# Gestational autoantibody exposure impacts early brain development in a rat model of MAR autism

**DOI:** 10.1038/s41380-025-02907-3

**Published:** 2025-02-10

**Authors:** Janna McLellan, Ana-Maria Iosif, Karol Cichewicz, Cesar Canales, Darlene Rahbarian, Melissa Corea, Melissa Bauman, Alex S. Nord, Judy Van de Water

**Affiliations:** 1 Department of Internal Medicine, Division of Rheumatology, Allergy, and Clinical Immunology, University of California, Davis, CA USA; 2 Department of Public Health Sciences, Division of Biostatistics, University of California, Davis, CA USA; 3 Center for Neuroscience, University of California, Davis, CA USA; 4 Department of Psychiatry and Behavioral Sciences, University of California, Davis, CA USA; 5 Department of Physiology and Membrane Biology, University of California, Davis, CA USA; 6 MIND Institute, University of California, Davis, CA USA; 7 Department of Neurobiology, Physiology and Behavior, University of California, Davis, CA USA

**Keywords:** Neuroscience, Autism spectrum disorders

## Abstract

Maternal autoantibody-related autism (MARA) is a subtype of autism characterized by the maternal production of specific patterns of autoantibodies during pregnancy, which significantly increases the likelihood of an autism diagnosis in their children. Multiple patterns of MARA autoantibodies (MARA-ABS) have been identified, and differences in the severity of the autism phenotype associated with each autoantibody pattern have been described. In this study, we utilized preclinical rat models to further elucidate the differential effects of MARA-AB exposure based on the known clinical patterns, including the originally reported pattern of lactate dehydrogenase A and B (LDHA/B) + collapsin response mediator protein 1 (CRMP1) + stress-induced phosphoprotein 1 (STIP1), as well as the more recently described patterns of CRMP1+CRMP2, CRMP1 + guanine deaminase (GDA), and STIP1+ neuron-specific enolase (NSE). We induced endogenous MARA-AB production in rat dams before pregnancy to expose offspring to the ABs throughout gestation. We found that in postnatal day 2 offspring exposed to MARA-ABS, the levels of brain and serum cytokines/chemokines/growth factors were altered based on the pattern of MARA-AB exposure. Further, bulk transcriptomic profiles of coronal sections containing hippocampal formation and the adjacent cortical and subcortical structures suggested changes in cellular proliferation and differentiation following MARA exposure. These combined observations demonstrate that gestational exposure to MARA-ABS alters early gene expression and immune signaling molecules, both of which may contribute to the altered neurodevelopment and behaviors associated with MARA.

## Introduction

Gestational insults on the developing brain, including hypoxic-ischemic events, infection, exposure to toxicants, and to maternal inflammation or autoantibodies can have impacts on long-term neurodevelopment, leading to neurodevelopmental disorders later in life [1–5]. More specifically, links between gestational insults from the maternal compartment have been identified as factors that can increase the risk of an autism diagnosis [5]. As the incidence of autism continues to rise, with a nearly 90% increase from 1 in 68 children in 2010 to 1 in 36 in 2020 [6], understanding how maternally derived exposures influence an autism diagnosis and behavioral severity is of the utmost importance.

In maternal autoantibody-related autism (MARA), the presence of maternally derived autoantibodies to specific proteins expressed in the fetal brain is associated with an autism diagnosis in the child [5, 7]. These target proteins include lactate dehydrogenase A/B (LDHA/B), collapsin response mediator proteins 1 and 2 (CRMP1/2), guanine deaminase (GDA), neuron-specific enolase (NSE), and stress-induced phosphoprotein 1 (STIP1) [8–10]. Early clinical studies identified that mothers with the combination of LDHA/B+STIP1+CRMP1 autoantibodies had children with a more severe autism phenotype compared to mothers of children with autism who lacked autoantibodies [8]. Later studies noted that the combinations of autoantibodies to CRMP1+CRMP2, CRMP1+GDA, and STIP1+NSE are highly prevalent in mothers of autistic children but are rare in mothers of typically developing children [11]. Furthermore, the CRMP1+CRMP2 MARA pattern has been found to correlate with more severe developmental delay in addition to autism in affected children [12]. Thus, MARA and the implications of gestational autoantibody exposure to these specific targets have become an area of intensive research. As maternal immunoglobulin G (IgG) can cross the placenta and enter the fetal compartment through neonatal Fc receptor (FcRn) uptake, it is believed that MARA autoantibodies (MARA-ABS) cause pathology when binding their targets within the fetal brain [13, 14]. However, despite the observed associations in clinical populations between the presence of the MARA-ABS and the diagnosis of autism in their offspring, the exact pathology induced by the MARA-ABS is still not well understood.

To address how gestational exposure to MARA-ABS impacts neurodevelopmental and behavioral outcomes, our lab developed endogenously driven mouse and rat models in which naïve females were immunized to generate antibodies against the original LDHA/B+STIP1+CRMP1 pattern [15–17]. Immunization of dams with specific epitopes targeted by MARA-ABS leads to the production of clinically relevant autoantibodies to which offspring are continuously exposed during gestation in the absence of elevated maternal cytokines and chemokines [15]. This model recapitulates the physiological method of exposure while ensuring that the maternal autoantibodies are the only alteration in the maternal compartment and should not be confused with the maternal immune activation model. In the MARA rodent models, we have found that exposed offspring had altered species-specific behavioral outcomes relevant to clinical autism, neuroanatomical changes, and IgG deposition in exposed offspring brains [15–17]. These models validated a pathological link between one pattern of MARA-ABS. However, further research is needed to clarify the specific differential effects of the additional clinically relevant MARA patterns on various aspects of brain development.

Therefore, we generated a novel rat model of MARA in which we immunized dams before pregnancy with the top three clinically relevant patterns of autoantigens, CRMP1+CRMP2, CRMP1+GDA, and STIP1+NSE, in addition to the original LDHA/B+STIP1+CRMP1 pattern. Using postnatal day (PND) 2 offspring, we aimed to identify changes in neurodevelopment in exposed offspring through analysis of brain and peripheral cytokine/chemokine/growth factor levels as well as brain transcriptomic profiles to determine how MARA-AB exposure might influence early neurodevelopmental trajectories.

## Methods and methods

### Peptide immunogen preparation

Peptides were synthesized by LifeTein LLC (Hillsborough, NJ) as Multiple Antigenic Peptides (MAPs), in which four copies of the same peptide epitope are synthesized on a lysine-based core (MAPs-4 system). The MAPs-4 system does not require a carrier protein, as the dense packing of multiple copies of an epitope combined with a high molar ratio produces a strong immunological response [9, 10]. The peptides corresponded to peptides from the clinically relevant epitopes of CRMP1, CRMP2, STIP1, NSE, GDA, LDHA, and LDHB [8–10] and are listed in Supplementary Table 1.

### Animal generation

Six-week-old male and female Sprague-Dawley rats were obtained from Charles River Laboratories (Portage, MI). All rats were pair-housed in a temperature/humidity-controlled vivarium on a 12-hour light-dark cycle, with food and water provided ad libitum. After two weeks of acclimation, female rat dams were randomly assigned to one of five treatment groups: CRMP1+CRMP2 (*n* = 5), STIP1+NSE (*n* = 7), CRMP1+GDA (*n* = 6), LDHA/B+CRMP1+STIP1 (*n* = 5), or adjuvant+saline control (*n* = 4).

**Fig. 1:**
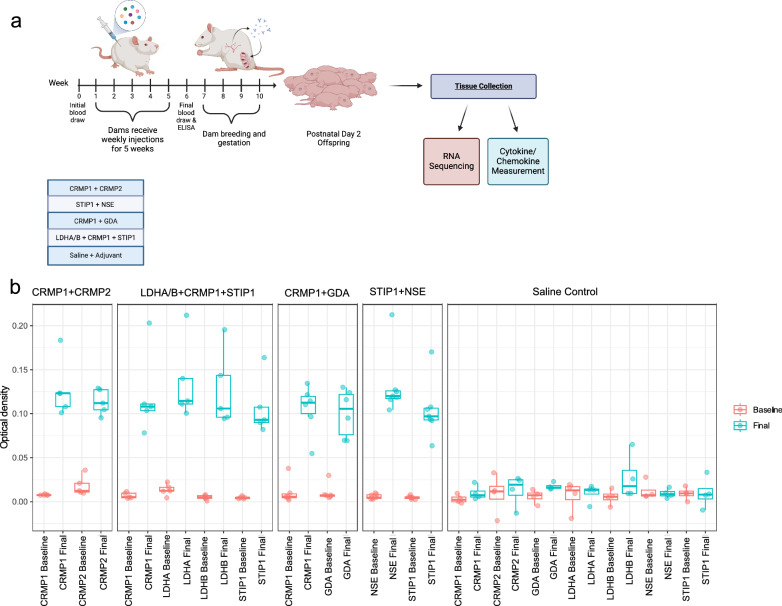
MAR rat model creation. **a** Timeline of autoantibody generation in naïve female rat dams and subsequent allocation of offspring. **b** Average optical densities of rat dams at baseline and after receiving immunizations.

Following random treatment assignment, a tail-vein blood draw was performed on each dam to obtain serum. Dams then received weekly subcutaneous injections for five weeks. Injection components comprised synthetic peptides according to treatment group and Freund’s adjuvant, with control dams receiving only injections of adjuvant and saline. A second blood draw was performed on each dam one week following the final injection to establish the dam’s antibody response. The schedule and injection mixture were based on our previous rodent studies [15]. To assess antibody response to injection, baseline and post-immunization dam sera were tested via enzyme-linked immunosorbent assay. Assay details have been described previously [16]. Supplementary Table 2 presents the number of dams in each treatment group and the average antibody responses per treatment group displayed as optical densities (OD), Fig. 1a summarizes the model generation, while pre- and post-immunization dam responses are shown in Fig. 1b.

Following the determination of the dam antibody response, animals were singly paired with breeder males for one week, after which they were singly housed. Weight was used to confirm pregnancy. Brain and blood samples were collected from offspring on PND2. Based on the number of available offspring per litter, dams were bred for a second time for additional offspring to collect tissue. For cytokine and chemokine measurement, tissue was collected from two litters, and sex allocations for experimental testing were limited based on the offspring availability. For RNA-sequencing, tissue was only collected from the dam’s first litter. Investigators were not blinded to pattern of AB exposure prior to experimental testing or data analysis. Sample sizes were chosen based on standards reported in associated literature and previous rodent studies using this model. For those brains allocated for RNA-sequencing, the brains were placed in RNA*later*™ Stabilization Solution (Invitrogen) and stored at −80 °C. Remaining brains were flash-frozen and stored at −80 °C until used for cytokine/chemokine/growth factor measurement. Trunk blood was collected from each animal for serum. Serum was stored at −80 °C until used for cytokine/chemokine/ growth factor measurement. Details of offspring allocation are provided in Table 1.

**Table 1:** Offspring allocations based on treatment group, sex, litter, and experimental use.

Tissue Allocation	CRMP1+CRMP2		STIP1+NSE		CRMP1+GDA		LDHA/B+CRMP1+STIP1		Control	
	Male	Female	Male	Female	Male	Female	Male	Female	Male	Female
	(pups per litter)		(pups per litter)		(pups per litter)		(pups per litter)		(pups per litter)	
Brain Cytokine/ Chemokine Measurement	12 (L1 = 8; L2 = 4)	12 (L1 = 8; L2 = 4)	12 (L1 = 9; L2 = 3)	12 (L1 = 8; L2 = 4)	12 (L1 = 5; L2 = 7)	12 (L1 = 5; L2 = 7)	16 (L1 = 4; L2 = 12)	14 (L1 = 9; L2 = 5)	16 (L1 = 5; L2 = 11)	14 (L1 = 0; L2 = 14)
Sera Cytokine/ Chemokine Measurement	12 (L1 = 8; L2 = 4)	12 (L1 = 8; L2 = 4)	12 (L1 = 9; L2 = 3)	12 (L1 = 8; L2 = 4)	12 (L1 = 5; L2 = 7)	12 (L1 = 5; L2 = 7)	20 (L1 = 8; L2 = 12)	18 (L1 = 13; L2 = 5)	20 (L1 = 9; L2 = 11)	18 (L1 = 1; L2 = 17)
RNA Sequencing	5 (L1 = 5)	3 (L1 = 3)	4 (L1 = 4)	4 (L1 = 4)	2 (L1 = 2)	4 (L1 = 4)	12 (L2 = 12)	13 (L2 = 13)	12 (L2 = 12)	12 (L2 = 12)

*LDHA/B* lactate dehydrogenase A/B, *CRMP1* collapsin response mediator protein 1, *CRMP2* collapsin response mediator protein 2, *GDA* guanine deaminase, *STIP1* stress-induced phosphoprotein 1, *NSE* neuron-specific enolase, *L1* litter 1, *L2* litter 2.

### Tissue preparation and multiplex assay

For PND2 brain samples, cell lysis buffer (Bio-rad) with cOmplete^TM^ protease inhibitors (Roche) was added and whole brain lysates were created using a handheld sonicator. Following a freeze-thaw cycle, tubes containing the samples were placed in a water bath sonicator for three minutes and then centrifuged at 7000 × *g* for eight minutes. The supernatant was removed and stored at 4 °C until total protein and cytokine/chemokine/growth factor levels were measured the following day. The total quantity of protein for each brain lysate was determined using a bicinchoninic acid assay (BCA) (Thermo Scientific, Rockford, IL). For BCA, samples were diluted (1:20) in PBS and run according to the manufacturer’s instructions.

To assess the levels of cytokines/chemokines/growth factors, we utilized a Millipore 27-plex magnetic bead kit (catalog # RECYMAG65K27PMX). The panel included interleukin (IL)-1α, IL-1β, IL-2, IL-4, IL-5, IL-6, IL-10, IL-12p70, IL-13, IL-17a, IL-18, tumor necrosis factor-alpha (TNFα), interferon-gamma (IFNγ), epidermal growth factor (EGF), vascular endothelial growth factor (VEGF), Leptin, macrophage inflammatory protein (MIP)-1α, MIP-2, monocyte chemoattractant protein-1 (MCP-1), granulocyte colony stimulating factor (G-CSF), granulocyte-macrophage colony-stimulating factor (GM-CSF), eotaxin, fractalkine, interferon gamma-induced protein-10 (IP-10), lipopolysaccharide-induced CXC chemokine (LIX), regulated on activation, normal T cell expressed and secreted (RANTES), and GRO/KC/CINC-1. PND2 serum samples were run according to kit instructions. For PND2 brain samples, we utilized the supernatants generated from the whole brain lysates described above. Brain supernatant samples were run neat following all other kit instructions and cytokine/chemokine/growth factor values were normalized based on total protein values obtained from the BCA assay.

### RNA-Sequencing and bioinformatics analysis

Seventy-one PND2 rat brains were collected for total RNA extraction (Table 1). On the day of the RNA isolation, brains were thawed on wet ice, and individual coronal slices (one per brain, ~150 um thick) were sub-dissected using the Rodent Brain Slicer Stainless Steel Matrix (BSMAS005-1, Zivic Instruments). To obtain the targeted region of interest, the brain was positioned in the slicer according to manufacturer instructions, and the target slice was collected by placing two razor blades in rostro-caudal positions 16th and 19th. The 150 um thick tissue slice collected in between these positions encompassed rat hippocampal formation and adjacent cortical and subcortical structures. Total RNA was obtained using Ambion RNAqueous Total RNA Isolation Kit (catalog # AM1912) and assayed via Agilent RNA 6000 Nano Bioanalyzer kit/instrument. Sample RIN scores were above 8.3. Poly-A-enriched mRNA libraries were prepared at Novogene using the Illumina TruSeq RNA Library Prep Kit and sequenced on the Illumina NovaSeq sequencer using the paired-end 150 method.

A total of 24 saline control and 47 MARA samples were stratified across 4 autoantibody combinations, as shown in Table 1. Samples were stratified across 3–9 dams (1 litter per dam) per autoantibody condition, with a median of 2 (male and female) animals per litter. The RNA-sequencing dataset is stratified across two experimental batches, which were processed and sequenced separately: 1) including control animals and LDHA/B+CRMP1+STIP1 animals, and 2) including control animals and all four MARA conditions. Additionally, a “Cohort” identifier was assigned to samples from litters born and tissue collected within a few days of each other. (Supplementary Table 3).

Reads were aligned to the rat UCSC rn7 genome using STAR (version 2.5.4b) [18], and gene counts were produced using featureCounts [19]. Data quality was assessed using FastQC [20], and principal component analysis (PCA) was used to determine sample outliers. Raw RNA-seq fastq files and a gene count matrix are available on GEO (GSE275431). Bioinformatic analysis was performed using R programming language version 4.2.1 (R Development Core Team, 2015) and RStudio integrated development environment version 2023.06.0 (Team R, 2018). Plots were generated using ggplot2 R package version 3.4.0 [21]. RNA-seq analysis script is available at: (https://github.com/NordNeurogenomicsLab/Publications/tree/master/MARA).

### Differential expression (DE) and gene ontology (GO) enrichment analyses

For DE analysis, we used the edgeR R package [19]. For models testing control versus MARA samples, we filtered our dataset to include genes expressed at a minimum of 1 count per million (CPM) in at least 8 samples. For models testing DE of individual MARA-AB patterns, this threshold was set to >1 CPM in at least 2 samples. DE models were adjusted to account for technical and sex-related variation using principal components or SVA batch correction methods [22]. Unless stated otherwise, DE analysis was conducted on samples from both experimental batches. SVA correction, closely resembling covariation captured by PC2, was used to account for technical variation. Replication of the control and LDHA/B+CRMP1+STIP1 samples across the two batches was used to determine the reproducibility of our MARA model. Reads per kilobase per million mapped reads (RPKM) were used to plot individual gene expression data. Gene ontology (GO) enrichment analysis was performed using the topGO R library [23].

### Statistical analysis

For analytes with small amounts of missing data (0.1 to 16%), where Luminex failed to detect the analyte, we imputed these values as below Limit of Detection (LOD) by batch divided by √2 [24]. These analytes included eotaxin, leptin, IL-2, and IFNγ, for comparisons between LDHA/B+CRMP1+STIP1 and control brain samples and IL-1β, IL-10, and IL-5 for sera samples. For analyses of CRMP1+CRMP2, STIP1+NSE, and CRMP1+GDA offspring, these included IL-5, TNFα, and IL-10 for the brain and IL-1β and EGF for the sera. For the analytes with more substantial missing data (ranging from 20% to 75%), we created dichotomous variables capturing whether or not the values fell under or over the detection limit. For comparisons between LDHA/B+CRMP1+STIP1 and controls, these included IL-12p70, GRO, and TNFα for the brain and G-CSF, eotaxin, IL-1α, IL-2, and IL-12p70 for the sera. For analyses of CRMP1+CRMP2, STIP1+NSE, and CRMP1+GDA offspring, G-CSF, IFNγ, GRO, and LIX in the brain, and G-CSF, eotaxin, IL-1α, IL-10, IL-12p70, and IFNγ in the sera were treated as dichotomous. When the missingness was high (>80%), variables were excluded from analyses. Thus, G-CSF, GM-CSF, LIX, IL-13, MCP-1, and MIP-2 were excluded from the analyses of the LDHA/B+CRMP1+STIP1 and control brain, and GM-CSF, IFNγ, IL-4, IL-6, IL-13, GRO, and MIP-2 from the analyses of the sera. Similarly, we excluded GM-CSF, IL-12p70, IL-13, MCP-1, and MIP-2 from the analyses of the CRMP1+CRMP2, STIP1+NSE, and CRMP1+GDA brain, and GM-CSF, IL-2, IL-4, IL-6, IL-13, GRO, and MIP-2 from the analyses of CRMP1+CRMP2, STIP1+NSE, and CRMP1+GDA sera.

Summary statistics, including mean, lower, and upper quartiles for each analyte prior to imputations or transformations in the brain and the sera, are displayed in Supplementary Tables 4, 5, respectively. Because data were hierarchical, with offspring nested within litters and litters nested within dams, the impact of autoantibody exposure on offspring brain and sera cytokines/chemokines/growth factors was assessed within a generalized linear mixed-effects models framework [25] that can accommodate normally distributed and binary data (via standard link functions) that are clustered (within dams). This flexible approach allows the use of all available data and accommodates unequal sample sizes across clusters. It also provides the ability to control for the effect of covariates of interest and to account for the intrinsic complexity of the data by modeling dam-specific random effects and residual correlations. Natural log or square-root transformations were employed if the normality assumption was not met. We accounted for within-dam correlation using a random intercept for the dam in all models. To avoid confounding due to batch differences, two separate sets of models were fitted, as cytokines for LDHA/B+CRMP1+STIP1 autoantibody-exposed and control offspring were processed in a different batch than those for CRMP1+CRMP2, STIP1+NSE, and CRMP1+GDA autoantibody-exposed offspring. Models fitted to LDHA/B+CRMP1+STIP1 and controls included a term for group (LDHA/B+CRMP1+STIP1 or control) and were adjusted for offspring sex (female or male) and litter number (first or second). Models fitted to CRMP1+CRMP2, STIP1+NSE, and CRMP1+GDA-exposed offspring included terms for group (CRMP1+CRMP2, STIP1+NSE, or CRMP1+GDA), coded using two dummy variables, and were adjusted for offspring sex (female or male) and litter number (first or second). To assess the statistical significance of the differences in cytokine/chemokine/growth factor concentrations among CRMP1+CRMP2, STIP1+NSE, and CRMP1+GDA autoantibody-exposed groups for both brain and sera samples, we first conducted two-degrees-of-freedom F-tests for group assessing overall differences between CRMP1+CRMP2, STIP1+NSE, and CRMP1+GDA-exposed offspring using the linear mixed-effects models described above. Then, for all analytes with significant overall F-tests for group, we constructed follow-up linear contrasts to identify all pairs with significant differences in cytokine/chemokine/growth factor concentrations.

Since the cytokines/chemokines/growth factors had different ranges and some were transformed for analyses, to evaluate the magnitude of the group differences, for the analytes that were analyzed as continuous variables, we used the linear mixed-effects models fitted for analyses to calculate standardized effect sizes (Cohen’s d) that account for clustering, imbalance in groups, and take covariates into account [19]. To assess the magnitude of the differences between any two groups (LDHA/B+CRMP1+STIP1 vs. controls in the first set of models or any of the CRMP1+CRMP2, STIP1+NSE, and CRMP1+GDA pair groups in the second set of models), we used the following formula:

$$d=\frac{t[1+{\left(\frac{{n}_{I}}{{n}_{O}}\right)}R]{\sqrt{1-R}({n}_{1}}+{n}_{2})}{\sqrt{{n}_{1}{n}_{2}}\sqrt{{n}_{O}-k}}$$

$$R=\frac{{s}_{B}^{2}}{{s}_{B}^{2}+{s}_{E}^{2}}$$

where *t* is the *t* value obtained for group (evaluating the difference between the two groups being compared) from the corresponding linear mixed-effects regression model, *n*_*1*_ and *n*_*2*_ are the numbers of offspring in each of the two groups being compared, *n*_*O*_ and *n*_*I*_ are the total number of offspring, and the number of dams, respectively $({n}_{O}{=n}_{1}+{n}_{2})$, *k* is the number of parameters (including the intercept), and *R* is the intraclass correlation, which consists of two variance components: ${s}_{B}^{2}$ (between-dam variance) and ${s}_{E}^{2}$ (within-dam variance or residual variance); they are obtained from the random-effect part of the mixed-effects model. To further enhance interpretability and enable comparisons with similar studies, we back-transformed the least-squares means for models where significant cytokines were square-root or log-transformed. The back-transformed least-squares means at each level of the group represent the expected cytokine level in the respective group at the average of the covariate levels, providing a more representative effect of the group while accounting for covariates. We then calculated and reported the differences between the back-transformed least-squares means for groups, allowing for a more explicit interpretation of group differences on the original scale.

All model assumptions (e.g., residual distribution, heterogeneity of variances, random effects structure, etc.) were validated both graphically and analytically. Hypothesis tests were two-sided, with α = 0.05. Analyses were conducted in SAS OnDemand version 9.4. (SAS Institute Inc., Cary, NC).

## Results

### Gestational autoantibody exposure alters cytokine/chemokine/growth factor profiles in brain and serum in LDHA/B+CRMP1+STIP1-exposed offspring compared to saline controls

**Fig. 2:**
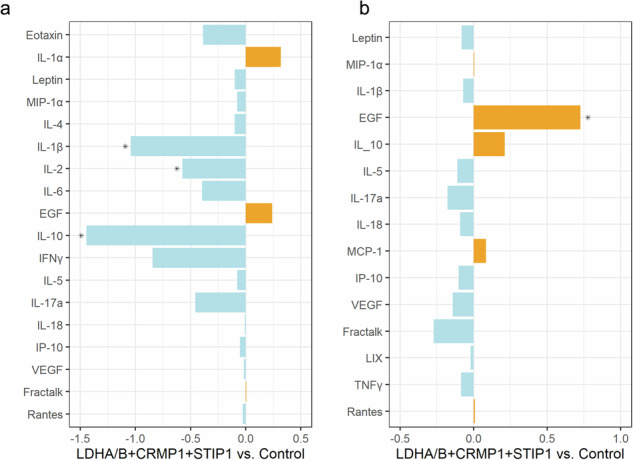
Effect size comparisons between LDHA/B+CRM1+STIP1-exposed and control offspring. Estimated effect sizes (Cohen’s d) for adjusted group differences between LDHA/B+CRMP1+STIP1 and control groups in PND2 offspring brain (**a**) and sera (**b**) cytokine, chemokine and growth factor concentrations were assessed using linear mixed-effects models. Cytokine, chemokine, and growth factors with higher concentrations in offspring from treated dams than controls are shown in yellow. Those with higher concentrations in offspring from control dams than those from treated dams are shown in blue. Comparisons with significant group differences (*p* < 0.05) are marked with asterisks (*).

We used linear mixed-effects models to compare the cytokine/chemokine/growth factor levels between PND2 offspring brains from dams immunized with LDHA/B+CRMP1+STIP1 to those from adjuvant/saline controls. In this comparison, we found significantly lower levels of IL-1β (estimated difference [est.] = −0.57, standard error [SE] = 0.14,d = −1.04, *p* = 0.0003), IL-2 (est. = −0.73, SE = 0.34, d = −0.57, *p* = 0.04), and IL-10 (est. = −0.32, SE = 0.06, d = −1.44, *p* < 0.0001) in the brains of the MARA-AB exposed offspring compared to controls. Both IL-1β and IL-10 were square-root transformed for analyses; thus, the reported differences translate to −8.22 pg/mL for IL-1β and −1.18 pg/mL for IL-10 on the original scale. Values for IL-2 were not transformed. We also observed relatively lower levels of IFNγ (est. = −1.51, SE = 0.48, d = −0.84, *p* = 0.09) levels in LDHA/B+CRMP1+STIP1-exposed offspring, translating to a difference of −11.66 pg/mL on the original scale, but this difference did not reach statistical significance. (Fig. 2a and Supplementary Table 6).

When we examined the serum levels of the same analytes, we found that autoantibody-exposed offspring had significantly higher levels of EGF (est. = 2.29, SE = 0.66, d = 0.73, *p* = 0.03) compared to saline controls (Fig. 2b and Supplementary Table 7). Values for EGF were natural-log transformed for analyses; thus, the reported difference translates to 81.89 pg/mL on the original scale. In both the brain and the serum comparisons, IL-12p70 was treated as binary due to low detectable values. Using logistic mixed-effects models, we found that detectable reads were significantly less likely in LDHA/B+CRMP1+STIP1-exposed offspring brains (est. = −2.26, SE = 0.84, *p* = 0.01) and sera (est. = −1.09, SE = 0.51, *p* = 0.04) than in controls (Supplementary Tables 6, 7). These data confirmed that exposure to the LDHA/B+CRMP1+STIP1 pattern resulted in changes in brain and peripheral cytokines/chemokines levels.

### Differential cytokine/chemokine/growth factor levels as a result of offspring exposure to clinically relevant MARA-ABS

**Fig. 3:**
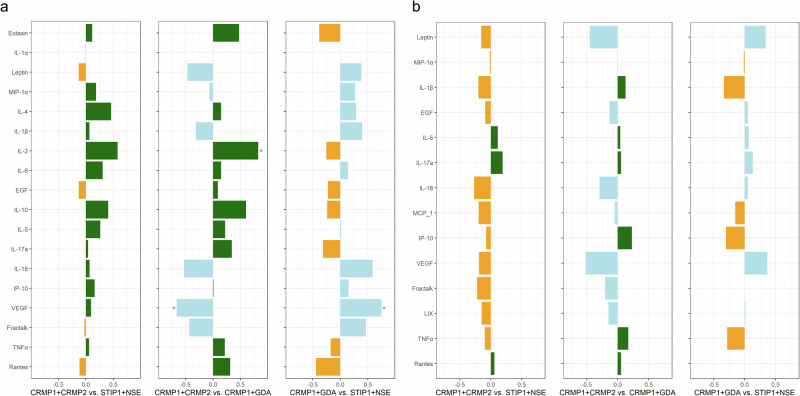
Effect size comparisons between CRMP1+CRMP2, STIP1+NSE, and CRMP1+GDA-exposed offspring. Estimated effect sizes (Cohen’s d) for adjusted pairwise group differences between CRMP1+CRMP2, STIP1+NSE, and CRMP1+GDA groups in PND2 offspring brain (**a**) and sera (**b**) cytokine, chemokine, and growth factor concentrations assessed using mixed-effects linear models. Cytokine/chemokines/growth factors with higher concentrations in offspring from CRMP1+CRMP2 dams compared to offspring from STIP1+NSE or CRMP1+GDA dams are shown in green. Those with higher concentrations in offspring from CRMP1+GDA dams are shown in blue. Those with higher concentrations in offspring STIP1+NSE dams are shown in yellow. Comparisons with significant group differences (*p* < 0.05) are marked with asterisks (*).

In addition to the LDHA/B+CRMP1+STIP1 pattern, we induced autoantibody production to three additional patterns of autoantibodies: CRMP1+CRMP2, CRMP1+GDA, and STIP1+NSE, to determine if there were differential effects based on autoantibody pattern. Using the same analytic framework, we found that IL-2 levels varied significantly between the treatments (*p* = 0.03), with significantly higher levels in the CRMP1+CRMP2 autoantibody-exposed brains compared to the CRMP1+GDA (est. = 0.90, SE = 0.33, d = 0.82, *p* = 0.01) brains. Values for IL-2 were not transformed for analyses. In addition, we found that the CRMP1+CRMP2 brains had significantly lower levels of VEGF (*p* = 0.04) compared to CRMP1+GDA (est. = −0.32, SE = 0.15, d = −0.66, *p* = 0.03). CRMP1+GDA autoantibody-exposed offspring also had higher levels of VEGF in the brain when compared to STIP1+NSE autoantibody-exposed brains (est. = 0.36, SE = 0.15, d = 0.74, *p* = 0.02) (Fig. 3a and Supplementary Table 8). Values for VEGF were square-root transformed for analyses; thus, the reported differences translate to −4.16 pg/mL when comparing CRMP1+CRMP2 and CRMP1+GDA autoantibody-exposed brains and 4.73 pg/mL when comparing CRMP1+GDA and STIP1+NSE autoantibody-exposed brains on the original scale. When examining serum levels, we found no statistically significant differences among the three treatment groups (Fig. 3b and Supplementary Table 9).

In the brain analysis, we treated IFNγ as a dichotomous variable due to low numbers of detectable values. We found that IFNγ was detected more frequently in the brain of CRMP1+CRMP2 autoantibody-exposed offspring compared to those exposed to autoantibodies to CRMP1+GDA (est. = 1.55, SE = 0.69, *p* = 0.03) and STIP1+NSE (est. = 1.32, SE = 0.65, *p* = 0.046) (Supplementary Table 8). In serum, we observed a similar pattern with the chemokine eotaxin, with detectable values significantly more likely in CRMP1+CRMP2 autoantibody-exposed offspring compared to the CRMP1+GDA group (est. = 2.73, SE = 1.02, *p* = 0.01) (Supplementary Table 9). Thus, the effects of MARA autoantibodies on offspring cytokine and chemokine levels in the brain and sera vary depending on the MARA-ABS the developing fetus is exposed to.

### Gestational MARA-AB exposure yields transcriptomic changes in PND2 brain

To investigate the systems-level effect of exposure to MARA-ABS during cortical development, we performed an RNA-sequencing study on PND2 cortical samples exposed to the four combinations of MARA-ABS and saline controls. The dataset consists of two experimental batches including, (1) the LDHA/B+CRMP1+STIP1 and control samples, and (2) all autoantibody combinations and independent controls, further stratified across sample collection cohorts. Data from experimental batches were combined, and SVA correction [22] or PCA-based correction was applied to adjust for technical variation, unless stated otherwise. Sequencing depth ranged from 24.9 to 36.2 million reads per sample (Supplementary Fig. 1a). Sample sex was verified using expression of female and male sex markers genes, Xist and Eif2s3y, respectively (Supplementary Fig. 1b, c). PCA did not indicate the presence of any outlier samples. However, it identified strong correlations with the experimental batch/cohort in PC1 and PC2 (Supplementary Fig. 2a, c, and d). PC1 explained 96.8% of the variance in the data (Supplementary Fig. 2b). We interpret this finding as a technical variation, likely caused by minor differences in tissue dissection, variation in developmental timing, or other differences in conditions during dissection and sample processing. Sample sex was significantly correlated with PC5-7.

**Fig. 4:**
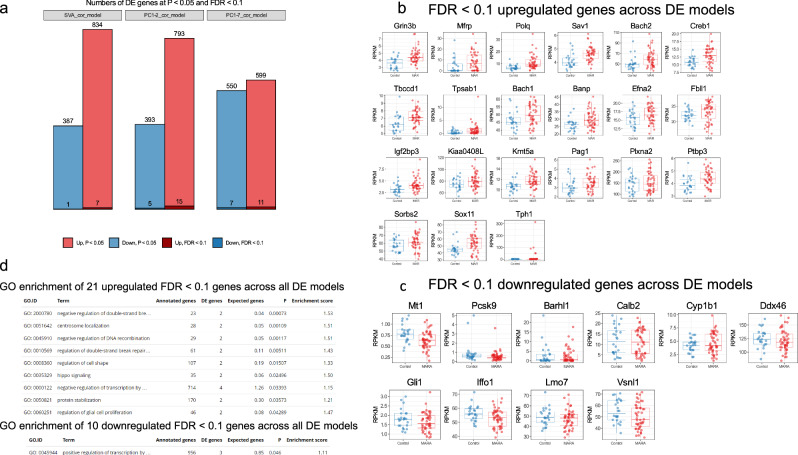
General MARA DE signature. **a** Number of DE genes passing thresholds of *p* < 0.05 and FDR < 01. **b** Upregulated genes across DE models passing FDR < 0.1. **c** Downregulated genes across DE models passing FDR < 0.1 (**d**) GO enrichment of top up- and downregulated genes across all DE models passing FDR < 0.1.

In our DE analysis, we first investigated the presence of a general MARA phenotype in cortical development. In the second part, we examined phenotypes of each autoantibody exposure separately. To maximize our chances of identifying a biologically relevant signature, we performed our DE testing using edgeR general linear models (GLM) including covariates correcting for technical and sex-related variation. To maximize the sensitivity of our approach and avoid DE model overfit, we applied three DE models to our data, with varying levels of bias/variance tradeoff: 1) DE model corrected for the first surrogate variable, equivalent to PC2 (SVA batch correction) [22] DE model corrected for PC1 and PC2; and 3) DE model corrected for the first seven PCs, correcting for most of the technical and sex-related variation. Across the three models, our DE analysis identified 8, 20, and 18 DE genes at a strict FDR < 0.1 significance threshold (Benjamini–Hochberg correction). After combining FDR < 0.1 gene sets, we found 31 unique genes, 21 upregulated and 10 downregulated. (Fig. 4a–c, Supplementary Tables 10–12). When we inspected the expression patterns of these genes, we found a wide expression range and variance across control and MARA conditions. To identify autoantibody drivers and genes with consistently up- or downregulated expression, we stratified by individual MARA-AB exposures (Supplementary Fig. 3a, b). We found that some of these genes were consistently upregulated across all or most peptide conditions (e.g., *Grin3b*, *Mfrp*, *Polq*, *Sav1*), whereas others changed expression in response to specific autoantibody pairs (e.g., *Bach1*, *Bach2*, *Pag1*, *Mt1* in response to anti-CRMP1+CRMP2 and anti-LDHA/B+CRMP1+STIP1; or *Efna2*, *Kiaa0408L*, *Calb2*, and *Vsnl1* in response to anti-LDHA/B+CRMP1+STIP1).

In order to interpret this signature at a systems level, we performed GO enrichment analysis on 21 upregulated and 10 downregulated genes. For upregulated genes we found enrichment of GO terms associated with regulation of transcription, DNA repair, and glial cell proliferation. For downregulated genes, we found only one significantly enriched term indicating enrichment of genes associated with regulation of transcription (Fig. 4d, Supplementary Tables 13−14). Being aware of the limitations of performing GO enrichment on such a small gene set, we expanded it to genes passing a more inclusive *p* < 0.05 threshold. In that analysis, for upregulated genes we found significant enrichment of GO terms associated with regulation of transcription and the immune response. We found enrichment of GO terms for the downregulated genes indicating altered synaptic transmission (Supplementary Tables 15−16).

**Fig. 5:**
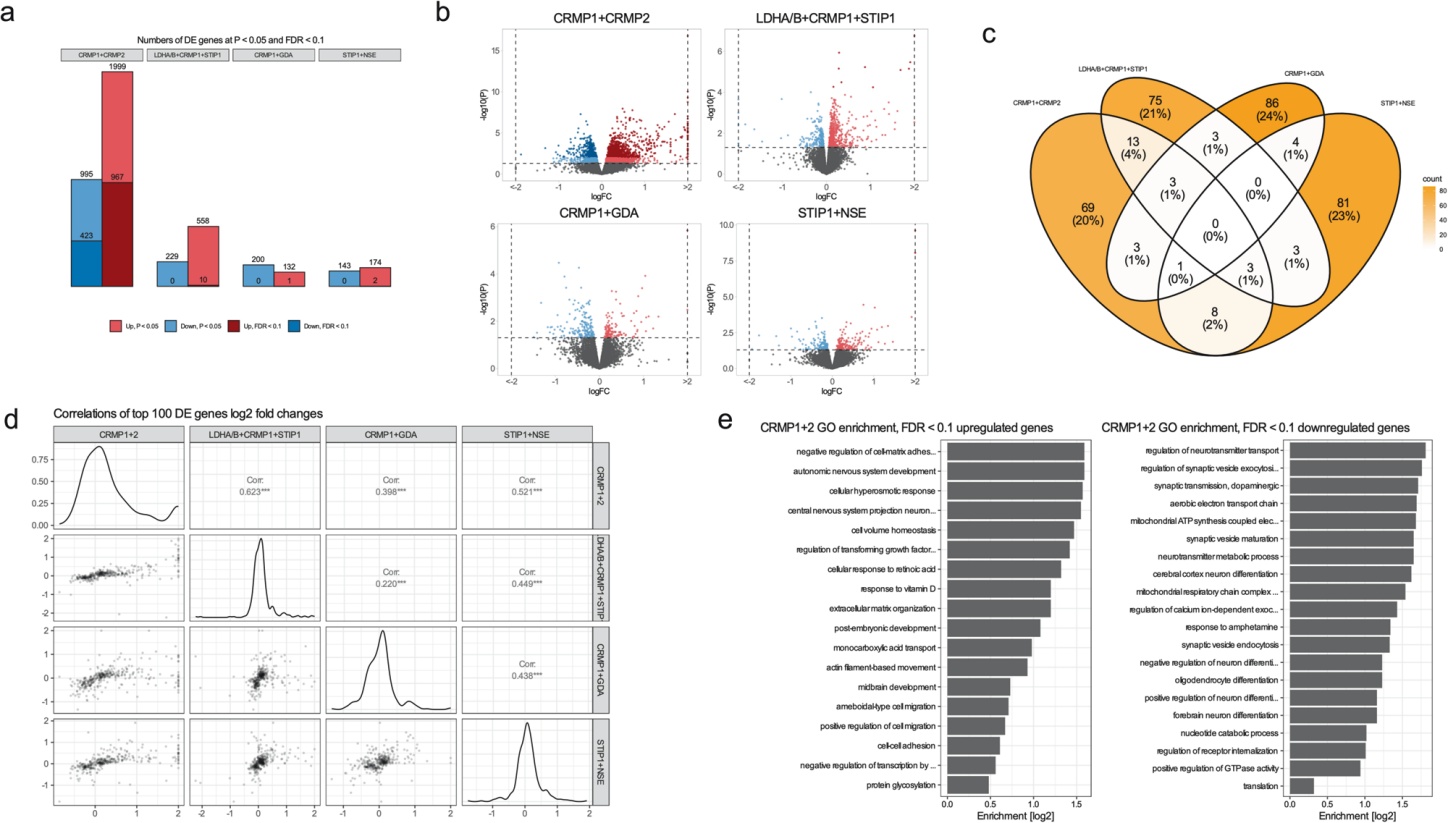
DE analysis by autoantibody exposure. **a** Numbers of DE genes passing thresholds of *p* < 0.05 and FDR < 01. **b** Volcano plots split by autoantibody exposure. **c** Intersections of 100 most significant DE genes by autoantibody exposure. **d** Correlations of top 100 DE genes log2 fold changes. **e** CRMP1+CRMP2 GO enrichment for up- and downregulated genes passing FDR < 0.1.

Next, we stratified our DE analysis by autoantibody exposure. We found that exposure to maternal CRMP1+CRMP2 autoantibodies elicits the strongest DE response with over 1390 DE genes passing FDR < 0.1, which aligns with our clinical observation that CRMP1+CRMP2 is associated with a more severe autism phenotype [12, 13]. Exposure to LDHA/B+CRMP1+STIP1 autoantibodies demonstrated a much weaker response with ten upregulated genes at FDR < 0.1. CRMP1+GDA and STIP1+NSE had only 1 and 2 upregulated genes at FDR < 0.1, respectively. There were no downregulated genes passing our FDR < 0.1 in these three exposure comparisons (Fig. 5a). DE of CRMP1+CRMP2 and LDHA/B+CRMP1+STIP1 was shifted towards upregulation in both the number of DE genes and log_2_ fold change effect size (Fig. 5a, b), demonstrating that *in utero* exposure to MARA-ABS has a strong downstream effect on gene expression.

Next, we asked if DE signatures of the individual autoantibody combinations are shared or are unique to each exposure condition. We intersected gene lists of 100 of the most significantly DE genes; 19/100 genes were shared between CRMP1+CRMP2 and LDHA/B+CRMP1+STIP1, 12/100 genes were shared between CRMP1+CRMP2 and STIP1+NSE, and fewer than 10 genes were shared between the other exposure pairs (Fig. 5c). Furthermore, we performed a quantitative correlation analysis between DE log2 fold changes. We found that the sign of DE of these genes, as signified by positive Pearson correlation values, is mostly concordant across all exposures. Most of these genes fell in quadrants 1 and 3, and few genes fell in quadrants 2 and 4, especially those with high fold change values (Fig. 5d). Thus, although qualitative intersections of DE gene sets do not reveal high similarities, quantitative correlations demonstrate similarities across autoantibody exposures.

Taking advantage of the two independent batch replicates of LDHA/B+CRMP1+STIP1 and saline control samples, the correlation of DE results of these replicate sets demonstrated high concordance with a ratio of directionally concordant to discordant genes at 1015/430 and 260/101, at *p* < 0.05 and FDR < 0.1, respectively (Supplementary Fig. 4a, b), demonstrating overall reproducibility of transcriptomic effects in this MARA model.

The DE response to CRMP1+CRMP2 is by far the strongest, and we examined this signature using GO enrichment analysis. At FDR < 0.1 in upregulated DE genes, we found significant enrichment of GO terms related to cell-matrix adhesion, and nervous system development. Downregulated genes were enriched for GO terms related to synaptic transmission, and neuron differentiation, energy production and metabolism, and translation (Fig. 5e, Supplementary Tables 17−18). GO enrichment for other autoantibody conditions was tested at DE *p* < 0.05 (Supplementary Fig, 5a–c, Supplementary Tables 19−24).

## Discussion

Mounting clinical [5, 7–12] and preclinical [15–17, 26] evidence strongly implicates exposure to MARA-ABs in altered neurodevelopment. Here, we utilized a translationally relevant rat model to determine the impact of gestational exposure to clinically relevant patterns of MARA-ABs on gene expression and immune signaling molecules in the early postnatal brain.

In the PND2 rat brain, the neurodevelopmental processes are most equivalent to what occurs during the third trimester in humans [27]. For rats, beginning at approximately embryonic day 15 and extending into the early weeks of postnatal life, neural progenitor cells begin to proliferate, differentiate, and migrate throughout the developing brain [28, 29]. Synaptogenesis also begins during gestation and peaks during the early weeks of life in the rat, requiring neurons to migrate to the cortical plate where they develop their synaptic connections [30]. Similar to humans, rodents have a hemochorial placenta that allows maternal IgG to cross the placenta via the FcRn receptor during gestation [31], beginning at approximately embryonic day 13 [32]. After birth, the passage and uptake of IgG continues via maternal milk, with FcRn receptors also expressed in the neonatal intestine [33, 34]. Due to the vulnerability of the fetal brain during key developmental processes and before the full formation of the blood-brain barrier [35, 36], it is believed that the MARA-ABS can access their protein targets once within the fetal compartment. This interaction could therefore alter the trajectory of early neurodevelopment and impact signaling molecules such as cytokines, chemokines, and growth factors, leading to lasting effects associated with MARA pathology [37]. To determine the downstream effects of MARA-AB exposure, we used our novel preclinical rat model in which dams are immunized to endogenously express CRMP1+CRMP2, STIP1+NSE, CRMP1+GDA, and LDHA/B,+CRMP1+STIP1 autoantibodies to examine levels of brain and peripheral cytokines/chemokines/growth factors in addition to brain-specific transcriptomic changes.

LDHA/B+CRMP1+STIP1-exposed brains had significantly lower levels of several pro-inflammatory cytokines than saline controls, including IL-1β, IL-2, and IFNγ. While the developing brain is vulnerable to inflammation, and inflammation is often considered to be pathological in nature, it is important to note that sufficient levels of this class of cytokines/chemokines are necessary for proper neurodevelopment. IL-1β, for example, has been shown to be highly expressed in the developing brain and can impact neural precursor cells via Wnt5 signaling, which is necessary for neuronal differentiation [38]. Deficiencies in IL-2 and IFNγ have been linked to alterations in learning and memory [39] and social behavior, respectively [40]. This suggests that a reduced level of these immune signaling molecules could negatively impact brain development in the exposed offspring.

In the serum, we found increased EGF in LDHA/B+CRMP1+STIP1 offspring compared to controls. While some studies have found a correlation between decreased levels of EGF and an autism diagnosis, studies in a younger cohort observed higher levels of peripheral EGF in autistic patients [41]. Accelerated brain growth and increased total cerebral volume are often described in autistic individuals. Increased brain volume was also observed in our MARA (LDHA/B+CRMP1+STIP1) rat model [15] and our original mouse studies [17]. As EGF is functionally important for the proliferation of cells that develop into neurons and glia [42], elevated EGF at an early postnatal timepoint suggests it could contribute to brain overgrowth in MARA. Observations of brain overgrowth in earlier preclinical studies conducted in mice [16, 17], rats [15], and nonhuman primates [26] strongly supports our current findings and suggests that this is a trait consistent with the MARA pathology.

Although little clinical research has formerly addressed cytokine/chemokine changes in children from mothers who express MARA-ABs, one such study that examined this relationship in newborn bloodspots determined that neonates gestationally exposed to MARA-ABs had significantly higher levels of the T cell chemoattracting cytokine IL-16 compared to neonates from women who did not express MARA-ABs [43]. While not measured in our preclinical model, IL-16 plays a role in EGF signal transduction [44]. This is of interest given our observation of significantly higher EGF in serum of LDHA/B+CRMP1+STIP1 offspring. Although the findings in human MARA neonates and our animal model offspring cannot be directly compared, the potential correlation between the findings in MARA neonates and MARA rat offspring further implicates EGF as an analyte of interest in MARA.

When assessing the additional clinical MARA-AB patterns, we found a significant decrease in VEGF in CRMP1+CRMP2 and STIP1+NSE-exposed offspring brains compared to CRMP1+GDA-exposed offspring. VEGF is required for the formation and survival of blood vessels both peripherally and in the brain and has also been shown to aid in proper neuronal migration [45]. Therefore, a decrease in this growth factor could indicate impairments in the blood-brain barrier and impacts on neuronal fates. We also found that CRMP1+CRMP2-exposed offspring have significantly higher levels of IL-2 and more detectable reads for IFNγ. Excess levels of these cytokines could result in more severe impacts on the neurological development of CRMP1+CRMP2 autoantibody-exposed offspring. In our clinical studies, CRMP1+CRMP2 has been the most prevalent MARA autoantibody pattern and is most highly associated with a more severe autism phenotype. In contrast, the CRMP1+GDA pattern is less commonly associated with increased odds of intellectual disability (ID) and is significantly higher in the autism with no ID population [12]. Although we did not observe many significant differences in cytokine levels among these three additional MARA exposure groups, we believe that the trending differences, as shown by medium to large effect sizes, could implicate the potential for differences in MARA severity based on autoantibody reactivity profile.

At the transcriptomic level, we observed changes in genes linked to the regulation of transcription, proliferation, and neuronal differentiation in offspring exposed to MARA-ABS. This phenotype mirrors the abnormal neuronal growth trajectory described in autistic individuals [46]. More specifically, we found upregulation of *wnt* signaling genes and genes involved in neuroblast formation. *Wnt* has previously been implicated in autistic patients, with excess numbers of cortical neurons and enlarged brain volumes having been linked to dysregulated *wnt* signaling [47, 48]. This finding is supported by our previous observation of increased regional brain volumes in LDHA/B+CRMP1+STIP1-exposed offspring [15] and the increased levels of peripheral EGF identified here. We also observed upregulation of genes that activate the mitogen-activated protein kinase (MAPK) cascade and downregulation of those that induce nuclear factor kappa-light-chain-enhancer of activated B cells (NFκB) signaling. Aberrant signaling of the MAPK pathway has been linked to several neurodevelopment disorders, including autism, as it plays a crucial role in proper neuronal development within the cerebral cortex [49]. NFκB signaling has been identified to play roles in neurogenesis and neurite outgrowth, particularly in the hippocampus, where it has impacts on learning and memory, as well as in the regulation of dendritic spine density [50, 51]. Immunologically, MAPK and NFκB are involved in the release of several cytokines/chemokines, including IL-1β, IL-2, IL-10, and IL-12 [52, 53]. All of these were lower in the LDHA/B+CRMP1+STIP1 autoantibody-exposed offspring compared to saline/adjuvant controls, with IL-2 being higher in CRMP1+CRMP2 autoantibody-exposed offspring compared to CRMP1+GDA autoantibody-exposed offspring. Thus, alterations in the functions of these pathways could have direct impacts on the release of these cytokines.

When we assessed the differential effects of the individual clinical patterns, we found the highest number of differentially expressed genes in the CRMP1+CRMP2-exposed offspring, suggesting a more profound impact of this MARA pattern. As previously mentioned, this mirrors what is observed clinically, with children from mothers with CRMP1+CRMP2 autoantibodies having a higher incidence of developmental delay [12]. These results reflect brain-specific alterations of RNA expression as a result of MARA autoantibody exposure, elucidating potential downstream changes that could be implicated mechanistically in altered neurodevelopment.

Our analyses should be considered in light of several study limitations. Experimentally, we were limited in the cytokine/chemokine/growth factors comparisons we could make between control offspring and those offspring exposed to the additional clinical MARA-AB patterns due to plate-to-plate variation. In this first study of additional MARA patterns, the statistical power to detect medium to large effect sizes was limited, and we elected to present *p*-values uncorrected for multiple comparisons, as well as effect sizes to offer a comprehensive view of the results. As a result, it is important to exercise caution when interpreting *p*-values that are not adjusted for multiple comparisons, as they may lead to an increased risk of Type I errors. Further, as this was the first study of the newly identified autoantibody patterns, we began our exploration of downstream effects from MARA-AB using bulk RNA sequencing to examine the systems-level impacts of autoantibody exposure on gene expression. Future studies are needed to test if single gene DE effects are linked to changes in protein levels, to map DE effects to the specific perturbed cell types, and to test for associated functional impacts in relevant pathways and processes.

In conclusion, we used our novel rat models to examine the brain and peripheral cytokines/chemokines/growth factors and brain transcriptomic profiles of PND2 offspring exposed to clinical patterns of MARA-ABS. We not only observed changes when we compared the original MARA pattern to controls, confirming that MARA autoantibody exposure directly impacts factors critical for healthy neurodevelopment, but we also confirmed that the effect on the offspring varied based on the specific MARA pattern. The changes we observed in exposed offspring validated previous findings from our preclinical studies and mirrored changes noted in the brains of autistic individuals, such as altered brain size and neuronal cell numbers. Thus, this novel study provides insights into the variable pathological effects of MARA-ABS, highlighting their impact on neurodevelopment, downstream expression of critical genes, and changes in key developmental immune molecules. Future studies will include direct comparisons between offspring exposed to each clinical pattern and controls and be adequately powered to examine differences in the presence of multiple confounders and covariates, as well as provide validation of the DEG studies at the protein level. Investigations are currently underway to further elucidate the clinical pattern-induced effects on behavior and other aspects of immune and neurodevelopment.

## Supplementary information

Supplementary Materials Combined

## Data Availability

Data that is not already available in the main text or Supplementary Materials are available from author upon request.
