# Supplementary material for: Gestational autoantibody exposure impacts early brain development in a rat model of MAR autism
Source: Mol Psychiatry. 2025 Feb 10;30(7):3018–28. doi: 10.1038/s41380-025-02907-3 (PMC12185337; doi:10.1038/s41380-025-02907-3)
Supplement: Supplementary file 1 — Supplementary Materials Combined [file 41380_2025_2907_MOESM1_ESM.pdf]

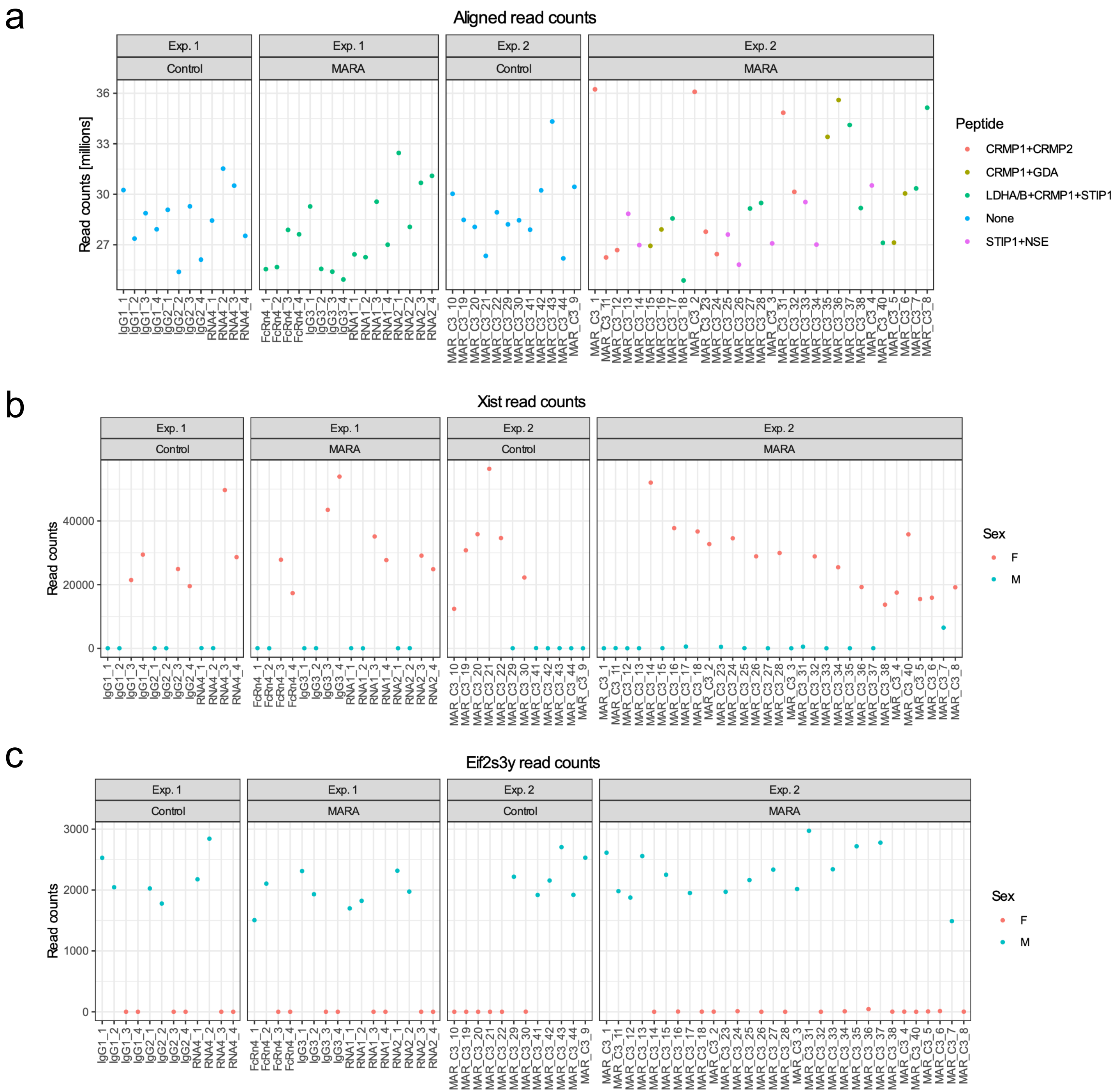
a
Aligned read counts
Exp. 1
Control
Exp. 1
MARA
Exp. 2
Control
Exp. 2
MARA
Read counts [millions]
Peptide
CRMP1+CRMP2
CRMP1+GDA
LDHA/B+CRMP1+STIP1
None
STIP1+NSE
b
Xist read counts
Exp. 1
Control
Exp. 1
MARA
Exp. 2
Control
Exp. 2
MARA
Read counts
Sex
F
M
c
Eif2s3y read counts
Exp. 1
Control
Exp. 1
MARA
Exp. 2
Control
Exp. 2
MARA
Read counts
Sex
F
M

a

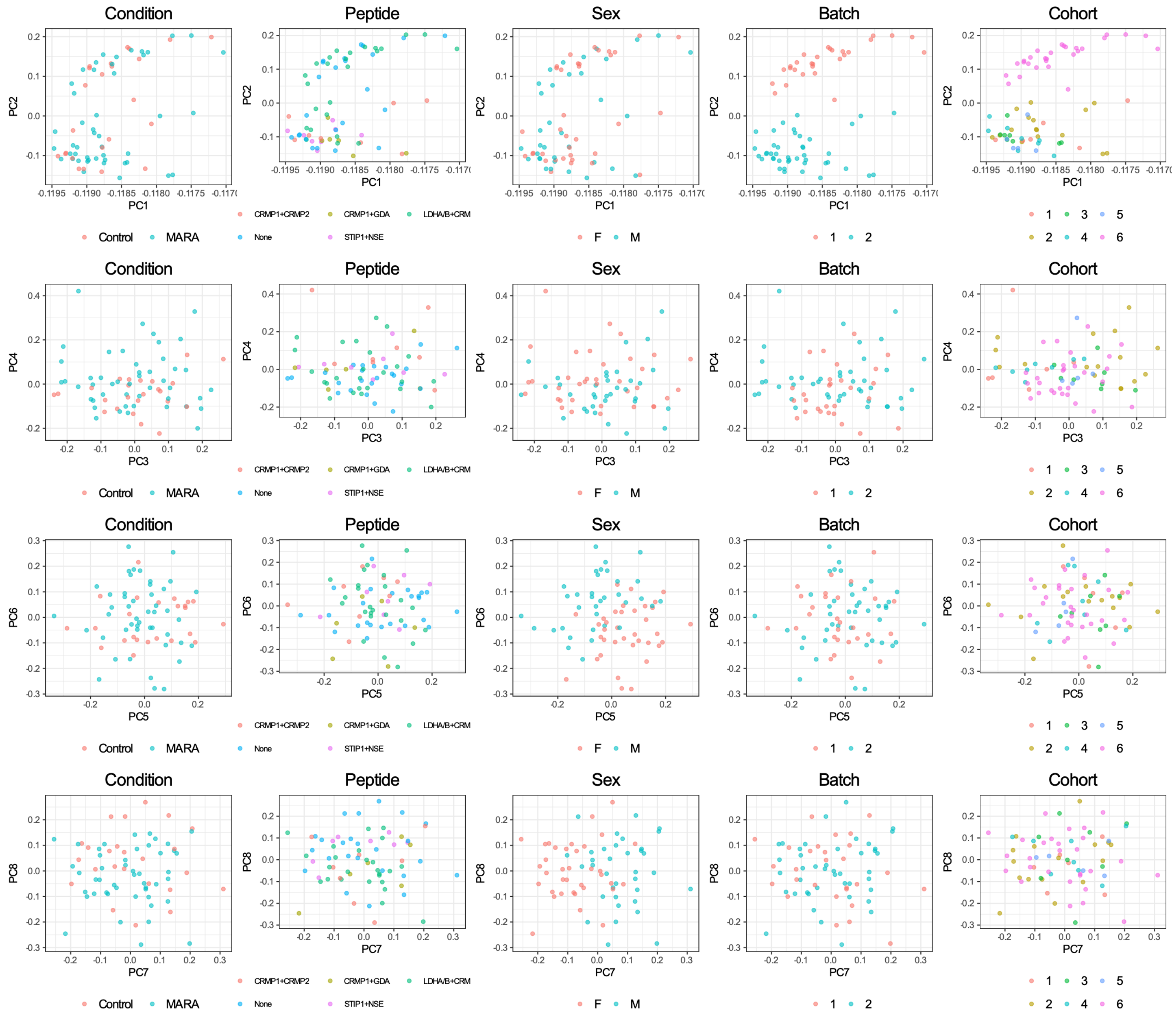

b

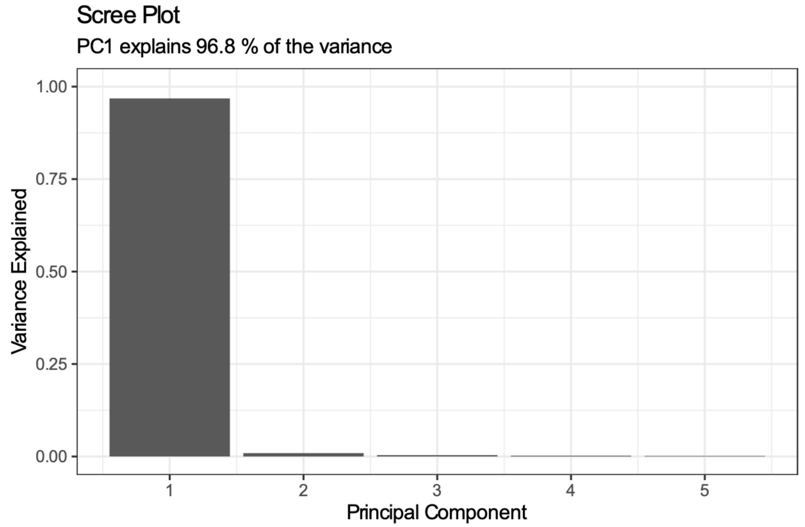

c

Correlations of PC eigenvalues and experimental variables; Pearson's R

	PC1	PC2	PC3	PC4	PC5	PC6	PC7	PC8	PC9	PC10
Condition	0.012	0.116	0.049	0.267	0.164	0.074	0.069	0.24	0.182	0.089
Peptide	0.161	0.087	0.017	0.268	0.241	0.027	0.013	0.297	0.216	0.149
Sex	0.077	0.068	0.086	0.197	0.476	0.492	0.661	0.029	0.073	0.058
Batch	0.349	0.935	0.016	0.251	0.15	0.046	0.018	0.01	0.023	0.023
Cohort	0.176	0.759	0.005	0.277	0.226	0.061	0.043	0.041	0.124	0.005

Color scale: 0.2, 0.4, 0.6, 0.8

d

Correlations of PC eigenvalues and experimental variables; P values.

	PC1	PC2	PC3	PC4	PC5	PC6	PC7	PC8	PC9	PC10
Condition	0.9212	0.3351	0.6853	0.0245	0.1713	0.5369	0.5653	0.0435	0.1298	0.4599
Peptide	0.1787	0.4715	0.8879	0.0236	0.0431	0.8208	0.9167	0.0118	0.0704	0.2162
Sex	0.5239	0.5739	0.476	0.1002	0	0	0	0.8101	0.5429	0.6287
Batch	0.0028	0	0.8964	0.0346	0.2127	0.7016	0.8802	0.9363	0.8464	0.8519
Cohort	0.1421	0	0.9696	0.0191	0.0578	0.6134	0.7236	0.7368	0.3032	0.9673

Color scale: 0.2, 0.4, 0.6, 0.8

a

FDR < 0.1 upregulated genes across DE models

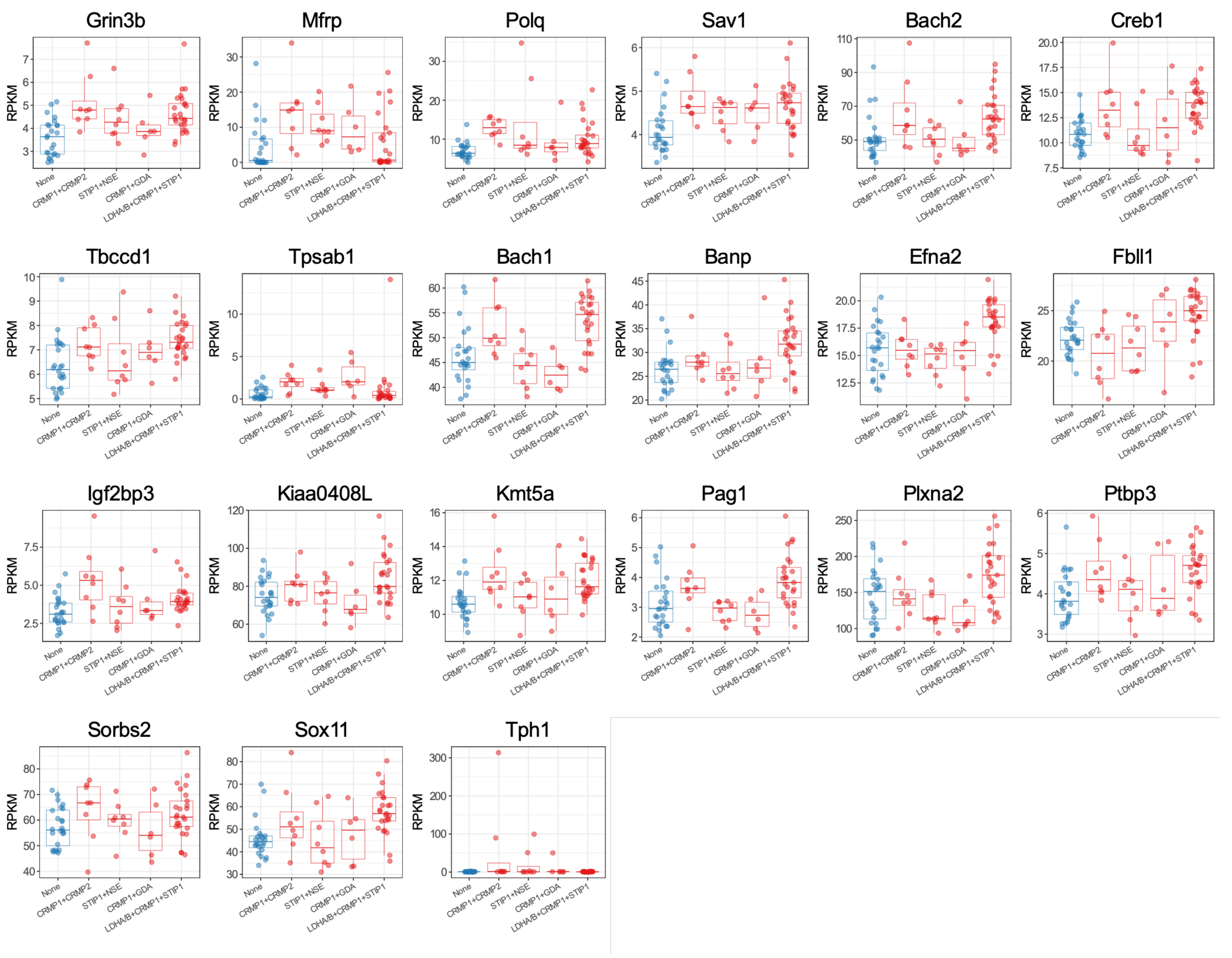

b

FDR < 0.1 downregulated genes across DE models

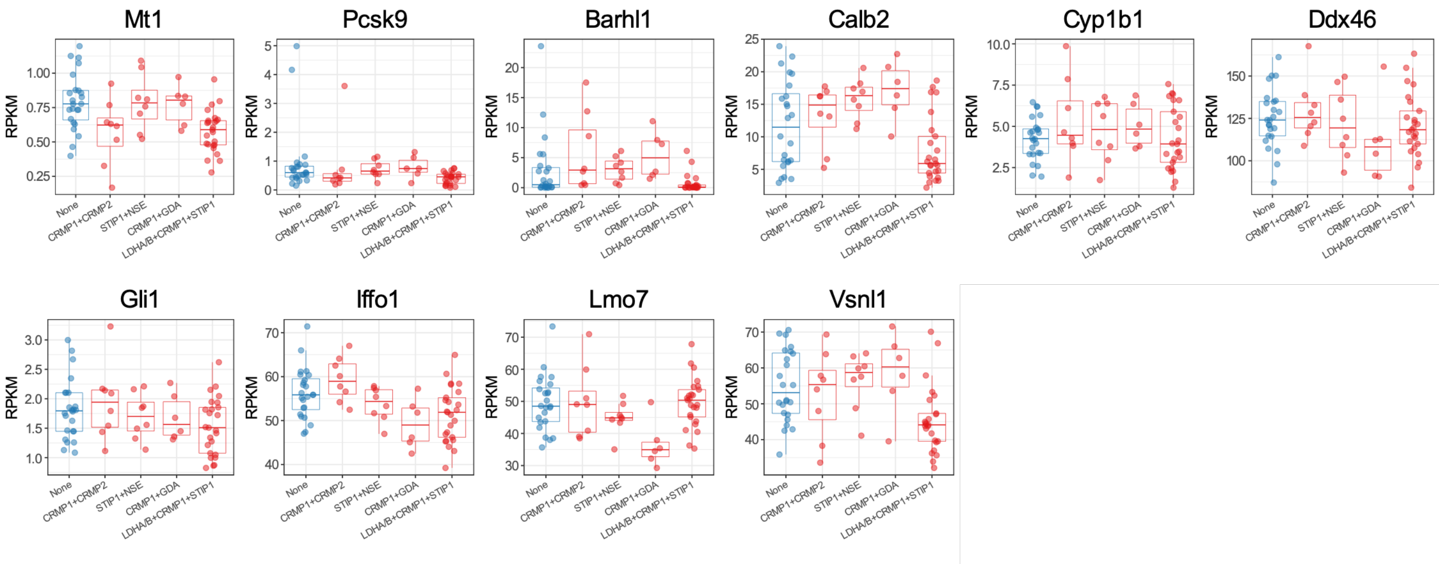

a b

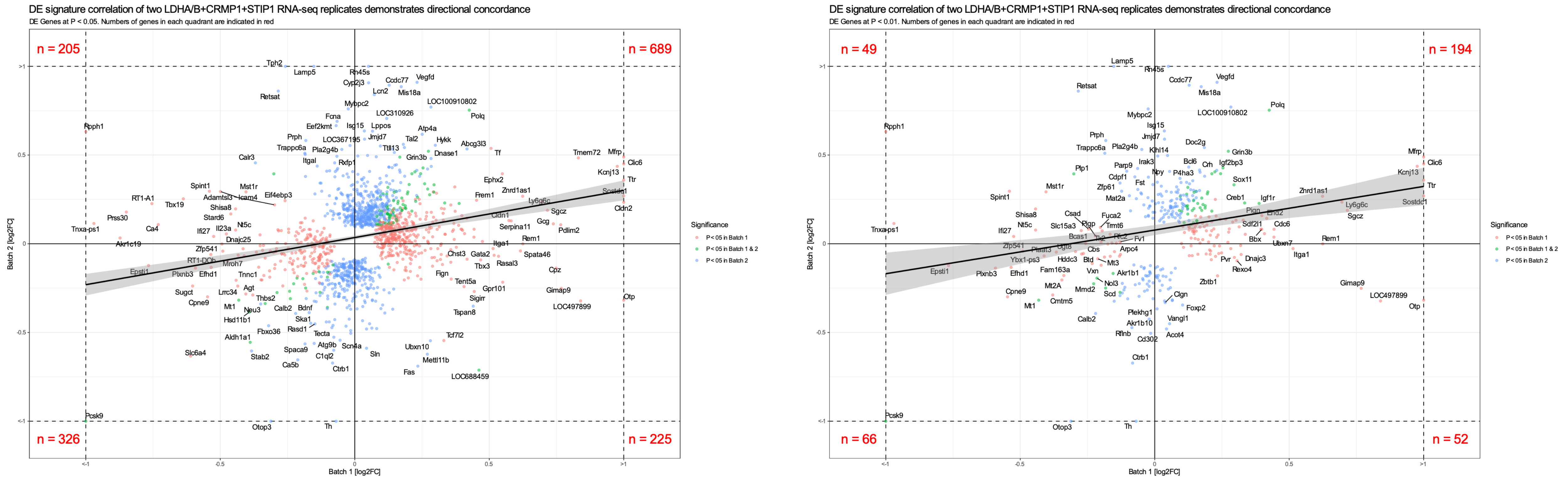

DE signature correlation of two LDHA/B+CRMP1+STIP1 RNA-seq replicates demonstrates directional concordance
DE Genes at P < 0.05. Numbers of genes in each quadrant are indicated in red
n = 205
n = 689
n = 326
n = 225
Batch 2 [log2FC]
Batch 1 [log2FC]
Significance
P < 05 in Batch 1
P < 05 in Batch 1 & 2
P < 05 in Batch 2
DE signature correlation of two LDHA/B+CRMP1+STIP1 RNA-seq replicates demonstrates directional concordance
DE Genes at P < 0.01. Numbers of genes in each quadrant are indicated in red
n = 49
n = 194
n = 66
n = 52
Batch 2 [log2FC]
Batch 1 [log2FC]
Significance
P < 05 in Batch 1
P < 05 in Batch 1 & 2
P < 05 in Batch 2

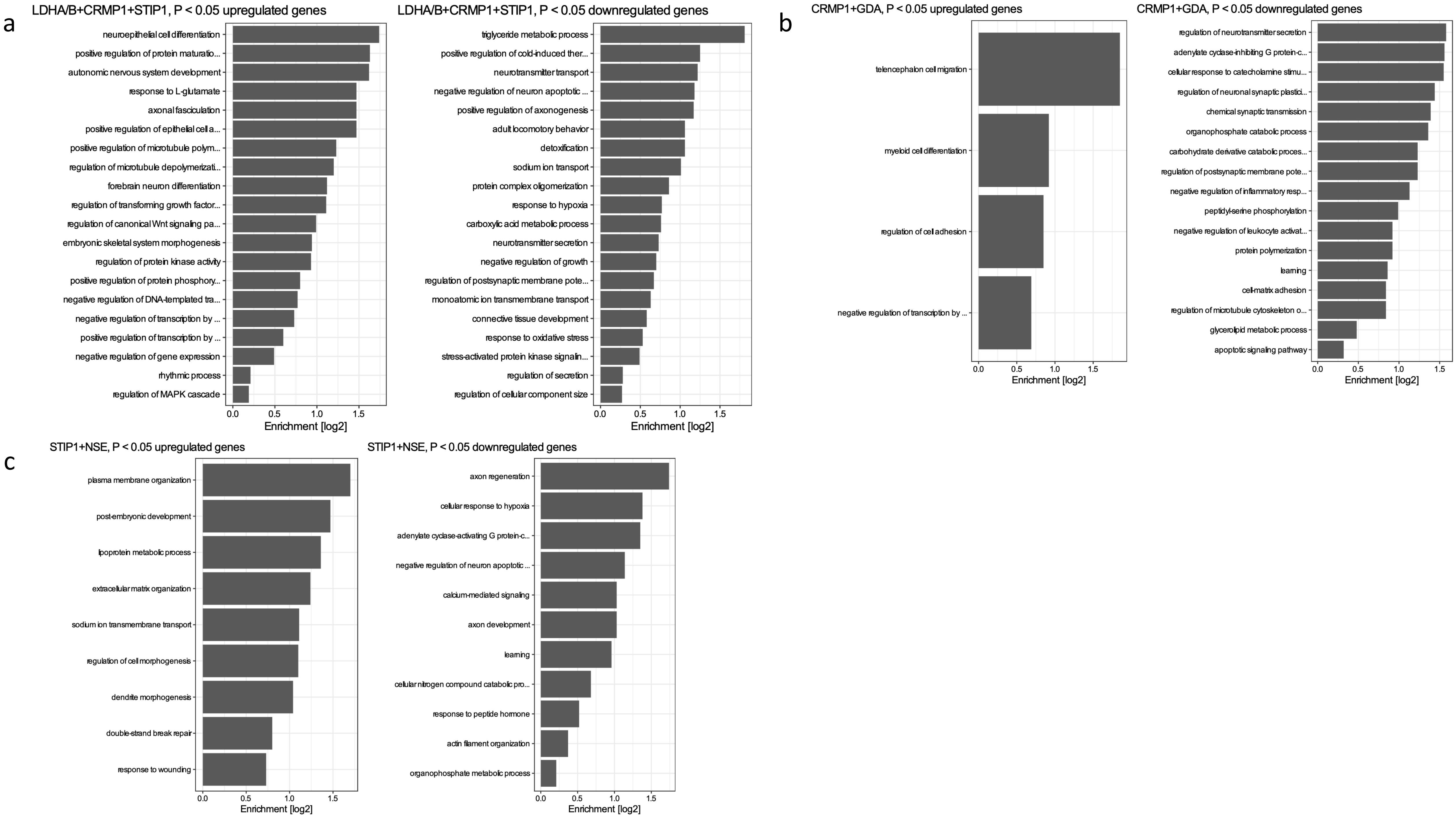
a
LDHA/B+CRMP1+STIP1, P < 0.05 upregulated genes
neuroepithelial cell differentiation
positive regulation of protein maturatio...
autonomic nervous system development
response to L-glutamate
axonal fasciculation
positive regulation of epithelial cell a...
positive regulation of microtubule polym...
regulation of microtubule depolymerizati...
forebrain neuron differentiation
regulation of transforming growth factor...
regulation of canonical Wnt signaling pa...
embryonic skeletal system morphogenesis
regulation of protein kinase activity
positive regulation of protein phosphory...
negative regulation of DNA-templated tra...
negative regulation of transcription by ...
positive regulation of transcription by ...
negative regulation of gene expression
rhythmic process
regulation of MAPK cascade
0.0
0.5
1.0
1.5
Enrichment [log2]
LDHA/B+CRMP1+STIP1, P < 0.05 downregulated genes
triglyceride metabolic process
positive regulation of cold-induced ther...
neurotransmitter transport
negative regulation of neuron apoptotic ...
positive regulation of axonogenesis
adult locomotory behavior
detoxification
sodium ion transport
protein complex oligomerization
response to hypoxia
carboxylic acid metabolic process
neurotransmitter secretion
negative regulation of growth
regulation of postsynaptic membrane pote...
monoatomic ion transmembrane transport
connective tissue development
response to oxidative stress
stress-activated protein kinase signalin...
regulation of secretion
regulation of cellular component size
0.0
0.5
1.0
1.5
Enrichment [log2]
b
CRMP1+GDA, P < 0.05 upregulated genes
telencephalon cell migration
myeloid cell differentiation
regulation of cell adhesion
negative regulation of transcription by ...
0.0
0.5
1.0
1.5
Enrichment [log2]
CRMP1+GDA, P < 0.05 downregulated genes
regulation of neurotransmitter secretion
adenylate cyclase-inhibiting G protein-c...
cellular response to catecholamine stimu...
regulation of neuronal synaptic plastici...
chemical synaptic transmission
organophosphate catabolic process
carbohydrate derivative catabolic proces...
regulation of postsynaptic membrane pote...
negative regulation of inflammatory resp...
peptidyl-serine phosphorylation
negative regulation of leukocyte activat...
protein polymerization
learning
cell-matrix adhesion
regulation of microtubule cytoskeleton o...
glycerolipid metabolic process
apoptotic signaling pathway
0.0
0.5
1.0
1.5
Enrichment [log2]
c
STIP1+NSE, P < 0.05 upregulated genes
plasma membrane organization
post-embryonic development
lipoprotein metabolic process
extracellular matrix organization
sodium ion transmembrane transport
regulation of cell morphogenesis
dendrite morphogenesis
double-strand break repair
response to wounding
0.0
0.5
1.0
1.5
Enrichment [log2]
STIP1+NSE, P < 0.05 downregulated genes
axon regeneration
cellular response to hypoxia
adenylate cyclase-activating G protein-c...
negative regulation of neuron apoptotic ...
calcium-mediated signaling
axon development
learning
cellular nitrogen compound catabolic pro...
response to peptide hormone
actin filament organization
organophosphate metabolic process
0.0
0.5
1.0
1.5
Enrichment [log2]

Supplementary Figure 1: Sequencing depth and validation of sample sex. a) Samples were sequence to a median depth of 28.4 million reads, ranging from 24.8 to 36.2 million reads. b, c) Sample sex was confirmed based on X and Y chromosome gene expression Xist (LOC100911498) and Eif2s3y, respectively. Figure facets indicate experimental batches and MARA conditions.

Supplementary Figure 2: Principal component analysis (PCA). a) Sample PCA eigenvalue scatter plots of the first 8 PCs color coded to indicate experimental variables: Condition (MAR vs Control), peptide treatments, sex, experiment batch, and cohort stratifying samples in experiment 2. b) Scree plot quantifying the amount of variation explained by the first 5 PCs. c) Pearson's absolute R correlation values calculated between PC eigenvalues and experimental variables and d) their corresponding P values indicate that experiment batch variable is correlated with PC1 and PC2. Correlation with the cohort is driven by experiment batch. Sample sex is correlated with PCs 5-7.

Supplementary Figure 3: a) Union of DE genes passing FDR < 0.05 across SVA, PC1-2, and PC1-7 – corrected models; a) upregulated; b) downregulated.

Supplementary Figure 4: Correlation of log2 fold changes in two experiment replicates testing DE between controls and LDHA/B+CRMP1+STIP1 autoantibody exposure. Genes indicated in red pass the significance threshold in batch 1, genes indicated in blue pass the significance threshold in batch 2, genes indicated in green pass significance threshold in both experiment replicates. Black lines indicate linear model fit, and shaded area indicates 95% confidence interval. Numbers in corners indicate gene counts in each quadrant.
a) DE genes at P < 0.05. b) DE genes at P < 0.01.

Supplementary Figure 5: GO biological process enrichment scores of DE signatures, calculate as $\log_2$ ratios of observed DE genes to expected number of genes in a GO term, passing enrichment significance of P < 0.05 [Fisher's exact test]. Enriched GO terms were filtered to include at least 5 DE genes, and terms not broader than 1000 genes. GO terms unrelated to brain function were removed. a) LDHA/B+CRMP1+STIP1 upregulated and downregulated DE genes at P < 0.05. b) CRMP1+GDA upregulated and downregulated DE genes at P < 0.05. c) STIP1+NSE upregulated and downregulated DE genes at P < 0.05.

Supplementary Table 1. Peptides used to generate autoantibody responses to the MARA proteins in rat dams

Protein	Peptides
Collapsin Response Mediator Protein 1	VDITSWYDGVREELE, VVPEPGSSLLTSFEK, VTSPPLSPDPTTPDY, VGSDADVVIWDPDKL
Collapsin Response Mediator Protein 2	HNSSLEYNIFEGMEC, MAERKQSGKAAEDEE, VKTISAKTHNSSLEYN
Stress-Induced Phosphoprotein 1	VDLGSMDEEEEIATP, VLLGVDLGSMDEEEE, PPPPPPKKETKPEPME, TNQAAVYFEKGDYNK
Neuron-Specific Enolase	DVAASEFYRDGKYDL, ERLAKYNQLMRIEEE, GGFAPNILENSEALE, GDQLGALYQDFVRDY, DFVRDYPVVSIEDPF, SIEDPFDQDDWMWS, LAKYNQLMRIEEELG, QLMRIEEELGDEARF
Guanine Deaminase	MDLNDTFPEYKETTE
Lactate Dehydrogenase A	ADELALVDVIEDKLK, VDVIEDKLKGEMMDL
Lactate Dehydrogenase B	DCIIIVVSNPVDILT, ESMLKNLSRIHPVSTMV, IAPVAEEEATVPNNKIT

Supplementary Table 2. Pre-gestational dam sera reactivity toward MARA antigens before injections (baseline) and one week post final injection (final)

	LDHA/B+CRMP1 +STIP1 n=5	CRMP1+CRMP2 n=5	CRMP1+GDA n=6	STIP1+NSE n=7	Control n=4
Protein	Average Optical Densities: Baseline; Final				
LDHA	0.014; 0.136	–	–	–	0.007; 0.01
LDHB	0.005; 0.127	–	–	–	0.005; 0.028
STIP1	0.005; 0.107	–	–	0.005; 0.104	0.009; 0.010
CRMP1	0.007; 0.121	0.008; 0.128	0.011; 0.105	–	0.003; 0.010
CRMP2	–	0.018; 0.114	–	–	0.009; 0.013
NSE	–	–	–	0.006; 0.132	0.012; 0.010
GDA	–	–	0.011; 0.101	–	0.006; 0.017

Abbreviations: LDHA/B: lactate dehydrogenase A/B; CRMP1: collapsin response mediator protein 1; CRMP2: collapsin response mediator protein 2; GDA: guanine deaminase; STIP1: stress-induced phosphoprotein 1; NSE: neuron-specific enolase.

Supplementary Table 3

Sample	Condition	Peptide	Sex	Age	Cohort	Dam_ID	Collection_Date	Batch	Sequencing_depth	Xist_counts	Xist_normalized	Eif2s3y_counts
**MAR_C3_1**	MARA	CRMP1+CRMP2	M	PND2	1	9_1	9/6/2021	2	36234952	33	9.10722884357622e-07	2613
**MAR_C3_2**	MARA	CRMP1+CRMP2	F	PND2	1	9_1	9/6/2021	2	36086969	32743	0.000907335830836887	2
**MAR_C3_3**	MARA	STIP1+NSE	M	PND2	2	9_2	11/8/2021	2	27081577	24	8.86211316276006e-07	2017
**MAR_C3_4**	MARA	STIP1+NSE	F	PND2	2	9_2	11/8/2021	2	30523869	17505	0.000573485622022556	2
**MAR_C3_5**	MARA	CRMP1+GDA	F	PND2	1	9_3	9/4/2021	2	27126679	15473	0.000570397872883739	6
**MAR_C3_6**	MARA	CRMP1+GDA	F	PND2	1	9_3	9/4/2021	2	30048556	15895	0.000528977166157336	12
**MAR_C3_7**	MARA	LDHA/B+CRMP1+STIP1	M	PND2	2	9_4	11/10/2021	2	30347372	6502	0.000214252489474212	1489
**MAR_C3_8**	MARA	LDHA/B+CRMP1+STIP1	F	PND2	2	9_4	11/10/2021	2	35141768	19166	0.000545390886423244	2
**MAR_C3_9**	Control	None	M	PND2	1	9_5	9/5/2021	2	30441695	21	6.89843321799262e-07	2531
**MAR_C3_10**	Control	None	F	PND2	1	9_5	9/5/2021	2	30029914	12412	0.000413321196990441	0
**MAR_C3_11**	MARA	CRMP1+CRMP2	M	PND2	2	9_6	11/5/2021	2	26246595	68	2.59081225583738e-06	1981
**MAR_C3_12**	MARA	CRMP1+CRMP2	M	PND2	2	9_6	11/5/2021	2	26681216	22	8.24550125451554e-07	1876
**MAR_C3_13**	MARA	STIP1+NSE	M	PND2	2	9_7	11/7/2021	2	28841270	45	1.56026416312458e-06	2558
**MAR_C3_14**	MARA	STIP1+NSE	F	PND2	2	9_7	11/7/2021	2	26980190	52040	0.00192882259168672	0
**MAR_C3_15**	MARA	CRMP1+GDA	M	PND2	2	9_8	11/7/2021	2	26933517	52	1.93067990340808e-06	2251
**MAR_C3_16**	MARA	CRMP1+GDA	F	PND2	2	9_8	11/7/2021	2	27910006	37749	0.00135252568559104	4
**MAR_C3_17**	MARA	LDHA/B+CRMP1+STIP1	M	PND2	2	9_9	11/9/2021	2	28564733	559	1.95695860346393e-05	1951
**MAR_C3_18**	MARA	LDHA/B+CRMP1+STIP1	F	PND2	2	9_9	11/9/2021	2	24880182	36692	0.00147474805449574	2
**MAR_C3_19**	Control	None	F	PND2	2	9_10	11/9/2021	2	28478004	30788	0.00108111509500455	0
**MAR_C3_20**	Control	None	F	PND2	2	9_10	11/9/2021	2	28059510	35838	0.00127721403545536	0
**MAR_C3_21**	Control	None	F	PND2	2	9_10	11/9/2021	2	26339240	56364	0.00213992506997165	0
**MAR_C3_22**	Control	None	F	PND2	2	9_10	11/9/2021	2	28929430	34632	0.00119712002621552	0
**MAR_C3_23**	MARA	CRMP1+CRMP2	M	PND2	3	9_11	1/15/2022	2	27773875	464	1.67063472417875e-05	1971
**MAR_C3_24**	MARA	CRMP1+CRMP2	F	PND2	3	9_11	1/15/2022	2	26444429	34560	0.0013068915195711	10
**MAR_C3_25**	MARA	STIP1+NSE	M	PND2	3	9_12	1/16/2022	2	27608350	61	2.20947648084728e-06	2164
**MAR_C3_26**	MARA	STIP1+NSE	F	PND2	3	9_12	1/16/2022	2	25818198	28889	0.00111893943953796	0
**MAR_C3_27**	MARA	LDHA/B+CRMP1+STIP1	M	PND2	3	9_14	1/16/2022	2	29154316	37	1.26910883452042e-06	2335
**MAR_C3_28**	MARA	LDHA/B+CRMP1+STIP1	F	PND2	3	9_14	1/16/2022	2	29483065	29946	0.00101570172571949	0
**MAR_C3_29**	Control	None	M	PND2	3	9_15	1/16/2022	2	28212293	29	1.02792070109296e-06	2218
**MAR_C3_30**	Control	None	F	PND2	3	9_15	1/16/2022	2	28452897	22239	0.000781607581119068	2
**MAR_C3_31**	MARA	CRMP1+CRMP2	M	PND2	4	9_16	3/15/2022	2	34843334	516	1.48091454164518e-05	2973
**MAR_C3_32**	MARA	CRMP1+CRMP2	F	PND2	4	9_16	3/15/2022	2	30143629	28868	0.000957681638133219	0
**MAR_C3_33**	MARA	STIP1+NSE	M	PND2	4	9_17	3/15/2022	2	29537176	25	8.46391002308413e-07	2341
**MAR_C3_34**	MARA	STIP1+NSE	F	PND2	4	9_17	3/15/2022	2	27010282	25460	0.000942604005393205	8
**MAR_C3_35**	MARA	CRMP1+GDA	M	PND2	4	9_18	3/13/2022	2	33410373	37	1.10744049460328e-06	2719
**MAR_C3_36**	MARA	CRMP1+GDA	F	PND2	4	9_18	3/13/2022	2	35600728	19253	0.000540803547612847	44
**MAR_C3_37**	MARA	LDHA/B+CRMP1+STIP1	M	PND2	4	9_19	3/12/2022	2	34116932	44	1.28968220237388e-06	2778
**MAR_C3_38**	MARA	LDHA/B+CRMP1+STIP1	F	PND2	4	9_19	3/12/2022	2	29181468	13714	0.000469955795232783	2
**MAR_C3_40**	MARA	LDHA/B+CRMP1+STIP1	F	PND2	5	9_24	5/23/2022	2	27113212	35793	0.00132013130720182	0
**MAR_C3_41**	Control	None	M	PND2	5	9_25	5/22/2022	2	27894483	85	3.04719754081838e-06	1919
**MAR_C3_42**	Control	None	M	PND2	5	9_25	5/22/2022	2	30232288	28	9.2616212176862e-07	2156
**MAR_C3_43**	Control	None	M	PND2	5	9_25	5/22/2022	2	34323510	53	1.54413112178795e-06	2705
**MAR_C3_44**	Control	None	M	PND2	5	9_25	5/22/2022	2	26191426	32	1.2217738736333e-06	1921
**IgG1_1**	Control	None	M	PND2	6	IgG1	07_2021	1	30249895	27	8.92565081630862e-07	2529
**IgG1_2**	Control	None	M	PND2	6	IgG1	07_2021	1	27366089	25	9.13539380800815e-07	2046
**IgG1_3**	Control	None	F	PND2	6	IgG1	07_2021	1	28875963	21450	0.000742832368915281	0
**IgG1_4**	Control	None	F	PND2	6	IgG1	07_2021	1	27919525	29426	0.00105395775895184	0
**IgG2_1**	Control	None	M	PND2	6	IgG2	07_2021	1	29077604	54	1.85709936760952e-06	2026
**IgG2_2**	Control	None	M	PND2	6	IgG2	07_2021	1	25389628	28	1.10281253431519e-06	1778
**IgG2_3**	Control	None	F	PND2	6	IgG2	07_2021	1	29283282	24903	0.000850416971704196	0
**IgG2_4**	Control	None	F	PND2	6	IgG2	07_2021	1	26116328	19526	0.000747654877056223	0
**RNA4_1**	Control	None	M	PND2	6	RNA4	07_2021	1	28439195	89	3.12948379867996e-06	2176
**RNA4_2**	Control	None	M	PND2	6	RNA4	07_2021	1	31524646	56	1.77638790932022e-06	2843
**RNA4_3**	Control	None	F	PND2	6	RNA4	07_2021	1	30511930	49692	0.00162860887528255	0
**RNA4_4**	Control	None	F	PND2	6	RNA4	07_2021	1	27528424	28643	0.00104048818777275	0
**IgG3_1**	MARA	LDHA/B+CRMP1+STIP1	M	PND2	6	IgG3	07_2021	1	29276466	33	1.12718522788919e-06	2312
**IgG3_2**	MARA	LDHA/B+CRMP1+STIP1	M	PND2	6	IgG3	07_2021	1	25566850	43	1.68186538427691e-06	1931
**IgG3_3**	MARA	LDHA/B+CRMP1+STIP1	F	PND2	6	IgG3	07_2021	1	25401441	43478	0.00171163517849243	0
**IgG3_4**	MARA	LDHA/B+CRMP1+STIP1	F	PND2	6	IgG3	07_2021	1	24936909	53930	0.00216265776965381	0
**FcRn4_1**	MARA	LDHA/B+CRMP1+STIP1	M	PND2	6	FcRn4	07_2021	1	25553039	52	2.03498300143478e-06	1505
**FcRn4_2**	MARA	LDHA/B+CRMP1+STIP1	M	PND2	6	FcRn4	07_2021	1	25674780	27	1.05161563214953e-06	2105
**FcRn4_3**	MARA	LDHA/B+CRMP1+STIP1	F	PND2	6	FcRn4	07_2021	1	27883579	27819	0.000997683977368902	0
**FcRn4_4**	MARA	LDHA/B+CRMP1+STIP1	F	PND2	6	FcRn4	07_2021	1	27621802	17334	0.000627547761004152	0
**RNA1_1**	MARA	LDHA/B+CRMP1+STIP1	M	PND2	6	RNA1	07_2021	1	26429304	50	1.89183945214751e-06	1699
**RNA1_2**	MARA	LDHA/B+CRMP1+STIP1	M	PND2	6	RNA1	07_2021	1	26260774	13	4.95034914050896e-07	1823
**RNA1_3**	MARA	LDHA/B+CRMP1+STIP1	F	PND2	6	RNA1	07_2021	1	29556955	35124	0.00118834974712382	0
**RNA1_4**	MARA	LDHA/B+CRMP1+STIP1	F	PND2	6	RNA1	07_2021	1	27002549	27711	0.0010262364490108	0
**RNA2_1**	MARA	LDHA/B+CRMP1+STIP1	M	PND2	6	RNA2	07_2021	1	32455969	29	8.93518230806789e-07	2317
**RNA2_2**	MARA	LDHA/B+CRMP1+STIP1	M	PND2	6	RNA2	07_2021	1	28060870	54	1.92438794663173e-06	1974
**RNA2_3**	MARA	LDHA/B+CRMP1+STIP1	F	PND2	6	RNA2	07_2021	1	30679013	29103	0.000948628953610731	0
**RNA2_4**	MARA	LDHA/B+CRMP1+STIP1	F	PND2	6	RNA2	07_2021	1	31094746	24846	0.000799041741649859	0

Supplementary Table 4. Summary statistics (mean, lower, and upper quartile) for PND2 brain cytokines (pg/ml), stratified by treatment group. Values are normalized for total protein.

	LDHA/B+CRMP1+ STIP1 n=30			CRMP1+CRMP2 n=24			CRMP1+GDA n=24			STIP1+NSE n=24			Control n=30		
Cytokine	Mean	Q1	Q3	Mean	Q1	Q3	Mean	Q1	Q3	Mean	Q1	Q3	Mean	Q1	Q3
G-CSF[a]	0.1	0.03	0.2	0.2	0.2	0.3	0.1	0.02	0.3	0.2	0.02	0.4	0.2	0.03	0.5
Eotaxin[b]	2.5	2.1	3.1	3.7	3.4	4.1	3.3	2.8	4.1	3.3	3.2	3.6	3.2	2.6	3.9
IL-1α	7.1	4.5	8.2	6.8	3.5	8.1	6.8	3.9	8.6	6.3	3.7	7.8	6.0	3.7	7.8
Leptin[c]	64.1	19.2	103	88.4	48.3	103	225	161	269	137	61.9	177	179	125	242
MIP-1α	24.3	21.8	26.2	22.5	20.5	25.1	23.8	20.7	26.0	20.4	17.5	23.9	27.8	24.8	30.4
IL-4	9.8	7.6	11.3	10.8	9.0	12.2	10.3	7.6	12.4	9.0	7.8	11.4	11.6	9.4	13.3
IL-1β	50.4	45.4	56.3	72.3	64.5	78.6	79.4	70.6	91.6	71.3	59.6	86.4	58.6	54.0	60.0
IL-2[d]	2.5	1.8	3.4	4.1	3.5	4.7	3.2	2.4	3.7	3.5	2.6	4.2	3.5	2.5	4.6
IL-6	224	205	243	289	254	315	273	227	308	263	229	294	263	241	279
EGF	1.5	0.8	2.0	0.8	0.3	0.9	0.5	0.3	0.6	0.8	0.3	1.1	0.3	0.2	0.5
IL-10[e]	2.9	2.3	3.3	2.8	1.9	3.7	1.5	0.03	2.3	1.8	1.1	2.5	4.1	3.7	4.4
IL-12p70[f]	2.3	0.5	4.3	3.4	2.6	4.2	2.9	2.9	2.9	6.5	2.9	9.8	3.1	0.5	4.5
IFNγ[g]	13.4	9.4	17.9	5.9	3.6	7.8	5.6	2.3	9.1	3.7	3.4	4.3	23.9	18.3	29.3
IL-5[h]	4.7	3.9	5.6	5.9	3.8	7.5	4.9	4.1	6.2	4.6	2.5	6.4	5.2	4.1	6.5
IL-17a[i]	2.5	2.0	2.9	2.5	2.0	2.9	1.8	1.2	2.4	2.0	1.3	2.6	2.9	2.4	3.4
IL-18	803	708	863	1063	950	1175	1163	1036	1285	1057	928	1215	953	834	1022
IP-10	78.5	69.8	85.6	69.2	62.1	71.4	68.8	57.1	80.7	66.8	54.4	73.4	85.9	79.1	95.0
GRO[j]	17.0	10.3	24.5	31.6	22.5	38.5	30.3	25.4	33.6	22.9	13.6	32.8	20.0	11.2	26.8
VEGF	52.8	50.1	57.5	42.2	36.9	45.9	45.5	38.0	51.1	41.8	37.4	46.3	53.6	48.5	59.1
Fractalkine	513	467	543	362	300	389	407	364	456	367	314	422	523	468	560
LIX[k]	6.8	1.8	11.9	5.4	3.0	7.2	3.0	0.9	4.3	5.0	2.4	7.0	3.9	2.8	5.0
TNFα[l]	2.2	0.7	3.3	1.4	0.2	2.3	1.4	0.1	2.3	1.5	0.1	2.8	1.9	0.4	3.1
Rantes	14.0	11.1	16.3	8.5	7.0	9.9	7.7	6.1	9.2	8.6	7.7	9.8	17.5	16.0	19.6

Abbreviations: LDHA/B: lactate dehydrogenase A/B; CRMP1: collapsin response mediator protein 1; CRMP2: collapsin response mediator protein 2; GDA: guanine deaminase; STIP1: stress-induced phosphoprotein 1; NSE: neuron-specific enolase.
Data Missing: [a]23 from LDHA/B+CRMP1+STIP1, 6 from CRMP1+CRMP2, 6 from CRMP1+GDA, 2 from STIP1+NSE, 29 from Control; [b]1 from LDHA/B+CRMP1+STIP1 and 1 from STIP1+NSE; [c]5 from LDHA/B+CRMP1+STIP1, 1 from Control; [d]2 from LDHA/B+CRMP1+STIP1; [e]3 from CRMP1+GDA and 3 from STIP1+NSE; [f]14 from LDHA/B+CRMP1+STIP1, 20 from CRMP1+CRMP2, 23 from CRMP1+GDA, 19 from STIP1+NSE, and 4 from Control; [g]4 from LDHA/B+CRMP1+STIP1, 10 from

CRMP1+CRMP2, 19 from CRMP1+GDA, and 17 from STIP1+NSE; [h]1 from CRMP1+CRMP2, 2 from CRMP1+GDA, and 5 from STIP1+NSE; [i]1 from STIP1+NSE; [j]13 from LDHA/B+CRMP1+STIP1, 12 from CRMP1+CRMP2, 13 from CRMP1+GDA, 14 from STIP1+NSE and 13 from Control; [k]30 from LDHA/B+CRMP1+STIP1, 4 from CRMP1+CRMP2, 14 from CRMP1+GDA, 9 from STIP1+NSE, and 30 from Control; [l]12 from LDHA/B+CRMP1+STIP1, 1 from CRMP1+CRMP2, 3 from CRMP1+GDA, 2 from STIP1+NSE, and 22 from Control.

Supplementary Table 5. Summary statistics (mean, lower, and upper quartile) for PND2 sera cytokines (pg/ml), stratified by treatment group.

	LDHA/B+CRMP1+ STIP1 n=38			CRMP1+CRMP2 n=24			CRMP1+GDA n=24			STIP1+NSE n=24			Control n=38		
Cytokine	Mean	Q1	Q3	Mean	Q1	Q3	Mean	Q1	Q3	Mean	Q1	Q3	Mean	Q1	Q3
G-CSF[a]	8.4	3.8	12.1	7.4	3.5	11.8	5.0	1.6	5.4	9.6	5.4	16.3	12.5	8.0	16.1
Eotaxin[b]	5.7	5.6	5.6	6.1	1.36	10.2	5.1	1.4	8.2	5.4	1.4	8.2	7.2	5.6	9.9
IL-1α[c]	123	28.6	126	109	43.71	138	61.1	29.5	102	80.8	29.5	70.4	62.2	8.1	67.3
Leptin	20331	4418	25167	15881	5225	22770	63855	34157	90582	33106	11336	49179	61502	37115	87427
MIP-1α	80.7	69.1	85.5	74.2	52.52	91.4	79.8	66.2	96.2	73.7	65.2	89.1	103	65.3	135
IL-1β[d]	5.9	2.0	8.1	10.0	3.58	10.6	6.7	3.6	8.7	9.7	4.2	13.7	7.6	4.1	10.0
IL-2[e]	29.2	8.4	22.7	9.3	3.97	9.8	34.3	9.8	50.9	19.3	12.7	25.9	15.3	4.8	22.7
EGF[f]	188	46.0	241	84.4	17.81	120	87.7	28.3	131	83.5	21.8	66.7	50.6	4.9	26.2
IL-10[g]	21.1	10.6	28.1	21.3	5.93	36.7	14.2	10.6	19.6	20.2	10.6	28.4	23.2	17.5	26.3
IL-12p70[h]	15.3	13.8	13.8	31.3	4.08	46.9	58.8	4.1	80.4	38.3	4.1	46.9	58.4	13.8	86.1
IFNγ[i]	51.2	33.9	69.3	72.4	22.42	69.3	40.1	2.5	47.0	64.2	30.8	103	44.7	6.9	62.0
IL-5[j]	63.7	39.5	82.1	82.4	72.16	92.7	83.8	68.7	98.3	78.6	61.2	94.1	75.2	60.2	93.3
IL-17a	31.2	26.4	36.1	31.2	26.02	35.1	31.5	26.7	33.7	28.3	25.3	33.0	38.8	34.9	45.9
IL-18	9203	5873	11711	8948	6827	10746	10115	7388	12196	10588	7124	11331	9627	6031	11101
MCP-1	1306	1041	1336	1334	1135	1461	1416	1203	1599	1695	1293	1718	1306	1130	1444
IP-10	406	329	432	415	325	473	391	331	440	414	346	454	476	430	527
VEGF	484	403	554	465	408	526	549	479	612	519	451	581	611	549	674
Fractalkine	153	134	168	159	142	175	168	150	182	169	147	192	172	152	190
LIX	4085	3379	4861	4270	2820	5383	4364	3583	5217	4090	3367	4883	4662	3545	5747
TNFα	2.4	1.8	2.8	5.2	4.44	5.9	4.3	3.2	5.2	5.4	4.4	6.1	3.2	2.6	3.9
Rantes	5890	3650	6620	4656	3.5	11.8	4950	3071	6770	4145	2287	5348	11857	3422	17389

Abbreviations: LDHA/B: lactate dehydrogenase A/B; CRMP1: collapsin response mediator protein 1; CRMP2: collapsin response mediator protein 2; GDA: guanine deaminase; STIP1: stress-induced phosphoprotein 1; NSE: neuron-specific enolase.

Data Missing: [a]21 from LDHA/B+CRMP1+STIP1, 16 from CRMP1+CRMP2, 12 from CRMP1+GDA, 18 from STIP1+NSE, and 16 from Control; [b]28 fom LDHA/B+CRMP1+STIP1, 2 from CRMP1+CRMP2, 11 from CRMP1+GDA, 6 from STIP1+NSE, and 23 from

Control; [c]13 from LDHA/B+CRMP1+STIP1,7 from CRMP1+CRMP2, 2 from CRMP1+GDA, 7 from STIP1+NSE, and 16 from Control; [d]3 from LDHA/B+CRMP1+STIP1, 3 from CRMP1+CRMP2, 6 from CRMP1+GDA, and 3 from STIP1+NSE, and 16 from Control; [e]26 from LDHA/B+CRMP1+STIP1, 18 from CRMP1+CRMP2, 21 from CRMP1+GDA, 20 from STIP1+NSE, and 9 from Control; [f]1 from CRMP1+CRMP2; [g]1 from LDHA/B+CRMP1+STIP1, 20 from CRMP1+CRMP2, 19 from CRMP1+GDA, and 15 from STIP1+NSE; [h]26 from LDHA/B+CRMP1+STIP1, 10 from CRMP1+CRMP2, 12 from CRMP1+GDA, 15 from STIP1+NSE,and 15 from Control; [i]35 from LDHA/B+CRMP1+STIP1,14 from CRMP1+CRMP2, 14 from CRMP1+GDA, and 16 from STIP1+NSE and 30 from Control; [j]1 from LDHA/B+CRMP1+STIP1 and 1 from Control.

Supplementary Table 6. Summary of the models evaluating differences between LDHA/B+CRMP1+STIP1 and Control PND2 Brain

**Cytokine/Chemokine**	**LDHA/B+CRMP1+STIP1 versus Control Difference**		
	**Estimate**	**SE**	**P-value**
Eotaxin[a]	-0.13	0.09	0.15
IL-1α	0.62	0.44	0.22
Leptin[b]	-1.52	2.94	0.63
MIP-1α	-0.15	0.36	0.70
IL-4	-0.06	0.16	0.72
IL-1β	-0.57	0.14	**0.0003**
IL-2[c, d]	-0.73	0.34	**0.04**
IL-6	-0.85	0.51	0.17
EGF	0.48	0.33	0.21
IL-10	-0.32	0.06	**<0.0001**
IL-12p70[e, f]	-2.26	0.84	**0.01**
IFNγ[g]	-1.51	0.48	0.09
IL-5	-0.08	0.22	0.76
IL-17a	-0.10	0.06	0.09
IL-18	-0.12	2.59	0.96
IP-10	-0.24	0.72	0.76
GRO[e, h]	0.58	0.65	0.37
VEGF	-0.02	0.27	0.94
Fractalkine	0.01	0.89	0.99
TNFα[e, i]	1.76	0.93	0.21
Rantes	-0.09	0.47	0.86

Abbreviations: LDHA/B, lactate dehydrogenase A/B; CRMP1, collapsin response mediator protein 1; STIP1, stress induced phosphoprotein 1; SE, standard error.
Bolded values represent significant comparisons with a p-value <0.05.
All variables were normalized for total protein before analyses. Variables were square-root transformed for analyses to improve normality with the exception of those notated.
Estimates are from linear mixed-effects models or logistic mixed-effects models fitted to variables transformed as described below, with fixed effects for group (LDHA/B+CRMP1+STIP1 or Control), adjusted for sex and litter, and including a random effect for dam-specific intercept.
[d]This variable was not transformed.
[e]These variables were treated as binary and analyzed using logistic mixed-effects models.
Data missing: [a]1 from LDHA/B+CRMP1+STIP1; [b]5 from LDHA/B+CRMP1+STIP1 and 1 from Control; [c]2 from LDHA/B+CRMP1+STIP1; [f]14 from LDHA/B+CRMP1+STIP1 and 4 from control; [g]4 from LDHA/B+CRMP1+STIP1; [h]13 from LDHA/B+CRMP1+STIP1 and 13 from Control; [i]12 from LDHA/B+CRMP1+STIP1 and 22 from Control.
The following variables were removed from the analysis due to missing data: G-CSF, GM-CSF, LIX, IL-13, MCP-1, and MIP-2.

Supplementary Table 7. Summary of the models evaluating differences between LDHA/B+CRMP1+STIP1 and Control PND2 Sera

**Cytokine/Chemokine**	**LDHA/B+CRMP1+STIP1 versus Control Difference**		
	**Estimate**	**SE**	**P-value**
G-CSF[a,b]	-1.63	2.71	0.58
Eotaxin[a, c]	-0.26	0.91	0.79
IL-1α[a, d]	0.10	1.25	0.94
Leptin[e]	-31.82	60.14	0.62
MIP-1α	0.01	0.28	0.97
IL-1β[e, f]	-0.24	0.61	0.72
IL-2[a, g]	-1.26	0.93	0.30
EGF	2.29	0.66	**0.03**
IL-10[h]	0.59	0.60	0.38
IL-12p70[a, i]	-1.09	0.51	**0.04**
IL-5 [j, k]	-4.64	9.54	0.65
IL-17a[j]	-6.00	5.92	0.36
IL-18	-0.13	0.27	0.64
IP-10	-0.10	0.16	0.57
MCP-1	0.15	0.24	0.55
VEGF[e]	-1.79	1.85	0.38
Fractalkine	-0.13	0.09	0.20
LIX	-0.02	0.18	0.91
TNFα[j]	-0.34	0.69	0.64
Rantes	0.04	0.55	0.94

Abbreviations: LDHA, lactate dehydrogenase A/B; CRMP1, collapsin response mediator protein 1; STIP1, stress induced phosphoprotein 1; SE, standard error.

Bolded values represent significant comparisons with a p-value <0.05.

Variables were natural log transformed to improve normality except for those notated.

Estimates are from linear mixed-effects models or logistic mixed-effects models fitted to variables transformed as described below, with fixed effects for group (LDHA/B+CRMP1+STIP1 or Control), adjusted for sex and litter, and including a random effect for dam-specific intercept.

[a] These variables were treated as binary and analyzed using logistic mixed-effects models.

[e] These variables were square-root transformed.

[j] These variables were not transformed.

Data missing: [b]21 from LDHA/B+CRMP1+STIP1, 16 from Control; [c]28 from LDHA/B+CRMP1+STIP1, 23 from Control; [d]13 from LDHA/B+CRMP1+STIP1, 16 from Control; [f]3 from LDHA/B+CRMP1+STIP1, 2 from Control; [g]26 from LDHA/B+CRMP1+STIP1, 9 from Control; [h]1 from LDHA/B+CRMP1+STIP1; [i] 26 from LDHA/B+CRMP1+STIP1, 15 from Control, [k]1 from LDHA/B+CRMP1+STIP1, 1 from Control.

The following variables were removed from analyses due to missing data: GM-CSF, IFNγ, IL-4, IL-6, IL-13, GRO, and MIP-2.

Supplementary Table 8. Summary of the models evaluating differences among CRMP1+CRMP2, STIP1+NSE, and CRMP1+GDA PND2 Brain

**Cytokine/ Chemokine**	**Overall Group**	**CRMP1+CRMP2 vs. CRMP1+GDA Difference**			**CRMP1+CRMP2 vs. STIP1+NSE Difference**			**CRMP1+GDA vs. STIP1+NSE Difference**		
	**P-value**	**Estimate**	**SE**	***P***	**Estimate**	**SE**	***P***	**Estimate**	**SE**	***P***
G-CSF[a, b]	0.16	0.35	0.71	0.63	-1.39	0.89	0.12	-1.74	0.92	0.06
Eotaxin[c]	0.23	0.18	0.11	0.11	0.04	0.10	0.68	-0.14	0.10	0.19
IL-1α	1.00	-0.003	0.29	0.99	-0.003	0.27	0.99	-0.001	0.27	0.99
Leptin	0.14	-4.68	2.26	0.06	-1.19	2.15	0.59	3.49	2.10	0.12
MIP-1α	0.53	-0.10	0.32	0.77	0.24	0.31	0.45	0.34	0.30	0.29
IL-4	0.36	0.11	0.23	0.64	0.32	0.21	0.18	0.20	0.21	0.37
IL-1β	0.47	-0.38	0.36	0.37	0.07	0.33	0.84	0.45	0.34	0.25
IL-2[d]	**0.03**	0.90	0.33	**0.01**	0.62	0.33	0.06	-0.28	0.34	0.41
IL-6	0.65	0.42	0.85	0.67	0.77	0.75	0.40	0.36	0.78	0.68
EGF	0.71	0.05	0.16	0.75	-0.07	0.15	0.63	-0.13	0.15	0.42
IL-10[e]	0.20	0.51	0.25	0.09	0.32	0.23	0.22	-0.19	0.24	0.45
IFNγ[a, f]	**0.04**	1.55	0.69	**0.03**	1.32	0.65	**0.05**	-0.23	0.72	0.75
IL-5[g]	0.68	0.20	0.27	0.52	0.22	0.24	0.45	0.01	0.25	0.96
IL-17a[h]	0.44	0.18	0.15	0.25	0.02	0.14	0.88	-0.16	0.14	0.28
IL-18	0.12	-1.50	0.87	0.09	0.19	0.85	0.82	1.69	0.88	0.06
IP-10	0.82	0.01	0.41	0.98	0.22	0.39	0.59	0.20	0.39	0.61
GRO[a, i]	0.84	0.24	0.65	0.71	0.36	0.63	0.57	0.12	0.65	0.85
VEGF	**0.04**	-0.33	0.15	**0.03**	0.04	0.14	0.76	0.36	0.15	**0.02**
Fractalkine	0.44	-1.23	0.86	0.34	-0.06	0.74	0.94	1.17	0.77	0.25
LIX[a, j]	0.24	1.83	0.96	0.11	1.11	0.91	0.26	-0.71	0.85	0.44
TNFα[k]	0.65	0.36	0.40	0.38	0.09	0.38	0.81	-0.27	0.37	0.49
Rantes	0.34	0.18	0.17	0.31	-0.06	0.16	0.70	-0.25	0.16	0.15

Abbreviations: CRMP1, collapsin response mediator protein 1; CRMP2, collapsin response mediator protein 2; STIP1, stress induced phosphoprotein 1; GDA, guanine deaminase; NSE, neuron specific enolase; SE, standard error.
Bolded values represent significant comparisons with a p-value <0.05.
All variables were normalized for total protein prior to analyses. Variables were square-root transformed to improve normality with the exception of those notated.
Estimates are from linear mixed-effects models or logistic mixed-effects models fitted to variables transformed as described below, with fixed effects for group (CRMP1+CRMP2, STIP1+NSE, or CRMP1+GDA), adjusted for sex and litter, and including a random effect for dam-specific intercept.
[a]These variables were treated as binary and analyzed using logistic mixed-effects models.
[d]This variable was not transformed.
Data missing: [b]6 from CRMP1+CRMP2, 2 from STIP1+NSE, 6 from CRMP1+GDA; [c]1 from STIP1+NSE; [e]3 from STIP1+NSE, 3 from CRMP1+GDA; [f]10 from CRMP1+CRMP2, 17 from STIP1+NSE, 19 from CRMP1+GDA; [g]1 from CRMP1+CRMP2, 5 from STIP1+NSE, 2 from CRMP1+GDA; [h]1 from STIP1+NSE; [i]12 from CRMP1+CRMP2, 14 from STIP1+NSE, 13 from CRMP1+GDA; [j]4 from CRMP1+CRMP2, 9 from STIP1+NSE, 14 from CRMP1+GDA; [k]1 from CRMP1+CRMP2, 2 from STIP1+NSE, 3 from CRMP1+GDA.
The following variables were removed from analyses due to missing data: GM-CSF, IL-12p70, IL-13, MCP-1, and MIP-2.

Supplementary Table 9. Summary of the models evaluating differences among CRMP1+CRMP2, STIP1+NSE, and CRMP1+GDA PND2 Sera

Cytokine/ Chemokine	Overall Group	CRMP1+CRMP2 vs. CRMP1+GDA Difference			CRMP1+CRMP2 vs. STIP1+NSE Difference			CRMP1+GDA vs. STIP1+NSE Difference		
	**P-value**	**Estimate**	**SE**	***P***	**Estimate**	**SE**	***P***	**Estimate**	**SE**	***P***
G-CSF[a, b]	0.58	-0.64	0.97	0.54	0.39	0.98	0.70	1.03	0.96	0.32
Eotaxin[a, c]	**0.03**	2.73	1.02	**0.01**	1.40	0.99	0.16	-1.32	0.79	0.10
IL-1α[a, d]	0.43	-3.40	2.55	0.20	-1.45	2.26	0.53	1.95	2.49	0.44
Leptin[e]	0.10	-117.37	51.1	0.04	-37.62	48.8	0.45	79.75	47.3	0.12
MIP-1α	0.99	-0.002	0.24	0.99	-0.02	0.23	0.94	-0.02	0.22	0.94
IL-1β[e, f]	0.53	0.26	0.59	0.67	-0.39	0.55	0.51	-0.64	0.56	0.28
IL-2[a, g]	0.51	0.93	0.82	0.26	0.55	0.75	0.47	-0.38	0.88	0.66
EGF[g]	0.90	-0.24	0.52	0.66	-0.15	0.48	0.76	0.09	0.50	0.86
IL-10[a, h]	0.72	-1.83	4.23	0.67	-3.32	4.08	0.43	-1.50	3.39	0.67
IL-12p70[a, i]	0.33	0.20	0.62	0.75	0.89	0.62	0.15	0.70	0.64	0.28
IFNγ[a, j]	0.53	-1.38	1.28	0.30	-0.30	1.24	0.81	1.08	1.21	0.39
IL-5[k]	0.93	1.39	10.1	0.90	3.55	9.33	0.72	2.16	9.59	0.83
IL-17a[k]	0.79	0.82	4.33	0.86	2.75	4.02	0.53	1.93	4.09	0.66
IL-18	0.59	-0.16	0.16	0.38	-0.13	0.15	0.41	0.03	0.15	0.87
IP-10	0.59	0.06	0.07	0.46	-0.02	0.07	0.80	-0.07	0.07	0.33
MCP-1	0.52	-0.06	0.23	0.79	-0.25	0.22	0.29	-0.18	0.21	0.41
VEGF[e]	0.21	-1.89	1.00	0.09	-0.65	0.94	0.51	1.24	0.94	0.22
Fractalkine	0.71	-0.04	0.06	0.50	-0.04	0.06	0.47	-0.0001	0.06	1.00
LIX	0.66	-0.20	0.24	0.42	-0.18	0.23	0.44	0.01	0.22	0.95
TNFα[k]	0.57	0.64	0.95	0.52	-0.33	0.90	0.72	-0.98	0.89	0.31
Rantes	0.95	0.11	0.40	0.78	0.12	0.38	0.76	0.002	0.37	1.00

Abbreviations: CRMP1, collapsin response mediator protein 1; CRMP2, collapsin response mediator protein 2; STIP1, stress induced phosphoprotein 1; GDA, guanine deaminase; NSE, neuron specific enolase; SE, standard error.

Bolded values represent significant comparisons with a p-value <0.05.

Variables were natural log transformed to improve normality except for those notated.

Estimates are from linear mixed-effects models or logistic mixed-effects models fitted to variables transformed as described below, with fixed effects for group (CRMP1+CRMP2, STIP1+NSE, or CRMP1+GDA), adjusted for sex and litter, and including a random effect for dam-specific intercept.

[a]These variables were treated as binary and analyzed using logistic mixed-effects models.

[e]These variables were square-root transformed.

[k]These variables were not transformed.

Data missing: [b]16 from CRMP1+CRMP2, 18 from STIP1+NSE, 12 from CRMP1+GDA; [c]2 from CRMP1+CRMP2, 6 from STIP1+NSE, 11 from CRMP1+GDA; [d]7 from CRMP1+CRMP2, 7 from STIP1+NSE, 2 from CRMP1+GDA; [f]3 from CRMP1+CRMP2, 3 from STIP1+NSE, 6 from CRMP1+GDA; [g]1 from CRMP1+CRMP2; [h]20 from CRMP1+CRMP2, 15 from STIP1+NSE, 19 from CRMP1+GDA; [i]10 from CRMP1+CRMP2, 15 from STIP1+NSE, 12 from CRMP1+GDA; [j]14 from CRMP1+CRMP2, 16 from STIP1+NSE, 14 from CRMP1+GDA.

The following variables were removed from analyses due to missing data: GM-CSF, IL-2, IL-4, IL-6, IL-13, GRO, and MIP-2.

Supplementary Table 10 - Control vs MARA SVA corrected DE genes

gene_id	gene_name	logFC	logCPM	LR	PValue	FDR
Tph1	Tph1	4.06227885847143	2.50795973480942	31.2980593815911	2.21300740800691e-08	0.000276116934299517
Grin3b	Grin3b	0.379704988141101	4.20064812856704	25.6729391593859	4.04459979755329e-07	0.00252321723896512
Creb1	Creb1	0.276672515266649	4.22612995879338	19.0257946337254	1.28963279617929e-05	0.0396671275284876
Sav1	Sav1	0.18909059936372	4.99551704474666	18.6386679817769	1.57983494985245e-05	0.0396671275284876
Mfrp	Mfrp	2.10429899639769	2.0949915325873	18.6269080466114	1.58960999350916e-05	0.0396671275284876
Polq	Polq	0.646216019453241	1.57945781164538	18.2568838005819	1.93027102942706e-05	0.0401399960569357
Tpsab1	Tpsab1	1.31401803396747	1.22419380637182	16.4746277433151	4.93054988626672e-05	0.0793160125839405
Mt1	Mt1	-0.374913403396475	2.43502279119147	16.4159378677832	5.08558227676144e-05	0.0793160125839405
Kmt5a	Kmt5a	0.15559427891718	5.17107660477408	15.5211709906727	8.15863742729892e-05	0.113105910200288
Ptbp3	Ptbp3	0.195115951091396	4.01677594696482	14.6688545622327	0.000128146252088646	0.117017483862454
Pag1	Pag1	0.245870259289077	3.14710387223561	14.5804829618231	0.000134298314357763	0.117017483862454
Tf	Tf	0.870735308619614	3.57086135328882	14.4521025334942	0.000143768274306005	0.117017483862454
Bach1	Bach1	0.13756590601881	5.54228809960513	14.288077006844	0.000156855271789504	0.117017483862454
Ramp	Ramp	0.205102422630022	4.77440910527257	14.2875216554744	0.000156901556859593	0.117017483862454
Chga	Chga	-0.141828615862243	6.9491239770925	14.174865466241	0.000166589910680661	0.117017483862454
Sox11	Sox11	0.252910510493653	7.93442708090919	14.0864178318593	0.000174600025458919	0.117017483862454
Vsnl1	Vsnl1	-0.175904078360979	7.36115736737418	14.0731617513886	0.000175834950296821	0.117017483862454
Pcsk9	Pcsk9	-0.862721839365857	0.471920188578388	14.06423059949	0.000176671925276593	0.117017483862454
Igf2bp3	Igf2bp3	0.407993748777943	3.1139154855475	14.0071436817775	0.000182117404113823	0.117017483862454
Chgb	Chgb	-0.130685321862144	7.35434097097437	13.9479887248713	0.000187938622652591	0.117017483862454
Tbccd1	Tbccd1	0.19687072158105	3.91730460123108	13.8581972570318	0.000197134918416887	0.117017483862454
Clic6	Clic6	1.5242782383307	3.29545508821838	13.7725375750575	0.000206330619569946	0.117017483862454
Bach2	Bach2	0.261226941618152	5.76845020116424	13.6397630767685	0.000221445376766321	0.120129930865799
Rnf165	Rnf165	0.159656185536467	5.62847522586128	13.2615424482084	0.000270906653527805	0.123007827582197
Zfp112	Zfp112	0.255432443392174	2.71524465229927	13.2103581669968	0.000279406163130356	0.123007827582197
Podxl	Podxl	0.196659418180065	6.22348596489426	13.1632519675375	0.000285477753212843	0.123007827582197
Abhd14b	Abhd14b	0.307540198034349	2.91244154627757	13.1479886722543	0.000287826509352916	0.123007827582197
Vxn	Vxn	-0.145794004348351	5.66854204913527	13.0225601120966	0.000307760791191188	0.137140406846159
Rai14	Rai14	0.180429655642448	4.87738512824919	12.8549436933935	0.000336588834654716	0.144814444482307
[illegible]	[illegible]	[illegible]	[illegible]	[illegible]	[illegible]	[illegible]

Dusp22	Dusp22	-0.130277775950847	3.04759028910124	6.61604025256783	0.0109106428852863	0.375636032358845
Mafb	Mafb	0.129484156902321	5.27804069735498	6.61069399033627	0.0109136814938124	0.375636032358845
Nfib	Nfib	0.190668045366798	6.09417960590382	6.60911350418101	0.0109145815733438	0.375636032358845
Nell1	Nell1	-0.145090927592955	5.86681264643493	6.60094234856053	0.0109192360711876	0.376242853852232
Syp	Syp	-0.12055480904107	9.22767525775413	6.5940177476986	0.0109232198299662	0.37639724812379
RGD1563	RGD156307	-0.0968747418076828	4.99036972902946	6.58972910146733	0.0109256877804126	0.37639724812379
Fnip2	Fnip2	0.10539755406433	5.39886777187907	6.58263176126381	0.0109297852739818	0.376792693943446
Nop53	Nop53	-0.103460580240164	6.6101726978087	6.5698237203516	0.0109372164097271	0.376894821226028
Hs6st2	Hs6st2	0.107873679529602	6.08091915362232	6.56082490834572	0.0109424802983375	0.376894821226028
Ciao3	Ciao3	-0.0892546222313226	4.38390395579986	6.55831312607425	0.0109439528214800	0.376894821226028
Rab3il1	Rab3il1	0.150870694802089	3.12670208023387	6.55684429249716	0.0109448149078743	0.376894821226028
Rai2	Rai2	0.129951027323455	3.75276803436137	6.55624304508713	0.0109451680019572	0.376894821226028
Cgref1	Cgref1	-0.137772210966343	5.80108749447993	6.53995543840045	0.0109547799116023	0.37778660832104
Armc7	Armc7	0.137864125471551	3.76057514701552	6.53485233597432	0.0109578100512062	0.37778660832104
Klk6	Klk6	1.11155474578649	-0.473347987883216	6.52768104672764	0.0109620833412894	0.37778660832104
Itgb4	Itgb4	0.302439487820517	2.9016158271775	6.52387514658786	0.0109643584213559	0.37778660832104
Helz	Helz	0.200368815435212	4.9539320190712	6.51893910476271	0.0109678147559250	0.37778660832104
Nfatc3	Nfatc3	0.135491459605885	4.09462491585009	6.51452566425647	0.0109699685733021	0.37778660832104
Fbn2	Fbn2	0.260051777525774	5.19251604736808	6.50845552705184	0.0109736271855517	0.37778660832104
Cma1	Cma1	0.869781301730812	2.05741973094236	6.50190888396421	0.0109775873835244	0.37778660832104
Tp53il1	Tp53il1	0.087298279387301	8.09985323491696	6.5018617690575	0.0109776158985326	0.37778660832104
Per2	Per2	0.170215701322623	4.90353361295768	6.49759887576627	0.0109802021643937	0.37778660832104
Ubxn7	Ubxn7	0.19893625436416	2.66348940822594	6.47695892687445	0.0109928180068955	0.37778660832104
Hmox2	Hmox2	-0.0971462684377383	5.38331417241765	6.47544709469713	0.0109937479659383	0.37778660832104
Btbd7	Btbd7	0.128120612019772	5.11608214422605	6.47191022388824	0.0109959267405796	0.37778660832104
Shprh	Shprh	0.129791296053406	6.59125685576308	6.47118532876685	0.0109963728214783	0.37778660832104
Mob1a	Mob1a	0.110742399945256	4.91427595394638	6.46281490826388	0.0110093037248525	0.37778660832104
Rgs20	Rgs20	-0.150878010046285	4.21926536884312	6.46266063012675	0.0110102585755169	0.37778660832104
Spcs3	Spcs3	0.117068039195183	4.39672894825249	6.46288096154292	0.0110150994151042	0.37778660832104
Sucla2	Sucla2	-0.0943982406257226	6.50385711367308	6.4580165429187	0.0110452988432231	0.37778660832104
Nefl	Nefl	-0.169117937774405	7.53123934589494	6.45223267628967	0.0110750650853095	0.37778660832104
Actl6a	Actl6a	0.108552887567813	4.12759752277944	6.45168994418986	0.0110846863444478	0.37778660832104
Pla2g5	Pla2g5	0.672838954900814	0.82782800719242	6.44727560629934	0.0111122613812472	0.37778660832104
Ca8	Ca8	-0.207006855629725	3.68739345821895	6.43560390749471	0.0111855122271201	0.378882904012712
Syt3	Syt3	-0.0972436413834007	6.03116406630749	6.43341161647253	0.0111993260864543	0.378882904012712
Cnot6	Cnot6	0.121307779164524	6.36622980570111	6.40586491999805	0.0113744003568914	0.379005045345909
Zcchc4	Zcchc4	0.179600181185294	2.78228238881971	6.40345248566637	0.0113898786757491	0.379005045345909
Zfp687	Zfp687	0.115881900779453	4.55479242823554	6.40053601638765	0.0114085912816374	0.379005045345909
Serpinb1a	Serpinb1a	0.495619867729393	1.9690365066213	6.39867253991106	0.0114205726112911	0.379005045345909
Sstr2	Sstr2	0.163593294706417	4.64630875283768	6.39323263265975	0.0114556234215582	0.379005045345909
Tspan7	Tspan7	-0.10687611990546	8.72151985027595	6.3898152728726	0.0114775972176991	0.379005045345909
Arhgap39	Arhgap39	0.0775495695525002	6.85207835206264	6.38504554354812	0.0115085808278775	0.379005045345909
Trappc2	Trappc2	-0.0957777673897768	5.7742550032384	6.37788514540603	0.011555104047087	0.379005045345909
Zbtb39	Zbtb39	0.110518009163438	4.94460604120737	6.37327094500919	0.0115851861149361	0.379005045345909
Mtif3	Mtif3	0.108023030456526	3.62376519776569	6.37011585845467	0.0116058018198381	0.379005045345909
Hddc3	Hddc3	-0.156520595796069	4.01128080672362	6.36578644893757	0.0116341520158515	0.379005045345909
Syt10	Syt10	-0.278279444779637	0.91505151731905	6.36518559601191	0.0116380921622819	0.379005045345909
Fzd1	Fzd1	0.123934973359795	5.5782173293027	6.3613624880642	0.0116631947353814	0.379005045345909
Hapln1	Hapln1	-0.340183395706045	2.23963870781145	6.35723894262978	0.0116903455937342	0.379005045345909
Rasd1	Rasd1	-0.244468499047293	1.97426322304082	6.35313292838917	0.0117174190722736	0.379005045345909
Rfng	Rfng	-0.083814254533258	6.0718840386492	6.35287492341214	0.0117191623896553	0.379005045345909
N4bp2l2	N4bp2l2	0.0974542842126948	4.76712564453598	6.35194762940902	0.0117252502607615	0.379005045345909
Sesn1	Sesn1	-0.2752089990602	2.48699143462026	6.34492097373906	0.0117717852703084	0.379031643541392
Ywhah	Ywhah	-0.0905449601215501	9.67640022879687	6.34242140858979	0.0117883848074921	0.379031643541392
Rwdd2a	Rwdd2a	-0.200116780266423	2.4449763226963	6.33808868871191	0.0118172084104824	0.379031643541392
Rasa1	Rasa1	0.103481885457318	6.70441212173355	6.33223876129352	0.0118562558553738	0.379308865403845
Lgi4	Lgi4	-0.398284371315503	2.02219982820294	6.32298884020339	0.0119182577229415	0.380317395419799
Tcf12	Tcf12	0.110978605823865	5.42591767559031	6.31071479822822	0.012001344650764	0.381882227001664
Tcf4	Tcf4	0.181254203057358	6.86733513041708	6.30273065889419	0.0120552132739055	0.382375530033582
Sat1	Sat1	-0.108803497107935	4.9839784857802	6.29656213609228	0.0120972359090772	0.382375530033582
Lyve1	Lyve1	0.548273437146667	2.50287637436842	6.29537498239024	0.0121053405757205	0.382375530033582
Rabep2	Rabep2	0.119573479263254	4.21544295062176	6.28424047047287	0.0121816272976067	0.383813544929896
Ctdspl	Ctdspl	0.144947332715637	4.17362352217483	6.2666209386701	0.0123033557656074	0.386607043402093
Pygo1	Pygo1	0.154913489769694	3.3611724776988	6.26246357211264	0.0123322586196227	0.386607043402093
Ubn1	Ubn1	0.0898814057933727	5.88229712365889	6.24568040179899	0.0124496547094481	0.387375200708802
Kcnq1	Kcnq1	0.371887890574572	-0.0153767110380226	6.24165123827456	0.0124780086475104	0.387375200708802
Clstn3	Clstn3	-0.0985883014366962	9.34943988785808	6.23544972490143	0.0125217790162674	0.387375200708802
Gria4	Gria4	-0.116748689183493	6.72477871458588	6.21908229816622	0.0126380602948304	0.387375200708802
Arpc4	Arpc4	-0.0843869800159933	7.5694139342677	6.21694604640583	0.0126533188093749	0.387375200708802
Gipc2	Gipc2	-0.337716251749638	0.472844480540808	6.21106718301434	0.0126954073052829	0.387375200708802
Tigd5	Tigd5	0.102374564860137	3.78685912177666	6.20583350053434	0.0127344365235922	0.387375200708802
Mavs	Mavs	0.135983703035277	3.60754016747649	6.20206451317431	0.0127601390882133	0.387375200708802
Dab1	Dab1	0.120607722149601	6.34529189867894	6.20180801001467	0.0127619882814797	0.387375200708802
Pde5a	Pde5a	0.145723016821962	3.87888892735071	6.19913329768248	0.0127812884693119	0.387375200708802
Naa15	Naa15	0.085378798566168	7.026058704521	6.19879361118947	0.0127837417736172	0.387375200708802
St8sia2	St8sia2	0.108118091167786	6.79296947464825	6.19209513803226	0.0128322176816255	0.387375200708802
Klhl14	Klhl14	0.212038691171603	4.02911285145508	6.18673128148709	0.0128711706495049	0.387375200708802
Msln	Msln	0.69087451938083	0.0678898861808414	6.17778899634738	0.0129365218743991	0.387375200708802
Zfp799	Zfp799	0.113810945060176	4.73004165784833	6.17259267400635	0.0129744330274238	0.387375200708802
Faim2	Faim2	-0.0846571737683212	6.20643719182305	6.1654739063108	0.0130267470621226	0.387375200708802
Fes	Fes	0.300854552038527	0.985737706429137	6.16016833772829	0.0130659869026062	0.387375200708802
Hexim2	Hexim2	-0.14414103568827	3.45743887900175	6.15920606257684	0.0130729873245679	0.387375200708802
Rnf38	Rnf38	0.112726445452123	5.22297224897305	6.15785957491349	0.0130829428881949	0.387375200708802
Thnsl1	Thnsl1	0.123843673096729	3.79015686537199	6.15432048896363	0.0131096470971701	0.387375200708802
Pde7a	Pde7a	0.119806649566389	5.43504545811372	6.1515137593739	0.0131299671040328	0.387375200708802
Gpr37l1	Gpr37l1	-0.222889872162081	4.77619436417893	6.14839424711293	0.0131532215807595	0.387375200708802
Prelp	Prelp	0.447250610770829	3.07200306389271	6.14680541325384	0.0131724159494706	0.387375200708802
Taf13	Taf13	-0.0995046030488356	4.89599260941768	6.14350885128803	0.0131895335976973	0.387375200708802
Eppin	Eppin	0.259924317217569	1.48808906652008	6.14121315369494	0.0132066676609118	0.387375200708802
Tmem150	Tmem150b	-0.195713622369485	3.29884485896819	6.14101095456451	0.0132081778811984	0.387375200708802
Hoxc4	Hoxc4	-2.35422046442037	0.330429119056374	6.1388162543988	0.0132245816096171	0.387375200708802
Arl5b	Arl5b	0.126466675035027	4.99763460001695	6.13444249625872	0.0132573342261097	0.387375200708802
Mrc1	Mrc1	0.557804059088673	2.17623718257449	6.1330091140245	0.0132680862037527	0.387375200708802
Slco5a1	Slco5a1	0.143680288384506	4.48416651883671	6.13033438058456	0.013288173687855	0.387375200708802
Isg15	Isg15	0.262333517400098	0.762026717850142	6.10729295214829	0.0134625171736227	0.390841655245568
Neu4	Neu4	-0.222592959832369	2.54821436750219	6.10464882325806	0.0134826739294749	0.390841655245568
Rnf111	Rnf111	0.0871280224768587	5.89395614259505	6.10150971061477	0.0135066442540749	0.390841655245568
Sertad2	Sertad2	0.145162875703722	3.04582942001817	6.08702147460528	0.0136178465240502	0.390841655245568
RGD1561	RGD15617	0.950452691756413	-0.791442274189595	6.08426629645491	0.0136390967705809	0.390841655245568
Ptrhd1	Ptrhd1	-0.174561879632132	3.00689687110043	6.07601637038905	0.0137029434578515	0.390841655245568
Satb2	Satb2	0.249841171104316	8.2176950083198	6.07433817835586	0.0137159680543518	0.390841655245568
Dmtf1	Dmtf1	0.107164204569368	6.5467115899697	6.07385125008355	0.0137197495305449	0.390841655245568
Pou3f2	Pou3f2	0.136713118904897	6.79315212420605	6.06725296645382	0.0137711052338248	0.390841655245568
Cpe	Cpe	-0.0912310134959354	10.0409459405147	6.06643926879379	0.0137774434300333	0.390841655245568
Phc3	Phc3	0.21320490410206	4.61310830807953	6.06555604433954	0.0137843349557643	0.390841655245568
Rnd2	Rnd2	-0.123403804152545	6.13590602440216	6.06527694102253	0.0137865134493909	0.390841655245568
Usp14	Usp14	-0.0635520103445157	7.64280551953949	6.06002789427223	0.0138275501022831	0.391216196431262
Rab27a	Rab27a	0.210404144366295	2.18612412934868	6.04206507727774	0.0139698935005363	0.392445768104767
Cdk12	Cdk12	0.12773334521592	5.26201332247922	6.04066445889492	0.0139800215620061	0.392445768104767
Gtf3c4	Gtf3c4	0.0881718324696067	5.81380052028336	6.03843129054122	0.0139977186647082	0.392445768104767
Bicra	Bicra	0.153310148901832	6.21684902522447	6.03034310077787	0.0140619996599084	0.392445768104767
Eno2	Eno2	-0.0935352727909706	8.30324740799687	6.02862000390703	0.0140757235797955	0.392445768104767
Phf2	Phf2	0.0871763893527114	6.81710876292514	6.027719025224	0.014082920123025	0.392445768104767
Rps6kl1	Rps6kl1	-0.0907191272023922	6.42300053809664	6.02199438301263	0.0141286716980354	0.392445768104767
Galnt9	Galnt9	-0.113708295719999	5.2230582917432	6.02132765968875	0.0141340100903691	0.392445768104767
Rfx3	Rfx3	0.156071869185249	4.97402754025038	6.01260423494571	0.0142040498008538	0.392445768104767
Gpr85	Gpr85	0.113931655980993	7.42312060219838	6.01222359094976	0.0142071135358682	0.392445768104767
Lama1	Lama1	0.230034644116185	4.15497380361687	6.00726920566806	0.0142470589633485	0.392445768104767
Rrn3	Rrn3	0.0609379521673058	6.24052011168355	6.00709669528018	0.0142484525135417	0.392445768104767
Rap2a	Rap2a	0.111281425408825	4.12388323667229	5.9838797564172	0.0144372091767875	0.395807792380343
Bnip3	Bnip3	-0.0905847991780171	6.26747710986782	5.97909677164333	0.0144764141273131	0.395807792380343
Sdf2l1	Sdf2l1	0.257772238526123	2.8394672655628	5.97757304564436	0.0144889267372811	0.395807792380343
Cdk19	Cdk19	0.114957919496884	7.93651162275723	5.97605498250934	0.0145010708964348	0.395807792380343
Nt5m	Nt5m	-0.159431402312772	2.88291606005459	5.96972725304374	0.0145617872015078	0.396897423932123
Mdk	Mdk	0.271352461235889	4.94728924564817	5.96211999964417	0.0146081680514184	0.397093827629829
Prkx	Prkx	0.105474579064024	5.97673051439203	5.95133528655622	0.0147061423854138	0.398888127266973
Ddah2	Ddah2	0.135424933868615	7.28044095394817	5.9436299679103	0.0147789496174782	0.399561976817656
Glul	Glul	-0.109289807295382	8.72937829865382	5.94071286003317	0.0147950335248663	0.399561976817656
Csnk2b	Csnk2b	-0.0770777024457354	6.84409959143782	5.93428101715849	0.0148491255175438	0.40015667188422
Cmpk1	Cmpk1	-0.0701577229685243	7.45973101321109	5.92302225141871	0.0149443028116362	0.40051760487
Tradd	Tradd	0.277852043264861	1.385053724983	5.91619416385884	0.0150024159017507	0.40051760487
Grap	Grap	0.250625882350399	1.72773043800451	5.91398690363972	0.0150213082657394	0.40051760487
Spon2	Spon2	0.252328584309941	2.12513777552988	5.91231191318046	0.0150354269944555	0.40051760487
Pdlim2	Pdlim2	0.501290476741787	0.461159805780923	5.90970938233184	0.0150576557664524	0.40051760487
Vamp7	Vamp7	0.10010282994582	4.1169074345331	5.89947274722706	0.0151454179444853	0.40051760487
Ctnnd1	Ctnnd1	0.0900280394288038	7.44184601787558	5.89768594287554	0.0151607907504196	0.40051760487
Slc6a3	Slc6a3	0.311998552141742	1.17972093159191	5.88104887672717	0.0152180510186598	0.40051760487
Tal2	Tal2	0.289888016242283	2.19817884723481	5.87131898367754	0.0153895171273366	0.40051760487
Pigv	Pigv	0.13441854119106	3.25101770426206	5.86981955244335	0.0154026306268918	0.40051760487
Mycn	Mycn	0.105007864957801	6.75409157442087	5.8692839686402	0.0154073174446165	0.40051760487
Ndufa5	Ndufa5	-0.106700497874227	6.20432801258769	5.864646594319	0.0154479600193166	0.40051760487
Tpm1	Tpm1	-0.0579739184797167	7.41462421557015	5.86370320875802	0.015456241382597	0.40051760487
Klhl9	Klhl9	0.0712587064460815	7.65546374825574	5.85897036823589	0.015497857410995	0.40051760487
Gfra1	Gfra1	-0.501236636242555	-0.288796361215567	5.85831516425232	0.0155036277320383	0.40051760487
Scg2	Scg2	-0.159634555736498	7.00362951140575	5.8577347691344	0.0155087410670867	0.40051760487
Sgk3	Sgk3	0.205442476261891	2.98727475643978	5.83782521462432	0.0156852023241639	0.40051760487
Abhd14a	Abhd14a	0.297102842676773	3.18001875055776	5.83636499601379	0.0156982282838897	0.40051760487
Gclm	Gclm	0.0991156302762197	4.410324375882	5.83167829488981	0.0157401003106285	0.40051760487
Mindy3	Mindy3	-0.0762374685930887	5.92509641356304	5.83108258521559	0.015745431090962	0.40051760487
Nuak1	Nuak1	0.110341206095861	5.45441771141513	5.83027687106824	0.0157526440838512	0.40051760487
Mgst3	Mgst3	-0.141705840715767	6.98896432024462	5.82944984491513	0.0157600534085544	0.40051760487
Lcn2	Lcn2	0.508708790345702	0.182144953119454	5.82615813538096	0.0157895684552792	0.40051760487
Plin2	Plin2	0.148537878643524	5.64407442325719	5.82026017226499	0.0158426013748893	0.40051760487
Rn45s	Rn45s	0.545258458905193	3.10433918736794	5.81974258104638	0.0158472640824955	0.40051760487
Agfg1	Agfg1	0.0600496665215903	6.93165167582349	5.80613074479856	0.015970394975445	0.40051760487
Gsta1	Gsta1	0.563798800529843	1.86516998829745	5.79994257040049	0.0160266980099966	0.40051760487
Ccng2	Ccng2	0.109469497107945	6.30854190113914	5.79792171465297	0.0160452290262577	0.40051760487
Napb	Napb	-0.130578270513608	5.88889408756919	5.79723762245685	0.0160513721701027	0.40051760487
Smtnl2	Smtnl2	-0.18062634687648	2.58197789167003	5.79487718906819	0.0160729375829624	0.40051760487
G3bp1	G3bp1	0.0565791407368795	7.71732820047604	5.78953268004215	0.0161218742949187	0.40051760487
Slc1a5	Slc1a5	0.266703889454639	2.70987709800138	5.78239477361544	0.0161783572724469	0.40051760487
Acp1	Acp1	-0.0897622120538478	5.32500186144471	5.7767627608358	0.0162394242186735	0.40051760487
Sox4	Sox4	0.0945000219658542	9.53675890198692	5.77405688552946	0.0162644457081591	0.40051760487
Mis12	Mis12	0.111516946607675	4.02147670041823	5.77271287966817	0.0162768886017376	0.40051760487
Mdga1	Mdga1	0.150170178987317	6.76178207325545	5.76584847125737	0.0163396630970671	0.40051760487
N4bp2l1	N4bp2l1	-0.194585074682613	2.88447562733557	5.76110081963672	0.0163848032582784	0.40051760487
Fignl1	Fignl1	0.234756115805704	1.23311807393152	5.75834302110866	0.0164105407284564	0.40051760487
Stt3a	Stt3a	0.0889610908293012	5.95702937071717	5.75489441825557	0.0164427837491018	0.40051760487
Ccnd3	Ccnd3	0.152623747335575	5.09913040989457	5.75431582361257	0.0164481986733374	0.40051760487
Sh2d3c	Sh2d3c	-0.0751510645592547	5.58823801769817	5.75208098252217	0.0164691365961842	0.40051760487
Rcsd1	Rcsd1	0.218228567213829	2.75049656213318	5.74553908866648	0.0165305816063839	0.40051760487
Hcn4	Hcn4	0.0767794778114808	6.00001230324133	5.74421556283182	0.0165430416011422	0.40051760487
Orai1	Orai1	0.166700629333701	3.54067603810549	5.74009479867164	0.0165819920528032	0.40051760487
Itpr1	Itpr1	0.203609669721033	6.3700640603748	5.73763783129376	0.0166051097839401	0.40051760487
Cipc	Cipc	0.0623972650693014	6.43431892011797	5.73708715727256	0.0166103188955121	0.40051760487
Cluap1	Cluap1	-0.13093587167302	5.66601801211604	5.73301000357422	0.0166489223554719	0.40051760487
Zfp398	Zfp398	0.133522789438581	4.35983909425984	5.72830084912623	0.016693627240478	0.40051760487
Ankrd13d	Ankrd13d	-0.0739167312122056	6.48096991324257	5.72541594790199	0.0167210752808257	0.40051760487
Nme4	Nme4	0.253063903932278	1.4815927642089	5.71897581112265	0.0167825172352775	0.40051760487
Pak6	Pak6	-0.101228537430865	7.59719179267442	5.71629740788835	0.0168081389847582	0.40051760487
Napg	Napg	-0.0682553158001254	6.60508468832824	5.70068654672517	0.016958278236701	0.40051760487
Bicral	Bicral	0.165453271081613	5.40239805405306	5.69860132813208	0.0169784375496384	0.40051760487
Obi1	Obi1	0.0899039424426942	4.72458749739095	5.69772406857049	0.0169869261462205	0.40051760487
Angel2	Angel2	0.0657651278121462	5.64158342902318	5.69720490320554	0.0169919517262972	0.40051760487
Rbm3	Rbm3	-0.0961121987983807	7.92876795598985	5.69023477092654	0.0170304338148893	0.40051760487
Bub1	Bub1	0.153323159685425	3.64163125748397	5.68054605889279	0.017056546278005	0.40051760487
Ddx24	Ddx24	-0.0550562962800858	7.10815094884637	5.68044525250836	0.0170575261009943	0.40051760487
Ube2b	Ube2b	-0.0673073490852144	6.91323325158535	5.67994292466124	0.0170624095182676	0.40051760487
Osbpl11	Osbpl11	0.078375717689201	6.10154191892587	5.68653023949319	0.0170955234220211	0.40051760487
Pds5a	Pds5a	0.101252835018629	6.22818579002956	5.6830592368628	0.0171294742185228	0.40051760487
Tulp4	Tulp4	0.109704509434176	7.97128093625028	5.68292268328985	0.0171308084432584	0.40051760487
Efnb1	Efnb1	0.0955549604428987	6.01119152547989	5.68210372246552	0.0171388060570839	0.40051760487
Htra3	Htra3	0.184556240310671	3.75959797200337	5.68154259704525	0.0171442880024925	0.40051760487
Wnt9b	Wnt9b	-0.470823731576542	1.37425428107037	5.68140618837805	0.0171456209253511	0.40051760487
Ugt8	Ugt8	-0.190478454016171	3.52808526744596	5.67885731096476	0.0171705470587449	0.40051760487
Klhl6	Klhl6	0.365475524562674	0.281505604485135	5.67805378432954	0.0171784127026763	0.40051760487
Plvap	Plvap	0.418243292156417	1.85426335006229	5.67544714783293	0.0172039544133025	0.40051760487
Amz1	Amz1	-0.115136880257121	4.50376759557526	5.67366563401942	0.0172214323512768	0.40051760487
Eps8l2	Eps8l2	0.363368352116834	1.49719828853561	5.67299250675496	0.0172280327647531	0.40051760487
Stmn4	Stmn4	-0.0825016879960777	8.89692410889796	5.67221520160358	0.0172356777797768	0.40051760487
Plscr3	Plscr3	0.0960432247044938	5.09547118880137	5.67077547462501	0.0172498289341204	0.40051760487
Sdha	Sdha	-0.072299706364742	7.3235906584823	5.66912361436996	0.0172660799266437	0.40051760487
Syngr2	Syngr2	0.210119457230812	2.53473067725491	5.66900526767984	0.0172672448270126	0.40051760487
Thbd	Thbd	0.270681208823363	3.21965217471259	5.66683070921666	0.0172886637008654	0.40051760487
Mitf	Mitf	0.355306453732505	1.85465786399963	5.66545241822061	0.0173021550673551	0.40051760487
Sox12	Sox12	0.104131026189749	7.33475991185423	5.65226481028571	0.0174229049698565	0.401548174776737
Hipk1	Hipk1	0.0832726760220243	7.28291423575199	5.65214433480065	0.017434040607211	0.401548174776737
Itpripl1	Itpripl1	0.254120423327641	2.40126843645148	5.65122081173421	0.0174432242861991	0.401548174776737
Lats1	Lats1	0.102905349810447	6.81237855068461	5.64557179391197	0.0174995086520059	0.401561973275561
Sufu	Sufu	0.132569484730021	3.90980736725917	5.64019492778654	0.0175532655422644	0.401561973275561
Srsf5	Srsf5	-0.114285029682903	7.7214596870384	5.63947054065306	0.0175605092578722	0.401561973275561
Gpt2	Gpt2	0.0898354722957265	5.88433823093525	5.63826777968983	0.0175725605083799	0.401561973275561
Lynx1	Lynx1	-0.248557395375376	4.90763730862225	5.63098647762466	0.0176456985177692	0.40249612505705
Srf	Srf	0.0733082751940927	6.19386149012672	5.62429500509028	0.0177131889511145	0.403298292012876
Prkd3	Prkd3	0.110493127852692	5.54216527115948	5.60574657759496	0.0178914516293785	0.406333452540238
Pign	Pign	0.193978909703359	3.72333608278364	5.60477163850854	0.0179116293097003	0.406333452540238
Snx13	Snx13	0.0845426842315716	5.44247551492773	5.58558626864493	0.018005784215865	0.406550278808699
Trio	Trio	0.115167229138767	8.51929505892958	5.58895500179232	0.0180740757125923	0.406550278808699
Slc15a3	Slc15a3	-0.143365329004015	4.2624626204462	5.58431362974981	0.0181220335110588	0.406550278808699
Pip5k1b	Pip5k1b	0.195128805857067	3.27016129848654	5.58006546447297	0.0181660435994886	0.406550278808699
Ddx6	Ddx6	0.108300143198469	7.23012659244688	5.57903378002819	0.0181760217582496	0.406550278808699
Zfp316	Zfp316	0.0670288180455656	6.13570469346763	5.57874720420026	0.0181797229002905	0.406550278808699
Igf2bp2	Igf2bp2	0.163306738762289	4.1041955234352	5.57663814886529	0.0181908550424194	0.406550278808699
Mcc	Mcc	0.183380193875517	2.81379799528052	5.57652200550829	0.0182028374951865	0.406550278808699
Gpr139	Gpr139	0.202146247886673	2.07236482284525	5.57466471947829	0.0182221535584488	0.406550278808699
Cldn1	Cldn1	0.506928686945372	2.56497719427573	5.57063793393047	0.0182641054818539	0.406550278808699
Tp53	Tp53	0.0980070233126666	5.80271316344116	5.56568769757217	0.0183158148234705	0.406550278808699
Adamtsl4	Adamtsl4	0.302061978463298	1.77445832881991	5.56080431192488	0.0183669740055373	0.406550278808699
Dctn6	Dctn6	-0.0870682671892389	6.37821084321534	5.56030701055784	0.0183721920924004	0.406550278808699
Tigd2	Tigd2	-0.120196670482431	3.17149692478559	5.55948994468299	0.0183912712333823	0.406550278808699
Erich1	Erich1	0.138997427173207	3.29416147298439	5.55671318913421	0.0184099469044574	0.406550278808699
Maf	Maf	0.121231281390087	4.99697037949037	5.53803929545839	0.0186074191164582	0.410185203001889
Lrrc27	Lrrc27	0.238062401271158	2.01287091175005	5.53427252120098	0.0186475163680303	0.41034403422644
Fndc1	Fndc1	0.246412184966576	2.66236665424972	5.52979797141379	0.0186952638960257	0.410670425989564
RGD1559	RGD15598	0.0895178963072023	7.29085830155622	5.51698808575634	0.0188142558462301	0.412557944100802
Anxa2	Anxa2	0.306800833596415	4.61074976788476	5.50915893245146	0.0189171416757869	0.413197095983956
Abt1	Abt1	0.639276917640369	-0.016032519479582	5.50605245814717	0.0189507722138426	0.413197095983956
Kat14	Kat14	0.103097910149132	4.31878392637032	5.50261494290496	0.0189880588055648	0.413197095983956
Pcdhga2	Pcdhga2	0.213991089931888	4.33882706669777	5.50242704659757	0.0189900960592193	0.413197095983956
Papola	Papola	0.0761042857412009	7.45585723773339	5.50068856821802	0.0190089871840018	0.413197095983956
Elf2	Elf2	0.0889421247768477	5.3915719890392	5.49708632080965	0.0190481870968118	0.413329096262032
Fbp2	Fbp2	-0.406308477074458	-0.288862930317695	5.48713114304145	0.0191569526611763	0.413921204002217
Nr1d2	Nr1d2	-0.113219164034603	4.93754388422801	5.48506858916434	0.0191795674268347	0.413921204002217
Kif5b	Kif5b	0.0751851488098372	6.89020097890563	5.48283616359502	0.0191930934597361	0.413921204002217
Rfc4	Rfc4	-0.100890025582601	4.50587973055055	5.48246322885147	0.0192081732080856	0.413921204002217
Tob1	Tob1	0.111266490804583	4.55547373208171	5.46814804087839	0.0193661367212544	0.416370014435367
Nedd8	Nedd8	0.100714723303668	7.56622010641626	5.46037468868106	0.0194524750823928	0.416370014435367
Mn1	Mn1	0.103917902322813	7.63113521859347	5.45826011010112	0.0194760306041602	0.416370014435367
Cxcl12	Cxcl12	0.262553456996881	5.89776772671204	5.45484756683719	0.0195141065933209	0.416370014435367
Aqp4	Aqp4	-0.157025534196137	7.05628899193622	5.45305310211442	0.0195341585841585	0.416370014435367
Nnt	Nnt	0.120319140257691	5.22890070825852	5.44937552563365	0.0195753226686346	0.416370014435367
Cavin1	Cavin1	0.289258904681268	4.30050118855768	5.44590203333084	0.0196142840058148	0.416370014435367
Rbms1	Rbms1	0.139283427891718	4.02697372799907	5.44408864497591	0.0196346561358255	0.416370014435367
Vps26c	Vps26c	0.0877372416287826	5.29498904003328	5.44149625421651	0.0196637840286738	0.416370014435367
Maip1	Maip1	-0.107476347995486	4.51357049388697	5.44041969391484	0.0196759412387181	0.416370014435367
Llgl2	Llgl2	0.490678551246827	0.451509434315716	5.43653244403704	0.0197197606593394	0.416370014435367
Yipf2	Yipf2	0.109274252752836	3.45249987322347	5.43631256175342	0.0197222632468784	0.416370014435367
Dlgap3	Dlgap3	-0.112912393393507	7.94814256352753	5.41968165234572	0.0198109900154767	0.419154537437266
Tmem169	Tmem169	-0.0717147792239489	5.4053000940518	5.41893895449056	0.0198221988876706	0.419154537437266
Ttll12	Ttll12	0.0728970724696372	5.83861891764922	5.41454607058319	0.0198695649532443	0.419154537437266
RGD1359	RGD13595	-0.283331489866677	0.0128001140074883	5.41207720049881	0.0198979061886723	0.419154537437266
Mcam	Mcam	0.158483314915739	4.57683099218105	5.40794829213283	0.0200452623987399	0.419154537437266
Fyco1	Fyco1	0.165810682896432	3.13403451632113	5.40703758829476	0.0200557232387632	0.419154537437266
Tex10	Tex10	0.094231194495725	5.15540538723022	5.39581691846284	0.0201839178357284	0.419478670095261
RT1-DOb	RT1-DOb	-0.476381558678829	1.02449974266311	5.39469524245814	0.0201980522941866	0.419478670095261
Drgx	Drgx	0.678071892380308	-0.237529339056561	5.38615538796647	0.0202971432368793	0.419478670095261
Triobp	Triobp	0.10661062498172	6.9786440529487	5.38298489653423	0.0203340584077381	0.419478670095261
Ppfia4	Ppfia4	-0.108683485374551	6.93490982692438	5.38067054549626	0.0203610508069043	0.419478670095261
Crtc1	Crtc1	0.0901843237560234	6.32147470921386	5.38048877824852	0.0203632172246725	0.419478670095261
Itch	Itch	0.0921178661712323	5.11958740196286	5.37736187955487	0.0203997029077189	0.419478670095261
Ska1	Ska1	-0.247518506404814	1.27405338998521	5.3762886263862	0.0204122570348724	0.419478670095261
Ndufs4	Ndufs4	-0.0761199686890776	6.56511013800137	5.37112146378048	0.0204728106722835	0.419478670095261
Ncoa2	Ncoa2	0.131717605946652	6.11477525290037	5.36476224965097	0.0205475890638595	0.419478670095261
Cox6c	Cox6c	-0.109989123238251	6.99153832579538	5.36409534097491	0.0205554476384817	0.419478670095261
Tmem18	Tmem18	-0.089905925392997	4.53217195120372	5.36351287504399	0.0205623137187905	0.419478670095261
Ybx1-ps3	Ybx1-ps3	-0.183124821242689	5.58551356612647	5.35750074648139	0.0206332232059631	0.419478670095261
Itpkb	Itpkb	0.0831709743108121	6.141494616423	5.35583237778559	0.0206518889642479	0.419478670095261
Prrc1	Prrc1	0.0979432136578917	4.25184186722676	5.35196887207057	0.0206989082708888	0.419478670095261
Eif2d	Eif2d	0.0933635456468478	4.86584451371885	5.34971609172347	0.020725645211326	0.419478670095261
Nxnl2	Nxnl2	-0.348830452919961	0.469269746662765	5.34913360983882	0.0207325703121195	0.419478670095261
Zfp111	Zfp111	0.138657329158585	3.44572714582147	5.34565679821059	0.0207739559048593	0.419478670095261
Wdr91	Wdr91	-0.0827872498714783	5.05159265915295	5.34123157249063	0.0208267542049782	0.419478670095261
Zfyve28	Zfyve28	0.138938347798067	3.67610206618645	5.34090729436395	0.0208306288195964	0.419478670095261
Rbm11	Rbm11	-0.171365900159625	2.45986945479045	5.34089685388571	0.0208307534559778	0.419478670095261
Caly	Caly	-0.116548406819126	7.52033855582099	5.33967057772652	0.0208418248382501	0.419478670095261
Llgl1	Llgl1	0.0709802896176471	6.86331577962588	5.33464151326303	0.0209056282670889	0.419478670095261
Bmt2	Bmt2	0.109509920196045	3.60326340765236	5.3326214677013	0.0209301218759895	0.419478670095261
Pitpnb	Pitpnb	0.050292663098824	6.91561898828441	5.32908306282935	0.020972418031215	0.419478670095261
Drg1	Drg1	-0.0925853868140387	5.66579303345675	5.32854093594642	0.0209789407076112	0.419478670095261
Arhgef19	Arhgef19	-0.184372932234164	2.1935701416208	5.3258097893127	0.0210111195325519	0.419478670095261
Adgre1	Adgre1	0.377242344300814	1.35787112775985	5.32436471023868	0.0210282734473443	0.419478670095261
Kif26b	Kif26b	0.166455668183402	6.18576663788305	5.32295972115142	0.0210462347507237	0.419478670095261
Rptor	Rptor	0.0676940591630689	6.40960115343418	5.32017973782149	0.0210798389920436	0.419478670095261
Lpar6	Lpar6	0.253290590039093	2.00412023983269	5.30644512254464	0.0212466669416127	0.421485035603425
Akr1b1	Akr1b1	-0.114077477955121	6.28788891903069	5.30631748368629	0.0212482237232151	0.421485035603425
Ppp1r2	Ppp1r2	-0.0742210620614639	6.61109894900523	5.30065647553334	0.0213173886215258	0.42206145694684
Bmp1	Bmp1	0.107367857389231	5.54511519941131	5.29412221901897	0.0213975125258291	0.42206145694684
Cenpl	Cenpl	0.129321753341414	3.25349930794973	5.29380851633893	0.0214013670234819	0.42206145694684
Vamp2	Vamp2	-0.0736539614708308	9.37555365007067	5.29282568382814	0.0214134478438484	0.42206145694684
Otop3	Otop3	-0.743134291986439	0.29913875003117	5.29014628127383	0.0214464185064006	0.42206145694684
RT1-T24-	RT1-T24-1	0.508012887882162	-0.346369647098818	5.28644359126376	0.0214920674806644	0.422067498656856
Rhobtb2	Rhobtb2	0.0709482957358526	6.35263049236098	5.28233874752005	0.0215427796775283	0.422067498656856
Akap10	Akap10	0.0922177678693619	4.72330520595254	5.28190102250803	0.0215482084352637	0.422067498656856
Rbpms2	Rbpms2	0.256853159551394	1.31536612852434	5.27461844543798	0.0216385298214228	0.422759014718365
Rgl1	Rgl1	0.0980518103285267	4.99607914817919	5.27308947882531	0.0216575424076503	0.422759014718365
Tpd52l1	Tpd52l1	-0.161046533395319	3.50552152882569	5.26983140760689	0.0217130770066442	0.422759014718365
Frmd4a	Frmd4a	0.0877781030921148	7.3863608853589	5.26915300086061	0.0217190452181431	0.422759014718365
Dse	Dse	0.203443062580679	2.96952182682046	5.25685575489088	0.0218604777368421	0.424266348162684
Sec24c	Sec24c	0.0540949641737317	7.26292341257642	5.25653624266528	0.0218644693514058	0.424266348162684
Meltf	Meltf	0.266168421988467	1.80975524273797	5.24610061946515	0.0219990097678242	0.424802561244086
Naa35	Naa35	0.0655915398343082	6.17505054428037	5.24471670000346	0.0220135126744315	0.424802561244086
Ipmk	Ipmk	0.0929985024936094	5.68590174811187	5.24279820229559	0.0220377872964534	0.424802561244086
Ctbp2	Ctbp2	0.107922212497669	5.56770526558747	5.23976242329067	0.0220762885557304	0.424802561244086
Pnoc	Pnoc	-0.2005518027462	3.31617054068626	5.23885113386343	0.0220878557618252	0.424802561244086
Tinagl1	Tinagl1	0.344659201652324	2.44688185205492	5.23817781645323	0.0220964062675092	0.424802561244086
Utp4	Utp4	0.0770761769665971	5.03626819781175	5.23286734659271	0.0221639654748161	0.425199245165961
Acsm5	Acsm5	0.711761002867126	-0.327049218205479	5.23120187384977	0.0221851974515541	0.425199245165961
Brd3	Brd3	0.0679182512308945	7.21291009800629	5.2260186993052	0.0222514095214065	0.42565762603688
Pklr	Pklr	-0.17188271557565	2.23532947125458	5.22197824750384	0.0223032534349928	0.42565762603688
St3gal1	St3gal1	0.134052114042307	4.5482703474215	5.22133159794147	0.0223114600807982	0.42565762603688
Rhobtb1	Rhobtb1	0.092813777087424	5.10956091097053	5.21214981881674	0.0224295837092599	0.427257888074894
Ino80c	Ino80c	-0.0824517854440731	5.11487724826686	5.20754269327309	0.022489098442408	0.42726492874221
Ap1g1	Ap1g1	0.0669269265232873	7.09301671346801	5.20579235621346	0.0225117381524653	0.42726492874221
Trabd	Trabd	0.0822284027520252	5.12655714629798	5.20417651822471	0.0225326859612138	0.42726492874221
Klhl42	Klhl42	0.11388729869816	4.07865267712652	5.20143222592116	0.0225682727854846	0.427290348222445
Rpl38	Rpl38	-0.19406891667115	6.78830972414155	5.19459268551572	0.0226572641660834	0.427901684618898
Ext2	Ext2	0.065945039307152	5.9764186594269	5.19333248213152	0.0226736982940774	0.427901684618898
Zfp541	Zfp541	-0.286093506894887	0.940905279121381	5.19305365185	0.022703447560929	0.427901684618898
Sst18	Sst18	0.0986559367264446	5.28450471807167	5.17583378848629	0.0229031772806057	0.430490032723613
Sv2a	Sv2a	-0.0790229438279486	8.52777369579101	5.17533265355805	0.0229097945418353	0.430490032723613
Qser1	Qser1	0.171442046270365	5.58403810964302	5.16643049613798	0.0230274857470553	0.431403480631096
Polb	Polb	0.080236477075479	5.49703594748605	5.16642579588813	0.0230275481899744	0.431403480631096
Cpne3	Cpne3	0.205846175767455	2.8149900168734	5.15794625873054	0.0231402448021059	0.432864819184221
Ccni	Ccni	-0.0815757016590351	9.1085379677542	5.15289851411581	0.02319422922284	0.433074813675261
Srgap1	Srgap1	0.169323938478223	5.98488177366599	5.15190455173294	0.0232208904663581	0.433074813675261
Insr	Insr	0.136457923591548	5.65147020470759	5.13850290864521	0.0234008195018027	0.43486836694943
Plxna4	Plxna4	0.147202926928881	8.24357652476167	5.13263039597604	0.0234665951559431	0.43486836694943

Eif5	Eif5	-0.0526032270399652	8.17395285917254	5.13244490892737	0.0234691028915344	0.43486836694943
Rprd1b	Rprd1b	0.0757564500365222	5.34780576868	5.13191055053019	0.0234898576694329	0.43486836694943
Fli1	Fli1	0.240782753460002	2.69378692794358	5.13061234317519	0.0235074329717902	0.43486836694943
Denr	Denr	-0.0652431457153908	6.11174931307552	5.12922869573085	0.0235261799864443	0.43486836694943
S100a13	S100a13	-0.208752760872554	2.51389584776406	5.123837246585	0.0235993765708066	0.435576068114872
C1d	C1d	-0.0773584059473943	5.94780176085726	5.11925249828276	0.0236618070348391	0.43608325904529
Elavl1	Elavl1	0.112056427049061	5.50419895421936	5.10790449258413	0.0238170702471952	0.438168975216469
Sned1	Sned1	0.344234812232799	3.86419515898861	5.10440695317943	0.0238652362614088	0.438168975216469
Ahnak	Ahnak	0.275710499635906	4.59797606561511	5.10330274529041	0.0238803320682214	0.438168975216469
Syt13	Syt13	-0.128010451087615	6.50205290826384	5.0974655064845	0.0239608295185572	0.438676630769572
Scrg1	Scrg1	-0.147283283911326	3.06111238430438	5.09391701727858	0.0240099018332734	0.438676630769572
A3galt2	A3galt2	0.360493598841558	0.509039937670383	5.09365886549	0.0240134759009071	0.438676630769572
Eif2ak3	Eif2ak3	0.0717379564714828	5.37295162321644	5.08974876729357	0.0240676780907667	0.439024005173241
Plagl1	Plagl1	0.184798708610518	5.81349153643192	5.07038867454909	0.0243379260103381	0.44146320690241
Cbwd1	Cbwd1	0.118835781077627	3.08720708505658	5.07024945375188	0.0243398807581773	0.44146320690241
Mycbpap	Mycbpap	0.258630038747898	1.17769940662639	5.07023021523955	0.0243401508918427	0.44146320690241
Trim21	Trim21	0.447264875917511	-0.206404588551928	5.07003259677109	0.0243429258915492	0.44146320690241
Slc7a1	Slc7a1	0.0944065910465559	7.28904702973759	5.06475372692949	0.0244171745253186	0.442167034183455
Caprin1	Caprin1	0.0654479638163851	8.85121139090431	5.05772895587167	0.0245163443307549	0.442958755275072
Elovl6	Elovl6	0.10116216909211	5.50411867352678	5.05662982652684	0.0245318986907249	0.442958755275072
Pgm5	Pgm5	0.25651889 7465	2.80214287113249	5.0428946325532	0.0247271386706604	0.445637526602235
Rnf14	Rnf14	-0.0635443426890572	8.82179533477305	5.03860513745489	0.0247884419720713	0.445637526602235
Igdcc3	Igdcc3	0.187514058651823	3.68277008622219	5.03584292210518	0.0248265682326944	0.445637526602235
Aqp1	Aqp1	0.620800591098673	3.71899382079758	5.02990701967821	0.0249132358841203	0.445637526602235
Nit1	Nit1	-0.0760739441252424	5.55788216273002	5.02261882140352	0.0250038601838343	0.445637526602235
Cald1	Cald1	0.17142796579 0115	4.59595543860824	5.02240017627689	0.0250214626159654	0.445637526602235
Ptk7	Ptk7	0.0884778742697132	6.18909496823904	5.02058892228449	0.0250475486217463	0.445637526602235
Cd24	Cd24	0.102326291 57675	9.70547849532603	5.01759894619606	0.0250909236515764	0.445637526602235
Serpinh1	Serpinh1	0.224513786387228	6.50614094344094	5.01661485190121	0.0251052030577828	0.445637526602235
Pcdhb6	Pcdhb6	0.398999070027302	1.91620746819739	5.01601517449303	0.0251138967958623	0.445637526602235
Mideas	Mideas	0.108634549 12334	4.17703057506237	5.01439696372426	0.0251373832847156	0.445637526602235
Atp5if1	Atp5if1	-0.113113208355737	6.62948820641166	5.01167510365886	0.0251769342980875	0.445637526602235
Ecm1	Ecm1	0.177409704744233	2.79471731261444	5.00714765577636	0.0252428655041608	0.445637526602235
Klhl25	Klhl25	0.130122811043395	5.48344743462455	5.00473822756521	0.0252780259562892	0.445637526602235
Ccl19	Ccl19	0.475465612011036	0.110836834001715	5.00443837898587	0.0252824051618508	0.445637526602235
Kdr	Kdr	0.163804057982373	5.60316432103507	5.00245776293396	0.0253112050741417	0.445637526602235
Diras2	Diras2	-0.103014314789882	5.72156158313895	5.00245871388282	0.0253113374016287	0.445637526602235
Cd93	Cd93	0.158763371387192	5.49549957043523	5.0016507722962	0.0253232155114294 8	0.445637526602235
Ngrn	Ngrn	-0.0751980980165383	6.23514624378596	4.99857117944865	0.0253682527016765	0.445802378815236
Prr5l	Prr5l	0.232102187463397	1.21293408 6494	4.99139615340879	0.0254735477154356	0.446159598434886
Gatm	Gatm	-0.0989412507695109	6.72227620998923	4.98913913928774	0.0255068952328385	0.446159598434886
N4bp2	N6amt1	-0.138463241717779	2.82857558806035	4.98884506277575	0.0255112305225692	0.446159598434886
C2cd3	C2cd3	0.110426037710298	5.57070565199188	4.98748305258074	0.0255316144331577	0.446159598434886
Eef1a2	Eef1a2	-0.100982642016829	8.9724402315145	4.982362909865	0.0256069705321215	0.446850598271721
Map2k3	Map3k3	0.0988566309956724	4.71004464707296	4.97942374079326	0.0256505025871887	0.446985490649136
Alyref	Alyref	0.0985102620211711	6.36824699658461	4.97568871921957	0.0257064128793567	0.447334607385961
Cchcr1	Cchcr1	0.176562724184703	4.11445308768665	4.97060304192132	0.0257816570731384	0.447419886975505
Hecw1	Hecw1	0.112299861533 24	7.02450147458759	4.97051075733263	0.0257830326789603	0.447419886975505
Nrm	Nrm	0.122238320499 39	3.17857200066778	4.96232690186538	0.0259053259781269	0.448759534868726
Ramp1	Ramp1	-0.232917778364128	1.98216717699046	4.96038036526216	0.025934502149392	0.448759534868726
Mtmr1	Mtmr1	0.0735059337192909	5.96142132797264	4.95813856837856	0.0259681320970762	0.448759534868726
Anapc1	Anapc1	0.0865682555870003	7.54275292785186	4.94871880560588	0.0261100109039291	0.449067748860863
Rexo18	Resp18	-0.175860812377596	4.77042364525182	4.94791886767911	0.0261220952662527	0.449067748860863
Scn4a	Scn4a	-0.292704993088028	0.458995924208588	4.94790018875332	0.0261223775112056	0.449067748860863
Btk	Btk	0.164015458476 58	2.73743541358857	4.94373654064217	0.0261853708069944	0.449067748860863
Lcp1	Lcp1	0.173906664631334	3.35152998824615	4.94233150446325	0.0262066636928079	0.449067748860863
Fat1	Fat1	0.198943248287174	7.73587221480981	4.94099402267258	0.0262271012648959	0.449067748860863
Tipin	Tipin	-0.114899945781529	4.3555049120237	4.94022062302888	0.0262386872752039	0.449067748860863
Slc35c1	Slc35c1	0.0658644331535862	5.76107741157742	4.93790257918712	0.0262739005104136	0.449067748860863
Fam219a	Fam219a	-0.0674205670914555	7.18244957910267	4.9275995885521	0.0264310076037317	0.450653248529677
Ddi2	Ddi2	0.0988275831539126	5.19358345193908	4.92708358210138	0.0264389018132343	0.450653248529677
Scaf8	Scaf8	0.0688626342170513	6.93917264502848	4.92128636626863	0.0265277582536458	0.451058961584139
Zfp260	Zfp260	0.0802685578074599	6.06389440575199	4.91726259486258	0.0265896149586498	0.451058961584139
RGD1563	RGD156388	0.208726210585318	2.05610988803475	4.91648474962447	0.0266015868974316	0.451058961584139
Sox5	Sox5	0.124043407927816	5.52307967746938	4.91611338624722	0.0266073091068307	0.451058961584139
Sh2b3	Sh2b3	0.100951021542747	4.26622278496705	4.90957723462423	0.0267081774172152	0.451724145644758
Iffo1	Iffo1	-0.0963931447363694	5.03854759520118	4.90888028480658	0.0267189564387137	0.451724145644758
Gabpa	Gabpa	0.0768119084924749	5.48037638711465	4.90621061382379	0.0267602874219488	0.451810698462322
Ntsr1	Ntsr1	0.153987502362533	6.24145256426258	4.90304776395243	0.0268093296713562	0.452027204161502
Fabp7	Fabp7	-0.120210773802443	8.88967382628282	4.89485522554634	0.0269368318806463	0.452509224634378
Psma1	Psma1	-0.0968087107661153	6.76897730580395	4.89424268006986	0.0269463895998306	0.452509224634378
Gdpd5	Gdpd5	0.0800568072964 48	6.53384376034335	4.89414849218518	0.0269478586526009	0.452509224634378
Itgb2	Itgb2	0.357425034865398	0.470725745959429	4.89189858402756	0.0269829977681279	0.452509224634378
Tns1	Tns1	0.166189054164927	4.28077775817071	4.8825512416053	0.0271294919073946	0.453535675158424
Trpc3	Trpc3	-0.154994150325725	4.65418230297233	4.88152393655987	0.0271456424808652	0.453535675158424
[illegible]	[illegible]	[illegible]	[illegible]	[illegible]	[illegible]	[illegible]

**Aff4**	Aff4	0.0872276705297614	8.5502540240775	4.24068810473688	0.0394661682129712	0.4872272231790837
**Galnt6**	Galnt6	-0.1267208174198	3.51039927564077	4.2379459550851	0.0395299648829758	0.4872272231790837
**Tecta**	Tecta	-0.350158987992314	0.568206514989456	4.23232835777272	0.0396375386689285	0.4872272231790837
**Pp2d1**	Pp2d1	-0.105815054034887	4.04657207341484	4.23247231425435	0.039657633798054	0.4872272231790837
**Rspo3**	Rspo3	0.173480685776629	2.71921172529944	4.23148060821849	0.0396808109141158	0.4872272231790837
**Cd4**	Cd4	0.324474756159511	1.38700559678987	4.23041765845859	0.0397055688475256	0.4872272231790837
**Aldoc**	Aldoc	-0.104808898554187	7.66673696772347	4.22944290187513	0.0397284786726454	0.4872272231790837
**Pgap4**	Pgap4	-0.0757867709155074	6.61472089728354	4.2268946355499	0.0397881744469569	0.4872272231790837
**Dcp1a**	Dcp1a	0.12155212407067	3.69766465855763	4.22457986999805	0.039842481807179	0.4872272231790837
**Rps27a**	Rps27a	-0.0807408678253141	8.63949494695986	4.22103005008961	0.0399259162782714	0.4872272231790837
**Agpat2**	Agpat2	0.448557424074167	-0.582364021771623	4.21833566024721	0.0399893672324887	0.4872272231790837
**Ybx3**	Ybx3	0.0747335724875453	7.18545445628715	4.216413093846	0.0400347069859206	0.4872272231790837
**Dctn4**	Dctn4	0.0538584911827061	6.3442724976317	4.21590822683297	0.0400466221721011	0.4872272231790837
**Pomc**	Pomc	0.605129496244752	1.58429055492772	4.21230846802769	0.0401316867193163	0.4872272231790837
**Acaca**	Acaca	0.0869994601908885	8.0774026434517	4.21155326193615	0.0401495567935839	0.4872272231790837
**Syncrip**	Syncrip	0.066364279728427	6.70392084785721	4.21036065124602	0.0401777939318904	0.4872272231790837
**Ifrd1**	Ifrd1	0.0769855435528377	5.79099849407914	4.20927554355556	0.0402035038602152	0.4872272231790837
**Dusp19**	Dusp19	0.11203469791 7298	3.56167035736536	4.20806887994925	0.0402321141450102	0.4872272231790837
**Axin2**	Axin2	0.0973109162936239	4.82475935348035	4.20709965465741	0.0402551101865935	0.4872272231790837
**Fubp1**	Fubp1	0.0844299340197681	7.40384612704385	4.20697771266731	0.0402580043846593	0.4872272231790837
**Iqgap1**	Iqgap1	0.125305294972702	5.22805301158635	4.20665303649079	0.0402657112855921	0.4872272231790837
**Slc16a8**	Slc16a8	0.446019276938239	0.821196977697183	4.20523107872863	0.0402994832576323	0.4872272231790837
**Mettl25**	Mettl25	-0.112238576024025	2.98765590528463	4.20450317237139	0.0403167828814471	0.4872272231790837
**Nectin4**	Nectin4	-0.271927905603647	-0.0229108517312 37	4.20340595751312	0.040342874175128	0.4872272231790837
**Casp8ap2**	Casp8ap2	0.103522549427454	4.81249914205287	4.20220029308342	0.0403715647928373	0.4872272231790837
**Chst7**	Chst7	-0.130754695917543	3.95658870754723	4.20194126140871	0.0403777216399638	0.4872272231790837
**Mrc2**	Mrc2	0.211219516693285	4.48387569866829	4.19557790103288	0.0405295370762555	0.48798442870 3253
**Zswim5**	Zswim5	0.0999198753522309	5.11550098823497	4.19455252504478	0.0405541034603469	0.487984428703253
**Klhdc8a**	Klhdc8a	-0.104008572419829	5.20060929113828	4.19173606314354	0.0406214786039514	0.487984428703253
**Tmem185b**	Tmem185b	-0.0849030309512585	4.5894481012396	4.19105518599607	0.0406377959494868	0.487984428703253
**Acvr1**	Acvr1	0.109508901388724	5.1621215483625	4.19007449014613	0.0406613106001458	0.487984428703253
**Itga1**	Itga1	0.286978955501822	2.18083946318497	4.18699110325042	0.0407352356755479	0.487984428703253
**Adarb1**	Adarb1	-0.0723880977857798	7.22862990674969	4.1859296301201	0.040760851950267	0.487984428703253
**Scamp1**	Scamp1	-0.0554358539121181	8.14451879228721	4.18543779257329	0.0407726807248855	0.487984428703253
**Igflr1**	Igflr1	0.244638949749243	0.467332905634323	4.18208387912931	0.0408534388677767	0.487984428703253
**Il23a**	Il23a	-0.296448893067521	0.0884305099079321	4.181398042976 7	0.040869973645774	0.487984428703253
**Lyst**	Lyst	0.152668559984952	5.70988241153186	4.17565195561 33	0.041008782081422	0.487984428703253
**Nefm**	Nefm	-0.104294601007599	8.00134287106473	4.17440520147764	0.0410389652779783	0.487984428703253
**Pigp**	Pigp	-0.102689196610851	3.42919815396755	4.17279364287364	0.0410780147536839	0.487984428703253
**Chrna5**	Chrna5	-0.126998270074076	3.17851452173783	4.17207544271056	0.0410954289411569	0.487984428703253
**Pla2g6**	Pla2g6	0.0811247123242761	4.69689903526585	4.17131333786173	0.0411139182260748	0.487984428703253
**Ece1**	Ece1	0.0807686739452705	5.2156293692194	4.17016266721237	0.0411418494783027	0.487984428703253
**Tcn2**	Tcn2	0.141164440671805	4.49986191492474	4.16908097073293	0.0411679789403769	0.487984428703253
**Itga6**	Itga6	0.0887942777053225	5.51072113639233	4.16744850167816	0.0412080062982206	0.487984428703253
**Ppp3r1**	Ppp3r1	-0.0550492830185161	8.4886950612897	4.16728195776506	0.0412118629402827	0.487984428703253
**Ankk8**	Ankk8	-0.0582743157147076	5.96026434896966	4.16610058813561	0.0412406114080589	0.487984428703253
**Lgi3**	Lgi3	-0.161005848872094	3.91815173005323	4.16523012411933	0.04126180750837	0.487984428703253
**Plpbp**	Plpbp	0.0736667364257539	4.52777305879409	4.16127076471431	0.0413583637811833	0.488498800536359
**Dus3l**	Dus3l	-0.0888786152006437	5.28945770979296	4.16023734862242	0.0413836045748924	0.488498800536359
**Txnrd3**	Txnrd3	-0.0939888656561418	4.60924830562335	4.15646279101826	0.0414759342828176	0.488861044439784
**Lrrc32**	Lrrc32	0.277697603232521	2.48075487821595	4.15578020455537	0.0414926541686087	0.488861044439784
**Tmem199**	Tmem199	-0.0839958571112945	4.91074186380181	4.14652834284585	0.0417199773395647	0.490729822781208
**Adck2**	Adck2	0.0948292436071962	4.05684202477659	4.14612447158382	0.0417299206298595	0.490729822781208
**Pick1**	Pick1	-0.0571040365728582	6.08821793808905	4.14349211520324	0.0417948639418644	0.490824841416421
**Tmem229a**	Tmem229a	-0.0970416977788507	5.60583203100129	4.14203280681556	0.0418309070891048	0.490824841416421
**Hykk**	Hykk	0.33379456081202	0.179650121194657	4.14101659461144	0.0418560255884485	0.490824841416421
**Tmem220**	Tmem220	0.34551724468974	-0.317627064966623	4.13929405796243	0.0418986280618979	0.490863210868826
**Krt8**	Krt8	0.556754276774879	2.08031300551329	4.13217236526569	0.042050426785886	0.491509483145833
**Zdhhc4**	Zdhhc4	0.108202580929887	3.94777775778817	4.13143374626294	0.0420936721705778	0.491509483145833
**Kif1c**	Kif1c	0.0911328850149338	5.90806360198307	4.13129830890334	0.0420970171864803	0.491509483145833
**Tesmin**	Tesmin	-0.349175182668818	0.354347519025666	4.13072218092566	0.042111375933521	0.491509483145833
**Zfp703**	Zfp703	0.0893057130154924	5.52538018160613	4.12248227729972	0.0423169442166256	0.493292532861436
**Mfap4**	Mfap4	0.180333283864892	5.05998467171957	4.11802995575317	0.0424294589838837	0.493292532861436
**Zfp707l1**	Zfp707l1	-0.138676538237563	2.65128245064944	4.1174431282782	0.0424431799427849	0.493292532861436
**Fxyd7**	Fxyd7	-0.198072141624118	5.3810961447087	4.11511366055029	0.0425016681774615	0.493292532861436
**Lrrc14b**	Lrrc14b	0.171794979785076	1.91119420849326	4.11488835178545	0.0425073308150386	0.493292532861436
**Col6a2**	Col6a2	0.264242001024741	5.51430821911932	4.11324742773772	0.0425485884517418	0.493292532861436
**Parvg**	Parvg	0.309050018014379	0.318973121532683	4.11208330702337	0.0425778830820165	0.493292532861436
**Lama4**	Lama4	0.134398125122888	5.68742852734042	4.11097766155433	0.042605726425 6174	0.493292532861436

Stx16	Stx16	0.0761802636242083	5.57826774100067	3.64946374869654	0.0560872673418081	0.519811467031011
Slc4a10	Slc4a10	-0.0806036974091647	6.29642987725465	3.64546359155717	0.0562221496734715	0.520157610947273
Amotl2	Amotl2	0.0637005204535697	6.81446546868422	3.6444739661317	0.0562555721862743	0.520157610947273
Tead3	Tead3	0.174604647512658	2.06871503184433	3.64443764207316	0.05625679855812	0.520157610947273
Nin	Nin	0.157907629400263	5.87019352484348	3.64289776861335	0.0563088484706115	0.520157610947273
Foxo1	Foxo1	0.260353851552642	0.711029622074986	3.64244792407685	0.056324063264558	0.520157610947273
Ptprj	Ptprj	0.0810128388571072	6.26296294732375	3.63981612424242	0.0564131643491533	0.520157610947273
Sox18	Sox18	0.149681703707124	3.94673353312114	3.63860008904415	0.0564543844242146	0.520157610947273
P4ha3	P4ha3	0.139140037907124	3.51938135427026	3.63799056841167	0.0564750574341433	0.520157610947273
Dmgdh	Dmgdh	-0.262501213065806	2.39797345477126	3.63710204918428	0.056505207585189	0.520157610947273
Zc3h10	Zc3h10	0.0917332676493721	4.06559181639442	3.63635076823927	0.0565307141495954	0.520157610947273
Ccdc102a	Ccdc102a	0.161236252369467	3.39751570408973	3.63512262920944	0.0565724367340827	0.520157916824723
Zwint	Zwint	-0.110318551616999	10.5288286199861	3.63038346669175	0.0567337431416481	0.521256921648264
Rttn	Rttn	0.141243675932999	3.22535976944708	3.62843817958691	0.0568000959322386	0.521482558459559
Ablim3	Ablim3	-0.101720000450843	7.45634670965142	3.62284713327395	0.0569912627931598	0.521888403737403
Vhl	Vhl	0.0597603966988149	6.31482623809951	3.62209740494208	0.0570169490960768	0.521888403737403
Slc6a7	Slc6a7	-0.161166179339076	6.01810001240743	3.61892423405888	0.0571258006786161	0.521888403737403
Tbr1	Tbr1	0.0779291434603608	7.79056562449604	3.61889369996656	0.0571268491827654	0.521888403737403
Lppos	Lppos	0.288624982947483	0.108784138095188	3.61545724624681	0.057244983533232	0.521888403737403
Dab2ip	Dab2ip	0.0457201983380077	7.97495138773481	3.61452987568649	0.0572769080061637	0.521888403737403
Utrn	Utrn	0.112680625836544	5.26616360872698	3.6143126352398	0.0572843898691374	0.521888403737403
LOC688390	LOC688390	0.201316299444468	1.17956447276774	3.61403521876503	0.0572939441628066	0.521888403737403
Clec4a1	Clec4a1	0.454254338182196	-0.712257039221319	3.61345877247655	0.0573138039620655	0.521888403737403
Sowahc	Sowahc	0.0669880522722624	4.96359329706866	3.61262755566253	0.0573424540171632	0.521888403737403
Ankib1	Ankib1	0.0597019937191288	6.67398257415387	3.61213700578335	0.0573593682329979	0.521888403737403
Htr7	Htr7	-0.197519422305257	2.5764568774906	3.61095196466658	0.057400253864841	0.521888403737403
Ccdc65	Ccdc65	-0.0696764435181089	4.5045579861096	3.60990678623742	0.0574363238828022	0.521888403737403
Dcaf11	Dcaf11	-0.0466999882621356	6.52021457605968	3.60896970760729	0.0574687121143599	0.521888403737403
Rab6a	Rab6a	-0.0501189464483048	9.04073192055332	3.60888263187355	0.0574717213060185	0.521888403737403
Arl5c	Arl5c	-0.296969153913439	-0.191826494152783	3.60653459115775	0.0575529287864436	0.521963049076887
Tjp1	Tjp1	0.0867274746316697	6.68408192869394	3.60482674763755	0.0576120714562908	0.521963049076887
Ech1	Ech1	0.0821508257307884	5.29013657153006	3.60383463242717	0.0576464580693769	0.521963049076887
Rab20	Rab20	0.369398565451984	-0.369332432260926	3.60381099745582	0.0576472775208745	0.521963049076887
Ets2	Ets2	0.0790879985211346	5.67839949999852	3.59617266026353	0.0579127566065029	0.523726320152917
Inhba	Inhba	0.168393519855544	3.07907217969709	3.59452175878883	0.0579703060458556	0.523726320152917
Maz	Maz	0.0679619176830438	8.21688009507752	3.5929605898154	0.0580247833514376	0.523726320152917
Irak3	Irak3	0.161045122201232	1.66105259867244	3.59205696478492	0.0580563438694753	0.523726320152917
Copz2	Copz2	0.346350063641588	1.41084048690325	3.59064454357529	0.0581057024598489	0.523726320152917
Trpm5	Trpm5	0.417383064530962	0.321825464577385	3.59024218940414	0.0581197722163136	0.523726320152917
Sec24d	Sec24d	0.122377961737771	3.88000701378927	3.58939670878359	0.0581493244063749	0.523726320152917
Ntsr2	Ntsr2	-0.256390785278996	1.36776938686341	3.58758157082052	0.0582129021840932	0.523726320152917
Chchd1	Chchd1	-0.109649411640846	4.71780066853123	3.58581690294267	0.0582712512367895	0.523726320152917
Col1a2	Col1a2	0.380156661741581	7.22923182365589	3.58400575436349	0.0583383166331785	0.523726320152917
Styl1	Stpl1	0.267606374004393	0.279597950654275	3.5813265793621	0.058432471324015	0.523726320152917
Spock3	Spock3	-0.152660632287787	6.26326152309664	3.58049594845059	0.0584616951033483	0.523726320152917
Ppp1r12a	Ppp1r12a	0.0909937032314554	6.09928933593105	3.5792780829832	0.0585045708591624	0.523726320152917
Inppl1	Inppl1	0.0912070493823012	5.36573210362409	3.57895389311254	0.0585159898519614	0.523726320152917
Notch2	Notch2	0.142161004521663	5.58056356486171	3.57808290355172	0.0585466804956881	0.523726320152917
Mfap2	Mfap2	0.104242183617814	4.80376327542914	3.57618810726518	0.0586135056266241	0.523726320152917
Sqstm1	Sqstm1	-0.0786179946890376	8.52798083552737	3.57529100567652	0.0586451726162375	0.523726320152917
Cebpg	Cebpg	0.0859821323902649	4.45287497480294	3.57490213287684	0.0586589051725645	0.523726320152917
Abcb9	Abcb9	-0.105897907804513	5.05897734749645	3.57437450719758	0.0586775430774219	0.523726320152917
Stard13	Stard13	0.134544832599468	2.99421086000989	3.57426179644009	0.0586815256531039	0.523726320152917
Colq	Colq	-0.453813283158969	-0.0363905644876477	3.57227286178937	0.0587518443046192	0.523824850099735
Sh3gl3	Sh3gl3	-0.0641239592588918	5.53753691203016	3.57071292329928	0.0588070587656013	0.523824850099735
Nras	Nras	0.0577875623217186	6.18449995298861	3.57038944516772	0.05881851526727	0.523824850099735
Vsig10	Vsig10	0.200953226594739	1.51320775641424	3.56924513886273	0.0588945192541467	0.524127516249421
Gatad2a	Gatad2a	0.0548617137266738	6.72987458126289	3.56368099678017	0.0590573527331587	0.524250583390729
RT1-S2	RT1-S2	0.737090049723667	-0.033247554757501	3.56290475307831	0.05908492612140323	0.524250583390729
Ap1s2	Ap1s2	-0.119540057082489	6.03680089332031	3.56184466476984	0.0591220031029198	0.524250583390729
Mmp16	Mmp16	0.0903999349853144	6.16683289664757	3.56120383820835	0.0591448305202149	0.524250583390729
Pip4p2	Pip4p2	0.0638452530290241	5.78029782218555	3.56114342828324	0.0591469829519913	0.524250583390729
Zfp644	Zfp644	0.06736570923621	6.61850211018478	3.56078576552282	0.0591604402944327	0.524250583390729
Parp9	Parp9	0.178768612252671	1.87557077309988	3.55895879816359	0.0592248770305894	0.524342218415817
Lemd3	Lemd3	0.0590287631255741	5.44343389590525	3.55795148366658	0.0592608301126305	0.524342218415817
Sgcb	Sgcb	-0.0756180536329978	6.12502462528537	3.55634958852346	0.0593180394580023	0.524342218415817
Tbx18	Tbx18	0.38635189987001	2.90947372596021	3.55447644501177	0.0593849677630797	0.524342218415817
Slc25a4	Slc25a4	-0.0794574239021048	9.32748400053094	3.55270590508311	0.0594126184105548	0.524342218415817
Zfp39	Zfp39	0.110302567340071	4.04835315842512	3.55203166467832	0.0594725896008921	0.524342218415817
Fbxo42	Fbxo42	0.0726980306464525	5.26379101204885	3.5517432852054	0.0594829259328177	0.524342218415817
Ptp4a3	Ptp4a3	-0.0752615496887465	5.74933047472199	3.55018386376438	0.0595388534479591	0.524342218415817
[illegible]	[illegible]	[illegible]	[illegible]	[illegible]	[illegible]	[illegible]

Ajuba	Ajuba	0.164198012607325	3.21485330255155	3.19960019582292	0.0736562740872885	0.545725995230059
Kcnj3	Kcnj3	0.100772553145178	4.05021554949921	3.19911381938814	0.0736781829429188	0.545725995230059
Epn3	Epn3	0.296391224592763	1.66667206671322	3.19864136896523	0.0736994711974953	0.545725995230059
Kremen1	Kremen1	0.183834865952491	2.55189553001888	3.19688246869373	0.0737787839580041	0.545989257086453
Pknox2	Pknox2	0.0754164252757466	5.95140845826085	3.1953485102144	0.0738480282809426	0.546156970042704
Smim1	Smim1	0.169856275930437	1.21555860280285	3.19437350087937	0.0738920775389098	0.546156970042704
Cd74	Cd74	-0.314115937032379	-0.268645230628443	3.19262063515112	0.0739712267903331	0.546156970042704
Chchd4	Chchd4	-0.174606610350724	3.99295434292813	3.19211305066808	0.073994309121926	0.546156970042704
P2ry14	P2ry14	0.325806649491854	-0.0189384961149385	3.19153862765849	0.0740203122819759	0.546156970042704
Ro60	Ro60	0.0864424093967493	6.05019354106933	3.19028797666289	0.0740769610788583	0.546236111915305
Ca3	Ca3	0.426735119942659	-0.441261607630321	3.18936841874385	0.0741185972168876	0.546236111915305
B3galt1	B3galt1	0.0694992851098678	5.18068755258223	3.18760065822523	0.0741988418436152	0.546401121700641
Rbm34	Rbm34	-0.0521925850030703	5.26455341301382	3.18694585081842	0.0742285726763314	0.546401121700641
Fam126a	Fam126a	0.0749819055545258	5.04645824819452	3.18463785737331	0.0743334665377263	0.546850626174063
Trhde	Trhde	-0.0880285802296326	4.80011432838651	3.17889754099762	0.0745950437278661	0.548247509786753
Pcdhb10	Pcdhb10	0.118103273568313	2.80333376703124	3.17708188107568	0.0746779861399049	0.548247509786753
Clec2l	Clec2l	-0.107108979231713	5.19106117826208	3.17704344499977	0.0746797430349983	0.548247509786753
Tle1	Tle1	0.0662744876574078	5.93939539593439	3.17661986347704	0.0746991076891464	0.548247509786753
Acot11	Acot11	0.37165159702275	-0.595596110802921	3.17315559815434	0.0748576842590828	0.548583215842565
Hexb	Hexb	0.0816995896323706	5.09421854590028	3.17258531589734	0.0748838235433805	0.548583215842565
Pspn	Pspn	-0.214523911615453	0.592987554710002	3.17190826891132	0.0749148682762727	0.548583215842565
Pfn2	Pfn2	-0.0590467179016535	9.24176161780681	3.17178074890593	0.0749207181049716	0.548583215842565
Sbno1	Sbno1	0.0607581518959429	6.76949503153215	3.16846127717955	0.0750731438013783	0.549376900416303
Tapbp	Tapbp	0.125098893632308	3.35141666620921	3.16543168808733	0.0752125482135354	0.550074429288793
Sinhcaf	Sinhcaf	0.122643054397124	3.99349898160505	3.16372344150436	0.0752912762353876	0.550327623660768
Atp4a	Atp4a	0.32674574067987	0.00846817365367772	3.15964984278258	0.0754793713489353	0.550490680951856
Mroh7	Mroh7	-0.249835060024573	0.563624465682461	3.15909666477168	0.0755049528080932	0.550490680951856
Rab4a	Rab4a	-0.058526707739504	5.67597179151827	3.15867201268376	0.0755245969681238	0.550490680951856
Xrn1	Xrn1	0.118756105268656	5.01032299026428	3.15832189863721	0.0755407971716643	0.550490680951856
Prr18	Prr18	0.0737695245221997	4.55239901461772	3.15708449763356	0.0755980831523792	0.550490680951856
Ecsit	Ecsit	-0.0699957105345921	4.40924915522475	3.15551693879326	0.0756707209652847	0.550490680951856
Nme6	Nme6	0.183335020909064	2.28517692493524	3.15523241875032	0.0756839131449814	0.550490680951856
Atg4d	Atg4d	0.0702934329220298	4.72479062623865	3.15247877462316	0.0758117176025104	0.550490680951856
Gna13	Gna13	0.0853450670212491	4.3685759463502	3.1516853344102	0.0758495180438517	0.550490680951856
Lrpap1	Lrpap1	-0.0592907540954161	6.63240259327132	3.15150375448258	0.0758570266520307	0.550490680951856
Syndig1l	Syndig1l	-0.113009928588119	4.29619392964742	3.15096920887405	0.0758818792634186	0.550490680951856
Txnip	Txnip	0.125833614408332	6.43686763601255	3.1508398145745	0.0758878965038871	0.550490680951856
Exoc4	Exoc4	0.0593609806580802	6.2712125155241	3.15036449956499	0.0759100045441946	0.550490680951856
Snx10	Snx10	-0.0671698257232559	6.58953944404052	3.14990742075199	0.0759312709649567	0.550490680951856
Pald1	Pald1	0.0980463412033749	4.19044020381319	3.14824510513351	0.0760086694691997	0.5507317871807
Gzmm	Gzmm	0.334472553509204	-0.0323684280191168	3.14509402944523	0.0761555108022914	0.551158122809859
S1pr2	S1pr2	0.0916018137592846	3.56004146602107	3.14508880678172	0.0761558550712669	0.551158122809859
St3gal4	St3gal4	-0.06469771321182	5.27829073615754	3.14353937526449	0.0762282220400997	0.551362044286565
Adhfe1	Adhfe1	-0.216897183583534	1.02292314355116	3.14161789327447	0.0763180685185994	0.551692098272632
Tma16	Tma16	-0.0996784085195832	3.28273843719774	3.13960331768794	0.0764123902347083	0.552054078146182
Bop1	Bop1	0.0616912965070603	6.46889125502068	3.13806540600572	0.0764844790787973	0.552069469537425
Fsip1	Fsip1	-0.167877701003994	1.40250770664734	3.13767022981569	0.0765030145732314	0.552069469537425
Gfra2	Gfra2	-0.0896276638987047	4.92488520097041	3.13659066468327	0.0765536755332553	0.552115729108817
S100a1	S100a1	-0.154714164840252	2.17297194257439	3.13563985806013	0.0765983240964575	0.552118596043616
Oxct1	Oxct1	-0.0649580863397426	8.41892580314217	3.13159846596231	0.0767884150000257	0.552487198740343
Sh3pxd2a	Sh3pxd2a	0.110443654172501	4.1824635827456	3.13112559773442	0.0768106889601937	0.552487198740343
Osbpl6	Osbpl6	0.116783150291407	5.47529599376242	3.13110807670082	0.0768116095743425	0.552487198740343
Ophn1	Ophn1	0.0719534617542845	5.22788094210789	3.12644992999524	0.0770313167355427	0.552487198740343
Gpd1	Gpd1	-0.140262691121434	3.19727960046612	3.12521030641952	0.0770899240518544	0.552487198740343
Spata20	Spata20	-0.130951179798279	3.05861856660898	3.12248807496393	0.0772187949681677	0.552487198740343
Rnft2	Rnft2	-0.0378323398932871	7.33731523605168	3.12087798624773	0.0772951259126319	0.552487198740343
Cfh	Cfh	0.284316276008149	3.04119149082586	3.12084451749655	0.0772967134570979	0.552487198740343
Prkdc	Prkdc	0.145669443267236	5.16647123891653	3.12065891503111	0.0773055178938755	0.552487198740343
Flrt2	Flrt2	0.101237699089866	5.11097637717754	3.12038034326823	0.0773196835267467	0.552487198740343
Hdhd2	Hdhd2	-0.0698709758567933	5.21324980494485	3.12001485656692	0.0773360785077355	0.552487198740343
Ptprf	Ptprf	0.0646896642262375	8.27349863558956	3.1193102050117	0.0773695291580508	0.552487198740343
Ccdc151	Ccdc151	-0.255463810338903	0.462335020455051	3.11679018467114	0.0774892849851287	0.552487198740343
[illegible]	[illegible]	[illegible]	[illegible]	[illegible]	[illegible]	[illegible]

Rexo4	Rexo4	0.124315964064639	5.26541945710072	2.82943249969783	0.0925510588801187	0.571487990370967
Plppr1	Plppr1	0.0471651322244005	6.3837855820498	2.82854613208617	0.0926021585473018	0.571487990370967
E2f6	E2f6	0.0648838885064725	4.69230057585082	2.82833543077985	0.0926143076484807	0.571487990370967
Rasgrp3	Rasgrp3	0.123811458798559	3.18327016276142	2.82569217000039	0.0927668912749094	0.571783822646561
Cltb	Cltb	-0.0773644521757717	7.45787018868581	2.82546122802745	0.0927802354952195	0.571783822646561
Mospd2	Mospd2	0.099847953277036	4.08750047577945	2.82455344646985	0.0928327088813213	0.571783822646561
ST7	ST7	0.0646361890068857	5.15182012747519	2.82433124712955	0.0928455578009083	0.571783822646561
Pik3r1	Pik3r1	0.0640015344355013	6.69309532707112	2.82225062842884	0.0929080732247336	0.571842617938895
Trappc8	Trappc8	0.0500481680961943	6.13180126201591	2.8215523279499	0.0930064152113909	0.571842617938895
Rps25	Rps25	-0.0722145708201094	7.84721157469516	2.82148736909367	0.0930101789688575	0.571842617938895
Pdia6	Pdia6	0.0591150022698835	7.80298070779834	2.82010540128319	0.0930902903012845	0.571842617938895
Dnase1	Dnase1	0.211939169225175	0.514971069028544	2.81953609842689	0.0931233140574313	0.571842617938895
Wdhd1	Wdhd1	0.125385022166683	3.37866050062996	2.81941922160794	0.0931300953475538	0.571842617938895
Tuba4a	Tuba4a	-0.146058884993725	5.98288295630084	2.81855893174331	0.0931800266025981	0.571867777629423
Zfp867	Zfp867	0.0867961505271624	3.43040160147656	2.8173166697311	0.0932521789002062	0.572029221306722
Arrdc4	Arrdc4	0.105846617804521	3.78921211223533	2.81584469526074	0.0933377531851845	0.572146923117429
Rpl9	Rpl9	-0.0564163848021154	8.28120464390271	2.81530484816818	0.0933691571622176	0.572146923117429
Appl2	Appl2	-0.109499540642106	4.10227789811564	2.81402190740807	0.0934438376328275	0.572146923117429
Ptgs1	Ptgs1	0.252717789304735	0.825555394319953	2.81285823482928	0.0935116316421208	0.572146923117429
Kazn	Kazn	-0.0502552048878727	6.00235140275586	2.81189607455763	0.093567726205862	0.572146923117429
Vgf	Vgf	-0.122088363042791	7.30605113898153	2.81186107033605	0.0935697676642649	0.572146923117429
Lamp1	Lamp1	-0.0674851868315541	8.01264888521432	2.81104082151455	0.0936175181466108	0.572146923117429
Ccr10	Ccr10	-0.259261522556755	0.19252245257275	2.81068789509324	0.0936382156773094	0.572146923117429
Cln8	Cln8	0.0774439472322267	4.4190012283772	2.80762123490483	0.0938173949748031	0.572858083235253
Eml2	Eml2	-0.0636745207977554	6.81380597959605	2.80671575621159	0.0938703717076767	0.572858083235253
Fbxo44	Fbxo44	-0.0537210715529833	5.8796309372893	2.80627241353096	0.0938963221700916	0.572858083235253
Tdrp	Tdrp	0.112901268841803	4.15959718601236	2.80552400727287	0.0939401469215381	0.572858083235253
Eml4	Eml4	0.0845307204584985	6.77028752123925	2.80477258300738	0.0939841705844804	0.572858083235253
Fkbp7	Fkbp7	0.148519121224309	2.44028013693716	2.80323248901584	0.0940744702659852	0.57293087116849
Plekha8	Plekha8	0.0946552085371732	5.24382414078402	2.80300272322297	0.0940879502304217	0.57293087116849
Ushbp1	Ushbp1	0.125010749761224	2.29423484905605	2.80097034171188	0.0942072772171577	0.573377657482184
Ptges3	Ptges3	-0.0499350254317861	8.0771823804662	2.79787964402076	0.094389058695559	0.573733404139588
Slc40a1	Slc40a1	0.105211479845657	4.28464569229734	2.79771549665027	0.0943987216948983	0.573733404139588
Svil	Svil	0.144901892801792	5.0685518320191	2.79763134320913	0.0944036770616794	0.573733404139588
S1pr1	S1pr1	-0.0819864975978109	5.25767608951376	2.79634745540768	0.0944793138796699	0.573830850695657
Pcdhga8	Pcdhga8	0.11480721157081	3.72827303765417	2.7953769306655	0.094536532509561	0.573830850695657
St7l	St7l	0.0718781733321156	4.65337714472106	2.79500158279436	0.094558673161038	0.573830850695657
Letm1	Letm1	0.0445935105946217	7.11230465504105	2.79241428802361	0.0947114387329524	0.574484490090932
Cpeb1	Cpeb1	-0.159790096623928	1.69444919503468	2.79136222999441	0.0947736308555176	0.574491463651983
Unc13b	Unc13b	0.123754885931524	6.23586391670874	2.79063680556089	0.0948165414493374	0.574491463651983
Icam5	Icam5	-0.145975024877592	5.1306068713874	2.78810427978944	0.0949665122746065	0.574491463651983
Atmin	Atmin	-0.0510137854974199	5.8623212508188	2.78744372177707	0.095005671564481	0.574491463651983
Mir1896	Mir1896	0.171381342365309	1.2160711285191	2.78736261120274	0.0950104811841823	0.574491463651983
Psmd1	Psmd1	-0.0430108217371484	7.97954217342761	2.78685786097586	0.0950404172530396	0.574491463651983
Tmem186	Tmem186	0.0506982163890684	5.8943618066607	2.78608257259026	0.0950862584851784	0.574491463651983
Rcbtb1	Rcbtb1	0.0611058808638234	6.11048565283324	2.78540070262824	0.095126897398135	0.574491463651983
Hint3	Hint3	-0.0972649816343421	3.27755534713496	2.7845805827403	0.0951756078913012	0.574491463651983
Pex1	Pex1	-0.055887291798602	5.4465215172655	2.78284798175584	0.0952714656645729	0.574491463651983
Scgb1c1	Scgb1c1	-0.245429653526558	-0.303031807154255	2.78233493785183	0.0953091255487791	0.574491463651983
Lrp10	Lrp10	0.0718895421508075	5.72238691129332	2.78233342400748	0.0953092156250671	0.574491463651983
Srp14	Srp14	-0.0659332370078808	5.96654053741173	2.78230077058117	0.0953111585813581	0.574491463651983
Sdsl	Sdsl	-0.190923128999969	1.22274365877693	2.78056465824242	0.0954145224480576	0.574765070732955
Rasl10a	Rasl10a	-0.124788266107988	2.52522599594056	2.77999135541509	0.0954488837027076	0.574765070732955
Zfp592	Zfp592	0.0844039382765276	5.56261660620807	2.77616731580308	0.0956768796887038	0.575643765092343
Spsb4	Spsb4	0.0608005046251937	5.13957333914634	2.7760000504793	0.0956868773584611	0.575643765092343
Atp6v0c	Atp6v0c	-0.0757047262117337	9.56123303273172	2.77503465713411	0.0957445804012355	0.575713337329024
Poli	Poli	0.11309232968446	3.02319414140312	2.77400088758138	0.0958064228484022	0.575807677205932
Smad2	Smad2	0.0581484006414889	5.9546363210022	2.77313596002762	0.0958581951686736	0.575841454565247
LOC100360	LOC100360	0.119829458068571	4.11547789478089	2.7709297417251	0.0959903917204029	0.57621499423974
Jade3	Jade3	0.100192104013007	3.34094821576761	2.76881879724088	0.0961252903793916	0.57621499423974
Aacs	Aacs	-0.0459371492713189	7.44982258804393	2.76806100316453	0.0961625835397454	0.57621499423974
Ncdn	Ncdn	-0.058493472208103	9.15315423803848	2.76787870789032	0.0961735389033241	0.57621499423974
Zdhhc8	Zdhhc8	0.0711037103019071	5.81528802624785	2.76720222588344	0.096214195803626	0.57621499423974
Hnmt	Hnmt	-0.112200122988757	3.1173070365352	2.76625893715707	0.096270801805836	0.57621499423974
Serinc2	Serinc2	0.16038456582285	4.44606734857148	2.76597155817028	0.0962882111313214	0.57621499423974
Ulk3	Ulk3	0.122632621091807	3.30662067674204	2.76584460819242	0.0962898326632891	0.57621499423974
Abi1	Abi1	-0.0361597306583422	6.78025077212969	2.76281861418768	0.0964178437738233	0.576454041568849
Cldn11	Cldn11	0.311950975526866	3.91237723412821	2.76265943968824	0.0964274355113177	0.576454041568849
Clgn	Clgn	-0.129817476742049	2.64959574885748	2.76298007862282	0.0964693849321027	0.576454041568849
Nmnat1	Nmnat1	0.121364497388848	2.72641339692146	2.76069152029205	0.0966064704556893	0.576730531893345
Tmem179	Tmem179	-0.0569516012621788	5.86609912808356	2.7603150186004	0.0966292081905251	0.576730531893345
Lmbr1l	Lmbr1l	0.124032235345485	3.86342964792751	2.75961577887797	0.0966653325494277	0.576730531893345
Lgals3bp	Lgals3bp	0.206503419482179	3.02611105157695	2.75911765845702	0.0967015580185693	0.576742514052433
Smim13	Smim13	0.0501580174341091	7.72936820586761	2.75814707835055	0.0967602481576454	0.576816825734802
Stk39	Stk39	0.0770178822618579	6.01076970253717	2.75523642328806	0.0969364858952956	0.577591468250645
Wfdc2	Wfdc2	0.0997235381345688	3.17117308725781	2.7534256200981	0.0970463049955	0.577969807841935
Akr1b10	Akr1b10	-0.16859638264733	3.22907012862652	2.75236684872286	0.0971105767400158	0.578034328223184
LOC300308	LOC300308	0.65670456951579	-0.520511506243757	2.7513538521853	0.0971721171057552	0.578034328223184
Slitrk5	Slitrk5	0.0405001009242051	6.56064563726248	2.75063152518774	0.0972160235965589	0.578034328223184
Rere	Rere	0.110036066787868	7.63173730742665	2.75019693441872	0.09724245052267	0.578034328223184
Col18a1	Col18a1	0.242885651846622	5.92306870520692	2.74695320580331	0.0974399080975593	0.578588616122372
Iscu	Iscu	-0.0807942598552562	5.25647612246336	2.74623720698963	0.0974835977304312	0.578588616122372
Cpne4	Cpne4	-0.143226170022274	5.96038396767015	2.74622941639812	0.0974840728192355	0.578588616122372
Zfp381	Zfp381	0.0914961743777934	5.40972480007159	2.74518287334158	0.0975479166585467	0.578588616122372
Usp15	Usp15	0.0494386540365182	6.26687370647116	2.74486101818312	0.0975675601764424	0.578588616122372
LOC688801	LOC688801	-0.224030653457003	0.151291863016056	2.743895761258	0.0976264971469551	0.578663090214993
Sars2	Sars2	-0.0717979630245665	3.5985163614266	2.73981265653929	0.0978762408881211	0.579713220858044
Stmn2	Stmn2	-0.063527238852704	11.2587349851222	2.73938763781811	0.0979022771076127	0.579713220858044
Slc4a7	Slc4a7	0.121952605862096	5.13202480510989	2.73872198227232	0.0979430696777076	0.579713220858044
Pgr15l	Pgr15l	-0.428158889037357	-0.682364097178134	2.73633863861129	0.0980894001426478	0.579876442976414
Npr1	Npr1	0.208098540527325	2.21253104136066	2.73619563771301	0.0980980573961562	0.579876442976414
Pde3a	Pde3a	0.118722080704375	3.8029771174578	2.73540522793452	0.0981466028598321	0.579876442976414
Btd	Btd	-0.0872140391418782	4.13039292344703	2.73323458956537	0.0982745170362665	0.579876442976414
Rnf44	Rnf44	0.101029978821184	6.27159987084847	2.73212971069689	0.0982865071752684	0.579876442976414
Zdhhc15	Zdhhc15	0.107779138790275	2.86116303606218	2.73256738123948	0.0983211140674352	0.579876442976414
Ttc38	Ttc38	0.188394963152969	0.87801134199961	2.73233714049982	0.0983352873836613	0.579876442976414
Pik3ca	Pik3ca	0.0562547958113723	6.94831874881173	2.7322210542345	0.0983424343462445	0.579876442976414
Rnf181	Rnf181	0.0718208856827046	5.09384473356311	2.72764796820115	0.098624432183882	0.581019536883458
Ube2v2	Ube2v2	-0.0490379826115768	6.41240207499318	2.72742012897398	0.0986385048693659	0.581019536883458
Arhgap22	Arhgap22	-0.12348096921834	2.34308216963304	2.72562296228135	0.0987495234036721	0.581019536883458
Fuz	Fuz	-0.0769179123682231	4.95529632348037	2.72537457899281	0.0987649482104372	0.581019536883458
Csrnp3	Csrnp3	0.123427423884297	5.28690227081283	2.72530897297059	0.0987691302179863	0.581019536883458
Zfp53	Zfp53	0.140274019093076	2.3203203370922	2.72297649549629	0.0989132226098618	0.581443855536472
Polr1e	Polr1e	0.0628557564491022	4.67727807110256	2.72263670211954	0.0989344638077758	0.581443855536472
Vwa5b2	Vwa5b2	-0.19195982649754	6.20224492829127	2.71748834266198	0.0992540851048722	0.582047655298253
Utp14a	Utp14a	0.0713254632683673	4.62188097706871	2.71667181076318	0.0993048880643145	0.582071527428009
Sh3bp2	Sh3bp2	0.0732851432751554	5.18584943046332	2.71579479980289	0.0993594701273884	0.582117642887782
Rps27	Rps27	-0.119291491022442	7.04851482004029	2.71151547764744	0.0996263065882817	0.584212527605993
Dipk1a	Dipk1a	-0.0594261515252136	5.8163807887639	2.71130137107686	0.0996396777066244	0.584212527605993
Stk3	Stk3	0.132616001295364	2.53984221128785	2.70749036505207	0.0998780060930979	0.585234840599616
Il6st	Il6st	0.0620849652313261	6.22517246897424	2.70561603564286	0.0999956497306526	0.585371866324656
Prtn3	Prtn3	-0.153512080201243	1.78873573023086	2.70517625714366	0.10002302684645	0.585371866324656
Pde1b	Pde1b	-0.13027473326871	5.70418959234579	2.70459822302689	0.100059287081497	0.585371866324656
Hdac11	Hdac11	-0.0716705106526838	6.11713490864476	2.7043958125214	0.100071987727057	0.585371866324656
Socs3	Socs3	0.205621686847751	0.811642773777182	2.70287767878556	0.100167282234544	0.585648262584754
Lgr5	Lgr5	-0.227903197828933	1.18839197961219	2.70214854385159	0.10021311538049	0.585648262584754
Pgr	Pgr	-0.114120208762072	3.32967361099323	2.69993712824127	0.100352203095782	0.585977305902885
Rab3a	Rab3a	-0.062581199440021	8.67810434807173	2.69920481373728	0.100398309572052	0.585977305902885
Bmpr2	Bmpr2	0.10764151472471	6.87365456453913	2.69901421354007	0.100410313278245	0.585977305902885
Cgnl1	Cgnl1	0.133624308274273	4.70566098155766	2.69783670767924	0.100478203079878	0.585984858903394
Cog6	Cog6	0.0775688808048546	4.5715356462464	2.69750308768316	0.10050553804402	0.585984858903394
Smim17	Smim17	-0.0939981915355489	3.29532123719723	2.69590603926457	0.100606284449571	0.586135063403106
Cep97	Cep97	0.128067841741856	3.13485308960428	2.69509261515643	0.100657639990698	0.586135063403106
Igfbp3	Igfbp3	0.133609975439267	5.17092277621712	2.69467745058368	0.100683863647792	0.586135063403106
Daam1	Daam1	0.0488752210723605	8.25417941742276	2.69411800990385	0.100719209420234	0.586135063403106
Adam9	Adam9	0.0573984957610863	5.63744985768466	2.69283342398429	0.100800423720454	0.586322935993024
Ubl4a	Ubl4a	-0.0600890077466369	5.89265043882301	2.69211858553703	0.100845648928042	0.586322935993024
Mrpl53	Mrpl53	-0.0651372063270288	5.22264306159564	2.69098800822915	0.100917158278856	0.586496641131269
Vdac1	Vdac1	-0.0518491428784039	8.20084091744848	2.68878244541899	0.101057007012534	0.586577294290972
Slc8a1	Slc8a1	0.0778604007112829	7.81072204238477	2.68686850855574	0.101190046506543	0.586577294290972
Calm2	Calm2	-0.0737519923148279	10.3549744973688	2.68635207811604	0.101211285208001	0.586577294290972
Mcl1	Mcl1	0.0521610592114728	6.62567237946828	2.68564478730286	0.101256231932911	0.586577294290972
Gp5	Gp5	-0.283069148831501	-0.08719782008965	2.68557884388608	0.101260423600164	0.586577294290972
Ndufb8	Ndufb8	-0.0892835286006794	7.03577402568983	2.68556834292288	0.101261218240151	0.586577294290972
Lonrf3	Lonrf3	0.150001699424774	2.32680188981665	2.68500766074828	0.1012967385588	0.586577294290972
Fam102b	Fam102b	-0.0678558452607234	7.22520725247576	2.68412266139887	0.101353033599743	0.586577294290972
Colgalt1	Colgalt1	0.0489257454489765	5.97489982344775	2.684022344754	0.101359053291571	0.586577294290972
Pusl1	Pusl1	0.102500803896526	3.69220654728838	2.68142515463467	0.10152483380976	0.586895535215038
Sox2	Sox2	0.0567226035658658	6.22451263267927	2.6809815379349	0.101552817571421	0.586895535215038
Gjb6	Gjb6	0.367094006227689	2.60161005062783	2.68042781473387	0.101588943504032	0.586895535215038
Klf9	Klf9	-0.1402011910928	1.75172691002894	2.67968071516563	0.101636104077197	0.586895535215038
Necap1	Necap1	-0.0443412819757761	7.44967642150269	2.67947028885701	0.101649535272878	0.586895535215038
Acp2	Acp2	-0.0415344600570234	5.79899356747453	2.67646539173435	0.101841545232194	0.587486188909463
Pfkm	Pfkm	-0.0399872288523556	7.9257151655279	2.67631343882938	0.101851265401891	0.587486188909463
Mrpl50	Mrpl50	0.0839821451603876	3.81688242014366	2.67565974768098	0.101893092487129	0.587486188909463
Tmem200a	Tmem200a	0.0785614221989615	4.85221382621148	2.67326133395684	0.102046718972218	0.587723222467412
Scel	Scel	-0.320227060684323	-0.546526890404269	2.67311035835803	0.102056397301565	0.587723222467412
Zdhhc14	Zdhhc14	-0.0791022884840588	5.235133887706	2.67281217124089	0.102075516797859	0.587723222467412
Rps2	Rps2	-0.0752111793736279	9.88020395052946	2.67137392766512	0.102167790746801	0.587832540880127
Hes1	Hes1	0.115156780372435	2.3651580909498	2.67104775601169	0.102188729756271	0.587832540880127
Mrpl1	Mrpl1	-0.0779746225646563	3.93061235750055	2.66942395428245	0.102293043667872	0.588151419764995
Snx9	Snx9	0.0735304368120472	4.03125341019631	2.66320561325119	0.102693582116475	0.58927693835021
Tmem81	Tmem81	0.241125548893022	0.0400914785760298	2.66226775864342	0.102754140691765	0.58927693835021
Tstd2	Tstd2	0.0794470343775892	4.34210953866766	2.66168856192367	0.102792852091922	0.58927693835021
Cpsf3	Cpsf3	0.0498460811350753	5.50823103825034	2.66124139179128	0.10282045938209	0.58927693835021
Mid1ip1	Mid1ip1	-0.0657348832917989	6.23842946942044	2.66106640161944	0.102831767167495	0.58927693835021
Snx33	Snx33	0.140141414085295	2.53674276979483	2.66105517360347	0.102832490890884	0.58927693835021
Riiad1	Riiad1	-0.233837604310245	0.573306202777922	2.66074237816137	0.102852720072328	0.58927693835021
Tlr6	Tlr6	0.23768159194268	0.31224238518713	2.65928246575207	0.102947165647518	0.58927693835021
Nsd1	Nsd1	0.0852492944212645	8.0825100708873	2.65851542497018	0.102996825256059	0.58927693835021
Nt5e	Nt5e	0.198526750156341	1.11680077310393	2.65841333246543	0.103003436912785	0.58927693835021
Mgat2	Mgat2	0.0686882709704218	5.65258774569635	2.65774882757245	0.103046417759404	0.58927693835021
Adamts2	Adamts2	0.152911677064281	3.24686894058032	2.65735302390316	0.103072121481196	0.58927693835021
Polr3b	Polr3b	0.0497628154978843	5.28794475570334	2.65680719672026	0.103101033609434	0.58927693835021
Slc25a29	Slc25a29	0.0814927521832217	4.45182626479605	2.65552654775642	0.103190589512609	0.589351635483342
Polr1a	Polr1a	0.0929685314768829	5.57546519481884	2.6552493768909	0.103208565650645	0.589351635483342
Kcnip1	Kcnip1	-0.0723708814405197	5.36897438820338	2.65432322608687	0.103268813293877	0.589421889668853
P2rx4	P2rx4	-0.109059803851947	2.67695319225087	2.65195491534266	0.103422638906812	0.589875578283537
Arhgef28	Arhgef28	-0.0833747426264836	5.3484038343177	2.65165494045341	0.103442154962922	0.589875578283537
Fmnl1	Fmnl1	-0.0797537346926365	5.55216131448756	2.64921335822512	0.103601152329711	0.590059292445216
Ptpn9	Ptpn9	0.0460162889396553	5.98610773305864	2.64905658346165	0.103611370183096	0.590059292445216
Lrrc55	Lrrc55	0.0755344803275923	5.13300290491261	2.64900431485422	0.103614777228574	0.590059292445216
Pou3f1	Pou3f1	0.122845586651486	7.02719588534494	2.64750840866731	0.103712272232053	0.590336688247867
Mcfd2	Mcfd2	0.0767037503607632	5.0445775079718	2.64561164244463	0.103836185707182	0.590772498635253
Mrpl2	Mrpl2	-0.0843003027011238	4.03979202194085	2.64454934573123	0.103906208950586	0.590901433054907
Rnf40	Rnf40	0.0453288294057117	6.2577989575892	2.642356526389	0.104049104199085	0.591406925731466
Scyl1	Scyl1	-0.0431370742625753	6.39106040673156	2.64080035843878	0.104151063673375	0.591406925731466
Pvalb	Pvalb	-0.653937746508338	-0.523873177197351	2.64032586389497	0.104182172882914	0.591406925731466
RGD735065	RGD735065	-0.0552050389939782	5.7652310876851	2.64028739576947	0.104184695418591	0.591406925731466
Fen1	Fen1	-0.0728488706092199	3.99294519756887	2.63816418311345	0.104324031865932	0.591763174970909
Kirrel3	Kirrel3	-0.0756889905295511	6.69614599776058	2.63722984058752	0.104385413028263	0.591763174970909
Fzr1	Fzr1	-0.0452539002926506	7.086813250804	2.63716403442324	0.104389730964893	0.591763174970909
Ankrd42	Ankrd42	0.0721916448505255	4.22113567450384	2.63634967434276	0.104443271293772	0.591792620460322
Rab5a	Rab5a	-0.0534658945231166	5.75243862181137	2.63443458071784	0.104569292555845	0.591792620460322
Gap43	Gap43	-0.0682049691194482	10.5614529046089	2.63273734042688	0.104681520163494	0.591792620460322
Gba	Gba	-0.0555297567510507	5.40049996365351	2.63287598216822	0.104671958731705	0.591792620460322
Sympk	Sympk	0.0386805857260473	6.69386829645754	2.63245598406277	0.104699887885638	0.591792620460322
Pdcd6ip	Pdcd6ip	0.0455128881057837	6.22340435864526	2.63168060204483	0.104750124015453	0.591792620460322
Nptx2	Nptx2	-0.104563777268771	4.95234590190657	2.63102385534673	0.104794117572317	0.591792620460322
Smg9	Smg9	0.0529545784443284	4.96873740688379	2.63035832183458	0.104838192665959	0.591792620460322
Slc45a3	Slc45a3	0.266906546287729	0.177507101569128	2.63009095382213	0.104855708650359	0.591792620460322
Cln5	Cln5	0.115650240327162	2.6859795129785	2.62977049866885	0.104876871892533	0.591792620460322
Sox1	Sox1	0.0735946367434858	6.90843807394319	2.62904813978163	0.104916868787227	0.591792620460322
Olfml2a	Olfml2a	0.182270085995788	4.74945153450842	2.62703742720871	0.105057574679506	0.592219637045297
Gmfb	Gmfb	-0.0568823887164702	6.89798277772617	2.62274374982954	0.105342149192276	0.593443943830852
Cox7a2	Cox7a2	-0.10130436189594	5.94193242084479	2.62259361965127	0.105352114737909	0.593443943830852
Hoga1	Hoga1	-0.151035858307558	1.26163340066742	2.62071697649465	0.105476772246478	0.593625833113931
Sdhaf3	Sdhaf3	-0.136449163923447	1.55920026658855	2.6206750356085	0.105479560151766	0.593625833113931
Gpr182	Gpr182	0.139849878688949	1.63794194320319	2.61773757737134	0.105675013707549	0.594457585215186
Mcrip2	Mcrip2	-0.132120495536889	2.54133737513706	2.61621217095441	0.105776668800367	0.594761374665921
Rpl34	Rpl34	-0.0651432918182006	7.94276380190626	2.61520104858424	0.105844109581367	0.594872502363384
Rgs9	Rgs9	-0.167565721371117	3.85740802804437	2.61417502057144	0.10591259325707	0.594980985893049
Bdnf	Bdnf	-0.161019029031305	2.6774205733791	2.61307849672986	0.105985768967572	0.595007679787737
Fhdc1	Fhdc1	0.0863940880236298	4.14038959068203	2.61269856797881	0.106011226430082	0.595007679787737
Spryd3	Spryd3	-0.0585041480832082	7.38405793862336	2.60847163764304	0.106294160374616	0.596327445590868
Cercam	Cercam	-0.126779914677591	2.40269298945984	2.606814158947	0.106405332137892	0.596543228457632
Plekhm1	Plekhm1	0.0688030494810647	5.43636307882398	2.60569038789242	0.106480777897444	0.596543228457632
Cdh5	Cdh5	0.162301002211111	4.3950696859099	2.60430055474928	0.106574168178962	0.596543228457632
Trappc5	Trappc5	-0.0664698848867625	6.00732757175132	2.60346103895186	0.106630623237729	0.596543228457632
Slc1a4	Slc1a4	0.0616401308643239	6.14022731040598	2.60320729677571	0.106647693136105	0.596543228457632
Ephb2	Ephb2	0.0664875492655498	6.93903364215047	2.60254404552055	0.106692325939742	0.596543228457632
Isoc2b	Isoc2b	0.179068898051644	1.53941005708179	2.6019442197917	0.106739442204428	0.596543228457632
Cyb5d2	Cyb5d2	-0.127500281512636	1.93394648069791	2.60158891422014	0.106756771223988	0.596543228457632
Adamts9	Adamts9	0.147985147905661	3.13552740243005	2.60034999228847	0.106840118556551	0.596543228457632
Alkbh8	Alkbh8	0.0966319387163189	4.06387776107393	2.59913035826833	0.106922322595278	0.596543228457632
Ccdc81	Ccdc81	-0.298894727397677	-0.321970758045169	2.59882987352295	0.106942646166339	0.596543228457632
Sox10	Sox10	-0.0850202577343658	4.91095049175246	2.59753757858498	0.107029892872899	0.596543228457632
Soat1	Soat1	0.130878890751401	2.7398352709182	2.59687816575476	0.107074441934156	0.596543228457632
Rtca	Rtca	-0.0594882564860282	6.07291452503573	2.59620032900011	0.107120256698524	0.596543228457632
Trpm2	Trpm2	-0.140823230727473	3.75440671475002	2.59608303561106	0.107128198720711	0.596543228457632
Szrd1	Szrd1	0.0517447174570004	6.49958664952347	2.59561678975273	0.107159715243209	0.596543228457632
Cmbl	Cmbl	0.169410983168121	2.19955586573287	2.59410038872264	0.107262327899154	0.596543228457632
Eci3	Eci3	0.256968730367801	1.18822607707513	2.59283453089567	0.107348069247257	0.596543228457632
Rnf130	Rnf130	0.0527160225261859	5.68833944759304	2.59280349083514	0.107350172652977	0.596543228457632
Loxl4	Loxl4	0.51035010463056	-0.895852877497527	2.5922892766265	0.10738501778371	0.596543228457632
Flnc	Flnc	0.124213607787619	4.43778695078368	2.58871363820478	0.107627714419098	0.596543228457632
Akap13	Akap13	0.104565542460607	5.15843082375728	2.58801930248201	0.107674910948087	0.596543228457632
Wdr86	Wdr86	0.205994864177805	1.47629429570913	2.58780570229965	0.107689434700317	0.596543228457632
Smad5	Smad5	0.0724269046396554	5.21799216578193	2.58726676710169	0.107726087818123	0.596543228457632
Rbbp8	Rbbp8	0.0584419078952707	5.42084652182708	2.5863345294684	0.107789525589128	0.596543228457632
Gnpnat1	Gnpnat1	0.0825244893915405	4.25925178649392	2.58621934748047	0.107797366278061	0.596543228457632
Snrpe	Snrpe	-0.0815151036559872	4.81940329641002	2.58620149329899	0.107798581707147	0.596543228457632
Atp11a	Atp11a	0.100394701485457	5.90119182411417	2.58580649599659	0.107825475123086	0.596543228457632
Hagh	Hagh	-0.0868986086318001	4.39189874806626	2.58263281626739	0.107998777006678	0.596543228457632
Nkain3	Nkain3	0.132075281369628	3.27799040481437	2.58293281577315	0.108021351711411	0.596543228457632
Ppp1r16a	Ppp1r16a	-0.0523850531132854	6.47351316070113	2.58272240191971	0.108035709342726	0.596543228457632
Aqp11	Aqp11	-0.0920377098957605	3.98320515707801	2.58266643523375	0.108039528593523	0.596543228457632
Kctd15	Kctd15	0.0645427703293736	6.29142471307612	2.58225521030225	0.108067595748394	0.596543228457632
Ppm1k	Ppm1k	-0.096194267059684	4.46450908709654	2.58224583015839	0.108068236060632	0.596543228457632
Zfyve9	Zfyve9	0.0452486476815241	6.57685992618828	2.58203678577526	0.108082507037621	0.596543228457632
Htr2a	Htr2a	0.121941842991128	2.88501687811074	2.58052716072639	0.108185627107646	0.596543228457632
Magi2	Magi2	0.0612122205802231	6.72216058039908	2.57963520310419	0.108246508079733	0.596543228457632
Skiv2l	Skiv2l	-0.0475380448421768	5.79227436265655	2.57959126404574	0.108249610963085	0.596543228457632
Mmrn2	Mmrn2	0.125007389256105	2.89581732995816	2.57867657802641	0.10831218501866	0.596543228457632
Spaca9	Spaca9	-0.229930370666878	0.325936031444282	2.57836837473418	0.108333209653054	0.596543228457632
Nell3	Nell3	0.271404539677445	0.282289272899038	2.57774767351111	0.108375772151162	0.596543228457632
Gck	Gck	-0.244144829043651	1.34990756066957	2.57759308806318	0.108386358128816	0.596543228457632
Rara	Rara	0.0572935778568112	4.84683220820113	2.57715551423579	0.108416329239724	0.596543228457632
Ntn4	Ntn4	-0.0878528089842486	3.94456042821795	2.57686334361042	0.108436346206773	0.596543228457632
Dusp3	Dusp3	-0.0722972511429312	5.86565477712869	2.57447278612297	0.108600278741824	0.596777112833574
Srfbp1	Srfbp1	0.0658441823036124	4.48771627807765	2.57419871207885	0.108619022118751	0.596777112833574
Prepl	Prepl	-0.05541255166158	7.59310944915075	2.5741515307979	0.108622330948549	0.596777112833574
Lrsam1	Lrsam1	-0.0801059643292967	5.1361430369013	2.57147267305433	0.108806396902596	0.597041263651912
Snhg4	Snhg4	0.183210950307187	1.42595689780767	2.57143527444265	0.108808969027373	0.597041263651912
Sh3bp1	Sh3bp1	0.158084431631382	2.20564894863074	2.57050888708891	0.10887270351784	0.597041263651912
Pcbp3	Pcbp3	-0.0738542396827719	5.89680702934252	2.57025003149397	0.108890519845617	0.597041263651912
Etnk2	Etnk2	-0.104150651816029	3.45829277101151	2.56997189096723	0.108909667073155	0.597041263651912
Clstn2	Clstn2	0.119677529598915	6.32923049759424	2.56836597982813	0.109020290555453	0.597119297819605
Synpr	Synpr	-0.132054181513949	6.47496785978438	2.56784986049633	0.109048975148343	0.597119297819605
Esam	Esam	0.110902778342865	4.29852932466896	2.56768155893133	0.109067474531608	0.597119297819605
Lfng	Lfng	-0.0848406741737929	5.11204517253317	2.56646579714874	0.109151344691375	0.597316370710038
Parp6	Parp6	-0.0362461539916337	7.99038412469285	2.56477813164163	0.109267886474924	0.597571623490718
Metap1d	Metap1d	0.0885687735866169	3.53870329526752	2.56440348736773	0.109293776132549	0.597571623490718
Nsun6	Nsun6	-0.0724482394544665	3.85254473501478	2.56279206661837	0.109405209669711	0.597629541932896
Trpm3	Trpm3	0.102136791285624	6.42516981227109	2.56261706297579	0.109417319098499	0.597629541932896
Atp6ap2	Atp6ap2	-0.0584722427048172	6.79803543237899	2.56148264554984	0.109495851182557	0.597629541932896
Lsr	Lsr	0.0994275878231374	4.08031986404327	2.56148103805201	0.109495963200978	0.597629541932896
Erc2	Erc2	-0.058556403278942	6.78588183545674	2.56070455698224	0.109549751580484	0.597661674888369
Pkm	Pkm	-0.0394752263797312	8.90402186555483	2.55861744239576	0.109694476012465	0.597743026717773
Smarcc1	Smarcc1	0.0514996359995022	7.08579231831233	2.55843361199383	0.109707233224285	0.597743026717773
Nppc	Nppc	0.183288492780562	1.77397289978922	2.55805909372371	0.109733228579542	0.597743026717773
Tle4	Tle4	0.0575957669611885	7.3270623377423	2.55772687599685	0.109756290516905	0.597743026717773
Ulk1	Ulk1	0.0692621185602572	6.97108382391236	2.55376933620884	0.11003146463016	0.598639118575214
Nat2	Nat2	-0.126821111472876	2.24091254321108	2.55309404050566	0.11007849423059	0.598639118575214
Tyw1	Tyw1	0.0641187290915017	4.56242020425729	2.55203381043147	0.110152376605272	0.598639118575214
Capza1	Capza1	0.0479634298438671	6.53354338181932	2.55182212723818	0.110167134080587	0.598639118575214
Slc9a4	Slc9a4	-0.263894183318895	0.380270398561177	2.55143419755161	0.110194184590966	0.598639118575214
Wdr55	Wdr55	0.0850685825638693	3.91934864651958	2.55122595491365	0.110208708452898	0.598639118575214
Mical3	Mical3	0.0893205034017099	6.87943616168955	2.54886612380639	0.110373441118542	0.599026128660251
Msn	Msn	0.0974605259429233	4.76834670202804	2.54882980273682	0.110375979408425	0.599026128660251
Rbm4	Rbm4	0.0712420232162789	5.1058236873221	2.5462905592378	0.110553544091311	0.599194114490391
Ace2	Ace2	0.264821386617683	1.14969899091818	2.54624254594781	0.110556904598807	0.599194114490391
Nf1	Nf1	0.112132539137404	6.82421345411545	2.54602169790041	0.110572364162662	0.599194114490391
Rfc3	Rfc3	-0.0709232117661921	4.52383139180699	2.54456315509687	0.110674517891196	0.599194114490391
Apaf1	Apaf1	-0.0576447874998983	4.62781418541151	2.54354203823767	0.11074609735412	0.599194114490391
Mtmr6	Mtmr6	-0.0364327181729771	7.14837289492826	2.54325989764752	0.110765894168151	0.599194114490391
Dis3l	Dis3l	-0.0542445436662318	5.25205378425755	2.54298821694617	0.110784941088148	0.599194114490391
Nsd3	Nsd3	0.0895639325084732	6.48201012447475	2.54232984538821	0.110831137226579	0.599194114490391
Twf1	Twf1	0.0503655096112557	6.06889036828983	2.54221574738796	0.110839145226907	0.599194114490391
Hic1	Hic1	0.210852283999664	2.80325907489324	2.54051221056245	0.110958785769312	0.599389026118401
Bap1	Bap1	-0.0383596512784294	8.23644694881233	2.54033443587472	0.110971279180372	0.599389026118401
Pnpo	Pnpo	-0.0980527386653806	4.56755600243498	2.5396509612181	0.111019325911474	0.599389065078952
Dctn5	Dctn5	-0.0517000856650434	6.12828705860684	2.53497532893724	0.111348628914494	0.600736957296058
Gstm2	Gstm2	0.274441410063829	2.28609970700461	2.53408193594176	0.111411670095219	0.600736957296058
Kcnmb2	Kcnmb2	0.131618099493021	1.95659739117221	2.53295861598989	0.111420375378925	0.600736957296058
Gabbr1	Gabbr1	-0.0530828816215397	10.0751694472431	2.53277043225642	0.111504268855849	0.600736957296058
Zfp597	Zfp597	0.0807251114599263	4.41042651991101	2.53264932130174	0.111513432486613	0.600736957296058
Fosb	Fosb	-0.313780478701432	0.514882348811999	2.53129712571322	0.111608435267008	0.600736957296058
Jmjd6	Jmjd6	0.0611789843255777	4.93134628412616	2.52959160894319	0.111729130242162	0.600736957296058
Crem	Crem	0.0993287670806363	3.24177520710678	2.52901761104923	0.111769782696421	0.600736957296058
Oxr1	Oxr1	-0.0536181558641782	6.84057510064656	2.52878055315171	0.111786576740432	0.600736957296058
Slc38a5	Slc38a5	0.10065945963547	5.22933476892567	2.52790476808538	0.111848643218011	0.600736957296058
Asb2	Asb2	-0.259066675341533	3.27017465976116	2.5272935467318	0.11189198507322	0.600736957296058
Smyd2	Smyd2	-0.0407882344820812	6.62344624838245	2.52682237295329	0.111925407648972	0.600736957296058
Prox1	Prox1	0.150544750981389	2.96101522736416	2.5265150396158	0.11194721417328	0.600736957296058
Rpl41	Rpl41	-0.112926787174807	8.86872433293809	2.5260507771341	0.111980164216093	0.600736957296058
Plod1	Plod1	0.119060144142736	4.32163713884563	2.5258953645079	0.111991196815792	0.600736957296058
Top1mt	Top1mt	0.0627073831126741	4.55515567677616	2.52497648510133	0.112056451091355	0.60082868081944
Mtus2	Mtus2	0.055662438350201	6.15804042909242	2.5229261734246	0.112202205076708	0.601068110754509
Ntm	Ntm	-0.0541819726512202	7.56258453080237	2.52263018213202	0.112223263960815	0.601068110754509
Atp5mc1	Atp5mc1	-0.0950782691724336	6.49025296103761	2.52231591583063	0.112245627799792	0.601068110754509
Setmar	Setmar	0.166247877467128	1.30225575617272	2.51952089970735	0.112444743081114	0.601876044368539
Phc2	Phc2	0.0489012928521391	6.98678428047718	2.51709890022318	0.112617527938574	0.602542408271695
Kcnj16	Kcnj16	-0.242957216125306	1.47987630204796	2.5159679111284	0.112698417652886	0.602652956171792
Lmtk2	Lmtk2	0.0547089197622255	8.01486430268699	2.51545817109878	0.112734791993665	0.602652956171792
Bex1	Bex1	-0.0839279663095816	6.97059685318489	2.51001184748672	0.113125081231139	0.60432551452042
Foxd1	Foxd1	0.276471540029013	2.29557872524053	2.50868296524681	0.1132205185697	0.60432551452042
Krt71	Krt71	-0.44341502568272	0.337616494267609	2.50832576893589	0.113246186733324	0.60432551452042
Ccsap	Ccsap	-0.080970216454157	4.46702781305559	2.50796876701621	0.113271847335441	0.60432551452042
Olfm3	Olfm3	-0.0660382801603785	5.82417627623467	2.50739149286262	0.113313354234766	0.60432551452042
Aptx	Aptx	0.0700567516020441	4.39405433990936	2.50704495694023	0.113338276751125	0.60432551452042
Cflar	Cflar	-0.0585843317381635	4.55150312097415	2.50584273203971	0.113424795251285	0.604529479864966
Dcaf17	Dcaf17	0.0988979652256536	3.71517286248568	2.50395291718052	0.113561084845411	0.604756428228936
Dlst	Dlst	-0.0479459695511561	6.13314982704557	2.50390349420362	0.113564503583845	0.604756428228936
Ptprcap	Ptprcap	-0.229794093825771	-0.311526418823731	2.50319868084065	0.113615255222899	0.604768574836224
Cfap300	Cfap300	0.139574754397377	3.14210339801149	2.50100554880657	0.113773632507866	0.605264380789838
Tns2	Tns2	0.102428966663263	4.52086589323536	2.50032326547004	0.113822932126724	0.605264380789838
Kat6a	Kat6a	0.0756684946772827	7.46810016250538	2.49989440873975	0.113853931370841	0.605264380789838
Snx14	Snx14	-0.0953207657611581	4.83551620128502	2.49689128100557	0.114071268780615	0.606161507911298
Ppt2	Ppt2	0.0659947191946365	4.38576186879149	2.4934568371424	0.114320381569521	0.607226549996982
Prrc2b	Prrc2b	0.0704212892206049	10.4306186186394	2.4905549560963	0.11453133326538	0.607905664640404
Ppp1cc	Ppp1cc	-0.0439287204335165	7.99448758384237	2.48770008876203	0.114739285233644	0.607905664640404
Rab40b	Rab40b	-0.123632166131733	3.63367514755545	2.48762103277083	0.114745050982147	0.607905664640404
Usp21	Usp21	0.0532449282956498	5.82939802383088	2.48713308356487	0.114780637446931	0.607905664640404
Samd4a	Samd4a	0.125687904281911	2.71116600322272	2.48653840415913	0.114824024474353	0.607905664640404
Dyrk1b	Dyrk1b	0.100725883920642	4.90028945164628	2.48636438885966	0.114836723823153	0.607905664640404
Kdm5b	Kdm5b	0.074648612339091	7.74119960814811	2.48563038828968	0.114890307024826	0.607905664640404

Ndn	Ndn	-0.082417589203715	9.21828216663268	2.48251801032177	0.115244667932503	0.607905664404
Fbxo30	Fbxo30	0.0610481919004262	5.47111378866426	2.48248545824563	0.115247048440481	0.607905664404
Map10	Map10	0.0967254323303732	2.26178949248815	2.48247497201718	0.115247815211443	0.607905664404
Zfp346	Zfp346	0.0430047233230933	5.8682945669975	2.48224857604891	0.115264373148385	0.607905664404
Ggt7	Ggt7	-0.0462246564630287	6.59196661005522	2.48278366688687	0.115298383279277	0.607905664404
Gpr12	Gpr12	0.0628599151081962	4.98321116094772	2.48247380581049	0.115121057126843	0.607905664404
Psmc5	Psmc5	-0.0532867756562929	6.83157740498819	2.48234714867031	0.115130326598883	0.607905664404
Rab15	Rab15	-0.0588816995029226	6.87158539546449	2.48072822372305	0.115248880884841	0.608110704906922
Podxl2	Podxl2	-0.0462952888487547	8.82146684494932	2.4793759395233	0.115348012273736	0.608110704906922
Syt11	Syt11	-0.0463016231851509	7.97625572378659	2.47936881397748	0.115348461530843	0.608110704906922
Pf4	Pf4	0.307584115361508	0.350255742805389	2.47915642456674	0.115364113049185	0.608110704906922
Bcl2l11	Bcl2l11	-0.116690857196566	3.61557624999456	2.4769609864905	0.115525278483094	0.608703082615527
Ppfia3	Ppfia3	-0.0383491415280642	7.79972658101095	2.47589493665311	0.115597008237344	0.608793267629059
Camk2n1	Camk2n1	-0.0959911086383886	6.63477160636252	2.47471422757634	0.115690468656326	0.608793267629059
Mtf1	Mtf1	0.0613462495981379	4.57380623855588	2.47414190707744	0.115732589467492	0.608793267629059
LOC1001	LOC100125	-0.0719084620561787	7.55142894182637	2.47407428156552	0.115737567589655	0.608793267629059
Msh5	Msh5	0.281917229301242	0.216586521844358	2.47085403281304	0.115974893864286	0.609773479131351
Tll2	Tll2	0.240682120107535	0.992842396191935	2.47022034078168	0.116021658009203	0.609773479131351
Dnel	Dnel	0.0634105480104091	5.09178240729418	2.46722193419243	0.116243217644599	0.610137038130893
Efs	Efs	0.0711500861793823	4.40915499748108	2.46685776059409	0.116262760375043	0.610137038130893
F2r	F2r	0.0594310442768577	6.04901336295007	2.4667930034832	0.116274950423875	0.610137038130893
Gnb3	Gnb3	0.58663475824245	2.47004075079293	2.46619023129477	0.116319560380284	0.610137038130893
Zfp777	Zfp777	-0.0441979847423272	5.58623978566508	2.4627433575311	0.116575018777913	0.610137038130893
Fbxo21	Fbxo21	0.0346526927309424	7.47139634560047	2.46232604502165	0.116605990239299	0.610137038130893
Dysf	Dysf	0.115348875368496	2.83252951521108	2.46191030554744	0.116636852995942	0.610137038130893
B4galt1	B4galt1	0.153443760879412	2.0576736122629	2.46189916505723	0.116637680098814	0.610137038130893
Fam53a	Fam53a	0.0900185658309647	3.22290229074315	2.46183209571485	0.11664265997446	0.610137038130893
Slc30a7	Slc30a7	0.0755389687204781	3.88963284867163	2.46176355012861	0.116647749657813	0.610137038130893
Abcg2	Abcg2	0.209882415024851	2.40886414816429	2.46110716828841	0.116696500094792	0.610137038130893
Gdap1l1	Gdap1l1	-0.0500022530208685	6.58922041382231	2.46025047857484	0.116760163547175	0.610137038130893
Upf3a	Upf3a	-0.0593839874643568	4.87952524751541	2.46013600577273	0.116768671038399	0.610137038130893
Rilpl1	Rilpl1	0.0750744767177582	4.35489332862366	2.46004486520338	0.116775448586245	0.610137038130893
[illegible]	[illegible]	[illegible]	[illegible]	[illegible]	[illegible]	[illegible]

Map4k4	Map4k4	[illegible]	[illegible]	[illegible]	[illegible]	[illegible]
Stag1	Stag1	[illegible]	[illegible]	[illegible]	[illegible]	[illegible]
Camk2a	Camk2a	[illegible]	[illegible]	[illegible]	[illegible]	[illegible]
Phkg2	Phkg2	[illegible]	[illegible]	[illegible]	[illegible]	[illegible]
Setd4	Setd4	[illegible]	[illegible]	[illegible]	[illegible]	[illegible]
Nt5c	Nt5c	[illegible]	[illegible]	[illegible]	[illegible]	[illegible]
Slc39a8	Slc39a8	[illegible]	[illegible]	[illegible]	[illegible]	[illegible]
Tmem117	Tmem117	[illegible]	[illegible]	[illegible]	[illegible]	[illegible]
Cbfa2t3	Cbfa2t3	[illegible]	[illegible]	[illegible]	[illegible]	[illegible]
Arhgef18	Arhgef18	[illegible]	[illegible]	[illegible]	[illegible]	[illegible]
Copa	Copa	[illegible]	[illegible]	[illegible]	[illegible]	[illegible]
Cux1	Cux1	[illegible]	[illegible]	[illegible]	[illegible]	[illegible]
Wdr33	Wdr33	[illegible]	[illegible]	[illegible]	[illegible]	[illegible]
Magee2	Magee2	[illegible]	[illegible]	[illegible]	[illegible]	[illegible]
Ube2j2	Ube2j2	[illegible]	[illegible]	[illegible]	[illegible]	[illegible]
Epc1	Epc1	[illegible]	[illegible]	[illegible]	[illegible]	[illegible]
Phgdh	Phgdh	[illegible]	[illegible]	[illegible]	[illegible]	[illegible]
Ttc27	Ttc27	[illegible]	[illegible]	[illegible]	[illegible]	[illegible]
Mak	Mak	[illegible]	[illegible]	[illegible]	[illegible]	[illegible]
Vangl1	Vangl1	[illegible]	[illegible]	[illegible]	[illegible]	[illegible]
Spire2	Spire2	[illegible]	[illegible]	[illegible]	[illegible]	[illegible]
Kctd6	Kctd6	[illegible]	[illegible]	[illegible]	[illegible]	[illegible]
Rad54l2	Rad54l2	[illegible]	[illegible]	[illegible]	[illegible]	[illegible]
Picalm	Picalm	[illegible]	[illegible]	[illegible]	[illegible]	[illegible]
Gtf3c5	Gtf3c5	[illegible]	[illegible]	[illegible]	[illegible]	[illegible]
Mef2a	Mef2a	[illegible]	[illegible]	[illegible]	[illegible]	[illegible]
Paqr9	Paqr9	[illegible]	[illegible]	[illegible]	[illegible]	[illegible]
Axx1	Axx1	[illegible]	[illegible]	[illegible]	[illegible]	[illegible]
Tmem200c	Tmem200c	[illegible]	[illegible]	[illegible]	[illegible]	[illegible]
Ivns1abp	Ivns1abp	[illegible]	[illegible]	[illegible]	[illegible]	[illegible]
Tifa	Tifa	[illegible]	[illegible]	[illegible]	[illegible]	[illegible]
Ndufb5	Ndufb5	[illegible]	[illegible]	[illegible]	[illegible]	[illegible]
Kifc3	Kifc3	[illegible]	[illegible]	[illegible]	[illegible]	[illegible]
Rpl35al1	Rpl35al1	[illegible]	[illegible]	[illegible]	[illegible]	[illegible]
Yeats4	Yeats4	[illegible]	[illegible]	[illegible]	[illegible]	[illegible]
Tnrc18	Tnrc18	[illegible]	[illegible]	[illegible]	[illegible]	[illegible]
Fndc8	Fndc8	[illegible]	[illegible]	[illegible]	[illegible]	[illegible]
Slc26a8	Slc26a8	[illegible]	[illegible]	[illegible]	[illegible]	[illegible]
Ddrgk1	Ddrgk1	[illegible]	[illegible]	[illegible]	[illegible]	[illegible]
[illegible]	[illegible]	[illegible]	[illegible]	[illegible]	[illegible]	[illegible]

Notum	Notum	0.162104206546197	1.52809302352242	1.95061392362746	0.162520709969954	0.668973795928164
Igf2r	Igf2r	0.0982975763179922	6.60530874891683	1.95019903620755	0.162565404284218	0.668973795928164
Fis1	Fis1	-0.0681186930447304	6.49318163798279	1.94876752517462	0.162719723550109	0.669388061567659
Ptar1	Ptar1	0.123667229124468	2.85747525541013	1.94707547075744	0.162902345478292	0.669716877992283
Kmt5c	Kmt5c	0.111033964003676	5.27651014232808	1.9465816958363	0.162955582257349	0.669716877992283
Crocc	Crocc	-0.116825613965954	5.01378814712386	1.94653541016596	0.162960682983455	0.669716877992283
Rpl7a	Rpl7a	-0.0516842865244132	8.81390246272857	1.94547604968013	0.163075184726885	0.669966769785298
Hsp90ab1	Hsp90ab1	-0.0439056726470034	11.1913467867025	1.94496889985771	0.163130032859106	0.669971500981916
Coq8b	Coq8b	0.0598567005997036	5.30783857296413	1.94262340654637	0.163383971624667	0.670745996556514
Sipa1l2	Sipa1l2	0.0482932636758843	6.77517165753562	1.94223441156159	0.163426130442559	0.670745996556514
Fv1	Fv1	-0.0444529755243942	7.46662062715715	1.9416129773785	0.163493506525183	0.670801868107436
Cabcoco1	Cabcoco1	-0.216693287111105	1.09695012825174	1.94054341046153	0.16360954358462	0.670894716199477
Mto1	Mto1	-0.0535775663973486	4.58224434249518	1.94041319301258	0.163623677277548	0.670894716199477
Pnrc1	Pnrc1	0.0604388988620194	4.92380630558316	1.93943946612946	0.163729408917868	0.67093248041243
Dlgap4	Dlgap4	0.0375205541362938	8.0850534147918	1.93822229449808	0.163861685734719	0.67093248041243
Cox6b2	Cox6b2	-0.128206521612574	4.13229201186603	1.93813278396189	0.163871416993491	0.67093248041243
Micu1	Micu1	-0.0530828864118062	6.73570302100983	1.93802182714184	0.163883482078314	0.67093248041243
Abcb1a	Abcb1a	0.107114595433266	3.28759647954711	1.93785379525009	0.163901755253433	0.67093248041243
Fgf13	Fgf13	-0.0527407802554607	7.91309990130012	1.93734341250473	0.163957272760366	0.670939618999374
Eif3g	Eif3g	-0.0522404775244673	6.46933636989386	1.93569352304136	0.164027996279482	0.67100895436535
Fam210b	Fam210b	-0.0911482042823396	4.40023821076986	1.93515157333503	0.16419593712261	0.671269260856354
Ttc9	Ttc9	0.0425953918122143	6.41399448728324	1.93512136407837	0.164199229312623	0.671269260856354
Selenom	Selenom	-0.0966997885403132	5.67941518332708	1.93454540390324	0.164262011581717	0.671287539478171
Ccn2	Ccn2	-0.0948531711283106	3.57515575143572	1.93409314642541	0.164311328922524	0.671287539478171
Mrpl44	Mrpl44	-0.0606795828249497	4.86186734394922	1.93241436347622	0.164494543278974	0.671816175611052
Angr1	Angr1	0.287914117221072	0.120514210654758	1.93184420385283	0.164556820882796	0.671850606725998
Ptgds	Ptgds	0.313977467348597	7.8387684505061	1.92950968195733	0.164812098131809	0.67266440700615
Oip5	Oip5	-0.112327923821342	2.17042798657395	1.92903581452757	0.164863970235217	0.67266440700615
Rdh14	Rdh14	0.0583438607326264	4.27499107933167	1.92574326348083	0.165224906610895	0.673309368492286
Tnfrsf11a	Tnfrsf11a	-0.120541517505715	1.87248442599184	1.9256460709517	0.165235574781284	0.673309368492286
Krt19	Krt19	0.225704074724386	0.874860978062298	1.92538127021109	0.165264544175266	0.673309368492286
Gabrg1	Gabrg1	-0.0849181127940273	5.16510528119461	1.92468073994689	0.165341575451762	0.673309368492286
Fam216a	Fam216a	-0.0535760456184059	5.5449773363085	1.92409671757414	0.165405743296038	0.673309368492286
RT1-O1	RT1-O1	0.481561385877523	-0.512471455677535	1.92409293526181	0.165406158959964	0.673309368492286
Rhot1	Rhot1	0.0360811543498725	6.42783295394347	1.92403044274941	0.165413026859456	0.673309368492286
Stard8	Stard8	0.131123864474554	2.38297340255565	1.92365989291635	0.16545375681633	0.673309368492286
Ube2l6	Ube2l6	0.22743386112068	0.403847624952936	1.92245741133226	0.165584909435807	0.673597322395967
Maff	Maff	-0.149812861611983	0.850973525608001	1.9220350552783	0.165632490581555	0.673597322395967
St6galnac2	St6galnac2	0.305835192880343	0.215495768289548	1.92043661081335	0.165808536627927	0.67409355213641
Coq5	Coq5	-0.0736251458028346	4.25274472777065	1.91878040798007	0.165991166773731	0.674616229728613
Rhoj	Rhoj	0.092488726988825	3.25164702046865	1.91729089904231	0.166155617820653	0.674850504063076
Nxn	Nxn	0.0894085472364849	4.61368387089093	1.91712519349709	0.166173923928872	0.674850504063076
Sox14	Sox14	0.560575070597681	1.05280756201529	1.91626157011174	0.166269368992651	0.674850504063076
Ptpn21	Ptpn21	0.112302121831611	2.5916501375545	1.91591794809923	0.166307362511188	0.674850504063076
Gypc	Gypc	0.154226286386524	1.77708153088101	1.91549379871289	0.166354273463662	0.674850504063076
St6galnac6	St6galnac6	-0.0514466992925062	6.28015564805359	1.91481348014739	0.166429548275531	0.674850504063076
Dapk2	Dapk2	-0.227419469651713	-0.161591550130674	1.91405347477323	0.166513686465519	0.674850504063076
Dpm3	Dpm3	-0.144388189288433	1.96712812396895	1.91297419671432	0.166632465923689	0.674850504063076
Nampt	Nampt	0.0448581556133074	5.76449400544546	1.91285559054868	0.166635601667886	0.674850504063076
Tomm40l	Tomm40l	-0.0494021142057534	4.52169659349615	1.91249481430574	0.166687502524926	0.674915999954518
LOC500X	LOC500321	0.18952726656299	0.687603713632114	1.91227469264366	0.166710800911029	0.674915999954518
Ppp2r3c	Ppp2r3c	0.0588784788820254	4.24751836865248	1.9122454755461	0.166714040811399	0.674915999954518
Cpne5	Cpne5	-0.0894319872762916	6.86643419977487	1.91140342407684	0.166807447355219	0.674987408242913
Abcd1	Abcd1	0.0506209927422717	4.2267360192098	1.91051668363272	0.166905875904778	0.674987408242913
Fzd10	Fzd10	-0.163925221917229	3.733917483487	1.91034492396402	0.166924948984579	0.674987408242913
Pcyt1a	Pcyt1a	0.0541642686943657	4.68665858576819	1.91011217576126	0.166950798528063	0.674987408242913
RGD1560	RGD156054	-0.0625832855204714	3.99689611857284	1.90901552007357	0.16707265729067	0.674987408242913
Nrn1	Nrn1	-0.111847465443364	5.40613134780336	1.90877242618291	0.16709966831405	0.674987408242913
Mmp28	Mmp28	0.10225284160384	1.94713156931293	1.90867630124983	0.16711037140838	0.674987408242913
LOC3047	LOC304725	-0.149085442631464	2.51033614052987	1.90683502223783	0.167315253716858	0.675596252629528
Birc6	Birc6	0.114036662321877	7.76296377068698	1.90565634728488	0.167446557944941	0.675907700899069
Prdm11	Prdm11	0.0910611256713618	3.92473414858315	1.9049730599956	0.167522730096574	0.675949270089481
Slc35d3	Slc35d3	-0.20435318125854	3.640029203673	1.90459218050252	0.167565207372507	0.675949270089481
Snapc5	Snapc5	-0.0589267270966093	4.57088012780646	1.9035214840162	0.167680914683166	0.676221601325747
Plrg1	Plrg1	-0.0448795582494586	5.72983992077228	1.90216204396244	0.167836517064667	0.67658294790651
Selenot	Selenot	-0.0404205364527488	7.84562500304937	1.90007239128347	0.16807021637176	0.67658294790651
RGD1359	RGD135912	0.0854307908807144	3.72379301744689	1.89991049695161	0.168088237614895	0.67658294790651
Cdc40	Cdc40	0.0510386422143156	5.06904825011476	1.8996559493603	0.168116845496497	0.67658294790651
Dpy19l3	Dpy19l3	0.064005634889119	6.88982603193232	1.89956890556353	0.168126590151967	0.67658294790651
LOC4978	LOC497899	0.27762852772151	-0.383472270025082	1.89952165740577	0.168131870559197	0.67658294790651
Arid4a	Arid4a	0.0612639904207553	5.74849658563103	1.89910396849979	0.168171927410429	0.67658294790651
Cyp2u1	Cyp2u1	0.0818344722337082	3.28022637445445	1.89815846862203	0.168284586780727	0.67658294790651
Atad2	Atad2	0.0976123468154149	3.31727832797117	1.89753041112075	0.168355005266474	0.67658294790651
Pik3cb	Pik3cb	0.0637826896972863	5.0085058766536	1.89749978465618	0.168361804257916	0.67658294790651
Ptpn22	Ptpn22	-0.188671102505727	-0.101329710485455	1.89704795426837	0.168409619395318	0.67658294790651
Adamts15	Adamts15	-0.126769009735008	4.07499447082951	1.89680345789277	0.168436550616873	0.67658294790651
Rbpjl	Rbpjl	-0.187519178216524	2.09249808897734	1.89635594504941	0.168486772497773	0.67658294790651
Pcp4l1	Pcp4l1	-0.271053806675099	4.00044794218575	1.89592020358129	0.168535689836276	0.67658294790651
Efemp2	Efemp2	0.104717855584032	4.40785779540179	1.89418826948917	0.168730168348676	0.676870557452367
Cyp4f4	Cyp4f4	-0.234642195475411	0.201589720777182	1.89414487454812	0.168735260733803	0.676870557452367
Ctcf	Ctcf	0.03909322514972	6.97723820018669	1.89366456894841	0.1687891616489	0.676870557452367
Ppl	Ppl	-0.239865063562779	-0.487614961448273	1.89317047543549	0.168844756478076	0.676870557452367
Raf1	Raf1	0.0344785441090385	7.67460234731411	1.89298954801155	0.168865115198524	0.676870557452367
Slc36a4	Slc36a4	-0.0592789236215273	5.72830135239149	1.89283139434379	0.168882913823988	0.676870557452367
S100a10	S100a10	-0.0859436046677749	6.48614154708522	1.89192742482962	0.168984687251824	0.676706563562994
Rims3	Rims3	-0.062902143429027	5.64562258946682	1.89084018861701	0.169107187152457	0.676706563562994
Arfgap2	Arfgap2	0.0458956584960719	6.55834111194314	1.89054095097259	0.169140920374457	0.676706563562994
Hcn2	Hcn2	-0.0668614298180551	6.56832498878284	1.88979099040837	0.169225497750015	0.676706563562994
Med1	Med1	0.0545849103691205	6.31450235372633	1.88915004425492	0.169297819386806	0.676706563562994
Slc39a14	Slc39a14	0.077326744198823	4.28594485674055	1.88913041033585	0.169300035254341	0.676706563562994
C1r	C1r	0.197595415798522	3.19827185324478	1.88887565203147	0.169328791474702	0.676706563562994
Fpgt	Fpgt	0.094113993731198	2.44189484064528	1.88862844395054	0.169356700711501	0.676706563562994
Gdap2	Gdap2	0.0517925326283027	5.00299917824163	1.88842186471083	0.169380027090425	0.676706563562994
Fam32a	Fam32a	-0.0482098882393794	6.68426952828668	1.8869271718525	0.169549913687267	0.677164467373891
Grb2	Grb2	-0.0347949743172858	9.18659539101555	1.88587311481474	0.169668128630384	0.677239084745397
Ago4	Ago4	0.10017476783922	4.78783862721853	1.88534372132988	0.169728038861366	0.677239084745397
Ryk	Ryk	0.0621676381024311	5.23807476829617	1.88532257542953	0.169730433437684	0.677239084745397
Tmtsf1	Tmtsf1	0.203113628984269	0.972629591722426	1.88340276175643	0.169947905160207	0.677887131440835
Prkaa2	Prkaa2	0.0667909692955977	5.52769918552667	1.88292998211156	0.170001508519786	0.677887131440835
Pdrg1	Pdrg1	-0.0634351281158846	5.28166123142481	1.88189217051981	0.170119245984438	0.67794759849259
Mrap2	Mrap2	-0.134406684819332	3.37406229308332	1.88183843105868	0.170125345105418	0.67794759849259
Crkl	Crkl	0.041768548381937	6.06829601790647	1.88105768264085	0.1702129482543	0.678079727863634
Vwf	Vwf	0.097800258270669	5.31062165665275	1.88043662981452	0.170284530212583	0.678101658581971
Fbxl17	Fbxl17	0.0350664770900468	6.43149265255929	1.88006245172403	0.170327049862138	0.678101658581971
Sfrp4	Sfrp4	-0.241157094171771	-0.076850965830940	1.87929719850781	0.170414048232637	0.678195783160706
Rpl27a	Rpl27a	-0.0582739984863077	8.61578716261075	1.87898841065068	0.170459403328158	0.678195783160706
Spata2	Spata2	0.0397607927465283	6.0493114861438	1.87757457363279	0.170610068500298	0.678578845124393
Wdfy3	Wdfy3	0.0938520668504469	8.15183643109149	1.8756224285425	0.17083252253743	0.678753083120995
Slfn2	Slfn2	0.177491036660558	1.18700216466023	1.87490843823983	0.170913967137802	0.678753083120995
Egr4	Egr4	-0.243787073280383	2.65685703795143	1.87440874698545	0.170970993263902	0.678753083120995
Fads2	Fads2	0.0362434524716264	7.87299446123942	1.87434856215	0.170977863182695	0.678753083120995
Fkbp5	Fkbp5	0.0501607250617713	6.06922941179164	1.87432856581509	0.170980145779421	0.678753083120995
Tbl1xr1	Tbl1xr1	0.0497629931088929	6.8927759462262	1.87379636415795	0.171040908836087	0.678753083120995
Maoa	Maoa	-0.0493346776474156	5.86917212213474	1.87365184926 8	0.171056271983185	0.678753083120995
Capn7	Capn7	0.0388272937829538	6.18416839308928	1.87298280027873	0.171145273716966	0.678753083120995
Pmfbp1	Pmfbp1	-0.236105002827185	0.790257505969461	1.87252278592985	0.171186421193341	0.678753083120995
Thbs2	Thbs2	-0.145135262890237	2.60941259384064	1.87242254364622	0.171197886306305	0.678753083120995
Ripply3	Ripply3	0.215213706004599	-0.234148204929188	1.8718872884974	0.17125908246734	0.678780041913813
Clic1	Clic1	0.110635788384292	3.96065467952466	1.86932909762886	0.171551938566122	0.679724845185615
Samd15	Samd15	0.114014651024405	1.68900341617622	1.86869419400521	0.171624710034368	0.679797303840892
Heph	Heph	0.168881191673681	2.55146674562149	1.86759714064982	0.171750535861481	0.679822968475778
Taf9	Taf9	-0.0538507520812164	4.78553234153021	1.86712417722176	0.171804814795693	0.679822968475778
Ap5m1	Ap5m1	0.120397902616209	4.13166825166794	1.86699260299581	0.171819918217851	0.679822968475778
Vamp8	Vamp8	0.211368157813386	1.68944159072687	1.86498672303466	0.172048867859002	0.680532321582118
Ldlr	Ldlr	-0.059741479019913	4.94966575404892	1.86445841970553	0.172111115389127	0.680532321582118
Gjb1	Gjb1	0.176064046831757	0.581768850536842	1.86422875522574	0.172137533614904	0.680532321582118
Prickle2	Prickle2	0.0586020303153253	7.26031400667404	1.86253856899658	0.172216953836514	0.680581124960116
Bhlhb9	Bhlhb9	-0.0381191051683275	6.28163176486594	1.86205979831604	0.172272070666081	0.680581124960116
Lrfn2	Lrfn2	0.0483480491848817	5.35013602344485	1.86208987806285	0.172313518774466	0.680581124960116
Spem2	Spem2	0.25375125863427	-0.51877593112484	1.86085712916815	0.172525907862071	0.681001572164936
Tubd1	Tubd1	0.0958267840880333	2.67304530094064	1.86082918291238	0.172529131170423	0.681001572164936
Ifrd2	Ifrd2	0.0974754235847589	3.54516677375197	1.86007068001619	0.17261664299806	0.681131516346233
Zswim4	Zswim4	0.0542429437103805	5.79740223877743	1.85931670606397	0.172703682891817	0.681259516737654
Wdr45	Wdr45	-0.0649444033210548	3.99729808370445	1.85606021536972	0.173080197371951	0.68231039227142
Dkk2	Dkk2	0.161115950434615	1.21303269286417	1.85578433283322	0.173112138186251	0.68231039227142
Gal3st1	Gal3st1	-0.10911387003584	2.26780636352138	1.85476214764757	0.17323054269947	0.68231039227142
Cox6b1	Cox6b1	-0.0736872966418579	6.80704145485416	1.85423992815943	0.173279477554623	0.68231039227142
Rtp4	Rtp4	0.256371683550045	-0.034609424342218	1.85419629768406	0.173296127921321	0.68231039227142
Morc3	Morc3	0.0636511464138269	5.8856990507091	1.85403175737126	0.173315204309135	0.68231039227142
Cybrd1	Cybrd1	0.183963706113323	1.77461330854722	1.85333324362257	0.173396215555781	0.68231039227142
Ttbk2	Ttbk2	0.0799856181347438	6.14414949317119	1.85322353686583	0.173407570240657	0.68231039227142
Rassf4	Rassf4	-0.136165777232676	1.92777683511318	1.85267269673446	0.173472863618474	0.682352118237863
Slc30a9	Slc30a9	-0.0348107993063796	7.3524763864357	1.85191397270385	0.173560952002688	0.682483453557371
Ciz1	Ciz1	0.0596201045406122	5.81844084188801	1.85111080074942	0.1736542568114	0.682560478022709
Dnajc11	Dnajc11	-0.0290712139470093	6.62574346735912	1.8507939438537	0.173691082221064	0.682560478022709
Eps15	Eps15	-0.0497023406221368	6.9246344220724	1.84995704893747	0.173787226812059	0.682560478022709
Fkbp1a	Fkbp1a	-0.0474785050216077	8.56490532704186	1.84916887529285	0.173880090030402	0.682560478022709
Bcs1l	Bcs1l	0.0619427004222239	4.39423164566762	1.84853802266444	0.173953526759845	0.682560478022709
Cldn10	Cldn10	-0.0995349707167236	2.71336631968603	1.84847675734083	0.173960660476192	0.682560478022709
Commd5	Commd5	-0.0584903654252762	4.15228041343639	1.84845255735118	0.17396347840925	0.682560478022709
Camkv	Camkv	-0.051353942662166	8.9491016711236	1.84780322126598	0.174039609025716	0.682571890083675
Aif1b	Aif1b	0.12286123047835	1.37137136260601	1.84706937405725	0.174124628567581	0.682571890083675
Phyhipl	Phyhipl	-0.0502332895860198	7.14082524456622	1.8469211807252	0.174141903927953	0.682571890083675
Piezo1	Piezo1	0.12228069558414	3.66269205890561	1.84654974591705	0.174185213143738	0.682571890083675
Inpp4a	Inpp4a	-0.0378986103409321	7.92744996600496	1.84484325040199	0.174384347965537	0.682896132735739
Ubap2l	Ubap2l	0.0416144535449578	7.80543484243325	1.84481681255903	0.174387435118533	0.682896132735739
Atad1	Atad1	-0.0354129238138193	7.14183536824564	1.84408260400924	0.174473193966105	0.682896132735739
Wdr26	Wdr26	-0.0566166736316555	5.2900076892586	1.8434639698385	0.17454549090941	0.682896132735739
Vwa3a	Vwa3a	-0.157024268839644	3.70119381281691	1.84314433511529	0.174582893749522	0.682896132735739
Pank3	Pank3	0.0477652963840903	8.23664517391738	1.84303894868729	0.174595350358821	0.682896132735739
Trim16	Trim16	0.0794585461814664	4.52603176769574	1.84188182893189	0.174730545472303	0.682990005870152
Map2k1	Map2k1	-0.0806390216342937	6.26793455197244	1.84144987223721	0.174781108287045	0.682990005870152
Rps6kc1	Rps6kc1	-0.0335185559935108	6.22480795879021	1.84109749460216	0.174822368636845	0.682990005870152
Gnptg	Gnptg	-0.0385692514123198	5.62903856330016	1.84023856203163	0.174922988895477	0.682990005870152
Sema4c	Sema4c	0.0666151741568991	6.47080054396518	1.84007375879337	0.174942302396133	0.682990005870152
Otud7b	Otud7b	0.0792564267608247	4.22442348765008	1.83893303129689	0.175076053997401	0.682990005870152
Fbxw8	Fbxw8	0.0394421341863006	6.4732579144545	1.83867787132053	0.175105987880159	0.682990005870152
Ppp1r3c	Ppp1r3c	-0.0724784147497426	4.04350495133626	1.83862190380412	0.175112554463985	0.682990005870152
Rgs6	Rgs6	0.0630416853784148	5.58088572604661	1.83755248964316	0.175238907261858	0.682990005870152
Cdk13	Cdk13	0.043204118814421	6.51456893868205	1.83732792402541	0.175264454055753	0.682990005870152
Ncor1	Ncor1	0.0565309446300116	8.12779668423836	1.83682035117528	0.175324078212135	0.682990005870152
Usp31	Usp31	0.0736301287762521	5.43474198006513	1.83663003874986	0.175346441324936	0.682990005870152
Cdh7	Cdh7	-0.0922553319778388	4.10527835262359	1.83652595766423	0.175358672636777	0.682990005870152
LOC1009	LOC100912	0.18436102558534	0.0691322108484472	1.83430298454761	0.175620142787923	0.682990005870152
Kcnn1	Kcnn1	0.0540033034654499	5.24838382143362	1.83374852742023	0.175685429033246	0.682990005870152
Ppp4r4	Ppp4r4	-0.128681258907761	2.17767239641794	1.83374564788355	0.175685768164805	0.682990005870152
Mrps35	Mrps35	-0.0715038136063173	5.01578409104664	1.83332569122507	0.175735235971779	0.682990005870152
RGD1309	RGD130974	0.0441082311501096	6.38380446308559	1.83298301560439	0.175775612534842	0.682990005870152
Chst3	Chst3	0.14576522453331	1.20382920447695	1.83255844252656	0.175825653691923	0.682990005870152
Pcdhb20	Pcdhb20	0.0834685733693626	3.49628813271932	1.83251801204525	0.175830418777381	0.682990005870152
Etv5	Etv5	0.0586934511308291	5.96644164284414	1.83228061167308	0.175860766519847	0.682990005870152
F2rl1	F2rl1	-0.195443286423155	0.348706268961045	1.83214063508555	0.175874913467638	0.682990005870152
Snap25	Snap25	-0.051497287287682	8.32181764869907	1.83208974753049	0.175880914231049	0.682990005870152
Prrc2c	Prrc2c	0.11538511574194	7.54384475792614	1.83093846455076	0.176055592584053	0.683462006525515
Polm	Polm	-0.124471100289948	2.74494497414802	1.82885929717445	0.176262348226154	0.684051390047194
Flt3	Flt3	-0.208198725570225	1.10089050274184	1.82833660877685	0.17632414336143	0.684076462910624
Wdr93	Wdr93	-0.231582549547267	-0.100268440614962	1.82752570676678	0.17642007642773	0.684237890453628
Cdh2	Cdh2	0.052423230055827	8.79176221974954	1.82613583383529	0.176584644191779	0.684519590147551
Slc9b2	Slc9b2	-0.101542996411037	3.14506933266204	1.82481937208456	0.176740683011028	0.684519590147551
Mcrs1	Mcrs1	-0.0380024073904825	6.89541072965226	1.82442229857565	0.176787793354703	0.684519590147551
Kxd1	Kxd1	-0.0542008744332389	4.37912548851856	1.82321520841813	0.17693103787518	0.684519590147551
Dnai2	Dnai2	0.285582421489713	-0.234695282090507	1.82257297582115	0.17700731341234	0.684519590147551
Lepr	Lepr	0.193510281188084	2.15526651816624	1.82175148148941	0.177104934496302	0.684519590147551
Fbln1	Fbln1	0.145072224616874	4.72724838248201	1.82114747148313	0.177176756777255	0.684519590147551
Daxx	Daxx	0.0428023241100768	5.61872955854055	1.82104763554179	0.177188624422481	0.684519590147551
Ptpn5	Ptpn5	-0.0920575389988068	7.35841293761207	1.8209640090816	0.177205707753558	0.684519590147551
Vwa5b1	Vwa5b1	-0.170498104232298	3.21941379816154	1.82079850229529	0.177218058235393	0.684519590147551
Lhx2	Lhx2	0.122161747906656	6.55485106337399	1.82073927218364	0.177225204359122	0.684519590147551
Calcrl	Calcrl	0.0885085758834008	3.33111655883565	1.8203154781506	0.177275729114775	0.684519590147551
Fam118b	Fam118b	0.0604627878882323	4.2943688237832	1.81986583894847	0.177329247137784	0.684519590147551
Lig3	Lig3	0.0524362436068563	5.52658186864355	1.8192209803324	0.177406032578391	0.684519590147551
Apod	Apod	0.190131513480329	2.67482574377022	1.81895657485614	0.177437528712083	0.684519590147551
Cd320	Cd320	0.0850686884471622	4.03627149988994	1.8181246794022	0.177536663534336	0.684519590147551
Pcdh7	Pcdh7	0.0752952667638017	6.49872834527348	1.8181213427785	0.177537061162984	0.684519590147551
Ldha	Ldha	-0.0600789642973986	8.36745620687481	1.81657047856922	0.177722044965489	0.684519590147551
Slc9b1	Slc9b1	-0.268749290474793	-0.446659730805509	1.8162623243019	0.177758827446367	0.684519590147551
Sema6c	Sema6c	0.0503385024546037	7.52016795714209	1.81621451246586	0.177764535211317	0.684519590147551
Tedc2	Tedc2	0.0664947164911806	3.5647408204487	1.81617583327323	0.177769152897731	0.684519590147551
Zfp532	Zfp532	0.0534007232194026	7.13941221528239	1.81617413184073	0.177769356025155	0.684519590147551
Hnrnph3	Hnrnph3	0.0699615438581446	6.14211614853984	1.81605324363798	0.177783789084409	0.684519590147551
Tbl3	Tbl3	-0.036035078648029	5.54507642294299	1.81433316607872	0.177989298872432	0.684519590147551
Arsb	Arsb	0.0642245184478274	5.79917475099716	1.81427608646754	0.177996123276357	0.684519590147551
Nts	Nts	-0.239986117075824	5.43373743783033	1.81425433868552	0.177998723387995	0.684519590147551
Icam1	Icam1	0.172918524092793	1.28110616795847	1.81415588775059	0.178010495150553	0.684519590147551
Ppp1r14c	Ppp1r14c	-0.0745741324610658	6.62259972809955	1.81353594968221	0.178084540947414	0.684519590147551
Mlycd	Mlycd	-0.0617708212009883	4.58887982104665	1.81199454784295	0.178269150229115	0.684519590147551
Cdk5r2	Cdk5r2	-0.0745107538349411	8.18899984602537	1.81164173605366	0.178311413639863	0.684519590147551
Rab11a	Rab11a	-0.0327359789681402	7.90439331074232	1.81082537620364	0.178409249948499	0.684519590147551
Il10rb	Il10rb	0.103182013669647	1.98389699508183	1.81057898909003	0.178438790306172	0.684519590147551
Cirbp	Cirbp	-0.0561027410100025	7.05769423822527	1.81035671271522	0.178465444764088	0.684519590147551
Sirpa	Sirpa	-0.0561581928545278	6.96684729496067	1.80988900852796	0.178521544926678	0.684519590147551
Erbb4	Erbb4	0.0914912677104043	5.54118960350541	1.80942061205776	0.178577748543276	0.684519590147551
Ripk1	Ripk1	0.0832842621455188	4.03197554985385	1.80901525674329	0.1786264042533	0.684519590147551
Rnf185	Rnf185	0.0378542830763759	5.6617042805109	1.80803154796715	0.178744544722552	0.684519590147551
Lsm4	Lsm4	-0.0749006279055646	5.67455854100026	1.80800767562648	0.178747412840853	0.684519590147551
Taf5	Taf5	0.0661442318411752	3.81106447670857	1.80746135655399	0.178813064324226	0.684519590147551
Erlin2	Erlin2	0.0381040421569428	6.16632398501948	1.80713295709558	0.178852541686369	0.684519590147551
Brca1	Brca1	0.123578050077487	2.57286955759294	1.80711714218005	0.17885444307381	0.684519590147551
Ints6	Ints6	0.067353583109117	4.89889321290313	1.80666371823637	0.178908967015197	0.684519590147551
Ca9	Ca9	0.225449959545829	-0.399047774874594	1.80649422398628	0.178929353538984	0.684519590147551
Sar1b	Sar1b	-0.0581091120073846	6.21526673035756	1.8056202850548	0.179034512201873	0.684519590147551
Csrnp2	Csrnp2	0.0456482922560544	6.86280152465256	1.80481884779979	0.179131009718885	0.684519590147551
Calcoco1	Calcoco1	0.0609180233086334	6.47499752895547	1.80472940673226	0.179141782649866	0.684519590147551
Pde12	Pde12	0.0662207030236123	3.30644546310713	1.80444028749884	0.17917661128106	0.684519590147551
Rcn1	Rcn1	-0.0611367121874054	6.58726279825202	1.80422724234705	0.179202280877798	0.684519590147551
Acbd5	Acbd5	0.0425701540619195	5.75329824733957	1.80421667085909	0.179203554731002	0.684519590147551
Ca11	Ca11	-0.0924464352985953	6.99836109446771	1.80394881962573	0.179235834015553	0.684519590147551
Sema3c	Sema3c	-0.0890492754455797	6.86054973295847	1.80321479820578	0.179324327071802	0.684647988251799
Ppp1r13b	Ppp1r13b	-0.0381712255966061	6.87905959460499	1.8019244243287	0.179519548764491	0.684934497494429
Rnf39	Rnf39	-0.116447704541174	1.9464242399484	1.80059552054827	0.179640516019891	0.684934497494429
Slitrk6	Slitrk6	-0.217246361834363	2.02206788104792	1.80041593210624	0.179662218943984	0.684934497494429
B3gat3	B3gat3	-0.0516662867161265	6.9531344857431	1.80018050135818	0.179690674577124	0.684934497494429
Cul4b	Cul4b	0.0448201358213902	6.20961340228806	1.80015783098423	0.179693408647284	0.684934497494429
Smc3	Smc3	0.0391058771606663	7.30380664897834	1.79986559 6354	0.179728744473572	0.684934497494429
Camk1d	Camk1d	-0.0753251744337235	6.01859934189135	1.79877110844642	0.179861132902784	0.685229726787186
Ca13	Ca13	0.226001478842175	0.353605873218693	1.79709724470428	0.180063820356425	0.685792517273232
Rsrc1	Rsrc1	-0.0526173568658386	4.57002901310257	1.79630304167664	0.180160092458634	0.685856970817665
Zfyve26	Zfyve26	0.085476412086307	5.04389631426657	1.7959221855945	0.180206264265349	0.685856970817665
Rab23	Rab23	0.0642644509960737	5.34712826436149	1.79559746387692	0.180245652585647	0.685856970817665
Nme3	Nme3	-0.0751180370601537	4.79145707080551	1.79445565196772	0.180384227349816	0.686072182345923
Gpr52	Gpr52	-0.122221963554099	3.1420775560092	1.79328201840143	0.180526792572384	0.686072182345923
Ube2ql1	Ube2ql1	0.0575329994079457	8.02769292733409	1.79273891159039	0.180592806701081	0.686072182345923
Trhr	Trhr	-0.189459164371882	1.44189452379928	1.79258059800399	0.180614490623841	0.686072182345923
Hcfc1	Hcfc1	0.0672782134576011	8.05613128999302	1.79253964704813	0.180617038225708	0.686072182345923
Arfip2	Arfip2	-0.0365369725751215	6.25337448150873	1.79223908598678	0.180653590494655	0.686072182345923
Cldn19	Cldn19	0.437605585461716	-0.359142018110324	1.79196128056526	0.180687382959742	0.686072182345923
Klhl5	Klhl5	-0.0362525027135597	7.3940359676401	1.79102073488173	0.180801846013133	0.686139603259785
Zbtb49	Zbtb49	0.0688221264660894	3.64899956043182	1.79081371656172	0.180827051109087	0.686139603259785
Ccdc91	Ccdc91	-0.0495527207126013	5.24051371548502	1.7896602449586	0.180967564174382	0.686139603259785
Coro1c	Coro1c	-0.0339665365768225	7.35490349350432	1.78934915137603	0.181005482459822	0.686139603259785
Hrc	Hrc	0.217192875887376	0.94189954033983	1.78905839678581	0.181040931217458	0.686139603259785
Cdca4	Cdca4	0.0620278068973845	4.19629360199414	1.78782150838271	0.181191815885675	0.686139603259785
Pltp	Pltp	0.147824808670616	5.41684957824238	1.78781163891813	0.181193020307823	0.686139603259785
Vrk2	Vrk2	0.170022504567691	0.7430351829188	1.78758014734086	0.181221276073773	0.686139603259785
Manf	Manf	0.0747984388691635	5.18889492879526	1.78744355787488	0.18123795044788	0.686139603259785
Disc1	Disc1	0.124740972106021	1.61559687840684	1.78655181689062	0.181346854788884	0.686139603259785
Pex5l	Pex5l	-0.0877403823674339	3.51170680392841	1.78644586194925	0.181359677449186	0.686139603259785
Ppm1e	Ppm1e	-0.0540027986769749	6.72271446575533	1.78625453525726	0.181383177301519	0.686139603259785
Adora2b	Adora2b	-0.126011468441729	2.24514230770779	1.7854970316254	0.181475768812217	0.686139603259785
Tram1	Tram1	0.0446356614120547	5.80937521361429	1.78549041152324	0.181476578244568	0.686139603259785
Glrb	Glrb	-0.0562981332642193	6.17755324375968	1.78505551921542	0.181529761189433	0.686139603259785
Gtpbp2	Gtpbp2	0.0570577335564026	6.08664477262962	1.78340744513962	0.181731467872999	0.686458807735388
Sytl2	Sytl2	-0.110376599078952	3.0952968556721	1.7823297888126	0.18174097735468	0.686458807735388
Prmt9	Prmt9	0.0483232956363213	4.58777485876488	1.78209223111229	0.181819083183117	0.686458807735388
Tdp2	Tdp2	0.0866942343950509	3.73051867362433	1.78216489497324	0.181883715590257	0.686458807735388
Kctd8	Kctd8	-0.116209644823193	4.27452180753512	1.78211910036681	0.181889328281894	0.686458807735388
Gpatch4	Gpatch4	0.0490788902816119	4.43126719229438	1.78030973337653	0.182111297563863	0.686859193191496
Dcun1d5	Dcun1d5	-0.0425404239737671	5.8683066851468	1.78014107843686	0.18213200377314	0.686859193191496
Ube2g2	Ube2g2	0.0646212609824654	5.80755718163002	1.77952600155662	0.18220754148724	0.687036414365757
Lhfpl4	Lhfpl4	0.0493556289628551	5.63247937285587	1.77789014094342	0.182408618539675	0.687085224960521
Dpp6	Dpp6	-0.0710998329302782	7.49768820159548	1.77776899676198	0.182423765603725	0.687085224960521
Rab43	Rab43	0.100137983907722	2.53882283337527	1.77706673420202	0.182509927483044	0.687085224960521
Rnf168	Rnf168	0.060534774649949	4.65030595897386	1.77705810075071	0.182510990056513	0.687085224960521
Mpp4	Mpp4	0.287933817591456	0.771455196482723	1.77590498150938	0.182652976175493	0.687085224960521
Syngr3	Syngr3	-0.0508675332288168	6.95741372999496	1.77511051348242	0.182750875254011	0.687085224960521
Kank1	Kank1	0.0798638706148213	4.32855942860614	1.77488510016097	0.182778663098391	0.687085224960521
Dnajc28	Dnajc28	0.130671463002118	1.68972622252168	1.77407017626653	0.182879163764074	0.687085224960521
Cdh22	Cdh22	-0.0942332159475487	5.42713794871801	1.7740565809036	0.18288084095822	0.687085224960521
Slc25a42	Slc25a42	0.0641945564597062	5.05287940718349	1.77397154225001	0.182891332168989	0.687085224960521
Tgif2	Tgif2	0.0946078768328272	2.79248507267741	1.77391703757426	0.182898056774222	0.687085224960521
Mfge8	Mfge8	-0.0460380777974586	7.58380479295222	1.7738434 30905	0.182907138573229	0.687085224960521
Uqcrh	Uqcrh	-0.0675407163289981	6.22674247212671	1.77250342187403	0.183074096633197	0.687085224960521
Myl12a	Myl12a	0.101947090257258	4.79068930179236	1.77242058627215	0.183082795918797	0.687085224960521
Sox9	Sox9	0.0935420123990543	5.1880219308139	1.77237713853184	0.183088162713835	0.687085224960521
Zfp672	Zfp672	0.0578243824814718	4.09434908262621	1.77226855310563	0.183101576740701	0.687085224960521
Coq6	Coq6	-0.049317571224546	5.38585724868326	1.77113010159865	0.183242281889234	0.687342649121373
Rnf128	Rnf128	-0.0892870267479136	3.55471550428219	1.77057246187833	0.18331124824158	0.687342649121373
Tmem150a	Tmem150a	0.100853161213308	4.58031047556346	1.77037687943707	0.183335444119254	0.687342649121373
Pex12	Pex12	0.0685064895402377	3.90235465720506	1.76955249276435	0.18343747251508	0.687404040284706
Gpr63	Gpr63	0.0922279829667053	2.63609792392738	1.76912208649029	0.183490765070891	0.687404040284706
Gnrh1	Gnrh1	-0.312116159887515	-0.416612339852423	1.7689094553815	0.183517100119288	0.687404040284706
Degs1	Degs1	0.0498527974620822	5.76502561225457	1.76760354583145	0.183678937386178	0.687797767990255
Hyal2	Hyal2	0.061731855288497	4.94102906000046	1.76717191367698	0.18373246459177	0.687797767990255
Lyl1	Lyl1	0.151398158504811	1.31039306052245	1.76597766989262	0.183880658827182	0.687953330612233
Tbc1d14	Tbc1d14	0.0423414831966791	8.02032007678559	1.76584837251905	0.183884295711453	0.687953330612233
Tmem209	Tmem209	0.0786671269712788	3.29659613294874	1.76422619445611	0.184098250117034	0.688383907673267
Bag5	Bag5	-0.0410969770820443	5.43770129172024	1.76413385619001	0.184109729895463	0.688383907673267
Slitrk2	Slitrk2	0.0844329262294847	4.21833353808077	1.76348573852572	0.184190329136685	0.688464715418773
Hspa1l	Hspa1l	0.106645099861568	2.17476271893862	1.76274730707546	0.184282209889557	0.688464715418773
Ptges2	Ptges2	-0.0623445343061262	5.18227439818817	1.76225664196826	0.184343291130952	0.688464715418773
Atp6v0a4	Atp6v0a4	0.0889455672538752	3.33456094297071	1.76203893036418	0.184370400945827	0.688464715418773
Tmsb15b	Tmsb15b2	0.0827980288879421	5.75185008371895	1.76174317859856	0.184407235627919	0.688464715418773
Slc29a4	Slc29a4	0.0490782928814665	7.65790684249178	1.76021032303478	0.184598283972846	0.688774558150795
Arrdc3	Arrdc3	0.0924498767307089	4.64154125110855	1.75880182620596	0.184774035218317	0.688774558150795
Igfbp7	Igfbp7	0.0972139918311242	5.53988256293193	1.75857241006923	0.184802680134545	0.688774558150795
Cep112	Cep112	0.136632739568722	2.12744252190424	1.75856717741564	0.184803333531472	0.688774558150795
Casc3	Casc3	0.0403987011637081	6.21373875015685	1.75681521137827	0.185022254171972	0.688774558150795
Irf2	Irf2	-0.0757929352472563	2.79019297300117	1.75665978751628	0.185041690002585	0.688774558150795
Scfd2	Scfd2	0.0480559304231159	4.45338521770405	1.75665299102574	0.185042565044435	0.688774558150795
Cdk8	Cdk8	0.0487652776403223	5.79185850295129	1.7566455875001	0.185043464614576	0.688774558150795
Snw1	Snw1	0.04319347440147	5.52935764149763	1.75587007822547	0.185140490182123	0.688774558150795
Dcaf15	Dcaf15	-0.051948571070211	6.06205338322889	1.75584269475585	0.185143908247506	0.688774558150795
Litaf	Litaf	0.0606635524944438	3.85295873714147	1.7554929664116	0.185187677475011	0.688774558150795
Trim17	Trim17	0.187536461582445	0.510734048325298	1.75538154283487	0.18520162528908	0.688774558150795
Pdk4	Pdk4	0.236743609072526	0.465912291732041	1.75533162619782	0.185207874164536	0.688774558150795
E2f4	E2f4	0.0522698858858721	5.18500392390382	1.75479138329514	0.185275529390832	0.68882082033134
Slc52a2	Slc52a2	0.0618359215506209	3.93899686615855	1.75227589198884	0.185590876683841	0.689787717719476
Mpeg1	Mpeg1	0.165015106592264	3.68479943310229	1.75121953214087	0.185723493621999	0.689905627786375
Tjp3	Tjp3	-0.15080875795401	2.28876492863833	1.75008058503579	0.185866600202582	0.689905627786375
Nr1i3	Nr1i3	-0.0642219373217986	3.66921969649239	1.75001752467529	0.185874529285411	0.689905627786375
Prpf6	Prpf6	0.0327138397093713	6.6129596382833	1.74973322772022	0.185910272870099	0.689905627786375
Mapk9	Mapk9	-0.0416237887402208	7.59393779627008	1.74951975763866	0.185937116908032	0.689905627786375
Nudt14	Nudt14	0.0980821608936277	3.72244893313236	1.7487562068125	0.186033170918716	0.689905627786375
Slc22a6	Slc22a6	0.293968301525163	4.98838094120087	1.74863314938413	0.186048656926981	0.689905627786375
Otud3	Otud3	0.0897357507889462	2.0176311246494	1.74829906212356	0.186090708902793	0.689905627786375
Ncoa6	Ncoa6	0.069010851012815	7.15839441628843	1.74700962808127	0.186252105920814	0.689905627786375
Hmx3	Hmx3	-0.525960511184385	0.102633680082524	1.74687544149918	0.186270015528336	0.689905627786375

Acss3	Acss3	0.222813977945868	0.332532174016294	1.74620651414551	0.186354337956245	0.689905527786375
Gdpd2	Gdpd2	-0.0944946832961029	4.08134437132804	1.74537262316517	0.186459517189655	0.689905527786375
Gcat	Gcat	0.0952070017316784	2.68495127036225	1.7450014203359	0.186506359446539	0.689905527786375
Ptcd2	Ptcd2	-0.0694482434144251	3.41505266657572	1.74493362306684	0.186514916292409	0.689905527786375
Ddx49	Ddx49	-0.0404914854341958	5.75868824425201	1.74488779865536	0.186520701667569	0.689905527786375
Fchsd2	Fchsd2	0.0394286620771959	7.23023050250058	1.74484460804786	0.186526151771108	0.689905527786375
Sun1	Sun1	0.0643752315914289	5.56006584387833	1.74455586581688	0.18656260224022	0.689905527786375
Spdya	Spdya	-0.123486505259928	1.36267922781735	1.74298183858389	0.186761453058874	0.690079689497294
Spindoc	Spindoc	0.0493117358168389	5.32577208501021	1.74295297743227	0.186765098551702	0.690079689497294
Hmbs	Hmbs	-0.0749950624560179	3.84021510624816	1.7428899467035	0.186775596011277	0.690079689497294
Clec14a	Clec14a	0.160700247306586	3.00386554181748	1.74072557887647	0.187046917615854	0.690196096585344
Zdhhc24	Zdhhc24	0.0651634721418289	3.8980126177737	1.74065949407203	0.187055286179164	0.690196096585344
Mon2	Mon2	0.0628036061288036	6.21035398825867	1.74019798286136	0.187113742960922	0.690196096585344
Fat3	Fat3	0.138964048486671	6.71468784847743	1.73983516919867	0.187159713247316	0.690196096585344
Mei1	Mei1	-0.249198298234842	-0.620325827983553	1.73922949810979	0.187236484038247	0.690196096585344
Zfp74	Zfp74	0.0639654835670774	4.66997672150334	1.73920845523276	0.187239151949827	0.690196096585344
Usp1	Usp1	0.0560035855195013	5.06953409498316	1.73910589524751	0.18725215555916	0.690196096585344
Ttc17	Ttc17	0.0423079818319066	5.69889122626716	1.73897585593703	0.187268644892187	0.690196096585344
Arx	Arx	-0.0580294491138287	6.50287762457035	1.73868951323034	0.187304956769013	0.690196096585344
Mterf4	Mterf4	-0.0741193509954431	3.51105320347337	1.7380204467333	0.187389844804574	0.690305017309322
Pdyn	Pdyn	-0.250384941525423	4.00324262857883	1.73714077071767	0.187501518146294	0.690512527128484
Smpdl3a	Smpdl3a	-0.12606851436284	1.98594321434852	1.73620590628544	0.187620282421539	0.690746020588238
Fundc2	Fundc2	0.0715123384073789	3.76995917745672	1.73522480369314	0.187745014870804	0.690848558982821
Rbm27	Rbm27	0.0557964478254806	5.65476732734461	1.73484331710959	0.187793541203726	0.690848558982821
Plk3	Plk3	-0.0565282888407376	4.56557373863935	1.73428005943248	0.187864452421516	0.690848558982821
Hba-a1	Hba-a1	0.165858024758457	9.01790291520873	1.73424551493038	0.187869612938103	0.690848558982821
Ccnj	Ccnj	0.0689684557207889	4.95779884670413	1.7337738128381	0.187929663207967	0.690865765790659
Entpd7	Entpd7	0.0965451557024641	3.16448997088024	1.73243110565916	0.188100720479477	0.691053773900394
Pms2	Pms2	-0.0527276852975262	4.98303896305617	1.73213271089465	0.188138759686348	0.691053773900394
Nfkb1	Nfkb1	0.0604927429173889	4.17903018321976	1.73168052816545	0.188196419401498	0.691053773900394
Mrpl45	Mrpl45	-0.0557675342298426	5.71559523496098	1.7316340380839	0.188202350221491	0.691053773900394
Ldhb	Ldhb	-0.0545084164121471	7.83134627064497	1.72908511564886	0.188527778913863	0.691283927043082
[illegible]	[illegible]	[illegible]	[illegible]	[illegible]	[illegible]	[illegible]

**Crot**	Crot	0.0853545898058087	3.89290836237202	1.56068994381346	0.211564227825796	0.711064770439272
**Srpk1**	Srpk1	0.0311827447801035	6.87329023881846	1.56055406724683	0.211584112615606	0.711064770439272
**Macir**	Macir	-0.0412034681167923	6.23748594968407	1.56017234246391	0.211639987805206	0.711064770439272
**Fkbp14**	Fkbp14	0.137348849859684	1.78812891445839	1.56016390563886	0.211641222948103	0.711064770439272
**Pex14**	Pex14	-0.0538168798814813	5.31173078760344	1.55975094108438	0.211701699006307	0.711064770439272
**Hap1**	Hap1	-0.149097132003292	7.9663597025928	1.55950722073584	0.211737387210978	0.711064770439272
**Taf7**	Taf7	0.0482319510758828	4.25477533273083	1.55795931853125	0.211964265579816	0.711064770439272
**Xpo5**	Xpo5	0.052438847815481	6.55102339542066	1.55700216042156	0.212104697311404	0.711064770439272
**Casp9**	Casp9	-0.0493835841294309	4.68595971554277	1.55671626048968	0.212146671212326	0.711064770439272
**Hgf**	Hgf	-0.14397351577605	2.01362767827785	1.55671447298764	0.212146933743582	0.711064770439272
**Lysmd3**	Lysmd3	0.0939977305206683	2.31280973556376	1.55518197964784	0.212372067864249	0.711064770439272
**Pkn2**	Pkn2	0.0563144954987039	5.50889888257885	1.5545730289989	0.212461605686518	0.711064770439272
**Rad1**	Rad1	0.0842300047604032	3.7630251090923	1.55426146460911	0.212507434304226	0.711064770439272
**Cs**	Cs	-0.028257131839817	8.00453712917875	1.55407829461808	0.212534382608973	0.711064770439272
**Commd1**	Commd1	-0.0668170365338359	4.99210996820999	1.55280879476223	0.212574038295852	0.711064770439272
**Loxl2**	Loxl2	0.164403082473405	2.47769186682032	1.55338168234845	0.212638908537129	0.711064770439272
**Rwdd4**	Rwdd4	0.0387215274272214	5.58057768493848	1.55268525612765	0.212742407828491	0.711064770439272
**Ephx4**	Ephx4	0.0989328091985287	3.81308808250985	1.55223924682451	0.212805171639082	0.711064770439272
**Chpf**	Chpf	-0.0534176692296855	7.10230562807527	1.55114548233388	0.212967153754426	0.711064770439272
**Nmi**	Nmi	-0.151449084565451	1.28238222563109	1.55113626133208	0.212967776305297	0.711064770439272
[illegible]	[illegible]	[illegible]	[illegible]	[illegible]	[illegible]	[illegible]

Lims2	Lims2	-0.0784419659259635	3.58134798227721	1.28807004359982	0.237040975647068	0.731148813063841
Cpeb4	Cpeb4	0.0617488817023255	6.89544656014072	1.28806728867853	0.237047437675041	0.731148813063841
Agpat3	Agpat3	-0.0301584384580321	7.81076572430668	1.28778556294825	0.23709469167285	0.731148813063841
Acvr2b	Acvr2b	0.0861709760255348	3.40085532882214	1.28745444482125	0.237148566903859	0.731148813063841
Apol3	Apol3	0.189358284545223	1.75136779811087	1.28617264528211	0.237365447117163	0.731148813063841
Kyat3	Kyat3	0.0618380258052586	4.15515819685843	1.28608856779138	0.237379571220115	0.731148813063841
Cpne8	Cpne8	-0.0835787815195043	4.27834982723142	1.28605210744496	0.237386032479238	0.731148813063841
Usp20	Usp20	0.0304616672151055	6.65758951292594	1.28598517882303	0.237395260660563	0.731148813063841
Bin1	Bin1	-0.0316457625453324	7.47539659065047	1.28516157035043	0.237535364278262	0.731148813063841
Aarsd1	Aarsd1	-0.0355064757430756	6.28699912987629	1.28509541873387	0.237546486591442	0.731148813063841
Arhgap35	Arhgap35	0.0387374136682064	9.22546077634553	1.28487274807939	0.237583930000491	0.731148813063841
Lcor	Lcor	0.118172477139743	3.00673486826994	1.28475277820961	0.237604105906602	0.731148813063841
Kcna5	Kcna5	-0.202426909437281	1.27093039697521	1.28362109732782	0.237794533636822	0.731148813063841
Grip1	Grip1	0.0296900222668687	6.45985608179565	1.28360264281645	0.237797640484376	0.731148813063841
Ltbp2	Ltbp2	0.255589285852522	-0.0182250045744469	1.28349569747482	0.237815648004588	0.731148813063841
Nova1	Nova1	-0.0772630707898472	6.58164025388482	1.28280788868571	0.237931486294181	0.731148813063841
Cst6	Cst6	-0.0616260521162614	5.44724278886197	1.28273799160183	0.237943262107686	0.731148813063841
Evc	Evc	0.0724181557906429	3.01575520474546	1.28202365383775	0.238063650056386	0.731148813063841
Procal	Procal	0.0845765391875707	2.2976887137511	1.28167317302439	0.238122743908898	0.731148813063841
Mthfd1l	Mthfd1l	-0.0636042241164836	5.09570550684789	1.2813437548732	0.238178300655988	0.731148813063841
Srgn	Srgn	0.129427424480034	3.48216481645582	1.28119233621383	0.238203845781667	0.731148813063841
Uqcrb	Uqcrb	-0.0736500288944726	5.79169131828388	1.28115600048349	0.238209975825428	0.731148813063841
Atp6v1d	Atp6v1d	-0.04958957683315	6.98248744725777	1.28097234616135	0.238240962280162	0.731148813063841
Smarcad1	Smarcad1	0.0442845649222878	6.65893743465809	1.28082492571774	0.2382658088252	0.731148813063841
Uggt1	Uggt1	0.0660672205376075	6.4270198713326	1.28082102998089	0.238266496266537	0.731148813063841
Lgi1	Lgi1	-0.0426441708915241	5.24574862266636	1.27951096160193	0.238487703854322	0.731632978593028
Sdf2	Sdf2	-0.0549531685118455	5.45035914685372	1.27919227134008	0.238541553003855	0.731632978593028
Eogt	Eogt	0.073215707961468	4.22018626053783	1.27744858520321	0.238836783937683	0.732235654949734
Tsen2	Tsen2	-0.056674431202526	3.98850158739674	1.27670788068812	0.238961847418905	0.732561909151273
LOC306766	LOC306766	-0.0295389476446917	6.71865109364501	1.27553675553401	0.239160283199847	0.732827223078834
Zdhhc5	Zdhhc5	0.0350926806821655	5.6476769879624	1.27550385140528	0.239165861374471	0.732827223078834
Col5a2	Col5a2	0.0957715456362727	5.78852006070407	1.27483720864149	0.239278910097919	0.732866624831165
[illegible]	[illegible]	[illegible]	[illegible]	[illegible]	[illegible]	[illegible]

**Bhlhe23**	Bhlhe23	-0.256302855527124	0.829247808994356	1.26015462858144	0.261621823537701	0.744645954589828
**Stx11**	Stx11	-0.135902620345322	1.02697178104114	1.25990882597568	0.261687280321592	0.744645954589828
**Spata7**	Spata7	-0.052304238941189	4.66855731778023	1.25977120890086	0.26169440206101	0.744645954589828
**Crym**	Crym	-0.0808922155716141	7.23871667426533	1.25921198820872	0.261800304693589	0.744645954589828
**Hivep1**	Hivep1	0.069052924884713	7.09918217255943	1.25917681653881	0.261806965507157	0.744645954589828
**LOC1003**	LOC100360	0.16825439688435	1.25676508803783	1.25885483964083	0.261867965161156	0.744645954589828
**Deaf1**	Deaf1	-0.031574890941837	7.47275529656446	1.25873080082647	0.261891469452193	0.744645954589828
**Svop**	Svop	-0.0450288985329633	7.33906324590314	1.25857722032748	0.261920575263728	0.744645954589828
**Map1b**	Map1b	0.0864527469431282	12.413474222323	1.25848265871059	0.261938498105706	0.744645954589828
**Trim14**	Trim14	0.16867722796066	0.601903207454328	1.25823304425816	0.26198581636686	0.744645954589828
**Ca4**	Ca4	-0.261440104395333	1.67972920129679	1.25788968133824	0.262050923413502	0.744645954589828
**Actr10**	Actr10	-0.0363526023042128	6.96984952960059	1.25788964060523	0.262054724111455	0.744645954589828
**Nmnat3**	Nmnat3	-0.152193176315944	0.768543855332424	1.25760473724502	0.262104968561271	0.744645954589828
**Casq2**	Casq2	0.259060529162397	-0.317191050051127	1.25751967225668	0.262121105438724	0.744645954589828
**Tcf7l1**	Tcf7l1	0.0742080982107618	3.36718247528531	1.25596349172731	0.262416531742308	0.745315517083719
**Nt5c1a**	Nt5c1a	0.135855254506897	0.852008750960022	1.25540619708583	0.262522428941336	0.745446597836107
**Zfp574**	Zfp574	0.0329567440526436	5.26274547077876	1.25283295446568	0.262802631620876	0.745644473963241
**Trak1**	Trak1	0.0384581575088695	7.64354146849859	1.25374125128125	0.262836119726668	0.745644473963241
**Tmem88**	Tmem88	0.117265167854393	2.09020341057987	1.25262418298276	0.262861405218226	0.745644473963241
**Lck**	Lck	0.06155368621624	3.26588486602919	1.25200010884389	0.262980245795659	0.745644473963241
**Anks1b**	Anks1b	-0.0351863441800503	7.01058196556189	1.25297960730934	0.262984150579506	0.745644473963241
**Neurl2**	Neurl2	-0.0969309473352966	1.66723784343828	1.2528728947863	0.263004478512544	0.745644473963241
**Brd4**	Brd4	0.0550184740408588	8.00368349824451	1.25284157213177	0.263010445612906	0.745644473963241
**Retreg3**	Retreg3	0.0284167130723371	6.21534201984532	1.25230195387917	0.263113271601132	0.745766535612749
**Pom121**	Pom121	0.0394064768385215	6.92202088089444	1.25009119750623	0.263535056769774	0.746713796283278
**Vav2**	Vav2	0.0358832171251938	6.93361099485661	1.24992308398904	0.263567168231486	0.746713796283278
**Myrip**	Myrip	-0.0520761019885955	4.37517456031132	1.24844102071212	0.26385244287804	0.747158693219072
**Lamb3**	Lamb3	-0.205791927817376	0.214281337166804	1.24830245310392	0.263876947361498	0.747158693219072
**Cox6b2**	Cox6b2	0.113354953151122	1.39139514895334	1.24800512120939	0.263933830606717	0.747158693219072
**Cwf19l2**	Cwf19l2	0.036949780921868	5.51950372474311	1.24784884180901	0.263963734884224	0.747158693219072
**Myl9**	Myl9	0.11301677208902	3.81468541736588	1.24611441748179	0.264295902153114	0.747906079543733
**Rfc5**	Rfc5	-0.0467494640985745	4.8705112732393	1.24519831997585	0.264471365955519	0.747906079543733
**Retreg2**	Retreg2	-0.0275892229332445	7.94652669770382	1.24493089362906	0.26452171102917	0.747906079543733
**Nyap1**	Nyap1	0.0362552522287948	8.30524971246161	1.24486305025173	0.264535879657064	0.747906079543733
**Usp8**	Usp8	0.0487489888122433	5.76860486442479	1.24462787087536	0.264581010763761	0.747906079543733
**Ttc1**	Ttc1	-0.0412838153288382	5.41800371474457	1.24459439447102	0.264587435690153	0.747906079543733
**Smim8**	Smim8	-0.0825205627659063	2.42718656247129	1.24282839790325	0.264715296756828	0.748098019849365
**Zfp563**	Zfp563	0.0541160203888191	4.21322376628839	1.24253939009389	0.264790015218263	0.748139723704317
**Scn10a**	Scn10a	-0.263821314541657	-0.820909827544172	1.24299727609056	0.264894186588312	0.748260781291825
**Scaf4**	Scaf4	0.0630343033678615	5.906629694603	1.24269234401497	0.264952803698588	0.748260781291825
**Eif2b4**	Eif2b4	0.0353477429219778	5.5489274392902	1.24197512255678	0.265090738822543	0.748275984894913
**Ap4e1**	Ap4e1	0.0773977077817018	4.06298402560363	1.24179259825068	0.265125855901918	0.748275984894913
**Uqcrc1**	Uqcrc1	-0.0450380156344811	7.74852308803556	1.2417289424945	0.26513810444982	0.748275984894913
**Elp6**	Elp6	-0.0501851268860266	4.52255130871443	1.23965724654844	0.265556400270574	0.748582230950575
**Chfr**	Chfr	0.0311039478820761	6.22128994020649	1.23942757550668	0.265581402502116	0.748582230950575
**Ampd3**	Ampd3	-0.0806262421109197	4.25435288871149	1.23922505850244	0.265620456031637	0.748582230950575
**Dok1**	Dok1	0.186090528361249	-0.063552864572825	1.23906592367613	0.265651147139185	0.748582230950575
**Rpl19**	Rpl19	-0.0480027480231763	8.78721912968177	1.23899896015865	0.265664065855386	0.748582230950575
**Vegfc**	Vegfc	0.0871946330912601	2.05223260460573	1.23873607996616	0.265714781136132	0.748582230950575
**Elavl4**	Elavl4	0.0389377380706848	7.62389252710572	1.23841607023078	0.265776534203972	0.748582230950575
**Robo1**	Robo1	0.0542697535155092	7.75390539045677	1.23838405862341	0.265782712391031	0.748582230950575
**Lhx5**	Lhx5	-0.127821370794852	2.27089889178894	1.23836378516349	0.265786625479767	0.748582230950575
**Opa3**	Opa3	-0.0441689939478052	5.25494215514247	1.23783774095443	0.26588818114498	0.748618027459836
**Rassf8**	Rassf8	0.0968201904423151	2.02515644481242	1.23767658476174	0.265919300879092	0.748618027459836
**Dnaja4**	Dnaja4	-0.0417786496665443	5.16586863123579	1.23697278616337	0.266055270341823	0.748831853835985
**Aldh1l2**	Aldh1l2	0.0640285420690804	5.79395591942546	1.23659284657259	0.266126707171858	0.748858185716893
**RGD1304**	RGD130466	0.0346518259074977	7.83006585128997	1.23630343036292	0.266184664074179	0.748858185716893
**Adra2a**	Adra2a	0.0545467524914707	5.19871240375324	1.23323080505746	0.266779642288788	0.750279090637755
**Plpp5**	Plpp5	0.0626934882490784	4.27861551854775	1.2330290750589	0.266818762876531	0.750279090637755
**Parp16**	Parp16	0.0596055726121232	3.36798927464813	1.23258824853929	0.26690427505249	0.750279090637755
**Cand2**	Cand2	0.0477243170358009	5.01262327315038	1.23139666628837	0.267135591576555	0.750279090637755
**Thrb**	Thrb	-0.0740227968829197	4.66297204826596	1.23128397273784	0.267157481202924	0.750279090637755
**Gtf2h4**	Gtf2h4	0.0560216477050333	4.10208006653336	1.23112964265442	0.267187461918328	0.750279090637755
**Rprd1a**	Rprd1a	0.0338094608966326	6.59668746317202	1.23068394834889	0.267274087069811	0.750279090637755
**Tnik**	Tnik	0.0557915675881138	7.22185295144761	1.23048383697947	0.26731296393687	0.750279090637755
**Rraga**	Rraga	-0.0432896415604364	6.73810094018329	1.23007224865614	0.267392987888933	0.750279090637755
**Snx24**	Snx24	-0.0475725342463677	4.31330898187483	1.2300665826228	0.267394087783445	0.750279090637755
**Sra1**	Sra1	-0.0553960338620325	5.373405681677	1.22897870330451	0.267605747926789	0.750279090637755
**Cpt1a**	Cpt1a	0.0525402709937101	5.16889406575922	1.22862834964023	0.267673957363089	0.750279090637755
**Ssb**	Ssb	-0.0316118931419696	6.84867332088777	1.22797122677281	0.267801949245013	0.750279090637755
**Tinf2**	Tinf2	0.0638985865433858	3.90249465092491	1.22788987600926	0.267817796748635	0.750279090637755
**Got1**	Got1	-0.0524898811626709	6.93327307863961	1.22786550008749	0.26782254941722	0.750279090637755
**Ajap1**	Ajap1	-0.05666829224922	5.23470713438072	1.22782795526291	0.267829865262928	0.750279090637755
**Keap1**	Keap1	-0.0312616317387918	6.27258322447458	1.22762762451597	0.267868905184057	0.750279090637755
**Emc9**	Emc9	-0.0681634048707161	3.4462319905742	1.22734062081361	0.267924848065857	0.750279090637755
**Ift46**	Ift46	-0.0409307278122906	5.26357488826882	1.22667702578695	0.26805425206764	0.750279090637755
**Vipr2**	Vipr2	-0.155924041933421	1.43955092716815	1.22663819943888	0.268061825814052	0.750279090637755
**Uhrf1**	Uhrf1	0.0821540983487577	3.45568945777434	1.22645738473068	0.268097100253021	0.750279090637755
**Snrpa1**	Snrpa1	-0.047955722285214	5.16093101800136	1.22633654562323	0.268120677512548	0.750279090637755
**Mrto4**	Mrto4	0.0390172721115331	5.17963058628535	1.22615039094807	0.268157003738074	0.750279090637755
**Gtf2i**	Gtf2i	0.0420167021419647	8.22680063961167	1.22586233805707	0.268213226287664	0.750279090637755
**Eif4ebp1**	Eif4ebp1	0.110568091821667	3.50246183154078	1.22581345325727	0.268222768876181	0.750279090637755
**Pigu**	Pigu	0.0364276233372148	5.14487369483428	1.22565763897758	0.268252188804843	0.750279090637755
**C3**	C3	-0.226731125631309	-0.70338462552975	1.22473104493376	0.268434176893759	0.750617038246803
**Acot1**	Acot1	-0.0857787309311875	4.73253229451638	1.2237462498865	0.268626599997578	0.750967611892805
**LOC1001**	LOC100125	-0.126685735281028	1.08492452864248	1.22347415044592	0.26867992462046	0.750967611892805
**Vsig8**	Vsig8	-0.206260696406576	-0.174124894599819	1.22213037828934	0.268942968263008	0.751534474242902
**Crebbp**	Crebbp	0.0794982398167529	6.52514753891194	1.22164227864989	0.269038594859911	0.751633351560034
**MGC9520**	MGC95208	-0.0637173748598065	3.17430729428184	1.22088366207	0.269187302682474	0.751827099042066
**Gpnmb**	Gpnmb	0.137873590796087	2.52987320405841	1.22067380195215	0.269228458645504	0.751827099042066
**Abcb7**	Abcb7	0.0615312088093882	4.03507430733126	1.21970134260006	0.269419277163312	0.752191598093949
**Prkcz**	Prkcz	-0.0290105875173555	7.30812960416791	1.21948520675078	0.269658135173836	0.752385352117766
**Maml3**	Maml3	-0.0945315290364716	3.22886723152131	1.21823799076414	0.269687068752997	0.752385352117766
**Ext2**	Ext2	0.0349819642812162	6.02383707828374	1.21790501091751	0.269772186618544	0.752385352117766
**Manbal**	Manbal	-0.0500648747420985	5.28018120890369	1.21784714025804	0.269783565734371	0.752385352117766
**Snapc2**	Snapc2	-0.0416173928973458	4.93658871356461	1.21760885083778	0.269830426934623	0.752385352117766
**Gja5**	Gja5	0.190555631105471	0.171774401500614	1.21730532956231	0.269890121140554	0.752385352117766
**Psmd9**	Psmd9	-0.0504988517399803	4.90344839232648	1.21703763266416	0.269942802204774	0.752385352117766
**Fzd2**	Fzd2	0.0854938142265228	4.10367823423195	1.21645897986285	0.270056696712218	0.752385352117766
**Apoh**	Apoh	-0.259157689275574	-0.080255808210555	1.21627400890448	0.270093121013364	0.752385352117766
**Traf2**	Traf2	0.081434220602888	2.88130350792311	1.21622109411935	0.270103540705766	0.752385352117766
**Bmerb1**	Bmerb1	-0.0309032044665952	8.15219425328156	1.21558587866275	0.270228667838335	0.752385352117766
**Sae1**	Sae1	-0.0372256641435887	7.43973839881307	1.21554678451331	0.270236371117347	0.752385352117766
**Ecrg4**	Ecrg4	0.247854361545147	1.4602576899424	1.21504576589035	0.270335118279522	0.752385352117766
**Mcm6**	Mcm6	0.0449546547945031	5.66834162468081	1.21502088889255	0.270340022531815	0.752385352117766
**Zfp668**	Zfp668	0.0421681185051881	4.85000879579022	1.2148669475622	0.270403894691936	0.752385352117766
**Chst15**	Chst15	0.0605374291151959	4.10179422850582	1.21444543571593	0.270453498639673	0.752385352117766
**Adcy7**	Adcy7	-0.0567409433901271	4.27062042676563	1.21412148873497	0.270517405032124	0.752395377204014
**Ccdc166**	Ccdc166	0.0725689574948084	2.4643974389561	1.2133628751484	0.270667134301627	0.752553569699263
**Drd2**	Drd2	-0.0645515987664432	2.94804940711362	1.21322219370194	0.270694912303462	0.752553569699263
**Trerf1**	Trerf1	0.0781262593431363	4.69769073086454	1.21233013578767	0.270871134986312	0.752875729823863
**Tmem110**	Tmem110	0.0429430584100579	4.60313013882926	1.21055552694818	0.271222129107547	0.7536834086581
**Prkrip1**	Prkrip1	0.0495082930180989	4.14454678820398	1.20958572036028	0.271414184435406	0.754002453180548
**Ppp1r1a**	Ppp1r1a	-0.0461862949650734	7.08097328111079	1.20936557807582	0.271457803934201	0.754002453180548
**Sult4a1**	Sult4a1	-0.0461632310571815	7.83146215604291	1.20884480540392	0.271561025963897	0.754121282178228
**Lrrc51**	Lrrc51	-0.177541950645183	-0.246138218673372	1.2084903116149	0.27163131804273	0.754145203893673
**Gpr45**	Gpr45	0.0578204979011893	3.77022997487448	1.20819180642882	0.271690525887799	0.754145203893673
**Herc3**	Herc3	-0.0349336004922724	6.67536842010273	1.20757241520946	0.271813432288029	0.754285777257606
**Tefm**	Tefm	0.0773660231175234	2.39912758315239	1.20732736315371	0.271862077448702	0.754285777257606
**Hypk**	Hypk	-0.0727328438103757	5.52385818752164	1.20616491194663	0.272092983478891	0.754337377329738
**Spock1**	Spock1	-0.0474149341728944	7.17177628126259	1.20605407912838	0.272115011816362	0.754337377329738
**Numa1**	Numa1	0.0728877612648137	7.63247061038632	1.20604158767165	0.272117494668907	0.754337377329738
**Zic1**	Zic1	-0.13446849424678	6.07285453145544	1.20601636435457	0.272122508244061	0.754337377329738
**Ubxn6**	Ubxn6	-0.0321668194656399	6.32972748551348	1.20513429578428	0.272297907560794	0.754378230186393
**Plaur**	Plaur	-0.235596932017341	-0.475269525130765	1.2049800715984	0.272328589576086	0.754378230186393
**Map1lc3b**	Map1lc3b	-0.0302264659972849	7.87983910726963	1.20455272721556	0.272413629908	0.754378230186393
**Tom1l2**	Tom1l2	0.0390519409221932	5.71620016621407	1.20436020820165	0.272451951218539	0.754378230186393
**Sms**	Sms	-0.0294053631542513	7.25800008528726	1.2041742823848	0.272488964946594	0.754378230186393
**Pitpnm1**	Pitpnm1	-0.0312201921629206	8.11737015398941	1.20385125253135	0.272553290718854	0.754378230186393
**Bbs7**	Bbs7	0.0574545789609694	3.73445959128625	1.20326321826614	0.272670440058337	0.754378230186393
**Amigo2**	Amigo2	0.0521290667472227	4.56551400305584	1.20265535541072	0.272791603510619	0.754378230186393
**Adamts1**	Adamts1	0.154882029812717	2.58152025301967	1.2024427000276	0.272834007317544	0.754378230186393
**Robo2**	Robo2	0.0439779396234231	8.34519284575917	1.20206559008829	0.272909223924065	0.754378230186393
**Thrap3**	Thrap3	0.0321926405747224	7.66973207411302	1.20134821624096	0.273052376741229	0.754378230186393
**Abcg1**	Abcg1	-0.0398440662151482	5.13232137404971	1.20133204899022	0.273055607087405	0.754378230186393
**Acly**	Acly	0.0283246019486287	8.97886065389947	1.20132035216761	0.273057942065577	0.754378230186393
**Cks2**	Cks2	0.100521882354752	2.1191903671658	1.20112756259861	0.273096431288082	0.754378230186393
**Wnk1**	Wnk1	0.0795452724532325	6.98978902887204	1.20019014284722	0.273283678178284	0.754378230186393
**Hnrnpul1**	Hnrnpul1	0.0219876752865025	8.12126709794994	1.20013003735624	0.273295688565629	0.754378230186393
**Ankfy1**	Ankfy1	0.0515665509170845	7.54304813792525	1.1999851431213	0.273322849027845	0.754378230186393
**Yy1**	Yy1	0.031688457302645	6.06119858460422	1.19957089897154	0.273407458625683	0.754378230186393
**Rnf43**	Rnf43	0.152091324104661	0.494669275390038	1.19950875168718	0.273419885105617	0.754378230186393
**Rps26**	Rps26	-0.053465256197242	7.72178516255349	1.19942298384942	0.273437035727673	0.754378230186393
**Stk4**	Stk4	0.0404869279406609	6.01492984185212	1.1989017758745	0.27354128843067	0.754378230186393
**Slc38a9**	Slc38a9	0.0782361659822345	2.28297354441581	1.19860593288701	0.273600485505476	0.754378230186393
**Aldh3b1**	Aldh3b1	0.164290800889331	0.963383445831683	1.19845567590338	0.273630557516703	0.754378230186393
**Tlcd1**	Tlcd1	-0.0844041748508579	2.51007872820862	1.19828358742463	0.273665003391282	0.754378230186393
**Spin1**	Spin1	-0.0323806874193831	6.59867163899778	1.19791587132515	0.273738626088055	0.754378230186393
**Nup210l**	Nup210l	0.131972983630465	0.117557815769858	1.19772350748697	0.273777150968519	0.754378230186393
**Saraf**	Saraf	-0.0462542508316723	7.24681228918169	1.19736590217762	0.273848765863862	0.754378230186393
**Rtkn2**	Rtkn2	0.0722257930170003	3.10510698428104	1.1969599009019	0.273930143855907	0.754378230186393
**Akna**	Akna	0.0827652017511889	4.53795556128156	1.19656262709279	0.274009782312594	0.754378230186393
**Tecr**	Tecr	-0.0330129357817179	8.85801680004369	1.19589598796273	0.274143483658043	0.754378230186393
**Zfpl1**	Zfpl1	0.0412788907568038	5.00579593852291	1.19569386247272	0.274184038170788	0.754378230186393
**Cert1**	Cert1	-0.0380540933436173	6.3658051894601	1.19568197439045	0.274186025513638	0.754378230186393
**Nat8b**	Nat8b	-0.0945044883527727	1.90083207715352	1.19561340899281	0.274200182474428	0.754378230186393
**Zc2hc1c**	Zc2hc1c	0.141203813758711	0.835564226305813	1.19540111276473	0.274242788903094	0.754378230186393
**Surf1**	Surf1	-0.0500903398779567	4.2985586529175	1.19495652429882	0.274332041741172	0.754378230186393
**Artn**	Artn	-0.213458780540493	-0.492167520104984	1.19483649189008	0.274356146963614	0.754378230186393
**Smad3**	Smad3	0.0995041438058843	4.79316851621516	1.19473366105083	0.274376796106754	0.754378230186393
**Micu3**	Micu3	-0.0405987871998252	5.18937654477423	1.19444497190481	0.274434782945903	0.754378230186393
**Trim66**	Trim66	-0.117361778282788	2.02436928912216	1.19354426263608	0.274615800583881	0.754709547111528
**Prr15**	Prr15	-0.156209143170844	0.685183256877512	1.19311710120319	0.27470170058822	0.754779368822505
**Anp32e**	Anp32e	-0.0380871357712947	6.55964431913035	1.19207323096408	0.274911758611273	0.75519023000217
**Lypd6b**	Lypd6b	-0.0677664663515354	4.32456881020231	1.19136517553737	0.275054257089405	0.755342610929559
**Slc6a9**	Slc6a9	-0.0462345156280556	6.22213763069981	1.19119666110741	0.275088308412592	0.755342610929559
**Fuca1**	Fuca1	0.0577580663412628	5.0466009861113	1.18953058160923	0.27542426398261	0.755808047140418
**Slc28a2**	Slc28a2	0.185518369587132	3.92402942885694	1.18898726827554	0.275533921460464	0.755808047140418
**Gcfc2**	Gcfc2	0.0859915016846732	3.53389770002798	1.18888792110867	0.275553990583469	0.755808047140418
**Psmc6**	Psmc6	-0.0342487728392177	6.86686320706268	1.18823882419614	0.275664891277003	0.755808047140418
**Hipk2**	Hipk2	0.0960425195399752	5.62447822758286	1.18809916803387	0.275713312175307	0.755808047140418
**Med23**	Med23	0.0672079793154079	6.14024134221525	1.188063015174	0.275720617527373	0.755808047140418
**Cog3**	Cog3	0.0521878976787876	4.96082079920476	1.18687459274664	0.275960895564683	0.756462128248033
**Phlpp2**	Phlpp2	0.0652909508353154	5.18507857764939	1.18677492114753	0.275981057445792	0.756462128248033
**Itfg1**	Itfg1	-0.0294545255188189	8.55928376767716	1.18639282905377	0.276058374775891	0.7565076721895
**Maob**	Maob	-0.0740323458600917	3.64564113850763	1.18543035597688	0.276252107515629	0.756712575231866
**RGD1311**	RGD131194	0.0394383649448544	4.794670068229	1.18542482117242	0.276254370456168	0.756712575231866
**Pdgfc**	Pdgfc	0.102809586914553	2.27151362981472	1.18452599622984	0.276436515093619	0.757045302638955
**Ifngr2**	Ifngr2	-0.037592171424579	5.83704459113194	1.18243390680491	0.276861058981576	0.758041564177995
**Myo18a**	Myo18a	0.0318693973719219	8.31909983852994	1.18155399625456	0.277039858146857	0.758294498822082
**Them4**	Them4	-0.0375559042502186	5.16422532097415	1.18095540098382	0.277161580720439	0.758294498822082
**Clstn1**	Clstn1	-0.0313027675051625	9.97370760478083	1.18090730457187	0.277171363753702	0.758294498822082
**Pxn**	Pxn	0.0479882789760243	5.48147001932008	1.18078254860822	0.277196538360785	0.758294498822082
**Pyurf**	Pyurf	-0.0724159941319251	3.76032590284579	1.17920496866775	0.277517907563666	0.758761436976108
**Lrrk1**	Lrrk1	0.117142735066439	2.28461594387418	1.17903095420978	0.277553362324489	0.758761436976108
**Prps1**	Prps1	-0.0319865461218048	7.16006623468567	1.17886556214926	0.277587065590658	0.758761436976108
**Arhgef40**	Arhgef40	-0.0691108348485817	6.3754477827735	1.17875067515352	0.277610480066998	0.758761436976108
**Taf8**	Taf8	0.0452196995682203	4.34809085864505	1.17789060182458	0.277791965208534	0.758898656340014
**Lama5**	Lama5	0.0946133022256724	3.96091271518932	1.17784677915617	0.277794784915685	0.758898656340014
**Ift88**	Ift88	-0.0621516907860136	4.84320660055069	1.17760967288108	0.277843156280635	0.758898656340014
**Zfp143**	Zfp143	0.0519022412338681	3.73478526300247	1.17628497586645	0.278113600339585	0.759445388849096
**Akap14**	Akap14	-0.178978249702835	-0.153193375589479	1.17602310868782	0.278166057868611	0.759445388849096
**Mrps36**	Mrps36	-0.0657207935390447	4.62141948661245	1.17479861865878	0.278417442903883	0.759968154694652
**Atp1b2**	Atp1b2	-0.046152768087924	8.08787305626152	1.1735064153919	0.278681936219457	0.760523725291551
**Hltf**	Hltf	0.0467485943387455	5.23849808321039	1.17250146849521	0.278887853446842	0.76081251990509
**Pex19**	Pex19	-0.0294435694453793	6.68677204462798	1.17239484059166	0.278909711152191	0.76081251990509
**Arnt**	Arnt	0.0925471144832379	4.57580870390833	1.17161573524596	0.27906949900587	0.761081997616656
**Slc6a8**	Slc6a8	0.0403009229550865	6.31630572363017	1.17062291048725	0.279273286150131	0.761228077192422
**Rpl4**	Rpl4	-0.0398217215953406	9.53913483228178	1.17037615877894	0.279323963291372	0.761228077192422
**Twnk**	Twnk	-0.0407425387811449	4.81552012557319	1.17029032653063	0.279341594261476	0.761228077192422
**Csmd1**	Csmd1	0.0936990487364957	5.7237170782367	1.17016614553317	0.279367104709794	0.761228077192422
**Tmem14c**	Tmem14c	-0.0507848606112532	5.27859720793828	1.16941232385248	0.279522022974699	0.761483904073214
**Dnajc18**	Dnajc18	0.040190148159172	5.06639373367088	1.16765890144067	0.279883207709853	0.762301415105234
**Gabrb2**	Gabrb2	0.0537188141005246	5.55206670358608	1.16722209262943	0.279972761701844	0.762350894484772
**Jak1**	Jak1	-0.0305796051034977	7.62152997912678	1.16670397880538	0.280079520554685	0.762350894484772
**Ppfibp2**	Ppfibp2	0.0990546559490557	1.5449986703918	1.1654418894708	0.280133542323583	0.762350894484772
**Khdrbs2**	Khdrbs2	0.0537155813254731	4.95230266829605	1.16638255671565	0.280145776225453	0.762350894484772
**Ccar2**	Ccar2	0.0311256076300266	7.80442882628418	1.16602496867027	0.280219510345282	0.762385266153093
**Fgd6**	Fgd6	0.0967015982718606	2.80361835540104	1.16537699149842	0.280353184500687	0.762390047680074
**Coa4**	Coa4	0.0833062931340173	2.6811389445023	1.16502238113882	0.280426372758795	0.762390047680074
**Mthfs**	Mthfs	0.102447910355315	2.30443720686273	1.16499345027728	0.280432344881743	0.762390047680074
**Lzic**	Lzic	-0.0245526916812676	6.55285716003373	1.16482127632222	0.280467889625033	0.762390047680074
**Kidins220**	Kidins220	0.0432772062541555	10.1288896693921	1.16351019308821	0.28073874508068	0.762966090693019
**Rnf25**	Rnf25	-0.0354343148563903	5.29695088426848	1.1626419884514	0.280918288643599	0.763098375288847
**RGD1311**	RGD131186	-0.0403891774893816	7.54313175340434	1.16204898162891	0.281041004681302	0.763098375288847
**Ddx25**	Ddx25	-0.0365752588998182	5.92455123125959	1.16181727059227	0.281088973127174	0.763098375288847
**Pde2a**	Pde2a	-0.0387345054059405	7.88534523397254	1.16140164304078	0.281175041588757	0.763098375288847
**Dnmt1**	Dnmt1	0.0398813052091328	7.32978091746438	1.16089887491414	0.281278992464681	0.763098375288847
**Mapk10**	Mapk10	0.0366339412349797	6.83620072908457	1.1603055003234	0.281402191370743	0.763098375288847
**Lrrc34**	Lrrc34	0.0712322575445617	2.77921268813293	1.15996385705862	0.281473036285611	0.763098375288847
**Trpc5**	Trpc5	0.051523996460122	5.28399754648945	1.15937345521559	0.281595518180314	0.763098375288847
**Ndp**	Ndp	-0.0910836787417083	2.56489774364533	1.15913052903726	0.281645934050408	0.763098375288847
**Galnt1**	Galnt1	-0.0354682700856974	5.64882799088475	1.15821460767783	0.28183612303407	0.763098375288847
**Senp2**	Senp2	-0.0347512649792091	5.12778897879307	1.15754189907373	0.281975912852789	0.763098375288847
**Pi15**	Pi15	0.223423656442271	0.0696608593373781	1.1575369485578	0.281976962688748	0.763098375288847
**Lpar2**	Lpar2	0.0920540843058813	2.47571455001287	1.15664896721462	0.282161600807095	0.763098375288847
**Gramd1b**	Gramd1b	0.052374049479669	5.81895453868449	1.15621974216721	0.282250914512037	0.763098375288847
**Zer1**	Zer1	-0.0339216924358746	7.46326601996877	1.15516728885629	0.282470061507141	0.763098375288847
**Dtx2**	Dtx2	-0.0859314001640759	4.8103075166189	1.15510648606271	0.282482728957977	0.763098375288847
**Tiparp**	Tiparp	0.0603588061203058	3.86723876156696	1.15485191572191	0.282535772222257	0.763098375288847
**Tmem212**	Tmem212	-0.276565141457675	-0.037197372503866	1.15439717729431	0.282630554643481	0.763098375288847
**Mphosph**	Mphosph9	0.0835380248245545	3.58197922245997	1.1537828404649	0.282758666499905	0.763098375288847
**Filip1**	Filip1	-0.114998287949443	3.43887695065894	1.15277210730951	0.282760905411058	0.763098375288847
**Ppef1**	Ppef1	0.161503280615081	0.234967307929228	1.15237638031675	0.282842842772299	0.763098375288847
**Mppe1**	Mppe1	-0.0963612362891305	4.07213345383778	1.15235569126839	0.2828477861437	0.763098375288847
**Dtx3l**	Dtx3l	0.0960146150264489	1.52318429422293	1.15274236826804	0.282975810919225	0.763098375288847
**Mff**	Mff	-0.0285741017394432	7.8809270384795	1.15233021864886	0.283061884048252	0.763098375288847
**Tst**	Tst	-0.0634408542320574	5.12394339488765	1.15225187422972	0.283078248206748	0.763098375288847
**Ndufa13**	Ndufa13	-0.069121166124114	5.88051886578349	1.15184894964792	0.283162851922312	0.763098375288847
**Higd1a**	Higd1a	-0.0519981469070372	5.44521381869426	1.15176116665107	0.283180779058775	0.763098375288847
**Matk**	Matk	-0.0795985250887506	6.48000702079185	1.15174016118021	0.283185168051215	0.763098375288847
**Drc1**	Drc1	-0.0882709069596236	3.37311639676241	1.15171014023915	0.283191443361665	0.763098375288847
**Surf4**	Surf4	0.0366681047545628	7.02171402306044	1.15162769817154	0.283208674477011	0.763098375288847
**Haus8**	Haus8	-0.0737590576091713	3.25461793697802	1.15154453729999	0.283226026209888	0.763098375288847
**Bnip2**	Bnip2	0.0578776623347212	4.80509001706013	1.15152888512716	0.283229329018687	0.763098375288847
**Klf5**	Klf5	0.0814160151295006	3.52699413503437	1.15152516190975	0.283230103126211	0.763098375288847
**Gcn1**	Gcn1	0.0477452877564789	7.76372028676322	1.1515052670356	0.283234247293615	0.763098375288847
**Mrpl34**	Mrpl34	0.0642856801136107	4.98939429810169	1.15137280100426	0.283261958616436	0.763098375288847
**Dclk3**	Dclk3	-0.0790303234405708	5.10346372320953	1.15105471319536	0.283328470035848	0.763098375288847
**Fanca**	Fanca	0.0819150421323401	3.85184213069528	1.15066667726818	0.283409634215429	0.763098375288847
**Mtdh**	Mtdh	0.033309936932272	6.02670457818285	1.15029013709566	0.28349051480101	0.763098375288847
**Baz2a**	Baz2a	0.0911294601842841	6.24738287734647	1.15001104343683	0.283546827853039	0.763098375288847
**Ppp2r5b**	Ppp2r5b	-0.0273328272884328	7.8881852488419	1.14980396411031	0.283590190549487	0.763098375288847
**Osbp**	Osbp	0.0260055816114102	6.9040989184763	1.14938800129827	0.283677298993515	0.763098375288847
**Ccnd1**	Ccnd1	0.0466419861878904	5.52676624715995	1.14935162028511	0.283684918321652	0.763098375288847
**B3gat1**	B3gat1	0.0415374062291039	8.1728754754568	1.149115001936	0.283734487338245	0.763098375288847
**Scn7a**	Scn7a	-0.207042973034898	0.460437920053429	1.14899029009371	0.283760616604865	0.763098375288847
**Hbp1**	Hbp1	0.0444723017822552	5.11283452304073	1.14889660002884	0.283780249016486	0.763098375288847
**Pih1d1**	Pih1d1	0.0501525512058973	4.10629974394045	1.1486122468302	0.283839843455183	0.763098375288847
**Cbx8**	Cbx8	0.0519100929517365	3.92040695326745	1.1484559247361	0.283872612024355	0.763098375288847
**Mfsd8**	Mfsd8	0.0596308535813252	3.46550240546688	1.1483720075227	0.283890204905273	0.763098375288847
**Apc**	Apc	0.0546902861506689	9.57439745026074	1.14817969401766	0.283930527809232	0.763098375288847
**Zfp691**	Zfp691	0.100852897375026	2.03519637403027	1.14810899645246	0.28394535298669	0.763098375288847
**Prelid3b**	Prelid3b	-0.0424187217175747	5.25312803511554	1.14767046071636	0.284037335206762	0.763098375288847
**Gpr155**	Gpr155	0.0479217056005052	4.88058326412515	1.14713325688446	0.284150064261803	0.763098375288847
**Hnrnpm**	Hnrnpm	0.0349810600750784	8.02553282742503	1.14710646864276	0.284155687036934	0.763098375288847
**Prelid2**	Prelid2	0.215991899128778	-0.337979448808851	1.14682226823648	0.2842153492271	0.763098375288847
**Frg1**	Frg1	0.0478257825211344	4.27201380573409	1.14672938435707	0.284234852032326	0.763098375288847
**Mpzl2**	Mpzl2	0.233183005189882	0.354142953238671	1.14614386472606	0.284357831032605	0.763098375288847
**Reln**	Reln	0.0913221211077644	9.26891969013977	1.14583826374789	0.284400822080277	0.763098375288847
**Fa2h**	Fa2h	-0.157791611549589	0.675169590249578	1.14578132657567	0.284434010151714	0.763098375288847
**Spag8**	Spag8	0.162155738583392	1.44405901429785	1.14556806864279	0.284479253931356	0.763098375288847
**Rfxap**	Rfxap	-0.0460173870146873	6.03522510983358	1.14541452550252	0.284511111350084	0.763098375288847
**Snx7**	Snx7	0.072198760890301	4.63989256867242	1.1453491360186	0.284524858932327	0.763098375288847
**Plch1**	Plch1	-0.0824605633919435	4.45380776464792	1.14489905522439	0.284619507432057	0.763098375288847
**Atp5po**	Atp5po	-0.0547562388167249	7.26907123773013	1.14479813328486	0.284640525654748	0.763098375288847
**Nat1**	Nat1	0.0719116585555257	3.04240312501263	1.1429854158489	0.285022376949752	0.763281236630243
**Tas1r1**	Tas1r1	-0.0769762522926612	2.55861347516929	1.14297134300722	0.285025342215443	0.763281236630243
**Auts2l1**	Auts2l1	0.0769452551787329	7.02383969011409	1.14254575439843	0.285115038811034	0.763281236630243
**Ofd1**	Ofd1	0.0438008759404021	4.67776288337657	1.1425215287177	0.285120145668056	0.763281236630243
**Cd151**	Cd151	0.0427035132953965	5.81867104521405	1.14250032254053	0.285124616094781	0.763281236630243
**Raph1**	Raph1	0.0720096431546024	6.36419950724592	1.14241813943043	0.285141730953637	0.763281236630243
**Lrrc7**	Lrrc7	0.0742242516323338	5.49785344243771	1.14240051868144	0.285145656725913	0.763281236630243
**Dnm1l**	Dnm1l	-0.0243460323886151	7.34483865556917	1.14198274825628	0.285233752385746	0.763281236630243
**Cd2bp2**	Cd2bp2	-0.0332669661894586	7.19447970095642	1.1418615803329	0.285259309642288	0.763281236630243
**Hr**	Hr	-0.0501919070984655	5.05296118323048	1.14104209054389	0.285432236207659	0.763580191072676
**Lcmt1**	Lcmt1	-0.0354436010860145	5.9585257301324	1.13971178129827	0.28571323781075	0.76396062887509
**Mid1**	Mid1	0.0884209174292983	3.04973429274867	1.13967685408051	0.285720620224847	0.76396062887509
**Syn3**	Syn3	0.055538702403424	6.29108095871622	1.13940215868057	0.285778689622373	0.76396062887509
**Sema3d**	Sema3d	0.0956723005815329	3.47910613341423	1.13915788380986	0.285830341234721	0.76396062887509
**Nf2**	Nf2	-0.0232045889420193	8.2275787283439	1.1388639901455	0.285892500126389	0.76396062887509
**Wdr77**	Wdr77	-0.0336820038873828	5.8075805721446	1.13858966887879	0.285950534911308	0.76396062887509
**Galnt18**	Galnt18	-0.0582149738615456	5.05544523035999	1.13834148309672	0.28600305341633	0.76396062887509
**Ahi1**	Ahi1	-0.0723859464608374	7.66157486110503	1.13802959319085	0.286069068689674	0.76397341235484
**Zfp384**	Zfp384	0.0528164057260942	6.1130414557349	1.13674671873325	0.286340813219068	0.764134020097307
**Ap3s2**	Ap3s2	-0.0469870414131261	5.45522630089103	1.13557370354161	0.286589573942667	0.764134020097307
**Nek6**	Nek6	-0.0400473865940896	5.45489299458923	1.13529355201285	0.286649024765932	0.764134020097307
**Dnmbp**	Dnmbp	0.0578834599196009	3.80923140406828	1.13513870949949	0.286681891151888	0.764134020097307
**Plekhg1**	Plekhg1	-0.0867269813103275	3.48698481342823	1.13499704748764	0.286711964078647	0.764134020097307
**Tfcp2**	Tfcp2	0.0414291173688825	5.10841060065265	1.13493775005971	0.286724553309261	0.764134020097307
**Timp1**	Timp1	0.094193051154717	2.63204563027934	1.13482919080262	0.286747602966897	0.764134020097307
**Tmem70**	Tmem70	-0.0396971104779912	5.2513588704841	1.13430013808703	0.286859966655254	0.764134020097307
**St8sia4**	St8sia4	0.0591655188220662	4.58012969217777	1.13404598384626	0.286913965804819	0.764134020097307
**Cnih1**	Cnih1	-0.047758746852286	5.98934143166765	1.13388848569001	0.286947435166609	0.764134020097307
**Smim7**	Smim7	-0.0422165666076082	6.13758964396928	1.13348890044683	0.287032796616121	0.764134020097307
**Dnajc14**	Dnajc14	0.0298479599239783	6.39096415806375	1.13322719498383	0.287066753365489	0.764134020097307
**Lhpp**	Lhpp	-0.0446474873264329	6.06775189002037	1.13320214136382	0.287093345632556	0.764134020097307
**Camkmt**	Camkmt	-0.0578974926445119	4.2962180229029	1.13308728831903	0.287117771536334	0.764134020097307
**Tcp11l2**	Tcp11l2	0.0579086421616283	3.81386124617269	1.13293670134541	0.287149801009049	0.764134020097307
**Qtrt1**	Qtrt1	0.0520875817122554	4.21477530946555	1.13284815171774	0.287168637413371	0.764134020097307
**Nox1ap**	Nox1ap	0.0608907269462983	4.59939834528105	1.13257147144434	0.287227503235862	0.764134020097307
**Btg4**	Btg4	0.18879825796459	-0.326419137564169	1.13255226399318	0.287231590466869	0.764134020097307
**Snx5**	Snx5	-0.0337072531507133	5.69778044513992	1.13094170388509	0.287574560879428	0.764883350264895
**B9d2**	B9d2	-0.0629230698532367	4.23022596768009	1.13018368501504	0.287736161937666	0.76495345661708
**Bud13**	Bud13	-0.0397057423912131	4.28408262702704	1.12990764804285	0.287795038579137	0.76495345661708
**Plcxd2**	Plcxd2	0.0351322980212959	7.61633439000076	1.12979781111786	0.287818470241968	0.76495345661708
**Psmb1**	Psmb1	-0.0506355943877622	7.25335685581751	1.12957300992919	0.287866434953869	0.76495345661708
**Pdxdc1**	Pdxdc1	0.058727025921192	4.91399632981602	1.12909386372941	0.287968701923196	0.76495345661708
**Recql5**	Recql5	0.0597151326844935	4.2893460141154	1.12909352891148	0.287968773401493	0.76495345661708
**LOC1001**	LOC100125	0.0620748073348726	3.22344845525031	1.12878218361445	0.288039948511373	0.764979658913667
**Lrch1**	Lrch1	0.0568789404372571	4.08245193020927	1.12818908706483	0.288166213091656	0.765011824624528
**Dhx58**	Dhx58	-0.131420020712761	0.773060535247038	1.12812676601137	0.288175256024407	0.765011824624528
**Tom1**	Tom1	-0.0443220991009049	5.25164053040692	1.12784252129047	0.288236001247087	0.765011824624528
**Atp5f1d**	Atp5f1d	-0.0503881674941082	7.56078874664223	1.1268820966911	0.288439232361594	0.765388409650279
**Champ1**	Champ1	0.0339632054709383	6.16254625826159	1.12608739361789	0.288611446237512	0.765665729804369
**Ndufa4**	Ndufa4	-0.0465549419947989	7.58659403782692	1.12583039548845	0.288666473751683	0.765665729804369
**Jpt2**	Jpt2	0.0436576920090893	5.20288257745136	1.12540187945882	0.288758255758312	0.765746388230811
**Zpr1**	Zpr1	0.0307282918298313	6.02060773376184	1.12464963062785	0.288819466556918	0.766011088871796
**Tax1bp1**	Tax1bp1	-0.0271598559640559	7.32484599783859	1.12419260119768	0.289017466268702	0.7660967249627
**Neurod6**	Neurod6	0.0569153630785587	7.85619693632111	1.1238192458448	0.289097555360027	0.7660967249627
**Npy5r**	Npy5r	0.13061178629685	0.625022870713121	1.12346594424498	0.289173368709842	0.7660967249627
**Nr2f1**	Nr2f1	0.0721904152773583	8.09292353709284	1.12235412328173	0.289197368125136	0.7660967249627
**Wasl**	Wasl	0.0380400974008076	5.9040595818039	1.12302527418912	0.289267965535257	0.766121079596881
**Mrps2**	Mrps2	0.0303251986146996	6.25693651891344	1.1219864096243	0.289512623497749	0.766603224996057
**Mib1**	Mib1	0.0475611154473048	7.12812813970386	1.12100821072733	0.289701463651373	0.766843594733233
**Col4a3**	Col4a3	-0.165252952126478	0.759375981992917	1.11983339657873	0.289932795803509	0.767128961397418
**Scrt2**	Scrt2	0.0590853799729151	4.58390822138617	1.11978792191286	0.289964124417651	0.767128961397418

Dqx1	Dqx1	0.160064723448249	0.912281195557529	1.1193098887141	0.290054171496388	0.767128961297418
Atp5mc3	Atp5mc3	-0.0544113225857759	7.58445777321075	1.11929954778699	0.290069307864127	0.767128961297418
Rbm38	Rbm38	0.0597457114713055	3.56428272181198	1.1192551294726	0.290078900366516	0.767128961297418
Rplp0	Rplp0	-0.0532534914870342	10.2635412659508	1.1189545867272	0.290143667688752	0.767137643909837
Por	Por	0.0287183792035514	6.82177816814924	1.11697974063539	0.290569724585995	0.767139784827127
Glrx5	Glrx5	-0.0632584205248335	6.07283219324635	1.11696275621384	0.290573392254258	0.767139784827127
Gpsm3	Gpsm3	-0.142111444018316	1.85701275214612	1.11687417188517	0.290592522517851	0.767139784827127
Trpv4	Trpv4	0.0307689471409774	8.24303369884383	1.11681234484843	0.290605658259883	0.767139784827127
Uhmk1	Uhmk1	-0.053244254131015	5.83489121411477	1.11665410627629	0.29064005377099	0.767139784827127
Plscr1	Plscr1	0.192955158328659	-0.303135888744707	1.11660799312328	0.290650014856807	0.767139784827127
Ubash3b	Ubash3b	-0.0471777404150616	5.06123656876285	1.11620735571969	0.290736576463592	0.767139784827127
Irf9	Irf9	0.0768278043575068	3.56275110161366	1.1158931800161	0.290804480180928	0.767139784827127
RGD1306	RGD130674	-0.0516432126696682	3.98092502624169	1.1158322777672	0.290817545503273	0.767139784827127
Vps18	Vps18	0.0305048235496761	5.69648925320143	1.11561411786919	0.290864811665816	0.767139784827127
Rarres1	Rarres1	-0.114495470258948	2.23683240141802	1.11557067691387	0.290874204785344	0.767139784827127
Dennd10	Dennd10	-0.0335976613095361	5.43449612975519	1.11553328677838	0.29088228917345	0.767139784827127
Abca17	Abca17	0.172897492379008	-0.0297243515181350	1.11507868452931	0.290980613704091	0.767147744856386
Rab2a	Rab2a	-0.0305641017321731	7.66480472271814	1.11473902898886	0.291054104383834	0.767147744856386
Sdad1	Sdad1	0.0662825864499079	4.16900687426329	1.11437487039981	0.291132922153847	0.767147744856386
Bag3	Bag3	-0.106149817775741	1.97008011964377	1.11299447166449	0.291217450002367	0.767147744856386
Ciao1	Ciao1	-0.0479277609555708	5.06178676803243	1.11288639025804	0.291238890537832	0.767147744856386
Uba7	Uba7	0.150253027776289	0.704852689536813	1.11281470472463	0.291254217150333	0.767147744856386
Hs3st2	Hs3st2	-0.0728648263927628	5.42976673678099	1.11283959896264	0.291455514714805	0.767542258834661
Bpifa1	Bpifa1	-0.234445778041908	-0.759251702524431	1.11222718569147	0.291598319763119	0.767626013098976
Dmac2l	Dmac2l	0.0577715152144548	3.67065600563931	1.11204039352341	0.291638842001548	0.767626013098976
Furin	Furin	0.0340545774133539	6.35176174555535	1.11184199980717	0.291681888238	0.767626013098976
Usp48	Usp48	0.0506747148868926	6.72685916806438	1.11094850149436	0.291875641105561	0.767823495081563
Prpf4	Prpf4	-0.0309175558525826	5.29829827955357	1.11082960880414	0.29190168106438	0.767823495081563
Edem3	Edem3	0.0407341603907726	5.88346603039955	1.11039543677859	0.291996004865467	0.767823495081563
Afdn	Afdn	0.0510282526689435	7.84863433123475	1.1103453593559	0.292006887405788	0.767823495081563
Cadps	Cadps	-0.0322727835993559	8.25578297652669	1.10956729858273	0.292176027551432	0.768116370787867
Rgs11	Rgs11	-0.121532411218493	3.02693560686559	1.10906922587418	0.292284367677349	0.768239320731047
[illegible]	[illegible]	[illegible]	[illegible]	[illegible]	[illegible]	[illegible]

Sh3bp5	Sh3bp5	0.0300624976072835	7.67074750036504	1.00483131791453	0.216144287560005	0.780434002405896
Dip2a	Dip2a	0.0480114838545076	6.8911774588336	1.0046460270443	0.216188910920301	0.780434002405896
Tcea2	Tcea2	-0.0395067882921937	5.95803546802871	1.00429427514882	0.216273645282183	0.780434002405896
Vps13b	Vps13b	0.0538538011139988	5.94814361344093	1.00424328644455	0.216285920556708	0.780434002405896
Emc2	Emc2	-0.0416282477471901	5.85128740843081	1.00366294649062	0.216425802362062	0.780434002405896
Smpd2	Smpd2	0.0544062905449578	2.89538512119372	1.00354061516632	0.216455296633591	0.780434002405896
Nphp3	Nphp3	0.0949612332671844	2.22521521491408	1.00335978653956	0.216498901292761	0.780434002405896
Rasgrp1	Rasgrp1	-0.138662060664027	5.04626216747675	1.00280848685307	0.216631889210369	0.780434002405896
Rpl39	Rpl39	-0.0598934838365861	6.52804542003812	1.00276872037563	0.216641484756881	0.780434002405896
Maco1	Maco1	0.0264222206581272	7.0249283886178	1.00272795878324	0.216651320817499	0.780434002405896
Six2	Six2	0.270944078177109	0.254657719590686	1.00245385710917	0.216717473890837	0.780434002405896
Idh2	Idh2	0.0499431260085686	5.62544339800021	1.00230980591411	0.216752247189424	0.780434002405896
Mboat1	Mboat1	0.0802935198492817	2.49773258626748	1.00163187753446	0.216915963197897	0.780683212797661
RGD1311	RGD131151	0.0462802060049214	4.3409564620237	1.00104149614504	0.21705862745236	0.780880476652803
Pacrg	Pacrg	0.108248641266142	2.12892826625002	1.00063655555432	0.217156529062269	0.780967438940188
Gtf2f1	Gtf2f1	-0.0291420534389531	6.60132214793699	1.00028768227875	0.217240907184934	0.780976750248815
Stim1	Stim1	-0.0301085127938856	5.91124837823957	0.999965219595808	0.217318923948871	0.780976750248815
Rps13	Rps13	-0.0480922309987498	7.76423743280146	0.999325897838099	0.217473651633985	0.780976750248815
Tpr	Tpr	0.0563511664533011	7.48610412597258	0.999109027055056	0.217526193216884	0.780976750248815
Cwh43	Cwh43	-0.224407647259819	-0.355707769661363	0.998599407751314	0.217649647682405	0.780976750248815
Trib2	Trib2	-0.0431585792923979	6.98883639163959	0.998475784361005	0.217679604717031	0.780976750248815
Ncam1	Ncam1	0.0502152988269855	10.1192318885549	0.998400256407791	0.217697908850122	0.780976750248815
Dhrs11	Dhrs11	0.0581418211097789	3.33099066708546	0.997899180477233	0.217819378208449	0.780976750248815
Phf12	Phf12	0.042568097162174	6.25721764943152	0.997849249244695	0.217831488829497	0.780976750248815
Zfp2	Zfp2	0.0380739891604808	4.90295639886474	0.997842628438026	0.217833089659833	0.780976750248815
Hcfc2	Hcfc2	0.0403766859519827	4.65597281898891	0.997774346363983	0.217849650858658	0.780976750248815
Dnajc8	Dnajc8	-0.0315873006103814	6.13475981994756	0.997138749134201	0.218003828705218	0.781203758701221
Dnase2	Dnase2	0.0868327012979529	2.25633179590419	0.99680557660465	0.218084701257741	0.781248586285832
Dgkz	Dgkz	-0.0304921108918247	8.21607671219857	0.995881598098777	0.218309098851667	0.781335328951545
Ipo4	Ipo4	-0.0249719038912099	7.6947492101291	0.994990658719473	0.218525665274463	0.781335328951545
Rrp1	Rrp1	-0.0254272061988067	6.2043773466718	0.994964789933405	0.218531956419127	0.781335328951545
Atp5f1b	Atp5f1b	-0.0406454790426933	10.2426287740756	0.994874124764458	0.218554006620699	0.781335328951545
Nostrin	Nostrin	0.118332444922747	1.92796552379941	0.994782513505442	0.218576288951799	0.781335328951545
Purg	Purg	0.0590811151888637	4.0274354267068	0.994758480399112	0.218582134620615	0.781335328951545
Unc119b	Unc119b	0.0442179284958191	5.41880135750411	0.994697721995379	0.218596914259741	0.781335328951545
Gsto1	Gsto1	-0.0523181391321941	6.4208210677949	0.994448728710779	0.218657491740051	0.781335328951545
Blvrb	Blvrb	0.054502503920753	3.78246438887691	0.99403258916481	0.218758767773588	0.781335328951545
Nck2	Nck2	0.0272632526773784	6.14115914341262	0.993891167980522	0.218793195123411	0.781335328951545
Glmn	Glmn	-0.0498519525876328	5.46090559486917	0.993826805011722	0.218808895516008	0.781335328951545
Gltpd2	Gltpd2	0.095784689522191	1.33296554394652	0.992924852357106	0.219028564228106	0.781529900140969
Ssr1	Ssr1	0.0266831366414094	7.06523908700598	0.992809574907106	0.219056658026812	0.781529900140969
Kcnma1	Kcnma1	0.0522495419010826	6.13025456721249	0.992714858502495	0.219079743409263	0.781529900140969
Tex261	Tex261	0.0375878921279193	5.07978053482311	0.992472586700828	0.219138802694417	0.781529900140969
Slc39a13	Slc39a13	0.0430827756138439	4.56745796061772	0.991865178538319	0.219286925470986	0.781739225641972
Coasy	Coasy	-0.0389942615999714	5.13885376464315	0.991342343411112	0.219414515215543	0.781898157022628
Paip1	Paip1	-0.0285001869675013	6.8798253824889	0.990814155174078	0.219543468245435	0.781944573832739
Slc25a5	Slc25a5	-0.0455090934938994	7.43811439061754	0.990427764584508	0.219637852452322	0.781944573832739
RGD1559	RGD155974	-0.0580636130444872	3.52440598990633	0.990243879078207	0.219682775622628	0.781944573832739
Nfe2l2	Nfe2l2	0.0851198843214436	3.18415624723704	0.989759406902508	0.219801184654126	0.781944573832739
Hs3st5	Hs3st5	-0.0500865561263114	5.58397469200487	0.989749229682943	0.219803672669662	0.781944573832739
Insyn1	Insyn1	-0.0321883343800241	6.18390247456481	0.989725383983114	0.21980950230572	0.781944573832739
Aurkb	Aurkb	0.0778652113854544	3.2563198337098	0.98917031215521	0.219945242021531	0.782036628816827
Kdelr2	Kdelr2	0.0301487995578375	6.30855807497684	0.989058849913029	0.219972508624662	0.782036628816827
Ubr1	Ubr1	0.0462050205735025	6.28983492663143	0.988251180415247	0.220170177217158	0.782348612636329
Smad1	Smad1	0.0263973865265592	6.33738596569045	0.988015623404777	0.220227857564814	0.782348612636329
Mblac2	Mblac2	-0.0378682548151177	5.00622951478627	0.987557663319524	0.220340036413952	0.782348612636329
Art3	Art3	-0.145493482711999	0.635256704110031	0.987410300529362	0.220376144415322	0.782348612636329
Man1a2	Man1a2	0.0350889453434823	6.0348924275222	0.987257159154268	0.220413674005902	0.782348612636329
Map3k6	Map3k6	0.0369407228103915	5.09214779795268	0.986772204487366	0.220532557591183	0.782485760333632
Prdm1	Prdm1	0.123393390487462	0.898990889733301	0.986278903428818	0.22065354680317	0.782519258514134
Wdr18	Wdr18	-0.0435940736595451	5.92259219716935	0.986204858270483	0.220671712635791	0.782519258514134
Dnajb2	Dnajb2	-0.033225946066171	6.16152065528021	0.985660861203208	0.220805215431828	0.782621278083744
LOC6873	LOC687399	0.0608003878676259	2.61311435272115	0.985292476978039	0.220895662476522	0.782621278083744
Sema4f	Sema4f	-0.0380022258286796	6.19837196386209	0.985267908495456	0.220901695814413	0.782621278083744
Gpr108	Gpr108	0.0440682487313742	4.69011472995479	0.984825136967721	0.221010453824157	0.782652544798421
Tyms	Tyms	0.0343382435858631	5.081391127818	0.984602130483303	0.221065249203998	0.782652544798421
Prkcsh	Prkcsh	-0.0321402965725112	7.11346276306749	0.983803160573011	0.221261667335522	0.782652544798421
Zfp26	Zfp26	0.0825399753377492	5.26064918253585	0.983701749824775	0.221286608077575	0.782652544798421
Ogdh	Ogdh	-0.0282942586558802	7.75181202884801	0.983181857177727	0.221414492778199	0.782652544798421
Polr2m	Polr2m	-0.0298419313075282	8.25256549419852	0.98315338349019	0.221421524641436	0.782652544798421
Igsf11	Igsf11	-0.0762962318840103	4.28532612355257	0.983100400674005	0.221434564023913	0.782652544798421
Tysnd1	Tysnd1	0.0378414120511837	5.34615685033816	0.982939945169193	0.22147405731755	0.782652544798421
Cul1	Cul1	-0.0216530405309786	7.47664327628971	0.982919602162063	0.221479064848671	0.782652544798421
Otos	Otos	-0.219907372047419	-0.621455258233526	0.981195445682587	0.221903847997196	0.783473028990689
Stard10	Stard10	-0.0654011937743203	3.90809457307903	0.981042073965341	0.22194167024407	0.783473028990689
Riok3	Riok3	-0.0262656469675521	6.73016291401439	0.980738749213955	0.222016488921607	0.783502287885118
Etv6	Etv6	-0.056490067888538	4.45823608446947	0.980176165990088	0.222155317489349	0.783655115734936
Zfx	Zfx	0.0411861011958764	5.48561994796447	0.979975250287211	0.222204918544059	0.783655115734936
Cwc25	Cwc25	-0.0479781407143585	4.31953500858841	0.979646082615	0.222286195566125	0.7837000315881
Lym7	Lym7	-0.0540199624806883	4.01271662290148	0.979135426145405	0.222412348763151	0.783804447717598
Sgsm1	Sgsm1	-0.0642585222385915	6.54460445029496	0.977900243589835	0.222717752568504	0.783804447717598
Arl8b	Arl8b	-0.0289183464533282	7.81342859107338	0.97769815589689	0.222767755866937	0.783804447717598
Akap2	Akap2	-0.0402838988189069	6.25588967587468	0.977417941332391	0.222837107303441	0.783804447717598
Cacng3	Cacng3	-0.0853226042550879	4.27191260468338	0.977288527591412	0.222869143070144	0.783804447717598
Shank3	Shank3	-0.0456056019308003	7.6718385121551	0.977098117202331	0.222916285897795	0.783804447717598
Ctsh	Ctsh	-0.0718637657921774	4.47319594712383	0.976944860316023	0.222954236485168	0.783804447717598
Ndufaf1	Ndufaf1	-0.0587511545206947	6.07635523767146	0.976605843884276	0.223038207670266	0.783804447717598
Ppil2	Ppil2	-0.0323986257760182	5.29101613658847	0.976601870284398	0.223039192064909	0.783804447717598
Nsmce2	Nsmce2	-0.041640177201658	4.48426613521369	0.976549398523105	0.223052191427608	0.783804447717598
Mansc1	Mansc1	0.110373792399649	1.42877185837571	0.976479080432071	0.223069613125364	0.783804447717598
Suv39h2	Suv39h2	0.0572119014732204	3.67601431892707	0.976425153581374	0.22308297464227	0.783804447717598
Nfia	Nfia	0.063506508886246	5.61905728721896	0.975955302421596	0.223199421059857	0.783839037888523
Rassf5	Rassf5	-0.0696181965354559	3.60194910470968	0.97569322487459	0.223264397582284	0.783839037888523
Slc35f6	Slc35f6	-0.036463815429742	4.82624457049524	0.975416198398285	0.22333009913929	0.783839037888523
Alkbh7	Alkbh7	-0.0870405383007482	4.50545806304813	0.975354013055224	0.223348523524263	0.783839037888523
Casp1	Casp1	0.190783720229247	-0.393063272944863	0.974916002201211	0.223457194781919	0.78395015914802
Thbs1	Thbs1	-0.103973183578841	4.34498564768963	0.974396982219147	0.223586026973193	0.784028621765872
Pbx1	Pbx1	0.0435668204968096	8.96455334826679	0.974090269762449	0.22366219161099	0.784028621765872
Klhdc9	Klhdc9	-0.0538684499878714	3.50822385643772	0.973759248240746	0.223744418382819	0.784028621765872
Uba5	Uba5	0.0369379616094287	5.86210053692481	0.973589166259515	0.223786679517062	0.784028621765872
Usp30	Usp30	-0.0232581456702452	6.08209139857725	0.973384337152467	0.223837582912665	0.784028621765872
Psap	Psap	-0.0347506231337222	9.68699929093202	0.973267611574443	0.223866595862892	0.784028621765872
Vat1	Vat1	-0.0360862436968191	8.20023914200156	0.972675092766252	0.224013923473373	0.784233117978133
Tpi1	Tpi1	-0.0474057326635237	8.28432882875275	0.972189487232797	0.224134732512471	0.784284726087045
Pin1	Pin1	-0.0481978472428332	6.51102345115122	0.971997003707244	0.224182636467243	0.784284726087045
Ptpn2	Ptpn2	0.0443791919464667	3.63389144880469	0.971445875223912	0.224319896161298	0.784284726087045
Amn	Amn	-0.12177559163635	1.43998212805014	0.971354619049833	0.224342573052703	0.784284726087045
Supt5h	Supt5h	-0.0284172276263689	7.76371264556	0.971086736974272	0.22440929932637	0.784284726087045
Iars1	Iars1	0.0410691641541152	6.66870823062319	0.970825074163372	0.224474493970714	0.784284726087045
Ece1	Ece1	-0.210844749598773	4.70224394063767	0.970822020319218	0.224475254954022	0.784284726087045
Srd5a3	Srd5a3	0.0582004638519805	3.80397565188002	0.970211574022485	0.224627418621378	0.784500542734639
Zbtb7a	Zbtb7a	0.0481995044641723	4.41295279450028	0.966717447822781	0.225500204571594	0.786426734916892
Prima1	Prima1	-0.188524463936038	1.95929403358976	0.9664129851989	0.225576402208551	0.786426734916892
Ap2a1	Ap2a1	-0.0227982881891967	8.07720570354085	0.966264508299773	0.225613568975208	0.786426734916892
Igbp1b	Igbp1b	-0.114198437618579	1.47854667394987	0.965930052802134	0.225697194611797	0.786473538017204
Zfp418	Zfp418	0.0631156363700791	3.09910735055621	0.965601191534219	0.225779684623644	0.786473538017204
Prx	Prx	0.108016600404693	1.10587231357698	0.965432092825459	0.225822050012898	0.786473538017204
Nmrk1	Nmrk1	0.107437298105867	1.29810782309426	0.964749475434824	0.225993395439527	0.786677760087417
Entpd6	Entpd6	-0.0277531262089798	6.41544511832807	0.964193640315962	0.226132548917974	0.786677760087417
Dpcd	Dpcd	-0.0391414238736531	5.56405098766494	0.964153821220258	0.226142539703078	0.786677760087417
Angptl6	Angptl6	-0.0998925988388612	0.974655424004148	0.964088786125494	0.226158856530593	0.786677760087417
C2cd2l	C2cd2l	-0.0309513684342734	7.03699031581324	0.963676518109416	0.226262316697227	0.786680696992105
Cdh9	Cdh9	-0.0745332270604096	4.00073726393144	0.963397253061142	0.22633243422606	0.786680696992105
Ap3s1	Ap3s1	-0.0300508806125021	5.9029120092195	0.963242701045985	0.226371231610263	0.786680696992105
Megf10	Megf10	0.0906082469585659	3.49536089426267	0.96262400277861	0.226426156658685	0.786680696992105
Mrps21	Mrps21	-0.0537035817448132	4.98701994672067	0.962828306327808	0.226475315288226	0.786680696992105
Ttc5	Ttc5	0.0307719421859063	6.36329982176462	0.96150362299506	0.226808321601322	0.787331058773836
Elk1	Elk1	0.0343228988642952	5.83529842594173	0.961131009692842	0.226902085133591	0.787404887299578
Thsd7a	Thsd7a	0.0741782983655218	5.18809789187979	0.960711125389971	0.227007772760951	0.787503454631699
Grap2	Grap2	-0.158572257934811	0.1102541730261	0.960387346291739	0.227089305467075	0.787503454631699
Tomm22	Tomm22	-0.0434576320416904	6.34270662871647	0.959223946279702	0.227379961472768	0.787503454631699
Dnase1l1	Dnase1l1	0.0678679335564465	2.97921614618203	0.959016695585603	0.22743474500024	0.787503454631699
Tkt	Tkt	0.0265176077037431	8.3223129049576	0.958909287890708	0.227454268195622	0.787503454631699
LOC1003	LOC100365	-0.0382080027227703	4.99340612290242	0.958928300106599	0.227457038675401	0.787503454631699
Txn2	Txn2	-0.0442277292946689	6.04055340213665	0.958543064145971	0.227554224888185	0.787503454631699
Lipg	Lipg	0.0828548268254875	1.93819756329071	0.957928897300164	0.227709242809393	0.787503454631699
Prr5	Prr5	0.0325245029500201	4.93557495105686	0.957763840542946	0.227750920318061	0.787503454631699
Khsrp	Khsrp	0.0395206099076759	6.70234582331486	0.957577504779721	0.227797978212714	0.787503454631699
Itm2b	Itm2b	-0.0361285625532564	8.19826659700916	0.957304709517274	0.227866889774796	0.787503454631699
Ppp1r9a	Ppp1r9a	0.0474984006038022	6.6801899675755	0.957261475796784	0.227877812753821	0.787503454631699
LOC1009	LOC100910	-0.0664948120133749	3.21284964975443	0.956750830081853	0.228006838409843	0.787503454631699
Elf4	Elf4	0.195170919525789	-0.456787220739946	0.956170528169545	0.228153597511891	0.787503454631699
Cacng8	Cacng8	-0.0397817828185891	5.87147522963126	0.955949422656388	0.228209532161533	0.787503454631699
Uba6	Uba6	0.0481852543414053	5.4830477342235	0.95584396874192	0.228236212592643	0.787503454631699
Max	Max	0.0343439599162367	5.03290790217494	0.955783711212902	0.228251456775051	0.787503454631699
Trim26	Trim26	-0.0332082924342886	5.1080594057398	0.955244472237872	0.228387944972344	0.787503454631699
Erlec1	Erlec1	-0.0311782918429689	5.7967002525968	0.95511271608671	0.228421304836837	0.787503454631699
Zfp161	Zfp161	0.0420171051694624	5.13897527447395	0.955081326811418	0.228429250543905	0.787503454631699
Spred2	Spred2	0.0343960206945749	5.25726881509299	0.954963089033065	0.228459194962217	0.787503454631699
Psmb3	Psmb3	-0.0470237092805953	6.42604949829607	0.95483412163943	0.228491855558594	0.787503454631699
Rpusd2	Rpusd2	0.0416720300158119	3.85563392816823	0.954829132185537	0.228493121837855	0.787503454631699
Slco3a1	Slco3a1	0.0493735056561412	4.83769574050728	0.954803892217868	0.228499514814655	0.787503454631699
Slc25a46	Slc25a46	-0.0237680730367749	6.56309597277468	0.954719399493982	0.228520916995912	0.787503454631699
Scube3	Scube3	0.0642599130597244	3.73295364920826	0.954327706432622	0.228620157844244	0.787590032543725
Ednra	Ednra	0.0765094786435702	3.98099582416692	0.953469482572079	0.228837739668269	0.78784339646116
LOC1009	LOC100910	-0.0660989587978085	4.57393756089892	0.953412628449541	0.228852160677224	0.78784339646116
Trpm7	Trpm7	0.073718808390625	6.06753417015433	0.952920503959916	0.228977320628183	0.787991224107859
Casp2	Casp2	0.0725730458913693	5.68179164808849	0.952554610100634	0.229069894642157	0.788062394520189
Nubpl	Nubpl	0.065160547187802	2.57220386432089	0.951334461688765	0.229379854050269	0.788653217786452
Zfp213	Zfp213	0.0337828858314202	4.86306209204905	0.950245301112048	0.229656865889285	0.788979207115684
Fam163b	Fam163b	-0.0832975436284585	5.52766185880086	0.950133061479774	0.22968543506342	0.788979207115684
Add3	Add3	-0.037564805898654	6.81590530623274	0.9498893882048	0.2297474541878	0.788979207115684
Ubxn2a	Ubxn2a	-0.0426525862265381	4.10263075899775	0.949449322192565	0.229859504672989	0.78919598078713
Usp29	Usp29	-0.0373342408318691	8.14745929360494	0.948733738249492	0.230041822895883	0.789382711466839
Zbtb3	Zbtb3	0.066786297470171	2.23011607906952	0.948646390722772	0.230064086376775	0.789382711466839
Mest	Mest	0.0593306621213418	5.77742865731708	0.947754690641617	0.230291480724097	0.78977516385484
Arhgap17	Arhgap17	0.066490968409165	3.67893982764744	0.94720368501985	0.230432098030469	0.789796351942625
Pygb	Pygb	-0.0278357268320996	7.7442637574937	0.947020314205702	0.230478912148863	0.789796351942625
Fgf1	Fgf1	0.202681936418829	-0.5511134912963	0.946975828121259	0.230490242325274	0.789796351942625
Tmod2	Tmod2	0.0413789258066284	7.04045980469409	0.945897273039265	0.230765814434396	0.789845568794764
Slc7a5	Slc7a5	0.0415987458184546	7.97108527355302	0.945869519356037	0.230772908839491	0.789845568794764
Foxb1	Foxb1	0.31012881135973	1.19637588899828	0.945759982110107	0.230798354242316	0.789845568794764
Limk1	Limk1	0.0252939183648028	6.95775522324949	0.945559810828265	0.230852397206211	0.789845568794764
Hs6st3	Hs6st3	0.0545161530361169	5.05971537989783	0.945395069721016	0.23089421911645	0.789845568794764
Gnl3	Gnl3	-0.0491894169342329	5.67601315111541	0.945245184802872	0.230932554948323	0.789845568794764
Drp2	Drp2	0.0657432283805634	4.73940272343088	0.9449556914585	0.231006638010748	0.789871268607136
RGD1560	RGD156002	-0.215319109058849	-0.419021433837826	0.944199827435847	0.231200063209893	0.790146406278565
Slc2a8	Slc2a8	-0.0379289035986805	5.60563382894648	0.944115881403797	0.231221558713303	0.790146406278565
Strada	Strada	-0.0343809723073635	4.98456099422551	0.943770188801057	0.231310127740366	0.790146406278565
Vps13a	Vps13a	0.0762650811126521	5.98617020049119	0.943470751236219	0.231386848791176	0.790146406278565
Usp38	Usp38	0.0364732098149279	5.16600254776707	0.943432459829253	0.2313966613814	0.790146406278565
Kcnn4	Kcnn4	0.204338891643715	-0.510395438362016	0.942113352268564	0.231734923532108	0.790801827604149
Zfyve27	Zfyve27	-0.0310655978444544	7.01665162651547	0.941401113348974	0.231917771470376	0.791086538567505
Gon7	Gon7	-0.0526307506720852	4.26961897242947	0.940710220111626	0.232095258132861	0.791230695237051
Ogfod3	Ogfod3	-0.0463638820949024	3.83056754283727	0.940671980261581	0.232105085433713	0.791230695237051
Zap70	Zap70	0.0685915775170829	2.44501915852811	0.939639073487477	0.232370680004163	0.791712288883913
Ccdc175	Ccdc175	-0.0798439638585849	2.42331929001614	0.939326851618333	0.232451018034297	0.791752501578068
Anapc13	Anapc13	-0.0725947933623923	3.50387769498257	0.938490076686264	0.232666458035531	0.792034106205895
Pex11a	Pex11a	-0.0670854206618809	3.79912514735058	0.938195410471849	0.232742368408348	0.792034106205895
Ago2	Ago2	0.0724948362321319	4.37876941204238	0.938015180547083	0.232788809690131	0.792034106205895
Dll4	Dll4	0.0700463240658725	2.51531726721293	0.937726358067362	0.232863250657231	0.792034106205895
Grk3	Grk3	-0.0637377940662803	4.11185043893415	0.937591894596715	0.232897888282752	0.792034106205895
Rras2	Rras2	0.0382030588871862	6.20769749250064	0.937279052425865	0.232978583508124	0.792034106205895
Trpt1	Trpt1	0.0677708923216918	2.27270329718542	0.93667260303868	0.233135034647	0.792034106205895
Baz2b	Baz2b	0.0817360922198514	5.75229612783661	0.936608623002905	0.233151543284828	0.792034106205895
Pum2	Pum2	0.0319827084781013	8.44180694141637	0.936528365526752	0.233172258319766	0.792034106205895
Trim13	Trim13	-0.0494431070012991	4.21666016760974	0.93640517213192	0.233204057343491	0.792034106205895
Ebag9	Ebag9	-0.0368752634135969	4.55422398783532	0.93557737177683	0.233417829861461	0.792391288225038
Pcdhb12	Pcdhb12	-0.0562162798677236	3.00411921122552	0.933485277823998	0.233958913438543	0.793253329701769
Tigar	Tigar	0.0367760238079098	5.06250647573699	0.933313500056919	0.234003391760851	0.793253329701769
Rnf138	Rnf138	0.0345038372773483	5.94373213811321	0.933260621349021	0.234017085516304	0.793253329701769
Kcnt1	Kcnt1	-0.12491732535388	5.44623527214441	0.933191974901789	0.234034863689436	0.793253329701769
Hadhb	Hadhb	0.0328675140473529	6.06207551729435	0.932038703318526	0.234333729043034	0.793811976645087
Oprl1	Oprl1	0.0308908031536345	5.79446738575215	0.931498884985385	0.234473743407277	0.793893321250494
Stac2	Stac2	-0.0858764505324956	4.11150400967241	0.930166576212557	0.234819644096682	0.794683248886115
Tekt4	Tekt4	-0.166462701262126	0.464744427015051	0.929819428656046	0.234909850824035	0.794726171306863
Borcs7	Borcs7	-0.0346852852644972	5.56348201760908	0.927833019758502	0.235426664730744	0.795556524970745
Tnfrsf21	Tnfrsf21	0.0235912181350106	7.0703887732308	0.927825258774824	0.235428668544694	0.795556524970745
Pofut2	Pofut2	0.036240989672608	4.9843121320662	0.927739285365412	0.235451060180419	0.795556524970745
Rasl10b	Rasl10b	-0.0600030698390791	6.5254224325175	0.927485531436176	0.235517163546765	0.795562072570636
Sesn2	Sesn2	-0.0710352782687999	3.88038769084469	0.926999065351222	0.23564612470636	0.795596650029817
Ubxn8	Ubxn8	-0.042256751193303	3.57986880271294	0.926543850354234	0.235762618500457	0.795596650029817
Fut11	Fut11	0.0420789682788743	4.21729332576033	0.926385447922037	0.235803928875436	0.795596650029817
Zbed3	Zbed3	0.0522377152186602	4.16186952865219	0.926072354607058	0.23588560526705	0.795596650029817
Pigq	Pigq	-0.0268808424968885	6.06372862879676	0.925673727901504	0.235989623219072	0.795596650029817
Sdc3	Sdc3	-0.0325097492588491	10.490216474207	0.925576573526669	0.236014993724619	0.795596650029817
Wnt16	Wnt16	-0.157552346653285	0.443934294326237	0.925252551670837	0.236098599267804	0.795596650029817
Lats2	Lats2	0.0565310849204409	4.25915806834978	0.924598513609283	0.236270442562626	0.795596650029817
Atad3a	Atad3a	-0.03367443481214	6.07630298903829	0.924327987694255	0.236341144200145	0.795596650029817
Ube4b	Ube4b	0.0349809274975087	7.67615189654464	0.924276673731057	0.236354557292018	0.795596650029817
Eif3f	Eif3f	-0.0335710510418697	8.08270447050176	0.9238745557779	0.236459692773717	0.795596650029817
Rps16	Rps16	-0.0548118798374407	9.09838593869973	0.923603700075262	0.236530523985066	0.795596650029817
Wasf1	Wasf1	0.0289136623447899	8.61457997892055	0.923225491918494	0.236629486232361	0.795596650029817
Paqr5	Paqr5	0.21125934720292	-0.028197091779083	0.923181070568219	0.236641110941775	0.795596650029817
Gpn2	Gpn2	-0.0421681625146439	3.966686384781	0.923066516830679	0.236671091213992	0.795596650029817
Ubxn1	Ubxn1	-0.0392265046427707	6.24985877154338	0.92203355678655	0.236679717968952	0.795596650029817
Esd	Esd	-0.0381256626106557	6.01400119693567	0.922696690325751	0.236767904266799	0.795596650029817
Akr1a1	Akr1a1	-0.0432947798409411	9.27414192075671	0.922510063115539	0.236816773636007	0.795596650029817
Mycbp2	Mycbp2	0.0589289418051387	9.20491123106111	0.922074528085358	0.236930854991752	0.795596650029817
Cd47	Cd47	-0.0319876630212398	6.86552904892524	0.922005335349368	0.236948987016867	0.795596650029817
Mtfp1	Mtfp1	0.062627819362164	3.39021890421664	0.921882374379206	0.236980945113747	0.795596650029817
Fgfr1op2	Fgfr1op2	-0.0309603394364277	5.00244023452923	0.921819190567646	0.236997765178966	0.795596650029817
Sumo1	Sumo1	-0.0302138144679589	6.72160210270863	0.921204292116634	0.237158963052191	0.795596650029817
Ttc7b	Ttc7b	-0.0278916486944359	5.85791617204352	0.921104665989104	0.237185090140623	0.795596650029817
Fam20b	Fam20b	0.0245832610726389	6.92305584916149	0.920835127873183	0.237255790469582	0.795596650029817
Trim8	Trim8	0.0253608797910739	7.60775086296811	0.920510348697654	0.23734100704107	0.795596650029817
Myh3	Myh3	-0.0901763085798433	1.67397817314922	0.919907823211233	0.237499176043738	0.795596650029817
Pclo	Pclo	0.105369266693764	7.23889270049043	0.919903098787231	0.237500416645523	0.795596650029817
Cmtm4	Cmtm4	0.0456434353055548	4.79883267489959	0.919865672825698	0.237510244663376	0.795596650029817
Prmt7	Prmt7	0.0288652484389682	5.61913772354534	0.919767344345388	0.237536067456765	0.795596650029817
Jmjd1c	Jmjd1c	0.0824532602127308	7.19972375817702	0.919413623296883	0.237629140387079	0.795596650029817
Slfn4	Slfn4	-0.245088054869204	-0.835784577316107	0.919112161932006	0.237708197574638	0.795596650029817
Mx1	Mx1	-0.170415752157268	-0.379414821972358	0.919053496321965	0.237723618000265	0.795596650029817
Trappc10	Trappc10	-0.0202482740639985	7.42847626941066	0.918808675801558	0.237840711702031	0.795596650029817
Pcdhb5	Pcdhb5	0.0493243774732443	3.77695242407665	0.918579994960503	0.237848095642833	0.795596650029817
Ndst1	Ndst1	0.0359092380008525	6.98848944817799	0.918551066824056	0.237853073032718	0.795596650029817
Pdzd9	Pdzd9	-0.0888755629295882	0.979038267278899	0.918416652148871	0.237891053415244	0.795596650029817
Mmp24os	Mmp24os	0.0758909425521504	2.99847714467567	0.917842767162284	0.238042029286543	0.795726450889709
Zfhx2	Zfhx2	0.11447801877797	5.58639709678165	0.91764674242836	0.238093620421838	0.795726450889709
Hspa4	Hspa4	0.0236789626493181	8.49378918808567	0.917480032873087	0.238137504417507	0.795726450889709
Ubr4	Ubr4	0.0767787735853643	8.31749649712148	0.916926630815752	0.238283234717786	0.795745737112256
Obsl1	Obsl1	0.0432673078391496	4.81905306352057	0.916820219571981	0.238311266219099	0.795745737112256
Ppp1r14d	Ppp1r14d	-0.171972026775744	-0.106287385816658	0.91643556845003	0.238412619121945	0.795745737112256
Cluh	Cluh	-0.0301938672799537	6.73725272254958	0.916076519781186	0.238507263255956	0.795745737112256
Kricap2	Kricap2	-0.042644417736201	5.17366713283361	0.915998511027048	0.238527820767929	0.795745737112256
Fibcd1	Fibcd1	-0.11024119281482	3.65304985476581	0.915996496271717	0.238528361993416	0.795745737112256
Chmp4bl1	Chmp4bl1	-0.0342848098552578	6.08783620380691	0.915602008868632	0.238632397068884	0.795840350015215
Mrpl48	Mrpl48	-0.0446075708633948	5.34124369298951	0.914969276049452	0.238799352427539	0.796047851499575
Pigb	Pigb	0.0998962877841665	1.2744567257871	0.914783882633927	0.238848292002424	0.796047851499575
Otulin	Otulin	0.0352381031967992	4.99068443146352	0.913598181802623	0.239161514289817	0.79663370007407
Cherp	Cherp	0.0366681023468342	6.8668733554359	0.912714140112513	0.239395303645561	0.796986397038567
Ppp2r5c	Ppp2r5c	-0.0282903716644614	7.19705107280766	0.912365622133407	0.239487527738873	0.796986397038567
Cbarp	Cbarp	-0.0324521421465782	8.96035685293929	0.912100142770832	0.239557802458124	0.796986397038567
Wdr3	Wdr3	0.0351824456193245	5.56648093190432	0.912064727986149	0.239567178541077	0.796986397038567
Stk17b	Stk17b	-0.070389009209481	2.6752675099792	0.911076069094918	0.239829067065338	0.797451058944558
Hdac1	Hdac1	0.0387195150543405	5.25857599441271	0.910129814989865	0.240079977230252	0.797889765097697
Pard3b	Pard3b	0.113460977220228	1.84036191492296	0.908320598236642	0.240560406412344	0.798109824521102
Flrt1	Flrt1	0.0628389403629237	5.08684993054967	0.908252153612942	0.240578600019637	0.798109824521102
Golgb1	Golgb1	0.0856516527200585	6.32180365277687	0.908076308881462	0.240625346298602	0.798109824521102
Slc24a2	Slc24a2	-0.0802744123303668	5.65060209700366	0.9078934071227	0.240673978298732	0.798109824521102
Stx1b	Stx1b	-0.0338021003107712	7.42852649413386	0.907832153402445	0.240690267227832	0.798109824521102
Cox6i1	Cox6i1	-0.0515515899165783	8.38238778776572	0.90772418197821	0.240718982140844	0.798109824521102
Mrnip	Mrnip	0.0404389420135588	4.09301876795752	0.907697895223547	0.240726026769488	0.798109824521102
Med25	Med25	-0.0270371748628201	7.25505369204622	0.907549343870237	0.24076548710624	0.798109824521102
Zic4	Zic4	-0.16935164221465	4.42431669874004	0.907479373456073	0.240784366871758	0.798109824521102
Mlh3	Mlh3	0.0424019669423511	4.80989633236603	0.907227802847245	0.240851035698982	0.798109824521102
Vamp3	Vamp3	0.0367331969391809	5.23994652513668	0.907128752033465	0.240877394796261	0.798109824521102
Mir3064	Mir3064	0.16427721125169	1.42627704397965	0.906756761198324	0.240976412364118	0.798191875622345
Wnt11	Wnt11	0.156688694947521	0.502309998502028	0.905737880409944	0.241247819335199	0.798399208471472
Bsn	Bsn	0.0686321673664135	8.00761329744799	0.905685294858031	0.241261834840756	0.798399208471472
Tfap2b	Tfap2b	0.282660037215102	3.13670156465715	0.905616142067018	0.241280267154587	0.798399208471472
Pantr1	Pantr1	0.0688849065579901	4.21962795821769	0.905463564046642	0.241320940769963	0.798399208471472
Agap2	Agap2	-0.0342746557691394	10.0698970288717	0.904868515518743	0.241479628748544	0.798567860393629
Ubqln2	Ubqln2	0.0293003958912211	8.24615200677789	0.904713266684411	0.241521046971259	0.798567860393629
Nckap1	Nckap1	-0.0221580009944839	9.06861470947889	0.904469309609858	0.241586144991593	0.798570418910082
Tmc5	Tmc5	-0.109917010265852	1.23779248541758	0.903799109742536	0.241765068628486	0.798797316796277
Orc6	Orc6	-0.0451767993761326	4.00754272855935	0.903625218521618	0.241811513103314	0.798797316796277
Sik3	Sik3	-0.0359600022081782	6.86642259341678	0.903386591923556	0.241875264221537	0.798797316796277
Dhdds	Dhdds	0.0407612683925946	5.51625754780868	0.902302734080749	0.242165013042334	0.798992472114676
Ndufaf1	Ndufaf1	-0.0453744595080874	3.72487519342216	0.902287383029282	0.24216911926997	0.798992472114676
Stradb	Stradb	0.0244897749205557	6.44462934481557	0.90220243755585	0.242191842396871	0.798992472114676
Qrich1	Qrich1	-0.0360709000573686	6.66774044949032	0.902116110584231	0.242214927162846	0.798992472114676
Fn3krp	Fn3krp	0.0359060912441706	4.82362201335038	0.901114127759463	0.242483148138545	0.799402152380668
Arl3	Arl3	-0.0412797507107349	6.44549240807983	0.900543216263813	0.242636096666884	0.799402152380668
Tmem53	Tmem53	-0.0663337305431582	2.90922442149876	0.900351781820223	0.242684722675792	0.799402152380668
Mir1249	Mir1249	-0.164229669250307	0.234281198936642	0.899950705734597	0.242794929094487	0.799402152380668
Marchf1	Marchf1	0.039983059717411	5.19309239428669	0.89992762801678	0.242801117207282	0.799402152380668
Fem1a	Fem1a	0.0315859657292586	6.05310119111381	0.89983041010418	0.242827188496999	0.799402152380668
Arf4	Arf4	0.0341919946034674	8.29203384317428	0.899786752902521	0.242838896961526	0.799402152380668
Rhd	Rhd	-0.199968921002718	-0.363742658017466	0.899511676624996	0.242912682331521	0.799424801466813
Rabggta	Rabggta	-0.033892031875508	5.74547782398721	0.899217672983909	0.242991568398059	0.799459321013461
Dync2h1	Dync2h1	0.0832931279739826	6.21503112177544	0.898495308576543	0.243185494843477	0.799761938580885
Fkbp1b	Fkbp1b	-0.0604634284125155	4.44216672017503	0.897002323880053	0.243586768209053	0.800404982098869
Nmnat2	Nmnat2	0.0358034967280401	6.3261401060105	0.896991320161071	0.243589721836302	0.800404982098869
Pfdn2	Pfdn2	-0.054815004233429	5.72082881680005	0.896727702802366	0.243660652255625	0.800420750082776
Lrch4	Lrch4	0.0826735413445212	4.35377470188216	0.895867340640777	0.243892251298075	0.800679328610792
Tspan15	Tspan15	0.0544485781578092	3.60113807899083	0.895838500620499	0.243900017794761	0.800679328610792
Slco4a1	Slco4a1	0.0873426167184265	3.7778545768551	0.895346233103325	0.244032629669147	0.800838641862303
Palm2	Palm2	0.0575620418015751	5.36476784756541	0.894649797061433	0.244220356778888	0.801126175892778
Ltc4s	Ltc4s	0.149729416058255	0.305630208578763	0.893964429098201	0.244405241872231	0.801242610755219
Tgif1	Tgif1	-0.0892884542752522	1.30388110155271	0.893759180086032	0.244457935678843	0.801242610755219
Fnip1	Fnip1	-0.0325307766473783	5.6689442784543	0.893750264889936	0.244463041122946	0.801242610755219
Poldip3	Poldip3	0.0262084159677684	7.0883687961712	0.893369877316488	0.24456573401564	0.801266377228206
Tmed4	Tmed4	0.0399541693328151	5.25852577702969	0.893035308437888	0.244656091502622	0.801266377228206
Sbk1	Sbk1	0.0309808082595282	9.86126939421456	0.892896802088728	0.244693507485502	0.801266377228206
Pkn3	Pkn3	0.0262238625398022	7.15037966237759	0.892751229675269	0.244730136488019	0.801266377228206
Rad9b	Rad9b	-0.0848075724966976	1.07451219508069	0.892117501246595	0.244904129572959	0.801376916035426
Otx1	Otx1	0.0699868617190214	3.49728029678194	0.891904091005856	0.244961838644804	0.801376916035426
RGD1566	RGD156636	0.0456933772112367	4.26888271796303	0.891821526201099	0.244984168533553	0.801376916035426
Celsr3	Celsr3	0.0765460786113416	8.41473550735593	0.89163505774016	0.245034607112472	0.801376916035426
Gnl2	Gnl2	-0.0319796426170031	5.78622380378544	0.891236745449658	0.245142381576666	0.801478037396625
Hist1h4b	Hist1h4b	0.0772342120842689	2.64100651316094	0.890506816458442	0.245339975223869	0.801787657655737
Relch	Relch	0.0325546897709225	7.05706980432556	0.890263612659439	0.245405891382264	0.801791475722141
Mgrn1	Mgrn1	-0.0212674946166736	7.83299620568228	0.889421108568413	0.245634230193555	0.80217230099051
Ccar1	Ccar1	0.043965908748374	6.80523725601608	0.888545316681459	0.245871817906049	0.802253532780369
Tmem229b	Tmem229b	-0.0310254344697583	8.22764801553625	0.888497171739694	0.245884885236893	0.802253532780369
Pik3c3	Pik3c3	0.0305207909715055	5.21987499316778	0.888406068395845	0.245909614015922	0.802253532780369
Nedd1	Nedd1	0.0690629501250521	2.51922071709125	0.887891650611301	0.246049290756923	0.802253532780369
Hccs	Hccs	-0.0407882470375858	4.64857960672345	0.887890347917967	0.246049644566308	0.802253532780369
Tnr	Tnr	0.0644620155694878	6.28116330092585	0.887372518479293	0.246190053271133	0.802253532780369
Gnl3l	Gnl3l	-0.0265594017367007	7.39043545611721	0.887359035302978	0.24619398807352	0.802253532780369
Acadl	Acadl	0.0518196880865939	4.53418419423814	0.887254318765031	0.246222447590862	0.802253532780369
Adamts18	Adamts18	0.0669529962958291	4.26905637497946	0.887160606884336	0.24624791809107	0.802253532780369
Timm9	Timm9	-0.0365411398387144	4.66381515611625	0.886701900257968	0.24637262928269	0.802393482282324
Sall1	Sall1	0.0567830225716888	5.14572551012566	0.885861237254318	0.246601343187846	0.802639309003385
Pla2g12a	Pla2g12a	0.0442990064423512	4.5495171513798	0.885749060125043	0.246632150207717	0.802639309003385
Gpr149	Gpr149	-0.0772268348562682	2.95880352875827	0.885002656114301	0.246871734897079	0.802639309003385

Ier5	Ier5	0.0545454858040637	5.61104118160981	0.884995266062887	0.346837158623026	0.802720258932044
Zfp202	Zfp202	0.0583284847970 53	4.63661989972565	0.884905349437616	0.346861654267746	0.802720258932044
Synpo2	Synpo2	-0.165818962059431	2.89947936529186	0.884765698759452	0.34689970678677	0.802720258932044
Nkd1	Nkd1	0.0548655102532509	4.20894186584065	0.884465834588009	0.346981406547568	0.802760431781393
Zmynd15	Zmynd15	0.19197210258 7624	-0.416581089845257	0.883758393291643	0.347174348013742	0.802882271735633
Dpp9	Dpp9	0.0415582759466401	6.22967014421647	0.883755882756298	0.347175005598374	0.802882271735633
Csnk1g1	Csnk1g1	0.0803504366998473	2.91922216395572	0.882564875686019	0.347227116958041	0.802882271735633
Mier1	Mier1	0.0415715419048012	5.91780575 54609	0.88222073223229	0.347321061671627	0.802950692324789
Dok7	Dok7	-0.0876017202779274	1.93491081065533	0.882747755460599	0.347450194275428	0.802989606644719
Pglyrp1	Pglyrp1	-0.151433945448137	0.119025 83694049	0.882581397827309	0.347495828882187	0.802989606644719
Mepe	Mepe	0.123969934805859	0.0654096965932814	0.882452328446409	0.34753096704989	0.802989606644719
Zfand5	Zfand5	-0.0249610628946552	7.58032745673994	0.882211596129903	0.347596654963655	0.80299267996 1688
Bok	Bok	-0.0441826400727184	6.20189256851005	0.881779271888831	0.347714813935312	0.80311694 4367066
Nob1	Nob1	-0.0333218702996782	5.69887370620055	0.881302896353091	0.347845074822108	0.803265109486479
Slc25a24	Slc25a24	0.0528077124271105	3.22909364209505	0.880941587829018	0.347943915774115	0.803348674521397
Rnu2	Rnu2	0.0289272751700113	7.70083891850042	0.880179804313691	0.348152765807692	0.803682157073557
Hcrtr1	Hcrtr1	-0.128656857897487	2.06801023573776	0.879062267475675	0.348458645437817	0.803853258656108
Zcrb1	Zcrb1	-0.0308965566565772	5.29688842571945	0.87882773894264	0.348522953351702	0.803853258656108
Elp5	Elp5	-0.0332648587228428	5.48665512 81725	0.878811579457468	0.348527384903031	0.803853258656108
Scg5	Scg5	-0.0656515359837968	6.40165765 69656	0.878787617022937	0.348533956429568	0.803853258656108
Trim59	Trim59	0.0668476546876777	3.18738528828591	0.878429631293407	0.348632151710064	0.803853258656108
Tdp1	Tdp1	0.0533976030261889	3.43519655290028	0.877779560655604	0.348810561842055	0.803853258656108
Praf2	Praf2	-0.0312386202090256	6.28379392765365	0.877702891201544	0.348831611745248	0.803853258656108
Ucp2	Ucp2	0.072139718861 9647	5.17847824520711	0.877363805775184	0.348924702234632	0.803853258656108
Atm	Atm	0.0704182312663179	5.02793772150153	0.877330393477308	0.348933907048814	0.803853258656108
Sfxn2	Sfxn2	0.138228892117546	2.03367093157911	0.877326525981751	0.348934966607302	0.803853258656108
Lrfn1	Lrfn1	-0.0278498864732614	7.28860108072959	0.876507234722938	0.349160080002257	0.803853258656108
Dzip3l	Dzip3l	-0.0535066615489859	4.47339734822846	0.876108110393062	0.349269858918873	0.803853258656108
Eps15l1	Eps15l1	0.0288038930265907	6.24529617777106	0.876094889143777	0.349276216132701	0.803853258656108
H2az2	H2az2	0.0579516702088699	5.30695736878355	0.875742090979941	0.349370544518097	0.803853258656108
Il17rd	Il17rd	-0.104000807255046	2.19032594802347	0.87557137418564	0.349417520181 79	0.803853258656108
Adam17	Adam17	0.0463640973287742	4.44195615109762	0.875209914342186	0.349517010629621	0.803853258656108
[illegible]	[illegible]	[illegible]	[illegible]	[illegible]	[illegible]	[illegible]

Ifih1	Ifih1	0.094545915930826	1.10865196641537	0.787106967515463	0.374976351026679	0.816881964805164
Tmem222	Tmem222	0.0607367003936053	4.30941306396139	0.786774721059032	0.375077164215263	0.816881964805164
Bdh2	Bdh2	0.105210067821637	1.06243453284657	0.786546447701539	0.375146451002456	0.816881964805164
Traf7	Traf7	0.0261235548738859	6.61454299588671	0.786538160341486	0.375148966765536	0.816881964805164
Kbtbd11	Kbtbd11	0.0543285768778471	5.80314762892913	0.785300874522235	0.375524830883481	0.817534486202847
Tmem168	Tmem168	0.0321274008181034	4.86889856829124	0.785120463199782	0.375579680605492	0.817534486202847
Bace1	Bace1	0.0218836414250757	6.77612447279053	0.784557253827764	0.375750983266557	0.817675727148344
Zscan25	Zscan25	0.0660610824270388	3.49883552172379	0.783966059620113	0.375930915720634	0.817675727148344
Rfc1	Rfc1	0.0297138045208854	5.55085947766088	0.783890591326873	0.375953893525811	0.817675727148344
Dgcr8	Dgcr8	0.0385970289925136	6.18985779064238	0.783292672644009	0.376136011389662	0.817675727148344
Romo1	Romo1	-0.0622055361730534	5.24844025035451	0.783128590151023	0.376186010352782	0.817675727148344
Emc3	Emc3	-0.0308026138884241	6.58637715558715	0.783069020599342	0.376204164605295	0.817675727148344
Ccno	Ccno	-0.194391248703527	0.775819109543983	0.782649593433533	0.376332032293605	0.817675727148344
Myorg	Myorg	-0.0629640798904062	3.17651721887925	0.78257543869884	0.376354634340849	0.817675727148344
Emc1	Emc1	0.0336571990135472	6.57966564853117	0.782418545933083	0.376402481631972	0.817675727148344
Mtrex	Mtrex	0.0243264580520659	6.0641201897715	0.782352409646649	0.376422653523827	0.817675727148344
Ppp2cb	Ppp2cb	-0.0257174473981148	7.76772799850285	0.782060874176992	0.376511591390202	0.817675727148344
Ubl3	Ubl3	-0.0368940458831204	6.94114889411789	0.782018159800245	0.376524624620428	0.817675727148344
Slc43a3	Slc43a3	-0.0760647944408452	2.34144557997063	0.781986455589547	0.376534295730878	0.817675727148344
Dgke	Dgke	0.0362886734125444	5.42338257826443	0.781895507548157	0.376562052432025	0.817675727148344
Gjb2	Gjb2	0.198202872348842	3.67161239762713	0.781506224821939	0.376680878225143	0.817734392219256
Wnt9a	Wnt9a	0.0820167629289705	1.90926723621768	0.781092064043859	0.376807361874939	0.817734392219256
Ube2j1	Ube2j1	-0.0274087113954563	6.23245056131489	0.781006155161336	0.376833598232318	0.817734392219256
Phf11b	Phf11b	-0.16722160235036	-0.176062972554034	0.780734972252105	0.376916453279674	0.817734392219256
Arfgap3	Arfgap3	0.0264985338242557	5.27755677544964	0.780253151123674	0.377063728262753	0.817734392219256
Hmgb2l1	Hmgb2l1	-0.168143184917097	0.148976552522867	0.779963268277928	0.377152373581726	0.817734392219256
Brwd1	Brwd1	-0.0251227930111649	8.54339348852073	0.779822585895424	0.377195404801368	0.817734392219256
Plekhh3	Plekhh3	0.0535980452346736	3.44063399078262	0.779741561868377	0.377220191001784	0.817734392219256
Pdss1	Pdss1	0.0372614506522373	5.77732537052005	0.779694906287375	0.377234464524135	0.817734392219256
Fry	Fry	0.052969589407711	8.38765232940876	0.779384765454383	0.377329360309666	0.817734392219256
Crip1	Crip1	-0.100436064327064	3.10979483773213	0.778879518167784	0.377484042137798	0.817734392219256
Cox18	Cox18	0.0455419403378248	3.96619393628814	0.778798725721707	0.377508784102183	0.817734392219256
Vps33b	Vps33b	-0.0298499321088885	5.06932486125231	0.778760466413438	0.377520501489831	0.817734392219256
Mad2l2	Mad2l2	0.0319474505790528	4.96081454348558	0.778614358442976	0.377565253176436	0.817734392219256
Casp7	Casp7	-0.0486694953737284	3.70730650753389	0.778591813735076	0.377572159459416	0.817734392219256
Gins4	Gins4	-0.0465741504682923	3.24906581111717	0.778376871768728	0.377638010192927	0.817735066650072
Fancl	Fancl	-0.0614328615613201	2.68602741378135	0.777671028294272	0.377854368833761	0.818045657188292
Zw10	Zw10	0.0330730363483701	4.49618454846204	0.777481247020802	0.377912572576205	0.818045657188292
Tbc1d17	Tbc1d17	-0.02732345754586	5.91794569333922	0.776489169315312	0.378217032861938	0.818280774743059
Ppwd1	Ppwd1	0.0485351930014841	3.41035880600639	0.776461379427566	0.378225565290938	0.818280774743059
Lrrc39	Lrrc39	-0.144700575383301	0.488944318174248	0.776277421570719	0.378282058922591	0.818280774743059
RGD1308	RGD13086	0.038523992143844	4.07391118045906	0.776272660272944	0.378283522378614	0.818280774743059
Chrna4	Chrna4	-0.0441671652355983	5.60807786807975	0.775660805958765	0.378471513578717	0.818545514807011
Egf	Egf	0.092956184993164	1.6816332342972	0.775260468836279	0.378594587749538	0.818669797062563
Crabp1	Crabp1	-0.189178937935186	4.49572664243886	0.774948098874319	0.378690657687231	0.818726696987977
Map3k10	Map3k10	-0.0308372047203936	8.28958657800859	0.774032858096622	0.378972032062026	0.818726696987977
Dmkn	Dmkn	-0.157358110471446	0.211902456881135	0.77399531091811	0.378983901723207	0.818726696987977
Stau2	Stau2	-0.0241764568093907	9.26334357463639	0.773937420165886	0.379001729350822	0.818726696987977
Rragb	Rragb	-0.0257431759822582	6.66524670216236	0.773857865093859	0.379026230499155	0.818726696987977
Pex3	Pex3	0.0360146240575193	4.63521787828218	0.773676564913558	0.379082075168649	0.818726696987977
Kcng3	Kcng3	0.106075228661129	1.1594692323231	0.773472292468838	0.379145006735644	0.818726696987977
Ctdnep1	Ctdnep1	0.0239731233904878	6.36814963882134	0.773469543426536	0.379145856578222	0.818726696987977
Ccnb1	Ccnb1	0.05297851351061	4.01454795404743	0.773068201442143	0.37926955009886	0.818813475737371
Lrrn2	Lrrn2	-0.028607713507771	7.65298245518678	0.772422640107592	0.379468631109429	0.818813475737371
Il4r	Il4r	-0.0496326228503725	3.47821976369501	0.772199385122128	0.379537513770153	0.818813475737371
Irag1	Irag1	0.127435663338519	0.92449430158785	0.772144825206141	0.379554350264249	0.818813475737371
Sdhc	Sdhc	-0.0361274887117789	6.98197811032895	0.772071669586055	0.379576926909772	0.818813475737371
Eno1	Eno1	-0.0253884376692334	9.42363946868554	0.771986451067512	0.379603228474759	0.818813475737371
Rexo5	Rexo5	-0.0718673963245766	2.50126487326109	0.771787146257608	0.379664753538134	0.818813475737371
Atp6v0e2	Atp6v0e2	-0.0409806747952121	6.98989679808677	0.771637191874888	0.37971104998128	0.818813475737371
Trappc11	Trappc11	0.0227303872301464	6.4506502826752	0.770789264009514	0.379973004075392	0.819083218287576
Ska3	Ska3	0.0500942766482585	2.82985562020814	0.770509218237429	0.380059567682105	0.819083218287576
Tbc1d2	Tbc1d2	0.0958501423876693	1.55140501719806	0.770481097263897	0.380068262181127	0.819083218287576
Nomo1	Nomo1	0.024127883168792	6.92190472293496	0.770109806578176	0.380183094926594	0.819083218287576
Pold4	Pold4	0.0713972450862309	1.62983080664049	0.769801709216409	0.380279402152666	0.819083218287576
Chat	Chat	-0.285147503417077	0.0397077187900275	0.769667425658071	0.380319956520015	0.819083218287576
Dnajb14	Dnajb14	0.0479405194070834	4.41070474991855	0.76945284915621	0.380386371047356	0.819083218287576
Brms1	Brms1	0.039008262380105	5.00571688575743	0.769289695090798	0.380436880554952	0.819083218287576
Lmbr1	Lmbr1	-0.0246339952593474	6.05662797034459	0.769186690942014	0.380468773631334	0.819083218287576
Rplp1	Rplp1	-0.0534155301306808	8.87169698445002	0.769109555226123	0.380492659563548	0.819083218287576
Srgap3	Srgap3	0.0536542272053174	7.70331297663755	0.767902994970754	0.380866566378782	0.819635507868394
Sox2	Sox2	-0.0567689901767185	6.75798883598376	0.767857870113247	0.380880554189384	0.819635507868394
Grhpr	Grhpr	-0.0524596484966531	4.11583519404708	0.766701207762665	0.381239498665759	0.820138770955507
Macroh2a2	Macroh2a2	0.0231872997016809	7.14269807964239	0.766680651579932	0.381245882146505	0.820138770955507
Ndufab1	Ndufab1	-0.0438521684152488	6.21831579267878	0.765875965888696	0.381495886679754	0.820469427162033
Orc4	Orc4	0.0401473291844557	5.20100048279779	0.765764160055291	0.381530641531948	0.820469427162033
Mcoln1	Mcoln1	-0.0246555855816905	5.74191247082547	0.765481300657406	0.381618598406214	0.820516134234712
Tnfaip2	Tnfaip2	0.110768145881377	1.3922541147752	0.765209335610535	0.381703175055451	0.820556601510486
Atp10a	Atp10a	0.0887194326332447	1.89753888242475	0.764210577682296	0.382014036822233	0.820559557279627
Sorcs1	Sorcs1	0.0438254585801026	5.9171243607689	0.763310664957672	0.382294440272076	0.820559557279627
Sirt6	Sirt6	0.0307442355034781	5.99278996219216	0.763294494923414	0.382299481352098	0.820559557279627
Shpk	Shpk	0.0691749190278884	2.0116505255225	0.763274964148527	0.382305576518772	0.820559557279627
Noct	Noct	-0.0299209607465786	5.44984627628418	0.763186269611744	0.382333223497929	0.820559557279627
Hhex	Hhex	0.114433020159774	1.04341124774201	0.763008414478918	0.382388683772777	0.820559557279627
Cyp1b1	Cyp1b1	0.123634298511843	2.87482757141164	0.762931449081151	0.382412697299608	0.820559557279627
Pqbp1	Pqbp1	-0.0352806622199862	5.87768293285935	0.762639723713733	0.382503688215407	0.820559557279627
Mrpl46	Mrpl46	-0.0368552763751538	4.54591347146771	0.762639056000005	0.382503896637684	0.820559557279627
Dpp10	Dpp10	-0.0307304194285949	6.17916004891733	0.762403034794595	0.382577543768877	0.820559557279627
Zswim2	Zswim2	-0.111747829052445	0.665091415260873	0.762277864966521	0.382616609424164	0.820559557279627
Fam151b	Fam151b	0.039515073129223	4.30814480362263	0.762145042089543	0.382658068787152	0.820559557279627
Tub	Tub	0.0301126028007995	6.50409151898677	0.762096251665774	0.382673301207253	0.820559557279627
Klhl1	Klhl1	0.0559300705720862	4.52375560425934	0.761974655470624	0.38271126491026	0.820559557279627
Socs6	Socs6	-0.0343506357121084	4.53324631523617	0.761929798721766	0.382725271040618	0.820559557279627
Psip1	Psip1	-0.0227103825948781	8.10296541881545	0.761730197980164	0.382787503448067	0.820559557279627
Atxn1	Atxn1	0.0521985213689005	5.75914871515432	0.761339119294149	0.382909773352022	0.820559557279627
Adh1	Adh1	0.217788545896973	0.565671108892067	0.761082395565166	0.382989689821369	0.820559557279627
Galt	Galt	-0.0697405921502896	3.64755538885125	0.761027961492559	0.383007016086805	0.820559557279627
RGD1311	RGD13117	-0.0296631734407285	7.26981865419034	0.760817573943093	0.38307278604013	0.820559557279627
Fbxl22	Fbxl22	-0.068809642196505	3.45855988813491	0.760687498205897	0.383113457460892	0.820559557279627
Ubxn11	Ubxn11	0.0494945161357181	3.81702826527784	0.760415780087527	0.383198436658067	0.820559557279627
Pex5	Pex5	0.0248111931211989	6.87999457164363	0.76035591526553	0.383217162801025	0.820559557279627
Pwp2	Pwp2	0.0248152225946189	5.99729866804671	0.760075066710009	0.383305032173634	0.820570398743069
R3hdm1	R3hdm1	0.0326894260672595	7.25151942850544	0.759276755541515	0.383554015258736	0.820570398743069
Rpl8	Rpl8	-0.0360511563988582	8.45798352540622	0.758942376577949	0.383659705960998	0.820570398743069
Pde4b	Pde4b	-0.0496582049731235	5.72494188403225	0.757962677727614	0.383966850521126	0.820570398743069
Naa20	Naa20	-0.0300086848055389	5.08528556346127	0.757745756403935	0.384034904258841	0.820570398743069
Nmt2	Nmt2	0.0332208614347331	6.15897219972141	0.757679821638042	0.384055593239111	0.820570398743069
Dll3	Dll3	0.0674402870398714	2.8258874906742	0.757024260185354	0.384261380120582	0.820570398743069
Cacng2	Cacng2	-0.0488837390433745	4.81560838865899	0.756649279745091	0.384379160393923	0.820570398743069
Cahm	Cahm	0.0773402827267347	1.32376559502783	0.756638854193675	0.38438243576201	0.820570398743069
Zcchc24	Zcchc24	-0.0319473877121962	5.92259904801111	0.756330501174062	0.384479328147146	0.820570398743069
Plppr3	Plppr3	0.0341485045416341	8.8194886274701	0.75624207654262	0.384507118822632	0.820570398743069
Chac2	Chac2	-0.0523688185875014	3.24925429044906	0.75601905364001	0.384577228127841	0.820570398743069
Itih4	Itih4	0.197108918178026	-0.344218648652221	0.755912101174559	0.384610855584138	0.820570398743069
Cactin	Cactin	0.0278031812544084	4.83201367330375	0.755574731802049	0.384716958941457	0.820570398743069
Cat	Cat	0.0388037442055044	6.62929452565818	0.755345654348602	0.384789024618772	0.820570398743069
Mrpl55	Mrpl55	-0.051206013251169	4.29083855571883	0.755216813053138	0.384829566469132	0.820570398743069
Acvr2a	Acvr2a	0.0532457327820271	6.29084152804972	0.755096278822165	0.384867498872718	0.820570398743069
Rassf7	Rassf7	0.0588371095257943	2.71029206487068	0.75489260891969	0.384931609032453	0.820570398743069
Ppargc1a	Ppargc1a	-0.0575809866640762	2.9373778404475	0.754721261102048	0.384985559293319	0.820570398743069
Slc25a16	Slc25a16	0.0321609841605178	4.68579436472334	0.754613305850015	0.385019549193869	0.820570398743069
Gpm6a	Gpm6a	-0.0292836165221042	10.1090836038735	0.754555814458271	0.385037655105227	0.820570398743069
Cnn3	Cnn3	0.0343436192610606	7.71129922856704	0.75449333030987	0.385057333981632	0.820570398743069
Rae1	Rae1	-0.0252436781407507	5.89617379893352	0.754370771445565	0.38509593705576	0.820570398743069
RGD1308	RGD13087	-0.105509325681044	0.651888981588754	0.754236829399862	0.385138100658741	0.820570398743069
Akirin1	Akirin1	-0.0247372924411296	6.41228301664777	0.754083788003086	0.385186351417101	0.820570398743069
Adcyap1	Adcyap1	0.0693488494929167	4.79033564252361	0.753892525268946	0.385246622501764	0.820570398743069
Slc39a9	Slc39a9	0.0308870087253451	4.9680989661858	0.753685376088384	0.385311921448222	0.820570398743069
Serpinf2	Serpinf2	0.173613776944336	-0.493211505312965	0.753456289942463	0.385384150352528	0.820570398743069
Inafm1	Inafm1	0.0563174593740982	4.99124935005753	0.753388657279814	0.385405478034443	0.820570398743069
Purb	Purb	-0.038705973595049	6.05290696044548	0.75326853666948	0.385443361708194	0.820570398743069
Scmh1	Scmh1	-0.032791279122291	6.16517722777186	0.753100420414881	0.385496391167649	0.820570398743069
Rpl18a	Rpl18a	-0.040914430220762	8.7364949121815	0.753047518261056	0.385513080411765	0.820570398743069
Ydjc	Ydjc	0.0507547844948755	6.15493725996668	0.75267438123106	0.385630824567011	0.820570398743069
Fbxw9	Fbxw9	-0.0332216518672499	5.80250359268009	0.752496189100083	0.385687071508119	0.820570398743069
F11r	F11r	0.0728980036308546	2.72736882639716	0.75218921448311	0.385783996647411	0.820570398743069
Cox14	Cox14	-0.0528424317016703	5.3737751496491	0.751901034535166	0.385875018652797	0.820570398743069
Adss	Adss	0.0221263783911015	6.49730932911897	0.751436457128563	0.386021817541681	0.820570398743069
Uso1	Uso1	0.0211106878022798	6.9174001261341	0.751374658316585	0.38604135452989	0.820570398743069
Ric8a	Ric8a	0.029282569595819	6.80047694891681	0.751190242957179	0.386099654270583	0.820570398743069
Kcna1	Kcna1	-0.0953680604870655	1.95428618548187	0.751084389099319	0.38613312380662	0.820570398743069
Srsf8	Srsf8	-0.0364810504308458	5.42688481921932	0.751010151244742	0.386156598260627	0.820570398743069
Ezh1	Ezh1	0.0324746913260173	5.44053974399488	0.75097840400619	0.386166538975335	0.820570398743069
Ung	Ung	-0.0652059315709297	2.77112342769223	0.750946850858949	0.386208245170348	0.820570398743069
Tsnax	Tsnax	-0.0259925418648384	7.13275458126523	0.750607828149993	0.386283853519525	0.820570398743069
Lsm11	Lsm11	-0.0464959984662947	5.30286575328747	0.750574547636987	0.386294388654319	0.820570398743069
Slc15a2	Slc15a2	-0.0403618197693299	4.97950798461195	0.750366579549878	0.386360194236428	0.820570398743069
Ipp	Ipp	0.0403807990166406	4.28190643009152	0.750301474791407	0.38638079885603	0.820570398743069
Errfi1	Errfi1	0.0382801259268025	4.78871696105442	0.750006598597196	0.386474141624472	0.820570398743069
Gabrq	Gabrq	-0.102091931500548	1.78705459544704	0.749951929312845	0.386488294443081	0.820570398743069
LOC1009	LOC100911	-0.0524673905820557	4.45230326340618	0.749891582960462	0.386510558098645	0.820570398743069
Spred3	Spred3	0.0510967430858671	4.18249982129899	0.749569882924888	0.386612442551432	0.820647064599219
Tmem45a	Tmem45a	0.146096987500897	0.0525520174250715	0.749308187390966	0.386695351158067	0.820683431939827
Smad6	Smad6	0.118884912198897	1.10874234064182	0.748031695649025	0.387100127138953	0.821402769781074
Blnk	Blnk	0.123094656223038	0.8344657813119	0.747139569755376	0.387383373765989	0.821864028987968
Lrrc1	Lrrc1	0.0579587205620925	3.01687913678865	0.746891897509968	0.38746206206001	0.821891218687989
Mrps7	Mrps7	-0.0443179963435063	4.16330564150314	0.746421715186322	0.387611507061324	0.821977179495789
Ksr1	Ksr1	0.0434651640596614	5.85279204818852	0.746349885071865	0.387634345127292	0.821977179495789
Fnd1	Fnd1	-0.0351194717417157	4.96055984276032	0.745213550986435	0.387998893027759	0.82257139414736
Lipa	Lipa	0.035456199372702	5.20156831258851	0.744872156053262	0.388104806789994	0.82257139414736
Acbd4	Acbd4	-0.0526988505155956	3.78120058613935	0.744716522986023	0.388154180715405	0.82257139414736
Tbc1d23	Tbc1d23	-0.0233280090681656	6.45920757196528	0.744625321234892	0.38818323936786	0.82257139414736
Tmem248	Tmem248	-0.0374300198777468	4.79277014051548	0.74389784680092	0.388415112629961	0.82257139414736
Abr	Abr	0.0213287823738877	9.17074279700971	0.743821624845687	0.388439418856201	0.82257139414736
Suv39h1	Suv39h1	-0.0292498879900107	4.81915385080611	0.743445714763467	0.388559323634023	0.82257139414736
Fez2	Fez2	-0.0400149388598794	4.71303371273848	0.743370797418727	0.388583226494405	0.82257139414736
Chchd3	Chchd3	0.0332791335563541	5.69354833050274	0.743312645264702	0.388601781753105	0.82257139414736
Ash2l	Ash2l	0.0265382566231475	6.01419151638673	0.743196207843532	0.388638938547831	0.82257139414736
Ppp1r10	Ppp1r10	-0.0288906085712862	6.61664308876236	0.743193613017951	0.388639766850532	0.82257139414736
Zcchc10	Zcchc10	0.0631372837856213	2.49198111284864	0.742644655321648	0.388815015568126	0.822776648525059
RT1-S3	RT1-S3	0.0747209775784226	2.21985524388117	0.742476780190898	0.388868630459888	0.822776648525059
Bckdha	Bckdha	-0.0401119319311743	5.09042260881018	0.741652589797219	0.389132008439291	0.822816945945798
Rapgef2	Rapgef2	0.0322562363725249	6.99128830442975	0.741465996730803	0.389198067198745	0.822816945945798
Srsf11	Srsf11	0.040447088498381	7.1434408041963	0.741352808899265	0.389227868535748	0.822816945945798
Senp8	Senp8	0.082729790300954	1.27406259091093	0.74118717152767	0.389280849962833	0.822816945945798
Fdx1	Fdx1	0.0579731365685628	3.14442158677978	0.740855064344508	0.389387108138622	0.822816945945798
Gtf2h5	Gtf2h5	-0.0431732854468052	6.23275205081892	0.740594866984011	0.389470387633225	0.822816945945798
RGD1565	RGD15657	-0.0403160218392447	4.21868990228051	0.740233110884039	0.389586214620436	0.822816945945798
Stk38	Stk38	0.0481732633048254	5.83595986792988	0.740001295988809	0.389660462960335	0.822816945945798
Mllt11	Mllt11	-0.0262782197175217	9.80526587008052	0.7392630215368	0.389897060900219	0.822816945945798
Tmed1	Tmed1	-0.0388522292245472	4.29747607483089	0.739250172754652	0.389901180422885	0.822816945945798
Map1s	Map1s	0.020940952369191	6.71637607543648	0.739099821236921	0.389949308917133	0.822816945945798
Ap3b1	Ap3b1	0.0432239303824519	4.89687556627469	0.739060475044525	0.389962007754044	0.822816945945798
Tmem255a	Tmem255a	-0.0804205800839748	3.62140328197557	0.73904283444314	0.389967564973519	0.822816945945798
Marcksl1	Marcksl1	0.0432579094149603	9.39725421102664	0.739001600197014	0.389980888970514	0.822816945945798
Ola1	Ola1	-0.0275182208199607	7.11674859648575	0.73894309700642	0.389999652291892	0.822816945945798
Tppp	Tppp	-0.0543082458035591	3.22093717070286	0.738924104028804	0.390005744055219	0.822816945945798
Dmxl1	Dmxl1	0.0577660802703436	6.04758389706181	0.738914671096808	0.390008769602417	0.822816945945798
Kif15	Kif15	0.0698806481251197	3.63197741942809	0.738099387015069	0.390270392237742	0.822841811653544
Mknk2	Mknk2	0.0250203159633586	7.04926254043884	0.737965363255949	0.390345537562741	0.822841811653544
Sephs2	Sephs2	-0.0333891205327061	6.04941883924107	0.737790558593449	0.390369561161584	0.822841811653544
Dnmt3b	Dnmt3b	0.0840500291485714	2.49782933826117	0.73758082725945	0.390436929116561	0.822841811653544
Iqcb1	Iqcb1	0.0368935959484042	4.99165500414311	0.737556341333587	0.390444795306121	0.822841811653544
Zfp395	Zfp395	0.0393746163408584	3.94098563477275	0.737403544854626	0.390493888823717	0.822841811653544
Zfp865	Zfp865	0.0386155149435103	3.81738200472848	0.736950595827523	0.390639465433558	0.822841811653544
Slc37a2	Slc37a2	0.112548810085887	0.422327545724812	0.73690867156599	0.390653598900032	0.822841811653544
Pink1	Pink1	-0.0321235197766928	6.27894375448672	0.736780429244547	0.390694177348854	0.822841811653544
LOC690Z	LOC690276	0.110215019422928	1.48274471375514	0.736663858150038	0.390731663590079	0.822841811653544
Dnajb3	Dnajb3	0.0670389548792216	2.26147325708832	0.73659439853359	0.390754002450781	0.822841811653544
Cbr3	Cbr3	-0.0716229491320645	2.24450736371996	0.73641427606924	0.390811940038383	0.822841811653544
Fus	Fus	-0.0316121966797637	8.62214580621509	0.735981419507759	0.390951221551328	0.822996185472569
Sf3b4	Sf3b4	0.0232176821051102	6.38136722665881	0.735692528943368	0.391044218278358	0.823037526002386
Tctex1d1	Tctex1d1	-0.159115064054383	-0.558990809084524	0.735510485976917	0.391102825983662	0.823037526002386
Rab40c	Rab40c	0.0418522684808554	4.52468668225941	0.734853035097028	0.391311416766646	0.823182870303976
Slc9a1	Slc9a1	-0.0391794755825847	6.04874729145788	0.734694379479379	0.391365776513813	0.823182870303976
Top2a	Top2a	0.0653698590048908	5.55287448577877	0.734323437236014	0.391485373779085	0.823182870303976
Avpi1	Avpi1	-0.0688658092701575	3.00678515153481	0.734199876432536	0.391525223241667	0.823182870303976
Inava	Inava	0.0825209719014966	1.730322285385	0.734139322938901	0.391544754407897	0.823182870303976
Dmd	Dmd	0.0468032308033883	5.91984850809116	0.733798904299817	0.391654580401637	0.823182870303976
Tbxa2r	Tbxa2r	0.0895412229717265	1.08736170779719	0.733491646116782	0.391753746087054	0.823182870303976
Smarca2	Smarca2	0.0285988033201016	8.09934526528918	0.73346381098839	0.391762723481856	0.823182870303976
Bub3	Bub3	0.0358351895994288	5.50488176800997	0.733454802713915	0.391765583485855	0.823182870303976
Zfp219	Zfp219	-0.0424271247851203	5.46430818309271	0.732652725350469	0.392024363024324	0.823587805599343
Ebp	Ebp	0.056103756212007	3.01916539397046	0.732434346632296	0.392095258281761	0.82358807029992
Chit1	Chit1	0.137464382481066	-0.311009186131327	0.732144568358656	0.392188923453671	0.823656170716748
Ppp4r3a	Ppp4r3a	0.0350280025120658	6.24730154312278	0.731605846988103	0.392363165721804	0.823680676669469
Shmt2	Shmt2	0.0367651024390256	5.80044850275789	0.731573821957917	0.392373494401544	0.823680676669469
Mtmr2	Mtmr2	0.0215449198688361	6.25303940628082	0.730865550389598	0.392602789405986	0.823680676669469
Tox	Tox	-0.0552547339569581	5.36366418850763	0.730803266305642	0.392622942483564	0.823680676669469
Osmr	Osmr	0.117585780683891	0.462312637444123	0.730731808701752	0.392646088362291	0.823680676669469
Thumpd2	Thumpd2	0.0661667779506431	3.6353487460823	0.730253250318711	0.39280111252702	0.823680676669469
Ccdc80	Ccdc80	0.0861669915412899	4.19181170222142	0.730197493401135	0.392819180687465	0.823680676669469
Urod	Urod	-0.0494262631481817	4.29631692998579	0.729981634689485	0.392889141474079	0.823680676669469
Anxa4	Anxa4	0.0991975483011297	2.08761884510183	0.72980960807384	0.392944908558242	0.823680676669469
Dnajc9	Dnajc9	-0.0322229116086883	5.38516049889184	0.729717482741329	0.392974778654738	0.823680676669469
Anapc4	Anapc4	-0.0313815120360388	6.41529571344195	0.729715677017083	0.392975364156824	0.823680676669469
Ccdc71	Ccdc71	-0.0289296601387097	5.03059432060217	0.72966192965167	0.392992792194706	0.823680676669469
Znhit1	Znhit1	-0.0433302006761375	5.53355392277538	0.729326594325929	0.393098008630407	0.823763452600199
Spg11	Spg11	0.0406721336968436	5.6503724783433	0.729113110227615	0.393170815105709	0.823777037797885
Zfp37	Zfp37	0.0390719288289749	5.05963629505876	0.728207653214575	0.393464775464647	0.824254523826796
Siae	Siae	-0.0390960034307915	3.96510817076212	0.727897855141329	0.393565425217056	0.824308210967081
Pomgnt1	Pomgnt1	-0.0265977061441126	6.74992395173752	0.727722134826827	0.393622531132634	0.824308210967081
Klf3	Klf3	0.0356681376774506	5.26745508258689	0.727492711698879	0.393697107420096	0.82432802941442
Hbs1l	Hbs1l	-0.0271226183189267	6.47682537119269	0.727259191256806	0.393773036444615	0.824346573779061
Slc9a3	Slc9a3	-0.0481589441375305	5.80637554967266	0.726397730408124	0.394053322108662	0.824795051157485
Gtpbp4	Gtpbp4	0.0411293788076544	4.11870957694515	0.725762717257169	0.39426011432182	0.825089474403447
Sptssb	Sptssb	-0.100978418211487	1.48634595670442	0.725522729502947	0.394338307383865	0.825114717620133
Sart3	Sart3	0.0260524021510218	6.05806594163968	0.724523126563525	0.394664237581882	0.825658231440165
Lca5l	Lca5l	0.177351050376828	-0.200024725363945	0.724167478882848	0.394780293584172	0.825762568847358
Bex3	Bex3	-0.0415620793118946	7.54313225503324	0.723117647483562	0.395123164498885	0.826178137776709
Prrt2	Prrt2	-0.0304981623106317	7.15408752594533	0.722926294001166	0.395218095463621	0.826178137776709
Agtr1a	Agtr1a	0.0961857691227797	1.58139642325078	0.722517726435559	0.395319288969262	0.826178137776709
Lrrc20	Lrrc20	0.0421746105620493	4.31483175511137	0.722349016273753	0.395374795664055	0.826178137776709
Dvl2	Dvl2	0.0443708166187805	4.77225461885502	0.722294890745107	0.395392140879008	0.826178137776709
Smarca5	Smarca5	0.0333689632924684	6.36058837138089	0.722181310377067	0.395429330632108	0.826178137776709
Grb10	Grb10	0.0385946707381923	6.22543716755543	0.722141115647943	0.395442481269737	0.826178137776709
Acta2	Acta2	0.160091193866494	3.13649425532667	0.721235768075006	0.395738847226073	0.82665889784693
Gamt	Gamt	-0.0361244348372057	4.45073032449816	0.720752650453016	0.395896711061068	0.82685022830749
Mob3c	Mob3c	0.110924615434617	0.292781892403748	0.720021871396613	0.3961367351203	0.827084458241411
Ptp4a1	Ptp4a1	-0.0262032917788508	6.99828570568289	0.720007522344258	0.396141438089647	0.827084458241411
Isca1	Isca1	-0.0335904314785538	7.0149399193638	0.719589510046831	0.396278596303701	0.827086010122226
Otog	Otog	-0.16205349234967	-0.34864640309077	0.719456215781733	0.396322338165333	0.827086010122226
Fancc	Fancc	-0.0377682166775252	4.81532520292321	0.719137053661775	0.396427117133305	0.827086010122226
Herpud1	Herpud1	0.0379080795453138	5.27176936344924	0.71897583760618	0.39648005857248	0.827086010122226
Micalcl	Micalcl	-0.136664411250699	-0.039159607288088	0.718958749149778	0.396485670816803	0.827086010122226
Npc2	Npc2	0.04077012505807	5.27851046502809	0.718793603286045	0.396539914449674	0.827086010122226
Fbxo17	Fbxo17	0.120312717720029	0.551027832537369	0.71851707621164	0.396630766213047	0.8271372238859
Rcc1l	Rcc1l	0.0405620232275946	4.62342320653133	0.718072827379502	0.396776781866536	0.827215107007325
RGD1308	RGD130814	0.0465647589149636	3.70002696571279	0.717767149611461	0.396877305257104	0.827215107007325
Pde4d	Pde4d	0.0330135743965078	5.74157319824934	0.717738727449984	0.396886655088469	0.827215107007325
Jam3	Jam3	-0.0373982577905801	4.433128815717	0.71742380961124	0.396990248588237	0.827215107007325
Cdc73	Cdc73	0.0489938049202298	3.76700362204002	0.717395376418672	0.396999604132393	0.827215107007325
Ptprn2	Ptprn2	-0.0266651817433237	7.93620192710515	0.715985022494408	0.397464060831709	0.827679072299433
Dr1	Dr1	0.0228557008317988	6.29987904079833	0.715916957094124	0.397485634329408	0.827679072299433
Cept1	Cept1	-0.0295932937966203	4.93610058637066	0.715653381213339	0.397570093432559	0.827679072299433
Tmem170b	Tmem170b	0.0517987904448945	4.32788733592164	0.715520726534123	0.397617134210292	0.827679072299433
Dusp7	Dusp7	0.0253858847749625	6.38436425817732	0.714753364909939	0.397870311935514	0.827679072299433
Lgr4	Lgr4	-0.0369018526559514	5.38032970185176	0.714732079402495	0.397877338043058	0.827679072299433
Hspe1	Hspe1	-0.0486564983092002	6.45174587488032	0.714582868745993	0.397926599846257	0.827679072299433
Rcc1	Rcc1	0.0396522205853648	4.12966238597282	0.714026016346558	0.398110503269834	0.827679072299433
Gstm6	Gstm6	-0.110270499421008	1.3853730120048	0.713682161724248	0.398223796418661	0.827679072299433
Mok	Mok	-0.0566185493832624	3.18289984156242	0.713341868455197	0.398336518958432	0.827679072299433
Mylip	Mylip	-0.0530760055302259	3.90088485678813	0.713297381444988	0.398351329726341	0.827679072299433
Aasdhppt	Aasdhppt	-0.0280174250225796	5.48118879664404	0.713231816087536	0.398373010547393	0.827679072299433
Fam111a	Fam111a	-0.249610378666789	4.92202911582568	0.712672547759013	0.398558015989995	0.827679072299433
Lancl3	Lancl3	0.0575782612967888	3.54049493601832	0.712547958465553	0.398599246615139	0.827679072299433
Zfr	Zfr	-0.0278106338502865	8.47011985226139	0.712491807623074	0.39861783124244	0.827679072299433
Lrrtm2	Lrrtm2	0.0261981068138533	6.18861623825165	0.712140290981743	0.398734201536474	0.827679072299433
Npas2	Npas2	0.0751078853020381	5.47168237808083	0.711871158809917	0.398823323119427	0.827679072299433
Kdm4b	Kdm4b	0.0276162280635576	7.43951993171177	0.711806598051952	0.398844722524469	0.827679072299433
LOC500	LOC500124	-0.0887083055099751	1.93405084910413	0.71175855326581	0.398860632287935	0.827679072299433
Slc6a17	Slc6a17	-0.0265975248384123	8.13495770770042	0.711753670913673	0.398862249688858	0.827679072299433
Polr2l	Polr2l	-0.0802121782991808	4.96362618036762	0.711716940189532	0.39887441796183	0.827679072299433
Crebrf	Crebrf	0.0604571010487973	4.50897283831478	0.711571227361958	0.398922695473859	0.827679072299433
Smc2	Smc2	0.0399786569058055	5.32429151895947	0.711530137640196	0.398936310832297	0.827679072299433
Slc9a7	Slc9a7	-0.0508227925902127	5.24658343896006	0.711457685348168	0.398957006112187	0.827679072299433
Poldip2	Poldip2	-0.0229122000090842	6.2959469882282	0.711401863003914	0.398978818823815	0.827679072299433
Kcnb2	Kcnb2	0.0590339697150619	3.46441923212655	0.711363961153182	0.398991381404172	0.827679072299433
Zfp287	Zfp287	0.0344554262425983	5.08980324160857	0.710990991309643	0.399115022990011	0.827679072299433
Snx16	Snx16	-0.0322451998684369	6.09011056493148	0.710911229471307	0.399141471686966	0.827679072299433
Rassf3	Rassf3	0.0628817469280018	4.52111486673413	0.710609461712636	0.399241558607004	0.827679072299433
Gm2a	Gm2a	0.0302418649514207	6.08922858021122	0.710540490547608	0.399264440516214	0.827679072299433
LOC1009	LOC100919	0.103208284364252	0.762786012245679	0.710497511064162	0.399278698749314	0.827679072299433
Slc43a2	Slc43a2	0.0348882230701584	5.69072346988794	0.709786501423203	0.399514697356185	0.828030710782611
Naa40	Naa40	0.0338226934982639	5.13114400322564	0.708842217105641	0.399828435881173	0.828543230757249
Plpp1	Plpp1	-0.0390938808554989	4.35645890632775	0.708410011690916	0.399972155214278	0.828703517412197
Ncl	Ncl	-0.0239362970994572	8.81516284978648	0.707900462767178	0.400141698691147	0.828805575978612
Gtse1	Gtse1	0.0744267497528793	2.86424436079998	0.707095008763801	0.400409888676581	0.828805575978612
Abhd4	Abhd4	-0.0465561059025646	6.51180706374249	0.706923323741258	0.400467098508295	0.828805575978612
Map3k4	Map3k4	0.0265752282553388	5.56034953970437	0.706904964258909	0.400473214127677	0.828805575978612
Xpo6	Xpo6	0.0212378763765892	7.40229349328913	0.706894990380695	0.400476530715391	0.828805575978612
Gtpbp1	Gtpbp1	0.0200866555845743	6.46230392120415	0.706867309287105	0.400485755023484	0.828805575978612
Gde1	Gde1	-0.0370010782987477	7.43418074450498	0.706672081805262	0.400550818822198	0.828805575978612
Cnot10	Cnot10	0.0287300290777006	5.92196476018508	0.706406695564326	0.400639288995225	0.828805575978612
Lrrfip1	Lrrfip1	-0.0259460563493607	5.12758721399905	0.706312808984912	0.400670594199263	0.828805575978612
Grk4	Grk4	-0.0609786241092155	2.59051065525864	0.706267568186753	0.400685690396168	0.828805575978612
Rpl36al	Rpl36al	-0.0465512098745633	5.85214836138522	0.705981093583404	0.400781228628726	0.828865800016843
B3gnt2	B3gnt2	0.0595331773979059	3.04058696314566	0.705469951080546	0.400951936763221	0.828979984445782
Zdhhc12	Zdhhc12	-0.0436871921261607	3.60100208634907	0.70541744377028	0.40096932003978	0.828979984445782
Nexn	Nexn	-0.219336764367942	2.79666172965461	0.704863944600234	0.40115414890957	0.829224704430865
Tax1bp3	Tax1bp3	0.0422678699334433	5.05674405644429	0.70445894397611	0.401289167576158	0.829366397854518
Sgo2	Sgo2	0.11810502725894	0.473500357899813	0.703789602801116	0.401513255087005	0.829692097336959
Hsd17b4	Hsd17b4	0.0261866738723625	6.15276610510476	0.703469407872478	0.40162070740632	0.829776712416576
Ddx18	Ddx18	-0.0366191414570538	3.82757445741771	0.703136994049872	0.401731622155427	0.829869451926037
Bambi	Bambi	0.0864086176026785	1.21781432307722	0.702868228384716	0.401821603049577	0.829916924557122
Mphosph6	Mphosph6	-0.0469034760652937	3.57602945686682	0.702242625886306	0.402031163965884	0.830212318232429
Peli2	Peli2	0.0501606074596672	4.74772231440546	0.70163589164288	0.402234556232366	0.830457400997542
Oaf	Oaf	-0.0669208053147412	4.15710971609448	0.701477113829843	0.402287807295756	0.830457400997542
Cdc123	Cdc123	-0.0276998018809176	6.11987389255597	0.700123891715258	0.402742067968815	0.831265437840204
Sec16b	Sec16b	-0.0882442149484927	0.939077583087527	0.699928759801438	0.402807623019305	0.831265437840204
Ccdc50	Ccdc50	0.0433758970395912	5.28836036052761	0.699238597229794	0.403039654925611	0.831606709857259
Actl6b	Actl6b	-0.0242635807283532	7.15474868888243	0.698730858363955	0.403210472748304	0.831821605238193
Ddt	Ddt	-0.0390085507346963	4.64128104094822	0.698403183522402	0.403320768641168	0.831863307654673
Otof	Otof	-0.0988692775946143	3.77232278121221	0.697822523537646	0.403516323205095	0.831863307654673
Htr6	Htr6	-0.124655443188192	1.27411829593086	0.697709681735205	0.403554346140856	0.831863307654673
Naa10	Naa10	0.0306925251948616	4.68394282199259	0.697585761272336	0.403596104432727	0.831863307654673
Rnpep	Rnpep	0.0205971448704759	6.81494503467838	0.697346041052064	0.403676902402896	0.831863307654673
Map3k11	Map3k11	0.0425037496785815	4.42553991152418	0.697228743789488	0.403716446166708	0.831863307654673
Ppp1r7	Ppp1r7	-0.022268274197985	6.62980060490936	0.69710444353958	0.403758356969687	0.831863307654673
Ell	Ell	0.0429031439683632	3.92116256318801	0.696913010040625	0.403822915758652	0.831863307654673
Elfn1	Elfn1	-0.0533635850746508	5.4408612090533	0.696898322206555	0.403830732905695	0.831863307654673
Cox6a1	Cox6a1	-0.0574850010744475	8.09344131567153	0.696594566520034	0.403930340583689	0.83193114220249
Efna1	Efna1	0.0705032103894539	2.24757609790299	0.696247475422247	0.404047476939119	0.832035050300279
Nipa1	Nipa1	0.0259432212605213	5.83588370395388	0.695945659807073	0.40414837398185	0.832107547718077
Mrpl36	Mrpl36	0.0489265211174091	4.74130812030219	0.69568882627779	0.404236113944629	0.832148819285124
Phka2	Phka2	0.0817145636108981	2.01900508821333	0.695033607576328	0.404457725858887	0.832467574949287
Prokr2	Prokr2	0.0588180949065226	3.1345605318915	0.694692370979162	0.404572902488293	0.832469886191233

Txndc15	Txndc15	-0.0307601290663522	6.5761330588218	0.693551495432743	0.404859006087756	0.822469886191233
Kitlg	Kitlg	-0.0824679589800871	7.74680774353574	0.693364190034586	0.405022446773881	0.822469886191233
C1qtnf2	C1qtnf2	-0.0810100692042346	2.38327414425283	0.693340390824304	0.405030508649685	0.822469886191233
Czib	Czib	-0.0330317031045098	5.02586476853222	0.693080059027878	0.405118710179216	0.822469886191233
Foxq1	Foxq1	0.0632680665223653	3.55391999017884	0.692991474535278	0.405148729347047	0.822469886191233
Dync1li2	Dync1li2	-0.0279482425988527	8.23675638772031	0.6929513626181	0.405162323096091	0.822469886191233
Tmem259	Tmem259	-0.0252379121403908	7.13277720555855	0.692832927980085	0.405202465191002	0.822469886191233
Slc7a6	Slc7a6	-0.0382947823025851	5.71256264282084	0.692564196428891	0.405293568517896	0.822469886191233
RGD1565	RGD156546	0.0479559257454709	3.35178665220507	0.692424850110072	0.40534082194688	0.822469886191233
Klhl15	Klhl15	0.0717403873243237	2.21649726894387	0.692416365888559	0.405343699206388	0.822469886191233
Pik3r2	Pik3r2	-0.0253680845847509	8.20955992001453	0.692344425060164	0.405368094402295	0.822469886191233
Papolg	Papolg	0.0513050625042534	5.20763729064751	0.691661479725227	0.405599824198279	0.822469886191233
Xpr1	Xpr1	0.0233029219534874	7.25520376514026	0.691576528794727	0.405628661963631	0.822469886191233
Faf2	Faf2	0.0277136667092196	4.98596150402713	0.691457692365724	0.405669007654952	0.822469886191233
Nab1	Nab1	0.0319731187277303	5.3748688594987	0.691456244386032	0.405669499289037	0.822469886191233
Elp1	Elp1	0.0305866601954931	7.54683577420321	0.691408066740593	0.405685857592696	0.822469886191233
Zmiz2	Zmiz2	0.0265001561138025	8.84222540132558	0.691366964583345	0.405699814237448	0.822469886191233
Esco1	Esco1	0.0345146302223466	4.52178980776973	0.691288419441683	0.405726486970336	0.822469886191233
Prim2	Prim2	-0.0601565200505764	3.14377696001322	0.690224863063847	0.406087906986366	0.822859801406807
Sft2d1	Sft2d1	0.0356855642086462	5.10136202486687	0.690199800551511	0.406096429458872	0.822859801406807
Acad9	Acad9	-0.023491253907498	6.13649802761839	0.690139965298407	0.406116777411158	0.822859801406807
Lrtomt	Lrtomt	-0.0594158679443606	1.96118040856775	0.689830058759688	0.406220148799367	0.822934888507758
Pex11b	Pex11b	-0.0379415390611223	5.1438304458576	0.689410611534768	0.406364925938536	0.82309483748523
Bpgm	Bpgm	-0.035320688153514	6.45689228869245	0.689205898940355	0.406434687482323	0.82310309685751
Nat10	Nat10	0.0351909917289434	5.23182401852987	0.688665874948988	0.406618537100586	0.823233340061443
Nkx6-1	Nkx6-1	-0.277417159045545	-0.128344185949233	0.688623826550676	0.40663282901612	0.823233340061443
Ncbp3	Ncbp3	0.0279447877967119	4.9689227785001	0.688082685346465	0.40681729708807	0.823272780386817
Dhx32	Dhx32	-0.0296532460568879	5.8208705003728	0.687979207185253	0.406852918871073	0.823272780386817
Trmt10c	Trmt10c	0.0341674830041916	4.50840297566972	0.687820465968613	0.406906711830581	0.823272780386817
Fam172a	Fam172a	0.0261016154564239	6.60260855274121	0.687783804402052	0.406919215428187	0.823272780386817
Smarcd2	Smarcd2	0.0436508370168046	4.21230275798842	0.687046248236996	0.407170881923742	0.823408508211471
Gid8	Gid8	-0.0237222946885738	5.67399326266117	0.686785855948251	0.407259798564161	0.823408508211471
Lgals9	Lgals9	0.0763109601795423	2.9294974150697	0.686500898353557	0.407357111017554	0.823408508211471
Havcr2	Havcr2	-0.152192576231147	-0.210953826136993	0.68634229675613	0.407411296635237	0.823408508211471
Mapkapk	Mapkapk2	0.0365496014806201	5.87716853658302	0.685875547377336	0.407570812929062	0.823408508211471
Smpd5	Smpd5	0.0736404369655784	3.1578248040981	0.68573297520372	0.40761855727471	0.823408508211471
Mcm10	Mcm10	0.0704811826290527	2.42741283190702	0.685601323372438	0.407664575624382	0.823408508211471
Vof16	Vof16	0.170770595088859	4.81623987005038	0.685409396809348	0.407730217975638	0.823408508211471
LOC4993	LOC499331	0.03037338062776	5.11719130396826	0.685308952187475	0.407764383135165	0.823408508211471
Mtarc2	Mtarc2	-0.0598255516135257	4.07978434472321	0.685247552682675	0.407785583657057	0.823408508211471
Anks4b	Anks4b	-0.152388903815295	-0.232037515804534	0.685226389180372	0.407792824218895	0.823408508211471
Stap2	Stap2	-0.089722638738178	1.26019431706487	0.684829697012788	0.40792857988258	0.823408508211471
Cdon	Cdon	0.0519620110591959	4.41460327178683	0.684807111393326	0.407936311080835	0.823408508211471
Fbxo25	Fbxo25	-0.0234949677608617	6.5259961817351	0.684666943357485	0.40798429640042	0.823408508211471
Vasp	Vasp	0.0350809013145955	5.66894365460636	0.684610713946597	0.408003548280502	0.823408508211471
LOC1002	LOC100294	-0.020997333175288	6.72633942896073	0.684349237162006	0.40809343052513	0.823408508211471
Lrrc58	Lrrc58	0.035416514516668	5.50204594569609	0.6842676694195	0.408121021542285	0.823408508211471
Atp5pf	Atp5pf	-0.04654244440653	6.51303890253672	0.683948548673566	0.408264532065803	0.823565343525613
Arl4c	Arl4c	-0.0242924110996881	7.22590004166965	0.683050016209009	0.408539436451939	0.823987904386591
Hk1	Hk1	-0.0208047803705946	9.15090610705203	0.681823880932026	0.408959302943227	0.824507868277363
Slc16a6	Slc16a6	0.0729981415488904	3.10838416453475	0.681757619881466	0.408982150347124	0.824507868277363
Prtg	Prtg	0.182243573280645	-0.469067867442084	0.681723721030224	0.408993798239246	0.824507868277363
Inka1	Inka1	0.103327541734487	1.09571442618768	0.681403715450429	0.409103778490855	0.824595788789488
Wdr46	Wdr46	0.0327319654748216	5.78492162405597	0.681150140057355	0.409190958704783	0.824597470947588
Fbxo38	Fbxo38	0.0305538445670912	5.37204066000694	0.681012228163482	0.409238384808635	0.824597470947588
G3bp2	G3bp2	0.0188650555668126	7.47656380815104	0.680297749974294	0.409484213855069	0.824688418258044
Nenf	Nenf	-0.0521313474243436	4.43786798996023	0.680228139128403	0.409508176212172	0.824688418258044
Galnt13	Galnt13	-0.0563075155584794	3.82819701289515	0.68022087248184	0.409510677861618	0.824688418258044
Abca1	Abca1	0.0696678974338167	4.62574570625586	0.679989856853894	0.40959021689796	0.824688418258044
Smyd5	Smyd5	0.0225089154760713	6.00317955288952	0.679961071451865	0.409617470946061	0.824688418258044
Ak3	Ak3	0.0410048697125119	5.51243580641801	0.679414888389076	0.409788278012804	0.824900121614264
Slc13a3	Slc13a3	0.108828083170719	4.34776802351741	0.678409043367054	0.410135103170756	0.82534045527313
Trim32	Trim32	0.020302199719499	7.13117444230491	0.678148065321068	0.410225161458292	0.82534045527313
Fam220a	Fam220a	0.0324912155988088	6.03787238233827	0.677889923325807	0.410314268694478	0.82534045527313
Cyb561a3	Cyb561a3	0.0699201353479007	2.57503905078818	0.67782049622329	0.410338242219549	0.82534045527313
Rasip1	Rasip1	0.0314692345568304	6.00207031814059	0.677817843480526	0.410339158076702	0.82534045527313
Slco1a4	Slco1a4	0.159071154739271	1.61141067707598	0.677066221423936	0.410598790988847	0.825521861628683
Exoc6b	Exoc6b	0.0225847575126234	6.91710526079372	0.676911686468998	0.410652206553261	0.825521861628683
Arl1	Arl1	-0.0328628092667263	5.73658697274327	0.676794615951742	0.410692678589428	0.825521861628683
Ints13	Ints13	0.039818847172827	4.81589064926934	0.676540880684127	0.410780409630886	0.825521861628683
Ppm1g	Ppm1g	-0.0206376158696385	7.74644279566513	0.676347112342761	0.410847427948746	0.825521861628683
Fbxo4	Fbxo4	0.0793869679830915	1.16921692790722	0.676225128472318	0.410889626114453	0.825521861628683
Snx15	Snx15	-0.0338451464907384	4.86348802423203	0.676203750924202	0.410897021956688	0.825521861628683
Pofut1	Pofut1	0.0422398033280698	3.46898334200131	0.675722873880176	0.411063439455777	0.82572405626896
En2	En2	-0.467166751851573	-0.577363981755115	0.675270863455381	0.411219957558672	0.825906062310126
Cdk14	Cdk14	-0.0335480727374413	7.29557303326843	0.674453674282827	0.411503045325881	0.825924217258761
Cog7	Cog7	0.0287582198841427	4.97118682172319	0.674237759163105	0.411578021354756	0.825924217258761
Cyb561d2	Cyb561d2	0.0464566698767922	4.63880527823542	0.674233854043464	0.411579375702092	0.825924217258761
Ampd2	Ampd2	-0.0203545115954402	7.2883928078818	0.67411079292004	0.411622058297739	0.825924217258761
Rmnd5b	Rmnd5b	0.0309897252720943	5.46897229010001	0.67401201022944	0.41165632486612	0.825924217258761
Slc18a2	Slc18a2	-0.0903936922742844	4.24511977752062	0.673944736807144	0.411679663641412	0.825924217258761
Sntb2	Sntb2	0.104697644314363	0.722629927762644	0.673787103677888	0.411734358019861	0.825924217258761
Rrm2	Rrm2	-0.0598199219185956	3.92701345385091	0.67323295760184	0.411926718556275	0.825924217258761
Selenop	Selenop	0.0759802929024185	6.98214411708024	0.673062596683465	0.411985879891625	0.825924217258761
Fam3a	Fam3a	0.05474852905302	4.56752227725541	0.673019815333461	0.412000739066125	0.825924217258761
Mtmr11	Mtmr11	-0.0624456548852756	3.50699979022635	0.672960824196323	0.412021229654734	0.825924217258761
Bmf	Bmf	0.0847442392299422	1.96830921399739	0.672927356475924	0.412032855345146	0.825924217258761
LOC3141	LOC314140	0.0463048627224975	3.40172393450261	0.672137209045538	0.41230748963254	0.826300606299306
Vps53	Vps53	-0.0192920695507962	6.2685121945801	0.671905262438393	0.412388123500317	0.826300606299306
Nrxn2	Nrxn2	-0.0288912305763345	10.275685050116	0.671726048211951	0.412450474616439	0.826300606299306
Crat	Crat	-0.0267500991815817	6.28766699013717	0.671614792938584	0.412489182655464	0.826300606299306
Pafah2	Pafah2	0.0642935140173509	2.28304777832678	0.671234199879839	0.412621638803129	0.826323374026446
Ankrd52	Ankrd52	0.0415939754255255	6.68171120130289	0.671084895545292	0.412673618637361	0.826323374026446
Copb2	Copb2	0.0211211984750574	7.5132201513001	0.670997864379096	0.412703922321737	0.826323374026446
Hip1	Hip1	-0.0325268379366499	7.68475696894793	0.670724660921039	0.412799071322473	0.826390713263185
Dnajb13	Dnajb13	-0.0783586691143867	1.64397753828339	0.669875144250753	0.41309514081111	0.826647675550284
Ybx2	Ybx2	0.11011899952906	3.67066660047768	0.669684733755446	0.413161544776835	0.826647675550284
Uap1l1	Uap1l1	0.077552308450502	2.4743099943842	0.669213451947826	0.413325967921442	0.826647675550284
Traip	Traip	-0.0508469762073074	2.97952930406674	0.669159861597228	0.413344677889506	0.826647675550284
Kpna3	Kpna3	0.0217602087285705	6.67031930189306	0.669006399456556	0.413398235984994	0.826647675550284
Mvk	Mvk	0.0317986137552079	5.99189479125904	0.668730606836675	0.413494525585892	0.826647675550284
LOC1009	LOC100912	-0.0844370415152117	5.46968522918501	0.66839892123572	0.413610373429474	0.826647675550284
Echs1	Echs1	0.0455500681113076	5.2380510780356	0.668116328676786	0.413709108541322	0.826647675550284
Gpr26	Gpr26	0.0479217441423736	4.55789614506814	0.667820543621829	0.41381249816894	0.826647675550284
Lmbrd1	Lmbrd1	-0.0238547123253617	5.68459991891929	0.667773834039799	0.413828828065611	0.826647675550284
Ttc30a	Ttc30a	-0.0626094973274705	2.46795571237134	0.667339356319523	0.413980769236113	0.826647675550284
Ripk4	Ripk4	0.103828958409069	1.39133797748563	0.667331305421499	0.413983585493257	0.826647675550284
Cops7a	Cops7a	0.0300228165815389	5.94524399340868	0.667156997663248	0.414044566641088	0.826647675550284
Ift20	Ift20	0.0311906314730756	5.25057967227476	0.667117048443657	0.414058544403891	0.826647675550284
Sema4d	Sema4d	0.0239834069361203	5.53713852513367	0.667037484452841	0.414086385286885	0.826647675550284
Dbi	Dbi	-0.0530485461772212	6.6453655255079	0.666953188605419	0.414115885158439	0.826647675550284
Cd63	Cd63	0.0466272747069	5.98620668064805	0.666908211416811	0.4141316264216	0.826647675550284
Vps28	Vps28	-0.0311052328256047	6.66670827279631	0.666888816499181	0.414138414605478	0.826647675550284
Mgat4a	Mgat4a	0.0358246332552754	5.72267547814175	0.666713040599916	0.4141999432455	0.826647675550284
Tm9sf2	Tm9sf2	0.0192864410200516	7.13703510451378	0.666244082492121	0.414364163625381	0.826735468742437
Map3k7	Map3k7	0.0247491170051844	7.16276199810443	0.666173790534046	0.414388786856864	0.826735468742437
Eif4a3	Eif4a3	-0.0292748652016142	6.66257676949309	0.666014501282085	0.414444593798851	0.826735468742437
Cyfip1	Cyfip1	0.0267289280388226	6.49682971593606	0.665347322654952	0.414678460049883	0.826902819346861
Calb1	Calb1	-0.0803198547950089	5.58923164398341	0.665242082311181	0.414715368217879	0.826902819346861
Fam53b	Fam53b	0.0362726576455874	4.72305998757278	0.665204038782704	0.41472871128069	0.826902819346861
Mfsd11	Mfsd11	0.0469055388389477	4.71543769852526	0.664228108890725	0.415071218334681	0.827132667964881
Homez	Homez	-0.0546138185141334	2.61416832234783	0.663832626260167	0.415210133978141	0.827132667964881
P3h4	P3h4	0.0685994463230515	4.1437576754905	0.66366242980736	0.415269937693831	0.827132667964881
Cited2	Cited2	0.0269860586263255	6.5005603884246	0.66322679035806	0.415423071040544	0.827132667964881
Gpr4	Gpr4	0.0834971022761656	2.0858954279439	0.663111797226705	0.415463506685882	0.827132667964881
Timp3	Timp3	0.0527923833438473	5.44352127266045	0.662610329008469	0.415639909152274	0.827132667964881
Usp32	Usp32	0.0261619091403341	7.19234578545405	0.662530744055722	0.415667914704997	0.827132667964881
Rmdn3	Rmdn3	-0.0279207476573866	5.10510907362518	0.662430596418034	0.415703160581937	0.827132667964881
Tef	Tef	-0.0279932874441381	6.53889906201976	0.662289446948155	0.415752843074898	0.827132667964881
Ogfod2	Ogfod2	0.0482993330742589	3.44548542547772	0.662244482637007	0.415768673077792	0.827132667964881
Ankrd46	Ankrd46	-0.0248509110254582	7.8791280692796	0.662158526549042	0.415798924992059	0.827132667964881
Irf6	Irf6	-0.0881615282647913	2.86078591740519	0.662127668771902	0.415809799654289	0.827132667964881
Mbd1	Mbd1	0.0224100130834961	5.96275715966732	0.66164373385547	0.415980242541364	0.827132667964881
Ppp1r1b	Ppp1r1b	-0.133865520873346	6.28658182421679	0.661636555172095	0.415982771668193	0.827132667964881
Rbl1	Rbl1	0.065507008573497	2.78934442068375	0.66155682894248	0.416010863603135	0.827132667964881
Churc1	Churc1	-0.0747794298208975	3.55020115283944	0.661523576730389	0.416022578182992	0.827132667964881
Stard5	Stard5	0.077243296921153	1.54900027240422	0.661388522608867	0.416070170194006	0.827132667964881
Strn	Strn	0.0393342838947579	5.77748972114734	0.661294031767127	0.416103472824251	0.827132667964881
Lpcat2	Lpcat2	0.0652925731316848	3.27113360019718	0.661254517404757	0.416117400554476	0.827132667964881
Il12rb2	Il12rb2	0.0757477897225682	3.13245001142226	0.660318056738262	0.416447679751309	0.827662050662111
Odc1	Odc1	0.0362688805385304	7.90636629134616	0.659266788268937	0.416818913963724	0.82817363474101
Ndufa10	Ndufa10	-0.0279536296479805	6.83928622964225	0.659217386705002	0.416836371208461	0.82817363474101
Tars2	Tars2	0.0297447594294713	5.24432773492947	0.658722189217599	0.417011421277821	0.828371578200098
Pcdhb21	Pcdhb21	-0.0781739451360201	2.02772893087074	0.658558812238567	0.417069198245468	0.828371578200098
Fads3	Fads3	-0.0285438183266023	5.60854904052788	0.658314948591439	0.417155460798456	0.828405248992442
Tubgcp5	Tubgcp5	0.0391773998224104	4.63727738343866	0.658131723502791	0.417220290942861	0.828405248992442
Iqsec3	Iqsec3	-0.0402618429641421	7.05819777942359	0.657743198884795	0.417357812011826	0.82854644452058
RGD1359	RGD135962	0.0210537327449314	5.51908121549452	0.65658124490421	0.417769493232084	0.829116703472261
Slc25a34	Slc25a34	-0.0662814258478079	2.32787479829877	0.656441427937459	0.417819071254864	0.829116703472261
Kctd10	Kctd10	0.0214543682873423	6.77389845964579	0.656082504204491	0.41794638290736	0.829116703472261
Gabarapl2	Gabarapl2	-0.0315065128427106	6.54696411898357	0.655916550117837	0.418005268970282	0.829116703472261
Aamdc	Aamdc	-0.0839209621988839	1.5075337256441	0.655775237914604	0.418055417290004	0.829116703472261
Arl4d	Arl4d	0.0549112339098512	4.40167797292851	0.65564166645008	0.418102828723038	0.829116703472261
Nr1h3	Nr1h3	-0.116421668670447	0.338476417432959	0.655614674673217	0.418112410674236	0.829116703472261
Iffo2	Iffo2	0.0399280315577525	4.06325031468618	0.654386393909711	0.418548780996609	0.829587841250744
Ywhag	Ywhag	-0.0261691067543933	10.1903411309432	0.653734676200806	0.418780591545833	0.829587841250744
Efr3a	Efr3a	0.0335537177005027	5.55682424940381	0.653725341243366	0.418783913200316	0.829587841250744
Zfp496	Zfp496	0.0279678412142522	5.28203082218211	0.65372150627897	0.418785277946738	0.829587841250744
Atp13a4	Atp13a4	0.116388893819503	0.552585644441933	0.653651887499479	0.418810053474518	0.829587841250744
Smc4	Smc4	0.0464322525792524	4.85896700317958	0.653526457306782	0.418854693549707	0.829587841250744
Mapk15	Mapk15	-0.0765166176772999	3.77779458096067	0.653415783165428	0.418894088773998	0.829587841250744
Exoc5	Exoc5	0.0221068654349601	6.61443193421752	0.653158092310612	0.418985828990743	0.829587841250744
Kif20b	Kif20b	0.064093917021526	2.89256688514735	0.652923257166705	0.419069473724265	0.829587841250744
Thbs3	Thbs3	0.0855648358171727	3.98946654381174	0.652860351972464	0.41909188168878	0.829587841250744
Masp1	Masp1	0.0315665557006903	6.62291716572865	0.652844443872084	0.419097548717409	0.829587841250744
Ralgapb	Ralgapb	0.0253738308225842	7.64891502701251	0.652469685663732	0.419231083998288	0.829587841250744
Cops9	Cops9	-0.0541623983898628	5.98189104373534	0.652459226235891	0.419234811850688	0.829587841250744
Cacnb2	Cacnb2	-0.0451036830186027	4.86057703497205	0.652105455281415	0.419360928703417	0.829587841250744
Ndor1	Ndor1	0.0543939835488253	4.7341281400477	0.651861147750807	0.419448055613321	0.829587841250744
Cacna1e	Cacna1e	0.101791137763592	6.64951532355015	0.651724739239711	0.419496714644074	0.829587841250744
Gdf10	Gdf10	-0.0552627359715782	4.30559240328264	0.651705428457717	0.419503603550723	0.829587841250744
Aimp1	Aimp1	-0.035775389449713	5.75557598207522	0.651099481716514	0.419719860263456	0.829587841250744
Pitx2	Pitx2	0.402811708575271	1.31502867444806	0.65093827797011	0.419777063251408	0.829587841250744
Gabre	Gabre	-0.165391351373512	1.09543405110169	0.650875768978082	0.419799743241865	0.829587841250744
Nudt9	Nudt9	-0.0352402623933377	5.21449452698275	0.650862308074124	0.419804550562275	0.829587841250744
F8	F8	0.0867147558566997	4.74155675488941	0.650572956385119	0.41990790717454	0.829587841250744
Sall3	Sall3	0.0599878085547238	4.65897011890954	0.650045603090831	0.420096375729816	0.829587841250744
RGD1311	RGD131174	-0.0482485224833762	3.27460251236853	0.649984680356873	0.42011815677526	0.829587841250744
Itgae	Itgae	0.0543185737145749	2.61642637086289	0.649636594134073	0.420242636583627	0.829587841250744
Crhr1	Crhr1	0.0366506295904659	4.55497713600627	0.649510424422722	0.420287768970844	0.829587841250744
RGD1311	RGD131134	0.0336383129789124	4.45902275967457	0.649385698926558	0.420332393841659	0.829587841250744
St8sia6	St8sia6	-0.157118095323949	-0.334828628943132	0.649275849861888	0.420371665341434	0.829587841250744
Dync1h1	Dync1h1	0.0734114704660345	10.078979122666	0.649262868806417	0.420376346499669	0.829587841250744
Otub1	Otub1	-0.0247957376481183	7.51810291540957	0.649036373065904	0.420457412109558	0.829587841250744
Mnat1	Mnat1	0.0410857026197192	3.87589996476037	0.649021328580943	0.420462794955124	0.829587841250744
Nploc4	Nploc4	0.0201767165996213	6.77008331000523	0.648841195400983	0.420527287307901	0.829587841250744
Cdh23	Cdh23	-0.124784031031361	2.90290656617397	0.648728152922558	0.420567765363641	0.829587841250744
Flnb	Flnb	0.0745167366875862	6.64625651123688	0.648316167759944	0.420715327526247	0.82974808291713
Rin1	Rin1	-0.0904126991154762	2.21551312812796	0.647826013371258	0.420891010438091	0.829964353264692
Casp4	Casp4	0.0721675384384572	1.52347574769933	0.647188394637084	0.421119699003504	0.830249730859794
Rps20	Rps20	-0.0410323053025612	7.56758082843243	0.64705185148506	0.421168695743841	0.830249730859794
Hba-a3	Hba-a3	-0.161635871581811	2.61023595743295	0.646831284317571	0.421247861402598	0.830272312025615
Psd	Psd	-0.0312186465877913	9.51961922350164	0.646397761957132	0.421403525440223	0.830288846603182
Prpf3	Prpf3	0.0342429520049046	4.95475157131925	0.646031739221861	0.421535019644606	0.830288846603182
Tap1	Tap1	-0.0765265272227286	2.04233320812543	0.646023716484834	0.421537902306946	0.830288846603182
Fmr1	Fmr1	0.030014369105522	6.53257678867314	0.645784928178693	0.421623717561702	0.830288846603182
Pop4	Pop4	-0.0348294432952753	4.92355493896653	0.645692680951313	0.421656881157981	0.830288846603182
Adnp	Adnp	0.0270957196276375	8.26575241062722	0.645684752430583	0.421659731295876	0.830288846603182
Layn	Layn	0.114937784512832	0.0876600499275626	0.645396811730842	0.421763227261765	0.830360872971102
Cables1	Cables1	0.0348132854126111	4.56982377253535	0.644857516201036	0.421957274357816	0.830568242385934
Syn1	Syn1	-0.031270661699963	8.70302865201234	0.644666577317935	0.422025996456427	0.830568242385934
Gabrg3	Gabrg3	-0.0817078971432348	3.25947123088742	0.644403868875537	0.422120540974412	0.830568242385934
Ccdc173	Ccdc173	-0.132187013395665	-0.107771310776394	0.64434263815707	0.422142625875764	0.830568242385934
Rafp1	Rafp1	0.115799607124001	2.85971306851535	0.644171809335731	0.422204149637946	0.830568242385934
Sdr39u1	Sdr39u1	-0.0321730478695864	5.14314276118311	0.643910198450243	0.422298394437246	0.83062174017127
Apoc1	Apoc1	-0.120279430156025	0.133932456176379	0.643715323140924	0.422368618234875	0.830627412819307
Tm2d3	Tm2d3	-0.0387933391306817	4.74202155097103	0.642916856100413	0.42265588149159	0.831064981298815
Sel1l	Sel1l	0.0248126855437173	7.6745550019282	0.642355347148353	0.422859176642398	0.831268928848323
Glb1	Glb1	-0.0447525539027987	4.4524150888819	0.642031951729152	0.422975954459478	0.831268928848323
Cep104	Cep104	0.0287267216789495	5.78734226472763	0.641727789634679	0.423085821218184	0.831268928848323
Usp19	Usp19	0.0293356033255758	7.02607647336238	0.641521691267425	0.423163921454805	0.831268928848323
Siglec5	Siglec5	0.14948777479797	0.825126722020957	0.641321825435753	0.423232550009524	0.831268928848323
Unc5c	Unc5c	0.0488851948410775	5.72885482379657	0.641285358722744	0.423245732747907	0.831268928848323
LOC2941	LOC294154	0.0190479917455267	6.9881865317595	0.641242109452851	0.4232613688724	0.831268928848323
Cldnd1	Cldnd1	-0.0248317579923553	6.61586867045256	0.641141419299927	0.423297774730286	0.831268928848323
Slc25a32	Slc25a32	0.0486531167204792	3.03335454940452	0.640737005064025	0.423444042926787	0.831377103144861
Flot2	Flot2	-0.0231151697140608	6.80805316834712	0.640451466060277	0.423547362053325	0.831377103144861
Dolpp1	Dolpp1	-0.0245747071654017	5.33673007025769	0.640363806719336	0.423579087471531	0.831377103144861
Med20	Med20	0.045115123118617	4.94830111215741	0.64024541912147	0.423621941328526	0.831377103144861
Lamtor2	Lamtor2	-0.0404020049380759	5.4743430632564	0.639879360779306	0.423754847917896	0.831380475748817
Hes6	Hes6	0.0445581638829521	7.64330881705021	0.639868253720721	0.423758508423945	0.831380475748817
Dmrta2	Dmrta2	-0.066472575172039	2.97841879817776	0.639324269047748	0.423955595080863	0.831409432940983
Fut8	Fut8	0.0324057581402685	6.05654166749498	0.639277601864801	0.423972509161961	0.831409432940983
Plcxd3	Plcxd3	-0.0855790096329944	4.91082232021048	0.639269617231122	0.423975403133763	0.831409432940983
Ccnd2	Ccnd2	0.0617069337523698	5.48681044708973	0.638800076189919	0.424145641293138	0.831613418885956
Ace	Ace	0.106203715261733	4.6219424211735	0.638450303318948	0.424272522470765	0.831731318630582
Htr3a	Htr3a	-0.0761255461317882	3.37521874410659	0.638006794817712	0.42443348854148	0.831916794361215
[illegible]	[illegible]	[illegible]	[illegible]	[illegible]	[illegible]	[illegible]

Gtf3c6	Gtf3c6	0.0316950233067 54	4.42036388781 08	0.610149700922683	0.434731450670636	0.846917093865518
Lpin3	Lpin3	-0.104540883744781	3.12259283210983	0.609962952201101	0.434801759633141	0.846917093865518
Fgd5	Fgd5	-0.0503124781611524	4.48413355583455	0.609793948183281	0.434865402836379	0.846917093865518
Pbxl	Pbxl	-0.0660959301054874	1.82297921459181	0.609437226702155	0.434999782936738	0.846917093865518
Col7a1	Col7a1	0.0811025336833901	2.432504963871	0.609398713152018	0.435014295113526	0.846917093865518
Abcc1	Abcc1	0.0841589941549767	6.06897834896772	0.609227480962502	0.435078825544094	0.846917093865518
Drap1	Drap1	-0.0283147135854042	6.64944749529744	0.608971880932899	0.435175177928852	0.846917093865518
Entpd4	Entpd4	-0.0256888477179788	5.55704910028798	0.608956745954437	0.435180884213349	0.846917093865518
Map3k12	Map3k12	-0.0277686406594513	7.53330694869406	0.608408740327683	0.435387577341287	0.846917093865518
Spata5	Spata5	0.0340705769694009	4.13922785716898	0.608359577245247	0.435406127655552	0.846917093865518
Mtch2	Mtch2	-0.0235794744663076	7.36084924052722	0.608164621727141	0.435479700540731	0.846917093865518
Ift22	Ift22	-0.0348584547617975	4.28583808802613	0.607836651340158	0.435603513763023	0.846917093865518
Zdhhc17	Zdhhc17	0.0270385443033033	5.93028017576813	0.607743658241255	0.435638629678886	0.846917093865518
Pflp	Pflp	-0.0991302172999517	7.62957935393979	0.607691266437158	0.435658415686603	0.846917093865518
Zfp787	Zfp787	-0.0326481461655588	4.07713594282358	0.607227268237075	0.435833706724975	0.846917093865518
Nxph4	Nxph4	0.08016549466275	4.26842731492322	0.607084462681044	0.435887678021752	0.846917093865518
Tm2d1	Tm2d1	-0.0309289798198405	4.90295383144755	0.60700203423823	0.435918835266918	0.846917093865518
Polr2f	Polr2f	-0.0422701708399154	5.42821751820798	0.60697844002615	0.435927754262422	0.846917093865518
Gdi1	Gdi1	-0.0212629660776757	10.0387939820329	0.606889822364506	0.435961214360053	0.846917093865518
Mrps17	Mrps17	-0.0396252914443166	4.64048846320871	0.606868031757337	0.435969494412281	0.846917093865518
Cygr1	Cygr1	0.0587144106186004	3.47270374140421	0.606826627209585	0.435985149055185	0.846917093865518
Dph3	Dph3	-0.0249032630350176	5.40395602066882	0.606817774372141	0.435988496648738	0.846917093865518
Ap3d1	Ap3d1	-0.0190000195337252	8.72631195423736	0.606379621715434	0.436154208213809	0.846917093865518
Zcchc9	Zcchc9	-0.0463293152237538	4.76147612896086	0.606116908684815	0.436253619983036	0.846917093865518
Fam227b	Fam227b	0.120037486475296	-0.234875899458327	0.606052140218862	0.436277754558863	0.846917093865518
Rpp25	Rpp25	-0.0428434348897721	4.20799217736048	0.60588123316586	0.436342826699485	0.846917093865518
Ap5b1	Ap5b1	-0.0395097209655296	3.94688516556697	0.605743202944353	0.436395086143642	0.846917093865518
Slc27a2	Slc27a2	-0.0916752809726588	4.14743489565911	0.605653805524079	0.43642890001846	0.846917093865518
Ldah	Ldah	0.0241055972111878	5.91268582294982	0.605572488821757	0.436459733223712	0.846917093865518
Trim65	Trim65	-0.05147028924922 7	3.24776825289331	0.605375309010275	0.436534496470896	0.846917093865518
Nutf2	Nutf2	-0.0223741169274494	6.3301480421813	0.605324132664948	0.436553808298542	0.846917093865518
Nol10	Nol10	-0.0311582797790882	4.86312442725325	0.605220715881579	0.436592990922739	0.846917093865518
Svbp	Svbp	0.0447514047418361	5.20684895564547	0.604962553857959	0.436690826738886	0.846943071923685
Pxmp4	Pxmp4	-0.0375605452441681	4.53926146607778	0.604406331818673	0.436901732440435	0.846943071923685
Gpr162	Gpr162	-0.0210743575045905	7.72411747794726	0.604339053211795	0.436927253286616	0.846943071923685
Prdx2	Prdx2	-0.0338812410128675	8.85889854094691	0.60362971666423	0.437195665290249	0.846943071923685
Kcnk5	Kcnk5	-0.109876318234199	0.271247387236312	0.603609061468447	0.437204309413564	0.846943071923685
Thoc3	Thoc3	-0.0254954554903198	5.91144924347426	0.603583812703562	0.437213896961964	0.846943071923685
Snta1	Snta1	-0.0399712767018952	4.30123322444858	0.603547388718724	0.437227729570136	0.846943071923685
Ly6h	Ly6h	-0.0377810201744353	8.52888801174104	0.603523443313239	0.437233024361869	0.846943071923685
Hps5	Hps5	0.0660659457105183	3.82549644516071	0.60350094258726	0.43724536694927	0.846943071923685
Isy1	Isy1	0.0359331711305151	4.78312553240293	0.603104279284928	0.437396048124257	0.846943071923685
Nck1	Nck1	-0.0443838796839018	4.43639384461035	0.602562933174084	0.437601818438824	0.846943071923685
Brd7	Brd7	0.0178236711573692	6.86788680929847	0.602532543567072	0.437613374181019	0.846943071923685
Rnf4	Rnf4	-0.0200674691449966	5.88155033328106	0.602100085681371	0.437777868255405	0.846943071923685
Fign	Fign	0.0932482178574658	3.20413566286057	0.601910260680745	0.43785010190022	0.846943071923685
Nt5c3a	Nt5c3a	-0.0256252803287071	6.15945970672417	0.60145984729764	0.438021569683653	0.846943071923685
Nipsnap2	Nipsnap2	-0.035429421805611	5.01141194855532	0.601132042581267	0.438146426243166	0.846943071923685
Stk16	Stk16	0.0239685491620 72	5.98692261327882	0.601110391919519	0.438154574621999	0.846943071923685
Rbl2	Rbl2	0.0248283358990017	5.84987158260795	0.600844418577601	0.438256023401366	0.846943071923685
Ndufa2	Ndufa2	-0.0489809534377574	5.102895150226	0.600677550017423	0.438319626772625	0.846943071923685
Sec61a2	Sec61a2	0.0177231650814561	7.02346919053341	0.600633188402043	0.438336537959165	0.846943071923685
Gask1b	Gask1b	0.0881528436110704	2.60644958845004	0.600609577921986	0.438345528898501	0.846943071923685
Atg14	Atg14	-0.0381536958356 15	4.28084206671315	0.600531746176358	0.438375212724305	0.846943071923685
Nptxr	Nptxr	-0.0275403344077047	9.63032772424776	0.600270019889206	0.438475019802289	0.846943071923685
Phkb	Phkb	0.0532073843036102	4.45114163524547	0.599919234230555	0.438608843539222	0.846943071923685
Uba2	Uba2	0.0206189434250765	6.88356855734885	0.599838098306577	0.43863988055774	0.846943071923685
Riox1	Riox1	0.0388518740506799	4.86102298689048	0.59963281622916	0.438718157623363	0.846943071923685

Glra3	Glra3	-0.110292730664446	0.777970908554127	0.537375005235418	0.463522649962974	0.859808662519971
Srm	Srm	0.0274105622841288	6.83219184995617	0.537243240826484	0.463577467672384	0.859808662519971
Rnf7	Rnf7	-0.0369032730568776	5.47491067761534	0.537003760666238	0.463677124634881	0.859808662519971
Sart1	Sart1	-0.0242378976133028	6.2839499948441	0.536900877487028	0.463719948796234	0.859808662519971
Aspa	Aspa	0.136118790919764	0.814448233294985	0.536881500772452	0.463728014878266	0.859808662519971
Mul1	Mul1	-0.0240864021404109	4.97828857112866	0.536709438807037	0.463799650143138	0.859808662519971
Ube2q1	Ube2q1	-0.0168525645339222	6.62987527966	0.536464386688628	0.463901704165213	0.859808662519971
Xkr6	Xkr6	0.0656558261958466	2.2011445617345	0.536405828264214	0.463926092438449	0.859808662519971
Rbis	Rbis	-0.0452396885821263	3.60188271942641	0.536287675423499	0.463975319284995	0.859808662519971
Sh3bp5l	Sh3bp5l	0.0242306446852091	5.96746282634234	0.536193343120559	0.464014624266205	0.859808662519971
RGD1308	RGD130811	0.0460865632912251	2.76438562996524	0.53564221869631	0.46424436478221	0.858287483792528
Poc5	Poc5	-0.0304780392720274	4.06805818344874	0.535542442493053	0.464285978675797	0.858287483792528
Txnl4b	Txnl4b	0.0398194595942585	3.89079403147789	0.535396061811362	0.464347035845314	0.858287483792528
Tjap1	Tjap1	0.0455158367773431	4.95936624669717	0.535263449976711	0.464402362737912	0.858287483792528
Erg	Erg	0.0691340258231655	2.17326090312087	0.53506906198119	0.464483482239382	0.858287483792528
Bcas2	Bcas2	-0.03357907062725	5.52787317437068	0.53482333221254	0.46458805940588	0.858287483792528
Fyn	Fyn	-0.0168671298966603	8.07513451082642	0.534661923028729	0.464653457562831	0.858287483792528
Stag3	Stag3	-0.154637781604479	0.858037385492906	0.5346137980083	0.464673555583756	0.858287483792528
Rft1	Rft1	-0.0415186025455681	3.35678045425645	0.534282501391651	0.464811950681206	0.858299326941083
Rdm1	Rdm1	0.0844735742633912	1.1849361526204	0.534128226745191	0.464876419445906	0.858299326941083
Slfn13	Slfn13	0.068748700732515	2.66802764187532	0.533942414958517	0.464954086000887	0.858299326941083
Polr1d	Polr1d	-0.0393839243640614	5.41241986195326	0.533939918922987	0.464955129447694	0.858299326941083
Onecut1	Onecut1	-0.195492974791535	0.0565897557526575	0.533772502745476	0.465024706775477	0.858300779058821
Rragd	Rragd	-0.0248029428108487	5.51026527664053	0.533608190760575	0.465093838921106	0.858301408291324
Ficd	Ficd	0.0982656428255816	2.3823231126747	0.532916326980001	0.465383349103132	0.858681101631526
Chd8	Chd8	0.0387451793021236	7.5697221056459	0.532478805823987	0.46556666622049	0.858681101631526
Tbce	Tbce	-0.0243845198253829	5.68526882765602	0.53222327311007	0.465673646248031	0.858681101631526
Ankrd24	Ankrd24	0.0493409232307411	3.44375631890316	0.532121032348783	0.465716496002089	0.858681101631526
Akr7a2	Akr7a2	0.0414544412152246	4.45908275298011	0.531996153785059	0.465768841962893	0.858681101631526
Pafah1b1	Pafah1b1	-0.0204265744315712	9.73296263174549	0.5318605719829	0.46582568511059	0.858681101631526
Zfp955a	Zfp955a	-0.0454359851059121	4.85551245406762	0.531663650713	0.465908264815362	0.858681101631526
Tpcn2	Tpcn2	-0.0854001337258791	2.14336134636872	0.531552265499215	0.465954985012994	0.858681101631526
Fbxo11	Fbxo11	0.0201182932017472	7.50502028079483	0.531469832958344	0.465990068362334	0.858681101631526
Ikzf1	Ikzf1	0.162801547995676	1.00169523413925	0.531186693118897	0.466108375961293	0.858681101631526
Ubtd1	Ubtd1	-0.0487211675339131	3.5610166573535	0.531164112566799	0.466117853212035	0.858681101631526
Psmd2	Psmd2	-0.0164949156655007	8.34684656886724	0.531146039355313	0.466125438915631	0.858681101631526
Hmgn3	Hmgn3	-0.0304970537644506	6.00039304286247	0.53094041826807	0.466211756288618	0.858713327902729
Atp1a2	Atp1a2	0.0454591850540631	8.34434108480073	0.530473729327881	0.466407761930788	0.858909626608569
Paxip1	Paxip1	0.0206460016950609	5.98239999558693	0.529942653217972	0.466630970255874	0.858909626608569
Parn	Parn	0.0265342737725676	5.07950091873137	0.529488311333203	0.466822063470741	0.858909626608569
Alg3	Alg3	0.0339498450904321	4.13931164166889	0.529341969941733	0.466883640400581	0.858909626608569
Slc25a37	Slc25a37	0.0926918707096204	0.776378682323226	0.529272137649613	0.466913028733851	0.858909626608569
Dnajc13	Dnajc13	0.0357429966758983	6.68515530311264	0.529021683334634	0.467018454718172	0.858909626608569
Lsm8	Lsm8	-0.0340430148031182	4.52241004729877	0.528968260483737	0.467040947416351	0.858909626608569
Flad1	Flad1	0.0232140442030066	5.10242136496729	0.528757707890954	0.467129613584314	0.858909626608569
Rtn4rl1	Rtn4rl1	-0.0321162235618633	6.10353184006247	0.528529211778604	0.467225866520004	0.858909626608569
Sec14l1	Sec14l1	0.0185432155413067	7.1381077663635	0.528298560587544	0.467323058532379	0.858909626608569
Cdc42ep	Cdc42ep2	0.039146482340589	3.85359622887839	0.528297526782535	0.467323491442457	0.858909626608569
Raly	Raly	0.0291559011594066	7.79538783658196	0.528288414075931	0.467327335867391	0.858909626608569
LOC6885	LOC688553	-0.117064028872195	0.270054741487633	0.528015798302221	0.467442255643479	0.858909626608569
Sv2b	Sv2b	-0.0302926417214856	6.9365989854595	0.527405150071658	0.467699835836581	0.858909626608569
Cldn23	Cldn23	-0.0842545915555602	1.20899370089305	0.527391526739976	0.467705584950623	0.858909626608569
Dap3	Dap3	-0.0230295961299032	5.60908349615892	0.527214704413694	0.467780215146876	0.858909626608569
Dnai3	Dnai3	0.123396529642627	1.89378978821861	0.527028447819802	0.467858847672795	0.858909626608569
Rpl29	Rpl29	-0.0312315205125542	8.92509542317528	0.526847340158838	0.467935327200865	0.858909626608569
Pex13	Pex13	-0.0349457392573843	4.21230270726524	0.526620982582301	0.468030943183565	0.858909626608569
Set	Set	-0.0278629298159855	5.8004370304835	0.526597845410997	0.468040718251728	0.858909626608569
Ptger2	Ptger2	0.129496096646732	-0.347093826365469	0.526404642999537	0.468122356776771	0.858909626608569
Ankrd27	Ankrd27	0.0401495179581787	4.94520749408132	0.526354778975886	0.468143430725217	0.858909626608569
Ifngr1	Ifngr1	0.0294682298377013	5.42123027526182	0.526250360917601	0.468187565589887	0.858909626608569
Gatd1	Gatd1	-0.0264248572182015	4.90656889020973	0.526084629781323	0.468257629919011	0.858909626608569
Ppp3ca	Ppp3ca	-0.0265221571953028	7.98933648967122	0.526049840620203	0.468272339467662	0.858909626608569
Pnpla1	Pnpla1	0.221355276409887	1.48289651865995	0.525732823830273	0.46840594934828	0.858909626608569
Tmem230	Tmem230	-0.0276160000028295	4.63265896685767	0.525726521195708	0.468409080858971	0.858909626608569
Asmt	Asmt	0.0910208482667497	1.59234503728135	0.525697159939767	0.468421501784975	0.858909626608569
Fxr1	Fxr1	-0.0275586903753779	6.0674388326691	0.525482161725492	0.468512470213645	0.858909626608569
Zfyve21	Zfyve21	0.0406644602877606	3.66396855298814	0.525269008916574	0.468602898055957	0.858909626608569
Htr1b	Htr1b	0.0624601029157724	3.89858882356662	0.52526554325982	0.468604153106539	0.858909626608569
Dpy19l4	Dpy19l4	0.03723781886188	4.06318139176634	0.525060318718943	0.468691040075763	0.858909626608569
Fgd1	Fgd1	-0.0226231285526248	5.87060102015237	0.524944984126336	0.468739881242171	0.858909626608569
Mfn1	Mfn1	0.0478277906884984	6.44739730618996	0.524828140619238	0.468785123958217	0.858909626608569
Olig1	Olig1	-0.0341765418706414	7.29440965421403	0.524724819623714	0.468832137838967	0.858909626608569
Eif3h	Eif3h	-0.0227845769187255	7.58652805201841	0.524703082858124	0.468842346765905	0.858909626608569
Helz2	Helz2	0.0772415435035308	1.77973198829429	0.524648894454789	0.468865389663458	0.858909626608569
Thra	Thra	0.0311220193803858	9.70785920121224	0.524343323720686	0.468994801338704	0.85901344474338
Def6	Def6	-0.0971415161685139	0.812721093837413	0.524135391722038	0.469083037589243	0.85901344474338
Slc6a3	Slc6a3	-0.482637568494671	0.697235701985881	0.524027730306088	0.469128605632876	0.85901344474338
Brf1	Brf1	0.0226332897228546	5.82761331446842	0.523496825005303	0.46935383577231	0.859079705678852
Sec11c	Sec11c	-0.0335673309769444	5.31708418111512	0.523413815371285	0.469389067332658	0.859079705678852
Myh7	Myh7	-0.0604424961111043	2.95020231492548	0.52340891789064	0.469391995003715	0.859079705678852
Rps6ka5	Rps6ka5	0.0322673037273592	5.85240255884789	0.5231102886371	0.469517614297321	0.859079705678852
Csad	Csad	-0.042858198449321	3.83808145523743	0.522837037740317	0.469633865127588	0.859079705678852
Insig1	Insig1	-0.0245437445599986	7.24588960163477	0.522624569393045	0.469724257677578	0.859079705678852
C1qa	C1qa	0.095898624734071	2.29183827804031	0.522558320016089	0.469752406015098	0.859079705678852
Dph6	Dph6	-0.0491054398390578	3.2227997761661	0.52252469826	0.469766700107588	0.859079705678852
Chd7	Chd7	0.0692345460215898	5.24813080725708	0.522365795046326	0.469834242247045	0.859079705678852
Mif4gd	Mif4gd	-0.0480372691745047	2.86872172522131	0.522320911230381	0.469853323039481	0.859079705678852
Rad17	Rad17	0.0344516877141334	4.26029298136759	0.521519038942649	0.470194422440933	0.859563576739041
Cacna1d	Cacna1d	0.0611572094578668	6.34576349098753	0.521345340546958	0.470268361580929	0.859563576739041
Gnpda2	Gnpda2	0.0469100011121841	2.93222001760765	0.521213160909205	0.470324640403149	0.859563576739041
Dtx3	Dtx3	-0.020493682480154	7.29553952897465	0.520744242885371	0.470524381739225	0.859693448050255
Sigirr	Sigirr	0.0898917139930051	0.429998829578761	0.520722828463654	0.470533506737533	0.859693448050255
Kifbp	Kifbp	-0.0212650369610062	6.62615301754888	0.52000297257861	0.470840413976091	0.8599732768338
Kcnh5	Kcnh5	0.0764096011636082	2.53680925045152	0.519951882899491	0.470862208095777	0.8599732768338
Elp2	Elp2	-0.016324434880255	7.08732923079561	0.519879880282133	0.470893438112408	0.8599732768338
Klhl24	Klhl24	0.0282994027589706	7.28208860115101	0.51929406850039	0.47114296726191	0.860082967988239
Gng8	Gng8	-0.0875864351288685	1.56846977766841	0.519074873424824	0.471236592059481	0.860082967988239
Bbs5	Bbs5	-0.0413778612377207	3.91292720471694	0.518949563468986	0.471290112561484	0.860082967988239
Pck2	Pck2	0.0289452827525682	4.46125780479527	0.518946083493248	0.471291596906005	0.860082967988239
Ndufaf4	Ndufaf4	-0.0246120776698553	4.78266924275199	0.518655465866338	0.471415786141122	0.860082967988239
Gsto2	Gsto2	-0.0578519825159401	2.4385392567065	0.518528951526342	0.471469864805884	0.860082967988239
Lage3	Lage3	-0.0408469503753151	5.7136137225875	0.518524809882642	0.471471635258633	0.860082967988239
Tceal7	Tceal7	0.0802060218993884	1.09771572038974	0.518441067870491	0.471507436548065	0.860082967988239
Dsc2	Dsc2	-0.070886282500622	1.37389981481609	0.518070417561333	0.471665946535215	0.860082967988239
Plcb3	Plcb3	0.0398667907202964	4.27952900793415	0.517969628477537	0.471709068037641	0.860082967988239
Klf1	Klf1	-0.0970320672775467	0.399617561266225	0.517963313977091	0.471711769651641	0.860082967988239
Vmp1	Vmp1	-0.0251936862077848	6.55061698095402	0.517380738082437	0.471961129021659	0.860245548256613
Ebna1bp	Ebna1bp2	0.0291547340779976	5.68786759403507	0.5171557248038	0.472057497074758	0.860245548256613
Spon1	Spon1	-0.0366085294484059	6.98290297396996	0.517012628867744	0.472118794177678	0.860245548256613
Olfm2	Olfm2	-0.0234523551397682	8.08246962129764	0.516934991146105	0.472194916197884	0.860245548256613
Aloxe3	Aloxe3	-0.0668132115789605	4.2760247224895	0.51646850408756	0.472352875243133	0.860245548256613
Prkn	Prkn	0.0440802065804122	3.08264528496788	0.516398320101757	0.472382112985304	0.860245548256613
Dfna5	Dfna5	0.0330258799274201	5.01169463790452	0.516376614402425	0.472391421146341	0.860245548256613
Calr	Calr	0.0296559964523487	9.23000317217738	0.51623161564576	0.472453609281721	0.860245548256613
Hsdl2	Hsdl2	-0.0237352393988932	4.44657325329203	0.5162070868111	0.472464130699149	0.860245548256613
Coq10a	Coq10a	0.0289992398038831	4.87714789347819	0.516115897952179	0.472503231481327	0.860245548256613
Plekha5	Plekha5	-0.0558654283437131	6.13814067773402	0.515985143941435	0.472559348275725	0.860245548256613
Azin1	Azin1	-0.0184485997419549	8.64255434855483	0.515137470809037	0.47292330157686	0.86051547842649
Gls	Gls	-0.0449086487015112	7.99333058126149	0.515134497867081	0.472924578824751	0.86051547842649
Acd	Acd	-0.0476177364430185	5.25280606008434	0.515131166758863	0.472926009956179	0.86051547842649
Cetn4	Cetn4	-0.0713402994506663	1.08615363155222	0.514997362140257	0.472983501725988	0.86051547842649
Aph1a	Aph1a	0.0242386152990525	6.41809030364984	0.514823764127669	0.473058108270581	0.860525726258746
Sytl5	Sytl5	-0.10337609854541	0.587979515677213	0.514272035080552	0.473295349714526	0.860792966788514
Galnt2	Galnt2	0.0251840592584176	7.84548797428815	0.514161285745871	0.473342994721167	0.860792966788514
Rfx1	Rfx1	0.0584460890214328	5.51744624654271	0.513740100971802	0.473524261842815	0.86099625789789
Fbxo3	Fbxo3	-0.0194149473221545	6.41304493125431	0.513580925476113	0.473592796181231	0.86099625789789
Psmc3ip	Psmc3ip	0.0493445699950598	2.99062948736231	0.513182959408716	0.473764214153797	0.861056448498796
Hspa13	Hspa13	0.0252031774481281	5.34783520862304	0.512757165434721	0.473947729989745	0.861056448498796
Katnbl1	Katnbl1	0.0356535399084888	4.17897676062684	0.512642141861233	0.473996893250916	0.861056448498796
Trmo	Trmo	-0.0617164289219283	1.86305423048291	0.512624017740009	0.474005139877452	0.861056448498796
Kifc1	Kifc1	-0.0488888780782289	3.57835641641529	0.512602941429293	0.474014228478592	0.861056448498796
Gne	Gne	0.0275705226194054	5.47332001608158	0.512282583257253	0.47415240926708	0.861056448498796
Gpr137b	Gpr137b	0.0520489007374013	2.46598888112694	0.51185866521385	0.474335358230352	0.861056448498796
Usp12	Usp12	0.0217182818998884	5.31934991145399	0.511857465210078	0.474335877277558	0.861056448498796
Pdxk	Pdxk	0.0294005263064176	4.64710735805016	0.51182058291247	0.474351799984596	0.861056448498796
Hdac5	Hdac5	0.0189291640365869	7.59086825005161	0.511687576105857	0.474409229460483	0.861056448498796
Mocs3	Mocs3	0.0358553235559992	3.78916634610539	0.511680717095665	0.474412190287788	0.861056448498796
Mrps5	Mrps5	-0.0226429775467343	5.1566985172619	0.511473395861678	0.474501728986505	0.861056448498796
Prkaca	Prkaca	-0.0193387300502674	7.43350494000932	0.511424029399535	0.474523053608858	0.861056448498796
Med28	Med28	-0.0283916075940637	5.51057088398646	0.511236154256167	0.474604223469233	0.861058568262314
Iftap	Iftap	-0.0386641747804702	3.64110595187313	0.511092906175598	0.474666127732041	0.861058568262314
Chchd2	Chchd2	-0.0482779781523187	7.0257217271463	0.510942227278591	0.474731257349861	0.861058568262314
Prpf19	Prpf19	0.0203703705418934	8.27203423635008	0.510755004490129	0.47481220295986	0.861080211675898
Afap1l2	Afap1l2	-0.0379854789624664	4.42670319869058	0.510387808612094	0.474971024885109	0.861112134137839
Inhbb	Inhbb	0.0454074019058171	4.05383120736871	0.510292915910497	0.47501208251895	0.861112134137839
Pard3	Pard3	0.0486060434027446	5.23821181310268	0.510182540173531	0.475059846518341	0.861112134137839
LOC6919	LOC691995	-0.0356789991423163	9.06091346541354	0.510076205167223	0.475105869811925	0.861112134137839
Tada1	Tada1	0.0253170303418619	5.07474893026363	0.509334195266085	0.475427219972575	0.861388541682386
RGD1359	RGD135926	-0.21240785268167	5.75302963869873	0.509323454470703	0.475431874204623	0.861388541682386
Slc10a3	Slc10a3	0.0591981156370384	3.12643530339789	0.50924588870921	0.475465487422184	0.861388541682386
Rhno1	Rhno1	-0.0307607319016565	4.30298900829586	0.50889619889936	0.475617073909423	0.861486742278846
Wdr48	Wdr48	0.0175248277170581	7.14767074571877	0.508682956818992	0.475709616719452	0.861486742278846
Rwdd1	Rwdd1	-0.0256384933202962	5.16591737957311	0.508426443315699	0.475820831250243	0.861486742278846
Mafg	Mafg	0.0282520051922603	5.43315703038605	0.508370426616383	0.475845051283373	0.861486742278846
Tek	Tek	0.0608449164172011	4.18945747625417	0.508324837401673	0.475864921678754	0.861486742278846
Plcg2	Plcg2	0.0787084395266431	1.03074321970801	0.507962342687362	0.476022275238991	0.861520687354976
Impdh2	Impdh2	0.022570240603768	6.4241120504331	0.507825290103114	0.47608178977187	0.861520687354976
Armc6	Armc6	-0.024943497783254	5.16020210376509	0.507804501379077	0.476090818250586	0.861520687354976
Fau	Fau	-0.033492815317622	8.25717204082583	0.50741968457541	0.476257992038211	0.861698228375895
Fez1	Fez1	-0.0270300598767568	8.6566278305533	0.506812771221419	0.476521847618049	0.862050615155923
Lemd2	Lemd2	-0.021684503924701	6.15797823184894	0.50647840555736	0.476667314095848	0.862050948520352
Dctn1	Dctn1	-0.0187361510638296	9.40882678802267	0.506394181758729	0.476703967240136	0.862050948520352
Xbp1	Xbp1	0.0306272063242907	7.07074431888405	0.505874121281423	0.476930092815861	0.862050948520352
Lhx9	Lhx9	-0.132088232453475	3.856911394639	0.505719825822145	0.476997604171402	0.862050948520352
G6pc3	G6pc3	-0.0297143687876433	5.32446650650394	0.505630866911599	0.477036360535489	0.862050948520352
Dnaja1	Dnaja1	-0.022438984628317	8.72564559710052	0.505524206561482	0.477082838450101	0.862050948520352
Hnrnpl	Hnrnpl	0.019813791386319	8.97136982772902	0.505518220321548	0.477085447136996	0.862050948520352
Dynll2	Dynll2	-0.0272072476272712	8.96683685068097	0.505403480316012	0.477135453093921	0.862050948520352
Micos13	Micos13	-0.0435213908667956	5.98957272851225	0.505304554934597	0.47717857358597	0.862050948520352
Nr3c1	Nr3c1	0.033075103509253	5.09831240743135	0.505211867917536	0.477218989328633	0.862050948520352
Usp6nl	Usp6nl	0.0218052565265924	6.17311511093906	0.504868757927092	0.477368608614087	0.862050948520352
Sema3a	Sema3a	0.0461693054229995	4.58164209625716	0.504784938933824	0.477405272904679	0.862050948520352
Gtf3c1	Gtf3c1	0.0264664765763074	7.72353085571891	0.504617343235211	0.477478297139143	0.862050948520352
Med11	Med11	-0.0456274058213822	3.60384821832998	0.50458446820392	0.477492632684971	0.862050948520352
Taok3	Taok3	0.0260888224452084	6.18443035301792	0.504238418893547	0.477643257616862	0.862101076509499
Cbfb	Cbfb	-0.0223840925410874	5.22352039620903	0.504139787828844	0.477686763254044	0.862101076509499
RGD1309	RGD130910	0.029448074573301	4.72597929866774	0.503887899562554	0.477796863448288	0.862101076509499
Pacsin3	Pacsin3	0.0531337526711717	4.42156326702075	0.503813674150479	0.477829202716325	0.862101076509499
Mark2	Mark2	0.0299934967545503	6.28054592345905	0.503642428465221	0.477904023648771	0.862101076509499
Iqub	Iqub	0.0687142953237519	2.43397326707254	0.503479781752816	0.477975113738294	0.862101076509499
Zfp958	Zfp958	0.106851847489484	4.25809681971666	0.503403568905226	0.478008437702755	0.862101076509499
Vps16	Vps16	-0.0187210504228766	6.16478757278686	0.503206174772309	0.478094734261763	0.862101076509499
Smim20	Smim20	-0.0495489105250433	3.87543985561477	0.503105110889067	0.478138920589784	0.862101076509499
Parpbp	Parpbp	0.0930001837537491	0.378400194813833	0.502107556515021	0.478575541070299	0.862514081941703
Folh1	Folh1	-0.0765355791635526	1.44550759584873	0.501989078699005	0.478627436708052	0.862514081941703
Pigm	Pigm	0.0315049580022973	4.01820099619281	0.501756291535766	0.478725055465077	0.862514081941703
Cd164	Cd164	0.0289469581233501	5.89713730960701	0.501723800782715	0.478743764429357	0.862514081941703
Ddb1	Ddb1	0.018328820887762	8.52071579160522	0.501640648829614	0.478780121051939	0.862514081941703
Limk2	Limk2	-0.0171420410331988	7.33700346428656	0.501634621754931	0.478782762805822	0.862514081941703
Coro1b	Coro1b	0.0229348546631624	6.43186382405122	0.500971421467412	0.479073598747674	0.862834328158119
Dennd1c	Dennd1c	-0.0630208517493741	2.22568806770958	0.500633223708384	0.479222021264031	0.862834328158119
Ficd	Ficd	0.0362505890080958	3.36007788879763	0.500309829861231	0.479363877351119	0.862834328158119
Eri1	Eri1	0.0249897954489058	4.8920524674182	0.500292409911758	0.479371667988133	0.862834328158119
Vti1a	Vti1a	0.0263780516033814	4.73708379157459	0.500103875231837	0.479454480870339	0.862834328158119
Grip2	Grip2	0.0455945467092591	6.6100775419051	0.500067354341994	0.479470528770888	0.862834328158119
Senp7	Senp7	0.0257369052891796	5.92370358132009	0.499815619764902	0.47958114846134	0.862834328158119
Foxj2	Foxj2	0.0262256848735086	6.17024221063344	0.499650971948462	0.479653522230995	0.862834328158119
Cela2a	Cela2a	0.0821582218597542	0.317406531109162	0.499643770275924	0.479656688257777	0.862834328158119
Inpp4b	Inpp4b	-0.0619167323016994	3.25526799398404	0.499610363679096	0.479671375040961	0.862834328158119
Klhdc10	Klhdc10	0.0247175828570277	6.19330092411453	0.499496985473883	0.47972122581012	0.862834328158119
Polr3k	Polr3k	-0.0522599673254024	5.62204237584678	0.499118026168716	0.479887910364273	0.863009722919434
Zfpm2	Zfpm2	0.066424026667479	5.43846526658732	0.498685745232223	0.480078164200789	0.863197821240587
Zfp706	Zfp706	-0.0213612330669058	7.02179513144059	0.498566037967151	0.4801308711557	0.863197821240587
Adamts1	Adamts14	-0.0962767211888216	0.842192244997859	0.498170364394483	0.480305153214026	0.863386744943294
Pcbp4	Pcbp4	0.0218219672469244	8.53400677675368	0.497997143416878	0.48038149437313	0.863399565042284
Kcnj11	Kcnj11	-0.0316203338147373	3.75262365251534	0.49768063168181	0.480521009086135	0.863524532617188
Tprn	Tprn	-0.0293516843262838	4.27615509678066	0.49751657349379	0.480593355271785	0.863524532617188
Tatdn2	Tatdn2	0.0204118025574534	6.26463602353992	0.497286625489195	0.480694787781126	0.863524532617188
Synpo	Synpo	-0.0539329668020731	4.04136849473155	0.497211686926804	0.480727851531537	0.863524532617188
Rpp25l	Rpp25l	0.0443887859284521	3.06151626035921	0.496990222365952	0.48082558603676	0.863575764640233
Ube2e3	Ube2e3	-0.0198887857584262	7.79399803319262	0.49665365089389	0.480974180790482	0.863718315158729
Rpl11	Rpl11	-0.0287782177634132	7.85751317428693	0.49607518440456	0.481229747224248	0.86379470144222
Ube2h	Ube2h	0.014506906921453	7.88104915967013	0.495976442139401	0.481273390820038	0.86379470144222
Pheta1	Pheta1	-0.0293542473952451	5.32209037582537	0.495758893389366	0.481369561167476	0.86379470144222
Uvrag	Uvrag	-0.027179822635256	4.16491585013141	0.495720277902258	0.481386655246033	0.86379470144222
Kcnh6	Kcnh6	-0.0692679633547408	2.60447140768569	0.49555598843439	0.481459317854035	0.86379470144222
Nrcam	Nrcam	0.0331764953850194	8.64075215104647	0.495462082475356	0.481500858051362	0.86379470144222
Pgm2	Pgm2	-0.0264964500057084	4.79786877866059	0.495461008143913	0.481501334237632	0.86379470144222
Begain	Begain	-0.0294959473039834	7.29005681203198	0.495261508364678	0.481589606779171	0.863828856208124
Sh2d1a	Sh2d1a	0.0567486934097988	3.23416739969889	0.494884916377387	0.481756309487687	0.863976726752669
Txlna	Txlna	0.0275932766345847	5.61367472050847	0.494555722213398	0.481902108699159	0.863976726752669
Mapk8ip1	Mapk8ip1	0.0455910481751267	4.40684229688336	0.494432523215179	0.481956248765418	0.863976726752669
Tnfrsf1a	Tnfrsf1a	0.0448158924341485	4.02159369794358	0.494432440902884	0.481956728226778	0.863976726752669
Kazald1	Kazald1	-0.107926279188909	0.476805886461755	0.493927609015884	0.482180499656223	0.863976726752669
Mks1	Mks1	0.04568970757973	3.11878439179668	0.493860605308441	0.482210212501055	0.863976726752669
Syt9	Syt9	-0.0598122120126627	3.45203102653476	0.493738046358075	0.482264569152185	0.863976726752669
Ccndbp1	Ccndbp1	-0.0379267585395958	5.20125546543465	0.493715078667407	0.482274756773396	0.863976726752669
Socs2	Socs2	-0.0426489330724065	3.63924862940797	0.493606670029877	0.482322847628812	0.863976726752669
Bax	Bax	0.0325772599983547	5.82379694523358	0.493512771153505	0.482364508224501	0.863976726752669
Mfsd9	Mfsd9	0.0330624227294732	4.20732603055689	0.493281159821095	0.4824672937234	0.864036805481105
Stx18	Stx18	-0.0263472997855107	4.85024786723103	0.492969624951712	0.482605604887087	0.864150466730221
Lrrc3	Lrrc3	-0.0368411016785525	3.86422427715678	0.492718867986454	0.482716980078184	0.864175948144367
Lhfpl2	Lhfpl2	0.0557894552261249	4.72403494099971	0.492565226391258	0.482785237362413	0.864175948144367
Arc	Arc	-0.103837110300569	2.97901006173379	0.492405071886779	0.482856414083631	0.864175948144367
Mtss1l	Mtss1l	0.0200821629984086	8.62409447775874	0.49232659164106	0.482891296823157	0.864175948144367
Jam2	Jam2	-0.0331146883594814	5.31460066843274	0.491564055408247	0.483230440533098	0.86465588618905
Pde8b	Pde8b	-0.0824072012030967	3.82737395787441	0.491264117150138	0.48336395203548	0.864722436926197
Bmper	Bmper	0.0308125637152406	4.01878363423662	0.491172860649343	0.483404594183916	0.864722436926197
Shbg	Shbg	-0.0955625930794379	0.574197923696571	0.490177527350696	0.483848124255205	0.865060317973607
Cetn3	Cetn3	-0.0292502420010767	5.28387689596117	0.490049639532934	0.483905162225402	0.865060317973607
Uck1	Uck1	0.0209109741867779	5.94364015812794	0.489999821782469	0.483927918237998	0.865060317973607
Sptbn2	Sptbn2	0.0258462045905775	9.80666209307854	0.489946399545147	0.483951215353116	0.865060317973607
Cpne1	Cpne1	0.034292291263105	4.89753780064028	0.489410294759409	0.484190476412035	0.865060317973607
Txndc12	Txndc12	-0.0283360848366461	5.21157384477086	0.48935869019612	0.484213517584738	0.865060317973607
Mxd4	Mxd4	0.0295859236352572	4.86444566643724	0.489334166985327	0.484224467702294	0.865060317973607
Arhgap26	Arhgap26	-0.0582326295428712	4.07505436524272	0.489293651638349	0.484242559544438	0.865060317973607
Gtf2f2	Gtf2f2	0.0294197722924261	4.4946667590198	0.489257797129345	0.48425857104432	0.865060317973607
Psmd3	Psmd3	-0.0209054368474955	7.64791960749881	0.488947068449896	0.484397368384144	0.865060317973607
Map3k5	Map3k5	0.0464018426849032	4.75757506609846	0.488922226591697	0.484408468743888	0.865060317973607
Galk2	Galk2	-0.0347753032822956	4.34923565828488	0.488779902770716	0.484472067221462	0.865060317973607
Cmas	Cmas	-0.0192106792100482	7.43019023009981	0.488654461871413	0.484528132867919	0.865060317973607
Kif2a	Kif2a	0.0177369318649873	8.00642760958172	0.488508647236685	0.484593317964278	0.865060317973607
Acy1	Acy1	0.0304479065617393	4.84725768517516	0.488376894724177	0.484652184511587	0.865060317973607
Oxa1l	Oxa1l	-0.019896441335176	6.3935177796433	0.488244825095165	0.484711294130261	0.865060317973607
Mbtps2	Mbtps2	0.0270677612047504	5.00700790067614	0.48810894523858	0.484772120162817	0.865060317973607
Nfkbie	Nfkbie	0.031132524241876	4.01648765272229	0.487770715146802	0.484923426157743	0.865183898276187
Rpl6	Rpl6	-0.0281680974961277	8.88886325770596	0.48764420213648	0.484980058070342	0.865183898276187
Cntn4	Cntn4	-0.0357820100116285	5.4058151393987	0.48729373864948	0.485136995093651	0.865328566492455
Mcm7	Mcm7	0.0277976826221647	6.02280891753987	0.487153406573157	0.485199858036596	0.865328566492455
Dctn2	Dctn2	-0.0208277068629726	8.00253130496036	0.486454534016268	0.485513132787019	0.865763521182459
Slc66a1	Slc66a1	0.0506111733553467	2.23084085861186	0.48589471468636	0.485764315854145	0.865926011272109
Ywhae	Ywhae	-0.0246965935830843	10.8431044541425	0.485678569937974	0.485861354503734	0.865926011272109
Pptc7	Pptc7	-0.0214219030057167	5.78192529753355	0.485483494689802	0.485948961501686	0.865926011272109
Armcx1	Armcx1	-0.0313600373193585	4.3325571613285	0.485198411766682	0.486077037284265	0.865926011272109
Msh4	Msh4	-0.177434353986639	-0.76023301104177	0.485155515419891	0.486096313762909	0.865926011272109
Txndc5	Txndc5	0.0261747185807678	6.454220235896	0.485135299448437	0.486105298675068	0.865926011272109
Tmem208	Tmem208	-0.0378679275760257	4.90599045871378	0.485096725804368	0.486122734173221	0.865926011272109
Ppp2r2d	Ppp2r2d	-0.0199388747432185	6.16476121482556	0.484872059601258	0.486223722438331	0.865926011272109
LOC1003	LOC100362	-0.031009298527543	5.32251467307325	0.484771622750948	0.486268880400773	0.865926011272109
Borcs5	Borcs5	-0.0287724259119636	4.4770086503288	0.484706256615262	0.486298273758465	0.865926011272109
Rcor3	Rcor3	-0.0321629351853248	4.5094896707446	0.484207810839422	0.486522507965277	0.866201674070028
Chmp2a	Chmp2a	-0.0224991589206509	6.25436286785181	0.483589000633856	0.486801128592229	0.866393785511798
Usp2	Usp2	-0.0552525748107945	3.05581314018983	0.483171748769436	0.486989146469078	0.866393785511798
Rars2	Rars2	0.036549481181898	3.23723756805821	0.483157941235689	0.486995370340362	0.866393785511798
Macroh2a1	Macroh2a1	-0.0173847166396904	7.20753366861601	0.48312415070054	0.487010600287472	0.866393785511798
Nucks1	Nucks1	-0.0181999755509313	7.08755394677719	0.482992749221175	0.487069842372141	0.866393785511798
Taf1c	Taf1c	0.0542609611311922	4.63895423637523	0.48289398297581	0.487114377293416	0.866393785511798
Clcn3	Clcn3	-0.019765095455269	7.19451050423724	0.482889305117965	0.487116486764868	0.866393785511798
Abcg4	Abcg4	-0.031184197390976	6.16775579161964	0.482701782069682	0.48720106242109	0.866420703510254
Stox1	Stox1	-0.0865544762259476	0.859664168492334	0.482243443780021	0.487407882306412	0.86663798176877
Patl1	Patl1	0.0315916828662355	5.69151761421415	0.481846663987483	0.487587042307207	0.86663798176877
Spdl1	Spdl1	0.0593217613749667	2.00182940301167	0.481837977558811	0.487590965757205	0.86663798176877
Zfp598	Zfp598	0.0309489723935173	6.14427226751595	0.481624343939345	0.487687475299487	0.86663798176877
Lpcat3	Lpcat3	0.0212630432033851	5.58261305261564	0.481531101254134	0.487729608010169	0.86663798176877
Gpr101	Gpr101	-0.149042667628684	1.53083889056265	0.481056019389909	0.487944372236638	0.86663798176877
Trib1	Trib1	0.0430077879446522	3.13743512427009	0.480874361195504	0.488026532663893	0.86663798176877
Atg4b	Atg4b	-0.0159695538685528	6.16198815150084	0.480463813629151	0.488212257937569	0.86663798176877
Lrrtm4	Lrrtm4	-0.0353875495512454	4.62234037521769	0.480332700920021	0.488271200697886	0.86663798176877
Pygo2	Pygo2	0.0235841727859066	6.48294066640783	0.480069165653269	0.488390989096446	0.86663798176877
Plxna3	Plxna3	0.0559068534464545	8.36707779251933	0.480068725907273	0.488391188262127	0.86663798176877
Plekhh2	Plekhh2	0.0704627090383665	3.00944990679565	0.479922937231876	0.488457224960873	0.86663798176877
Tnk2	Tnk2	-0.032886442360067	6.95685632018527	0.479778890975302	0.488522574894892	0.86663798176877
Cct6a	Cct6a	-0.0183184745573473	8.47850330827717	0.479754107299065	0.488533717019481	0.86663798176877
Fut10	Fut10	-0.0640637282598877	1.55845471338881	0.479706853458246	0.488555129963092	0.86663798176877
Ascc1	Ascc1	-0.02085919830135	5.28717275284277	0.479645681707794	0.488582852085722	0.86663798176877
Tmem250	Tmem250	0.0269616088643044	5.20017076040242	0.47951662851618	0.488641345643494	0.86663798176877
H1f0	H1f0	-0.034852750308884	6.90914307084445	0.479406621111289	0.488691211330564	0.86663798176877
Rpl10a	Rpl10a	-0.0322747518571664	7.72025817855935	0.479210598622942	0.488780100727789	0.86663798176877
Strn4	Strn4	-0.0168327524017947	8.32919809755477	0.479169256083352	0.488798850612642	0.86663798176877
Flacc1	Flacc1	0.107814124548976	0.567878580264477	0.478962724654693	0.48889253574834	0.86663798176877
Rbm7	Rbm7	0.0286796297014943	4.52624139088785	0.478940564268582	0.48890259881413	0.86663798176877
Ankx3	Ankx3	-0.035326475116602	5.02178859744832	0.478838011831769	0.488949121448887	0.86663798176877
Phlpp1	Phlpp1	0.0219752500977864	6.79375477334452	0.478658067186302	0.489030788582499	0.86663798176877
Pcdhga1	Pcdhga1	-0.115768859224924	3.22908517691853	0.478144026293094	0.489264201296299	0.86663798176877
Arsi	Arsi	0.12639291796552	0.0476463403840005	0.478036567391058	0.489313019468163	0.86663798176877
Zfp846	Zfp846	-0.0370215313179501	5.56238741183013	0.477725621824817	0.489454326559297	0.86663798176877
Rhbdd1	Rhbdd1	0.0849475635614103	0.780051310631974	0.477685911639163	0.489472377475587	0.86663798176877
Txndc9	Txndc9	-0.0244062708728832	5.99580035164216	0.477608829260305	0.48950741970037	0.86663798176877
Plekho1	Plekho1	0.0246011995788028	5.8168765820359	0.47750143171065	0.489556250285078	0.86663798176877
Rev3l	Rev3l	0.0379870466157073	8.09481013394823	0.477322773131675	0.48963749948608	0.86663798176877
Slc7a8	Slc7a8	0.0349007719589527	5.78216232272483	0.477260496607016	0.489665826445594	0.86663798176877
Tbx19	Tbx19	-0.118801497617952	0.543780531729539	0.477243864875576	0.489673391979369	0.86663798176877
Specc1	Specc1	-0.0375607205984727	4.07480345571172	0.477218894857307	0.489684841926547	0.86663798176877
Scn2b	Scn2b	-0.0452833202480455	3.58702510350901	0.476621076318722	0.489958829739036	0.866962557118357
Cyb5r1	Cyb5r1	0.0369182679520148	4.75601857046204	0.476510461379602	0.490007209489353	0.866962557118357
Cct7	Cct7	-0.0209748249842682	8.52610011358957	0.476294386091979	0.490105618328706	0.8669735529351
Fils1	Fils1	0.0347922859926793	5.78028105536959	0.475929017998852	0.490272113841677	0.8669735529351
Gns	Gns	0.0225496268226113	6.01433153141288	0.475895119472483	0.490287565835668	0.8669735529351
Abcb1b	Abcb1b	0.0729882109518932	1.69516722410925	0.475765890514905	0.490346571019295	0.8669735529351
Septin1	Septin1	-0.0850679147634449	0.83023121730921	0.475078351184504	0.490660119764779	0.8669735529351
Agtpbp1	Agtpbp1	-0.0222005704062545	8.86518982814008	0.474721062139102	0.490823248321721	0.8669735529351
Hexim1	Hexim1	-0.0213067827532182	5.49406033592262	0.474669572273648	0.490846754220063	0.8669735529351
Nfe2l3	Nfe2l3	0.0391447019447076	3.97885489471093	0.474546572064902	0.490902935098041	0.8669735529351
Pagr1	Pagr1	0.0490586308473245	4.58409571380291	0.474461183457493	0.4909419430282	0.8669735529351
Gadd45gi	Gadd45gip1	-0.031505491699529	5.62770832525152	0.474399757181502	0.490970007488723	0.8669735529351
Fam20c	Fam20c	0.0179633041054783	7.3587217342202	0.474342204879086	0.490996304437595	0.8669735529351
Ttk	Ttk	0.0569721664586661	2.81861601560221	0.474308557543125	0.491011679758075	0.8669735529351
Pomp	Pomp	-0.0354059676894496	6.61228581367404	0.474166179213427	0.491076749148536	0.8669735529351
Utp3	Utp3	-0.0247615621823475	5.04055966116749	0.47404250547752	0.491133281889084	0.8669735529351
Myadml2	Myadml2	0.0905190682036764	0.802860646249713	0.473906147758257	0.491195825254003	0.8669735529351
Parva	Parva	-0.0399236879180635	3.71830398515335	0.473853898983395	0.491219608582647	0.8669735529351
Nudcd3	Nudcd3	-0.0189184216148046	6.0080752257326	0.473683040673166	0.491297658319818	0.8669735529351
Brwd1	Brwd1	0.0497157011021234	6.06793172949044	0.473529499395099	0.491367899271253	0.8669735529351
RGD1562	RGD156320	-0.0303090340763791	4.50650692844255	0.473095149210458	0.491566690729889	0.8669735529351
Cyp51	Cyp51	-0.0211082618788503	8.15093739420738	0.473041938871844	0.491591056706136	0.8669735529351
Ica1l	Ica1l	-0.0322826976859775	4.69659832752649	0.47290440319702	0.491654032999889	0.8669735529351
Mettl11b	Mettl11b	-0.0988542580249955	0.507576572605579	0.472670275771804	0.491761275017004	0.8669735529351

Pter	Pter	-0.0708758564308256	2.39961180296349	0.472660780814223	0.491765625005559	0.8669735629851
Slc3a2	Slc3a2	0.0305059107062863	7.55302572507329	0.472572829309635	0.491805876056232	0.8669735629851
Tekt5	Tekt5	0.144082770345438	0.0656108142754129	0.472452693342731	0.491860973673752	0.8669735629851
Mospd3	Mospd3	-0.0295783375892318	5.45975763854337	0.472185294948623	0.491983544861542	0.8669735629851
Wdr5b	Wdr5b	0.0494282872492996	2.085360182362	0.471942602132081	0.492094835642845	0.8669735629851
Masp2	Masp2	0.0518729465560546	4.15088553525634	0.471904048843811	0.49211251876297	0.8669735629851
Tnfaip8	Tnfaip8	-0.0595321160791148	2.15178992483084	0.471886988017033	0.492120344341583	0.8669735629851
Ankrd13c	Ankrd13c	-0.0260617833398099	6.98390141639837	0.471688260413416	0.492211513429837	0.8669735629851
Pdpk1	Pdpk1	0.0273544761072609	5.65887947283924	0.471592759745754	0.492255325762238	0.8669735629851
Cep85	Cep85	0.0448622422382724	4.93667708504994	0.471496485860001	0.492299518510447	0.8669735629851
Foxp1	Foxp1	0.0386928497462456	6.29400901170617	0.471346244586191	0.492368494204913	0.8669735629851
Fam91a1	Fam91a1	-0.0186524668208705	6.58960130213257	0.4712806229509	0.492398611341758	0.8669735629851
Gab1	Gab1	0.0305237867492771	5.14304920461474	0.470944088697445	0.492599104251966	0.8669735629851
Ascc2	Ascc2	-0.0283205277670974	4.58783646594095	0.470798736840401	0.492619941584831	0.8669735629851
Rbbp9	Rbbp9	0.0290205966147082	4.57174071820825	0.470753826713784	0.492636069589316	0.8669735629851
Riok2	Riok2	0.0280578398471784	4.02583377318015	0.470599233934654	0.492711822032549	0.8669735629851
Man2c1	Man2c1	-0.0409068956990321	6.60526209391754	0.470572665802946	0.492723373789596	0.8669735629851
Kcmf1	Kcmf1	-0.0180142078087856	6.56379469622701	0.470114049083319	0.492934709043346	0.86722311967482
Bc1	Bc1	-0.187292729560765	-0.674485366542314	0.469744116390899	0.493104910518102	0.867400249235169
Cap1	Cap1	0.0216896089298937	8.095955840435	0.468919093559172	0.493484856737844	0.867946230267561
Rpl15	Rpl15	-0.0322336658742489	9.04146357481119	0.468472683387176	0.493690645935504	0.868026596741882
Bbof1	Bbof1	-0.0603286657074161	1.83980807605799	0.468141717028089	0.49384332100256	0.868026596741882
Krt77	Krt77	-0.13601446606515	1.08006038854254	0.468116640236019	0.493854880369486	0.868026596741882
Phf10	Phf10	0.0244132133015366	5.59251557861014	0.468084993810798	0.493869482566205	0.868026596741882
Hck	Hck	-0.0921420802333473	0.907977579150374	0.468065660049768	0.493878401079636	0.868026596741882
Adal	Adal	0.0627780675047392	2.62927036808607	0.467561016500881	0.49411135997548	0.868236069189871
Myo5a	Myo5a	0.0367248804542561	8.56343548353445	0.467506022314609	0.49413675781977	0.868236069189871
Irf8	Irf8	0.107527718667014	0.452905402052605	0.466834747089726	0.494446948199408	0.868310953888463
Tomm7	Tomm7	-0.0626679028155679	4.69084097403732	0.466810118821897	0.494458234916193	0.868310953888463
Ube2f	Ube2f	0.0258076294220228	4.34927099672114	0.466630008387668	0.494541621247519	0.868310953888463
Tubg2	Tubg2	-0.026501971767316	4.57587009823862	0.4665596329667	0.494574170523836	0.868310953888463
Coro1a	Coro1a	0.0332681746307863	6.72002325308783	0.466500716223185	0.494601422821073	0.868310953888463
Elp4	Elp4	-0.0314014568829562	4.23491821633818	0.466410012732133	0.494643383216837	0.868310953888463
Btrc	Btrc	0.0228879458649058	6.26268626043905	0.466223405169778	0.494729728662056	0.868310953888463
Pnma3	Pnma3	-0.0589808554235554	5.38841631366342	0.466096256366612	0.494788576276341	0.868310953888463
Txlng	Txlng	-0.0443782369097016	2.72983835715537	0.465954916768275	0.494854005766001	0.868310953888463
Pgap1	Pgap1	0.0502205454281753	6.20463217887768	0.465908911078174	0.494875306008845	0.868310953888463
Ppp2ca	Ppp2ca	-0.0207574342373241	8.27958559065106	0.465649825877858	0.494995705923018	0.868400087570515
Ntrk2	Ntrk2	0.0257498146530736	8.80040809771973	0.46491032102108	0.495338024973616	0.868753391834709
Iba57	Iba57	-0.0395707453714549	2.87275282062514	0.464899211447715	0.495343176935727	0.868753391834709
Gdap1	Gdap1	-0.0226437786467166	6.69371971313226	0.464369502258464	0.495588929392729	0.868753391834709
Pex6	Pex6	0.0247248748508841	6.15221438813968	0.464267225517361	0.495636400145774	0.868753391834709
Mtln	Mtln	0.0521785223058466	3.68917166678876	0.464243053328175	0.495647624259598	0.868753391834709
Atp5mg	Atp5mg	-0.0350188799070491	6.90145328976368	0.464038876183342	0.495742423595371	0.868753391834709
Haus1	Haus1	-0.0312865086378167	4.22574976857482	0.463891674142651	0.49581078837367	0.868753391834709
Fam122a	Fam122a	-0.0296234784476821	4.20892261449237	0.463881004114022	0.495815744452668	0.868753391834709
Alcam	Alcam	0.0245654534619688	7.43780012857696	0.463743142952111	0.495879785038017	0.868753391834709
Bag4	Bag4	0.0185720067272689	5.22288501796367	0.463370917264996	0.49605277004685	0.868753391834709
Eral1	Eral1	0.0347875310822287	4.83829168881823	0.463370466300418	0.496052979684146	0.868753391834709
Atraid	Atraid	-0.0308452919762227	4.65753525170512	0.463208870752652	0.496128109284412	0.868753391834709
Cfl1	Cfl1	-0.0303587921852262	10.5019411566664	0.463197676055465	0.496133314574655	0.868753391834709
Tnc	Tnc	0.0492875146646587	8.05171268746963	0.462978906392223	0.496235058233212	0.868753391834709
Mog	Mog	0.0298261115310207	5.08737683610361	0.462845842158388	0.496296913306883	0.868753391834709
Map7d1	Map7d1	0.0158676235650647	8.36742404063793	0.462815349944599	0.496311146669697	0.868753391834709
Atf7ip	Atf7ip	0.0343973660451916	7.50383163678513	0.462424828075996	0.496492901732916	0.868803873903896
Tfap4	Tfap4	0.0441269463890962	3.83679102105802	0.462358663562839	0.496523706863861	0.868803873903896
Ralyl	Ralyl	-0.0316264902568731	5.6577616381799	0.462304592374565	0.496548883931128	0.868803873903896
Gli2	Gli2	0.0651184879611664	2.84082527717664	0.462113317185214	0.496637964325842	0.868837896929426
Epha2	Epha2	0.0728823878988996	1.57240942050298	0.461943695932793	0.496763582379366	0.868935821862799
Nup107	Nup107	0.0280898525795116	5.05822799406768	0.46150186654031	0.496922909120341	0.868975024290845
Tcf19	Tcf19	0.0477286037730467	3.30700415055125	0.461364087975215	0.49698706073058	0.868975024290845
Gar1	Gar1	0.0397544893300625	4.05482188473524	0.46123276541762	0.497049401550513	0.868975024290845
Dmrtc1a	Dmrtc1a	0.0725575626895124	1.20529645530606	0.461049673243575	0.49713428178551	0.868975024290845
Hbb-b1	Hbb-b1	0.0959451171147083	5.85301004171136	0.461000031118587	0.497156977847352	0.868975024290845
Vps37d	Vps37d	0.0288915047812285	5.77951308076243	0.460899541752255	0.49720387098562	0.868975024290845
Rabgef1	Rabgef1	-0.0246230774325445	5.04952012427222	0.460720826687642	0.497287281785058	0.868990077707587
Coa8	Coa8	-0.0363601817189436	3.97386268094532	0.46017246346765	0.497543383184446	0.869104699957749
Gpr27	Gpr27	0.03562582927306	6.49243597554734	0.460111817725405	0.49757171965667	0.869104699957749
Gmps	Gmps	0.0299314468228196	5.64886999068034	0.459880685140561	0.497679740405501	0.869104699957749
Lipt1	Lipt1	0.0716206889478187	1.05477961971305	0.459698497777275	0.497764914340175	0.869104699957749
Snn	Snn	0.0193465266228532	8.77037140271182	0.459676522882773	0.497775189430255	0.869104699957749
Slc44a5	Slc44a5	-0.0500996398571841	3.82047612623491	0.459547689975309	0.497835319816293	0.869104699957749
Hdhd5	Hdhd5	0.0262888107874285	4.20349705218896	0.459529765570032	0.497843819867897	0.869104699957749
Stk35	Stk35	0.0271230752825311	4.19418299049193	0.459375305399256	0.497916066793115	0.869104699957749
Rai1	Rai1	0.0436945257255643	7.26589935643209	0.459250132612823	0.497974633225154	0.869104699957749
Ints2	Ints2	0.0505450708656087	5.02880034249392	0.458988051702192	0.498097288631179	0.869197184650521
Igsf6	Igsf6	0.145203486363004	0.890526630378756	0.45786406893329	0.498623898784378	0.869829626321902
Cops3	Cops3	0.0190095455564494	6.25907701381414	0.457693846148921	0.498703723982607	0.869829626321902
Fas	Fas	-0.107467844239441	-0.312966076815757	0.45764598374587	0.498726185547853	0.869829626321902
Tox2	Tox2	-0.0594011076157577	4.83605611399241	0.456541100898107	0.499244946480034	0.870712775682329
Dcps	Dcps	0.0294658162927085	4.48098150790348	0.456177126589751	0.499416034021301	0.870814358197077
Fgfbp3	Fgfbp3	-0.052452137012916	5.65005240467304	0.456025737624529	0.499487230921535	0.870814358197077
Zc3h13	Zc3h13	0.0331625852612993	6.50494541696041	0.455971858960481	0.499512572061912	0.870814358197077
Sars1	Sars1	-0.0182218135402384	7.53218837252244	0.455677880110677	0.499650879659829	0.870932783950396
Chst8	Chst8	0.0644057554134654	3.75196861528522	0.45498204678124	0.499978505923098	0.871026617508083
Ethe1	Ethe1	-0.0388521919405439	4.12682167386446	0.454981928998653	0.499978561310697	0.871026617508083
Arhgap24	Arhgap24	0.0620913097777952	2.77756424204926	0.454910184376345	0.500012362282477	0.871026617508083
Rsad2	Rsad2	0.103262340765886	0.790962638225014	0.45486778768559	0.500032238321488	0.871026617508083
Efl1	Efl1	0.0277207832321634	4.63572247175033	0.454823534402863	0.500053190767645	0.871026617508083
Pdp1	Pdp1	-0.0278746505674383	5.61275958565177	0.454225515115269	0.500335125046409	0.871099599947736
Mustn1	Mustn1	0.0724762809003565	2.5773904086697	0.454165570507413	0.500363400621495	0.871099599947736
Nectin1	Nectin1	-0.0214892049976845	7.92763181572664	0.453942291865687	0.500468743934687	0.871099599947736
Pax7	Pax7	0.343946723355594	-0.7899752913832	0.45291336566648	0.500482394129786	0.871099599947736
Cpq	Cpq	-0.0726207155633326	1.96487724424273	0.453857452814841	0.500504056859228	0.871099599947736
Prodh1	Prodh1	0.0483362223053952	3.26192931393942	0.453845419435393	0.500513988200499	0.871099599947736
Grik5	Grik5	-0.0242077615627496	8.42022923861384	0.453355638052798	0.500745707991153	0.871381338717659
Drd2	Drd2	-0.110533231132617	4.17365129410425	0.453060920403303	0.500884944900173	0.871459751303954
Plk2	Plk2	0.0254654446635719	8.05183880510159	0.452797540237199	0.501009432367667	0.871459751303954
Cspg4	Cspg4	0.0507329102987827	5.26110875872346	0.452763376270738	0.501025583928154	0.871459751303954
Ddx1	Ddx1	-0.0151917786383422	7.45984314448883	0.452525103889653	0.501138255618678	0.871459751303954
Itga8	Itga8	-0.0650612764022482	3.58559748340276	0.452521426598793	0.501139994838973	0.871459751303954
Hey1	Hey1	0.0512436905111302	3.26637069697512	0.452129670564318	0.501325328026324	0.871660570658556
Timm23	Timm23	-0.0265024997098429	6.25263030513713	0.451354320683166	0.501692506782741	0.872177434103283
Pdcl	Pdcl	0.0266664470739785	5.09648203628271	0.451106620704458	0.501809905804326	0.87219399878028
Yju2	Yju2	-0.0264645532193482	5.00370794150342	0.450964767635952	0.501877157337481	0.87219399878028
Rab14	Rab14	0.0169218885556937	6.63666301994896	0.450891813169186	0.501911750508841	0.87219399878028
Mir3597-2	Mir3597-2	0.0882229790427772	0.650309113812378	0.450719013149685	0.501993704129539	0.872214934747843
Zdhhc1	Zdhhc1	-0.0306724764485662	3.87183121240618	0.450131066206678	0.502272729180403	0.872578229181828
Nup43	Nup43	0.0372180705538932	3.88030076625189	0.449772119166475	0.502443191195724	0.872704072021851
Dstyk	Dstyk	0.0285611637539486	5.34482211807736	0.449560345686265	0.502543813144493	0.872704072021851
Cyp4f6	Cyp4f6	-0.0668448758995699	2.11228869126697	0.449505348814512	0.502569948950859	0.872704072021851
Srrm3	Srrm3	0.0276838561927058	6.57866031705501	0.449389640727603	0.502624946826082	0.872704072021851
Mrpl35	Mrpl35	-0.0323707866672797	4.48149060593784	0.449070080709703	0.50277688929499	0.872824405468001
Galc	Galc	-0.0383674112011338	3.01620247374002	0.44894967167636	0.502834160976516	0.872824405468001
Fcgbp	Fcgbp	-0.0698689629100291	1.99919865747291	0.448565786686387	0.503016827404872	0.872840701532
Nup93	Nup93	0.0246058258910539	5.38430700226052	0.448552274693422	0.503023258007601	0.872840701532
Hgh1	Hgh1	0.0242861096812541	5.12198111057968	0.448488919807048	0.503053417064728	0.872840701532
Cyp39a1	Cyp39a1	-0.110316468905021	-0.221733296220604	0.448148052365696	0.503215729259809	0.872865562620836
Sft2d3	Sft2d3	0.0381670838592778	3.29676790258196	0.448077128377058	0.503249512650323	0.872865562620836
Sidt2	Sidt2	-0.0208951501737965	6.98192820669733	0.448018127936429	0.503277519419275	0.872865562620836
Ednrb	Ednrb	-0.0524276603709092	5.35681986378311	0.447598664650329	0.503477521614363	0.873090901623684
Tmem86a	Tmem86a	0.0458543201995996	3.49216111992254	0.447417241480778	0.503564023848148	0.87311955608023
Pecam1	Pecam1	0.0426181805885607	4.205224297044	0.44706882821373	0.503730217862067	0.873280358053204
Plcd3	Plcd3	-0.0383372022208538	3.56247910327983	0.446832541067955	0.503842980767115	0.873360498892928
Tbrg1	Tbrg1	0.0274196576525588	4.76730218701252	0.446425176243295	0.504037488105825	0.873576290804192
Antkmt	Antkmt	-0.0327681148806435	5.09475435571993	0.44563015007 32	0.504417464813411	0.874113431732906
RGD1307	RGD13071C	0.0376448126250257	8.02720131848659	0.445407063162648	0.504524175545669	0.874140078808995
Slitrk1	Slitrk1	0.0205671892767482	6.56030797159147	0.44521120958928	0.504617891407435	0.874140078808995
Pxylp1	Pxylp1	0.038148210981199	4.78460680058791	0.44515870025154	0.504643022173695	0.874140078808995
Coro7	Coro7	0.0268219852952118	5.95246123446823	0.444669429700539	0.504877288159256	0.874221590770263
Nnat	Nnat	-0.0420819614059286	11.0546757882816	0.444530009889926	0.504943937953727	0.874221590770263
Tg	Tg	0.10196802812183	-0.001492386182500	0.444314205252304	0.505047888673879	0.874221590770263
RGD1564	RGD15648C	0.0387014522191886	3.90920151456857	0.444073978260207	0.505162645573654	0.874221590770263
Slc4a8	Slc4a8	0.0592581941094371	5.19439627527836	0.444066992566135	0.505165994958051	0.874221590770263
Acss2	Acss2	-0.0277046482559986	5.11561052492508	0.444052044545195	0.505173162114297	0.874221590770263
Stub1	Stub1	-0.0245298814710864	6.37978306478958	0.444036645386724	0.505180545760487	0.874221590770263
Papss2	Papss2	-0.0694580798063856	2.05847758847306	0.443594125590377	0.505397604230224	0.874464542889137
Nipsnap1	Nipsnap1	-0.0191625922875621	7.08642806006075	0.443451670066906	0.505461168912115	0.874464542889137
Fbxl17	Fbxl17	-0.0346541392845454	3.26323916816007	0.443064321847778	0.505647132864549	0.874561407069233
Rpia	Rpia	0.0400022154061884	4.19425032598158	0.442859278253451	0.505745622292551	0.874561407069233
Snx12	Snx12	0.0252201899894478	4.79410746099175	0.442646776739892	0.505847727696933	0.874561407069233
Crlf1	Crlf1	0.104246779337693	4.51726307598315	0.442642460356089	0.505849801955352	0.874561407069233
Ptpmt1	Ptpmt1	-0.0396407007533658	3.97873140784635	0.442605488870385	0.505867570314872	0.874561407069233
Tgds	Tgds	-0.0508840055428884	4.10555387297781	0.442372418450103	0.505979626340548	0.874578278924592
Isyna1	Isyna1	0.0285143991892279	7.58920716780309	0.441809228087223	0.506250508224285	0.874578278924592
Gmeb1	Gmeb1	0.0280636031291556	4.58157500318854	0.441614879610125	0.506344056358921	0.874578278924592
Septin3	Septin3	0.0209432394852438	8.91134573862832	0.441590311283541	0.506355876942304	0.874578278924592
Cage1	Cage1	0.0665520447855013	1.564398544686	0.441556779088895	0.506367205665174	0.874578278924592
Fbxl19	Fbxl19	0.0152441417386404	7.98061145142678	0.441323996239511	0.506484110086315	0.874578278924592
Tmco3	Tmco3	-0.0230291332357191	5.14915514410324	0.441276863123107	0.506506810923615	0.874578278924592
Clip1	Clip1	0.0233545757158244	6.97538624966785	0.441177811747792	0.506554522973031	0.874578278924592
Snrnp27	Snrnp27	0.029156464750652	4.58947923817813	0.441029456150225	0.506625998810307	0.874578278924592
Nhs	Nhs	0.0637222163283234	3.65381533296291	0.440937367583222	0.506670374618388	0.874578278924592
Lysmd2	Lysmd2	-0.0327232719820327	4.40653865510131	0.440852323072136	0.506711361964249	0.874578278924592
Fjx1	Fjx1	0.0301363717136285	7.67687042343645	0.440837571150695	0.506718472256622	0.874578278924592
Psma4	Psma4	-0.0227385391683106	6.97137110306188	0.440360651809087	0.506948435451268	0.874768892083041
Col11a1	Col11a1	-0.0721807972976889	5.17884256987019	0.440319497189478	0.506968287983957	0.874768892083041
Ess2	Ess2	-0.0249798577178131	4.89674598810375	0.440106178815377	0.507071211823698	0.874768892083041
Dpm2	Dpm2	-0.0317662900343754	5.84174491864142	0.43982822494236	0.507205375511648	0.874768892083041
Hacd3	Hacd3	0.0166019722842672	7.50472897379191	0.439747444376096	0.507244378288238	0.874768892083041
Mepce	Mepce	0.0203822913463811	5.45379671274305	0.439739086188308	0.507248414219828	0.874768892083041
Slc2a5	Slc2a5	-0.110516838260471	0.305319222988191	0.439155969118872	0.507530114662466	0.874815403447123
Zranb2	Zranb2	-0.0341030149538609	7.24437202056247	0.439042544702957	0.507584940601063	0.874815403447123
Chuk	Chuk	-0.0586320494106727	5.03247143736884	0.43897850482162	0.507615800048051	0.874815403447123
Spint1	Spint1	-0.0740132712587523	2.72432279740063	0.438901719689078	0.507653025342986	0.874815403447123
Csrp2	Csrp2	0.0352811110596722	5.97586116739035	0.438609308055951	0.507794412749886	0.874815403447123
Nxpe4	Nxpe4	0.0765420409865408	0.465496525370048	0.438420583482007	0.5078857591565	0.874815403447123
Gba2	Gba2	-0.0295277492858132	7.04690045882948	0.438275714075814	0.507955870948641	0.874815403447123
Xab2	Xab2	0.0193996520421602	5.58059083457062	0.438246880318171	0.507969863645336	0.874815403447123
Tram1l1	Tram1l1	-0.0273120435449989	5.63538765660957	0.438223521956272	0.507975471859381	0.874815403447123
Hnrnpu	Hnrnpu	0.0225682648261608	9.57040992389983	0.437991563365969	0.508093438500552	0.874815403447123
Ptch1	Ptch1	0.0454428431403631	5.46346330705398	0.437878028568204	0.508149417383944	0.874815403447123
Siva1	Siva1	-0.0433013479733258	3.13915816811084	0.437811496060796	0.508180648763093	0.874815403447123
Clic5	Clic5	0.0728497223499078	1.98146044723361	0.437732461338541	0.508218935031601	0.874815403447123
Vps45	Vps45	-0.0217626574380016	5.95514145876306	0.437651532021267	0.508258143751559	0.874815403447123
Ctla2a	Ctla2a	0.0879249497181923	0.704702624054745	0.437017499555779	0.508565505221661	0.874954791516646
Sntg2	Sntg2	-0.0714738687260412	1.18770340918161	0.436781761036968	0.508679866297881	0.874954791516646
Mrpl52	Mrpl52	-0.0344295420222816	5.58520191497398	0.436752338018536	0.508694143055831	0.874954791516646
Gabra3	Gabra3	-0.0251694890738191	5.89513686667957	0.436693055186751	0.508722910613783	0.874954791516646
Shh	Shh	-0.0912019355150912	1.42622079187104	0.436653522040552	0.508742091155023	0.874954791516646
Niban3	Niban3	0.119324306024301	3.04012136704685	0.436616880602244	0.508759879173841	0.874954791516646
Dusp4	Dusp4	0.0460184888794911	4.95467689789484	0.436468096875841	0.508835690302872	0.874955868190606
Phlda1	Phlda1	0.0355650080112162	4.86586235242194	0.435912673049492	0.509103860211183	0.874955868190606
Cnbd2	Cnbd2	-0.0650079069709519	1.37521501045001	0.435625906150136	0.509241235148044	0.874955868190606
Armc8	Armc8	-0.0149042052966971	6.19516073325986	0.43557698245268	0.509265019703056	0.874955868190606
Ndst4	Ndst4	-0.123999667235019	2.55546814026238	0.435429566692224	0.509336894416782	0.874955868190606
Ahr	Ahr	0.0433174339563822	3.91376956551059	0.435268396419385	0.509415083438916	0.874955868190606
Ndufs3	Ndufs3	-0.0263867440030423	5.84065878320257	0.435105251420545	0.509494453064474	0.874955868190606
Sytl1	Sytl1	0.137609863720271	-0.277246701629451	0.434859472160085	0.509614062175225	0.874955868190606
Rex1bd	Rex1bd	-0.0356101632418676	4.9908521597968	0.434829699507555	0.509628556563992	0.874955868190606
Kcnj5	Kcnj5	-0.0848098815075409	0.718466133998236	0.434827027453672	0.509629855098891	0.874955868190606
Cdk5rap1	Cdk5rap1	-0.0285062213093347	3.97681102152279	0.434760712768849	0.509662137347708	0.874955868190606
Atp5f1a	Atp5f1a	-0.0227643644339458	9.57366446675338	0.434680758622445	0.509696195129471	0.874955868190606
Slc30a3	Slc30a3	0.0564451305863334	4.13744301905542	0.434644963181796	0.509718490318569	0.874955868190606
Lonp1	Lonp1	-0.0157721964310887	6.88753264853874	0.434596150366573	0.509742262232709	0.874955868190606
Mgmt	Mgmt	0.104621766327783	0.326854255552267	0.434379390281137	0.509847835213796	0.875012141286247
Prdx6	Prdx6	0.030528745096764	5.93943978320345	0.433837562815199	0.510111897585491	0.875012141286247
Jph3	Jph3	-0.0225834485638128	7.86759636064022	0.433834678967521	0.510113303675792	0.875012141286247
Mxi1	Mxi1	-0.027888579694003	5.68428313296701	0.433727045293764	0.510165767892455	0.875012141286247
Slc39a6	Slc39a6	0.0185405404530413	7.05714108430886	0.433429534987233	0.510306029211995	0.875012141286247
Skp1	Skp1	-0.0263870172084687	8.99082940749038	0.433349730233743	0.510349847620362	0.875012141286247
Adgrl2	Adgrl2	0.0369634412546078	7.98649318195294	0.433258850512158	0.510394294800572	0.875012141286247
Smyd4	Smyd4	0.0321829012217079	3.91577141205997	0.433246004450147	0.510400466680724	0.875012141286247
Frzb	Frzb	0.102571027145814	2.05363066870568	0.433232028030983	0.51040679989431	0.875012141286247
Pcdhgc5	Pcdhgc5	-0.106278013224298	2.17180138566477	0.433030782832475	0.510505524362612	0.875023979906603
Qtrt2	Qtrt2	0.0346838041814219	3.82907609289771	0.432932762522796	0.510553384124394	0.875023979906603
Slc30a6	Slc30a6	-0.0312050543011318	3.72512054638886	0.432731780195553	0.510651540789473	0.875042167580393
Brd2	Brd2	0.0145889324550117	8.14790888477941	0.432622665033847	0.510704844627146	0.875042167580393
Rims1	Rims1	-0.0295957681668667	7.06203799469444	0.432469108704822	0.510779874731429	0.875051558015309
Zfp91	Zfp91	-0.0162318820181106	7.22979052348189	0.431978806455978	0.511019669843057	0.875167812050892
Tceal1	Tceal1	-0.0305372577587355	4.78670884486243	0.431787082018175	0.51111335474029	0.875167812050892
RGD1561	RGD156114	-0.0270537728900327	4.94517909999348	0.431782639765771	0.511115528035386	0.875167812050892
Dcaf5	Dcaf5	0.0150956954947726	6.98743631823401	0.431627574574357	0.511191401029324	0.875167812050892
Lrrc73	Lrrc73	-0.0293157629015636	6.38634906892597	0.43128210421551	0.511360018316808	0.875167812050892
Tmem79	Tmem79	-0.066111540502047	0.730689721002733	0.431188436698392	0.511406375962968	0.875167812050892
Zfp763	Zfp763	-0.0315960429828463	4.69465984578677	0.430846790496304	0.511573723334745	0.875167812050892
Ctc1	Ctc1	0.0275845798346942	6.57669414521247	0.430540133085358	0.511724032144083	0.875167812050892
Xpo1	Xpo1	0.0283213162296767	7.46925354978924	0.430460385589284	0.511760186656185	0.875167812050892
Hsd17b7	Hsd17b7	0.036235284444697	3.54830127085338	0.430451654002626	0.511762508583312	0.875167812050892
LOC1001	LOC100125	-0.0597817165761002	1.68759491007028	0.430451476833806	0.511762595450772	0.875167812050892
Capsl	Capsl	0.13575518955122	0.535916041428763	0.43043917480513	0.511772530526996	0.875167812050892
Slc47a2	Slc47a2	-0.0795671338713871	1.06381945025327	0.43033079739083	0.511826698050124	0.875167812050892
Dynlrb1	Dynlrb1	-0.0272994082289034	7.61534741970326	0.430174896425996	0.511903141717061	0.875167812050892
Magi3	Magi3	0.0222066456548911	6.3056507068681	0.429917966877959	0.512029203504498	0.875167812050892
Lpp	Lpp	0.0902462140308095	0.708222738852453	0.429909899517111	0.51203226063558	0.875167812050892
Grin2c	Grin2c	-0.107863411575396	2.12203272880294	0.429856064110481	0.512059583835739	0.875167812050892
Eif3el1	Eif3el1	0.0232542490373072	7.02050550273534	0.429752742606638	0.512110298612131	0.875167812050892
Tmem138	Tmem138	-0.0420156337052625	2.98981411788849	0.429447722760699	0.512260066744197	0.875303869182053
Med9	Med9	0.0324423050978216	4.67456654353849	0.428742947885211	0.512606410022761	0.875488588723758
Abi3	Abi3	0.106862808043733	0.309732225823503	0.428590497207921	0.512681381444233	0.875488588723758
Acot2	Acot2	-0.0380352041627389	3.29783799802478	0.428554722183172	0.512698977464085	0.875488588723758
Chrm5	Chrm5	-0.0958275546609332	1.22263540040032	0.428404144641533	0.512773050842569	0.875488588723758
Tdrd7	Tdrd7	-0.023337581532019	5.30458844057628	0.42839906772524	0.512775548648337	0.875488588723758
Arhgap21	Arhgap21	0.036454684228341	8.32220866834717	0.428336192087457	0.512806494727348	0.875488588723758
Metap1	Metap1	-0.015724630454712	6.39351845802299	0.428229764439801	0.512859348800348	0.875488588723758
Ltk	Ltk	-0.0785627893172385	2.23289496295121	0.427882692418734	0.513029711804576	0.875659605223762
Mgat4c	Mgat4c	-0.0591248846371976	3.75481294298657	0.427610373737451	0.513163838972881	0.875768731214695
Tp73	Tp73	0.0557780725386606	2.87637372285565	0.427163807305448	0.513383916443497	0.875980942583723
Zfp689	Zfp689	0.0224138110915847	4.80845438094978	0.426970882403195	0.51347904560608	0.875980942583723
Copb1	Copb1	0.0157231265730586	6.972763345894	0.426919281924739	0.513504494438687	0.875980942583723
Rnasel	Rnasel	0.0336458141095913	3.93821810363458	0.426788479724905	0.513569014586835	0.875980942583723
Miga2	Miga2	-0.019334814799086	5.84141800802172	0.426388871255298	0.513766214877736	0.876197520917102
Neurl1b	Neurl1b	0.031412281906745	5.40228603498301	0.426244519658795	0.513837482454921	0.876199298700294
Fam229a	Fam229a	-0.0911373675466706	-0.109976420036969	0.426026820054366	0.513944940477829	0.876229932795072
Tspan13	Tspan13	-0.0205889415126769	8.03462474775908	0.425716969807059	0.514098084750022	0.876229932795072
Dpf3	Dpf3	0.0599212880094816	2.32229350049922	0.425628550699187	0.514141784969459	0.876229932795072
Ciita	Ciita	-0.0264539634802224	8.11256182925246	0.425474091780126	0.514218170040084	0.876229932795072
Slc46a1	Slc46a1	-0.0238786758834683	4.90572047632601	0.4254687480067	0.514220783358582	0.876229932795072
Hdgfl2	Hdgfl2	-0.0194659779933606	6.97672124604702	0.42533146365806	0.514288664878217	0.876229932795072
Sec11a	Sec11a	0.0265224041296035	5.4893511386456	0.425213430539074	0.51434704077932	0.876229932795072
Eif2s2	Eif2s2	-0.0226991241596964	7.25585789593399	0.425060124749693	0.514422878801868	0.876239489257633
Naxe	Naxe	-0.0310722806386347	5.42946294502003	0.424396181849815	0.514751546308634	0.876679640089111
Ik	Ik	-0.0159139987367078	6.82983047308062	0.424171655947175	0.514862774870951	0.876749098408604
Lmo7	Lmo7	-0.0347616758016354	6.19080945862093	0.423787404158602	0.515053227709318	0.876854028674831
Emid1	Emid1	-0.0475452051507983	4.63183575343778	0.423578233982773	0.515156904213323	0.877010873771269
Retreg1	Retreg1	-0.0293074483848217	6.35479751465863	0.423254345588603	0.515317626747829	0.877045196922713
Dbn1	Dbn1	0.01931467826443	9.88987750521403	0.423254321765562	0.515317651570122	0.877045196922713
Gtf2h1	Gtf2h1	0.0168309818583062	5.81890990470242	0.422500102366506	0.515692170747808	0.877562904312657
Nap1l1	Nap1l1	-0.0171290774487008	8.01678601420502	0.422213432700453	0.515834645686661	0.877653358559167
Itpa	Itpa	-0.0274162513607147	6.26226625003708	0.422110120429437	0.515886008790008	0.877653358559167
Cacnb4	Cacnb4	0.0423327508468701	5.01373091591487	0.421714787270886	0.516082636638132	0.877660225582836
Hes7	Hes7	0.0391445177970663	4.09257338441766	0.421613648824042	0.516132962166085	0.877660225582836
Morf4l2	Morf4l2	-0.0191649812968139	8.77678802817674	0.421321157340778	0.516278547575183	0.877660225582836
Mesp2	Mesp2	-0.0687880129269092	1.2361226293441	0.421173299647052	0.516352170453809	0.877660225582836
Prss30	Prss30	-0.136392608956161	-0.170558334288819	0.42099315214206	0.516441896243506	0.877660225582836
Carf	Carf	-0.0449011153616684	3.54152806421583	0.420947646765029	0.516464565352081	0.877660225582836
Dxo	Dxo	-0.0308018845385172	3.84243278662192	0.420824053834401	0.516526143600853	0.877660225582836
Tmeff1	Tmeff1	-0.0153288611663688	8.27465228796211	0.420803310052925	0.516536490125364	0.877660225582836
Atp2b2	Atp2b2	-0.0311071608892219	8.1237743335244	0.420790897889668	0.51654261538708	0.877660225582836
Fam117a	Fam117a	0.0601443121625278	1.79397964288834	0.420688857951342	0.516593467715023	0.877660225582836
Agrn	Agrn	0.0377410391948297	9.76881947606353	0.420291482186959	0.516791636151823	0.877701548268022
Atp2b1	Atp2b1	-0.0245659641971459	8.25790229119031	0.41989739191921	0.516988248139148	0.877701548268022
Imp4	Imp4	0.021358238772329	5.59328476013904	0.419867685881177	0.517003073810325	0.877701548268022
Tfpt	Tfpt	0.0358606476102731	4.2915085735112	0.419777707218387	0.517047984875178	0.877701548268022
Abhd5	Abhd5	0.0289672720020905	4.11987928896476	0.419558456445458	0.517157448157813	0.877701548268022
Slc35a4	Slc35a4	0.0210667969752904	6.22104829204012	0.419439488710061	0.517216861059834	0.877701548268022
Cnot3	Cnot3	0.0247726537444192	5.84296124694421	0.419381596705165	0.517245776855298	0.877701548268022
Ip6k1	Ip6k1	0.0202722264430551	6.64389957728822	0.419185122599302	0.517343832516467	0.877701548268022
Aatf	Aatf	0.0256893621043503	4.64651041467897	0.419118346109194	0.517377300531915	0.877701548268022
Sfmbt1	Sfmbt1	-0.030125833759262	5.38534641838113	0.419102091323964	0.517385423571112	0.877701548268022
Ankrd49	Ankrd49	-0.0228289360494161	4.49312667188267	0.419089747917887	0.517391592117624	0.877701548268022
Prlr	Prlr	0.113837377267692	-0.234265855688219	0.418797321161244	0.517537763430573	0.877765749987904
Rps14	Rps14	-0.0370130553768422	8.34968006128746	0.418732583452382	0.517570138868399	0.877765749987904
Pold2	Pold2	0.0276348758417558	4.61456704717579	0.417852159971986	0.518010724932305	0.878148328258538
Lnc001	Lnc001	0.0665049430636064	1.35977763227254	0.417692121373484	0.518090882956271	0.878148328258538
Epor	Epor	-0.102305488583813	0.0350651121558275	0.417644658973501	0.518114659483275	0.878148328258538
Bard1	Bard1	0.0570830330215257	2.00886095334857	0.417640758802513	0.518116613378624	0.878148328258538
Pde4c	Pde4c	-0.107001226324525	-0.133313200670496	0.417578847874296	0.518147631052285	0.878148328258538
Pih1d2	Pih1d2	-0.0500771681677102	2.02221080469822	0.417065867201572	0.518404762308389	0.87826868154978
Rpa1	Rpa1	0.0275973373221636	5.12386705173333	0.416847680023792	0.518514068009067	0.87826868154978
Ppp2r2b	Ppp2r2b	-0.0149740757820371	7.96586262814642	0.416787329703148	0.518544472948665	0.87826868154978
Thoc2	Thoc2	0.0382638824723284	6.75340665445014	0.416760026930788	0.518558171209079	0.87826868154978
Amigo3	Amigo3	0.0512407368251607	1.85942864019641	0.416413960338339	0.518731853758535	0.878290747860395
Slc7a11	Slc7a11	0.0863736020356687	5.62153391743765	0.416303843978657	0.518787128965922	0.878290747860395
Star	Star	0.116867267317459	-0.3626032571404	0.416124222282235	0.5188773449993	0.878290747860395
Cd40	Cd40	0.0991839446504472	0.494086831125489	0.416014686179153	0.518932368965731	0.878290747860395
Clmp	Clmp	0.0198448857227898	7.73756167811842	0.415923398521748	0.518978230243961	0.878290747860395
Ssu72	Ssu72	-0.0283680389430735	5.91760248754211	0.415890664532057	0.51899465768505	0.878290747860395
Pbk	Pbk	-0.0538439473291612	2.79709112150993	0.415579152974331	0.519151244692239	0.878536563139166
Ebf2	Ebf2	-0.105343716898081	1.47188749725213	0.415268838154891	0.519307393469595	0.878635402809012
Pcmtd2	Pcmtd2	0.0299881637560401	6.05311409887221	0.415085479270235	0.519399450933853	0.878635402809012
Mkrn1	Mkrn1	0.0158516993760833	6.98312929024013	0.415042154308866	0.519421337765525	0.878635402809012
Mir3577	Mir3577	0.0577760292968941	1.29483645303573	0.414929325795168	0.519479145130336	0.878635402809012
Dgkq	Dgkq	-0.0455316558011642	6.21555766398663	0.414569523226788	0.51965926037764	0.878635402809012
S100a11	S100a11	0.05050164395167	3.77340454781829	0.414515584895209	0.519686425261586	0.878635402809012
Slc25a10	Slc25a10	-0.0268538272569311	5.19174684826911	0.414483481265215	0.519702594592491	0.878635402809012
Ndufb6	Ndufb6	-0.0421283405880051	5.80028414526664	0.414325268880074	0.51978229346229	0.87865030732272
Fam162a	Fam162a	-0.0264508914653405	4.90635123726523	0.413718408638474	0.520088196170864	0.879029827923276
Rnf152	Rnf152	0.0330534457174865	7.23308437470277	0.413601331038781	0.520147248501847	0.879029827923276
Castor2	Castor2	0.0251028817606252	7.41878039000345	0.413110575144785	0.520394907676511	0.879055353472174
Scarf2	Scarf2	0.0446497954141373	4.50512852081803	0.413015723141697	0.520442798557302	0.879055353472174
Stx5	Stx5	-0.0204762034292122	6.63502512943804	0.412976563298203	0.520461562721655	0.879055353472174
Ago3	Ago3	0.08203271137628708	2.35292579861182	0.41295635571572	0.520472777515288	0.879055353472174
Ins6	Ins6	0.198447062770019	-0.47478426124207	0.41287349980702	0.520514621838296	0.879055353472174
Arl2	Arl2	-0.0274349759392337	5.26493477294605	0.412594808910136	0.520655412409196	0.879158458895994
Zfp157	Zfp157	-0.0277367626728104	5.04004181480466	0.41247373119235	0.520716600034575	0.879158458895994
Cdh20	Cdh20	-0.0409952403249283	3.80480210376387	0.412270395338993	0.520819386033113	0.879171599828241
Eif3c	Eif3c	0.0229935952198868	8.5717075842105	0.412077165336456	0.520917096716204	0.879171599828241
Tmem63c	Tmem63c	0.0430572263165051	5.59842094704143	0.412040229250942	0.520935772824412	0.879171599828241
Haus3	Haus3	-0.0349751169880777	3.80302092014373	0.411709532990072	0.521103086372318	0.879330292673629
Dtnbp1	Dtnbp1	-0.0278572098368168	5.04375155492788	0.411162791918102	0.521379907406102	0.87964982023578
Zfp251	Zfp251	0.0202658966495182	5.49054607306749	0.410730590506574	0.521598918240376	0.87964982023578
Nhlrc2	Nhlrc2	-0.0519054899713367	4.24296003754991	0.410643376828403	0.52164313332249	0.87964982023578
Pip4k2b	Pip4k2b	-0.0171838123120484	7.56803169712113	0.41044909116686	0.521741652407928	0.87964982023578
Naa60	Naa60	-0.0147945241372887	6.7584040916523	0.410349677525713	0.521746426746603	0.87964982023578
Ppcdc	Ppcdc	0.0390549729153574	2.71742952656598	0.410338860175682	0.521797064875253	0.87964982023578
Apex1	Apex1	0.024099746095585	6.36893315860353	0.410228310279493	0.521853648829115	0.87964982023578
Ap2m1	Ap2m1	-0.0226580040421422	9.85260064104325	0.410228307452101	0.521853648263624	0.87964982023578
Psmc3	Psmc3	-0.0211909071273539	7.53061754716412	0.409640387127601	0.522152137868509	0.879787150596881
Lrrn4cl	Lrrn4cl	0.117860353782513	-0.528715426912328	0.409509424049816	0.522218607382713	0.879787150596881
Ogt	Ogt	0.0402566284348434	7.96860336542017	0.409357528598235	0.522295778682224	0.879787150596881
Zfp422	Zfp422	0.0217253049063191	5.98163977751718	0.409207582183309	0.522371979049391	0.879787150596881
Ccdc125	Ccdc125	0.0585075700084778	1.68709475023444	0.408946306307371	0.522503888613955	0.879787150596881
Grin3a	Grin3a	-0.03324155345545	6.655929653954	0.408758810565669	0.522595019859846	0.879787150596881
Dhrs7	Dhrs7	-0.0379250672538153	4.63931365642173	0.408712907602435	0.522623506523238	0.879787150596881
Pip5k1a	Pip5k1a	0.0260228259993043	5.45636097019388	0.4087108747866	0.522624540590538	0.879787150596881
Tpgs1	Tpgs1	-0.0363447964306439	5.95389286035994	0.408639186665417	0.522661009739232	0.879787150596881

**Jade1**	Jade1	0.0287299387058169	5.2018135800075	0.408338707566507	0.522813918343718	0.879787150596881
**Anapc10**	Anapc10	-0.0340615789952333	3.65265408384465	0.408213880452653	0.522826555597558	0.879787150596881
**Plk4**	Plk4	0.0435362876309052	2.98866312152459	0.408052628507604	0.522959567575751	0.879787150596881
**Pnisr**	Pnisr	-0.0657884501413129	7.43722427649233	0.407921105985186	0.523026560180791	0.879787150596881
**Trex1**	Trex1	0.0392674772485031	4.02385697820962	0.407428848950914	0.523277413200961	0.879787150596881
**Wdr82**	Wdr82	-0.0374380227115389	3.60582665560534	0.40742292826403	0.523280431258207	0.879787150596881
**Taf9**	Taf9	-0.0291247917419621	6.67563293012006	0.407285819841306	0.523350340613874	0.879787150596881
**Ptk2b**	Ptk2b	0.0459382272616174	6.79620005124268	0.407184365160745	0.523402080989866	0.879787150596881
**Th**	Th	-0.165295925856253	2.04732882495795	0.407107117601853	0.523441482177077	0.879787150596881
**Nfs1**	Nfs1	-0.0294440996625208	4.87168180475119	0.407008697581844	0.523491690292048	0.879787150596881
**Cfap126**	Cfap126	0.0824343809624606	2.72086077209494	0.406843149252254	0.523576162598307	0.879787150596881
**Zmynd11**	Zmynd11	-0.0167665068911837	8.16401941895182	0.406707469648822	0.523645412188101	0.879787150596881
**Abhd11-a**	Abhd11-as1	0.0457008317088967	2.23110547496761	0.406599325292156	0.523700619685761	0.879787150596881
**Kcnk4**	Kcnk4	-0.0813297472948998	2.43002244119674	0.406590875859976	0.523704923540484	0.879787150596881
**Sdccag8**	Sdccag8	-0.0296808598156896	5.3819102522121	0.406583714523634	0.52370858860768	0.879787150596881
**RGD1564**	RGD156454	-0.0280340881334442	4.50774191145671	0.406453124867923	0.523775271158644	0.879787150596881
**Farp2**	Farp2	-0.0315908103250346	3.70551756213348	0.406430483227297	0.523786823964552	0.879787150596881
**Ilvbl**	Ilvbl	-0.0261641636080087	4.45227625843718	0.406328417377217	0.523838963034722	0.879787150596881
**Ormdl1**	Ormdl1	-0.0464966642024443	2.38945147451568	0.40602752610107	0.523992609389686	0.879826896021766
**Kcnv2**	Kcnv2	0.278274213360253	-0.921965795664406	0.405833178883218	0.524092032021418	0.879875277019409
**Unc50**	Unc50	0.0264425582351282	4.63989040763616	0.405669674208866	0.524175631251114	0.879897221625424
**Calcr**	Calcr	-0.164754476381509	4.43960952323934	0.405237608528608	0.52439665868231	0.880249846680907
**Card10**	Card10	0.0521222365242966	2.22424545319639	0.404980756451586	0.524528132347454	0.880352096648371
**Mettl4**	Mettl4	0.0812425407441022	0.616067124385872	0.404277447589988	0.524888431764052	0.880589643953691
**Fam222b**	Fam222b	0.0258672117858323	5.5291138519506	0.404158239425477	0.524949544754921	0.880589643953691
**B4galt6**	B4galt6	-0.0236821206530831	7.71967738392138	0.404099739607517	0.524979526709811	0.880589643953691
**Phf13**	Phf13	0.0315519388313597	4.81213226802324	0.404071789115257	0.524993872094746	0.880589643953691
**Bckdhb**	Bckdhb	-0.0404301751682337	4.80604388868912	0.403927029320059	0.525068112495244	0.880589643953691
**Tmem263**	Tmem263	0.0307938239148679	3.92251320605698	0.403878499041717	0.525093005542567	0.880589643953691
**Cpd**	Cpd	0.0301549739878846	6.09206223675333	0.403596025373261	0.52523793884741	0.880591201144543
**Car7**	Car7	-0.101277206940128	0.4612138461014	0.403585542792968	0.525243318683177	0.880591201144543
**Ccdc116**	Ccdc116	0.0654406832250726	1.62068037763175	0.403117121376262	0.52548382055266	0.880591201144543
**Prnp**	Prnp	-0.0234898733574329	8.14696422687638	0.403087548338902	0.525499010832728	0.880591201144543
**Rbm46**	Rbm46	0.0640249027328544	1.31359623389	0.403082334572979	0.525501688980431	0.880591201144543
**Ubox5**	Ubox5	0.0275159125153355	4.16278584045263	0.403051518095829	0.525517518932617	0.880591201144543
**Syt4**	Syt4	0.0285309260726906	9.23489166430801	0.402512163011892	0.525794714938535	0.880899794672886
**Cdkn2d**	Cdkn2d	-0.0285973226123656	6.22999732879053	0.402246818007512	0.525931181885262	0.880899794672886
**Grik3**	Grik3	0.0309655825367975	6.43854207787714	0.402229186354432	0.525940252081348	0.880899794672886
**Endog**	Endog	-0.0543581244332203	2.92042918895324	0.402212775992595	0.525948694262871	0.880899794672886
**Ywhaq**	Ywhaq	-0.0211785898669231	9.99123184713084	0.401887789309056	0.526115930839092	0.880899794672886
**Zdhhc18**	Zdhhc18	0.0206287541413787	5.32686994461856	0.401883065127009	0.526118363610599	0.880899794672886
**Qrfpr**	Qrfpr	0.0773712532927234	0.861202713394232	0.401822090800792	0.52614975052924	0.880899794672886
**Zfp410**	Zfp410	0.0190783004798617	5.30880868564048	0.401675809425328	0.526225065801811	0.880899794672886
**Pgam1**	Pgam1	-0.0279539343243258	7.57049493741584	0.401607111775107	0.526260442503941	0.880899794672886
**Il18**	Il18	-0.0981218529979529	-0.00644448929371	0.401520956198319	0.526304151108961	0.880899794672886
**Rnf139**	Rnf139	-0.0220536703644178	5.20349549628657	0.401416214565685	0.526358769646206	0.880899794672886
**Wdr62**	Wdr62	0.0512882694673472	2.8909152855932	0.401190592981266	0.526475024840848	0.880776198034227
**Parvb**	Parvb	0.0295223385207938	5.59656635300301	0.400902525655155	0.526623522813751	0.880906514834048
**Syce2**	Syce2	0.0555677497588512	1.82960340022327	0.400288146794409	0.526940483228588	0.881318553797639
**Tmem47**	Tmem47	-0.0302880442572407	6.18824707967061	0.399779878976614	0.527203061736668	0.881424988568412
**Sstr3**	Sstr3	-0.0547332702935339	2.46717150779355	0.399666068705741	0.527261763209631	0.881424988568412
**Zic5**	Zic5	-0.0681528454316643	3.05444428052814	0.399572756626128	0.527309985622182	0.881424988568412
**Edf1**	Edf1	-0.0398649477534645	6.49611655879213	0.399559751719607	0.527316709998165	0.881424988568412
**Nab2**	Nab2	0.0458721567013462	4.12573556340846	0.399481139947582	0.527357329483305	0.881424988568412
**Fan1**	Fan1	0.0239757624716727	3.55034042525891	0.399026287168077	0.527592551560634	0.881646116760274
**Prom1**	Prom1	0.0287521098908187	4.6611437764161	0.398952032727788	0.527630967079423	0.881646116760274
**Sptlc1**	Sptlc1	-0.0228186446523364	4.80583682766966	0.398720569923135	0.527750748543227	0.881728885534962
**Mphosph**	Mphosph10	-0.0227361443881584	4.61129913812587	0.398510188129976	0.527859662357755	0.881738845225453
**Edem1**	Edem1	0.0339019706143058	6.1341028579877	0.398407129556578	0.527913030021588	0.881738845225453
**Prc1**	Prc1	0.0427242128071343	4.62206999168409	0.39829861194333	0.527968717077772	0.881738845225453
**Emc8**	Emc8	-0.0298161413250189	4.79636746524549	0.398140119001347	0.52805134326866	0.881758811611759
**Bmp8a**	Bmp8a	-0.0860836541264229	0.165521797634505	0.397750773881469	0.528252142864893	0.881792294756236
**Zcchc2**	Zcchc2	0.0211979126508752	6.03468729824297	0.397379636091451	0.528445634068249	0.881792294756236
**Mrrf**	Mrrf	0.0264559454426917	4.5585809894778	0.397260443526904	0.528507480129521	0.881792294756236
**Fah**	Fah	-0.063452147280821	1.84525874934567	0.396861761015131	0.528714440824784	0.881792294756236
**Mpc2**	Mpc2	-0.0282285006508358	6.60014049127784	0.396811052174272	0.528740777478284	0.881792294756236
**Pcdh8**	Pcdh8	0.0261940213768855	7.00546799380676	0.396499580441642	0.528902578858232	0.881792294756236
**Kif22**	Kif22	0.0380115764793042	3.8492243505117	0.396490825421574	0.52890712302294	0.881792294756236
**Akap11**	Akap11	0.0235859682458733	8.1867805003485	0.396290231935147	0.529011381601396	0.881792294756236
**Upp1**	Upp1	-0.127214356574928	-0.081445974600931	0.396200274228619	0.529058146806303	0.881792294756236
**Dusp14**	Dusp14	-0.0594989398932566	2.06959521876391	0.39578326233287	0.529275030514767	0.881792294756236
**Ranbp17**	Ranbp17	0.0399237767409024	2.52868386219501	0.39577144635318	0.529281178210581	0.881792294756236
**Slc17a8**	Slc17a8	-0.0929818124520724	2.04595821122702	0.395685710657432	0.529325789180805	0.881792294756236
**Nup214**	Nup214	0.0375441323297721	6.06752911450291	0.395498527747264	0.529423209760873	0.881792294756236
**Otulinl**	Otulinl	-0.0303363201214199	2.69401724103984	0.395123136693009	0.529618681504478	0.881792294756236
**Actr5**	Actr5	-0.0231559192578649	4.41627219022918	0.395001602847223	0.529681993758074	0.881792294756236
**Prpsap1**	Prpsap1	-0.0205544731095573	6.09334116464971	0.394845823608357	0.529763165795242	0.881792294756236
**Rybp**	Rybp	0.0157989459676263	7.23586969694544	0.394843011209609	0.529764521459795	0.881792294756236
**Aoc3**	Aoc3	-0.128219852961642	-0.0536829592426103	0.394710251617298	0.529833826601514	0.881792294756236
**Ddx55**	Ddx55	0.0359767582942744	4.15722545309115	0.394671763913351	0.529853889671582	0.881792294756236
**Cfap94**	Cfap94	0.062207536101871	2.17246709207898	0.394652167644637	0.52986410544043	0.881792294756236
**Srp68**	Srp68	0.0149471501944711	6.25368290291939	0.394559657874403	0.529912336665118	0.881792294756236
**Rnf19b**	Rnf19b	-0.0207852288972472	6.23515830682814	0.394400388313116	0.529995260490108	0.881792294756236
**Wnk2**	Wnk2	0.0304725169347549	8.34172554871454	0.39428152309531	0.53005734134825	0.881792294756236
**Arl13b**	Arl13b	-0.0407220009558232	4.71347086525541	0.394271217040114	0.530062768847121	0.881792294756236
**Spn**	Spn	0.106439784372888	-0.181267773379731	0.394163613134609	0.530118909144726	0.881792294756236
**Hsd17b8**	Hsd17b8	0.0309436524198998	4.31946608354	0.393961174961319	0.530224554503649	0.881792294756236
**Magohb**	Magohb	0.0434765127251995	2.87984618313978	0.393821404829758	0.530297517699212	0.881792294756236
**Fdft1**	Fdft1	0.0170878067087544	7.87471840508083	0.393304246505688	0.530567643146129	0.881792294756236
**Sorbs3**	Sorbs3	0.0314339274947065	4.37113409498386	0.393220520799409	0.530611393239157	0.881792294756236
**Thbs4**	Thbs4	-0.0902445698485249	0.442138534263868	0.393096311631851	0.530676322773745	0.881792294756236
**Cmpk2**	Cmpk2	-0.0298530765789931	4.40254669427069	0.392968817609679	0.530743314271393	0.881792294756236
**Pip4p1**	Pip4p1	-0.0261570185022049	6.28417210915356	0.392871075438975	0.530794090329041	0.881792294756236
**Clcn4**	Clcn4	-0.0153411921286761	7.27541798303596	0.392854804503145	0.530802599611862	0.881792294756236
**Fxn**	Fxn	-0.0234983209204875	4.4207452191941	0.392772499211816	0.530845648933669	0.881792294756236
**Lgals3**	Lgals3	0.097107272660555	0.852268052979024	0.392715708891203	0.530875353075559	0.881792294756236
**Tmem65**	Tmem65	-0.0161755977090326	7.18047919422338	0.392675182975381	0.530896553286904	0.881792294756236
**Polg2**	Polg2	0.0401404093904138	2.42015159223946	0.392573519969119	0.530949743033448	0.881792294756236
**Arl11**	Arl11	0.104878349526114	0.245022417415791	0.39249552260671	0.530990499854093	0.881792294756236
**Cabp4**	Cabp4	0.105485896206227	1.96816980476663	0.39241898702161	0.531030612235348	0.881792294756236
**Ctps1**	Ctps1	0.0150027578889647	7.26951469754893	0.392396880742942	0.531042193196173	0.881792294756236
**Tcta**	Tcta	-0.0262567753257329	5.2097718783857	0.392340104788197	0.531071901147855	0.881792294756236
**Lpar4**	Lpar4	0.0463461637531811	2.49620070165225	0.392330720460749	0.531076808270066	0.881792294756236
**Camsap2**	Camsap2	0.0227769901904578	8.23546310815429	0.3922658521732	0.531110771426875	0.881792294756236
**Iqcal**	Iqcal	-0.0880756878638782	0.501850737521702	0.391971606293367	0.531264858202201	0.881930765970804
**Spice1**	Spice1	0.0336730175949374	3.90420582528481	0.391809584117489	0.531349728063002	0.881954327765341
**Cda**	Cda	-0.144857580777339	-0.403785307318688	0.391545326227643	0.531488229228676	0.881989452677863
**Tor1b**	Tor1b	0.0247318862700978	4.51225880302827	0.391499450589949	0.531512278166615	0.881989452677863
**Selenov**	Selenov	0.12234077482483	-0.736849893636819	0.391327089963227	0.531602650613608	0.8820221105992
**Ak2**	Ak2	0.0197714237931416	5.69726732162539	0.39109511714161	0.531724322762707	0.882106694631072
**Cacul1**	Cacul1	-0.0161542848380337	6.13135987445406	0.390797055695458	0.531880732786257	0.882248857082442
**Mcm2**	Mcm2	0.0324658082974912	4.46754001455759	0.390602964432732	0.531969928846523	0.882274540903638
**Smoc2**	Smoc2	0.0869981876791719	1.2190877127612	0.390237390350734	0.532174645710857	0.882469801727772
**Kcnj14**	Kcnj14	0.0449396054149719	3.74273336779243	0.390140470205623	0.532225573896087	0.882469801727772
**Kcns2**	Kcns2	-0.0434740355208345	3.16897324344617	0.389985889986328	0.532306918657148	0.882469337547865
**Slc7a6os**	Slc7a6os	-0.0220473186664779	5.24881671541691	0.389779561753443	0.532415296183765	0.88254891065296
**Snai1**	Snai1	-0.0335540581287347	4.47267212010347	0.389597764487362	0.53251090957318	0.882590145954379
**Cyhr1**	Cyhr1	0.0318708961036406	3.71806252967539	0.389212280710495	0.532713751248111	0.882744381285852
**Slc39a12**	Slc39a12	-0.175160715478558	1.44507912654152	0.388876434295469	0.532890587510295	0.882744381285852
**Tnfrsf19**	Tnfrsf19	0.0387449925509691	4.11712515227481	0.388794377317765	0.532933809837485	0.882744381285852
**Nprl2**	Nprl2	0.0340746996432216	4.93466305892192	0.388784168430433	0.532939187664418	0.882744381285852
**Prr22**	Prr22	0.0801757084905485	1.42539352808293	0.388642525255648	0.533013813427175	0.882744381285852
**Hsdl1**	Hsdl1	0.0191231890416796	6.65323472855579	0.388095266932211	0.533302313227738	0.882744381285852
**Slc31a2**	Slc31a2	0.0469407711705605	2.00194965273987	0.387803279697401	0.533456357074269	0.882744381285852
**Tppp3**	Tppp3	-0.0556682935173323	2.9335728888625	0.387592567575493	0.533567046667657	0.882744381285852
**Hif1an**	Hif1an	0.0263429705162729	4.25761200893478	0.387501111048124	0.533615857097727	0.882744381285852
**Snx18**	Snx18	-0.0228930250221229	5.36275476478472	0.387464150889897	0.53363537247644	0.882744381285852
**Rpl7**	Rpl7	-0.0286746511288917	8.93829002534976	0.387211214347147	0.53376896048246	0.882744381285852
**Rabgap1l**	Rabgap1l	0.0333635586539841	4.68066372379828	0.387079601562476	0.533838495494241	0.882744381285852
**Hunk**	Hunk	0.0445145545583326	3.87967812555046	0.386911965054068	0.533927086575529	0.882744381285852
**Tcn2**	Tcn2	0.0302749451874076	4.50270549951643	0.386841059808211	0.533964566098899	0.882744381285852
**Rufy1**	Rufy1	0.0234922940096966	5.02451925821876	0.386752648475593	0.534011305679865	0.882744381285852
**Ube2n**	Ube2n	-0.0189143978185576	6.78810796550122	0.386710283921275	0.534033704934292	0.882744381285852
**Spag9**	Spag9	0.0192080454890369	8.86016134562248	0.386685896446602	0.534046546862236	0.882744381285852
**Kifap3**	Kifap3	-0.0157526307457269	8.92689147899534	0.386574743112739	0.534105379646368	0.882744381285852
**Rpl27**	Rpl27	-0.0330870320549816	7.91057251356041	0.38649966548283	0.534145088695037	0.882744381285852
**Igsf8**	Igsf8	-0.0271996190532436	6.54194151330936	0.386495445809472	0.534147320650675	0.882744381285852
**Hnrnpa2b**	Hnrnpa2b1	-0.0234511472865249	9.26146980737316	0.386375791206007	0.534210618200471	0.882744381285852
**Mybl2**	Mybl2	0.0500732372574858	3.10383604853831	0.386281722484756	0.534241341715387	0.882744381285852
**Dhx30**	Dhx30	0.0186549962426165	7.61040032440419	0.386280381557697	0.534261100020326	0.882744381285852
**Chmp6**	Chmp6	-0.0260297558672556	5.89652834690022	0.386175897187485	0.534316393136411	0.882744381285852
**Cldn9**	Cldn9	-0.110510175107581	0.873032008069484	0.386034258312222	0.534391364969338	0.882744381285852
**Ctdsp1**	Ctdsp1	0.0369505244316395	4.83909050668347	0.385634234961827	0.534603207033372	0.882744381285852
**Ems2**	Ems2	-0.0457054922027968	3.42724854825476	0.385507990159894	0.534670094635246	0.882744381285852
**Capn10**	Capn10	0.0192055392123735	6.07728445330637	0.385469922841737	0.534700865223975	0.882744381285852
**Ero1a**	Ero1a	-0.0270675682347147	4.78398567791694	0.385426483294978	0.534713287038897	0.882744381285852
**Rnf113a2**	Rnf113a2	0.0345729620855089	3.50664670854259	0.385294236347669	0.534783281268283	0.882744381285852
**Klf9**	Klf9	-0.0438280174279483	4.69685796452792	0.385268153027333	0.534797209115954	0.882744381285852
**Rnf10**	Rnf10	0.0169247709117677	8.64337640066299	0.384943772300701	0.534969216711529	0.882911496945734
**Ppp1r12b**	Ppp1r12b	0.0395762472129028	6.94094244166044	0.384785250569621	0.535053312195652	0.88293349772056
**Amelx**	Amelx	0.158181778257159	-0.620947236769842	0.384642738383391	0.535128935124946	0.882941513297269
**Slc26a1**	Slc26a1	0.0423358801201521	2.71219559831418	0.38442059306383	0.535246853420822	0.883019303203865
**Optc**	Optc	-0.0864196110026699	0.583768816310447	0.384146657102107	0.535392327981952	0.883142527264783
**Aspg**	Aspg	-0.117890717369225	-0.006574307268429	0.38394982721006	0.535496899453077	0.883198257035828
**Septin10**	Septin10	-0.0314282259891758	3.41295000322899	0.383785818617413	0.535584008860187	0.883225175584574
**Selenow**	Selenow	-0.0353175119316512	9.00531056326594	0.383283155956997	0.535091717811668	0.883872743484295
**Mtg2**	Mtg2	-0.0224986962237517	5.03220972764788	0.383269505821173	0.535916440675633	0.883872743484295
**Dot1l**	Dot1l	0.0643711976521362	6.66335193315982	0.382505825318426	0.536265149473175	0.883872743484295
**Mir6315**	Mir6315	-0.0365312717730146	2.8556117846255	0.382393545324952	0.536325027017748	0.883872743484295
**Nceh1**	Nceh1	-0.0361071947917689	4.61179043070707	0.382382528759614	0.53633089211506	0.883872743484295
**Pou6f2**	Pou6f2	0.0885585469434237	0.540216943220534	0.381812440474818	0.536634841543985	0.884081550098074
**Usp34**	Usp34	0.0374673274463264	8.08129580868825	0.381674514190479	0.536708424419471	0.884081550098074
**Flii**	Flii	0.0245452994949394	6.91647731721872	0.381656675030115	0.53671794284887	0.884081550098074
**Fastk**	Fastk	-0.0216537237205009	6.17621012208903	0.381552616305747	0.536773471527913	0.884081550098074
**Cd46**	Cd46	0.0848805520385596	1.8520209625687	0.381480652176862	0.536811876742166	0.884081550098074
**Dusp10**	Dusp10	-0.0526356558269319	1.93908546579903	0.381330472376241	0.536892048825029	0.884096884412021
**Cnih3**	Cnih3	-0.0435784273051245	2.87754716670322	0.380975847155981	0.537081388251843	0.884291961100323
**Inf1**	Inf1	-0.0861817906586377	3.04586430133214	0.380287849881455	0.537449224228826	0.884780827934169
**Pnrc2**	Pnrc2	0.0238832729862286	5.72980799585657	0.379697270237273	0.537765296720089	0.885184380894005
**Dpf1**	Dpf1	0.0185449869842764	8.14445247832551	0.379527562213958	0.537856185537185	0.885217204451584
**Myh2**	Myh2	0.0862050584534605	1.12539206292656	0.37917928070388	0.538042798989789	0.885407544578687
**Sord**	Sord	0.0313882723859089	4.74084624809611	0.378942369488584	0.538169806649329	0.885414056092879
**Six3**	Six3	-0.135954765848803	2.9445822192616	0.378907165927515	0.538188683654628	0.885414056092879
**Spc25**	Spc25	-0.0497813077107964	2.53986122151459	0.37858372751498	0.53836217227958	0.885459794229443
**Ror2**	Ror2	0.0436607323015888	3.33151056564576	0.378343371795495	0.538491171846913	0.885459794229443
**Trim15**	Trim15	0.0445479512605515	3.0282198843119	0.378244304344072	0.538544355842388	0.885459794229443
**Tmem175**	Tmem175	0.0279872782444702	5.03476080676446	0.377987894944612	0.538676681461138	0.885459794229443
**Mas1**	Mas1	-0.0835138516236376	1.92524364283878	0.377984006069312	0.538684141769187	0.885459794229443
**Gss**	Gss	0.028792133170918	3.72707960840058	0.377963381727014	0.538695220332438	0.885459794229443
**Aldh7a1**	Aldh7a1	-0.0313019157906129	5.25259851133192	0.377918481750868	0.538719340225672	0.885459794229443
**Mpig6b**	Mpig6b	0.152778600983448	-0.00396314216125	0.377481911119958	0.538953965211694	0.885647728699496
**Eif3d**	Eif3d	0.0188047023307993	6.90621651971721	0.377330565170539	0.539035346621907	0.885647728699496
**Celf5**	Celf5	0.0319301880428779	9.03976972128807	0.377004812647129	0.539210584993398	0.885647728699496
**Mtx1**	Mtx1	-0.0189250519181884	5.8612444827171	0.376921007128971	0.539255685032235	0.885647728699496
**Tprg1l**	Tprg1l	-0.0278529120794089	6.91092227192954	0.376880352944547	0.539288330458974	0.885647728699496
**Kif27**	Kif27	0.0450400293565297	3.37356119185614	0.376647377212606	0.539402987379752	0.885647728699496
**Insig2**	Insig2	0.0290944839301505	3.30574228790963	0.37647953749174	0.539493376470088	0.885647728699496
**Man2b2**	Man2b2	-0.0753788895012074	4.61303059982323	0.376347951303956	0.539564260831387	0.885647728699496
**Gmeb2**	Gmeb2	0.0290616994258506	3.79979819239993	0.37627693328983	0.539803524718998	0.885647728699496
**Kif19**	Kif19	-0.0461036671011678	3.12521131822821	0.376085198191063	0.539705854746593	0.885647728699496
**Pnkp**	Pnkp	-0.0256952184622959	4.06925656615759	0.376061094523955	0.539718847278101	0.885647728699496
**Trim23**	Trim23	-0.0180738244397768	6.63820052900597	0.375979748973123	0.539762700048808	0.885647728699496
**Hint1**	Hint1	-0.0301808779103782	7.28348661818172	0.375941624063543	0.539783253561656	0.885647728699496
**Myo1e**	Myo1e	0.0445282153843627	3.45761781629961	0.375832099735476	0.539842310278113	0.885647728699496
**Gucy1b1**	Gucy1b1	-0.0219104832480522	7.49210032337058	0.375491053391528	0.540026292060195	0.885647728699496
**Lin37**	Lin37	0.0296111977590119	3.89363564371443	0.375457161382304	0.540039174514448	0.885647728699496
**Pkhd1l1**	Pkhd1l1	-0.110714335240555	0.16767905883307	0.375295228619099	0.540131960302184	0.885647728699496
**Ptgfrn**	Ptgfrn	-0.0176785198485165	7.5764589824591	0.375120550059652	0.540226273917164	0.885647728699496
**Ddx46**	Ddx46	-0.0327480964619326	6.90701817740271	0.375067181928259	0.540255092714477	0.885647728699496
**Nol8**	Nol8	0.0271123403860175	4.8860441381847	0.374957080571281	0.540314582777751	0.885647728699496
**Ust**	Ust	0.0571203638490329	2.74209246096502	0.374950272355903	0.540318223116339	0.885647728699496
**Zfp282**	Zfp282	0.0138558222023316	6.43307491830523	0.374698854478379	0.540453524102986	0.885854387074598
**Pcgf6**	Pcgf6	0.0543653472105501	4.28451144554172	0.374679997921959	0.540464254483128	0.885854387074598
**Ndufa9**	Ndufa9	-0.0198888569095144	6.64642493721943	0.374547995551339	0.540535597817479	0.885854977540209
**Adamts5**	Adamts5	-0.0581854600176231	1.85383905265091	0.374101579184195	0.54077700116323	0.885915670467178
**Decr1**	Decr1	-0.0497990639622655	3.5656169856775	0.373902600958374	0.540884653277979	0.885915670467178
**Chodl**	Chodl	-0.0509885534950588	3.84706526564992	0.373690397465992	0.540999525208899	0.885915670467178
**Dexi2**	Dexi2	0.0252763415562299	4.56200404152073	0.373647783571265	0.541022596742172	0.885915670467178
**Upf2**	Upf2	0.0267487322301996	5.25598385324398	0.373582844404096	0.541058047624471	0.885915670467178
**Metrn**	Metrn	-0.0506114474273672	4.87759629658429	0.373392390839723	0.541160904858616	0.885915670467178
**C2cd2**	C2cd2	0.0278865645500159	4.66005889012917	0.373097198352205	0.5413208510241	0.885915670467178
**Il27ra**	Il27ra	0.096354198093319	0.124927268953662	0.373075111833472	0.541332821685266	0.885915670467178
**Erich2**	Erich2	-0.09238605724943	0.980431949897477	0.373073417875944	0.541333739812596	0.885915670467178
**Tmsu1ap**	Trnau1ap	0.0315509414309866	5.45779499522258	0.372552707107189	0.541616101501674	0.886177785716717
**Traf3ip2**	Traf3ip2	0.0594282246645586	1.34053252560283	0.372516116599222	0.541635953328912	0.886177785716717
**Mageh1**	Mageh1	-0.0251223104815342	6.09135168822267	0.371844064705966	0.542000817665606	0.886659476729221
**Mtpn**	Mtpn	-0.0150536358890369	8.21375419615196	0.371423460489474	0.542229380829723	0.886890253585301
**Zfp105**	Zfp105	0.0350752136670153	3.9355759371322	0.371225458274747	0.542337044471136	0.886890253585301
**Polr2j**	Polr2j	-0.0446962130162583	4.42846655234351	0.370951144200731	0.542486268034233	0.886890253585301
**Kcnq5**	Kcnq5	-0.0232620722585782	5.99627086557065	0.370628834470425	0.542661644431148	0.886890253585301
**Cenpi**	Cenpi	-0.0499469365233052	2.47082175521687	0.370551874900211	0.542698157391666	0.886890253585301
**Zmym1**	Zmym1	0.0300545094952468	4.23879904891991	0.370340218801644	0.54281887814058	0.886890253585301
**Kcnk2**	Kcnk2	-0.0219486850486876	5.92820276708451	0.37025687093815	0.542864284766177	0.886890253585301
**Mrpl16**	Mrpl16	0.0272654068753057	4.00759514480256	0.370240726339816	0.542873080903003	0.886890253585301
**Pdzrn3**	Pdzrn3	0.0348914411333026	6.07090036112682	0.369990405556682	0.543009497978022	0.886890253585301
**Pcdhb9**	Pcdhb9	0.0366819855987783	3.32869979755979	0.36978338719338	0.543122364348042	0.886890253585301
**Kcnq2**	Kcnq2	0.0288301422684443	7.56223704125863	0.369724373733092	0.543154546398731	0.886890253585301
**Gtf3c3**	Gtf3c3	0.0323605400417902	4.87334330447527	0.369685562029574	0.543175713662138	0.886890253585301
**Polr1b**	Polr1b	0.0338109302995191	4.44613913998419	0.36951759076901	0.543267339956273	0.886890253585301
**Sema4b**	Sema4b	0.0177526901342911	5.7729155243043	0.369370123264986	0.543347805236219	0.886890253585301
**Baiap2l1**	Baiap2l1	0.0671936967066859	1.33299728808308	0.369236485412898	0.543420743289716	0.886890253585301
**Vax1**	Vax1	-0.0964088088832305	1.69118918057564	0.368988840590475	0.543555953154579	0.886890253585301
**Hsp90b1**	Hsp90b1	0.0256734495110458	8.64019198591834	0.368946356887161	0.543632774900538	0.886890253585301
**Ddhd2**	Ddhd2	-0.0593532604069607	5.29956517441144	0.368713472890136	0.543706372081939	0.886890253585301
**Fat2**	Fat2	-0.202170771824856	0.0702146392017855	0.368611732364883	0.543761967043362	0.886890253585301
**Rassf6**	Rassf6	-0.0845930300108101	0.350234559119154	0.368538676928324	0.543801893839321	0.886890253585301
**Daw1**	Daw1	-0.144890560041926	0.19740573119868	0.368512925745471	0.543815963290738	0.886890253585301
**Planp**	Planp	-0.0204880940697296	6.91385538835262	0.368484142697504	0.543831701819778	0.886890253585301
**Usp39**	Usp39	-0.0202568355930462	5.31742782725619	0.368384687266449	0.543886071147038	0.886890253585301
**Map4k6**	Map4k6	-0.0153481997399967	5.86646547800638	0.368206139935992	0.543983703867604	0.886890253585301
**Cav2**	Cav2	-0.0489612757786585	3.41663922694672	0.368033961261672	0.544077893755663	0.886890253585301
**Adat2**	Adat2	0.0420298107475735	2.74808829224051	0.36773676123766	0.544240519017184	0.886890253585301
**Cgrrf1**	Cgrrf1	-0.0413790157788858	2.7097137587915	0.36770282107014	0.544259092335642	0.886890253585301
**Arhgef7**	Arhgef7	0.0141159582433783	8.21758747298725	0.367514449980707	0.544362223403365	0.886890253585301
**Itga3**	Itga3	0.0200235027544981	6.54573482145548	0.367418534512971	0.544414761417435	0.886890253585301
**Cep57l1**	Cep57l1	-0.0294156011164841	3.48566810114099	0.367237562543465	0.544513897114543	0.886890253585301
**Mfap1a**	Mfap1a	0.0212548001133101	5.7302558642324	0.367090071094232	0.544594717024803	0.886890253585301
**Chordc1**	Chordc1	0.025090013653454	5.42012245415394	0.367041337140257	0.544621426324761	0.886890253585301
**Hsf2**	Hsf2	0.0205505331890171	6.21593511591388	0.367011076768286	0.544638012149129	0.886890253585301
**Commd10**	Commd10	-0.026760248974922	5.21984040530418	0.366796193189955	0.54475581754419	0.886890253585301
**Babam2**	Babam2	-0.0213788701436866	5.14930982842341	0.366690992388307	0.544813508866007	0.886890253585301
**Enoph1**	Enoph1	-0.0201382750177468	5.99953458484401	0.366666830068255	0.544826760896243	0.886890253585301
**Terf2ip**	Terf2ip	-0.020431430741797	6.63691236736786	0.366571416793064	0.544879098951145	0.886890253585301
**Ap2a2**	Ap2a2	-0.0172631107478049	8.67232936797793	0.366518203465631	0.544908288549416	0.886890253585301
**Suxd2**	Suxd2	0.0853478521966209	1.05861250393987	0.36622140368334	0.545071165749072	0.886890253585301
**Psmb2**	Psmb2	-0.0265095602537989	6.38027599042132	0.366150459106208	0.545110111688021	0.886890253585301
**Scn8a**	Scn8a	0.0402900019824072	6.71992412314141	0.366102243170488	0.545136582388785	0.886890253585301
**Map2k7**	Map2k7	0.0294575833830055	4.68563895883009	0.366019825129643	0.545181832887584	0.886890253585301
**Prr13**	Prr13	0.0294206032675091	4.77464365940774	0.365820366937228	0.545291387847086	0.886890253585301
**Myh6**	Myh6	0.0901529763750823	1.73911023398162	0.365770994445313	0.545318651258044	0.886890253585301
**Suclg1**	Suclg1	-0.0242345589506949	6.50036624516859	0.365613108301218	0.545405264818404	0.886890253585301
**Kirrel2**	Kirrel2	0.0944188362892303	1.76260803590888	0.365103769423726	0.545685205275739	0.886890253585301
**B3gntl1**	B3gntl1	-0.0818911363365411	1.79103391791769	0.364820297130166	0.54584127717179	0.886890253585301
**Zfp503**	Zfp503	-0.102105768397504	2.9744177197611	0.36481126226577	0.545846246968687	0.886890253585301
**Scara3**	Scara3	0.0398004372305296	3.94358496584668	0.364727175066097	0.545887028207757	0.886890253585301
**Lnc215**	Lnc215	0.102806160272083	6.28102454721083	0.364454332082175	0.546042760516324	0.886890253585301
**Tmem132**	Tmem132b	-0.0288026136398176	6.16771407822955	0.364431965901559	0.546055078770866	0.886890253585301
**Zfp414**	Zfp414	0.0227070448827291	5.24330356440407	0.36434989149308	0.54607549877647	0.886890253585301
**Eif3k**	Eif3k	0.0241942328287776	5.31865745587174	0.364217887257103	0.546172611653405	0.886890253585301
**Tnn**	Tnn	-0.0775212762557096	1.34764922226765	0.363933157858327	0.546329933196692	0.886890253585301
**Usp51**	Usp51	-0.0653288136750479	1.25541679465209	0.363915384587719	0.546339788536841	0.886890253585301
**Cyp26b1**	Cyp26b1	-0.0480900512470517	5.39775297492802	0.363756083107478	0.546422052572659	0.886890253585301
**Mynn**	Mynn	0.0231871218694373	4.92365572253674	0.363714145553978	0.546450695064162	0.886890253585301
**Exoc7**	Exoc7	0.0146111181556988	6.21723200999741	0.363677550204237	0.546470878920995	0.886890253585301
**Slbp**	Slbp	-0.0204998054319689	5.56565728282783	0.363396527554698	0.546625915551803	0.886890253585301
**Hdgf**	Hdgf	0.0196079102240599	7.06021041843959	0.363312592388397	0.546672237548834	0.886890253585301
**Nudt2**	Nudt2	0.0323413999073139	3.76604663809356	0.36314477599655	0.546764882501766	0.886890253585301
**Gnb4**	Gnb4	-0.0305995017640509	6.94478641247357	0.363103861608203	0.546787407958071	0.886890253585301
**Psmd11**	Psmd11	-0.0143756768347286	7.04097165925721	0.363086218501365	0.546797204762724	0.886890253585301
**Efhd2**	Efhd2	-0.0250592434228273	5.99113720740768	0.362975835684279	0.54685827700972	0.886890253585301
**Gdi2**	Gdi2	-0.0184391312988473	8.18652988872279	0.362967402584574	0.546862816479235	0.886890253585301
**Socs5**	Socs5	-0.0157921178157698	6.22344205176871	0.362923853658982	0.546942106841302	0.886890253585301
**Mtf2**	Mtf2	0.0262615961582526	5.15853168007206	0.362764336538923	0.546974987622762	0.886890253585301
**Dhx15**	Dhx15	0.0173953142929934	7.34830922611554	0.362694650731974	0.547013490800986	0.886890253585301
**Lhx6**	Lhx6	0.0424125683238655	5.94247792123352	0.362623724926738	0.547047158098299	0.886890253585301
**Slc25a15**	Slc25a15	0.0424189977983184	2.62187093489783	0.362432062952	0.547158622666045	0.886916048703946
**B3galnt2**	B3galnt2	0.0511777085751	4.18665793840444	0.362060040240323	0.547364361967657	0.886916048703946
**Dhx36**	Dhx36	-0.0250041758575589	7.34659892066426	0.362018755716619	0.54738702023606	0.886916048703946
**Dpp8**	Dpp8	-0.0168676455940683	8.09889423318697	0.36179894331792	0.547508841599109	0.886916048703946
**Rngtt**	Rngtt	0.0172339493722962	6.13377309298736	0.361662812323339	0.547595272246328	0.886916048703946
**Camk4**	Camk4	-0.040708951757182	5.21916920808178	0.361364898039277	0.547749132817064	0.886916048703946
**Nat1**	Nat1	0.10117469089216	0.0341866637696193	0.360917989959497	0.547996844327501	0.886916048703946
**Zfp385d**	Zfp385d	-0.0716821526078587	2.2630748989021	0.36088909435968	0.54801286945462	0.886916048703946
**Morf4l1**	Morf4l1	-0.0215035738899366	8.48480153098892	0.36088869727727	0.548013089557863	0.886916048703946
**Mid32**	Mid32	0.0308535172098655	3.96484108919957	0.360101283723819	0.548449906483533	0.886916048703946
**Sec24p**	Sec24p	0.0175576835371795	6.00883670858172	0.360012755164245	0.548499153149772	0.886916048703946
**Rab3c**	Rab3c	0.0365469640926895	5.42105225132379	0.359969868913639	0.548674578273869	0.886916048703946
**RGD1560**	RGD156045	-0.0980741109644186	-0.437805584576459	0.359661909344254	0.548694062256332	0.886916048703946
**Ehmt2**	Ehmt2	0.0184110368218684	8.43559383370181	0.359582454047761	0.548738221304371	0.886916048703946
**P2ry13**	P2ry13	0.123655326523678	-0.251751071590097	0.359462809907299	0.54880473078522	0.886916048703946
**Shmt1**	Shmt1	0.0483765473885441	2.12759341956656	0.35940178716853	0.548838655406858	0.886916048703946
**Chaf1b**	Chaf1b	0.0421130191290503	2.4496899467286	0.359223348767056	0.548932222520275	0.886916048703946
**Oprk1**	Oprk1	-0.102208063426436	3.20839382903406	0.359180218302748	0.548957424244517	0.886916048703946
**Zfp286a**	Zfp286a	0.0328280099640857	4.58617988505396	0.359174286896041	0.548965217350792	0.886916048703946
**Syvn1**	Syvn1	0.0245487409987472	6.25235791317273	0.359158323241118	0.548974053183962	0.886916048703946
**Dexi1**	Dexi1	-0.0203226997870208	5.770654120928	0.359049258948395	0.549034783029754	0.886916048703946
**Pcdhgb8**	Pcdhgb8	0.0465289415794265	3.34617707916134	0.358969226328014	0.549079268798878	0.886916048703946
**Ifi47**	Ifi47	0.125065929928096	-0.365210487277804	0.358871784173544	0.549133489253576	0.886916048703946
**Timm21**	Timm21	-0.0265408823658745	3.94949758364205	0.358780510264829	0.54918429009681	0.886916048703946
**Txndc11**	Txndc11	-0.016758700738549	5.72537997986504	0.358772679925933	0.549189649173592	0.886916048703946
**Pyroxd2**	Pyroxd2	0.0571212220514636	1.95807374805509	0.358707278322524	0.549225057966883	0.886916048703946
**Xiap**	Xiap	0.0312364010572435	4.47158881097721	0.358675597478829	0.549242698204638	0.886916048703946
**Nbr1**	Nbr1	-0.0150546986957759	7.92819086447335	0.358619892144842	0.549273656910859	0.886916048703946
**Slc9a3**	Slc9a3	-0.154292362687346	-0.611131976390916	0.358529232336181	0.549324198900126	0.886916048703946
**Pgam2**	Pgam2	-0.039227498920294	2.51293939155201	0.358214280709396	0.549499965481601	0.886916048703946
**Ndufs7**	Ndufs7	0.030972944328566	5.69106810090476	0.358178654246227	0.549519341162045	0.886916048703946
**Ppp1r8**	Ppp1r8	-0.0212632214576933	5.53331937656957	0.358129282803624	0.549547023895484	0.886916048703946
**Wnt2b**	Wnt2b	-0.0804876920076947	1.42117089985817	0.358107285269284	0.54955929291493	0.886916048703946
**Brd8**	Brd8	0.0174061233518615	6.40623673034551	0.357947820281257	0.549648171413455	0.886916048703946
**Ncbp2**	Ncbp2	0.0207136605436459	6.33774286487558	0.357757524804857	0.549754294212762	0.886916048703946
**Gtf3a**	Gtf3a	0.0241332105868419	5.32809150381687	0.357726655727272	0.549770720570822	0.886916048703946
**Acot13**	Acot13	-0.0348889422278838	2.96011963698505	0.35731934622981	0.550002742772299	0.886916048703946
**Sdf4**	Sdf4	-0.0199907724290097	5.9758108986838	0.357223[illegible]	0.5500[illegible]	[illegible]
[illegible]	[illegible]	[illegible]	[illegible]	[illegible]	[illegible]	[illegible]

**Abcb1**	Abcb1	-0.037134848525429	3.67891996353793	0.355036065628845	0.551276086566185	0.887452699069477
**Dpyd**	Dpyd	0.0453765544864196	3.29548553176792	0.355016883437395	0.551286794868937	0.887452699069477
**Ccdc28b**	Ccdc28b	-0.0279634987350671	5.27867296887467	0.354943480143984	0.551384076206813	0.887452699069477
**Meis1**	Meis1	-0.0781245322132188	3.77544360788807	0.354697852817619	0.551471371082115	0.887452699069477
**Icam4**	Icam4	-0.0846500486016566	0.362725756401498	0.354409307204548	0.55162767838827	0.887452699069477
**Tdrkh**	Tdrkh	0.0166769010758731	5.91223170799113	0.354179626018998	0.551756622148388	0.887452699069477
**Commd1**	Commd1	-0.0289398857828831	5.15867781468785	0.353986629807167	0.551865024964069	0.887452699069477
**Kctd21**	Kctd21	-0.0455026831220435	1.85766129270725	0.353759450537716	0.551992672512628	0.887452699069477
**Large2**	Large2	-0.0674796401228183	2.12404854201228	0.353669756158129	0.552043647392548	0.887452699069477
**Samd8**	Samd8	-0.0183765671015609	6.11373634134861	0.353621594495415	0.552070158155795	0.887452699069477
**Snip1**	Snip1	0.0214574412923887	4.99651217754922	0.353523107796022	0.552125527737359	0.887452699069477
**Mybbp1a**	Mybbp1a	0.015920803703332	6.85434226337632	0.353254314624934	0.552276897380229	0.887452699069477
**Cnih2**	Cnih2	0.0324556927715571	9.2632152221793	0.353234142568397	0.552288045211465	0.887452699069477
**Asb14**	Asb14	-0.0925026309045109	0.770857586223098	0.353084335736327	0.552372233913026	0.887452699069477
**Cpsf4**	Cpsf4	-0.0298367725778605	4.97533113801565	0.352891191552857	0.552481041926758	0.887452699069477
**Arhgap11a**	Arhgap11a	-0.0384626398358567	4.04413538172505	0.35285712793619	0.55250021824569	0.887452699069477
**Tanc1**	Tanc1	0.0395787813902932	3.46013730508974	0.3528180769786	0.552522203759054	0.887452699069477
**Adprs**	Adprs	-0.0236011456545229	6.25091490997326	0.352779372131382	0.552544558098861	0.887452699069477
**Adam23**	Adam23	0.0290932863658185	6.50372132152504	0.352771852264547	0.552548173885797	0.887452699069477
**Rgs17**	Rgs17	-0.0334536211824787	5.03216490499098	0.352708979143017	0.552583634073242	0.887452699069477
**Ccdc60**	Ccdc60	-0.138957431002728	-0.263636219782147	0.352704137770957	0.55258636042885	0.887452699069477
**Pclaf**	Pclaf	0.0492028516978428	2.45692909801763	0.352185268587384	0.552878701574086	0.887698078346924
**Ephb6**	Ephb6	0.0269834354844682	7.32752352471573	0.352167625021693	0.552888647529173	0.887698078346924
**Fam83f**	Fam83f	0.0832311974237157	0.885082627390214	0.352054207873394	0.552952589958507	0.887698078346924
**Pik3r5**	Pik3r5	-0.109295436560112	-0.160798854925063	0.351817858618915	0.553085883928912	0.887777940169923
**Pcdha1**	Pcdha1	-0.04504163258249	3.10297758628036	0.351713705003533	0.553144642683034	0.887777940169923
**RGD1310**	RGD131055	0.0843864191432399	0.34876128617685	0.351372483926816	0.553337226617499	0.887972807267722
**Smarce1**	Smarce1	0.0154385532130837	8.08985129196572	0.350894191146296	0.55372042813718	0.888244503348157
**Atoh8**	Atoh8	0.0686757166254723	0.862908329466683	0.350624754843217	0.553759684416131	0.888244503348157
**Snap47**	Snap47	-0.0280866804272125	4.54347694522088	0.350346329996186	0.553917140860958	0.888244503348157
**Phb2**	Phb2	-0.0248467113337842	7.1484600377629	0.350223794928638	0.553986472530058	0.888244503348157
**Actn3**	Actn3	-0.100595682012504	-0.104131989266819	0.350131140181162	0.554038904185883	0.888244503348157
**Melk**	Melk	0.0501689832125677	2.2382555686367	0.349879332611224	0.554181444894883	0.888244503348157
**Tm7sf2**	Tm7sf2	0.0286437997204553	5.3276130644106	0.349859390392254	0.554192736545287	0.888244503348157
**Sf3b5**	Sf3b5	0.0366886524884074	5.56337029671004	0.349707992293247	0.554278475098156	0.888244503348157
**Mtch1**	Mtch1	-0.0234176298059604	9.46448065328595	0.349665598905232	0.554302493134059	0.888244503348157
**Ptk2**	Ptk2	0.0153967965101645	7.524656623775	0.349499563882763	0.554396551582378	0.888244503348157
**Tmem252**	Tmem252	0.102219888180908	0.466756368773912	0.349430435361327	0.554435723983584	0.888244503348157
**Crlf2**	Crlf2	0.0378522429733264	3.52593740493238	0.349072596265053	0.554638590853208	0.888244503348157
**Grik5**	Grik5	0.0283119036774695	4.36764739004894	0.349056721490026	0.554641906727688	0.888244503348157
**RGD1563**	RGD156304	0.0522215265313457	1.5170039099254	0.348988829659469	0.554680087907824	0.888244503348157
**Ddx51**	Ddx51	-0.0278342094370431	5.01570238770878	0.348817707750129	0.554783161206126	0.888244503348157
**Sgsm3**	Sgsm3	-0.0143602574698689	6.21540423429005	0.348620343703644	0.554895160920853	0.888244503348157
**Taf9b**	Taf9b	-0.0238098819393176	5.02915094823808	0.348529379736494	0.554941118542443	0.888244503348157
**Galm**	Galm	0.063505413191309	2.07684020897839	0.34851076753823	0.554957361421841	0.888244503348157
**Cacybp**	Cacybp	-0.0184424360868732	7.18492794198312	0.348500529073142	0.554963173921882	0.888244503348157
**Wdfy2**	Wdfy2	0.0395112611294895	4.88382061241487	0.348443883021318	0.554995334649154	0.888244503348157
**Plscr4**	Plscr4	0.0746813107733978	1.28625542673542	0.348432963372176	0.555001534571875	0.888244503348157
**Farsa**	Farsa	-0.0168210036778108	5.90752785029469	0.348250067909959	0.555105299614089	0.888296788917274
**Ost4**	Ost4	0.0374498516055165	5.47198712437429	0.347805061260715	0.555358269171034	0.888480788608821
**Zeb1**	Zeb1	-0.0308967556099154	6.43205450944488	0.347797089202061	0.555362801182992	0.888480788608821
**Lmx1b**	Lmx1b	-0.344001481316982	0.0573845695850213	0.34725066488329	0.55567360328876	0.888802565087225
**Mycbp**	Mycbp	0.0306189958995646	3.32036337753984	0.347042634150981	0.555792016223216	0.888802565087225
**Psmc2**	Psmc2	-0.0215798982874203	7.0061898073851	0.346870123085523	0.555890247314567	0.888802565087225
**Pou2f1**	Pou2f1	0.0692342900937763	3.01996067628172	0.346816082524498	0.555921025808229	0.888802565087225
**Capn3**	Capn3	-0.102194982190651	-0.520054090336943	0.346275671988441	0.556228991301704	0.888802565087225
**Rbfa**	Rbfa	-0.024318211020133	4.74992356450422	0.346112002636033	0.556322325920348	0.888802565087225
**Cx3cr1**	Cx3cr1	0.101281591828589	1.81298275662745	0.346046218793802	0.556359848298049	0.888802565087225
**Nrxn1**	Nrxn1	0.0284495931967379	8.54365635986599	0.345983780309695	0.556395467152398	0.888802565087225
**Dusp2**	Dusp2	-0.0428384562487987	3.8425693731896	0.345942475035267	0.556419032580896	0.888802565087225
**Eif4g2**	Eif4g2	-0.0179085089504937	10.8084570082349	0.345812799873855	0.556493027069567	0.888802565087225
**Itga7**	Itga7	-0.0384247452320484	3.5761650820904	0.345797377965582	0.55650182828917	0.888802565087225
**Grm5**	Grm5	-0.0278773082540712	7.36617437345925	0.345730823273479	0.556539807993807	0.888802565087225
**Pdzd2**	Pdzd2	0.0325244501990992	6.65262608368355	0.345522706809918	0.556658625814456	0.888802565087225
**Dars**	Dars	0.0189350535740611	6.34643741277888	0.345495568784571	0.556674122320325	0.888802565087225
**Ptgis**	Ptgis	0.0603113294559842	1.27355034696962	0.345201033350222	0.556842361652652	0.888802565087225
**Tacc1**	Tacc1	-0.0244956594565323	6.139953206	0.345182399305514	0.556853008708859	0.888802565087225
**Nr3c2**	Nr3c2	0.0317985372192185	4.45274051581072	0.345162257583851	0.556864517653178	0.888802565087225
**Oma1**	Oma1	0.0609628890224809	1.49354197673761	0.345040771596743	0.556933944106601	0.888802565087225
**Ift122**	Ift122	-0.0251706792741643	5.94943852540054	0.344982335590743	0.556967344798623	0.888802565087225
**Mir22**	Mir22	-0.0607507621975215	1.33805902053539	0.344876773567826	0.557027691295831	0.888802565087225
**Mettl27**	Mettl27	0.0743885892635461	1.30812066668182	0.344800829656685	0.557071108012181	0.888802565087225
**Jpt1**	Jpt1	-0.0256609480306499	8.92587043276315	0.344616670723482	0.557176436956408	0.888802565087225
**Nmd3**	Nmd3	0.0208046872234796	4.73478456489581	0.344373691588856	0.557315463965304	0.888802565087225
**Bet1l**	Bet1l	0.0247605125828118	4.7079576026841	0.344322889501242	0.557344533152374	0.888802565087225
**Esrrb**	Esrrb	0.195168670501036	0.800215449626501	0.344298090984847	0.557358725100716	0.888802565087225
**Pole**	Pole	0.045568820814801	3.96415350091278	0.344292847229781	0.557361728218669	0.888802565087225
**Slc25a28**	Slc25a28	-0.0326520582561621	5.45220331892217	0.344292852400483	0.557361826734922	0.888802565087225
**Rusf1**	Rusf1	-0.0277744206261526	4.94976467707546	0.343965234621649	0.557549006946578	0.888787875498244
**Kif5a**	Kif5a	-0.0226385568860035	9.22613104892238	0.343768808358298	0.557661830067868	0.888853686972062
**Fgf18**	Fgf18	0.0590841551668534	1.36207063267714	0.34264345257805	0.557733664020546	0.888854633529861
**Asic1**	Asic1	-0.0171998407622123	7.56260747145825	0.34218662961396	0.557995588891509	0.889035662449071
**Nae1**	Nae1	-0.0185111068097473	5.6392298218619	0.342185652939795	0.557996249130036	0.889035662449071
**Acot5**	Acot5	0.0826708915438741	0.0067265251877711	0.342060665790702	0.558067852929165	0.889035662449071
**Kbtbd4**	Kbtbd4	0.020595047227673	4.30937280068187	0.342948404572889	0.558132277085533	0.889035662449071
**Pbx3**	Pbx3	-0.0765280167059072	5.99206132295726	0.342497601531354	0.558391060135899	0.889334344819136
**Eif2b3**	Eif2b3	0.0235399452805845	4.87209109624889	0.34229837315165	0.558505552267658	0.889346596309871
**Rgs8**	Rgs8	-0.0526277944603555	5.77000071110403	0.342120518209541	0.558607768571954	0.889346596309871
**Crb2**	Crb2	0.0404479516881704	2.94383396828059	0.3421121428661	0.558612592905446	0.889346596309871
**Pigt**	Pigt	0.0160924099012191	6.91467059657341	0.341924583397336	0.55872041697838	0.88938373088596
**Arfgef2**	Arfgef2	0.0277093310210588	5.60081273626778	0.341823624970544	0.558778477712194	0.88938373088596
**Timp2**	Timp2	-0.0186004272651324	8.40894390031048	0.341484517325405	0.558973581241135	0.889580787391025
**Snx30**	Snx30	-0.0244225858576183	5.32392327387349	0.341309506163171	0.559074323699008	0.889627541473349
**Cox11**	Cox11	0.0235804284114245	4.56062673991317	0.341103026677231	0.55919322496637	0.889687288572612
**Slc35d2**	Slc35d2	-0.0723285335696031	0.155732212575007	0.34058189657091	0.559493532635286	0.889687288572612
**Ssr3**	Ssr3	-0.0166860340749146	7.59527906392201	0.340550614597127	0.559511569057108	0.889687288572612
**Tfpi**	Tfpi	0.0451289995498573	4.06443629519567	0.340416368161627	0.559588984882962	0.889687288572612
**Rps3**	Rps3	-0.0279325720001818	8.70403534287702	0.340352545083815	0.559625220064593	0.889687288572612
**Trpc4**	Trpc4	0.0370877170256075	5.2938000355167	0.340219587776545	0.559702499116643	0.889687288572612
**Yy2**	Yy2	-0.0340715491445547	2.98043391152745	0.340045854322078	0.55980275513132	0.889687288572612
**Fbxl12**	Fbxl12	-0.031423107826101	3.06524585048095	0.339974026453349	0.559844208950401	0.889687288572612
**Pelp1**	Pelp1	-0.0167514800537239	7.04564347732428	0.339948321579467	0.559859226380657	0.889687288572612
**Tubb3**	Tubb3	-0.0228052422464219	11.8335752347889	0.339887183526471	0.55989434895789	0.889687288572612
**Hp**	Hp	0.0822618824229646	1.16723397801459	0.339813456769861	0.559936917991829	0.889687288572612
**Adcy1**	Adcy1	0.0263698823098444	8.41812027825196	0.339632106139033	0.560041078585606	0.889687288572612
**Pttg1ip**	Pttg1ip	-0.0241643806795204	6.08275817051542	0.339529596471863	0.560100873656356	0.889687288572612
**Rpl36**	Rpl36	-0.0390852139761509	7.85388892733056	0.339489615570784	0.560123973712553	0.889687288572612
**Dcaf13**	Dcaf13	0.0219077025030127	5.15588296580226	0.339390235939064	0.560181400899771	0.889687288572612
**Slc19a1**	Slc19a1	0.0303385932211843	4.24226400870368	0.339096871273426	0.56035098835297	0.889843361862626
**Wdr26**	Wdr26	0.0164606748571689	7.54646767542925	0.338749218317282	0.560552088050018	0.889867952907934
**Ripor2**	Ripor2	0.024367061252105	6.09137501242322	0.338708852657047	0.560575562120846	0.889867952907934
**Tsfm**	Tsfm	-0.0290648893664728	4.57712217719048	0.338617276360736	0.560628445602263	0.889867952907934
**Med10**	Med10	-0.0299161083627765	4.80175482815461	0.33853304768427	0.560677200898526	0.889867952907934
**Il1rapl2**	Il1rapl2	0.0616877977660749	1.08494165170087	0.338379991215575	0.560765817570433	0.889867952907934
**Aco1**	Aco1	0.0136763631858517	6.93435855942812	0.338330626332365	0.560794398790882	0.889867952907934
**Cckar**	Cckar	0.0661232476502485	0.842675953817728	0.338103587197814	0.560925918010355	0.889913301616486
**Jph1**	Jph1	0.0425380591511733	2.80420584193928	0.337936368845057	0.561022817774976	0.889913301616486
**Ccdc12**	Ccdc12	-0.03578236788445	3.73529570891521	0.337911984975435	0.561036950430014	0.889913301616486
**Rgs12**	Rgs12	0.0153713472035478	6.76053775245756	0.337679243637598	0.561172458274024	0.889951542015893
**Mcee**	Mcee	-0.0559796018175042	2.42650396413941	0.337624348188555	0.561203713439212	0.889951542015893
**Uri1**	Uri1	0.0204180185625625	5.86052783875142	0.337455374411604	0.561301723576892	0.889993849925833
**Sdc4**	Sdc4	-0.0449752529324182	3.21604833033323	0.336978200688733	0.561578676741003	0.890280308259102
**Pcid2**	Pcid2	-0.0211938124771139	4.90176575750069	0.336896295324522	0.56162624123782	0.890280308259102
**Abo**	Abo	-0.0463239574244281	2.11591908406044	0.336754974555731	0.561708327929214	0.890280308259102
**Dhodh**	Dhodh	0.0290516411311317	3.71741390192927	0.336527591681772	0.561840452442937	0.890280308259102
**Atp7b**	Atp7b	0.0443432854616102	2.38108506376111	0.33648081827851	0.561867538297489	0.890280308259102
**Dnajc7**	Dnajc7	-0.0140437177610706	7.43196689298983	0.336310373064215	0.561966726582568	0.890280308259102
**Ccdc47**	Ccdc47	-0.0141912716729904	7.76609030900545	0.336247374061081	0.562003358535022	0.890280308259102
**Gcsh**	Gcsh	-0.0257187673405222	5.03755577264088	0.336097022923553	0.562090804946346	0.890280308259102
**Stxbp2**	Stxbp2	-0.0505318803448957	2.76721675455129	0.335921791205166	0.562192754083976	0.890280308259102
**Ddias**	Ddias	0.0674959732418251	0.826212330079958	0.33591634179821	0.562195925097408	0.890280308259102
**Mfsd5**	Mfsd5	-0.0236998604147445	5.44113875238235	0.335331504578299	0.562536442144046	0.89040451760163
**Large1**	Large1	0.0141347090368963	7.78807581821454	0.335314338787214	0.562546442784658	0.89040451760163
**Crppa**	Crppa	0.0377613452148971	2.6155280619338	0.335310029070662	0.562548953641725	0.89040451760163
**Eef1b2**	Eef1b2	-0.0222306892010979	7.65231882157295	0.335148750496515	0.562642930639044	0.89040451760163
**Eif2ak4**	Eif2ak4	0.0374606897670676	5.57223743340008	0.335074777028661	0.562686048995361	0.89040451760163
**Tmem120b**	Tmem120b	0.0380296880972473	3.49746755362307	0.335031987252336	0.562710987403653	0.89040451760163
**Yif1a**	Yif1a	-0.0372584579825203	3.16682525985774	0.334924063502307	0.562773906051651	0.89040451760163
**Scarb2**	Scarb2	-0.0241738995274555	7.3283107700522	0.334724749747323	0.562890138893587	0.890475500881486
**Sema6d**	Sema6d	-0.029471409239654	6.84400454017221	0.334595629934967	0.562965463276129	0.890481755283504
**Tcf7l2**	Tcf7l2	-0.108330515181798	7.90791966809785	0.334331326717347	0.563119708113089	0.890522785780822
**Engase**	Engase	-0.0313818935595352	4.0011689449881	0.334103585779417	0.563249760698137	0.890522785780822
**Tnpo2**	Tnpo2	0.0135746446379646	8.54928863219488	0.33410593229897	0.563251310285269	0.890522785780822
**Il13ra1**	Il13ra1	0.0500585091282335	2.2447542826332	0.334062124482905	0.563276895518397	0.890522785780822
**Nphp4**	Nphp4	0.0318532738999455	4.56292796412268	0.333791943014603	0.563434739675625	0.89065847636295
**Gtpbp10**	Gtpbp10	-0.0309181983273812	3.28982814995475	0.333487093435835	0.563612929881532	0.890828306215359
**Wdr13**	Wdr13	0.0174750001238123	7.24128522603596	0.333299601263377	0.563722592506544	0.89088876325708
**Il11ra1**	Il11ra1	0.0413521551530852	3.41277220418045	0.333168360232918	0.563799371819224	0.890897258648994
**Htr1d**	Htr1d	-0.125448740473521	-0.325086889370032	0.332916811908072	0.563945925084882	0.891015836340898
**Mtss1**	Mtss1	-0.0150228580488348	5.48443968578356	0.33271645353701	0.564063902951701	0.891089556486247
**Cfap161**	Cfap161	-0.123556415865846	1.13303039457415	0.332256499724522	0.564333390564675	0.89124199501771
**Pls1**	Pls1	0.096358601730892	0.346607810999174	0.332077948714584	0.564438070825692	0.89124199501771
**Pwwp2b**	Pwwp2b	-0.04198486675499	3.11282890479183	0.332005177925993	0.564480745281917	0.89124199501771
**Tmco1**	Tmco1	-0.0264354184186972	4.29351545339096	0.331966823703992	0.564503351172673	0.89124199501771
**Celf4**	Celf4	-0.0247185669905401	9.23274087167568	0.331942423292851	0.56451755122425	0.89124199501771
**Rpl13**	Rpl13	-0.0326234987204681	8.84776594279082	0.331724809909147	0.564645218247007	0.891298275175656
**Rapgef6**	Rapgef6	-0.0446186658674162	5.07860581432171	0.331411891428756	0.564828836881072	0.891298275175656
**Xpnpep1**	Xpnpep1	-0.0140944154524975	7.12747187336775	0.331368918303632	0.564854129041528	0.891298275175656
**Fnbp1**	Fnbp1	0.0229798575445857	5.86000682262779	0.331332381770622	0.564875584688139	0.891298275175656
**Hipk3**	Hipk3	0.0272075322136948	5.43173101770899	0.331272141984241	0.564910375898781	0.891298275175656
**Hspb8**	Hspb8	-0.0615444782689439	2.66396250562651	0.330932252943256	0.565110658732225	0.891299484021239
**Spata6l**	Spata6l	-0.0733185768275119	0.636137549654689	0.330908626578321	0.565124545037768	0.891299484021239
**Nat9**	Nat9	0.0289908639615907	3.98824243826438	0.330907089840856	0.565125448272182	0.891299484021239
**Serf2**	Serf2	0.0386912845104483	6.06821355730339	0.330709316737625	0.565241714899805	0.891370181598347
**Meis2**	Meis2	-0.0591113279033702	7.84553991005055	0.330428853383877	0.565406613884996	0.891395823876391
**Abcd4**	Abcd4	0.0409456806048499	3.5879493309325	0.330344558204189	0.565456270129794	0.891395823876391
**Rpl10**	Rpl10	-0.024728155235986	9.03823461403049	0.329963742517223	0.565680437983772	0.891395823876391
**Naa38**	Naa38	-0.0364882758735617	4.01265756123609	0.329416172575947	0.566003069198856	0.891395823876391
**Pik3r6**	Pik3r6	0.0638520501772755	0.900462491955659	0.329388499607056	0.566019383683451	0.891395823876391
**Rbpms**	Rbpms	0.0906960976112193	0.938571792015766	0.329383623102871	0.566022258712881	0.891395823876391
**Tpm2**	Tpm2	-0.0653350038270683	2.85827182315473	0.329242886277001	0.56610524450629	0.891395823876391
**Nudt16**	Nudt16	0.0355747233988202	4.73848626968863	0.329072368052917	0.566205232624235	0.891395823876391
**Cacna1b**	Cacna1b	0.0418036225874251	7.7775151187607	0.329048509617891	0.566219897926165	0.891395823876391
**Adgrb2**	Adgrb2	-0.0169982322545883	9.22627740216105	0.328866383542511	0.566327366166252	0.891395823876391
**Apool**	Apool	0.0471486112847994	2.43213105320712	0.328737481787214	0.566403451909375	0.891395823876391
**Nolc1**	Nolc1	0.0137239402734709	7.78887985233281	0.328619515926832	0.566473099986603	0.891395823876391
**Pabpc1**	Pabpc1	-0.018692988131719	8.97870870150403	0.328173031831561	0.566736858590326	0.891395823876391
**Fbxo31**	Fbxo31	-0.0213882441749621	6.26122470164861	0.328155418840652	0.566747268275896	0.891395823876391
**Tal1**	Tal1	0.111187899290612	2.82408810063494	0.328092045634079	0.566784726277369	0.891395823876391
**Fosl2**	Fosl2	0.0374008824692038	4.27949629681208	0.328076884728231	0.56679380649576	0.891395823876391
**Agap3**	Agap3	-0.0160314578620631	8.2646456625831	0.32805704259065	0.566805417741509	0.891395823876391
**Trim41**	Trim41	-0.017702257468083	5.97909480991541	0.327853828382723	0.566925566899166	0.891395823876391
**Traf3ip3**	Traf3ip3	-0.101492350168941	-0.528451016424988	0.327801910763839	0.566956278228859	0.891395823876391
**Mgat3**	Mgat3	-0.0144549775437864	6.87641368330279	0.32750892602599	0.567130801103768	0.891395823876391
**Uimc1**	Uimc1	-0.0231772141722802	5.07203255284037	0.327461315085661	0.567157795274577	0.891395823876391
**Csgalnact1**	Csgalnact1	0.028398265530989	4.33406282096756	0.327358657928173	0.567218560893126	0.891395823876391
**Slc31a1**	Slc31a1	0.032971674939632	4.89782672823601	0.326744852705573	0.567582152680165	0.891395823876391
**Palb2**	Palb2	-0.049556847025161	1.93922545495667	0.326734493261952	0.567588293065854	0.891395823876391
**Etv3**	Etv3	0.0522530062590254	3.01801978016747	0.326586532762306	0.567676008114689	0.891395823876391
**Bicap**	Bicap	-0.0152789564572457	7.70394896111223	0.326476190433022	0.567740253142287	0.891395823876391
**Ppp1r14a**	Ppp1r14a	0.0810786844998952	0.553203994346533	0.32617933765431	0.567917540721322	0.891395823876391
**Pth2r**	Pth2r	-0.0836163245544152	1.36208894037361	0.326095751362175	0.567967145653584	0.891395823876391
**Rbm41**	Rbm41	-0.0419882318343639	3.18470721269348	0.325907496306002	0.568078897957652	0.891395823876391
**Egr2**	Egr2	-0.104683651812285	1.11735833324646	0.325759661345359	0.568166686014744	0.891395823876391
**Mr1**	Mr1	0.0922936823912259	0.393772612193347	0.325650098813528	0.568231645187614	0.891395823876391
**Alox12**	Alox12	0.102197965579853	-0.205997547422853	0.325648865187802	0.568232496928849	0.891395823876391
**Aste1**	Aste1	-0.0407185500875304	2.52988545303268	0.325535106981374	0.568300082481337	0.891395823876391
**Wnt8b**	Wnt8b	-0.204151743144682	-0.268003067416734	0.325520874202681	0.568308528521514	0.891395823876391
**Psd2**	Psd2	0.0152866872822229	7.44597076710618	0.32543594526458	0.568359008896864	0.891395823876391
**Trmt1**	Trmt1	0.0173043641794715	5.53959277282048	0.325364397085698	0.568401533486786	0.891395823876391
**B2m**	B2m	0.037820941935542	6.36444163587667	0.325356265370289	0.568406366942788	0.891395823876391
**Tbkbp1**	Tbkbp1	0.0323279877272606	6.42096562610925	0.325354389075912	0.568407482218052	0.891395823876391
**Wdr70**	Wdr70	0.0201588780100476	4.73882185805531	0.325177500341756	0.568512644583164	0.891395823876391
**Scn4b**	Scn4b	-0.0892676144037805	1.20791496716148	0.325076749108817	0.568572558281527	0.891395823876391
**Mfsd6**	Mfsd6	-0.0217006108507944	6.2870598414758	0.324952425532011	0.568646508930537	0.891395823876391
**Kdm2a**	Kdm2a	0.0280988534332748	7.0169104321651	0.324882003236937	0.568688405552108	0.891395823876391
**Slc25a30**	Slc25a30	0.0293152338940966	4.02985173045585	0.324879014849806	0.56869018357844	0.891395823876391
**Sntb1**	Sntb1	0.0510976315094825	2.594102689572	0.324817326171015	0.568726889441393	0.891395823876391
**Rpe**	Rpe	-0.0237989846896852	4.61698807832712	0.324550337923178	0.568885805466126	0.891395823876391
**Ebi3**	Ebi3	0.0445657370633688	2.1803814430888	0.324169491479183	0.569113227604645	0.891395823876391
**Rad52**	Rad52	0.0518875088038378	3.51648806805906	0.324154353090449	0.569121661995835	0.891395823876391
**Eid1**	Eid1	-0.0239296158410988	9.40790564565482	0.324122161330664	0.569140844438641	0.891395823876391
**Map4**	Map4	-0.0168985227461466	9.00825968403801	0.324107427203039	0.569149624639122	0.891395823876391
**Zfp667**	Zfp667	0.0362009979062282	4.39587355673059	0.324031054075732	0.569195240314767	0.891395823876391
**Cenpx**	Cenpx	-0.029654003973079	3.87589642432984	0.323945061702357	0.569306014889011	0.891395823876391
**A2m**	A2m	0.0374290035668069	4.21598708054113	0.323820119740873	0.56932088666351	0.891395823876391
**Hk2**	Hk2	0.0473851262516185	2.2512390524749	0.323790321189868	0.569338655067195	0.891395823876391
**Arhgef9**	Arhgef9	-0.0195281118514428	6.64110376776025	0.323421046933134	0.569558938060127	0.891395823876391
**Gars**	Gars	-0.0175010184042108	7.86795063114875	0.323304252584286	0.569628543980053	0.891395823876391
**Mns1**	Mns1	0.0464199629577841	2.54395422860555	0.323301312913536	0.569630398667335	0.891395823876391
**Bhmt**	Bhmt	0.0710212940808669	2.60555307861237	0.323283711339059	0.569640905254878	0.891395823876391
**mrpl11**	mrpl11	-0.0265126515712247	4.72594147595328	0.323224087255227	0.569676498287002	0.891395823876391
**Kcnh4**	Kcnh4	-0.0483701165600163	4.70383658648572	0.32311077697338	0.56974415295062	0.891395823876391
**RGD1565**	RGD156501	-0.0349067230765532	3.22324288764792	0.323103708330358	0.569748372907569	0.891395823876391
**Slc5a3**	Slc5a3	0.0639776274096747	2.49143672331981	0.323049065521971	0.569781004278764	0.891395823876391
**Dgcr6**	Dgcr6	-0.0234063224749234	6.34980289920145	0.322780850964037	0.569941228529802	0.891395823876391
**rnf141**	rnf141	-0.0165508112222527	5.98746095366683	0.322717863840606	0.569978867654179	0.891395823876391
**Hmgcll1**	Hmgcll1	-0.0224874907594821	5.09135630144353	0.322620551372552	0.570037029291851	0.891395823876391
**Has1**	Has1	0.118867221660367	0.0046592811940334	0.322497367322427	0.570110668872663	0.891395823876391
**Tceal8**	Tceal8	-0.0214972010039081	5.83408851755535	0.322346128500797	0.570201107299571	0.891395823876391
**Slc25a25**	Slc25a25	0.0181022016179193	5.86323319159047	0.322185220306494	0.570297357602346	0.891395823876391
**Trmt9b**	Trmt9b	0.0388990090826216	2.61406296820495	0.322142289182977	0.57032304744198	0.891395823876391
**Mink1**	Mink1	-0.0383974893233066	7.52527377375118	0.322131515549486	0.570329489199487	0.891395823876391
**Nisch**	Nisch	0.0205219183053478	7.87365388879084	0.322092859986215	0.570352202657809	0.891395823876391
**Lyar**	Lyar	-0.0230466144194246	4.23046657215859	0.321887118637527	0.570475756799893	0.891395823876391
**Rad54l**	Rad54l	0.0447737381071185	1.86415441064002	0.321881002427865	0.570479418193638	0.891395823876391
**Usp53**	Usp53	0.0315574218815469	4.18068372107123	0.321877908578955	0.570481270305878	0.891395823876391
**Rhbdd2**	Rhbdd2	0.021600672577507	5.54385404347594	0.321771747051393	0.570544830323963	0.891395823876391
**Cdc23**	Cdc23	0.0180871524409641	6.70491071856108	0.32163191344938	0.570628571341767	0.89141513517256
**Mcmdc2**	Mcmdc2	0.0479446677446106	2.46181964645415	0.320324657550813	0.571412602967627	0.89252817316213
**Pus7**	Pus7	0.0257588919453971	4.27319761829172	0.320053527970103	0.571575478282721	0.892670827860961
**Cpped1**	Cpped1	0.0430773734672757	2.30841451897576	0.319917471803524	0.571657245583134	0.892686790145873
**Cry1**	Cry1	-0.0598962824181264	2.93854419303335	0.319416050809977	0.571958788505338	0.892865255941836
**Pnpla2**	Pnpla2	0.0345281052180852	4.342738794279	0.319338887319674	0.572005220555161	0.892865255941836
**Cnpy2**	Cnpy2	0.0245188742123739	5.10689142596235	0.319167304731891	0.572108494507133	0.892865255941836
**Knl1**	Knl1	0.0442640460213432	3.40509343255253	0.319113080982543	0.572141137082567	0.892865255941836
**C2cd4c**	C2cd4c	0.0256278244277186	5.26261822688633	0.319021101477148	0.572196521775805	0.892865255941836
**Ttc30a1**	Ttc30a1	-0.0806524520058578	0.211432278291583	0.318988310437682	0.572216268529785	0.892865255941836
**Sncg**	Sncg	0.122861230031965	1.10877833301195	0.31872939994875	0.572372233788851	0.892865255941836
**Prickle3**	Prickle3	0.0626689085130914	1.00166494156538	0.318677323525368	0.572403611229275	0.892865255941836
**Hat1**	Hat1	-0.0272332160765686	4.65618689609096	0.318655687009063	0.572416548965681	0.892865255941836
**Wrap73**	Wrap73	0.0214410138000077	4.91401593082566	0.318538732112653	0.572487140140834	0.892865255941836
**Bcap31**	Bcap31	-0.0210098947879705	5.58171224677703	0.318109278698836	0.572746125770793	0.893157523916659
**Serp1**	Serp1	-0.0128559852169481	7.78386408186631	0.317804570888825	0.572930014134015	0.8933264013273
**Secisbp2**	Secisbp2	0.0327356192060335	4.28740952086313	0.317365473819976	0.57319521680015	0.893484030950549
**Wdr37**	Wdr37	-0.0163826866613658	6.43059805184479	0.317154097384226	0.573322968624721	0.893484030950549
**Hddc2**	Hddc2	0.0346490506756198	3.1646803571395	0.317118194940555	0.573344672933743	0.893484030950549
**Dlgap2**	Dlgap2	0.0408258975576616	4.18088764902227	0.317077976811376	0.573368988150539	0.893484030950549
**Sprtn**	Sprtn	-0.0340838891880898	3.60182228396526	0.316951403673343	0.573445525436295	0.893484030950549
**Tmem39a**	Tmem39a	0.0358589516272553	3.50661091305482	0.316932810763745	0.573456770045243	0.893484030950549
**Wdr46**	Wdr46	-0.0249687957150538	4.88689119783733	0.316763481884401	0.573559196659639	0.893532038548924
**Gps1**	Gps1	-0.0160584377965322	7.097422962981	0.316526122046163	0.573702825730107	0.893644229888208
**Trrap**	Trrap	0.0581067976845626	8.20203942236785	0.316379108509267	0.573791837007246	0.893671295760754
**Ddx23**	Ddx23	0.013478091677786	6.80918640746584	0.315974548963275	0.574036895975729	0.893849429811753
**Dynll1**	Dynll1	-0.0235157608014451	8.3535637027407	0.315953767864613	0.574049488547293	0.893849429811753
**Eif2a**	Eif2a	0.0195406035404759	5.52114948332999	0.315827119251125	0.574126253767261	0.893857404953846
**Eef1d**	Eef1d	-0.0136080741264459	6.62263636210746	0.315621208306851	0.574251098436769	0.893940231465448
**Slirp**	Slirp	-0.0363145045439508	3.54551665458329	0.315497797765417	0.574325946645673	0.893945218778576
**Fdps**	Fdps	0.0243932999488333	8.14599410432057	0.315180808823721	0.574514579032809	0.893981214211114
**Cul4a**	Cul4a	0.0128749754949682	6.55927375170046	0.315142829698893	0.574541346929057	0.893981214211114
**RGD1308**	RGD130817	0.0264281954357825	5.12841478327359	0.315005000719388	0.574625034017279	0.893981214211114
**Pold1**	Pold1	0.0328957767824819	3.88429625481123	0.314987474282825	0.574635676682947	0.893981214211114
**Wbp2**	Wbp2	-0.0130186846125548	6.83825850018909	0.314618508808871	0.574859778799506	0.894057273989239
**Cmss1**	Cmss1	[illegible]	[illegible]	[illegible]	[illegible]	[illegible]
**Sin3b**	Sin3b	0.0194176318278688	6.47865722302704	0.314437673200331	0.574969723004245	0.894057273989239
**Creg1**	Creg1	0.0475576376841461	3.87382507718638	0.314435272805952	0.574971192313028	0.894057273989239
**Mrpl21**	Mrpl21	-0.02847700904925432	4.10534483264301	0.314032563444179	0.575216121341851	0.894326672286701
**Mocs2**	Mocs2	-0.025255961410879	4.56210000827948	0.313954443320981	0.575318423562463	0.894374295484532
**Rprm**	Rprm	0.0384282104060423	7.76239299584699	0.313526805245805	0.575530047666357	0.894508295572912
**Uhrf2**	Uhrf2	0.0357941515266365	6.62393606893947	0.313487348884152	0.575548053486683	0.894508295572912
**Hdac3**	Hdac3	-0.017880808170211	6.56356516000503	0.313082816052891	0.575794578786394	0.894601305154698
**Ociad1**	Ociad1	-0.0168542372056083	7.44307650148011	0.313072778076396	0.575800696711759	0.894601305154698
**Tnnt2**	Tnnt2	0.119543922909081	2.80262861093222	0.312945369659261	0.575878387915252	0.894601305154698
**Cyren**	Cyren	0.0389106494700507	2.79185520146908	0.312918698807151	0.575894660816104	0.894601305154698
**Cbln4**	Cbln4	-0.0673250798619658	4.73880142112331	0.312529066029697	0.576132380176576	0.894852926272468
**Stt3b**	Stt3b	0.0203055329792494	6.65188020293838	0.312418163038643	0.576200080987129	0.894852926272468
**Ufm1**	Ufm1	-0.020972835247688	5.35914273085371	0.312191372134002	0.576338574771425	0.894956614489492
**Dok4**	Dok4	0.0241262278668201	7.20391547130457	0.311789481865662	0.576584768547248	0.895227497466527
**Ptrh2**	Ptrh2	0.0208173333088625	5.22971451894685	0.311532670633866	0.576741196835697	0.895337837303201
**Atl2**	Atl2	-0.0193073103529385	5.86148703211811	0.31143759969293	0.576799353710277	0.895337837303201
**Spata9**	Spata9	-0.0406532045931258	2.48673091075687	0.31126487828945	0.576905040782626	0.895390495564726
**Dhx57**	Dhx57	0.0204017637088598	7.63956380861687	0.311016867920983	0.577056863932997	0.895514737722886
**Gimap1**	Gimap1	0.0828741055707524	0.277312499017546	0.310820874897047	0.577176900058718	0.895589625921232
**Zbtb7b**	Zbtb7b	0.0647537457436346	1.17860770949266	0.310680825673523	0.57726264246434	0.895611288463657
**Gclc**	Gclc	0.0154918144163013	5.74327043741801	0.310151194143124	0.577587421401553	0.895818791945778
**Rffl**	Rffl	0.0401680494407628	2.45145813018179	0.310054140181819	0.57764696466419	0.895818791945778
**Mff2**	Mff2	-0.0210342018778217	8.60027996722177	0.309916592625647	0.57773127807982	0.895818791945778
**Zfp580**	Zfp580	-0.0217746849810944	6.22309859520802	0.309909595954892	0.577735666167053	0.895818791945778
**Rhbdf2**	Rhbdf2	0.0642098184332735	1.77711847092537	0.309877494623868	0.577755375283738	0.895818791945778
**Prkg2**	Prkg2	0.048388355702393	2.01103439197054	0.309460528174779	0.578011408832072	0.895938118497989
**Acp6**	Acp6	0.0326967677253949	3.53907772255222	0.309398222736924	0.578049694948034	0.895938118497989
**Apom**	Apom	-0.0792631871396381	0.0523065216951443	0.309335008473909	0.578088537628963	0.895938118497989
**C17h6orf52**	C17h6orf52	0.0870180460997458	0.136335611896128	0.309284522060693	0.578119563258765	0.895938118497989
**Stx**	Stx	-0.0478857530243681	3.11738732010211	0.309070842971607	0.578250913622149	0.895998950869051
**Ms4a6bl**	Ms4a6bl	0.127845022208822	-0.057670291476407	0.308987045878396	0.578302440588578	0.895998950869051
**Ctnna2**	Ctnna2	-0.013368071323663	8.26186264275513	0.308822205966263	0.578403212469847	0.896043814500562
**Hpf1**	Hpf1	0.018670444095812	5.48265683851604	0.308330519477038	0.578702761792068	0.896292601926249
**Dtnb**	Dtnb	-0.0274032881718933	5.27646532212309	0.308328861177969	0.57870747784967	0.896292601926249
**Dyrk3**	Dyrk3	0.0297745628696182	3.6212943070116	0.308064605376437	0.578870256154084	0.896382086820975
**Wars2**	Wars2	0.0351120910126757	2.74370324435818	0.30800182599755	0.578908940899528	0.896382086820975
**Cyb5d1**	Cyb5d1	0.0358335547347992	2.43125527279408	0.30737423508527	0.57929594705617	0.896870024993154
**Yars1**	Yars1	-0.0195417737449473	7.02548786194921	0.307076527084782	0.579479710405473	0.897043219197157
**Sri**	Sri	0.0222065119482706	4.72034398651302	0.306940829584016	0.579625279723213	0.897157252835446
**Frem2**	Frem2	-0.0942921395649157	2.1140146969516	0.306500964783652	0.579835212582494	0.89735475952321
**Rbm5**	Rbm5	0.0371515207639486	7.53327392127959	0.306401638276625	0.579896724055113	0.89735475952321
**Nupl2**	Nupl2	-0.0268498273970549	3.83494251333994	0.306201592736976	0.580020447428015	0.897434911031665
**Aplar**	Aplar	0.0153415343871157	6.51308084061552	0.306016494408332	0.580134978527462	0.897500823256558
**Map2k2**	Map2k2	-0.0167008882785306	7.17397832523369	0.305802017285082	0.580267723383735	0.897506146754211
**Gpr160**	Gpr160	0.0973337626848128	-0.426014224950259	0.305722982968547	0.58031667047761	0.897506146754211
**Slc26a6**	Slc26a6	-0.0368129175986158	3.65355500144883	0.305662350248582	0.580354219124227	0.897506146754211
**Coq3**	Coq3	-0.0271685188550877	4.18241186392161	0.304932735738284	0.580806436713315	0.897779076109364
**Kif16b**	Kif16b	0.0287442721571493	3.67189307523537	0.30489404424174	0.580836540931176	0.897779076109364
**Tvp23b**	Tvp23b	0.0264259963505103	4.28928097465535	0.304834912712984	0.580867121423818	0.897779076109364
**Pik3r3**	Pik3r3	0.0334073903536602	4.83087275590409	0.304601167084759	0.581012177454548	0.897779076109364
**Cep44**	Cep44	-0.0362962809084825	3.41466504125679	0.304516907358888	0.581064494508514	0.897779076109364
**Ggct**	Ggct	-0.032083300882926	3.00033622219704	0.30436669005158	0.581157760356622	0.897779076109364
**Pdia5**	Pdia5	0.04802223360081	2.75030868423817	0.304347561920082	0.581169639922416	0.897779076109364
**Snupn**	Snupn	0.0213527970123581	4.40561285805335	0.303882163588004	0.581458204995455	0.897779076109364
**RGD1304**	RGD130463	-0.0383394919728839	2.83713128242925	0.303840847285784	0.581484513537445	0.897779076109364
**Repin1**	Repin1	0.0221023426946874	4.98404854153029	0.303838771409374	0.581485804195647	0.897779076109364
**Lrfn3**	Lrfn3	-0.02083220784519587	5.78489795428327	0.303835182334723	0.581488035685329	0.897779076109364
**Vcan**	Vcan	0.0536808845553777	7.83179206014053	0.303794080468094	0.581513591796003	0.897779076109364
**Arhgap45**	Arhgap45	0.0661864162214932	1.03025813375808	0.303679398172849	0.581585532249802	0.897779076109364
**Fzd5**	Fzd5	0.0930794595454738	1.17476130821375	0.303654350859404	0.581600489024874	0.897779076109364
**Pdcd4**	Pdcd4	0.0169695696944871	6.14200337513976	0.303538171572818	0.58167276025945	0.897779076109364
**Trmt12**	Trmt12	0.0323141910918335	3.03294320258932	0.303523354205339	0.581681978942702	0.897779076109364
**Tmem42**	Tmem42	-0.0303547268522801	4.28609442386108	0.303135497102659	0.581923390147149	0.898040586130609

Paics	Paics	-0.0201860077939033	5.67965072326451	0.302981733218495	0.582019152049605	0.8980772891523
Tufm	Tufm	0.0189331590750882	6.39764852832388	0.302739189197503	0.582170269298252	0.898199387903757
Nfkbib	Nfkbib	0.0281081222953749	3.87529086850271	0.302326247477795	0.582427734499779	0.89940595826430 5
Numbl	Numbl	0.0164591194776 9	6.89588952917991	0.302281892189722	0.582455378261069	0.898405958264305
Mrps22	Mrps22	0.0192280294074838	4.78109758514289	0.302178076720345	0.582520173272383	0.898405958264305
Synm	Synm	0.0408584551219451	4.41934204698075	0.301791451354774	0.582761515291845	0.898667090136738
Ttbk1	Ttbk1	0.0196740087907231	6.63066294279026	0.30136369928897	0.583028764158909	0.89887750510347
Cyp4x1	Cyp4x1	-0.0352267525636322	4.57848012588924	0.301342445838429	0.583042049274856	0.89887750510347
Pcsk1	Pcsk1	-0.0534421263287925	3.10566021747634	0.300972830481362	0.58327249713204	0.899061517980162
RGD1564	RGD156417	-0.0659471134936539	0.56973288090784	0.300778431344675	0.583394825330621	0.899061517980162
Pcdhga10	Pcdhga10	0.0326278944648255	4.72125558633659	0.300740551226809	0.58341853375122	0.899061517980162
Slc25a11	Slc25a11	0.0222273646853685	6.41083961368231	0.300600153673287	0.583506422760463	0.899061517980162
Slc39l2	Slc39l2	-0.0345540422141163	3.32373256609025	0.300575763548061	0.583521693724721	0.899061517980162
Rps3a	Rps3a	-0.023697638198304	8.63644627225816	0.300410507712797	0.583625183713853	0.899109941622143
Alpk3	Alpk3	0.0894792153501899	0.385790857971024	0.300072112586787	0.583836588281286	0.899204152447567
Abcb6	Abcb6	-0.0251974301806219	6.7181767731443	0.300037891787738	0.583859667001015	0.899204152447567
Tada2a	Tada2a	0.0194405647248959	5.31315937589884	0.299933009221597	0.5839244210141	0.899204152447567
RGD1560	RGD156085	-0.0319098856428956	3.82209144756425	0.299849829968778	0.583977321736721	0.899204152447567
Amz2	Amz2	-0.013093049715148	8.19057323820454	0.299738057145888	0.584046682009704	0.899204152447567
Brinp1	Brinp1	-0.0219538780635525	6.96676832519845	0.299169497528896	0.584404168247415	0.899631079109003
Rps4x	Rps4x	-0.0207994969370548	8.70568418541909	0.29904937753939	0.584478890251894	0.899631079109003
Ppp2r5a	Ppp2r5a	0.0217621974283812	5.29025670093216	0.298900843891587	0.584572214774976	0.899631079109003
Sctr	Sctr	0.0668703872872957	1.08326831566915	0.298738851163463	0.584674130056121	0.899631079109003
Gpr173	Gpr173	-0.0239074295426203	3.73174984882563	0.298722366277389	0.584684493106909	0.899631079109003
Lypd3	Lypd3	0.0430345497578035	2.44385282252335	0.298468830927751	0.584843922504974	0.899666555432947
Spa17	Spa17	-0.0572644632287431	1.68795263259079	0.298330453004056	0.58493097470545	0.899666555432947
Smim11a	Smim11a	-0.0329291848223711	3.52310446494955	0.298069574598237	0.585095162576135	0.899666555432947
Sprn	Sprn	-0.0247614034374801	4.60952061698537	0.298009341409871	0.585133084482189	0.899666555432947
[illegible]	[illegible]	[illegible]	[illegible]	[illegible]	[illegible]	[illegible]

Gfm2	Gfm2	0.030363721814394	4.36342319176632	0.249462223577879	0.617453997122261	0.914478094711812
Bcl11b	Bcl11b	-0.0423607045458363	7.96275580425983	0.249315490080193	0.617557474810309	0.914478094711812
Fam43a	Fam43a	-0.0308840852581665	3.93907280144932	0.249287672344341	0.617577095584068	0.914478094711812
Cds1	Cds1	-0.0243617018153991	4.72780785199233	0.249249714498305	0.617603872649098	0.914478094711812
Phactr3	Phactr3	-0.0136931015638118	6.77383803641147	0.249197072254496	0.617641011792614	0.914478094711812
Rasl12	Rasl12	0.0542473168886987	1.40105010010832	0.249029456382779	0.617759297570091	0.914500418886001
Rps6kb1	Rps6kb1	0.0169327692580647	5.69578900465396	0.248948945329959	0.617816121413796	0.914500418886001
Gpr165	Gpr165	-0.0539907497809386	3.37236439235321	0.248801058709745	0.617920556856558	0.914500418886001
Washc2c	Washc2c	-0.018092120150094	7.29484639487925	0.248727797263129	0.617972302284819	0.914500418886001
Ddx39a	Ddx39a	0.0196449432547089	5.30454162710209	0.248565649553254	0.618022564081651	0.914500418886001
Fendrr	Fendrr	-0.0978537841452961	-0.295858436868948	0.248187188099479	0.618354436256586	0.914672043803374
Slc35a2	Slc35a2	-0.0176480553183428	4.95032622340508	0.248155410961751	0.618376914443802	0.914672043803374
Ptpn23	Ptpn23	0.027723161060928571	6.96906857888684	0.24799543047007	0.618490106660141	0.914672043803374
Gabrb3	Gabrb3	0.0181882846174145	7.24469029823248	0.247901755529909	0.618556406263584	0.914672043803374
Ctif	Ctif	0.021403004357895	5.92983602129208	0.24789885022379	0.61855775486028	0.914672043803374
Pde4a	Pde4a	-0.0254726882216517	4.92643355424672	0.247834293169205	0.618604163213473	0.914672043803374
Rnf113a1	Rnf113a1	-0.0292236819901348	3.21842071781127	0.247767140258063	0.618651709257752	0.914672043803374
Ddo	Ddo	0.0731351208499852	1.27627740143652	0.247655327822948	0.618730886288761	0.91468071898299
Drc7	Drc7	-0.0648168645299722	1.03088737178907	0.247464344554118	0.618866196665395	0.914772365335166
Il10ra	Il10ra	-0.0703725182720354	0.562170057599246	0.24718019195258	0.619067626949368	0.914961713035686
Rap1gap	Rap1gap2	-0.0176891567943043	6.50417594500371	0.247065850749934	0.619148721960976	0.914973185349651
Epb41l3	Epb41l3	-0.0167474597272583	7.06033474823608	0.246937356940455	0.619239882526292	0.914999527982064
Fut9	Fut9	0.0421047131472558	3.56728174042173	0.246519827089655	0.619536234123346	0.915329022280283
Nusap1	Nusap1	-0.0375344487627617	3.521842022486	0.2459346407026	0.619952228163083	0.915835261414766
Zbtb5	Zbtb5	0.0128784616690867	6.24805155062183	0.245631509421642	0.620168003284996	0.915939096798081
Tubgcp6	Tubgcp6	0.0262235397646746	5.26127576728907	0.245589615531344	0.62019782987343	0.915939096798081
Zdhhc22	Zdhhc22	-0.0402456140531737	2.97723685119407	0.245526456319681	0.620242802416044	0.915939096798081
Liph	Liph	0.0330922147949658	2.65646832949789	0.245106167100488	0.620542253155293	0.916070187688235
Atp5pb	Atp5pb	-0.0208900375133906	7.70678005853972	0.245104364428528	0.620543538224497	0.916070187688235
E2f3	E2f3	-0.0263146891168033	3.47281675463008	0.24503712549081	0.620591474991257	0.916070187688235
Abraxas2	Abraxas2	-0.0179527730680608	4.90594489664413	0.244980219576888	0.620632053416578	0.916070187688235
Clns1a	Clns1a	-0.0201364930793132	6.24144297741109	0.244728271718913	0.620811771871778	0.916070187688235
Ntf3	Ntf3	-0.0515406676780703	3.32904948768228	0.24465318293511	0.620865356776801	0.916070187688235
Psph	Psph	-0.0382348598862868	3.94346904419091	0.24464704309645	0.620869728744961	0.916070187688235
Ruvbl1	Ruvbl1	-0.0190311377348602	6.1927047503888	0.244579115012516	0.620918937026481	0.916070187688235
Sulf1	Sulf1	0.0374585240078238	4.61650875864932	0.244338248243054	0.621090212216862	0.916105388212805
Mtmr7	Mtmr7	0.0132882240252558	5.95157709494322	0.244303457574981	0.621115062865242	0.916105388212805
Zfp131	Zfp131	0.0175585551998542	5.31015586154666	0.244236260619978	0.621163067463359	0.916105388212805
LOC6846	LOC684871	0.0502075706005444	1.36825080319649	0.243893712667685	0.621407906518305	0.916209973220193
Slc34a1	Slc34a1	-0.0597888592420819	0.473065883349584	0.243865873747615	0.621427814017815	0.916209973220193
Adgrg2	Adgrg2	-0.0513671152355856	1.6653526231447	0.243632229665932	0.621594232146494	0.916209973220193
Zbtb15os	Zbtb15os1	-0.0417449616741908	1.93903041182394	0.243387670940834	0.621769995782307	0.916209973220193
Dph2	Dph2	0.0232103856306153	4.2898045721376	0.243345877680484	0.621799849526142	0.916209973220193
Snrpf	Snrpf	0.0316173307534758	4.63927352043699	0.243263126890454	0.621859183951892	0.916209973220193
RT1-DMb	RT1-DMb	-0.0840182664111722	0.928190528810354	0.243060946666134	0.622004026314797	0.916209973220193
Iqsec2	Iqsec2	-0.0186404945135629	6.91029482512344	0.243058166375164	0.622006018659458	0.916209973220193
Wbp1	Wbp1	0.0194448576244876	5.28606746729683	0.242946144394871	0.622086304877233	0.916209973220193
Itpr2	Itpr2	0.0349916113943958	4.23004892066648	0.242919181900731	0.622105632247624	0.916209973220193
Wdr12	Wdr12	-0.0160056443236157	5.49402311620372	0.242815851641964	0.622179714716048	0.916209973220193
Nsl1	Nsl1	0.0358747155210941	2.26143157992101	0.242802868808154	0.62218902463231	0.916209973220193
Ccdc32	Ccdc32	-0.0210294708705002	5.87125067832255	0.242709528708439	0.622255965955176	0.916209973220193
Cmtr1	Cmtr1	-0.0112749214390982	6.90549408719159	0.242701074071277	0.622262027175436	0.916209973220193
Mier2	Mier2	-0.0232678090188562	5.78632932288329	0.242591570560947	0.622340579903902	0.916217512148789
Ino80	Ino80	0.0255445625593735	5.56073922452156	0.242402813609871	0.622476037098219	0.916308881487429
Ctdp1	Ctdp1	0.0160696393646439	5.5256282871318	0.242115566814007	0.622682298103665	0.91650431118514
Actr3	Actr3	-0.0119611511585307	8.55945461963999	0.24160535711033	0.623049038090024	0.91688674589647
Srpx2	Srpx2	0.131421249690918	-0.356939453630062	0.241483473418853	0.623136719286088	0.91688674589647
Smc6	Smc6	0.0182609921948052	6.49124411302705	0.241356785953954	0.623227885261935	0.91688674589647
Pcgf5	Pcgf5	-0.0508254652183211	1.5636300895386	0.241240808995032	0.623311298057445	0.91688674589647
Kdelr1	Kdelr1	-0.0135455481069451	6.67325682087237	0.241143828040521	0.623381199810519	0.91688674589647
Prkaa1	Prkaa1	-0.0221186936507291	5.111719176412	0.241141264288885	0.623383046039894	0.91688674589647
Cacna1h	Cacna1h	0.0341460291271726	7.51370695287822	0.240942322264004	0.623526346925875	0.916989418976208
Ikbkb	Ikbkb	0.0294887146223577	4.69882169968899	0.240679408459772	0.623716561284516	0.917161053052081
Slc22a23	Slc22a23	0.0140569294365067	7.49055537152906	0.240441189500586	0.623887645938093	0.917304520164389
Cort	Cort	-0.0699978803143177	0.623738349946239	0.240101979454323	0.624132468541646	0.917431614229361
Cyp4f1	Cyp4f1	0.0823361044665243	-0.280173915806265	0.240051303217671	0.624161841864128	0.917431614229361
Papss1	Papss1	0.0144764001864942	7.13953703224483	0.239881620115987	0.624291632800076	0.917431614229361
Vrk1	Vrk1	-0.022054850066225	3.9987308673051	0.239715621122521	0.624411583635037	0.917431614229361
Eif2b1	Eif2b1	-0.019223479460385	5.30912695503614	0.239693913221679	0.624427274190844	0.917431614229361
Six6	Six6	-0.189660785973669	-0.074840046708150	0.23951017615909	0.624559506837705	0.917431614229361
Frmpd1	Frmpd1	-0.029579974383498	4.37862715088154	0.239477299882083	0.624583891429284	0.917431614229361
Hells	Hells	0.0365839159513493	2.57481867005178	0.239381751664276	0.624653003251793	0.917431614229361
Zfp444	Zfp444	0.0170774948547871	5.85595107302303	0.239245228659485	0.62475178257697	0.917431614229361
Nagk	Nagk	0.0217408285361977	5.26425618347177	0.239212420917212	0.624775525368434	0.917431614229361
Dck	Dck	-0.0248366155651205	4.30611979221028	0.239114864545185	0.624846152864621	0.917431614229361
Slc30a4	Slc30a4	-0.0127102796553148	6.70679772587807	0.239022302505795	0.624913152805436	0.917431614229361
Aip	Aip	-0.0238841656245663	5.49260994008347	0.23897037116987	0.62495075788779	0.917431614229361
Pdgfa	Pdgfa	0.0170507841165164	5.67730182272985	0.23889754219595	0.625003504123553	0.917431614229361
Utp18	Utp18	-0.0191575888090267	5.23471549470029	0.238474828608743	0.625309988191623	0.917491110086619
Cstb	Cstb	0.0301082672315765	4.90778672926478	0.238440617587173	0.625334658218924	0.917491110086619
Gorasp1	Gorasp1	0.0178532670248566	5.78883852014291	0.238405504460275	0.625360122665272	0.917491110086619
Cryab	Cryab	-0.05272230883311	1.30108217280779	0.238397828763931	0.625365609678441	0.917491110086619
Mrps26	Mrps26	-0.0246354287917384	5.50780710689502	0.238295721159198	0.625439746203719	0.917491110086619
Egfl8	Egfl8	0.0473544879591109	2.04092252497202	0.238206538536872	0.625504468269526	0.917491110086619
Tmie	Tmie	-0.0437818858199265	2.75738239651685	0.238072644713355	0.625601630887594	0.917491110086619
Cenpm	Cenpm	-0.0407197768564203	2.04223326827604	0.237982752982352	0.625666888165832	0.917491110086619
Arf2	Arf2	-0.016220157767591	6.95688532779543	0.237941503718684	0.625769458508702	0.917491110086619
Canx	Canx	-0.0153489640965727	9.51560150056589	0.237927842638183	0.625779081809499	0.917491110086619
Grm6	Grm6	-0.05706752788962	2.84503327837994	0.237622520183946	0.625928546621254	0.917591985091747
Fam149b	Fam149b1	-0.013947972171869	5.6154753622561	0.237503464105217	0.626015087760894	0.917591985091747
Mrpl18	Mrpl18	-0.022603250804284	4.46219079894768	0.237429556947249	0.626068812141223	0.917591985091747
Trappc2b	Trappc2b	0.0277879882102216	3.56188532145693	0.237091883428803	0.626314445034531	0.917747867230232
Gdpd1	Gdpd1	0.0131474336408433	6.20768751596012	0.237081117002347	0.626322280152715	0.917747867230232
Tmem129	Tmem129	0.0259241342711272	3.92975269899442	0.236799573057432	0.62652724804227	0.917786218202741
Tmem17	Tmem17	-0.02433784461738383	4.05273273627123	0.236779518775407	0.626541853585859	0.917786218202741
Arsa	Arsa	-0.0254322312414979	4.30387186766409	0.2367420716282	0.62656912840979	0.917786218202741
Tmem160	Tmem160	-0.0332922476659452	5.22827119801215	0.236547220577997	0.626711084967317	0.917886395954598
Camk2b	Camk2b	0.017886175123221	9.77184669054704	0.236208452138982	0.626958083107966	0.918140375931701
LOC6886	LOC688613	-0.021725568713909	4.66551536757545	0.236026490636327	0.627090832737707	0.918141557366564
Trh	Trh	-0.117092353895642	1.79862966043333	0.235880238316412	0.627197587667251	0.918141557366564
Pdzrn4	Pdzrn4	-0.0266223920276295	4.12750783850641	0.235825064577867	0.627237868025832	0.918141557366564
Lrat	Lrat	-0.119696107535305	-0.069407714170325	0.235736218480572	0.627302746987276	0.918141557366564
Tm6sf1	Tm6sf1	-0.0171900798078658	5.35054307241125	0.235612434388834	0.627393162463205	0.918141557366564
Gpat4	Gpat4	0.0100979129888596	7.13555734497978	0.235602513545459	0.62740041020336	0.918141557366564
Osbpl7	Osbpl7	-0.0200650104997637	4.83389760674695	0.23548060344529	0.627489487860677	0.918164224233337
Ric8b	Ric8b	-0.0182688672174483	5.08124452564601	0.235082122513871	0.627780118486802	0.9183232514549344
P3h3	P3h3	0.0555487199513457	6.77940184145063	0.234968777027451	0.627863791971794	0.918323251454934
Aars	Aars	-0.0151003473905108	9.08784414116865	0.234813324798708	0.627977562474097	0.918323251454934
Lig4	Lig4	0.0263013001048622	4.13773277900549	0.234776633214274	0.628004425012395	0.918323251454934
Abca7	Abca7	-0.0433107928558211	3.84093544311192	0.234776146859659	0.628004781098355	0.918323251454934
Abhd8	Abhd8	-0.0245401188630783	7.54538665528945	0.234630818948231	0.628111203834873	0.918323251454934
Vrk3	Vrk3	0.0208660517649574	4.54213281622652	0.234619105783892	0.628119783005859	0.918323251454934
Cyc1	Cyc1	-0.0196150370489818	7.30022523019527	0.234524118506596	0.628189692180679	0.9183237448131614
Kctd5	Kctd5	0.0163628739475231	6.2638944396461	0.234338988497122	0.628325032219182	0.918417458645587
Abhd15	Abhd15	-0.0780868974548088	-0.4388620856748	0.234054555384891	0.628533598737893	0.918459613021141
Arpc5l	Arpc5l	-0.0177588190394476	5.67152670649575	0.234042141573937	0.628542704992723	0.918459613021141
Mob1b	Mob1b	0.0263838495399617	4.82629454511341	0.233648786123808	0.628831408400766	0.918459613021141
Vipas39	Vipas39	-0.0131178290980646	6.23346188312919	0.233397022988882	0.629016347452194	0.918459613021141
Rufy3	Rufy3	0.0129602207173242	8.24450256685423	0.233353070096427	0.629048646786427	0.918459613021141
Rpf1	Rpf1	0.0222429428329609	3.69141009485068	0.233326260242322	0.629068350186106	0.918459613021141
Krt18	Krt18	0.072604658268155	2.65014470505582	0.23215002587583	0.629197905083979	0.918459613021141
Rab34	Rab34	0.0256848782352741	4.43028831247909	0.233101853304313	0.629233255083229	0.918459613021141
Clip3	Clip3	0.0161774961978609	9.01319755660645	0.233045108805863	0.629275061225432	0.918459613021141
Ube2r2	Ube2r2	0.011466365622678	7.26626356008153	0.232909154439938	0.629375074249834	0.918459613021141
Med21	Med21	-0.0338901827582139	3.6875132445103	0.232838648102529	0.629426955435004	0.918459613021141
Prcc	Prcc	0.0130148471588294	6.82704886701059	0.232831103128447	0.6294325078811	0.918459613021141
Kcnq3	Kcnq3	0.0285298347256076	6.76602474626341	0.23281354888039	0.629445425977406	0.918459613021141
Med18	Med18	0.0215457334448911	4.19262235329987	0.232781721555583	0.629468851171636	0.918459613021141
Mzf1	Mzf1	-0.0377745763478884	5.72032748593255	0.232702190596655	0.629527393457083	0.918459613021141
Mknk1	Mknk1	-0.0272672214948831	3.32938869173397	0.23269638540016	0.62953166711203	0.918459613021141
Ap3m1	Ap3m1	0.0139530245849143	6.19927779255455	0.232392276627633	0.629754900312124	0.918595266709217
Sypl2	Sypl2	-0.108117013423639	1.50181982716658	0.232361279224321	0.629779476190736	0.918595266709217
Agmo	Agmo	0.0883032181284988	-0.537457071609528	0.232270399646836	0.629845447719592	0.918595266709217
Kcnc3	Kcnc3	-0.025934584600099	6.66329058894754	0.231987297322097	0.63005417673734	0.918790508526164
Npbwr1	Npbwr1	0.0768482178227785	0.110215731131214	0.231797702158943	0.63019405143472	0.918790508526164
Uqcc3	Uqcc3	-0.0305238664248824	3.58954794528066	0.231772245053961	0.630212827895631	0.918790508526164
Psmd14	Psmd14	-0.0176046894925796	6.51721601214398	0.231689457470999	0.630273941049566	0.918790508526164
Rnf146	Rnf146	0.0186738687026641	5.85982670586568	0.231501826944367	0.630412475578948	0.918888520207295
Ptprc	Ptprc	0.0799089960797633	0.472219083481289	0.231189888984943	0.630636217272876	0.91903385269604
Arhgef11	Arhgef11	0.0261114400326756	6.79491228375234	0.231038711684424	0.630754706782999	0.919169175021196
Rpusd4	Rpusd4	0.0231513267619278	3.7684909817605	0.230526877712339	0.631123805965092	0.9193292929648114
Zbtb48	Zbtb48	0.025361176555852	3.80192817847262	0.23027803018644	0.631317749906089	0.9193292929648114
Wnt10a	Wnt10a	-0.104299634352688	-0.675147703234398	0.23023611341425	0.631348809210276	0.9193292929648114
Gja1	Gja1	0.0344283333221762	6.89574331440674	0.230211767308802	0.631366850676512	0.9193292929648114
Nfatc2	Nfatc2	-0.0589872442276396	1.71508756494898	0.230174682787592	0.631394234088621	0.9193292929648114
Klc4	Klc4	0.0217237694987519	5.21743959824355	0.230153588922484	0.631409968009038	0.9193292929648114
Mettl2	Mettl2	-0.0199714085862864	4.21952887280979	0.230134574808677	0.631424061405361	0.9193292929648114
Pmf1	Pmf1	0.03683735172726	2.36367909667841	0.230015061122579	0.631512661121261	0.919414640934853
Mvb12a	Mvb12a	0.0187178290074464	4.96440197876044	0.22978794443815	0.631712382153633	0.919595970921658
Stk36	Stk36	0.0514043223416003	4.64958175362636	0.229684945459028	0.63178451571399	0.919595970921658
Cyp27a1	Cyp27a1	-0.0962845340678647	0.0367489074662172	0.229455024822769	0.631929214084989	0.919679373862468
Kars	Kars	-0.0127325550149259	7.24514539894798	0.229318192541064	0.632029847304845	0.919679373862468
Adipor1	Adipor1	0.0176065725322755	6.61005589821441	0.229273490967742	0.632063046475168	0.919679373862468
RGD1561	RGD156164	0.0693141222362506	0.542125896351194	0.229164048982601	0.632144367457234	0.919690446917434
Cnpy3	Cnpy3	0.0177489104482915	6.00220887713317	0.228879358766818	0.632356014836606	0.919694290627493
Hibch	Hibch	-0.0202612522658768	4.18741340387466	0.228876819224922	0.632357903537096	0.919694290627493
Tmem87b	Tmem87b	0.0262801710080529	4.58932636069009	0.228863051669293	0.632368142926446	0.919694290627493
Zc3h8	Zc3h8	0.0307965956445493	4.10028425574896	0.228668822535425	0.632512637946339	0.919797224202386
Gga3	Gga3	0.0189138140590614	6.51842948268302	0.228546812553219	0.632603444739427	0.91981768247149
Fbxw11	Fbxw11	0.00987540807681935	7.49667208511356	0.228312206215186	0.632778135880859	0.91981768247149
Oga	Oga	-0.0151648898081185	7.66724824909613	0.22829139362409	0.632793638548573	0.91981768247149
Sdcbp	Sdcbp	0.016011560383295	7.43235028399651	0.228209479380794	0.632854662416392	0.91981768247149
Eda	Eda	-0.0218081733360626	4.01864122395307	0.228143830186512	0.632903504460451	0.91981768247149
Camsap1	Camsap1	0.0118219609287549	8.41493555127845	0.228056005121942	0.632969032756288	0.91981768247149
Uba52	Uba52	-0.0247028224260888	8.43027061271632	0.227815884580153	0.633148067503625	0.919891974023703
Mstn	Mstn	-0.0598640978211367	0.52397521197719	0.227789683362182	0.633167510236079	0.919891974023703
Qars1	Qars1	-0.0106269252644684	6.45322039709332	0.227596227528224	0.633311946086086	0.919914725811171
Ccnt2	Ccnt2	0.0523470630180771	6.29890639881941	0.227381191384239	0.633472471232465	0.919914725811171
Tyk2	Tyk2	0.0380611507509856	4.27621287887241	0.227377242472002	0.633475418978669	0.919914725811171
Cdc26	Cdc26	-0.0162005324445348	5.41749558857914	0.227372528574106	0.63347818579543	0.919914725811171
Lrfn4	Lrfn4	-0.0139881328209244	7.10797162905654	0.227113235112007	0.633672631847474	0.920090006698584
Rcan3	Rcan3	-0.0427689333851122	4.03419699478159	0.22686215468012	0.633860317918631	0.920136261118738
Cers5	Cers5	0.0146251814576719	6.29138555614582	0.226855024466566	0.633865649711538	0.920136261118738
Slc25a45	Slc25a45	0.0964686574588829	-0.418083281260127	0.226774690017756	0.633925727384521	0.920136261118738
Spsb1	Spsb1	-0.0273634193767632	3.24526951771352	0.226543659765071	0.634098580665588	0.920280096657502
Pex16	Pex16	-0.0266479778756805	4.66449458778014	0.226294985134217	0.634284754435251	0.920306111550731
Ltb	Ltb	0.0671432428849681	0.6888921462667	0.22628401669138	0.634292968002683	0.920306111550731
Cstf3	Cstf3	-0.017995630582354	4.99955863087011	0.226142779249372	0.634398767412072	0.920306111550731
Rpn1	Rpn1	-0.015668543597484	8.40832104805444	0.226125723266108	0.634411546481353	0.920306111550731
Pdgfb	Pdgfb	0.0268603733229644	4.14030356824282	0.226004097727284	0.63450269065203	0.920331326582815
Sh2d4a	Sh2d4a	-0.0757209150826372	0.239708362754115	0.225795355919772	0.634659188425749	0.920392863538114
Abcb10	Abcb10	0.015074257907685	5.99824389415734	0.225750738717892	0.63469265030712	0.920392863538114
Zfp637	Zfp637	-0.0198522517294638	6.07795275063508	0.225615852821529	0.634793761271107	0.920422902748356
Wnk4	Wnk4	0.0376822207521214	3.64339210367822	0.225459270546722	0.634911344655444	0.920422902748356
Atp6v1c2	Atp6v1c2	-0.0589589407280542	0.513414001447213	0.225428191861951	0.634934673716046	0.920422902748356
Pibf1	Pibf1	-0.0270860154953284	4.14780764969919	0.225282658988711	0.635043945449234	0.920427563864105
Lamtor4	Lamtor4	-0.0298881433821003	4.71542307205756	0.225091527447099	0.635187509155334	0.920427563864105
Kcna4	Kcna4	-0.030402374862344	3.72565998897658	0.225058205045272	0.635212555178755	0.920427563864105
Mau2	Mau2	0.0301497682703434	7.28173407889638	0.225030947980414	0.63523303787239	0.920427563864105
Bcl10	Bcl10	0.0210800828147274	4.68882268523025	0.224739665686975	0.635452032978497	0.920595966045893
LOC2889	LOC288978	0.0537285477502949	0.834871359949908	0.224680185969483	0.635496758479865	0.920595966045893
Gmip	Gmip	0.0288546145945707	5.70664232608592	0.224496091659267	0.635635270780274	0.920689722953968
Ube2d2	Ube2d2	-0.01215856927078075	7.27475711738773	0.224342890717729	0.635750591978106	0.920749870703521
Fbxo7	Fbxo7	-0.0148417702430575	6.02581590074458	0.22415818287282	0.635889693950063	0.920844441900527
Agpat4	Agpat4	0.0122934315979047	5.77688314573646	0.22404146421718	0.635977529995514	0.92086490535616
Rftn1	Rftn1	-0.0421627036977172	4.4244740925556	0.223702825049443	0.636232920135669	0.921127656584656
Sumo2	Sumo2	0.0240835940066567	7.92617394821712	0.223414976512988	0.636450106680569	0.921245865968122
Ralgapa1	Ralgapa1	-0.0220103334318509	7.28996524241092	0.223398902706478	0.636462239682651	0.921245865968122
Dpm1	Dpm1	0.0212254276013267	4.34104771527711	0.223059074348221	0.636718877635366	0.921403891921631
Pla2g3	Pla2g3	0.0514240073906177	3.14454527797085	0.223007751063975	0.636757657683098	0.921403891921631
Shld2	Shld2	0.029708847291146	2.79263816884526	0.222961036871403	0.636792958849341	0.921403891921631
Fam160a	Fam160a1	0.0557113284947876	1.72441001518307	0.222295680050106	0.637296262809742	0.92189221938791
Pdpr	Pdpr	0.0252394373506757	4.80835000543472	0.222181165260508	0.637382981955611	0.92189221938791
Mmp17	Mmp17	-0.025827077789296	6.34755829995934	0.222008066536304	0.637514113656426	0.92189221938791
Sptan1	Sptan1	0.0255899885482637	10.817168478967	0.221764191847569	0.637698968049165	0.92189221938791
Meaf6	Meaf6	-0.0148552421608507	6.09930532547312	0.221761718120632	0.637700843743688	0.92189221938791
Zmat2	Zmat2	0.0191790115360118	5.94805186154218	0.221699003438715	0.637749401195124	0.92189221938791
Dnaaf4	Dnaaf4	-0.0445732947511335	1.24588324823128	0.221672047191618	0.637768845032892	0.92189221938791
P2ry6	P2ry6	-0.0617676840628417	3.67680380310314	0.221605158988915	0.637819581668711	0.92189221938791
Tmem202	Tmem202	0.0863366515399045	-0.153120731111113	0.221536779813533	0.637871455851897	0.92189221938791
Ddx31	Ddx31	0.040500648124818	3.24005278727647	0.221400310876149	0.63797501693207	0.92189221938791
Gpr146	Gpr146	-0.0515501643008684	1.3416969388621	0.221280440078019	0.63806601448098	0.92189221938791
B4galt7	B4galt7	0.0178075672524156	5.1715649198727	0.221257358298999	0.63808354001263	0.92189221938791
Foxp4	Foxp4	-0.0152836805233889	6.90052908875403	0.22123851991617	0.63809784444794	0.92189221938791
Surf2	Surf2	0.0325045611953494	4.80986746326489	0.221071883555098	0.63822440781689	0.92189221938791
Clk2	Clk2	0.0278977452558978	5.22752189069613	0.221052993807206	0.638238758601649	0.92189221938791
Scamp5	Scamp5	-0.0201982308278826	6.47388854899473	0.220677694589396	0.638524041102389	0.922197529903289
Zbtb22	Zbtb22	-0.0196046426088171	5.61332285817538	0.220556949430264	0.638608270517925	0.92220349701507
Fastkd1	Fastkd1	0.0307244384701425	4.49555960382105	0.220460878742173	0.638688976080392	0.92220349701507
Dnttip1	Dnttip1	-0.0176644507143254	5.31440382017973	0.220269864982351	0.638835296778531	0.92220349701507
Kif18b	Kif18b	0.0395684620950749	3.01072274832341	0.22024997060366	0.638849519301822	0.92220349701507
Tnip2	Tnip2	-0.0542411070990558	0.944720872758096	0.220186654135851	0.638897734086581	0.92220349701507
Pym1	Pym1	-0.0191030133047568	5.07074241247249	0.219932594965532	0.639090520329796	0.922284248598021
Agk	Agk	-0.0158670074450579	5.77578140354668	0.219919167129873	0.639101515860154	0.922284248598021
Shank2	Shank2	0.0242520207081337	8.01928914621933	0.219646657040607	0.639309277221135	0.922477366789489
Lrp2	Lrp2	0.059872058359227	3.37393781674665	0.219477907485048	0.639438014913103	0.922493674152266
Hbb-bs	Hbb-bs	0.0775181618759416	5.20026537347439	0.219405234582837	0.639493473891834	0.922493674152266
Sebp1	Sebp1	-0.0274755268862985	3.26489778327678	0.219313037224779	0.639563846736803	0.922493674152266
Trna7	Trna7	-0.0231602205184728	6.01769779823473	0.219112337851215	0.639717106266894	0.922493674152266
Qpctl	Qpctl	0.0139764393717947	5.34113356858464	0.219095558758291	0.639729922949479	0.922493674152266
Dlk1	Dlk1	-0.0781240621196982	4.02103958218041	0.218604223106993	0.640105494429612	0.922493674152266
Traf3ip1	Traf3ip1	0.030054572536391	4.18303567248707	0.218581007277962	0.640123325307097	0.922493674152266
Slc35a3	Slc35a3	0.021006074246543	3.66893213846687	0.218482650147557	0.640198502807717	0.922493674152266
Slu7	Slu7	0.0130785940678466	5.75699313068329	0.218405166149793	0.640257797745749	0.922493674152266
Ptger1	Ptger1	0.0282962950165107	4.90325101652704	0.218270302295025	0.640361033422405	0.922493674152266
Cnot11	Cnot11	-0.0165881933806911	4.70752228903204	0.218213336525977	0.640404651290618	0.922493674152266
Ybey	Ybey	0.0321286664725562	2.71458021332918	0.218201428992785	0.640413768583528	0.922493674152266
Rad51d	Rad51d	0.0286597095823812	2.84131211020732	0.21796527710103	0.640594667714977	0.922493674152266
Entr1	Entr1	-0.0311534057384329	6.50329743822659	0.217907989260226	0.640638568589434	0.922493674152266
Pbx2	Pbx2	-0.0185512234349499	5.67735187087691	0.217805856944175	0.640716854946581	0.922493674152266
Cyp19a1	Cyp19a1	-0.0845194790161081	0.0249322253392465	0.217581465560613	0.64088893153582	0.922493674152266
Nup88	Nup88	-0.011283484883522	6.29523140186534	0.217457968866047	0.640983681437046	0.922493674152266
Sdhaf2	Sdhaf2	-0.0164291174380748	5.30071858546653	0.217424462667985	0.641009394894991	0.922493674152266
Snrpc	Snrpc	0.0266961378117969	5.57443208364589	0.217385857428561	0.64103894693282	0.922493674152266
Crls1	Crls1	-0.0172707714641607	5.32832823364729	0.217350166945344	0.641066418113246	0.922493674152266
Adra2c	Adra2c	-0.0541418903475956	3.59129579800065	0.217277852492385	0.641122085619176	0.922493674152266
Plpp7	Plpp7	0.0269408571447849	4.27225240882479	0.217223632267256	0.641155883577316	0.922493674152266
Tmem132	Tmem132e	-0.0279910220479385	5.21404954331299	0.217225117178046	0.641162422711241	0.922493674152266
Tmed9	Tmed9	-0.0180127834569238	6.6089640422295	0.217163598112222	0.641209665157117	0.922493674152266
Cul9	Cul9	0.0323286885819817	6.63782542061154	0.217100227910592	0.641258237665764	0.922493674152266
Osgep	Osgep	-0.0219158129512849	4.24888480880055	0.217089166422689	0.641266834506783	0.922493674152266
Kctd7	Kctd7	0.0254466687050542	3.8297851828934	0.217020866730296	0.6413192276689	0.922493674152266
Ocel1	Ocel1	-0.0244486402498755	5.12910320068209	0.216925173912486	0.641392835665311	0.922493674152266
Lman1	Lman1	0.0185069750858901	4.81245158970616	0.216775326750309	0.641508019148085	0.922493674152266
Dlgap5	Dlgap5	-0.0354582758152924	3.06265508097611	0.216626574160074	0.641622408179837	0.922493674152266
Wnt3	Wnt3	-0.0395456681601978	2.98545467858675	0.216546698127985	0.641683853187249	0.922493674152266
Lbh	Lbh	-0.0129949288057976	6.26623551823785	0.21654324340367	0.641686511017674	0.922493674152266
Slk	Slk	0.0251723318514513	4.96512476292692	0.216196567424177	0.641953351244966	0.922606344275239
Gfra3	Gfra3	0.0942225344208045	-0.489912106144076	0.21606891037284	0.642051675796359	0.922606344275239
Veph1	Veph1	0.0370384448249666	2.75285872179945	0.216022694120298	0.642087281381565	0.922606344275239
Map1lc3a	Map1lc3a	-0.0254506285646865	8.00312487629907	0.216022189291564	0.642087670323574	0.922606344275239
Evpl	Evpl	0.0805369106165263	0.144774628492488	0.215831344497957	0.642234748853881	0.922606344275239
Clasrp	Clasrp	-0.0459273364765832	6.14624707056865	0.215805868547406	0.642254388407213	0.922606344275239
RGD1310	RGD131041	-0.0167159894469859	5.59948201549076	0.215769410849326	0.642282496303176	0.922606344275239
Sphkap	Sphkap	0.0230487734058778	6.72938596825316	0.215636775214008	0.642384779142637	0.922647046087565
Opcml	Opcml	0.0210380734005512	7.53085802389156	0.21546839362567	0.642514682809756	0.922669254218258
Necab3	Necab3	0.0320273551495258	4.68327425282442	0.215425038413841	0.642548140640486	0.922669254218258
Vars2	Vars2	-0.0247126505408575	5.4305690244863	0.215306886489955	0.642639263742241	0.922693911819557
Dguok	Dguok	0.0301751662855541	3.39384532906948	0.215156676330473	0.642755330566678	0.922753765987857
Dag1b	Dag1b	-0.0167760978280874	4.72840283890125	0.215061481568178	0.642828863757093	0.922753765987857
Alms1	Alms1	0.0346549786215297	5.46248013517816	0.214929300685114	0.642930999673448	0.922794211771035
Arhgap15	Arhgap15	-0.0672291124998637	0.20293001735441	0.214773064175318	0.643051772543539	0.922849391141729
Rnf8	Rnf8	0.014292417937643	4.92438804715101	0.214688230497991	0.643117372643483	0.922849391141729
Lrrc24	Lrrc24	0.0176934449293518	4.62418697193827	0.214210050047306	0.643487432974012	0.922980924374696
Rfc2	Rfc2	-0.0224182538820016	4.14315525580161	0.214132058478985	0.643547837511998	0.922980924374696
Apobec2	Apobec2	-0.0722025863061433	0.0352402729561888	0.214065828797001	0.643599134710452	0.922980924374696
Rnf125	Rnf125	0.0679534687190642	-0.013989964726356	0.213991880814227	0.643656428253148	0.922980924374696
Fam193b	Fam193b	0.0321077579745699	6.28330546035487	0.213929507263728	0.643704778572269	0.922980924374696
Ppp1r36	Ppp1r36	-0.0357674680439445	2.58165151608921	0.213870377583717	0.643750607904294	0.922980924374696
Rbbp4	Rbbp4	0.0159734358432157	6.16889706667195	0.213824727267337	0.64378599666143	0.922980924374696
Mtbp	Mtbp	-0.0358469106642271	1.78238493558748	0.213643421815321	0.643926584687534	0.922980924374696
Glod4	Glod4	-0.0171124230783609	6.09609555488327	0.213595301626611	0.643963920057855	0.922980924374696
Irak1	Irak1	0.017061404415216	6.7689507650617	0.213523333046624	0.644019756780851	0.922980924374696
Polr3e	Polr3e	-0.0182418780475335	5.39586652206072	0.213519467633255	0.644022756079675	0.922980924374696
Adamtsl3	Adamtsl3	-0.0441322616289431	2.40777622823183	0.213307825702742	0.644187026178212	0.922991404346814
Ece2	Ece2	0.0461952342627183	3.66311432255683	0.213279154864651	0.644209287223895	0.922991404346814
Lsm3	Lsm3	-0.0260737580957576	4.23852720345627	0.213224158577851	0.644251994907212	0.922991404346814
Prph	Prph	0.0507540945030524	3.55605052674048	0.213063679556456	0.644376547938315	0.923009871182094
LOC3020	LOC302022	-0.0494120952073854	2.37189765507046	0.212944299418822	0.644469415266495	0.923009871182094
Coprs	Coprs	0.0207428303497431	4.96895937294472	0.212921911901695	0.644486815559702	0.923009871182094
Gpaa1	Gpaa1	-0.0136818598229264	6.26044201202112	0.212803103817023	0.644579175569806	0.92303619575169
Rnf6	Rnf6	-0.018900071921803	4.9619218159269	0.212469083386402	0.644839006771883	0.923119697147423
Cenpw	Cenpw	0.0423832889055942	1.98112502155997	0.212465888416774	0.644841415475848	0.923119697147423
Itpr3	Itpr3	0.0387377178921366	3.16559051133547	0.212292366591541	0.644976573201186	0.923119697147423
Zbtb4	Zbtb4	0.017543200946853	6.08268745093083	0.212141157929764	0.645094337762072	0.923119697147423
Prkcq	Prkcq	-0.0429288373508882	3.22536524190569	0.211960289875307	0.645235268535369	0.923119697147423
Zbtb20	Zbtb20	0.0632713876562622	3.77809990362873	0.211909231412335	0.645275066023172	0.923119697147423
Pnma1	Pnma1	0.0277462823268811	2.62629304487632	0.211593105570707	0.645521598561904	0.923119697147423
Metrnl	Metrnl	-0.0426296018425881	2.39906671529459	0.211382114557438	0.64568626696008	0.923119697147423
Serpine1	Serpine1	-0.0681148778658737	0.117144635871917	0.211381608428525	0.64568666208677	0.923119697147423
Slc22a15	Slc22a15	-0.0292582503441101	4.70164051769749	0.211254016650798	0.6457862821843	0.923119697147423
Lrrc4c	Lrrc4c	-0.01488619696875	6.27671317663974	0.210996812411956	0.645987232112037	0.923119697147423
Tor2a	Tor2a	0.0174803389250922	4.64978747432077	0.210872877904698	0.646084109903892	0.923119697147423
Nt5dc?	Nt5dc?	-0.0714576187191502	-0.053682708887112	0.21075883705339	0.646173284428365	0.923119697147423
Tuba1b	Tuba1b	-0.0240215226305618	10.787862525902	0.210745203370671	0.646183947274833	0.923119697147423
Mrpl22	Mrpl22	-0.0332582839336865	3.42790850332454	0.21065756720887	0.646252497122984	0.923119697147423
Pramef8	Pramef8	0.0271649146302025	2.61249946103105	0.210655869106632	0.646253747334172	0.923119697147423
Sgtb	Sgtb	0.0168164712042664	5.05085442259606	0.21057913298236	0.646313863769228	0.923119697147423
Fkbpl	Fkbpl	0.0282981087531176	3.52047241856715	0.210387547495671	0.646463817731329	0.923119697147423
Slc25a53	Slc25a53	0.0307500393572209	2.60239114507206	0.210358638766053	0.646486921558174	0.923119697147423
Wnt7a	Wnt7a	-0.0243035672932809	5.68584860676496	0.210342806883499	0.646497986786561	0.923119697147423
Mir207	Mir207	-0.0401489321615691	2.02841670823126	0.210336316378601	0.646503930330503	0.923119697147423
Bnip3l	Bnip3l	-0.012548470056473	7.32908175892347	0.210322184911744	0.646514212871016	0.923119697147423
Wbp11	Wbp11	0.0108469340861029	6.96022283892611	0.210303250733261	0.646529822989135	0.923119697147423
Dag1	Dag1	-0.012752858757436	7.92124065534848	0.210258978285076	0.646564495197662	0.923119697147423
Rxrb	Rxrb	0.016500052145418	6.43232925327123	0.210172872774001	0.646631080112447	0.923119697147423
Cdc16	Cdc16	0.0110522270204469	6.65513260477616	0.21013450845723	0.646661998096039	0.923119697147423
Ctsf	Ctsf	-0.0150646297679028	6.00904000673445	0.210128538731674	0.646666675232715	0.923119697147423
Cct6b	Cct6b	0.0803289105552244	-0.282476889001078	0.209990367150326	0.64677495423765	0.923119697147423
Dnajb9	Dnajb9	-0.0199818919669699	4.27927553826545	0.209980010205193	0.646783077249862	0.923119697147423
Akt1	Akt1	0.0127115853212174	8.22689968575159	0.209738189704971	0.646972685627518	0.923284707603173
Wdr82	Wdr82	0.0117786158587117	8.78009030259711	0.209641454089675	0.647048573815123	0.923287403618491
Rmdn2	Rmdn2	-0.0426929648058041	0.936530948048518	0.209492429154537	0.647165523962511	0.923348684102645
Gabarap	Gabarap	-0.0173260646299274	7.2831423151413	0.209193169858608	0.647400525482	0.923432494370645
Vwa5a	Vwa5a	-0.0354893682408031	3.14067092032429	0.209077045755052	0.647491768726857	0.923432494370645
Mmp15	Mmp15	0.0149141553105743	6.81866407805444	0.208874219685832	0.647651213484306	0.923432494370645
Hmg1l1	Hmg1l1	0.0968228254016028	-0.550842307298534	0.208827418776664	0.647688017482757	0.923432494370645
Mrpl20	Mrpl20	-0.0265401685220516	5.62977725307318	0.208807425224279	0.647703741826854	0.923432494370645
Ap5s1	Ap5s1	0.0294722656994944	2.97608259999238	0.208802121053217	0.647707913554889	0.923432494370645
Zdhhc6	Zdhhc6	-0.0162984384931052	5.36861582394053	0.208758350950163	0.647742341166297	0.923432494370645
Mpp3	Mpp3	0.0161678734822429	6.22452616085062	0.208581233627882	0.647881698815449	0.923525642623313
Ggnbp2	Ggnbp2	-0.0131340135416128	6.59634929228452	0.208389102365317	0.648032949419675	0.923536001752954
Zfp52	Zfp52	-0.0466125828044741	2.20819189581598	0.208382853747926	0.648037003714604	0.923536001752954
RGD1563	RGD156394	0.0625278649132131	0.722113820758363	0.20803798415659	0.64830957731926	0.923562756287237
Zfp612	Zfp612	0.0408172044607549	5.28871382200597	0.207993469182624	0.6483449842737	0.923562756287237
Gpr75	Gpr75	0.0259602134238535	3.09361040738224	0.207930073232923	0.648394651412482	0.923562756287237
Polr1f	Polr1f	-0.0225713776726054	4.69308419079595	0.207922216270084	0.648400846673741	0.923562756287237

Rps11	Rps11	-0.01987709897606	8.27236900626081	0.207663023921569	0.648805300674483	0.923562756287237
Fpgs	Fpgs	0.0234470551003272	4.83093804899568	0.207654214952584	0.648612252000891	0.923562756287237
RGD15662(	RGD15662(	-0.0223801326666668	3.96312557091697	0.207564492648793	0.64868306373455	0.923562756287237
Kif11	Kif11	0.0175552928519802	5.43240861430898	0.207492880913964	0.648739594497468	0.923562756287237
Eif4ebp3	Eif4ebp3	-0.038133932495448	2.89531674304737	0.207424674049165	0.648793448929356	0.923562756287237
Laptm4a	Laptm4a	0.023797391662346	7.71143126375571	0.207244669388089	0.648935629012703	0.923562756287237
Ip6k2	Ip6k2	0.0164682506124155	6.38364922555256	0.206889522859077	0.649216365001797	0.923562756287237
Tcte1	Tcte1	-0.0186428164096385	4.74591063080263	0.206687557087079	0.649376144502967	0.923562756287237
Kif3a	Kif3a	-0.0147072714577185	7.91105859135071	0.206675868537552	0.649385394451101	0.923562756287237
Ccnj	Ccnj	0.0280904182157568	4.12636891849829	0.206616555271253	0.649432237957835	0.923562756287237
LOC3613(	LOC361346	-0.0262021301522104	3.47884254865855	0.206463076564333	0.649553846486488	0.923562756287237
Ube2g1	Ube2g1	0.0141080300221236	6.52113621103899	0.206423964715716	0.649584826744259	0.923562756287237
Nudt18	Nudt18	-0.0348216598972692	3.05704262679359	0.206376255418468	0.649622906600101	0.923562756287237
Ndel1	Ndel1	-0.0116618643537724	6.26946535168004	0.206361752023376	0.649634094616573	0.923562756287237
Drc3	Drc3	-0.0373080043551691	2.0144304515975	0.20604261840645	0.649887300999494	0.923562756287237
Kcnh1	Kcnh1	-0.0266836674045588	4.36420324652716	0.205938718661159	0.649969390235147	0.923562756287237
Irx2	Irx2	-0.110690799447032	2.24680055674573	0.205800160267017	0.6500793018991	0.923562756287237
Nup58	Nup58	0.0135689076348935	6.02322374575161	0.205774197378972	0.650099901726209	0.923562756287237
Tpst1	Tpst1	0.0181180018328332	5.4644612445635	0.205683498731887	0.650171878077791	0.923562756287237
Klhl29	Klhl29	0.0275715023150856	6.63054819446867	0.205526360915861	0.650296598119488	0.923562756287237
Nanp	Nanp	-0.0215163250922704	3.65509532265952	0.205517603216575	0.650303578267416	0.923562756287237
Prrt3	Prrt3	-0.0172001719916306	5.21361856807956	0.205515685005828	0.65030510164199	0.923562756287237
Ndufa7	Ndufa7	-0.0248687223020467	5.56916286558351	0.205488902453211	0.650326369779459	0.923562756287237
Ascl2	Ascl2	-0.0766577773796538	0.668254224768975	0.205341547117357	0.650443415158716	0.923562756287237
Pard6b	Pard6b	0.0291014278683284	3.28354133178324	0.205282326311824	0.650490468921327	0.923562756287237
Med22	Med22	-0.0251245018409078	5.03754042782419	0.205254590337447	0.650512509292494	0.923562756287237
Hist3	Hist3	-0.0389569387085432	2.14287012291693	0.205212969929143	0.650545586286274	0.923562756287237
Exd1	Exd1	-0.0424791076651911	2.61520442602044	0.205176505470703	0.650574568013779	0.923562756287237
Klhl17	Klhl17	0.0369091688082763	5.04247859176708	0.205108455689119	0.650628664746269	0.923562756287237
Xrcc1	Xrcc1	-0.0200784176948965	6.34915408352766	0.205030541538392	0.650690615342022	0.923562756287237
Fam168a	Fam168a	0.0149837302409223	8.12298148710429	0.205004077159302	0.650711650760564	0.923562756287237
Jakmip1	Jakmip1	0.0137081918910163	6.8982753438501	0.204924141866051	0.650775238098651	0.923562756287237
[illegible]	[illegible]	[illegible]	[illegible]	[illegible]	[illegible]	[illegible]

**Steap3**	Steap3	-0.0321700389781397	2.4938986327457	0.173610065246535	0.676923157139068	0.928296245149475
**Rtf1**	Rtf1	0.00998040434940045	7.36280926165	0.173598703778453	0.676932131072397	0.928296245149475
**Syngap1**	Syngap1	-0.0148909148866917	9.22982581577374	0.173555537090316	0.676971029462689	0.928296245149475
**Suox**	Suox	-0.0195731903512072	4.03394448099544	0.173190558482226	0.677291685290704	0.928559040337649
**Nmt1**	Nmt1	-0.0118523894156345	6.27596900494179	0.173064528766027	0.677402493440584	0.928559040337649
**Klhl20**	Klhl20	-0.0140887455563135	5.10957495918077	0.172980638859521	0.677476292149757	0.928559040337649
**Aar2**	Aar2	0.0160060970294595	5.48243062641232	0.172747823887347	0.677681090518174	0.928559040337649
**Eif1b**	Eif1b	0.0162625671615188	7.02367887560307	0.17264048255312	0.677775710336476	0.928559040337649
**Dhx34**	Dhx34	0.0194495249928135	4.26186658183214	0.172569430461105	0.677838120493808	0.928559040337649
**Mical1**	Mical1	0.0274719647848763	5.34045598564553	0.172506608575716	0.677893648028818	0.928559040337649
**Kyat1**	Kyat1	0.0183219389160917	4.17492986015173	0.172426715735071	0.677964052843049	0.928559040337649
**Hmgcs2**	Hmgcs2	0.0323406521470635	4.05281897158789	0.172398265219954	0.67798912992164	0.928559040337649
**Tmem196**	Tmem196	-0.0290906781787103	3.28627965360197	0.172191216770429	0.678171701215865	0.928559040337649
**Preb**	Preb	0.0143269038186781	5.84291195272333	0.172138154164301	0.678218511626193	0.928559040337649
**Pm20d2**	Pm20d2	-0.0145104157722585	5.44523658208684	0.172088219628989	0.678262570166348	0.928559040337649
**Tbc1d10b**	Tbc1d10b	0.00941776975175258	7.08267695299815	0.172044760534085	0.678300921255146	0.928559040337649
**Pkp1**	Pkp1	0.0626002553921001	0.76219208612785	0.171935894964056	0.67839772277492	0.928559040337649
**Sirt5**	Sirt5	-0.0130462303073837	6.70312246785875	0.171892929388868	0.678434951812069	0.928559040337649
**Stoml1**	Stoml1	-0.0152516013594525	5.85711151827215	0.171873900419804	0.678451754715445	0.928559040337649
**Tfap2d**	Tfap2d	-0.0774960505217782	2.45384352588996	0.171853324895005	0.678469915649336	0.928559040337649
**Brsk1**	Brsk1	-0.0114212601123338	8.84451707278567	0.171816704974141	0.678502265829794	0.928559040337649
**Rragc**	Rragc	0.0123575367343233	6.13984166382838	0.171509642263558	0.678773611215564	0.928741243287446
**Nrep**	Nrep	-0.0148402605047925	8.70821234389626	0.171464112831167	0.678813868915109	0.928741243287446
**Golga5**	Golga5	-0.0153110160512326	5.03936961851664	0.171400352973073	0.678870256712738	0.928741243287446
**Cdca8**	Cdca8	-0.0277991632872014	3.47384005447819	0.171329256438128	0.678933147393187	0.928741243287446
**Mad1l1**	Mad1l1	-0.0169880400197987	4.22919730714947	0.170903961560882	0.679309674918078	0.929154441345413
**Atg4a**	Atg4a	-0.0300179598863613	3.22967054451592	0.170302201304873	0.679843371656977	0.929609466146116
**Abca2**	Abca2	0.0290842522375298	7.65848609918591	0.170281072172898	0.679862130693838	0.929609466146116
**Tagln**	Tagln	0.0549334061154458	2.79339989250222	0.170276868018732	0.679865863475459	0.929609466146116
**Isoc1**	Isoc1	-0.0145621426830252	7.41645100556003	0.169961320643402	0.680146185529838	0.929804229787602
**Morn4**	Morn4	-0.0144342310198306	6.74108494023785	0.169949764903403	0.680157345938242	0.929804229787602
**Fam122b**	Fam122b	0.0316819374568869	2.22839841305785	0.169830375558959	0.680263602291665	0.929846241089871
**Nedd8**	Nedd8	-0.0208472132420446	6.74943284621788	0.169625467821191	0.680444880951244	0.929960105525502
**Ptpa**	Ptpa	-0.0124659758564877	7.81919486872334	0.169421736285102	0.680626241023096	0.929960105525502
**Hcfc1r1**	Hcfc1r1	-0.0200849806889209	6.80304950867221	0.169413772659013	0.680633232764126	0.929960105525502
**Gla**	Gla	-0.015877829975537	4.75137650777246	0.169355673518098	0.680685078911342	0.929960105525502
**Smim15**	Smim15	0.0182732056814302	4.6919392076311	0.169318069036606	0.680718573676718	0.929960105525502
**Mamdc4**	Mamdc4	-0.0379365959391711	2.43981926805804	0.168522170104708	0.681428555283546	0.930211378893641
**Kpna1**	Kpna1	0.0123964386235786	6.12479003949237	0.168471884589088	0.6814733888897	0.930211378893641
**Prmt3**	Prmt3	0.014401913057644	5.56332249219393	0.168410656532338	0.681526187345759	0.930211378893641
**Vps26a**	Vps26a	0.015035236454862	5.05546060762865	0.168381184692159	0.681554525469085	0.930211378893641
**Rbak**	Rbak	-0.0232330278239739	3.63799403791246	0.16837832156884	0.681557088557642	0.930211378893641
**Slc6a11**	Slc6a11	-0.0185115546190801	7.03447293578138	0.168251234772882	0.68167069004653	0.930211378893641
**Ggps1**	Ggps1	-0.019309617270978	4.49009938320861	0.168076985823955	0.681826805004667	0.930211378893641
**Nkx6-2**	Nkx6-2	-0.118977210885219	-0.502693619114277	0.16807010034637	0.68183269684792	0.930211378893641
**Sertad1**	Sertad1	0.0459739530414932	1.47432634826435	0.168041664432351	0.681858128215815	0.930211378893641
**Sfr1**	Sfr1	-0.0182135101935876	6.28534854880665	0.168004497641292	0.68189139712264	0.930211378893641
**Dhcr24**	Dhcr24	-0.0138352933754239	7.17032024042768	0.167989415219445	0.681904894530364	0.930211378893641
**Pkn1**	Pkn1	0.0152228411297519	7.04798120152938	0.167945359228522	0.682033847328367	0.930211378893641
**Ifi27**	Ifi27	-0.0564268797752378	3.79446663420602	0.167772392574108	0.682099188633387	0.930211378893641
**Pus3**	Pus3	0.0224719003774223	3.90107192582095	0.167516287547265	0.682328660923858	0.930211378893641
**Mxd3**	Mxd3	0.0497580330201522	1.35286636625235	0.167218194962212	0.682506303004676	0.930211378893641
**Nfe2l1**	Nfe2l1	-0.0111343406885904	7.23423200385306	0.167207607424108	0.682544080301223	0.930211378893641
**Wipi2**	Wipi2	-0.012424948718982	5.49373444632526	0.167197534572747	0.682559686118266	0.930211378893641
**Zfpm1**	Zfpm1	-0.0203486161294888	4.36385998233532	0.167186642706289	0.682570486435303	0.930211378893641
**Armcx6**	Armcx6	-0.0197601965933218	4.16184694227348	0.167172152589648	0.682636434012812	0.930211378893641
**Tpd52**	Tpd52	-0.0228340255417947	4.78196305437681	0.167166008302928	0.682642845959949	0.930211378893641
**Acadm**	Acadm	-0.0278465859995097	3.80224092659831	0.16704048888333	0.682755524844522	0.930211378893641
**Slc52a3**	Slc52a3	0.0540144807506645	0.833482133220306	0.167023281829273	0.682770975498135	0.930211378893641
**RGD1566**	RGD15664	-0.0689995856866812	8.79165065472962	0.166935520748822	0.682849792765358	0.930211378893641
**Tbc1d24**	Tbc1d24	-0.0134968819064463	7.33700050150751	0.166794247377403	0.682976719661849	0.930211378893641
**Dffb**	Dffb	-0.0217088168336512	3.4277675242407	0.166772593573029	0.682996180040194	0.930211378893641
**Hmgcl**	Hmgcl	0.0199069703729812	4.26224004268042	0.166771110302548	0.682997513116546	0.930211378893641
**Ap1s3**	Ap1s3	-0.0576223458263362	2.1429145198008	0.166753883217623	0.683012996631384	0.930211378893641
**Foxp3**	Foxp3	-0.0815300326598185	-0.44718810612489	0.166741490846633	0.683024134789571	0.930211378893641
**Nrgn**	Nrgn	-0.0357267478552099	8.60964790400495	0.166696507018827	0.683064571084679	0.930211378893641
**Epha4**	Epha4	-0.0214624969060726	8.03418567977292	0.166489155451302	0.683251043280121	0.93036377463779
**Tars1**	Tars1	0.012484939104868	6.34726293717793	0.166405896700674	0.683325956836232	0.930364246784447
**Egr3**	Egr3	-0.0410141477348032	3.19791632195615	0.166266197902047	0.683451701194505	0.930425857649686
**Fxyd1**	Fxyd1	0.0578809720570607	2.26071273163705	0.166118528839441	0.683584678164261	0.930425857649686
**Bphl**	Bphl	0.0323153430208548	2.90708600912736	0.16610716756685	0.683594921621758	0.930425857649686
**Irak1bp1**	Irak1bp1	-0.017610455982734	4.93825076525672	0.165966245425913	0.683721900889629	0.930497181226232
**Pi4ka**	Pi4ka	0.0145004590154275	7.8459351354646	0.165862829486429	0.683815124835238	0.93052255562976
**Cntnap5b**	Cntnap5b	-0.0374079908360079	3.3647367302453	0.165677242556171	0.683982506526314	0.930531682431909
**Nudcd1**	Nudcd1	0.0177257576561625	3.91555414863521	0.16565504846216	0.684002529892412	0.930531682431909
**Tbc1d25**	Tbc1d25	-0.0142727216937927	4.81223543547203	0.165607350125576	0.684045571152158	0.930531682431909
**Acsm3**	Acsm3	-0.0479992842410042	1.33734180362247	0.165393330785463	0.684238779427498	0.930623310894656
**Cdc37**	Cdc37	-0.0120324995682608	7.41891611852062	0.165367506309451	0.684262103600812	0.930623310894656
**Cd9**	Cd9	0.0318449243652124	4.85799601160986	0.165072426405293	0.684529745746169	0.930786290268586
**Cars2**	Cars2	-0.0185972193838991	4.87685748395872	0.165069788776769	0.684531137252909	0.930786290268586
**Atl3**	Atl3	-0.0279569792332857	3.19356215260052	0.164663542772253	0.684898700848817	0.931184601775165
**Extl2**	Extl2	0.0172874894810399	4.48178194502383	0.164557207753354	0.684994997923307	0.931214051416094
**Sgsm2**	Sgsm2	0.0113157497997464	7.16815748404068	0.164376798385341	0.685158459703349	0.931234797006067
**Snrpb**	Snrpb	0.0197736812543703	6.56145102671229	0.164203628855418	0.685315460026736	0.931357602459426
**Slc20a2**	Slc20a2	-0.0195782291577781	5.25709215410826	0.164130622333602	0.685381678656769	0.931357602459426
**Tgfbi**	Tgfbi	0.0481958101308423	2.55037079610784	0.164111335705186	0.685399174944494	0.931357602459426
**Lnc012**	Lnc012	-0.117477125863662	-0.721538049180039	0.163879201410481	0.685609854611519	0.931482014463366
**Heca**	Heca	-0.0151824275153941	6.30851301042034	0.163797454772983	0.685684087297104	0.931482014463366
**Fdxr**	Fdxr	-0.0194825567351585	3.96931691895224	0.163751661337642	0.685725680932472	0.931482014463366
**Xk**	Xk	0.0239989663000255	2.73088561021189	0.163681572170738	0.685789355202411	0.931482014463366
**Has2**	Has2	0.030958221156604	2.79163631318503	0.163470127770879	0.685981543267822	0.931576307368942
**Wscd2**	Wscd2	0.0219704916002669	4.61754001076185	0.163414381448732	0.686032238961652	0.931576307368942
**Ccnb2**	Ccnb2	-0.0310352212918922	3.25858157791538	0.163358825589356	0.686082767365008	0.931576307368942
**Kansl3**	Kansl3	0.011412523974861	7.02841151053271	0.163018638370396	0.686392400234854	0.931625264870945
**Eps8**	Eps8	0.0136022957969963	5.53384645819454	0.162887575219853	0.686511792005264	0.931625264870945
**Disp2**	Disp2	-0.0242270768314664	8.52386552223711	0.162848500205712	0.686547398188593	0.931625264870945
**Hfe**	Hfe	0.102119453348239	0.401530576345532	0.162837705001095	0.686557235928352	0.931625264870945
**Ppt1**	Ppt1	-0.015888790857487	5.59689481099767	0.16277476472154	0.686614601263801	0.931625264870945
**Noc2l**	Noc2l	0.0259895973982899	6.53651479500763	0.162754680183256	0.686632898718213	0.931625264870945
**Maged1**	Maged1	-0.010564896586719	9.45132812729338	0.16267098795247	0.686709214423555	0.931625264870945
**Slc35b2**	Slc35b2	0.014922757991837	5.44166893094534	0.162534103079508	0.686834067577181	0.931625264870945
**Aldh1a3**	Aldh1a3	0.0848039952223778	1.83241432340243	0.162512025301126	0.686854208297605	0.931625264870945
**Il6r**	Il6r	0.0452162560348741	1.46392042490843	0.162499652467204	0.686865497428114	0.931625264870945
**Invs**	Invs	0.0469982372066213	3.75940474367715	0.162132405627403	0.687200803210084	0.931976741483837
**Garem2**	Garem2	-0.0224880456325	4.15380506937	0.161790481440534	0.687513385707076	0.932196594437304
**Zfp692**	Zfp692	0.0474191622066833	4.74215457661986	0.161686563922004	0.687608461735241	0.932196594437304
**Pigc**	Pigc	0.0164163312021802	4.85135543508688	0.161579506414867	0.687706447721056	0.932196594437304
**Sbf1**	Sbf1	-0.0093150055722515	8.30698940842186	0.161573921238443	0.687711553517557	0.932196594437304
**Eno4**	Eno4	0.0574141400613178	1.32058099349373	0.161297671017564	0.687964582207372	0.932196594437304
**Ints14**	Ints14	0.0110140428555815	5.58567201352107	0.161261234554559	0.687997973664407	0.932196594437304
**Tm9sf4**	Tm9sf4	0.0112731129588027	5.62026452944024	0.161245853834764	0.688012070327802	0.932196594437304
**Qdpr**	Qdpr	-0.0178201138922195	6.48882051881666	0.161149943841366	0.688099990911969	0.932196594437304
**Aak1**	Aak1	0.0159750990043528	7.65181217587694	0.161060423310218	0.688182081698665	0.932196594437304
**Snx6**	Snx6	0.0118726682988563	6.49252823720821	0.16105416968918	0.688187817291002	0.932196594437304
**Gosr1**	Gosr1	-0.0141951071603953	5.07854079261267	0.160991541105851	0.688245265067685	0.932196594437304
**Tcerg1**	Tcerg1	0.0250112373931817	7.52099880299169	0.160974968015988	0.688260497706218	0.932196594437304
**Trmt10b**	Trmt10b	-0.0179407558368774	4.14288529558917	0.16084983029463	0.688375301079586	0.932196594437304
**Tmem205**	Tmem205	0.0262584067763402	3.5247807081549	0.160460676402652	0.688733553194551	0.932196594437304
**Ankrd23**	Ankrd23	-0.0649275475446282	0.142647825517007	0.160430027957901	0.688760908827565	0.932196594437304
**Psme4**	Psme4	0.0222115021126589	7.13655242402266	0.160400672985162	0.688787517888897	0.932196594437304
**Swsap1**	Swsap1	-0.017350496887463	4.58439559679396	0.160358867276003	0.688826331200594	0.932196594437304
**Selenoi**	Selenoi	0.0198452496183303	6.53537966328899	0.16032128889937	0.688860886007844	0.932196594437304
**Arpc1a**	Arpc1a	0.013822754323085	7.63462295685376	0.160228499039228	0.688946230041361	0.932196594437304
**Rab2b**	Rab2b	0.0121175682360086	5.24621456784984	0.160226595086584	0.688947981891447	0.932196594437304
**Frmpd4**	Frmpd4	-0.0164080695815221	5.55963514574796	0.160168778863103	0.689001173753058	0.932196594437304
**Gbx1**	Gbx1	-0.176860015161458	0.43419099434947	0.160104779165575	0.689060068419272	0.932196594437304
**Ctxn1**	Ctxn1	0.0169930708566887	9.03150155975063	0.160069416527458	0.689093537019224	0.932196594437304
**Nsdhl**	Nsdhl	0.0155532164325228	5.7630564860474	0.160002120433824	0.689154555249017	0.932196594437304
**Arhgap18**	Arhgap18	-0.0278396048353755	3.86764993892279	0.159848198545518	0.689296315107796	0.932287276270899
**Tnks2**	Tnks2	-0.0147774426066417	8.05092657070949	0.15959094204171	0.689533405927224	0.932409489080415
**Acr**	Acr	0.0809126211787648	0.809082055633219	0.159502751211193	0.689614734702606	0.932409489080415
**Plec**	Plec	-0.0418189408013067	7.11437367528656	0.159465354238753	0.689667864176465	0.932409489080415
**Kif21a**	Kif21a	0.0225719117734004	7.38909770944436	0.159425884653882	0.689685595473523	0.932409489080415
**Tmem260**	Tmem260	0.0210673934153268	4.7454826995672	0.159132282235106	0.689956663862605	0.932575287536574
**Phf20l1**	Phf20l1	0.023246161762863	6.38081676700897	0.159069584047174	0.690014576436358	0.932575287536574
**Erbb2**	Erbb2	0.0312910797751508	2.96413969626442	0.158999732055879	0.690079112550914	0.932575287536574
**Zc3h3**	Zc3h3	0.0154111032351626	5.46653012727515	0.158969329739278	0.690103720764821	0.932575287536574
**Pde7b**	Pde7b	-0.0551824407848431	2.78877121426187	0.158835820902796	0.690230051566341	0.932640907941156
**P2rx2**	P2rx2	-0.0306191803517625	3.99764635874904	0.158735612618443	0.690323285007016	0.932665254686794
**Anapc11**	Anapc11	-0.0200060968945607	5.8023651691651	0.158493875928883	0.690546970665509	0.93276240314923
**Cxxc1**	Cxxc1	0.0124180195427744	5.96402492877528	0.158419167989075	0.690616121444138	0.93276240314923
**Nacc2**	Nacc2	-0.0251000064425754	4.61670193566521	0.158415575878536	0.690619465241291	0.93276240314923
**Septin5**	Septin5	0.0113232956118364	9.34565598964291	0.158233258887556	0.690789034921854	0.932889514234416
**Gng10**	Gng10	-0.0117771037360285	7.31379496753418	0.158079652926119	0.690930722572341	0.932934618957588
**Gba2**	Gba2	-0.106029171440319	3.37265210028344	0.158035898543289	0.690971292280762	0.932934618957588
**Nkapd1**	Nkapd1	-0.0117240782566758	5.93513900080006	0.157571104628502	0.691403658088345	0.933409433436229
**Atcay**	Atcay	-0.0180744504083785	8.68179015524544	0.157496635066622	0.691471846206065	0.933409433436229
**Cyp26a1**	Cyp26a1	-0.135937627282958	0.179335459209925	0.157266801054083	0.691685381580966	0.93357934103621
**Gcnt2**	Gcnt2	0.0328188939743827	1.96118982664754	0.157199467492852	0.691748097121689	0.93357934103621
**LOC100911**	LOC100911	-0.0398769648394907	2.21700845121212	0.157052802542371	0.691884432157411	0.933662348963878
**Pabpn1**	Pabpn1	-0.0319651842486509	7.95496251172042	0.156921625745952	0.692006661562029	0.933726302185513
**Fam181b**	Fam181b	-0.0213711834890138	4.56686130087307	0.156136628786228	0.692738627668238	0.934577857255908
**Gorab**	Gorab	0.0245614333637889	3.57623848718849	0.156084213963219	0.692787577282992	0.934577857255908
**Pde10a**	Pde10a	0.0273186255485804	6.6681595586657	0.155992075827015	0.692873647094564	0.934587896433589
**Senp3**	Senp3	0.0112332602356232	6.55593210896909	0.155826070981796	0.693028792860086	0.934587896433589
**Gipr**	Gipr	-0.0590884013048961	6.15365900237193	0.15571831384861	0.693129552227425	0.934587896433589
**Usp40**	Usp40	0.0371905417735588	4.99981573418866	0.155711068431783	0.693136347326808	0.934587896433589
**Ubxn2b**	Ubxn2b	0.0197664285322872	5.21349765479731	0.155675557237799	0.693169543447659	0.934587896433589
**Slc36a1**	Slc36a1	0.0188177233021494	4.63050051013399	0.155385251558826	0.693441240548563	0.93485319916796
**Mb21d2**	Mb21d2	-0.0123380818340858	6.20446133729555	0.155179158152392	0.693635237597252	0.934869459746908
**Cstf2t**	Cstf2t	0.0108462639595749	6.8094814221691	0.155167709319933	0.693645030603523	0.934869459746908
**Mpp5**	Mpp5	-0.0222539879671385	3.84267886898694	0.154950719708033	0.693849467427921	0.934869459746908
**Ckb**	Ckb	-0.0162897983136413	9.48515660311475	0.154927404449772	0.693870326242658	0.934869459746908
**Slc25a1**	Slc25a1	0.0126666487198551	6.74399923481643	0.154854557126832	0.693938676475113	0.934869459746908
**Clcn5**	Clcn5	-0.0310921810954783	3.14934950742175	0.154825210888343	0.693966206572558	0.934869459746908
**Cript**	Cript	-0.0173877580917379	5.65820546889498	0.154738888642996	0.694047226656826	0.934869459746908
**Rapgef3**	Rapgef3	-0.0268832881850281	5.34453316497532	0.154689948302263	0.694093168286759	0.934869459746908
**Adam11**	Adam11	0.0349129392425476	6.10212598859135	0.154620394458476	0.694158477234631	0.934869459746908
**Stat5b**	Stat5b	-0.0264364446686964	4.59019154775209	0.154574229171857	0.694201833738487	0.934869459746908
**Sox8**	Sox8	-0.0254431183919414	4.84292329794749	0.154390568607781	0.694374394111893	0.934909225166735
**Rab1a**	Rab1a	-0.0111638329305812	8.14732049687835	0.154382512547507	0.694381965987026	0.934909225166735
**Slco4c1**	Slco4c1	0.0512810483840877	0.870134141756977	0.153946409652008	0.69479219990264	0.935360624433793
**Ccdc120**	Ccdc120	0.0135317199182695	5.66808320914061	0.153780781097758	0.694948178087129	0.935459675045109
**Rell1**	Rell1	0.0433461809898959	0.894004790224701	0.15364119706183	0.695079705250503	0.935545790982797
**Thsd1**	Thsd1	0.0242295161727729	3.48491080360318	0.153361107766642	0.695343835690497	0.935622474779191
**Glp1r**	Glp1r	-0.126221957826364	-0.754453871103952	0.153319995410072	0.695382628965934	0.935622474779191
**Kntc1**	Kntc1	0.0238182611724174	4.56670916652979	0.153288575784018	0.695412286306151	0.935622474779191
**Rbmx**	Rbmx	0.0127517278212128	7.27361085284828	0.153262776889276	0.69543662988717	0.935622474779191
**Klhl4**	Klhl4	-0.0267719097776456	3.99552216105162	0.153106643601916	0.695584042343456	0.935641768238262
**Clba1**	Clba1	0.0215578966138674	3.40987353863221	0.153088742418703	0.695600949194976	0.935641768238262
**Rpa3**	Rpa3	0.024346464620379	3.65410541976994	0.152970783108632	0.695806893214606	0.935725305643421
**LRRTM1**	LRRTM1	0.0166484852405966	6.52590568877206	0.152563800607934	0.696097429681628	0.935725305643421
**Gpsm1**	Gpsm1	-0.0085519637809210	8.29745185121239	0.152491898879927	0.696165208303759	0.935725305643421
**Ostf1**	Ostf1	-0.0234459496563731	3.77564353111695	0.152454702836216	0.696200507261809	0.935725305643421
**Aldh1b1**	Aldh1b1	-0.0181770661705141	4.60876686736791	0.152452687519596	0.696201223925068	0.935725305643421
**Slc38a6**	Slc38a6	-0.0389200341491679	2.72842636064881	0.15241798404351	0.696235273129049	0.935725305643421
**Hsd17b11**	Hsd17b11	0.0285262096135801	3.10318647640052	0.152270064399318	0.696375374420056	0.935725305643421
**Tagln3**	Tagln3	-0.0161914842048107	6.99746872660648	0.152269420162568	0.696375984776874	0.935725305643421
**Nap1l4**	Nap1l4	0.0110302396609292	8.15709166873494	0.15219076145042	0.696450518068932	0.935725305643421
**Sstr4**	Sstr4	-0.0265294286365138	3.45837957059685	0.152151569985439	0.696487562329835	0.935725305643421
**Cilp**	Cilp	0.0812646331774916	-0.366169876113749	0.1521512621137	0.696488011021115	0.935725305643421
**Npff**	Npff	-0.0676988883835436	0.827234275651151	0.152071442984138	0.696563621005869	0.935726130454538
**Gna12**	Gna12	0.0114042281693662	8.21909452751589	0.151884693383671	0.696740745120253	0.935805864009867
**Tmem128**	Tmem128	-0.018679030590299	4.99809167976862	0.151743310339427	0.696874924413678	0.935805864009867
**Prex1**	Prex1	0.014187307486526	7.36710442068591	0.151710556681351	0.696906018477779	0.935805864009867
**Tgm4**	Tgm4	0.032456340516911	1.31923629708783	0.151692697561045	0.696922985263518	0.935805864009867
**Stard3nl**	Stard3nl	-0.0137770449675371	5.96700720369891	0.151481897987587	0.69712320702417	0.935873979178522
**Tada2b**	Tada2b	0.00959827982705384	6.03737901491352	0.151451637387811	0.697142460309208	0.935873979178522
**Dcp2**	Dcp2	0.0210245769438941	4.56844456039298	0.151402425724484	0.697198736592479	0.935873979178522
**Cdc20**	Cdc20	0.0304946642076213	3.93550964961753	0.15119152706815	0.697399283081145	0.936042475796413
**Mturn**	Mturn	-0.0136604147370084	7.64700643379223	0.151032064628637	0.697551032507748	0.936145438201788
**Rabl2**	Rabl2	0.0175388426400695	4.50891360274169	0.150808613206905	0.697763813208442	0.93618110344023
**Taf5l**	Taf5l	-0.0140955741898696	4.72911181868092	0.15073880068546	0.697830321408888	0.93618110344023
**Ptcd3**	Ptcd3	-0.0125540943681263	6.08984711888726	0.150550810587326	0.698009528784319	0.93618110344023
**Lrguk**	Lrguk	-0.0480067277323422	1.32425969114388	0.150501869026428	0.698056214958884	0.93618110344023
**Ccpg1os**	Ccpg1os	0.0379471583671606	1.83314532828073	0.150398463899592	0.698154862432841	0.93618110344023
**Tnk1**	Tnk1	-0.0462489698937967	0.697451569232457	0.150360388464328	0.698191195850766	0.93618110344023
**Hsd11b2**	Hsd11b2	-0.0630924082938469	2.24937322514675	0.150322294883111	0.698227551880629	0.93618110344023
**Eif3j**	Eif3j	-0.0120167994821821	6.23886590520404	0.150283504807156	0.698264578081847	0.93618110344023
**Grm2**	Grm2	-0.0302184695208067	5.26662341611587	0.150146510271028	0.698395387160905	0.93618110344023
**LOC100366**	LOC100366	0.0343789370666437	1.38858310507421	0.150011819967289	0.698524062948246	0.93618110344023
**Pcdha6**	Pcdha6	-0.0272164284932558	4.03331734379193	0.149930768584657	0.698601527094017	0.93618110344023
**Spata1**	Spata1	-0.0415936693993498	0.628467321400567	0.149847900094244	0.698680752792566	0.93618110344023
**Trak2**	Trak2	0.011755920448887	7.51256627376442	0.149733309687761	0.698790347461924	0.93618110344023
**Ush2a**	Ush2a	0.0661517839471984	0.401082804333468	0.14971894109506	0.698804093036302	0.93618110344023
**Cct8**	Cct8	-0.0102905750785111	8.21727689128355	0.149706519471962	0.698815976672333	0.93618110344023
**Asic3**	Asic3	0.0746689771735108	-0.438699252435588	0.149559991534335	0.698956528880936	0.93618110344023
**Spout1**	Spout1	-0.0145836858536224	4.56489347126696	0.149350891211387	0.699156863145288	0.93618110344023
**Mob3b**	Mob3b	0.0342514510538063	2.18231192752148	0.149180589484374	0.699319649284616	0.93618110344023
**Mras**	Mras	0.00914527807556983	7.28426504440638	0.149138614790154	0.699359891549434	0.93618110344023
**Rpusd1**	Rpusd1	-0.0181165773995728	4.42014558413291	0.149093747225557	0.699402914481058	0.93618110344023
**Gucy1a1**	Gucy1a1	0.0204158045572792	7.25582809963848	0.149074088879223	0.699421768961973	0.93618110344023
**Copg2**	Copg2	-0.0119447566399472	6.79570868794901	0.148982750566184	0.69950937972859	0.93618110344023
**Snapc1**	Snapc1	-0.016213092920518	3.90960446914913	0.148925096847051	0.69956459773097	0.93618110344023
**Prkag1**	Prkag1	-0.0155801548901246	6.48796872145062	0.148856811989677	0.699620523808884	0.93618110344023
**Ccdc124**	Ccdc124	-0.0203160012380872	6.2009158136406	0.148848566546732	0.699638148577278	0.93618110344023
**Sycp3**	Sycp3	-0.0578806023474947	0.997317630761515	0.148784132230134	0.699700003300814	0.93618110344023
**Cntnap1**	Cntnap1	-0.0362896214274305	4.79163892794353	0.148679856894091	0.699800128874659	0.93618110344023
**Cdk5rap3**	Cdk5rap3	0.0139436704079172	5.51682166070374	0.148457283546897	0.700014014139392	0.93618110344023
**Tmem265**	Tmem265	0.0313607699162559	3.21548695702214	0.148444103943433	0.700026694402558	0.93618110344023
**Map6d1**	Map6d1	-0.0214940986854563	3.44739327195226	0.14840066807351	0.700068445256953	0.93618110344023
**Gemin8**	Gemin8	0.0182535737329499	3.89070399863137	0.148315803609024	0.700149964008563	0.93618110344023
**RGD1302**	RGD130296	-0.0173435344042018	4.79556729655294	0.148304894868354	0.700160466480881	0.93618110344023
**Toe1**	Toe1	-0.0179497822758784	3.97715460127319	0.147927607102908	0.700522730183615	0.93618110344023
**LOC1025**	LOC102547	0.0357742183522572	1.67344632257948	0.147735804099195	0.700708438261691	0.93618110344023
**C1qtnf1**	C1qtnf1	0.0306497582555969	2.21324808155971	0.147641025766553	0.700799948768275	0.93618110344023
**RGD1308**	RGD130870	-0.0141676843794546	5.28275071704474	0.147467202510896	0.70096763651777	0.93618110344023
**Srsf6**	Srsf6	-0.0147163214652677	7.63140192507612	0.147458861238349	0.70097568621615	0.93618110344023
**Cfap157**	Cfap157	0.0393224357047579	2.36443248882787	0.147205443214403	0.701220370498916	0.93618110344023
**Ust**	Ust	0.0227614273275517	3.2326642364139	0.14719874659549	0.70122587356671	0.93618110344023
**Tacc2**	Tacc2	-0.00913060540969	7.55426535608057	0.147139756568095	0.70128382276168	0.93618110344023
**Dcaf10**	Dcaf10	-0.0144006887195739	5.08768278566193	0.146716812732181	0.701692747094699	0.93618110344023
**Stn1**	Stn1	-0.0171211140733309	4.21063971290037	0.146649125170427	0.701758364293664	0.93618110344023
**Hspbap1**	Hspbap1	0.0299450486528095	3.02160481823233	0.146601473698155	0.701804500682318	0.93618110344023
**Nup54**	Nup54	0.0146948037079704	5.01416706864686	0.146471089607346	0.701930792884799	0.93618110344023
**Nat14**	Nat14	0.0196298857296115	4.82978525284845	0.146442240702143	0.701958722907939	0.93618110344023
**Cobl**	Cobl	-0.0180882598723708	6.08027647085384	0.146408378252858	0.701991544288724	0.93618110344023
**Ercc6**	Ercc6	0.0262682556723262	5.60877614743365	0.14625451155014	0.702140698883228	0.93618110344023
**Ikbke**	Ikbke	0.049806675647284	1.1259542075786	0.146242252162097	0.702152576962521	0.93618110344023
**Selenos**	Selenos	-0.0183180237311323	4.86667729283765	0.146105267564195	0.702285440541198	0.93618110344023
**Pds5b**	Pds5b	0.0147611355078028	7.66698080391139	0.145916876625478	0.702468280579804	0.93618110344023
**Rit1**	Rit1	-0.0119344857862829	5.58922727395964	0.145848675845322	0.702534505182304	0.93618110344023
**Cacng7**	Cacng7	-0.0121035898882962	6.05490571430339	0.145823104313706	0.702559340329947	0.93618110344023
**Qsox1**	Qsox1	0.0132017758285871	5.39236885207385	0.145812206374927	0.702569925198112	0.93618110344023
**Tshr**	Tshr	-0.0245778797085988	3.3106345748808	0.145771218355186	0.702609726794468	0.93618110344023
**Rack1**	Rack1	-0.0154274368313504	8.94561260048216	0.145425224798394	0.702946083941406	0.93618110344023
**Carnmt1**	Carnmt1	0.0220162592530266	2.73210086026519	0.145267906460603	0.703099166403845	0.93618110344023
**Exoc6**	Exoc6	-0.0138305163688881	5.54341139640276	0.145246620546478	0.703121832297689	0.93618110344023
**Ube2l3**	Ube2l3	-0.0092195281792886	7.55498946090857	0.145225396525123	0.703130812827614	0.93618110344023
**Vars1**	Vars1	0.00967587240787212	7.56078489189366	0.145222364845158	0.703142525956858	0.93618110344023
**Ap3b2**	Ap3b2	-0.0154648934262305	8.18760757640929	0.145198804320083	0.703166340578453	0.93618110344023
**Hhip**	Hhip	0.0714321737890924	1.22929625196315	0.145185864726961	0.703178958892136	0.93618110344023
**Alg8**	Alg8	0.0303103941230587	2.98245624477243	0.145184988526097	0.703179888937843	0.93618110344023
**Ssmem1**	Ssmem1	-0.0491161245198264	0.322586238090982	0.145105206854815	0.70325758513205	0.93618110344023
**Harbi1**	Harbi1	0.0229123023848195	3.26402721821537	0.145097697219065	0.703264899632636	0.93618110344023
**Reep6**	Reep6	0.0207276598681271	3.61403469562121	0.145064792303104	0.703316435976759	0.93618110344023
**Usp36**	Usp36	0.0203173390822021	5.28362678604159	0.145037741810086	0.703322304896615	0.93618110344023
**Mir760**	Mir760	-0.0405164182086761	0.497888641591751	0.144995798118167	0.703364172353822	0.93618110344023
**Rassf1**	Rassf1	-0.0164924008345745	4.75340638525233	0.144907270054972	0.703450451095106	0.93618110344023
**Tmed10**	Tmed10	0.0119474329171978	6.886019896929	0.144879472769084	0.703477548231827	0.93618110344023
**Mfsd12**	Mfsd12	0.0196707941281133	5.19889379402572	0.144827576873681	0.703528145242453	0.93618110344023
**Lars2**	Lars2	-0.0139418325215508	4.57851258008138	0.144781220308978	0.703573305251374	0.93618110344023
**Syngr1**	Syngr1	-0.0134209145891823	6.07295902254468	0.144712937544114	0.703639951877701	0.93618110344023
**Ly6e**	Ly6e	-0.0133952421232981	5.86588629998833	0.144696964871738	0.703665529881944	0.93618110344023
**Deup1**	Deup1	0.0627019877257762	0.574970996283622	0.144684730681647	0.703667468530002	0.93618110344023
**Lef1**	Lef1	-0.0452721522374903	3.40653301717292	0.144632825347875	0.70371712904986	0.93618110344023
**Hapln3**	Hapln3	-0.0428381559768272	0.932190712146409	0.144446428254466	0.703890006114168	0.93618110344023
**Rpl18**	Rpl18	-0.0167282677361473	7.88562749081263	0.144365447520197	0.703979165595544	0.93618110344023
**Nfatc2ip**	Nfatc2ip	0.0235174687210385	2.84157692818237	0.144216118390652	0.704125081605658	0.93618110344023
**Ypel5**	Ypel5	-0.0104373673602646	6.34805367487436	0.143935696453645	0.704399327667411	0.93618110344023
**Nmbr**	Nmbr	-0.0459959418946796	1.90555076027596	0.143708789987944	0.70462146395247	0.93618110344023
**Actr6**	Actr6	-0.0165035328978787	4.2339535896805	0.143677174767369	0.704652427076421	0.93618110344023
**Asns**	Asns	0.0224772717157941	6.02356961097783	0.143649161596883	0.704679868304933	0.93618110344023
**Clec1a**	Clec1a	0.0392132039430095	1.02309339067449	0.143537557583578	0.704789224101121	0.93618110344023
**Eif2b2**	Eif2b2	0.0160564555255489	5.41042948813318	0.143498875268503	0.704856554700642	0.93618110344023
**Tpgs2**	Tpgs2	-0.0128913057953716	7.21393220311968	0.143324099579722	0.704998517572072	0.93618110344023
**Fndc3c1**	Fndc3c1	0.102248462988323	-0.413750995485851	0.143298818731864	0.705023216993819	0.93618110344023
**Dctd**	Dctd	0.0267657741926452	3.23236880713794	0.14321070975636	0.705109767652983	0.93618110344023
**Brix1**	Brix1	-0.0136596727839883	5.48974570742155	0.143185516093183	0.70513449273054	0.93618110344023
**Hint2**	Hint2	-0.0260446679241479	3.77520353456263	0.143140328633805	0.705178845963385	0.93618110344023
**Kcnk9**	Kcnk9	0.0304773916620774	3.94002406768387	0.143052601727817	0.705264976158011	0.93618110344023
**Bod1**	Bod1	0.0160857837759605	6.00110972669683	0.143047309939362	0.705270172596286	0.93618110344023
**Ube3b**	Ube3b	0.00948899911219064	6.6478700464001	0.14297425040462	0.705341926938521	0.93618110344023
**Supt7l**	Supt7l	-0.0129374930268883	4.47065167054601	0.142939145876025	0.70547467307744	0.93618110344023
**Letmd1**	Letmd1	-0.0184451082238505	5.45227792601618	0.142787350982857	0.705525580861823	0.93618110344023
**Magoh**	Magoh	-0.0170128698091457	4.58153380388055	0.14276261593551	0.705549889941585	0.93618110344023
**Ppp6c**	Ppp6c	0.0108635518024028	5.84514168647775	0.142753807631999	0.705558466314899	0.93618110344023
**Cant1**	Cant1	-0.0148634388074497	5.31951675215945	0.142746152125234	0.705566085218683	0.93618110344023
**Plekha4**	Plekha4	-0.0268555647053842	2.57006563384206	0.142708978933179	0.705602635699646	0.93618110344023
**Tial1**	Tial1	0.0300164989091941	6.02910334172484	0.142704432213606	0.70560710662766	0.93618110344023
**Vkorc1**	Vkorc1	0.0231258389074284	3.92198120430676	0.14270100356714	0.705610478174119	0.93618110344023
**Aamp**	Aamp	-0.0145145228966258	7.04539129225548	0.142503469114985	0.705804801124828	0.93618110344023
**Eif4e2**	Eif4e2	-0.0115525314763252	5.49612331462661	0.142486964850768	0.705821044027685	0.93618110344023
**Gramd2b**	Gramd2b	-0.0248438914812653	3.71706403555536	0.142472329186077	0.705835448821274	0.93618110344023
**Bace2**	Bace2	-0.0434888574740125	1.87257386804963	0.142471027167218	0.705836730342308	0.93618110344023
**Rpap2**	Rpap2	-0.0128306681154936	4.62556217363648	0.142322328680249	0.705982131906085	0.93618110344023
**Psmg2**	Psmg2	0.0182160738978139	4.03317888859402	0.142297857429959	0.706007233500563	0.93618110344023
**Shd**	Shd	-0.0127561768765263	6.04593036304464	0.14225905127524	0.706045458245806	0.93618110344023
**Plcb1**	Plcb1	0.0142212535274066	7.18831660029807	0.142236709737091	0.706067457912729	0.93618110344023
**Bcar1**	Bcar1	0.0121727249321887	6.65945934891552	0.142180457783972	0.70612289276791	0.93618110344023
**Serpinb9**	Serpinb9	0.0282556701313457	2.97756920415985	0.14215627345596	0.706146725252166	0.93618110344023
**Serinc3**	Serinc3	-0.0108720411080618	7.07434972058397	0.142043528605534	0.706257860978351	0.93618110344023
**Paqr6**	Paqr6	-0.0559412506709343	0.695856382388366	0.14199355890571	0.706307132563166	0.93618110344023
**Syap1**	Syap1	0.0175275812313457	4.54049171937569	0.141904912358012	0.706394566805612	0.93618110344023
**Dok6**	Dok6	-0.020458897210745	5.12981564708023	0.141882330772544	0.706415858995065	0.93618110344023
**Glt8d1**	Glt8d1	-0.0245121038635451	5.37840567592492	0.141863818070769	0.706435011992205	0.93618110344023
**Hps4**	Hps4	-0.0205211090865459	5.17408990451459	0.141839205050218	0.706459396662168	0.93618110344023
**Marchf9**	Marchf9	-0.0140873846110969	6.89950407101688	0.141761821664048	0.706535767416112	0.93618110344023
**Arhgdia**	Arhgdia	-0.0115842220389127	8.96735391828258	0.141652435307421	0.706643763018503	0.93618110344023
**Mettl6**	Mettl6	-0.0157951972221761	4.0674081332599	0.14146005100055	0.70683381698981	0.93618110344023
**Atp5mc2**	Atp5mc2	-0.0200970501966483	7.41333384279029	0.14142366329493	0.706869760378445	0.93618110344023
**Fam118a**	Fam118a	-0.0130982830560239	4.90701490445106	0.141406521029722	0.70688672463421	0.93618110344023
**Spaca6**	Spaca6	-0.0522081003084168	2.81887656778587	0.141317444250305	0.706974791370135	0.93618110344023
**Brpf1**	Brpf1	0.018223914530282	5.61728276770823	0.141192629871256	0.707098243783589	0.93618110344023
**Ppfia1**	Ppfia1	0.0165229628129641	5.98479303524548	0.141075523596143	0.707214128836858	0.93618110344023
**Napa**	Napa	-0.0119412916194741	6.62061414693105	0.141072635001755	0.707216988001533	0.93618110344023
**Nhlh2**	Nhlh2	-0.0365619762019413	3.97984195867617	0.141027961134185	0.707261210974212	0.93618110344023
**Tll1**	Tll1	0.0411329478321885	3.12958433303446	0.141027758597506	0.707261410494882	0.93618110344023
**Afap1l1**	Afap1l1	-0.0261719499823243	3.2459382805452	0.141011061670234	0.707277941954767	0.93618110344023
**Pkp2**	Pkp2	0.0408955229196035	1.41455492245416	0.140906187678532	0.707381795964543	0.93618110344023
**Pycr3**	Pycr3	0.0162682667787866	5.8714893414616	0.140845301983857	0.707442307875716	0.93618110344023
**Lpcat4**	Lpcat4	-0.0169612530237888	5.40791232512828	0.140738384173147	0.707548058208666	0.93618110344023
**Pafah1b2**	Pafah1b2	0.00947223932775146	7.70384357764737	0.140727748391562	0.707558601174489	0.93618110344023
**Pstk**	Pstk	0.0166817375115758	5.40019732264922	0.14065456226392	0.707631153970027	0.93618110344023
**Nt5dc3**	Nt5dc3	-0.0167898623906968	7.43035288565621	0.140653747672928	0.707631961733174	0.93618110344023
**Zfyve16**	Zfyve16	0.0254362016912370	4.98062763205513	0.140391250528822	0.70789236575972	0.936426319718409
**Chid1**	Chid1	0.0140505823781916	5.55710387306239	0.140207828811879	0.708074676785071	0.936567928729673

Fam110b	Fam110b	-0.0124376947425175	6.58842824090446	0.139920626106068	0.708359933423108	0.936747806645562
Zc3h15	Zc3h15	0.00964815700582712	7.00180811828033	0.139919827646687	0.708360628011611	0.936747806645562
Pygl	Pygl	-0.0247355402540389	3.91418637220169	0.139800525749131	0.708479387288973	0.936794205722584
Grik2	Grik2	0.0239482444230172	5.99559828881096	0.1397337162431	0.708545877967783	0.936794205722584
Txnl1	Txnl1	-0.0102544190953067	6.80737030662267	0.13946824822311	0.708810218722297	0.936967246796836
Sult2b1	Sult2b1	0.0198002737176153	4.6618204308346	0.139424179496885	0.70885412805538	0.936967246796836
Hnrnpa1	Hnrnpa1	-0.0149914370874246	10.1246619190755	0.139314437781536	0.708963507215852	0.936967246796836
Saxo2	Saxo2	-0.0763817927720422	-0.225134475871163	0.139300763160577	0.708977140099496	0.936967246796836
Dcaf7	Dcaf7	-0.0106581375862331	8.81808515710562	0.139145747280025	0.709131736164485	0.937072301644173
Ube2i	Ube2i	0.00986031985052115	6.8410167878632	0.138928311738212	0.709348748234297	0.93725980426989
Lamp2	Lamp2	0.0177144243552097	5.45281449029492	0.138611006920286	0.70966578242453	0.937382288669781
Eno3	Eno3	-0.0334808189435288	2.21776558604064	0.138589704326709	0.709687081634108	0.937382288669781
Fbxl4	Fbxl4	-0.0201928371986404	3.62151557612967	0.138496180253362	0.709780612964511	0.937382288669781
Hmces	Hmces	-0.01710442695022	4.56453379934567	0.138476266461005	0.709800532942458	0.937382288669781
Plekhj1	Plekhj1	-0.0175326228376967	4.75005785587422	0.13845971326792	0.709817092518401	0.937382288669781
Snai2	Snai2	0.0760288675252368	-0.500917709717018	0.138372454229318	0.709904403837793	0.937398375138549
Wipf3	Wipf3	0.0196657632779482	5.18100419571392	0.138080763294226	0.710196497215219	0.93768482552929
Clk4	Clk4	-0.0290400871996244	4.78101507509332	0.137997893106371	0.710279746241788	0.93769552363335
Immt	Immt	0.00954393497778637	6.84596452903324	0.137758637121308	0.71051947608584	0.937897826289846
Eapp	Eapp	-0.0147182485615499	4.73147792849572	0.137694999360122	0.710583333124482	0.937897826289846
Zfp428	Zfp428	-0.0191101195613378	6.49713382320634	0.137475541918732	0.710803675578573	0.938004040836753
Dstn	Dstn	-0.0147852234175727	7.49731924710629	0.137343007580531	0.710936941213564	0.938004040836753
Smim5	Smim5	0.0881640507173712	-0.837916572019262	0.137329281438198	0.710950637000829	0.938004040836753
Abcg5	Abcg5	0.0795688214260638	-0.0769484929588848	0.137215477352686	0.711064511837234	0.938004040836753
Slc25a20	Slc25a20	0.0135561233208991	5.60986124304257	0.136978904862531	0.711303055102476	0.938251471612771
Cenpk	Cenpk	0.0331059760907	1.6602142953311	0.136588117249374	0.711696724758795	0.938710094220365
Cps1	Cps1	0.0420511439225688	0.970816036139332	0.136559702522405	0.711725373964121	0.938710094220365
Ddr1	Ddr1	-0.0141312061182493	6.41144590375823	0.136391660120054	0.71189487255565	0.938834407026407
Ogfod1	Ogfod1	0.0207321673418385	3.52347060677337	0.136206590038725	0.71208168168697	0.938981520017789
Psmd5	Psmd5	-0.0130639238581635	5.52866904999751	0.13605873387943	0.712231034489557	0.938991409100894
Polk	Polk	-0.0272236306468173	2.1080187787475	0.136012754310258	0.712277497640897	0.938991409100894
Sema6a	Sema6a	0.0161102682514596	7.4867708816629	0.135975893735574	0.712314954487454	0.938991409100894
Snrnp48	Snrnp48	0.0291276505885164	3.70276650080824	0.135846717959097	0.712445354566353	0.939064091371686
Sf3b1	Sf3b1	-0.0211794623436678	8.80740767554055	0.135579072256803	0.712716180444167	0.939249873271641
Slc16a14	Slc16a14	-0.0265334255419732	4.03042638132398	0.135497120365819	0.712799155951999	0.939249873271641
Phkg1	Phkg1	-0.0475144725043843	0.488560199301738	0.135366354399807	0.712931642406592	0.939249873271641
Nufip1	Nufip1	-0.010702113939902	5.53854469837469	0.135339370280214	0.712958988261705	0.939249873271641
Dnajc17	Dnajc17	-0.0204282813611475	3.60793198779801	0.135157899259539	0.713142973211115	0.939249873271641
Asap1	Asap1	0.0141587297828151	6.30926067440702	0.135141413113004	0.713159694678041	0.939249873271641
Ccdc146	Ccdc146	-0.0611151520940801	0.927675130825687	0.134971548107671	0.713332051768874	0.939249873271641
Abcc4	Abcc4	-0.0264419223825789	3.94697347609099	0.134866779360753	0.713438418955531	0.939249873271641
Rac1	Rac1	0.00944120159961138	7.72367867633644	0.134693400093411	0.713614546491001	0.939249873271641
Foxo3	Foxo3	0.0155351570021024	4.812851179152	0.134634343608546	0.713674568458231	0.939249873271641
Pappa1	Pappa1	0.0639148147167775	0.544188473033008	0.134488110765858	0.713823256292965	0.939249873271641
Phyhd1	Phyhd1	-0.0382506037498586	3.06314723450155	0.134465396431051	0.713845342992133	0.939249873271641
Ifnar1	Ifnar1	-0.0078073449993664	7.17312681028321	0.134389856747013	0.713923211340973	0.939249873271641
Sema5a	Sema5a	-0.0199562802383283	6.21409994364957	0.134264865546269	0.714050446742957	0.939249873271641
Nudt6	Nudt6	-0.0301892287346201	1.94067419565549	0.134179964890098	0.714136875897253	0.939249873271641
Tmem214	Tmem214	-0.013422549568247	6.21036969446031	0.134106678685782	0.714211524025465	0.939249873271641
Cckbr	Cckbr	0.0395034455602701	2.11605741661159	0.1339819092824246	0.714338580177395	0.939249873271641
Ikbip	Ikbip	0.0243249175629843	3.56909892885398	0.133923870219277	0.714397830609016	0.939249873271641
Epb41l1	Epb41l1	-0.0111076284649743	9.24606501201038	0.133901892305685	0.714420442711612	0.939249873271641
Fam8a1	Fam8a1	0.0083850073596248	7.25180800355205	0.133872594427174	0.714450113475753	0.939249873271641
Kcnip3	Kcnip3	0.0270029541407095	2.89270048112299	0.133841930915779	0.714481384654184	0.939249873271641
Gnaq	Gnaq	0.0121572910577835	7.11523000393602	0.133777684365072	0.71454691756845	0.939249873271641
Ube4a	Ube4a	0.00901932567879008	7.32921619364517	0.133758276084042	0.714566717946474	0.939249873271641
Gmpr2	Gmpr2	-0.0159119674733921	4.44532291540265	0.133722707739217	0.714603434635671	0.939249873271641
Sesn3	Sesn3	0.0196659002994911	6.09800899673375	0.133646115595951	0.714681176347	0.939249873271641
Pigx	Pigx	0.0106587121736238	5.89838291671773	0.133601544954594	0.714726675252192	0.939249873271641
Spcs1	Spcs1	-0.0148941186159488	5.88021960786435	0.133583778274541	0.714744814253586	0.939249873271641
Gpt	Gpt	0.0389862377263046	4.7321475508848	0.133566537152475	0.714762418187721	0.939249873271641
Med30	Med30	-0.0209671111648326	4.38187796874603	0.133559719648218	0.714769379471326	0.939249873271641
Med8	Med8	0.0205562310572584	3.50986749966021	0.133248908152682	0.714984726299408	0.939330378609856
Map9	Map9	0.0123226393465025	7.47390915165905	0.133232120725917	0.715001894081181	0.939330378609856
Sil1	Sil1	-0.0254971555441589	3.21105422698988	0.133217345956488	0.715119233073444	0.939330378609856
Cryzl1	Cryzl1	-0.0107414901972388	6.00339010652387	0.133205072822453	0.715131783795461	0.939330378609856
Camkk1	Camkk1	-0.0115719812631866	5.86160009123788	0.133072918737483	0.715266968410439	0.939359018899832
Cntfr	Cntfr	-0.0157165005416229	5.8531230065633	0.133035166682665	0.715305600143484	0.939359018899832
RGD1564	RGD15646(	-0.0308941241607746	5.5812136503973	0.13268167383626	0.715667631703086	0.939359018899832
Iah1	Iah1	-0.0188697523233522	4.81411562116649	0.132507791930479	0.715845914223875	0.939359018899832
Hdlbp	Hdlbp	0.0113814277336897	7.6147029860505	0.132497138719231	0.715856841353141	0.939359018899832
Adam8l1	Adam8l1	-0.049096777152037	0.572731947429275	0.132340944580761	0.716017108744734	0.939359018899832
Btc	Btc	-0.056456847952512	0.03329267701115	0.13233474121769	0.716023476090633	0.939359018899832
Mirlet7c2	Mirlet7c2	-0.0825775211176126	-0.261148956989589	0.132334731710372	0.716023485949402	0.939359018899832
Ier2	Ier2	0.0255868123032756	3.88750480678999	0.132271960667566	0.716087925724793	0.939359018899832
Ppme1	Ppme1	-0.0081504221051779	7.42812562384854	0.132219349383125	0.716141949061581	0.939359018899832
Prpsap2	Prpsap2	0.0116373392530756	6.23401778671977	0.132146261090689	0.716217018219884	0.939359018899832
Coq4	Coq4	-0.0143478048969977	3.99281778810893	0.132085659455669	0.716279281948777	0.939359018899832
Rbx1	Rbx1	-0.0131691866590473	7.11283196771696	0.132019818153168	0.716346946294815	0.939359018899832
Prr7	Prr7	0.0188570246942264	6.67465445134867	0.131895658408595	0.71647459581862	0.939359018899832
Ripk2	Ripk2	0.0220105434915971	3.17066696987364	0.131800707518984	0.716572261406354	0.939359018899832
Tsc2	Tsc2	-0.0188952174259068	7.34027906113955	0.13161137589988	0.716767125038344	0.939359018899832
Dscc1	Dscc1	0.035799621120845	1.50023616126114	0.13156165410718	0.716818325927771	0.939359018899832
Ccdc22	Ccdc22	-0.0157089361014283	4.45588498941374	0.131498295467964	0.716883585191677	0.939359018899832
RGD1308	RGD130894	-0.01336627510798	5.00807648255556	0.131422937966747	0.71696122642249	0.939359018899832
Unc119	Unc119	-0.0179880489075415	5.55765330016006	0.131404221647045	0.716980513850777	0.939359018899832
Kcnk12	Kcnk12	0.0272185920301846	3.2267994132643	0.131300469866432	0.717087458696203	0.939359018899832
Aldh1a1	Aldh1a1	-0.0803685452945584	2.24383181821142	0.131282603725793	0.717105880681399	0.939359018899832
Pou3f4	Pou3f4	-0.0499090782166478	3.22352597828548	0.131265165576252	0.717123861751465	0.939359018899832
Mad2l1bc	Mad2l1bp	0.0210961579329999	3.48749938266859	0.13124218547685	0.717147558078429	0.939359018899832
Zc3h6	Zc3h6	0.0201681797734804	4.18830103156009	0.131109736525843	0.717284189664834	0.939359018899832
Pigrkt	Pigrkt	-0.0172189484547065	4.87871743038666	0.13109492198565	0.717299476744653	0.939359018899832
Chd2	Chd2	0.0298868409829171	5.87241945289228	0.131063598361322	0.717331802715824	0.939359018899832
Mmd	Mmd	-0.0112738349281955	8.57992094089713	0.130985765436366	0.717412124544552	0.939359018899832
Glrx3	Glrx3	-0.0130770304067725	6.97808410505583	0.13092614649085	0.717473704682017	0.939359018899832
Ralbp1	Ralbp1	0.00987855352613631	6.38946262462817	0.130921604453363	0.717478395208535	0.939359018899832
Fam83h	Fam83h	0.0217589378363261	3.76521321556684	0.130882497568805	0.717518784289059	0.939359018899832
Reck	Reck	-0.036382714655683	5.06806645013033	0.130772867168787	0.717632045907786	0.939359018899832
Cct5	Cct5	-0.0127942665696842	8.45240320285165	0.130721782963391	0.717684840432364	0.939359018899832
Ddx47	Ddx47	-0.0109830819700325	5.76772524670908	0.130485513808736	0.717929171822183	0.939359018899832
Amer1	Amer1	0.017081155789518	4.84008954566275	0.130432824828645	0.717983692808801	0.939359018899832
Rnf208	Rnf208	-0.0115085915144621	6.87868126162269	0.130421101205769	0.717995825757549	0.939359018899832
Tmem231	Tmem231	0.0168520132080239	4.22418292543345	0.130395698812869	0.718022117180265	0.939359018899832
Nr2c1	Nr2c1	0.0214697165584066	4.15876261826293	0.130363279534592	0.718055675269542	0.939359018899832
Scn2a	Scn2a	-0.0159879336513226	8.58434089372402	0.13033032621216	0.718089790996839	0.939359018899832
Padi2	Padi2	0.0226597937212763	4.19904465503655	0.130237091803039	0.718186340599611	0.939365222518162
Commd2	Commd2	-0.0131247128747438	5.13214973681456	0.130169178007229	0.718256693809534	0.939365222518162
Rdh11	Rdh11	0.0128766685114705	5.2981208121703	0.130107701384333	0.718320396573358	0.939365222518162
Ndrg3	Ndrg3	0.0141172263602423	6.82521122247365	0.129780163849347	0.718660081493015	0.939572858425559
Krt2	Krt2	-0.0522305585172008	2.49442526226476	0.129557754965035	0.718891015010893	0.939572858425559
E2f1	E2f1	0.0183182195328276	4.15579088613892	0.129535651107176	0.718913978308477	0.939572858425559
Fndc3b	Fndc3b	0.025218573810847	4.25053872850599	0.129523857119972	0.718926127675084	0.939572858425559
Ppp1r3e	Ppp1r3e	0.0233201629125375	3.54656077322141	0.129523458846393	0.71892664557438	0.939572858425559
Abhd17a	Abhd17a	0.0162181898180167	6.99065076260443	0.129453670624486	0.718999165677059	0.939572858425559
Irf2bp1	Irf2bp1	0.0122137982636705	6.64381191343553	0.12937551181377	0.719080410251801	0.939572858425559
Ddx28	Ddx28	0.0177632058904159	3.85355031400011	0.129374358859607	0.719081608932809	0.939572858425559
Tagap	Tagap	0.0532284907610919	-0.190816316555936	0.12916224520834	0.719302237987506	0.93961986775042
Tfrc	Tfrc	0.0151237684828476	6.74989730772858	0.129152089155383	0.719312806753884	0.93961986775042
Gramd1c	Gramd1c	-0.0364974625944666	2.07716022961824	0.129122587809615	0.719343510198827	0.93961986775042
Vav3	Vav3	0.02296020283502723	4.24371165165893	0.129015538652808	0.719454953622604	0.939667063287448
Golga7b	Golga7b	-0.0125980423767586	5.80826283856721	0.128380334241612	0.720117310024827	0.940034057366188
Slc25a19	Slc25a19	0.0182668863098386	3.64950425352845	0.128374761855937	0.72012312877705	0.940034057366188
Tmpo	Tmpo	0.0149014556816691	6.7187923894714	0.128203231216119	0.720302312887144	0.940034057366188
Nup85	Nup85	0.0115982469145851	5.45670370254939	0.128178527388426	0.720328119644315	0.940034057366188
Ntn1	Ntn1	-0.0645184196430677	4.25126988614835	0.128148626255225	0.720359380625724	0.940034057366188
Tmprss5	Tmprss5	-0.0811587068656934	-0.629855524165714	0.128131435895952	0.720377351768225	0.940034057366188
Nbl1	Nbl1	0.0269616307016605	5.17374094462277	0.128081419655487	0.720429641654163	0.940034057366188
Noc4l	Noc4l	-0.0143220949131257	5.46170978255306	0.128067960086675	0.720443715036651	0.940034057366188
Slc5a6	Slc5a6	-0.0107181587713015	6.07592890668313	0.128067210657171	0.720444498667851	0.940034057366188
Cradd	Cradd	0.0437564168175577	0.951787594744119	0.128004126638978	0.720510460465022	0.940034057366188
Fam135a	Fam135a	0.0171928465124888	5.97093879995192	0.127952287670695	0.720564697014524	0.940034057366188
Kcnf1	Kcnf1	-0.0244506158227459	5.15371459823252	0.127679880233141	0.720641505975454	0.940035971777913
Adamts6	Adamts6	0.0420667321368669	2.58034976891433	0.127676621305093	0.720853255340237	0.940213889491965
Agtrap	Agtrap	-0.0215773668105467	3.08780695394462	0.12757713319872	0.720957496680071	0.940251561208032
Cacng4	Cacng4	0.0102571205118913	7.32585712716298	0.127369571958234	0.721175100328095	0.940346300325127
Cxcl16	Cxcl16	0.0533157380251344	1.61208177527847	0.12736406864137	0.721180873630531	0.940346300325127
Ppp6r2	Ppp6r2	0.00972457937505374	7.45548119608853	0.127274538048965	0.721274798963909	0.940370498084921
Thap1	Thap1	0.0181916213343999	3.19121298234478	0.127154410684551	0.721400882812161	0.940436612145788
Tox3	Tox3	0.0170399343656509	5.43808524477778	0.126718450703521	0.721859024528438	0.940935546285136
Fcrl2	Fcrl2	-0.0625904408601577	4.17746320916833	0.126614811454232	0.721968067559446	0.940965548535327
Nuak2	Nuak2	0.0273438274546781	2.17766313793128	0.126513858489673	0.722074332583368	0.940965548535327
Sash3	Sash3	0.0647830173045219	-0.224830069073517	0.1264542516017	0.722137098378179	0.940965548535327
Ginx3	Ginx3	-0.0218871852205375	2.87566755554654	0.126220668625756	0.722383220925843	0.940965548535327
Rangrf	Rangrf	-0.0218575443601279	3.239124086349	0.126043072408244	0.722570522833704	0.940965548535327
Cdc5l	Cdc5l	0.00955489737678933	6.46686104722174	0.125928768549011	0.722691130927756	0.940965548535327
Icmt	Icmt	0.0136365487557488	4.37853284906595	0.125912203449481	0.722708638932156	0.940965548535327
Lsm2	Lsm2	-0.0186580831434948	4.21958910789928	0.125907426960943	0.722713681456964	0.940965548535327
Tra2b	Tra2b	0.00964397781597564	7.52077258228629	0.125815619134691	0.722810623642032	0.940965548535327
Ppp1ca	Ppp1ca	-0.0144240760332732	7.86984438134408	0.125643320302331	0.722992656943352	0.940965548535327
Cyb5b	Cyb5b	-0.00919999111105179	6.71188733387441	0.125616974288363	0.723020514086992	0.940965548535327
Slc16a10	Slc16a10	0.0695718885718294	-0.104171329845996	0.125614805472034	0.723022701004847	0.940965548535327
Fbxo41	Fbxo41	0.0105820679226893	7.52511376766769	0.125603343314332	0.723034923579139	0.940965548535327
Pskh1	Pskh1	0.0243443313006164	2.40652839528889	0.125576121173928	0.723063703116285	0.940965548535327
Trim35	Trim35	0.00909300526979398	8.54449687322188	0.125254940087302	0.723403524598142	0.940965548535327
Rdx	Rdx	0.0104110554383606	6.77188169871946	0.125227194337356	0.723432900624743	0.940965548535327
Tpd52l2	Tpd52l2	-0.0145894857442143	4.95557031190763	0.125172360523834	0.723490976048808	0.940965548535327
Galk1	Galk1	0.0234640232021619	5.85005386300528	0.125168898514815	0.723494643012296	0.940965548535327
Txnrd1	Txnrd1	-0.00809066876485100	7.237279483046	0.12512976257629	0.723536098747823	0.940965548535327
Adcy9	Adcy9	-0.0158855656094931	4.74208634229978	0.125032085805213	0.72363960109194	0.940965548535327
Slc16a3	Slc16a3	0.0616726001673157	0.0260550360746975	0.124954221549935	0.723722130032444	0.940965548535327
Prxl2c	Prxl2c	-0.0433382257338554	0.69720225003245	0.124934757045999	0.72374277843964	0.940965548535327
Nr5a1	Nr5a1	0.166385223301433	-1.02300496231609	0.124856498836241	0.723815165480988	0.940965548535327
Med31	Med31	-0.0179573691504475	3.77094915718268	0.124750491480931	0.723938241009802	0.940965548535327
Arhgap25	Arhgap25	-0.0350185933549364	2.02500969136564	0.124705493328065	0.723985998165124	0.940965548535327
Gpn1	Gpn1	-0.00977884246331291	5.71818105550384	0.124619610058375	0.724077174159371	0.940965548535327
Chtf18	Chtf18	-0.0226308929010082	3.09946104522009	0.124606271187595	0.724091338233883	0.940965548535327
Sgce	Sgce	0.0138988006832553	4.68314512096528	0.124576702022587	0.724122726723174	0.940965548535327
Exosc3	Exosc3	0.0197407395580357	3.18539327364104	0.124575094484652	0.724124448996155	0.940965548535327
Pawr	Pawr	0.0316411952123741	2.00723209306214	0.124498465514904	0.724205844456212	0.940965548535327
Gsta4	Gsta4	0.029474648258497	3.76115441850835	0.124431503939874	0.724276996201901	0.940965548535327
Lypd5	Lypd5	0.0411762278521465	1.02340022022341	0.124391647867917	0.724319356453949	0.940965548535327
Tfap2a	Tfap2a	0.103971577254098	0.134295080730559	0.124343280889953	0.72437077048063	0.940965548535327
Cdc42	Cdc42	-0.0111554450680524	8.3833009210963	0.123964941579627	0.724773251866303	0.941298213439332
Thap6	Thap6	-0.0330009527020701	1.42223343691629	0.123960815622482	0.724777745973525	0.941298213439332
Fbxl21	Fbxl21	0.0230443575654746	2.71259473064397	0.123768315865049	0.724982847942428	0.941368243138247
Fiz1	Fiz1	-0.0100780481517367	5.25405709884468	0.123761767173093	0.724989828536502	0.941368243138247
Nfyb	Nfyb	0.0142867829958452	5.79345750091207	0.123697812227427	0.725058012066887	0.941368243138247
Cfp	Cfp	0.0457284009362473	3.3091699860293	0.123611295443723	0.725150280710521	0.941388046523094
LOC6838	LOC683897	0.0160610975317831	3.91709841327943	0.12351162366685	0.725256623912113	0.941388046523094
Zbtb25	Zbtb25	0.0268003387762526	3.50735216767567	0.123471342823592	0.725299614588964	0.941388046523094
Dpy30	Dpy30	0.0196541363539	5.18747383662867	0.123157375732148	0.725634977687044	0.941492346920784
Polr2k	Polr2k	-0.0238588144069314	3.83904180859997	0.123138617489616	0.725655025206576	0.941492346920784
Bccip	Bccip	-0.0130940621442476	4.23906803294133	0.123092625802258	0.725704195025813	0.941492346920784
Pygm	Pygm	0.0393708634468265	2.70067659010718	0.123028088449038	0.725773209458921	0.941492346920784
Mrpl54	Mrpl54	-0.0240151801760518	5.06602160526115	0.123014562610393	0.725787676186174	0.941492346920784
Zcchc7	Zcchc7	0.0360313249132414	4.61549578576534	0.122972450885489	0.725832723012825	0.941492346920784
Epcam	Epcam	-0.0416504753383443	2.67556342970349	0.122889942434881	0.725921007283181	0.941508982107303
Rwdd3	Rwdd3	0.0319798287177354	2.18552015398604	0.122576455986146	0.726256742359855	0.941704758630265
Depdc5	Depdc5	0.010285547280256	5.97141894003736	0.122541663983988	0.726294033217703	0.941704758630265
Rpp14	Rpp14	-0.0133966644305042	5.22905871459969	0.12253761000504	0.726298378861348	0.941704758630265
Amt	Amt	-0.0242702065337001	5.4601397619461	0.122361612653521	0.726487111898156	0.941846572582989
Ubr3	Ubr3	-0.0106862480779381	7.58979254801264	0.122294865458713	0.726558728957884	0.941846572582989
Arntl2	Arntl2	-0.0480771933636976	1.04738752894154	0.122158089007741	0.726705553151795	0.941939038715452
Nans	Nans	-0.0132429582034577	6.32726951811494	0.122064647806933	0.726805911896232	0.941971264436407
Grpel1	Grpel1	-0.0191871664798084	5.45845826306345	0.12188625054911	0.726997635215768	0.942064307549643
Jmjd8	Jmjd8	-0.0202930721506909	5.11754064370264	0.121805313124909	0.727084670575068	0.942064307549643
Cish	Cish	0.0306600636141569	2.59002770128813	0.121787142941583	0.72710421429054	0.942064307549643
Pelo	Pelo	-0.0158517832774673	4.76255616113555	0.121657810445434	0.727243368771353	0.942145768210691
Dnajc5	Dnajc5	-0.0088730298554925	6.97090149883939	0.12157213084835	0.727335600618188	0.94216842958584
Decr2	Decr2	0.027335047632222	2.55214043454676	0.121242500948472	0.727689706497635	0.942185079657403
Elof1	Elof1	-0.0169028355944773	5.16815102710274	0.121143762825909	0.727797282169614	0.942185079657403
Utp11	Utp11	0.0126442196046322	5.59754427722304	0.121140130206193	0.727801201186081	0.942185079657403
Prmt1	Prmt1	0.0132247301274873	7.98952624294513	0.12113727313826	0.727804282552329	0.942185079657403
Tincr	Tincr	0.0495409493302395	3.72112106784893	0.120998822481553	0.727953700803627	0.942185079657403
Selenok	Selenok	-0.0123881132089164	6.09077447783203	0.120987328889792	0.727966097248833	0.942185079657403
Taf6	Taf6	0.0105668952086758	5.97899540047043	0.120984125867356	0.727969567368839	0.942185079657403
Eif4enif1	Eif4enif1	0.00928599272154812	6.20544398132664	0.120975236104464	0.727979164995483	0.942185079657403
Gna11	Gna11	0.0132842533887846	5.90609944164174	0.120810820535155	0.728156747762143	0.942185079657403
LOC1009	LOC100913	0.00879568238547463	7.91496420076037	0.120794036255901	0.728174880825169	0.942185079657403
Ube2d1	Ube2d1	0.00981662865316971	6.8009282831794	0.120734722757319	0.728238865670491	0.942185079657403
Cmc1	Cmc1	0.0557768871514677	1.75324037095595	0.120720257700299	0.728254621160214	0.942185079657403
Psmd10	Psmd10	-0.0161367049465656	3.93220483163474	0.120424512850171	0.72857452647712	0.942501230363403
Adra1d	Adra1d	0.0465208393597707	1.45301678338919	0.120346843267498	0.728659614126876	0.942512287835479
Polr1c	Polr1c	0.0152317854058841	4.89502328505486	0.120204077531682	0.72881325634707	0.942581237087822
Otud6b	Otud6b	-0.0101021237816206	5.15674925679503	0.120137358818965	0.728885560569579	0.942581237087822
Avp	Avp	-0.106132192018809	3.09968559777863	0.119945352066679	0.729093767171785	0.942581237087822
Klhl36	Klhl36	-0.0159467581294483	3.92021822689582	0.119901047190609	0.729141836611378	0.942581237087822
Gapdhs	Gapdhs	-0.0317837782946085	1.9205881741923	0.119898423121488	0.729144683957969	0.942581237087822
Diaph3	Diaph3	-0.027793020385816	3.49738585805348	0.119879452917431	0.729165268041321	0.942581237087822
Mast3	Mast3	0.015606048770981	7.18024110998567	0.119776941937796	0.729276529981027	0.942627513658269
Dhrsx	Dhrsx	0.0175761035444919	5.18004900811078	0.119436708761185	0.729646229017356	0.942925925450243
Selenoo	Selenoo	-0.0207829098578588	4.22688629490558	0.119418787138414	0.729665718547554	0.942925925450243
Pcyt1b	Pcyt1b	-0.0122580769272805	5.75600514341264	0.119310801858394	0.729783044239142	0.942925925450243
Ralgds	Ralgds	0.00755590561715175	7.90158237502951	0.119258894822508	0.729839671849882	0.942925925450243
Snx11	Snx11	0.0121233247785349	5.14073459662515	0.119200617474977	0.729897057380163	0.942925925450243
Lars1	Lars1	0.0102643785096113	6.99672341598248	0.119110202443544	0.730001557451707	0.942925925450243
Cdadc1	Cdadc1	-0.0156942925938289	4.24274368782336	0.119076192655032	0.730036422204805	0.942925925450243
Jmjd4	Jmjd4	0.01998657865977	3.25954193504216	0.118815534262075	0.730322705422742	0.943181394958185
Wdpcp	Wdpcp	0.0251199465578467	2.96765936069567	0.11875805657818	0.730385400183216	0.943181394958185
Ppm1d	Ppm1d	0.0162260275573785	5.06175982120476	0.118475265098476	0.730693999765015	0.943442537973666
Nudt1	Nudt1	-0.0335007418036534	1.71795934513508	0.118434305488073	0.730738854450389	0.943442537973666
Ciao2a	Ciao2a	0.0207310385744797	3.7894707598064	0.117960780313055	0.731256798538717	0.943737744299428
Txlnb	Txlnb	0.0415329872192442	1.16377427605861	0.11789679093587	0.731326876226719	0.943737744299428
Acat1	Acat1	0.0121018902905508	7.45080342521642	0.117822448468533	0.731397368630465	0.943737744299428
Wdr35	Wdr35	0.0110259393918058	5.5559743648522	0.117684852790838	0.731559145471844	0.943737744299428
Rnf115	Rnf115	-0.0107473127208066	5.5498508050396	0.117658932216859	0.731587567131215	0.943737744299428
Tmem134	Tmem134	0.016814124063313	4.92936220980089	0.117655724858878	0.731591085443856	0.943737744299428
Shisa5	Shisa5	-0.0155182994253983	5.0880024603806	0.117652256758895	0.731594888652087	0.943737744299428
Cdo1	Cdo1	-0.012584740956617	6.39970118803152	0.117592881430845	0.731660010389424	0.943737744299428
Cfap77	Cfap77	0.0439452296331441	1.36374742200009	0.117579304796251	0.73167490418675	0.943737744299428
Rab18	Rab18	-0.0091529856845614	7.22387013621525	0.117534658521619	0.731723887020331	0.943737744299428
Peg3	Peg3	-0.0274179057190855	8.8956285813463	0.117287032867598	0.731995752748358	0.943829625881072
Slc2a6	Slc2a6	-0.01035444727115	5.68289772714958	0.1169191070626	0.732400290345299	0.943829625881072
Cdkn1b	Cdkn1b	0.0146291506867359	6.40921451089255	0.116903851608015	0.732417079144357	0.943829625881072
Vstm2b	Vstm2b	-0.0183798677895685	5.84754976815154	0.116842414330378	0.732484703947169	0.943829625881072
Capns2	Capns2	0.0925049522109809	-0.621953349581063	0.116813919889983	0.732516074986181	0.943829625881072
Rpl22	Rpl22	0.016273214967526	6.30610722030824	0.116756119696049	0.732579723103358	0.943829625881072
Cog4	Cog4	0.0100408551494216	5.72146101243457	0.1166985166209493	0.732657879478901	0.943829625881072
Porcn	Porcn	0.0121481960047099	5.20184718081639	0.116640225786448	0.732707395784834	0.943829625881072
Edem2	Edem2	0.0148560399079889	4.44273228817983	0.116612045481543	0.732739450862085	0.943829625881072
Hmgcs1	Hmgcs1	0.0115779910321125	10.3153403809475	0.116501331140576	0.732860500201877	0.943829625881072
Tpm3	Tpm3	-0.0093194965909146	8.57218480591	0.116494528745775	0.732868001140152	0.943829625881072
Gpr89b	Gpr89b	0.0128445461171043	4.91640708268159	0.116269562375584	0.73311620737615	0.943829625881072
Grk6	Grk6	-0.0071270390828107	6.76627268652858	0.116065412102905	0.733341678226229	0.943829625881072
Ticd3b	Ticd3b	0.00757292139916012	7.71297654490395	0.116055463816245	0.733352672171113	0.943829625881072
Atxn2l	Atxn2l	0.0263027559565553	8.21702017217051	0.115973215497505	0.733443577443489	0.943829625881072
Ppp6r1	Ppp6r1	-0.0106706611289629	7.23885122627738	0.115935511385679	0.733485262084342	0.943829625881072
Ppp1cb	Ppp1cb	-0.0089924790078948	8.4560491979439	0.115761901542612	0.733677298071328	0.943829625881072
Hscb	Hscb	-0.0190387541063014	3.42925041585723	0.115702543586792	0.733742992860863	0.943829625881072
LOC6906	LOC690617	-0.0140322687335005	4.49548866327059	0.115688426008526	0.733758605957991	0.943829625881072
Coq10b	Coq10b	-0.0165580067802958	4.24188803664321	0.115590930968352	0.733866571757818	0.943829625881072
LOC1001	LOC100125	0.0111759599538763	4.67114624512966	0.115426123682075	0.734049170245086	0.943829625881072
Nbn	Nbn	-0.0134452137648813	4.85535948225316	0.115340241078052	0.734144381607723	0.943829625881072
Cdc42se1	Cdc42se1	0.010591320238049	5.68724962322509	0.115271979316773	0.734220086234453	0.943829625881072
Wif1	Wif1	-0.0194957758293063	4.02491545570283	0.115233996231282	0.734262221758054	0.943829625881072
Lamp5	Lamp5	0.1003692812958	2.77111320898364	0.115159289581432	0.734346227703282	0.943829625881072
Tpbg	Tpbg	0.028187527610403	5.09281897327395	0.114902259612307	0.734630553671582	0.943829625881072
Ndufa6	Ndufa6	-0.0194222503976957	5.23007992858879	0.114855323542045	0.734682771521391	0.943829625881072
Itih3	Itih3	0.0418071621947163	4.93307893115879	0.114795846977308	0.7347488237272348	0.943829625881072
Setd5	Setd5	0.0140642127578005	7.75129644031123	0.114764898680107	0.734783241026215	0.943829625881072
Tut1	Tut1	0.012242939490802	4.82977942596082	0.114577147714186	0.734992105677379	0.943829625881072
Kdm8	Kdm8	0.0171472729934685	3.73784986706228	0.114569847577108	0.735000230608286	0.943829625881072
Ticd5	Ticd5	0.0252128539027355	2.56564009427241	0.114562157453534	0.735008788899708	0.943829625881072
Arl6ip5	Arl6ip5	0.0188242951749829	5.78522966336383	0.114517795483579	0.735058172085232	0.943829625881072
Ppp2r3b	Ppp2r3b	-0.0163659547805287	5.17509600019182	0.114413693184751	0.735174832110219	0.943829625881072
Slc4a1ap	Slc4a1ap	-0.0102746552849322	5.66839278198967	0.114370725746014	0.735221961240559	0.943829625881072
Maea	Maea	-0.0103502547838044	6.93414851891716	0.114348130980211	0.735247135058575	0.943829625881072
Gas2l2	Gas2l2	0.0843018494780831	-0.013406236703850	0.114334118341887	0.735262748544871	0.943829625881072
RGD1305	RGD130535	-0.0155751038894227	5.63470805599029	0.114323427730998	0.735263518080898	0.943829625881072
Clpb	Clpb	0.010490403566741	5.70523891524964	0.114167742358489	0.735448213383379	0.943829625881072
Tmem150	Tmem150c	0.0181252855760665	6.53149211198564	0.114145221066004	0.735473219031284	0.943829625881072
Plac9	Plac9	0.071042104251328	0.0677062653574395	0.114087628052516	0.735537573171726	0.943829625881072
RT1-N3	RT1-N3	-0.0218729211808205	3.53230960039851	0.114069610414496	0.735557674925005	0.943829625881072
Tmem25	Tmem25	-0.0252588540817379	2.93256117403335	0.113996106353831	0.735636698582392	0.943829625881072
Abcc5	Abcc5	0.036674685988133	6.73417452523821	0.113987377428003	0.735649442223838	0.943829625881072
Csnk1e	Csnk1e	0.00941220755766668	7.76877712325137	0.113945820617006	0.735695719795263	0.943829625881072
Rab27b	Rab27b	-0.0336451379941132	2.18945213374518	0.113931171397908	0.735712182233831	0.943829625881072
Cyp7b1	Cyp7b1	0.0271844928855926	2.48441815498211	0.113862334235264	0.735789054229884	0.943829625881072
Polr2d	Polr2d	-0.0171156785485662	4.19209497628069	0.113852227907365	0.735800341768786	0.943829625881072
Rpp21	Rpp21	-0.01820345681593707	5.09654897128095	0.11284170012515	0.735812100614098	0.943829625881072
Gas7	Gas7	0.0113757392121402	9.27312298206971	0.113825089688063	0.735830654619342	0.943829625881072
Gpx2	Gpx2	0.0709218547540887	0.121085414476424	0.113802863640487	0.735855403670087	0.943829625881072
Tbc1d10c	Tbc1d10c	-0.0830350825229737	-0.320125478374862	0.113786280543295	0.735874010655576	0.943829625881072
Depdc1	Depdc1	0.0343655987439084	1.55259412812309	0.11372928768084	0.735937595748743	0.943829625881072
Pigy	Pigy	-0.02240331788306	3.22054362132579	0.11361711362613	0.736063093690144	0.943829625881072
LOC4981	LOC498154	-0.0164098383409505	4.11827418547139	0.113600940847838	0.736074468873724	0.943829625881072
Marveld2	Marveld2	0.0466811676086407	1.75339932593405	0.113575829416803	0.73610926182759	0.943829625881072
Mfsd10	Mfsd10	-0.0268782841107967	5.04425143183732	0.113137585205322	0.736599928861997	0.944313928132934
Dnajb11	Dnajb11	-0.010923341280777	6.02476277880683	0.113087462872244	0.736656115203285	0.944313928132934
Fyttd1	Fyttd1	-0.0127644773728843	7.40929129258465	0.112893677787056	0.736873472287793	0.944313928132934
Gins1	Gins1	0.0271135152811185	2.43092854806158	0.112849695861307	0.736922832169176	0.944313928132934
Spag6l	Spag6l	0.0568841635420903	2.39433437852792	0.112845829064022	0.736927061131913	0.944313928132934
Nkx2-2	Nkx2-2	-0.0298052846644677	2.6941273770983	0.1128086872018982	0.736937115094849	0.944313928132934
Zfp18	Zfp18	0.0154498876028285	3.92262744094887	0.112549842480547	0.737259643212418	0.944313928132934
Cacnb1	Cacnb1	-0.0100958721090085	7.18338465727878	0.112485801570956	0.737331529455024	0.944313928132934
Rpa2	Rpa2	-0.018734325601757	3.90252125892961	0.112467953120024	0.737351712247836	0.944313928132934
Faxc	Faxc	0.0162362909105441	5.15786527543839	0.112404857566133	0.737422678576102	0.944313928132934
Epb41l4a	Epb41l4a	-0.0241531618649379	4.23806856207269	0.11239409109929	0.737434787963546	0.944313928132934
Dmac1	Dmac1	-0.0144257620956583	4.04426244124378	0.112330777150156	0.737506023994095	0.944313928132934
Akt	Akt	0.0304460698420369	2.54023223165734	0.112308098812733	0.737531545362826	0.944313928132934
Prkar1a	Prkar1a	-0.0085349111808250	8.34200687822145	0.112303461278222	0.737536787136005	0.944313928132934
Mrpl13	Mrpl13	-0.0158147578268239	4.02597505057475	0.112296829652536	0.737544228228159	0.944313928132934
Lyplal1	Lyplal1	-0.0215413242801795	3.05189813527381	0.112186496142321	0.737668441457061	0.944376066494947
Dok3	Dok3	-0.0405017280811162	0.648680476102949	0.112118132051016	0.737745439632062	0.944377741898867
Lrpprc	Lrpprc	-0.0085764257708136	7.47347907971387	0.112033669585337	0.737840605395916	0.944402670652638
Stmn1	Stmn1	0.0171441698686946	11.5379101140319	0.111765311666716	0.738143235202935	0.944588362598736
Taf12	Taf12	-0.01423681037624876	3.6882192346711	0.111735380189934	0.73817701435784	0.944588362598736
Ibsp	Ibsp	-0.0523998496578371	0.0597951270728717	0.111659082973247	0.738263142051466	0.944588362598736
Aard	Aard	-0.0293882520561119	2.28359063038684	0.111615800655778	0.738311903669621	0.944588362598736
Mat2a	Mat2a	0.0185109525811411	6.98009953504807	0.11151642854247	0.738424252092646	0.944588362598736
Tmem41a	Tmem41a	-0.0195642043460654	3.64189344053405	0.111490999605401	0.738452997515174	0.944588362598736
Eya2	Eya2	-0.0546213087582031	2.42808380566078	0.11142408316941	0.738528626008109	0.944588362598736
Rap2c	Rap2c	-0.0094589794694527	6.20831375763526	0.111369616405727	0.738591333294323	0.944588362598736
Nsa2	Nsa2	-0.0108343006168624	6.20925515483997	0.111191423884393	0.738791761215671	0.944679477842702
Rmc1	Rmc1	-0.0111413144520939	5.53063708315321	0.111068961700653	0.738930404834511	0.944679477842702
Atp1b3	Atp1b3	-0.0108652044746108	6.55856832696054	0.110957521825242	0.739056621967056	0.944679477842702
Hadha	Hadha	0.00836550402778316	6.75630237831693	0.110898892900959	0.739179972189614	0.944679477842702
Cstf1	Cstf1	-0.0106059577171963	5.12679991163346	0.110621917626439	0.739437199400618	0.944679477842702
Manea	Manea	0.0190951361616745	3.79769042492291	0.110529592708984	0.739530661768843	0.944679477842702
Sbds	Sbds	0.0134305773487168	4.79712772528077	0.110505878687697	0.739568923593099	0.944679477842702
Zfx2	Zfx2	0.0192554311031408	4.25307684396694	0.110494231593819	0.739582160296426	0.944679477842702
Sbsn	Sbsn	-0.0493829659047042	0.944931884100821	0.110294697073385	0.739808876548542	0.944679477842702
Edn1	Edn1	-0.0509219921311224	0.155426083840688	0.110098301646957	0.740032248540532	0.944679477842702
Erl2	Erl2	-0.0178594869024742	3.80924214420707	0.110066852962326	0.740068037504794	0.944679477842702
Enpp6	Enpp6	0.0619334414425343	-0.331371120262493	0.109828362270636	0.740329233117379	0.944679477842702
Pla2g15	Pla2g15	-0.0122571392436927	4.91420795651331	0.109826147852708	0.740342150838189	0.944679477842702
Cdipt	Cdipt	-0.0113490736165677	6.712294861482	0.109816855288585	0.740352739827447	0.944679477842702

Ide	Ide	0.0113912973329012	6.09983067298018	0.109770432533367	0.740405546566539	0.944679477842702
P2ry12	P2ry12	0.0545135068855053	1.96494042084836	0.109759220609931	0.740418426349032	0.944679477842702
RGD1309	RGD130977	0.0195720270314628	2.91603229408504	0.109703553268838	0.740481889952096	0.944679477842702
Cspp1	Cspp1	0.0380620884973795	5.22480509270337	0.109699679922514	0.740486305259814	0.944679477842702
Ftx	Ftx	-0.0294634042281663	3.364385618677	0.109690914138312	0.740496300352133	0.944679477842702
Slc25a17	Slc25a17	-0.0102089724244433	5.55984671799872	0.109620825089316	0.740576234588036	0.944679477842702
Grk2	Grk2	-0.0081293162569644	7.49779119167989	0.109579437642488	0.740623448914674	0.944679477842702
Higd1b	Higd1b	-0.0362207216527759	1.31482220789719	0.109520824602285	0.740690230931202	0.944679477842702
Gtf2b	Gtf2b	-0.0142887214104094	4.39702938852984	0.109437237397692	0.74078574476221	0.944679477842702
Alg6	Alg6	0.0182584616825464	3.22543923803259	0.109369841889709	0.740862705400148	0.944679477842702
Plce1	Plce1	0.0259689803083376	4.16175522624776	0.109332524222737	0.740905226705342	0.944679477842702
Zc3hc1	Zc3hc1	0.0126831329642837	5.40913153120182	0.1091578749367	0.741104927379822	0.944679477842702
Slc5a7	Slc5a7	-0.0425007094717181	3.68269534864525	0.109138882555641	0.741126643431691	0.944679477842702
Trpc4ap	Trpc4ap	-0.0073508138399083	7.7580977174083	0.109124156427029	0.741143482805811	0.944679477842702
Krt1	Krt1	-0.0284511676466387	1.84834512351542	0.108966557800201	0.741322776292442	0.944679477842702
Msantd3	Msantd3	0.0117485857012151	4.8181747597221	0.108960037162667	0.741331239043028	0.944679477842702
Prrt1	Prrt1	-0.0164275095758677	6.63541434497283	0.108956850316411	0.741334771968562	0.944679477842702
Neurl1	Neurl1	-0.0322547733031116	4.11077274897132	0.108929600776008	0.741366076207747	0.944679477842702
Dbf4	Dbf4	0.0247212725143004	2.51273058789994	0.108891523159088	0.741409655564976	0.944679477842702
Snd1	Snd1	0.00714163761186663	7.53019446624096	0.108865800238385	0.741462021308306	0.944679477842702
Syt4	Syt4	0.0552596704171257	-0.462270905885147	0.108826526977055	0.741484093486383	0.944679477842702
Bcas3	Bcas3	-0.0095363905714902	5.05493668240016	0.108785790970778	0.741530753421187	0.944679477842702
Dmac2	Dmac2	0.0130978761697378	4.4603623207125	0.108775127825403	0.74154296756474	0.944679477842702
Scn11a	Scn11a	-0.0693529755579474	0.120475026872184	0.108725259536442	0.741600103891064	0.944679477842702
Casp3	Casp3	0.0108838009194788	7.49459014778431	0.108672604519613	0.741660446695675	0.944679477842702
Nfu1	Nfu1	-0.0142059888704438	4.92946442457279	0.108596769672801	0.74174738286148	0.944679477842702
[illegible]	[illegible]	[illegible]	[illegible]	[illegible]	[illegible]	[illegible]

Rita1	Rita1	-0.0128965509938605	5.29099222040077	0.0852517927434562	0.770302606566049	0.950778525080944
Mairdl	Mairdl	-0.0509578631909805	0.254865007602549	0.0851543639734871	0.770430211670661	0.950778525080944
Prok2	Prok2	-0.0605009424881112	-0.531623862082186	0.0849755236943616	0.770654636942087	0.950778525080944
Lztfl1	Lztfl1	0.0142478329579961	4.20933163100018	0.0849334199840825	0.770719881820637	0.950778525080944
Gsta6	Gsta6	0.0308173125564984	1.50130655599256	0.0846408567879635	0.77110407853518	0.950778525080944
Fars2	Fars2	-0.0138977140944785	4.66614889184917	0.0846163911536451	0.771136239645608	0.950778525080944
Ngly1	Ngly1	0.011082767984334	4.72312514110021	0.0845899962829484	0.771171994371267	0.950778525080944
Cep55	Cep55	-0.0280451790509651	1.81525312828726	0.084564399924183	0.771204601063191	0.950778525080944
Cdc42bpb	Cdc42bpb	-0.0073228204754977	8.67379518880334	0.0845248945365995	0.771256560531799	0.950778525080944
Prkab1	Prkab1	-0.011152794199842	5.23171235505696	0.0845028293971453	0.771285587442546	0.950778525080944
Tmtc2	Tmtc2	-0.0177719722602565	4.04083517783978	0.0844548322269279	0.771335582732707	0.950778525080944
Josd2	Josd2	0.0180332709727048	3.44230478618572	0.0844083942282623	0.771409864206572	0.950778525080944
Mapt	Mapt	0.00966079602647193	11.6345523623808	0.0843874320004971	0.771437460861804	0.950778525080944
Hnrnpul2	Hnrnpul2	-0.0096322668852853	7.55449056418033	0.0843779958222797	0.771449884705606	0.950778525080944
Spag1	Spag1	-0.0255285304982828	1.69315574251489	0.0842687302314644	0.771593800666996	0.950778525080944
Slc25a39	Slc25a39	-0.0106021075266885	5.98922311942234	0.0842663585156274	0.771596925624282	0.950778525080944
Hnrnpf	Hnrnpf	0.00945850549024068	7.82635221460567	0.0842635279437269	0.771600642053456	0.950778525080944
Adamts19	Adamts19	0.0342924495518242	2.64538946771813	0.084245525814361	0.771624363526638	0.950778525080944
Nrtn	Nrtn	-0.0398235281666463	1.17498250862243	0.084243944693068	0.771626460284495	0.950778525080944
Arg2	Arg2	0.013053695148885	4.73000217450603	0.0841040135620261	0.771810943719148	0.950911932930157
Btbd1	Btbd1	-0.0072176380856752	8.08606629013033	0.0840048755516136	0.771941746733565	0.950929052989399
Bend7	Bend7	0.0280388677358185	1.56322562923896	0.0839779639753786	0.771977268282211	0.950929052989399
Ormdl3	Ormdl3	0.0105618123031565	5.61001225996028	0.0839075463292218	0.772070244643611	0.95094969816568
Tmem63a	Tmem63a	0.0541713095746202	-0.278126428382239	0.0835595829688458	0.772530301521184	0.951392241987308
Fth1	Fth1	-0.0120488847719734	9.37144608388935	0.0835204946430395	0.772582046631033	0.951392241987308
Sbsfip	Sbsfip	-0.0248364906408974	2.51521680070368	0.0834099534642831	0.772729452051936	0.951478623926971
Arl6	Arl6	-0.0128973259271905	4.7806597973152	0.0833236892758507	0.772842777029636	0.951482644276435
Dnajc4	Dnajc4	0.0137686012052937	3.39414306660501	0.0832924190310109	0.772884234971681	0.951482644276435
Caln1	Caln1	0.0179815734956045	5.04919452269481	0.0831773474023976	0.773036869026078	0.951525810938706
Wdr31	Wdr31	-0.0144716347054642	3.39609787753525	0.0831510069407173	0.773071823794635	0.951525810938706
Myo10	Myo10	0.0169107019270336	5.78689306757967	0.0830595094109446	0.773193291023568	0.951551123509254
Pid1	Pid1	-0.0160020063390531	6.150604454449	0.0830206389544868	0.773244917949854	0.951551123509254
Rnls	Rnls	0.0184215648110687	2.49551136257131	0.0829374565223446	0.77335543476495	0.951593270173795
Ap1m2	Ap1m2	0.0519254228561724	0.669225285445239	0.0828064326173319	0.77352964015623	0.951679096706853
Acta1	Acta1	-0.0134422643162069	4.03473088568009	0.0827388947507586	0.77361949465802	0.951679096706853
Reep1	Reep1	0.0077623689495135	9.36570469677566	0.0826625165465629	0.773721158538713	0.951679096706853
Chm	Chm	0.0106538136435818	4.6563359647468	0.0826001837652086	0.773804164825363	0.951679096706853
Aifm2	Aifm2	-0.0111230758447412	4.56213093226191	0.0825251480575417	0.77390413212283	0.951679096706853
Usp22	Usp22	-0.0091406987252482	9.19657379585884	0.0825121283536561	0.773921482800923	0.951679096706853
Mycbp	Mycbp	-0.0285888411977686	1.61658909181964	0.0824465204057238	0.774010670905176	0.951679096706853
Mrpl27	Mrpl27	-0.0160076837101744	5.24709747495367	0.0823700559086973	0.774110911701608	0.951679096706853
Mxra8	Mxra8	-0.0264562817015113	3.45011359407002	0.0823694954880096	0.774111658275134	0.951679096706853
Znhit6	Znhit6	0.0104751964682113	5.55007749840011	0.0823116257069287	0.774188869528407	0.951680248778909
Slc39a11	Slc39a11	-0.0095212319569019	8.21999632989286	0.0820503696995303	0.774537805236309	0.951840866736933
Atrip	Atrip	0.0209630079407232	4.86934289778414	0.0820424509275597	0.774548390994256	0.951840866736933
Tubgcp3	Tubgcp3	0.0111952185755922	5.72319202314898	0.0820424481969937	0.774548394644552	0.951840866736933
Oxsr1	Oxsr1	0.00750885499216097	6.90908564523331	0.08194308358059	0.774681271601716	0.951844566725636
Kif18a	Kif18a	-0.0277793724717256	1.92266910559649	0.0818522779609552	0.774802779090392	0.951844566725636
Gpd1l	Gpd1l	0.00895619598379872	6.22316755032127	0.0818374133026794	0.774822676518808	0.951844566725636
Gemin4	Gemin4	0.0129258308894676	3.99779280276633	0.0817632663760932	0.774921956790901	0.951844566725636
Map4k1	Map4k1	-0.0166052493191985	2.78627504032445	0.0815950152722493	0.775147420199456	0.951844566725636
Nipal2	Nipal2	0.0193237056941637	2.63985397120986	0.0815795745980452	0.775168123929812	0.951844566725636
Spidr	Spidr	0.0275536587202772	1.53717719998198	0.0815704638286832	0.775180341159418	0.951844566725636
Csf2ra	Csf2ra	-0.024847224057843	3.8874485903182	0.0815510512709352	0.775206375195857	0.951844566725636
Apba3	Apba3	-0.0165322119055947	4.10710199733635	0.0815121224934501	0.775258592389535	0.951844566725636
Kcnj12	Kcnj12	0.0185511556886532	2.95985405187663	0.0814243927371763	0.775376318234164	0.951844566725636
Usp7	Usp7	0.0118364860288228	7.43689364032739	0.0814137737673803	0.775390572749809	0.951844566725636
Lrp11	Lrp11	-0.0129057268930946	7.6217531282355	0.0813233037389583	0.775498624698212	0.951883553289615
Exoc8	Exoc8	0.0110220929456144	5.2971049529234	0.0812131359703869	0.775660088844097	0.951915005742666
Slc30a1	Slc30a1	-0.0115967682034173	5.01317963858739	0.0810950623959954	0.775818864965	0.951915005742666
Irf3	Irf3	-0.0264355931267366	5.249537060191	0.0809993426533424	0.77594767313708	0.951915005742666
Uros	Uros	0.0187617095643903	2.80376372203082	0.0809912887261177	0.775958514900004	0.951915005742666
Ostm1	Ostm1	-0.0100009500288544	5.60102269843963	0.0809523068387108	0.776010998454277	0.951915005742666
Tmem192	Tmem192	-0.0154987462866531	3.43174034168619	0.0809062552762896	0.776073017921138	0.951915005742666
Zfp639	Zfp639	-0.0104504634604483	4.57380919319443	0.0808626751505344	0.776131726578912	0.951915005742666
Zswim8	Zswim8	0.0111785795690289	7.64352406847035	0.0808605455227109	0.776134595930123	0.951915005742666
Dlg3	Dlg3	0.0075303246660464	7.26110920913347	0.0807484204849702	0.77628572515621	0.952006781283078
Synj2	Synj2	-0.0274604600886901	3.61171340520278	0.0806112540399937	0.776470761104844	0.952049743213192
Arg1	Arg1	0.0252554017342233	2.63549183101272	0.0806043296795593	0.776480106495496	0.952049743213192
Mrpl32	Mrpl32	-0.0165895969385183	3.45505639461125	0.0805527971916149	0.776549670409422	0.952049743213192
March5	March5	-0.0067145657218262	6.68227334574365	0.0803764238128934	0.776787939685006	0.952122946075089
Itgbl1	Itgbl1	0.0225455090014329	2.96273593769207	0.0802803827377545	0.776917803884001	0.952122946075089
Mier3	Mier3	0.00980282925314338	5.56326179143758	0.0801876762201025	0.777042832766769	0.952122946075089
Sez6	Sez6	-0.010958524820576	9.14373661765853	0.0801810382213546	0.777052223234103	0.952122946075089
Cd55	Cd55	-0.0278449251707586	2.42528199195467	0.0801324502273957	0.777117998587421	0.952122946075089
Rbks	Rbks	0.0238498685594078	2.19717118977369	0.0801132981120674	0.777143921428217	0.952122946075089
Serinc4	Serinc4	0.0488129611378528	0.0665122357367751	0.0800843883278617	0.777183082941474	0.952122946075089
Alg5	Alg5	-0.0116376846471702	4.16885756771543	0.079975341901509	0.777330829448794	0.952122946075089
Dhx40	Dhx40	-0.0097271775976482	5.36889629724275	0.0799027680708591	0.777429219701139	0.952122946075089
Lrrn1	Lrrn1	-0.0130545658847929	5.9449076500212	0.0798298816444147	0.777529710404189	0.952122946075089
Slc12a6	Slc12a6	0.0124059292495975	6.61510622384807	0.0797586774858096	0.777624302990875	0.952122946075089
Trpc6	Trpc6	0.0290573427429169	1.4109268880574	0.0796761364766212	0.777736781104515	0.952122946075089
Gfm1	Gfm1	-0.0108573524754715	4.9620181148169	0.0796751865356473	0.777738071125505	0.952122946075089
Gpatch1	Gpatch1	-0.0145680345961589	4.27403979668522	0.0796144600887558	0.777820563770772	0.952122946075089
Crmp1	Crmp1	0.00994525310402848	11.2987776340535	0.0795819181201693	0.777864783672069	0.952122946075089
Chst4	Chst4	-0.00882335232989378	5.94740279329829	0.079530811857268	0.777925958861636	0.952122946075089
Bicdl1	Bicdl1	-0.0136267811767503	6.2387384446759	0.0795306215758939	0.777934494464004	0.952122946075089
Ezr	Ezr	0.0209435854299761	6.07483050068574	0.07944776690411	0.778047179201818	0.952122946075089
Cntnap5a	Cntnap5a	0.02485201772860358	2.66810507949105	0.0793573837491834	0.778170160263171	0.952122946075089
Alkbh3	Alkbh3	0.0138575934616891	4.484710900188	0.0793468755654914	0.77818446326956	0.952122946075089
Ppif	Ppif	-0.012237886374126	4.53016204870957	0.0793267249247265	0.778211894211249	0.952122946075089
Wdr45b	Wdr45b	0.00990291438726818	5.0315224681775	0.0791232340443599	0.778489111221287	0.952357973449297
Poglut3	Poglut3	-0.0152101182356438	4.63316992859833	0.0790736996503369	0.778556650571678	0.952357973449297
Ttl	Ttl	-0.0082508837032900	6.13342748304261	0.0788705034096884	0.778833944489363	0.952595658172123
Api5	Api5	0.00755467666552041	6.76808099784456	0.0788194650673333	0.778903655099143	0.952595658172123
Ubald1	Ubald1	-0.0108697703478723	6.04088629381498	0.0787093564966383	0.779054129537224	0.952686305423498
Eepd1	Eepd1	-0.0134587108076011	4.64532700048442	0.0783568036844713	0.779536690604485	0.953104871809743
Gorasp2	Gorasp2	0.00697312228939757	6.80682082465885	0.0782705052674473	0.779654994647428	0.953104871809743
Mettl14	Mettl14	-0.0145596521033263	4.76010530324266	0.0782485298055065	0.779685130650994	0.953104871809743
LOC100909	LOC100909	0.032329723605066	1.68120702022353	0.0782362549445708	0.779701965741929	0.953104871809743
Gk	Gk	0.0121882329646778	4.21027808387538	0.0779627950951394	0.780077388433361	0.953372550603199
Golga3	Golga3	0.0102496251931388	5.71475976084216	0.0779216523385031	0.780133933161673	0.953372550603199
Ypel3	Ypel3	-0.0147960140952103	6.44775837033586	0.0779050014170366	0.780156822002578	0.953372550603199
Acot3	Acot3	-0.0167351276124086	3.98688923507557	0.0777508206713518	0.780355113643072	0.953372550603199
Cbx3	Cbx3	-0.0083264003781559	7.93227092701603	0.0777347508269131	0.780391004871026	0.953372550603199
Ttc33	Ttc33	0.0114745962066285	4.37675334134817	0.0775999647281137	0.780576628213328	0.953372550603199
Lpxn	Lpxn	0.0212150767845176	1.97845411321326	0.0775414770597465	0.780657191769285	0.953372550603199
Ipcef1	Ipcef1	0.0201782398096009	3.13801286972978	0.0775198203038201	0.780687040866037	0.953372550603199
Acaa1b	Acaa1b	-0.0279685139000667	1.93526676763812	0.0775191740617061	0.780687931638237	0.953372550603199
Pnma8b	Pnma8b	-0.008822501371029	8.58788194695663	0.0774832790129657	0.780737415222924	0.953372550603199
Notch3	Notch3	0.0155652002900279	6.47708743189619	0.077417248351594	0.780828474911621	0.953372550603199
Tnrc6a	Tnrc6a	0.0219145466001134	6.92254975153782	0.0774104279051437	0.780837868248545	0.953372550603199
Rnf34	Rnf34	0.00866829735359243	5.61778670640739	0.0772856165064226	0.781010126937598	0.953489564951116
Hnrnpr	Hnrnpr	0.00737985560827132	7.00073052634785	0.0769384585451007	0.781490005920167	0.953557941007009
C2cd5	C2cd5	0.0181702206151267	6.72508377289757	0.0769015900354475	0.781541037965396	0.953557941007009
Gzf1	Gzf1	0.0107330692968661	4.65016812860058	0.076876552983486	0.781575687004044	0.953557941007009
Ager	Ager	-0.0407589145493948	2.55306390687728	0.0768615017885139	0.781596541605353	0.953557941007009
Ppp1r37	Ppp1r37	-0.0064426920803391	7.22392528577455	0.0767370013852089	0.781769016380432	0.953557941007009
Itgb3bp	Itgb3bp	-0.0194213870372974	2.79310219692981	0.0767271004910768	0.781782738915998	0.953557941007009
Pak3	Pak3	0.0168015106455433	6.64809170975641	0.0766095426020144	0.781945745665272	0.953557941007009
Gmfg	Gmfg	0.0240767097238341	1.95383736198138	0.0765098600495291	0.782084027428395	0.953557941007009
Kcnmb4	Kcnmb4	0.0136427196557249	5.66821875508395	0.0765093498812961	0.782084780028448	0.953557941007009
Mmp9	Mmp9	-0.060572391841927	-0.554711458339281	0.0764824721461369	0.782120705707407	0.953557941007009
Tmem106c	Tmem106c	0.0131882412587393	4.08249986556457	0.0764545095314904	0.782147325389838	0.953557941007009
Pxmp2	Pxmp2	-0.0220552148459873	2.02087186616807	0.0764440100780774	0.782175502882443	0.953557941007009
Slc35g3	Slc35g3	0.0422710349053737	-0.155880007427133	0.0764412651373263	0.782179315094408	0.953557941007009
Kcnk1	Kcnk1	0.016191538370554	4.57364902620936	0.0763958926592932	0.782242200609483	0.953557941007009
Hcrtr2	Hcrtr2	0.0194951191402728	2.2697196816432	0.0763844730718546	0.782258205037094	0.953557941007009
Lrif1	Lrif1	0.0200500954116831	3.12038351902059	0.0763135632617846	0.782356750286592	0.953557941007009
Col23a1	Col23a1	0.0224234772947061	4.29205026250955	0.0763073670726442	0.782365363636191	0.953557941007009
Katna1	Katna1	0.0155014425802524	3.85271968115029	0.0761402658032324	0.782597793938646	0.953671068093712
Acot6	Acot6	-0.0552452871599671	-0.483813205682343	0.0761307412333707	0.782611055448868	0.953671068093712
Cdkn2aipnl	Cdkn2aipnl	0.0111908581192212	5.56713528747647	0.0760380507466891	0.782740105775465	0.953689177742435
Mbnl2	Mbnl2	0.0169340394967001	5.15552236470309	0.0760103940701506	0.782778782500623	0.953689177742435
Surf6	Surf6	0.009390322751828533	5.62219915664863	0.0759450697734929	0.783009069922374	0.953815155947286
Gatc	Gatc	0.00840797314500448	5.6059437194483	0.0757793459072954	0.783102150878639	0.953815155947286
Phka1	Phka1	0.0106290385823265	4.92493607630699	0.0757716302335467	0.783111521802036	0.953815155947286
Nudt21	Nudt21	-0.0080828788216004	6.54074900039114	0.0756012743759271	0.783349382687193	0.953976825514851
Edc4	Edc4	-0.0099882158715657	7.29749618198223	0.0755670703222648	0.783397175140271	0.953976825514851
Ank1	Ank1	0.0160007997949855	5.22933614164017	0.0754315248603774	0.783586683849643	0.954114477836835
Lrrc47	Lrrc47	-0.0084061371665655	6.52619363851146	0.0752020253208912	0.783907968699875	0.954412542491212
Vmac	Vmac	0.0256686366132141	1.93713605712394	0.0750517332506178	0.784118655132614	0.954575805960788
Rilpl2	Rilpl2	0.0149340885540074	4.26042675225498	0.0748785909074883	0.784361654538745	0.954679120755176
Utp25	Utp25	0.0143289229658176	4.54912197756009	0.0748549612079188	0.784394841431164	0.954679120755176
Npy1r	Npy1r	0.0119456615621586	5.07507324766086	0.0747891295029603	0.784487328920674	0.954679120755176
Sh3rf1	Sh3rf1	0.0120006435881167	6.03359408852172	0.0747389135327126	0.784557907157176	0.954679120755176
Brpf3	Brpf3	-0.0124032113827286	4.99304982248128	0.0747188203071983	0.784586014604759	0.954679120755176
Pdk3	Pdk3	-0.0126428885691542	4.47941537088459	0.0743494017984716	0.785106231007901	0.955218960924972
Ighmbp2	Ighmbp2	0.00881119993778165	5.26208692790017	0.0742418085582912	0.785257964144052	0.955232679915003
Pcdh15	Pcdh15	-0.0265906267399826	3.58552035369914	0.0742328340344045	0.785270625782494	0.955232679915003
Mirlet7b	Mirlet7b	-0.0509837310665724	-0.314570427280182	0.0741383130495166	0.785404029652539	0.955301821185204
Polr1g	Polr1g	-0.0166280516877462	3.7100370103875	0.0739548070713099	0.785663288014904	0.955365586255821
Slc27a3	Slc27a3	0.00847015922370564	6.1259682324383	0.0739536286889475	0.785664953941535	0.955365586255821
Rnaseh1	Rnaseh1	-0.0194299470750517	3.46383797416375	0.0738182150612658	0.785856486662929	0.955365586255821
Tmem101	Tmem101	0.0110332205132095	4.24599179653718	0.0737010069552682	0.786022423093978	0.955365586255821
Plekhg5	Plekhg5	0.0143164477224561	4.82709083087386	0.0736289789516604	0.786124466649524	0.955365586255821
Chrd	Chrd	-0.0260871230571805	4.19515659089441	0.0735787695968071	0.78619563063746	0.955365586255821
Cuedc2	Cuedc2	-0.0163530523689033	2.92730635460603	0.0734207872143884	0.786419717038467	0.955365586255821
Ubxn4	Ubxn4	0.00657817271061686	6.79073961456976	0.073381898708881	0.786474917209227	0.955365586255821
P2rx6	P2rx6	0.0289540997798936	2.01255693110768	0.0733801182211096	0.786477444892996	0.955365586255821
Pcgf3	Pcgf3	0.00958723298807482	5.58711242220041	0.0733362132057636	0.786539785417111	0.955365586255821
Ccdc92	Ccdc92	-0.0099811993437306	5.84919892375247	0.0733228974623046	0.786558696236761	0.955365586255821
Atg5	Atg5	-0.0117211630187935	5.06484944442738	0.0733050923498269	0.78658398590695	0.955365586255821
Gmnn	Gmnn	-0.0193189694348583	3.02030099257201	0.0732458097249318	0.786668006897908	0.955365586255821
Astn1	Astn1	0.011412170203205	8.43109194132355	0.0732117550801421	0.786716611848184	0.955365586255821
Capns1	Capns1	0.0110610863102681	7.80130623925631	0.0732091974630436	0.786720247340421	0.955365586255821
Aftph	Aftph	-0.0071317067715719	6.36616350666035	0.0732058952113519	0.786724941266465	0.955365586255821
Chtop	Chtop	0.00623529330212796	7.89680947523357	0.0731703522396714	0.786775471552688	0.955365586255821
Cyp2d4	Cyp2d4	-0.0281826885437726	1.32222628486296	0.0730614623883241	0.78693035843856	0.955365586255821
Lingo2	Lingo2	-0.019518223731927	4.44774829394427	0.0730277036679318	0.786979270837996	0.955365586255821
Polr3h	Polr3h	-0.0122547068599181	4.70054077281645	0.0730210612110227	0.786987857300502	0.955365586255821
Gsr	Gsr	-0.0094182715635507	5.827762294217	0.0728593561104134	0.787218166660811	0.955526768524722
Ak2	Ak2	0.0138287032668868	4.16758689844066	0.0728203249431072	0.787273798223463	0.955526768524722
Cyp2s1	Cyp2s1	-0.0173512193005283	2.85788142353459	0.0727534285851998	0.78736918320956	0.95554418605686
Gpbp1l1	Gpbp1l1	0.012761997377422	4.11389118503248	0.0725906705924828	0.78760145056201	0.95554418605686
Cdk17	Cdk17	-0.0067042111750568	7.12979800060269	0.0725473869647928	0.787663266420333	0.95554418605686
Gstm4	Gstm4	-0.0154544127426302	4.33112105090012	0.0725026450956534	0.78772718567035	0.95554418605686
Stk32a	Stk32a	0.0349272872659096	0.937016844437513	0.0724536767560551	0.787797167200728	0.95554418605686
Slc6a5	Slc6a5	-0.103453975796664	2.16301647271921	0.0724529144823856	0.787798256292024	0.95554418605686
Dcun1d2	Dcun1d2	-0.012960658087249	3.6122308896879	0.0723558135585307	0.787937101277906	0.95554418605686
Emd	Emd	0.0106721919507474	5.63909824325604	0.0723329489908622	0.787969808026643	0.95554418605686
RGD1310	RGD131035	-0.0095405474803055	6.67309731200169	0.0723121082886067	0.787999627996323	0.95554418605686
Cdkn3	Cdkn3	0.0238699853090262	2.08841930335825	0.0721721889661967	0.788198506870263	0.95554418605686
Psma6	Psma6	-0.0101612108722168	6.99236937023683	0.0721109581395467	0.788287563959887	0.95554418605686
Fer	Fer	0.0113676622569614	4.74893262803889	0.0720065214736829	0.788437367063468	0.95554418605686
Kcnh3	Kcnh3	0.0168879154989648	6.09403224918876	0.0719923636836757	0.788457598567716	0.95554418605686
Trub2	Trub2	-0.009755942387732	5.08032735760882	0.0719423616542514	0.7885294168265	0.95554418605686
Rnf180	Rnf180	-0.0081390106201200	6.05005769649244	0.0718730400073326	0.788628906198786	0.95554418605686
Gnai2	Gnai2	-0.0090323575941227	8.56403933754885	0.0718625218815703	0.78864400614559	0.95554418605686
Ovca2	Ovca2	-0.0178979798079085	2.93382285496416	0.0718320422135363	0.788687769811112	0.95554418605686
Prrx1	Prrx1	-0.0139180838429173	3.48707395220513	0.0717949913058558	0.788740901986469	0.95554418605686
Pax6	Pax6	-0.024578772546419	3.85337346029628	0.0717438775017314	0.788814415530849	0.95554418605686
Plppr2	Plppr2	-0.0071881311049288	8.7704518785979	0.0716618389640187	0.788932238007335	0.95554418605686
Tnfrsf1b	Tnfrsf1b	-0.0306770295112801	1.13941643673248	0.0716541891856508	0.788943235460752	0.95554418605686
Tfg	Tfg	0.00690778322546368	6.65156185453185	0.0715783658541724	0.789052393025396	0.95554418605686
Arnt2	Arnt2	0.00711894221251355	8.82771102386648	0.0715291590068148	0.789123200918832	0.95554418605686
Mettl9	Mettl9	-0.0075892577402934	7.07226792986054	0.071485658494023	0.789185817781689	0.95554418605686
Camlg	Camlg	0.00967491112275778	5.6514493526459	0.0714738922415989	0.789202760063792	0.95554418605686
Tug1	Tug1	0.0096276293058032	6.96654471746032	0.0712054803144184	0.789589621343469	0.955849908219607
Bbs10	Bbs10	-0.012266480425821	3.62670267602124	0.0711924091160725	0.789609480750701	0.955849908219607
Ccdc167	Ccdc167	0.0189797490070686	2.06968392378482	0.0710997609306858	0.789742208744093	0.955919047196357
Dhx35	Dhx35	0.0123635112734791	4.11326044928207	0.0710378332269102	0.789831646970456	0.955934567780617
Ripor1	Ripor1	0.00846632110365978	7.14643152751599	0.0706591027222672	0.790379523948034	0.956286223489773
Pten	Pten	-0.0104469992740387	6.37182522689486	0.0704384753291833	0.790699427423132	0.956286223489773
Psmg1	Psmg1	0.0105421807918593	5.45798016119774	0.07036009301911607	0.790813296213029	0.956286223489773
Rnf19a	Rnf19a	0.00728984605632378	6.30092542605316	0.0703225408491122	0.790867738845868	0.956286223489773
Kdm4c	Kdm4c	-0.0134076893778314	4.82880454843389	0.0703049842264676	0.790893240152474	0.956286223489773
Spem1	Spem1	0.0192952814293907	2.37612306824832	0.0702082245487148	0.791033846491428	0.956286223489773
Tnfaip1	Tnfaip1	0.00573986424934351	7.11487653004461	0.0701868153238294	0.791064971217197	0.956286223489773
Alad	Alad	0.0114731041240264	4.99345028801907	0.0701728529078309	0.791085272676316	0.956286223489773
Zmf4	Zmf4	0.0262889921885689	1.48983570521111	0.0701285109524932	0.791149760235001	0.956286223489773
Ubiad1	Ubiad1	0.0107041251528093	4.58307180445123	0.0701183348372751	0.791164562678786	0.956286223489773
MGC1162	MGC116202	0.00677226915187001	8.17087392501175	0.0700696029885863	0.791235465225203	0.956286223489773
Adtrp	Adtrp	-0.0400845106711172	0.180477264143419	0.0700647811518138	0.79124248222101	0.956286223489773
Fhl4	Fhl4	-0.0319360780987882	0.362970127602656	0.0699472413717999	0.791413612043762	0.956286223489773
Emp2	Emp2	-0.0265074680557153	2.4438801184892	0.0699450677230828	0.791416778174827	0.956286223489773
Mcart1	Mcart1	0.00757706905366671	6.18379183826668	0.0698733461433747	0.791521277178138	0.956286223489773
Sfswap	Sfswap	-0.0164557379628172	6.83291031454676	0.0698658235813178	0.791532226269669	0.956286223489773
Sec61g	Sec61g	-0.0185670848362963	5.66657642304735	0.0698184260810137	0.791601335012732	0.956286223489773
Klhl23	Klhl23	-0.0066502833447442	6.885759826293	0.0698057532937574	0.791780278792536	0.956286223489773
Hmg20b	Hmg20b	0.0160570112751867	3.59148471547989	0.0696867512745385	0.79179341674253	0.956286223489773
Itgb1	Itgb1	0.00955584529721337	7.19363244576564	0.0696830297537474	0.791798848261829	0.956286223489773
Uchl3	Uchl3	0.010078269307446	5.44210125436838	0.0695866202826786	0.791939599032512	0.956286223489773
Dkk3	Dkk3	-0.0139804287323185	6.47053481971612	0.0695371929603823	0.792011821792583	0.956286223489773
Ggact	Ggact	-0.0230968804541983	2.57653810679155	0.0695268874266901	0.792026880558301	0.956286223489773
Snrf3	Snrf3	0.00880155117597175	8.27978451226541	0.0694758865904147	0.792101349046339	0.956286223489773
Ccdc39	Ccdc39	0.0227472720178784	4.99752028348652	0.0694463972124653	0.792144536448022	0.956286223489773
Hnrnpk	Hnrnpk	-0.0076043074397490	9.53495449132954	0.0694415856903845	0.792151571958896	0.956286223489773
Tnfsf12	Tnfsf12	0.017158155887325	2.69621216016866	0.0692737534060228	0.792397143243573	0.956286223489773
Ptov1	Ptov1	-0.0097469094938422	8.74426542234065	0.0692593226794642	0.792418273048388	0.956286223489773
Tmem25a	Tmem25a	0.00910670858932005	7.126258144351	0.0691265637813601	0.79261277223899	0.956286223489773
Agap1	Agap1	-0.0092436813102456	8.02363592234775	0.0691261499652027	0.792613378829444	0.956286223489773
Ddhd1	Ddhd1	0.0092661507214313	5.98237755802597	0.0690423172331123	0.792736301710935	0.956286223489773
Asrnd1	Asrnd1	0.00785807761015034	6.05528482519731	0.068890512424197	0.792959094419017	0.956286223489773
RGD1310	RGD131055	0.00994184032611413	5.10664871459021	0.0687905258471417	0.793105986068015	0.956286223489773
Prrxl1	Prrxl1	-0.0264996719685015	2.09913071065308	0.0687855902361179	0.793113234250267	0.956286223489773
Tmbim4	Tmbim4	0.0129777655773343	4.42489113799216	0.0687494272398606	0.793167860194073	0.956286223489773
Stat1	Stat1	-0.0166239648715157	3.95322889176409	0.0687104638426348	0.79322367884163	0.956286223489773
Vta1	Vta1	0.011043296696608	6.27303158127843	0.0686546836025173	0.793305723997186	0.956286223489773
Fig4	Fig4	0.00746859509745653	6.00867960981775	0.0686489996459372	0.793314086224522	0.956286223489773
Spag7	Spag7	-0.0099066427406682	5.09776557054191	0.0686000220983232	0.793386158205112	0.956286223489773
Cthrc1	Cthrc1	0.0227705968508687	2.40328856817976	0.0685314083736088	0.793487171541234	0.956286223489773
Tifab	Tifab	0.0365331182266271	1.17808999300013	0.0685195885010899	0.793504578248844	0.956286223489773
Stxbp5l	Stxbp5l	0.0205503385808847	4.21301139694508	0.0684928682569787	0.793558665087399	0.956286223489773
Thg1l	Thg1l	0.0150287577821571	2.90424770654302	0.0684816746112773	0.793560423519849	0.956286223489773
Ddost	Ddost	-0.0087571562723755	7.10061138683993	0.0684773247764809	0.793566821665866	0.956286223489773
Zfp335	Zfp335	0.0157407074400158	5.82170149066068	0.0683360075080373	0.793775137689868	0.956286223489773
Rab32	Rab32	-0.0346094210078949	0.5140198909247	0.0682203396308978	0.793945807029062	0.956286223489773
Cct3	Cct3	-0.0087476204077039	8.28657025855142	0.0682199836206081	0.793946332565006	0.956286223489773
Sdhaf1	Sdhaf1	0.0140660332379997	6.05229208416981	0.0681422162195986	0.79406116652197	0.956286223489773
Clint1	Clint1	0.00980102122796355	5.16809867004075	0.0681418953623947	0.794061640455445	0.956286223489773
Pcsk7	Pcsk7	-0.0132586047392961	5.29943637312703	0.0680948902568872	0.794131083889181	0.956286223489773
Taok2	Taok2	0.00715517516666815	7.51900661575882	0.0679907454076343	0.794285034508143	0.956286223489773
Cenpq	Cenpq	0.0256744726679962	1.6097286692103	0.0679583748840997	0.79433291141684	0.956286223489773
Cox6a2	Cox6a2	-0.0656213565014881	0.158276576365276	0.0679401610594255	0.794359855526127	0.956286223489773
Ppa1	Ppa1	-0.0114144168865255	6.78085459650518	0.0679230560330524	0.794385162878045	0.956286223489773
Erh	Erh	0.0116436202482133	7.25393214400411	0.0679420567756035	0.794505046719719	0.956286223489773
Abhd11	Abhd11	0.0118978632650006	4.5575565693811	0.0677956835970264	0.794573720974281	0.956286223489773
Gal3st4	Gal3st4	0.0218996508970203	1.84755340197549	0.0677361637435538	0.794661897110717	0.956286223489773
Zfp483	Zfp483	0.0246822690410398	3.70503374165685	0.0677348252545897	0.794663880500776	0.956286223489773
C4a	C4a	0.0341837770778055	2.8809644941589	0.0677128795140024	0.794694920893049	0.956286223489773
Acadsb	Acadsb	-0.0145915746229646	4.67082095301613	0.0675345545213446	0.794960880357912	0.956286223489773
Epb41l5	Epb41l5	-0.0192418892190135	3.47828958759647	0.0675047357845813	0.795005241491179	0.956286223489773
Lcat	Lcat	-0.0239645377433925	2.04925929275728	0.0674165665353793	0.795136075683624	0.956286223489773
Htr4	Htr4	0.0345692665497497	1.01711226047201	0.0674089726113891	0.795147357180563	0.956286223489773
Vps50	Vps50	-0.0065478773665961	6.60757819852297	0.0673763359820037	0.795192877684456	0.956286223489773
Lin7c	Lin7c	0.00917788039616521	6.28128784195276	0.0673714396757958	0.795203125892513	0.956286223489773
Ppp1r21	Ppp1r21	-0.0062609358747528	6.15597397885939	0.0673435640985858	0.795244555846836	0.956286223489773
Camta1	Camta1	-0.0113674405120322	8.47761775120172	0.0673035090795651	0.795304103470183	0.956286223489773
RGD1560	RGD156028	-0.0420225328085434	1.03304426520415	0.0672455108541763	0.795390358812095	0.956286223489773
Mef2d	Mef2d	-0.0104422089491746	6.32261068895356	0.0672318853470983	0.795410529748887	0.956286223489773
Cxadr	Cxadr	0.00759453382034925	7.77190420573808	0.0671767482235205	0.795492675195827	0.956292716872491
Pcdhga7	Pcdhga7	-0.0141497654036804	4.6180458882537	0.0671019887423928	0.795603980129492	0.956323437958398
P2rx7	P2rx7	0.0202539045143243	3.13723225348811	0.0669883215731204	0.795773226984715	0.956339470617755
Tyw5	Tyw5	-0.0142858864881563	2.4025220527524	0.0669297845740164	0.795860613051087	0.956339470617755
LOC6893	LOC689316	-0.0179968818382862	2.38584578548864	0.066857988872616	0.795952792199892	0.956339470617755
Caskin2	Caskin2	-0.00998209941527939	4.99387055472965	0.0667841307044625	0.796077953895113	0.956339470617755
Minar1	Minar1	0.0207032586075512	3.80694716423883	0.0666874146974808	0.796222410571383	0.956339470617755
Ing4	Ing4	0.00896990771569066	6.13018758571243	0.06665185470363	0.796255628313475	0.956339470617755
Mbd6	Mbd6	0.033072987280022	5.88428332381365	0.0665857225033335	0.796374420325656	0.956339470617755
Stip1	Stip1	-0.0072324831368290	7.71271327856059	0.0665828171271627	0.796378765107999	0.956339470617755
Psmb9	Psmb9	0.0350877063153034	0.736824216529161	0.0665571039351533	0.796417221758061	0.956339470617755
Tomm70	Tomm70	0.00715720189626463	7.7240700096432	0.0665403211429449	0.796442326372242	0.956339470617755
Psmf1	Psmf1	0.00992522269294811	5.05259747557096	0.0665342922048922	0.796451345611052	0.956339470617755
Mtpap	Mtpap	-0.0127687476502408	4.49873854269747	0.0663764413962724	0.796687644308471	0.956531152620838
Uxs1	Uxs1	0.00802111567187725	5.77676064523007	0.0663111771049429	0.796785430859615	0.956556511193631
Pon2	Pon2	-0.0138322175450845	4.61187100567485	0.0660362158181158	0.797197974341142	0.956868868625366
Odf2	Odf2	-0.0121282293931574	6.05191340053701	0.0658838550166022	0.797426815742082	0.956868868625366
Ero1b	Ero1b	0.0269728649050947	3.20897402610707	0.0658305772665813	0.797507108908252	0.956868868625366
Abcc10	Abcc10	0.016958778972525	4.19008607827081	0.0657727443880276	0.797594138676802	0.956868868625366
Wdr75	Wdr75	0.00959655670145124	5.01289340928214	0.0657497693064428	0.7976287248254	0.956868868625366
Flt3lg	Flt3lg	0.0338951519272535	0.640815867280152	0.0657231995775334	0.797668730302858	0.956868868625366
Vps9d1	Vps9d1	-0.0167562364194035	6.44001873581224	0.0657072614965557	0.797692732068827	0.956868868625366
Plcd1	Plcd1	-0.0208649413899678	4.2783196868498	0.0657006584837063	0.79770267670736	0.956868868625366
Gsap	Gsap	0.0261894180292565	1.58411216405967	0.065586555499145	0.797874608648508	0.956868868625366
Dtymk	Dtymk	0.0107750477254868	5.03885936478923	0.0655496987439577	0.797930178961255	0.956868868625366
Irf7	Irf7	0.0524539519327309	0.0780092829968243	0.0655435083707516	0.797939514051873	0.956868868625366
Tomm40l	Tomm40l	-0.0124373418184273	3.21139234757481	0.0654058450807667	0.798147080974846	0.956868868625366
Apobec3	Apobec3	-0.0467776442737953	-0.338712948345023	0.0653550368061531	0.798223954581279	0.956868868625366
Raver2	Raver2	0.0165982703457307	4.54637257860137	0.0653536472657237	0.79822605329168	0.956868868625366
Fggy	Fggy	0.0212005847127247	1.84781940415644	0.0652781233281559	0.798340157624568	0.956868868625366
Ccdc117	Ccdc117	-0.0105585127793918	3.99553454347048	0.0652747345741744	0.798345279126211	0.956868868625366
Rcor2	Rcor2	0.0130924813237471	5.40987548200702	0.065216461348909	0.798433370954435	0.956868868625366
Naca	Naca	-0.0102177786172388	8.12308928800019	0.0651854280974362	0.798480301132349	0.956868868625366
Zfp518a	Zfp518a	0.0181258558050652	3.91031888523778	0.0651705942776875	0.798502737847825	0.956868868625366
Zmynd10	Zmynd10	-0.0273803438308882	4.3536394698336	0.0649307917968213	0.798856278213454	0.956988260881686
Rhbdf1	Rhbdf1	-0.0123689794724949	4.55939521817007	0.0649130447535455	0.798892725671588	0.956988260881686
Klhdc2	Klhdc2	-0.0078107302793585	7.81111232686753	0.0649061282410815	0.798903193500142	0.956988260881686
Ghdc	Ghdc	-0.0237218058629835	2.82889366788737	0.0648868703837294	0.798932254003821	0.956988260881686
Evl	Evl	-0.0067952282443455	9.20032058425737	0.064851611480286	0.79898587109117	0.956988260881686
Ikzf5	Ikzf5	-0.0097823528170739	4.8439946745674	0.0647568150303144	0.79912969414055	0.957069564916821
Eif3m	Eif3m	-0.0094283488944489	6.57627705223925	0.0646770935489513	0.799250732576786	0.957121738205256
Chka	Chka	-0.0095704704589060	5.865643767281	0.0646077147283525	0.799356132750149	0.957156091009844
Tirrd1	Tirrd1	-0.0105708248786385	5.68985231158175	0.064526185880759	0.7994952757198	0.957230837266668
Urb1	Urb1	0.0239610474980099	5.57914607620735	0.0644075988773665	0.799660486219397	0.957303053069788
Chchd6	Chchd6	-0.0138982975370967	5.25775046520583	0.0643414775650371	0.799761159949468	0.957303053069788
Alkal2	Alkal2	-0.0413944915997635	0.0977615972125111	0.0643245119446448	0.799787000142313	0.957303053069788
Wnt4	Wnt4	-0.0176230122273858	3.51204835034914	0.0641988417482935	0.799978519111775	0.957303053069788
Chpt1	Chpt1	0.0135377744948977	3.53091739973386	0.0641826381214585	0.800003227662156	0.957303053069788
Stx7	Stx7	-0.0077729695280882	9.08944792894918	0.0641270724435685	0.800087983745397	0.957303053069788
RT1-CE4	RT1-CE4	0.0391363531309935	0.848071511166304	0.0640627925419466	0.800186080671425	0.957303053069788
Nqo2	Nqo2	0.0424774461953655	1.41603435331534	0.0639550121880461	0.800350680797677	0.957303053069788
Smco4	Smco4	0.0286471998056242	1.18044147610431	0.0639062531211974	0.800425040357378	0.957303053069788
Noxo1	Noxo1	0.045020273997365	-0.315056723087948	0.0638118522407041	0.800569540314427	0.957303053069788
Cfap52	Cfap52	0.0277274520530598	3.26422637194741	0.0638101532228887	0.800572128299773	0.957303053069788
Ppp1r35	Ppp1r35	0.0172020514839536	3.38409551489049	0.063775347557528	0.800625388583615	0.957303053069788
Dagla	Dagla	-0.0096556625372107	6.82396845894114	0.0637577866383339	0.800652262454095	0.957303053069788
Adm	Adm	-0.0384250713125986	-0.0851661405284443	0.0637527466764567	0.800659975661399	0.957303053069788
Nr2f6	Nr2f6	-0.0122766049245832	5.62085485178361	0.0637177022334896	0.800713618892148	0.957303053069788
Cd59	Cd59	-0.0123881549555143	6.03597335224432	0.0636654250384039	0.800793665093102	0.957303053069788
Spata13	Spata13	-0.0213743886271499	3.94437299282642	0.0635660745804643	0.800945888205211	0.957303053069788
Stoml2	Stoml2	0.00804667775546775	5.59582082282626	0.0635268534368991	0.801021198828573	0.957303053069788
Pdia3	Pdia3	0.00805153603282144	8.54297985139053	0.0634745712358225	0.801086202117113	0.957303053069788
Hrh2	Hrh2	-0.0291184604519309	1.22440998149045	0.0634638659421665	0.801102624983768	0.957303053069788
Ndufaf8	Ndufaf8	-0.0172682054921128	3.86924309728717	0.0634220254538889	0.801166825271061	0.957303053069788
Plaat1	Plaat1	-0.0478977868026528	-0.155495886611205	0.0632145677319471	0.801485484098684	0.957569938131256
Nrg2	Nrg2	0.0144461576015728	3.50982248255787	0.0631767224392448	0.801543675089639	0.957569938131256

Nek4	Nek4	0.0122659016496095	4.09027162234578	0.0631063559293636	0.801651919983988	0.957570762423249
Pdlim5	Pdlim5	0.0180938264958189	3.92698192615411	0.063064369499898	0.801716538239468	0.957570762423249
Mesd	Mesd	0.0103115431407837	5.8688951176912	0.0630265523254477	0.801774605685674	0.957570762423249
Rtl8a	Rtl8a	0.0102681346712366	5.75057092968459	0.0629724953166146	0.80185801523152	0.95757871898346
Cbx5	Cbx5	0.0110439980499073	5.6982317911171	0.0627814345561291	0.802152581052917	0.95762866827583
Slc30a5	Slc30a5	-0.0080760058645680	5.41581722058776	0.0627533230744319	0.802195961801913	0.95762866827583
Spire1	Spire1	-0.0070039200098625	7.50614767859609	0.0626884891117427	0.802296050908832	0.95762866827583
Gopc	Gopc	-0.0056942102676244	6.77707007667299	0.0626670987612883	0.802329084881592	0.95762866827583
Hpse	Hpse	-0.0247773880625934	1.75702130943102	0.0625860063852102	0.802454373517106	0.95762866827583
Msmo1	Msmo1	0.00857918847162421	7.08738981243459	0.0625850843038833	0.802455798638841	0.95762866827583
Psma5	Psma5	-0.0116150422031607	6.48785670856716	0.0625568483498316	0.802493985490957	0.95762866827583
Srrm1	Srrm1	0.0130284135782935	7.40780919111875	0.0625094844604348	0.802572679871594	0.95762866827583
Gxylt1	Gxylt1	0.017180817680818	5.22402918615048	0.0624090781235411	0.802728029361049	0.95762866827583
Acaa1a	Acaa1a	0.0129719842083021	5.16500788808679	0.0623850819606275	0.802765176088855	0.95762866827583
As3mt	As3mt	-0.0265286146537567	2.24010482768563	0.0623789553934415	0.802774561297586	0.95762866827583
Fbxw5	Fbxw5	-0.0071534424760788	6.15752247868982	0.06231306877158	0.802876700050939	0.95762866827583
Fabp3	Fabp3	-0.0164362191490416	4.78005442841815	0.0622169024836694	0.803025725759241	0.95762866827583
Mfn2	Mfn2	-0.0050208624983144	7.64321477436386	0.0621935159594358	0.803061998003899	0.95762866827583
Spop	Spop	0.00779897352830954	6.76234324068109	0.0620783904644426	0.803240612824224	0.95762866827583
Top2b	Top2b	0.00824143936673238	9.06265265864799	0.0620771025189129	0.80324261203958	0.95762866827583
Cyp4f5	Cyp4f5	0.0329812537694817	1.17174001170393	0.0620093148265113	0.803347866690739	0.95762866827583
Pak1	Pak1	0.00715891809876008	7.73425547416954	0.0619718861689028	0.803406319469102	0.95762866827583
Phlda3	Phlda3	-0.0183185960070212	3.62263228783491	0.0619462435684852	0.803445852969578	0.95762866827583
Arid3a	Arid3a	0.021526723287464	2.79085925687954	0.0619181191447495	0.803489563604066	0.95762866827583
Smim38	Smim38	-0.0503421331446794	-0.367140353283405	0.0618688631129913	0.803566142130396	0.95762866827583
Aurkaip1	Aurkaip1	-0.0121456247988879	5.07204648072459	0.0617695665798976	0.803720617261849	0.95762866827583
Pnlip	Pnlip	-0.0424163388121106	1.12110371937521	0.0617566559839449	0.803725247249843	0.95762866827583
Upf3b	Upf3b	-0.0117173550829497	5.25654770507704	0.0617559069650122	0.80374187819063	0.95762866827583
Eefsec	Eefsec	0.00884719144436748	4.8714231807033	0.0615718232597481	0.804028623142843	0.95785032238749
Gpld1	Gpld1	-0.0287829057792481	3.47489594975818	0.0615379512449863	0.804081453255246	0.95785032238749
Amacr	Amacr	-0.0121991806139451	4.06795457610418	0.0614220684410469	0.804262258878081	0.957918981858566
Rab5c	Rab5c	-0.0084770079216774	7.83859991410305	0.0614026085898871	0.804292638771367	0.957918981858566
Erp29	Erp29	-0.0088174457427603	6.45226499076221	0.0611872150359147	0.804629243884961	0.958129446454172
Tsc22d1	Tsc22d1	-0.0068003731075187	9.48146411286663	0.0611726540988187	0.804652021583157	0.958129446454172
Med27	Med27	0.0129558117073772	4.39671321884062	0.0611421650036306	0.804699725045545	0.958129446454172
Lrrtm3	Lrrtm3	0.00940327919222714	5.38817705864508	0.0610897659014142	0.804781738559597	0.958135663259551
Nr2e1	Nr2e1	-0.0156161616477943	3.21903824231722	0.0609178457387998	0.805051085296358	0.95836488801094
Tmem144	Tmem144	0.0323667318258879	1.54148830010096	0.0608593439100673	0.805142823849017	0.95837070270432
Shank1	Shank1	0.0140454284035504	9.96707135055391	0.0608085754867602	0.805222488268689	0.95837070270432
Vldlr	Vldlr	0.0105945925955832	5.97684115890078	0.060767856317355	0.805286402753233	0.95837070270432
Esrra	Esrra	-0.0131870394770825	4.98790198820356	0.0606921931899908	0.805403656109112	0.958418828543003
Brf2	Brf2	-0.0113295651865653	3.93826354075522	0.0605113283264745	0.805689565669505	0.958511755445102
Pex2	Pex2	0.0115586403808504	3.96321269674597	0.060498008645709	0.805710541999625	0.958511755445102
Sbno2	Sbno2	0.0143143806275221	5.50370513827639	0.0604789237443839	0.805740577079858	0.958511755445102
Arhgef39	Arhgef39	0.0169806094993201	2.94530139816712	0.0603010678595837	0.806020721777698	0.958511755445102
Abcc3	Abcc3	0.0317196913498808	1.15354253485129	0.0602211035531184	0.806146918053819	0.958511755445102
Nedd4	Nedd4	0.00703397071384044	10.1140017891375	0.0601576886990856	0.806246880501984	0.958511755445102
Tceal9	Tceal9	-0.0120940375537481	5.94829452198111	0.0600915794796322	0.806351253940401	0.958511755445102
Hipk4	Hipk4	-0.0195969088528223	2.71293981858641	0.060060250975269	0.806400726734493	0.958511755445102
Fam189b	Fam189b	-0.0109558510583442	6.07200601049917	0.0600319573847559	0.80644543768065	0.958511755445102
Tubb5	Tubb5	0.00936449979815063	12.1967504843877	0.0600313650432973	0.806446373654333	0.958511755445102
Tial1	Tial1	-0.0166571764268886	6.40989729511557	0.0600221669869399	0.806460908118071	0.958511755445102
Bdh1	Bdh1	-0.0082940737247516	6.12166617892569	0.0600012612601404	0.806493947045815	0.958511755445102
Ppp1r11	Ppp1r11	-0.0083842192128850	6.00429947123659	0.0599774899736047	0.806531522051657	0.958511755445102
Scn8a	Scn8a	-0.0303671930122768	5.75288222360526	0.0599320800740202	0.806603322904319	0.958511755445102
RGD1560	RGD15658	-0.0236098898696672	1.51903206090523	0.0599126327600814	0.806634081283447	0.958511755445102
Rmdn1	Rmdn1	-0.0105120495752749	3.79231917327468	0.0596729681959519	0.807011990965928	0.958527229621208
Myrf	Myrf	0.0203163352853801	3.62037932771352	0.0596563932349312	0.807039851396755	0.958527229621208
Rap1a	Rap1a	0.00760283243885815	5.67886508520525	0.0596514303967197	0.807047719445413	0.958527229621208
Ugcg	Ugcg	-0.0058960483241769	7.58328389088504	0.0596494405631915	0.807050874210845	0.958527229621208
Pyroxd1	Pyroxd1	-0.0135535218035828	3.55454621725232	0.0596007425262499	0.807128099505838	0.958527229621208
Tgs1	Tgs1	0.00781491491968897	5.95667490207979	0.059562560641524	0.807188671581681	0.958527229621208
Polr2i	Polr2i	0.0141113202924856	4.55374321442599	0.0595320050667993	0.807237160212291	0.958527229621208
Dlst	Dlst	-0.0050072512439134	7.29987364414064	0.0595040943892617	0.807281558870631	0.958527229621208
Apbb3	Apbb3	-0.0154624009248589	5.8170414928541	0.0594681565439856	0.807338523778094	0.958527229621208
Cbr4	Cbr4	0.022718931018439	2.90250867740846	0.0593878916262582	0.807466032123373	0.958587409296217
Sp100	Sp100	0.0426340931379162	-0.063539479234850	0.0592477688805104	0.807688846760891	0.958734802268621
Mab21l1	Mab21l1	-0.0350909720691544	3.39435184820577	0.0592131944588346	0.807743868033	0.958734802268621
Ccdc153	Ccdc153	-0.0413092147719614	1.97286745843864	0.0591044236195728	0.807917075539538	0.958849172596481
Triap1	Triap1	-0.0101877813551265	4.64781669097903	0.0590021146854323	0.808080147212404	0.958951492939811
Gtf2ird1	Gtf2ird1	-0.0108187482436033	5.7191023822826	0.0588505639887176	0.808321981229394	0.959147252607857
Syne4	Syne4	0.0268342745869625	1.25990809913914	0.0587562236032307	0.808456532974618	0.95921568675583
Ramac	Ramac	-0.0112700244398588	4.79028770256829	0.0586149214886262	0.80869865923818	0.959290496236134
Rcan1	Rcan1	-0.0095437941753667	6.64072687171301	0.0586140587672617	0.808700039781848	0.959290496236134
Mcm5	Mcm5	0.0144796843346305	3.97285667402354	0.0584001005893153	0.809042752581269	0.959290496236134
Tnfrsf10b	Tnfrsf10b	0.0298075470628202	0.949748788808283	0.0583991326770246	0.809044304434994	0.959290496236134
Shisa3	Shisa3	-0.0676720028748205	0.986318027106845	0.058317779137937	0.809174789939765	0.959290496236134
Hectd2	Hectd2	-0.0095546226775475	5.65268908446213	0.0583163228227876	0.809177126830773	0.959290496236134
Sp110	Sp110	-0.0276290501329683	1.57505676220586	0.0583146270644407	0.809180810321661	0.959290496236134
Racgap1	Racgap1	0.0150889775817231	3.98603141570955	0.0583104019334115	0.809186527232636	0.959290496236134
Pcyox1l	Pcyox1l	-0.0079916753254110	5.23377401373713	0.0582122454459508	0.809344202355766	0.959290496236134
Ccnf	Ccnf	-0.0142248542129096	3.85059551744229	0.058189237370712	0.809381158611861	0.959290496236134
Kdsr	Kdsr	-0.0104520568908617	3.88821824704964	0.0581523582031807	0.809440411161456	0.959290496236134
Dad1	Dad1	0.0120197165018382	6.21686082235248	0.0580744231220586	0.809565576254223	0.959290496236134
Pgap6	Pgap6	-0.0156655109579875	3.96596515879447	0.0580737975447789	0.809566698214464	0.959290496236134
Dnai1	Dnai1	0.0342364889918875	0.684417975586768	0.0580108937120206	0.809668203914675	0.959290496236134
Ebf3	Ebf3	-0.0514479603066967	4.21174090118474	0.0579458208880781	0.809772606305099	0.959290496236134
Zmym6	Zmym6	-0.016674578643829	4.77659105801836	0.0579281237671978	0.809801109966329	0.959290496236134
Tubb2b	Tubb2b	-0.0106148864256012	12.5506912861427	0.0579122794378009	0.809826824737934	0.959290496236134
Sh3bgrl	Sh3bgrl	0.00831319073470553	8.26285772970425	0.0577034196388979	0.810163305951227	0.959544652652028
Plpp6	Plpp6	-0.0089587070333376	5.07579632549283	0.0576838831651187	0.810194831613298	0.959544652652028
Unc93b1	Unc93b1	0.0218504899245143	2.55093688745483	0.057454501465148	0.81054923238921	0.959720410690036
Scnm1	Scnm1	0.0126122517581753	3.3758671765371	0.0574503282266647	0.810572153169664	0.959720410690036
Slc38a4	Slc38a4	0.0265234743046798	1.42717245456137	0.0574053126639882	0.810644971597438	0.959720410690036
Elf1	Elf1	0.0192940923190592	2.44782066281898	0.057401543154306	0.810651070630944	0.959720410690036
Tsg101	Tsg101	0.00652072901588885	7.03001010922549	0.057280441389592	0.810847125048327	0.959861440154457
Stx8	Stx8	-0.0107956981302769	4.2547221449294	0.0571353880842196	0.811082243723843	0.959963741479578
Ptger4	Ptger4	-0.0256279626051786	0.658694652530895	0.0571169224919004	0.811112197461739	0.959963741479578
Tmem41b	Tmem41b	0.00714258764510076	5.4495537890206	0.0570102242014351	0.81128536088858	0.959963741479578
Scamp4	Scamp4	-0.0080550160430400	5.26835892389993	0.0569988548949331	0.811304165306898	0.959963741479578
Slc2a13	Slc2a13	0.00868145530702271	5.93851679061135	0.0569361257025562	0.811405745591992	0.959963741479578
Fgf14	Fgf14	0.0148966843820696	3.30565872647311	0.0569221744160089	0.81142841798005	0.959963741479578
Vwa1	Vwa1	0.0124401562069752	5.64790957664491	0.0568705154479687	0.811512394546737	0.959963741479578
Hmgcr	Hmgcr	0.00637487420942263	9.14292311032928	0.0568061111047635	0.811617146649809	0.959963741479578
Cox20	Cox20	0.0155572379365779	5.06779362049153	0.0568006741256681	0.811625992535711	0.959963741479578
Mphosph	Mphosph8	0.00809729332362733	6.03635401278695	0.0567098950518205	0.81177369123233	0.959976345009125
Ostc	Ostc	-0.0106152854494188	5.31999058820257	0.0566999063574897	0.811790527866577	0.959976345009125
Rybp	Rybp	0.0103960053647081	5.07799504370335	0.0564972804422936	0.812120325759085	0.960275322115583
Dync2i1	Dync2i1	-0.0117907846699785	5.09417671122744	0.0563749882163508	0.81232000602327	0.960420486262049
Syt7	Syt7	0.0105783842982174	6.97831273642086	0.0562642714375841	0.812500930817561	0.960543311901716
Tmem158	Tmem158	0.0157779403699743	7.25270780262407	0.0561532242761018	0.812682612043946	0.960585017347581
Spag4	Spag4	-0.0255267567492881	4.04582756009039	0.0561485978119549	0.812690185290804	0.960585017347581
Smc5	Smc5	-0.0121707723068969	6.28741520422418	0.0560184872535388	0.812903306553528	0.96073971263187
Cdt1	Cdt1	0.0154637647388298	3.29158529199193	0.0559747145596319	0.812975064996977	0.96073971263187
Wdr83	Wdr83	-0.0107872177025065	4.34887065807334	0.0559165613772876	0.813070443831353	0.960761428893247
Echdc2	Echdc2	-0.0239481259580589	1.91400360132735	0.0557939881535611	0.813271635213218	0.960850147741488
Lrrc71	Lrrc71	0.0138134599226707	4.48902770847218	0.055714126034382	0.813402856957515	0.960850147741488
Pa2g4	Pa2g4	-0.0068966676301702	7.45947918368391	0.0556762552851211	0.813465123674187	0.960850147741488
Lsm6	Lsm6	-0.0127386778426238	4.37604282685718	0.0556432929311015	0.813519342650251	0.960850147741488
Slc37a4	Slc37a4	-0.0122395464131979	4.88871463157282	0.0556364654146932	0.813530573113816	0.960850147741488
Tollip	Tollip	-0.0051867980958495	7.19258612361082	0.0555742186549821	0.813632995296701	0.96088015923492
Sde2	Sde2	0.00946812853125551	5.50919526249598	0.0554201828976844	0.813886709697981	0.961032667817193
Rab35	Rab35	-0.0066452894698185	7.21588888818297	0.0554023043190455	0.813916181940528	0.961032667817193
Zfp830	Zfp830	-0.0123041536324806	3.73474734942228	0.0553277168019828	0.814039850553303	0.961058084525193
Lurap1l	Lurap1l	-0.0187101953918155	2.15844022560997	0.0552210958403876	0.814215180639881	0.961058084525193
Marchf2	Marchf2	-0.0111962661279323	5.82876569593835	0.0552107227378826	0.814232212189237	0.961058084525193
Hebp2	Hebp2	0.0219450437235828	1.15805579720892	0.0551950235117999	0.814258243252109	0.961058084525193
Zfp36	Zfp36	0.0270570856205649	1.57329209493984	0.0551417065242958	0.81434633818273	0.961058084525193
Atp6v1f	Atp6v1f	-0.0118190489646285	6.54967574008491	0.0551093236291349	0.81439885596817	0.961058084525193
Cry2	Cry2	-0.0073703136023432	6.95251112712105	0.0549854520983217	0.814604774414621	0.961139220478727
Gng2	Gng2	0.00772892633311214	10.6170105873365	0.0549370864841698	0.814684846975561	0.961139220478727
Mcu	Mcu	0.0106237180453267	5.53790956854888	0.0549169892711227	0.814713221459869	0.961139220478727
Smpd3	Smpd3	0.00590649242456421	8.32985322884582	0.0548576217287228	0.814816470481383	0.961139220478727
Col27a1	Col27a1	0.0228094055174656	3.68978572594285	0.0547485022047169	0.81499741674341	0.961139220478727
Dnajc15	Dnajc15	-0.0127075359258059	4.26038718860277	0.0546929968003411	0.815089522520899	0.961139220478727
Lrp3	Lrp3	0.00767826036344514	7.46774190037728	0.0546899153485265	0.81509590217688	0.961139220478727
Rps6ka4	Rps6ka4	-0.0098537514163254	5.15071709672794	0.0546453657657864	0.815168600860816	0.961139220478727
Tbcb	Tbcb	-0.0098562068179079	7.10274160257234	0.0546425675130706	0.815173247726439	0.961139220478727
Cmip	Cmip	0.00808035963585485	8.69514828800092	0.0545891729057431	0.815261940525666	0.961139220478727
Srsf4	Srsf4	0.0058548310841504	6.73339731727062	0.0545551225601884	0.815318524816826	0.961139220478727
Arl4a	Arl4a	0.0095346782849197	4.9308306761945	0.0544651773968354	0.815468083799439	0.961139220478727
Trpv6	Trpv6	0.0144079091290703	4.93156162184911	0.0544639966858256	0.815470047927211	0.961139220478727
Prlhr	Prlhr	-0.0443827614522801	0.0215896350155328	0.054110507579793	0.816059094237508	0.961619247902356
Septin6	Septin6	-0.0074067524674090	6.58749363455884	0.0540979400987992	0.816080073707322	0.961619247902356
Pld1	Pld1	-0.0172147912362209	3.6032603334029	0.0540647411913255	0.816135506452089	0.961619247902356
Myom1	Myom1	0.0355922707826272	0.19302569888251	0.0540347442061773	0.816185608342225	0.961619247902356
RGD1560	RGD156561	-0.0446508190249529	0.361836003536945	0.0539219524042664	0.816374127988839	0.961750542433835
Scn3a	Scn3a	-0.0111053735648599	7.54261025057837	0.0538218903997745	0.816541545137564	0.96180222088042
Npffr1	Npffr1	0.0324331194532251	0.66741679408805	0.0535548026108362	0.816972440915895	0.96180222088042
Dnajc30	Dnajc30	0.0129285690550094	4.26415892328159	0.0535007438126343	0.817079977071765	0.96180222088042
Uhrf1bp1	Uhrf1bp1l	-0.0071768919265628	6.58935663496197	0.0534712563140616	0.817129500831206	0.96180222088042
Plekhh1	Plekhh1	0.0257673523063491	3.62473779406489	0.0534502843588914	0.817147931718021	0.96180222088042
Ptma	Ptma	-0.0093188474086008	10.0324632600291	0.0534363657904677	0.817188117464947	0.96180222088042
Nadk	Nadk	-0.0065232262747206	5.77664693207934	0.0534245729094849	0.817207934250443	0.96180222088042
Kbtbd2	Kbtbd2	0.00800677228091835	5.78195279192831	0.0533884344625903	0.817268675735797	0.96180222088042
Noxa1	Noxa1	0.0485386794212303	-0.519110995767887	0.0533548916434796	0.817325073872302	0.96180222088042
Kit	Kit	0.0112276686415718	6.65442953946603	0.0533438271652784	0.81734368153107	0.96180222088042
Fitm2	Fitm2	0.00886485245343158	4.87334328267259	0.0533098657523954	0.817401158078009	0.96180222088042
Rbm8a	Rbm8a	-0.0117959420561458	5.92274444676498	0.0533074428529408	0.817404885095022	0.96180222088042
Chac1	Chac1	-0.0218612445628818	3.78841644851025	0.0532522207306698	0.817497818571226	0.96180222088042
Thsd7b	Thsd7b	-0.0150934158388898	4.81827407774252	0.0532355196863818	0.817525934803816	0.96180222088042
Jrk	Jrk	-0.0083408098290068	4.494339468564	0.0531124139129494	0.817733327019845	0.96180222088042
Cox19	Cox19	0.00614521405431996	6.15198697435615	0.0530679849528071	0.817808321489761	0.96180222088042
LOC6821	LOC682102	0.0221561585826525	1.65623095375459	0.0530493115823631	0.817839721554207	0.96180222088042
Riok1	Riok1	0.0103073313225776	4.56521129520571	0.0530351540538661	0.81786361344115	0.96180222088042
Azin2	Azin2	-0.0188531974128772	2.22881485262297	0.0529967057157066	0.817928487667518	0.96180222088042
Vpreb3	Vpreb3	-0.0294629871414163	0.367280830071958	0.0529782036978901	0.817959715119814	0.96180222088042
B4galt3	B4galt3	0.00682314148786047	6.17770849322186	0.0528095904207931	0.818244561259327	0.962045016090337
Cnot2	Cnot2	0.00597823742813244	5.9712391885634	0.0527647413415295	0.818320410015769	0.962045016090337
Ppm1h	Ppm1h	-0.0086260257457511	5.71480457303262	0.0527144755035067	0.81840545766129	0.962054352293189
Zdhhc21	Zdhhc21	0.0133377805853678	5.29352842830533	0.0526257022139447	0.81855576258246	0.962140390943582
Fubp3	Fubp3	-0.0135586170737172	5.27646302175575	0.0524365168781458	0.818876524247676	0.962426753416565
Knstrn	Knstrn	0.0195724722634873	2.65947190770184	0.0523248252299248	0.819066165177265	0.96252204864995
Lima1	Lima1	0.0132740666606761	4.88471939232979	0.0522978232420795	0.819111894891814	0.96252204864995
Rab1b	Rab1b	0.00888046247400218	5.65983940925823	0.0519960939330062	0.819625623400885	0.962997748847858
Grb14	Grb14	-0.0085457316713149	5.06715224447181	0.051969430913104	0.819671080510079	0.962997748847858
Zim1	Zim1	-0.0219787752240625	4.01080379056548	0.0517899569116892	0.819976736387747	0.963269488753217
H2az1	H2az1	-0.0105577012152151	7.32949597055798	0.0516569892362782	0.820204571135934	0.963443201069765
Myh11	Myh11	0.027243668515604	2.55404746184835	0.0513573720827765	0.820717956266882	0.963504764221749
Prpf38a	Prpf38a	-0.0082276204635381	5.50153208829995	0.0513364643219845	0.820753828807143	0.963504764221749
Sirt4	Sirt4	0.0156950156708581	2.15800808783794	0.0513280149704087	0.820768223597934	0.963504764221749
Cdip1	Cdip1	-0.0067881007399501	6.68716396527867	0.0512419478806621	0.820916113620984	0.963504764221749
Rnf215	Rnf215	0.00944791805288365	5.42028037634596	0.0512108472867254	0.820969546450797	0.963504764221749
Cln3	Cln3	-0.0135932538760608	3.62624411174908	0.0512006586026104	0.820987054977302	0.963504764221749
Mms19	Mms19	0.00860093864202511	5.84837126521268	0.0511525212559292	0.821069800946744	0.963504764221749
Chmp5	Chmp5	-0.0082578033834808	6.49010158180654	0.0511443036471775	0.821083930039242	0.963504764221749
Ckap4	Ckap4	-0.0074315352620799	6.28109725754059	0.0511423089820013	0.821087358928066	0.963504764221749
Csnk1g3	Csnk1g3	-0.0062204674855705	6.67154378828141	0.0509624919570939	0.821396850252631	0.963504764221749
Arhgap5	Arhgap5	0.0109144487508401	6.00857148852479	0.050804647145398	0.821668996584218	0.963504764221749
Zdhhc7	Zdhhc7	-0.0075538152626334	5.50732522061952	0.0508025007331767	0.821672700365926	0.963504764221749
Adra1a	Adra1a	-0.0346420811754327	1.03070957776431	0.0507540941300938	0.821756354490878	0.963504764221749
Rmnd1	Rmnd1	-0.0140885093554161	3.21235423432284	0.0507342562195715	0.821790330855255	0.963504764221749
Catsperg	Catsperg	0.0325491192139498	4.05358835102683	0.0507294130238307	0.821798866986365	0.963504764221749
Agfg2	Agfg2	0.0117490010868748	4.46548735046592	0.0507111584795226	0.82183039353636	0.963504764221749
Perp	Perp	0.0170503975008128	1.9529633517169	0.0506109735272133	0.822003524419294	0.963504764221749
Patj	Patj	-0.0269282781940711	3.09978338323189	0.0505516495026402	0.82208882802712	0.963504764221749
Gpr137c	Gpr137c	-0.0121038635813765	3.93362609826901	0.050558120764336	0.822094920491146	0.963504764221749
Recql	Recql	0.00851868479614725	4.83611593381576	0.0505092739572817	0.822179458963645	0.963504764221749
Akr1e2	Akr1e2	0.014934947094204	2.64212557321737	0.0504883607922793	0.822215658212225	0.963504764221749
Cdc25b	Cdc25b	-0.0102709782485734	5.2089414869266	0.0504780508837115	0.822233507838531	0.963504764221749
Slc19a2	Slc19a2	0.0138945552298576	4.53651427795131	0.0504778018626447	0.822233713911825	0.963504764221749
Tcf15	Tcf15	0.0316991612088875	0.480930662035579	0.050426446745135	0.822322287828421	0.963504764221749
Sc5d	Sc5d	-0.0078057205122453	6.30739780203161	0.0503188207465968	0.822509427764534	0.963504764221749
Ppp5c	Ppp5c	-0.0073148449130823	7.02948371873158	0.0502627636642572	0.822606574678404	0.963504764221749
Cd109	Cd109	-0.0234447002577064	1.05200547678005	0.050260698732707	0.82261025797542	0.963504764221749
Pdcl3	Pdcl3	-0.0078468792275254	5.77555409383872	0.0502383290907581	0.82264906384455	0.963504764221749
Bclaf1	Bclaf1	0.00709470319864168	7.59681073827637	0.0501648371296438	0.822776519078501	0.963504764221749
Mst1r	Mst1r	-0.0245763710997081	1.57629952777581	0.0501487968093386	0.822804558274716	0.963504764221749
Lrrc40	Lrrc40	0.0115799695882304	5.51120423187064	0.0501220224812187	0.822851081477104	0.963504764221749
Blmh	Blmh	-0.0072928181088553	6.65120127719889	0.0500633878220924	0.822953010944354	0.963504764221749
Creb3	Creb3	-0.0081461468981707	5.74715418257086	0.0500116262782271	0.823043044409112	0.963504764221749
Rims2	Rims2	-0.0106918032562843	6.47029515167581	0.0499737575917294	0.823108943781578	0.963504764221749
Hibadh	Hibadh	0.00882409785476124	4.75208674502943	0.0499440884577922	0.82316059254925	0.963504764221749
Gal3st3	Gal3st3	-0.0067617378480583	6.00796770324529	0.0499352598158112	0.82317596477867	0.963504764221749
Rrh	Rrh	-0.0458238558185146	0.0900747354804368	0.0499153187273293	0.823210690997273	0.963504764221749
Lmnb2	Lmnb2	0.00798817836236863	6.24507037154321	0.0498103604688313	0.823393764070702	0.963504764221749
Atp13a5	Atp13a5	0.0222378261051884	2.62138773312725	0.049782093716388	0.823442881512958	0.963504764221749
Adipor2	Adipor2	-0.0094986779465288	5.13645197855474	0.0497126214731844	0.823564072790893	0.963504764221749
Ddx58	Ddx58	0.0222363648336227	1.67988633630379	0.0496956297677116	0.823593749326262	0.963504764221749
Ruvbl2	Ruvbl2	0.00722948097999404	6.05376625828364	0.0495695664567108	0.823813958702653	0.963504764221749
Mocs1	Mocs1	-0.0141649877146929	2.69562608619589	0.0495197306424089	0.823901095132741	0.963504764221749
Irf5	Irf5	0.0290961881166865	0.678932983573319	0.0495061426905892	0.823924860970322	0.963504764221749
Map7d3	Map7d3	-0.0351260520604084	-0.007052899052403	0.0495011623237929	0.823933572675944	0.963504764221749
Sh3gl1	Sh3gl1	-0.0065556146565337	6.77705314220921	0.0494863374729562	0.823959507170507	0.963504764221749
Trmt10a	Trmt10a	-0.0108880087567694	3.65577399821003	0.0493118692291858	0.824265027573485	0.963504764221749
Rpph1	Rpph1	-0.0952258203670323	0.276655483448934	0.0492658850739159	0.824345471855819	0.963504764221749
Ckap2	Ckap2	0.0156255198038072	3.52551094767717	0.0492624571842128	0.824351658592962	0.963504764221749
Ins5	Ins5	-0.047274521456591	0.751214836446172	0.0492582746702084	0.824358993623063	0.963504764221749
Eif2s3y	Eif2s3y	-0.0955996696280211	5.21549300547342	0.0492387489125008	0.824393240993277	0.963504764221749
Cplx1	Cplx1	-0.0084135091338041	7.43510165025929	0.049222471260947	0.824421796765978	0.963504764221749
Bola3	Bola3	0.0116188115013164	3.93543288241342	0.0490610830425453	0.824705187479101	0.963504764221749
Lactb2	Lactb2	-0.0125188233704608	3.6305789004244	0.0488222713320283	0.82512542908251	0.963504764221749
Thumpd3	Thumpd3	0.00786752319847436	5.40187940728173	0.0488007357388085	0.825163378623896	0.963504764221749
Atg7	Atg7	-0.0100310791702391	4.07208714015377	0.0487385210376686	0.825273061270679	0.963504764221749
Gpr174	Gpr174	0.139167488265256	-1.47127712808049	0.0486770151747038	0.825381566584549	0.963504764221749
Gem	Gem	0.0220614377078302	2.23651472072054	0.0486455246613022	0.825437148319516	0.963504764221749
Sugp2	Sugp2	-0.0050732344157560	7.53607212578152	0.0485969190002677	0.825522975649883	0.963504764221749
Carhsp1	Carhsp1	0.00885120291919525	6.7495080804116	0.0485505253069016	0.825604921514197	0.963504764221749
Rtn2	Rtn2	-0.0068617236600551	7.72806375326159	0.0484236204252895	0.825829051493746	0.963504764221749
Hmgb1	Hmgb1	0.0064148925322839	8.50360149003937	0.0484186636814865	0.825838123042044	0.963504764221749
Crtc2	Crtc2	0.0202458568886117	5.01743355982774	0.0483954547915459	0.82587919999822	0.963504764221749
Tacstd2	Tacstd2	0.0329250433289012	0.896217186271275	0.0483888930611476	0.82589074454852	0.963504764221749
Tnni3	Tnni3	0.0382470950884783	0.438764245905747	0.048192139574823	0.826239486221933	0.963504764221749
Megf8	Megf8	0.0159686107403989	8.02551651844633	0.0480729686224978	0.826451212015884	0.963504764221749
Mrps18b	Mrps18b	-0.0099780529997606	4.85470384807294	0.0480540626451997	0.826484620870395	0.963504764221749
Gpsm2	Gpsm2	0.0124939617758389	3.34811451161105	0.048044142120105	0.826502247462593	0.963504764221749
Ahsa1	Ahsa1	-0.0077909749415485	7.06016239019279	0.0480439941085251	0.826502510461072	0.963504764221749
Atg101	Atg101	-0.0093369381700662	5.34845445359051	0.0480408887042145	0.826508029482711	0.963504764221749
Mdh1b	Mdh1b	0.0291583834315658	0.578913703876197	0.0480187699884356	0.826547336823609	0.963504764221749
Abcf1	Abcf1	-0.0048309136799329	7.42274321851131	0.0480125329369514	0.826558422755658	0.963504764221749
Nrf1	Nrf1	0.00912625748043148	4.35005600552209	0.0479223894126335	0.826718720786955	0.963504764221749
Gpc5	Gpc5	0.0148637055200013	4.87320442599888	0.047782797521772	0.82700114890029	0.963504764221749
Map3k7cl	Map3k7cl	-0.0243144397173239	0.789890944666275	0.0477357334052613	0.827051176221539	0.963504764221749
Actr2	Actr2	0.00520774921142824	8.08907870588925	0.0477212570882317	0.827076987818216	0.963504764221749
Mir3593	Mir3593	0.0248271933437993	0.905448885306112	0.047686716753617	0.827138590640809	0.963504764221749
Mtmr10	Mtmr10	-0.0105209682687668	3.85590218279178	0.0476773174056362	0.827155258455537	0.963504764221749
Hic2	Hic2	0.0143021183571208	3.91517853315725	0.0475420861137508	0.827396794067866	0.963504764221749
Rgs7bp	Rgs7bp	-0.0123028028899106	4.4400012068287	0.0474709954285117	0.827523860307967	0.963504764221749
Trdmt1	Trdmt1	-0.0238272879431879	3.57439899956416	0.0473776591258286	0.827690828087231	0.963504764221749
Setd3	Setd3	0.00421271841663443	7.16854780615233	0.0473703719534484	0.827703883419764	0.963504764221749
Slc9a3r1	Slc9a3r1	0.0109969432610745	5.16455986555633	0.0472821920178603	0.827861808798329	0.963504764221749
Gja4	Gja4	0.0165733771129543	2.90783912987678	0.0472489980071062	0.827921297269501	0.963504764221749
Cdc42ep	Cdc42ep6	0.0369151839585426	2.08007368163688	0.0471681363673895	0.828066305073001	0.963504764221749
Mir149	Mir149	0.0473432844186147	0.847309434790421	0.0471446188307709	0.82810385030667	0.963504764221749
Tacr2	Tacr2	0.026590613999847	0.996145391405857	0.0470375503877278	0.828300757585859	0.963504764221749
Ddx19b	Ddx19b	-0.0103010624687438	4.95226175977087	0.0470115685718824	0.828347445668839	0.963504764221749
Cdk11b	Cdk11b	0.00558037621044134	6.85373636286539	0.0469403732504077	0.828475449500032	0.963504764221749
Lnc-hc	Lnc-hc	0.0180619440199657	2.19325259041922	0.046969118818492	0.82860366140398	0.963504764221749
Dync1i2	Dync1i2	0.00517112069657536	8.57731248822571	0.0468556571169003	0.828627895218544	0.963504764221749
Ttc8	Ttc8	0.00996402194284612	4.25896566295932	0.0468414501232033	0.828653474664122	0.963504764221749
RGD1561	RGD156151	-0.0485190724535989	-0.555883091159613	0.0468314349289898	0.828671509287918	0.963504764221749
Actn1	Actn1	0.0109671539788515	5.80322651306061	0.0467884383231336	0.828748867353752	0.963504764221749
Scap	Scap	0.00473241803422245	7.43185163168861	0.0467493201038565	0.828819451805636	0.963504764221749
Zfp707	Zfp707	0.0135134949904269	2.91447538028808	0.0466549825386516	0.828989581645575	0.963504764221749
Ranbp10	Ranbp10	0.0181906760240641	5.87223718390468	0.0466523053072478	0.828994414288046	0.963504764221749
Acap1	Acap1	-0.0372842208516131	1.31927730285895	0.0466332596617889	0.829028784735569	0.963504764221749
Plekhn1	Plekhn1	0.0278492922802836	4.27793956297174	0.0465848984451016	0.829116092114902	0.963504764221749
Sema5b	Sema5b	-0.0164807064307789	4.8695172170582	0.0465724763704287	0.829138525579607	0.963504764221749
Ube2z	Ube2z	-0.0063473740631888	7.62905433506446	0.0465440166837041	0.829189933718749	0.963504764221749
Glra1	Glra1	-0.0620262413617656	-0.387255986141997	0.0465035857382109	0.82925870782999	0.963504764221749
Pdzk1	Pdzk1	0.0115497343324856	3.66962806502069	0.0464507461380919	0.829358528200647	0.963504764221749
Cttn	Cttn	-0.0052103880562250	7.49808457190423	0.0463840946250747	0.829479123316601	0.963504764221749
LOC1001	LOC100174	0.0147243039724015	3.25318222422265	0.0463765035462453	0.829493705586101	0.963504764221749
Zhx3	Zhx3	-0.0096073957027140	6.0183619600885	0.046374147539737	0.829497119406308	0.963504764221749
Arpp19	Arpp19	0.0101679403435937	5.12853516637594	0.0463710969825115	0.829502641308728	0.963504764221749
Med17	Med17	-0.0089640546145245	5.32167686346338	0.0463592929463353	0.829525820521521	0.963504764221749
Cox16	Cox16	-0.0110889541494874	3.44717396108315	0.0463255816296311	0.829585052558669	0.963504764221749
Tmem181	Tmem181	0.00591360222683614	5.95522599932479	0.0462984577286392	0.829634183922015	0.963504764221749
T2	T2	-0.0456261682160326	-0.303557727626652	0.0462710572177025	0.8296838134993	0.963504764221749
Lsg1	Lsg1	-0.0081092193180148	4.96723837228905	0.0462266458742491	0.829764334368853	0.963504764221749
LOC1003	LOC100369	0.0102889660443148	3.60902940649088	0.0462230045103032	0.82977093672479	0.963504764221749
Dclk1	Dclk1	0.00828222061404058	10.1630128095394	0.0462073564136176	0.829799312240279	0.963504764221749
Ism1	Ism1	-0.0277059216728507	0.805911192254415	0.0461862939898765	0.829837513777237	0.963504764221749
Atox1	Atox1	-0.0120396127973042	5.18505094164227	0.0461725770906156	0.829862397420643	0.963504764221749
Ubl7	Ubl7	0.00879902471898768	6.32996498014172	0.0460458018785571	0.830092382210389	0.963504764221749
Dhrs4	Dhrs4	0.0182935841323122	2.89902728205458	0.0460106829647771	0.830156380020127	0.963504764221749
Plk1	Plk1	0.0130047879475479	3.53152575862544	0.0459881548891516	0.830197321475883	0.963504764221749
Ak6	Ak6	-0.0117855463917636	3.80376699486978	0.0458984148927186	0.830360564748534	0.963504764221749
Lmod1	Lmod1	-0.047985903193833	-0.094629869021114	0.0457822099380252	0.830572184850683	0.963504764221749
Wnt7b	Wnt7b	0.0125529382505879	4.86935812789235	0.0457648677371741	0.83060415523085	0.963504764221749
Fbl	Fbl	0.00924700927068715	5.61121230626849	0.0456763221078171	0.830765242960247	0.963504764221749
S1pr5	S1pr5	0.0387142141964514	-0.331089725642322	0.0456625385351401	0.830790411442206	0.963504764221749
Wdr7	Wdr7	0.00699980618482696	7.12475219888035	0.0456015188647854	0.8309017988739	0.963504764221749
Tlcd4	Tlcd4	-0.0134171559159865	3.24044113018258	0.0455761195705833	0.830948186597473	0.963504764221749
L1cam	L1cam	0.0110312583608932	9.5139070928874	0.0455666017563111	0.830965463226387	0.963504764221749
Gphn	Gphn	-0.0062582588580819	6.74552854945709	0.0455628374613465	0.830970440055163	0.963504764221749
Pde9a	Pde9a	-0.0128177898875156	5.24145521626393	0.0455048034042397	0.831078872013738	0.963504764221749
Npas1	Npas1	0.0111908113211627	5.51425865803986	0.0454954688092321	0.831095572258904	0.963504764221749
Vps4a	Vps4a	-0.0056782025218070	7.31989734478576	0.0454778007307439	0.831127877667935	0.963504764221749
Trim3	Trim3	-0.0051567702824247	7.36170835312958	0.0454520202092382	0.831175028528137	0.963504764221749
Rbmxl1	Rbmxl1	0.00659730755839126	6.90685516330318	0.0454423633320929	0.831192877084097	0.963504764221749
LOC4994	LOC499407	0.0251050200742854	0.712774098541035	0.0454211429564566	0.831231519707226	0.963504764221749
Mis18bp1	Mis18bp1	0.0204231686107239	2.16725642808726	0.0453811238518824	0.831304765952262	0.963504764221749
Stxbp4	Stxbp4	0.0189616042567862	2.11805399058302	0.0453260179177561	0.831405580623341	0.963504764221749
Ranbp6	Ranbp6	0.00663265003172885	6.10700874946469	0.0452965437727428	0.831459682505744	0.963504764221749
Gng13	Gng13	-0.0322312540598215	0.0681579760979866	0.0452140679571045	0.831610890556232	0.963504764221749
Slc2a1	Slc2a1	0.00971549099099161	5.71804740980096	0.0451645265764364	0.83170176888862	0.963504764221749
Arhgap4	Arhgap4	-0.0264278304086195	4.01887520597914	0.0451126116334137	0.831797094612906	0.963504764221749
Rspo2	Rspo2	0.013297114067726	3.23727654622669	0.0450977383725828	0.831824410078729	0.963504764221749
Jagn1	Jagn1	0.00929296791066817	5.02326424388277	0.0450758442053214	0.831864628246748	0.963504764221749
Snrpg	Snrpg	0.0191964139242497	4.16102719331483	0.0450502378849125	0.831911678550982	0.963504764221749
Slitrk3	Slitrk3	-0.0112737531087449	4.97459852351014	0.0450395264449384	0.831931364403673	0.963504764221749
B3galt6	B3galt6	0.00737242640496352	5.56481326754145	0.045019359041774	0.831968825259049	0.963504764221749
Scamp3	Scamp3	0.00542367202933424	6.40393540952124	0.0449661004405613	0.832066379202978	0.963504764221749
Nsmce1	Nsmce1	0.0119346294496031	4.46491061053403	0.0449518806212552	0.832092534745431	0.963504764221749
Nabp1	Nabp1	-0.0163842358861194	2.48802429603099	0.0449017416998047	0.83218480815562	0.963504764221749
Tmem115	Tmem115	0.00784395446857105	5.28454832088145	0.0448875974745064	0.832210848278571	0.963504764221749
Alkbh6	Alkbh6	0.0106511687373908	4.51192379790114	0.0448816525340376	0.832221794446833	0.963504764221749
Cd81	Cd81	-0.0080365147402699	7.94959830919522	0.0448421450309906	0.832294557249635	0.963504764221749
Zfp451	Zfp451	0.010784198182067	4.93941229461105	0.0448237945359754	0.832328406985703	0.963504764221749
Msra	Msra	-0.0143876668185988	5.59079170439413	0.0448092166142207	0.832320217795401	0.963504764221749
Fam126b	Fam126b	0.0124698404975678	3.99303247206357	0.0448180659470268	0.83233892118866	0.963504764221749
Tmbim6	Tmbim6	0.00810032682651148	7.27812756232841	0.0447489069833758	0.832456964111889	0.963504764221749
Slfnl1	Slfnl1	-0.0201158636922479	2.33439038312366	0.0446948566651994	0.83258457494014	0.963504764221749
Dtwd1	Dtwd1	0.0134382691337745	2.64644671756413	0.0446726438534384	0.832607115949246	0.963504764221749
Fermt3	Fermt3	0.0310105260406067	0.791960092105732	0.0446293221216365	0.832687100210593	0.963504764221749
Adgrl1	Adgrl1	0.00763174402006716	9.73657424807477	0.0446107348966223	0.832721428956337	0.963504764221749

Strbp	Strbp	-0.0062662087867580	7.75129710898989	0.0446079087832558	0.832726650311409	0.963504764221749
Trpc1	Trpc1	0.0079937883555758	5.24083390349991	0.0445990378572105	0.832743037677819	0.963504764221749
Yipf3	Yipf3	0.0057601404189025	5.90885384259031	0.0444891228844639	0.832946226299711	0.963504764221749
Rab11fip	Rab11fip4	0.0078788821209949	7.17719569365917	0.0444645218359341	0.832991739648856	0.963504764221749
Crip3	Crip3	0.0382044297188837	-0.260126197798121	0.0444329017247043	0.833050257983378	0.963504764221749
St6galnac	St6galnac3	-0.0106495385604067	4.46204973106375	0.0443950133985354	0.833120405253387	0.963504764221749
Stard3	Stard3	-0.0064279228988712	5.74279374116396	0.0442941352855328	0.833307326114528	0.963504764221749
P4ha2	P4ha2	0.0176381425416292	3.3906159676313	0.04429403227671	0.833307517096903	0.963504764221749
C1galt1	C1galt1	-0.0108475111947 97	3.10928352863604	0.0442558675111258	0.833378292027639	0.963504764221749
Il17rb	Il17rb	0.0188331772893413	1.70701981576219	0.0442373085494694	0.83341272034716	0.963504764221749
Pparcc1b	Ppargc1b	0.0236355298983539	1.92286870549347	0.0442180823569913	0.833448394385689	0.963504764221749
H1f4	H1f4	0.0790579592798161	-0.580808712076562	0.0442106841104533	0.833462123927654	0.963504764221749
Pnkd	Pnkd	0.0066960860306471 6	6.45948393349761	0.0440414942407301	0.833776431648881	0.963595948326592
Popdc2	Popdc2	0.0182352562868805	1.87098456358954	0.0440243717036068	0.833808275658372	0.963595948326592
Slc25a44	Slc25a44	-0.0057712510621710	6.0546295036131	0.0440137585939518	0.833828015004703	0.963595948326592
Fgfr4	Fgfr4	0.0275867641918754	0.634264141409873	0.0440019842069717	0.83384992104506	0.963595948326592
Ell3	Ell3	-0.0198916074023644	1.77220871367917	0.043957017223808	0.833933602372533	0.963603404037979
Spata22	Spata22	-0.0294403625760943	0.560580774603734	0.0438870862446379	0.83406382923496	0.963623373764994
Traf6	Traf6	0.0147909189095796	3.15959380920469	0.043839091819855	0.834153268249143	0.963623373764994
Hid1	Hid1	-0.0059476290163838	7.04413127880033	0.0437967151920304	0.834232280737424	0.963623373764994
Mfsd1	Mfsd1	-0.0076198012309858	5.2759879069127	0.0437798142782313	0.834263804125332	0.963623373764994
Mrps9	Mrps9	0.0079846091795513 5	4.93015201707416	0.0437405602992769	0.834337044704916	0.963623373764994
Nlgn2	Nlgn2	0.0061157685016573 6	8.84392449465783	0.0436695024046614	0.834469712738886	0.963675801174065
Clcn2	Clcn2	-0.0141815512204547	6.32700208363244	0.0435395182308298	0.834712690408379	0.963675801174065
Zfand4	Zfand4	-0.0135972503340014	2.35702732867864	0.0435232792970908	0.834743072211369	0.963675801174065
Dusp16	Dusp16	0.0155084534336735	2.36745028851118	0.043513620485534	0.83476114591429	0.963675801174065
Sptbn4	Sptbn4	-0.0124355920087799	7.49344616862085	0.0434441437856492	0.834891213410098	0.963675801174065
Npepps	Npepps	0.0071511956548767 6	8.20409040868525	0.0434293223155038	0.834918974761752	0.963675801174065
Uba1	Uba1	0.0055996205213132	9.52010616975881	0.0434271247439781	0.834923091343403	0.963675801174065
Pmvk	Pmvk	0.0096988714021458 2	5.59272235111663	0.0433303487500041	0.835104484021445	0.963796008429893
Ubac1	Ubac1	0.0074000288345272 3	6.32287200525621	0.0431889241187733	0.835369944655168	0.963957430908619
Scand1	Scand1	-0.0146555103225198 2	6.32734217832574	0.0430954375852934	0.835545672237734	0.963957430908619
Kcnab3	Kcnab3	0.0177959442983268	2.17931304146567	0.0430921498801435	0.835551859925184	0.963957430908619
Ins2	Ins2	0.0065598023775072 82	6.70252625002417	0.0430913255346263	0.835553387459355	0.963957430908619
Knop1	Knop1	0.0099066970615735 8	3.83940938429845	0.0429890354985218	0.835745919403912	0.964090406472135
Trmt112	Trmt112	-0.0123422272072541	5.10634049820409	0.0429445401870192	0.83601827174521	0.964277261425324
Tomm40	Tomm40	-0.0084794962471471	6.29890623415237	0.0427522509719722	0.836192474005272	0.964277261425324
Eci2	Eci2	0.0091092150224402 2	4.82813436337832	0.0427506277741614	0.836195539656794	0.964277261425324
Lmo4	Lmo4	-0.0094512020232939	8.37435191405632	0.042716857044334	0.836259145550831	0.964277261425324
Abhd16a	Abhd16a	0.0080958049870737	5.61700207393567	0.0426983422173208	0.836294321221722	0.964277261425324
Foxj3	Foxj3	0.0055656558003173 7	7.236902254439	0.042578441175948	0.836521085120243	0.964347001821672
Zswim3	Zswim3	-0.0114075600606289	3.82003344710313	0.0425738023638331	0.836529864998858	0.964347001821672
Fbxl20	Fbxl20	0.011694344345353	3.5279931454707	0.0424891897564308	0.836690098850883	0.964347001821672
Leo1	Leo1	-0.0059307752936797	5.94158962119057	0.042445068701582	0.83677371841417	0.964347001821672
Mitd1	Mitd1	0.0153699621444818	2.72867225575881	0.0424188933670541	0.836823727387603	0.964347001821672
Slc12a3	Slc12a3	-0.0363186194354443	-0.128075718 97662	0.0423492578483007	0.836955458202647	0.964347001821672
Eln	Eln	0.0178177658944796	6.55120535564716	0.0423243578779591	0.837002725037841	0.964347001821672
Hspb6	Hspb6	-0.0130018884890454	3.07345096418565	0.042314128015314	0.837022128278264	0.964347001821672
Eif1ad	Eif1ad	-0.0078145007389540	4.80656545445935	0.0422713648252824	0.837103368258377	0.964347001821672
Palmd	Palmd	0.0103235093473908	4.5169399995737	0.0422585556667983	0.837127705115855	0.964347001821672
Eef1a1	Eef1a1	-0.0107422365762585	12.1486044812232	0.0421098677702219	0.837410486426352	0.964438276299828
Taf1d	Taf1d	-0.0221385508259664	4.34413559817467	0.0420320332943049	0.837558722999315	0.964438276299828
Gkap1	Gkap1	0.0078680469201514 4	5.14324942499866	0.0420300204151 6081	0.837562208340009	0.964438276299828
Spata25	Spata25	-0.0174355116459633	1.49396243195282	0.0420257365323735	0.837570721499144	0.964438276299828
Mtfmt	Mtfmt	-0.0102486645154453	3.6673198889049	0.0420138232037317	0.837593424947386	0.964438276299828
Rad21	Rad21	0.0057238650645509 8	7.86619160223514	0.0415631253981567	0.838454785463121	0.965255108041573
Dcaf8	Dcaf8	0.0073250305549988 7	6.55471986082598	0.0415532540430673	0.838473522518841	0.965255108041573
Cnot8	Cnot8	0.0064007080547981 3	6.23610126117899	0.0414491964743817	0.838673316449252	0.965255108041573
Anapc5	Anapc5	-0.0064732998052701 6	8.90888998565684	0.0414247388915214	0.838720266027758	0.965255108041573
Ntrk1	Ntrk1	-0.0756664531279434	0.625256834470229	0.0413478040516679	0.838869048858094	0.965255108041573
Nav3	Nav3	0.0087995830874516	7.44475765827499	0.0413255219400526	0.838910874342518	0.965255108041573
Fam214a	Fam214a	-0.0175584118721944	5.47304125825564	0.0413057161865709	0.838948952220096	0.965255108041573
Plp1	Plp1	-0.0222253061681621	4.63672051708977	0.0411118013737711	0.839322655118675	0.965255108041573
Prkce	Prkce	0.0070852887461724 5	7.73247451366314	0.0410667030127811	0.839409222560479	0.965255108041573
Psmb4	Psmb4	-0.0103806204887468	7.6330789896253	0.0410428594421006	0.83945521480032	0.965255108041573
Lingo3	Lingo3	0.0136086656705867	4.46215614525886	0.0410161509535953	0.83950674974321	0.965255108041573
Cul3	Cul3	0.0052113723141587 6	7.56414815120113	0.0410116799162168	0.839515378469484	0.965255108041573
Scarb1	Scarb1	0.0066568765753161 6	6.02394913775104	0.0409691425864454	0.839597496421618	0.965255108041573
Gpr150	Gpr150	0.0167380807762667	1.98343272381561	0.0409517336002736	0.839631117145155	0.965255108041573
Rpl17	Rpl17	0.01469457269128	8.17887926100519	0.0409289861108181	0.83967561534025	0.965255108041573
Commd9	Commd9	0.0073791835496884	5.19642216456554	0.04091716440049	0.839697900176031	0.965255108041573
Wdr25	Wdr25	-0.0133544730058555	2.26178860171905	0.0408803815237988	0.839768999186331	0.965255108041573
Gnb1	Gnb1	-0.0059875819478590 2	10.154922251995 7	0.0406545822890223	0.840206134927273	0.965255108041573
Ina	Ina	-0.0064874905202307	9.19083918260011	0.0405557983918072	0.840397775837051	0.965255108041573
Cenpa	Cenpa	0.0179415474573811	2.22885334964891	0.0405371998795232	0.840433884136303	0.965255108041573
Arf1	Arf1	-0.0069029138465103	8.23458869377229	0.0405117147836336	0.840483376473743	0.965255108041573
Mrps16	Mrps16	-0.0108584735716836	4.44232417221826	0.0405063708970346	0.840493756414669	0.965255108041573
Dscam	Dscam	0.0075568310666112 6	6.71816532897177	0.0404954761452814	0.840514820537762	0.965255108041573
Trpv3	Trpv3	0.0373623021444 08	-0.024363764644109	0.0404447561080019	0.840613488199113	0.965255108041573
Adarb2	Adarb2	0.0130080405014989	4.41859778864692	0.0404420311305529	0.84061878565089	0.965255108041573
Rgs2	Rgs2	-0.0167772271505816	4.99577952465474	0.0403885591926785	0.840722710889438	0.965255108041573
Hopx	Hopx	-0.0188301538190123	2.90054787978547	0.0402921247089125	0.840910495598625	0.965255108041573
Pmpcb	Pmpcb	0.0073081530858580 3	6.10980200793008	0.0402782815851737	0.840937462035659	0.965255108041573
Lmbrd2	Lmbrd2	0.0087162144517863 3	5.74193670567864	0.0401870888305638	0.841115226335107	0.965255108041573
Chmp3	Chmp3	0.0067766079465861 5	5.14535287148033	0.0401837416953299	0.841121754979437	0.965255108041573
Eif4a1	Eif4a1	0.0077722103745896 8	9.72464774812411	0.0401627036344578	0.841162796545671	0.965255108041573
Mdga2	Mdga2	0.0100006194285582	4.99412949828206	0.0401475307678538	0.841192403087686	0.965255108041573
Bad	Bad	-0.0094938211694457	5.79199071379169	0.0401429880706701	0.84120126830298	0.965255108041573
Mutyh	Mutyh	0.0233427185518549	4.45227662559996	0.0400635708494406	0.841356337860235	0.965255108041573
Dazap1	Dazap1	-0.0060336281899501	6.88088923542362	0.0400162603392218	0.841448792047061	0.965255108041573
Exosc4	Exosc4	-0.0140227451033 88	4.1211912873418	0.04000458893428	0.841471608244594	0.965255108041573
Ghr	Ghr	-0.0130345158330575	3.12176449186995	0.039937744235786	0.841603353725314	0.965255108041573
Dbnl	Dbnl	0.0054444504542761 6	6.94237162862437	0.0399263011616569	0.841624747174679	0.965255108041573
Fam168b	Fam168b	0.0055861715945803 3	9.11156142144828	0.0399054619017889	0.841665527002676	0.965255108041573
Tspy26	Tspy26	0.0079429216875074 4	5.2195816232225	0.0399014552680512	0.841673380676294	0.965255108041573
Sf3b6	Sf3b6	-0.0121862347560585	4.65541157000724	0.0398531628153194	0.841767953679403	0.965255108041573
Fbxl8	Fbxl8	0.0410185769459827	-0.649549060986264	0.039851528665051	0.841771133282905	0.965255108041573
Naaladl1	Naaladl1	-0.0127984391375689	2.92205103302278	0.0398190814176047	0.841834732484206	0.965255108041573
Pts	Pts	0.0122908070445857	3.09005158180301	0.0397969899087167	0.841878014572429	0.965255108041573
Hnrnph1	Hnrnph1	0.0078042724702624 5	9.09337172104909	0.0397885902283974	0.841894501854548	0.965255108041573
Wwp2	Wwp2	0.0062870056316121 6	5.12238828199615	0.0397792097263324	0.841912894470755	0.965255108041573
Inka2	Inka2	-0.0133084590162952	4.79454816061572	0.0397659613420132	0.841938874794938	0.965255108041573
Polr2g	Polr2g	0.0092410792254072 5	5.08321219076446	0.0397205990345029	0.842027865208371	0.965268843754179
Plbd2	Plbd2	0.0084445520151554 2	5.83922294497225	0.0396195117552622	0.842226365439816	0.965407290913 42
Inpp5b	Inpp5b	-0.0115228123472128	5.89958987821475	0.0394577503875837	0.842544558951723	0.965683303057748
Chek2	Chek2	-0.0198289403221390 1	1.40074262676296	0.0394178565120503	0.842623528112391	0.965685107032085
Ccdc34	Ccdc34	0.0080944950826770 3	4.53633744926491	0.039371813614693	0.842713874042367	0.965699945483708
Sertm2	Sertm2	0.0423960602728141	1.17256420064168	0.0393331717984 9362	0.842792938242111	0.965701854205787
RGD1560	RGD156050	0.016382003086872	1.65838979205872	0.039208742655596	0.843035693726517	0.965891308597406
Ankrd10	Ankrd10	0.0126525598885633	6.60801345569704	0.0389352669555478	0.843576961618119	0.966119508577917
Inpp5f	Inpp5f	0.0073227921038726 8	6.59328824296399	0.0389081716272415	0.84363069655439	0.966119508577917
Mboat4	Mboat4	0.0284256732804577	-0.115776114292803	0.038874498989415	0.843697502580972	0.966119508577917
Cntnap1	Cntnap1	0.0220751973695821	2.29928275809022	0.0388556165405731	0.843734978240703	0.966119508577917
Golph3	Golph3	0.0060887195114030 1	6.10464830143531	0.0388193792224979	0.843808910214424	0.966119508577917
Cnksr3	Cnksr3	-0.0159233077042741	2.95111862490252	0.0388166748800529	0.843812294943207	0.966119508577917
Wee1	Wee1	-0.0094985488542316	3.94924196254485	0.0388007221820601	0.843843978852668	0.966119508577917
Mif	Mif	-0.0097426092914179	7.67710834948887	0.038795514696119	0.843854324315311	0.966119508577917
Dnp3	Dnp3	-0.0124094252215791	5.16612419404611	0.0385591901241611	0.844324534908819	0.966569155166267
Usp3	Usp3	0.0077912382495974 5	4.99009098996035	0.0384888651516474	0.844464767867292	0.966594032078486
Eif3a	Eif3a	0.0069076567095280 3	8.37101417841237	0.0384454148010391	0.844551445235406	0.966594032078486
Cfap206	Cfap206	-0.0463846867343485	0.441492047644335	0.038431772838 3934	0.844578675781 01	0.966594032078486
Rasef	Rasef	-0.0329137032614533	-0.292743174572305	0.0383003250190299	0.844841314647444	0.966717793316177
Zyx	Zyx	-0.0071857955120535	5.74818135785975	0.0382594299109537	0.844922720205819	0.966717793316177
Egln3	Egln3	0.0138816918068619	4.57496923745102	0.0382316600515082	0.844978696536468	0.966717793316177
Btf3	Btf3	-0.0076370211700017	7.91537241789362	0.0381637905779099	0.845114612763129	0.966717793316177
Syt6	Syt6	-0.0144161607264627	4.27721061090551	0.0380964267949808	0.845249640413226	0.966717793316177
Pim2	Pim2	0.0089390813845809 2	4.63488189115646	0.0380738078358807	0.845295006877613	0.966717793316177
Trpm4	Trpm4	0.0123358512614907	5.4923652835193	0.0380700459429738	0.84530255340197	0.966717793316177
Med4	Med4	-0.0073901756569547	4.88029015206584	0.0380688017772406	0.845306654250179	0.966717793316177
Cideb	Cideb	-0.0343298825870884	-0.381182390765993	0.0378918918674742	0.845660382498046	0.966897093737366
Slc9a3r2	Slc9a3r2	-0.0103647955705260	5.25025858726864	0.0378810362841193	0.845682214534856	0.966897093737366
Atf5	Atf5	0.0084324131765075 7	4.52709659810525	0.0378742227092127	0.845695918207813	0.966897093737366
Nek2	Nek2	-0.016061129486219	2.74537268671845	0.0378240084678509	0.845796858500646	0.966924010556401
Fam83d	Fam83d	-0.0167146815454527	2.07467649823838	0.0377471588545077	0.845951727484745	0.967012341074576
Alg1	Alg1	-0.0105218988733442	3.8056427678541	0.0375915035854248	0.846265706247037	0.967282632543448
Srpk2	Srpk2	-0.0045671704474971	7.58392644464705	0.0375342110074826	0.846381443311647	0.967326304686232
Med16	Med16	-0.0057685036828391	7.09417246774698	0.0373530999653866	0.846747910773071	0.967635017149149
Grpel2	Grpel2	-0.0055388848542813	5.19592652995 34	0.0372899954003358	0.846875815557365	0.967635017149149
Pc	Pc	-0.0071928468359613	5.77198129025 11	0.0372528021570417	0.846951254032852	0.967635017149149
Slc15a5	Slc15a5	0.0361080658636213	-0.474352217369923	0.0372374791752037	0.846982344759043	0.967635017149149
Sgta	Sgta	0.0056322739242015 5	7.37112664373969	0.0371341657736366	0.847192143723129	0.967635017149149
LOC6898	LOC689986	0.0083614637542824 3	5.7504086277419	0.0370334970516524	0.847396863487406	0.967635017149149
Nifk	Nifk	-0.0074782979073410	5.21254567441788	0.0369727306922556	0.84752057754781	0.967635017149149
Ntmt1	Ntmt1	0.0119886890488746	4.90740931437589	0.0369673204895315	0.847531578904472	0.967635017149149
LOC1003	LOC100362	0.0348052975976139	-0.43693254466048	0.0369219436312136	0.847624055682057	0.967635017149149
Runx2	Runx2	-0.0311607502613645	0.259038138812497	0.0369214574526627	0.847625046622124	0.967635017149149
Pcgf1	Pcgf1	-0.0118542663870671	3.10783086679161	0.0369118806496516	0.847644567665234	0.967635017149149
Orc2	Orc2	0.0117271861798505	4.58834293336875	0.0368889667638257	0.847691285242809	0.967635017149149
Mark4	Mark4	-0.0049898867717544	7.60109923215051	0.0368753646188272	0.847719432768674	0.967635017149149
Snrf7	Srsf7	0.0090923293495321 1	6.91823456811714	0.036866401648723	0.847737306440438	0.967635017149149
Aars2	Aars2	-0.0071482958209312	4.53987216089842	0.0368214249365906	0.84782907932053	0.967651246129002
Ltа4h	Lta4h	0.0074220847098628 6	4.89620837864463	0.0367391921625568	0.847997021834398	0.967754398740308
Napepld	Napepld	0.0186120267221655	0.970676234181926	0.0366250154169165	0.848230526372565	0.967879022511442
Ube2t	Ube2t	0.0210492903037631	0.910710537068441	0.0365894436320104	0.848303351817474	0.967879022511442
Cdkn2b	Cdkn2b	0.0322200865971836	-0.131688222029829	0.0365720656037638	0.848338942869891	0.967879022511442
Polh	Polh	0.0104436997025018	3.10564418378588	0.0364764853669755	0.848534852737967	0.967894880644892
Triqk	Triqk	-0.0096870976763110	4.91238434360885	0.0364465688012388	0.848596227082306	0.967894880644892
Snu13	Snu13	-0.0069612361527384	6.0649413645064	0.0364413574004061	0.848606921029592	0.967894880644892
Chkb	Chkb	0.010973464230753	5.80530567805992	0.036413967121959	0.848663139717314	0.967894880644892
RGD1564	RGD15640 5	0.0109060864953501	4.65621723903396	0.0363699823303883	0.848753462543788	0.967909418897618
Psmc4	Psmc4	-0.0061815258339448	6.6655993234724	0.0361784900675701	0.84914736433866	0.968270121080 67
Ccdc90b	Ccdc90b	-0.0081674714474431	4.38446429271748	0.0360547064816572	0.849402562329834	0.968370660294764
Limd2	Limd2	-0.0096207688952194	6.23383577322295	0.0360294302051329	0.849454728851735	0.968370660294764
Armc1	Armc1	-0.0057429170671776	5.91611937323947	0.0360034952379051	0.849508274523558	0.968370660294764
Fscn1	Fscn1	-0.0058388285492267	9.62867400270341	0.0359852261706174	0.849545984418249	0.968370660294764
Camk2n2	Camk2n2	-0.011062585383931	6.13091532350516	0.0358970020769931	0.849728351423176	0.968412624296194
Cdca3	Cdca3	-0.0132061398563811	3.29733300840138	0.0358280785791152	0.849870967816824	0.968412624296194
Tbc1d22b	Tbc1d22b	-0.0062096858912562	6.21612226386236	0.0358199054997783	0.849887888960494	0.968412624296194
Unc13c	Unc13c	-0.0179474610852076	4.57764052424404	0.0357156218381363	0.850103968522561	0.968412624296194
Itgb5	Itgb5	0.013745560 6011962	5.79942591826129	0.0357144771859852	0.850106342074523	0.968412624296194
Snf8	Snf8	-0.0075177758594492	5.13492840236699	0.0356788859224935	0.850180164489636	0.968412624296194
Acvr1c	Acvr1c	0.021428621444033	1.22339734176096	0.0356641345351179	0.850210772578797	0.968412624296194
Ncor2	Ncor2	0.0107864565832464	7.73270488867871	0.0356424245018161	0.850255831294328	0.968412624296194
Coro2a	Coro2a	-0.0081682534554048	4.4052952826407	0.0356160975668953	0.850310491363048	0.968412624296194
Ralgps2	Ralgps2	0.0094656974545054 6	5.22713276632571	0.0355927623483723	0.85035895751 26	0.968412624296194
Gltb1l2	Glb1l2	-0.0275582715025099	0.209290516419343	0.0355172571911879	0.850515891016356	0.968502945251015
Myef2	Myef2	-0.0076212888627486	7.97222623030635	0.0353763602578709	0.850809200481809	0.968521191348587
Nod1	Nod1	-0.0170082230629739	3.01244985848018	0.0353079996612564	0.850951726725159	0.968521191348587
Rbfox3	Rbfox3	0.0059574818552332 4	6.40273007424973	0.035245357049611	0.851082458914233	0.968521191348587
Pkib	Pkib	0.0328975713642599	0.785947777823642	0.0352316134262765	0.851111154856426	0.968521191348587
Alkbh1	Alkbh1	0.0102884879417886	4.63441546476411	0.0352263256835954	0.85112219769021	0.968521191348587
Thoc5	Thoc5	0.0050250136588886	6.37371369486125	0.0351955089071367	0.851186572011224	0.968521191348587
Gtf2h2	Gtf2h2	-0.0120609635805879	3.41699325002785	0.0351261935651479	0.851331474124035	0.968521191348587
Apln	Apln	0.013321165923 8131	3.43815735889476	0.0350463219176902	0.851498827584338	0.968521191348587
Slc29a1	Slc29a1	-0.0142441620378235	5.18255942416525	0.0350249995849481	0.851543283781238	0.968521191348587
Armc9	Armc9	0.0048141760843604 6	6.10105632829622	0.0349995096820379	0.851596686749893	0.968521191348587
Prokr1	Prokr1	0.0193485212921846	1.39717127625809	0.0349805216360232	0.851636460036559	0.968521191348587
Ppef2	Ppef2	-0.0196042150881471	2.4891724882292	0.0349252176546315	0.851752448184126	0.968521191348587
Ymel1l1	Yme1l1	0.0051511422609492 2	6.90649654481551	0.034904142285491	0.851796666245689	0.968521191348587
Ntrkb	Ntrkb	0.0129324936213555	6.03573729032393	0.0348151062233484	0.851983624686865	0.968521191348587
Col15a1	Col15a1	0.0177912110416036	3.86064223008177	0.0347527145019484	0.852114782644851	0.968521191348587
Nudt19	Nudt19	-0.0093610685642172	4.28265828315791	0.0347256413828916	0.852171732822466	0.968521191348587
Smc1a	Smc1a	-0.0050386658016407 9	7.54023866879298	0.0346932294285702	0.852239822779717	0.968521191348587
Bves	Bves	0.0210885551312323	1.05038245210704	0.0346293517439946	0.852374471094007	0.968521191348587
Spcs2	Spcs2	0.0059747352989604 8	6.77987548807639	0.0346179836828853	0.852398425835884	0.968521191348587
Pak1ip1	Pak1ip1	0.0074053472256915 5	4.40011217857087	0.0346128189191 29	0.852409310354009	0.968521191348587
Snx22	Snx22	0.0154376757772895 2	1.97806708307973	0.0346099155951194	0.852415429353894	0.968521191348587
Polr2c	Polr2c	-0.0075675278762482	5.47977214899959	0.0346028380159932	0.85243014049063	0.968521191348587
Lypla1	Lypla1	-0.0073737447745728	5.303901608053	0.0345817912395887	0.852474717549708	0.968521191348587
Cdc42ep3	Cdc42ep3	0.0073296620833337 7	5.71759765545877	0.0345620695777527	0.8525163071325	0.968521191348587
Lrwd1	Lrwd1	0.0081259320351699	4.77888798394712	0.0345455249972915	0.852551206268518	0.968521191348587
Ac1576	Ac1576	0.0106910426473607	2.92417271721124	0.0345185584431817	0.852608108049857	0.968521191348587
Yipf5	Yipf5	-0.0077562335934903	5.91497929473913	0.0344982954261752	0.852650879914977	0.968521191348587
Trmt13	Trmt13	-0.0223134988609251	2.82104581746554	0.0344627073072701	0.852726031990932	0.968521191348587
Mccc1	Mccc1	0.0086118270476217 2	3.82653737151134	0.0344077805029102	0.852842100747988	0.968521191348587
Sptbn1	Sptbn1	0.0088282973378686 8	10.0819839692239	0.0343990067326203	0.852860649943651	0.968521191348587
Lctl	Lctl	0.0150065091066796	1.29631285030916	0.0342910709461535	0.853089044488655	0.96854549781065
Sh2b1	Sh2b1	0.0044660228148478 08	6.91220965763765	0.0342400385107027	0.853197158708895	0.96854549781065
Smug1	Smug1	0.0088881843688236 1	3.73950785816918	0.0342153105605921 9	0.853250011710168	0.96854549781065
Bche	Bche	0.0218699665377076	1.20001910825685	0.0342010195884939	0.853279879818217	0.96854549781065
Aldh2	Aldh2	-0.0092475152906777	5.90887057704349	0.0341388925741635	0.853411620642779	0.96854549781065
Tmem38b	Tmem38b	-0.0101230032659477	3.5237292124319	0.0341360477981283	0.853417728360095	0.96854549781065
Dmpk	Dmpk	0.0231802789471798	5.14069671864881	0.0341344908568004	0.853421032225761	0.96854549781065
Rab8b	Rab8b	0.0096908485863610 6	3.54811975303892	0.0338408542851821	0.854045532622273	0.969041434152921
Fam117b	Fam117b	-0.0054712131731061	7.76755866782653	0.0338102207054405	0.854111080014544	0.969041434152921
Fhl2	Fhl2	0.0092001087158114 4	4.26267416854353	0.0337752093539276	0.854185806127232	0.969041434152921
Tuba1c	Tuba1c	0.0129077265376378	4.19720470713694	0.0337024377814146	0.854341199009113	0.969041434152921
RGD1560	RGD156008	0.0067286800712769 3	5.60889944371275	0.0336872009501619	0.85437376066056	0.969041434152921
Polr2h	Polr2h	0.0078649998076194 5	4.48729318296248	0.0336797970581257	0.854389214929139	0.969041434152921
Rfx2	Rfx2	0.020186669073245	2.06377584147647	0.0336720751428743	0.854406092579649	0.969041434152921
Atp9b	Atp9b	-0.0065312796400503	5.77598184362055	0.0336351249478834	0.854485106651299	0.969042962705713
Tsen34	Tsen34	0.0255015659480063	0.141257470071185	0.0335434620764019	0.854681311120981	0.969177380610422
Washc3	Washc3	0.0108573092035656	3.21468537593841	0.03345269324889 7	0.854875875222275	0.969309914249003
Slc22a3	Slc22a3	-0.0202238600585461	3.53581475180417	0.0333964279487304	0.854996617732478	0.96935872780083
Dyrk1a	Dyrk1a	0.0050034616184305 08	7.46821279399265	0.0332851953436233	0.855235626801 19	0.969541605996588
Bles03	Bles03	0.0095092871645120 8	5.64732282456616	0.0332435373763484	0.855325245000834	0.969553741343085
Apex2	Apex2	0.011197165255615 3	2.85832147856044	0.0331436233677421	0.855540403117473	0.969553741343085
Barhl2	Barhl2	-0.020666264905726	2.27533384502995	0.0331108461093521	0.855611088411469	0.969553741343085
Mzt2b	Mzt2b	0.0066840401147763 4	4.98355178253216	0.0331031201071079	0.855627729417691	0.969553741343085
Lurap1	Lurap1	0.0143359214482355	2.18149649687679	0.0330998198324295	0.855634867821106	0.969553741343085
Ppm1l	Ppm1l	0.0088743210106221 8	7.03825191145429	0.0330166858654053	0.855814287562221	0.969669985280951
RGD1305	RGD130517	0.0077226333247775	5.06733887520623	0.0329765024130424	0.855901095143571	0.969679284854839
Xrcc6	Xrcc6	-0.0076342627761126	4.47871634601996	0.0327505258897521	0.856390288806268	0.970078889493047
Lamtor3	Lamtor3	-0.0068289778453253	5.27294850806741	0.0327113460538939	0.856475282372713	0.970078889493047
Tbk1	Tbk1	0.0071805883924715 8	5.03011061382214	0.0327059188183298	0.856487059922 69	0.970078889493047
Sirt1	Sirt1	-0.0094678554054006	4.82980794432115	0.0326558149479444	0.856595837161476	0.970114029251497
Kcnn3	Kcnn3	0.0161559195912878	3.2996709342894	0.0326142306215473	0.856686183632398	0.970128291267148
Crk	Crk	0.0055141051325221 1	6.05075847611363	0.0325727163177721	0.856776437317134	0.970142445630808
Zfp772	Zfp772	-0.0167872382461946	1.50974380043337	0.0324727696196589	0.856993968160769	0.970300703558885
Mall	Mall	-0.0333875240332714	-0.232008563835 51	0.032365425812003	0.857227985959844	0.970445469103008
Paxx	Paxx	-0.0078952857752768	5.26278528072105	0.0322884029 88276	0.857396147895493	0.970445469103008
Faap20	Faap20	-0.0117651061872919	3.03249012300063	0.0322790519868335	0.857416577807527	0.970445469103008
Ube2v1	Ube2v1	0.0067299500842803 3	6.36388919648803	0.0322715616823412	0.857432944729627	0.970445469103008
Rtel1	Rtel1	-0.0150280085408933	6.52202611581501	0.0321201192725908	0.857764278265185	0.970732418257525
St3gal3	St3gal3	-0.0073841526064313	4.74555877811732	0.0320430077242406	0.857933298209091	0.970835038558941
Atg10	Atg10	0.0181322036076302	1.29019642291897	0.031944964805561	0.858148499486358	0.970991097123516
Tle2	Tle2	-0.0177584265464862	4.72718823024303	0.0318050870527173	0.858456117761165	0.971071020354872
Cmtm7	Cmtm7	0.0235132722094718	1.46572042385017	0.0317882195130608	0.858493258869162	0.971071020354872
Lat	Lat	-0.0330646200471676	0.406804185362824	0.03177512905242	0.858522091904945	0.971071020354872
Tns3	Tns3	0.0070899726609651 3	5.67645010777944	0.0317645212625948	0.858545460297066	0.971071020354872
Ndufs6	Ndufs6	0.021218179571292	0.927899489071205	0.0317163837012799	0.85865155538425	0.971071020354872
mrpl24	mrpl24	-0.0067675436165617	4.81531185446535	0.0317007146567079	0.858686107844458	0.971071020354872
Snap29	Snap29	-0.0042538557859745	6.23681935628751	0.0315058551980698	0.859116538708857	0.971215158255765
Pcdha8	Pcdha8	0.0120436063383916	3.22232732616334	0.0315019077992744	0.859125272404369	0.971215158255765
C1qc	C1qc	0.0230768642663413	2.32442024098228	0.0314928679724318	0.859145275321622	0.971215158255765
Arpp21	Arpp21	0.0174424246714102	7.6578434729031	0.0314510196499498	0.859237913992332	0.971215158255765
Agpat5	Agpat5	-0.0047268626643478	6.17918385989618	0.0314247673264134	0.859296060609349	0.971215158255765
Pdlim1	Pdlim1	-0.0179207787571709	2.45710824621193	0.0314217743177352	0.859302691454274	0.971215158255765
Chml	Chml	-0.0139385604978741	3.0087272582062	0.0313810783767536	0.859392883401439	0.971215158255765
Prorsd1	Prorsd1	0.0212359113732167	0.721977592107133	0.0313615029858099	0.859436286638527	0.971215158255765
RGD1560	RGD156080	0.0149559156276082	2.75405081290286	0.0312528453958265	0.859677473530441	0.971217913920128
Il1rap	Il1rap	0.0127457776367436	3.1957429763588	0.0311780065688843	0.859843709730007	0.971217913920128
Pgm3	Pgm3	-0.0073007991899332	4.87631655125075	0.0311594831012627	0.859885053273476	0.971217913920128
Caps2	Caps2	0.0352464617415754	-0.291907128443664	0.031149571896762	0.859907108563309	0.971217913920128
Cnnm3	Cnnm3	0.0067280213101767 5	6.1642852545291	0.0310793807420495	0.860063407078859	0.971217913920128
Trpc7	Trpc7	0.0131359939007435	3.52406363684454	0.0310146367517348	0.860207727518195	0.971217913920128
Cep295	Cep295	0.0208186909228079	5.39301291513947	0.0309973904413852	0.860246210020137	0.971217913920128
Rfesd	Rfesd	-0.0103362682036896	3.19310054467737	0.030965747444057	0.860316826884434	0.971217913920128
Chp1	Chp1	-0.0052121509777272	6.67538534219 12	0.0309444297221341	0.8603643997388	0.971217913920128
Jak3	Jak3	-0.0121571572998871	2.55358204551676	0.0309025042542572	0.860458076640339	0.971217913920128
Erc1	Erc1	0.0070876294482711 3	7.03363946296167	0.03072285679596 7	0.860857657409624	0.971217913920128
MGC1161	MGC11619	0.0190037992725695	1.17848988889431	0.0307109096727132	0.860888903119411	0.971217913920128
Bcl2l2	Bcl2l2	0.0077070242861096 5	5.66347135799105	0.0306886665152177	0.860936776917974	0.971217913920128
Fam50a	Fam50a	0.0071186434903089 7	4.91792365066096	0.0306142025365332	0.861103876401666	0.971217913920128
Prkd1	Prkd1	-0.0117872633898334	3.81651758291283	0.0306109812764817	0.861111109733046	0.971217913920128
Tango2	Tango2	-0.0069975770568913	5.08591547860002	0.0306008232277376	0.86113369758107	0.971217913920128
Dgkg	Dgkg	0.0121641362066797	4.17785871405883	0.0305188228479523	0.861318217632503	0.971217913920128
Kif3b	Kif3b	-0.0038653609805346	7.97590621976928	0.0305142368588776	0.861328532150268	0.971217913920128
Stx17	Stx17	0.011139956950 32	2.985746637164	0.0304607683812108	0.861448849075638	0.971217913920128
Pdzd4	Pdzd4	-0.0044382247654427	8.58609166203821	0.0304092607402779	0.861564856591911	0.971217913920128
Nabp2	Nabp2	0.0067748882630036	6.51162964541652	0.0303798575210836	0.861633830302564	0.971217913920128
Wsb1	Wsb1	-0.0168873816710984	7.25871934971701	0.030361826690779	0.861671778982212	0.971217913920128
Ect2	Ect2	0.0135036545945598	3.58111146177842	0.030353134925079	0.861691380658567	0.971217913920128
Tsen54	Tsen54	-0.0109561463715312	3.99371543628005	0.0303070011266016	0.861795470062352	0.971217913920128
Gpalpp1	Gpalpp1	0.0080384248947989	4.15435015288154	0.0302428926753049	0.861940024444385	0.971217913920128
Coq8a	Coq8a	0.0083561925870698 8	3.82092678827557	0.0301960221533264	0.862046201264034	0.971217913920128
Fam174a	Fam174a	0.0088878525386928 2	4.85855642989911	0.0301712698075391	0.862102188147619	0.971217913920128
Cpsf1	Cpsf1	-0.0065908413340706	6.75674724757733	0.030141446260302	0.862169678925145	0.971217913920128
Arhgap1	Arhgap1	-0.0053995177002944	6.80405330462293	0.0301216386043919	0.862214518391571	0.971217913920128
Hs2st1	Hs2st1	0.0039585365267372 1	7.22238263202655	0.0300775114540386	0.862314473294408	0.971217913920128
Col2a1	Col2a1	-0.0246901084577428	5.04339843812696	0.0300673600457718	0.862337478274925	0.971217913920128
Psme2	Psme2	0.0075504537796421 6	5.11699026336672	0.0300642945347747	0.862344426082718	0.971217913920128
Egfl7	Egfl7	-0.0138315704855544	4.25479685697726	0.029988893470005	0.862515432285819	0.971217913920128
Ube2e2	Ube2e2	-0.0067137456427996	5.90464574851776	0.0299726813127492	0.862552230853888	0.971217913920128
Nfatc1	Nfatc1	0.0175150029379473	2.03409680029511	0.0299657367846322	0.862567996342314	0.971217913920128
Rbm20	Rbm20	-0.0297090412778418	0.647545338475352	0.0299286173440123	0.862652296933044	0.971217913920128
Cyth1	Cyth1	-0.0062996275658556	5.25185657410405	0.0299159898818847	0.862680966290714	0.971217913920128
Akap8	Akap8	-0.0123034385336884	7.0645454018136	0.0298865296593378	0.862793399674601	0.971217913920128
Rasgrf2	Rasgrf2	0.0101489950416586	5.27807328885422	0.0298581751627296	0.862812423738817	0.971217913920128
Mtmr3	Mtmr3	0.0040868667323015 8	7.59553989968849	0.0298421953405992	0.862848775596545	0.971217913920128
Pno1	Pno1	-0.0083141041967337	4.56868610715627	0.0297841148688818	0.862980985028474	0.971217913920128
Lrrc57	Lrrc57	-0.0070124012788406	4.34487714126519	0.0297121149552382	0.863145063905939	0.971217913920128
Ttc26	Ttc26	0.0080577726030218	4.15834946413116	0.0297030943885419	0.863165635060401	0.971217913920128
Thumpd1	Thumpd1	0.0046655542732725 6	6.60222740474177	0.0296614237790891	0.863260705642524	0.971217913920128
Gnpda1	Gnpda1	-0.0074999235305890	4.42600201176371	0.0296590491829772	0.863266407716646	0.971217913920128
Pacs1	Pacs1	0.0056221183553606 7	7.64215078978419	0.0296571858780155	0.863270378169748	0.971217913920128
Hcn3	Hcn3	0.0056289996183118 1	6.0926335027798	0.0296201497017066	0.863354938330522	0.971217913920128
Usp33	Usp33	-0.0089271073911557	7.43515727809206	0.0296168089810473	0.863362277170681	0.971217913920128
Amy1a	Amy1a	0.0510070562696205	-0.0398590040878 18	0.0295912684011057	0.863420919024106	0.971217913920128
Arntl	Arntl	0.0063727046747686 8	5.4187612179115	0.0295477796432664	0.863520332209816	0.971217913920128
Zfp513	Zfp513	-0.0081599909932172 7	5.27944459125852	0.0295102354587853	0.863605988123422	0.971217913920128
Tuba1a	Tuba1a	0.0075932293614923	12.9853969502735	0.0294898480963184	0.86365287797749	0.971217913920128
Dleu7	Dleu7	-0.0141779334448927	3.92320018888882	0.0294772538721304	0.863681710943644	0.971217913920128
Man1a1	Man1a1	0.0121561505133741	4.27560019325154	0.0294748860117551	0.863687132584176	0.971217913920128
Utp15	Utp15	0.0066636078610287 3	5.44615703368898	0.0294395558016163	0.863768054073325	0.971217913920128
Nip7	Nip7	-0.0098507730498529	4.33689513865985	0.0293952201856058	0.863869672597993	0.971217913920128
Klf15	Klf15	0.0091805055941692 8	3.45895625816174	0.0293665032668144	0.863935534714278	0.971217913920128
Cpsf6	Cpsf6	0.0078518831447207 1	7.81265679002326	0.0293586793656857	0.863953484539513	0.971217913920128
Hs3st6	Hs3st6	-0.0318144576205607	-0.078572004876404	0.0292966763872755	0.864095820755726	0.971219005912036
Eif4h	Eif4h	0.0063630353529976 7	8.74765749045343	0.0292904436479804	0.86411013742222	0.971219005912036
Tmem147	Tmem147	-0.0080375891580185	5.76051365254589	0.0292221992462203	0.864266998207768	0.971267225247802
Slc25a35	Slc25a35	0.0229286816832882	0.661062298369174	0.0291833284591831	0.864356427572675	0.971267225247802
Fibin	Fibin	0.0240056203919436	1.29700163807996	0.0291702317190783	0.864386572826128	0.971267225247802
Trip6	Trip6	0.0109734579391091	3.5899248327202	0.0290743149250545	0.864607560683985	0.971428053548319
Map3k1	Map3k1	-0.0113996924831798	4.08887676578759	0.0289708402655577	0.864846382549445	0.971608888844281
Mrgpre	Mrgpre	0.0080849018699565 42	4.33816523747739	0.0289243730200042	0.864953772786919	0.971626188233403
Atp2a2	Atp2a2	-0.0055647862865382	9.5845247132904	0.0288968044863935	0.86501752825258	0.971626188233403
Ltbp1	Ltbp1	-0.0131136116513175	2.61394241210703	0.0287917868239163	0.865260680992759	0.971746779025649
Mapk1	Mapk1	-0.0041690711693443	8.69805627624548	0.0287821692811338	0.865282053867754	0.971746779025649
Coq7	Coq7	-0.0086927692517031	4.23900351528064	0.0287390903870798	0.865382632365788	0.971776701723961
Rpn2	Rpn2	0.0044607539057359 6	7.61053093128254	0.0286520481879506	0.865584933462423	0.971913526250059
Hecw2	Hecw2	0.0106998221010568 8	5.29764983728657	0.0284287094723368	0.866103486671755 8	0.972280042193851
Zgpat	Zgpat	0.0126013640988514	5.36989113279161	0.0284172906926585	0.866131506018699	0.972280042193851
Dnajc21	Dnajc21	-0.0118349920239259	3.09945657406778	0.0284114558188122	0.866145120540567	0.972280042193851
Zfp467	Zfp467	0.010019895588459	4.52348890178102	0.028265210121603	0.866490326687388	0.972519578083469
Scfd1	Scfd1	-0.0082133242408990	3.51169432658433	0.0282018735110938	0.866635096055466	0.972519578083469
Abcg3l4	Abcg3l4	0.026051222941511	0.712158975773379	0.0281971965921031	0.866649053286285	0.972519578083469

Hmox1	Hmox1	0.018715245160179	2.42306812912614	0.0281331178090909	0.866796245293676	0.972519576083459
Arl8a	Arl8a	-0.0057260227508985	9.00004971287805	0.0281216970188183	0.866823032880005	0.972519576083459
Nrdc	Nrdc	-0.0039364589211262	7.56138016814764	0.0281203568551405	0.866826176614912	0.972519576083459
Panx2	Panx2	-0.0087783498977517	6.28523937964723	0.0280591120948372	0.866969925884123	0.97256888716827
Poglut1	Poglut1	0.00611617388612545	5.00524372093356	0.0280264543873017	0.86704664362568	0.97256888716827
Usp16	Usp16	-0.0086777925475925	5.00455121514973	0.0279692155149007	0.867181217053913	0.97256888716827
Fam234a	Fam234a	-0.0076582152198870	4.53293101750436	0.0279689149481097	0.86718192432051	0.97256888716827
Tepsin	Tepsin	0.00857801339470493	5.13882921482479	0.0278782695529058	0.867394464475326	0.972700128129357
Rhbdl3	Rhbdl3	-0.0117466327166603	3.47169162262522	0.0278188619816149	0.867535386663317	0.972700128129357
Uqcc1	Uqcc1	-0.0071035489794033	4.76781245864035	0.0277898401541137	0.867603863047127	0.972700128129357
Btg1	Btg1	0.00820529350322191	6.46042946557509	0.027786908648693	0.867610781914853	0.972700128129357
Eqtn	Eqtn	-0.0484115802033934	-0.567480057509151	0.0276040822675157	0.868043027883427	0.972906635214794
Kat8	Kat8	-0.0049770863702445	5.19645834321559	0.0276032643166602	0.868044965027928	0.972906635214794
Mpg	Mpg	0.0102485398770326	2.99988612215701	0.0275883657391773	0.868080254154225	0.972906635214794
Akt1s1	Akt1s1	0.00585506172546764	6.15544441619964	0.0275771264634059	0.868106882251046	0.972906635214794
Afg3l1	Afg3l1	-0.0072752822050072	5.69607742350666	0.0274643524220295	0.86837437581686	0.97299829512869
Rxylt1	Rxylt1	-0.0067791314733671	4.80398014409178	0.0274532898340993	0.868400646009197	0.97299829512869
Hmmr	Hmmr	-0.0092052084518678	3.63143611471905	0.0274408993931464	0.868430550993007	0.97299829512869
Osbpl1a	Osbpl1a	0.00705290685412124	7.07887739465664	0.0274112184297239	0.868500602135788	0.97299829512869
Ctrl	Ctrl	0.0220446967405687	1.7829049818599	0.027266463283766	0.868845124875826	0.973234876903607
Nsg2	Nsg2	0.00604487428910093	10.7731412768082	0.027256958177162	0.868867782221951	0.973234876903607
Arl5a	Arl5a	0.00547009075885695	5.16526323382254	0.0272164080865878	0.86896447565205	0.973255814649919
Rpain	Rpain	-0.0103835428268373	3.01695102479769	0.027094506740255	0.869255611072176	0.973494503127865
Adgrb3	Adgrb3	-0.0046003342540754	7.45859504604889	0.0269816602395494	0.869525720165663	0.973576971203142
Gtf3c2	Gtf3c2	0.00588306616609851	6.27290242492188	0.0269761465242908	0.869538932630968	0.973576971203142
Pimreg	Pimreg	-0.0137578041639438	2.88557620998339	0.0269618501927056	0.869572197259681	0.973576971203142
Tmem59	Tmem59	-0.0062464334563198	7.05954126076435	0.0269129298725801	0.869690517316462	0.973576971203142
Mars1	Mars1	-0.0045748921541440	7.09969324697777	0.0268835929708331	0.869760925208213	0.973576971203142
Nrarp	Nrarp	-0.0084381611736874	5.28967121823486	0.0268683901279587	0.869797427201583	0.973576971203142
Ssr2	Ssr2	0.00799104848145957	6.20484575342746	0.0268160345387258	0.869923214069682	0.973630421774975
S100pbp	S100pbp	0.00763232312947221	5.55570550174986	0.0267581291561925	0.870062481519179	0.97369894895539
Cds2	Cds2	-0.0092175731984923	5.5682114000163	0.02672064269386	0.870152722218366	0.973712602252785
Gale	Gale	-0.0081739698607851	4.15234959396004	0.0266296072652992	0.870372141609881	0.97387079293183
Ctbp1	Ctbp1	0.00467183625323992	8.61856996282281	0.0265429858839647	0.87058128021527	0.974017452765955
Ilf2	Ilf2	0.00469788200635721	7.57908649637736	0.026420488423021	0.87087763578203	0.97426165711263
Agpat1	Agpat1	0.00421186232190165	7.74212525299276	0.0263455724072941	0.871059226452416	0.974377440240882
Prps2	Prps2	0.00839958227552268	5.3020724146091	0.0263066956259905	0.871153565221685	0.974395610333569
Prpf31	Prpf31	0.00524094733112118	5.28245033163835	0.0262527122791596	0.871284680648591	0.974400561296722
Nrp2	Nrp2	-0.0217315302891297	6.39919160779168	0.0262405731262305	0.871314183177304	0.974400561296722
Nupr2	Nupr2	0.0112505237035889	4.24334411847125	0.0261429367950967	0.871551731485941	0.974534478790949
Epn1	Epn1	-0.0052030520345139	8.54359638884982	0.0261271654202346	0.871590145774481	0.974534478790949
Thtpa	Thtpa	-0.0047932704323232	6.77162730029574	0.026057349033664	0.871760340401331	0.974591632615392
Adam15	Adam15	-0.0063898672490007	5.5066611300665	0.0260421247901022	0.871797484300745	0.974591632615392
Sdk2	Sdk2	0.0126095683360077	4.81637207206206	0.0259882304264067	0.871929064624978	0.974630781765417
Tjp2	Tjp2	0.0088580787134072	4.47344186350367	0.0259638097177231	0.871988732615801	0.974630781765417
Nelfa	Nelfa	0.00519050820138644	5.14112241110434	0.0258650806194325	0.87223025491205	0.974719542994852
Slc9a9	Slc9a9	-0.0120661389539883	2.59766331703485	0.0258583303171847	0.872246785523087	0.974719542994852
Rbm28	Rbm28	0.00653093362210382	4.75834228379208	0.0258171198011325	0.872347752749643	0.974719542994852
Lrrc56	Lrrc56	-0.0092582303285035	3.61689064057347	0.0257906858744619	0.872412560331332	0.974719542994852
Aspm	Aspm	0.0146282198078898	4.26013388792189	0.0257718533005118	0.872458752597922	0.974719542994852
Gmds	Gmds	0.00769163637640274	4.53648122248513	0.0257181299701514	0.872590619626967	0.974779582870952
Myt1	Myt1	-0.0123630688819146	5.05765153405325	0.025582348492745	0.872922071682315	0.974884355214327
Igfbpl1	Igfbpl1	0.014231950013215	6.12295374984746	0.0255264042192636	0.873062377008848	0.974884355214327
Tep1	Tep1	0.0110659851534735	4.5735938694209	0.025517407857194	0.873084557871324	0.974884355214327
Gosr2	Gosr2	-0.0047010349825017	6.4353951526658	0.0254645601187136	0.873214936958311	0.974884355214327
Lama3	Lama3	-0.0208967015230366	4.00649482839061	0.0254382375501052	0.873279928347123	0.974884355214327
Fuom	Fuom	-0.0133590482294402	2.17495557975048	0.0254253944401626	0.873311650973386	0.974884355214327
Zfp641	Zfp641	-0.0084608303741079	4.56359786555366	0.025401298895499	0.873371189908534	0.974884355214327
Rab33b	Rab33b	-0.0059685532994638	5.29444132425469	0.0254006862350806	0.873372703633571	0.974884355214327
Prelid3a	Prelid3a	-0.0098689654696775	4.21770771624145	0.0253946517643442	0.873387619115456	0.974884355214327
Ptpru	Ptpru	0.0105298810447032	6.00982273069139	0.0252298040569343	0.873795780483003	0.975252701769863
Znhit2	Znhit2	0.0109692140596784	3.95407347254546	0.0251081191856599	0.874097948820839	0.975414800890269
Mss51	Mss51	0.0254559070451904	1.51738166195771	0.0250838601227912	0.874158278693754	0.975414800890269
Angel1	Angel1	-0.0097837922332121	4.01448939692765	0.0250742705210794	0.874182135274587	0.975414800890269
Golph3l	Golph3l	0.00694518973570601	4.52562717523874	0.0250442340698172	0.874256889095571	0.975414800890269
Rps27l	Rps27l	0.0125751482176057	3.62080698301485	0.0250141123055272	0.874331901251028	0.975414800890269
Prkar2a	Prkar2a	0.00608808725329522	6.23533937974361	0.0249470915023802	0.874498675966587	0.975527081293019
Ccdc189	Ccdc189	-0.0155803126596064	1.27325656823792	0.0248323120951882	0.874783602292697	0.975527081293019
Ddx3	Ddx3	-0.0728465229270367	4.84637068124661	0.0248144854034535	0.874830206773261	0.975527081293019
Tmed5	Tmed5	0.0082579471857316	3.75905166750565	0.0247858568946739	0.874901826976482	0.975527081293019
Nppa	Nppa	-0.0192489655619611	1.086905070367	0.0247454519852108	0.875003004599412	0.975527081293019
Ythdf1	Ythdf1	-0.0034036986492731	6.7440029202184	0.0246852387062404	0.875153926481567	0.975527081293019
Ormdl2	Ormdl2	0.00643867115511303	3.75798628584738	0.0246597249299098	0.875217923307644	0.975527081293019
Sgk1	Sgk1	0.00922032797919986	5.70265207618322	0.0246342650068527	0.875281837256495	0.975527081293019
Irf2bpl	Irf2bpl	0.00477953863475268	7.68324133373495	0.0245688893476354	0.875446578648198	0.975527081293019
Kif20a	Kif20a	0.00946575548981136	4.70350272033002	0.0245631232012755	0.875460583737306	0.975527081293019
Dclk2	Dclk2	-0.0042522594014320	8.11308233000467	0.0245517612424351	0.875489155638143	0.975527081293019
Pnpla7	Pnpla7	-0.0101231616145526	2.84172670536513	0.0245291060438291	0.875546148857304	0.975527081293019
Prep	Prep	-0.0042212876080083	6.91197427889767	0.0245123303553343	0.875585915429154	0.975527081293019
Pdcd2	Pdcd2	0.00653117476912073	4.46979593102555	0.02447452772563	0.87568355442005	0.975527081293019
C1s	C1s	0.0180728659845666	1.97105754336254	0.0244418304106659	0.875765946085552	0.975527081293019
Hsd17b1	Hsd17b13	0.0258220665712949	0.910280792152457	0.0244228586701532	0.875813779744604	0.975527081293019
Nlrp1a	Nlrp1a	-0.0321008239145687	0.0397026740761828	0.0244145229510701	0.87583477979279	0.975527081293019
Pla2g7	Pla2g7	0.0277578080284674	0.422493712588142	0.0244121767563001	0.875840722799616	0.975527081293019
Il3ra	Il3ra	-0.0127166386071826	4.48889964917321	0.0243815177113618	0.875918080606371	0.975527081293019
Slc17a7	Slc17a7	-0.0099560095471087	7.61172907787376	0.0242174684304217	0.876332853007685	0.975888117844454
Dynlrb2	Dynlrb2	0.0323100085580912	1.3190932297249	0.0241789849738723	0.876430360463535	0.975888117844454
Tsc22d3	Tsc22d3	0.00607224502950198	5.85381340345688	0.0241606292086232	0.876476897376368	0.975888117844454
Fam13a	Fam13a	0.00973020721359923	2.96205941136666	0.02406961801573	0.87670790365835	0.97590769066967
Ubp1	Ubp1	-0.0094581315674646	7.10546090748636	0.0239728104897292	0.8768954113768426	0.97590769066967
LOC6828	LOC682870	-0.0144915468690581	1.64504608977122	0.0239680780631417	0.876966162711682	0.97590769066967
Rab3gap	Rab3gap2	0.00794911746056974	6.07427618888933	0.0239528248848621	0.877005006230939	0.97590769066967
Cep76	Cep76	-0.0079796524515921	3.27639455682353	0.0239215173336405	0.877084773655367	0.97590769066967
Basp1	Basp1	0.00758122893722321	10.4914900892945	0.0239173649132295	0.877095408439176	0.97590769066967
Dhrs1	Dhrs1	0.0121456545625085	4.56902037070482	0.023909401899413	0.87711565695406	0.97590769066967
Siah1	Siah1	-0.0047734347734942	6.19800994127368	0.0239076163225818	0.877120208637468	0.97590769066967
Rgs3	Rgs3	-0.0069567756848767	6.0160142121113	0.0238154774851438	0.877355346219795	0.975970840423443
Sdhb	Sdhb	-0.0053282844413123	6.76307171036908	0.0238022221208354	0.877389212210677	0.975970840423443
Med29	Med29	-0.0070375737347191	4.80179137452369	0.0237604151295727	0.877496087427431	0.975970840423443
Cops8	Cops8	-0.0057578062532906	6.74415797383036	0.0237128058075743	0.877615353844434	0.975970840423443
Arpin	Arpin	0.0102009420593312	2.6411064066158	0.0237110695565602	0.877622409383248	0.975970840423443
Cog8	Cog8	-0.0048708074775447	5.49048708012254	0.023701720949532	0.87764629554789	0.975970840423443
Irs3	Irs3	-0.029896819485913	-0.21882282225242	0.0236495023781202	0.877780091637736	0.976032635537299
Rab10	Rab10	-0.0038710890906924	8.80597582868245	0.0236118701500345	0.877876607907281	0.97604711303024
Adam1a	Adam1a	0.0126591046742355	3.96583372160889	0.0235632073017626	0.878001531471697	0.97604711303024
Rabac1	Rabac1	-0.0078202166408060	7.29913908377902	0.023548132628477	0.878040258808243	0.97604711303024
Dpp3	Dpp3	0.00453199990272382	6.56559142632857	0.0235225431927475	0.878106022582707	0.97604711303024
Prox1	Prox1	0.0204079180088088	4.63228606767829	0.0234677570220612	0.878246948239634	0.97604917564998
Polr3c	Polr3c	-0.0053755733694934	4.69461847141797	0.0234474600869774	0.878299200480059	0.97604917564998
Rfx5	Rfx5	0.0104216015249532	5.34076330753415	0.0234155555196907	0.878381382108921	0.97604917564998
Myh14	Myh14	-0.0102562176509072	3.70448203183681	0.0233523178262942	0.878544442926979	0.97604917564998
Sema3e	Sema3e	-0.0126561757702375	3.6080750766725	0.0233078835071439	0.878659153717452	0.97604917564998
Bhlha15	Bhlha15	-0.0168952762487848	1.24328044193318	0.0232981956792102	0.878684178482437	0.97604917564998
Apba2	Apba2	-0.0040244754991686	8.05313677869128	0.0232880858318083	0.878710299049563	0.97604917564998
Mcat	Mcat	-0.0071711302172816	4.59161911189446	0.0232772715550666	0.878738248056716	0.97604917564998
Faim	Faim	0.00698429785265548	4.19471935180836	0.0232487717446546	0.878811929089675	0.97604917564998
Rab29	Rab29	-0.0103142091465646	2.35013365786214	0.02309894754469	0.879200044854856	0.976393320841481
Nup62	Nup62	0.00465510656092615	6.25419211081936	0.0230473724426616	0.879333946655423	0.976399072200075
Tp53inp2	Tp53inp2	-0.0040681302112280	8.0047307312675	0.0230345921287798	0.879367177206236	0.976399072200075
Slc26a4	Slc26a4	-0.0272982955260578	-0.082756022888954	0.0230065688960082	0.879439991459033	0.976399072200075
Nol12	Nol12	0.00878928967207940	3.37959356716224	0.0229222882269227	0.87965933443646	0.976463786472329
Rexo2	Rexo2	-0.0067165298471375	5.89856585678403	0.0229004713949763	0.879716180578944	0.976463786472329
Kat7	Kat7	0.00394896768032238	6.68182737894781	0.0228778258159821	0.879775215416631	0.976463786472329
LOC6858	LOC685883	0.0223072092457299	0.558222158167589	0.0228639803863473	0.879811323837615	0.976463786472329
Tmed3	Tmed3	0.00784646602416723	4.2705430235167	0.0228204020290974	0.879925047796779	0.976503141631274
Sec61b	Sec61b	-0.0083465192510987	5.22685760384404	0.0227821287831852	0.880024992816513	0.976527199872967
Def8	Def8	0.00475854908896713	5.95529335231504	0.0227144613338268	0.880201978249293	0.976570465516345
Dcun1d1	Dcun1d1	0.00590904269974767	4.79089032822919	0.0227073767018737	0.88022052217655	0.976570465516345
Ddx54	Ddx54	0.00443019412448464	6.37564853869608	0.0226732758839561	0.880309555007297	0.976582405781634
Mrpl47	Mrpl47	0.00667059915397085	3.80320586302986	0.0226240966476681	0.880438717043405	0.9765838857601437
Ccnc	Ccnc	0.00533076897559837	5.6112201496227	0.0225812802433225	0.880551056455708	0.976647750611583
Als2	Als2	0.00418633172684906	6.16589049283094	0.0225007626862972	0.880762609569623	0.976647750611583
St18	St18	0.0218517913858883	3.23542566804374	0.0224920685974155	0.880785475635292	0.976647750611583
Arvcf	Arvcf	0.00368517218708973	7.40856151529771	0.022466877666524	0.880851755235472	0.976647750611583
Tctn1	Tctn1	-0.0074529603066622	4.46575803744251	0.0223719473000727	0.881101867531414	0.976647750611583
Ifnlr1	Ifnlr1	0.036093214575168	0.281034014927426	0.0223382035402153	0.881190900612026	0.976647750611583
Slc25a14	Slc25a14	-0.0060818811645386	4.63593349678165	0.0223278436064831	0.881218251755502	0.976647750611583
Urgcp	Urgcp	0.00479789129556087	5.61813202176314	0.0223268938867704	0.881220758240939	0.976647750611583
Mrpl51	Mrpl51	-0.0051534712667601	5.20358702275928	0.0222925295213923	0.881311526078261	0.976647750611583
Dcxr	Dcxr	0.0139094175898549	1.7153018824301	0.022265881979757	0.881381695170257	0.976647750611583
Mylpf	Mylpf	-0.0201265498958677	0.190692819138236	0.0222438144006745	0.881440320183024	0.976647750611583
Bmpr1a	Bmpr1a	-0.0060294316691191	5.96693329351041	0.0222311329895888	0.88147387123944	0.976647750611583
Ufsp2	Ufsp2	-0.0061662192942409	4.72136500943618	0.0222193284845505	0.881505081941757	0.976647750611583
Izumo1	Izumo1	0.0303042593476779	0.347002078475062	0.022125992219614	0.881752418449023	0.976647750611583
Mrps10	Mrps10	0.0101531089963188	3.6846589749999	0.0221025147743603	0.881814709076539	0.976647750611583
Efcab1	Efcab1	0.0247253224605662	1.89380019448509	0.0220900652792205	0.881847753944117	0.976647750611583
Snx19	Snx19	0.00626509165982251	5.97549823735196	0.0220842942882911	0.881863075189913	0.976647750611583
Tmem132	Tmem132a	-0.0052641748071638	9.53402670046959	0.0220702480737884	0.881900374659249	0.976647750611583
Cabp1	Cabp1	-0.0099105910880368	4.82338023727395	0.0220575987831779	0.881933975005266	0.976647750611583
Tmem51	Tmem51	0.0156921344020062	2.08060078395316	0.0219699104614056	0.882169318114484	0.97682167040619
Tpk1	Tpk1	-0.0098978930397485	2.89862063702204	0.0218813060324123	0.882403290815488	0.976913716637067
Sqor	Sqor	0.0310985646630474	-0.011332577535292	0.0218546450677195	0.882474433964896	0.976913716637067
Rbm43	Rbm43	0.0179211477257867	1.27496463332286	0.0218122813803916	0.882587570136885	0.976913716637067
Bmyc	Bmyc	0.00777508293906145	5.87621338072142	0.0217858914689657	0.882658103608569	0.976913716637067
Acy3	Acy3	-0.0145729344701642	1.56584269889257	0.0217748523421051	0.882687621214402	0.976913716637067
Mon1b	Mon1b	0.0044696114346605	5.24679922934635	0.021738477323602	0.882784928222911	0.976913716637067
Rps10	Rps10	-0.0073675880379545	7.84079101850189	0.0217324823029267	0.882800983637774	0.976913716637067
March6	March6	-0.0045806505237427	8.83782017528722	0.0216733630177686	0.882959354239803	0.976913716637067
Tpx2	Tpx2	-0.0104066276543747	4.30209207112713	0.0216455148874353	0.883034030569017	0.976913716637067
Cox5a	Cox5a	-0.0182192857215886	0.771947003289543	0.0216446978801592	0.88303541688566	0.976913716637067
Rbm48	Rbm48	0.0085213351094258	3.0945502952939	0.0215807402265398	0.883207915501417	0.976956942010975
Lrrc2	Lrrc2	0.0208374827248984	0.0359634827572053	0.0215721150253714	0.883231089675707	0.976956942010975
Rnh1	Rnh1	0.00738111446667945	4.97653583042252	0.0215307417660568	0.883342317127744	0.976993088747372
RGD1304	RGD13045	-0.0071019046452264	4.07848994717427	0.0214730574495547	0.883497577428428	0.976993088747372
Cep70	Cep70	-0.0074879803992932	4.28954442263968	0.0214726482887944	0.883498679196649	0.976993088747372
Gfra4	Gfra4	-0.0134523492382853	2.56748203518767	0.021388859511258	0.883724512035	0.977082751991577
Odf3	Odf3	0.0203212152941729	0.20741672395011	0.021363176707041	0.883793917570337	0.977082751991577
Samd10	Samd10	0.00591699853702475	5.21404485990707	0.0213554837017114	0.883814694155401	0.977082751991577
Sec5d	Sec5d	-0.0202075791034267	2.47890676214763	0.0213092314338361	0.883939688278449	0.977121185656393
Osr1	Osr1	-0.0083241176878639	8.40116489888445	0.0212846827929525	0.884006086694667	0.977121185656393
Plk5	Plk5	-0.0114472181517478	3.63963194205118	0.0212314942018565	0.884150092684626	0.977193777993233
Clspn	Clspn	-0.0113181347124677	2.92654470549934	0.0211506411879299	0.884369327069061	0.977236381700338
Ints10	Ints10	0.00805045357256561	6.29273805311096	0.0211285498035352	0.884429305285632	0.977236381700338
Vps11	Vps11	-0.0036663198207251	6.13182492675097	0.0211183120179896	0.884457111926948	0.977236381700338
Sv2c	Sv2c	-0.0199301442691538	4.02147342449234	0.0211018198754402	0.884501926216552	0.977236381700338
Paqr7	Paqr7	-0.0080086953088401	3.98370246057919	0.0210263292649344	0.884707252852654	0.97727284527585
Ergic2	Ergic2	-0.0040157582466553	5.82383896119092	0.0210141268394758	0.884740451235624	0.97727284527585
Tp53bp1	Tp53bp1	0.00669808922115975	8.29483808247352	0.0210033239914509	0.884769901437525	0.97727284527585
Ccnl1	Ccnl1	0.00999467039098752	5.19667950674774	0.0209258861014491	0.884953479409619	0.977350740823464
Rps7	Rps7	-0.0052955994156938	9.07321730324291	0.0209129424420951	0.885216370722185	0.977350740823464
Ccdc58	Ccdc58	-0.0090516202732428	2.938002355541	0.0208864279884651	0.885288778284885	0.977350740823464
Bbox1	Bbox1	-0.0381636297604249	-0.382921019532413	0.0208626504567579	0.885353752609212	0.977350740823464
Pank4	Pank4	-0.0067214227519386	5.37266343345626	0.0208041535354084	0.885513758858851	0.977440914014856
Slc7a14	Slc7a14	-0.011815151533555	4.22760149172404	0.0207215504330378	0.885540095230971	0.977537557748694
Fcgr2a	Fcgr2a	0.0196209441954255	0.963118276701283	0.020715027560442	0.885557987916446	0.977537557748694
Chrnb2	Chrnb2	0.00423508319410816	6.11374152549978	0.0206773716080235	0.885661336451113	0.977565153476893
Ggh	Ggh	-0.0070587687224413	4.70126376711267	0.0205792982042868	0.885930065239278	0.977776251969866
Slc1a1	Slc1a1	-0.0045764761006551	5.93508270346036	0.0205446927974364	0.886026247241752	0.977794930730174
Srsf1	Srsf1	0.00432980705153273	8.53667548576082	0.0204780870324086	0.886209888599421	0.977854123299907
Clec11a	Clec11a	0.00831173305481904	3.1650340127957	0.0204683974516655	0.886236629500308	0.977854123299907
Polrmt	Polrmt	0.00664596799191914	5.16984549328935	0.020389200648772	0.886455436114973	0.97798042425349
Recql4	Recql4	-0.0095240412167312	2.87717824866748	0.0203702483020862	0.886507862331248	0.97798042425349
Mms22l	Mms22l	-0.0121721421444427	2.66186988141536	0.0202811281873352	0.886754721788701	0.978040093237636
Csnk2a2	Csnk2a2	0.00396479202485268	5.67154214799262	0.0202578842488819	0.88679145420825	0.978040093237636
Ercc8	Ercc8	-0.0038278710009457	6.18583770750339	0.0202565844395283	0.886797112761776	0.978040093237636
Plp2	Plp2	0.0142193894365111	2.2505843767355	0.0201798290829487	0.887035898542429	0.978070682952973
Psmd8	Psmd8	-0.0061016955770866	6.75129136625221	0.0201251923034391	0.887187993402652	0.978070682952973
Aplm1	Aplm1	-0.0077927508908405	4.77529000275737	0.0201072185394366	0.887238043842957	0.978070682952973
Shtn1	Shtn1	0.00358328364105871	8.29183216295537	0.0200721961906041	0.887335634029279	0.978070682952973
Las1l	Las1l	0.0116957991142385	6.32294888429546	0.0200701501503744	0.887341338028477	0.978070682952973
Kcnh7	Kcnh7	0.0111664515427921	3.86115590855479	0.0200446342635558	0.887412496746334	0.978070682952973
Mrpl3	Mrpl3	0.00572200257697814	5.58949612083273	0.0200285443934831	0.887457391937306	0.978070682952973
Gas6	Gas6	0.00689667506012852	6.65216885893539	0.0200075079172422	0.887516117284063	0.978070682952973
Pim3	Pim3	-0.0050639216127696	5.92727715458592	0.0200005471406968	0.887535555894604	0.978070682952973
Idua	Idua	0.0164932637850488	5.30716377959919	0.019743490088196	0.887608747541598	0.978070682952973
RGD1311	RGD13111	0.00492580231216861	4.83870791123265	0.0196106264336998	0.887786950613602	0.978138213014446
Lrrc41	Lrrc41	-0.0037998532917120	6.60984254291391	0.0198963953871143	0.887826822344201	0.978138213014446
LOC1025	LOC10255	-0.0085538189699922	3.78241944023547	0.0197755296802358	0.888165806713049	0.978309835539407
Ppa2	Ppa2	0.00631019650408137	4.77495454545699	0.0197620904529435	0.888203564079119	0.978309835539407
RGD1310	RGD13108	0.00725490626640295	3.50688422875507	0.0197461974668798	0.888248232120469	0.978309835539407
Iqgap3	Iqgap3	-0.0114416660368027	3.70655118615211	0.0197291251281513	0.888296235218878	0.978309835539407
Ddx20	Ddx20	-0.0006032853290145	4.65217880992754	0.0196589773005442	0.888493695806876	0.97844093932766
Mrps11	Mrps11	-0.0055944880017421	5.04136178477072	0.0196112487207571	0.88862825341096	0.978460717617928
Tlk1	Tlk1	0.00414890358958497	6.85823357807505	0.0195969850850465	0.888669498203398	0.978460717617928
Dennd6a	Dennd6a	0.01055900420953	5.40124276867222	0.0195636345981995	0.888762654787152	0.978478041452333
Rsrc2	Rsrc2	0.00401586991245212	6.43316577870674	0.0195087354039174	0.88891782702976	0.97856252113202
Dgka	Dgka	-0.0111774320882371	3.90680982692616	0.0194791123845448	0.889001648782004	0.978569465859556
Hyal1	Hyal1	-0.0182113363186605	1.35961659944475	0.0193525044699605	0.889360634842173	0.978877261902416
Ptprt	Ptprt	0.0109603766142786	6.66049723889503	0.019298216574508	0.889514645596706	0.978938063724028
Hspb11	Hspb11	-0.0099029804875527	2.30739276142	0.0192573729277967	0.889631160730743	0.978938063724028
Pank2	Pank2	-0.0058362676196679	4.42607523419196	0.0192503163399351	0.88965125467394	0.978938063724028
Sparcl1	Sparcl1	-0.0072290611846126	9.10440825712478	0.0192108057375719	0.889763822260512	0.978947577720017
Slc30a10	Slc30a10	-0.0060977957312739	4.94545851437944	0.0191922228595089	0.889816821264945	0.978947577720017
Rspo1	Rspo1	0.0175928149595739	0.699967390498635	0.0191340532874733	0.889982960805852	0.979043921202245
Lpin1	Lpin1	0.00851431714429272	4.39419396789555	0.0190560309820469	0.89017657795671	0.979170692223679
Ctsl	Ctsl	-0.0078322753433177	6.6783789993049	0.0190257603942712	0.890292658220782	0.979191975924597
Edar	Edar	-0.0280968476034996	-0.143386788974783	0.0189781574389514	0.890429623396872	0.979191975924597
Sat2	Sat2	0.0074618157030509	4.05808754817802	0.0189642242586672	0.890469098858864	0.979191975924597
Neurl4	Neurl4	0.00290773102926827	7.93744202182195	0.0189240751862627	0.890584373668811	0.979191975924597
Mxd1	Mxd1	-0.0059652682550409	4.56355966754838	0.0189143606983655	0.890612192421843	0.979191975924597
Hnrnpab	Hnrnpab	0.00556584378735216	8.99210060727886	0.0188807106012242	0.890705557948628	0.979191975924597
Prxl2b	Prxl2b	-0.0082948792657779	4.10916051309809	0.0188385324076183	0.890830397604503	0.979191975924597
Actg1	Actg1	0.00535008464135328	11.8317416836492	0.0188240371980726	0.890872142882518	0.979191975924597
Gpr135	Gpr135	-0.0076381007628682	3.45281740160403	0.0188125883276175	0.890902244986457	0.979191975924597
Dnai4	Dnai4	0.00861385860257222	3.69289706168934	0.0187812571417538	0.890995441738676	0.979208149969834
RGD1565	RGD15652	0.00833089901106634	2.86017659493383	0.0186537807101459	0.891363698580082	0.979441834653125
Scaf1	Scaf1	-0.0030332256732870	8.21107420132797	0.0186532056521156	0.891365074326676	0.979441834653125
Cdk10	Cdk10	0.00430966637830145	5.61035251247112	0.0186018932153734	0.891514540912708	0.979519806224168
Mctp2	Mctp2	0.0243325351244497	-0.280568124399121	0.0185053330585134	0.891794158884115	0.97964491913683
Fgfrl1	Fgfrl1	-0.0088950524040927	3.54260215746445	0.0184850732063993	0.891853041240227	0.97964491913683
Trim47	Trim47	0.00941955966266096	2.87931002321734	0.0184436493341735	0.891973542543255	0.97964491913683
Ephb1	Ephb1	0.00505881966651354	7.15242758147419	0.0184187093659318	0.89204615767753	0.97964491913683
Sec62	Sec62	-0.0043494650416657	6.43486759802998	0.0184078973632088	0.892078236124151	0.97964491913683
Irx3	Irx3	0.0356407671341577	0.564225655814776	0.0184003966547976	0.892099508794795	0.97964491913683
Mrpl37	Mrpl37	0.00479450588673753	5.88715287315028	0.0183670108224661	0.892196842631397	0.979662640795339
Dnpep	Dnpep	0.00417575465770014	7.15763193336401	0.0183410191540361	0.892272681734249	0.979662640795339
Pcmt1	Pcmt1	-0.0043805632565152	6.91850942409871	0.0182966145342287	0.892432973761826	0.979752418509244
Eps8l1	Eps8l1	-0.0202231806382362	-0.547441201443625	0.018169573594605	0.892774206520085	0.979952618575728
Cyrib	Cyrib	-0.0032139662659568	7.48509449209266	0.0181460738027468	0.892843250965999	0.979952618575728
Mapk8ip3	Mapk8ip3	-0.0060948603104928	8.3640272396518	0.0181293026259084	0.892892492583055	0.979952618575728
LOC4982	LOC498265	-0.0112616748890182	2.91697222562135	0.0181166986006218	0.892929498830684	0.979952618575728
Alg14	Alg14	0.00669062439440068	3.90025658058114	0.0179255232013497	0.8934925077583	0.980429083798832
Grb7	Grb7	0.029433003661129	-0.152479223188139	0.0178917542806118	0.893592308949053	0.980429083798832
Tsnaxip1	Tsnaxip1	0.0230286531523047	-0.081110905526948	0.0178893601484467	0.893599306147428	0.980429083798832
Ube2s	Ube2s	0.00646558581620694	7.36996322019112	0.0178610803751748	0.893683215042121	0.980434775514329
Rab6	Rab6	-0.0030080579942460	6.78488517229407	0.0177814529799142	0.893918871152981	0.980483703690104
Fam20a	Fam20a	0.0153189319122939	1.2226794974569	0.0177479144124533	0.894018367099668	0.980483703690104
Rnf13	Rnf13	0.00478343359432128	5.6948716302007	0.0177452259393619	0.894025457903493	0.980483703690104
Mipep	Mipep	0.00589407469089678	4.78884786015545	0.0177344022553498	0.894055484067108	0.980483703690104
Rhoq	Rhoq	0.00677971203272993	3.16881669460246	0.0177134647995274	0.894120668476878	0.980483703690104
Pip4k2c	Pip4k2c	0.0039115529085021	5.49420822785699	0.0175997496154565	0.894450077070231	0.980581837511607
Zfp523	Zfp523	0.00385886569370928	6.02958829351735	0.0175990680176729	0.894461108807429	0.980581837511607
Vps29	Vps29	-0.0055112400107477	6.21913016465145	0.0175481815268625	0.894612906770765	0.980581837511607
Adra1b	Adra1b	-0.0109079538645872	3.60359003156868	0.0174947176234355	0.89477263479009	0.980581837511607
Fndc7	Fndc7	0.0212881700997766	0.813631021538533	0.0174924965984502	0.894779275662517	0.980581837511607
RGD1309	RGD13094	-0.0080851589234930	5.04705897864969	0.0174855523565896	0.894800041737614	0.980581837511607
Arf5	Arf5	-0.0063947124477056	8.09312454570741	0.0174326223880919	0.894955461937475	0.980581837511607
RGD1309	RGD13091	-0.0218921353727573	0.188826188701292	0.0174273068735196	0.894974204841752	0.980581837511607
Rtn4ip1	Rtn4ip1	0.00837141130096626	3.75835692451709	0.0173802235598401	0.89511553313161	0.980581837511607
Lmntd2	Lmntd2	0.0174313609827147	0.390859056762533	0.0173769644327263	0.895125370692043	0.980581837511607
Vps37c	Vps37c	0.00623631075922401	4.5942753080717	0.0173740588511606	0.895134028275497	0.980581837511607
Gnb2	Gnb2	0.00567003977246657	9.45701577907896	0.0173503403306252	0.895205219686886	0.980581837511607
Clic2	Clic2	0.0216661315446138	0.0931392205872761	0.0173414742867237	0.895231843679579	0.980581837511607
Tmem9	Tmem9	0.00524095762656834	6.09470046594827	0.0172708159254427	0.895444273608417	0.98064544343816
Ift43	Ift43	0.00982357251137701	2.74835254680399	0.0172698748603295	0.895447105641657	0.98064544343816
Lyrm1	Lyrm1	0.00636125490744564	4.27251097862223	0.0172037176775219	0.895646409668868	0.980777524489468
Cd99	Cd99	0.00657863398280986	5.26711961861941	0.0170805254315098	0.896018578071581	0.980972954428071
[illegible]	[illegible]	[illegible]	[illegible]	[illegible]	[illegible]	[illegible]

**Evi5**	Evi5	-0.00378126280922242	5.92872980943083	0.0151961288369735	0.901891218220486	0.982099991073685
**Gstk1**	Gstk1	-0.00739208964523970	3.44491261845406	0.0151692166182116	0.901977728261979	0.982099991073685
**Tfam**	Tfam	0.00499721760338717	5.25959047627635	0.0151573688277296	0.902015822387895	0.982099991073685
**Taf1a**	Taf1a	0.00468145012164702	4.35164750264708	0.0151360534589813	0.90208439563245	0.982099991073685
**Lgalsl**	Lgalsl	-0.00274249285720666	7.17762101518576	0.0151266278803206	0.902114734102384	0.982099991073685
**Ube2d3**	Ube2d3	0.00316401432752071	7.64703035779731	0.0151225834877309	0.902127754884628	0.982099991073685
**Armcx2**	Armcx2	-0.00350180474114	7.61701929603092	0.0150599002158657	0.902332685554333	0.982194922949417
**Yars2**	Yars2	-0.00531765747902249	3.90698799427365	0.0150400543820126	0.902393841535243	0.982194922949417
**Gpr84**	Gpr84	-0.0249523892542425	-0.462810784404732	0.0150223186281871	0.902451117792107	0.982194922949417
**Med24**	Med24	0.00611120865874248	6.68097385318585	0.0149520581405405	0.902678369419697	0.982235656478487
**Ripk3**	Ripk3	0.0221671848015267	-0.1965426226083	0.0149233030833074	0.902771534719904	0.982372268204526
**Mpv17l2**	Mpv17l2	-0.00870886237608861	4.36613046729623	0.0148870213109268	0.902889212285246	0.982414642162991
**Homer2**	Homer2	-0.00544516561739803	4.90051071810509	0.0148381919598606	0.903047817075408	0.982415526590563
**Prmt2**	Prmt2	-0.00359484745105021	7.10127943668804	0.0148210038873589	0.903071187324217	0.982415526590563
**Rock1**	Rock1	0.0152655234830831	6.80525705473107	0.0148052997548973	0.903154805043223	0.982415526590563
**Oxt**	Oxt	0.0690201610245257	1.57271216888263	0.0147898847199741	0.903204986794129	0.982415526590563
**Mrps27**	Mrps27	-0.00430749794564877	4.54145561019963	0.0147275241645781	0.903408232618729	0.982527393712547
**Clic3**	Clic3	0.0115424617831231	1.69554946324366	0.014670712644417	0.903593835847461	0.982527393712547
**Cpsf2**	Cpsf2	0.00296380212210036	7.30689081665977	0.0146398248840072	0.903694881422955	0.982527393712547
**Sla2**	Sla2	-0.0231439939792062	0.471497404370775	0.0146197616889623	0.903760573693477	0.982527393712547
**Zfp367**	Zfp367	0.00646906853822186	3.08717259203138	0.0145987528915299	0.903829411156944	0.982527393712547
**Myd88**	Myd88	-0.0120773036240866	1.74737552188968	0.0145924477156854	0.903850080510602	0.982527393712547
**Ldhd**	Ldhd	-0.0101674435078907	1.99260056643123	0.0145897105849144	0.90385905466411	0.982527393712547
**Rufy2**	Rufy2	0.0113719979156215	4.87289609625749	0.0145123891391112	0.904112946706225	0.982702857704861
**Ift80**	Ift80	0.00601405243289905	3.67247267773609	0.0144926033098471	0.904177992021464	0.982702857704861
**C1ql3**	C1ql3	-0.0134612225396348	4.07442247420521	0.0144674769241106	0.904260694110506	0.982707140529291
**Atpaf2**	Atpaf2	0.00481259882510041	4.39702498829168	0.0144245190511469	0.904402253617037	0.982775280454605
**Vps33a**	Vps33a	-0.00363114958741110	5.82001755613386	0.0143609917051251	0.904611989976711	0.982909273584845
**Hirip3**	Hirip3	0.00543216046306582	4.97422974693475	0.0142988523723962	0.904818262031422	0.982909273584845
**Fn3k**	Fn3k	-0.0128403704912 97	0.903409144123041	0.0142751182467188	0.904896248277474	0.982909273584845
**Bco2**	Bco2	-0.022182183870791	0.0851834599163266	0.014252541326897	0.904971130577215	0.982909273584845
**Suclg2**	Suclg2	0.00556418754381672	4.83935736778616	0.014249016937022	0.904982825037669	0.982909273584845
**Vipr1**	Vipr1	0.015787103726057	0.558140567322979	0.014225670628412	0.905260328702475	0.982909273584845
**Gulo**	Gulo	-0.0544426753346063	-1.25664385118873	0.0142206774677476	0.905276913066537	0.982909273584845
**Hgs**	Hgs	0.00295517556417653	7.38622210752596	0.0141787150686028	0.905216404502035	0.982955124347942
**Pole2**	Pole2	0.0123134646970033	1.23520394942584	0.0141605974410695	0.905276695930905	0.982955124347942
**MGC94207**	MGC94207	-0.0045812034254060	4.96261206246903	0.0141270588809004	0.905388407884948	0.98299087758271
**Ift57**	Ift57	-0.00446186642672962	5.93980559329773	0.0140471765896919	0.905652026740451	0.983194794104291
**Matr3**	Matr3	-0.0037037190302004	9.5088305882256	0.0139929582205235	0.905836423378294	0.98320283258801
**Sptb**	Sptb	-0.0083869639770882	5.70666723282165	0.0139813854790276	0.905875192111838	0.98320283258801
**Cnnm2**	Cnnm2	0.00535153564142967	4.65292888386034	0.0139674691391676	0.905921829432067	0.98320283258801
**Tomm34**	Tomm34	-0.0036146222790382	5.52902958755649	0.0139508259294985	0.905977636156274	0.98320283258801
**Adamts4**	Adamts4	-0.0139981882044032	1.48690486005753	0.0139084284801525	0.906119915817259	0.983271715920328
**Banf1**	Banf1	0.006459419596057	6.80622517082732	0.0138806273468219	0.906213392880736	0.983287533944117
**Taldo1**	Taldo1	0.00525265580085134	7.70348259768692	0.0138469138445865	0.906326923982581	0.983311360361879
**Ocrl**	Ocrl	0.00367852147208788	6.07082955597093	0.0138273046645913	0.906392879239743	0.983311360361879
**Thoc6**	Thoc6	-0.0055349456528952	4.27081240555079	0.0137946923767497	0.906502823423416	0.983345233703178
**Ston1**	Ston1	-0.0117261126541882	2.31562882460276	0.0136708584181303	0.906921508094126	0.983536501294726
**Thap4**	Thap4	0.00473093469873888	4.69377401112278	0.0136452392059923	0.907008368864541	0.983536501294726
**Ccdc59**	Ccdc59	-0.00575973504481	3.42847459888207	0.0136036780355369	0.90714645453312	0.983536501294726
**Drd1**	Drd1	0.0162235869409085	4.47247324175348	0.0135999647779795	0.907162128235894	0.983536501294726
**Bdkrb2**	Bdkrb2	-0.0286074402472042	-0.559722914981574	0.0135585217977905	0.907302990948747	0.983536501294726
**Tmub1**	Tmub1	-0.00487189081480079	5.3565492702967	0.0135254904810012	0.907415465410932	0.983536501294726
**Cask**	Cask	-0.0043270762046781	7.4785970058839	0.0135204767241927	0.907466534549076	0.983536501294726
**Mei4**	Mei4	0.0117542103507609	1.06883558671285	0.0134946281349784	0.907520680195471	0.983536501294726
**Capn1**	Capn1	-0.010367274025106	3.32231418125856	0.0134945572030176	0.907520922154497	0.983536501294726
**Sst**	Sst	-0.0116718408284785	6.87477629372944	0.0134852867560795	0.907552550518362	0.983536501294726
**Ccdc126**	Ccdc126	-0.0096261188355096	3.21255267569117	0.0134754321757242	0.907586183877816	0.983536501294726
**Pip5k1c**	Pip5k1c	-0.0029728348740058	8.8161265604238	0.0134640131033805	0.907625177289795	0.983536501294726
**Slc2a10**	Slc2a10	-0.0114164251423601	2.37700154171445	0.0133490704212303	0.908021999343187	0.983812716475653
**Idh1**	Idh1	0.00722719666454988	8.40289945258914	0.0133229426664697	0.908108566248136	0.983812716475653
**Wdr43**	Wdr43	0.00319697852460145	5.77938371218443	0.0132970223817921	0.908197259994894	0.983812716475653
**Nup160**	Nup160	0.00502118205556787	5.20452627496385	0.0132849744243515	0.908238675083537	0.983812716475653
**R3hdm2**	R3hdm2	0.00367353128021647	7.70044542985463	0.0132290968824768	0.908430315418255	0.983812716475653
**Rab11b**	Rab11b	0.00356198552064583	8.523697454129	0.0132074617489764	0.908505582678151	0.983812716475653
**Snx17**	Snx17	0.0044006596873635	6.70436719320374	0.0131898239859191	0.908566392890988	0.983812716475653
**Skor1**	Skor1	0.0516985371924297	0.527307158399221	0.0131688189054273	0.908638942102432	0.983812716475653
**Rab4b**	Rab4b	0.00529185725782996	5.70983349086525	0.0131553132921027	0.908685597753209	0.983812716475653
**Bend6**	Bend6	-0.0049467498051309	7.68148850010098	0.0131436001011309	0.908726081064496	0.983812716475653
**Cep95**	Cep95	-0.0140035247032267	4.98871249225872	0.0130987191812437	0.908881368616039	0.983812716475653
**Ints4**	Ints4	-0.00463220597291246	5.27405069045192	0.0130841430254378	0.908931858939482	0.983812716475653
**Trmt1**	Trmt1	-0.0050536898904413	6.58902209296817	0.0130724681482494	0.908968855438831	0.983812716475653
**Mtfr1l**	Mtfr1l	-0.0033048028877461	6.97988150048923	0.013056357243272	0.909028187960032	0.983812716475653
**Pitpnm2**	Pitpnm2	-0.0048239923116 87	7.69210462252872	0.0130170483263612	0.909164642032523	0.983812716475653
**Agbl5**	Agbl5	0.0042064415435515	5.54904507817491	0.0130137154480465	0.90917622113273	0.983812716475653
**Net1**	Net1	-0.00569866542790581	5.06797323526773	0.012989204156027	0.909261063558325	0.983812716475653
**Arpc2**	Arpc2	0.00327165989001427	8.24951753416873	0.0129762953960031	0.909299370553597	0.983812716475653
**Kcnc1**	Kcnc1	-0.0070203330074694	5.39989788465769	0.012938790663199	0.909436922684285	0.983876223387936
**Tfb2m**	Tfb2m	-0.0049476529522674	4.47168592196373	0.0128076805499049	0.909894967985155	0.98402135600562
**Rag1**	Rag1	0.00435772849842835	6.01590341874744	0.0128076233847565	0.909895168213436	0.98402135600562
**Dnph1**	Dnph1	0.00883708768842636	2.97150277094577	0.0127730672664228	0.910016288417855	0.98402135600562
**Slc43a1**	Slc43a1	-0.0161611186800598	0.223811140653532	0.0127593209616745	0.910202994210433	0.98402135600562
**Hpcal4**	Hpcal4	0.00551385408999233	5.42983749818601	0.0127632528286057	0.910050367653267	0.98402135600562
**Htr5a**	Htr5a	-0.0108247255094938	2.38941114256702	0.012699771621433	0.910273742305275	0.98402135600562
**Tgfbrap1**	Tgfbrap1	0.00340899064231321	5.98499567769607	0.0126695694194296	0.910380047485283	0.98402135600562
**Bpnt2**	Bpnt2	-0.0033138940403354	8.21118589988434	0.0126508620580494	0.910410719346398	0.98402135600562
**Faap24**	Faap24	0.00885342499664033	2.09872096235871	0.0126465520739887	0.910461149771506	0.98402135600562
**Specc1l**	Specc1l	0.00358317506042043	6.32887083387715	0.0126350122908576	0.910501838661052	0.98402135600562
**Tbcc**	Tbcc	0.00634602139436206	4.66249403432301	0.0126268254942516	0.910530716642818	0.98402135600562
**Dusp8**	Dusp8	-0.0037259210701097	7.61118149472882	0.0125778941849201	0.910704219787768	0.98402135600562
**Ndufaf2**	Ndufaf2	-0.0089571978971785	2.84112005270825	0.0125694269232238	0.910736984834123	0.98402135600562
**Map3k6**	Map3k6	-0.0156406027577127	0.879512947563102	0.0125151278794533	0.910925665227887	0.98402135600562
**Dele1**	Dele1	-0.0040380217093445	5.4701349815078	0.0124896568354416	0.911015977225147	0.98402135600562
**Nus1**	Nus1	-0.002798295659095	6.70246990548478	0.012480415234749	0.911048767969999	0.98402135600562
**Rnpc3**	Rnpc3	-0.0093471190487117	4.06255622904055	0.0124456807900205	0.911168567871648	0.98402135600562
**Tecpr1**	Tecpr1	0.00328167415737054	6.79829414487716	0.012440690816554	0.911189857022393	0.98402135600562
**Nsrp1**	Nsrp1	0.00463944472900316	4.2436093827292	0.0124330512818389	0.911217016480566	0.98402135600562
**Chrne**	Chrne	-0.0138914768515128	1.84454218276489	0.012424080416426	0.911249097570172	0.98402135600562
**Puf60**	Puf60	-0.0033497055353739	7.52498062081237	0.0124178949069371	0.911271535736833	0.98402135600562
**Fhit**	Fhit	0.00951063534206342	2.10627411483005	0.0123815520184181	0.911400322202009	0.98402135600562
**Tada3**	Tada3	-0.0033771257569953	6.09513766343961	0.012375210169921	0.911422923079049	0.98402135600562
**Timm44**	Timm44	0.00526812527551133	5.16039554815598	0.0123522678751158	0.911504729483043	0.98402135600562
**Cant**	Cant	-0.0063105281561544	4.87192415368927	0.0123232700202223	0.911608238595482	0.98402135600562
**Gcdh**	Gcdh	-0.0052771074383923	5.40818484773137	0.0122864706967789	0.911739773051394	0.98402135600562
**Hdhd3**	Hdhd3	-0.0093783597955947	1.72519006202296	0.0122750964854177	0.911780469039187	0.98402135600562
**Pdgfd**	Pdgfd	-0.0157100557574119	0.665582034858938	0.0122523653587169	0.911858274215464	0.98402135600562
**Oasl2**	Oasl2	-0.0162047731274978	0.112007321752462	0.0122452971830072	0.911887179072361	0.98402135600562
**Samd12**	Samd12	0.00700826814741811	3.22041412645256	0.0122244710096382	0.911961834757204	0.98402135600562
**Kctd1**	Kctd1	0.0052973283565067	5.26369711499576	0.0122093860601069	0.912015950055807	0.98402135600562
**Naa30**	Naa30	-0.0031483467560932	6.30909669162411	0.0121613729353243	0.912188418535427	0.984104653204673
**Trim34**	Trim34	0.0173230636368204	0.654384674246137	0.0121408860031499	0.912262823640007	0.984104653204673
**Kcnh8**	Kcnh8	0.00941305537233044	2.73325173660883	0.0121220931571031	0.912329768663817	0.984104653204673
**Exosc2**	Exosc2	0.00424298675758368	4.91217867160781	0.0120998697397593	0.912409991658603	0.984106027770046
**Rsph4a**	Rsph4a	0.0213800878805777	1.97267823478836	0.0120718879176221	0.912510414117361	0.984129348858355
**Elmod3**	Elmod3	-0.0064887830528419	2.95370915817933	0.0120443469788327	0.912610230970458	0.984143564905623
**Ppp4r3b**	Ppp4r3b	0.00337451401382845	6.44025342056307	0.0120246728222639	0.912681348884371	0.984143564905623
**Samp**	Samp	-0.0050110281364655	5.82614343571802	0.0119898624636363	0.91280732525655	0.984148684141372
**Dipk1b**	Dipk1b	-0.0050286493256878	6.90780305402942	0.0119797792209511	0.912843850610202	0.984148684141372
**Scx**	Scx	-0.0129172943935163	1.00193790547195	0.011941382017099	0.91298308157866	0.984199912254746
**Rxfp2**	Rxfp2	-0.0218751963825438	-0.206123643521993	0.0119233892144427	0.913049129145522	0.984199912254746
**Dbr1**	Dbr1	-0.0052277882225704	3.97854021654522	0.0118911570733218	0.913165543407638	0.984240366715368
**Stx19**	Stx19	-0.0275658058454235	0.0331265366503184	0.0118502113489717	0.913314583539385	0.984315976403291
**Dpysl4**	Dpysl4	0.00349355299265059	7.4310963082248	0.0117540200895743	0.913665742653081	0.984609385967797
**Ogg1**	Ogg1	0.00586820290431563	3.29988316296805	0.0117076018054284	0.913835498816856	0.984707273403295
**Mrpl19**	Mrpl19	0.00420554057020635	4.47116478869293	0.0115951211745448	0.914248988936735	0.985067755613859
**Sar1a**	Sar1a	-0.0025320512218524	7.5730238733155	0.0115517743002371	0.914408840973766	0.985147171596317
**Lix1**	Lix1	0.00807192003709962	3.86308723796785	0.011514673581928	0.914545870057978	0.985147171596317
**Rbm25**	Rbm25	-0.0059146579951564	7.32921684554927	0.0115034460769076	0.914587404304414	0.985147171596317
**Irf4**	Irf4	0.0201827915588263	-0.417715977805785	0.011489622278944	0.914638521741744	0.985147171596317
**Ccdc61**	Ccdc61	0.0063941066304588	3.34409975052018	0.0114551491011596	0.914765187209842	0.985199632088339
**Rpp40**	Rpp40	0.00794892224147846	2.25499258393776	0.0113826281837373	0.915031636604941	0.985285791954495
**Zfp579**	Zfp579	-0.0033435788005296	5.75630537870383	0.011378679869672	0.915050083856859	0.985285791954495
**Zfp133**	Zfp133	-0.0050238075969077	3.68366438096043	0.0113437529778153	0.915180057375585	0.985285791954495
**Ctbs**	Ctbs	-0.0066274890351317	2.70716463483188	0.0113243712474329	0.915252275914733	0.985285791954495
**LOC680254**	LOC680254	-0.0081913214413095	2.83579591023212	0.0113240835935713	0.915253348216343	0.985285791954495
**Csde1**	Csde1	0.00346573243252596	8.37146362942834	0.0112745040653621	0.915438374513359	0.985285791954495
**Avpr2**	Avpr2	0.0198202255110546	1.13034074064287	0.0112605638714112	0.915490098279119	0.985285791954495
**Lrrc45**	Lrrc45	0.00684899941581118	5.86570412690547	0.0112300522662387	0.915603493640291	0.985285791954495
**Crnde**	Crnde	-0.0268921998348379	-0.584377287483586	0.0112254636482731	0.915621794065328	0.985285791954495
**Meis3**	Meis3	-0.0048852895983471	6.07847178828047	0.0112178254096591	0.915650398824159	0.985285791954495
**Wdr74**	Wdr74	0.00460409752587924	4.87849259834384	0.0112006278415322	0.915714826783708	0.985285791954495
**Mmel1**	Mmel1	-0.0125408142688922	0.51654072594328	0.0110814532347163	0.916162771965256	0.985600312049678
**Rexo1**	Rexo1	-0.0035096378732043	7.08465287905914	0.0110808256149255	0.916165137384962	0.985600312049678
**Ankrd37**	Ankrd37	-0.0162389346795106	-0.004990447636080	0.0110590531544794	0.916246105219718	0.985603245970896
**Dohh**	Dohh	-0.0048913767099355	6.26545136650438	0.0110355947772894	0.916335823168844	0.985613971179039
**Adk**	Adk	-0.0049285546391986	5.50331492085973	0.0110096515807436	0.916433786205322	0.985634372186148
**Eif2s1**	Eif2s1	-0.0034805404935150	6.15645870885127	0.0109844082010042	0.916529330464474	0.985652168200019
**Sez6l**	Sez6l	-0.0045164353932680	7.93321321886102	0.0109438465466329	0.916683025943353	0.985732492863502
**Sntg1**	Sntg1	0.00682946027548166	3.09635915325734	0.0108885142147983	0.916893152658802	0.985748513424027
**Katnip**	Katnip	-0.0053889067764280	4.95971559096716	0.0108776228701544	0.916934576717652	0.985748513424027
**Rcbtb2**	Rcbtb2	0.00392864621355174	5.60699477571922	0.0108775029197587	0.916935033052758	0.985748513424027
**Plppr5**	Plppr5	0.00321392461975825	6.24140244661675	0.0108328868728265	0.917104945578977	0.985765424016541
**Stk19**	Stk19	0.00564417656680893	3.56377155283946	0.0108245508023757	0.91713672125738	0.985765424016541
**Tmem218**	Tmem218	-0.0058144697897354	3.82368107008395	0.0108111932122199	0.917187689942136	0.985765424016541
**Lrrc9**	Lrrc9	-0.0086418610424258	2.0244565358553	0.0107807223351486	0.917304054367381	0.985805571605669
**Nosip**	Nosip	-0.0041546994014776	4.84910666908793	0.0107430601802534	0.917448111306807	0.985875470224359
**Pwwp3b**	Pwwp3b	-0.0099341538459556	1.72776297541793	0.010670260922474	0.917727292211701	0.986010041770299
**Cd2ap**	Cd2ap	-0.0090040298919327	3.44401749828497	0.0106653684880484	0.917746088879403	0.986010041770299
**Mbp**	Mbp	0.00883066506838701	5.72613796716103	0.0106408754242662	0.917839871791344	0.986010041770299
**Mat2b**	Mat2b	0.00436550568552418	5.08835716646213	0.0106066504734059	0.917972023564056	0.986010041770299
**Marcks**	Marcks	-0.0035296705869267	9.72809458071319	0.0105712479782554	0.918108555485228	0.986010041770299
**Gstt1**	Gstt1	-0.0116959790172035	1.09606830140804	0.0105691721163197	0.918116563047745	0.986010041770299
**Znrf2**	Znrf2	0.00375128749322259	6.84162924425113	0.0105665912340922	0.918126526030884	0.986010041770299
**Cth**	Cth	0.00750602113610252	2.26490904467218	0.010506121608131	0.918360309950179	0.986136895042081
**Gnao1**	Gnao1	0.00352298680921277	9.52189013856617	0.0104831478222849	0.918449307931376	0.986136895042081
**Rbm42**	Rbm42	0.00421331225895256	6.43651467156412	0.0104687985132585	0.918504945617835	0.986136895042081
**Cadm2**	Cadm2	-0.0048635758708420	5.89945812606988	0.0104544055234612	0.918560791390484	0.986136895042081
**Asah2**	Asah2	0.0130650582797853	1.54854262405948	0.0103971518053498	0.91878322518556	0.986290925942958
**Hemk1**	Hemk1	-0.0054572498488249	3.34642988024136	0.0103594297737502	0.918930281690878	0.986348274595222
**Oprm1**	Oprm1	-0.0146656950301696	2.13400094273672	0.0103151726318345	0.919103042559392	0.986348274595222
**Ppp2r5e**	Ppp2r5e	0.00208385352278426	7.3109705798871	0.0103000625818627	0.919159806728907	0.986348274595222
**Sgcd**	Sgcd	0.0133285664559648	1.01378149404898	0.0102873117023705	0.919211991769458	0.986348274595222
**Stx4**	Stx4	0.00451442392820623	4.53945675475757	0.010251229884827	0.919353310188483	0.986348274595222
**Rbm45**	Rbm45	0.00376051458281913	4.81122886587185	0.0101811847614997	0.919628368900001	0.986348274595222
**Polr3gl**	Polr3gl	0.0050385140749657	3.74007855900786	0.0101633346409287	0.919698516851462	0.986348274595222
**LOC100912**	LOC100912	0.00561915668103835	4.46042648112479	0.0101630793014209	0.919699622174848	0.986348274595222
**Lrp12**	Lrp12	-0.00326536856135538	6.53517974478826	0.0101326160499084	0.919819653858581	0.986348274595222
**Tor3a**	Tor3a	0.0178052870236097	2.90884467566005	0.0101196290844712	0.919870880669907	0.986348274595222
**LOC498276**	LOC498276	-0.013828186898776	0.980536125961988	0.0100991955572454	0.919951547553003	0.986348274595222
**Fech**	Fech	0.00442885853489833	4.74230753788073	0.0100695263534685	0.91996801673643	0.986348274595222
**Sumf1**	Sumf1	-0.0050734561935599	3.92917060624365	0.0100521388854495	0.920094092350187	0.986348274595222
**Espl1**	Espl1	0.00669162900953082	3.47233184270401	0.0100597252881913	0.920107600826444	0.986348274595222
**Myl12b**	Myl12b	0.0033669296413752	8.00609077853519	0.0100422556334721	0.92017676922199	0.986348274595222
**Dimt1**	Dimt1	0.00460988674760375	4.52096442190403	0.0100328823151099	0.920209928642096	0.986348274595222
**Tars3**	Tars3	-0.0040112758697286	4.9232717550681	0.0100182114889762	0.920272067688668	0.986348274595222
**Dyrk2**	Dyrk2	0.00501378692429076	6.57940386013172	0.0100149600884533	0.920285042739667	0.986348274595222
**Srsf2**	Srsf2	0.00317893076278267	8.45162472735168	0.00996707598713176	0.920475126941576	0.986348274595222
**Il34**	Il34	0.0117411854957374	3.00179286768393	0.00996275563404683	0.920488236286509	0.986348274595222
**Spr**	Spr	0.00644061517728173	3.87278119218399	0.00996161114645133	0.920496858971448	0.986348274595222
**Yod1**	Yod1	0.00671108590069884	2.57244737594868	0.00989502561599582	0.920762174163609	0.986547844314242
**Ftl1**	Ftl1	0.00393529660003845	9.94728881738138	0.00977389982033827	0.921247018347563	0.986609285221667
**Cdc42se2**	Cdc42se2	0.00306789821918108	7.24698732412391	0.00977155238691552	0.921256445344507	0.986609285221667
**Rfk**	Rfk	0.00648242429436998	5.10426804884157	0.00976280536525331	0.921292385944621	0.986609285221667
**Kcnj6**	Kcnj6	-0.0068308626875436	6.27641036299705	0.00975517844288731	0.921322233869173	0.986609285221667
**Psme1**	Psme1	-0.0037960775565301	5.9598959476096	0.00974089595853986	0.921379501929232	0.986609285221667
**Nacc1**	Nacc1	0.00297204364217846	6.42943195097319	0.00972970644154714	0.921424685652011	0.986609285221667
**Supt20h**	Supt20h	-0.0069427351036472	5.75814323962783	0.00972318863266253	0.921450923154834	0.986609285221667
**Egln2**	Egln2	0.00273393780670486	6.86727921010031	0.00969740708657696	0.921554794146817	0.98671922823059
**U2af1l4**	U2af1l4	-0.0043065970259250	4.68977381931119	0.00961685000360291	0.921880251226506	0.986834978660571
**Msl3**	Msl3	0.00320139117258764	5.39070758653584	0.00960607442620187	0.921923888624595	0.986834978660571
**Chd5**	Chd5	-0.0059277274896072	8.42048815328852	0.00960155187890165	0.921942212173689	0.986834978660571
**Dars2**	Dars2	0.00469164334659361	3.61004863899494	0.00958646629858474	0.922003361087379	0.986834978660571
**Plin5**	Plin5	0.0195103149780599	-0.501421132450589	0.00953710697592669	0.922203777729341	0.986834978660571
**Chrm1**	Chrm1	-0.0070564043159209	4.81717338962799	0.00951196674111543	0.922306057402228	0.986834978660571
**Camk2g**	Camk2g	-0.0035070795096614	5.54434474886915	0.00950231754863498	0.922341277022351	0.986834978660571
**Stambp**	Stambp	-0.0035171214959990	4.91897746199145	0.00949652718635008	0.922368838801427	0.986834978660571
**Klf16**	Klf16	0.00469912911255585	5.93345594073974	0.00949510559013333	0.922374731196568	0.986834978660571
**Prkrir**	Prkrir	0.002939918900775	6.17462518802193	0.00942578747241958	0.922616818501062	0.986885508224234
**Tusc2**	Tusc2	0.00385895744726812	6.25019854630324	0.00942098470832775	0.922677350761252	0.986885508224234
**Rom1**	Rom1	0.00977528594289442	2.28706975597783	0.00941295254698105	0.922710216289376	0.986885508224234
**Foxo1**	Foxo1	-0.0081780168947989	4.50728240898271	0.00940808067074322	0.922730345671549	0.986885508224234
**Npr2**	Npr2	0.00582143309605486	5.28707099619458	0.00934870362758033	0.92297261831228	0.987007998182851
**Selenoh**	Selenoh	-0.0061257035053316	5.45242547068952	0.00933984507698564	0.923010805674241	0.987007998182851
**Dmap1**	Dmap1	-0.0037931912099800	5.24758482221383	0.00926479011874183	0.923319018979421	0.987192936336697
**Kctd4**	Kctd4	-0.0061968224425402	4.7104359393096	0.00925915385790985	0.923342275153217	0.987192936336697
**Ccne1**	Ccne1	-0.0074384844040379	3.58380243886275	0.00923931814068624	0.923424177556445	0.987193906808222
**Srxn1**	Srxn1	-0.0034981901635456	4.93298769254128	0.00916332642665196	0.92373877418261	0.987403737889402
**Cebpz**	Cebpz	0.00274592695625416	6.32979214318451	0.00914345973924924	0.923821236768709	0.987407378889402
**Ptms**	Ptms	-0.0044620274444542	10.4333371668745	0.00912022231865078	0.923917763787422	0.987407378889402
**Fcf1**	Fcf1	-0.0041779287329376	4.68187756801803	0.00911046457478903	0.92395839299331	0.987407378889402
**Dscaml1**	Dscaml1	0.00573744653598297	6.08896561057831	0.00909622135799282	0.924017577466591	0.987407378889402
**Syndig1**	Syndig1	-0.0050431695735649	4.92916936248408	0.0090727408454736	0.924115512718258	0.987427357385091
**Lratd1**	Lratd1	-0.0041141854465337	6.69631084388522	0.00900856313244889	0.924383572599216	0.987629203230041
**Lsamp**	Lsamp	-0.0039830276961137	6.12700547568801	0.00894263126035355	0.924659964627717	0.987797232071472
**Tbpl1**	Tbpl1	0.0031606470032668	5.75504857429106	0.00891722382238752	0.924766707008527	0.987797232071472
**Mcf2l**	Mcf2l	0.00381250613340959	8.86865247977214	0.00888175086694129	0.924916094747068	0.987797232071472
**Mlip**	Mlip	-0.0083763235712185	3.84101531276196	0.00887780823710256	0.924932712287734	0.987797232071472
**Smoc1**	Smoc1	0.00510213124497476	4.60023226288985	0.00883009826218739	0.925134097893951	0.987797232071472
**Pecr**	Pecr	-0.0054060733097345	3.62058006489568	0.00882438765678078	0.925158239270685	0.987797232071472
**Csgalnact2**	Csgalnact2	-0.0054615958220633	4.09605185834993	0.00882414055132585	0.925159284126502	0.987797232071472
**Ncoa4**	Ncoa4	0.00221738520157948	7.25334216526003	0.00880060213563638	0.925258876805434	0.987797232071472
**Sypl1**	Sypl1	0.00453469955187044	4.57094172920035	0.0087794404006134	0.925348528297075	0.987797232071472
**U2af1**	U2af1	0.00378213416665558	6.15237487466086	0.00876743047020057	0.925399458807555	0.987797232071472
**Clvs1**	Clvs1	0.00328964921459932	6.23434487673345	0.00876454329812759	0.925411705192229	0.987797232071472
**Cpa2**	Cpa2	0.0098642158563884	1.429605110019	0.00864014579836692	0.92594138897903	0.988051375700538
**Hadh**	Hadh	-0.0055261224292886	4.23364906123014	0.00860387634870108	0.926096547181348	0.988051375700538
**Mpp2**	Mpp2	0.00563943049815535	5.62521800762788	0.00858132892745627	0.926193126920973	0.988051375700538
**Junb**	Junb	-0.0076171711858770	3.88909997304575	0.00856932942720534	0.926248934841569	0.988051375700538
**Atp2b4**	Atp2b4	-0.0055449462019589	6.36346681186786	0.00856253630294497	0.926273796718752	0.988051375700538
**Oaz2**	Oaz2	-0.0027689968824056	7.82624598426281	0.00855996630824762	0.926284823268073	0.988051375700538
**Crbn**	Crbn	-0.0031970818274207	5.22325472989085	0.00854022939794554	0.926369580868826	0.988051375700538
**Abcg3l1**	Abcg3l1	0.0122915787908928	0.475423610516859	0.00852446428287124	0.926437422376543	0.988051375700538
**Thnsl2**	Thnsl2	-0.0108537592668193	1.51822359463041	0.00852226095004188	0.926446865927081	0.988051375700538
**Golga4**	Golga4	0.00567181809339373	6.52827373777575	0.0085087648632225	0.926505001235497	0.988051375700538
**Jkamp**	Jkamp	0.00257090902016474	5.44648463826935	0.00849874480773138	0.926548165890992	0.988051375700538
**Timm22**	Timm22	0.00515677352481673	4.65610454389796	0.00845438912280372	0.926739551407097	0.988051375700538
**Pgam5**	Pgam5	0.00295823329018426	5.96829278725576	0.00838966718828839	0.927019723708167	0.988051375700538
**Fam219b**	Fam219b	0.00432687315394922	4.32647947396478	0.00838861531794952	0.927024287393416	0.988051375700538
**Sfxn4**	Sfxn4	0.0065814144699686	2.71381715425192	0.00838811975101085	0.927026435664937	0.988051375700538
**Lrrc43**	Lrrc43	0.00964920680467013	1.27467738014458	0.00835388677792537	0.927175080018551	0.988051375700538
**Hnrnpd**	Hnrnpd	-0.0032509779440973	8.14147526099024	0.00834600093107677	0.927209328305229	0.988051375700538
**Dclre1b**	Dclre1b	-0.0033896054522851	4.26404551052303	0.00833130498038059	0.927273301504546	0.988051375700538
**Ankzf1**	Ankzf1	-0.0060591177633745	5.26425644651141	0.00827761411390782	0.927507374908928	0.988051375700538
**Ap1s1**	Ap1s1	-0.0046126549705496	7.10200977577896	0.00826269703857246	0.927572543929614	0.988051375700538
**Adcy3**	Adcy3	-0.0025886309303389	6.1555956300158	0.00825124318027548	0.927622623253376	0.988051375700538
**Nvl**	Nvl	-0.0066840068971343	4.18041891517075	0.00822805875236278	0.927679874779868	0.988051375700538
**Gnb5**	Gnb5	0.00319706632540877	5.85257066700295	0.00822846171377734	0.927722234206758	0.988051375700538
**Pced1b**	Pced1b	-0.0049282087337835	3.14050988721402	0.00820019031294085	0.927845267465612	0.988051375700538
**Atpsckmt**	Atpsckmt	-0.0041111423729835	4.20170503312415	0.00819087914268858	0.92788635787642	0.988051375700538
**B4galnt1**	B4galnt1	0.00250940910509663	6.99352660805593	0.00816998855401607	0.927978900018746	0.988051375700538
**Sgip1**	Sgip1	-0.0044321265797617	7.93948755886544	0.00815769743766737	0.928032949232884	0.988051375700538
**Bub1b**	Bub1b	0.0073433884779412	3.46879937288791	0.00815502785793688	0.928044791701566	0.988051375700538
[illegible]	[illegible]	[illegible]	[illegible]	[illegible]	[illegible]	[illegible]

Kcnj9	Kcnj9	-0.0040195836164250	4.43719980975089	0.00675529288971433	0.93449515716237	0.98867271856714
Trip4	Trip4	-0.0036965903517512	3.98331298458504	0.00674352508847947	0.934552060430398	0.98867271856714
Nrbp2	Nrbp2	-0.0073595526889321	7.72701519868446	0.00672629667141678	0.934635578266674	0.988677245645807
Nudt18	Nudt18	0.00351272215167526	5.80799266991801	0.0066888280519149	0.934816994867481	0.988749832103921
Rsl24d1	Rsl24d1	0.00293568901052606	6.10580179846052	0.00667518032475266	0.934883865440003	0.988749832103921
Rad51c	Rad51c	-0.0092697119385925	1.08998005772118	0.00666325338982965	0.934941935739557	0.988749832103921
Ccdc130	Ccdc130	-0.0040046499445379	4.1561526979564	0.00662889630777386	0.935109507433141	0.988797245285256
Csrp1	Csrp1	-0.0065412877018740	4.19970239664232	0.00659889912617239	0.935257151207481	0.988797245285256
Scn1a	Scn1a	0.0060052259230805	6.04626400650686	0.00659415508201278	0.935279397919649	0.988797245285256
Zfp512b	Zfp512b	-0.00465147523075467	7.8999806026788	0.00655347487139897	0.935429804203888	0.988797245285256
Snx2	Snx2	-0.0027212849517650	5.88051230941785	0.006544882100678	0.935520634064957	0.988797245285256
Efhb	Efhb	-0.0187905241250103	0.132762061292182	0.00653912442472742	0.935549382590878	0.988797245285256
Pef1	Pef1	0.00367498903619253	5.55032097371716	0.00652950440637312	0.935596754198217	0.988797245285256
Rrp7a	Rrp7a	-0.0039862649702525	4.00352305095716	0.00650322039876272	0.935726228699575	0.988797245285256
Sertm1	Sertm1	0.0066130552445032	6.81182251252994	0.00647412258254576	0.93586987149615	0.988797245285256
Cd302	Cd302	-0.0121142293869377	0.882728810908107	0.00646638977744374	0.935908099479705	0.988797245285256
Cdk20	Cdk20	0.00311406523852381	4.83439171954987	0.00646559445981154	0.935912032525644	0.988797245285256
RGD1562	RGD15626(	0.00480025823309214	3.80314078842977	0.00646039262120723	0.935937762933235	0.988797245285256
Ptpra	Ptpra	0.00203103813871892	7.84044754111345	0.00640352134284055	0.936219704645712	0.988899029436191
Fosl1	Fosl1	0.00371828247196313	3.67380522199384	0.00638842140840351	0.936294787839619	0.988899029436191
Helq	Helq	0.00371017382594231	3.82984585242865	0.0063744139451014	0.936364569001341	0.988899029436191
Btbd3	Btbd3	-0.0054977807400006	6.00672863084843	0.00631642058647571	0.936654046361518	0.989218931903501
Whrn	Whrn	0.00348895092481103	5.23433347627168	0.00629973491226921	0.936737538260672	0.989223488262895
Dclre1a	Dclre1a	-0.0040037445039342	4.00497875890257	0.00624627715582449	0.937006062620959	0.989423209488973
Avpr1a	Avpr1a	0.0108290678821174	1.50393866484812	0.00622757589404087	0.937100238891755	0.989438917925821
Mmgt1	Mmgt1	-0.0029497808817440	6.24758201391584	0.00619881607865935	0.937245346248046	0.989475980325195
Umps	Umps	0.00269852824879906	5.10652862371081	0.00618919801257789	0.937293946784682	0.989475980325195
Rasl11a	Rasl11a	-0.0081074628131920	1.00740581808332	0.00609188013808318	0.937787869101291	0.989650026219813
Cirp	Cirp	0.00308019242311871	4.96519720110736	0.00608226395310854	0.93783180629187	0.989650026219813
Zfp110	Zfp110	0.00442787556267154	4.92802532995438	0.00605555721833186	0.937973256790444	0.989650026219813
Nox4	Nox4	-0.0118323893688961	0.843956831575179	0.00603644490294694	0.938071020241925	0.989650026219813
Trmt11	Trmt11	0.00656558544634646	1.81927587488389	0.00603546683920797	0.938076027424886	0.989650026219813
Gga1	Gga1	-0.0021237556580075	6.28846077552948	0.00598776123026568	0.938320753003637	0.989650026219813
Acyp1	Acyp1	0.00665879104714715	3.40593572242913	0.00595261613050013	0.938501672325894	0.989650026219813
Cby1	Cby1	0.00420668197284874	4.74373343494217	0.00592675615395422	0.938635127179801	0.989650026219813
Fam104a	Fam104a	-0.0024782528773239	5.53068509434476	0.00587554745935282	0.938900295232515	0.989650026219813
Slc38a7	Slc38a7	0.00275350297683287	5.3704867984259	0.00586682708545389	0.938945045632986	0.989650026219813
Thap3	Thap3	0.00396782724478044	4.25676366983606	0.00585428787954256	0.939010718553404	0.989650026219813
Zfp608	Zfp608	-0.0038674031205527	6.09219888759385	0.00584913982370949	0.939037488105261	0.989650026219813
Rhebl1	Rhebl1	0.00616146362996921	3.88881462670959	0.00584735463462493	0.939046773737883	0.989650026219813
Zfp879	Zfp879	0.00555328296118376	2.54423201098468	0.00584066970768049	0.939081557950909	0.989650026219813
Pdcd2l	Pdcd2l	-0.0044534702758896	4.08436753625563	0.00582567842700144	0.939159636146018	0.989650026219813
Mfap3l	Mfap3l	0.00438440881903575	3.98004459062881	0.00582092580270282	0.939184357908774	0.989650026219813
Ubfd1	Ubfd1	0.00280755769674453	5.56148711201896	0.00581345419129065	0.939222377726573	0.989650026219813
Bcap29	Bcap29	-0.00368986863961972	4.23332320120819	0.00580928538905032	0.939245132343759	0.989650026219813
Fkbp4	Fkbp4	-0.00243898484940955	7.58545012685854	0.00580400533657985	0.939272693669032	0.989650026219813
Gpx7	Gpx7	0.00550945886155025	3.54831606496996	0.00580193386160488	0.939283510611301	0.989650026219813
Gpx8	Gpx8	-0.0046343874998952	3.94423795741439	0.00580014410101626	0.939292858045919	0.989650026219813
Spsb3	Spsb3	-0.0022702909059887	6.05823406047314	0.00579417129839754	0.939324062877947	0.989650026219813
Acsl5	Acsl5	0.00257109414157312	6.01355435340614	0.00578741142933836	0.939359396249649	0.989650026219813
Mrps14	Mrps14	-0.0039759436690700	4.78835437098165	0.00578573754987133	0.939368153451354	0.989650026219813
Gria2	Gria2	-0.0049385493321603	8.75217589660469	0.00571806411472954	0.939723214440206	0.98994851693435
LOC1009	LOC100911	0.0235139460372095	9.07525592495559	0.00568159347052278	0.939915279164378	0.989981341707171
Slc18a3	Slc18a3	0.0150681375740799	1.97597930536507	0.00564711898431389	0.940097502123289	0.989981341707171
Rgl3	Rgl3	-0.0126032848836407	1.57634788992188	0.00562505395672019	0.940214425692751	0.989981341707171
Cdh15	Cdh15	-0.0059857289905791	2.32049487687479	0.00558142914016457	0.940446276228885	0.989981341707171
Nucb2	Nucb2	-0.0028215338960882	5.56967477517254	0.00557902604259652	0.940459074278373	0.989981341707171
Gak	Gak	0.00161960361876287	6.79697360016365	0.00555658525182423	0.940578720055943	0.989981341707171
Plekha3	Plekha3	0.00284691807016475	4.54536546469472	0.00555641658311146	0.940579620252447	0.989981341707171
Arcn1	Arcn1	0.00169928990992916	7.87516120537973	0.00553674502670987	0.940684703107212	0.989981341707171
Ctr9	Ctr9	0.00192161491317871	7.3086257510127	0.00553015267050228	0.940719960583939	0.989981341707171
Nrip1	Nrip1	-0.0034541044313524	6.42118579143734	0.0055209739042823	0.940769086003496	0.989981341707171
Cadm1	Cadm1	-0.0034328414203129	7.15512883089237	0.00549335391264094	0.940917158282913	0.989981341707171
Rab33a	Rab33a	-0.0036768148874925	5.1830270239536	0.00547581242358319	0.941011393751327	0.989981341707171
Ttc14	Ttc14	0.00807738683435022	4.60438026202308	0.00546689228214348	0.941059372223218	0.989981341707171
Chmp1a	Chmp1a	-0.00252321032164972	7.0480082167896	0.00546439990612413	0.941072784917602	0.989981341707171
Adamts1(	Adamts10	0.00496087247166576	4.82960488478296	0.00540938197055141	0.941369655489518	0.989981341707171
Lsm1	Lsm1	-0.0031768879626161	4.16487865110691	0.0054073423176817	0.941380685151531	0.989981341707171
Esr1	Esr1	-0.0076128307706486	1.51989569095791	0.00537628292248371	0.941548977859857	0.989981341707171
Osgepl1	Osgepl1	-0.0047173291988270	3.68593577896503	0.00533664524485289	0.941764462725396	0.989981341707171
Ube2m	Ube2m	0.00270604433372545	7.71510163822041	0.00531254209327989	0.941895889763399	0.989981341707171
Nub1	Nub1	-0.0016123024835425	6.56725743817963	0.00529829660757031	0.941973708947202	0.989981341707171
Slc44a2	Slc44a2	0.00337677193118727	6.15688853634043	0.00528076045971204	0.942069644210471	0.989981341707171
Exog	Exog	0.00333781380820224	4.77937430259196	0.00524688316318844	0.942255435676387	0.989981341707171
Tmem143	Tmem143	-0.00311374163321275	3.98282253429555	0.00524387803650939	0.942271945610178	0.989981341707171
L3mbtl2	L3mbtl2	-0.0037155414574806	5.94297273818669	0.00524233312546585	0.942280435082899	0.989981341707171
Rnf166	Rnf166	-0.0026570977918373	4.61440304186602	0.00524039680145449	0.942291077193057	0.989981341707171
Pou6f1	Pou6f1	0.00334829733882674	4.8987429496713	0.00522996063682347	0.942348578853917	0.989981341707171
Cog2	Cog2	-0.0032538752514263	5.21352192980624	0.00522614917111497	0.942369443561155	0.989981341707171
Ccdc107	Ccdc107	-0.0027642617796522	5.23383869329774	0.00522458001292136	0.942378080976888	0.989981341707171
Rpap1	Rpap1	0.00307117026679693	5.15726403558495	0.00522330051696684	0.942385124914786	0.989981341707171
Bora	Bora	-0.0070634802412455	2.46962170529296	0.0052231081348566	0.942386184100314	0.989981341707171
Dlgap1	Dlgap1	0.00204615727645276	7.69930793482177	0.00519822367122913	0.942523354550786	0.989981341707171
Lrrc49	Lrrc49	-0.0018782587113318	6.95734154273831	0.00519182143106655	0.942559803398589	0.989981341707171
Copg1	Copg1	-0.0018191406228941	7.48662727664438	0.00518466067432399	0.94259825673869	0.989981341707171
Cyb561d1	Cyb561d1	0.00817884610323386	2.98698270534975	0.00517134697830102	0.942671877994299	0.989981341707171
Gria3	Gria3	-0.0035139835197319	6.72000739445475	0.0051672002232408	0.942694827915819	0.989981341707171
Tspan4	Tspan4	-0.0023882372665280	5.36305428839867	0.00513529423979975	0.942871718816155	0.989981341707171
Bcl7b	Bcl7b	-0.0030096034312806	4.18340124055073	0.00513057348554291	0.942897939464839	0.989981341707171
Casr	Casr	0.0117849300663649	1.26826636364955	0.00511120560343299	0.943005637270643	0.989981341707171
Nr2c2ap	Nr2c2ap	-0.0030829908832545	4.47988596560272	0.00509482213673351	0.943096900265131	0.989981341707171
Ccnl2	Ccnl2	0.00718462674587791	5.25380442862813	0.00508861318375308	0.943131525278313	0.989981341707171
Tube1	Tube1	0.00509381036964583	3.524251149974	0.00508662516245261	0.943142616291596	0.989981341707171
Plekhd1	Plekhd1	-0.0105420285895568	1.64611197749028	0.00506305549596675	0.943274275211611	0.989981341707171
Kif13a	Kif13a	-0.0026447850637119	6.39934697543114	0.0050600641754014	0.943291118573468	0.989981341707171
Rida	Rida	0.00301241841067453	2.9084589857891	0.00504738768547242	0.943361965434888	0.989981341707171
Anp32a	Anp32a	-0.0026191602788383	7.2337746750668	0.00504351627051847	0.943383654166402	0.989981341707171
Mcm8	Mcm8	0.00579255104824758	2.28428447304632	0.00503950780831985	0.943406119491726	0.989981341707171
Ddit3	Ddit3	-0.0041165174078920	3.28492099326495	0.00501756022458721	0.943529283669343	0.990027320859675
Nsmce2	Nsmce2	-0.0037112127089182	4.48481987835161	0.00491699905562371	0.944097100189662	0.990042142297388
Brap	Brap	-0.0015603584567381	6.45135812298357	0.00488242873815954	0.944236720722401	0.990042142297388
Lnpk	Lnpk	-0.0022878461064982	5.37244314820295	0.00488705323702021	0.944267313881081	0.990042142297388
Hbegf	Hbegf	-0.0034078452722626	3.89033765043138	0.00487754211744829	0.944321485205546	0.990042142297388
Zkscan3	Zkscan3	0.00257732159360197	5.30901679888188	0.00487285041223238	0.944341955847176	0.990042142297388
P4hb	P4hb	-0.0022015344847871	8.02677032205464	0.00485022676615188	0.944477357698831	0.990042142297388
Yrdc	Yrdc	0.00292291312232026	4.9520659368874	0.00484188882305624	0.944526168098809	0.990042142297388
Ulk2	Ulk2	-0.0017525630513226	8.06264757668059	0.00483899927913711	0.944541554170596	0.990042142297388
Timm8a1	Timm8a1	0.00311438641359286	4.84377179466704	0.00481285866234771	0.944691311434196	0.990457574089767
Gpn3	Gpn3	-0.0030875688251256	4.19935233677027	0.00480556724759396	0.944733156146895	0.990457574089767
Pcdh17	Pcdh17	0.00558367599526133	6.95580118311804	0.00478056309799513	0.944876895291602	0.990515782638298
Eme1	Eme1	0.0106722428286227	0.167781108869813	0.00476436300571947	0.944970225040293	0.990515782638298
Glipr1	Glipr1	-0.0043654253264774	2.72473614726233	0.0047545493805714	0.945026928506797	0.990515782638298
Galnt3	Galnt3	0.00896017155247026	1.61397941705361	0.00472540072799177	0.945195344107936	0.990609181724882
Mkrn2	Mkrn2	0.00196168235289186	5.37416215145238	0.0045920074663357	0.945973221110364	0.991321844411548
Pdxp	Pdxp	-0.0026807058530044	7.58414235512804	0.00457577703662082	0.94606863853147	0.991321844411548
Brox	Brox	0.00158549440882555	7.58578787235	0.00455960721904347	0.946163868816261	0.991321844411548
Wdr73	Wdr73	0.00243505065029342	5.17810799266984	0.00454418210691898	0.94625487146197	0.991321844411548
Fktn	Fktn	-0.0024905748811848	5.72923150260665	0.00452135612874827	0.946389821723694	0.991321844411548
Fbxo15	Fbxo15	-0.0065363368319156	1.44773572312015	0.00450297445586045	0.946498745582997	0.991321844411548
Samd5	Samd5	-0.0055316817329443	3.56336719998533	0.00448754307345212	0.946590358526719	0.991321844411548
Ppcs	Ppcs	-0.0036843004315143	3.83534705472133	0.00447697062976005	0.946653218002054	0.991321844411548
Ttc3	Ttc3	-0.0023206414065300	10.6618325152117	0.00447238252982629	0.946680519750433	0.991321844411548
Slc39a1	Slc39a1	-0.0031239974061348	5.21935966144163	0.00444930819584499	0.946818038640961	0.991321844411548
Aven	Aven	0.00426264776098757	2.72153567253241	0.00444542398549963	0.946841223148743	0.991321844411548
Naa50	Naa50	-0.0036724555569496	5.83941834098666	0.00442873591910567	0.946940948053553	0.991321844411548
Ubac2	Ubac2	-0.0026480524324239	5.39926186445509	0.00442306905858914	0.946974855151286	0.991321844411548
Bicd1	Bicd1	-0.0041246739172810	5.11979340622079	0.00440932648979242	0.947057173167344	0.991321844411548
Commd3	Commd3	-0.0029331527752607	6.90920023452476	0.00439849943867898	0.947122118010745	0.991321844411548
Pigbos1	Pigbos1	-0.0067947646635981	1.47489184534719	0.00435224401793022	0.947400484302976	0.991321844411548
Zdhhc2	Zdhhc2	0.00238355488603758	6.00520199321374	0.00433551549475908	0.947501522528634	0.991321844411548
Grcc10	Grcc10	0.0037429202054793	6.74291036742098	0.004332342510559325	0.947514101501308	0.991321844411548
Zfand6	Zfand6	0.00227245834021562	5.97584977995478	0.0043203823234137	0.947593093815366	0.991321844411548
Ccdc112	Ccdc112	0.00298188525906158	4.75687285800271	0.00431119431205929	0.947648708853274	0.991321844411548
Chst10	Chst10	0.00200879995086926	6.67712346900616	0.00430492563317131	0.947686789147744	0.991321844411548
Ebf1	Ebf1	-0.0121813958872857	3.74735018386583	0.00429918262058483	0.94772164516855	0.991321844411548
Rtbdn	Rtbdn	-0.012005648371906	3.02385536842689	0.00429717172912082	0.947733855375574	0.991321844411548
Miat	Miat	-0.0104791573674676	9.4028167739521	0.00428921752273936	0.947782181773291	0.991321844411548
Lmo1	Lmo1	0.00414199447522658	4.95902727498667	0.00426550529598682	0.947926514905122	0.991389700458609
Kcne5	Kcne5	0.00591048848099706	3.42575676991677	0.00424707598731278	0.948038970028423	0.99142420828469
Akap17a	Akap17a	0.00236669365104318	6.29201054438561	0.00421412841768642	0.94824062765346	0.991470726320704
Ube2c	Ube2c	0.00549019273982407	2.85038079365775	0.00420056538658287	0.948323870934634	0.991470726320704
Yaf2	Yaf2	0.001932230300908146	6.27232038596857	0.00419836107396776	0.948337412674358	0.991470726320704
Phldb3	Phldb3	-0.0071872613029677	1.59124997152361	0.00418796810259882	0.94840130789754	0.991470726320704
Dexi	Dexi	-0.0031078008790652	4.28784692364465	0.00417360008534473	0.948489772667319	0.991480135185166
Calr3	Calr3	0.00777434755669642	0.807237727320667	0.00415557816924661	0.948600951183786	0.991494181862383
Dhtkd1	Dhtkd1	0.0040181483445596	4.4228857973536	0.004145675945324	0.948662141786738	0.991494181862383
Exoc3l4	Exoc3l4	0.00964371326055854	0.514652711794832	0.00411962141279159	0.948823434694938	0.991509443158
Fam227a	Fam227a	0.00888988606735877	5.10045657094535	0.00411781209209039	0.948834720790777	0.991509443158
Tmeff2	Tmeff2	-0.002848542025840	6.22924463459123	0.00409046830839088	0.949004548587851	0.991603965656781
Atg16l2	Atg16l2	-0.0065588855023813	1.65708958366254	0.00404881689754433	0.949264593326471	0.991791519604165
Slc22a2	Slc22a2	0.0194449936529068	0.414983023035061	0.00400881649645985	0.949514871141871	0.991827227935881
Cabyr	Cabyr	-0.0066549186892617	1.90093405446521	0.00398759365037111	0.949649195900033	0.991827227935881
Cul2	Cul2	-0.0016272583497285	6.69644952370605	0.00397589971490337	0.949722286648274	0.991827227935881
Ddx39b	Ddx39b	-0.0036256120229142	8.53681015193638	0.00396036115370773	0.949821763187717	0.991827227935881
Trim5	Trim5	0.00586563383787012	2.20656898830379	0.00393492767749931	0.949982228227914	0.991827227935881
Rgs10	Rgs10	0.00369380098045301	5.28813896493165	0.00393271553501506	0.949996345756279	0.991827227935881
Htra1	Htra1	0.00700367647477103	3.37036476614	0.00393261363291231	0.949996992743631	0.991827227935881
Vac14	Vac14	-0.0015192854454704	6.40032629119837	0.00393223403542581	0.949998767981208	0.991827227935881
Nhlh1	Nhlh1	0.0120985428160602	1.46404243697767	0.00392990358494671	0.950014202217017	0.991827227935881
Oard1	Oard1	-0.0037702579580137	3.00351028623739	0.00388468577151713	0.950302230346898	0.992044923697979
Mtrf1l	Mtrf1l	-0.0028808801989366	3.53310265574585	0.00384968183239209	0.95052635567856	0.992195878842248
Coa3	Coa3	0.0031174150326367	4.86222446882169	0.00382612577273278	0.950677758089622	0.992270904109291
Dtx2	Dtx2	0.00299096808567276	4.26243911757236	0.00378656319992388	0.950933096105752	0.99244074828918
Suv39h1	Suv39h1	-0.0020891062502177	5.32454327363822	0.00377629806383339	0.950999566106141	0.99244074828918
Phospho	Phospho2	-0.0026291718538987	4.4259416296847	0.00372574531614589	0.951328242104725	0.992700717298708
Xylb	Xylb	-0.0065284332696527	3.33316252089681	0.0037089099955634	0.951438195878108	0.992732427661076
Unc13a	Unc13a	-0.0042745761231843	7.83319598898858	0.00368971903253623	0.951563840057475	0.992780502750825
Atf7ip2	Atf7ip2	-0.011799015684793	-0.207347149478675	0.0036497805950404	0.951826375219154	0.992971378232532
Sec61a1	Sec61a1	0.0015032715364267	7.15812926781569	0.00362985143365099	0.95196585104224	0.993033853645517
Rbm33	Rbm33	0.00595046719982487	6.46003704981814	0.00359414282440833	0.952229099661195	0.993203006638219
Xrcc2	Xrcc2	-0.0037186470549746	3.15356336546816	0.00358002052181745	0.952287213946703	0.993203006638219
Dusp1	Dusp1	-0.0030440457288509	4.67435612382384	0.00355150922347036	0.952478566918847	0.993229917843552
Gsx1	Gsx1	-0.0077849659214007	0.787113703423843	0.00354282187983248	0.952529228883959	0.993229917843552
RGD1309	RGD130959	0.00253022549786438	5.63228106093783	0.00352348252562784	0.952666224897123	0.993229917843552
Aif1	Aif1	0.0069156634061509	2.12028583013994	0.00352345221710948	0.952666428103745	0.993229917843552
Doc2b	Doc2b	-0.0030264198301131	5.75235767709775	0.00350839348502063	0.95276756638775	0.993229917843552
Ncapd3	Ncapd3	0.00265059498511733	4.95172551079564	0.00349466332681263	0.952859971636895	0.993229917843552
Rhbg	Rhbg	-0.0097726632951067	-0.093122262361565	0.00349313768461457	0.952870250588067	0.993229917843552
Trmu	Trmu	0.00367085094905376	2.5238614894161	0.00347585226218904	0.952988219132572	0.993269903108838
Rab12	Rab12	-0.0019448623315426	7.26771149194694	0.00345506071353441	0.953127526185568	0.993325064180115
Insrr	Insrr	-0.0124187435187878	0.146515777346085	0.00344431808522927	0.953200368151681	0.993325064180115
RGD1564	RGD156414	-0.0132571678215737	-0.509884217162712	0.00342674602904935	0.953319764208997	0.993368851898072
Siah2	Siah2	0.00242688254514758	4.67828560252979	0.00340286431239178	0.953482525749543	0.993426243576297
Gckr	Gckr	-0.0082844764656657	0.636394574742128	0.00339498926656745	0.953536322278571	0.993426243576297
Tfb1m	Tfb1m	-0.0029340415126010	4.37991579552536	0.00336456457394262	0.953744751900246	0.993560429945677
Cab39	Cab39	0.00121229780985796	7.81921774183709	0.00333880808662457	0.953921941411007	0.99364499977248
Kctd18	Kctd18	0.00243371442059196	5.12632744887192	0.00332962549316645	0.953985208966461	0.99364499977248
Ppie	Ppie	0.00257804469425858	4.08296036327427	0.00325066610305669	0.954533554533479	0.993846197409214
Ptprv	Ptprv	0.0110492580997509	-0.041417302906141	0.00324145861810621	0.954597922218495	0.993846197409214
Mkks	Mkks	0.0023816580044889	5.05113566356056	0.00322396615347031	0.954720461678015	0.993846197409214
Smagp	Smagp	-0.0085961228800954	-0.0109886007153921	0.00322102128483775	0.954741053871503	0.993846197409214
Scrn2	Scrn2	0.00369561758032487	2.89432052623992	0.00321941003788848	0.954759453529887	0.993846197409214
Spata24	Spata24	0.00622657308674954	1.15982069421774	0.00321446372382994	0.954787168586853	0.993846197409214
Vps35	Vps35	0.00164231736489602	7.97987359083863	0.00320184570414739	0.954875899665975	0.993846197409214
Snrk	Snrk	0.00140865439126791	6.68143726350485	0.00320116853680474	0.954880666829589	0.993846197409214
Acap3	Acap3	-0.0020217389063780	7.72165394582022	0.00319909529125795	0.954895264448846	0.993846197409214
Snrnp70	Snrnp70	-0.0044489656361511	7.53184794142511	0.00317112738699959	0.955092580053843	0.993922532584579
Rev1	Rev1	0.00224660318343055	6.3483964226255	0.00316614180054842	0.955127928643833	0.993922532584579
Cdk5rap2	Cdk5rap2	-0.0033894212222356	5.65372690180486	0.00313420201152326	0.95535459774836	0.993999663039315
Hnrnpa3	Hnrnpa3	0.00192365679368158	9.18621857015524	0.00313182749482621	0.955371495298972	0.993999663039315
Cse1l	Cse1l	0.00147214239828375	7.08826676007996	0.00312206312049312	0.955441048235293	0.993999663039315
Eml5	Eml5	-0.0066149164580021	5.16561467073716	0.00308169928313369	0.955729944809874	0.994112038480179
Sf3a1	Sf3a1	-0.0017086956642076	7.105574006509	0.00306375447934215	0.955858679156557	0.994112038480179
Rala	Rala	-0.0016847623141240	6.84014676719241	0.0030537350684483	0.955930842489087	0.994112038480179
Pcdha5	Pcdha5	-0.0030340714422557	3.80883638524319	0.00304243252318582	0.956012390231577	0.994112038480179
Gsx2	Gsx2	-0.0101581263761359	0.380404513548795	0.00304117509966773	0.95602147175219	0.994112038480179
Reep3	Reep3	-0.0025137367429720	5.25517362899129	0.00304039348398533	0.956027117874783	0.994112038480179
Parm1	Parm1	-0.0040072818566408	3.08375155247838	0.00300113458243345	0.956311653178256	0.994189213548987
Klhl22	Klhl22	-0.00165788901617575	6.45923958609031	0.00299496992560933	0.956356501726498	0.994189213548987
[illegible]	[illegible]	[illegible]	[illegible]	[illegible]	[illegible]	[illegible]

Plcl2	Plcl2	0.00098211135934698	6.32747140637506	0.00152269627304863	0.96887315713533	0.996393971299255
Vwa7	Vwa7	-0.0081304788253871	-0.216814128131303	0.00151504824457049	0.968951284280411	0.996393971299255
Brms1l	Brms1l	-0.0011523638079682	6.15924966663855	0.00152171271978967	0.968885464245329	0.996393971299255
Dhps	Dhps	-0.0015717070878174	6.22619150865821	0.00151142784235958	0.96898838520596	0.996393971299255
Dync1li1	Dync1li1	0.00178306741532875	8.28256191417493	0.00149481308580324	0.969159222291432	0.996393971299255
Pbxip1	Pbxip1	-0.0024549734997705	5.15173982770742	0.00148316857155351	0.969279521647855	0.996393971299255
Fibp	Fibp	-0.0015187290290947	5.10890523893792	0.00148191963950239	0.969292452380872	0.996393971299255
Brca2	Brca2	-0.0034811772851753	3.45157717881482	0.00146830513026554	0.969433764803974	0.996393971299255
Dpagt1	Dpagt1	0.00141317511292278	5.17181225008968	0.00144401206154754	0.969687555493248	0.996393971299255
Yif1b	Yif1b	0.00200823795437449	5.50387711948429	0.00143912461835782	0.969738872497307	0.996393971299255
Dio3	Dio3	0.00798801885890141	1.71749295028038	0.00142902144794784	0.969845230796319	0.996393971299255
B4galt2	B4galt2	0.00132679886580174	7.74926376571439	0.00142670238940923	0.969869697115242	0.996393971299255
Lrrk2	Lrrk2	0.00277068656789716	3.07291632351918	0.00142486714307921	0.969889073281946	0.996393971299255
Cpt1b	Cpt1b	-0.0056202450447119	3.93344541802227	0.00141939954723114	0.969946873263454	0.996393971299255
Mgat4b	Mgat4b	-0.0011272098225131	7.75721636381721	0.00140876882555938	0.970059663601229	0.996393971299255
Fancc	Fancc	-0.0025786994700792	2.55512643268483	0.00139581682020305	0.970197464022622	0.996393971299255
Rad23a	Rad23a	-0.0015528877554248	5.20103001120658	0.00139536643600735	0.970202268189676	0.996393971299255
Ccdc163	Ccdc163	-0.0032875176383473	1.54764912254738	0.00139010815345841	0.970258428835204	0.996393971299255
Dnaja2	Dnaja2	-0.0010287116105238	7.63168718070692	0.00138142821165579	0.970351278655499	0.996393971299255
Pdzd3	Pdzd3	0.00394197892989705	2.48834980622456	0.00136776774968439	0.970498286042192	0.996393971299255
G2e3	G2e3	-0.0030952379050566	4.79497631445427	0.00135404476458551	0.970539446107898	0.996393971299255
Usp13	Usp13	0.00178438051718386	5.28582095350602	0.00134153968940486	0.970782387272234	0.996393971299255
Htatip2	Htatip2	0.00400830462304145	1.26034820808522	0.00134074913827931	0.970790993408088	0.996393971299255
Ssl5	Ssl5	0.0019845621084997	7.10176956193463	0.00133940330366045	0.970805650437907	0.996393971299255
Terf1	Terf1	0.00238482064014098	3.68776556414766	0.00132969328614425	0.970911727554896	0.996393971299255
RGD1560	RGD156041	0.003022023378657041	1.72563222799559	0.00132222182687596	0.970993310445184	0.996393971299255
Osnad1	Osnad1	0.00178326494487426	3.67145553278549	0.00131622326679803	0.97105926342669	0.996393971299255
Slc41a2	Slc41a2	-0.0014613789673710	5.6957363511524	0.00131612789544278	0.971060311484852	0.996393971299255
Slc6a15	Slc6a15	0.0009744689899688	6.98989600813553	0.00130411070605874	0.971192676801326	0.996393971299255
Lypla2	Lypla2	0.001251322530934898	6.65253594326325	0.0013002968415492	0.971234812790903	0.996393971299255
Ddx42	Ddx42	-0.0012434694558271	7.3966466223415	0.00128014412498945	0.971458496752428	0.996393971299255
Taz	Taz	0.00232768775242064	5.46040961322081	0.00127799495898273	0.971482455009099	0.996393971299255
Cep57	Cep57	-0.0011677150685106	5.42209250942561	0.00127309924403107	0.971537106282243	0.996393971299255
Atp6v1g1	Atp6v1g1	0.00154550761193537	6.84293413825126	0.00125539718100232	0.971735599605994	0.996393971299255
Smurf1	Smurf1	-0.0010166882992882	6.88980342405551	0.00125379108108348	0.971753678080219	0.996393971299255
Aimp2	Aimp2	0.00149982389584345	5.54017155444763	0.00125118678552383	0.971783016801128	0.996393971299255
RT1-T24-	RT1-T24-3	0.00693048442622795	-0.288968524190242	0.00124057008765988	0.971902938943825	0.996393971299255
Maf1	Maf1	0.00141779969243084	6.98265545812857	0.00123801413535318	0.971931884167561	0.996393971299255
Timm10b	Timm10b	0.00226425722186887	3.70267693820218	0.00123213222191873	0.971998601785886	0.996393971299255
Pam	Pam	-0.0011725705998082	7.21168086543925	0.0012242100874289	0.972088740514859	0.996393971299255
Plod2	Plod2	0.00182101467602644	3.88959026784446	0.00121668081162886	0.972174898087775	0.996393971299255
Rab13	Rab13	0.00238480069730192	2.83381546407059	0.00120189754123007	0.972343013686751	0.996393971299255
Slc4a11	Slc4a11	0.00485643570632274	2.36111432634221	0.00120062982274936	0.972358749452155	0.996393971299255
LOC1009	LOC100910	-0.0019582905532489	4.11289047611556	0.00118497432620221	0.972539482807648	0.996393971299255
Akap7	Akap7	-0.0018659769154549	5.05822396490213	0.00118213404162507	0.97258080252352	0.996393971299255
[illegible]						

**Erfb**	Erfb	7.31771012653285e-0	4.3645046397998	1.70570325508379e-06	0.998857942515703	0.99968484790081
**Cep162**	Cep162	9.15098078607969e-0	4.03169870692031	1.17412549035549e-06	0.999135432144654	0.99968484790081
**Myo1b**	Myo1b	4.48729323013636e-0	6.67370861464223	8.35087064388063e-07	0.999270868469444	0.99968484790081
**Ykt6**	Ykt6	2.93886921291936e-0	6.42677097423405	8.16256658708886e-07	0.99927913594625	0.99968484790081
**Drosha**	Drosha	-4.69312389623316e-0!	7.63551682701651	6.88425615180677e-07	0.999337983944411	0.99968484790081
**Zfp330**	Zfp330	-2.24681465116738e-l	5.75602237294236	5.01550385881623e-07	0.99943493642351	0.99968484790081
**Arl10**	Arl10	-3.06554256482104e-l	5.26159275082346	4.83922420357885e-07	0.999444955278913	0.99968484790081
**Fam167a**	Fam167a	-5.59417362940367e-0!	2.81421495846184	3.75420910359026e-07	0.99951112238511	0.99968484790081
**Foxj1**	Foxj1	-7.37000652284895e-l	4.43286175021938	3.55007571783972e-07	0.999524600365173	0.99968484790081
**Fam76b**	Fam76b	1.33655475291403e-0	5.07131000088492	5.12378477424136e-08	0.999819392618065	0.99989953203716
**Ablim2**	Ablim2	-8.68416877254108e-l	5.28454852075009	1.79056789411545e-12	0.999998932223948	0.999998932223948

Supplementary Table 11 - Control vs MARA PC12 collected DE genes

gene_id	gene_name	logFC	logCPM	LR	PValue	FDR
Grin3b	Grin3b	0.379520038173682	4.20064453527082	25.3834696352447	4.69927731009361e-07	0.00586322882998038
Mfrp	Mfrp	2.06811235552179	3.69516034252233	21.0089109292759	4.57629662621637e-06	0.0285492265026508
Creb1	Creb1	0.270919166959885	4.22611878136392	18.9802701379764	1.32077131884773e-05	0.054930879150877
Sav1	Sav1	0.18542137155442	4.99552485439093	17.7173862851333	2.56273537564503e-05	0.0598096873612458
Mt1	Mt1	-0.365931896499064	2.42504550708286	17.6132716026431	2.70692672501932e-05	0.0598096873612458
Polq	Polq	0.632479161327029	1.57938661551887	17.5364977967534	2.81845094307506e-05	0.0598096873612458
Tpsab1	Tpsab1	1.3202767989754	1.22437270422486	16.5474261914561	4.74493430787075e-05	0.0845732822704334
Pag1	Pag1	0.244214851806276	3.14707772499115	15.8140252162161	6.98825725592433e-05	0.0885034516074374
Barhl1	Barhl1	-1.66715558281716	1.40820628245054	15.5625022752435	7.98220449707762e-05	0.0885034516074374
Pcsk9	Pcsk9	-0.91810399498134	0.472068935351261	15.5493973406156	8.03772659638241e-05	0.0885034516074374
Ptbp3	Ptbp3	0.191409493362758	4.01676922010506	15.4652419628818	8.40364854760056e-05	0.0885034516074374
Kmt5a	Kmt5a	0.154808591584789	5.17107611957198	15.4410311392261	8.51199342221086e-05	0.0885034516074374
Calb2	Calb2	-0.238090031385432	6.72548895598413	14.9292571614179	0.000111618489140753	0.0984919136626789
Bach2	Bach2	0.252956448742004	5.76844530988513	14.6275752747104	0.000130983870558536	0.0984919136626789
Vsnl1	Vsnl1	-0.18136110616791	7.36115804961273	14.5655487519925	0.000135366947335152	0.0984919136626789
Bach1	Bach1	0.138142248229783	5.54228623883832	14.4945692681676	0.000140564192946707	0.0984919136626789
Igf2bp3	Igf2bp3	0.387904486968866	3.11388292998661	14.4415978774488	0.000144556409859258	0.0984919136626789
Sox11	Sox11	0.250952652196575	7.93442631250243	14.4126370244372	0.000146813787055892	0.0984919136626789
Banp	Banp	0.204723567490783	4.77440069504209	14.3482380138916	0.000151921725601118	0.0984919136626789
Tbccd1	Tbccd1	0.19676154884933	3.91729897743888	14.2758492315215	0.000157877556585968	0.0984919136626789
Chga	Chga	-0.147231280822516	6.94912385252598	14.0775346017997	0.000175426610045595	0.103523760086695
Vxn	Vxn	-0.152122257768927	5.66854589143373	14.0004137203029	0.000182770412859989	0.103523760086695
Clic6	Clic6	1.41857628584211	3.29570048301689	13.9192414335444	0.000190834854692153	0.103523760086695
Rnf165	Rnf165	0.159962536210239	5.62847069922599	13.7907628807294	0.000204338424668549	0.106230438534562
Chgb	Chgb	-0.130877489038557	7.35424091890033	13.6179508395617	0.000224603307946210	0.107724217559731
Rai14	Rai14	0.182927647932743	4.87737871424553	13.6142142808545	0.000224479414647192	0.107724217559731
Cntn2	Cntn2	0.226950067647531	7.35853763417247	13.5257851828938	0.000235307919706972	0.108738454228039
Zfp112	Zfp112	0.254799830015353	2.71522281386264	13.2256032897357	0.000276150810506322	0.123054773667407
Tf	Tf	0.787160302511447	3.57101054881915	12.8621010008303	0.000335662476175103	0.144415886732302
Timeless	Timeless	0.19170042680766	4.03645638967977	12.6549735304761	0.000374567898273342	0.15026843648424
Podxl	Podxl	0.186748199106091	6.22348315525184	12.5068152230788	0.000405470174795888	0.15026843648424
Dusp26	Dusp26	-0.171849417116728	7.01642599549749	12.4104231088346	0.000426944121467541	0.15026843648424
Eng	Eng	0.210364764327721	4.90411039261654	12.3983230188894	0.000429719592189199	0.15026843648424
Mapk7	Mapk7	0.107748935200161	5.9634003385466	12.3376947562191	0.000443802847889977	0.15026843648424
Sh2b2	Sh2b2	0.119092999343771	5.69097619871784	12.3290238927651	0.000445869546634986	0.15026843648424
Mmd2	Mmd2	-0.171858926076578	6.10580526631719	12.3110941785382	0.000450276352134648	0.15026843648424
Mtmr4	Mtmr4	0.104891961981025	7.49484728243046	12.2945587358533	0.000454280762092169	0.15026843648424
Hsd11b1	Hsd11b1	-0.336504571410982	2.39706723706824	12.2807348394052	0.000457658131794592	0.15026843648424
Efna2	Efna2	0.145268444323782	4.89644112772446	12.164543098338	0.000487065949469359	0.152164455375797
Map4k5	Map4k5	0.127764210405741	6.00431980099067	12.1616430316315	0.000487823853092241	0.152164455375797
Kcnj13	Kcnj13	0.844828437074727	1.87948724978361	12.0382677313643	0.000521193847035982	0.154677796637565
Cdc6	Cdc6	0.297656151618371	1.54897029965337	12.0229114975235	0.000525505494566881	0.154677796637565
Lrrc2	Lrrc2	0.182236377553256	4.34273899146244	11.9962661244	0.000533072473784987	0.154677796637565
Rcor1	Rcor1	0.14630339420995	5.51146301488404	11.7313340654997	0.000614565224624545	0.167084508732238
Mllt3	Mllt3	0.185783794595088	5.77367325002642	11.7273046439399	0.000615897294839958	0.167084508732238
Nfyc	Nfyc	0.102083608579249	5.76109721662776	11.6918431524547	0.000627746838841517	0.167084508732238
Shroom	Shroom4	0.295721907581656	2.51287160621192	11.6813252109224	0.000631305479114108	0.167084508732238
Src1	Src1	0.251307310456089	2.57833078244245	11.6477937654856	0.000642787242061025	0.167084508732238
Nol3	Nol3	-0.201748922212069	4.53869502518247	11.5570282111176	0.000674926392929218	0.171860844437914
Mcmbp	Mcmbp	0.10276983520025	6.26679743614037	11.5184376996471	0.000689092787253736	0.171956214131297
Abhd14b	Abhd14b	0.28870940885226	2.9124866526906	11.3262724255498	0.000764191453847316	0.186954745091235
Abcg3l3	Abcg3l3	0.540048663198601	0.97367341014385	11.2584234941198	0.000792198109340368	0.188231126746901
Unc13d	Unc13d	0.359523858202142	1.09385521540087	11.2422279792891	0.000799657118838008	0.188231126746901
Mt2A	Mt2A	-0.316784487121401	2.61289595099587	11.1987355237954	0.000819520862282201	0.189126103126213
Ctdspl2	Ctdspl2	0.258078357283035	4.62937809581767	11.1288328791753	0.000849960561525956	0.191511923427859
Tbc1d2b	Tbc1d2b	0.120570150943429	5.47102873167037	11.1080137003858	0.000859955499815341	0.191511923427859
Sipa1	Sipa1	0.227019856926099	4.54570104468676	11.0414357139645	0.000890678349589037	0.193155361359141
Xpo7	Xpo7	0.0806063755888029	7.0092215294943	11.0199932839145	0.000901343926940732	0.193155361359141
Eif4ebp2	Eif4ebp2	0.12517988967864	5.89373883878729	10.9842645193749	0.000918887414629589	0.193155361359141
Zbed4	Zbed4	0.121242401127315	5.24556186091966	10.9642709684606	0.000928854927406306	0.193155361359141
Lix1l	Lix1l	0.116094571893436	6.70249947044568	10.8254684299828	0.00100112362428805	0.204772856232475
Gab2	Gab2	0.126181357988128	5.16596120158352	10.7611377959468	0.00103652640637834	0.205568872956809
Noc3l	Noc3l	0.136979966864788	4.55108905688813	10.7585673643372	0.00103797098726739	0.205568872956809
Niban2	Niban2	0.112921780109416	6.31151795328761	10.5957739196751	0.00113346443418375	0.216026018640405
Pcdha11	Pcdha11	-0.222104366034154	2.9222478293645	10.5823581033254	0.00114171935408655	0.216026018640405
Zbed5	Zbed5	0.226440042569884	3.17429042702109	10.5807385632996	0.00114271998319041	0.216026018640405
Tmtc4	Tmtc4	0.153540217478297	6.46003617909151	10.5182090231685	0.00118140040296285	0.219822944420563
Nrip3	Nrip3	-0.158546818079968	6.08594519436782	10.4925225126063	0.00119858621628583	0.219822944420563
Atp1b1	Atp1b1	-0.11846511934161	9.4588327307969	10.4175437017547	0.00124923805200637	0.221512260601574
Bcorl1	Bcorl1	0.164574718460345	5.82503408887519	10.4029108152792	0.00125916827541932	0.221512260601574
Fam162a	Fam162a	-0.240503756424805	3.27690747282829	10.3984786497854	0.00126050897673613	0.221512260601574
Ccdc134	Ccdc134	0.226773347142348	2.52008791342134	10.2989539253737	0.00133032530625289	0.228797821069235
Agt	Agt	-0.288644527181501	4.20241995729193	10.2742733759756	0.00134867989931883	0.228797821069235
Pum3	Pum3	0.110541612049083	6.11548368873544	10.2633643088882	0.00135687994396885	0.228797821069235
Kdm6b	Kdm6b	0.167907555107535	7.01886291073935	10.1628662533014	0.00143297664746424	0.230936333906972
Grin2b	Grin2b	0.225262076907689	5.9421777723742	10.1593490695156	0.00143571303861109	0.230936333906972
Arhgap2	Arhgap29	0.251000666887937	5.16486267857568	10.1513887011274	0.00144182581033502	0.230936333906972
Cldn2	Cldn2	1.2036102111224	2.3023978754201	10.1491229329832	0.00144389912997867	0.230936333906972
Sostdc1	Sostdc1	0.860934043061058	3.23704158885397	10.0987222434674	0.00148372164501977	0.233033803552131
Ackr3	Ackr3	0.191275049376358	3.63477801689735	10.0857965223654	0.00149416592725273	0.233033803552131
Ehd2	Ehd2	0.351104310475241	2.54980604938503	10.060996820395	0.00152441265384988	0.233275638050431
Plekhg2	Plekhg2	0.21872984659877	5.27783705227795	10.0027264849872	0.00156308638626343	0.237611225438117
Tspan2	Tspan2	-0.165461751201732	6.0649247011209	9.95077576085511	0.00160781630526366	0.237611225438117
Aldh18a1	Aldh18a1	0.100185689515756	6.69216607852412	9.89776298962505	0.00165479806192496	0.237611225438117
Zeb2os	Zeb2os	0.213985739099182	3.71911425462274	9.87240197400841	0.00167776334266218	0.237611225438117
Optn	Optn	-0.165843117251963	5.73216728504524	9.8678003129862	0.00168196478855278	0.237611225438117
Kiaa0408	Kiaa0408L	0.124786333321406	8.26348275615343	9.86522023417564	0.00168432513528524	0.237611225438117
Mcm4	Mcm4	0.139498115613478	5.4921262761906	9.86064085428315	0.00168852278034298	0.237611225438117
Sfxn1	Sfxn1	-0.0834504043564448	7.0027972216178	9.85389425197297	0.00169491056055081	0.237611225438117
Ehmt1	Ehmt1	0.0949042219126714	7.35261795012286	9.82159233031798	0.00172474955728	0.239107780029175
Slain2	Slain2	0.106775015769229	7.06391372582162	9.78273415348109	0.00176158172294953	0.241520276453201
Fbxl14	Fbxl14	0.110641572480368	5.1822967198869	9.65191987600656	0.00189152787895653	0.256529544736311
Gfod2	Gfod2	0.117374220287033	5.42613656298905	9.61373088927775	0.00193127881422587	0.257213330290281
Mcpt2	Mcpt2	1.2146341690287	-0.360297679514211	9.60753123675967	0.00193780981384038	0.257213330290281
Fam107b	Fam107b	0.151981976722375	5.59589559046621	9.54349216395899	0.00200659599408535	0.257499449710008
Igf1r	Igf1r	0.270934297852508	5.16851261712294	9.5432604831593	0.00200684929178564	0.257499449710008
Nfya	Nfya	0.167254042981607	5.29001370860555	9.53525669783262	0.00201561980208886	0.257499449710008
Barx1a	Barx1a	0.225685474746597	2.69367213264132	9.52888711638372	0.00202251711722215	0.257499449710008
Fzd6	Fzd6	0.182951914263449	3.47713044230332	9.46910046621591	0.00208961814477761	0.260925089960222
Tuft1	Tuft1	-0.242917096721327	2.30284478539087	9.45916830905932	0.00210096310776783	0.260925089960222
Abhd2	Abhd2	0.140109955321451	5.92504990316394	9.4367872979002	0.00212675722556743	0.260925089960222
Smurf2	Smurf2	0.0884995265747843	6.29538970454721	9.42835399906641	0.00213655972158227	0.260925089960222
Rit2	Rit2	-0.127371098556023	6.3919928981106	9.41345815143707	0.00215388607187053	0.260925089960222
Ttr	Ttr	1.34869137369849	9.36454832444042	9.3849124376343	0.00218778502971246	0.262471094381946
Pcdha12	Pcdha12	0.226981139533689	3.42808951021097	9.32791848357368	0.0022568857607707	0.266597116422557
Ccdc77	Ccdc77	0.519979785919205	2.70897628948111	9.31555158197382	0.00227216966877595	0.266597116422557
Virma	Virma	0.130897415882665	5.82068924605768	9.27336411390387	0.00232510258804825	0.266597116422557
Cmtm5	Cmtm5	-0.285011980524887	2.60750632776027	9.26747485697847	0.00233259072703145	0.266597116422557
Tph1	Tph1	2.2354773891763	2.50868065046245	9.25963587417874	0.00234259579937002	0.266597116422557
Zmrd1as	Zmrd1as1	0.44168938613592	0.960297394409467	9.24511351499854	0.00236124658664752	0.266597116422557
Rnpepl1	Rnpepl1	0.116084404856634	5.38610710327769	9.23299886719499	0.00237692031532511	0.266597116422557
Slc12a7	Slc12a7	0.191191070367534	4.45005792409594	9.21483154060003	0.00240062300173283	0.266597116422557
Tmem25	Tmem254	-0.148350752314746	5.31046192986908	9.18911727394525	0.00243459272104799	0.266597116422557
Lin7b	Lin7b	-0.183197270963243	4.67953074510845	9.17004502564281	0.00246008516890241	0.266597116422557
Mybph	Mybph	-0.127113801672122	4.96970265415367	9.15176707745767	0.00248477992349379	0.266597116422557
Plcd4	Plcd4	-0.218553145858403	3.17680971205077	9.14571797776007	0.00249164584469835	0.266597116422557
Trim67	Trim67	0.143186944500226	7.13238298079008	9.13432113467523	0.00250858524372337	0.266597116422557
Plxna2	Plxna2	0.16582778261575	8.4533872390282	9.12512873754027	0.00252122142645261	0.266597116422557
Dnajb12	Dnajb12	-0.0984626439662436	5.19702765789875	9.09453019072826	0.0025637505789779	0.268155449835295
Cpne9	Cpne9	-0.427581372696067	2.27463832295869	9.07615224971481	0.00258962936227082	0.268155449835295
Maml1	Maml1	0.12112500742302	4.79782174242505	9.06848121783514	0.00260052972910721	0.268155449835295
Slc5a5	Slc5a5	0.591303422080914	2.34803771799679	8.97961885988983	0.00273007588752121	0.272108804301016
Pwp1	Pwp1	0.11173670949827	5.19627468030969	8.97168100741526	0.00274196208322153	0.272108804301016
Amd1	Amd1	0.0874642106351517	7.18525748897658	8.95586048767713	0.00276580881040396	0.272108804301016
Tmem10	Tmem106a	0.51821907677624	0.561054963081879	8.94697820506417	0.00277928953194496	0.272108804301016
Smarca1	Smarca1	0.0903779474994204	6.44290420631453	8.92150092610822	0.00281832797995268	0.272108804301016
Vegfd	Vegfd	0.366628890085705	1.57245512721932	8.91479717825715	0.00282869226070641	0.272108804301016
Rbm47	Rbm47	0.887476049121105	0.232645840943359	8.88756105922471	0.00287120012341251	0.272108804301016
Rasd2	Rasd2	-0.1921595196437	5.12157748323706	8.87840391615167	0.00288562709172295	0.272108804301016
Dnajc25	Dnajc25	-0.310489475075558	3.61107789182474	8.87163287332751	0.002896358978043	0.272108804301016
Map3k13	Map3k13	0.180880976660647	4.39758354043004	8.87048046522922	0.00289818853384797	0.272108804301016
Ntn3	Ntn3	0.118785347159734	4.87711912040061	8.85840086791464	0.00291743047379511	0.272108804301016
Pgpep1	Pgpep1	0.105907062729347	5.19035328567084	8.85521339369332	0.00292252949688242	0.272108804301016
Psca	Psca	1.00522443208774	0.0606074294774182	8.84855770379866	0.00293320585122378	0.272108804301016
Igf2bp1	Igf2bp1	0.368334572900625	1.7159039620162	8.82888350958077	0.00296483485907365	0.272108804301016
Epha3	Epha3	0.167268087797742	5.48881417358708	8.82826573882116	0.00296600123306289	0.272108804301016
Pcbd1	Pcbd1	-0.217233214172299	3.83052591707661	8.80593401692745	0.00300252397274575	0.273448843856364
Pak2	Pak2	0.115136988039108	5.985010288092	8.7645120860282	0.00307149232714004	0.276224975546545
Pak5	Pak5	0.0995486417033897	6.67683430047396	8.76107036970564	0.00307728393050972	0.276224975546545
Tmem37	Tmem37	0.55905964779612	0.288325522368222	8.74440293215656	0.00310552767088695	0.276769953711654
Prdm8	Prdm8	0.181726414683222	6.41069031256153	8.71851623352649	0.00314994296970003	0.277058334881328
Tmem15	Tmem151a	-0.133325274597631	6.63144101067129	8.71307059701942	0.00315936609418321	0.277058334881328
Itsn1	Itsn1	0.127925658814139	7.4554324718003	8.7038481052965	0.00317539006876893	0.277058334881328
Lrp5	Lrp5	0.152790718223166	4.67772156682625	8.61190191200134	0.00333973278472825	0.289374016889067
Eml1	Eml1	0.160888588577781	6.34738775441993	8.54309749280878	0.00346834919155281	0.289602068921981
Septin11	Septin11	0.0772587200660376	7.17794684737104	8.52536056598364	0.00350231356265264	0.289602068921981
Arhgef2	Arhgef2	0.109529778106519	6.90705192878201	8.51677795826507	0.0035188695859807	0.289602068921981
Zeb2	Zeb2	0.161974577324688	7.80905732013095	8.51545040208266	0.00352143750534544	0.289602068921981
Afap1	Afap1	0.109891468865738	7.38193807582284	8.51374482911834	0.00352473956437586	0.289602068921981
Zfat	Zfat	0.156826259584122	3.57725773134224	8.50262241772931	0.00354634995953045	0.289602068921981
Enpp2	Enpp2	0.47621901200872	5.90578994975665	8.4877441798419	0.00357540858371277	0.289602068921981
Manba	Manba	-0.201657964721977	3.13774733167187	8.48309188801878	0.0035834614837128	0.289602068921981
Zfp84	Zfp84	0.125252092710821	5.1474069019617	8.47670111056269	0.00359723838620388	0.289602068921981
Rorb	Rorb	0.217882071785473	4.36887041526513	8.44859364885805	0.00365325832236886	0.289602068921981
Ache	Ache	-0.203896080138491	6.35817489111059	8.44835984397519	0.00365373002107969	0.289602068921981
Flt1	Flt1	0.157198386732384	5.01566186912378	8.43676084285958	0.00367710645929013	0.289602068921981
Snrpn	Snrpn	-0.278263205073236	0.835825786422785	8.43199117548593	0.00368676408416087	0.289602068921981
Slc41a1	Slc41a1	0.16091665710302	5.87230725337904	8.42802617558878	0.00369481205142776	0.289602068921981
Atic	Atic	0.0944322193957418	6.6944942829181	8.4221321209957	0.00370680855629303	0.289602068921981
Six5	Six5	0.221731427865151	2.74417645509027	8.41938625918993	0.00371915520497092	0.289602068921981
Ppp2r3a	Ppp2r3a	0.13064182628921	6.69009737217747	8.40740867575804	0.00373685063688698	0.289602068921981
Mertk	Mertk	0.291101802502573	1.90551457988869	8.38544547945229	0.00378237495110185	0.291215435827137
Gng5	Gng5	0.15959485218701	4.12287012006029	8.3705777708048	0.00381344363123509	0.291215435827137
Lmnb1	Lmnb1	0.0974646100908188	6.94452628279954	8.36375411222527	0.00382778965100989	0.291215435827137
Fam107a	Fam107a	-0.17886485668074	4.64977677189537	8.35088325197128	0.00385499889196703	0.291269604607229
Bicd2	Bicd2	0.0737043998723757	7.02611401400486	8.3413914660917	0.00387519069295505	0.291269604607229
Dynlt3	Dynlt3	-0.128675564367645	4.52881172006919	8.30210259558589	0.00395982063531179	0.295855866866977
Psen1	Psen1	0.0809775731248845	5.66065886662355	8.29074967012917	0.00398475307307285	0.295839071980535
Lcp2	Lcp2	0.348688162238436	1.27435886002684	8.27555262115349	0.00401824307344471	0.296660466543414
Cdh3	Cdh3	0.526156769759681	2.13899867882185	8.22037731914791	0.00414225865754677	0.303676552847367
Rwdd2a	Rwdd2a	-0.214700414822926	2.44503390607182	8.2046214515107	0.00417838255312004	0.303676552847367
Zfp709	Zfp709	0.118241954453227	4.12447455759133	8.18725522681778	0.00421857019294503	0.303676552847367
Ddx21	Ddx21	0.0777752176815905	6.74149726301096	8.18362751531863	0.00422701472956615	0.303676552847367
Prdm2	Prdm2	0.108858006897408	6.58214084599995	8.16101515633484	0.00428000407668511	0.303676552847367
Sh3d21	Sh3d21	0.430154778140604	0.254197290017326	8.15678206609796	0.00429004238971876	0.303676552847367
Midn	Midn	0.0859204420099571	7.7637067897627	8.1534219207758	0.00429797468491033	0.303676552847367
Ercc3	Ercc3	-0.0822880314351756	5.38004253318493	8.14409439855432	0.00431873332856933	0.303676552847367
Slco1a5	Slco1a5	1.38543334290358	1.23637150822686	8.13522260706206	0.00434135171134075	0.303676552847367
Pou3f3	Pou3f3	0.157225602290936	8.18283459148457	8.12883862756379	0.0043566644994533	0.303676552847367
Shc1	Shc1	0.184973782363166	4.03563748199666	8.11356538205122	0.00439352285854548	0.304075455507344
Zfp786	Zfp786	0.142781433521379	4.38728222909944	8.09708982936713	0.00443362854088547	0.304075455507344
Mfsd14b	Mfsd14b	0.0993622127595184	5.32533412939839	8.09632914217296	0.0044354999457895	0.304075455507344
Rnf182	Rnf182	0.172337449337909	4.55032410377621	8.07769557294456	0.00448133862597877	0.305528205672717
Kcnip4	Kcnip4	-0.225652502340471	3.1427977032677	8.04009787133901	0.00457530648328504	0.309025320488312
Mapk13	Mapk13	0.966011379585743	-0.504310112525258	8.02957373189439	0.00460196686613373	0.309025320488312
Ascl1	Ascl1	0.15190368195833	4.16695107451071	8.02768303514183	0.00460677322153685	0.309025320488312
Ica1	Ica1	-0.104068389719683	5.70892667864665	8.00021381015071	0.0046622253121256	0.311072648232038
Trim36	Trim36	0.157803263458149	4.2889068774623	7.97956221894415	0.00473083808356424	0.312406959491818
Smtnl2	Smtnl2	-0.2086409192436	2.59200518210492	7.96738158553978	0.00476277843212335	0.312406959491818
Zfp951	Zfp951	0.378557954784483	0.51245487920951	7.9638917857666	0.00477186988937297	0.312406959491818
Aifm3	Aifm3	0.340797022719387	3.20638849946815	7.95348013768813	0.00479949967315666	0.312406959491818
Shoc2	Shoc2	0.082087090807416	6.58367889385293	7.95049727171995	0.00480741654423571	0.312406959491818
Mafk	Mafk	0.124656499298464	4.23298354852004	7.90467643467233	0.00493071604413997	0.318758295765464
Spen	Spen	0.152371504562085	7.37826252881381	7.88447886505152	0.00498693436790054	0.318995822581566
Spats1	Spats1	0.256978713291473	1.86431123615067	7.8724885406686	0.00501925315007392	0.318995822581566
Araf	Araf	-0.0756168001250171	7.01559893191486	7.8629690062048	0.00504576966188603	0.318995822581566
Ccnjl	Ccnjl	0.151569522974745	3.18014785589435	7.86028346385289	0.00505324695810128	0.318995822581566
Mxi1	Mxi1	0.506752217120072	3.37795880800532	7.85394651752526	0.00507098917711805	0.318995822581566
Afmid	Afmid	-0.252462093248655	1.89617528648114	7.83693239992381	0.00511893727624803	0.318995822581566
Egflam	Egflam	0.260717501569202	3.60432479946663	7.82890082943727	0.00514172577874499	0.318995822581566
Fgd3	Fgd3	0.121249334676912	5.39390655179897	7.82717840241401	0.00514662796402966	0.318995822581566
Mn1	Mn1	0.128202531593133	7.63113443476761	7.82092519820719	0.00516447512731296	0.318995822581566
Neu3	Neu3	-0.314237199768474	1.1775964582763	7.80634280506069	0.00520631638537027	0.319520800499892
Fzd8	Fzd8	0.119672280833432	4.70019219000328	7.79275262834748	0.00524562168058933	0.319520800499892
Dusp22	Dusp22	-0.144376158638542	3.04759201002087	7.79131379133324	0.00524980076159957	0.319520800499892
Ppm1a	Ppm1a	-0.0717564398067093	6.71259469959345	7.78210010180908	0.00527664214130519	0.319695456296492
Aff3	Aff3	0.121851969328786	6.72666768660045	7.76809552544221	0.00531770856796483	0.320526810640083
Abcf3	Abcf3	-0.0726765174730446	6.05533303298843	7.74924336606691	0.00537350497215978	0.320676731876198
Cnmd	Cnmd	0.916471699750453	-0.670953650115742	7.74466521031108	0.00538714469381085	0.320676731876198
Tvp23a	Tvp23a	-0.0854442114759847	5.0664132387777	7.74126421983219	0.00539730012775529	0.320676731876198
Tle3	Tle3	0.105898400637962	7.35462991888341	7.7091250911749	0.00549420897843819	0.320922980739083
RGD156	RGD15601	0.11256910702619	8.67746162162262	7.70173987570994	0.00551676530745774	0.320922980739083
Fbxo34	Fbxo34	-0.135342889474226	5.09847054206706	7.68188683016426	0.00557778181868851	0.320922980739083
Slc4a5	Slc4a5	0.819428889127006	2.56192160306004	7.68082716346399	0.00558105805263527	0.320922980739083
Mbd3	Mbd3	0.120417594455668	7.03689120684984	7.67312268942759	0.00560493750859604	0.320922980739083
Rc3h2	Rc3h2	0.178555978010813	5.47367893627331	7.67135335130871	0.00561043613950553	0.320922980739083
Eloa	Eloa	0.0895168287352518	5.54880989136171	7.66562645361129	0.00562827155930671	0.320922980739083
Plxnb3	Plxnb3	-0.34642261976268	2.57133220245382	7.66495404092434	0.00563036949053475	0.320922980739083
Eya3	Eya3	0.103628523796535	5.0618648389909	7.65006921893181	0.00567701745425008	0.320922980739083
Folr1	Folr1	1.00251342197982	2.81387183176656	7.6424485412163	0.00570106772243797	0.320922980739083
Dnaaf5	Dnaaf5	0.122322754825498	3.77761390070416	7.64157662637238	0.0057038040659272	0.320922980739083
Mypf	Mypf	0.294769823887665	2.69857430526574	7.63958780008243	0.00571009871957013	0.320922980739083
Phf3	Phf3	0.0944753603741169	6.72678438450826	7.61714914913972	0.00578158142063288	0.322226309682138
Neurod2	Neurod2	0.161314256161359	7.38876467534566	7.59827808216885	0.00584240718012003	0.322226309682138
Ttll13	Ttll13	0.345530327554555	1.49124847696157	7.59219197420767	0.00586211529973137	0.322226309682138
Tmem97	Tmem97	0.17111976909159	5.09956431840126	7.5903303585779	0.00586821949727647	0.322226309682138
Tmem38	Tmem38a	-0.128613877191045	4.94203127973487	7.58500862728224	0.00588556818625204	0.322226309682138
Clcnka	Clcnka	-0.622749078747924	0.49171911277785	7.58413350991147	0.00588842608700228	0.322226309682138
Limch1	Limch1	0.108701776890532	7.85238003252118	7.5680123695338	0.00594132685332996	0.323711507200864
Aldoa	Aldoa	-0.114520243940067	8.9925086455862	7.53372776325448	0.00605544826534715	0.328494904377115
Mis18a	Mis18a	0.428568921760559	1.1612722679939	7.510352713922	0.00613453234238684	0.331344415740089
Mt3	Mt3	-0.223525973448236	6.02713177088365	7.47965465862583	0.00623996919115116	0.335586618956966
Gabra5	Gabra5	-0.116248247591512	7.21240832872981	7.45559159616919	0.00632386911003726	0.335899522575291
Scg2	Scg2	-0.173109802098353	7.00363103454298	7.45483650793462	0.00632662248454178	0.335899522575291
Echdc1	Echdc1	0.127216291904308	5.00308980534508	7.45432079603281	0.0063284353454511	0.335899522575291
Nfkbil1	Nfkbil1	-0.117992774427296	4.52222598964083	7.43444922708997	0.00639889443238855	0.337269211287813
Mcpt1l1	Mcpt1l1	0.881244544121047	2.54795142912095	7.43228002449197	0.00640641204417823	0.337269211287813
Mtrr	Mtrr	0.138496774064886	3.48068957881077	7.41887782973106	0.00645430568935589	0.338362908145103
Rec114	Rec114	-0.3380103779507094	0.74624692515536	7.3988039588236	0.00652659652082515	0.33892014086796
Zfp319	Zfp319	0.103728956094319	5.36896415523994	7.39550224278254	0.00653871653741146	0.33892014086796
Rbm14	Rbm14	0.0895929265065983	6.41646152224151	7.39338273591169	0.00654642573929457	0.33892014086796
Srebf2	Srebf2	0.0745243011784972	8.29788137167631	7.366940907531	0.00664338538562104	0.340280684086599
Pgap3	Pgap3	0.123952926290652	3.59169880686276	7.3641687991471	0.00665362130306679	0.340280684086599
Il1rl1	Il1rl1	0.739064151220092	-0.874275167126383	7.36392876252462	0.00665452327619861	0.340280684086599
Faim2	Faim2	-0.089647724658882	6.20643994686779	7.34889544318532	0.00671786649774108	0.342087887292871
Nefl	Nefl	-0.187986107488899	7.5312395403935	7.32546282061145	0.00679507228011719	0.342087887292871
Kl	Kl	0.88514470869308	2.76836708295074	7.32245948753479	0.006809815600851	0.342087887292871
Atp13a3	Atp13a3	0.105547870065284	6.08744461170951	7.30962515755833	0.00685982223401686	0.342087887292871
Ctns	Ctns	0.121720938421238	3.29008761423438	7.29826372859167	0.00690981508255723	0.342087887292871
Ppp4r1	Ppp4r1	0.0726339420486849	6.71578195559161	7.28944486842375	0.00693609061035612	0.342087887292871
Lmo1a	Lmo1a	0.640921972058657	1.92558645480113	7.28573665282574	0.00695042236608946	0.342087887292871
Mtfr1	Mtfr1	0.197808859888886	3.04022840045464	7.28533370456329	0.00695198157941612	0.342087887292871
Cux2	Cux2	0.162481874084595	7.23086258580345	7.28003626544671	0.00697251283433424	0.342087887292871
Meltf	Meltf	0.31295175531235	1.80974090583096	7.27412049111871	0.00699551378872007	0.342087887292871
Slc2a4	Slc2a4	0.468180959085773	0.481970278099985	7.27157039429331	0.00700545264189018	0.342087887292871
Ppp1r18	Ppp1r18	0.127704879715129	5.65604406745832	7.25840346127835	0.00705700000467623	0.342087887292871
Dram2	Dram2	0.129026419482084	3.89165393798913	7.25659264883875	0.00706411942135924	0.342087887292871
Gspt1	Gspt1	0.0613485436009452	7.29877462648794	7.25011497953789	0.00708964725092534	0.342087887292871
Nell1	Nell1	-0.153883903895446	5.86681553523212	7.24279950552914	0.00711859013445247	0.342087887292871
St6gal1	St6gal1	0.111326451021009	5.87848826252123	7.23331631784086	0.00715628891635508	0.342087887292871
Acer2	Acer2	0.268784495166068	2.5857207026604	7.22476447005936	0.0071904606103855	0.342087887292871
Slc16a13	Slc16a13	0.140750610579539	3.925802463768	7.21882985588277	0.00721427211267543	0.342087887292871
Megf6	Megf6	0.386578470968106	3.2112249992169	7.2155446130535	0.00722748815209899	0.342087887292871
Crnal	Crnal	0.623477585683311	2.05767471652186	7.21288276880657	0.00723821444943161	0.342087887292871
Me1	Me1	-0.14308091603037	6.43640189486671	7.18226786968656	0.00736275821363544	0.346350131232342
Syp	Syp	-0.121110565203838	9.22767547119005	7.17711928046135	0.00738381720027273	0.346350131232342
Chst12	Chst12	0.116492881641224	4.71714827077578	7.15942745862844	0.00745709956732681	0.347599954582281
Srf	Srf	0.081176900040053	6.19086126424639	7.14545267496408	0.00751543270288829	0.347599954582281
Phyhip	Phyhip	-0.166833569133014	6.06529288875163	7.14504240853739	0.00751714966648854	0.347599954582281
Card6	Card6	0.319734779489631	1.69322150112556	7.14388786712885	0.00752199065257343	0.347599954582281
Fam89a	Fam89a	-0.182424877416215	3.16115975753669	7.11729288222297	0.00763438988085921	0.350276827138956
Fndc1	Fndc1	0.276041585597215	2.66237740056946	7.10823235870276	0.00767307357312063	0.350276827138956
Cnot6	Cnot6	0.119142178863252	6.36622631404181	7.10214338783092	0.00769918282699589	0.350276827138956
Nop53	Nop53	-0.101257702503622	6.61017439407972	7.08274240882641	0.00778298082922802	0.350276827138956
Top1	Top1	0.0951373313854091	7.02837175859882	7.07596122564538	0.00781248998037759	0.350276827138956
Ttyh1	Ttyh1	-0.116488308669636	8.28215780610217	7.07013990169092	0.00783779135184055	0.350276827138956
Man2a1	Man2a1	0.12169262144685	5.38037552515051	7.06620908864113	0.00785512709607831	0.350276827138956
Bcas1	Bcas1	-0.175483028006784	4.84824621217337	7.06435754411629	0.00786325059265529	0.350276827138956
Atp6v0a2	Atp6v0a2	0.111032890406909	4.60357247761916	7.0603190673427	0.00788099601000389	0.350276827138956
Spcs3	Spcs3	0.122522077984033	4.39673253474973	7.05619739693396	0.00789914922127704	0.350276827138956
Ptpn12	Ptpn12	0.085463626324212	5.9430992897236	7.05257224871773	0.00791515091741713	0.350276827138956
Kank4	Kank4	0.316281944280139	2.0429057727339	7.0521543462891	0.00792584216144507	0.350276827138956
Vamp5	Vamp5	0.453515872647681	1.50867594835271	7.04585570435692	0.00794488595658609	0.350276827138956
Slc22a4	Slc22a4	-0.29664530894892	0.612914797067858	7.03783930042418	0.00798052586561096	0.350608229666296
Hapln1	Hapln1	-0.319640623195714	2.23960461817775	7.00385362918865	0.00810170422579017	0.354894082895382
Slco5a1	Slco5a1	0.153190956912045	4.49415362164941	6.98411010198801	0.00822365325257498	0.358764061651671
Gria4	Gria4	-0.129726020117207	6.72477844511129	6.97486470411593	0.0082662472103494	0.359365736737036
Rhbdl1	Rhbdl1	-0.112550104777924	5.83622311283748	6.95046319857125	0.00837975339290898	0.360985452854377
Pdik1l	Pdik1l	0.107648007877335	5.52189606200712	6.94440435962409	0.00840818298956236	0.360985452854377
Plxn1	Plxn1	0.118893506397399	4.75891939611399	6.94188584482545	0.00842002953401558	0.360985452854377
Col8a1	Col8a1	0.616789727556693	1.08394301716522	6.93726343415587	0.00844181680380956	0.360985452854377
Jdp2	Jdp2	-0.162922990510782	3.19320639678507	6.93591918006617	0.0084481636317607	0.360985452854377
Rai2	Rai2	0.134234105693457	3.75276523407571	6.92078754842968	0.00851996461343682	0.361853777272832
Cdr2	Cdr2	0.15141054283612	4.29964573635895	6.91405562407112	0.00855207962319364	0.361853777272832
Klhl14	Klhl14	0.232681338358274	4.02911273607274	6.91334244754393	0.00855549124753431	0.361853777272832
Ndrg4	Ndrg4	-0.105501278267533	9.65871900147746	6.89744366268563	0.00863190900582768	0.362276796226653
Tgfb1	Tgfb1	0.190509756249883	3.14992440271242	6.88890314258589	0.00867324721370981	0.362276796226653
Sh3glb2	Sh3glb2	-0.0940868895279056	6.90142498846385	6.88817158063526	0.00867679750049193	0.362276796226653
Spast	Spast	0.0695714457983771	7.71698809812902	6.88305220335385	0.00870169409751785	0.362276796226653
Tgfbr2	Tgfbr2	0.29762208420326	3.44189094656365	6.8812072539439	0.00871067074360792	0.362276796226653
Tspan17	Tspan17	-0.101254916872982	6.54402421785885	6.87086163350033	0.00876123997267401	0.363169405775587
Kcng1	Kcng1	0.139853509055281	5.58011168699605	6.86434405911989	0.00879325196663827	0.363289419820972
Nr4a1	Nr4a1	-0.202068955191278	3.69456750571334	6.85378700800585	0.00884535868724322	0.364236106735095
Sgcz	Sgcz	0.379108330299752	0.125708851647342	6.83581269842035	0.00893480341133076	0.366709020273599
Trpv4	Trpv4	0.68157803219008	0.811568032981015	6.82790581387017	0.00897444275507022	0.367128289688561
Efcc1	Efcc1	0.369541066346948	1.02598486130487	6.81861133789558	0.00902126880656139	0.367827814630185
RGD156	RGD156307	-0.100002641978644	4.99037081557594	6.78820279672115	0.0091762243551391	0.372937300589291
Dmbx1	Dmbx1	-1.28274353012558	0.570904650767211	6.78222834570025	0.00920688784155419	0.372972686035947
Tspan7	Tspan7	-0.10447889817842	8.72152031784173	6.77285462586454	0.00925546777883112	0.37326511579511
Cd248	Cd248	0.276834596733763	4.30359123934778	6.76927712675146	0.00927403910368947	0.37326511579511
Ca8	Ca8	-0.21500778665422	3.68739825981823	6.75963308225287	0.00932429310271245	0.374081045152872
Per2	Per2	0.158807832652236	4.90352255494056	6.74880097512654	0.00938212229903011	0.375079275465558
Cgref1	Cgref1	-0.132249312530466	5.80108942118493	6.74200108776542	0.00941689373941145	0.375079275465558
Tal2	Tal2	0.317552967147264	2.19822281989774	6.73714531348462	0.00944256060159088	0.375079275465558
Fat4	Fat4	0.225765502198466	7.73640756051051	6.73207810731623	0.00946942147725021	0.375079275465558
Cpa3	Cpa3	0.880219625715104	2.29329621127553	6.72244757031811	0.00952068817915762	0.375816539276423
Sertad2	Sertad2	0.151482592623653	3.0459199785024	6.70761882446939	0.00960018282702373	0.376124686586772
Ncs1	Ncs1	0.0719081034849024	7.28292912025497	6.701053473904	0.00963559632833735	0.376124686586772
Cdk2ap1	Cdk2ap1	0.129961338897683	6.49668166375914	6.69665455987018	0.00965939856318292	0.376124686586772
Sh2d3c	Sh2d3c	-0.0792578611475057	5.59834099611106	6.68897185500584	0.00970111321670302	0.376124686586772
Lamc1	Lamc1	0.162542608829703	6.55845305486286	6.68882944948429	0.00970188872786135	0.376124686586772
Nfib	Nfib	0.178659972364925	6.09417423849552	6.68792127186826	0.00970683249822045	0.376124686586772
Nfatc3	Nfatc3	0.135462304104784	4.0946208649422	6.68103982343203	0.00974437638626465	0.376410347731091
Lcn2	Lcn2	0.542610124753118	0.182378234626251	6.66419852344543	0.00983688846004244	0.378811288801219
Kdm1a	Kdm1a	0.068566105378755	7.32232119316282	6.65587050909724	0.00988296777862672	0.379414735598771
Rem2	Rem2	-0.168757605756855	4.64557416743688	6.64168952303496	0.00996194143714254	0.380179254657204
Frem1	Frem1	0.395574147556823	2.12935102729946	6.626533723748	0.0100470591993077	0.380179254657204
Mafb	Mafb	0.134640567401641	5.27804075475831	6.60596292975669	0.0101637825582492	0.380179254657204
Pou3f2	Pou3f2	0.152067300185353	6.79315126357713	6.60472956008519	0.0101708249831437	0.380179254657204
Uck2	Uck2	0.0868004776565852	5.77511242147321	6.60059854803031	0.0101944491734854	0.380179254657204
Shprh	Shprh	0.125225587717557	6.59125327196581	6.59647265557651	0.010218100227085	0.380179254657204
Ywhah	Ywhah	-0.0887852920910322	9.67640038129297	6.58524512620101	0.0102825147705133	0.380179254657204
Ano6	Ano6	0.145438490030083	5.24002424145454	6.58512964552869	0.0102258109578773	0.380179254657204
Trip11	Trip11	0.146394332566191	5.0306454682428	6.58471085365747	0.0102282160464404	0.380179254657204

Adamts7	Adamts7	0.168656409097765	5.20616831129454	6.59098849797671	0.0102496244346417	0.380179254657204
Dnajc3	Dnajc3	0.124989278237933	4.30469603191223	6.58947788979323	0.0102583252623042	0.380179254657204
Rasd1	Rasd1	-0.249692537892174	1.97428619525719	6.58640695626112	0.0102760266309595	0.380179254657204
Rgs7	Rgs7	-0.073067204352405	6.31457866105452	6.58243394304727	0.0102989972007802	0.380179254657204
Rnd2	Rnd2	-0.125418327952232	6.12590886501649	6.55817498513152	0.0104403286794789	0.383445436297035
Atp6v0e1	Atp6v0e1	0.165136309934331	3.45039359369656	6.55570926954843	0.0104489419204129	0.383445436297035
[illegible]	[illegible]	[illegible]	[illegible]	[illegible]	[illegible]	[illegible]

**C1d**	C1d	-0.0745013066961761	5.94780293817942	5.07556471826433	0.0242653665811028	0.45044034032127
**Syngr2**	Syngr2	0.195831885519853	2.51483611688634	5.07334902958136	0.0242964133234123	0.45044034032127
**Atp6v1b**	Atp6v1b2	-0.0628311508912515	8.38612561776971	5.06989528753043	0.0243448542098116	0.4506687625754
**Serpinh1**	Serpinh1	0.223200170669505	6.50615001587692	5.06312275153761	0.024440162095739	0.451762818471905
**Lca5**	Lca5	0.129141606619146	3.78000648258023	5.05614221280915	0.0245388024493367	0.452678687916031
**S100a3**	S100a3	-0.401150169802972	-0.381488286282667	5.05379588094355	0.024572050617968	0.452678687916031
**Sx18**	Sx18	0.0979795758919856	5.2845019719663	5.04726388488663	0.0246648572822814	0.452678687916031
**Stmn4**	Stmn4	-0.0754075638375988	8.89608418812048	5.04717905159224	0.0246660607139174	0.452678687916031
**Ncoa2**	Ncoa2	0.119178373546183	6.11476972685286	5.04032576608456	0.0247638326881849	0.452678687916031
**Lpl**	Lpl	-0.16765884913502	5.66354011444577	5.04002344408535	0.0247681548101398	0.452678687916031
**Lama1**	Lama1	0.222583823032084	4.15497035399622	5.0393064603217	0.0247784082412625	0.452678687916031
**RGD130**	RGD130622	0.119070923762087	3.28613495440885	5.03919807456001	0.0247799586261401	0.452678687916031
**Arrdc2**	Arrdc2	0.164739265614277	3.10537239969599	5.03494170225629	0.0248408228405059	0.453128939007298
**Snrf5**	Snrf5	-0.107645800671784	7.72145931420773	5.03116313194128	0.024895173934391	0.453246878529718
**Bmt2**	Bmt2	0.105647298777716	3.82026738171356	5.02943386571752	0.0249200415702001	0.453246878529718
**RGD135**	RGD13586	-0.272554550440519	0.0127769074930536	5.02689477338139	0.0249566051707444	0.453251182991817
**Nr1d2**	Nr1d2	-0.1090434526827	4.93754290218125	5.02319818094794	0.0250099845722951	0.453559525666462
**Leng1**	Leng1	0.153692623928812	2.79691475797367	5.00678173073501	0.0252482021252012	0.457215961170008
**Nid2**	Nid2	0.267540444354566	5.40966655367923	4.99350219155588	0.0254426647235711	0.457399942586444
**Prrc1**	Prrc1	0.0914394276742795	4.2518440922239	4.9921494476987	0.0254625611046229	0.457399942586444
**Sh2b3**	Sh2b3	0.102544374068175	4.36622336937592	4.98545528732412	0.0255612580399378	0.457399942586444
**Ecm1**	Ecm1	0.179500126860296	2.79474008119408	4.9833129121919	0.0255929285173258	0.457399942586444
**Alyref**	Alyref	0.0986789474535061	6.3682497294378	4.98300967567619	0.02559741451833	0.457399942586444
**Cox6c**	Cox6c	-0.0936361759450236	6.99153851142979	4.98147629972752	0.0256201113877057	0.457399942586444
**Mex3b**	Mex3b	0.0912858627674616	6.86181993231583	4.98064496477095	0.0256324254579193	0.457399942586444
**Rfc4**	Rfc4	-0.0969063482186946	4.50584689267873	4.97867158948995	0.0256616805153271	0.457399942586444
**Jun**	Jun	-0.0889267528848131	6.27608518637197	4.97246742676118	0.0257538826381868	0.457399942586444
**Eef1a2**	Eef1a2	-0.0981138761732877	8.97244029291911	4.96882350422996	0.0258081965334203	0.457399942586444
**Ccdc85a**	Ccdc85a	-0.101369373740038	5.61691839312979	4.96721176906826	0.0258322579325414	0.457399942586444
**Ets2**	Ets2	0.0882235744962349	5.67839828885241	4.96718955641067	0.0258325897053705	0.457399942586444
**Slc15a3**	Slc15a3	-0.134948312626631	4.26246519276628	4.96687173076384	0.0258373372995246	0.457399942586444
**Zfp260**	Zfp260	0.081794248307408	6.06389535771632	4.96476765264392	0.0258687903258386	0.457399942586444
**Gnai1**	Gnai1	0.507379333397631	1.86555033249258	4.95512580877575	0.0260134300801201	0.457399942586444
**Qser1**	Qser1	0.152157455072438	5.5840266972959	4.9543711774443	0.0260247880031883	0.457399942586444
**Gabpa**	Gabpa	0.0766812226280877	5.48037628119841	4.95083501652895	0.0260787014004926	0.457399942586444
**Egr1**	Egr1	-0.258993040800074	3.97643596256767	4.94704763882781	0.0261353632055391	0.457399942586444
**Polb**	Polb	0.0768276329211285	5.49703367959035	4.94589427087225	0.026152770686904	0.457399942586444
**Atp1a3**	Atp1a3	-0.103618652016869	10.5814080530612	4.94545680559702	0.026159325197943	0.457399942586444
**Aplnr**	Aplnr	0.20505109814485	4.34500706579626	4.94472579928464	0.0261702282880541	0.457399942586444
**Slc7a1**	Slc7a1	0.0897849615496787	7.289047041764	4.94300755677395	0.0261964180841523	0.457399942586444
**Adap1**	Adap1	-0.0778828746579616	8.05973792057984	4.94281670703329	0.0261993085548095	0.457399942586444
**Zcchc12**	Zcchc12	-0.121735537997671	8.32296214999215	4.9419179173743	0.0262129949042563	0.457399942586444
**Ska1**	Ska1	-0.236085084048039	1.27402803614041	4.94166911974998	0.0262167081680619	0.457399942586444
**Tmem13**	Tmem130	-0.113836908803609	9.47888865263381	4.94107560819205	0.0262257116413962	0.457399942586444
**Sestd1**	Sestd1	0.0935785244152418	6.22072885031994	4.9384696464307	0.0262652816882154	0.457399942586444
**Tmem59**	Tmem59l	-0.0710333202970587	6.4489792712821	4.93718410378287	0.0262848247848214	0.457399942586444
**Lztr1**	Lztr1	-0.0575089077696756	6.82768739380139	4.93389840562546	0.0263348633518335	0.457620333865859
**Dlg1**	Dlg1	0.0993779307260723	4.86269222537022	4.92924347411489	0.0264058753195537	0.457620333865859
**C2cd3**	C2cd3	0.106145318981384	5.57068829632988	4.92912575954567	0.0264075213460691	0.457620333865859
**Tipin**	Tipin	-0.11451585629945	4.2555106749461	4.92250357592364	0.0265090759293721	0.458743051831867
**Pgap4**	Pgap4	-0.0786848104205486	6.61472370793332	4.91591939389917	0.0266102970895699	0.45985689271056
**Elavl1**	Elavl1	0.110616471252588	5.50416459720681	4.90135742363572	0.0268355932699118	0.463022899698099
**Tmem16**	Tmem169	-0.0669409416602551	5.4052996441633	4.89857548337673	0.0268788593121035	0.463022899698099
**Alg2**	Alg2	-0.0625005678821286	8.31083482247293	4.89675444079887	0.0269072202911502	0.463022899698099
**Ehbp1l1**	Ehbp1l1	-0.0711258311238054	6.13480017698659	4.89176704250225	0.0269850536053046	0.463022899698099
**Dse**	Dse	0.197578437607589	2.96850059805093	4.88586989888701	0.0270617062058591	0.463022899698099
**Kdr**	Kdr	0.163930927329728	5.60316752958896	4.88204324175533	0.0271374770774208	0.463022899698099
**Ncoa5**	Ncoa5	0.0625198790309685	7.16538242068874	4.88000030935532	0.0271696142974971	0.463022899698099
**Iffo1**	Iffo1	-0.0971780367327076	5.03854361946905	4.87535081780919	0.0272429027299096	0.463022899698099
**Rhobtb1**	Rhobtb1	0.0882139664952168	5.10956032571253	4.87522920431861	0.0272448224407506	0.463022899698099
**Cldn15**	Cldn15	0.433171665620224	-0.556249025943017	4.87309586757871	0.0272785192041748	0.463022899698099
**Cdk4**	Cdk4	0.0823750248125673	7.55590221853411	4.87244043963027	0.027288882675113	0.463022899698099
**Anapc1**	Anapc1	0.0814625675976496	7.54275163008755	4.8722599033576	0.0272917375553528	0.463022899698099
**Gstt3**	Gstt3	0.239959929466389	2.5965832371137	4.87223580128006	0.0272921171315854	0.463022899698099
**Rundc3b**	Rundc3b	-0.0542369359113646	6.76193188569799	4.87065311863739	0.0273171598659673	0.463022899698099
**Fgf9**	Fgf9	-0.185483815730587	2.71480893549913	4.86821685667366	0.0273557530343003	0.463022899698099
**Trim21**	Trim21	0.444971345364453	-0.206375647106477	4.8662302850687	0.0273873645649753	0.463022899698099
**Cisd2**	Cisd2	-0.0669861098892621	6.19141090206511	4.86290488708252	0.0274400974372205	0.463288356866306
**Thbd**	Thbd	0.249617565968365	3.21971292814725	4.85966849632302	0.0274916179861494	0.463520969747548
**Ybx1-ps**	Ybx1-ps3	-0.174313183404731	5.59551594788926	4.85338559751733	0.0275918237872573	0.464594376644548
**Ivd**	Ivd	-0.0869357567494335	5.44592340216789	4.84428528902929	0.0277378847758769	0.465823708697451
**Pign**	Pign	0.181454816795088	3.7333186625005	4.84031282201875	0.0278018513057961	0.465823708697451
**Erfe**	Erfe	-0.255527211558652	1.60387299395	4.83959105251388	0.0278134900430599	0.465823708697451
**Mitf**	Mitf	0.331657288491228	1.85480284628697	4.83677206097494	0.0278589956831312	0.465823708697451
**Ctbp2**	Ctbp2	0.0993699599188247	5.5677030780047	4.83624136423026	0.0278675711049634	0.465823708697451
**Whamm**	Whamm	-0.130405622355106	2.79387265050893	4.83491964544437	0.027888940482247	0.465823708697451
**Slc35c1**	Slc35c1	0.0655448007610758	5.76107867048647	4.83164490414047	0.0279418595573825	0.466085333419066
**Hecw1**	Hecw1	0.102449153757086	7.02449927380671	4.82574396824293	0.0280377624604855	0.466607262022389
**Atp5if1**	Atp5if1	-0.103433915183197	6.62848941435347	4.82511182103468	0.0280480641225288	0.466607262022389
**Alx4**	Alx4	0.60732182049465	-0.0157922128909878	4.8204872598068	0.0281223940614587	0.466755655054883
**Scaf8**	Scaf8	0.0656298077466433	6.93917211036508	4.81967006574754	0.0281318338277406	0.466755655054883
**Fli1**	Fli1	0.235004215534662	2.69383512557266	4.80013858604089	0.028457447771494	0.471520972296555
**Cpz**	Cpz	0.475257056809023	1.32803859386665	4.78713885564633	0.0286730173181427	0.472910717781319
**RT1-DO**	RT1-DOb	-0.455789487348977	1.02458456976595	4.78665299965896	0.0286809604349359	0.472910717781319
**Fam219a**	Fam219a	-0.0669044698888782	7.18244867510854	4.78322256856363	0.0287382922959163	0.472910717781319
**Fam241b**	Fam241b	-0.0952151418031782	5.32455769038003	4.78234511734816	0.0287528984756319	0.472910717781319
**Fubp1**	Fubp1	0.0888884480849117	7.40394478995217	4.778505022402	0.0288171277933465	0.474001635443797
**Usp25**	Usp25	0.109583976003255	5.52213348024328	4.77575316048503	0.0288632179104693	0.474001635443797
**Elovl6**	Elovl6	0.0956847493919006	5.50415408412707	4.77299185974607	0.0289095626672679	0.474001635443797
**Tecta**	Tecta	-0.376512523788509	0.568056045647785	4.77294003384276	0.0289104147289196	0.474001635443797
**Jup**	Jup	0.0908679595503784	7.07153422311294	4.76820365692674	0.0290265589843943	0.474057961828877
**Gpr139**	Gpr139	0.190136702674278	2.07237246510511	4.76583304826421	0.0290300074792061	0.474057961828877
**Zfp541**	Zfp541	-0.277580000930126	0.940865126584573	4.7649363059348	0.0290451339857555	0.474057961828877
**Lrrc34**	Lrrc34	-0.23077513497683	1.85117226785225	4.76349767803232	0.0290694183208878	0.474057961828877
**Fyco1**	Fyco1	0.156859796742185	3.13405913043657	4.75878200439552	0.0291491682147397	0.474057961828877
**Cspg5**	Cspg5	-0.0969662361641016	8.49409215928885	4.75546642916299	0.0292053765263284	0.474057961828877
**Ppp1r32**	Ppp1r32	-0.242393623631334	1.96498197578528	4.75404977784112	0.0292294271239692	0.474057961828877
**Lgi4**	Lgi4	-0.334707974900364	2.0221240340345	4.75398766577952	0.0292304820779556	0.474057961828877
**Pdha2**	Pdha2	0.157026702500844	3.51937982295103	4.75249766752611	0.0292558011227246	0.474057961828877
**Denr**	Denr	-0.0619115087419016	6.11174888090241	4.74990616790113	0.0292998920397849	0.474156618651617
**Yipf2**	Yipf2	0.104132055371773	3.45249972482577	4.74348323362893	0.0294094683131036	0.475313388785742
**Fzd1**	Fzd1	0.104070426329106	5.57922024126461	4.74062530969476	0.0294583620833296	0.475487589150974
**Hira**	Hira	0.0548898828475884	7.41102495070596	4.73485579436141	0.0295573259614313	0.475820339588977
**Arl6ip4**	Arl6ip4	-0.0832334315597052	5.19489939524549	4.73432561494837	0.0295662655469604	0.475820339588977
**Klhl25**	Klhl25	0.129965364412404	5.48345248426997	4.72878285201272	0.029661849734942	0.475820339588977
**LOC498**	LOC498675	-0.154220803870807	2.16508350418956	4.72841111073945	0.0296682805492024	0.475820339588977
**Wdr91**	Wdr91	-0.0782610028599447	5.05159163506275	4.72794970255573	0.0296762409880584	0.475820339588977
**Adamtsl**	Adamtsl4	0.282414888267609	1.77458340228501	4.72514285249034	0.0297248950865433	0.475975765082364
**Cd93**	Cd93	0.154818764349983	5.49650509342146	4.72335467853253	0.0297556281152716	0.475975765082364
**Heatr1**	Heatr1	0.0946515318935464	5.20706285678773	4.71761288941043	0.0298551612154749	0.476856289507657
**Kbtbd7**	Kbtbd7	0.0982159925366152	5.65207964962554	4.71328355556128	0.0299304315218279	0.477547307030494
**Ntsr1**	Ntsr1	0.155399363015126	6.24145443008745	4.70805162537569	0.030011181711179	0.478201514728777
**Slc50a1**	Slc50a1	0.192768595086255	2.20120771937208	4.70653888727172	0.0300480874847608	0.478201514728777
**Cblb**	Cblb	0.13720269675691	5.2095853694906	4.70275176351288	0.0301143651075217	0.47863821496301
**Rbms1**	Rbms1	0.123523098033914	4.02896321377187	4.69934589893697	0.0301740971814736	0.47863821496301
**Zfp707l1**	Zfp707l1	-0.147600504552462	2.651386629634	4.6970067189062	0.0302153434719999	0.47863821496301
**Nrn**	Nrn	0.118836242060232	3.17858766089469	4.69622323360494	0.030228974384135	0.47863821496301
**Prdm4**	Prdm4	0.0748607816128248	5.59178578421376	4.69372291568467	0.0302729881309706	0.478656115794247
**Calhm2**	Calhm2	0.319649072845308	0.881898614851892	4.69180294832246	0.0303068311507137	0.478656115794247
**Trpc3**	Trpc3	-0.155809708112432	4.65417630269453	4.68922640930818	0.0303523083952016	0.479768332562804
**Asb8**	Asb8	-0.0606046794425057	5.96026638845715	4.68081473618926	0.0305012791443116	0.480510681671181
**Ldb1**	Ldb1	0.0740800494942164	7.38155550550786	4.67137937634057	0.0306692855464881	0.481660859862542
**Zfp703**	Zfp703	0.0971172468969436	5.52538067235656	4.66818062116793	0.0307264612777177	0.481660859862542
**Tmem98**	Tmem98	0.173018806326601	2.4972108170103	4.66785224182685	0.0307323373352683	0.481660859862542
**Plvap**	Plvap	0.356759534851436	1.85454651550897	4.66540655188874	0.030776136330801	0.481660859862542
**Shc2**	Shc2	-0.0622562938867952	6.29231519701195	4.66520920820032	0.0307796723255737	0.481660859862542
**Stt3a**	Stt3a	0.0790790127560109	5.95703005166151	4.66374587144559	0.0308058121720212	0.481660859862542
**Acot4**	Acot4	-0.243253938034545	2.0443098076829	4.66035351647859	0.0308668356303464	0.482009396945841
**Uaca**	Uaca	0.144798898155961	4.56494085948678	4.65611446628468	0.030943129303229	0.482596936357985
**Rspo3**	Rspo3	0.17780588574157	2.71918973368654	4.65010654682761	0.0310516200414785	0.483125556618509
**Eif5**	Eif5	-0.0509489990515817	8.17395264957607	4.64981314565786	0.0310551187311088	0.483125556618509
**Cdk5r1**	Cdk5r1	0.0698060699914267	8.15291918249051	4.64528942640738	0.0311370716514072	0.483807276450547
**Slc3a1**	Slc3a1	-0.0888151334182903	5.91842002923078	4.64171489739206	0.0312028064168564	0.484166005509594
**Pigh**	Pigh	-0.121003365537074	3.22472150972001	4.63848736302499	0.0312625461722881	0.484166005509594
**Mtmr1**	Mtmr1	0.0711203642536396	5.96142305961235	4.63771757964055	0.0312765729294488	0.484166005509594
**Scp2**	Scp2	-0.0859637481433806	6.40435526708742	4.63297769746524	0.0313630865090767	0.484903632121895
**Map2k3**	Map2k3	0.0954290119654144	4.71005005018312	4.62824680514335	0.0314496846941407	0.485017559932985
**Cfap20d**	Cfap20dc	-0.148102751318337	3.02107113469772	4.62638342504547	0.0314838620369972	0.485017559932985
**Chp2**	Chp2	-0.400280844541581	-0.31845308870289	4.62124736558319	0.031578266232918	0.485017559932985
**Sugt1**	Sugt1	-0.0805152894990458	5.86798505312711	4.61798185927387	0.0316384419436465	0.485017559932985
**Tlr4**	Tlr4	0.499896060688619	-0.567527802118611	4.61620045404095	0.0316713195341344	0.485017559932985
**Ctsk**	Ctsk	0.327423330520302	3.15363573224877	4.61508970169064	0.0316807525466908	0.485017559932985
**Bgn**	Bgn	0.296944628175928	5.07282781734407	4.61379835344249	0.0317157091192765	0.485017559932985
**Dusp19**	Dusp19	0.11622811025356	3.56167570094745	4.61315380753626	0.031727629188736	0.485017559932985
**Klhl42**	Klhl42	0.104488710434987	4.07866373136451	4.61162124393351	0.0317559870630115	0.485017559932985
**Slc22a17**	Slc22a17	-0.0761614713805119	9.2262738355212	4.61144911559547	0.031759184618915	0.485017559932985
**Sp4**	Sp4	0.0864451017228296	5.33642582714267	4.60389205512236	0.0318994594317875	0.486564248570187
**Snph**	Snph	-0.0963697127039462	6.72078003829421	4.59885625372824	0.0319932932446275	0.487399657891596
**Ears2**	Ears2	0.165066880593783	2.36749240932309	4.59510065342486	0.0320634601155879	0.487872916905109
**Raver1**	Raver1	0.0622644681329018	6.55768340969781	4.58349159619038	0.0322813725366143	0.488060820764603
**Slc13a4**	Slc13a4	0.505899339666346	4.22209832167271	4.58314615192823	0.0322878804491637	0.488060820764603
**Cbwd1**	Cbwd1	0.112609044675001	3.08721696586049	4.58282500905661	0.0322939817874729	0.488060820764603
**Gdpd5**	Gdpd5	0.0769887079812226	6.53394201866986	4.58003021795997	0.0323466444183414	0.488060820764603
**Tmem13**	Tmem132d	-0.186431709875746	3.57386276016226	4.57773270464355	0.0323900451413579	0.488060820764603
**Zswim6**	Zswim6	0.101988893078109	5.1154929570945	4.57473451440746	0.0324467733043426	0.488060820764603
**Zfp217**	Zfp217	0.182889920821428	2.20657459460251	4.57396276298698	0.0324613922378471	0.488060820764603
**Ncbp1**	Ncbp1	0.0600954192730111	6.49251310947181	4.57383067265418	0.0324638950533589	0.488060820764603
**Ccdc86**	Ccdc86	0.0930935092065331	3.89865902096537	4.56209965036846	0.0326869770318645	0.488060820764603
**Zfp638**	Zfp638	0.0845361529699519	7.40348099822083	4.5614316418643	0.0326997282229706	0.488060820764603
**Dld**	Dld	-0.0528036524985174	6.88836494276063	4.56037900702311	0.0327198218434145	0.488060820764603
**Klhdc8a**	Klhdc8a	-0.102563650774791	5.20061195096954	4.56036064231064	0.0327201826941909	0.488060820764603
**Bmp1**	Bmp1	0.099354738060756	5.5451148796565	4.55606829191	0.0328017648705028	0.488060820764603
**Syncrip**	Syncrip	0.0672822382354674	6.70391906567321	4.5553406484352	0.0328162390320373	0.488060820764603
**Pcnx1**	Pcnx1	0.0897003250610132	7.9429316874763	4.55258424843363	0.0328689101297513	0.488060820764603
**RGD156**	RGD15617	0.772547540337785	-0.79076172478881	4.55104851613866	0.032888594291647	0.488060820764603
**Tpst2**	Tpst2	0.108131989140181	4.25088127048958	4.54883376311665	0.0329411762224382	0.488060820764603
**Laptm5**	Laptm5	0.282854436356715	2.5682475407348	4.5440963032203	0.0330325700759875	0.488060820764603
**Tfe3**	Tfe3	-0.0808180086695346	4.60458278019407	4.54230532463565	0.0330477196246549	0.488060820764603
**Atp6v1a**	Atp6v1a	-0.0589442513443894	8.75187409810398	4.53982776421187	0.0331129892817644	0.488060820764603
**Zbtb38**	Zbtb38	0.0885852728005829	5.90618314734676	4.53936234253781	0.0331239094988311	0.488060820764603
**Mycbpap**	Mycbpap	0.242712916127499	1.17781844786861	4.53848551412761	0.0331409008654714	0.488060820764603
**Ptk7**	Ptk7	0.0841076377990824	6.18969759186387	4.53658941858765	0.0331776330244034	0.488060820764603
**Ntan1**	Ntan1	-0.0745643754377171	5.67197381808983	4.53655089238915	0.0331783809778361	0.488060820764603
**G4**	G4	0.128803483500934	3.46659059644767	4.53588564346169	0.0331912777442976	0.488060820764603
**Nptn**	Nptn	-0.0607501081228316	8.25830156639006	4.53443284042586	0.0332194630813411	0.488060820764603
**Med13**	Med13	0.115104694705935	6.47888874589236	4.53425385259321	0.0332229372926281	0.488060820764603
**Mccc2**	Mccc2	-0.0746369962635937	4.91132420162385	4.53258335157183	0.0332553805594769	0.488060820764603
**Emp1**	Emp1	0.328191086371653	3.24463792979658	4.53245933547743	0.0332577904298519	0.488060820764603
**Vps37a**	Vps37a	0.0890811338672914	4.27792144928526	4.53202976478858	0.0332661292218329	0.488060820764603
**Akr7a3**	Akr7a3	-0.385238913902338	0.0607632697753094	4.52719295857048	0.0333602947538296	0.488060820764603
**Emp3**	Emp3	0.3535579864801	2.06559644382749	4.52670129540077	0.0333698812732107	0.488060820764603
**Klf10**	Klf10	0.0925385029655982	4.57985543623849	4.52405245780503	0.0334215780334666	0.488060820764603
**Fkbp10**	Fkbp10	0.146376318147813	4.15156416367274	4.52182643386735	0.033465087892176	0.488060820764603
**Tbrg4**	Tbrg4	-0.0731600862689776	5.4097164052272	4.52083421580573	0.0334845007579092	0.488060820764603
**Arhgap1**	Arhgap10	0.14756886261763	2.61877227947767	4.51799968858346	0.0335400185644463	0.488060820764603
**Pop1**	Pop1	0.118561490018589	3.75817676216567	4.51792421269046	0.033541503201103	0.488060820764603
**Smarca4**	Smarca4	0.0656402088300783	8.78271848137594	4.51686639739746	0.0335622492759501	0.488060820764603
**Aen**	Aen	0.0864185201513978	4.6861721353324	4.50572585522966	0.0337815555775833	0.490358421968347
**Rbx**	Rbx	0.188210574926268	2.73740642020643	4.50309791876307	0.0338216371889656	0.490358421968347
**Pi16**	Pi16	-0.125260044768146	4.13836401524056	4.50286316512135	0.0338381503017349	0.490358421968347
**Adgre1**	Adgre1	0.364011711066322	1.35798171347925	4.49465462972938	0.0340009811872915	0.49214645275387
**Rbm24**	Rbm24	0.134702027238095	4.17084865128789	4.48695096957344	0.0341545419214052	0.492990905898871
**Cpne3**	Cpne3	0.189557326641084	2.81496194537176	4.48581712401563	0.0341772045267869	0.492990905898871
**Gatm**	Gatm	-0.0936783837079837	6.72227705857902	4.48186994974525	0.0342562208835827	0.492990905898871
**Sdc1**	Sdc1	0.123348344659205	5.00802443977914	4.479565670783	0.0343024372557963	0.492990905898871
**Smim17**	Smim17	-0.115960414766107	3.29532213755771	4.47798867831614	0.0343341041789473	0.492990905898871
**Boc**	Boc	0.12271897032598	4.98169971904707	4.47144329083645	0.0344658663581358	0.492990905898871
**Ern1**	Ern1	0.161319264630653	3.20749515060007	4.46918584908703	0.0345114303626016	0.492990905898871
**Lgi3**	Lgi3	-0.176293289226605	3.9181461052536	4.46252232980982	0.0346463017228624	0.492990905898871
**Tesmin**	Tesmin	-0.358512303958199	0.354471178604878	4.46184421806012	0.0346600577572625	0.492990905898871
**Etnk2**	Etnk2	-0.131572116135089	3.45831819457908	4.46039981117266	0.0346893773034338	0.492990905898871
**Gabarap**	Gabarapl1	-0.071006677588596	7.92815601011869	4.45952467114761	0.0347071541012088	0.492990905898871
**Tsku**	Tsku	0.105989253572088	4.47372223415906	4.45891274156293	0.0347195899402811	0.492990905898871
**Psmd12**	Psmd12	-0.0600823775892485	6.49237057287717	4.4552913414552	0.0347932806893995	0.492990905898871
**Aplp2**	Aplp2	-0.0603724485437296	8.65062180182748	4.45437242967884	0.0348120053423306	0.492990905898871
**Zkscan5**	Zkscan5	-0.0629591470228346	5.11670856751019	4.45391964621365	0.0348212355798579	0.492990905898871
**RGD156**	RGD15656	0.0901967865575343	7.08798321747152	4.4518785235631	0.0348626767592248	0.492990905898871
**Lrrc14**	Lrrc14	0.0753512115459463	4.81171686817513	4.45152944002263	0.0348700036857467	0.492990905898871
**Cfap70**	Cfap70	-0.185210535944072	2.87205795889217	4.45130680182602	0.0348745478115662	0.492990905898871
**Zfp455**	Zfp455	0.223430493157804	1.32281062728866	4.44789613431242	0.0349442719272967	0.492990905898871
**Casp8ap**	Casp8ap2	0.103436381468631	4.81248871574638	4.44590817941453	0.0349849776341219	0.492990905898871
**Tmem10**	Tmem109	0.103599456589497	4.97814821989233	4.44399203354335	0.0350242599029225	0.492990905898871
**Eps8l2**	Eps8l2	0.301509477047808	1.49747344486657	4.44023664826152	0.0351013816822326	0.492990905898871
**Rem1**	Rem1	0.321563820330549	0.396454533024035	4.43946912625098	0.0351171655801617	0.492990905898871
**Aldoc**	Aldoc	-0.107543199551167	7.66673753218873	4.43906222620984	0.0351255363889199	0.492990905898871
**Colec12**	Colec12	0.444220394320364	4.28686084752039	4.43870578070901	0.0351328709729898	0.492990905898871
**Nrsn1**	Nrsn1	-0.0899992791716132	5.74538047799387	4.43848179449813	0.0351374807534499	0.492990905898871
**Apmap**	Apmap	-0.0742652986902905	5.12933005878231	4.43669820747697	0.0351742107070707	0.492990905898871
**Cchcr1**	Cchcr1	0.170276574523002	4.11445185350086	4.43625064752828	0.0351834337474476	0.492990905898871
**Dnajc16**	Dnajc16	-0.0781571583952281	4.66074471212563	4.43519638772105	0.0352051692840254	0.492990905898871
**Zfp68**	Zfp68	0.0895284998755642	6.62318922882955	4.43209819870304	0.0352567302115806	0.492158442656969
**Txnrd3**	Txnrd3	-0.0974518340501356	4.60505261829175	4.42515540282617	0.0354128882508741	0.493986001820005
**Hcrt**	Hcrt	-1.09741644923041	-1.14068343107209	4.42020698368003	0.0355157279710423	0.493986001820005
**Pcdhgc3**	Pcdhgc3	0.0744912221647703	7.67074730824801	4.41950091983341	0.035530427079466	0.493986001820005
**Kdm2b**	Kdm2b	0.0542651644931175	7.78426710388944	4.41785854965978	0.0355646431993753	0.493986001820005
**Zdhhc9**	Zdhhc9	0.0500733668860558	7.01490053890011	4.41309806287118	0.0356514745851822	0.493986001820005
**Ifrd1**	Ifrd1	0.0809660572282162	5.7910006621628	4.41314912515442	0.0356629684077973	0.493986001820005
**Map3k14**	Map3k14	0.0997083213701642	4.62127244284308	4.4107791704431	0.035712525635185	0.493986001820005
**LOC363**	LOC363337	-0.196595467105423	2.25812087227849	4.41035118150742	0.0357214865722754	0.493986001820005
**Fhod1**	Fhod1	0.194543428814943	2.55884089634026	4.40983896933226	0.0357322140102199	0.493986001820005
**Itgb2**	Itgb2	0.34112824008774	0.470882887818916	4.40914541973973	0.0357467446332881	0.493986001820005
**Ahnak**	Ahnak	0.265318595551199	4.5979912556468	4.40868209319578	0.0357564552763171	0.493986001820005
**Scn4a**	Scn4a	-0.279399585126708	0.45880091681359	4.40471871448484	0.0358396247475604	0.493986001820005
**Lcp1**	Lcp1	0.163791102950697	3.35157368873224	4.39721965008369	0.0359975721819292	0.493986001820005
**Rpl38**	Rpl38	-0.173287323770057	6.78830958366159	4.39657264566736	0.0360112328000033	0.493986001820005
**Nup205**	Nup205	0.103722052513124	6.02568559531106	4.39587022850945	0.0360260695236796	0.493986001820005
**Esm1**	Esm1	0.475110628803068	0.246361361186576	4.39401214857259	0.0360653474554826	0.493986001820005
**RT1-CE**	RT1-CE5	0.400631052367942	1.29702693688023	4.39089549376755	0.0361355694256292	0.493986001820005
**Scrg1**	Scrg1	-0.136071951917705	3.06112176770407	4.38867682520053	0.0361783798978569	0.493986001820005
**LOC498**	LOC498122	-0.123364243371859	4.02864057764875	4.38775290259223	0.036197991707677	0.493986001820005
**Pear1**	Pear1	0.175688567391575	4.00889862031467	4.38767796658604	0.0361995828208872	0.493986001820005
**Prkar1b**	Prkar1b	-0.0760825614324085	9.36882381252293	4.38722011283844	0.0362093060013692	0.493986001820005
**Mapre3**	Mapre3	-0.0867772814757435	7.17375560942803	4.38678543694922	0.0362185284962376	0.493986001820005
**Ptprn**	Ptprn	-0.0779831592760326	7.4948465319156	4.38641398298344	0.0362264219680456	0.493986001820005
**Bex2**	Bex2	-0.114703469520115	7.57702727540151	4.38246536827803	0.0363104415666824	0.494078078455556
**Aldh1a2**	Aldh1a2	0.4580617835351	4.1899722522668	4.38072687759676	0.036347493832768	0.494078078455556
**Osbpl2**	Osbpl2	0.0655236155561222	6.56484998606726	4.37930669082741	0.0363777914933202	0.494078078455556
**C1ql2**	C1ql2	-0.395021515707672	2.30061990045531	4.37616698358946	0.0364448665415238	0.494078078455556
**Mfap4**	Mfap4	0.183777739700981	5.05989710474322	4.37571317604827	0.0364545721497811	0.494078078455556
**Zfyve1**	Zfyve1	0.0650141641324541	5.50927647133618	4.37115249725196	0.0365522620350412	0.494078078455556
**LOC100**	LOC100364	0.446849604124302	-0.477400631746847	4.36985659972466	0.0365800701752838	0.494078078455556
**Pik3cg**	Pik3cg	0.222380150149761	2.40995381916385	4.3692912588003	0.0365922085356862	0.494078078455556
**Lad1**	Lad1	-0.334464953522436	0.212978028166084	4.36766672428296	0.0366271121774555	0.494078078455556
**Prodh2**	Prodh2	-0.265160174142393	0.274397875001358	4.36717995070357	0.0366375774487824	0.494078078455556
**App**	App	-0.0792036395689662	9.92737010076545	4.36572973392063	0.0366687745972465	0.494078078455556
**Mbtps1**	Mbtps1	0.0525842707552567	6.73752230702182	4.36291926575127	0.0367293243212374	0.49436019884151
**Tmem10**	Tmem100	-0.133055585616127	3.67714421149065	4.34923682034318	0.0370255323804389	0.496823315407602
**Nit2**	Nit2	0.169354693208273	3.94892406475901	4.34849612303541	0.0370416414774065	0.496823315407602
**Zdhhc13**	Zdhhc13	0.0965209542936234	5.03638875942623	4.34385434398927	0.0371427486683661	0.496823315407602
**Elavl3**	Elavl3	0.073812910597621	8.20716166469833	4.33919455859983	0.0372447573892144	0.496823315407602
[illegible]	[illegible]	[illegible]	[illegible]	[illegible]	[illegible]	[illegible]

Piga	Piga	0.185557485544407	1.70831345967692	4.17444357127056	0.0410380360196039	0.506821706284042
Tmem19	Tmem199	-0.0924763576997285	4.9107470469211	4.17269143783536	0.0410804925867632	0.506821706284042
Aff4	Aff4	0.0830422432011873	8.55025321915274	4.16829120730772	0.0411873196637117	0.506821706284042
Fgd2	Fgd2	0.258525926388172	0.954753945095183	4.16825048254267	0.0411883097252158	0.506821706284042
Adam10	Adam10	0.0625953715082316	7.05846117682217	4.16478406075153	0.0412726742271872	0.506821706284042
RT1-A1	RT1-A1	-0.417916910910132	0.928117417891992	4.16221028577988	0.0413354303718789	0.506821706284042
Ramp3	Ramp3	-0.195714826033832	2.16065930036628	4.16215560017335	0.0413367648638775	0.506821706284042
Srrm4	Srrm4	0.101388776626384	6.31697627218517	4.16064047426698	0.0413737564604249	0.506821706284042
Gspt2	Gspt2	-0.0929963906545721	4.48382148034683	4.15877889125264	0.0414192543767945	0.506821706284042
Rb1	Rb1	0.0924707768167635	6.09128764064801	4.15806609662009	0.0414366893070095	0.506821706284042
Gbe1	Gbe1	-0.0963214052798803	5.10998276970228	4.15801218333061	0.0414380083381362	0.506821706284042
Tmem13	Tmem135	-0.0568718128068085	5.65658725238783	4.15788734441528	0.0414410627883083	0.506821706284042
Msi1	Msi1	0.0814751401887254	6.00508233629098	4.15296577635828	0.0415616681246001	0.507823070246659
Fxyd7	Fxyd7	-0.191239065615637	5.38110094801107	4.15156137833223	0.0415960511414615	0.507823070246659
Galnt6	Galnt6	-0.129161611915046	3.51038638378219	4.14815666715353	0.0416798738597677	0.508273748247924
Dctn4	Dctn4	0.0518239483979513	6.34427144010393	4.14575281413089	0.0417390918400993	0.508273748247924
Kcnh2	Kcnh2	-0.105809671143645	5.8477446858782	4.14315414788608	0.0418032084200228	0.508273748247924
Mdc1	Mdc1	0.110574967422733	4.69144456939178	4.13685182037603	0.0419591327457865	0.508273748247924
Fmo5	Fmo5	0.295365061098547	0.576547917699189	4.13591981325902	0.0419822434929505	0.508273748247924
Cd34	Cd34	0.158851055763807	3.2476031269078	4.13588107434632	0.0419832068620396	0.508273748247924
Patz1	Patz1	0.0508724876070751	6.41306120468783	4.13415175183428	0.0420261249197119	0.508273748247924
Sec14l2	Sec14l2	-0.117164624078872	3.91420371958994	4.13143882052008	0.0420935467094497	0.508273748247924
Tmem20	Tmem204	0.183623961837942	2.51089242583609	4.13090773185182	0.0421067589263451	0.508273748247924
Dus3l	Dus3l	-0.0902608902079947	5.38945664100092	4.13050674785124	0.0421167371497501	0.508273748247924
Tnrc6b	Tnrc6b	0.121780332800349	7.08040884917169	4.12982160820897	0.0421313024249043	0.508273748247924
Slc35g2	Slc35g2	-0.208678444698376	2.74229650581877	4.12988464850838	0.0421621102090487	0.508273748247924
Neu1	Neu1	-0.115837450458759	4.21824348572899	4.12866315957034	0.0421626456228742	0.508273748247924
Acox3	Acox3	0.108583719521751	4.70636658307439	4.12368239936207	0.0422869381866764	0.508626370011217
Arpc1b	Arpc1b	0.174485592234807	4.35568223475343	4.12316855712808	0.0422997827789811	0.508626370011217
Apoc4	Apoc4	-0.432429732933425	-0.628456662729369	4.12244825185564	0.0423177952632553	0.508626370011217
Serpini1	Serpini1	-0.0818091431030015	7.15162330513205	4.11974734228482	0.0423854105324089	0.508626370011217
Llgl2	Llgl2	0.419859239171352	0.451912179211517	4.11917008838296	0.0423998733379062	0.508626370011217
Tinagl1	Tinagl1	0.298596605982741	2.44710887781828	4.11687992309972	0.0424573133700699	0.508626370011217
Nepro	Nepro	0.140659362663344	2.52629140612003	4.11476241539312	0.042510495734259	0.508626370011217
Zbtb11	Zbtb11	0.0909462527530402	5.56492435877285	4.11171955786214	0.0425870414248087	0.508626370011217
Atp6v0a	Atp6v0a1	-0.0712635150740984	8.93830554922982	4.11144437778331	0.0425939709567428	0.508626370011217
Zdhhc4	Zdhhc4	0.108949035307484	3.94778205303441	4.11118970072499	0.0426003852417826	0.508626370011217
Adarb1	Adarb1	-0.0725612371254677	7.32862917489373	4.10625957960799	0.0427247553750621	0.509031647012838
Rab6a	Rab6a	-0.0531349643442646	9.04073206235023	4.10286155361915	0.0428106981777401	0.509031647012838
Mrfap1	Mrfap1	-0.0601741698874896	8.51275026032231	4.10230701262727	0.0428247408612907	0.509031647012838
Gimap9	Gimap9	0.352590986522591	0.0978056475177948	4.09985265816961	0.0428869290189558	0.509031647012838
Tns1	Tns1	0.150735949168444	4.28077310373656	4.09961652831349	0.0428929409894281	0.509031647012838
Apip	Apip	0.116415790333778	3.16688964882827	4.09929962895607	0.0429009812703753	0.509031647012838
Cox7b	Cox7b	-0.100919308578526	5.50352195914142	4.09642727991337	0.0429739320714937	0.509031647012838
Zmym4	Zmym4	0.0641663102118013	7.03580002412226	4.0959140258815	0.0429869811925733	0.509031647012838
Mmp2	Mmp2	0.272355306150517	4.90549568729632	4.09356134832797	0.0430468522536727	0.509031647012838
Kctd11	Kctd11	0.330273549714114	-0.200596655958523	4.09207962993492	0.0430846024970568	0.509031647012838
Hoxb3	Hoxb3	-1.64332644665321	-0.310703111241681	4.08990404395779	0.0431400911914962	0.509031647012838
Atl1	Atl1	-0.0525046268012401	7.08979563195254	4.08943772882361	0.0431520074750354	0.509031647012838
Lbhd2	Lbhd2	-0.352742218642948	1.22772507099729	4.08803839071494	0.0431877370519574	0.509031647012838
Osbpl8	Osbpl8	0.0769093187926386	6.96582399867492	4.08597030541117	0.0432406173968173	0.509031647012838
Msh3	Msh3	0.0969415290722861	4.25491665967007	4.08578122361772	0.0432454553045423	0.509031647012838
Nelfb	Nelfb	0.0587201674233974	6.91260822768741	4.08409977280145	0.0432885050324585	0.509058131281795
Antxr1	Antxr1	0.127090868818986	4.79698968982753	4.08133474028905	0.0433593095024206	0.509128548247451
Tmem25	Tmem255b	-0.197877184741955	1.77967416110047	4.0806837154381	0.0433761037739072	0.509128548247451
Cd163	Cd163	0.634511360356556	-0.288404500337446	4.07779516029848	0.0434503211512006	0.509620354326626
Igflr1	Igflr1	0.243817638618533	0.447281723862439	4.0684034050583	0.0436656943218533	0.510846093286874
Diras2	Diras2	-0.0908394112696836	5.72155561401687	4.06788818980955	0.0437058803277879	0.510846093286874
Aqp1	Aqp1	0.523648344849177	3.71916456371504	4.06371506368052	0.0438140070571533	0.510846093286874
Prr5l	Prr5l	0.206649273064142	1.21304777905964	4.0634934440246	0.0438197568822503	0.510846093286874
Tmem18	Tmem185b	-0.0831109356978901	4.58945545228992	4.06300757573844	0.0438325425797163	0.510846093286874
Stk10	Stk10	0.122862809609304	3.17801929690397	4.06183256750239	0.0438628737275535	0.510846093286874
Cbll1	Cbll1	0.0725823618813623	5.56980128742329	4.05950489108799	0.0439233758832532	0.510846093286874
Pigp	Pigp	-0.100782246960413	3.42920606826955	4.05929211381465	0.0439291710379305	0.510846093286874
Smad4	Smad4	0.0621756728221119	6.66201430689757	4.0591750554085	0.0439319406745865	0.510846093286874
Emilin1	Emilin1	0.172532790859796	4.47195279452657	4.05623940117082	0.0440084283150222	0.511259925592674
Mlec	Mlec	0.085686501855185	5.4824529775969	4.0536555406888	0.0440758031830506	0.51147319468001
Cpne7	Cpne7	-0.176254855557609	4.89569722536611	4.0520584819334	0.0441175175436118	0.51147319468001
Arl6ip6	Arl6ip6	0.103975671542164	3.99234161761124	4.05082490623987	0.0441497660162144	0.51147319468001
Lyst	Lyst	0.143931517963733	5.70987032484306	4.0487304753412	0.0442045763556416	0.511512517491115
Fzd7	Fzd7	0.202089503542144	2.75991826547706	4.04756325059242	0.0442351531917058	0.511512517491115
Slc45a1	Slc45a1	-0.0660303938073666	6.12238970933159	4.04331262351681	0.0443465707441577	0.512327656248663
Mtm1	Mtm1	0.0852260202252212	4.27838932430474	4.0406251924281	0.0444173638708897	0.512669240532619
Calml4	Calml4	0.425563886340921	1.41144843263843	4.03483017411392	0.0445703503001586	0.513520251695515
Scamp1	Scamp1	-0.0538858078586772	8.16451890975789	4.03424996834798	0.0445858487186007	0.513520251695515
Gpr3	Gpr3	-0.388277085155838	-0.517653888624877	4.0331170867957	0.0446154258299604	0.513520251695515
Hoxa3	Hoxa3	-1.81796496721047	-0.537478732712223	4.03152862844176	0.0446574601287592	0.513540212006017
Stra6	Stra6	0.270806096441863	3.11518984471994	4.02158468625149	0.044921486211983	0.516100721426412
Fam214b	Fam214b	0.0857656183082806	5.67024705681369	4.01492350854704	0.0450992690909274	0.517373795475261
Psma1	Psma1	-0.0908173719787815	6.76897825465148	4.01219930042483	0.0451454077969975	0.517373795475261
Entpd1	Entpd1	0.116144969870583	4.41016859798929	4.01277782861194	0.0451568933776195	0.517373795475261
Rtkn	Rtkn	-0.127468389053482	3.19884378850093	4.01018282547657	0.0452262168832187	0.517487054151237
Cd68	Cd68	0.273912617221631	0.785137209244123	4.00931508238543	0.0452495292200869	0.517487054151237
Apoo	Apoo	-0.0861679643773395	4.34325182918694	4.00655251801301	0.0453237371803129	0.527608610120648
Nrbf2	Nrbf2	0.0945628581249401	3.73188097960554	4.00396247778003	0.0453934272327558	0.527608610120648
Zfp9	Zfp9	0.110514812111552	5.16289635267018	4.00306667494034	0.0454175568067957	0.527608610120648
Zc3h10	Zc3h10	0.0953212147066157	4.06559691958141	4.00103851966396	0.0454722376525489	0.527608610120648
Fat1	Fat1	0.169484663405929	7.72580984757591	4.0006580382535	0.0454824765237355	0.527608610120648
Mboat2	Mboat2	0.060684974770572	6.00279452594	3.99943710564515	0.0455154621748038	0.527608610120648
Tti1	Tti1	0.081801185414816	4.905070843751	3.99697076459875	0.0455821170345066	0.527608610120648
L2hgdh	L2hgdh	0.168232674282265	3.31136144125224	3.99588384030189	0.0456115246797594	0.527608610120648
RGD130	RGD130746	-0.119125837608645	3.57020380508057	3.99507122780377	0.0456335233736245	0.527608610120648
Susd4	Susd4	-0.0927515659249682	5.82005566709624	3.9935107853921	0.0456757966428671	0.527617571213036
Lap3	Lap3	0.0685550835208754	5.16812857812879	3.99006874301092	0.0457693968911319	0.527812524227846
Ankrd13	Ankrd13a	0.0681049879432363	6.37277358184534	3.98761004385544	0.0458305949603062	0.527812524227846
Parvg	Parvg	0.306581729591784	0.319016663362764	3.98688636413272	0.0458530061705167	0.527812524227846
Pcdha10	Pcdha10	-0.116023496637954	3.65163702229072	3.98640967589349	0.0458687041594016	0.527812524227846
Dchs1	Dchs1	0.123515034733006	7.98517469585329	3.98441805830254	0.045922961400982	0.527812524227846
Ablim3	Ablim3	-0.110332199697966	7.45634543447105	3.98371941410379	0.0459420104448366	0.527812524227846
Srgap1	Srgap1	0.140973948826227	5.98487381802952	3.97983428581688	0.0460453598876727	0.528384388637816
Fchsd1	Fchsd1	-0.0970242997981222	5.2730147058943	3.97642534201765	0.0461413868298626	0.528384388637816
Chst7	Chst7	-0.127258135191345	3.95660497365213	3.97406384468	0.0462061310450733	0.528384388637816
Ophn1	Ophn1	0.0799821366425816	5.22787445018977	3.97337034701567	0.0462253626273208	0.528384388637816
Col6a2	Col6a2	0.263474009559531	5.51432467756464	3.97129945501879	0.046282043081635	0.528384388637816
Gpc2	Gpc2	0.136535666090616	7.74859499304545	3.97121729462044	0.0462843015477999	0.528384388637816
Chchd5	Chchd5	0.138654107570607	3.19844541039617	3.97078777962108	0.0462961084505232	0.528384388637816
Mark1	Mark1	0.0568569915172035	7.7659101030102	3.96970690490797	0.0463258252658852	0.528384388637816
Prkci	Prkci	0.0469450400058137	6.69757605697053	3.96758577989204	0.0463842201179857	0.528384388637816
Anxa6	Anxa6	0.0891401279463502	6.12717426022896	3.96544227083912	0.0464433200307972	0.528384388637816
Mybpc2	Mybpc2	0.245562047790775	1.37768395286678	3.96479822084962	0.0464610899646328	0.528384388637816
Col4a1	Col4a1	0.130080381728226	8.01841443738527	3.96370336823452	0.046491314489518	0.528384388637816
Tgm2	Tgm2	0.251208360273364	4.54678970587099	3.9598244775128	0.0465985620258069	0.528990396789488
Adcy6	Adcy6	0.10010578232855	6.34998042475217	3.95737386269562	0.0466664535430639	0.528990396789488
Lama4	Lama4	0.121477784790259	5.89742881859012	3.95636318745119	0.0466944835244085	0.528990396789488
Asf1a	Asf1a	0.0807057626506412	4.81728829230646	3.95362492939123	0.0467705153899934	0.528990396789488
Atp13a2	Atp13a2	-0.0541614477044676	8.90489651907517	3.95195526041593	0.0468169402858458	0.528990396789488
Acsm5	Acsm5	0.61191182008582	-0.326496827441821	3.95150191898369	0.0468295538227232	0.528990396789488
Pp2d1	Pp2d1	-0.102952017913019	4.06657046589473	3.95112564575493	0.0468400462735421	0.528990396789488
Zbtb9	Zbtb9	0.107748080611942	3.86638712228241	3.94422035727188	0.0470326430096491	0.528990396789488
Clptm1l	Clptm1l	0.0492868321320141	7.21924129881641	3.94366660041849	0.0470482140566572	0.528990396789488
Tmem22	Tmem220	0.227401773269205	-0.317521286014415	3.94325642076188	0.0470595975880683	0.528990396789488
Zbtb12	Zbtb12	0.0901305178459804	5.64584564416425	3.9399588103082	0.0471519319916537	0.528990396789488
Cd4	Cd4	0.310095592476007	1.38703053408898	3.93758972399964	0.047218385008436	0.528990396789488
Auh	Auh	-0.0879538537359042	5.27093817937559	3.93698113648917	0.0472354722175821	0.528990396789488
Plxdc1	Plxdc1	0.162559854181834	2.09868879727787	3.93589182057269	0.0472660723892502	0.528990396789488
Eva1a	Eva1a	0.218837712576198	1.55037093162646	3.93468480086123	0.0473000037234561	0.528990396789488
Txnl4a	Txnl4a	-0.0954933880094005	4.39761012662087	3.93275146798718	0.0473544062602812	0.528990396789488
Cpxm2	Cpxm2	0.364379513618984	2.61578508825462	3.93180474030545	0.0473810705300808	0.528990396789488
Akap9	Akap9	0.119517908762316	8.04259642343285	3.93058583670672	0.047415446421712	0.528990396789488
Tbc1d20	Tbc1d20	0.0681579237071141	4.85372032862773	3.92911184574105	0.0474570016317928	0.528990396789488
Abhd17c	Abhd17c	0.0564411778188261	7.00869493223503	3.92867701336191	0.0474692745595541	0.528990396789488
Mpped1	Mpped1	0.164866964203214	9.4373679235546	3.92699794888688	0.0475166967525529	0.528990396789488
Ankrd29	Ankrd29	-0.151921586181186	3.51969022068406	3.92286434748009	0.0476336560645484	0.528990396789488
Zfand3	Zfand3	0.0561609090159324	6.72840165159506	3.92100552582424	0.0476863496718499	0.528990396789488
Hoxb5	Hoxb5	-2.2793837096758	1.25727159936887	3.92098039490767	0.0476870627881061	0.528990396789488
Ugdh	Ugdh	0.0687953925326321	5.73809277234822	3.91970805632061	0.0477231670726861	0.528990396789488
Pak4	Pak4	0.0980580000587837	4.1384594540506	3.91675496492917	0.0478070761332693	0.528990396789488
Gfap	Gfap	-0.168208823077702	5.99287221256998	3.91621142087607	0.0478225373230348	0.528990396789488
Sox18	Sox18	0.155041034246154	3.94675179481352	3.91578280370388	0.0478347331283782	0.528990396789488
Tmem17	Tmem179	-0.0640237949786331	5.86607325447977	3.91534005814406	0.0478473243862576	0.528990396789488
Rasgrp4	Rasgrp4	0.274185335345101	0.650123785221985	3.91432150761146	0.0478763272881456	0.528990396789488
Chrna5	Chrna5	-0.118797834324363	3.178481908153	3.91309878289385	0.0479111784951796	0.528990396789488
Zfp710	Zfp710	0.0894173027995642	4.12642934986951	3.91308971059952	0.0479114370994819	0.528990396789488
Dapk1	Dapk1	0.0586933996023857	7.32479995411405	3.91283942548261	0.0479186007518104	0.528990396789488
Lrrc14b	Lrrc14b	0.168188153831394	1.91115492024646	3.90313381071558	0.0481961270766839	0.521212687228475
Bmi1	Bmi1	0.0772442968187042	5.74546407212012	3.90275284361063	0.0482070562684668	0.521212687228475
Phf20	Phf20	0.0575810898222739	6.769835103079	3.90005338721215	0.0482845732947254	0.521598880609124
Sqstm1	Sqstm1	-0.0786762451674766	8.52798137362191	3.8973456081964	0.0483624614863289	0.521988262945438
Katnal1	Katnal1	0.0837500604405304	4.4202780443343	3.89524206475507	0.0484230606008236	0.522042808380823
Mmp23	Mmp23	0.292903047525446	0.594530100415175	3.8942662616368	0.0484511959692608	0.522042808380823
Hif1a	Hif1a	0.0551175726221072	7.37821666865177	3.88848362457773	0.0486183015234739	0.522811339942172
Satb1	Satb1	0.131275506863609	6.96323725886576	3.88837937477257	0.0486213196394188	0.522811339942172
Lppos	Lppos	0.300017656828463	0.108800542519723	3.88658713407495	0.0486732237935301	0.522811339942172
Cdh18	Cdh18	-0.115990912189402	4.41671980629609	3.88285651666118	0.048781493972229	0.522811339942172
Vcpip1	Vcpip1	0.0745718825010947	6.00914470742017	3.87923109244215	0.0488869615900465	0.522811339942172
Tmem17	Tmem179b	0.137193708501392	2.34284756439875	3.87875944708813	0.0489006773560174	0.522811339942172
Hpgds	Hpgds	0.383814312995066	-0.403073236896657	3.87772319482156	0.0489306705286893	0.522811339942172
Prpf18	Prpf18	0.0688166678862003	6.26199968380869	3.87643952294359	0.0489683000404323	0.522811339942172
Marf1	Marf1	0.085040394106919	7.14102137547908	3.87613642748334	0.0489771618624534	0.522811339942172
Ca5b	Ca5b	-0.30399643526015	-0.046498071393033	3.87501973757203	0.0490097335780922	0.522811339942172
Zfp148	Zfp148	0.0611421712106446	6.32894380242079	3.87491540840277	0.0490127796718149	0.522811339942172
Sorbs2	Sorbs2	0.105640045691469	6.6133209274263	3.87281961410166	0.0490740128401563	0.522811339942172
Pcp4	Pcp4	-0.168519643627672	6.19551374311056	3.87214706525738	0.0490936799275759	0.522811339942172
Cemip2	Cemip2	0.105449147674991	6.07328002165271	3.87001073618336	0.0491562070061557	0.522811339942172
Arpc5	Arpc5	-0.0545103889649896	7.70067873921894	3.86934332727047	0.0492050679428409	0.522811339942172
Hip1r	Hip1r	-0.0597363341085406	6.51610726031126	3.86685935767252	0.0492485966005266	0.522811339942172
Elovl4	Elovl4	-0.0658500560629614	5.81708821972144	3.86470185265551	0.0493119543470867	0.522811339942172
Scoc	Scoc	-0.0707659347984734	7.38645910962288	3.86215719014086	0.0493864980646529	0.522811339942172
Lrrc32	Lrrc32	0.269818253854604	2.48085688331817	3.86177069316316	0.0493981696449525	0.522811339942172
Ctu1	Ctu1	0.0923764793400591	5.1041760076403	3.86174806352138	0.0493988055814094	0.522811339942172
Ptprk	Ptprk	0.105080768377968	7.22808687327877	3.86116330446023	0.0494160545468061	0.522811339942172
Dgki	Dgki	0.0959734897365596	5.52058221598807	3.86020223804404	0.0494443681279909	0.522811339942172
Htr2a	Htr2a	0.148089982506196	2.88497586358012	3.85436276710888	0.0496167711426661	0.523065112276286
Tcerg1l	Tcerg1l	-0.112823819388676	4.6508555945192	3.85400925208693	0.0496272581760984	0.523065112276286
Il23a	Il23a	-0.281208620861649	0.0883585616106642	3.85397717386163	0.0496281776236224	0.523065112276286
Tmco6	Tmco6	0.100627148175497	3.55290323902922	3.85371084177548	0.0496360577811271	0.523065112276286
Jakmip2	Jakmip2	0.065801905334785	6.66959601375854	3.85162127780876	0.0496979290928096	0.523275157207582
Zfp362	Zfp362	0.0626677595734878	5.7285769582035	3.84247183474392	0.0499698021063543	0.525279446817286
Rell2	Rell2	-0.0626330729986488	6.68044785877104	3.84115780094675	0.0500089770980605	0.525279446817286
Cav1	Cav1	0.157782956229988	4.89434295956773	3.84096977042789	0.0500145854627664	0.525279446817286
Add2	Add2	0.068429647500448	8.29689764556949	3.83663389927514	0.0501440955233738	0.52582417057353
Rab26	Rab26	-0.107426992856047	4.60037458088653	3.83539336134123	0.0501812148662976	0.52582417057353
Rnf216	Rnf216	0.0500882068017564	6.3215085936805	3.83467026572702	0.050202864708463	0.52582417057353
Htr7	Htr7	-0.196156143863387	2.57648435227058	3.83299036606856	0.050253199851915	0.52582417057353
Sod1	Sod1	-0.0969740767537671	6.56924913462816	3.82906479142309	0.0503710306225914	0.52582417057353
Socs4	Socs4	0.129095432071394	2.82629479969685	3.82795967165774	0.0504043457239469	0.52582417057353
Wasf2	Wasf2	0.126002605790856	3.32783609021675	3.82387359814041	0.0505272990122195	0.52582417057353
Ppp3r1	Ppp3r1	-0.0512071846874467	8.48869511765123	3.82317131020047	0.0505484790195499	0.52582417057353
Tank	Tank	-0.127074119112568	3.61013630147043	3.82221559506742	0.0505773171075772	0.52582417057353
Tlr3	Tlr3	0.326186094072063	0.595170317232995	3.82084784292175	0.0506186184037906	0.52582417057353
Plxnd1	Plxnd1	0.0778967212533109	3.9220004923526	3.81930003524092	0.050665399746367	0.52582417057353
Hmgn1	Hmgn1	0.0731793994034724	6.88460424065784	3.81719429632151	0.0507291175760359	0.52582417057353
Pnpt1	Pnpt1	0.10126652061563	4.58127143431914	3.81542040937793	0.0507828595356963	0.52582417057353
Nras	Nras	0.0589007245567633	6.18449792148798	3.81452857177338	0.0508098686138833	0.52582417057353
Enah	Enah	0.0929988878850836	6.36234919707804	3.81337277836275	0.0508449284192469	0.52582417057353
Tpsb2	Tpsb2	0.730015198902981	0.431472586226273	3.81335442702743	0.0508455267806083	0.52582417057353
Clic4	Clic4	0.124654170622048	4.42755277609274	3.81041837021399	0.0509347268050064	0.52582417057353
Rest	Rest	0.156554463657885	2.83445252907511	3.81031415601893	0.0509381448520562	0.52582417057353
Phrf1	Phrf1	0.0611647713596332	6.69094854034047	3.80876920543223	0.0509849021413823	0.52582417057353
Zfp652	Zfp652	0.112549152354556	4.64645297065386	3.80871855122095	0.0509864441134759	0.52582417057353
Adamts3	Adamts3	0.11288125234285	5.54271398545694	3.8083011321785	0.0509991527037814	0.52582417057353
Itga1	Itga1	0.267931205625414	2.19081685844715	3.80777289539655	0.0510152095526614	0.52582417057353
Pnck	Pnck	-0.127443025197354	6.09342182254338	3.80709964570688	0.0510357514277807	0.52582417057353
Ankib1	Ankib1	0.0585711513947289	6.6739808182098	3.80447580135184	0.0511157704129317	0.526080817174139
Pi4k2a	Pi4k2a	-0.0698135173087526	5.03330659287245	3.80349950851281	0.0511455823140363	0.526080817174139
Col13a1	Col13a1	0.410200664365203	2.26240436875445	3.79773392308928	0.051321996403236	0.526430253134836
Slc22a12	Slc22a12	-0.148560188713153	2.63002197055366	3.79640012664996	0.0513628991877006	0.526430253134836
Nin	Nin	0.156488116809442	5.87018546277187	3.79529817027655	0.0513967884404093	0.526430253134836
Ptgr2	Ptgr2	-0.0812032444412615	4.97048615308599	3.79476882079269	0.0514129690022032	0.526430253134836
Sec24b	Sec24b	0.0603214184778893	6.58256498886292	3.79105943188817	0.0515267171732061	0.526430253134836
Gtpbp6	Gtpbp6	-0.0722316168308859	5.86576029799056	3.78706666776252	0.0516500896589701	0.526430253134836
Znfx1	Znfx1	0.0937909035649964	4.68791861760621	3.78679995634499	0.0516583212867862	0.526430253134836
Irak3	Irak3	0.16475282929023	1.66103488195787	3.78574144814034	0.0516910041605258	0.526430253134836
Tnnc1	Tnnc1	-0.253471205458937	0.463010051118263	3.78562012134513	0.0516947513776321	0.526430253134836
RGD156	RGD156388	0.179744290050473	2.05607512729939	3.78419128523535	0.0517389070167086	0.526430253134836
Mmp16	Mmp16	0.0897000267436529	6.16662809043384	3.78378213169826	0.0517515584375483	0.526430253134836
St3gal4	St3gal4	-0.068889351420182	5.27829550214935	3.78222135476909	0.051799869221975	0.526430253134836
Calm3	Calm3	-0.0762838690834851	9.60641967594077	3.78140927067668	0.0518249941360229	0.526430253134836
Ccny	Ccny	0.0558204632039475	5.97081932682635	3.7809267552821	0.0518401264713443	0.526430253134836
Mfap2	Mfap2	0.107913446741266	4.80376548128269	3.7802207957086	0.0518618166849006	0.526430253134836
Cdkl1	Cdkl1	0.129320229619964	4.78390047173747	3.77859335158967	0.0519122847347077	0.526430253134836
Arid1a	Arid1a	0.100262886808222	8.16295571870848	3.77764229342397	0.0519418016882119	0.526430253134836
Vwa5b2	Vwa5b2	-0.224200602296268	6.2022365175909	3.77571419640931	0.0520018964337102	0.526430253134836
Gtpbp3	Gtpbp3	-0.0958792181550406	3.95051111775328	3.77298573872154	0.0520865786249791	0.526430253134836
Rpl22l1	Rpl22l1	-0.121339792263483	5.5378312300999	3.7727775862856	0.0520930602532763	0.526430253134836
Utp23	Utp23	0.120584670076879	2.88773196330189	3.77125728231532	0.0521373096774632	0.526430253134836
Sephs1	Sephs1	0.0492260350321693	6.45405994475415	3.7701245584197	0.0521757473035922	0.526430253134836
Serpinf1	Serpinf1	0.299617129242127	3.72219324728235	3.7675160456188	0.0522540579628242	0.526430253134836
Slc25a18	Slc25a18	-0.18050967871631	4.15186760548505	3.76737524647464	0.0522615817957724	0.526430253134836
Tmem26	Tmem26	0.265940241330259	0.503426852415545	3.76495956668153	0.0523371238178965	0.526430253134836
Evi2a	Evi2a	0.271147578793584	0.792795001903265	3.76404486698658	0.0523657576062127	0.526430253134836
Rnf135	Rnf135	0.203747719403725	1.59056386409084	3.76402930796584	0.0523662448150882	0.526430253134836
Kdm3a	Kdm3a	0.0847049971000196	5.10523504074033	3.76169961862212	0.0524392499146918	0.526430253134836
Cytl1	Cytl1	-0.378784936224691	-0.330159848076014	3.76136926057949	0.0524496110084674	0.526430253134836
Tjp1	Tjp1	0.0866357648822759	6.68408008535574	3.76115260948145	0.0524560923549853	0.526430253134836
Pla2g4a	Pla2g4a	0.303671114880017	0.438836045842634	3.7590017081222	0.0525238285240248	0.526430253134836
Gck	Gck	-0.29562624174366	1.35003402813768	3.7588369755812	0.0525291067686861	0.526430253134836
Tie1	Tie1	0.151650415716543	5.5642905188885	3.75052586564753	0.0527908972967316	0.52734529552386
Inip	Inip	0.0784777584910281	4.56580307879804	3.74773459170152	0.0528791340634982	0.52734529552386
Nup98	Nup98	0.0829659386446685	5.96812347006146	3.74568241977259	0.0529441038551484	0.52734529552386
Lrp6	Lrp6	0.0950023762272627	5.59428989947439	3.74563433328753	0.0529456272400053	0.52734529552386
Panx1	Panx1	0.0619528526137638	6.22958488459028	3.74382546143679	0.0530029662087477	0.52734529552386
Thpo	Thpo	0.312960200904834	0.171843918561735	3.74373718328607	0.0530057661973993	0.52734529552386
Zfp39	Zfp39	0.112399682902485	4.04833998936794	3.74271287470073	0.0530382315514104	0.52734529552386
Vhl	Vhl	0.0599787809205845	6.31482949276984	3.74246506362722	0.053046132426531	0.52734529552386
Col1a1	Col1a1	0.403400706097358	7.40454614125632	3.74095137592012	0.0530942063748824	0.52734529552386
Shfl	Shfl	-0.150879586478788	4.34623902963407	3.74031002734981	0.0531145891891323	0.52734529552386
Mterf3	Mterf3	0.0906580041267663	3.63478577131634	3.73858948522616	0.0531693109861278	0.52734529552386
Omg	Omg	-0.101104889121241	4.97873395207426	3.73854549357236	0.0531707109219506	0.52734529552386
Lypd1	Lypd1	-0.128023782630049	5.22618030151989	3.73793041174574	0.053190288611417	0.52734529552386
Epstl1	Epstl1	-0.362709785460028	0.0428614846739274	3.73531547742287	0.0532738056852467	0.52734529552386
Omd	Omd	0.392085988093166	0.0216920065008616	3.73311388496643	0.0533438599758585	0.52734529552386
LOC688	LOC688765	0.144498522657807	2.15162085340683	3.73296240555332	0.0533488973929045	0.52734529552386
Carm1	Carm1	0.0470049624118966	6.81734434989855	3.73234121809053	0.0533686295538874	0.52734529552386
Rbpj	Rbpj	0.0771101989198716	6.26843870057785	3.73184530374742	0.0533811900494218	0.52734529552386
Nectin3	Nectin3	0.103738723874384	4.91430375540512	3.72811785274838	0.0535036509647197	0.527454184859403
Magee1	Magee1	-0.0805552811465834	7.54128847948398	3.72611503343687	0.0535678505666096	0.527454184859403
Pgp	Pgp	-0.0736348315325516	6.04649856384261	3.72283188804101	0.0536723654829677	0.527454184859403
Zfp609	Zfp609	0.0784454675250442	7.25544031511605	3.72282288977638	0.0536725563116304	0.527454184859403
Urb2	Urb2	0.0825702251440405	4.57969308166417	3.72216904214676	0.0536945766535722	0.527454184859403
Slc22a8	Slc22a8	0.176591434313537	4.02833094567992	3.72075651905621	0.0537400171871738	0.527454184859403
Dcn	Dcn	0.374162769624253	4.16329778559167	3.7184681077449	0.053813721313669	0.527454184859403
Sfpq	Sfpq	0.0715803322203573	8.97067244918837	3.71776122097126	0.0538365100474584	0.527454184859403
Fstl4	Fstl4	0.137177202855891	3.79377813208809	3.71646808988038	0.0538782248838497	0.527454184859403
Pla2g6	Pla2g6	0.0785036223827352	4.69693857462205	3.71519991485398	0.0539193675292475	0.527454184859403
Ash1l	Ash1l	0.122050095822004	7.32425399815259	3.71488183469407	0.0539294418701967	0.527454184859403
Cdc42ep	Cdc42ep4	0.0913171212657349	6.89434063218979	3.71465980959954	0.0539366614755431	0.527454184859403
Ppp1r12	Ppp1r12a	0.0871598988811175	6.09928405127464	3.71450006914438	0.0539417776058293	0.527454184859403
Cycs	Cycs	-0.086257123611503	6.9950628961314	3.71210907914367	0.0540190928352668	0.527796571108554
Pcsk4	Pcsk4	-0.188894146553606	2.36509445417615	3.70725482252845	0.054176421727497	0.529489564094429
Vezt	Vezt	0.0544557682849582	6.74672747089278	3.70479356589925	0.0542563806186291	0.529489564094429
Sh3gl3	Sh3gl3	-0.0641117941805442	5.53754089900787	3.70431246218407	0.0542720265976458	0.529489564094429
Dip2c	Dip2c	0.0617086331170451	7.40773542507058	3.70387806438507	0.0542861559592519	0.529489564094429
Tcn2	Tcn2	0.134952851038535	4.49987991164068	3.70223905854024	0.0543395242429813	0.529489564094429
Dock6	Dock6	0.136974307126454	4.38883685952584	3.70146435971799	0.0543647359241204	0.529489564094429
Ccdc8	Ccdc8	0.127268977702238	3.39679962485645	3.70079580671525	0.054386519219143	0.529489564094429
Bcl2	Bcl2	0.151482299503999	2.0716174271423	3.69315861069971	0.0546360581747258	0.529003434280649
Hexa	Hexa	0.0729721121144282	5.20495181499871	3.69159862776891	0.0546871773828674	0.529003434280649
Pcsk2	Pcsk2	-0.0872175392071303	6.0804285495693	3.6915312977721	0.0546893853218369	0.529003434280649
Slc25a4	Slc25a4	-0.0757768418769146	9.32748434537571	3.69074035508154	0.0547153243309593	0.529003434280649
Thy1	Thy1	-0.117351639411712	6.87963902917174	3.68900176686468	0.054772387502554	0.529003434280649
Mypop	Mypop	0.106603582413672	3.8444832250135	3.68859448764014	0.0547857641581057	0.529003434280649
Capn2	Capn2	-0.0541421990177035	6.76002009581101	3.68830802576517	0.0547951747699056	0.529003434280649
Itgb8	Itgb8	0.12276263219622	4.66061893280183	3.68796621202459	0.0548064059881857	0.529003434280649
Plau	Plau	0.410125010666344	-0.268836085518569	3.68725582338921	0.0548297555730198	0.529003434280649
Grin2d	Grin2d	-0.115400093380479	6.69690099709536	3.68623326934454	0.054863384143557	0.529003434280649
Cebpg	Cebpg	0.0866002040642411	4.45287376585578	3.68084967881029	0.0550407943772368	0.53014119748622
Adck2	Adck2	0.0894312335484935	4.05694189733712	3.68007535146842	0.0550663614604585	0.53014119748622
Inppl1	Inppl1	0.0932260394498854	5.36573438221561	3.67708227053484	0.055165306796063	0.5306842967575
Uqcr10	Uqcr10	-0.104651337525709	5.84735169670647	3.66734203342	0.055488567985916	0.532012739432647
Cdk16	Cdk16	-0.0285559250131585	8.39779643212988	3.66624786886648	0.0555250498921986	0.532012739432647
Scn1b	Scn1b	-0.150614644513835	3.98239305264483	3.66570201430784	0.0555432400270677	0.532012739432647
Iqgap1	Iqgap1	0.112683487050632	5.32890496734654	3.66534049227843	0.0555552909174606	0.532012739432647
Sebp3	Sebp3	0.0661240855211179	7.36592001657039	3.66518059220935	0.0555606218732089	0.532012739432647
Itga5	Itga5	0.128631423485143	3.50597069332075	3.66481526929604	0.0555728035177744	0.532012739432647
Agpat2	Agpat2	0.416843971114711	-0.582210652858919	3.66380199444349	0.0556066058809269	0.532012739432647
Pcdha9	Pcdha9	-0.134435584372249	2.84939430212393	3.66184975735164	0.0556684519410579	0.532012739432647
Pfdn1	Pfdn1	-0.0830752639875593	6.08528977073574	3.66093767328135	0.0557027761837273	0.532012739432647
Srsf10	Srsf10	0.0777240652978782	5.66359362836217	3.65965681504616	0.0557451142165532	0.532012739432647
Cfap45	Cfap45	-0.189990059454214	1.76293790578924	3.65568018359751	0.0558783246189493	0.532012739432647
Mkl1	Mkl1	0.0682742927796561	5.72635619832853	3.65558446220201	0.0558815455232376	0.532012739432647
Med14	Med14	0.0891123598051395	6.35033620338087	3.65099618387359	0.0560356857458876	0.532012739432647
Tmod4	Tmod4	-0.156821384763606	1.27903520217473	3.65049610991925	0.0560525126427321	0.532012739432647
Entpd2	Entpd2	-0.118831205369475	4.03125676412729	3.65028523863104	0.056059609881825	0.532012739432647
Fam131b	Fam131b	-0.0579794826404848	7.7735629711288	3.64980866826478	0.0560722862068822	0.532012739432647
Yap1	Yap1	0.13858083226397	3.69917409896526	3.64741111571854	0.0561564376819617	0.532012739432647
RT1-DM	RT1-DMa	-0.153732002167133	2.51768552281005	3.64429072634474	0.0562583843956426	0.532012739432647
RGD131	RGD131184	0.0808229843675863	3.90755361981165	3.64423658847134	0.0562635922508214	0.532012739432647
Usb1	Usb1	0.0910659724463535	3.37258723970313	3.6442314432588	0.0562637661009007	0.532012739432647
Tab1	Tab1	0.0577136785489141	6.09054818526287	3.64240775118904	0.0563254222177096	0.532012739432647
Gprasp1	Gprasp1	-0.0822282127165167	9.33862929612852	3.64149167295819	0.056356758886541	0.532012739432647
Unc45a	Unc45a	-0.0430442887132013	6.73515686792858	3.64127076411875	0.0563638982135233	0.532012739432647
Car2	Car2	0.174596809407098	4.44636809582794	3.64102230537773	0.0563723098470651	0.532012739432647
Dcaf11	Dcaf11	-0.0486162690598146	6.52021422198273	3.63866646425941	0.0564521336719901	0.532012739432647
Rgs4	Rgs4	-0.0722287801293032	6.9652951681483	3.6309882521646	0.0565090702968273	0.532012739432647
Cpeb1	Cpeb1	-0.18094281799547	1.69449436460625	3.63637082769391	0.0565298254744482	0.532012739432647
Snurf	Snurf	-0.234188103885357	0.230924589621417	3.63563227225296	0.056555119076948	0.532012739432647
Col1a2	Col1a2	0.389031728495739	7.22933845526089	3.63506651866558	0.0565743437169064	0.532012739432647
Plcl1	Plcl1	0.0884187488020634	5.26046654176631	3.63425331781337	0.0566019889302912	0.532012739432647
Zfp397	Zfp397	0.148778881207915	3.483566928031	3.63251352395312	0.056661180942302	0.532012739432647
Bcl11a	Bcl11a	0.0923644958322605	7.56284999576459	3.63231727791536	0.0566678633251035	0.532012739432647
Glg1	Glg1	0.069919549579229	7.37090130359631	3.62985704503867	0.0567585113985591	0.532351895560829
Fam120a	Fam120a	0.0538508990010857	7.25844275593456	3.62709189036759	0.0568460519843843	0.532351895560829
Ephb3	Ephb3	-0.0952016060198286	4.61094062717877	3.62623212924963	0.056871990973058	0.532351895560829
Minar2	Minar2	-0.0652332870580314	6.1369630882262	3.62587687741654	0.0568875828928794	0.532351895560829
Kcnj8	Kcnj8	0.202440903002476	2.13087691151429	3.62500730636147	0.056917322167039	0.532351895560829
Glis2	Glis2	0.0838096410008296	5.26953806221713	3.6222581545936	0.0569771880816965	0.532512622278148
Srgap2	Srgap2	0.0783325990857896	7.13555114413732	3.61956683872487	0.0571037357106394	0.533279220712191
Maz	Maz	0.0703006324796926	8.2168803774796	3.61813824388834	0.0571527971462348	0.533279220712191
Lrrc8c	Lrrc8c	0.0907182735139657	4.20071043822242	3.61703705818804	0.0571906624446528	0.533279220712191
Sgcb	Sgcb	-0.0787940300966449	6.12502214680188	3.61539033499065	0.0572473863020618	0.533279220712191
Osbp2	Osbp2	-0.0532838768615845	6.40266621969012	3.61201062685193	0.0573637279088562	0.533279220712191
Slc8b1	Slc8b1	0.277562601807713	2.89542985151717	3.61170134697698	0.0573743995782118	0.533279220712191
Stam2	Stam2	0.0711631301212246	4.77542321351465	3.61032729132585	0.0574218179250325	0.533279220712191
Vasn	Vasn	0.112755291467521	4.00490079426056	3.609258199114	0.057458743525974	0.533279220712191
Gjc1	Gjc1	0.129974199451665	5.4754943144625	3.60908458021758	0.0574647425651086	0.533279220712191
Kcnab2	Kcnab2	-0.0818138817400752	4.66715247032646	3.6078496740267	0.0575074213892226	0.533279220712191

Gfpt2	Gfpt2	0.0877373866682061	4.2194155795845	3.60721568397079	0.0575293605097343	0.532279220712191
Bicc1	Bicc1	0.218445974819762	2.2674195036429	3.60557695457753	0.0575960838235882	0.532324792210334
Htr1f	Htr1f	-0.234445476426762	-0.65663029664638	3.60200582515388	0.0577099011330015	0.532324792210334
Arih1	Arih1	0.0493741708921952	7.35217326285075	3.60124005319764	0.0577364890057881	0.532324792210334
Sema3f	Sema3f	0.0912965243435632	5.9998921636611	3.60096028939996	0.0577462056211989	0.532324792210334
Mrc2	Mrc2	0.200182096428731	4.48389395393507	3.60058733477382	0.0577599615945542	0.532324792210334
Heatr5b	Heatr5b	0.0706952877678386	7.07333169416858	3.59964738133525	0.0577918280891527	0.532324792210334
Rtn3	Rtn3	-0.0576134133996156	10.4442802570852	3.59691782179171	0.0578867967704589	0.532816380861061
Rpl23a	Rpl23a	-0.0745236767681092	8.51623971206991	3.59432456281311	0.0579771842402523	0.532942511706062
Ccdc102	Ccdc102a	0.161104916250344	3.3975503084525	3.59407005868536	0.0579860626239484	0.532942511706062
Grid2	Grid2	-0.131145031963436	4.32267665935152	3.58960574850092	0.0581420353037824	0.534822793814933
Cdc37l1	Cdc37l1	0.0569558706750656	5.40742241455611	3.58701862926059	0.0582326272029374	0.534822793814933
Clec4a1	Clec4a1	0.456337824967605	-0.712094705468663	3.58623051653009	0.0582602539545767	0.534822793814933
Zkscan7	Zkscan7	0.132804982426122	3.18097319628301	3.58566534937406	0.0582800740752508	0.534822793814933
Slc12a4	Slc12a4	0.125615919704636	4.54198043232465	3.58124448276216	0.058435359006846	0.534822793814933
Gfra2	Gfra2	-0.104439886005012	4.92488835932526	3.57974360324462	0.0584881779898018	0.534822793814933
Spart	Spart	0.101048933098983	4.48177223845061	3.57874128808485	0.0585234797363602	0.534822793814933
Slc16a8	Slc16a8	0.400640037482248	0.821631106150269	3.57707305085039	0.0585822856082552	0.534822793814933
Map7d2	Map7d2	-0.0998010044693046	5.39207856643475	3.57392962495111	0.0586932630349946	0.534822793814933
Rps4y2	Rps4y2	-0.283617748672563	2.67765968731835	3.57384774328968	0.0586961568191484	0.534822793814933
Stk32c	Stk32c	-0.0763593825550606	6.49082823986039	3.57370426865832	0.0587012277293479	0.534822793814933
Vgf	Vgf	-0.148913373072399	7.30605038641749	3.57368351690542	0.0587019612095727	0.534822793814933
Tmem12	Tmem123	0.165440722459412	3.82172418508324	3.57302229815586	0.058725327389947	0.534822793814933
Angptl4	Angptl4	-0.362821540453613	-0.214175275210498	3.57278912742996	0.0587335830741036	0.534822793814933
Kdm1b	Kdm1b	0.0635908523641323	5.89808900789031	3.57204567425119	0.0587598716274868	0.534822793814933
Ptgr1	Ptgr1	0.363033148062588	0.081495189958514	3.57183050018884	0.0587674962186642	0.534822793814933
Psd4	Psd4	0.332489657849799	-0.265598521965777	3.56989494796707	0.0588360333271237	0.535056258837627
Rttn	Rttn	0.133709430443556	3.22533948219837	3.5686406674473	0.0588804992446522	0.53507064025895
Wdr54	Wdr54	-0.0763463912828241	5.57769472433796	3.56689204653406	0.0589388830344061	0.535212293537308
Cspg4b	Cspg4b	0.246827085216924	1.9502781208861	3.56209668033806	0.0591130283882192	0.536402367509694
Cox7c	Cox7c	-0.125123075468983	6.69862203922915	3.56062203250976	0.0591655636892657	0.536488908539948
Bbc3	Bbc3	0.105532826650843	5.82019211720037	3.55764828202179	0.0592716565217047	0.536744995581577
Pip4k2a	Pip4k2a	0.0570949147027851	5.40490831905139	3.55741906641108	0.059279843224446	0.536744995581577
Cd74	Cd74	-0.234989351765653	-0.268448880188465	3.55467817879352	0.0593778211403401	0.537207727137425
Ptger3	Ptger3	-0.157782497765678	3.14901312501298	3.55358182275192	0.0594170604672315	0.537207727137425
Rab20	Rab20	0.258349177417089	-0.369045614192009	3.54916049003269	0.0595755851562901	0.538250960170189
Snap91	Snap91	-0.0642150260827596	9.07738787035662	3.54472001040558	0.0597352480162762	0.539302959116554
Stx16	Stx16	0.0740869600424212	5.57826597089521	3.54094619520926	0.0598712981680486	0.540140410152827
Arhgap2	Arhgap22	-0.13823129774849	2.34312962419336	3.5383744591924	0.0599642008472852	0.540587680456608
Cmklr1	Cmklr1	0.319777845350048	0.260018844697078	3.53586775103257	0.0600512808758802	0.540892289160273
Ctsz	Ctsz	0.192988010856066	2.96554366463333	3.53007156640115	0.0602651868560207	0.540892289160273
RGD130	RGD130755	0.0588068669078594	5.32011507967011	3.52699488406114	0.0603774908273829	0.540892289160273
Bcan	Bcan	-0.0852372529836812	8.0039109548887	3.52570197952561	0.0604242227282384	0.540892289160273
Ttc4	Ttc4	-0.08317246739539	3.57770014952896	3.52166712933016	0.0605714913473734	0.540892289160273
Rab3b	Rab3b	-0.0995610503838166	6.00208368862382	3.52092491473222	0.0605986212714483	0.540892289160273
Plxnb2	Plxnb2	0.0677172190852099	7.90145291926656	3.51950726649945	0.06065047603874	0.540892289160273
Sod3	Sod3	0.37439353248201	2.72672336678547	3.51901902212936	0.0606683459814134	0.540892289160273
Olfml3	Olfml3	0.253026261125271	3.31246510701831	3.51704274695948	0.0607407357166019	0.540892289160273
Ccdc65	Ccdc65	-0.0685089088942164	4.504561164561	3.51668967312848	0.0607536672691393	0.540892289160273
Kctd3	Kctd3	-0.0378959801032177	7.37790014495959	3.51628712426493	0.0607684279642825	0.540892289160273
Slco2b1	Slco2b1	0.186563709308831	2.98806268269498	3.5146217385619	0.0608295410535106	0.540892289160273
Ece1	Ece1	0.0754659930253686	5.21563228801156	3.51391531100108	0.0608554796527382	0.540892289160273
Cavin2	Cavin2	0.206165756810772	2.93418891832706	3.51358748155172	0.0608675208894561	0.540892289160273
Htr1a	Htr1a	0.153699652974644	2.05267049459504	3.51266965208557	0.0609012464263223	0.540892289160273
MGC941	MGC94199	-0.0582138672240926	5.35511485923218	3.51247162716869	0.0609085254368808	0.540892289160273
Slc38a1	Slc38a1	0.0609007082385621	6.84455507852291	3.51210464045035	0.0609220176026348	0.540892289160273
Egfl6	Egfl6	-0.168766808239448	2.10424774280462	3.51098113598346	0.0609633427155768	0.540892289160273
Fndc5	Fndc5	-0.111162089788255	4.56761194609386	3.51010359701505	0.0609955543255288	0.540892289160273
Tnpo1	Tnpo1	0.0905319853073558	7.58704918369335	3.50773182346936	0.0610830276468843	0.540892289160273
Fhl3	Fhl3	0.126884228805386	2.8523401068692	3.50706532110064	0.0611076085186163	0.540892289160273
Bdnf	Bdnf	-0.191901339823586	2.67744176117255	3.5060405710534	0.061145422617906	0.540892289160273
Tcf20	Tcf20	0.0637000569009405	7.57840111364041	3.50527412963913	0.061173720926635	0.540892289160273
Foxc1	Foxc1	0.275150228896719	3.97236303425497	3.50466946700348	0.0611960928730761	0.540892289160273
H6pd	H6pd	-0.114741730015683	2.86366209933551	3.50423664969249	0.0612120486449858	0.540892289160273
Cmtr2	Cmtr2	0.0882510209764772	3.99588080168585	3.50372657676882	0.0612309016893454	0.540892289160273
Naa25	Naa25	0.0626603998841761	5.86117860570548	3.50327170681966	0.0612477195649677	0.540892289160273
Arhgdig	Arhgdig	-0.110739637003482	6.29141154155294	3.50197819469925	0.0612955713814572	0.540892289160273
Dab2ip	Dab2ip	0.0451094569825622	7.97495146034347	3.50180341367236	0.0613020402310282	0.540892289160273
Cpne6	Cpne6	-0.17227788596201	5.37780984049642	3.50138376954514	0.0613175747196097	0.540892289160273
Nap1l2	Nap1l2	-0.0587992555974756	6.51058241216054	3.49981538917689	0.0613756705180861	0.540892289160273
Enkur	Enkur	-0.154426116347693	3.41793574951405	3.49810807188628	0.0614389794295276	0.540892289160273
Sin3a	Sin3a	0.0596890840927532	5.98547448298647	3.49620112987929	0.0615097728168112	0.540892289160273
Rhobtb3	Rhobtb3	0.116384136471273	4.22537452586758	3.49584289754989	0.0615230815350316	0.540892289160273
Elk3	Elk3	0.153076469328994	3.88567577089956	3.49541067152088	0.0615391432810226	0.540892289160273
Scrn3	Scrn3	-0.131366901432007	2.67123160216954	3.49488638778926	0.0615586319313607	0.540892289160273
Mapk8	Mapk8	0.052131403510714	8.38129236983444	3.48319684417316	0.0619948663329064	0.544341975535308
F13a1	F13a1	0.311791631670674	1.44676039854703	3.48032314963962	0.0621026111238048	0.544904557255362
Lpgat1	Lpgat1	0.0526182586115868	6.58180644463892	3.47721124294814	0.0622195125352819	0.545261010682078
Bckdk	Bckdk	0.081347133064813	5.82177547780863	3.47691538929102	0.062230638713274	0.545261010682078
Proser3	Proser3	0.118728395300754	4.37970393462006	3.47487767197387	0.062307328808113	0.545549853711457
Ogn	Ogn	0.377777318941253	4.67443237037501	3.47267849561563	0.0623902084377746	0.545557196045289
Gatad2a	Gatad2a	0.0551658155244746	6.72997564709894	3.47184526235671	0.0624216408958664	0.545557196045289
Zwint	Zwint	-0.108211645128384	10.528828635152	3.47001962340898	0.0624905689663668	0.545557196045289
Slc35c2	Slc35c2	0.125324398223045	5.17038827549734	3.46883643581425	0.062535294836727	0.545557196045289
Csf1r	Csf1r	0.230351191119386	4.28130079741884	3.46760008876751	0.0625820457764349	0.545557196045289
Zfp644	Zfp644	0.0659262495055574	6.618500600928	3.46592932828869	0.0626452958612254	0.545557196045289
Habp4	Habp4	-0.0962174352980523	5.39119880395452	3.46578965936394	0.0626505988110737	0.545557196045289
Nudt7	Nudt7	0.27152507192241	1.10690027718827	3.46559482013537	0.0626579676150436	0.545557196045289
Pfkfb3	Pfkfb3	0.0770835740496657	5.04535690503536	3.46369622909659	0.0627299417059402	0.545624040016607
Glo1	Glo1	-0.0612071043284435	6.30882551204091	3.46161915864202	0.0628087823175374	0.545624040016607
Serpina1	Serpina11	0.321375653030116	-0.244313169830985	3.46145789037763	0.0628149080898883	0.545624040016607
Fbxo42	Fbxo42	0.0715355789464506	5.26378722574007	3.46075228460622	0.0628417180014317	0.545624040016607
Itga6	Itga6	0.0810234541431481	5.51071817502172	3.45938151834395	0.0628938259435295	0.545706808809052
Nme6	Nme6	0.190676379867517	2.28518094342623	3.45557097728263	0.0630389587943995	0.545710568028797
Dnajb4	Dnajb4	-0.0815504294322643	4.93593268458733	3.45406114402545	0.0630965589405736	0.545710568028797
Eea1	Eea1	0.114502228502921	5.03872723028163	3.45321608002776	0.0631288224632647	0.545710568028797
Fgf3	Fgf3	-0.610041073501526	-0.707634410802501	3.45292751988006	0.0631398434022886	0.545710568028797
Pkia	Pkia	-0.0577879795350596	8.92844132510459	3.45143798947237	0.0631967654621729	0.545710568028797
Gatad2b	Gatad2b	0.11978332984162	5.01817947734655	3.45015509743739	0.0632458347282736	0.545710568028797
Crtac1	Crtac1	-0.0958030741406975	5.52823844072064	3.44940067100671	0.0632747097054325	0.545710568028797
Nqo1	Nqo1	-0.1363398523874	3.02645579037581	3.4490462352985	0.0632882764320781	0.545710568028797
Ppic	Ppic	0.165867635435919	3.90404605500773	3.44827442959658	0.0633178417097714	0.545710568028797
Cep120	Cep120	-0.0898877556593403	6.45732083684919	3.4479142449546	0.0633316424225132	0.545710568028797
Edn3	Edn3	0.366988209469911	0.603273481216093	3.44563673244733	0.0634189810964897	0.546068375411429
Dap	Dap	0.126398354405989	3.79321799143641	3.44455003898524	0.0634606992342169	0.546068375411429
Cxcl14	Cxcl14	-0.178270494968628	5.18745205261803	3.44291784087032	0.0635234140721502	0.546221314526684
Pdha1	Pdha1	0.091307590123492	4.72881425670044	3.43978732914017	0.0636438845625612	0.546497723484732
Clgn	Clgn	-0.150579739857741	2.64955545194255	3.43786478508989	0.0637179899361473	0.546497723484732
Amigo1	Amigo1	0.0711013963831275	4.17044749223479	3.43727654506309	0.0637406923052334	0.546497723484732
Foxn1	Foxn1	0.254142399868489	0.711085493232284	3.43723750961688	0.0637421884694649	0.546497723484732
Gpr21	Gpr21	0.159643740420723	2.48495575552759	3.43559282725703	0.0638056922572694	0.546497723484732
Smim1	Smim1	0.174785054091115	1.21557186995866	3.43529469360055	0.0638171994619904	0.546497723484732
Shox2	Shox2	-0.601878509518074	2.26397613818067	3.4317722436929	0.0639534382593315	0.54726728833432
Efemp1	Efemp1	0.420611012360283	1.86725552529982	3.43070472536779	0.0639947883049828	0.54726728833432
Glyr1	Glyr1	0.0514228875124844	7.06246835329182	3.42899274090515	0.0640611611197233	0.547459662527936
Bscl2	Bscl2	-0.0859895127387426	5.30001270210957	3.42679842182564	0.0641466908103871	0.547815373885858
Mal2	Mal2	-0.0756110496445101	6.18988136479583	3.42525686220353	0.0642140279455449	0.547880874322292
Vps39	Vps39	0.0492068671531909	6.84177276224603	3.42433315547493	0.0642421931476728	0.547880874322292
Abcb9	Abcb9	-0.106181121554439	5.05897498332111	3.42255535187441	0.064311393453634	0.548096486421442
Lemd3	Lemd3	0.0581614589178306	5.44343404187474	3.420089501509	0.064407521276931	0.548541353222539
Sugp1	Sugp1	-0.0686410371851024	5.66882221541361	3.41481046389661	0.0646128323090716	0.549310318955691
Pex1	Pex1	-0.0612473921484372	5.44461937707313	3.41430669232246	0.0646323557983539	0.549310318955691
Sort1	Sort1	0.0668043736253489	6.84705082522006	3.41186114798445	0.0647294032042543	0.549310318955691
Ybx3	Ybx3	0.0632666653893039	7.18545649081036	3.41032551805542	0.0647896647610244	0.549310318955691
Chrm3	Chrm3	-0.0943041686275483	5.2109856348862	3.40988145711025	0.064903174689834	0.549310318955691
Flrt2	Flrt2	0.100716444803279	5.11096757229305	3.40768618024502	0.0649823781953646	0.549310318955691
Fam126a	Fam126a	0.077971730802677	5.04645494309128	3.40765943236185	0.0649844301664218	0.549310318955691
Ino80d	Ino80d	0.161614029040889	3.47189135408268	3.40677212340473	0.0649292978297763	0.549310318955691
Cybc1	Cybc1	0.0838998403881184	4.52251360124362	3.40596846815075	0.0649606712772713	0.549310318955691
Ythdc1	Ythdc1	0.0547015633740564	6.47343594465318	3.40435065919482	0.0650247018154054	0.549310318955691
Igsf3	Igsf3	0.0812585158109153	8.08903688338962	3.40320436956718	0.0650698981235428	0.549310318955691
Slc4a10	Slc4a10	-0.0786053295414696	6.29642847800458	3.40273681530977	0.065088342641535	0.549310318955691
Trmt1l	Trmt1l	0.0778073391449694	4.65459965360071	3.40232422463106	0.0651046235393304	0.549310318955691
Slc6a7	Slc6a7	-0.166435141782654	6.01809551969368	3.40208140993261	0.0651142070798644	0.549310318955691
N4bp1	N4bp1	0.0939150698084098	5.95590411536278	3.39823040516208	0.0652624451866014	0.550126892831513
Ttc19	Ttc19	-0.0764219455375971	5.47883692388628	3.39734647257048	0.0653013893891633	0.550126892831513
Slc38a2	Slc38a2	0.0638995353735479	7.85272453803423	3.39406409091134	0.0654314884472068	0.550126892831513
Acsbg1	Acsbg1	-0.133836899778125	6.0289496032614	3.39272696400907	0.0654845629591007	0.550126892831513
Olfml2b	Olfml2b	0.106896185930918	4.1530355565381	3.39230011644457	0.0655015151868551	0.550126892831513
Synrg	Synrg	0.0493820985519841	7.17192487775602	3.39219215470767	0.0655058038245815	0.550126892831513
Pvr	Pvr	0.12186557681345	2.41739271544838	3.39183565806515	0.0655199673131979	0.550126892831513
Copz2	Copz2	0.337853465463038	1.41116612857729	3.39073460445083	0.0655637324389044	0.550126892831513
Rnf41	Rnf41	0.0502973625954131	7.12539763176091	3.38471408795109	0.0658035909276059	0.55013970586115
Sphk1	Sphk1	0.342458870636466	1.73069533172881	3.38428342114171	0.0658207842433428	0.55013970586115
Tpp1	Tpp1	0.0904433498036977	4.52742253133402	3.38330955068837	0.0658596826246056	0.55013970586115
Krt8	Krt8	0.479835123207186	2.08069247497013	3.38262931902565	0.0658868667524055	0.55013970586115
Exoc4	Exoc4	0.0617547999945556	6.27121127403227	3.38210540549597	0.0659078120991296	0.55013970586115
Parp9	Parp9	0.174544090664571	1.87563917879784	3.38173931064392	0.0659224522908245	0.55013970586115
Rarres2	Rarres2	0.326959440989275	0.886215839796202	3.38169706558593	0.0659241419017193	0.55013970586115
Dmgdh	Dmgdh	-0.255438635917697	2.39793485124033	3.38140336595521	0.0659358898281299	0.55013970586115
Fbxo36	Fbxo36	-0.27608960168884	0.0394740680266762	3.38039330766132	0.0659763090279776	0.55013970586115
Stau1	Stau1	0.0405476331751445	7.11892310342664	3.37964720587537	0.066006182549823	0.55013970586115
Fancd2	Fancd2	0.150091163162589	3.17748722887195	3.37756347854103	0.0660896904244566	0.550193211582494
Gper1	Gper1	0.19095207856082	0.934203218007728	3.3771214713077	0.0661074188959282	0.550193211582494
Lrpap1	Lrpap1	-0.0582341240956473	6.63240369315488	3.3761876064649	0.066144892361163	0.550193211582494
Axin2	Axin2	0.0919069860837021	4.82475893712623	3.37503575201337	0.0661911435824156	0.550211124901931
Grid2ip	Grid2ip	-0.231369359208171	2.91364266023099	3.36949906251545	0.0664138884642692	0.550910397618549
Mlh1	Mlh1	0.0977671589775159	4.17971029918314	3.36802630943366	0.0664732366642886	0.550910397618549
Lum	Lum	0.401836270500914	3.66638983714039	3.36783240171833	0.0664811716892532	0.550910397618549
Uchl5	Uchl5	-0.0716678035942032	4.40952708361165	3.366995499879	0.0665165709199726	0.550910397618549
Tardbp	Tardbp	0.0629545234522023	7.56765611837549	3.36658252780141	0.0665316323529201	0.550910397618549
Cyb5r2	Cyb5r2	0.2569083134341	0.866523779312585	3.36372491857013	0.0666471585382602	0.550910397618549
Kazn	Kazn	-0.0546217107948473	6.00235212285788	3.36311800485362	0.06667172195796	0.550910397618549
Ptprb	Ptprb	0.124861405916308	4.49499146939363	3.36250608320358	0.0666964978381232	0.550910397618549
Sncb	Sncb	-0.0814564441722936	8.05127362437138	3.36134618132111	0.066743487710234	0.550910397618549
Capg	Capg	0.28826500872284	1.55900830884783	3.36014469824873	0.0667921967796651	0.550910397618549
Pex26	Pex26	0.0757312103632397	4.96782500127388	3.35985110551347	0.0668041083121568	0.550910397618549
Ntm	Ntm	-0.061205544606052	7.56258467291019	3.35982626680315	0.0668051159410807	0.550910397618549
Slco2a1	Slco2a1	0.209321153671412	3.12430242342636	3.35199842831178	0.0671234766555144	0.552895172610894
Itm2a	Itm2a	0.150223586237247	5.76090964114234	3.35116263005149	0.0671575649627239	0.552895172610894
Tgfb2	Tgfb2	0.115291166526897	6.23210221218255	3.35064378194092	0.0671787354073889	0.552895172610894
Cbx2	Cbx2	0.12850402078318	4.0177034752194	3.345540918029	0.0673873268757529	0.554246326584563
LOC3671	LOC367195	0.25626036725022	0.450022060532111	3.3441803400112	0.0674430604969054	0.554328305546699
Ro60	Ro60	0.0875888349866536	6.05010100580956	3.34191336648283	0.0675360322647182	0.554738042820204
Bmp5	Bmp5	0.372613961630228	1.59300180208659	3.33857206860752	0.0676723147145745	0.555213890722105
Crb3	Crb3	0.301353034526078	0.969802358005421	3.33734784618088	0.0677236873672837	0.555213890722105
Ifitm1	Ifitm1	0.299332130514529	0.97451539679406	3.33645151506323	0.0677605942391426	0.555213890722105
Etv1	Etv1	-0.0619742970309725	6.79335561540016	3.33617556800361	0.0677719608535518	0.555213890722105
Golm2	Golm2	0.0599913228300032	7.738667988265	3.33320497607897	0.0678944525341556	0.555600277549595
Asrgl1	Asrgl1	-0.0887488022532564	5.11301750649925	3.33085834215203	0.0679913828028385	0.555600277549595
Esyt1	Esyt1	-0.191066890603514	5.041281427037	3.33079151798574	0.0679941452136487	0.555600277549595
Tceal5	Tceal5	-0.105124741335503	4.78353427409608	3.33071652995361	0.0679972448359566	0.555600277549595
Dram1	Dram1	0.170935204719278	1.46012321719507	3.32913224223107	0.0680627756864633	0.555771761937174
Slc66a2	Slc66a2	0.0849423540902609	5.61345504680186	3.32796376090918	0.0681111505134821	0.555803024824381
Kbtbd3	Kbtbd3	0.0935790999674643	3.73320441954174	3.32465782935979	0.0682482145200789	0.556529468897587
Runx1t1	Runx1t1	0.109920249969616	6.74131078870111	3.32169850090699	0.0683711585633967	0.556529468897587
Vangl2	Vangl2	0.0714847718226724	5.91780447106249	3.3208230378054	0.0684075746957162	0.556529468897587
Vsig10	Vsig10	0.194878878023761	1.51326506541445	3.32035419626901	0.0684270853554199	0.556529468897587
Sned1	Sned1	0.29191412172254	3.86421875215784	3.31976510449211	0.0684516086205273	0.556529468897587
Zfp507	Zfp507	0.0769718158419724	4.89065867282654	3.31922624328786	0.0684740490727786	0.556529468897587
Ak5	Ak5	-0.129774950765252	6.02509781086658	3.31830559281053	0.0685124070487246	0.556529468897587
Wnk3	Wnk3	0.106961362260584	5.2650035680069	3.31662521954388	0.0685824773834488	0.556668012432034
Lrba	Lrba	0.112559901247122	4.74059915615119	3.3157575005347	0.0686186890640539	0.556668012432034
Phc2	Phc2	0.0559759574482652	6.98678445610486	3.31035261709238	0.0688374480440548	0.557110576139242
Cd180	Cd180	0.273805049297663	0.625183885083286	3.30890823531588	0.068905255885355	0.557110576139242
Pank1	Pank1	-0.0844317138205496	4.46557312333133	3.30852365254783	0.0689213842973055	0.557110576139242
Arl5c	Arl5c	-0.285053734274194	-0.191862962161665	3.30703481550513	0.0689838603895804	0.557110576139242
Tgfb1i1	Tgfb1i1	-0.080650881603201	5.23424802105845	3.30682616115764	0.0689926209854046	0.557110576139242
Dpf2	Dpf2	0.0641582264156756	5.95861152657799	3.30667090489681	0.0689991403902274	0.557110576139242
Slc25a33	Slc25a33	-0.0745762657845276	4.50858689944922	3.30613568862748	0.0690216219109828	0.557110576139242
Wnt5a	Wnt5a	0.130976909056049	3.9456820430657	3.3054968578362	0.0690484513655065	0.557110576139242
Ca12	Ca12	0.326528672748618	1.69708400903189	3.30486304567246	0.0690751030926831	0.557110576139242
Samd4b	Samd4b	0.0498209580686512	6.62157209748067	3.29884421450767	0.0693286481920213	0.558783745520182
Stx6	Stx6	0.0542058066708684	6.90035084120397	3.29781446341789	0.0693721264575428	0.558783745520182
Akr1b10	Akr1b10	-0.183159114058494	3.22905485640596	3.29527300641242	0.0694795570795426	0.559070190847586
Nkd2	Nkd2	0.0787498853162115	5.64267605534728	3.29437711032494	0.0695174701852468	0.559070190847586
Retsat	Retsat	0.291543064275126	5.00288097974098	3.29380344820087	0.0695502283338822	0.559070190847586
Cercam	Cercam	-0.145037192716741	2.40268477213272	3.29273732812747	0.069589820444522	0.559070190847586
RGD135	RGD135815	0.102144546110986	3.11168749349375	3.28999885178818	0.0697032070859258	0.559936351171836
Hexb	Hexb	0.0836550794792352	5.09621945025971	3.28475162066091	0.0699262078580013	0.559936351171836
Diaph1	Diaph1	0.0632817597560156	6.45801740787448	3.28473919082368	0.0699267370844724	0.559936351171836
Ntng2	Ntng2	0.129829145488436	6.62509759282125	3.2844986029612	0.0699369741271382	0.559936351171836
Mrpl49	Mrpl49	0.0822683483021135	4.72098117854841	3.28444651976194	0.0699392001424188	0.559936351171836
Cyb5r4	Cyb5r4	0.0621676831778331	5.21482928946827	3.2840876253024	0.0699544965216129	0.559936351171836
Rps27a	Rps27a	-0.0716640194380242	8.62649493844318	3.28258269954127	0.0700186551238048	0.559936351171836
Blvra	Blvra	0.0650942060864458	5.5622111994257	3.28187289936537	0.0700489493087653	0.559936351171836
Derl3	Derl3	0.179939125376895	2.25279602902178	3.27921247289387	0.0701625884586876	0.559936351171836
Zmynd8	Zmynd8	0.0643170778223568	6.94767258610362	3.27814748963623	0.070208134121813	0.559936351171836
Stk11	Stk11	-0.0485375986402648	6.93079573173863	3.27583304793587	0.0703072240208377	0.559936351171836
Mia2	Mia2	0.074231254648808	4.43225356412139	3.27558771854046	0.0703177362649768	0.559936351171836
Nptx2	Nptx2	-0.115069773573469	4.95035070240109	3.27264836030204	0.0704438173082497	0.559936351171836
Enpep	Enpep	0.301708722786602	1.79182219829747	3.27076325023307	0.0705248047719401	0.559936351171836
Pomc	Pomc	0.525952713671596	1.58452396248145	3.27018437531567	0.0705496942103563	0.559936351171836
Colq	Colq	-0.436460330253686	-0.036358191530709	3.26957243953844	0.0705760153711426	0.559936351171836
Hdhd2	Hdhd2	-0.0675958876756737	5.21325420223221	3.26766011615161	0.0706583378534378	0.559936351171836
Ech1	Ech1	0.0749758930467292	5.29014582734609	3.26549783403411	0.0707515445829491	0.559936351171836
Cntn1	Cntn1	-0.0778628822210326	8.60976798407343	3.2649941890136	0.0707727049756561	0.559936351171836
RGD156	RGD156516	0.124981273258935	3.04774807971853	3.26282386674422	0.0708668900589815	0.559936351171836
Kif5c	Kif5c	0.0823847548090862	5.90806614382107	3.26173737912032	0.0709138555605725	0.559936351171836
Zbtb45	Zbtb45	0.059857419496526	5.62054309446154	3.26168490250965	0.0709162249062645	0.559936351171836
Kcna2	Kcna2	0.113105087402551	3.82378381207395	3.26076235844623	0.0709561203377066	0.559936351171836
Myh1	Myh1	-0.247979806528149	0.928033271960266	3.26032065480345	0.0709748865850805	0.559936351171836
Rab6b	Rab6b	-0.0782600270472414	10.0540558615381	3.25910173388006	0.0710280249774633	0.559936351171836
Ptp4a2	Ptp4a3	-0.0680959049927076	5.74933211176877	3.25750934621171	0.0710970279818723	0.559936351171836
Kremen1	Kremen1	0.18659884361164	2.55192721789443	3.2567186056617	0.0711313348363136	0.559936351171836
Tab2	Tab2	0.0588701960582802	6.69172299919003	3.25641029081923	0.0711447122042498	0.559936351171836
Myo5b	Myo5b	-0.0845729889277061	5.52235834429079	3.25623359645148	0.0711610589871387	0.559936351171836
Tmc8	Tmc8	0.253201530451048	1.01192844363387	3.25478045001248	0.0712154736199006	0.559936351171836
Tbx15	Tbx15	0.419589085223734	0.260454497750899	3.25332960413036	0.0712785272141974	0.559936351171836
Ywhab	Ywhab	-0.0511733645251826	9.82192159476188	3.25306440133285	0.0712900583628568	0.559936351171836
Tmem22	Tmem229a	-0.0846248203169543	5.60593047464414	3.25297428835613	0.07129397831449	0.559936351171836
Nrn1	Nrn1	-0.148234324013629	5.40613344469718	3.25253277562687	0.0713131282343519	0.559936351171836
Islr	Islr	0.358276167251979	4.74127752961195	3.25150215638204	0.0713580230282177	0.559936351171836
Zfp580	Zfp580	0.110584396683645	3.02674895403574	3.2489292190204	0.0714701336129687	0.559936351171836
Psma3	Psma3	-0.0622931175968076	5.76597925338013	3.2488497819377	0.0714735976179147	0.559936351171836
Uqcrfs1	Uqcrfs1	-0.071134849713046	7.08890095160596	3.24643688144931	0.0715789112518368	0.559936351171836
Pik3r1	Pik3r1	0.0647631635484056	6.69309294048266	3.24626122802972	0.0715865749533851	0.559936351171836
Utrn	Utrn	0.1053138854439	5.26615473620743	3.24622759600383	0.0715880645176605	0.559936351171836
Gyg1	Gyg1	-0.0780604836120857	5.07748609671697	3.24525700426484	0.0716304557789451	0.559936351171836
Ret	Ret	-0.196760870455124	4.22316740577203	3.24409912844988	0.0716548453232435	0.559936351171836
Fxyd5	Fxyd5	0.163633735184253	2.69900343983459	3.24374861423669	0.0716964209704363	0.559936351171836
Nsun7	Nsun7	-0.173649550131436	1.49193746842438	3.24351525588455	0.07170662055791	0.559936351171836
Slc6a4	Slc6a4	-0.454628363664286	3.19380037975581	3.24321806605799	0.0717196283537199	0.559936351171836
Foxc2	Foxc2	0.352423606829018	2.04579737064691	3.24311517241708	0.0717241421766124	0.559936351171836
Syt17	Syt17	-0.148551507379796	4.82456139489937	3.24244874248592	0.0717533184394596	0.559936351171836
MGC116	MGC116121	0.0889767257322271	4.21193252573884	3.24184632033534	0.071775324615381	0.559936351171836
Rsph1	Rsph1	-0.16488647863464	2.62755010434872	3.23495836817963	0.0720821354290424	0.560956677650905
Tacr3	Tacr3	-0.203786410560038	1.75898889346158	3.2349352577079	0.0720831524397578	0.560956677650905
Rapgef5	Rapgef5	0.0723357904908762	6.84712138418122	3.23412155298092	0.0721188705366319	0.560956677650905
Fbxo2	Fbxo2	-0.15313893403875	3.08161765885277	3.23319881761154	0.0721596110947806	0.560956677650905
Spock3	Spock3	-0.140638605900914	6.26326206245227	3.22957208068733	0.0723195836257495	0.561658744265763
Bop1	Bop1	0.0660477913159221	6.46889264505558	3.22911088881445	0.0723399536775732	0.561658744265763
Stard13	Stard13	0.129248390739447	2.9942188136049	3.22807474987879	0.0723857405951883	0.561664729730202
Ogfr	Ogfr	-0.0537770763751945	6.65890790896457	3.22544684331195	0.072502002628659	0.561859804464097
Adhfe1	Adhfe1	-0.223057082789703	1.02292324479098	3.22467188273617	0.0725363274316185	0.561859804464097
Rogdi	Rogdi	-0.0586781512189752	6.35582974728883	3.22422239408734	0.0725562132914064	0.561859804464097
Gpd1	Gpd1	-0.141405784640605	3.197291716743	3.22230202468366	0.0726414065099053	0.561859804464097
Ep400	Ep400	0.0918765104239615	7.63662326093549	3.22228642296133	0.0726420988593554	0.561859804464097
Tuba8	Tuba8	-0.238210285288179	0.877728638659433	3.22059560465914	0.0727171668706843	0.561859804464097
Dtx4	Dtx4	0.0916136125269005	5.75522692875368	3.22039445887879	0.0727261027658505	0.561859804464097
Cplx3	Cplx3	0.280651929586659	3.02383516636726	3.21855681865804	0.0728077944329527	0.562142853428187
Hook3	Hook3	0.126809911236482	4.39727539585744	3.21660842938975	0.0728945170789548	0.562464372042364
Chchd4	Chchd4	-0.17593595609299	3.99298402204037	3.21390162890366	0.0730151801499556	0.562948458422506
Cnr1	Cnr1	0.079680298828965	8.56308635909893	3.21317760804364	0.0730474917197769	0.562948458422506
Hist3h2a	Hist3h2a	0.116538118251511	5.1348701485267	3.21104486791528	0.0731427607324215	0.563324707196557
Ctxn3	Ctxn3	-0.32493657610739	-0.085090619985644	3.20904236938972	0.0732323232381661	0.563676632827019
Sinhcaf	Sinhcaf	0.123312654037268	3.99348449848599	3.20616047006132	0.0733614483116349	0.564193000332972
Plppr1	Plppr1	0.0542471948544264	6.38378569311685	3.20546542524682	0.0733926238600353	0.564193000332972
Pitpna	Pitpna	-0.0419737956787243	8.10196296720567	3.20451952590798	0.0734350751415202	0.564193000332972
Epb41l4b	Epb41l4b	-0.213963959579446	2.35604787289946	3.20346231855405	0.0734825525301457	0.564210343334541
Hcn1	Hcn1	0.0877964271518	4.3173594632488	3.19604022189046	0.0738167951909122	0.565692494164918
RT1-S2	RT1-S2	0.712209324474544	-0.033216269434346	3.19571834973436	0.0738313270749342	0.565692494164918
Pard6g	Pard6g	0.10397608953817	3.60675936878771	3.19515119105429	0.0738568406281316	0.565692494164918
Cubn	Cubn	0.277742860340958	0.902436632517579	3.19461760154748	0.0738810468662671	0.565692494164918
Ptprj	Ptprj	0.0749082737115164	6.26295977255526	3.19414773094999	0.0739022814369493	0.565692494164918
Sec24d	Sec24d	0.115448304376316	3.88002795887081	3.19105292994202	0.074042306652001	0.566079239327555
Agbl4	Agbl4	-0.160490452190798	1.67643526580999	3.19102557485212	0.0740435456510529	0.566079239327555
Tmem15	Tmem151b	0.0698038830925327	6.45172038682455	3.18669112312464	0.0742401412994553	0.566964490323484
Prkag2	Prkag2	0.0478634183158752	6.56963048244622	3.18607658314538	0.0742679695716471	0.566964490323484
Erp44	Erp44	0.0593552028430724	5.12610025574304	3.18481555215357	0.0743253849894366	0.566964490323484
N4bp3	N4bp3	0.106121642517665	4.12339796855634	3.18383650936634	0.0743699234773415	0.566964490323484
Sorl1	Sorl1	0.101246374082843	6.32769395050034	3.18352848976031	0.0743839417880525	0.566964490323484
Scgb1c1	Scgb1c1	-0.263648766583013	-0.303071454210612	3.1802090041266	0.0745351954177074	0.567532082376894
Marchf3	Marchf3	-0.130130362128296	2.82521965210669	3.17890837712319	0.0745945490124404	0.567532082376894
Pkd2	Pkd2	0.0841733046790063	5.51925952632188	3.17884805288946	0.0745973031162186	0.567532082376894
Tspan8	Tspan8	0.263435881206896	1.30624359778107	3.17689108592441	0.0746867077239231	0.567864748565606
Slitrk5	Slitrk5	0.0452459868859589	6.56064487949746	3.17323021235579	0.0748542649803124	0.568477178188703
Mgll	Mgll	-0.117082246897415	4.56145045452954	3.17314042247841	0.0748583797198075	0.568477178188703
Micu2	Micu2	-0.0927573674490263	6.85606142770287	3.17165343634414	0.0749265579839154	0.568603689529867
Gprc5b	Gprc5b	0.130639534787573	4.14992836084928	3.17026537536833	0.074990260746701	0.568603689529867
Rnmt	Rnmt	0.0566605798915259	6.2775294601582	3.16979729861382	0.0750117554673528	0.568603689529867
Dennd11	Dennd11	0.0989541273180995	4.57733816166503	3.16758521213437	0.0751134270733387	0.568889320246399
Sstr1	Sstr1	0.113843428273431	7.10976815017699	3.16689396982135	0.0751406267344766	0.568889320246399
Atp6v0c	Atp6v0c	-0.0752099688626337	9.56123339291173	3.16449882174138	0.0752555676430087	0.569389456398965
Crem	Crem	0.109607140134391	3.34177856474711	3.16357857933949	0.075297956484585	0.569389456398965
Arhgap2	Arhgap28	0.141238622789609	3.41686463774944	3.16166888525422	0.0753860806709664	0.569710556348666
Tead3	Tead3	0.165863687817013	2.06874972576113	3.15746904225634	0.075580275505164	0.570832383461217
Tbc1d19	Tbc1d19	0.063553546580708	5.09570755555863	3.15541734004461	0.0756753287229056	0.570941366648247
Acsl6	Acsl6	-0.0714515727928622	5.76785314688079	3.1543428751326	0.07572517405685	0.570941366648247
Xpnpep	Xpnpep3	0.100104279388693	3.52257424814936	3.15419611414525	0.0757319837644097	0.570941366648247
Bdp1	Bdp1	0.163073483241764	2.206530967886	3.15281729716738	0.0757959933106536	0.571078809889508
Sbno1	Sbno1	0.0582522698137035	6.76949327988822	3.15090001464443	0.0758850969481632	0.571320341179011
Slc24a3	Slc24a3	-0.0970761054684274	7.04544412909404	3.15015777071277	0.0759196221587561	0.571320341179011
Bcor	Bcor	0.0730818977116703	5.77671313746324	3.14707669702251	0.0760631178151767	0.572055166352201
Ier3	Ier3	0.31364133259454	1.75377915322622	3.14142292325758	0.0763271914721517	0.572895402408456
Fzd3	Fzd3	0.1071233896551092	5.64787878583363	3.13796402972272	0.076489223610016	0.574567229230686
Ap1s2	Ap1s2	-0.109824509915062	6.0366010784314	3.1348921885595	0.0766334532892927	0.575081980428056
Klf2	Klf2	0.152929168866319	4.25624263711513	3.13319176528989	0.0767134120890705	0.575081980428056
Kif13b	Kif13b	0.118254286288548	4.57408089149449	3.1316012842938	0.0767854512179568	0.575081980428056
LOC500	LOC500877	0.154284191861075	1.84800979190802	3.13164509653818	0.0767862188406731	0.575081980428056
Lyrm2	Lyrm2	0.0731457238710764	4.62500249328721	3.13115209349026	0.0768094416661721	0.575081980428056
Arrb2	Arrb2	-0.0520982516450322	5.48121841211739	3.12856615701035	0.0769313758026423	0.575081980428056
Gstz1	Gstz1	0.0884981015331674	4.27435924952821	3.12773913536745	0.0769868098300061	0.575081980428056
Tk2	Tk2	-0.0712947073757342	5.02771840820881	3.12719118525274	0.0769962943341632	0.575081980428056
Acot11	Acot11	0.372105642734015	-0.595605654306361	3.1266002197904	0.0770242145452114	0.575081980428056
Metap1d	Metap1d	0.0958399700478424	3.53868180561896	3.12444090413724	0.0771260402905011	0.575081980428056
Smtn	Smtn	0.0909951654584977	3.74251485541804	3.12422557042441	0.0771365148880152	0.575081980428056
Lxn	Lxn	-0.0999589444136796	5.36524120417101	3.12375122305986	0.0771589685292645	0.575081980428056
Pdia4	Pdia4	0.0966144081035616	6.10137585402407	3.12315744003368	0.0771870856355549	0.575081980428056
Lmntd1	Lmntd1	0.391846565178559	-0.706133546863141	3.12279177100605	0.0772044064655215	0.575081980428056
Hspa8	Hspa8	-0.0651311460331244	6.21232854818121	3.12184904685852	0.0772491276490681	0.575081980428056
Pkm	Pkm	-0.0432007180260886	8.90402197234659	3.11848787742504	0.0774085857146727	0.575437316986686
Shisa8	Shisa8	-0.171816338869845	3.20358387141129	3.1178512875596	0.0774388252008859	0.575437316986686
Styxl1	Styxl1	0.248727615297492	0.279728074653371	3.11664582356532	0.0774961460753303	0.575437316986686
Spata31	Spata31	0.100357911821873	3.29464621356718	3.11514406638959	0.0775676191066534	0.575437316986686
Crabp2	Crabp2	0.218040697397862	2.34877012337953	3.11421943600251	0.0776116570316523	0.575437316986686
Hdac11	Hdac11	-0.0770988850880335	6.11713600414023	3.11138439373433	0.0777468510837582	0.575437316986686

Tbc1d5	Tbc1d5	0.0821048857808889	5.454490350237	3.10981627484069	0.0778168605365426	0.575437316986686
Nfatc4	Nfatc4	0.194202297497328	2.21024710505835	3.1089940511047	0.0778610357688699	0.575437316986686
Cux1	Cux1	0.0707881338571297	7.10541403012875	3.10845850163614	0.0778866432632	0.575437316986686
Fbxo44	Fbxo44	-0.0582773835025466	5.87963268313794	3.10686132648321	0.077972642121006	0.575437316986686
Kpnb1	Kpnb1	0.0645474077946405	7.81218105609329	3.10610381063269	0.0779993412696587	0.575437316986686
Ccdc88	Ccdc88	-0.324197555030289	-0.321847567735319	3.10458667188048	0.0780720461043355	0.575437316986686
Gpi	Gpi	-0.0549712368018857	8.31688256011382	3.10386658706727	0.078106579776677	0.575437316986686
Klf9	Klf9	-0.155508326013042	1.75173382319371	3.10323282627685	0.0781368871444855	0.575437316986686
Elovl5	Elovl5	0.0594587167472485	5.23395891572992	3.1032220600332	0.0781375038108498	0.575437316986686
Sowahc	Sowahc	0.0610707173820877	4.96358587079017	3.10282967052222	0.0781515368357596	0.575437316986686
Ticam2	Ticam2	0.179889192905666	1.06501909430532	3.10264980704809	0.0781649712236127	0.575437316986686
P2ry14	P2ry14	0.324019472998941	-0.0187522172178826	3.10257801781137	0.0781684177490385	0.575437316986686
Mdfic	Mdfic	0.166866229827532	3.03731368746164	3.10247968729516	0.0781731287587107	0.575437316986686
Sptlc2	Sptlc2	-0.0508958630491822	6.21630291767154	3.10074052358263	0.0782568898095771	0.575712686473286
Myadm	Myadm	0.0802050555443414	4.86580952226735	3.09950910440428	0.0783158061261409	0.575808815990489
Eprs	Eprs	0.0632895520567863	7.99253175541667	3.09762506435198	0.0784065992755625	0.576126124358778
Tspan14	Tspan14	0.0845849228568427	6.40974795855129	3.09343412218706	0.0786087469262933	0.576822959153223
Cyp46a1	Cyp46a1	-0.0864563879250456	5.70654255737765	3.08997603720182	0.0787759682190091	0.576822959153223
Clec10a	Clec10a	0.568284871081924	-0.453272806716619	3.08995401687723	0.0787770342740307	0.576822959153223
Alpl	Alpl	0.100312472791088	4.28588949806768	3.08961462224169	0.0787934671212635	0.576822959153223
Kank3	Kank3	0.103762775019767	3.81254414294591	3.08957932515034	0.0787951763516727	0.576822959153223
LOC688801	LOC688801	-0.235904084309497	0.151441250039468	3.08642012583925	0.0789483196277768	0.576822959153223
Cks1b	Cks1b	0.0805032468577849	4.52110247703322	3.08542231546014	0.078996755418654	0.576822959153223
Pogk	Pogk	0.0587955755448622	6.92530592226484	3.08432676497319	0.0790499725823698	0.576822959153223
Iscu	Iscu	-0.0802719723407135	5.35648358965029	3.0834675454492	0.0790917368151285	0.576822959153223
RGD1311066	RGD1311066	-0.215841214816895	0.722758161612355	3.08326518717864	0.0791015763262109	0.576822959153223
Btd	Btd	-0.0914352925888997	4.13041312673367	3.0831989088448	0.0791053829159018	0.576822959153223
Tmem242	Tmem242	-0.0882175680532596	4.28022575682514	3.08265906548909	0.079131056445832	0.576822959153223
Ebf4	Ebf4	-0.139461002206419	4.66634175709597	3.08131281087503	0.0791965769469986	0.576822959153223
Cnp	Cnp	-0.0936801065401252	6.42771349187741	3.08029313423273	0.0792463422075729	0.576822959153223
Osbpl6	Osbpl6	0.106451116944684	5.47508613204533	3.07938801071037	0.079290356053677	0.576822959153223
Alkbh4	Alkbh4	0.0934789749069866	3.41435264776638	3.07916441672348	0.0793012576352593	0.576822959153223
[illegible]	[illegible]	[illegible]	[illegible]	[illegible]	[illegible]	[illegible]

Pvalb	Pvalb	-0.711161831991435	-0.533732152253079	2.76445529712893	0.0963794881728931	0.595310333630291
Kcnj16	Kcnj16	-0.265499369179741	1.47993509142776	2.76312971441818	0.0964593637996732	0.595407070152767
Gabbr1	Gabbr1	-0.0557451825752685	10.0751694785213	2.76054589293798	0.0966152584895892	0.595407070152767
Wfdc2	Wfdc2	0.0985298946723799	3.17119349170823	2.75831880236347	0.0967498553416533	0.595407070152767
Pstk	Pstk	-0.118591017628697	3.71755199488029	2.75804943701725	0.0967662546537355	0.595407070152767
Fam53a	Fam53a	0.0960311844171692	3.22290596039503	2.75749654966462	0.0968002120141413	0.595407070152767
Atp5mf	Atp5mf	-0.0876981391164725	6.6956017211035	2.75702380123741	0.0968282202777306	0.595407070152767
Tmsa-ps	Tmsa-ps1	-0.284595903327739	-0.156132836355195	2.75682803529828	0.0968400718452073	0.595407070152767
Pou3f1	Pou3f1	0.121020608175714	7.02719869183129	2.75679734397723	0.0968419300250701	0.595407070152767
Fzr1	Fzr1	-0.0456462395446114	7.09681420512229	2.7565505143237	0.0968613917112631	0.595407070152767
Dgat2	Dgat2	-0.0832556376882992	5.02010416121752	2.75459052726549	0.0969756417684696	0.595407070152767
Npr3	Npr3	0.223986786604563	3.05567787907942	2.75454785885867	0.0969782229794517	0.595407070152767
Smarcc1	Smarcc1	0.0512782226904298	7.08579063918976	2.75441917319066	0.0969860325832503	0.595407070152767
Pla2g4b	Pla2g4b	0.197564055612016	4.62862422388946	2.75350841577523	0.0970412807710033	0.595407070152767
Pex10	Pex10	0.0973331910714059	3.15914339311328	2.75314666395171	0.0970632348073027	0.595407070152767
Ndufb8	Ndufb8	-0.0852679634622254	7.0357757919026	2.75229802543669	0.0971147583295426	0.595430388048011
Gjd2	Gjd2	-0.117258805232627	4.55691822310194	2.75123473486831	0.0971793561374864	0.595518074496717
Ca14	Ca14	0.249630607158969	1.62520802860919	2.75049181190597	0.0972245185340676	0.595518074496717
Cyba	Cyba	0.18955496739789	2.47232034415774	2.74922689768161	0.097301465527342	0.595696950507909
Cwf19l1	Cwf19l1	-0.0629868595815332	5.56026240325131	2.74799362578343	0.0973765515477818	0.595864263688903
Klhl7	Klhl7	0.0509719035775735	7.54731171958526	2.7466771274222	0.0974567744314762	0.59606293067722
Vps39	Vps39	0.0689812651993884	5.94504891501534	2.74582595117366	0.0975086905296823	0.596088097547154
Sugct	Sugct	-0.298536749368223	-0.273327236006872	2.74468916392529	0.0975792716286592	0.596196710986403
Usp49	Usp49	0.08249528291575	5.01209812914999	2.74351736378609	0.0976496137546712	0.596196710986403
Nectin2	Nectin2	0.0590354325557686	5.20575026056865	2.74317897643591	0.0976702900742332	0.596196710986403
Mrpl2	Mrpl2	-0.0804003754068746	4.03980175237754	2.74140729282643	0.0977786223675891	0.596277659680417
Zar1	Zar1	-0.298895183932126	0.582382693144801	2.74084406456298	0.0978130892622463	0.596277659680417
Rgma	Rgma	-0.071200348678823	8.32641113169875	2.74062611687832	0.0978264301807978	0.596277659680417
Hrk	Hrk	-0.0999902891154432	5.55789620298782	2.73289264473952	0.0983010325231706	0.59826348694649
Rab4a	Rab4a	-0.054237356415115	5.67597338226606	2.73231062398457	0.0983369199484976	0.59826348694649
Pgr15l	Pgr15l	-0.428148326784735	-0.68236188861292	2.7317988882142	0.0983684214131291	0.59826348694649
Pik3ca	Pik3ca	0.0552959239043399	6.94831739899723	2.73118534148073	0.0984065264086836	0.59826348694649
[illegible]	[illegible]	[illegible]	[illegible]	[illegible]	[illegible]	[illegible]

Fkbp5	Fkbp5	0.0561655468927832	6.06922958950476	2.43097788036293	0.118958869217897	0.629718844222191
Aldh3a2	Aldh3a2	-0.0575277943422073	5.88681518738018	2.42989332950322	0.119040998732676	0.629754005367272
Kctd15	Kctd15	0.0636890573161701	6.29142670301342	2.42886544748495	0.119119084636746	0.629754005367272
Nsun4	Nsun4	-0.0871468264005976	3.61108726778139	2.42882777795004	0.119121847383553	0.629754005367272
Lrrc10b	Lrrc10b	-0.190163675254787	1.87391099189932	2.42823239803124	0.119167204189479	0.629754005367272
Msn	Msn	0.0960408039403805	4.76835264477368	2.42712150385347	0.11925168781978	0.629823714520489
Fgf13	Fgf13	-0.0582166680779007	7.91310048096515	2.42351111749642	0.119526758773096	0.630948889449398
Tspan1	Tspan1	-0.176472166681015	1.83238423440904	2.42327179230448	0.119545016803589	0.630948889449398
Sfrp2	Sfrp2	-0.0932247607968277	3.89634692854266	2.42213244718221	0.119631679357646	0.631103393898035
Plscr2	Plscr2	0.225309932194122	0.96164400287513	2.42154908068595	0.119676522031887	0.631103393898035
Nt5dc2	Nt5dc2	-0.0937815055321567	6.15291202099851	2.42058769831394	0.11974999921028	0.631103393898035
Wdr5	Wdr5	0.0409016005669652	6.25982671202058	2.4202385113259	0.119776615913324	0.631103393898035
Fads6	Fads6	-0.117981458538689	3.49180307090146	2.41732550240576	0.119999649708884	0.631157792589093
Setmar	Setmar	0.163614700178753	1.30233399489885	2.41716140925246	0.120012222815062	0.631157792589093
Epn3	Epn3	0.246671164243033	1.66695559104057	2.41688207702181	0.120025865234063	0.631157792589093
Gata2	Gata2	0.237061313967843	1.77342012994264	2.41626403912082	0.120081006695048	0.631157792589093
Cck	Cck	-0.10731430973428	6.67490561830634	2.4158974547195	0.120109118132799	0.631157792589093
Nle1	Nle1	-0.0702532290982479	4.16316318817411	2.41540836501232	0.120146625206846	0.631157792589093
Twf2	Twf2	0.0733025023629702	5.09785701728286	2.41501862738771	0.120176540474363	0.631157792589093
LOC300(	LOC300308	0.622201276478042	-0.500256581109855	2.41482206992404	0.120191625807581	0.631157792589093
Zdhhc14	Zdhhc14	-0.0738028395297576	5.23512545487691	2.41233362893805	0.120382768572735	0.631596668761032
Skiv2l	Skiv2l	-0.0462534915235601	5.79227348822264	2.41075144988221	0.120504508362756	0.631596668761032
Ankrd40	Ankrd40	0.0524633001624341	5.54233560629426	2.41059152686738	0.120516818253472	0.631596668761032
Atp6v1c	Atp6v1c1	-0.0438198546186658	6.64277802070673	2.40950985766115	0.120600121055654	0.631596668761032
Mtmr6	Mtmr6	-0.0352147572277325	7.14837316003017	2.4092047594862	0.120623628952651	0.631596668761032
Grp	Grp	-0.144503225976226	3.33722391566209	2.40834412932313	0.120689967322726	0.631596668761032
Tmem81	Tmem81	0.228689944028982	0.0399839880958582	2.40797095288801	0.120718745859036	0.631596668761032
Fen1	Fen1	-0.0682415799173144	3.992951879517	2.40785527172498	0.120727688223782	0.631596668761032
Ufl1	Ufl1	0.0533019975910441	4.59853921735932	2.40781481171521	0.12073078905146	0.631596668761032
Xrcc5	Xrcc5	0.0730924898629748	4.41397875931422	2.40663668644101	0.12082170094714	0.631807380736572
Armc10	Armc10	0.0486815698644091	6.61666622426225	2.40548399804618	0.120910723363512	0.631867057300993
Sf1	Sf1	0.0448992673542042	8.03481352634904	2.40517755934094	0.120934401926326	0.631867057300993
Cyp2j3	Cyp2j3	0.412442596822	-0.528024760851085	2.40344791427229	0.121068148213543	0.632071077609042
Ube2v2	Ube2v2	-0.0459441218956261	6.41240226850387	2.40336236531957	0.121074767611254	0.632071077609042
Sars2	Sars2	-0.067239644541607	3.59951102183698	2.40023284531104	0.121317191813735	0.632251286726131
Fbn1	Fbn1	0.120581377999789	5.19117901188281	2.40015406212247	0.121323300138308	0.632251286726131
Cdc7	Cdc7	0.0724965278888877	4.78564154989406	2.39872922515638	0.121433859042185	0.632251286726131
Hpca	Hpca	-0.0914066667934785	7.04597115380169	2.39840887539725	0.121458731328283	0.632251286726131
Slc45a3	Slc45a3	0.252692359130721	0.177553697297466	2.39833464907494	0.121464495142142	0.632251286726131
Ndufb2	Ndufb2	-0.0965669885249657	5.82262692682435	2.39635344515858	0.121618451439428	0.632251286726131
Rilpl1	Rilpl1	0.0743376398838921	4.35490265282621	2.39585895484472	0.121656811235368	0.632251286726131
Mex3c	Mex3c	0.0566967023194926	6.08967824944517	2.39555968933027	0.121680193649536	0.632251286726131
Lcmt2	Lcmt2	0.0890749808671656	3.12241659759881	2.39551880243116	0.121683374973191	0.632251286726131
Wnt5b	Wnt5b	0.230488752348876	1.0509173814564	2.39483752598617	0.121736397233118	0.632251286726131
Rpl23	Rpl23	-0.0690200272361426	7.69785224732357	2.39440100340656	0.12177038428767	0.632251286726131
Spata2	Spata2	0.0529014604069881	5.90800892890957	2.39410470324353	0.121793459810485	0.632251286726131
Pde3a	Pde3a	0.108290384798319	3.80296444492223	2.39384956974948	0.121813334761173	0.632251286726131
Tor1aip2	Tor1aip2	0.0489653498233068	5.82558702816057	2.39378050064568	0.121818713899674	0.632251286726131
Aptx	Aptx	0.0689222240619424	4.39405380685652	2.3925473075817	0.121914827751308	0.632487029460736
S1pr3	S1pr3	0.112636721961438	3.75435657190803	2.39120873195324	0.122019250175645	0.632540382704252
Ccdc159	Ccdc159	0.133383128391917	2.75213713641468	2.391115782377	0.122026504862478	0.632540382704252
Commd7	Commd7	-0.0781436427743297	5.12500571683571	2.3898213538646	0.122127594338362	0.632578379769904
Spopl	Spopl	0.0918283845000116	3.03458140989158	2.38972343798522	0.122135234179954	0.632578379769904
Rab5a	Rab5a	-0.0506216203973458	5.75343895432482	2.38729470802525	0.122325152881901	0.633299142171058
Mtap	Mtap	0.104142185520017	2.596100170598	2.38621940020694	0.122409342989609	0.633399639773954
Colgalt1	Colgalt1	0.0459265918170194	5.87490243742662	2.38575024300095	0.122446095306145	0.633399639773954
Fblim1	Fblim1	0.143511901637348	3.09403928717276	2.38339441792762	0.122630828361153	0.633953064249954
Ldlrad4	Ldlrad4	0.0836991854702387	4.42065886350249	2.38285989652708	0.122672801801788	0.633953064249954
Mrpl1	Mrpl1	-0.0729573903329587	3.9306168916133	2.38176733281863	0.122758597414029	0.633953064249954
Chst5	Chst5	-0.137715609800011	2.12304400388442	2.38130145881353	0.122795207905722	0.633953064249954
Nmnat1	Nmnat1	0.113302278654924	2.72639236923908	2.38114978776265	0.122807129621875	0.633953064249954
Tm9sf1	Tm9sf1	0.0633832746177472	4.82775306028968	2.37901919188615	0.122974734335894	0.634555732137695
Stmn2	Stmn2	-0.0595098932819663	11.2587341251754	2.37495817887816	0.123294899835486	0.635864797539215
Ncoa1	Ncoa1	0.0638434708527499	7.76829932677698	2.37372938351599	0.123391958797857	0.636023023988106
Fbxo30	Fbxo30	0.0596808271678107	5.47111194232786	2.37336812372192	0.123420509780222	0.636023023988106
Smad2	Smad2	0.0520782555135531	5.95463740994214	2.37281126451788	0.123464523680413	0.636023023988106
Tpm4	Tpm4	0.0780925304577223	5.89357124898965	2.37219822288875	0.123513968670843	0.636023023988106
Slc39a8	Slc39a8	0.119393667438635	3.35183465827679	2.37042082291774	0.12365371331258	0.636479942657201
Tmem20	Tmem200c	0.0950487700738224	4.96551684717588	2.36942794573193	0.123732385313234	0.636622256310607
Ttc9b	Ttc9b	-0.0884616617382415	7.15593830647841	2.36851310193549	0.123804922679127	0.636656377102589
Trip10	Trip10	0.103152788205617	3.30155356855034	2.36805743594002	0.123841069746572	0.636656377102589
Pusl1	Pusl1	0.0952564930258354	3.69220734587583	2.36736992620339	0.123895702127658	0.636674907515153
Scel	Scel	-0.302728014393408	-0.546579149605573	2.36376138252295	0.124182443542314	0.637440300632098
Ccdc51	Ccdc51	0.0867214741668042	3.55213800825726	2.36348779950358	0.12420421840357	0.637440300632098
Ptges3	Ptges3	-0.0454533849137585	8.07718371648348	2.36253175873647	0.124280344344568	0.637440300632098
Idi1	Idi1	0.0669562574966892	7.2532133682174	2.36231762357728	0.124297402224643	0.637440300632098
Ubtf	Ubtf	0.0344168571273752	7.75112469307272	2.36229385703326	0.124300092284836	0.637440300632098
Zfp322a	Zfp322a	0.0788916575023321	4.58097752636145	2.3585603435549	0.124517277934259	0.638190779096146
Socs3	Socs3	0.194452171225933	0.811657914370816	2.35890623270787	0.124569502516968	0.638190779096146
Gdap1l1	Gdap1l1	-0.0482328493410326	6.58922107340861	2.35768932192605	0.124666726104912	0.638190779096146
Il6st	Il6st	0.0588148228222675	6.22517356502493	2.35706251916794	0.124716826620382	0.638190779096146
Syt11	Syt11	-0.045174222700612	7.97625568914667	2.35687218769612	0.124748053452109	0.638190779096146
Sema4g	Sema4g	-0.0478113019307925	6.83716671345532	2.35607264642138	0.124796018780531	0.638190779096146
Zfyve9	Zfyve9	0.0411547817242221	6.57685838285873	2.35543805324922	0.124846810686741	0.638190779096146
Cabcoco	Cabcoco1	-0.23461875091777	1.09711522485765	2.35474691640378	0.124902154410544	0.638190779096146
Sfrp1	Sfrp1	0.0828766088200204	6.17101189385457	2.35468917858122	0.124906779077726	0.638190779096146
Gstm7	Gstm7	-0.0769206527419246	5.91461715914553	2.35275118227746	0.125062118538135	0.638667617116532
Kirrel3	Kirrel3	-0.0707275480470237	6.69614529748184	2.35224806196796	0.125102481063782	0.638667617116532
F2r	F2r	0.0584318655754701	6.04901346882381	2.35131250119863	0.125177574349231	0.638789609470492
Dsel	Dsel	0.0615036503629127	5.09178201137715	2.34915369010995	0.12535109673547	0.639133171961507
Alkbh8	Alkbh8	0.0882081897387757	4.06386639966823	2.34903657697362	0.125360461816254	0.639133171961507
Chpf2	Chpf2	-0.0652079631530836	5.25863611082798	2.34856276118178	0.125398573772683	0.639133171961507
Hbb	Hbb	0.230483195664126	6.76813843224366	2.34735856465133	0.12549549436979	0.639365979278018
Cbs	Cbs	-0.100082822912792	5.03553018664413	2.34497262941974	0.125687698538027	0.639822863199022
Ndst3	Ndst3	0.124795497179841	2.65711832047699	2.34497308506172	0.125687732443761	0.639822863199022
Glcci1	Glcci1	0.0693452439728905	5.382861175706	2.34423637147425	0.125747168431629	0.639864363967321
Lin54	Lin54	-0.0557291957250245	4.61319715870065	2.34343710340052	0.125811686473658	0.639921680445102
Magee2	Magee2	-0.0903088214857627	4.17674896269369	2.34068382711095	0.126034216655682	0.640577950345262
Tmem12	Tmem126a	-0.0809595115366017	3.59423107657201	2.34059472325689	0.126041425671044	0.640577950345262
Tyw1	Tyw1	0.0615215809891291	4.56242676151052	2.33829669852027	0.126227507581114	0.64125261856873
Rgs5	Rgs5	0.135485425747014	4.38096651352429	2.33768656439443	0.126276864320219	0.64125261856873
Git1	Git1	-0.0394401830367198	8.4276393223432	2.33704351230482	0.126329112671581	0.641256443776825
Nsd1	Nsd1	0.0753403077843128	8.08250877559766	2.33485854881273	0.126522730651914	0.641722931132793
Bcl6b	Bcl6b	0.161453337854059	2.29621794016759	2.33442678335867	0.126541563602299	0.641722931132793
Ppp1r16	Ppp1r16b	0.0776721152183972	5.05291682475739	2.33364044951823	0.126605483272468	0.641722931132793
Ctf1	Ctf1	0.145930502604982	1.49495224137201	2.33282725280458	0.126671624349143	0.641722931132793
Gstm2	Gstm2	0.265170591206152	2.29626636961894	2.33274674897373	0.126678174190917	0.641722931132793
Top1mt	Top1mt	0.0604309104733709	4.55515745654079	2.33202960910514	0.126736532763053	0.641758028275007
Kifc2	Kifc2	-0.103954999572215	7.94923744763178	2.32964913757493	0.126930484111609	0.642373123137037
Ppt2	Ppt2	0.0644978155254219	4.38576635152263	2.32927522860233	0.126960677931869	0.642373123137037
Mrpl53	Mrpl53	-0.0595088803594511	5.22264546676279	2.32758338538143	0.127099056630219	0.642429218375626
Gpr151	Gpr151	0.432702415812838	0.337742033721257	2.32745305969566	0.127109700005605	0.642429218375626
Eps15	Eps15	-0.0568728829436382	6.92483452502864	2.32724698017414	0.127126520332483	0.642429218375626
Ube2l6	Ube2l6	0.240975918859859	0.40397414215711	2.32605001970367	0.127224345622452	0.642526331431007
Ggn	Ggn	0.148185144696066	1.26545391388306	2.32486913705807	0.127320927421852	0.642526331431007
Mospd2	Mospd2	0.0912560295740201	4.08749976992511	2.32444499322344	0.127355627125935	0.642526331431007
Pcdhga3	Pcdhga3	-0.0862475088066858	4.36811907477062	2.32429880538704	0.127367619181687	0.642526331431007
Rpl24	Rpl24	-0.0645718193800946	8.02245000898094	2.32286358701851	0.127484223466403	0.642526331431007
Dctn5	Dctn5	-0.0472016257462784	6.12829006786331	2.32147778966328	0.127598752179313	0.643167387333385
Ncdn	Ncdn	-0.0517292752539193	9.15315421545827	2.32045201431476	0.127682918225072	0.643167387333385
B4gat1	B4gat1	-0.0536520090314282	6.56157851155074	2.32042677577031	0.127684889855317	0.643167387333385
Tent4a	Tent4a	0.0486192586540405	6.42095741995048	2.31940803288964	0.127768641549069	0.643329031722251
Mrps15	Mrps15	-0.0619165415618271	5.02288542585432	2.31755877388332	0.127920645044798	0.64383456564096
Rab3a	Rab3a	-0.0562870103639546	8.67610469419583	2.31674337387511	0.127987732396316	0.643912474640659
Bmpr2	Bmpr2	0.09202988670842	6.87365081766882	2.31602576424459	0.128046806410121	0.643950021585759
Fem1b	Fem1b	0.0502084736709896	7.17131833909649	2.31467956513692	0.128157708151611	0.643987204980313
Kdelr3	Kdelr3	0.184541022228936	2.13705311611246	2.31448734992233	0.12817355182908	0.643987204980313
Rnf130	Rnf130	0.0479083448530127	5.68833852587	2.31405687993158	0.128209041987098	0.643987204980313
Rps27	Rps27	-0.107241941226534	7.04861608968749	2.31180203995361	0.128386862617219	0.644620879225371
Nupr1	Nupr1	0.379106651171183	1.85921992358477	2.31043739760175	0.128507883866105	0.644756103690256
LOC100(	LOC100360	-0.221216918231853	-0.064290767718093	2.30976721329834	0.128563302571201	0.644756103690256
Zfp472	Zfp472	0.0930352723375617	2.94225917980843	2.30959193718129	0.128577800948288	0.644756103690256
Cxxc5	Cxxc5	-0.0584131508657586	6.12855615295655	2.30801602709323	0.128708236881513	0.644756103690256
Sh3bgrl	Sh3bgrl2	0.106524409677153	2.44796285110867	2.30789974200321	0.128712070085442	0.644756103690256
Lmtk2	Lmtk2	0.0514852880053223	8.01486366716777	2.30770276214212	0.12873418297039	0.644756103690256
Usp21	Usp21	0.0515418684805421	5.82939834465404	2.30720377122672	0.128775523795473	0.644756103690256
Rbbp5	Rbbp5	0.0516233058849706	5.36565679912919	2.30568450282239	0.128901484518906	0.645127887020763
Ehd4	Ehd4	0.0922483089162203	3.5559832762358	2.3045469778091	0.128995885203585	0.645179299725084
Cdkl3	Cdkl3	-0.0752075384352388	4.31000099016155	2.3029237672946	0.129130725241715	0.645179299725084
Coro6	Coro6	-0.247129537244882	1.09599039308111	2.3015264890748	0.129246824529629	0.645179299725084
Smo	Smo	0.102689139238129	4.08293388796416	2.301448593209	0.129253404111376	0.645179299725084
Icam5	Icam5	-0.118932282494215	5.13069489148262	2.30140734821938	0.129256864326027	0.645179299725084
Hacd1	Hacd1	-0.107670888896775	2.43521020475471	2.30129846688805	0.129257575012082	0.645179299725084
Lgals8	Lgals8	-0.0596918034000832	4.89496867247637	2.30076561184202	0.129310046386396	0.645179299725084
St8sia1	St8sia1	0.0850433616203387	4.33332120589123	2.30012312025966	0.1293637434244	0.645179299725084
Grm3	Grm3	0.0881852989076262	7.06208576540983	2.299525264339	0.129413547636017	0.645179299725084
Slit2	Slit2	0.0911487191517194	5.21747298316414	2.29934159650564	0.12942885206475	0.645179299725084
Setd2	Setd2	0.0596418478141382	7.29805507617697	2.29589952639167	0.12973274973175	0.646426909905371
Fastkd2	Fastkd2	0.0735204543837207	3.43292177332919	2.29326598092812	0.129950625159044	0.64719146151454
Rhog	Rhog	0.0941544751265402	3.40501233487806	2.29226509912175	0.130020051844976	0.647350433707012
Mapk8ip	Mapk8ip1	-0.0545325136931952	9.20578102544659	2.29047291379451	0.130170255687779	0.647826760756449
Qsox2	Qsox2	0.0711958996221463	4.80346445040398	2.2889440598342	0.130223006048723	0.647863878177798
Pgbd5	Pgbd5	-0.0590301449649652	5.58552976712543	2.2880195720435	0.130292202957923	0.647876524754147
Mvb12b	Mvb12b	0.0458996588709409	6.90501166809402	2.28818326757976	0.130362423484111	0.647876524754147
Rere	Rere	0.0976890018123999	7.62173503151774	2.28775185541743	0.130398678782352	0.647876524754147
Dock7	Dock7	0.0675569887875391	7.07389646579979	2.28729265592278	0.130437270983221	0.647876524754147
Golim4	Golim4	0.0712285582133298	4.45892951298496	2.28598167239313	0.130547519252298	0.64816609538835
Bloc1s2	Bloc1s2	-0.0660908933778644	5.6763996342246	2.28227313263105	0.130859655226079	0.648378114765856
Igf2	Igf2	0.316839414619188	7.2705799850095	2.2802288812163	0.131032525248217	0.648378114765856
Snx14	Snx14	-0.0932739250159456	4.83550737585681	2.28014332509591	0.13103976360999	0.648378114765856
Pcolce	Pcolce	0.29269937903242	2.91593869119596	2.28012884999504	0.131040986608759	0.648378114765856
Kdm5b	Kdm5b	0.0680062438289552	7.74119839286134	2.2799399936537	0.131056864215291	0.648378114765856
Atad2	Atad2	0.105063838982174	3.31725958802566	2.27978979684256	0.131069626793577	0.648378114765856
Fmnl1	Fmnl1	-0.0745476551933331	5.55215558846481	2.27966420944111	0.131080205774186	0.648378114765856
Prr16	Prr16	0.114189773998816	2.49317018719078	2.2796012513694	0.131085563564857	0.648378114765856
Tmem11	Tmem117	-0.0999538997159014	3.91401457238368	2.27913148516029	0.131125394464845	0.648378114765856
Acads	Acads	0.0813474616218642	4.1860066813613	2.27880702889745	0.13115272073742	0.648378114765856
Nalcn	Nalcn	-0.099622414293552	6.72482621899966	2.27809912338939	0.131161846731508	0.648378114765856
Osr1	Osr1	0.315919631000952	2.52942492652262	2.27737553026641	0.131273845859792	0.648621131252926
Cd48	Cd48	0.122978587828066	3.65727639019017	2.276805848705	0.131317002287801	0.648621131252926
Epc1	Epc1	-0.0507014221360753	6.4622919722278	2.2747543063896	0.131495862306786	0.64881194872966
Cpne4	Cpne4	-0.128298891013216	5.96083742579739	2.2746161069535	0.131507694640174	0.64881194872966
Ptgfr	Ptgfr	-0.237927302967176	0.514108011096489	2.27459336683002	0.131509611151504	0.64881194872966
Cbln2	Cbln2	-0.112153656335535	5.73557375681594	2.27387535845041	0.131570526128114	0.648855960185962
Capza1	Capza1	0.0453777526709884	6.52354343746109	2.27185773257283	0.131741899197937	0.649003689847406
Ppm1j	Ppm1j	0.134515782580942	3.01607001462997	2.27071617244333	0.131838965654128	0.649003689847406
Ccdc184	Ccdc184	-0.0585653949517876	6.20373691045218	2.26895012814155	0.131989288162603	0.649003689847406
Arhgef3	Arhgef3	-0.0772433959844271	3.88763012481913	2.26772696469495	0.132093515366486	0.649003689847406
Notum	Notum	0.173641685128369	1.52910591671566	2.2688490804449	0.13216837688746	0.649003689847406
Tln1	Tln1	0.0789124313693819	6.03966557584097	2.26675639172304	0.132176283676204	0.649003689847406
Wdhd1	Wdhd1	0.107302835994122	3.37863585686364	2.2664959630252	0.132198503162429	0.649003689847406
P2rx3	P2rx3	0.208919169710398	0.0512608964925671	2.26580712050656	0.13225729143206	0.649003689847406
Sox1	Sox1	0.0629111370630009	6.90843623644906	2.26548696298046	0.132284625147415	0.649003689847406
Tafa5	Tafa5	-0.0716103542741034	6.31537914574992	2.26541259157986	0.132290675570178	0.649003689847406
Cdc42ep	Cdc42ep1	0.163185641325488	2.84873433646462	2.26334189401784	0.132467824982396	0.649003689847406
Arid5a	Arid5a	-0.156486246508297	1.87831843988206	2.26326681186815	0.13247434601669	0.649003689847406
Wac	Wac	0.0398580631825304	7.40752035152747	2.26310346388364	0.13248831675625	0.649003689847406
Fbxl5	Fbxl5	-0.0385728406658503	6.4063054187879	2.26178960749463	0.132600747425754	0.649003689847406
Tent2	Tent2	0.0727937750923779	4.49276828075811	2.26131678939078	0.132641223971408	0.649003689847406
Foxd1	Foxd1	0.264517991872588	2.29575170453034	2.26100580497517	0.132667870522031	0.649003689847406
Shisa2	Shisa2	0.173499896423929	1.71962197291109	2.26027662989978	0.132730349521043	0.649003689847406
Pde1b	Pde1b	-0.120607058756449	5.70418525971764	2.25983472089131	0.132836827536053	0.649003689847406
Gpr182	Gpr182	0.128041721871641	1.62791367182168	2.2587325472717	0.132862676170659	0.649003689847406
Ythdf3	Ythdf3	0.0492070636130141	6.74768541265284	2.25856342007498	0.132877274400678	0.649003689847406
Rgs9	Rgs9	-0.158918334827713	3.85738204804663	2.25844512132817	0.132887425546488	0.649003689847406
Srebf1	Srebf1	-0.0730443239889666	5.14729774940932	2.25827952286319	0.132901628968118	0.649003689847406
Impact	Impact	-0.107736396868537	6.96489597078068	2.2581037656247	0.13291672527033	0.649003689847406
Myc	Myc	0.0653526397098368	5.83852717161253	2.25804113725724	0.132922101502534	0.649003689847406
MGC109	MGC10934(	-0.0656529104262118	6.18504300909115	2.25774417213498	0.132947597306235	0.649003689847406
Lhfpl5	Lhfpl5	-0.085735774072231	4.49748857966811	2.25768237271889	0.132952903753304	0.649003689847406
Ace2	Ace2	0.247655057223786	1.14994782809627	2.25659225718145	0.133046546140854	0.649206787719786
Snx33	Snx33	0.130545293893616	2.52677362466829	2.25399111518836	0.133270285544096	0.649730645722111
Gmfb	Gmfb	-0.0522168118617917	6.89798244762695	2.2538638637115	0.133281241967104	0.649730645722111
Atp6v1g	Atp6v1g2	-0.0535415741605996	6.99361838436632	2.25352844094143	0.133310126877548	0.649730645722111
Gzmm	Gzmm	0.278802610121902	-0.032035567315638	2.25272842834468	0.133379047290477	0.649754790487433
Ppm1k	Ppm1k	-0.089758481658093	4.46450027884027	2.25168015713692	0.133469417229602	0.649754790487433
Fcer1g	Fcer1g	0.152814887944452	2.12887387140811	2.25039516697805	0.133580286688241	0.649754790487433
Ryk	Ryk	0.0686879099182899	5.33807834004243	2.25036608716246	0.133582796886647	0.649754790487433
Acyp2	Acyp2	-0.0949658562659694	3.61324692937399	2.24877259685591	0.133720429730477	0.649754790487433
Dusp3	Dusp3	-0.0665859923962071	5.86565409867188	2.24846886500244	0.133746681616785	0.649754790487433
Abcg2	Abcg2	0.200983909014181	2.40893774096233	2.2482495840447	0.133765627897562	0.649754790487433
Kcnip2	Kcnip2	-0.0706084699711905	5.91365624282391	2.24810344390254	0.133778273008549	0.649754790487433
Serinc2	Serinc2	0.142571920662089	4.44605327734129	2.24762280268371	0.1338198381128	0.649754790487433
Cox7a2	Cox7a2	-0.0897512006697637	5.94193651011053	2.24743777490281	0.133835842875106	0.649754790487433
Rsbn1l	Rsbn1l	0.0702713878622248	4.22939030147828	2.24659946989585	0.133910979184	0.649886570061407
Wnt6	Wnt6	0.351769731648106	1.80705727541045	2.24582344590681	0.133975571912923	0.649927375877253
Pdcd11	Pdcd11	0.0778831360649277	6.85569573724575	2.24404117845646	0.134130026387243	0.649969084971316
RGD735	RGD735065	-0.0505354524695058	5.76523355159173	2.2440035742266	0.134132287380534	0.649969084971316
Bcs1l	Bcs1l	0.0680385190784481	4.39423376055628	2.24396857746241	0.134136322337182	0.649969084971316
Tbl1xr1	Tbl1xr1	0.0515807347517757	6.89277386272132	2.2430371890069	0.134217121553589	0.650088131065316
Tll2	Tll2	0.217766350923149	0.99276642246003	2.24075024313072	0.134415748180057	0.650551921366968
Akna	Akna	0.106845600917508	4.52789893168801	2.24067109697772	0.134422628091454	0.650551921366968
Abi1	Abi1	-0.0318618373362271	6.78025028079374	2.24013435217296	0.134469295920927	0.650551921366968
Rps6kc1	Rps6kc1	-0.0367287250796677	6.22480857005524	2.23934300557418	0.134538133416786	0.650622670791178
Rpl41	Rpl41	-0.102754491988516	9.8687250285784	2.23867207602866	0.134596526966985	0.650662898818026
Galnt17	Galnt17	-0.0895633842962958	5.41509404212712	2.23486862447641	0.134928092144229	0.651298659186457
Tepp	Tepp	0.144958499578955	1.32889758098538	2.23477171470055	0.134936552163066	0.651298659186457
Rbm4	Rbm4	0.0665757340160557	5.10582994619198	2.23437827177685	0.134970904984995	0.651298659186457
Papolb	Papolb	0.213653435885846	0.387335440417583	2.23426873514927	0.134980470749268	0.651298659186457
Epha10	Epha10	-0.142329469010029	4.36908514148953	2.23417054283482	0.134989046457562	0.651298659186457
Rps25	Rps25	-0.0623054534244152	7.8472118052205	2.23248881035066	0.135136016633883	0.651755732330871
Relt	Relt	-0.0956759725269516	2.28872640941977	2.23112305341979	0.135255504769682	0.651925357080772
St8sia3	St8sia3	0.0614443083911634	5.94559999890764	2.23080479356847	0.135283365852303	0.651925357080772
Dnajc24	Dnajc24	-0.0982532601661296	3.09975621552459	2.22989729490298	0.13536284530771	0.651925357080772
Tns2	Tns2	0.0971094656287217	4.52086717317731	2.22989935980912	0.135380197580814	0.651925357080772
Anxa7	Anxa7	0.0584041042865896	5.49943739030833	2.22837098681215	0.135496628454515	0.65223436737538
RGD135	RGD1358(	0.0904882332151984	3.72381558929897	2.22707407013023	0.135610429980636	0.652263204012807
Madcam	Madcam1	0.161385198896352	1.70923535088445	2.2269328090394	0.135622841703224	0.652263204012807
Ttc13	Ttc13	-0.0329885462119726	6.39996406280187	2.22575315494767	0.135726457146354	0.652263204012807
Grn	Grn	0.0541437011222831	5.89185742784853	2.22541570490544	0.135756113516805	0.652263204012807
Cnot7	Cnot7	0.0418035046173939	6.47774073503587	2.22532587823633	0.135764009042339	0.652263204012807
Minpp1	Minpp1	0.0605799271292789	5.45993113120178	2.22472827815633	0.135816549587588	0.652264485315908
Prune1	Prune1	-0.0442518027118265	6.32570938471289	2.22324880061961	0.135946722079436	0.652628419155491
Fdxacb1	Fdxacb1	-0.114932626329739	2.61068371096179	2.22230346860145	0.136029670667431	0.652785239621618
B4galt1	B4galt1	0.146947626878371	2.05773440914166	2.22154260855075	0.136097015589576	0.652785239621618
Nampt	Nampt	0.0488083221788826	5.76449385989478	2.2211200926106	0.136134262522678	0.652785239621618
RGD156	RGD1566(	-0.0708823151335131	4.34983943532787	2.22011482298918	0.136222916227854	0.652858417290022
Cntf	Cntf	-0.149849658342367	1.54581847344427	2.21908856280188	0.136313511666816	0.653101278073001
Has3	Has3	0.0715303467769889	4.41602134402344	2.21772609798812	0.136433874880329	0.653101278073001
Rad54l2	Rad54l2	0.0680136808252564	5.82592186950024	2.21754361328389	0.136450005004462	0.653101278073001
Map4k4	Map4k4	0.0475208045433512	9.19552698859532	2.2174091429828	0.136461892436989	0.653101278073001
Rdh14	Rdh14	0.0610396054280372	4.27499905546099	2.21608147822084	0.136579322822656	0.653345907098115
Ddx10	Ddx10	0.0660660948690987	4.94691135782159	2.21473305543457	0.136698704199509	0.653345907098115
Gins2	Gins2	0.0697639077321815	5.39924525704438	2.21382280356778	0.136779377803198	0.653345907098115
Ctcf	Ctcf	0.0423642888904542	6.97723760164495	2.21253057758515	0.13689095233851	0.653345907098115
Fstl5	Fstl5	-0.0611682795282092	6.92862094556207	2.21247237376008	0.136899611627975	0.653345907098115
Prag1	Prag1	0.0729831246605428	5.86763083159115	2.21244073197879	0.136901823682871	0.653345907098115
Rpl9	Rpl9	-0.0482412821845615	8.28120494988956	2.21176255450229	0.136962110187943	0.653345907098115
Dok5	Dok5	0.098185319677481	6.8107525133087	2.2114338568943	0.136991299318809	0.653345907098115
Khdrbs1	Khdrbs1	0.0383309251471154	8.49623173249713	2.21107411257712	0.137023237956033	0.653345907098115
Rpl35al1	Rpl35al1	-0.090270822490511	5.56127078569287	2.2109231465143	0.137036646539694	0.653345907098115
Pcdhga8	Pcdhga8	0.105132203262826	3.72825297062729	2.20981671092528	0.137126074243647	0.653522547111529
Frk	Frk	0.391752906237351	-0.107025621882053	2.20859175926986	0.137243803682151	0.653834359008096
Sobp	Sobp	0.0722376956659376	7.69465367845748	2.20744689956508	0.137345799804704	0.654070055024156
Gemin5	Gemin5	0.0621556487974448	5.28523615918139	2.20241872264623	0.137794376564462	0.655387908500363
Phgdh	Phgdh	-0.0739098383249417	6.12376764144955	2.20084574125736	0.13793498932547	0.655387908500363
Renbp	Renbp	-0.141110941554464	1.31520998909765	2.20052080515501	0.137964135945101	0.655387908500363
Zc3h18	Zc3h18	0.0448791947298903	5.97427195752446	2.20048776705853	0.137967074904105	0.655387908500363
Twsg1	Twsg1	0.0772445737696395	4.19109499106858	2.2002025063948	0.13799260824689	0.655387908500363
Tspan33	Tspan33	-0.109308012342718	3.44607061755127	2.20012612169521	0.137999446249659	0.655387908500363
Soat1	Soat1	0.120058997802695	2.73987971471723	2.19983570752129	0.138025447763451	0.655387908500363
Fam241a	Fam241a	0.179579449890754	2.41007613002519	2.19930817319909	0.138072693293557	0.655387908500363
Pcdha13	Pcdha13	-0.116629732862862	3.60543030941475	2.19905604337862	0.138095280231422	0.655387908500363
Lss	Lss	0.0796910037546392	7.14562119005827	2.19840521474613	0.138153603575727	0.655415403731691
Fzd10	Fzd10	-0.177035273538428	3.73394192838251	2.19748747322053	0.138235890982389	0.655556532288811
Prpf38b	Prpf38b	0.0580245328833175	6.22893498625527	2.196677875026	0.138308521331272	0.65565182297123
Pf4	Pf4	0.293090067583264	0.350481782878032	2.19553455527583	0.138411184582899	0.655735615372208
Vdac2	Vdac2	-0.0443049320472811	7.73798569801921	2.19517167361704	0.138443783983884	0.655735615372208
Best1	Best1	-0.190016899427185	0.689561557710305	2.19472549309794	0.138483878056085	0.655735615372208
Tm4sf1	Tm4sf1	0.212207195990809	0.973000566387946	2.19326990697515	0.138614768929407	0.656106400581261
Kcnn1	Kcnn1	0.0627454693406912	5.24838343854339	2.19180072671787	0.1387470229491	0.656378905035821
Chst1	Chst1	-0.0532057679689142	6.74143534927288	2.19146177817832	0.138777554819628	0.656378905035821
Arhgap3	Arhgap30	0.322752086980971	-0.115797203408095	2.18982135115425	0.138925428056133	0.656829316352306
Kiss1r	Kiss1r	-0.0820307206979362	3.82970166871242	2.18551262248539	0.139314672981753	0.658420142014435
Zfp777	Zfp777	-0.0415670407281444	5.58623925015358	2.18418508803022	0.139434867384372	0.65858963987948
Arv1	Arv1	-0.0601059782821935	4.77857717802631	2.18395038639046	0.139456105529082	0.65858963987948
Bap1	Bap1	-0.0347331466877386	8.2364472434725	2.18305582823224	0.139537155236732	0.658723074494402
Baz1b	Baz1b	0.0847891293500604	7.34026465047287	2.18089407011254	0.139732266515686	0.659283712531873
Ufc1	Ufc1	-0.0556130129095642	5.94913822327661	2.18012332224787	0.139802258184045	0.659283712531873
Slc8a1	Slc8a1	0.0645591564743232	7.81072124539647	2.18000029692703	0.139814434828832	0.659283712531873
Cdca4	Cdca4	0.0682010328826895	4.19629016802206	2.17825622313376	0.139972975930425	0.659781949635027
Wdr33	Wdr33	0.0437954527779344	6.04304132589787	2.17577676993741	0.140198712575271	0.660596426284613
Ccdc93	Ccdc93	0.0784712164988211	4.1570779555263	2.17497269453632	0.1402720056917	0.660892266898957
Gfer	Gfer	0.0743569481784176	3.98139180587299	2.17383780727405	0.140366401111919	0.66078802742728
Zbtb18	Zbtb18	0.11637983861803	8.82806592293758	2.17358873018799	0.140398257650855	0.66078802742728
Commd4	Commd4	-0.0670035906031006	5.97310187859355	2.17211608584579	0.140532738584489	0.661171565532717
Rfc3	Rfc3	-0.064745633650266	4.52383703662656	2.17122179181543	0.140614477146249	0.661306758897002
Fbxo21	Fbxo21	0.0322817130216893	7.471396481778	2.17049030564817	0.140681373935701	0.661372080857477
Unc13b	Unc13b	0.102475751763033	6.33586212098735	2.16906514098588	0.140811812576723	0.661733237209808
Gys1	Gys1	0.0480018623903171	5.12910205201909	2.16849242884032	0.140864268496373	0.661733237209808
Tapbpl	Tapbpl	0.145359626834057	2.58439224524726	2.16776073257304	0.140931318049791	0.661799042268439
Tgfbr1	Tgfbr1	0.0620624097317069	6.34093914944967	2.16669772275361	0.141028791867117	0.662007612351623
Erc2	Erc2	-0.053881077227616	6.78588044031764	2.16602876332855	0.141090171317116	0.66204665946734
Rab36	Rab36	-0.0903483090982876	4.54405028857139	2.16482842144578	0.141200382652526	0.662102808147613
Abhd18	Abhd18	0.107031547179742	3.30196091158394	2.16458632742166	0.141222622627986	0.662102808147613
Got2	Got2	-0.0497345186141437	8.12719844607211	2.16416502603413	0.141261334879293	0.662102808147613
Thbs2	Thbs2	-0.158269350877444	2.60947006751993	2.16231232477463	0.14143171619716	0.662422275876231
Mical3	Mical3	0.0764141878122394	6.87843286635316	2.16135590539598	0.141519758938658	0.662422275876231
Atp6v1e	Atp6v1e1	-0.0562508908299586	7.77655229571081	2.16127205827956	0.141527494216946	0.662422275876231
Lgals3b	Lgals3bp	0.181589736348384	3.02622219978262	2.159817523744	0.141661512406217	0.662422275876231
Rims3	Rims3	-0.0686292285994846	5.64562220107085	2.15973835689238	0.141668811334029	0.662422275876231
Fbxo24	Fbxo24	0.196597475731779	0.416607402726375	2.15949580228676	0.141691167494682	0.662422275876231
Nsun6	Nsun6	-0.0652837843740427	3.85254909152113	2.15937277718963	0.141702522084277	0.662422275876231
Fpgt	Fpgt	0.100751371371094	2.44187567607683	2.15789276567162	0.141839086886774	0.662422275876231
Tmem30	Tmem30a	-0.0458851522186714	6.72553401061474	2.1574412810631	0.141880768331338	0.662422275876231
Scg3	Scg3	-0.0384470735555057	7.96061368067427	2.15727308691935	0.141896312935578	0.662422275876231
Ank	Ank	0.0954133764939398	2.28122643714771	2.1570870203002	0.141913500313951	0.662422275876231
Tmem17	Tmem176b	0.149163079286613	3.87450951005611	2.1555157881013	0.142058731816835	0.662818464794158
Fezf2	Fezf2	0.131424982604559	6.07326468346688	2.15481889543156	0.142123200207229	0.662818464794158
Smg6	Smg6	0.0590077316583141	5.59027599470426	2.15391301698494	0.142207050703989	0.662818464794158
Slc9b2	Slc9b2	-0.1121548045238	3.14504714379112	2.15366086461759	0.142230402339532	0.662818464794158
Myo1f	Myo1f	0.201615156246916	1.46138389486155	2.15231796834641	0.142354827849889	0.662818464794158
Mri1	Mri1	0.0783885496223814	5.07795910518658	2.15230399468005	0.142356123236894	0.662818464794158
Setd4	Setd4	-0.0687305179569914	4.81042298203348	2.1521517218543	0.14237024009364	0.662818464794158
Dlgap4	Dlgap4	0.0390168029879408	8.08505289467966	2.15110857301917	0.142466890321273	0.663021498782483
Rsph9	Rsph9	-0.0701041298923391	4.43298301116813	2.14967384669319	0.142600179487707	0.663028904961915
Garre1	Garre1	0.0460473994282432	5.90483477613522	2.14942547659906	0.142623250520535	0.663028904961915
Cnpy4	Cnpy4	0.0992771447096755	3.02432251313516	2.14889418949381	0.142664251893077	0.663028904961915
Arf6	Arf6	0.0523314206480492	6.06451457979604	2.1488026482567	0.14268114208736	0.663028904961915
Slc22a6	Slc22a6	0.326320726483525	4.98841030036442	2.1474580988385	0.142806055019477	0.663262425267442
Akap13	Akap13	0.0920178300705344	5.15842375534684	2.14711885749848	0.142837712330477	0.663262425267442
Dis3l	Dis3l	-0.0499834048072041	5.25205430247078	2.14640220171862	0.142904418461585	0.663310073214738
Inpp4a	Inpp4a	-0.0423842368273106	7.92744958577706	2.14586605845674	0.142954298881408	0.663310073214738
Tagln2	Tagln2	0.12000595461161	5.18612612394211	2.1445834579941	0.143073864335886	0.663618067500281
Adamts1	Adamts15	-0.146197782908222	4.0749980782273	2.14322657793157	0.143200426732223	0.663947177280017
Mtus2	Mtus2	0.0519258051819165	6.15803865888141	2.1418697107964	0.143327114212355	0.663947177280017
Fip1l1	Fip1l1	0.0452134882106085	6.17327862443021	2.14189634501265	0.143343310051018	0.663947177280017

Zfp516	Zfp516	0.100226506179657	3.5988087741435	2.14154260933687	0.143357673767121	0.663947177280017
Fmo1	Fmo1	0.125504954821785	4.49734079560001	2.13991946592887	0.143509424954434	0.664403374826055
Cbx7	Cbx7	-0.152247635113129	1.86768297301759	2.137700238154	0.143717199824243	0.664783354622445
Kcnk15	Kcnk15	-0.0903392003108573	3.6416326132778	2.1365182928157	0.143827985321757	0.664783354622445
Tmod1	Tmod1	0.145125803015126	1.51987228052484	2.13527903577958	0.143944267104288	0.664783354622445
Zfp35	Zfp35	0.0707394613177698	4.21420502019315	2.13489220019793	0.143980582344755	0.664783354622445
Zmat3	Zmat3	0.0629628395823226	6.32792345262405	2.13430098038503	0.144036107172534	0.664783354622445
Eid2b	Eid2b	-0.0490654490913193	5.38734886431535	2.13429261798878	0.1440368926888	0.664783354622445
Plekhb2	Plekhb2	-0.0494246219780205	8.30442952405679	2.13423541710976	0.144042265965809	0.664783354622445
Phax	Phax	-0.0541742615738846	5.84304252507375	2.13402311066341	0.144062211359148	0.664783354622445
Tspyl2	Tspyl2	-0.0722855070386703	5.6446861769742	2.13366398487683	0.144095856957375	0.664783354622445
Zfp94	Zfp94	-0.0802310626358086	3.86370758511702	2.13310693037014	0.144148318728784	0.664783354622445
Wdr24	Wdr24	-0.0608096523431637	5.29000640629026	2.13200588861636	0.144251867808009	0.664783354622445
Arid4a	Arid4a	0.0642259680187248	5.74849206629894	2.13147288332319	0.144301935285989	0.664783354622445
Cbfa2t3	Cbfa2t3	0.0899403351050461	5.04054114634669	2.13122075873402	0.144325773440031	0.664783354622445
Tmx2	Tmx2	-0.0522638778362484	6.37042735345297	2.13061130861878	0.144383164272942	0.664783354622445
Kpna2	Kpna2	0.0601883129296458	6.25363895338593	2.12990103994265	0.144450081452482	0.664783354622445
Dnajc12	Dnajc12	-0.121805815872223	3.66040777322489	2.12984363077375	0.144455491709317	0.664783354622445
Serping1	Serping1	0.244436062555566	2.39008517416554	2.12940037946923	0.144497271598627	0.664783354622445
Pamr1	Pamr1	-0.158562541652165	1.74701436523658	2.12889143624827	0.144564123315931	0.664845767273451
Nxph1	Nxph1	-0.0528495942626296	6.48889527127936	2.12746449761344	0.144679903143639	0.665133069831682
Zdhhc15	Zdhhc15	0.0946119638563473	2.86115252582597	2.12474541528636	0.144936886125685	0.665328328261579
Atp5pd	Atp5pd	-0.0651109282975497	6.99515826383769	2.12460474718215	0.144950168622152	0.665328328261579
Kcnmb2	Kcnmb2	0.120501404838866	1.95661139487968	2.12389150647358	0.145017863211428	0.665328328261579
Fntb	Fntb	0.0649980304301117	5.38598102346118	2.12328065217318	0.145075497035951	0.665328328261579
Grem1	Grem1	0.129816021256762	2.87508559779841	2.1230094117826	0.145101185581217	0.665328328261579
Picalm	Picalm	0.037512203893818	7.28194073716966	2.12279816037468	0.145121196260095	0.665328328261579
Myo5c	Myo5c	0.287034589133405	2.04521093405533	2.1226356978915	0.145136587548882	0.665328328261579
Ranbp1	Ranbp1	-0.0492843350247216	7.0010108836891	2.12250500008574	0.145148970868864	0.665328328261579
Sult1d1	Sult1d1	-0.351871613443376	-0.530960732709256	2.12187454708385	0.145208721570472	0.665357774158933
LOC102[illegible]	LOC102548[illegible]	0.0649781264522679	4.57826415161346	2.12007136872168	0.145379769491446	0.6657914657863
Ca9	Ca9	0.243282076876445	-0.398033759574107	2.11975193762467	0.145410094114584	0.6657914657863
Taf2	Taf2	0.0481447558955776	5.95034556495871	2.11701961040723	0.145669774239022	0.666725793536417
Cend1	Cend1	-0.0694401798857255	6.50936336784325	2.11604231220754	0.145762783459452	0.666816849732626
Kcnj2	Kcnj2	0.129052347818135	2.51603572580008	2.11522084563418	0.145841014010469	0.667030180281752
Cdh7	Cdh7	-0.10336574225367	4.10527265049352	2.11285546391613	0.146066540419404	0.667603488202899
Abcd1	Abcd1	0.0532644008250697	4.22673219072392	2.11269646839812	0.146082068276131	0.667603488202899
Fmi2	Fmi2	0.0511174353991424	6.29538352246588	2.11178888156435	0.146168363912678	0.667603488202899
Map10	Map10	0.0889834542799137	2.36181192459419	2.11131560853133	0.146213558650386	0.667603488202899
Gpr63	Gpr63	0.0986277022544891	2.63604693090159	2.11110284293073	0.146223897031219	0.667603488202899
Mrpl42	Mrpl42	-0.0937994285640436	5.22596534925346	2.11009786657712	0.146320915298199	0.66780205931808
Lypd6b	Lypd6b	-0.0872023801509659	4.32467865932261	2.10855352568313	0.146477715778867	0.668045493895396
Rcn3	Rcn3	0.12670645514637	3.77708327197938	2.10791661031423	0.146538702456044	0.668045493895396
Tm7sf3	Tm7sf3	0.0634816763387384	5.42093300963416	2.10744924716046	0.146583472158412	0.668045493895396
Spag5	Spag5	-0.105876963673262	3.39639720948968	2.10729317494426	0.146598442386847	0.668045493895396
Pcdha4	Pcdha4	0.0701118229035183	4.5526257537475	2.1050604672615	0.146812528607047	0.668054562104361
Nkain3	Nkain3	0.124568592612762	3.27797670413843	2.10441123128777	0.146874866612967	0.668054562104361
Creld2	Creld2	0.0852829824921633	4.54221125136903	2.1030586110935	0.147004814796405	0.668054562104361
Trmt6	Trmt6	-0.0700809541476212	4.29011218327693	2.10279523833019	0.147030132485168	0.668054562104361
Pisd	Pisd	0.0559513501818846	5.42716988340929	2.10272154026791	0.147037217867097	0.668054562104361
Asf1b	Asf1b	0.131527944380573	1.3713779204876	2.10223213280696	0.147084279628522	0.668054562104361
Tp53i13	Tp53i13	0.111778021728441	2.28290954819249	2.10127753451938	0.147176123323155	0.668054562104361
Rpf2	Rpf2	-0.0536872842878265	5.20734631751114	2.10098731251668	0.147204055896027	0.668054562104361
Cib1	Cib1	0.107692950840287	2.62960041476321	2.1002882377182	0.147271364109351	0.668054562104361
Apod	Apod	0.204049815648917	2.67490887271214	2.09981767459547	0.147307069811338	0.668054562104361
Kalrn	Kalrn	0.0701035465981877	7.49319770610815	2.09856627513338	0.147437320881422	0.668054562104361
Akirin2	Akirin2	0.0426573905759035	6.93747837179159	2.09716372362681	0.147572692025178	0.668054562104361
Vps4b	Vps4b	0.0714969681843472	5.20965383719841	2.09708747052721	0.147580023592868	0.668054562104361
Fap	Fap	0.248408532753368	1.06459859081726	2.09688634417752	0.147598978709401	0.668054562104361
Paqr9	Paqr9	0.075549237157808	4.39492147279725	2.09584139849974	0.14770037966720	0.668054562104361
Ifrd2	Ifrd2	0.103091624334218	3.54516376231949	2.09557217193526	0.147726398377768	0.668054562104361
Ccr5	Ccr5	0.281484552013288	1.16614924605728	2.09557006754109	0.147726601771921	0.668054562104361
Axs1	Axs1	-0.112551275769084	3.48800094730359	2.09527851419894	0.147754784807627	0.668054562104361
Lrfn2	Lrfn2	0.0508098260998801	5.35013738394239	2.09524850193769	0.147757685493706	0.668054562104361
Efemp2	Efemp2	0.109000355653339	4.40784445163562	2.09510485812964	0.147771573007791	0.668054562104361
Timmdc1	Timmdc1	-0.0572078338620368	6.94318862826155	2.0945142287158	0.147828890713809	0.668054562104361
Ano1	Ano1	-0.214896760508831	1.42154813484516	2.09402328981643	0.147876188688684	0.668054562104361
Rps29	Rps29	-0.0988391392650937	8.06888876520489	2.09355473363217	0.147921523273645	0.668054562104361
St6galnac6	St6galnac6	-0.0536911829742679	6.28015736560652	2.09236516275954	0.148036729822257	0.668054562104361
[illegible]						

Vcl	Vcl	0.0912858940182743	5.30934993867851	1.90521577188359	0.167495668284931	0.689384444407477
Nme3	Nme3	-0.0736999642537687	4.79147924649275	1.90461988857129	0.167582116836561	0.689384444407477
Atp11a	Atp11a	0.0872600154292069	5.90118757283794	1.9044542780248	0.167580589876403	0.689384444407477
Calcoco(	Calcoco1	0.0627120021793406	6.47499510677848	1.90385223022783	0.167647764998886	0.689433475244266
Manf	Manf	0.076096507346733	5.1887033307942	1.90321505387148	0.167718893288641	0.689498725281226
Gcat	Gcat	0.100290159063974	2.6849582101781	1.90240937514744	0.167808881077723	0.689641439132657
Anxa1	Anxa1	0.17214446549145	2.51898729584829	1.8996040059051	0.168122650067191	0.689655919003291
Moap1	Moap1	-0.106064135342631	1.79252521797826	1.8988724528833	0.168204591968975	0.689655919003291
Rad51ap	Rad51ap1	-0.0765581584410246	3.27426494347619	1.89795377109237	0.168307528077978	0.689655919003291
Akr1c14	Akr1c14	0.200390509502356	1.8092664252397	1.89745989973393	0.168362912838325	0.689655919003291
Lhfpl6	Lhfpl6	0.0633290336748854	6.2481157274431	1.89737312863983	0.168372644456042	0.689655919003291
Fundc2	Fundc2	0.0746127506473498	3.7696797637859	1.89714573332777	0.168398150526946	0.689655919003291
Scnn1a	Scnn1a	-0.262914344311141	-0.028457728221569	1.89652560592169	0.168467723682584	0.689655919003291
Nmb	Nmb	-0.260989317313062	0.0004501854274148	1.89628718615927	0.168494490447332	0.689655919003291
Rpl37	Rpl37	-0.090490286770051	7.21461115197462	1.89563721441546	0.168567467572383	0.689655919003291
Fzd4	Fzd4	0.0833151846299628	3.33833949119732	1.89539039347733	0.168595201828571	0.689655919003291
Ccdc25	Ccdc25	-0.046127907518936	5.38069353684344	1.89535468157896	0.168599200917219	0.689655919003291
Sema3g	Sema3g	0.104738504809667	3.35005169554749	1.89398895088672	0.168752692796999	0.689655919003291
Mak16	Mak16	0.0556734062545447	4.15929124511895	1.8932621759853	0.168834438959305	0.689655919003291
Tbcel	Tbcel	0.0761494049748571	3.46104908924118	1.89300908374094	0.168862916918299	0.689655919003291
Opa1	Opa1	-0.0420707171669606	7.22272940495058	1.89296155628117	0.16886826532601	0.689655919003291
Atg9b	Atg9b	-0.204226250551146	-0.0590076365171 39	1.8928494642032	0.168880880153217	0.689655919003291
Dnajb5	Dnajb5	0.0416894892239868	7.09225547174107	1.8924340345228	0.168927641977821	0.689655919003291
Micb	Micb	0.155879367368874	1.61813009698279	1.89215342456207	0.168959236552423	0.689655919003291
Spats2	Spats2	0.0402082442898613	6.04931215618142	1.89203734227541	0.168972308527626	0.689655919003291
Igfbp2	Igfbp2	0.125386998716239	7.78561521876266	1.89196970705279	0.168979825434991	0.689655919003291
Cyp27b1	Cyp27b1	0.189635265436673	0.205858515880633	1.89173126297097	0.169006781544297	0.689655919003291
Pdcd10	Pdcd10	-0.0511044934765602	5.53499198961719	1.89149531101356	0.169033361782385	0.689655919003291
Tmem50	Tmem50b	-0.0566521095415417	6.80492636968287	1.8905697887794	0.169137699136853	0.689655919003291
Aifm1	Aifm1	0.0550722417449829	4.66585809869003	1.88987746504515	0.169215743033139	0.689655919003291
Rnf146b	Rnf146b	0.108346672129781	2.45833708458572	1.88984867570417	0.169218990052498	0.689655919003291
Col5a1	Col5a1	0.121360540911025	5.51131583999192	1.8895692436338	0.169250514706105	0.689655919003291
Hnrnph3	Hnrnph3	0.0717034513719434	6.14211374867907	1.88878606983509	0.169338904493795	0.689794812722522
Babam1	Babam1	0.0614634521303005	5.89586391081318	1.88686032824508	0.169556470913979	0.690241921680085
Scube1	Scube1	0.0887672382716163	5.96063991837711	1.88683523224431	0.169559308325728	0.690241921680085
RGD621(	RGD621098	-0.0490756741112045	5.16222519783191	1.8858701845909	0.169668460175758	0.690460984218177
Deaf1	Deaf1	-0.0385688514932586	7.4727552642277	1.88468221762343	0.169802928066952	0.690525545456813
Robo1	Robo1	0.0640513783846995	7.7539034944186	1.88462806445109	0.169809071295233	0.690525545456813
Mlycd	Mlycd	-0.0613889967960988	4.58888933961842	1.88422623063665	0.169854585515096	0.690525545456813
Rnf20	Rnf20	0.0380774073628133	6.80462894268164	1.88191832703745	0.170116277460331	0.690525545456813
Ephx4	Ephx4	0.116339958342734	3.81310088017341	1.88175185163085	0.170135171904649	0.690525545456813
Efcab14	Efcab14	0.0702351909755234	4.42897052358842	1.87973282232234	0.170364505960282	0.690525545456813
Zbtb49	Zbtb49	0.0708670867432529	3.64898816217986	1.87887596025001	0.170461857191922	0.690525545456813
Mrap2	Mrap2	-0.129133795651083	3.37407698099839	1.87824940836849	0.170533361155176	0.690525545456813
Timm17t	Timm17b	0.0727341660911529	4.20847721529825	1.87770987673	0.170587705928073	0.690525545456813
Arrdc3	Arrdc3	0.0964507423826016	4.64153582118737	1.87746656307844	0.170622368944824	0.690525545456813
Brca1	Brca1	0.125024298859426	2.57281178598349	1.87658768805085	0.170722486598871	0.690525545456813
Ankrd44	Ankrd44	0.0847051673091444	5.80033936520219	1.87515228960032	0.170886146093575	0.690525545456813
Rpl31	Rpl31	-0.0585389836139706	8.11680990098649	1.8747808792736	0.170928522434685	0.690525545456813
Lgals1	Lgals1	0.177529256885088	4.16095446805444	1.87470949802309	0.170936669141802	0.690525545456813
Nsn	Nsn	0.087472545688707	4.61370302498188	1.87445689489567	0.170965497531797	0.690525545456813
Bfd1	Bfd1	0.0795020563956295	3.51658677479735	1.87417347485898	0.170997850708811	0.690525545456813
Chrna4	Chrna4	-0.0687464274051665	5.60807814039983	1.87386674392656	0.171032872811914	0.690525545456813
Tigd3	Tigd3	0.0782397358549409	3.27172138672797	1.87381466873131	0.171038819321906	0.690525545456813
Crim1	Crim1	0.0617632777203118	6.72204877629315	1.87378719898841	0.17104195648539	0.690525545456813
Lyl1	Lyl1	0.157505118225853	1.31098543200248	1.87294318080776	0.171138373720763	0.690525545456813
Commd!	Commd5	-0.0581335574033031	4.15228805882226	1.87280106330882	0.171154611476444	0.690525545456813
Pgm1	Pgm1	-0.0428938839063005	6.17552916083852	1.87255450913388	0.171182794965255	0.690525545456813
Itga4	Itga4	0.114834928643249	2.30978386822609	1.8721576194591	0.171228169249652	0.690525545456813
Ttc9	Ttc9	0.0435581501027626	6.41398527515693	1.87130690498728	0.171325473100101	0.690525545456813
Galnt4	Galnt4	0.144576767989439	1.25807064903185	1.87086203356259	0.171376382391489	0.690525545456813
Nedd9	Nedd9	0.0601652743167668	4.50512675169075	1.8707649398505	0.171387495714523	0.690525545456813
Idh3g	Idh3g	-0.0380372703798354	6.84418676406279	1.87062622655229	0.171403374241793	0.690525545456813
Spire2	Spire2	0.0640591411804346	5.64923026159898	1.87052176080576	0.171415333556678	0.690525545456813
Atp6v0d	Atp6v0d1	-0.0449266585187712	8.19651061021413	1.87001079319192	0.171473843356378	0.690525545456813
Mtmr12	Mtmr12	0.0758785368148516	3.35628927371399	1.86930935261188	0.171554201168979	0.690525545456813
Tyrobp	Tyrobp	0.155245761128263	1.64596058772646	1.86917444112609	0.171569661739546	0.690525545456813
Mei1	Mei1	-0.264218936798153	-0.620282227086001	1.8687609358889	0.171617058538204	0.690525545456813
Gmpr	Gmpr	-0.0830256343623203	4.45524494294012	1.86842910659114	0.17165510435815	0.690525545456813
Fam32a	Fam32a	-0.0466338388071966	6.69427143295412	1.86839296823387	0.17165924837926	0.690525545456813
Baiap3	Baiap3	-0.177574577188237	5.49292880839588	1.86772974068651	0.171735321755461	0.690525545456813
Mpeg1	Mpeg1	0.17117120733073	3.69479086555249	1.86772434782597	0.171735940484896	0.690525545456813
Mak	Mak	0.159739739003045	1.65962690822352	1.86740339538454	0.171772791321324	0.690525545456813
Poll	Poll	-0.0771440224140075	5.13200736622202	1.86723747003863	0.171791811121263	0.690525545456813
Tfip11	Tfip11	-0.0370872693961336	5.60596001144268	1.86626076087595	0.171903854320959	0.690525545456813
Med1	Med1	0.0512963758522391	6.31449957143293	1.86607471069949	0.171925325655331	0.690525545456813
Pcsk1n	Pcsk1n	-0.080004776943063	8.69325289449193	1.86583012939617	0.171953423878682	0.690525545456813
Larp7	Larp7	-0.0434168036298418	5.35453448752917	1.8643110348411	0.172128068491497	0.691004475729861
Map2k4	Map2k4	-0.0410773978580941	5.84654944286668	1.86365081029787	0.172204035091668	0.691043895488295
Slitrk6	Slitrk6	-0.235050177315863	2.0220088486161	1.86326314303217	0.17224865872955	0.691043895488295
Scn3b	Scn3b	-0.0481659596751854	8.29968411978926	1.86174582041287	0.172423442722173	0.691426809083482
Usp10	Usp10	0.0313696054012483	7.02631462175072	1.86104852901387	0.172503833752454	0.691426809083482
Otud3	Otud3	0.0914724636225153	3.01792541689354	1.85920068275344	0.172717081278666	0.691426809083482
Lasp1	Lasp1	0.0323815398278225	8.56853233976015	1.8589545900163	0.172745504056509	0.691426809083482
Pde12	Pde12	0.0675453062375192	3.30643394500928	1.85828240227861	0.172823166807486	0.691426809083482
Asah1	Asah1	-0.0480337163652956	5.14295165996378	1.85804581585741	0.172850510644812	0.691426809083482
Nf1	Nf1	0.0891072920391295	6.82420906635845	1.85769810427968	0.172890707266897	0.691426809083482
Negr1	Negr1	-0.0787491776358126	7.28318005717659	1.85764936097995	0.172896343014642	0.691426809083482
Tubd1	Tubd1	0.0963616911788472	2.67304813369917	1.85719328773533	0.172949084885908	0.691426809083482
Ccn1	Ccn1	-0.193252312904349	1.24117148026305	1.85673738958081	0.173001825007081	0.691426809083482
Mapkbp:	Mapkbp1	0.0677963554979627	6.50162189185062	1.85594704354926	0.173093299230005	0.691426809083482
Prickle2	Prickle2	0.0582669435110318	7.26031349054832	1.85513071781494	0.173187828748164	0.691426809083482
Cwc27	Cwc27	-0.0574168482584409	3.72193310461807	1.85511418268064	0.173189754312665	0.691426809083482
Pcbp3	Pcbp3	-0.0606924981839972	5.89680492204379	1.85505517652877	0.173196590264111	0.691426809083482
Nova1	Nova1	-0.0930495538611719	6.58163840082516	1.85482471184324	0.173223292918578	0.691426809083482
Ppp4r4	Ppp4r4	-0.137226258640766	2.17794047047296	1.85476069607938	0.173230710907667	0.691426809083482
Gpr153	Gpr153	-0.0962127825988725	5.45657354683681	1.85302235408389	0.173432285476287	0.692009794015874
Dpp6	Dpp6	-0.0760556406806195	7.49768752238231	1.85130007330659	0.173632263821253	0.692296224076583
Ankrd50	Ankrd50	0.0416868239209153	6.217169176623	1.85124074625465	0.173639157160343	0.692296224076583
Mmp19	Mmp19	0.157032363022384	1.1896182900246	1.85097078708073	0.173670528280813	0.692296224076583
Ca13	Ca13	0.233199732454859	0.353715102833067	1.85009346488906	0.173772524231674	0.692297244566004
Gpr34	Gpr34	0.25750099508601	1.39005854091599	1.84995665143975	0.173788436138091	0.692297244566004
Hsbp1	Hsbp1	-0.0496342041808736	8.72968827129747	1.84872735325924	0.173931483283209	0.692297244566004
Dnali1	Dnali1	-0.134392601221972	1.77440611401625	1.84839362255528	0.173970341206731	0.692297244566004
Gsn	Gsn	0.0906018978558477	4.3222721876733	1.84784851489037	0.174033802293157	0.692297244566004
Cfap97	Cfap97	0.0683523988561981	4.41514469830807	1.84745465280956	0.174079723740609	0.692297244566004
Tril	Tril	-0.0836822457532626	6.39726327518392	1.84723694791408	0.174105095940195	0.692297244566004
Sf3a2	Sf3a2	0.0481198675053708	6.03528573214333	1.84670326130531	0.174167311925016	0.692297244566004
Fam216a	Fam216a	-0.0505620183352049	5.54498101487846	1.84581845806495	0.174270516701496	0.692297244566004
Rsrc1	Rsrc1	-0.0542987881384252	4.57002903040506	1.84499950818983	0.174366102982801	0.692297244566004
Vwa5b1	Vwa5b1	-0.177106290388132	3.21941404246616	1.84479240757027	0.174390384950536	0.692297244566004
Ppp2r3c	Ppp2r3c	0.0581608384280953	4.24752267486186	1.84469581158917	0.174401565235057	0.692297244566004
C1r	C1r	0.194386793058852	3.19836785404313	1.843164081105	0.174580549947447	0.692297244566004
Dnai2	Dnai2	0.2801879502792	-0.234410423067401	1.84315246649041	0.174581907938223	0.692297244566004
Ciz1	Ciz1	0.0584105261091888	5.81842562380991	1.84313465416417	0.174583990590928	0.692297244566004
Rps21	Rps21	-0.104756896468933	7.26035579875722	1.84289905802265	0.174611526703674	0.692297244566004
Gskip	Gskip	0.0703864906257142	3.82007800545085	1.84258710082216	0.174648025680554	0.692297244566004
Prkacb	Prkacb	-0.0568012855659852	7.72866402928962	1.84240327290186	0.174669530006715	0.692297244566004
Ankrd12	Ankrd12	0.0655158234653538	7.90262569181774	1.84118130322892	0.174812554387006	0.69264409053244
Gypc	Gypc	0.149632303890635	1.77725034281365	1.83776271793731	0.175213396902509	0.693608806682118
Tmem21	Tmem212	-0.340225471717998	-0.0369107780343021	1.8375381355871	0.175239767108296	0.693608806682118
Eif3g	Eif3g	-0.0494650090142207	6.40933989500891	1.8375307482595	0.175240634597634	0.693608806682118
Rnf185	Rnf185	0.0383823365874697	5.66170591935419	1.8372092081373	0.175278397651699	0.693608806682118
RGD130!	RGD130674	0.0643600609547714	6.38080427352559	1.83578629519893	0.175445623266458	0.693783105241622
Prrc2a	Prrc2a	0.0500592680370152	9.69426519845329	1.83568684514087	0.175457317850945	0.693783105241622
Prkaa2	Prkaa2	0.0653017971333324	5.52768631586413	1.83541526140742	0.175489258647316	0.693783105241622
Efna5	Efna5	0.0931607015305254	3.70702335551095	1.83357970315794	0.175705313316013	0.694417229725654
Skap2	Skap2	0.133081557868535	2.88721540553685	1.83269141534562	0.175809979444423	0.69461085588069
En2	En2	-0.765464867840537	-0.57715246452232	1.83214297049582	0.175874638077	0.694646362547239
Ttc27	Ttc27	-0.0675820301719282	5.01657362119088	1.83058424894453	0.176058552501967	0.694858411052548
LOC100!	LOC100912	0.185291406109896	0.069094655191718	1.83044904372279	0.176074633962124	0.694858411052548
Emilin3	Emilin3	-0.133152326037052	1.53686665819951	1.83027218957223	0.17609539919437	0.694858411052548
Usf2	Usf2	0.0287932524718953	6.57483993575005	1.8287059831574	0.176280464784164	0.695305966788379
Fads2	Fads2	0.0361078584348307	7.87298473520972	1.82836931262297	0.176320275623152	0.695305966788379
Ptgds	Ptgds	0.30410632492349	7.82877495669408	1.82756742881485	0.176415129068117	0.695460249652365
Spdef	Spdef	-0.219745491013348	0.819360729601852	1.82555196921307	0.176652664213855	0.695802307485401
Rpl7a	Rpl7a	-0.0491119675254616	8.81390285194234	1.82526089614551	0.176688332191185	0.695802307485401
Rasal2	Rasal2	0.0929779805713355	4.49227996502746	1.82491700284498	0.176729105481542	0.695802307485401
Sp5	Sp5	-0.178704842500806	1.62636739054934	1.8242626706776	0.176806716086378	0.695802307485401
Foxn3	Foxn3	0.0635863298958715	5.44575235194682	1.82415661562408	0.176819299021835	0.695802307485401
Dcaf15	Dcaf15	-0.0540249071241611	6.06205336191894	1.8234810763519	0.176899472844972	0.695802307485401
Tram1	Tram1	0.0451223484731229	5.808378506648	1.82330531071042	0.176920339800186	0.695802307485401
Pdk4	Pdk4	0.243604750745646	0.466023449633319	1.8230720065867	0.176948042129613	0.695802307485401
Fam118t	Fam118b	0.06075166437332	4.29437363665468	1.81912427694724	0.177417551827567	0.697428731617062
Snw1	Snw1	0.0450209252022212	5.52935890888287	1.81706799029903	0.177662679628889	0.698172359449654
Cdyl2	Cdyl2	0.0731643687428404	3.86624828588642	1.81563022325319	0.177834304897207	0.698462407006771
Ncor1	Ncor1	0.053263227499785	8.12738659122593	1.81550585115113	0.177849160188984	0.698462407006771
Rbm27	Rbm27	0.0549116916076615	5.65476235712491	1.8150432534765	0.177904426502165	0.698462407006771
Arfgap2	Arfgap2	0.0645756044039433	6.55834232783747	1.81377443716404	0.178056113162383	0.698720332957418
Cyyr1	Cyyr1	0.151764299608178	1.16532282115417	1.81355699074371	0.178082123812182	0.698720332957418
Atp6ap1l	Atp6ap1l	-0.238107196538592	1.97359326285684	1.81298601340686	0.178150444246586	0.698768655411711
Cox7a2l	Cox7a2l	-0.0579988578038845	7.07268661464349	1.81159589828299	0.178316894504309	0.699201726188014
Zfp532	Zfp532	0.0518786448609076	7.13841059044203	1.80915662664659	0.17860823319199	0.70012407336892
Dact2	Dact2	-0.0938474372879807	3.49427168547751	1.80785449775021	0.178765817506067	0.700521703808943
Fkbp14	Fkbp14	0.144985512280134	1.7882329606022	1.80571282937899	0.179023373218335	0.70111274998481
Prpf6	Prpf6	0.0332042368822351	6.61295925288752	1.80566582175186	0.179029031123414	0.70111274998481
Daxx	Daxx	0.0428439086797831	5.6187290034376	1.8034886302457	0.179291308044563	0.701919564001257
Lig3	Lig3	0.0521556234730832	5.52657811783436	1.80210858369627	0.179457786285023	0.702108481290159
Slc13a5	Slc13a5	-0.138493896743834	2.67516311451717	1.80176508185365	0.17949925148322	0.702108481290159
Piezo1	Piezo1	0.122906665710318	3.66269727655055	1.80149566144072	0.179531784242065	0.702108481290159
Hspa5	Hspa5	0.0655794466609112	8.54712565139986	1.80122348330821	0.179564652063549	0.702108481290159
Rpl36a	Rpl36a	-0.0720043572202419	7.11632486261024	1.8000464057983	0.179706884745576	0.702444865519596
Il1r1	Il1r1	0.21387221454858	1.11757637011223	1.79923785293197	0.179804692058289	0.702554837985626
Dhx9	Dhx9	0.0640745767222174	8.09842554218306	1.79838744402722	0.179907567349242	0.702554837985626
Dlg4	Dlg4	-0.0389607314112973	9.35364924695718	1.79758818765458	0.180004364920013	0.702554837985626
Zswim4	Zswim4	0.0522102955946317	5.7974001438953	1.79722795426231	0.180047983286792	0.702554837985626
Ndufb5	Ndufb5	-0.053998050499079	6.15124394085204	1.79622877231229	0.180169087385233	0.702554837985626
Gad1	Gad1	-0.127119014756443	7.33603526376565	1.79507950002318	0.180308499441463	0.702554837985626
Sla	Sla	0.118286967400988	7.16603007943897	1.79504885758584	0.180312218218567	0.702554837985626
Sppl2a	Sppl2a	0.0924850598006203	4.15970011298614	1.79482767249711	0.180339063951291	0.702554837985626
Epha5	Epha5	0.0510998314858627	8.08191064354211	1.79446046028232	0.180383643535696	0.702554837985626
Ptges2	Ptges2	-0.062655677258591	5.19227935586671	1.79410201388561	0.180427171243394	0.702554837985626
Tbl1x	Tbl1x	0.0471840586251297	6.92193868327093	1.79408419678037	0.180429336384886	0.702554837985626
Fam221a	Fam221a	-0.142283445409812	2.34188770748032	1.79403162757535	0.180435719988333	0.702554837985626
Psmc1	Psmc1	-0.0476761883261874	7.48301125284118	1.79377312814941	0.18046711996281	0.702554837985626
Cd83	Cd83	-0.110328932440517	3.35371789039704	1.79321424663462	0.180535029017253	0.702593372881102
Selenot	Selenot	-0.0394690434677937	7.84562494642557	1.79237135240005	0.180637504057885	0.702593372881102
Tmem19	Tmem198b	0.0930550134729494	3.7546400346129	1.79059400983562	0.180852805923835	0.702593372881102
Mrm2	Mrm2	0.0866556527853936	2.74464814184222	1.79051281972428	0.180862693443971	0.702593372881102
Nrros	Nrros	0.129864410492619	1.8303140145035	1.79026914071447	0.18089237425649	0.702593372881102
Rps6ka3	Rps6ka3	0.0580274224157632	6.1471777646464	1.79023963338875	0.180896968717366	0.702593372881102
Gja5	Gja5	0.229591941256476	0.171875064533951	1.78998570280214	0.180927904910522	0.702593372881102
Wdr41	Wdr41	0.0364908701593619	6.09845328886429	1.78958039466291	0.180977296016039	0.702593372881102
Itgb3	Itgb3	0.231516547576316	-0.0451843542779971	1.78921322361082	0.181022052964214	0.702593372881102
Pde3b	Pde3b	0.0596169835248872	4.41214919607019	1.78906496332445	0.181040129345587	0.702593372881102
Zmynd1!	Zmynd19	0.0383547236989027	6.19362327388728	1.78865790969961	0.181333894178908	0.703407600580398
Macf1	Macf1	0.102327378547035	9.31487761336301	1.78642222612353	0.181362687432585	0.703407600580398
Pcyt1a	Pcyt1a	0.0524279016772391	4.69666551507139	1.78579406937719	0.181439454645454	0.703496661159518
Bhlhb9	Bhlhb9	-0.0377778004813236	6.39163125552018	1.78498804668314	0.181538014016794	0.703623038219976
Icam1	Icam1	0.173189746665304	1.28123580264938	1.78441158828244	0.181608540584787	0.703623038219976
Sfrp4	Sfrp4	-0.237138782123152	-0.0769252513388214	1.78410724205557	0.181645788851097	0.703623038219976
Acot1	Acot1	-0.100467904104971	4.73254679409724	1.78366276282884	0.181700202730381	0.703623038219976
Slfn2	Slfn2	0.175016252130953	1.18702366676544	1.78200719862728	0.181903047054665	0.703706079617817
Rab43	Rab43	0.100335818894616	2.53884692054829	1.7808442841393	0.182045687379526	0.703706079617817
Ldlr	Ldlr	-0.0572084611964323	4.94966073059783	1.78021784311314	0.182122578834382	0.703706079617817
Tbc1d14	Tbc1d14	0.064008118278447	8.02032063263738	1.78011422742122	0.182135300594408	0.703706079617817
Pcdhb2	Pcdhb2	0.149550760801332	1.48341917473215	1.78002056901697	0.182146800701182	0.703706079617817
Grik1	Grik1	-0.0608683815582985	5.19862768073842	1.78001649971214	0.182147300381121	0.703706079617817
Astn2	Astn2	-0.0912107658329293	5.48272530556308	1.77904089053345	0.18226714366824	0.703706079617817
Ndufv2	Ndufv2	-0.0567279794440376	6.57385970055106	1.77756620871212	0.18244846619636	0.703706079617817
Cyp2u1	Cyp2u1	0.0795799360620274	3.28023180069888	1.77750653848607	0.182455807469478	0.703706079617817
Ppard	Ppard	0.0603068967046431	4.50783645543197	1.77731291590336	0.182479621358913	0.703706079617817
Pcsk5	Pcsk5	-0.0848043158772724	4.31278458231505	1.77713365058281	0.182501691903823	0.703706079617817
Atad1	Atad1	-0.0346464586809786	7.14193552585588	1.77704706268275	0.182512348581604	0.703706079617817
Slc35a1	Slc35a1	0.0534764132617985	5.22878868814852	1.77627219472665	0.182607746515423	0.703706079617817
Pou4f2	Pou4f2	-0.972535518156231	-0.906456778804955	1.77611882749434	0.182626622865604	0.703706079617817
Grm1	Grm1	-0.0989134903614347	5.37581000604565	1.77565146626632	0.182684209228557	0.703706079617817
Cdk13	Cdk13	0.0401572119804409	6.51456717298181	1.7754495929259	0.182709084430723	0.703706079617817
Lsr	Lsr	0.0842693911257545	4.08033616356563	1.77499734802817	0.18276482512198	0.703706079617817
Snx4	Snx4	-0.0320710437022636	6.69367763244633	1.77463825476443	0.182809098600876	0.703706079617817
Card9	Card9	0.101846630754297	1.73748868131111	1.77443957862908	0.18283359920404	0.703706079617817
Mir3561	Mir3561	0.223314185135811	-0.434712669844965	1.77430942137413	0.182849652169669	0.703706079617817
Slc6a12	Slc6a12	0.340919404522241	-0.592762587972387	1.77297237974071	0.183014651030537	0.704123897905645
Eogt	Eogt	0.0808943353995479	4.2201679410973	1.77165045420251	0.183177954168898	0.704325554710708
Myo7a	Myo7a	0.208687941096229	0.666665927297785	1.77132821229711	0.183217787698663	0.704325554710708
Gtf3c5	Gtf3c5	0.0424457431284157	5.49640924144686	1.77108612710651	0.183247719362458	0.704325554710708
Vps13d	Vps13d	0.0787371238256697	6.92532251963413	1.77070191707701	0.183295235127768	0.704325554710708
Ankle2	Ankle2	0.0726138145258956	5.0235397354575	1.7694473116092	0.183450493367561	0.704325554710708
S100a16	S100a16	-0.0864708801001303	5.1145734466671	1.76931882711382	0.183466402013043	0.704325554710708
Strn3	Strn3	0.0385064533972529	6.62213679650424	1.76867654205429	0.183545852216131	0.704325554710708
Plk3	Plk3	-0.0576786257602465	4.56557468610772	1.76816881720415	0.183608852380885	0.704325554710708
Dus2	Dus2	0.0586330710898351	4.50099490690653	1.76783488009802	0.183650256788674	0.704325554710708
Orai3	Orai3	0.0709976732209421	3.52712548254632	1.76716854058746	0.18373288296376	0.704325554710708
Clec14a	Clec14a	0.161905028586057	3.00393430976126	1.76703139696305	0.183749894136182	0.704325554710708
Ppp1r3d	Ppp1r3d	-0.0700768260572553	3.49171358262779	1.76639986611491	0.183828249752787	0.704325554710708
Nif3l1	Nif3l1	0.0805406946043781	3.62810289608915	1.7661406883189	0.183860421309048	0.704325554710708
Nek11	Nek11	0.104262141356264	2.14052982016422	1.76543915365701	0.183947529353889	0.704325554710708
Dst	Dst	0.0900666153673646	8.99287213204249	1.76512032119206	0.183987132459634	0.704325554710708
Tomm40l	Tomm40l	-0.0451792713185526	4.52169815603061	1.76506466024207	0.183994047456414	0.704325554710708
Hsp90ab	Hsp90ab1	-0.0419992203362625	11.1913469107261	1.76451085488044	0.184062865479485	0.704325554710708
Glrb	Glrb	-0.0562711210222337	6.17755371940304	1.76434755281265	0.1840831637342	0.704325554710708
Gle1	Gle1	0.0469412277233709	4.98185096038292	1.76241379308947	0.18432372535822	0.705029773542154
Maff	Maff	-0.126764653302267	0.850972819018658	1.76169287000844	0.184413502225603	0.705057863576248
Chrnb4	Chrnb4	0.27258814589908	0.525874953234884	1.76089580874784	0.184537750335384	0.705057863576248
Zfand2a	Zfand2a	-0.0381233317348975	6.11813010092056	1.76030499896341	0.184586477330065	0.705057863576248
Gaa	Gaa	-0.0682984138056571	7.08558055548188	1.76028596883927	0.184588850427622	0.705057863576248
RT1-O1	RT1-O1	0.464417282858687	-0.512589324454803	1.76008741789613	0.184613612270866	0.705057863576248
Ncoa3	Ncoa3	0.0500938108664737	5.69436110400714	1.75913300020709	0.184732694188571	0.705086228180547
Dnajc28	Dnajc28	0.130608058302609	1.6898946394798	1.75878280176856	0.184776410518519	0.705086228180547
LOC500	LOC500475	-0.171092428052582	0.950234831400315	1.75847864777357	0.184814388725265	0.705086228180547
Coq8b	Coq8b	0.0569577443063242	5.30783747498005	1.75780746163637	0.184898228486313	0.705086228180547
Sspn	Sspn	0.1217151074861	2.78412827986608	1.75711582858796	0.184984668521385	0.705086228180547
Rph3al	Rph3al	-0.121497887348947	1.70356635973698	1.75608887061532	0.18511312892223	0.705086228180547
Zfp329	Zfp329	0.0966877369661148	5.58451897447717	1.75499678895875	0.185249797524763	0.705086228180547
Arhgap3	Arhgap31	0.0873934541761421	4.01255313803915	1.75497340610644	0.18525272556943	0.705086228180547
Ndufa12	Ndufa12	-0.066979404677529	5.90368475231493	1.75472382772614	0.18528400560189	0.705086228180547
Klf5	Klf5	0.0992476948255596	3.52697539767432	1.75338998090756	0.185451131500734	0.705086228180547
Pkig	Pkig	-0.0578486868895093	5.77992573505249	1.75304304010157	0.185494826738508	0.705086228180547
Prelid1	Prelid1	-0.0475430894087773	7.20268554896998	1.7526667797851	0.185541801945158	0.705086228180547
Raf1	Raf1	0.0323031187949454	7.67460268830179	1.75266135255362	0.185542512797449	0.705086228180547
Uchl1	Uchl1	-0.0539375852316666	10.1682535681539	1.75238660485686	0.185576980996971	0.705086228180547
Nkain4	Nkain4	-0.0736837227298873	3.92842144165617	1.75196365146459	0.185630064353406	0.705086228180547
Ccdc24	Ccdc24	-0.194785749561276	0.162774363743513	1.75190023920761	0.185638024067575	0.705086228180547
Col14a1	Col14a1	0.157408062759047	3.34592325260556	1.75166908538503	0.185667168158302	0.705086228180547
Bag5	Bag5	-0.040973685829116	5.4377049013379	1.75156676304045	0.185679889613052	0.705086228180547
Vrk2	Vrk2	0.175232525725328	0.743082772400517	1.75094612302801	0.185757834470135	0.705086228180547
Pacsin1	Pacsin1	-0.0872511698901543	5.48441321925353	1.74988817177088	0.185890791349603	0.705086228180547
Mvp	Mvp	0.117561603355858	2.69100527837622	1.7490937472452	0.185990701526405	0.705086228180547
LOC102!	LOC10254	0.0694300705064111	5.20582712576743	1.74908296734233	0.185992057681401	0.705086228180547
Paqr8	Paqr8	-0.0866725823490975	4.48873231439185	1.74897460065857	0.186005691282912	0.705086228180547
Spaca5	Spaca5	0.225048368716521	-0.722907034943199	1.74891691405797	0.186012949301852	0.705086228180547
Dopey2	Dopey2	0.0788273429622433	6.99389550345684	1.74875111810954	0.186033811266359	0.705086228180547
Dancr	Dancr	0.14764998018217	2.05391247675468	1.74828552621312	0.186092410940322	0.705094142136258
Iws1	Iws1	0.0507613354238864	5.55182581385623	1.74637118881443	0.186333575800187	0.705208077516553
Rab11a	Rab11a	-0.0315603251814253	7.80436369850041	1.74630007977423	0.186342540758997	0.705208077516553
Scfd2	Scfd2	0.0680808344214996	4.4533869374294	1.74587461139001	0.186396192810198	0.705208077516553
Zfp207	Zfp207	0.0365054674996083	7.24886760000298	1.74568993752163	0.186418295512402	0.705208077516553
Hyal2	Hyal2	0.0613350035508607	4.94103704588244	1.7455064009486	0.186442639061267	0.705208077516553
Kcng2	Kcng2	-0.134945736673129	2.98060839071198	1.74535607458287	0.186461605171685	0.705208077516553
Akap14	Akap14	-0.214884467527081	-0.153107403496544	1.74471780875457	0.186542157740646	0.705298940039407
Klf14	Klf14	-0.316071447614945	-0.221519195131478	1.74364590958823	0.186677528187447	0.705599461135952
Nfic	Nfic	0.0693958598847308	3.9821566666555	1.74221847098038	0.186857976381217	0.705885888117516
Ipo9	Ipo9	0.0412977925164771	7.31879457476289	1.74214616876247	0.186867122581723	0.705885888117516
Wdr45	Wdr45	-0.0633495331635345	3.9972981400309	1.74148281934794	0.186951053364516	0.705953330220866
Acat2	Acat2	0.0581411413239166	8.12559989917373	1.74111084337306	0.186998137082629	0.705953330220866
Lamb2	Lamb2	0.11114209343818	5.4949343942806	1.7398803591408	0.187153986742041	0.706258476282952
Prorp	Prorp	-0.0555794579180386	3.90527655148026	1.7395790234385	0.187192176082073	0.706258476282952
Ntn4	Ntn4	-0.0739370221616334	3.94455395857371	1.73829361418665	0.18735518241282	0.706659797756493
Kmt5c	Kmt5c	0.105529138762748	5.27650247758643	1.73784145765041	0.187412559617934	0.706662588804161
Ube2j2	Ube2j2	-0.0404278467697743	6.52149326181566	1.73719190811032	0.187495024224319	0.706758944787563
Slc25a22	Slc25a22	-0.0336893157828186	7.33715134105726	1.73589376485667	0.187659956053043	0.707014811059692
Flnc	Flnc	0.106255455230399	4.42779667566662	1.73576780797995	0.187675968119716	0.707014811059692
Igfbp7	Igfbp7	0.0962412478153215	5.52988540474556	1.73494388039423	0.187780735089669	0.707195964900028
Shq1	Shq1	0.105512679707789	1.96893614630673	1.73244782845162	0.188098587630059	0.708105754465574
Ints6	Ints6	0.0643951033128448	4.89868788510472	1.73142202755717	0.188229393342252	0.708105754465574
Herc2	Herc2	0.0801715081877324	8.32594973972928	1.73123081933805	0.188241028832296	0.708105754465574
Olfm1	Olfm1	-0.0612265889073075	9.25491466916718	1.73126581680754	0.188249321757018	0.708105754465574
Tex30	Tex30	-0.0822911578811754	2.96743108265708	1.73052782227119	0.188343503811847	0.708246503032072
Rprd1a	Rprd1a	0.0394450840921016	6.59668595097089	1.7292882574671	0.188501806395549	0.708628212934367
Ptar1	Ptar1	0.113405269451543	2.85741982949699	1.72830248026708	0.188627821755267	0.708793887749852
Znrf1	Znrf1	-0.0616006293674367	4.17035248628078	1.72805491527299	0.188659493565541	0.708793887749852
Sema3c	Sema3c	-0.0877472871887176	6.86054869434232	1.7246858513216	0.189090988725988	0.710096403180397
Glyctk	Glyctk	0.0721292662657287	2.77907000187806	1.72445958861429	0.189120008637369	0.710096403180397
Degs1	Degs1	0.0480598510750807	5.76503097578491	1.72371084655354	0.189216077487374	0.710243381110099
Pik3cb	Pik3cb	0.061574409367817	5.00850135166914	1.72255548764429	0.189364429679705	0.710450276048047
Dnajc11	Dnajc11	-0.0280884949312003	6.62574337551593	1.72204534301966	0.189429677182084	0.710450276048047
Hnrnpdl	Hnrnpdl	-0.0552242600589342	7.58037898528013	1.72185164769235	0.189442018787517	0.710450276048047
Tmem15	Tmem150a	0.0993929465376843	4.58030767003767	1.72142936578693	0.189509158142401	0.71048851146116
Pdrg1	Pdrg1	-0.0562238230859197	5.28186625952121	1.71931272428814	0.189781526409835	0.711295953900311
Paip2	Paip2	-0.0426488187307332	6.79632518234634	1.71748221571767	0.19001746192348	0.711644047486082
Ube2g2	Ube2g2	0.0633009443534592	5.80756302285752	1.71737837520335	0.190030855670364	0.711644047486082
Fbxo2	Fbxo2	-0.0360657243208911	6.74230693665008	1.71726469556635	0.190045521055634	0.711644047486082
Smarcd3	Smarcd3	-0.0437245857981408	7.60154799089661	1.71631384268419	0.190168226630883	0.711679256837462
Mthfd2l	Mthfd2l	-0.0901491636876635	2.86638798801811	1.71630782394368	0.190169002348008	0.711679256837462
C1galt1c	C1galt1c1	0.0750801838582919	3.82361995862264	1.71001874483257	0.190982888471722	0.714510981502749
Sar1b	Sar1b	-0.0551603975476795	6.21536009423365	1.70950240928371	0.191049943075943	0.714547404004358
Snap25	Snap25	-0.0506535050568183	8.31981734685015	1.70880147224675	0.19116692290402	0.714770681394503
Sbf2	Sbf2	0.0480348967271255	7.97549336926865	1.7071706960507	0.191352870085816	0.715251575812079
C3ar1	C3ar1	0.283872906660446	-0.294631694279683	1.70551596784657	0.191568185845438	0.715820161340961
Vezf1	Vezf1	0.0381205065450663	6.42585718497913	1.70517920119971	0.191612043052125	0.715820161340961
Tbc1d9b	Tbc1d9b	-0.0501820091120346	6.6489510272654	1.70501477487147	0.191633457615113	0.715820161340961
Exo1	Exo1	0.118205722066967	1.37636497801681	1.7046270689976	0.191683967445018	0.715820161340961
March10	March10	-0.160104377594742	0.639529475061581	1.70418003674515	0.191742225526959	0.715634982919496
Fundc1	Fundc1	-0.0517317218964904	4.62936248653068	1.70245097502798	0.191967754733474	0.716182750022655
Fbln5	Fbln5	0.203065138893449	2.95984652412735	1.70217491689078	0.192003790881284	0.716182750022655
Snapc3	Snapc3	0.0532131124524603	4.95897306709009	1.70041674404951	0.192233485099519	0.716721365973555
RGD130	RGD130458	0.060443312699907	4.30117190769336	1.69988716356031	0.192304765229624	0.716721365973555
Zdhhc24	Zdhhc24	0.0649146945095493	3.89801897673788	1.69912817101852	0.192402032800971	0.716721365973555
Mpi	Mpi	-0.0385216123470716	5.36915087664176	1.69907442601195	0.192409066493687	0.716721365973555
Kras	Kras	0.0421784970680842	5.51375347477314	1.69887317472696	0.192435407230216	0.716721365973555
Foxa1	Foxa1	-0.457342392777653	-0.0049316415748778	1.69656682468703	0.192737561176542	0.717477449722537
Prmt9	Prmt9	0.0474211782809857	4.58777753712972	1.69601975873875	0.192809329571657	0.717477449722537
Fam180a	Fam180a	0.288576206427145	1.77391115512856	1.69600761143843	0.192810923212552	0.717477449722537
Irf2	Irf2	-0.0746193950305463	2.79021251088945	1.69429225649245	0.193037424109044	0.718106161412903
Pfkl	Pfkl	-0.0356376572388566	7.47780923988846	1.69366617538257	0.193118390798264	0.71819319028435
Serinc5	Serinc5	0.0562075517314081	5.70434981099658	1.69278782396709	0.19323387864197	0.718408552986465
Rpl35	Rpl35	-0.0524481879589695	8.39171127768493	1.69162551757643	0.193386828731289	0.718763021173752
Ptpn20	Ptpn20	-0.286746969645508	-0.363139774298182	1.69029215231443	0.193562461547431	0.718998135648248
Rhoj	Rhoj	0.0873889629027379	3.25167852289189	1.69001000823226	0.193599648871344	0.718998135648248
Ccdc33	Ccdc33	-0.285278647318776	0.491551460080486	1.68983215210034	0.193622965118066	0.718998135648248
Arr3	Arr3	-0.331997978630611	0.350273261013855	1.68724024813446	0.193965165053863	0.719625109189424
Etv5	Etv5	0.0547650288329579	5.96644083374923	1.6866913015148	0.194037703305862	0.719625109189424
Polr3g	Polr3g	-0.0737935468801341	3.27817065285869	1.68668851108379	0.194038072116599	0.719625109189424
Sp8	Sp8	-0.164510246708957	3.42044180835724	1.68629979135801	0.194089457154009	0.719625109189424
Ankh	Ankh	0.0459095125907659	6.47455286004344	1.68610651762889	0.194115012018431	0.719625109189424
Cldn10	Cldn10	-0.095849236715151	2.71332864670255	1.68591331938372	0.194140560834464	0.719625109189424
LOC689	LOC689574	0.0726284984966675	4.49712174528234	1.68472981390842	0.194297155167114	0.719637836683081

Traf4	Traf4	0.0485086708288666	6.91309958506964	1.68447986379746	0.194330404836704	0.719637836683081
Pfn3	Pfn3	0.147514028421484	1.44060287214444	1.68436985969618	0.194344811436996	0.719637836683081
Eif2ak2	Eif2ak2	-0.0558084226174116	4.07685706686848	1.68416450735597	0.194372005259436	0.719637836683081
Wdfy1	Wdfy1	0.0409453369463022	5.7382807762355	1.68238062819135	0.194608423145005	0.720105016652
Csrnp2	Csrnp2	0.0439975027799654	6.86280177077687	1.6815252549473	0.194721891976942	0.720105016652
Zfp358	Zfp358	0.0447701368500203	6.03637383490313	1.68140952106119	0.194737264245065	0.720105016652
Mrpl42	Mrpl42	0.0672186798450389	4.26043871090851	1.6810207601705	0.194788872708682	0.720105016652
Itpripl2	Itpripl2	0.185440653723858	0.507978206445948	1.68079885768375	0.194818323659963	0.720105016652
Cdk5r2	Cdk5r2	-0.0709526519311626	8.18899044814617	1.68060202518852	0.194844476738136	0.720105016652
Rab7b	Rab7b	-0.153052118793072	1.16191146288919	1.67913784067746	0.195039052597011	0.720440621536424
Coq5	Coq5	-0.0672861157889267	4.2527444525962	1.67887247459678	0.195074341497282	0.720440621536424
Wdr20	Wdr20	0.0449243685271438	4.67928560093704	1.67847715898865	0.195126925461522	0.720440621536424
Slc2a3	Slc2a3	-0.0580835905257 84	5.39349514139362	1.67819160332917	0.195165249963382	0.720440621536424
Prmt8	Prmt8	-0.0505171455915374	6.02680186018049	1.67676989071614	0.195354211520863	0.720710713802775
Bak1	Bak1	0.0454280282013134	4.92369270200427	1.67676439376295	0.195354843823113	0.720710713802775
Rtkn2	Rtkn2	0.0865213729299308	3.10510077251021	1.67607684696898	0.195446550586039	0.720760843157323
Caskin1	Caskin1	-0.0607537274219604	8.50921277204657	1.67579552695082	0.19548406614125	0.720760843157323
Sirpa	Sirpa	-0.0544344405035285	6.96684703317543	1.67411538352033	0.195708218277191	0.721265333682978
Tmem33	Tmem33	0.0452510547758756	6.07842366733692	1.67362765449411	0.195773343769745	0.721265333682978
Cep83os	Cep83os	0.107455147991572	2.61404549352687	1.67347062987308	0.195794316356936	0.721265333682978
Tsr2	Tsr2	-0.0370445152638241	5.84725715442821	1.67246826352884	0.195928256837329	0.721545708547626
Otx2	Otx2	0.282899840958855	3.27784126207371	1.67157169041585	0.196048151688309	0.72156060000498
Phyhipl	Phyhipl	-0.0474646229726537	7.14082556060858	1.67123686012687	0.196092949131275	0.72156060000498
Coch	Coch	-0.0966312125333637	4.21148027625401	1.67114086830753	0.196105794230736	0.72156060000498
Calm1	Calm1	-0.0487218322114687	10.724569005369	1.67038676575569	0.19620673839129	0.721719184819613
Tle5	Tle5	-0.0443706658984226	9.01750592357489	1.66942584858022	0.196335454957297	0.721956473279057
Gpatch2l	Gpatch2l	0.0445769854096132	5.36520022000572	1.66821686818606	0.196497527154093	0.721956473279057
Arid5b	Arid5b	0.0053505483700752	5.08191688254941	1.66765199218315	0.196573326107287	0.721956473279057
Camk2a	Camk2a	-0.103628853496865	6.92915738961605	1.66694312598393	0.196668476939655	0.721956473279057
Sat1	Sat1	0.0917192618795179	3.56469304189777	1.66644151488786	0.196735842899882	0.721956473279057
Rundc3a	Rundc3a	-0.0307461813051246	7.75267970362564	1.66642632941041	0.196737882678054	0.721956473279057
Bpifa1	Bpifa1	-0.286329361358864	-0.759150768998273	1.66622336183777	0.196765148528396	0.721956473279057
Rwdd4	Rwdd4	0.0402616526796048	5.58057810848155	1.666116598504	0.196779493538829	0.721956473279057
Fst	Fst	0.175284947157563	2.47591194082844	1.66565991598264	0.196840862797953	0.721956473279057
Cntn5	Cntn5	0.0770808082979263	5.78329153662226	1.66559284752516	0.196849877542306	0.721956473279057
Tmem209	Tmem209	0.0775022311213498	3.29659495317008	1.66357189659218	0.197121769769794	0.722591597036159
Sarm1	Sarm1	0.0397679792246186	7.02509164491955	1.66340686229494	0.197143849791449	0.722591597036159
Mrpl34	Mrpl34	0.0747013859441703	4.98940355016903	1.6615607729162	0.197392723144524	0.722591597036159
Apol3	Apol3	0.205963669506589	1.75150287257991	1.66136780637204	0.197418748553884	0.722591597036159
Slc26a2	Slc26a2	0.0820624806598 06	3.24282485070092	1.66105062817137	0.197461529849374	0.722591597036159
Gpx3	Gpx3	-0.117210539221724	5.10802475984748	1.66052282538539	0.197532747814413	0.722591597036159
Mcm6	Mcm6	0.0525971427830297	5.66834162264959	1.66035006254185	0.19755603875347	0.722591597036159
Fbxo9	Fbxo9	-0.0388031357765714	6.14768979114139	1.65967218611269	0.197647590440408	0.722591597036159
Kcnk6	Kcnk6	0.163782443813292	0.911078673850457	1.65956223701731	0.197662440098794	0.722591597036159
Pgrmc1	Pgrmc1	-0.0443322469703706	7.98531039437569	1.65875037403296	0.197772130329904	0.722591597036159
Smim12	Smim12	-0.0753346093294386	3.84821997761883	1.65827743887711	0.197836061166883	0.722591597036159
Litaf	Litaf	0.0586708766422868	3.85297210782822	1.65639433879424	0.198090857009884	0.722591597036159
Rnf121	Rnf121	0.0441841631583714	4.47380590520417	1.6562716447192	0.198107471678862	0.722591597036159
Cul4b	Cul4b	0.0429230557724876	6.20961191169021	1.65573730386452	0.198179868731722	0.722591597036159
Sox9	Sox9	0.0920957741421983	5.18801341005389	1.65570918319347	0.198183658566476	0.722591597036159
Slc7a3	Slc7a3	-0.0980115049049893	4.91694631325257	1.65554677967282	0.19820568294267	0.722591597036159
Erbb4	Erbb4	0.0854936512271183	5.54117873928896	1.65542738326697	0.198221842029058	0.722591597036159
Uqcrh	Uqcrh	-0.0609738414780536	6.22674534459526	1.65494624038145	0.198287056108845	0.722591597036159
Ggta1	Ggta1	0.12685418107454	1.45582811396834	1.65450810454627	0.198346462916648	0.722591597036159
Heph	Heph	0.161345015033651	2.55152948117771	1.65417872091702	0.198391127769941	0.722591597036159
Spdya	Spdya	-0.121315143875796	1.36261259421676	1.65397214209015	0.198419162426658	0.722591597036159
Hcn2	Hcn2	-0.0629832342858813	6.56833489847618	1.65374531279045	0.198449839630028	0.722591597036159
Nos1ap	Nos1ap	0.0688200809946838	4.59938023032896	1.65363075451282	0.198465485533104	0.722591597036159
Homer1	Homer1	0.0527301601772747	6.64627965764347	1.65300431487269	0.198550520554656	0.722591597036159
Slc16a12	Slc16a12	0.16442318453524	1.92090965891262	1.6528434442956	0.198572364566285	0.722591597036159
Sez6l2	Sez6l2	-0.0498633090023764	8.73485304022014	1.65284012853366	0.198572813337062	0.722591597036159
Zmiz1	Zmiz1	0.0702124140605642	8.61205728637583	1.65178496884726	0.198716161184132	0.722591597036159
Cpox	Cpox	0.0700203560142133	4.38118980763057	1.65137332559608	0.198772117172648	0.722591597036159
Narf	Narf	0.0451515445937989	6.56777678091357	1.65010656444924	0.198944418325498	0.722591597036159
Vamp8	Vamp8	0.197882927679078	1.68866877723402	1.64980554808811	0.198985396101325	0.722591597036159
Cd300f	Cd300f	-0.223197252004443	-0.0411778958691 98	1.64944233732521	0.199034848965325	0.722591597036159
Ptcd2	Ptcd2	-0.0675490899634924	3.41505616626015	1.64943081915573	0.199036417349128	0.722591597036159
Tiam1	Tiam1	0.0649525850193046	6.02461869232334	1.64941395304979	0.199038713981 21	0.722591597036159
Fbln1	Fbln1	0.128559681373876	4.7272707794135	1.6490765589879	0.199084662955752	0.722591597036159
Arfip2	Arfip2	-0.0328641657636636	6.2533755987857	1.64848815026154	0.199164826880409	0.722591597036159
Cul5	Cul5	0.0550057208811427	5.76331591639797	1.64797225822765	0.199235142420366	0.722591597036159
Gucd1	Gucd1	0.0827489174511773	3.12474856855863	1.64794909455018	0.199238301652146	0.722591597036159
Gpcpd1	Gpcpd1	-0.0592051217888571	6.18976944947376	1.64766891694094	0.199276501125685	0.722591597036159
Ly6i	Ly6i	-0.198674158985201	-0.27289520895463	1.64704446522438	0.199361672989963	0.722591597036159
Nfkbiz	Nfkbiz	0.191519649755131	-0.0754449426284 73	1.64536709567929	0.199590668916549	0.722591597036159
Ccnj	Ccnj	0.0657406823721217	4.95779325848246	1.6449875380234	0.199642529303463	0.722591597036159
Stard8	Stard8	0.122240794327283	2.38302083259509	1.64448775898938	0.199710840130476	0.722591597036159
Spring1	Spring1	0.0545990810940519	5.15894632609577	1.64433685174338	0.199731471839565	0.722591597036159
Adcy7	Adcy7	-0.0706051149732087	4.27061734197451	1.64429052077319	0.199737805243611	0.722591597036159
Peg12	Peg12	0.127837709649938	1.11666255859167	1.64420462507709	0.199749550271085	0.722591597036159
Trpc5	Trpc5	0.0615829426036 41	5.28398930446208	1.64411967771658	0.199761126767396	0.722591597036159
Fcsk	Fcsk	0.0743506236349127	3.97197817641965	1.64332687940177	0.199869617520094	0.722591597036159
Amigo2	Amigo2	0.0598743445970093	4.50551643444257	1.64329806429916	0.199873560543829	0.722591597036159
Ddx3y	Ddx3y	-0.374409865348225	-0.644049452838336	1.64271628334701	0.199953190266075	0.722591597036159
Rbp1	Rbp1	0.127103750158536	4.58938602849754	1.64269180663155	0.199956541299648	0.722591597036159
RGD156	RGD156054	-0.0582268114884947	3.99688836245183	1.64254486517888	0.199976658819336	0.722591597036159
Serpine2	Serpine2	-0.0727052115387946	7.05302661610764	1.64107516600426	0.200178014886781	0.723109754445471
Il17ra	Il17ra	-0.0812420514571661	4.03962559401889	1.64004718947727	0.200318993670378	0.723409575684735
Lpcat1	Lpcat1	-0.0276492363231405	7.55178393410793	1.63889512037036	0.20047712942985	0.723562512184025
Nfkb1	Nfkb1	0.0590823865230919	4.17903165471538	1.63634011275584	0.200828356722369	0.723562512184025
Mgp	Mgp	0.240628644695781	1.74875496314076	1.63591186345104	0.20088730080647	0.723562512184025
Smpd3a	Smpd3a	-0.123402265844844	1.98592308501837	1.63489622106334	0.201054723724532	0.723562512184025
Mrps35	Mrps35	-0.0659710916071472	5.0157895276048	1.6345377235406	0.201076554697284	0.723562512184025
Il10rb	Il10rb	0.0982431795573547	1.98388897192586	1.63376298519865	0.201182363867333	0.723562512184025
Samd15	Samd15	0.106824537017939	1.69896176465842	1.63354259793874	0.201213756860743	0.723562512184025
Gal3st1	Gal3st1	-0.101431879070654	2.26782673323313	1.63281752623402	0.201313787593953	0.723562512184025
Xpc	Xpc	0.0535729297138198	5.14851882452781	1.63279900673741	0.201316343305539	0.723562512184025
Nog	Nog	-0.075970173253012	3.29573301487089	1.63271215845559	0.201328328838117	0.723562512184025
Entpd7	Entpd7	0.0926710177060064	3.16446014117806	1.63244180317174	0.201365621466058	0.723562512184025
Tmem9b	Tmem9b	-0.0487272621526212	5.41076788949616	1.63182142632965	0.201437494159086	0.723562512184025
Brd1	Brd1	0.0400328888960688	6.51184006726544	1.6316414130612	0.201476168315817	0.723562512184025
Elmo1	Elmo1	-0.0561887367555 52	6.52147503280379	1.63089828676831	0.201578847887858	0.723562512184025
Mybl1	Mybl1	-0.136729738958808	2.35308973464267	1.63087128253289	0.201582580287847	0.723562512184025
Chst3	Chst3	0.140807812868362	1.31087197059526	1.6307239166221	0.201602949951959	0.723562512184025
Taf9	Taf9	-0.0502801093052561	4.78552930475455	1.63062237156416	0.201616849189783	0.723562512184025
Cldn5	Cldn5	0.10049509326421	6.75610076756499	1.6305228767541	0.201630742607862	0.723562512184025
Prrt4	Prrt4	-0.0504366713201017	5.07239145040465	1.63051639706212	0.201631639847176	0.723562512184025
Rnf168	Rnf168	0.0574070547018939	4.65030713322554	1.62922742517749	0.201809929805538	0.723562512184025
Ubap2l	Ubap2l	0.0374897152501751	7.9054341654193	1.62896706409162	0.201845877638215	0.723562512184025
Slc6a13	Slc6a13	0.258491098270785	4.423304107547	1.62850814068206	0.201909517876981	0.723562512184025
Bspry	Bspry	-0.0964067557797534	2.80915602602532	1.62816867610053	0.201956505884435	0.723562512184025
Srgn	Srgn	0.137107630593089	3.48222570798413	1.62814922121838	0.201959228471966	0.723562512184025
Gng11	Gng11	0.153785484831202	2.49381179609878	1.62800955053179	0.201978576855154	0.723562512184025
Plrg1	Plrg1	-0.0417248189746487	5.72994222880621	1.62777053778966	0.20201072100335	0.723562512184025
Dapk2	Dapk2	-0.210296659932675	-0.161594302333937	1.62746528380545	0.202053994153646	0.723562512184025
Rrad	Rrad	-0.133559777687997	1.77693867961748	1.62676754611198	0.202150725917069	0.723562512184025
Myt1l	Myt1l	0.0501862597574767	8.43834058396368	1.62555971997605	0.202318303314982	0.723562512184025
Cops6	Cops6	-0.0482511221040897	6.70474431347892	1.62542969882182	0.202336352583736	0.723562512184025
Kxd1	Kxd1	-0.0514358880216606	4.37912452436689	1.62493214870511	0.202405438972226	0.723562512184025
Ing2	Ing2	-0.0601669717608224	4.16418658806921	1.62445162231276	0.202472187923869	0.723562512184025
Stmn3	Stmn3	-0.0647267041056752	9.47751179657465	1.62435433468798	0.202485705108385	0.723562512184025
Rps6ka6	Rps6ka6	-0.0709452255016926	4.8609399188751	1.62371645320741	0.202574358938263	0.723562512184025
Casp9	Casp9	-0.0484803677515211	4.69598569226238	1.62333892724776	0.202626849652178	0.723562512184025
Synpr	Synpr	-0.0972461876838277	6.47496714513037	1.62293071512319	0.202683624950214	0.723562512184025
Nptx1	Nptx1	-0.0658237010884585	5.8994865521017	1.62278089168807	0.202704467519207	0.723562512184025
Mllt10	Mllt10	0.0585196772004234	5.87738057585001	1.62235735704677	0.202763400948086	0.723562512184025
Trim66	Trim66	-0.142788559228528	2.02432639398582	1.62230476502125	0.202770720248526	0.723562512184025
Cox5b	Cox5b	-0.085689491318049	6.76934659061488	1.62212982728553	0.202795234774685	0.723562512184025
Gpam	Gpam	0.036083440272868	6.72563655702562	1.62078059227032	0.202982979478184	0.723562512184025
Klf15	Klf15	0.182483618386199	0.15121450007345	1.62032237798705	0.203046703029758	0.723562512184025
Cd164l2	Cd164l2	0.193188388581367	0.0879195400161502	1.61984850671642	0.203112912445419	0.723562512184025
Mthfd1l	Mthfd1l	-0.0682829517386283	5.09570159711979	1.6195671392999	0.203152154822968	0.723562512184025
Lrig2	Lrig2	0.0734805325857528	5.22890653953848	1.61946311128617	0.203166665293133	0.723562512184025
Ahi1	Ahi1	-0.0911764773005385	7.66157402136108	1.61927392890898	0.203193056430976	0.723562512184025
Pola2	Pola2	0.0798121851400655	3.16996770881234	1.61888811998062	0.203246893087057	0.723562512184025
Slc30a9	Slc30a9	-0.03226660908576	7.35347630680323	1.61873718262473	0.203267958364921	0.723562512184025
Dvl3	Dvl3	0.0474034615799853	6.79227257681697	1.61852990316713	0.203296893483305	0.723562512184025
Pantr1	Pantr1	0.0949446052925033	4.21963047213834	1.61834947502929	0.203322223000501	0.723562512184025
Tcp11l1	Tcp11l1	0.0718190009355297	5.53486576065026	1.61809881302741	0.203357084981326	0.723562512184025
Psmb10	Psmb10	-0.0782988446252 81	3.75444792769686	1.61788994688431	0.203386255534408	0.723562512184025
Kmt5b	Kmt5b	0.0455331726992056	7.16198357322524	1.61754169914518	0.203434903644838	0.723562512184025
Bin2	Bin2	0.130906725607706	1.77304225222145	1.61708793405993	0.203498312141322	0.723581772752146
Ccnh	Ccnh	0.0496349824065792	5.49433987415753	1.61438579720021	0.203876388384027	0.724173108419296
Taf7	Taf7	0.0493784268936895	4.25477545752116	1.61432555524403	0.203884826726606	0.724173108419296
Rlbp1	Rlbp1	-0.0568946023 0842	5.51976106926981	1.61430042558111	0.203888345460211	0.724173108419296
Efnb3	Efnb3	-0.0452765174068481	8.55361221765059	1.61407070007188	0.203920529867949	0.724173108419296
Pcdhb20	Pcdhb20	0.0773150783260437	3.49628294938239	1.6138259727895	0.203954821109674	0.724173108419296
LOC691	LOC691807	-0.0601788814725725	5.33461125404171	1.61320828274384	0.2040414022082	0.724274415885268
Zfyve26	Zfyve26	0.0782873003544455	5.04088454295273	1.61221431014297	0.204180817487013	0.724563156935569
Mterf1	Mterf1	0.098623901298637	2.86276228542514	1.61141342114477	0.204293232425169	0.724746310749128
Nek6	Nek6	-0.0469186870173256	5.45488819394089	1.61101912908922	0.204348603126988	0.724746310749128
Actr1b	Actr1b	-0.0342976323992742	7.26146082232998	1.61048043727246	0.204424280582311	0.724808681109829
Vamp1	Vamp1	-0.118209680636015	4.52831018432458	1.60981449328678	0.204517880586403	0.724934544339929
Spem2	Spem2	0.239459061738515	-0.518818243029948	1.60918909773353	0.2046058274283	0.725019097235503
Birc2	Birc2	0.0497451781493324	5.47053738383031	1.60846480061054	0.204707738141023	0.725019097235503
Phex	Phex	-0.240695950117084	-0.365510647345856	1.60840567509794	0.204716059915098	0.725019097235503
Safb	Safb	0.0450055105196085	7.48518562055544	1.60748545505967	0.204845630121543	0.725272113231127
Acsf2	Acsf2	-0.0655668581262 6554	4.97004200524347	1.60659889125403	0.204970547270056	0.725385980470088
Olp5	Olp5	-0.102845956677067	2.17039410662174	1.60522195451308	0.20516475075281	0.725385980470088
Atp5f1c	Atp5f1c	-0.0522617720506927	7.80748798817894	1.60485677035909	0.205244524976215	0.725385980470088
Dpy19l3	Dpy19l3	0.0573615290375159	6.88982390799414	1.60458042780051	0.205255303328257	0.725385980470088
Hpcal1	Hpcal1	-0.0784972898838904	5.9521280761237	1.60448427963685	0.205268878866174	0.725385980470088
Postn	Postn	0.15190137254 0761	3.86376466411609	1.60434755445338	0.205288185457791	0.725385980470088
Aass	Aass	-0.0451926737351605	5.30722115863842	1.60426922123833	0.205299247643641	0.725385980470088
Cks2	Cks2	0.114879638599652	2.1192521259074	1.60396020564025	0.205342893565789	0.725385980470088
Sipa1l2	Sipa1l2	0.0642013368143148	6.77517092364072	1.60342642840883	0.205418309608159	0.725446999428532
Dnajc10	Dnajc10	-0.0294397858040524	7.21752040334939	1.60283962298793	0.20550134338975	0.725524878741911
Mplkip	Mplkip	0.0568151617455186	4.07083462017736	1.60165578487483	0.20566871964612	0.725757313285235
Tbl3	Tbl3	-0.0339126101174664	5.54507699019012	1.60122228201305	0.205730081541376	0.725757313285235
Asgr1	Asgr1	0.264810862740829	0.120762704455553	1.60089452917051	0.205776487556678	0.725757313285235
Otud7b	Otud7b	0.0747241192759574	4.22441381871731	1.59975266779226	0.205938264830517	0.725757313285235
Grik4	Grik4	-0.082025785184243	4.7615640143702	1.59939305792405	0.205989244477176	0.725757313285235
Mapkapk3	Mapkapk3	-0.104783446931975	2.69106062772314	1.59931038769773	0.206000966225361	0.725757313285235
Wwox	Wwox	-0.0927421588024034	2.12862768025905	1.59922867580291	0.206012552884677	0.725757313285235
Trim17	Trim17	0.181040342545636	0.510653775760405	1.5988986423361	0.20605935901529	0.725757313285235
Napsa	Napsa	0.176935602679088	0.0418930453977048	1.59847682974313	0.206119199783071	0.725757313285235
Tmem200a	Tmem200a	0.0599271744296907	4.85221431490734	1.59788090302709	0.206203776360004	0.725757313285235
Hmbs	Hmbs	-0.0680049340352034	3.84023356578328	1.5972968139241	0.206286712658899	0.725757313285235
Cdk2	Cdk2	0.0704354992109644	3.52210380817141	1.59716676613181	0.206305183837218	0.725757313285235
Rpgr	Rpgr	0.0682035940187367	4.28770767732 55	1.59689221853525	0.206344185282139	0.725757313285235
Alas2	Alas2	0.172430184648551	3.18572354727858	1.59603682123214	0.206465742184686	0.725757313285235
Adora2b	Adora2b	-0.117324718168599	2.24519105454768	1.59570712848589	0.206512634302842	0.725757313285235
Chst15	Chst15	0.0699823017432052	4.10179077477608	1.59544790628603	0.206549503595076	0.725757313285235
Vtn	Vtn	0.114331047612343	3.87991200403087	1.59542195130986	0.206553195468981	0.725757313285235
Ttc39a	Ttc39a	-0.1388248655211	1.42535897114307	1.59476822298098	0.206646208458884	0.725870881126743
Sec63	Sec63	0.0396979984928205	6.72757968652855	1.59344389585158	0.206834814952465	0.725870881126743
Hspa1l	Hspa1l	0.10101483742801	2.17479049883996	1.59338276197822	0.206843496544145	0.725870881126743
Ddx3x	Ddx3x	-0.0465633666374846	8.8304984306896	1.59311839049587	0.20688116802254	0.725870881126743
Ptp4a2	Ptp4a2	0.0382985387096499	6.50112723907406	1.59159295373786	0.207098892747824	0.725870881126743
Pltp	Pltp	0.13552924886579	5.41687463941674	1.59098021152407	0.207186144838359	0.725870881126743
Cdk8	Cdk8	0.0465254731083792	5.79185612543629	1.59050886005645	0.207253446982587	0.725870881126743
Ndufv3	Ndufv3	-0.0558106243152506	6.18061715619841	1.58931902773053	0.207423452735884	0.725870881126743
Ndfip2	Ndfip2	-0.0449667797958686	6.62284566901081	1.58928582448268	0.20742819761987	0.725870881126743
Ripk1	Ripk1	0.0751031097564433	4.03186062829555	1.58908759998397	0.20745653876499	0.725870881126743
Nck2	Nck2	0.0359887949614879	6.14115916082546	1.58893389742283	0.207478516273383	0.725870881126743
Cox6b1	Cox6b1	-0.0640832380132959	6.80704384048661	1.58849782300657	0.207542314818064	0.725870881126743
Pigk	Pigk	-0.0581747443254171	5.1841967498646	1.58838874279294	0.20755648863558	0.725870881126743
Pcdh11x	Pcdh11x	-0.0940372764949834	5.80378174059824	1.58770218274276	0.207654735216563	0.725870881126743
Arhgef37	Arhgef37	0.112889488700636	2.50888892981678	1.58723473144949	0.207707329895953	0.725870881126743
Fzd2	Fzd2	0.0962695178336004	4.10369436769887	1.58684989094352	0.207776946385863	0.725870881126743
Arf3	Arf3	-0.0386328147302126	9.70112282484012	1.58680321660997	0.20778346018491	0.725870881126743
Wwtr1	Wwtr1	0.127074593669082	2.93564133805842	1.58657627612372	0.207815671225701	0.725870881126743
Gpr61	Gpr61	-0.0649655620664434	3.86891731336622	1.58651229665078	0.207825137878331	0.725870881126743
Fat3	Fat3	0.120334626376698	6.71457868050567	1.58613501872205	0.207879200378714	0.725870881126743
Wrap53	Wrap53	0.0609848909418628	3.80553882572362	1.58566434333233	0.207946673516382	0.725870881126743
Chd1	Chd1	0.0606173047544549	5.66105714108737	1.58520132609158	0.208013072425117	0.725870881126743
Sike1	Sike1	0.0381732071271846	6.10064571487102	1.58427935855581	0.208145361563248	0.725870881126743
Gtpbp2	Gtpbp2	0.0535079889635853	6.08664248600934	1.58344430608435	0.208265265788903	0.725870881126743
Zfp189	Zfp189	0.0755936628898989	3.59380695537681	1.58285081375463	0.208350534237086	0.725870881126743
Aass	Aass	0.171984341398225	0.422170040865324	1.58228645864795	0.208431654634582	0.725870881126743
Lrrcc1	Lrrcc1	-0.0681315211152876	4.44185161317 28	1.5822110747963	0.208442492300075	0.725870881126743
Luzp1	Luzp1	0.0557281696132269	5.29057384681701	1.58217878405032	0.208447136185333	0.725870881126743
Chpf	Chpf	-0.0542938852197716	7.10230711788007	1.58193928057933	0.208481576801162	0.725870881126743
Ptpn5	Ptpn5	-0.085888305727112	7.25840336493193	1.58153421784358	0.208539840155807	0.725870881126743
Gpr37	Gpr37	-0.0573949366795191	4.11562981404976	1.58146482044593	0.208549824061308	0.725870881126743
Dtd2	Dtd2	-0.0723750046038446	2.94808397112356	1.58097455712945	0.208620372250174	0.725870881126743
Coq6	Coq6	-0.0468820000557 53	5.38585375022134	1.58093346247623	0.208626288964364	0.725870881126743
Elovl1	Elovl1	0.0730141079948159	4.1641766828237	1.58079739213255	0.208645872984483	0.725870881126743
Epdr1	Epdr1	-0.0632194380212991	4.47138772292171	1.58046823455774	0.208693260983441	0.725870881126743
Pcdhga5	Pcdhga5	0.101281714557447	2.69033841935222	1.58026477832863	0.208722558440234	0.725870881126743
Orai2	Orai2	0.0720259357438388	6.51235017531523	1.58015717096508	0.208738055741184	0.725870881126743
Rab8a	Rab8a	0.0414450026266451	5.82976949486788	1.57952482320525	0.20882915214923	0.725970330054083
Tceanc2	Tceanc2	0.0776545632669087	3.11819277289414	1.57915102720952	0.208883023554874	0.725970330054083
Ccdc127	Ccdc127	0.05496429382 9682	4.10198431732736	1.57806951143947	0.209038884053234	0.725979464805258
Sdf2	Sdf2	-0.0569363010201067	5.45036652204882	1.57801180912986	0.209047308914266	0.725979464805258
Vwa3a	Vwa3a	-0.145829535834147	3.70116715358519	1.57716303377647	0.209169809273953	0.725979464805258
Ppp4r2	Ppp4r2	0.0353291402131267	6.96963094589952	1.57704252789348	0.209187206850519	0.725979464805258
Rab5if	Rab5if	-0.0530276874111474	5.9435080273565	1.57621726370274	0.209306408599488	0.725979464805258
Cst3	Cst3	-0.0760089074309906	8.52165363236622	1.57615482919952	0.209315429845709	0.725979464805258
Mfsd3	Mfsd3	-0.0587349572536035	4.11144493716671	1.57553282960928	0.209405169526904	0.725979464805258
Cxxc4	Cxxc4	0.0399285293154516	6.75452458912187	1.57481429165345	0.209509227031429	0.725979464805258
Sapcd2	Sapcd2	-0.0647754588481976	3.99620790956054	1.57465194540423	0.209532722535883	0.725979464805258
Sp140	Sp140	0.183113411448645	0.440450392769743	1.5746304291291	0.209535835379921	0.725979464805258
B3gat2	B3gat2	-0.0574457373174 38	5.62697285418491	1.57462512985076	0.209536600355739	0.725979464805258
Fadd	Fadd	0.117880121128294	1.39112708612767	1.57429840646222	0.209583876911801	0.725979464805258
Rpl27a	Rpl27a	-0.0511888151125 7652	8.61578738011379	1.57386039782266	0.209647274889181	0.725997515622733
Pank3	Pank3	0.0424797643288553	8.32664470841771	1.57291316543071	0.209784455355146	0.726103884995366
Gnaz	Gnaz	-0.0434659007280477	6.8518027004295	1.57207331498095	0.209906173429775	0.726103884995366
Ptpn6	Ptpn6	0.158121850998327	1.8589595843	1.57169782970124	0.209960618919044	0.726103884995366
Pygb	Pygb	-0.0368677339040130 6	7.74426461381157	1.57136948717876	0.210008242402205	0.726103884995366
Phpt1	Phpt1	-0.0560393341558831	5.81759113951961	1.57091418338132	0.210074301787866	0.726103884995366
Bag3	Bag3	-0.129838443637498	1.97014449794588	1.57075825418983	0.210096930982473	0.726103884995366
Igf2r	Igf2r	0.0844701243870634	6.60530481200342	1.57089331978845	0.210106355441053	0.726103884995366
Rrm2b	Rrm2b	0.067851728991147	3.49443900935861	1.57043705257981	0.210143554437626	0.726103884995366
Sacm1l	Sacm1l	-0.0582688952050082	5.74881427399581	1.56931035220647	0.210307195550273	0.726305123888689
Cdh2	Cdh2	0.0466367777873549	8.79176176167555	1.56845814785744	0.21043103693 8656	0.726305123888689
Adar	Adar	0.0491720290059086	6.82154597132278	1.56841134667626	0.210437874737384	0.726305123888689
Ldha	Ldha	-0.0546895646652846	8.36745688194889	1.5689381492585	0.210652226382726	0.726305123888689
Bbs1	Bbs1	-0.0499955189291636	5.52133075126257	1.56652100407818	0.210712968408643	0.726305123888689
Usp1	Usp1	0.0524715864196036	5.06952630681224	1.56546503915947	0.210866824111505	0.726305123888689
Atxn3	Atxn3	0.079661750786801	3.79781242315846	1.56528671352982	0.210892819588792	0.726305123888689
Mrps34	Mrps34	-0.0728580887170816	5.47610712957746	1.56476810494743	0.210968441453238	0.726305123888689
Hba-a1	Hba-a1	0.158275207311658	9.01790447238978	1.56462755911953	0.210988940924413	0.726305123888689
Kmt2e	Kmt2e	0.0639520490734886	8.10203106880854	1.56452796369547	0.211003468967676	0.726305123888689
Bfsp1	Bfsp1	-0.0876721563557398	2.44791804251209	1.56422628271157	0.211047482588867	0.726305123888689
Qrsl1	Qrsl1	0.0582130973694114	3.54520179017194	1.5638640588091	0.211100343751587	0.726305123888689
Cit	Cit	-0.128649795938036	5.78331433017106	1.56382922328515	0.21110542799882	0.726305123888689
Slc44a1	Slc44a1	0.0520792245098388	5.5431467685936	1.56372961449252	0.21111996750342	0.726305123888689
Fbxw8	Fbxw8	0.0359194188239686	6.47326027996996	1.56367026729833	0.211128620744896	0.726305123888689
Bcat1	Bcat1	-0.0389937726580334	9.98489288612336	1.56349177240838	0.211154698181379	0.726305123888689
Slco1c1	Slco1c1	0.0594425927779274	6.3255065756789	1.56324039476237	0.211191394225228	0.726305123888689
Sytl2	Sytl2	-0.107820901220284	3.09527127866608	1.56275822830175	0.211261819197671	0.72634184818936
Vwf	Vwf	0.0893492949096717	5.31061968057358	1.56237030861367	0.211318498751884	0.72634184818936
Tcea3	Tcea3	0.292643221775281	-0.0673896811502176	1.5613281578364	0.211470858355367	0.726665353814354
Tex15	Tex15	-0.138523329088968	3.88546317078714	1.56047405918753	0.2115958223909	0.726894569382218
Zfp426	Zfp426	-0.0496343087211273	4.92382632789758	1.55986738851171	0.211685995281582	0.726926595730399
Armc5	Armc5	-0.0532248622388944	5.44844923683369	1.55947287621206	0.211742418013856	0.726926595730399
Usp11	Usp11	-0.0314773032224206	7.82073178642957	1.558843064236	0.211834698330435	0.726926595730399
Get3	Get3	-0.0504900354976686	6.53079219433008	1.55881923704881	0.211838190436461	0.726926595730399
Fxn1	Fxn1	0.116019767814877	5.15831539434843	1.55820077093768	0.212248808368482	0.72907458355 2328
Vav2	Vav2	0.0384559050382306	6.93382921375671	1.55537842273606	0.212343193120732	0.729074583552328
Tmsb15b2	Tmsb15b2	-0.0749105175595026	5.75195535901586	1.55521587186736	0.212381785912196	0.729074583552328
Plagl2	Plagl2	0.0796658756273431	3.46313978242432	1.55495017647944	0.21240614603935	0.729074583552328
Zfhx4	Zfhx4	0.118140934040899	5.77261062077228	1.55437543680912	0.212490668581819	0.72816426033929
Adgrf5	Adgrf5	0.0948146543988807	4.15805813535226	1.55379235032218	0.212576458306408	0.729258232705841
Snx24	Snx24	-0.0533494789072475	4.3133219588614	1.55319730239511	0.212664052486656	0.72835832634532
RGD156	RGD156348	0.0825266321427511	3.29017803581675	1.55228067143802	0.212799067599 57	0.72940924579 0238
Dpm3	Dpm3	-0.127161347413276	1.96714659116101	1.55204049102234	0.212834461786987	0.729409246790238
Sgsm1	Sgsm1	-0.08475911498938	6.5446014361394	1.55167502444255	0.212888332180827	0.729409246790238
Ppp1r13b	Ppp1r13b	-0.0354717010460382	6.87905884325778	1.55131034698932	0.212942102408817	0.729409246790238
Vstm4	Vstm4	0.173496194799232	1.89213415487002	1.55080055425478	0.213017296298817	0.729409246790238
Mterf4	Mterf4	-0.0691921191406495	3.51106273755456	1.55019082691829	0.213107270390434	0.729409246790238
Myl9	Myl9	0.123599380068157	3.81471776589686	1.5491090159105	0.213267023458635	0.729409246790238
Diablo	Diablo	-0.0482732393026771	4.91228922386631	1.54908987415161	0.213269850394687	0.729409246790238
Tgif2	Tgif2	0.0895736777673576	2.7925053041049	1.54885419671612	0.213304672199589	0.729409246790238
Rps23	Rps23	-0.0562697117282931	8.287907611189	1.54879511433265	0.213313402796673	0.729409246790238
Eloc	Eloc	-0.0439222468442976	6.55310934742272	1.54862411743507	0.213338673380743	0.729409246790238
Hook1	Hook1	-0.0637281664938517	5.98489017512469	1.54798135498495	0.213433695115588	0.728594312984219
Serpinb6b	Serpinb6b	-0.12035129090 8064	1.64655296301	1.54724110284181	0.213543191381624	0.728768708662067
Trhr	Trhr	-0.180214382325174	1.44193926545095	1.54634002637826	0.213676566452804	0.729024478980485
Ccser1	Ccser1	0.0777859784972252	3.58360730820173	1.54352559801279	0.214092306258782	0.730151179403384
Aplp1	Aplp1	-0.0422019618548153	10.3471807289596	1.54332312887101	0.214123841102587	0.730151179403384
Csrnp1	Csrnp1	-0.127710929260815	0.903920881097744	1.54078719244791	0.214500671174051	0.7302051102585
Rbp4	Rbp4	0.16406333421815	2.47202871783583	1.54071853493966	0.21451088436328	0.7302051102585
Acaa2	Acaa2	-0.101030217440175	5.34694171323197	1.54047881045743	0.214546549257902	0.7302051102585
LOC680	LOC680039	-0.0365808969102133	7.35497379394123	1.54011012612478	0.214601413853023	0.7302051102585
Spry2	Spry2	-0.0396471748442157	5.32973933788391	1.53942492198475	0.214703424953505	0.7302051102585
Tfdp1	Tfdp1	0.0344079706741677	6.72700116652945	1.53940980673034	0.214705675621501	0.7302051102585
Rpl30	Rpl30	-0.0887530472209085	7.0054032652 2393	1.5390673484462	0.214756681867843	0.7302051102585
Hivep3	Hivep3	0.0712280906609274	6.37365404909792	1.53884211858987	0.214790225795803	0.7302051102585
Acp4	Acp4	-0.209284905459628	0.996460861086522	1.53880539180361	0.214795707807073	0.7302051102585
Trmt2a	Trmt2a	0.0552223537773328	5.30531323999002	1.53820796394728	0.214884743340937	0.7302051102585
Oscar	Oscar	-0.279376527758872	-0.647010809280559	1.53802631434857	0.21491182354361	0.7302051102585
Tdp2	Tdp2	0.0813204905469374	3.73051364389427	1.53762355374913	0.214971881307247	0.7302051102585
Zfp629	Zfp629	0.0499254665220826	5.84133025938779	1.53744945330755	0.214997868524511	0.7302051102585
Hist1h2bq	Hist1h2bq	0.0980614963811849	4.14448315088757	1.53674585207765	0.215102829213742	0.7302051102585
Galnt16	Galnt16	-0.0422245286636755	6.43276018945276	1.53655907257016	0.215130707856175	0.7302051102585
Lepr	Lepr	0.178713147674257	2.1554093967362	1.53643216928501	0.215149651845854	0.7302051102585
Zdhhc3	Zdhhc3	-0.0308007658155166	6.36378628474711	1.53600373530676	0.215213622689417	0.7302051102585
Gpank1	Gpank1	-0.0625786599389932	4.19764747936691	1.53574882606392	0.215251693243444	0.7302051102585
Tubb4a	Tubb4a	-0.0385706562862536	8.86866233511963	1.53515827088434	0.21533992807406	0.7302051102585
Dcdc5	Dcdc5	0.319889362302708	-0.201783200988321	1.53386163246928	0.215533806517209	0.7302051102585
Mcrs1	Mcrs1	-0.0348190711386331	6.89541155598841	1.5338554851436	0.215534726183942	0.7302051102585
Ltv1	Ltv1	0.0594275578030667	4.64860025565464	1.53350017094299	0.215587890674227	0.7302051102585
Senp6	Senp6	0.0438809936672732	7.16217150272482	1.53229806888407	0.215767843026054	0.7302051102585
Nr4a2	Nr4a2	0.129132741305656	6.09400841985276	1.53208274302439	0.215800121100679	0.7302051102585
Lrrc8d	Lrrc8d	0.0327643693689208	6.30207426149782	1.5320260515006	0.215808625051009	0.7302051102585
Galnt1	Galnt1	-0.0404081359532761	5.64882855205907	1.53172682579034	0.215853463880487	0.7302051102585
Cln6	Cln6	0.0783022306204251	3.15575186713263	1.53094530878521	0.21597062618014	0.7302051102585
Lgi1	Lgi1	-0.0449697893404926	5.24674795030319	1.53024167633438	0.21607617705787	0.7302051102585
Sun1	Sun1	0.0596290648886313	5.56008060565259	1.52922560195322	0.216228696279205	0.7302051102585
Ap5m1	Ap5m1	0.104905021897721	4.13164109330943	1.52810828892284	0.216396579005454	0.7302051102585
Ldhb	Ldhb	-0.0476852354460903	7.82134691248171	1.52778900440482	0.216444579279374	0.7302051102585
Crym	Crym	-0.0891535169782132	7.22871835450863	1.52759976416291	0.21647303507279	0.7302051102585
Cd53	Cd53	0.185050906076135	0.835106422080294	1.52750650436285	0.216487060058735	0.7302051102585
Anpep	Anpep	0.145369617161144	2.82123747270827	1.52712673987739	0.216544182589308	0.7302051102585
Lnc134	Lnc134	0.0805997606217818	2.77921598204353	1.52701164607322	0.216561498019644	0.7302051102585
S100a10	S100a10	-0.0789392750112376	6.4861438307454	1.52695940098124	0.216569358650661	0.7302051102585
Cfap298	Cfap298	-0.0492157694459651	4.80978976327685	1.5267365890051	0.216602886049906	0.7302051102585
Ddx49	Ddx49	-0.0382449669960006	5.75869011724816	1.52670885593226	0.216607064538532	0.7302051102585
Nr1h2	Nr1h2	0.0413616950332159	5.60587580232849	1.52669800158402	0.21660868840487	0.7302051102585
Snca	Snca	-0.0709418131665098	7.20899905115754	1.52663938906122	0.216617514046539	0.7302051102585
Vsx2	Vsx2	0.245663125114335	0.291468577348393	1.52659785093382	0.216623795740322	0.7302051102585
Arfip1	Arfip1	0.0669560545711939	3.74410973010234	1.52608112821848	0.216701550019834	0.7302051102585
Eva1b	Eva1b	0.169620358967791	2.59634515596525	1.52575589416373	0.216750524071649	0.7302051102585
Cep112	Cep112	0.12886408289409	2.12742835179569	1.52563791188826	0.216768298773032	0.7302051102585
Ccrn2	Ccrn2	0.0654041155880338	6.01617049280616	1.52560530055415	0.216773240335304	0.7302051102585

**Gapdh**	Gapdh	-0.0506973771627336	10.2658831559493	1.52511438330122	0.216847172208457	0.730258995315768
**Arl6ip1**	Arl6ip1	-0.0235829024770445	7.87817598069505	1.52461744918141	0.216922070624174	0.730312108790562
**Leprot**	Leprot	0.0489990305237668	5.6814534411758	1.52340180776032	0.217105423293957	0.73073222725619
**Neurod(**	Neurod6	0.0718064256300652	7.85619661521089	1.52224418271639	0.217280197153299	0.731123252385282
**Dcx**	Dcx	0.0532150541187383	11.1959421551708	1.52152107833894	0.217389453800875	0.731139973612275
**Dus1l**	Dus1l	-0.0589319898281274	4.62381019914473	1.52068378649784	0.217516045290334	0.731139973612275
**Rabl3**	Rabl3	-0.0801122473859136	3.06077782554311	1.52040326407336	0.217558477572606	0.731139973612275
**Atf1**	Atf1	0.0767495965708801	3.81216224067687	1.52007296978928	0.217608451076867	0.731139973612275
**Igsf21**	Igsf21	-0.0639140041291796	6.75493205362415	1.52002066932448	0.217616365388717	0.731139973612275
**Prrc2c**	Prrc2c	0.100546550526588	7.54394092471371	1.5198869012859	0.217636760599182	0.731139973612275
**Casc3**	Casc3	0.037090193698885	6.21373774222307	1.51901272131401	0.217768859093882	0.73131534366701
**Slain1**	Slain1	0.0446369698438917	5.95448353850408	1.51844714359977	0.217854628030676	0.73131534366701
**Sh3gl1**	Sh3gl1	0.0532950300542795	4.74447324573224	1.51803056890352	0.217917770317055	0.73131534366701
**Rbm4b**	Rbm4b	0.0416805773493745	5.4520282007456	1.51774286157263	0.217961385370951	0.73131534366701
**Atp6v0a**	Atp6v0a4	0.0808028201570316	3.32454291190414	1.51737607285415	0.218017003877984	0.73131534366701
**Nos1**	Nos1	0.0560601915065978	5.96414218913084	1.51722022597518	0.218040641054843	0.73131534366701
**Dcun1d3**	Dcun1d3	0.0665061187587373	3.45009713769385	1.51551323952534	0.218299738546426	0.73198758415312
**Pex5l**	Pex5l	-0.0807412112732862	3.51168642848453	1.51389496367099	0.218545710136429	0.732194168195473
**Slc52a2**	Slc52a2	0.0577032634716769	3.93900661281079	1.51298665592266	0.218683814222034	0.732194168195473
**Tmem43**	Tmem43	0.0394066758820633	5.2505698934801	1.5128272427791	0.218708180575175	0.732194168195473
**Asb2**	Asb2	-0.190661056736486	3.27011221607251	1.51270317248694	0.218727069155448	0.732194168195473
**Rps8**	Rps8	-0.0502773334188935	8.75493414763757	1.51228013426163	0.218791487535232	0.732194168195473
**Zfp292**	Zfp292	0.0675666737829641	6.74634633680947	1.51223292772471	0.218798677340687	0.732194168195473
**Lhx2**	Lhx2	0.110529247802399	6.55465546625447	1.5120776849832	0.218822322096883	0.732194168195473
**Stom**	Stom	0.0666532921972526	3.93310066157468	1.51128917696047	0.218942474755753	0.732194168195473
**Eola2**	Eola2	-0.0708306398018941	3.35899806118276	1.51121269954169	0.218954122406068	0.732194168195473
**Rab23**	Rab23	0.0569531732289566	5.34712247121745	1.51108107808623	0.218974197538979	0.732194168195473
**Ltbp2**	Ltbp2	0.267188156756428	-0.0182107742820801	1.51037251174669	0.219082847321923	0.732194168195473
**Rpp38**	Rpp38	0.0983312814366122	2.78416308946929	1.5104920340004	0.219065546896369	0.732194168195473
**Gtpbp8**	Gtpbp8	-0.0775477002570396	3.10532945218483	1.50983472478989	0.219149047060081	0.732277091635983
**Zbtb1**	Zbtb1	0.0816859932856787	3.97833572056452	1.50910185481125	0.219276217061446	0.732505852818116
**Krt10**	Krt10	-0.102637198184724	2.39719915302313	1.5081708672239	0.219418408088268	0.732784656776586
**Sema4c**	Sema4c	0.0606209405552312	6.47080039281907	1.50767182276547	0.219494688549425	0.732843254350595
**Cerk**	Cerk	-0.0325272090417918	6.76414782823556	1.50650558357419	0.219673078585584	0.733242643539249
**Nckap1l**	Nckap1l	0.147921650345565	2.06910280945313	1.50606281828176	0.219740846714529	0.733258249051872
**Vipr2**	Vipr2	-0.178149152925571	1.4394977542024	1.50487903856667	0.219922165634378	0.733258249051872
**Fchsd2**	Fchsd2	0.0361042079502132	7.23022963847843	1.50470884750597	0.219948247967724	0.733258249051872
**Cldn19**	Cldn19	0.3890068446408	-0.258590252886156	1.50440243450988	0.219995216070282	0.733258249051872
**Hcfc1**	Hcfc1	0.057692946575461	8.05612990850132	1.50420845614128	0.220024855975378	0.733258249051872
**Med15**	Med15	0.0439150328203256	6.08308072549374	1.50412719947375	0.220037415315216	0.733258249051872
**Jag1**	Jag1	0.0789423661727746	3.93651729552292	1.50261137301312	0.220269986328586	0.733258249051872
**Crkl**	Crkl	0.0358110663094041	6.06609541417272	1.50249052450604	0.220288549835872	0.733258249051872
**Lifr**	Lifr	0.0834580055775926	4.24688687723983	1.50244238424817	0.220295943331903	0.733258249051872
**Chrnb1**	Chrnb1	0.15221171294968	0.679629579069109	1.50145972716688	0.220446895603172	0.733258249051872
**Pias2**	Pias2	0.0611516922838037	5.50484528685091	1.50136389809798	0.220461623135586	0.733258249051872
**Tsga10**	Tsga10	0.0813887530271384	5.06627107929141	1.50086873328964	0.220537741492054	0.733258249051872
**Tchp**	Tchp	-0.0476362099028361	5.1796349028578	1.50055070418653	0.220586646531047	0.733258249051872
**Pex14**	Pex14	-0.0495400775564476	5.31173494688137	1.50051495491801	0.220592144697847	0.733258249051872
**Pitpnm1**	Pitpnm1	-0.0356052704020997	8.11737001355311	1.50037206153187	0.220614123053738	0.733258249051872
**Ccng1**	Ccng1	-0.0419059940341743	7.48887202855791	1.49986821444618	0.220676252710905	0.733258249051872
**Ago4**	Ago4	0.0943886614299164	4.76782616494893	1.49984560786537	0.220695119198168	0.733258249051872
**Dusp11**	Dusp11	0.0708289498801746	4.38761050519157	1.49939759963264	0.220764074319354	0.733258249051872
**Usp31**	Usp31	0.0627843871558029	5.43473388170213	1.49919882152265	0.220794663073245	0.733258249051872
**RGD131(**	RGD131058	0.128861015855071	3.8117111640786	1.49765699179585	0.221032224228335	0.733758779090851
**Nme1**	Nme1	-0.0604335718052696	6.97080734184676	1.49737759180866	0.221075305084258	0.733758779090851
**Tfdp2**	Tfdp2	0.0707138616003908	3.64160690645014	1.49652140052065	0.221207378005756	0.733758779090851
**Grin1**	Grin1	-0.0481789908956322	8.17940948925442	1.49627540341267	0.221245342504141	0.733758779090851
**Coq9**	Coq9	0.0465569600327137	4.90752690387561	1.49621809908988	0.221254187344249	0.733758779090851
**Tent4b**	Tent4b	-0.0447602501389857	6.56241682426123	1.49582782482886	0.221314436864086	0.733758779090851
**Polm**	Polm	-0.111953472021905	2.7449091150633	1.49523615342474	0.221405814970267	0.733758779090851
**Tert**	Tert	0.119487252362253	2.59196451870895	1.49511254032775	0.221424911552646	0.733758779090851
**Micu1**	Micu1	-0.0446542880992594	6.73570335489648	1.49479252173553	0.221474357782812	0.733758779090851
**Slc39a14**	Slc39a14	0.0681439540335257	4.39592627957633	1.49414616644599	0.221574271403466	0.733894925484746
**Uckl1**	Uckl1	-0.062058564634002	4.87884574537227	1.49350409380938	0.221673574856092	0.734028979161215
**Sds**	Sds	0.182580594986028	0.36936713236405	1.49163546384798	0.221962881685448	0.734791954076442
**Map2k1**	Map2k1	-0.0647612414667901	6.26793626193923	1.49066187292436	0.222113794922681	0.735039894686008
**Tex264**	Tex264	-0.0565495967482087	5.62432756242419	1.49039230340129	0.222155601737037	0.735039894686008
**Capn7**	Capn7	0.0345846098458721	6.18416818895044	1.48916665423499	0.222345803162641	0.735474174459246
**Zpr1**	Zpr1	0.0359130807860304	6.02060856409901	1.48880428906757	0.222432138593206	0.735568054658743
**Ago1**	Ago1	0.0450344633215035	7.57696310346052	1.48807652141106	0.222515138374381	0.735664245229771
**Pxdc1**	Pxdc1	-0.0832452671946746	2.87444692922973	1.48711121758961	0.222665186665731	0.735840887409346
**Hdx**	Hdx	0.134540497994193	2.23624876967868	1.48689126494308	0.222730528237315	0.735840887409346
**Snhg11**	Snhg11	-0.206219471473709	6.30861186598032	1.48655625893603	0.222751545383113	0.735840887409346
**Snapc5**	Snapc5	-0.0509966670411331	4.57087922730501	1.48580446815556	0.2228997057948	0.735872913607294
**LOC500(**	LOC500331	0.166360367440823	0.687547656314709	1.48516324574331	0.222968428663127	0.735872913607294
**Pms2**	Pms2	-0.0490850777578325	4.98303360274203	1.48451217378338	0.223069883126587	0.735872913607294
**Slc29a4**	Slc29a4	0.0448789621880408	7.65790726185522	1.48433808059598	0.223097020878859	0.735872913607294
**M6pr**	M6pr	0.0438806521826446	5.6506038009438	1.48405024841207	0.223140337563718	0.735872913607294
**RGD131(**	RGD131194	0.0643096722742889	4.79457203562793	1.48402759894593	0.223145430185616	0.735872913607294
**Lin7a**	Lin7a	-0.11344388504564	2.46257529965381	1.48348736396619	0.223229687988618	0.736056241288051
**Pex12**	Pex12	0.0620595605329448	3.90236245664806	1.48303282285633	0.22330061125751	0.736095568470265
**Ncoa6**	Ncoa6	0.0595754305375284	7.1582917135075	1.48242258717265	0.223395870415879	0.736111556577645
**Srd5a1**	Srd5a1	-0.0526657792232921	4.45074001713161	1.4819823612638	0.223464620984042	0.736111556577645
**Fam110a**	Fam110a	0.0493638296261147	5.34181195227464	1.48186919823257	0.223482453820319	0.736111556577645
**Phf8**	Phf8	0.0614572700734589	5.6093372432285	1.48130740057871	0.223570079345522	0.736205827382527
**Rad1**	Rad1	0.0821088832907847	3.76304600958942	1.47966964283762	0.223826216886903	0.736696846984312
**Nmi**	Nmi	-0.149327670083604	1.28248655721081	1.47959895310434	0.223837280349245	0.736696846984312
**Ipo4**	Ipo4	-0.0296241910481204	7.69474880213254	1.47901010129125	0.223929465378634	0.736805891226058
**Bcar3**	Bcar3	-0.0755816757081524	3.31334625849622	1.47824056684703	0.224050004953716	0.737008149698791
**Cst6**	Cst6	-0.0614928812346791	5.44724995409836	1.47710876459638	0.224227431357567	0.737397380350122
**Uba3**	Uba3	0.0365248828209018	6.5210900564922	1.47608639559741	0.224387847322782	0.737730479854142
**Dhx33**	Dhx33	0.0451418658309091	4.58893824541322	1.47569480337587	0.224449320984628	0.737738212009274
**Rdh12**	Rdh12	-0.12221828232551	1.4863014974787	1.4744519285741	0.224643819270115	0.737828966504749
**Zfp74**	Zfp74	0.0560606284318174	4.66997137571895	1.47443682538915	0.224646865217983	0.737828966504749
**Dync2li1**	Dync2li1	0.0557549580005593	3.93427309489879	1.47436859188847	0.224657692774829	0.737828966504749
**Ttpal**	Ttpal	-0.0346193055664854	5.92888665444212	1.47279151014563	0.224905772259394	0.738460347494857
**Cnksr2**	Cnksr2	0.0695323990619381	6.45934579262081	1.47078054657312	0.225222581415549	0.739153313284654
**Sdc3**	Sdc3	-0.0407285483685852	10.4902165632218	1.47069985552967	0.225235304729363	0.739153313284654
**Proca1**	Proca1	0.0874171885267762	2.29768870558526	1.46813673157649	0.225639905715686	0.740240656879124
**Mastl**	Mastl	0.117604873418323	1.27215828125519	1.46764952043538	0.22568526764913	0.740240656879124
**Marchf8**	Marchf8	0.0343408672115898	6.49628571080244	1.46742938152948	0.225751717735063	0.740263911216919
**Frmd8**	Frmd8	0.0405412582087444	5.46601304164683	1.46581856520024	0.226006659047726	0.740904947289747
**Taf1b**	Taf1b	0.0584988178874564	3.70208418686678	1.46479787309112	0.22616826914099	0.741170027379658
**Snx7**	Snx7	0.0826222021002918	4.62990454644855	1.46455818157558	0.226206256653181	0.741170027379658
**Jund**	Jund	-0.0582558131051527	8.20721071568965	1.46417516605075	0.226266874777175	0.741174335598533
**Rtn4rl2**	Rtn4rl2	0.0672431761067455	5.69781972582838	1.46348980877707	0.226375670942653	0.741188376808866
**Tspyl1**	Tspyl1	-0.0296818634829246	7.48276680082171	1.46339904834542	0.22639007005038	0.741188376808866
**Sae1**	Sae1	-0.0356513133838961	7.42873937227423	1.46174101083041	0.226653310778831	0.741655276282733
**Svop**	Svop	-0.0455453852080467	7.32908510442408	1.46122651125674	0.226735070789982	0.741655276282733
**Mmp28**	Mmp28	0.0895469882209688	1.9471694846254	1.46087187115296	0.226791447881944	0.741655276282733
**Birc6**	Birc6	0.0393539768975816	7.76296091079724	1.46063253086695	0.226829505297082	0.741655276282733
**Parp1**	Parp1	0.0294997076297461	6.77500443578988	1.46013933319438	0.226907952685878	0.741655276282733
**Kctd8**	Kctd8	-0.10566424030899	4.27452989173724	1.46000418698799	0.226929454557801	0.741655276282733
**Mrpl30**	Mrpl30	-0.0570817690654977	4.32829335067783	1.45963797323088	0.226987721772623	0.741655276282733
**Ppm1f**	Ppm1f	0.050191520436657	4.43381074184804	1.45915617233675	0.22706443027575	0.741655276282733
**St3gal5**	St3gal5	-0.0380195342240754	6.96417492327373	1.4586643098685	0.227142762621952	0.741655276282733
**Mbnl1**	Mbnl1	0.0692594020143381	3.44685754082794	1.45859080233463	0.227154471971269	0.741655276282733
**Klf12**	Klf12	0.0810320079272153	4.32757893867034	1.45838950963687	0.227186540513811	0.741655276282733
**Lhfpl4**	Lhfpl4	0.0451318860984194	5.63247807610746	1.4562782930434	0.227523053185797	0.742090518436904
**Cartpt**	Cartpt	-0.25723868826553	2.59752683400414	1.45626012055976	0.227526113334882	0.742090518436904
**Tubb4b**	Tubb4b	-0.0449657503490812	8.56180291629241	1.45609809028998	0.227551978803613	0.742090518436904
**Haus7**	Haus7	0.0782464266130336	4.15700285688644	1.45599466124382	0.227568488783292	0.742090518436904
**Nudt22**	Nudt22	0.0727483951088557	2.91748078991741	1.45540945596626	0.227662096740163	0.742090518436904
**Apeh**	Apeh	-0.0323883875653149	5.81396340630704	1.45531688603469	0.227676725541113	0.742090518436904
**Etfa**	Etfa	-0.0588963837029491	4.91470672954392	1.45494028744075	0.227736880395871	0.74209276904199
**Rtcb**	Rtcb	0.0415098873964401	6.49234453674554	1.45390522095812	0.227902358066966	0.742433545429717
**Grip1**	Grip1	0.02942842908294	6.45885460912829	1.45281905629348	0.228076149215967	0.742433545429717
**Rangap1**	Rangap1	-0.0282784641867546	8.12771750899547	1.45273072600099	0.228090289382117	0.742433545429717
**Plpp5**	Plpp5	0.0688512158133645	4.2798125911348	1.45223710731707	0.228153313808269	0.742433545429717
**Fkbp3**	Fkbp3	-0.0449132554462356	6.74155969649453	1.45172850737872	0.22825080128818	0.742433545429717
**Timm29**	Timm29	-0.0357465164066174	6.54531112605137	1.45171409896114	0.228252109876784	0.742433545429717
**Msl1**	Msl1	0.0260412014343472	7.91012006335022	1.45167974370288	0.228258614577385	0.742433545429717
**Sgpp1**	Sgpp1	-0.0442953053881706	5.35026179717431	1.45049973840517	0.228447782187612	0.742433545429717
**Ercc8**	Ercc8	0.109162378276961	1.54138030230757	1.45039979664052	0.228463812595312	0.742433545429717
**Adi1**	Adi1	0.0847075509681026	3.26207878165414	1.45030587955179	0.228478877893633	0.742433545429717
**Coa7**	Coa7	-0.0589976969418816	3.52258002967892	1.45007640253428	0.228515693472586	0.742433545429717
**Arhgap2**	Arhgap20	0.04444239766627	6.22570614745444	1.44982919544443	0.228555522000124	0.742433545429717
**Ind1**	Ind1	-0.168742785126893	3.04590825065388	1.44905009192334	0.228680434457724	0.742521463068937
**Timm8b**	Timm8b	-0.0676094213388942	5.81636059185323	1.44842116877779	0.228781458358778	0.742521463068937
**Myrip**	Myrip	-0.056727717318973	4.37517856410751	1.44816325538914	0.228822902505153	0.742521463068937
**Zfp563**	Zfp563	0.0586476207891189	4.21322060948908	1.44801267833301	0.228847102935523	0.742521463068937
**Glrx2**	Glrx2	-0.0382455382550956	5.54077631939682	1.4477051981203	0.228896530082258	0.742521463068937
**Taf5**	Taf5	0.0567898223419863	3.81106892272575	1.44735660659556	0.228952581374333	0.742521463068937
**Srek1ip1**	Srek1ip1	-0.0920988607271803	3.12908741066615	1.44636260082696	0.229112501904547	0.742521463068937
**Sdr42e1**	Sdr42e1	-0.144015822314363	1.67550393515488	1.44624455385812	0.229131502812075	0.742521463068937
**Exoc1**	Exoc1	0.0322772361815219	5.66622929908843	1.44602991511579	0.229166056047318	0.742521463068937
**Cavin4**	Cavin4	-0.10612238261826	2.49431352044738	1.44585759795683	0.229177899309067	0.742521463068937
**Inha**	Inha	-0.0602138153965224	4.4920433942987	1.4452385496635	0.229293507301527	0.742644582401205
**Mepe**	Mepe	0.169492355549489	0.0603585424025103	1.4447967640205	0.229364210760548	0.742644582401205
**Lrrc6**	Lrrc6	0.203342556742448	-0.0874968740379379	1.44461331004676	0.229394263090025	0.742644582401205
**Bin1**	Bin1	-0.0324275447719426	7.47539629055843	1.44361048251136	0.229507586172617	0.742818716647403
**Sumo3**	Sumo3	-0.034736082756642	7.17237545824848	1.44301043517779	0.229652807207589	0.743090532967542
**Rps17**	Rps17	-0.0625028746981508	7.74190763418518	1.44265192807669	0.22971068250828	0.743090532967542
**Enho**	Enho	-0.0598071107934924	6.23549622269726	1.44180330177296	0.229847749826681	0.743174162450374
**Ghrl**	Ghrl	0.0833699748922862	2.85433054511808	1.44173273938332	0.229859151266636	0.743174162450374
**Adamts2**	Adamts2	0.11416310435453	3.24688221890231	1.44138576356757	0.229915225379373	0.743174162450374
**Pttg1**	Pttg1	-0.110198893795186	2.20960389457034	1.43981075746315	0.230153782389518	0.743752588092227
**Syt12**	Syt12	-0.13029246209875	2.4360342158468	1.43875260536428	0.230341292972686	0.744165798140946
**Otud4**	Otud4	0.0645829101551679	5.90366813431837	1.43829332977115	0.230415714916242	0.744213532231415
**Cybrd1**	Cybrd1	0.162380607275016	1.77479135540623	1.43742065008047	0.230557191774925	0.744477764436393
**P3h2**	P3h2	0.25583248235825	-0.484953779636211	1.43582354922496	0.230816378437959	0.74449695766701
**Gas8**	Gas8	-0.0806237740161136	3.62031905066895	1.435797181433	0.230820660520675	0.74449695766701
**Stx1a**	Stx1a	-0.0438368328479267	7.74629975161112	1.43554705623436	0.23086128496438	0.74449695766701
**Get1**	Get1	0.0344955696498375	7.00530971241476	1.43553016780043	0.230864028225915	0.74449695766701
**Cd300a**	Cd300a	0.265591012857985	-0.266207970368548	1.43541520555294	0.230882703162605	0.74449695766701
**Rhoap**	Rhoap	-0.0500568203948446	6.03522878277694	1.43517854480154	0.230921153015254	0.74449695766701
**Otud5**	Otud5	-0.0262854938034926	7.24841797250184	1.43455670649625	0.231022218782935	0.74463038588341
**Adcy4**	Adcy4	0.123062147756535	1.70067199409544	1.43403469802644	0.231107208317972	0.744711941679582
**Ttc17**	Ttc17	0.0375060015124423	5.69868768218695	1.43334947078674	0.231218580009256	0.744759781100126
**Tti9**	Tti9	-0.168871606758295	1.66948357688868	1.43320903414744	0.231241425600055	0.744759781100126
**Ptpre**	Ptpre	0.0553094008859824	4.22577764384678	1.43139668995489	0.231536632157096	0.745428639999608
**Aldh4a1**	Aldh4a1	-0.0591687507378398	4.58821899049378	1.43120060575689	0.231568598913078	0.745428639999608
**Fbxl7**	Fbxl7	0.074116741820379	3.20626624419221	1.43035916829504	0.231705820816533	0.745643298242724
**Fhdc1**	Fhdc1	0.0637755742673409	4.14038220467945	1.42985207653668	0.231788581184091	0.745643298242724
**Nudt14**	Nudt14	0.0869150372581983	3.72247989281449	1.4296928802935	0.231814567114172	0.745643298242724
**Mob4**	Mob4	-0.0338412061885826	6.61271305309765	1.42883981590908	0.231953874463956	0.74581141188806
**Ciapin1**	Ciapin1	0.0405918200621025	6.14238323977259	1.42813978977803	0.232068265893367	0.74581141188806
**Hsp90aa**	Hsp90aa1	-0.0459242387259034	10.4778710652719	1.42700891556754	0.232253205952901	0.74581141188806
**Aarsd1**	Aarsd1	-0.0360977841044027	6.28699983141287	1.42691785703751	0.232268105158868	0.74581141188806
**Mrps18a**	Mrps18a	-0.0545539981362994	5.60116452186876	1.42672228996808	0.232300108192415	0.74581141188806
**Abcg1**	Abcg1	-0.0438525612073469	5.12232432379043	1.42664060944207	0.232313476152622	0.74581141188806
**Edrf1**	Edrf1	0.0623574125532881	5.11101438648885	1.42631979427561	0.232365990242673	0.74581141188806
**Slc35d1**	Slc35d1	0.070138838064887	3.50347976277022	1.42517482941863	0.232553857003585	0.74581141188806
**Man1c1**	Man1c1	0.08089121994745	3.84319382949991	1.42485300998202	0.232606270133199	0.74581141188806
**Zbtb44**	Zbtb44	0.0586086628668142	4.37853071894826	1.42468617325122	0.232633619335189	0.74581141188806
**Nup155**	Nup155	0.046145491392338	5.52884940354034	1.42455034927664	0.232655887545942	0.74581141188806
**Sf3b3**	Sf3b3	0.0353810851513448	7.36494218032115	1.42348230455212	0.232831082189687	0.74581141188806
**Flot1**	Flot1	-0.0505128198873454	6.54068677247515	1.42317434385261	0.232881627515507	0.74581141188806
**Uqcrc2**	Uqcrc2	-0.0362162598062854	7.68887989348544	1.42316446186387	0.232883249657295	0.74581141188806
**Npepo**	Npepo	0.0822681336528475	2.67285514207494	1.42274380307954	0.232952297600216	0.74581141188806
**Gjc2**	Gjc2	0.231929980679545	0.398675956412443	1.42257521414119	0.232980000139554	0.74581141188806
**Tmem21**	Tmem215	-0.175478124689775	1.21523913872813	1.42231361733549	0.233022968122898	0.74581141188806
**Sec23ip**	Sec23ip	-0.0567537757585217	6.37186413500663	1.42228760154619	0.233027187599367	0.74581141188806
**Gal**	Gal	-0.18539940105945	0.693520870599435	1.42182394648036	0.233103423618084	0.74581141188806
**Tubgcp2**	Tubgcp2	0.0320955634407479	6.36570904110199	1.42169849958431	0.233124040601739	0.74581141188806
**Tdg**	Tdg	-0.0300633702358257	7.11754808451032	1.42135255777119	0.233180906963953	0.74581141188806
**Tlk2**	Tlk2	0.0316097933208484	6.04912707947154	1.42044039205427	0.233330930313769	0.74581141188806
**Git2**	Git2	0.0752752317080616	6.2483292985709	1.42021498278625	0.233368021209396	0.74581141188806
**Cal1**	Cal1	-0.0818477803852556	6.99835834138967	1.42014013687614	0.233380338646074	0.74581141188806
**Septin2**	Septin2	0.0335602492563094	7.19008242216822	1.42005687016254	0.23339404275305	0.74581141188806
**Ppp3cb**	Ppp3cb	-0.0329640356655047	8.8807274086676	1.41982561615739	0.23343046173884	0.74581141188806
**Unc5a**	Unc5a	0.0564562834001889	6.47726217681228	1.41950486657957	0.233484916767548	0.74581141188806
**Sall1**	Sall1	0.0706869974710173	5.14572118630498	1.41856082764907	0.233640429089432	0.74581141188806
**Fam210b**	Fam210b	-0.076539696454761	4.40023741708194	1.41817494852927	0.233704031281085	0.74581141188806
**Eef1akmt**	Eef1akmt	0.037382182969199	5.21894764489975	1.41792039779287	0.23374599885715	0.74581141188806
**Gpr157**	Gpr157	-0.178471793483123	0.185018913030266	1.41750168424736	0.233815051638773	0.74581141188806
**St6galna**	St6galnac2	0.263516862410289	0.215827436326726	1.41713280287279	0.233875896803994	0.74581141188806
**Radx**	Radx	-0.170079156577008	0.51847692536752	1.416855526701	0.233921602088684	0.74581141188806
**Arhgef40**	Arhgef40	-0.0859706014440866	6.2754444922001	1.41681973288846	0.233927569313072	0.74581141188806
**Pld2**	Pld2	-0.0757216972728815	5.29219043449419	1.41662958882044	0.233958953765318	0.74581141188806
**Hnrnpm**	Hnrnpm	0.0393834993375876	8.02553301640183	1.41610786494236	0.234045093257768	0.745895435141041
**Cpne2**	Cpne2	-0.0406319623102352	5.7221324910378	1.41558377455491	0.234131601982932	0.745895523278381
**LOC100(**	LOC100302	0.169370179885916	0.297776208049783	1.41538391866723	0.23416468419343	0.745895523278381
**Isca2**	Isca2	-0.0541945623106856	4.09631485319071	1.41421759952672	0.234357507808085	0.746067588194594
**Fam217b**	Fam217b	0.0452050707366432	4.7236465354221	1.41408461804385	0.234379505158253	0.746067588194594
**Scrn1**	Scrn1	-0.0470332561385884	7.22023306914146	1.41385102418432	0.234418151865415	0.746067588194594
**Arrdc1**	Arrdc1	0.0536081224764866	4.60589386682391	1.41361092079733	0.23445788357065	0.746067588194594
**Mrps2**	Mrps2	0.0339872908810265	6.35693639649138	1.41282526598759	0.234587948890651	0.746080644257453
**Spindoc**	Spindoc	0.0441478891404093	5.32577668382867	1.41243785123694	0.234652117314146	0.746080644257453
**Armt1**	Armt1	0.0587908476755995	3.71237907011377	1.41233269715278	0.234669537952111	0.746080644257453
**Pir**	Pir	-0.110050452961244	1.7341155684152	1.41201967737574	0.234721404487719	0.746080644257453
**Txn1**	Txn1	-0.0620690269339007	6.88919693086268	1.41148761542323	0.234809597545703	0.746080644257453
**Zmynd12**	Zmynd12	-0.228977839302585	-0.284805109135404	1.41129582585219	0.234841396205794	0.746080644257453
**Timm10**	Timm10	-0.0718380404973994	4.67442206633017	1.41105966852464	0.234880561885331	0.746080644257453
**Usp20**	Usp20	0.0305296119841965	6.65758960044345	1.4104992988633	0.234973507888414	0.746185914462648
**Trim26**	Trim26	-0.04082118135934	5.10805773104129	1.40985112593496	0.235081107088984	0.746221556418017
**Parp16**	Parp16	0.0631182692661815	3.36799741160758	1.40971165708041	0.23510434706093	0.746221556418017
**Krt19**	Krt19	0.192984821474416	0.875077058071414	1.40762118970984	0.235451690984183	0.747123964498894
**Arid3b**	Arid3b	0.0943868907217179	3.1206223037083	1.40687489859965	0.235575854719949	0.747327894569234
**Nt5c1a**	Nt5c1a	0.144722756869954	0.851952072873284	1.40641115897813	0.235653076352722	0.74734968258465
**Clip2**	Clip2	0.0341909734791604	8.95498364187774	1.40546982098368	0.235809886900277	0.74734968258465
**Tec**	Tec	0.102275371166107	3.0752585343032	1.40511180113806	0.235869543391113	0.74734968258465
**Pigq**	Pigq	-0.0332142914115597	6.06372943301505	1.40461464525252	0.235952454130685	0.74734968258465
**Atp1a1**	Atp1a1	-0.0407116830477994	8.26541926187249	1.40436932096734	0.23599337166146	0.74734968258465
**Msantd4**	Msantd4	0.0370712245973895	6.03109074959453	1.40423429448313	0.23599921441455	0.74734968258465
**Donson**	Donson	-0.0810468475792246	5.07877337854998	1.40386540395947	0.23607744671856	0.74734968258465
**Ube2ql1**	Ube2ql1	0.0496234706887171	8.02768390759442	1.40315944790001	0.236195290410654	0.74734968258465
**Clu**	Clu	0.106917555325944	7.26001388434174	1.40261347052997	0.23628648030863	0.74734968258465
**Was**	Was	0.209107066957386	-0.319909018260527	1.40206516545705	0.23637810033006	0.74734968258465
**Ddx19a**	Ddx19a	0.042198218509946	5.42391635807852	1.40185137565445	0.236397118857007	0.74734968258465
**Nrsn2**	Nrsn2	-0.0889762809117515	5.48557081224757	1.4016254595646	0.236451605257572	0.74734968258465
**Piwil2**	Piwil2	-0.11801524465205	1.32705134970482	1.4015256418335	0.236468295445963	0.74734968258465
**Popdc3**	Popdc3	0.128416701911976	1.08371675820824	1.40149962271601	0.236472662971741	0.74734968258465
**Pdgfc**	Pdgfc	0.111423986777448	2.27149032632651	1.4014673283333	0.236478046511517	0.74734968258465
**Actr8**	Actr8	0.0383755596692624	5.43252380139822	1.40103952107751	0.236549598475123	0.747386513085367
**Frg1**	Frg1	0.0531052305448868	4.2720161859673	1.40007235808053	0.236711455915076	0.747708565937317
**Ccn2**	Ccn2	-0.0782398518543477	3.5751634388581	1.3984439116355	0.236984284349051	0.748281401047701
**Apls1**	Apls1	-0.0585212087351884	4.02145136291376	1.39827413075742	0.237012751217481	0.748281401047701
**Zbtb43**	Zbtb43	0.0611324185446457	5.17243465361762	1.39658759520932	0.237295754970391	0.748885361691264
**Anks1b**	Anks1b	-0.0379244276859541	7.01058095122906	1.39621379013315	0.237358525801553	0.748994043677435
**Ptpn22**	Ptpn22	-0.157750199545855	-0.101255794285398	1.39583852454327	0.237421581785616	0.749003559023801
**Ankmy2**	Ankmy2	-0.0318416136933908	7.01420038025741	1.39540162420857	0.237495008449102	0.749037907239168
**Coa5**	Coa5	0.0344409597855727	7.35310359705415	1.39487737145392	0.237583152150152	0.749037907239168
**Zbtb33**	Zbtb33	0.0537095519975768	4.49514997969061	1.39470245479234	0.237612570077152	0.749037907239168
**Rnf144a**	Rnf144a	0.0476478128048055	4.6655501705684	1.39239369675148	0.238001277187569	0.750048863026368
**Fkbp1a**	Fkbp1a	-0.0391127588212874	8.56490573669618	1.39208387403535	0.238053498243521	0.750048863026368
**Ice2**	Ice2	0.0749409592976771	3.12113278149305	1.39151282930602	0.238149783382361	0.750162799106721
**Commd1**	Commd1	-0.0622387186873016	4.99211400904946	1.39024100043727	0.238364404549412	0.750649337909702
**Ankrd54**	Ankrd54	0.0479878026641605	4.56224546623205	1.38881297151809	0.23872234068555	0.7513292404621
**Klhl1**	Klhl1	0.0760598380482887	4.5237521071729	1.38785522611056	0.238767634726314	0.7513292404621
**Acot7**	Acot7	-0.0405982937736997	8.34882153567259	1.38758121891394	0.238813998866234	0.7513292404621
**Vat1**	Vat1	-0.0444514532287902	8.20023965217563	1.38686337832615	0.238935514892607	0.7513292404621
**Grem2**	Grem2	-0.0598303772141924	5.31410941333544	1.38590556431188	0.239097770556084	0.7513292404621
**Casp1**	Casp1	0.225279059010633	-0.392892601882361	1.38558740929778	0.239151696272106	0.7513292404621
**Ncam2**	Ncam2	-0.072644479058487	6.64765246525108	1.38537584037193	0.239187547390636	0.7513292404621
**Lhx6**	Lhx6	0.0731151029946835	5.94247630256673	1.38529962802011	0.239200486512005	0.7513292404621
**Exoc2**	Exoc2	0.0312124462400483	7.27011903108958	1.38511424329685	0.239231922912756	0.7513292404621
**Nostrin**	Nostrin	0.127951928330131	1.92802930733611	1.38510690647517	0.239233167149055	0.7513292404621
**Srpk1**	Srpk1	0.028469450551608	6.87328952300179	1.38505074131223	0.239242692342384	0.7513292404621
**Trerf1**	Trerf1	0.0834996446527769	4.89767132722023	1.38457770192775	0.239322934579079	0.751392112416499
**Orc5**	Orc5	-0.0465147791127495	4.23621905541201	1.38331633713145	0.239537061263048	0.751875198334353
**Dnajc27**	Dnajc27	-0.0376988215982936	6.19808365600488	1.38222238805585	0.239705972986408	0.752094708541018
**Efhc2**	Efhc2	0.134140102019853	1.08406800879814	1.38207324286795	0.239748314141053	0.752094708541018
**Zbtb7c**	Zbtb7c	-0.0968515573588206	3.39676965267677	1.38129086440536	0.239881388523498	0.752094708541018
**Ikbkg**	Ikbkg	0.0835737838635512	2.12523072169731	1.3812221279567	0.239893084162424	0.752094708541018
**Retreg2**	Retreg2	-0.0273455395235394	7.96652719167385	1.38113219995739	0.239908386630861	0.752094708541018
**Fga**	Fga	-0.190849524630826	-0.489567815694622	1.37947636545322	0.240190378512563	0.752789588721742
**Plekhg1**	Plekhg1	-0.0993730169379153	3.48697485464299	1.3778295845498	0.240471194230126	0.753277986511976
**Tmem88**	Tmem88	0.123209000481568	2.09025877545017	1.37751559121628	0.240524786195624	0.753277986511976
**Adam19**	Adam19	0.0566095042921446	6.0285099915091	1.3775006884055	0.24052733014857	0.753277986511976
**Pfkfb4**	Pfkfb4	-0.0428083086224801	4.65046228106444	1.3766075443372	0.240679852184132	0.753566923212776
**Spock1**	Spock1	-0.0505887852576266	7.1717753790542	1.37564099776948	0.240845041688388	0.753654837960869
**Slc38a9**	Slc38a9	0.0838543613219233	2.39294693984492	1.37516121843365	0.240927090513783	0.753654837960869
**Emc9**	Emc9	-0.0730783857416537	3.44624186716477	1.37501264074228	0.24095252622472	0.753654837960869
**Pik3cd**	Pik3cd	0.053816100588818	6.44752041137963	1.37423927108888	0.241084851578631	0.753654837960869
**Ankrd17**	Ankrd17	0.0509167012263244	7.93750224305111	1.37409562347194	0.241109441761453	0.753654837960869
**Mlst8**	Mlst8	-0.0477523189850976	4.73046632429818	1.37354339737292	0.24120403078524	0.753654837960869
**Tor4a**	Tor4a	0.0913516113729754	2.54316448135835	1.37233284258302	0.241411473156081	0.753654837960869
**Ndufa13**	Ndufa13	-0.0721888342269173	5.88052693432354	1.37229554362061	0.241417868822415	0.753654837960869
**Pogz**	Pogz	0.0609101049766352	7.038850684971	1.37223388381334	0.241428442103658	0.753654837960869
**Ranbp2**	Ranbp2	0.0714323390667425	7.26337967762786	1.37204926052871	0.241460104249526	0.753654837960869
**Emg1**	Emg1	0.0454233611874583	5.31028221480326	1.37199666603769	0.241469124914463	0.753654837960869
**Smarca2**	Smarca2	0.0381676708189788	8.09934448452862	1.37195154388774	0.24147514906656	0.753654837960869
**Asb1**	Asb1	0.0437916317660217	5.03674521686813	1.3714282904658	0.24156663698868	0.753654837960869
**Dph5**	Dph5	0.0612172562186054	3.80835849632359	1.37112967322163	0.241617823538249	0.753654837960869
**Amdhd2**	Amdhd2	0.0586057831024608	3.88118292173759	1.3707082164043	0.241690239607048	0.753654837960869
**Tnfrsf12**	Tnfrsf12a	0.129525654448777	0.653921640384557	1.37001202962777	0.241809816859051	0.753654837960869
**Slc10a4**	Slc10a4	-0.167700860128877	4.2617614780032	1.36947609905812	0.24190191752922	0.753654837960869
**Slc35d3**	Slc35d3	-0.173129147342224	3.62998688823482	1.36928692052633	0.241934428397855	0.753654837960869
**Hspa9**	Hspa9	-0.0276547039531296	7.79706297867029	1.36845263227797	0.242077820867263	0.753654837960869
**Grin3a**	Grin3a	-0.0598635430685525	6.65592935629649	1.36843622829525	0.242080743083624	0.753654837960869
**Por**	Por	0.0319118834624713	6.82177893670921	1.36842372356641	0.242082894499059	0.753654837960869
**Galr2**	Galr2	-0.0582335888958736	3.95378188209834	1.36810096594666	0.242138432148022	0.753654837960869
**Ddah1**	Ddah1	-0.0748958699369405	7.49111474169424	1.36783981753108	0.242183379914643	0.753654837960869
**Ankra2**	Ankra2	-0.0507059098549644	4.95370651583896	1.36775634129363	0.242197749636363	0.753654837960869
**Rras2**	Rras2	0.0480827705646159	6.20788895828573	1.36763782249122	0.24221815342014	0.753654837960869
**Atf7**	Atf7	0.113587960362197	3.76795794781563	1.36720388965249	0.242292875649527	0.753699379077324
**Surf1**	Surf1	-0.0537853042884417	4.29956659196701	1.36562459340917	0.242565063942296	0.754358001696916
**Nr1i3**	Nr1i3	-0.0566711607916592	3.66499947795022	1.36389137507492	0.242864209214107	0.755100109236087
**Adra2a**	Adra2a	0.0573118439663604	5.19871934028478	1.36323928629665	0.242976873008972	0.755147606474284
**Slitrk2**	Slitrk2	0.0714751198116372	4.21832637645451	1.36268698390035	0.243072346160788	0.755147606474284
**Slc6a9**	Slc6a9	-0.0504261469187494	6.22213743655838	1.36204394463893	0.24318356205264	0.755147606474284
**Sec23b**	Sec23b	-0.112644866307168	4.74790671362404	1.3614974734011	0.243278124920252	0.755147606474284
**Vim**	Vim	0.0813413987856735	7.95377797726791	1.36132943186441	0.243307212264374	0.755147606474284
**Lrfn1**	Lrfn1	-0.0340449702705285	7.28882165294199	1.36118188077386	0.243332756342941	0.755147606474284
**Ldlrap1**	Ldlrap1	0.123587025573343	2.00456037448794	1.36106061185181	0.243353752989394	0.755147606474284
**Idnk**	Idnk	0.116548152618328	1.88294052973036	1.36086285708001	0.243387996912935	0.755147606474284
**Kcnd3**	Kcnd3	-0.0740112247150856	6.64173779473472	1.3603998631235	0.243468193851276	0.755147606474284
**Xpo5**	Xpo5	0.0478190564556713	6.55102104059351	1.36015294153424	0.24351097724987	0.755147606474284
**LOC100(**	LOC100125	-0.131877427528553	1.08492036487785	1.35986981591917	0.243560064897615	0.755147606474284
**Oca2**	Oca2	0.304752349529216	-0.304901612977673	1.35945306841026	0.243632240370476	0.755147606474284
**Eif2ak1**	Eif2ak1	0.0333875444291045	6.20376165606659	1.3592570254316	0.243666287061431	0.755147606474284
**Zfp57**	Zfp57	0.0421324775732003	5.75679245282253	1.35841149167346	0.243812973740588	0.755414569962978
**Fam120b**	Fam120b	0.0370640955679557	6.84344422850524	1.35764925032878	0.243945302819239	0.755620341784534
**Shisa6**	Shisa6	0.0803852763651495	4.1391446571017	1.35707858442272	0.244044430745042	0.755620341784534
**Fads1**	Fads1	-0.028164148386226	7.54989684172809	1.35621856175281	0.244193914432684	0.755620341784534
**Mgat1**	Mgat1	0.04076796749499	5.94048095898645	1.35604071119678	0.24422484122525	0.755620341784534
**Vmo1**	Vmo1	0.264974028879	-0.219561482264212	1.35550503733253	0.244318019511674	0.755620341784534
**Pkn2**	Pkn2	0.0499479778458101	5.50889317248814	1.35488099472985	0.244426823884194	0.755620341784534
**Khk**	Khk	-0.0876998732576853	2.70227742147763	1.35469524189508	0.244458962492431	0.755620341784534
**Btk**	Btk	0.246896921985104	-0.659152504839773	1.3544648010561	0.244499088307498	0.755620341784534
**Plpp2**	Plpp2	0.160925007959927	0.88588539389771	1.35429325505234	0.244528964200382	0.755620341784534
**Ap5z1**	Ap5z1	0.0486044347561847	4.2174562559001	1.35407891792235	0.244566298716832	0.755620341784534
**Dnaja4**	Dnaja4	-0.04373774985792	5.16587267328221	1.3538321315707	0.244609294048028	0.755620341784534
**Ttbk2**	Ttbk2	0.0631800161607272	6.14414352150863	1.35383176881305	0.244609357254767	0.755620341784534
**Mtor**	Mtor	0.0396207079156628	7.65251402035227	1.35320699600855	0.244718246982314	0.755675337041106
**Nr0b1**	Nr0b1	-0.256133559170254	-0.63825940073172	1.35305323824815	0.244745053857747	0.755675337041106

Gcnt1	Gcnt1	0.108977701778645	2.31254713942256	1.35159544115231	0.244999393204941	0.756115952376365
Macrod1	Macrod1	-0.0993933171223785	3.44294368192444	1.35136686094675	0.245039302446549	0.756115952376365
Mtdh	Mtdh	0.0345485706933182	6.0267020882319	1.35119358572547	0.245069950904865	0.756115952376365
Hcrtr1	Hcrtr1	-0.167603289754201	2.06801546065581	1.35075797438472	0.245145650417476	0.756163728123323
Parp2	Parp2	0.0553356044692756	4.2348027106798	1.35038237860658	0.245211279984489	0.756179224046278
Rsph6a	Rsph6a	-0.142045155773421	1.49487466921059	1.34905888785664	0.245442710686402	0.756705881206409
Tshz3	Tshz3	0.0775536285290022	6.43428465149707	1.34842244796663	0.245554095988029	0.756808623155906
Gnl1	Gnl1	-0.0382682442935047	6.82952106375899	1.3481754052906	0.245597348323585	0.756808623155906
Igf1	Igf1	-0.123533407988218	2.88602411691803	1.3477239926176	0.245676405885332	0.756865312680507
Trim2	Trim2	0.0401879049301781	9.36902552077074	1.34692542551912	0.245816338804657	0.756912473109721
Sult4a1	Sult4a1	-0.0461865299031452	7.82146319838258	1.34573562580888	0.245949609071105	0.756912473109721
Elf4e3	Elf4e3	0.0673257345792658	3.37488369339201	1.34517588226806	0.245947768397349	0.756912473109721
Pmp22	Pmp22	0.0857931305058033	4.35518409289371	1.34562982722083	0.246042569960024	0.756912473109721
Hey2	Hey2	-0.0854727250328976	3.08434576573358	1.34550106585745	0.246066172568977	0.756912473109721
Trappc6	Trappc6a	0.13819022880794	2.00548809182898	1.34546545436757	0.246072419246846	0.756912473109721
Ampd3	Ampd3	-0.0901136648536494	4.25435528535218	1.34521508940702	0.246116366386643	0.756912473109721
Cog1	Cog1	0.0628021550934794	5.62044118291276	1.34470454592151	0.246206031299047	0.757001583066065
Zfp574	Zfp574	0.0342659527208908	5.36274686828963	1.34395428754136	0.246337823687508	0.757072632708946
Gnb3	Gnb3	0.421744141434677	2.47016418459945	1.34299116282322	0.246507158709957	0.757072632708946
Prkch	Prkch	-0.105685864445552	4.84795932880461	1.34146219720635	0.246776271336534	0.757072632708946
Hspa12a	Hspa12a	0.0308671955677478	7.9705586493363	1.34111773820492	0.246836849000483	0.757072632708946
Ddx25	Ddx25	-0.0380744590814723	5.82455243359606	1.34111096544389	0.246838142229021	0.757072632708946
Agpat3	Agpat3	-0.0297115950533641	7.81076540715479	1.34077616802607	0.246897125804869	0.757072632708946
Ddx59	Ddx59	-0.0529422584703052	3.95442418410821	1.34065497162605	0.246918495583105	0.757072632708946
Dis3	Dis3	0.0636369217452336	4.90638592851958	1.34038274674757	0.246966481339952	0.757072632708946
Brinp2	Brinp2	-0.0264894443532064	6.52043626018688	1.34036502814189	0.246969605503147	0.757072632708946
Nt5c3b	Nt5c3b	-0.0661351711840361	5.55001695202685	1.34011428387001	0.247013815031181	0.757072632708946
P3h1	P3h1	0.0724855094810782	3.78151716780678	1.34005532273773	0.247024212174298	0.757072632708946
Tmem39	Tmem39b	0.0688395574765274	4.6702282988425	1.33961932269792	0.247101110807689	0.757072632708946
Zfp423	Zfp423	-0.104990483025634	6.30684074570495	1.33923808642551	0.247168324954261	0.757072632708946
Slit3	Slit3	0.0879323987658243	4.08041934272631	1.33808570372051	0.247371840516221	0.757072632708946
Cyp4f4	Cyp4f4	-0.197634565614581	0.201473214860424	1.33771288892035	0.247437743130257	0.757072632708946

[illegible]

**Rbm38**	Rbm38	0.0629165967670228	3.56428949664957	1.21988604251009	0.269362391196604	0.767101146611116
**Kansl2**	Kansl2	0.0282842533531294	6.88298152469457	1.21983570747333	0.269392896897952	0.767101146611116
**Ganab**	Ganab	0.0260638909173207	7.57848913424634	1.21978178279306	0.269403481478932	0.767101146611116
**Magt1**	Magt1	0.0773011716282754	2.64396795608604	1.21974504094923	0.269410693632276	0.767101146611116
**Fosb**	Fosb	-0.192143484192297	0.514861920143005	1.21917181412329	0.269523245089846	0.767246527204304
**Otulin**	Otulin	0.0404126459745747	4.99068887222819	1.21898576558603	0.269616760470347	0.767282265256858
**Htatsf1**	Htatsf1	-0.0453428982901044	6.66024074084151	1.21848008494362	0.269659142309035	0.767282265256858
**Elmo3**	Elmo3	0.15771528536409	1.31983348644343	1.21811024904014	0.269731825451559	0.767315118770884
**Cacng2**	Cacng2	-0.0610528404574041	4.81580013152566	1.21738532851706	0.269874391363213	0.76746229757775
**Npat**	Npat	0.0613284327512159	4.84309980800573	1.21722165596216	0.269906593072948	0.76746229757775
**Rtn1**	Rtn1	-0.0382903049814717	10.8685779179361	1.2160343231409	0.270140324039989	0.767828415244896
**Usp9x**	Usp9x	0.0634389608267806	9.09387812050545	1.21574040595669	0.270198221180324	0.767828415244896
**Tmub2**	Tmub2	-0.0294848856125485	6.17549608251577	1.21533664690598	0.270277782236312	0.767828415244896
**Magi1**	Magi1	-0.0524307125956702	6.46938542174462	1.21521282153419	0.270301970985687	0.767828415244896
**RGD130[illegible]**	RGD130877	-0.132895204511692	0.651916748974063	1.21477056649906	0.270388937728815	0.767828415244896
**Peak1**	Peak1	0.0819598701179532	3.91813245572636	1.21469342587969	0.270404588970582	0.767828415244896
**Sod2**	Sod2	-0.0412498352942446	5.90292470717956	1.21389257514259	0.270562575310719	0.767868521862234
**Rraga**	Rraga	-0.0402064766378077	6.73810295688551	1.21354027513513	0.2706321111872	0.767868521862234
**Ptchd1**	Ptchd1	0.063468704855345	3.31348837334507	1.21339801292654	0.270660196791166	0.767868521862234
**Col26a1**	Col26a1	0.0864730030129155	5.08061152107422	1.21264825865464	0.270808274558428	0.767868521862234
**Prdm1**	Prdm1	0.128171477712631	0.898970666109445	1.2125666737282	0.270824393833542	0.767868521862234
**Fyb1**	Fyb1	0.186659690505515	0.215162816387169	1.21255048770838	0.270827591955482	0.767868521862234
**Rad9b**	Rad9b	-0.0976368512517746	1.07452544101701	1.21243955315469	0.270849651227985	0.767868521862234
**Tekt4**	Tekt4	-0.187702898387804	0.454941907409006	1.21187787288562	0.270960532625888	0.768008760920793
**Asmtl**	Asmtl	-0.0566817376366856	5.16362880940112	1.21131626349476	0.271071576091412	0.768149001782538
**Cnot1**	Cnot1	0.0403757194180884	7.62239215925057	1.21049128505318	0.271234845970641	0.768437141956333
**Pcdhb8**	Pcdhb8	-0.0836825072S058	3.49015213(illegible)	1.2081052306734	0.271509401089385	0.768889454479422
**Ccdc28a**	Ccdc28a	0.0902704493075417	1.85880015679914	1.20864454093038	0.271600733345099	0.768889454479422
**Kif21b**	Kif21b	0.0644025567593567	9.04352525721949	1.2080585412163	0.271716863963854	0.768889454479422
**Slitrk4**	Slitrk4	0.0458897492965826	4.84918701552721	1.20801075527615	0.27172644854	0.768889454479422
**Prps1**	Prps1	-0.0315431282577488	7.16006724989483	1.20777732451301	0.271772764249139	0.768889454479422
**Slc12a2**	Slc12a2	0.0438153787110846	6.2487295129771	1.20767440142917	0.271793190296477	0.768889454479422
**Atp6v1d**	Atp6v1d	-0.0449587508079847	6.98248892600992	1.20750975308599	0.271825870298047	0.768889454479422
**Cytip**	Cytip	0.192128698711229	0.300058248680859	1.20718867094611	0.271889613986225	0.768895447349531
**Lrrk1**	Lrrk1	0.120563104109766	2.28463926946999	1.20599405716523	0.272126942284 23	0.769283165669315
**Casq2**	Casq2	0.252815739890437	-0.317040489214583	1.20587792516798	0.272150027521177	0.769283165669315
**Lrrc28**	Lrrc28	-0.0405654215227477	4.78189961215388	1.20496273947956	0.272332037972122	0.769574232135899
**Tlcd1**	Tlcd1	-0.0852183968B3927	2.51010116152063	1.2045611150665	0.272411960429256	0.769574232135899
**Hlx**	Hlx	0.159532370437888	0.0419846131786872	1.20429896203282	0.272464208883874	0.769574232135899
**Nop10**	Nop10	-0.0537510605422675	5.99459020562648	1.20382259410857	0.272559112853228	0.769574232135899
**Septin9**	Septin9	0.0320331370675758	7.63263270729379	1.20389476758405	0.272584461905986	0.769574232135899
**Cry1**	Cry1	0.0683650547782593	4.99692138654822	1.20293994081463	0.272734898966618	0.769574232135899
**Mdh1**	Mdh1	-0.0442226459160014	8.39726588510432	1.2026988957524	0.272782922518667	0.769574232135899
**Alg12**	Alg12	0.0514311710944288	3.85588542887923	1.20253608456567	0.272814727249797	0.769574232135899
**Lcor**	Lcor	0.106837802001227	3.00657380182699	1.20221582480065	0.272879236209534	0.769574232135899
[illegible]	[illegible]	[illegible]	[illegible]	[illegible]	[illegible]	[illegible]

Onecut1	Onecut1	-0.285162261207767	0.0565484648019107	1.08673785712961	0.297195126116923	0.786196368240332
Adap2	Adap2	0.148513689159542	0.942734432878808	1.0864199522734	0.297265795123214	0.786196368240332
Pcdhb5	Pcdhb5	0.0538597276410336	3.7769488198787	1.08631572591308	0.297288968931465	0.786196368240332
Smad1	Smad1	0.0273027582887405	6.23738700236831	1.0855177825276	0.297466461455929	0.786499054796689
Ppp1r9a	Ppp1r9a	0.0498802131605598	6.68018655568493	1.08458873744328	0.297673287405487	0.786770897606914
Trak1	Trak1	0.0349643732848972	7.64354087921162	1.08437691927102	0.297720468660542	0.786770897606914
Snrpa	Snrpa	0.0645243913846252	5.24407734875826	1.08420642581559	0.297758468826914	0.786770897606914
Usp47	Usp47	0.0284250000875264	7.97770066430347	1.08301751662725	0.298023496396491	0.787198928374375
Prdm11	Prdm11	0.0640277084579888	3.92472272456924	1.08291389400788	0.298046624800877	0.787198928374375
Cops5	Cops5	-0.0337131450224536	6.54704066880619	1.08254166293307	0.298129663724108	0.787251600907025
Pi15	Pi15	0.206901384572362	0.0701307130913127	1.08185284349398	0.298283432177551	0.787398446594424
Tex261	Tex261	0.0284959992860076	5.07978352066002	1.08153821774919	0.298353745585626	0.787398446594424
Aox1	Aox1	0.165231583857361	1.19580288617	1.08144467313191	0.298374597453263	0.787398446594424
Lrrc23	Lrrc23	-0.0739323874878282	4.21424907543263	1.08100189701727	0.298473510548822	0.787492914170046
Qrich1	Qrich1	-0.0380177299705422	6.66773867783221	1.08010632897435	0.298673768238536	0.787679625285302
Ajap1	Ajap1	-0.0514452009709997	5.32470753153799	1.0794249439234	0.298826223557286	0.787679625285302
Ttc21b	Ttc21b	0.0809650465435313	4.34188311032811	1.07933789976765	0.298845710581755	0.787679625285302
Plcxd2	Plcxd2	0.0345488536744251	7.61633419286721	1.07930372897371	0.298853368878635	0.787679625285302
Commd6	Commd6	-0.0485992579984465	4.72152375843344	1.07927441970776	0.298859929959099	0.787679625285302
Psmd6	Psmd6	-0.0347116481658506	7.05616592143613	1.07894055172589	0.298934682726415	0.787710250556907
Cntnap5c	Cntnap5c	-0.0931189360730931	3.57887141434512	1.07762315963615	0.299229880502456	0.788216042096677
Gjb2	Gjb2	0.235223524178806	3.67168849401697	1.07752015733929	0.299252976780251	0.788216042096677
Atp5mc3	Atp5mc3	-0.0503157813232472	7.58448942296458	1.07718988643006	0.299327094033055	0.788264881175893
Col17a1	Col17a1	0.278790115228325	-0.788086710354597	1.07628040462054	0.299576053933463	0.788511974045122
Tax1bp3	Tax1bp3	0.0515717188240166	5.05674978088754	1.07597235992772	0.299600317010957	0.788511974045122
Tiparp	Tiparp	0.0585664228881659	3.86734270189275	1.07575174688968	0.299649866873172	0.788511974045122
Gramd1b	Gramd1b	0.0481696591942072	5.81894955414824	1.07561174852167	0.299681316095373	0.788511974045122
Ubxn6	Ubxn6	-0.030765848275465	6.32972877202723	1.07520440370509	0.299772846514535	0.788586526716325
Tas1r1	Tas1r1	-0.0750908275813381	2.55858370467164	1.07489522747197	0.299842328571672	0.788593584215726
Hsf4	Hsf4	-0.0882240915281257	2.25235509637547	1.07455381355548	0.299919107878696	0.788593584215726
Calca	Calca	0.257391557739798	-0.448369188275555	1.07434908936992	0.299965151731012	0.788593584215726
Gen1	Gen1	0.112266967076519	1.59748154250868	1.07385607048545	0.300076072362884	0.788719013032853
Nudc	Nudc	-0.0327243940142532	7.31165439925632	1.07321975419989	0.300219310487238	0.788782368276682
Trim46	Trim46	0.0246528895231919	7.30229798052572	1.07317090189989	0.300230311034741	0.788782368276682
Tox	Tox	-0.0649798217673925	5.30365963472296	1.0728187270107	0.300309629039126	0.788782368276682
Hus1	Hus1	0.0566190639090309	4.1667389348558	1.07231182534882	0.300423842510193	0.788782368276682
Pnpla8	Pnpla8	-0.0274586896942875	7.54824493228143	1.07223250604619	0.300441719545365	0.788782368276682
Gpnmb	Gpnmb	0.129686829394336	2.52988165116779	1.07185671656366	0.300526423946376	0.788782368276682
Pdcd5	Pdcd5	-0.0405285052065382	5.76108167616909	1.07178452671426	0.300542708887341	0.788782368276682
Gpm6b	Gpm6b	-0.0295349183436967	8.32280972438159	1.07140222258228	0.300628703810387	0.788842131954195
Myl3	Myl3	0.179076272880568	0.217654921327655	1.07038344248257	0.300858851299502	0.789280043662559
Nlrp3	Nlrp3	0.176336630726008	0.0744041794333572	1.06930519437572	0.301102438866855	0.789620369388812
Mblac2	Mblac2	-0.0381753272096677	5.00623199743229	1.06923209090773	0.301118862686517	0.789620369388812
Cpt1a	Cpt1a	0.0502003753866932	5.16888825956241	1.06843158783687	0.301299981181729	0.789929036170272
Gpr155	Gpr155	0.0458752876717381	4.88057427504026	1.06684504592092	0.301659164107742	0.790521786688346
Sulf2	Sulf2	-0.0575084656838418	7.82432016036136	1.06680235054642	0.301668827676953	0.790521786688346
Mcur1	Mcur1	0.0734621045849006	3.03306366442933	1.06605269542167	0.301838893041303	0.790521786688346
Cdc14b	Cdc14b	0.126820139047864	1.26862351290489	1.06577771418017	0.301901111487319	0.790521786688346
Kdelr2	Kdelr2	0.0311399840888155	6.30855967709507	1.06556949303015	0.301948567988587	0.790521786688346
Gucy1a1	Gucy1a1	0.0543459752118239	7.35582646046534	1.0655045843269	0.301963065966863	0.790521786688346
Zfp524	Zfp524	0.0833545562718468	2.45089926491097	1.06546689242414	0.301971584289203	0.790521786688346
Kpna4	Kpna4	0.0296101377373342	6.62789392308896	1.06502938396424	0.302071122488528	0.790521786688346
Pus1	Pus1	0.0698814088584302	4.20675712210101	1.06491745092988	0.302096301909917	0.790521786688346
Sting1	Sting1	0.180895154389709	-0.158411743452045	1.06352064757444	0.302413578215338	0.790886787958411
Atp5f1e	Atp5f1e	-0.0679326827907396	6.88570177521258	1.06352018415653	0.302413680549589	0.790886787958411
Cwc25	Cwc25	-0.0499961759617225	4.31952953886584	1.06342209753609	0.302435979556001	0.790886787958411
Shpk	Shpk	0.0802328656578494	2.01164697263471	1.0629051138301	0.302553529884858	0.790886787958411
Rnf150	Rnf150	0.0605217779248969	3.67224731182584	1.06284903754124	0.302566283901682	0.790886787958411
Wfdc6a	Wfdc6a	0.237496749771356	-0.294498241603007	1.06255572681549	0.302633005593845	0.790886787958411
Mpdu1	Mpdu1	-0.0464015028907355	4.69530652144735	1.06236819149809	0.302675676244403	0.790886787958411
Mansc1	Mansc1	0.114116747945724	1.42883113187303	1.06171926063183	0.302823388811022	0.790962233118949
Kcnh6	Kcnh6	-0.101961750905407	2.60452652349645	1.06132664558191	0.302912802813897	0.790962233118949
Ccne2	Ccne2	0.0702251790339032	3.92058803083759	1.06093170861358	0.303002780010246	0.790962233118949
Arhgap12	Arhgap12	0.0360994501134875	6.27099989274092	1.06089582950498	0.303010956168222	0.790962233118949
Zfp161	Zfp161	0.0640988081466768	5.13897503389034	1.06084931816303	0.303021555108026	0.790962233118949
Gnrh1	Gnrh1	-0.241784752908631	-0.417080726947467	1.05932281283403	0.303369696448387	0.791705412636796
Styx	Styx	0.0623701354723819	4.19552385188102	1.05901746646004	0.303439385055242	0.791721707932717
Ift46	Ift46	-0.0380360254798472	5.26357727782559	1.05781664778241	0.303713685867925	0.791922472061573
Slc2a12	Slc2a12	0.0961771004643481	3.13418438109113	1.05763812277905	0.303754493136871	0.791922472061573
Orc3	Orc3	0.0645222716363044	4.48002258316845	1.05760315387553	0.303762487202672	0.791922472061573
Neurl2	Neurl2	-0.0885556178017401	1.66734886626386	1.05719523420958	0.303855759765134	0.791922472061573
Uggt1	Uggt1	0.0556418125859848	6.42701563332885	1.05718319481565	0.303858513186053	0.791922472061573
Acox1	Acox1	0.0297406473724663	6.62792009310099	1.05688294264477	0.303904315779501	0.791922472061573
Fzd9	Fzd9	-0.0621077495588915	4.51810208112088	1.05673680801766	0.303960625046315	0.791922472061573
Jak1	Jak1	-0.0289156486671316	7.62152937249455	1.056220990526	0.304078574571186	0.791996400682455
Shc3	Shc3	0.0605287290473503	5.42383278788956	1.05570825608573	0.304196054385703	0.791996400682455
Tead2	Tead2	0.0709910932062939	3.68812874385367	1.05549806947421	0.304244221411754	0.791996400682455
Glrx5	Glrx5	-0.0601558298076204	6.07283565424224	1.05489058821242	0.304383422773877	0.791996400682455
Edem3	Edem3	0.0386118891756741	5.88346196172129	1.05470264991963	0.304426504889698	0.791996400682455
Abracl	Abracl	0.0514448216797178	4.7490392435883	1.05445111322509	0.304484178070088	0.791996400682455
Tnr	Tnr	0.0668544830079028	6.2811570032765	1.05381085543465	0.304631042296974	0.791996400682455
Dtx3l	Dtx3l	0.0920189808130141	1.52322524280741	1.05311368404439	0.304791065779172	0.791996400682455
Hadhb	Hadhb	0.0346173205726298	6.06207746642657	1.05292648852974	0.304834051753116	0.791996400682455
Npy5r	Npy5r	0.127229267700876	0.625008909865867	1.05259982705763	0.304909082285719	0.791996400682455
Drp2	Drp2	0.0677834186380803	4.72837999712848	1.05256585179341	0.304916864427409	0.791996400682455
Lsm11	Lsm11	-0.0548348725757262	5.30297012349031	1.05238457150261	0.304958537172834	0.791996400682455
Atp5f1d	Atp5f1d	-0.0473132579114026	7.56079019526015	1.05213432480394	0.305016064313095	0.791996400682455
Btbd6	Btbd6	-0.0229669610509852	6.44859157984308	1.05148707998116	0.305164847444376	0.791996400682455
Bzw2	Bzw2	0.0328650560166993	7.70082942438908	1.0513967300728	0.305185166590114	0.791996400682455
Akirin1	Akirin1	-0.0290033436470197	6.41229841944705	1.05127728344925	0.305213100336218	0.791996400682455
Tmem248	Tmem248	-0.0438015147539425	4.7927814666176	1.05106532632412	0.305261860189507	0.791996400682455
Cabin1	Cabin1	0.0315099777502283	7.22473844302959	1.05104185215259	0.305267227937595	0.791996400682455
Met	Met	0.134248807458457	5.7444342825251	1.05086039254087	0.305309013941496	0.791996400682455
Mvk	Mvk	0.0377393214411259	5.9619961755882	1.05054520453238	0.305381554784162	0.791996400682455
Mmaa	Mmaa	0.0704952504323056	4.11763797631975	1.05054465093302	0.305381715592304	0.791996400682455
Prokr2	Prokr2	0.0720788604109672	3.12453165474501	1.05029909203578	0.305438164270911	0.791996400682455
Sox6	Sox6	0.0582727645501352	4.25987694942595	1.05006127823769	0.305492974356684	0.791996400682455
Elk1	Elk1	0.035847805417104	5.82519550955535	1.04972907871873	0.305569490708761	0.791996400682455
Mir3064	Mir3064	0.17725315077252	1.42633218905156	1.04970119870346	0.305575812511689	0.791996400682455
Gcc2	Gcc2	0.0354632106783506	6.18494747232483	1.04913156313027	0.305707180458996	0.792172064504028
Trappc13	Trappc13	-0.0324066823849296	5.97362966644289	1.04874470910676	0.305796368829215	0.792228640756254
Depdc1b	Depdc1b	0.117879032904156	1.40106486559568	1.04728546422683	0.306133098159432	0.792513978242537
Igfbp5	Igfbp5	-0.0677911871152855	6.55967510554099	1.04676210795799	0.306253751700013	0.792513978242537
Peli2	Peli2	0.0631787985802387	4.74771781003964	1.04674672813796	0.306257526097604	0.792513978242537
Ifih1	Ifih1	0.106930993653964	1.10965581521611	1.04649354831912	0.306316038531355	0.792513978242537
Aebp2	Aebp2	-0.0512564492778437	3.86020408935254	1.04634114236177	0.306351262063614	0.792513978242537
Tcp11	Tcp11	-0.213981133883999	0.0835424690624987	1.04618223954162	0.306387992718363	0.792513978242537
E2f7	E2f7	0.0934447382779553	2.36272718465628	1.04603510389792	0.306422008506464	0.792513978242537
Spred1	Spred1	0.0486820985710643	4.51678998918966	1.04593741010549	0.306444596628583	0.792513978242537
Gon7	Gon7	-0.0545185351052015	4.26960555082485	1.04580892125953	0.306474308329231	0.792513978242537
Recql5	Recql5	0.0572268618322265	4.28833289282158	1.04541292530695	0.306565609950012	0.792587101935935
Lrrc59	Lrrc59	-0.0270541003747436	7.59675168132671	1.04469277810798	0.306732320428437	0.792777855835533
Pex19	Pex19	-0.0264825771882796	6.68677218934523	1.04445612971259	0.306787351659467	0.792777855835533
Ydjc	Ydjc	0.0606748004970893	6.15494117786766	1.04427646318421	0.306828961275129	0.792777855835533
Ptpn2	Ptpn2	0.0652217912692592	3.62388764655595	1.04379708894387	0.306940010942142	0.792896587272278
Klf6	Klf6	-0.0363094871649884	7.05982146545487	1.04342421291418	0.307026425715198	0.792955943479006
Pom121	Pom121	0.0345241238048227	6.92201898271185	1.04268301024375	0.307198094705573	0.793161082024556
Phf11b	Phf11b	-0.196039554573106	-0.176031728918296	1.04224999627695	0.307298759105767	0.793161082024556
Ddn	Ddn	-0.0624018541862238	7.20445997128084	1.04217119208953	0.307317047205243	0.793161082024556
Gng3	Gng3	-0.0403345777217469	8.05639727705875	1.04195736748841	0.307366657600916	0.793161082024556
Purg	Purg	0.0510967627524881	4.02743082479328	1.041501130697	0.307472692547298	0.793161082024556
Mpc1	Mpc1	-0.0424807377645041	6.66457427258189	1.04123856893571	0.307533588426618	0.793161082024556
Trappc9	Trappc9	0.0279151736088377	6.72145155876377	1.04110889369485	0.307563712765553	0.793161082024556
Slc18a2	Slc18a2	-0.117239672476909	4.24511410837753	1.04089018492166	0.307614528880635	0.793161082024556
Hpdl	Hpdl	0.0789059691868578	2.72279472280397	1.04054220950555	0.307695401856705	0.793205687802914
Hsd17b12	Hsd17b12	-0.0321158911078708	6.44477663610106	1.03994943644278	0.307833222944917	0.793397073946855
Skp2	Skp2	0.0497162773609856	4.44695868067371	1.0394534668875	0.307949080511139	0.793517524664388
Trappc10	Trappc10	-0.0212338721971011	7.42847590924276	1.03891422224737	0.308074129588817	0.793517524664388
Map7	Map7	-0.0729457082054881	4.10482794802292	1.0388515317856	0.308088725717724	0.793517524664388
Six6	Six6	-0.384010140260067	-0.0747542526513999	1.03852766582209	0.308164145262215	0.793517524664388
LOC100361	LOC100361	0.147627846775747	1.25661496567514	1.03838252333356	0.308197950437094	0.793517524664388
Supt5h	Supt5h	-0.0301754475812075	7.76371209714074	1.03783355617965	0.308325985522908	0.793580931891566
Irf9	Irf9	0.0751155751123466	3.56277986510212	1.03753211068578	0.308396123063564	0.793580931891566
Cul1	Cul1	-0.0223427476459066	7.47664336450517	1.0373509582802	0.308438370233766	0.793580931891566
Serinc1	Serinc1	-0.03579232979544	9.66816525367575	1.03718530918507	0.308476899252552	0.793580931891566
Dll1	Dll1	0.0664347612662979	4.61080718078549	1.03622855521959	0.308700235294485	0.793677941051087
Zfp263	Zfp263	0.0625021651699673	4.26528301587335	1.03619482645854	0.308708108937149	0.793677941051087
Megf10	Megf10	0.0942510568508246	3.4953323025851	1.03598080807984	0.308758068468344	0.793677941051087
Rrs1	Rrs1	0.038336514376715	4.94252614822383	1.03593236518964	0.308769153321105	0.793677941051087
Perm1	Perm1	0.171190982108459	0.827023752323635	1.03544888221539	0.308882308888448	0.793713104825086
Cd2bp2	Cd2bp2	-0.0331268094018074	7.19447980715094	1.03533009297695	0.308910061475564	0.793713104825086
Aldh3b1	Aldh3b1	0.153631716777119	0.96351628128247	1.03479210496729	0.309035791504245	0.793777067616127
Mad2l1	Mad2l1	0.0520258865026141	3.71479865820993	1.03414156475165	0.309187918986307	0.793777067616127
Dffa	Dffa	0.0319784930009259	5.47297857169687	1.03409755462751	0.309198209182906	0.793777067616127
Thoc7	Thoc7	-0.0412641645886536	5.9635558412689	1.03392721128589	0.309238060201196	0.793777067616127
Amn	Amn	-0.12724937595023	1.4400784986432	1.03345425465419	0.309348741179447	0.793777067616127
Timp1	Timp1	0.0888256681771502	2.63211538213298	1.03340452362806	0.309360382181573	0.793777067616127
Tst	Tst	-0.0572958926724256	5.12394925741544	1.03314311231014	0.309421582580631	0.793777067616127
Dll4	Dll4	0.0738304764536301	2.51531045210099	1.0329408093164	0.309468865612866	0.793777067616127
Hltf	Hltf	0.0435618040455613	5.23849457242873	1.03277611613241	0.309507528568763	0.793777067616127
Mff	Mff	-0.0269002428972544	7.88092747418991	1.03235755666619	0.309605587941989	0.793778682728415
Bsdc1	Bsdc1	-0.0291696111157615	6.2118695178211	1.03184742322317	0.309725153399981	0.793778682728415
Ttc1	Ttc1	-0.0356068298687471	5.4180063974193	1.0316932268621	0.309761285460416	0.793778682728415
St6galnac5	St6galnac5	-0.0584352569688262	5.59088801447947	1.03126914589075	0.309860768119607	0.793778682728415
Angpt2	Angpt2	0.104203357787453	2.16253263289189	1.03119561668576	0.309878017059112	0.793778682728415
Pex1	Pex1	0.0303120641028525	6.1922176990931	1.03114507377578	0.309889874454605	0.793778682728415
Fancb	Fancb	-0.101800090977783	1.80882588246338	1.03081469512052	0.309967396016907	0.793814285735416
Sema4a	Sema4a	-0.0339472131110606	5.71459076282185	1.02991466118419	0.310178712144651	0.794052288807033
Gas2	Gas2	-0.078732611914041	2.16979919714916	1.02941500376531	0.310296106065259	0.794052288807033
Hsf2bp	Hsf2bp	0.159965692360355	0.178765908175741	1.02860924724716	0.310485528447155	0.794052288807033
Lhx1	Lhx1	-0.191382103962785	1.90228214227195	1.02806850754594	0.310612751790491	0.794052288807033
Det1	Det1	0.0674774800903756	2.69525507231043	1.0280579596707	0.310615223917736	0.794052288807033
Btg4	Btg4	0.180406034740748	-0.326490249343998	1.02776176703877	0.310684964553062	0.794052288807033
Pla2g12a	Pla2g12a	0.0452791495404782	4.54952434295074	1.02769809013901	0.310699932931345	0.794052288807033
Rplp0	Rplp0	-0.0507291813962776	10.263541452689	1.02746376722903	0.310755101067339	0.794052288807033
Tdrd5	Tdrd5	0.164955311521215	-0.096733967188117	1.02678507470308	0.310914858070136	0.794052288807033
Lcmt1	Lcmt1	-0.032780339596159	5.95852833857457	1.02677862995478	0.31091647756766	0.794052288807033
Rab2a	Rab2a	-0.0279943484966831	7.66480554866593	1.02671909000193	0.310930436008463	0.794052288807033
Gpr83	Gpr83	-0.127511150691174	1.66716165333723	1.02660306122347	0.310957848404732	0.794052288807033
Med23	Med23	0.0609039820378698	6.14023649752929	1.02645331856542	0.310993128277594	0.794052288807033
Micu3	Micu3	-0.037831968728927	5.18937151178871	1.02618552544259	0.311056262255937	0.794052288807033
Fam98a	Fam98a	0.0353679326263986	5.74991548088617	1.02616685482455	0.311060666672887	0.794052288807033
Scn5a	Scn5a	-0.202002825508096	3.33485436660446	1.0257917698367	0.311151471885584	0.794052288807033
Fuca1	Fuca1	0.0522332323672614	5.06651417864291	1.02566476263157	0.311179065503252	0.794052288807033
Slc2a8	Slc2a8	-0.0402995296659087	5.60563302358731	1.02548574594579	0.311221299522814	0.794052288807033
Coasy	Coasy	-0.0403794213489113	5.12865283240642	1.02528139198973	0.311269515472886	0.794052288807033
Qpct	Qpct	-0.0534480775219482	4.37792974470736	1.02435820525011	0.311487456553914	0.794209862251132
Fam151b	Fam151b	0.0463425719709578	4.30813922468895	1.02380885180478	0.311617229408759	0.794209862251132
Arf4	Arf4	0.0370290568161989	8.29203465915569	1.0236068378587	0.311664982228905	0.794209862251132
Vps18	Vps18	0.0295313434389464	5.69649032687015	1.02347838182043	0.311695345752816	0.794209862251132
Tmem45a	Tmem45a	0.171511634699422	0.0526487694080474	1.02341462694082	0.311710417132781	0.794209862251132
Tox4	Tox4	0.0233070783506922	6.61768366700855	1.02331570374458	0.311733380397201	0.794209862251132
Tac3	Tac3	-0.1294955159376	0.809752225694373	1.02313360017182	0.311776861850288	0.794209862251132
Sf3b4	Sf3b4	0.0274649356890937	6.38136777226534	1.02265455505196	0.311890167752518	0.794336318238043
Sorcs2	Sorcs2	-0.0652880340064875	6.30988600098916	1.02229467862942	0.311975322654653	0.794391040971859
Wdr18	Wdr18	-0.043555521707852	5.92259660224681	1.0218260428434	0.312086257928768	0.794511372225531
Shank3	Shank3	-0.0473238865282763	7.67183694751406	1.02113474767511	0.312249994967785	0.794720650772792
Fnip1	Fnip1	-0.0344382281627275	5.66894226689042	1.02094122295042	0.312295852427587	0.794720650772792
Cenpc	Cenpc	0.0370898115512451	5.3451502639437	1.02028230893744	0.312452054037919	0.794804272415186
Cpne8	Cpne8	-0.0676888909906124	4.37834019723867	1.01970236991227	0.312589618239316	0.794804272415186
Nup50	Nup50	0.0283584463696299	5.75779946946721	1.01880234557442	0.312803264689733	0.794804272415186
Cwh43	Cwh43	-0.22841359988025	-0.355782798300161	1.01859182707934	0.312853240976874	0.794804272415186
Lrrtm4	Lrrtm4	-0.0496403935256276	4.62234852675176	1.01843023179222	0.312891652159509	0.794804272415186
Ptpn7	Ptpn7	0.150049491307675	0.51847235777864	1.01840658772397	0.312897269382029	0.794804272415186
Apex1	Apex1	0.0698071980735375	4.0626959761305	1.01834722146283	0.312912443058052	0.794804272415186
Grpr	Grpr	-0.12687927702194	0.210715640655266	1.01830518849311	0.312921360776939	0.794804272415186
Myh8	Myh8	0.230684746892983	-0.312321755368696	1.01820580757291	0.312944951219271	0.794804272415186
Nlgn3	Nlgn3	0.0335978858903872	7.68967796432784	1.0181184753872	0.312965728169897	0.794804272415186
Dpp10	Dpp10	-0.0352305453928445	6.17915917350035	1.01768849289844	0.313067926656483	0.794902041755381
Pgk1	Pgk1	-0.0360454722353193	7.76262224466229	1.01711277708921	0.313204846851327	0.795087868598985
Gnl2	Gnl2	-0.0238004285087741	5.78622041685139	1.01615142985831	0.313432643465269	0.795387189687144
Errfi1	Errfi1	0.0436879705783306	4.78872092680409	1.01609534448766	0.313447045911025	0.795387189687144
Ubxn8	Ubxn8	-0.0452494472529047	3.57987877341445	1.01591706088139	0.313489455389139	0.795387189687144
Ypel2	Ypel2	0.0675519313746573	3.16275955982958	1.01587169325968	0.313500352979085	0.795387189687144
Pyurf	Pyurf	-0.0662683840632521	3.76033701898067	1.01538652018607	0.313615842322386	0.795387189687144
Sh3bgr	Sh3bgr	-0.173987868842477	-0.172553780501885	1.01529457206818	0.313638542313165	0.795387189687144
Hspa2	Hspa2	0.0742040896503692	3.26607152582947	1.01485792164545	0.313741825166652	0.795387189687144
Me3	Me3	-0.0709271966823376	3.82744240907286	1.01480760635025	0.313753823779938	0.795387189687144
Faf1	Faf1	0.0293029183677036	6.46111929485818	1.01444633063013	0.313839887134453	0.795210522507929
Dennd10	Dennd10	-0.0320055810159636	5.43449697496978	1.01423455165922	0.313890504396213	0.795210522507929
Il17rc	Il17rc	0.0957804903518918	1.64223080710084	1.01392636572312	0.313964037331707	0.795225341816426
Rps26	Rps26	-0.0472108711097885	7.72178582608835	1.0119245988158	0.314442205823891	0.796268352477756
Map1lc3b	Map1lc3b	-0.0273785213717881	7.87993948411675	1.01168495378244	0.314499514387303	0.796268352477756
Mprip	Mprip	0.0299194162207692	8.89491101292149	1.01136180425642	0.314576861625461	0.796302597408329
Metap2	Metap2	-0.0252700629270831	7.16401725710384	1.00954932001002	0.315010832182317	0.797200583281735
Cdc14a	Cdc14a	0.0794740930583738	2.48194416127656	1.00934672070267	0.315059395380479	0.797200583281735
Pfdn2	Pfdn2	-0.055907276103579	5.72082506365387	1.00843757568039	0.315277438675206	0.797226836882649
Arl4d	Arl4d	0.0686116673581411	4.40168302794971	1.00785237784868	0.315417893087836	0.797226836882649
Abcf2	Abcf2	-0.0222023967184721	6.80326043664213	1.00741135212616	0.315523798600102	0.797226836882649
Hemgn	Hemgn	-0.200598655891574	-0.611512810985407	1.00728790926105	0.315553449832631	0.797226836882649
Ebpl	Ebpl	-0.101604939188362	3.69578168946817	1.00673504277717	0.315686294266677	0.797226836882649
Txndc17	Txndc17	0.0671053098683091	4.8048538201477	1.00653849223833	0.315733529665046	0.797226836882649
Grk3	Grk3	-0.0686423199301119	4.11183406309734	1.00629325628493	0.315792500691996	0.797226836882649
Ppef1	Ppef1	0.149746474742622	0.235049153163911	1.00613606925057	0.315830300076489	0.797226836882649
Lmna	Lmna	-0.0361619096723016	5.64139620458804	1.00583882353728	0.315901772060324	0.797226836882649
Col5a2	Col5a2	0.0807237066275147	5.78852551642397	1.00574814814037	0.315923610344457	0.797226836882649
RGD15663	RGD15663	0.0486456705090778	4.26887230729865	1.00524159915057	0.316045509629427	0.797226836882649
Ednra	Ednra	0.0799090080945257	3.88031258061871	1.00513189053921	0.316071894672977	0.797226836882649
Glb1	Glb1	-0.0555438915208641	4.4504329482612	1.00500364925942	0.316102792721001	0.797226836882649
Elf4	Elf4	0.200314309673849	-0.456598235224899	1.00496233561244	0.316112499011778	0.797226836882649
Morc2	Morc2	0.0652161466240441	6.59655880497224	1.00488194681533	0.316132096129496	0.797226836882649
Gnal	Gnal	-0.125068552553123	7.4305852908552	1.00461370018827	0.316196696882979	0.797226836882649
RGD155874	RGD155874	-0.0601011199374626	3.5243958834095	1.00450885808678	0.316221950280039	0.797226836882649
Padi3	Padi3	-0.162155976132732	0.671808891636564	1.00436859287586	0.316255740206673	0.797226836882649
Zfp2	Zfp2	0.0386025596710239	4.90295487760164	1.00355684528549	0.316451383319526	0.797226836882649
Psmd9	Psmd9	-0.0436715262674405	4.90345529459756	1.00322216887091	0.316532091421852	0.797226836882649
Ankrd39	Ankrd39	-0.0682703992023771	2.70113088921846	1.00311922693657	0.316556821564948	0.797226836882649
Prickle1	Prickle1	0.0652531918872968	5.88130339967173	1.00310300001757	0.316560836581713	0.797226836882649
Calcr	Calcr	-0.248855956916309	4.4396314142545	1.0027082895253	0.316656040746973	0.797226836882649
Prnp	Prnp	-0.0326897652562351	8.1409653331012	1.00270710248458	0.316656353744762	0.797226836882649
Cnih1	Cnih1	-0.0439646271979955	5.98834263014567	1.00230330318326	0.316753817034477	0.797226836882649
Itfg1	Itfg1	-0.0264571808722245	8.55928404206066	1.00202661507076	0.316820622750282	0.797226836882649
Tor1aip1	Tor1aip1	0.0476316030378259	3.99711512880402	1.00187258680069	0.316857820526254	0.797226836882649
Traf1	Traf1	0.124580205196772	0.515512106453648	1.00163579723004	0.316915016284038	0.797226836882649
Ift20	Ift20	0.0375233118923551	5.25057777156323	1.00160379763262	0.316922746732982	0.797226836882649
Higd1a	Higd1a	-0.0450928660745886	5.44521852905126	1.00109312640829	0.317046147763419	0.797308773719753
Prelid2	Prelid2	0.202275725484819	-0.337834936222575	1.00041894708946	0.317209156162643	0.797308773719753
Med25	Med25	-0.0287967665670927	7.25505358915419	1.00035223531331	0.317225292236908	0.797308773719753
Prrg1	Prrg1	0.0942490899151892	1.76413517106238	1.00025812179799	0.317248055585182	0.797308773719753
Slfn4	Slfn4	-0.264527182157729	-0.835861811960544	0.999885986898909	0.317338094412544	0.797308773719753
Fmo3	Fmo3	0.281687680908166	-0.067244969131615	0.999712092120589	0.317380183171125	0.797308773719753
Kitlg	Kitlg	-0.103710572068953	7.74868634908515	0.999573057994795	0.317413827387099	0.797308773719753
Pin1	Pin1	-0.0457454464242684	6.51102700190489	0.998210830648536	0.317501529120665	0.797308773719753
Mfsd8	Mfsd8	0.0549817196239881	3.40559538085632	0.999091498522833	0.317520439738185	0.797308773719753
Phf12	Phf12	0.0623682799430235	6.25721447201777	0.998079578879853	0.317775640177767	0.797408641186672
Camk4	Camk4	-0.0659245207099	5.21915904092888	0.997988023221723	0.317797827589799	0.797408641186672
Sdad1	Sdad1	0.060583703806641	4.16898911582277	0.997932548579428	0.317811288250712	0.797408641186672
Uba7	Uba7	0.144880952715368	0.704999910595751	0.997872481002645	0.317825853379925	0.797408641186672
Cmtm3	Cmtm3	0.076487379526065	3.95218410639323	0.997048892225408	0.318025644772697	0.797437774477801
Cited4	Cited4	-0.140727887355937	2.86384578967463	0.996970398801012	0.318044694989241	0.797437774477801
Rassf7	Rassf7	0.0664865671607462	2.71030426176734	0.996773689701563	0.318092432945726	0.797437774477801
Mapk8ip1	Mapk8ip1	0.0384878537719266	5.50278178532786	0.996717153644127	0.318106166658739	0.797437774477801
Mgat5b	Mgat5b	-0.0261737210952166	7.46687398188132	0.996507667558852	0.318157028240001	0.797437774477801
Pkn3	Pkn3	0.0280854631830008	7.15037947552372	0.99621714546187	0.318227582497264	0.797454417918933
Ogdh	Ogdh	-0.0287437617935475	7.75181198887222	0.995510096398526	0.318399377552414	0.797712205690458
Klhdc9	Klhdc9	-0.0535306458998838	3.5082425186639	0.99503241792452	0.318515510274104	0.797712205690458
Cpm	Cpm	0.0840042352882893	3.31194284259292	0.995004671551484	0.318522257654072	0.797712205690458
Psmc6	Psmc6	-0.0318268561724606	6.86695423938672	0.994480869468141	0.318649671380288	0.797871151903797
Zbtb16	Zbtb16	0.087512687016861	3.69416851901921	0.993773440700366	0.318821858191765	0.798028390600717
Ttc23	Ttc23	0.107721125962778	1.79510220224332	0.99309747023195	0.318840356523589	0.798028390600717
Snf9	Snf9	0.0551344788737368	4.49873608800616	0.99325085732346	0.318949132472426	0.798140459455367
Capn3	Capn3	-0.170189643353905	-0.500012492562996	0.991673271995532	0.319322373141337	0.798942644242959
Ndufs2	Ndufs2	-0.0358470965889067	7.52679844072219	0.990370760472589	0.319651774279092	0.799657824786555
Atf6	Atf6	0.0481390546318072	4.7444459251799	0.988730015937826	0.320052967884616	0.800357442580417
Il17rd	Il17rd	-0.112756289804012	2.19032554535187	0.988571922566521	0.320091659736009	0.800357442580417
Pnldc1	Pnldc1	0.143808778809135	0.943296253206829	0.988188250098915	0.320185595492805	0.800431886743613
Xkr7	Xkr7	0.0430893964044068	4.69980328988797	0.987267898520628	0.320411061974523	0.800518423744625
Zbtb40	Zbtb40	0.100549482833528	4.10879679962348	0.98726158944261	0.320412598216055	0.800518423744625
Pja1	Pja1	-0.0269288955739947	8.36091815541161	0.987175010455609	0.320423808160253	0.800518423744625
Zap70	Zap70	0.0706719309901503	2.44504889307693	0.986893211454907	0.320502863543161	0.800518423744625
Tgif1	Tgif1	-0.0947727779066158	1.30391755776153	0.986282556153232	0.320652405374535	0.800518423744625
Gda	Gda	-0.0958556640105671	5.95998849936585	0.986233049499273	0.320664796440157	0.800518423744625
Spint1	Spint1	-0.111472388648103	2.7243797920587	0.986034549454114	0.320713500652172	0.800518423744625
Slc32a1	Slc32a1	-0.0982936271562677	7.06677606502991	0.985953141311939	0.320733477622776	0.800518423744625
Galnt18	Galnt18	-0.0510798850677126	5.05544788224933	0.985321378482243	0.320886109651483	0.800739188024309
Pold4	Pold4	0.0799431532511556	1.62998770718754	0.984789000064492	0.321019332227655	0.800811459349022
Ndufs5	Ndufs5	-0.0487920175796786	5.42415104120793	0.983579184201588	0.321316757879489	0.801493240216349
Tp53inp1	Tp53inp1	0.0799563038275434	2.03625016778789	0.983083366310858	0.321438756428833	0.801637290418258
Spry1	Spry1	0.0541907094884368	3.07259451823198	0.982692412137055	0.321534995747296	0.801637517901865
Ciao1	Ciao1	-0.0452064760228967	5.06178982204651	0.982561029578662	0.321567346084702	0.801637517901865
Six2	Six2	0.271611376207882	0.254848475784875	0.981075614575829	0.321933398458282	0.802290339102589
Lmbr1	Lmbr1	-0.0287111173572521	6.05682716005471	0.980976580352434	0.321957820620875	0.802290339102589
Anks1a	Anks1a	0.0241236028308402	6.73328235930765	0.980659633610645	0.322036254219333	0.802325547903228
Gcfc2	Gcfc2	0.0749741860537133	3.52386388616917	0.98020631143261	0.322147876503166	0.802443412882811
Kcns3	Kcns3	0.101454859450574	1.86240445652349	0.979662808670653	0.322282067446588	0.802617436233749
Pfn1	Pfn1	0.0395462287883513	7.86729598353092	0.978789616818867	0.322497813060551	0.802994454930495
Sparc	Sparc	0.0683090076434069	8.60284698559634	0.978336922833924	0.322609738026647	0.803112986938517
Orc1	Orc1	0.102319738790631	1.15820255304569	0.977888863020929	0.322720568228155	0.803228511825792
Fam136a	Fam136a	0.0382712120892892	6.14000919179463	0.977434371337743	0.322833040432419	0.803348184151434
Fbxo27	Fbxo27	-0.0492158068936758	3.82371120284826	0.97643146876692	0.323081409878937	0.803587625300198
Stk4	Stk4	0.0360530066110422	6.01492920483073	0.975306576073116	0.323360290394883	0.803587625300198
Slc10a1	Slc10a1	-0.197012021143853	0.159630466257201	0.975288944896874	0.323364603989519	0.803587625300198
Adam17	Adam17	0.0494549956271562	4.44195320165017	0.975284980775946	0.323365647353494	0.803587625300198
LOC500124	LOC500124	-0.105825845027143	1.93400277098071	0.975231912076954	0.323378811896323	0.803587625300198
Tmem178a	Tmem178a	-0.0482172943968866	5.06618245928209	0.975099742204733	0.323411602686626	0.803587625300198
Nmnat3	Nmnat3	-0.133909718688637	0.768676229424967	0.974751702068062	0.323497970366854	0.803587625300198
Tmem110	Tmem110	0.0375764780865439	4.60313348648388	0.974529005034668	0.323553249578508	0.803587625300198
Syn3	Syn3	0.0490216366182678	6.29107828901894	0.974469032142213	0.323568138297637	0.803587625300198
Pomgnt2	Pomgnt2	-0.04026163864724	5.26214240299421	0.974448189214279	0.323573313257048	0.803587625300198
Mrpl4	Mrpl4	-0.0301624426882782	6.04929971665957	0.972926580711801	0.323951378628214	0.804366438038652
Vgll4	Vgll4	0.0395181268570932	4.64176731880202	0.972430529708738	0.324074758985216	0.804512687596209
Ptcd1	Ptcd1	-0.0350001114677195	4.60295475333764	0.972011531584013	0.324179024527485	0.804611436051209
Wdsub1	Wdsub1	-0.0492158622719896	3.45748356878008	0.971527061492502	0.324299662926416	0.804647907470133
Pik3ap1	Pik3ap1	0.120874636890356	1.54018186584669	0.971271359580021	0.3243633100684	0.804647907470133
Tmcc2	Tmcc2	-0.0338676579581416	8.01834920967887	0.971175488338105	0.324387190396311	0.804647907470133
Ttc5	Ttc5	0.0301522044871892	6.36330134271618	0.970406451891549	0.324579831895831	0.804809231237216
Ctsh	Ctsh	-0.0685761283644825	4.4732190645532	0.970396817935921	0.324581223586672	0.804809231237216
Ubqln2	Ubqln2	0.0305651165478625	8.3461515304548	0.969940309807056	0.324694385015416	0.804822686815176
Mphosph9	Mphosph9	0.0740464750652722	3.5819441635879	0.969695951014614	0.324756019537357	0.804822686815176
Macroh2a2	Macroh2a2	0.0259591977555861	7.14269864451667	0.968888309420237	0.324957587871523	0.804952234085143
Dnajc2	Dnajc2	-0.0322533204183432	6.12454009267595	0.968721386384573	0.324999268560539	0.804952234085143
D2hgdh	D2hgdh	-0.0468235126177876	3.6840147587118	0.968587487038022	0.325032708343445	0.804952234085143
Robo3	Robo3	-0.199068131287288	1.43121910296637	0.96852103291296	0.325049306167147	0.804952234085143
Prmt7	Prmt7	0.0295646853023705	5.61913920733222	0.96776717750997	0.325237670342982	0.804952234085143
Nudt15	Nudt15	-0.112968094923158	0.643567437281069	0.967431331329031	0.325321623974138	0.804952234085143
Smox	Smox	0.0385290937215171	4.69486721800126	0.967094975182029	0.325405753843376	0.804952234085143
Ryr2	Ryr2	0.0745204463668065	6.49397211512183	0.966189751960854	0.325632285625472	0.804952234085143
Cdh1	Cdh1	0.151411800035586	1.96709865258386	0.966185340432261	0.325633390119792	0.804952234085143
Atp5f1b	Atp5f1b	-0.0382598107063466	10.2426289636662	0.965978300613116	0.325685231313115	0.804952234085143
Gpr4	Gpr4	0.100780463621713	2.08595662205867	0.965701322797528	0.325754601529668	0.804952234085143
Abat	Abat	-0.0927994540536396	5.65403348456245	0.965683155190817	0.325759152364122	0.804952234085143
Ppp2r5b	Ppp2r5b	-0.0245452546709862	7.88818553808052	0.965626066781226	0.325773453082901	0.804952234085143
Haus8	Haus8	-0.0676443914013805	3.35461522518831	0.965532269889367	0.325796951377262	0.804952234085143
Them6	Them6	0.0631235342328121	3.77996865985723	0.965223642734713	0.325874284383662	0.804952234085143
Asb4	Asb4	-0.268285151498838	2.63448798257305	0.964550324604716	0.326043083285575	0.804952234085143
Tkt	Tkt	0.0272670542506408	8.32231326217276	0.964317729008171	0.326101421162483	0.804952234085143
Gpr162	Gpr162	-0.0262857012674546	7.72411798412631	0.964046604118875	0.326169942088106	0.804952234085143

Stard10	Stard10	-0.0641578726704105	3.90810532319132	0.964041981930137	0.326170600028275	0.804952346951 43
Crebbp	Crebbp	0.0690132059944502	6.52514307286534	0.964022391657736	0.326175515529901	0.80495234695143
Manbal	Manbal	-0.04374875229032	5.39018445956991	0.963887442840871	0.326209378935737	0.80495234695143
Zfyve27	Zfyve27	-0.0315056834250606	7.016661551127	0.963292672721813	0.326358693175321	0.80495234695143
Bbs7	Bbs7	0.0532667293748285	3.73445885212131	0.963110392996438	0.326404308029849	0.80495234695143
Kctd10	Kctd10	0.0267341434374917	6.77389839916218	0.962811372822564	0.326479569418111	0.80495234695143
Tyrnd1	Tyrnd1	0.037809290496632	5.34616185883758	0.962669276184991	0.326515244964682	0.80495234695143
Numa1	Numa1	0.0634730779157878	7.63246841971654	0.962224553456124	0.326627038838085	0.80495234695143
Ppp1r14	Ppp1r14c	-0.0518845120831177	6.62259758329114	0.962012147507608	0.326680438824679	0.80495234695143
Hdac2	Hdac2	0.0292871403488668	8.14679013276767	0.962005660267593	0.326682089693239	0.80495234695143
Slc25a16	Slc25a16	0.0862910139645373	4.68579195279883	0.961876917404538	0.326714462419216	0.80495234695143
Lhx9	Lhx9	-0.18422537978847	3.85692142973681	0.96177420957244	0.326740271425824	0.80495234695143
Pard3b	Pard3b	0.12019886261217	1.84031962222221	0.961664182810267	0.326767964001682	0.80495234695143
Pglyrp1	Pglyrp1	-0.160320936062976	0.118892273123591	0.96130868624023	0.326857375349741	0.805013713430461
Pacc1	Pacc1	0.0603963206338833	5.06115930175115	0.960395796571177	0.327087177276152	0.805238935171389
Mmp24os	Mmp24os	0.0777902053268458	2.99851046256312	0.960344401261182	0.327100121395947	0.805238935171389
Cdc42ep	Cdc42ep2	0.0519279447164812	3.85359770436008	0.960069105191785	0.327169467402045	0.805238935171389
Atxn1	Atxn1	0.0584751246041091	5.75913929185841	0.959920244548982	0.327206872935717	0.805238935171389
Hcfc2	Hcfc2	0.0392607575266703	4.65596693331915	0.957977351402903	0.327697009284389	0.80596617644713
Zfp819	Zfp819	0.106584841096551	0.990977120002772	0.957863505736462	0.327725753606689	0.80596617644713
Trip12	Trip12	0.0391005958553424	8.08222044665559	0.957783461819162	0.327745865497043	0.80596617644713
Otog	Otog	-0.193822422128834	-0.348716420616735	0.957610906718472	0.327789543012102	0.80596617644713
RGD156	RGD156002	-0.225597905582649	-0.419145380887417	0.957469682380437	0.327825466495887	0.80596617644713
Cherp	Cherp	0.036481325773358	6.86687124206407	0.957121612507808	0.327910625555566	0.805994538426769
Txn2	Txn2	-0.042311900422434	6.04055848008254	0.956674782612883	0.328026087999355	0.805994538426769
Spata5	Spata5	0.0624555195288957	4.12822119791741	0.956405282865146	0.328094226207536	0.805994538426769
Abca17	Abca17	0.163545589738396	-0.029778900357496	0.955951884866202	0.328208809237138	0.805994538426769
Usp48	Usp48	0.0458432422240721	6.72665576864554	0.955942082014676	0.328211389056416	0.805994538426769
Ppil2	Ppil2	-0.0318825728482456	5.29031855600446	0.955889880220679	0.328224593231259	0.805994538426769
Cntn4	Cntn4	-0.0517426710456726	5.40581291229798	0.954574795388055	0.328557549809797	0.80665328413161
Ldb3	Ldb3	0.125355895291961	0.336467341484191	0.953297034261446	0.328881483137593	0.807021580059981
Tmem14	Tmem14c	-0.0448003399587106	5.27983320954205	0.953105898248943	0.328929975703398	0.807021580059981
Tmem14	Tmem14a	-0.045749486598836	4.36984459285379	0.952485398829385	0.329087466046535	0.807021580059981
Wbp1l	Wbp1l	0.0276246428071979	6.11030257643235	0.952435268098689	0.329100194485579	0.807021580059981
Igbp1b	Igbp1b	-0.112774644501344	1.47856665212529	0.952327347642623	0.329127597696236	0.807021580059981
B3gat1	B3gat1	0.0377012966784362	8.17287499953279	0.952219733080113	0.329154926251926	0.807021580059981
Pcp4l1	Pcp4l1	-0.182773838168895	4.00045409069025	0.95212504277977	0.329178975204262	0.807021580059981
Cttnbp2	Cttnbp2	0.0673534137215416	6.95603604316994	0.951901611314042	0.329235730425438	0.807021580059981
Rnpep	Rnpep	0.0242856199433457	6.81494487589629	0.951673364002033	0.329293722376001	0.807021580059981
Slc39a13	Slc39a13	0.0432326782203578	4.56745884302335	0.951341488958571	0.329378067767706	0.807079762674326
Otx1	Otx1	0.0713762705508752	3.49727292655855	0.950543102244495	0.329581093837817	0.80718155016875
Rfc5	Rfc5	-0.0411643811432315	4.87051712459762	0.950412539030492	0.32961405685269	0.80718155016875
Tyro3	Tyro3	-0.0349045838190225	6.80951545512599	0.949526670149027	0.329839808614824	0.80718155016875
Zfx	Zfx	0.0407820857077384	5.48561799932898	0.949523492376784	0.329840617883743	0.80718155016875
Retreg3	Retreg3	0.024366957699411	6.2153440347192	0.949332186948944	0.329889341637785	0.80718155016875
Syk	Syk	0.0782060364506143	2.28994132782141	0.94929215385207	0.329899528914638	0.80718155016875
Nmrk1	Nmrk1	0.106943285372135	1.29819181727852	0.949034548971916	0.329965166355667	0.80718155016875
Brd4	Brd4	0.0465461080111758	8.00368215140939	0.949009530831702	0.329971540904042	0.80718155016875
Col4a3	Col4a3	-0.157915495701104	0.759244615297845	0.94889058419173	0.330001850397585	0.80718155016875
Sil2	Sil2	0.0971221619814934	6.2548884714217	0.948237485201872	0.330168339639038	0.807420330873655
Ccdc82	Ccdc82	0.0511113114412879	4.04048515494532	0.947788320314963	0.330282900968365	0.807420330873655
Pde6d	Pde6d	-0.0267108448193832	5.6935180553966	0.94774634579931	0.330293692974426	0.807420330873655
mrpl9	mrpl9	-0.0389659112292246	5.57735697341423	0.947104980267654	0.330457293461609	0.807528426065474
Gpn2	Gpn2	-0.0430557953627733	3.96669306609345	0.947065908323417	0.330467271258934	0.807528426065474
Mrps36	Mrps36	-0.0561547275970803	4.62142857291807	0.946417211878284	0.330632964952946	0.807631541004908
Dnlz	Dnlz	-0.0509697967173821	5.01712652989942	0.946393791178579	0.330638928544768	0.807631541004908
Cnn3	Cnn3	0.0388197145699318	7.71129998652767	0.945843903578208	0.330779456930356	0.807816653771785
Sh3bp5	Sh3bp5	0.0286669378812157	7.67074812590847	0.945302306798469	0.330917964247057	0.807996710444332
Pdgfrl	Pdgfrl	0.164089166310079	0.717521643466296	0.944969954947674	0.33100373304098	0.808048048748251
Fxd1	Fxd1	-0.0284472346960666	4.96056320588855	0.944524585915624	0.331116961415007	0.808103091155673
Ppm1n	Ppm1n	0.146311693220922	0.544492948475331	0.943879854631 39	0.331282035738135	0.808103091155673
Slc35e4	Slc35e4	0.0547859637418 54	5.22915211932711	0.942972189603907	0.331514640939262	0.808103091155673
Tpi1	Tpi1	-0.0450243120620135	8.28432975342273	0.942918372573573	0.331528428327946	0.808103091155673
Slc43a3	Slc43a3	-0.083482196686571	2.34144966172988	0.942809065670581	0.331556467381472	0.808103091155673
Ints11	Ints11	0.0351962349807272	5.57357799482 42	0.942634676848742	0.331601189999254	0.808103091155673
Dnajb13	Dnajb13	-0.0885317551791306	1.66404037583366	0.942478980080416	0.331641125814731	0.808103091155673
Prcp	Prcp	0.0497935858426781	4.40370588896477	0.942361949748822	0.331671147989157	0.808103091155673
Hrc	Hrc	0.153098259400232	0.941800683803292	0.942353838897626	0.331673222880639	0.808103091155673
Zfp40	Zfp40	0.0572990336381074	5.16295813297819	0.942351010478816	0.331673954460987	0.808103091155673
Ndufc2	Ndufc2	-0.0491557359084953	5.99185315726367	0.940986617788539	0.332024237916905	0.808798597518396
Atg4c	Atg4c	-0.0352097684775021	4.76545163228268	0.939889978403 08	0.332306128001052	0.808867564521259
Ptprh	Ptprh	0.118161466606796	1.74190470985122	0.939227405844649	0.332476612303884	0.808867564521259
Smpd2	Smpd2	0.0533106137568366	2.8953912456383	0.939047778720386	0.332522846966193	0.808867564521259
Phf23	Phf23	0.0446367400931201	5.48058497371212	0.938719775357427	0.332607300283107	0.808867564521259
Rpl19	Rpl19	-0.0417584260014306	8.7872194535234	0.938602292615876	0.332637555626763	0.808867564521259
Ppib	Ppib	-0.0354336219889756	7.20564728731494	0.938426906640657	0.332682729627678	0.808867564521259
Ndufa1	Ndufa1	-0.0761358067043775	5.04977060782451	0.938333824698148	0.332706707980187	0.808867564521259
Rrp15	Rrp15	0.0379473628858841	5.11079999158737	0.938163487697814	0.332750593573985	0.808867564521259
Gtf2i	Gtf2i	0.0365827448366499	8.32690014849773	0.937830604254536	0.332836379897966	0.808867564521259
Raph1	Raph1	0.0650250449424576	6.2941934936549	0.93771782 91912	0.332865446488741	0.808867564521259
Slco3a1	Slco3a1	0.0497513598505555	4.83768928001071	0.937575667475668	0.332901840970517	0.808867564521259
Masp1	Masp1	0.0407709478346883	6.62291694789455	0.937283434968677	0.332977453261 46	0.808867564521259
Stk25	Stk25	-0.0232177244738671	7.75096295482887	0.937222438152673	0.332993018464565	0.808867564521259
Mlh3	Mlh3	0.0434589024649048	4.8098824426181	0.936788999156349	0.333104999300136	0.808867564521259
Hist1h4b	Hist1h4b	0.0795994160329 35	2.64103299042217	0.936710085387475	0.333125362148545	0.808867564521259
Wdr3	Wdr3	0.0347515169853611	5.56647788307107	0.936472237908681	0.333186746179682	0.808867564521259
Tcea2	Tcea2	-0.0380751832909292	5.95803616448257	0.936387112515717	0.333208719135958	0.808867564521259
Tyms	Tyms	0.0336247830217552	5.0813896131311	0.935918189365851	0.333329791822985	0.808867564521259
Ext1	Ext1	0.0309779036704844	5.63027757057053	0.935894002064828	0.333336041123755	0.808867564521259
Ang	Ang	0.09625661 2949423	2.47094217337719	0.935467468174437	0.333446225313185	0.808867564521259
Zic1	Zic1	-0.119917893336025	6.07285848852433	0.935406702490766	0.333461926539763	0.808867564521259
Itih4	Itih4	0.218851330492147	-0.344230911821899	0.935341477479469	0.333478781109029	0.808867564521259
Zfp438	Zfp438	0.0949628279139645	1.49204937531438	0.934942499033781	0.333581904509012	0.808960431984246
Stradb	Stradb	0.0249394787395805	6.44462990507455	0.934535089805889	0.333687251138428	0.809058653799878
Naa10	Naa10	0.0354034055220736	4.68394157989566	0.933893538658637	0.333853231053881	0.80914709054736
Slc7a6	Slc7a6	-0.0437518222487004	5.71255773449834	0.933892777365756	0.333853429807889	0.80914709054736
Strap	Strap	-0.0281928288932365	7.52241830689345	0.932981459335221	0.334115297330623	0.809276885421199
Wnk1	Wnk1	0.0691429229778069	6.969784873052	0.932719251885729	0.334157324630337	0.809276885421199
Fancl	Fancl	-0.0671115190836796	2.69607429184949	0.932278455774352	0.334271568819 48	0.809276885421199
L3hypdh	L3hypdh	0.0632327375096183	2.66786774185181	0.932122339660016	0.334288709041148	0.809276885421199
Kif15	Kif15	0.0772476564979787	3.62195254626457	0.931728821580094	0.334414094458132	0.809276885421199
Tnik	Tnik	0.0457968974573066	7.22185129262663	0.931478866348627	0.334478937213 07	0.809276885421199
Ccnd2	Ccnd2	0.0733869515479168	5.48690248706364	0.931410653643752	0.334496633569943	0.809276885421199
Pdcd6	Pdcd6	-0.04024061 0567552	5.32192713839314	0.930876728962966	0.334635211193006	0.809276885421199
RGD155	RGD155990	-0.0526787211883882	6.10514787820417	0.930750280329889	0.334668043072339	0.809276885421199
Zfp384	Zfp384	0.0480325590 90154	6.11303731055914	0.930563051707395	0.334716659178256	0.809276885421199
Kif1b	Kif1b	0.0452543232544424	9.55354001257865	0.93024656444115 4	0.334798842885835	0.809276885421199
Ccar1	Ccar1	0.0427475782274493	6.80523409988835	0.929707954880598	0.334938824317606	0.809276885421199
Otos	Otos	-0.215662141328737	-0.621225971650425	0.929358008382039	0.335029801775 52	0.809276885421199
Gabre	Gabre	-0.206291431542931	1.09544726076272	0.929224532634677	0.335064510884372	0.809276885421199
B3galt4	B3galt4	0.0716466294866179	2.58069455816043	0.929105747555238	0.335095143777766	0.809276885421199
Rps18	Rps18	-0.050192932720433	8.3664573012118	0.929004898093638	0.335121625243649	0.809276885421199
Pdxdc1	Pdxdc1	0.0520355410600709	4.91398501190626	0.929004371048407	0.335121772337577	0.809276885421199
Usp30	Usp30	-0.0226198552785781	6.08209146062826	0.928596189922907	0.335227967526551	0.809276885421199
Pabpc5	Pabpc5	0.119553930492367	0.512771841017434	0.928355300567318	0.335290664176673	0.809276885421199
Cavin3	Cavin3	0.0746149768099888	4.31120811342894	0.928286808787192	0.335308492769385	0.809276885421199
Ankfy1	Ankfy1	0.0445849975454369	7.54304651169221	0.928218700291282	0.335326222849882	0.809276885421199
Eps15l1	Eps15l1	0.0289988538291117	6.24528393247254	0.92818907915369	0.335333894249227	0.809276885421199
Cd47	Cd47	-0.0321780488691226	6.86552803230547	0.927812025182831	0.335432115082139	0.809357280966901
Rassf5	Rassf5	-0.0706139551901256	3.80193473509011	0.927472269557967	0.335520616770441	0.809414295329619
Rps19l2	Rps19l2	-0.0464029662697667	8.54006656551234	0.927097720066252	0.335617896732975	0.809491977988803
Kin	Kin	0.0704824640150262	2.99665204806114	0.926405277408522	0.335798756867625	0.809525488126936
Wasf1	Wasf1	0.0294493781537466	8.61458006983484	0.926016547176829	0.335900166520888	0.809525488126936
Cnppd1	Cnppd1	0.0322421444279605	5.64098400843389	0.92597581212219	0.335910769521311	0.809525488126936
Sub1	Sub1	-0.0342941575906181	7.21631132797402	0.925792647642751	0.335958594721034	0.809525488126936
Afdn	Afdn	0.0436488327310389	7.84863290797356	0.92555504078851	0.336020614828518	0.809525488126936
Rbm12	Rbm12	0.0304935019686337	6.09413592959058	0.925554030151901	0.336020878657482	0.809525488126936
Mylip	Mylip	-0.060726624107212	3.9008916915479	0.924622144971465	0.336264267478624	0.809827008180907
Man1a2	Man1a2	0.0342840280462919	6.03488977126206	0.924265608225667	0.336357446827501	0.809827008180907
Cdpf1	Cdpf1	0.087086129360531	2.73201238622844	0.924193470224722	0.336376307588272	0.809827008180907
Gadd45a	Gadd45a	-0.0761087593575151	2.56883531698891	0.923850851556473	0.336465891719052	0.809827008180907
Fem1a	Fem1a	0.0325569858536115	6.0531035423055	0.923817103508508	0.336474717512 01	0.809827008180907
Elac2	Elac2	0.0293809652767014	5.68366464452853	0.923045911405339	0.336676484313934	0.810003071823008
Wasl	Wasl	0.0323503492974466	5.90405722621558	0.922854087777324	0.336726896257804	0.810003071823008
Wfdc1	Wfdc1	0.170829288981091	1.42868363250116	0.922809101073294	0.336738473474889	0.810003071823008
Ext2	Ext2	0.0300467265232531	6.02394032811924	0.922365994701693	0.336854505248774	0.810079117 60747
Cplane2	Cplane2	0.109646731617775	0.000818812902933	0.921896284527485	0.336977562005052	0.81007911760747
Lrch1	Lrch1	0.0506995334012457	4.08243756502782	0.921706024730391	0.337027424257682	0.81007911760747
Lym7	Lym7	-0.0525201879855154	4.01272726332556	0.921454757111896	0.337093290265209	0.81007911760747
Nubpl	Nubpl	0.063323736068345	2.57224034545863	0.921258405095223	0.337144772989765	0.81007911760747
Fdx1	Fdx1	0.0636775848 47339	3.14444762431513	0.921201701688341	0.337159642360791	0.81007911760747
Sfxn3	Sfxn3	-0.0292852433988231	7.43375774698012	0.920130791530646	0.337440633026349	0.810411779612984
Tdp1	Tdp1	0.0547956206789 22	3.43517874152127	0.919889576649823	0.337503967501143	0.810411779612984
Ppargc1	Ppargc1a	-0.0634329721247984	3.93735474419887	0.919557313075146	0.33759123424 37	0.810411779612984
Myo18a	Myo18a	0.0268229074458823	8.31909941782783	0.91952572438111	0.337599522372317	0.810411779612984
Npas2	Npas2	0.0872730790678252	5.47167842908 56	0.919436926201641	0.337622860497579	0.810411779612984
Opa3	Opa3	-0.0377665465722206	5.25494492870491	0.919070461793019	0.337719157058347	0.810487001849777
Tubg2	Tubg2	-0.0378949670670785	4.57587042804535	0.918508064416038	0.337867010981545	0.810614294099411
Tsks	Tsks	0.16212956809335	-0.0877907200389 87	0.918374508850761	0.337902135418052	0.810614294099411
Zbtb7a	Zbtb7a	0.0468007651511312	4.4129421081963	0.916961988439482	0.338273921286196	0.811224837680952
Rpl26	Rpl26	-0.0359835508851086	8.30023791668441	0.916913574515583	0.338286673920496	0.811224837680952
Fads3	Fads3	-0.0336483126380911	5.80855220330649	0.916362355496794	0.338431915158926	0.811363240037713
Rnase4	Rnase4	-0.162193912029175	1.17198248698373	0.916201002216695	0.338474446132587	0.811363240037713
Arnt	Arnt	0.0796858721119 37	4.57579068140243	0.91506365625726	0.338774441881951	0.811580553685461
Hps3	Hps3	0.0371614901147227	4.79778411469086	0.914806202298388	0.338842399624062	0.811580553685461
Stxbp	Stxbp	-0.0341068568482375	7.42852534682295	0.914419399693351	0.338944534761538	0.811580553685461
Usp8	Usp8	0.0411294278498077	5.7680013605336	0.914178448833127	0.339008179686573	0.811580553685461
Sardh	Sardh	0.0745705595202854	3.02626222441375	0.91398867429713	0.339058316377908	0.811580553685461
Park7	Park7	-0.0437742395779733	6.82559946488408	0.91331720775672	0.339235795230546	0.811580553685461
Usp38	Usp38	0.0351360880184414	5.16600082106893	0.912782943956266	0.339377098523209	0.811580553685461
Rpl28	Rpl28	-0.0421578504447323	8.15241608778495	0.912075471157578	0.339564334243989	0.811580553685461
Pycr1	Pycr1	0.0552756515993281	3.91325717851 83	0.912067402144061	0.339566470544209	0.811580553685461
Rpsa	Rpsa	-0.0442914898570145	10.0399918396511	0.911968231607504	0.339592701296396	0.811580553685461
Mgrn1	Mgrn1	-0.0215481158634803	7.82299614102339	0.911957978849259	0.339595442537335	0.811580553685461
Riok3	Riok3	-0.0252931147177007	6.73016198146931	0.911880495852182	0.339615859684974	0.811580553685461
Wfikkn1	Wfikkn1	0.0865888908623829	1.58792636990963	0.911688927112358	0.339666693468068	0.811580553685461
Gca	Gca	0.0611503397839887	3.62573160418444	0.911653646629858	0.339676038021223	0.811580553685461
Zc2hc1a	Zc2hc1a	0.026241421765477	6.36068365388877	0.911526180466893	0.339709802184271	0.811580553685461
Foxp2	Foxp2	-0.104806912884153	6.29811087882538	0.911372962366315	0.339750393680358	0.811580553685461
Marcksl1	Marcksl1	0.0471909990651435	9.39725464888163	0.911246941320144	0.339783784851288	0.811580553685461
Ttc7b	Ttc7b	-0.0278483548284981	5.85791584450244	0.910850410741389	0.339888880384383	0.811580553685461
Nat8l	Nat8l	0.0278184804751816	6.42788589354078	0.910832981793632	0.339893500714664	0.811580553685461
LOC362	LOC362863	-0.0914685888108071	1.46497951309252	0.910615304891849	0.339951212931246	0.811580553685461
Phf1	Phf1	-0.0505826093414177	5.47046683634108	0.910602342606424	0.339954650010332	0.811580553685461
Pwp2	Pwp2	0.0276135605764481	5.99729895032558	0.910445970975701	0.339996117188922	0.811580553685461
Cdkn2c	Cdkn2c	-0.0728024453259666	2.98189464494752	0.907698441298798	0.340725828334676	0.812776860425551
Fgfr1	Fgfr1	0.0492161167705253	6.30389975027915	0.907683785602111	0.340729723702176	0.812776860425551
Cnnm1	Cnnm1	0.0594437104378 01	4.34145400603113	0.907491215772964	0.340780950414828	0.812776860425551
Ogfod3	Ogfod3	-0.0461714460810645	3.83057558 83029	0.907100845298375	0.340884821745887	0.812776860425551
Trpt1	Trpt1	0.0672548336427834	2.2726917072479	0.906389989935204	0.341074155732 98	0.812776860425551
Spin1	Spin1	-0.0282397854179467	6.59997116352507	0.906249281459587	0.341111557221 24	0.812776860425551
Spi1	Spi1	0.131525765187586	1.06534161218139	0.90608739204923	0.341154684505587	0.812776860425551
Smyd3	Smyd3	-0.0391045771930642	4.02301403501 31	0.906033852298979	0.341168968829559	0.812776860425551
Entpd6	Entpd6	-0.0265650011472475	6.41544655657696	0.906010308963594	0.341175221984511	0.812776860425551
C1qtnf4	C1qtnf4	-0.0497387000345503	8.14536679 56583	0.905902130295829	0.341204567185664	0.812776860425551
Dpf1	Dpf1	0.0283620717027277	8.14446249754255	0.90583461758552	0.341275342470727	0.812776860425551
Psme3ip	Psme3ip1	-0.0332957090902803	5.84202093091531	0.905347749947545	0.341351818371411	0.812776860425551
Lrrc20	Lrrc20	0.0472586743137704	4.31483864224425	0.905297929677806	0.341365128949472	0.812776860425551
Ndp	Ndp	-0.0903558955584762	2.56492238759	0.90513228178294	0.341409275105419	0.812776860425551
Psmd7	Psmd7	-0.0273135253723833	7.03571034887878	0.90419757247583	0.341658675588527	0.812911053236117
Lrrc39	Lrrc39	-0.154774007754278	0.488747526270766	0.904100682445539	0.341684528297664	0.812911053236117
Zfp26	Zfp26	0.0794942708880125	5.26063618060377	0.903997685302384	0.341712046974317	0.812911053236117
Neurod1	Neurod1	-0.083130739571746	5.12955176872515	0.903157173111524	0.341936565272866	0.812911053236117
Pik3r4	Pik3r4	0.0318787194581562	5.31092337561702	0.903128611108805	0.341944198452812	0.812911053236117
Egfem1	Egfem1	-0.0755528162390116	3.76778174570548	0.903026622991703	0.341971456597 22	0.812911053236117
Pcna	Pcna	0.0366104248123767	6.15092575982804	0.902619657150652	0.342080054652872	0.812911053236117
Nsfl1c	Nsfl1c	-0.0293148570237017	7.19489659 27943	0.902217479944838	0.342187818371879	0.812911053236117
Fam72a	Fam72a	0.172454477235214	-0.532261847364984	0.901919030135545	0.342267669313488	0.812911053236117
Ndufaf1	Ndufaf1	-0.0456685043847165	3.72487799787658	0.901878967468109	0.342278384734269	0.812911053236117
Baz2a	Baz2a	0.0779432987384452	6.24737528114836	0.901844732888055	0.342287551353875	0.812911053236117
Tnfrsf21	Tnfrsf21	0.0232182367537809	7.07038924293329	0.901741294754899	0.342315234767406	0.812911053236117
Mcm3	Mcm3	0.0663492359428013	3.78408580057362	0.901718178051311	0.342321421 90323	0.812911053236117
Cog7	Cog7	0.0329331338797198	4.97118655268451	0.901470703637692	0.342387667346999	0.812911053236117
Comt	Comt	-0.0382612709586236	5.31696672703503	0.901094889613977	0.342490677797047	0.812911053236117
Tent5a	Tent5a	0.116537822334647	1.37922272897867	0.900982275562541	0.342518463470589	0.812911053236117
Kidins22	Kidins220	0.0267641276472 08	10.1288894997614	0.899618765489144	0.342882954691881	0.812911053236117
Lrp1	Lrp1	0.0895727933794509	9.43268 6577441	0.899468522503241	0.342924259766784	0.812911053236117
Slc35e2b	Slc35e2b	0.0399270980957574	5.17047618328 16	0.899212960938058	0.342992832918761	0.812911053236117
Ak3	Ak3	0.0472918255221 27	5.51243667225472	0.899061574546948	0.343033462161342	0.812911053236117
Rcc1	Rcc1	0.0645122447178548	4.12966126 12697	0.89901122301891	0.343046970988264	0.812911053236117
Cdk14	Cdk14	-0.0385803181387596	7.29557232121569	0.898895473517712	0.343078049008779	0.812911053236117
Pink1	Pink1	-0.0351679163867261	6.27894546036339	0.898786636806541	0.343107266862 24	0.812911053236117
Zbtb3	Zbtb3	0.0653331346848581	2.23011961357836	0.898763076388974	0.343113592453294	0.812911053236117
Ghitm	Ghitm	-0.0374796206836458	6.79186224950239	0.898216789403028	0.343260630588003	0.812911053236117
Entpd5	Entpd5	-0.0560302490024961	2.96820230657575	0.897406130988486	0.343478176178037	0.812911053236117
Efcab7	Efcab7	0.0803708635257764	1.97891008432779	0.897052062822175	0.343573392873404	0.812911053236117
Selenof	Selenof	-0.0484122892077044	7.39307597005012	0.896780109906118	0.343646551708963	0.812911053236117
Pdk2	Pdk2	-0.0372767594338177	5.04542710676709	0.896545886486 29	0.343709583254 41	0.812911053236117
Acvr2b	Acvr2b	0.0579636599478065	3.40083461352528	0.895575685896731	0.343970809089696	0.812911053236117
Aurka	Aurka	0.0549554065195255	3.10799990854208	0.895466019946644	0.344000731274417	0.812911053236117
Lancl3	Lancl3	0.0628352409453807	3.54046361661775	0.895265375127302	0.344054418526001	0.812911053236117
Cars	Cars	-0.0402168529152271	6.80659129882776	0.89520245894515	0.344071373849327	0.812911053236117
Zfp418	Zfp418	0.0602177506209005	3.09908262930824	0.895077936687236	0.344104961756982	0.812911053236117
Mgat3	Mgat3	-0.023286175071287	6.87641430506365	0.894855516849839	0.344164891871973	0.812911053236117
Cahm	Cahm	0.0831383603550137	1.32374241736834	0.89475365610987	0.344192354928537	0.812911053236117
Unc119b	Unc119b	0.0415041129402889	5.41880691081962	0.894594084420518	0.344235383563641	0.812911053236117
Tmem53	Tmem53	-0.0611546420987584	2.90926116580304	0.894582515668048	0.344238503369767	0.812911053236117
Rab24	Rab24	-0.0385861403371642	5.83883584212851	0.894575352543413	0.344240435106298	0.812911053236117
Terf2	Terf2	-0.0290328804829714	5.52587990265909	0.894432497077401	0.344279893411447	0.812911053236117
Iars1	Iars1	0.0378225582655396	6.66870625310344	0.894390228022175	0.344290364184794	0.812911053236117
Slc35f6	Slc35f6	-0.0347139279271272	4.82624683602886	0.894360079261226	0.344298496394583	0.812911053236117
Nob1	Nob1	-0.0237758403806444	5.68987469682169	0.893947882630532	0.344409703795098	0.812911053236117
Mical2	Mical2	-0.0686780001541696	5.44203908182066	0.89385508017395	0.344434751500216	0.812911053236117
Prpf40a	Prpf40a	0.0269530948225407	6.78538576 05166	0.893755412598832	0.344461651679471	0.812911053236117
Ccdc148	Ccdc148	-0.0703844452073026	2.06705810025256	0.893449700258879	0.344544180854297	0.812911053236117
Spryd4	Spryd4	-0.0413069292551748	4.27136459132143	0.893425566424348	0.344550697629888	0.812911053236117
Sorcs3	Sorcs3	-0.0568908999944187	5.39195863 22634	0.893081894646883	0.344643507979648	0.812911053236117
Ube3d	Ube3d	-0.0812138000616982	2.54426071312287	0.893027766518351	0.344658126728705	0.812911053236117
Apbb1	Apbb1	-0.0261047115142291	8.57294312637304	0.891716993831742	0.345012463473005	0.81334742954771
LOC688	LOC688553	-0.140399580350658	0.270210574992452	0.891671366376983	0.345024785085974	0.81334742954771
Zbtb8b	Zbtb8b	0.0439479658529524	4.15742084928498	0.891298888115088	0.345125564152022	0.81334742954771
Samd11	Samd11	-0.0875877311325287	3.40713953797734	0.891059928958327	0.345190228792534	0.81334742954771
Adcyap1	Adcyap1r1	-0.0340832176109671	8.24015176648738	0.890976580103943	0.345212798970679	0.81334742954771
Dmac2l	Dmac2l	0.0518144839616889	3.67068692502876	0.890897277565614	0.345234270007588	0.81334742954771
Nphp3	Nphp3	0.0893350637774848	2.2251749082097	0.890296830218745	0.345396880417971	0.813490636708785
Crebzf	Crebzf	-0.048130948954426	6.65554886004063	0.889732258120771	0.345549600949647	0.813490636708785
Ikzf1	Ikzf1	0.209406165121204	1.00154710913 96	0.889719273789865	0.345553393371754	0.813490636708785
Pja2	Pja2	-0.0237554044446595	9.45891515556473	0.889309086710797	0.345664833668 0289	0.813490636708785
Cand1	Cand1	0.0284098212957638	7.88492679845232	0.889179821258864	0.345699665350222	0.813490636708785
Hs6st1	Hs6st1	0.0285667860223096	7.10685888 10583	0.889116891965386	0.345716734020706	0.813490636708785
Nap1l3	Nap1l3	-0.0314734098352306	6.49522092540749	0.888988911592776	0.345751450386045	0.813490636708785
LOC314	LOC314140	0.0536829998538585	3.40171499485861	0.887866214207349	0.346056199331389	0.813876535556257
Ube2k	Ube2k	0.0265443665085227	7.78892452480237	0.887568007331595	0.346137206808092	0.813876535556257
Map11	Map11	-0.0232233747048266	6.71652985474423	0.887238412684127	0.346226770618758	0.813876535556257
Arhgap2	Arhgap26	-0.0827918215324455	4.07503493329873	0.887202160141342	0.346236623775005	0.813876535556257
Grk4	Grk4	-0.0679328568081264	2.59047545525886	0.887183790540512	0.346241616633214	0.813876535556257
Arfgap3	Arfgap3	0.0282673557603143	5.27755724691328	0.886745941440822	0.346360652914496	0.814002988588 09
Ins5	Ins5	-0.200314676150517	0.751228094 7819	0.886330216336276	0.346473725761 6	0.814101982749166
RGD156	RGD156114	-0.0404600171838853	4.94518228807992	0.886111363045961	0.346533271650302	0.814101982749166
Snx16	Snx16	-0.0361112930877384	6.09011199460044	0.885381169646593	0.346732042985908	0.814278370867883
Paip1	Paip1	-0.0270946114069067	6.87982594687166	0.88517178408577	0.346789071188234	0.814278370867883
Pxdc1	Pxdc1	0.0391867601834953	5.77733872544879	0.885116461681292	0.346804140642136	0.814278370867883
Dennd1c	Dennd1c	-0.0809499398375152	2.2256649807034	0.884225874979052	0.347046852758547	0.814545517166102
Entpd3	Entpd3	-0.121912950846375	3.20947050132 75	0.884219882193321	0.347048486756031	0.814545517166102
Elavl4	Elavl4	0.0323151208087375	7.62388209456639	0.883364350259612	0.347281863852782	0.814556229759462
Phlpp2	Phlpp2	0.0533380912880562	5.18508884712861	0.883359230958129	0.347283223675762	0.814556229759462
Trim59	Trim59	0.0659679084780703	3.18736073061598	0.883304488821111	0.34729820117996	0.814556229759462
Akr1a1	Akr1a1	-0.0414048420425017	9.27414239678388	0.883027637793319	0.347373772663561	0.814556229759462
Ebp	Ebp	0.0616585231961309	3.01917895803693	0.883006754220666	0.347379474116382	0.814556229759462
Mrpl58	Mrpl58	-0.0388587555876116	4.67689778780525	0.88268682814988	0.347466806123702	0.814607918100375
LOC499	LOC499331	0.0347928785459199	5.11719355254 85	0.882161569348291	0.347610325787937	0.814791289659231
Smcr8	Smcr8	0.0401823780648784	4.72373082114785	0.881617722227407	0.347758980638 64	0.814872570158784
Dnase1l1	Dnase1l1	0.0657703750146793	2.97919930366745	0.88155686118337	0.347775622032181	0.814872570158784
Guk1	Guk1	-0.0425978874621426	6.6993642696437	0.881225104215602	0.347866352719476	0.814932122208206
B3galt2	B3galt2	0.0373297407766675	5.91689866656225	0.880542320308344	0.348053185294 61	0.815193085405208
Fbxo32	Fbxo32	0.0982040530619307	1.84757842384681	0.880340559323098	0.348108420216314	0.815193085405208
Ctdnep1	Ctdnep1	0.0254889834130396	6.36815051987694	0.879852519670997	0.348242076259749	0.815311950 15387
Idh2	Idh2	0.0469781570867427	5.62545030990884	0.879678069850851	0.348289868904394	0.81531195015387
Khdrbs2	Khdrbs2	0.0461567362439615	4.95230603602658	0.879191269318003	0.348423280173487	0.815399300949983
Cd276	Cd276	0.0441186330561618	5.55523557042247	0.878939662931245	0.348492261692385	0.815399300949983
Nup35	Nup35	0.0368901692469381	4.93689539968714	0.878490463003324	0.348615462956277	0.815399300949983
Mospd1	Mospd1	0.0603055582977026	3.31738494276161	0.878360684487739	0.348651065352208	0.815399300949983
Zfp37	Zfp37	0.0429445907352588	5.05963318811862	0.878036425422025	0.348740051177964	0.815399300949983
Il4r	Il4r	-0.0542942188311246	3.47822013207652	0.87785498187808	0.348789856330049	0.815399300949983
Srm	Srm	0.0359042209650045	6.82219281165393	0.877599803135126	0.348859917704655	0.815399300949983
Pcdhac2	Pcdhac2	-0.0368907665394606	5.71128479476027	0.876351569983299	0.349202905922796	0.815399300949983
Mrm3	Mrm3	0.0526396858744 82	3.52653650665702	0.875437239417415	0.349454425697637	0.815399300949983
Ppp2r2c	Ppp2r2c	-0.0372796509226796	7.60227801556375	0.875431352288405	0.349456055753245	0.815399300949983
Mest	Mest	0.0575005642708925	5.77743231571338	0.875029221976604	0.349566759660763	0.815399300949983
Rab3d	Rab3d	-0.0332505490782124	5.64168304380385	0.87500455177134	0.349573552745701	0.815399300949983
Elp5	Elp5	-0.0332202806719883	5.48666030906246	0.874799442968062	0.349630037613514	0.815399300949983
Zmynd15	Zmynd15	0.187757679676804	-0.416740505218657	0.874700821754558	0.349657201307048	0.815399300949983
Klhl29	Klhl29	0.0538205160761359	6.63054381931313	0.874405376892257	0.349738594199002	0.815399300949983
Caap1	Caap1	0.0428896747867754	4.12943461160151	0.874248525597856	0.349781816096838	0.815399300949983
Wdr47	Wdr47	0.0222001136039611	8.42522534999257	0.874240932690874	0.349783908579187	0.815399300949983
Sephs2	Sephs2	-0.035039834625317	6.04842312532373	0.874190830034728	0.349797716487786	0.815399300949983
Ndufa4	Ndufa4	-0.0390590014094 67	7.58658512770508	0.874041031772833	0.349839004165922	0.815399300949983
Capn6	Capn6	0.118612112572804	1.57179125514141	0.874025912957535	0.349843171610547	0.815399300949983
Tmem25	Tmem255a	-0.0891991806822262	3.62141442157206	0.874024059012768	0.349843682648104	0.815399300949983
Rps16	Rps16	-0.0512720720403076	9.09838647931764	0.873987848697 03	0.349853664345434	0.815399300949983
Sdhc	Sdhc	-0.0358091689178111	6.98198061025914	0.873875257147256	0.349884675439716	0.815399300949983
Sema6b	Sema6b	-0.0244522363120066	7.79129970882226	0.873721806236347	0.349927011831825	0.815399300949983
Rnf43	Rnf43	0.130764239678495	0.494774551290605	0.873424116327058	0.350009109464172	0.815399300949983
Afg3l2	Afg3l2	-0.0251264239152106	5.93295469406914	0.873361646249286	0.350026340938375	0.815399300949983
Cacnb2	Cacnb2	-0.054781332202528	4.86056741943548	0.872668576883937	0.350217591120183	0.815692530 22338
Mcoln1	Mcoln1	-0.0264713943821662	5.74191272006021	0.87214871192338	0.350361140441011	0.815787142310245
Nexmif	Nexmif	0.0552154743839484	8.11947089368495	0.872047926208348	0.350388979373295	0.815787142310245
Glra3	Glra3	-0.14452320400225	0.777880916439198	0.871495935370504	0.350541503179862	0.815815755764322
Hmgxb4	Hmgxb4	0.0316962081260179	4.78061460814328	0.871143855960128	0.350638825521403	0.815815755764322
Ndufaf6	Ndufaf6	-0.0639261007809 42	3.42964902944568	0.871138194507779	0.350640400930375	0.815815755764322
Slco1a4	Slco1a4	0.176707837268542	1.61154039996358	0.869721104608885	0.351032520235027	0.816652631330915
Map7d1	Map7d1	0.0216122156433597	8.36742377475712	0.869520885920985	0.351087979038152	0.816652631330915
Osbp	Osbp	0.0223473770984781	6.90408931385748	0.869172826427736	0.351184407050145	0.816724598548865
Stat5a	Stat5a	0.112647077015454	1.38355691657198	0.868864734408158	0.351269789950863	0.816770986872329
Tmem16	Tmem164	0.0280350227944328	6.2438530148229	0.868542876451372	0.351359018087574	0.816820934071435
Prn20d1	Prn20d1	0.210646164820528	-0.589945952927641	0.868253546095616	0.351439255023188	0.816820934071435
Slc6a1	Slc6a1	-0.0601358270989432	8.70322854601888	0.867965607691161	0.351519130743791	0.816820934071435
Mrpl48	Mrpl48	-0.0415914094647526	5.34124946891427	0.867843069468451	0.351553121039189	0.816820934071435
Nanl1	Nanl1	0.250002041422645	-0.679391157108918	0.867210059047338	0.351728853815625	0.817077063686008
Mrpl57	Mrpl57	0.0560626246 2649	4.00872147062022	0.866748713067011	0.351856677605334	0.817158968323 747
Aebp1	Aebp1	0.156305661941409	4.18815854079178	0.86661146847041	0.351895108651512	0.81715898323 747
Pcdh12	Pcdh12	0.0850325348886527	2.44717087996322	0.865693074695116	0.352150413654178	0.817599685739335
Csf1	Csf1	0.0585511 71737078	3.76918518689279	0.865050499046276	0.352329194044435	0.817710021954848
Ube2a	Ube2a	-0.0410972913629393	4.10262889068708	0.864910229964839	0.352396074641509	0.817710021954848
Ric8a	Ric8a	0.0319419096198784	6.80047810241587	0.864516949074253	0.352449916164261	0.817710021954848
Zfp61	Zfp61	0.0651221747441278	4.90676527513015	0.864386641617671	0.352514025658125	0.817710021954848
Gpr108	Gpr108	0.0417195405497636	4.69011215522931	0.864316559484794	0.352532526222677	0.817710021954848
Zmiz2	Zmiz2	0.0290541250526466	8.84222504444968	0.864020264715293	0.352616089229892	0.817710021954848
Bckdha	Bckdha	-0.0411585248383408	5.09043061711579	0.863874529878359	0.352656698576504	0.817710021954848
Gulp1	Gulp1	0.0837024338283224	2.92172922076661	0.863233586984535	0.352835374596 63	0.817828504780709
Senp2	Senp2	-0.0298203811089554	5.12778501085213	0.863081200539888	0.352877873640456	0.817828504780709
Yeats2	Yeats2	0.0369336725821969	6.42563660282385	0.86296894732304	0.352909184402755	0.817828504780709
Gtf2e2	Gtf2e2	0.0435269499762301	4.64445666117661	0.862634417871732	0.353002517124587	0.817867924926858
Cactin	Cactin	0.0298847063771889	4.82201432729251	0.862287939524123	0.353099219095608	0.817867924926858
Marveld	Marveld1	0.0834456340902 53	3.29322512722503	0.862251243219958	0.353109463138654	0.817867924926858
Ireb2	Ireb2	0.0281571597174199	6.48104981520839	0.861740614220494	0.353252051114873	0.818026325493 74
Kcnj4	Kcnj4	-0.100822752834727	4.12948318982899	0.861323175877148	0.353368674958791	0.818055267504273

Osf	Osf	-0.0716520082206252	4.15713958869014	0.861226544317049	0.353395679397935	0.818055267504273
St8sia4	St8sia4	0.0515116737713828	4.58012994886226	0.860799647993087	0.353515012517007	0.818079196779131
Uqcrq	Uqcrq	-0.061571370109616	6.82380183426241	0.860259738985292	0.353666015688273	0.818079196779131
Itgb1bp1	Itgb1bp1	0.0417879647775291	4.35213736994063	0.860216386117472	0.353679144555679	0.818079196779131
Gatd3a	Gatd3a	-0.0347106260070784	6.28208420469126	0.860077475639649	0.353717011483765	0.818079196779131
Inafm1	Inafm1	0.0593720362984777	4.9912599907299	0.859900575439184	0.353766516327411	0.818079196779131
Irf1	Irf1	0.0970676262582768	1.3448246254951	0.859466532743927	0.353888021369928	0.818079196779131
Prx	Prx	0.103272294340686	1.10587256714509	0.859328422957628	0.353926695601261	0.818079196779131
C1qbp	C1qbp	-0.0367165540682172	6.88916417741559	0.859028071042999	0.354010821961046	0.818079196779131
Nfasc	Nfasc	0.0284209982240201	8.09711863570129	0.858977517228936	0.354024984353264	0.818079196779131
Casp2	Casp2	0.0675800477705286	5.68178376285589	0.858846518749473	0.354061686511766	0.818079196779131
Otof	Otof	-0.119615197718947	3.77230585750833	0.858581722188166	0.354135890810404	0.818099150090986
Herpud2	Herpud2	0.0267823480062181	5.70248560717407	0.85811623263983	0.354266387916466	0.818176394937778
Uso1	Uso1	0.022510034282059	6.91739984460102	0.857732659732219	0.354373969684927	0.818176394937778
Atg3	Atg3	-0.0300138370667285	5.60257513470559	0.857722421928898	0.354376841434799	0.818176394937778
Dolpp1	Dolpp1	-0.0282597291980656	5.32673302344532	0.8573762237019	0.354473948951085	0.818176394937778
Cldnd1	Cldnd1	-0.0270857378982471	6.61597044306072	0.85724250827159	0.354521508255573	0.818176394937778
Fkbp2	Fkbp2	-0.0482775490145372	5.11804309385294	0.856978268469689	0.354585685512278	0.818176394937778
Txndc15	Txndc15	-0.0332058669871626	6.57613574100608	0.856826314126266	0.354628351672958	0.818176394937778
Nagpa	Nagpa	-0.0540817146678995	2.89756137726744	0.85572261479988	0.35493846237229	0.8184407992561
C1qtnf2	C1qtnf2	-0.083814531925759	2.38334372531875	0.855465815255727	0.355010668567144	0.8184407992561
Pafah1b(	Pafah1b3	0.0402001559068324	7.06341631670704	0.85517449514046	0.355092607645958	0.8184407992561
Amn1	Amn1	-0.0412326378728135	4.98689175164135	0.854998560278972	0.355142104461414	0.8184407992561
Tsnax	Tsnax	-0.0262831447009482	7.1327560652327	0.854845282625739	0.355185234688627	0.8184407992561
Glmn	Glmn	-0.0434637222956495	5.46089903227338	0.854225821799758	0.355359615550925	0.8184407992561
Adamts1	Adamts18	0.061471450340318	4.26904354585793	0.854015589238957	0.355418823533129	0.8184407992561
Fgd6	Fgd6	0.0850515872580099	2.80358186673877	0.854001176480878	0.355422880135945	0.8184407992561
Cox6l1	Cox6l1	-0.0470873246916627	8.38238878787847	0.853970436912505	0.355431541619627	0.8184407992561
Pcdhb12	Pcdhb12	-0.0540523666247808	3.00410500764379	0.853946939966752	0.355438165885943	0.8184407992561
Zfp385b	Zfp385b	-0.0404402679182291	4.57852939794105	0.853728810226386	0.355499611216685	0.8184407992561
Ppp1r14	Ppp1r14d	-0.168506152105297	-0.106443281869625	0.853620589600957	0.355530105952367	0.8184407992561
Ebf2	Ebf2	-0.155621954580612	1.47190201173019	0.852687147052293	0.355793266758219	0.818876459366119
Dph3	Dph3	-0.0293259082926604	5.4039604952611	0.852136444696761	0.355948649727056	0.818876459366119
Fut11	Fut11	0.0403854328956527	4.21730268529135	0.852088700796557	0.355962125248478	0.818876459366119
Csnk1g2	Csnk1g2	0.0258844652182407	7.31606018326982	0.851926730179258	0.356007846005813	0.818876459366119
Top2a	Top2a	0.0689754360009174	5.55286779911542	0.851636513784371	0.356089767871842	0.818876459366119
Mknk2	Mknk2	0.0271901136526124	7.04606352687758	0.851464613809981	0.35613833558787	0.818876459366119
Supt4h1	Supt4h1	-0.044898685004891	4.61291562981788	0.851048023679674	0.356256025953755	0.818876459366119
Ssb	Ssb	-0.02657048257373	6.84867328375221	0.85056580802737	0.356392294430564	0.818876459366119
Rps6ka5	Rps6ka5	0.0404891340443365	5.85239769808921	0.85046479426662	0.356420885812086	0.818876459366119
Flcn	Flcn	0.026598307496452	5.79501105447295	0.85014892544988	0.356510208070149	0.818876459366119
Got1	Got1	-0.040866804773808	6.9332724167618	0.849930595389026	0.356571972734164	0.818876459366119
Rbbp7	Rbbp7	-0.0251888488919259	7.49227981087514	0.849857106751529	0.356592764712456	0.818876459366119
Guf1	Guf1	-0.0376157200878044	5.07717944700996	0.849822693819604	0.35660292165995	0.818876459366119
Mrgprf	Mrgprf	0.212272494496885	1.61172259450463	0.849696577504872	0.356638188682925	0.818876459366119
Ccnb1	Ccnb1	0.0560138013469199	4.01455178513719	0.84931744806353	0.356745500115599	0.81897214642361
Klhl28	Klhl28	0.0517849399849065	4.16633752682057	0.848151607577051	0.357075765417854	0.819318041324064
Pex11b	Pex11b	-0.0410836770782254	5.14383967786271	0.848071286383401	0.357098534666342	0.819318041324064
Paqr4	Paqr4	0.0313142927178285	5.96671982605224	0.848033900529224	0.357109133393185	0.819318041324064
Olfm2	Olfm2	-0.0292524917731163	8.08247042211411	0.847858581836605	0.357158838403589	0.819318041324064
Slc9a1	Slc9a1	-0.0431699544828953	6.04874398573544	0.847136089016301	0.357363794469202	0.819627511689749
Chrm4	Chrm4	0.074738150720305	3.76416719068135	0.846278559780018	0.357607251367267	0.820045152602649
Adh1	Adh1	0.227011429109579	0.565862823720232	0.846036728272509	0.35767595133181	0.820051974415104
Cbr3	Cbr3	-0.0789832181557705	2.2445470518152	0.845308635771715	0.357882898744631	0.820231428625614
Sccpdh	Sccpdh	-0.0296877442244412	6.87179547272866	0.845001757715117	0.357970172678452	0.820231428625614
Gtf2h5	Gtf2h5	-0.0435717242762682	6.23275619054883	0.844869415085824	0.35800781900394	0.820231428625614
Stk17b	Stk17b	-0.0679174298705176	2.67527708588742	0.844635679429409	0.358074320987144	0.820231428625614
Cebpd	Cebpd	-0.0954180379872286	1.59382998862234	0.844536537962377	0.358102520638167	0.820231428625614
Rassf8	Rassf8	0.0803721206991015	2.02519098188814	0.843874707905137	0.358290948660298	0.820231428625614
Hdac8	Hdac8	0.0509578675218887	3.46449351879325	0.843773596522844	0.358319745846296	0.820231428625614
Sbk1	Sbk1	0.0307206118869581	9.86126953847388	0.843642121495975	0.358357195555835	0.820231428625614
Jpt2	Jpt2	0.0374705146813364	5.20288826228448	0.84331620580646	0.358450053482977	0.820231428625614
Pigb	Pigb	0.0951009258446902	1.27436455308398	0.843281900482994	0.358459829472325	0.820231428625614
Tmem22	Tmem223	0.0593867267279299	4.30943902858344	0.84322032979809	0.358477356760077	0.820231428625614
Ldah	Ldah	0.0294425271060856	5.91268869039079	0.842193626065466	0.358770145436806	0.820732344845246
G6pc3	G6pc3	-0.0404888801858626	5.32446710551121	0.841923577063298	0.35884720575269	0.820732344845246
Prss12	Prss12	0.116680757220649	5.33720406247143	0.841710990950432	0.358907884658186	0.820732344845246
St8sia6	St8sia6	-0.183934980793037	-0.334863288447637	0.841530555667276	0.358959397757535	0.820732344845246
Traip	Traip	-0.0578648189822934	2.97952780119218	0.841227472289731	0.359045848982336	0.820779828774754
Prr5	Prr5	0.03093995013182758	4.92557412636912	0.840755612621223	0.359180754641555	0.820827109116355
Arl8b	Arl8b	-0.0269318315566003	7.81342895009994	0.840679232287723	0.359202582012928	0.820827109116355
Rxfp3	Rxfp3	-0.10019035239758	1.46858099316125	0.838513852318774	0.35952594630957	0.821032789972409
Fanca	Fanca	0.0651949771286664	3.85181856945292	0.838477986960404	0.359540109484306	0.821032789972409
Cstf2	Cstf2	-0.0431417525059731	6.93093141282401	0.838378049830202	0.359574709464447	0.821032789972409
Ier5	Ier5	0.0527285221404602	5.61104597040413	0.838318184794389	0.359591843126536	0.821032789972409
Erf	Erf	0.0686968790549539	5.06143939622697	0.838229485045422	0.359617221481864	0.821032789972409
Snn	Snn	0.0257962403231506	8.77037159229421	0.838857037003756	0.359723863507582	0.821125987007725
Agtr1a	Agtr1a	0.106197976275463	1.59147302039813	0.838332631387544	0.359874074862548	0.821192979306871
Marchf1	Marchf11	0.0578017677013005	3.18881263785611	0.838295043207019	0.35988494498189	0.821192979306871
Herpud1	Herpud1	0.0613809925006364	5.27177465185127	0.838046108424386	0.359956183324883	0.821205576768092
Ccnd1	Ccnd1	0.0601295929423544	5.52676897747061	0.837743427195392	0.360042950264872	0.821253362057551
Nf2	Nf2	-0.0201732456289646	9.2275767918233	0.837156307937484	0.360211326685872	0.821343380066632
Pum2	Pum2	0.0287685840306833	8.44180652240303	0.836986255145611	0.360260128267098	0.821343380066632
Tmsb10	Tmsb10	-0.0543456522791106	10.4768373769459	0.83673861615388	0.360331197182734	0.821343380066632
Rpl39	Rpl39	-0.0532802924369782	6.52804932623851	0.836687987966386	0.360345729140398	0.821343380066632
Setd6	Setd6	-0.046262071374592	3.72794819168385	0.835969978736046	0.36055190841435	0.821423337516513
Stat2	Stat2	0.0683636576247545	4.620009581964	0.835809885639362	0.360597807710756	0.821423337516513
Ngf	Ngf	-0.165286960689603	-0.163509287280966	0.835541161521959	0.360675122576207	0.821423337516513
Snx29	Snx29	0.0293587251201498	5.10043662060551	0.835537297170397	0.360676223203236	0.821423337516513
RragB	RragB	-0.0272456340134844	6.66504723298149	0.835419872526849	0.360709980670191	0.821423337516513
Dgcr8	Dgcr8	0.0284063655499299	6.18995485744689	0.834799267632036	0.360888431720181	0.821497481213026
Hcar1	Hcar1	0.25217404553278	-0.71869027485174	0.834291075808432	0.361034647296827	0.821497481213026
Stim1	Stim1	-0.0274691416550316	5.91124895945359	0.834029444658142	0.361109954960346	0.821497481213026
Pnma8a	Pnma8a	0.0312709203946317	5.60437183809472	0.833724746105446	0.361197686414398	0.821497481213026
Nedd1	Nedd1	0.0672201777199194	2.51920380570381	0.833680282580186	0.361210490908788	0.821497481213026
Tnfaip8	Tnfaip8	-0.0782648971116306	2.1518640103351	0.833645967107695	0.361220365290708	0.821497481213026
LOC688	LOC688459	-0.166960604068725	0.952675492357208	0.833528759447105	0.361254132132706	0.821497481213026
Ap2a1	Ap2a1	-0.0211896216148744	8.0772056408441	0.83347620670493	0.36126926980972	0.821497481213026
Bex3	Bex3	-0.0402168806576995	7.54313395789613	0.832419137660324	0.361573941647084	0.821925000675997
Rmnd5b	Rmnd5b	0.0343971299899114	5.46897668932152	0.832351566052943	0.361593429406953	0.821925000675997
Srd5a3	Srd5a3	0.0535804168211605	3.80396179540272	0.831906828916964	0.361721728482747	0.822076868174724
Poc5	Poc5	-0.036986574562937	4.06806173333411	0.831608005947842	0.361807969103917	0.822123116100814
Atad3a	Atad3a	-0.0320352998707567	6.07630265416215	0.831000430314241	0.361983403609711	0.822371982308516
Nckap1	Nckap1	-0.022217188831733	9.06861467146397	0.830632189932493	0.362089794170485	0.822463928975996
Echs1	Echs1	0.0500706067442366	5.22806067798494	0.830175411463699	0.362221811860075	0.822614041969086
Zkscan1	Zkscan1	0.0411969726802799	5.49652771347316	0.829130241550672	0.362524149335822	0.823150800075169
Pbx4	Pbx4	-0.078708085654417	1.82296173585149	0.828746628103772	0.362635205088732	0.823253175744561
Gnat1	Gnat1	-0.101206321905537	0.573732677769792	0.828121638080844	0.362816229854983	0.823514321388143
Slc25a34	Slc25a34	-0.0774619058273796	2.32788563184347	0.827789928736034	0.362912663472857	0.8235324379106
LOC100	LOC100362	-0.0338822517166364	4.89341315405655	0.827638644222773	0.362956229547999	0.8235324379106
Pik3c3	Pik3c3	0.0293606362673402	5.21997405355036	0.827212349991299	0.363079847605289	0.82358746793208
Plcb2	Plcb2	0.126145816730079	0.197249865532677	0.826792340871606	0.36320170002133	0.82358746793208
Lrrtm2	Lrrtm2	0.02794921375663	6.18861478082276	0.82641652042998	0.363310780424257	0.82358746793208
Evc	Evc	0.0560761377398335	3.01676693122997	0.826297032228897	0.363345470605098	0.82358746793208
Sumo1	Sumo1	-0.0276598430645204	6.72183344711676	0.826080971869047	0.363408210358202	0.82358746793208
Rassf3	Rassf3	0.06997245046577	4.52112937437351	0.825750885695753	0.363504095305606	0.82358746793208
Sirt6	Sirt6	0.0320496535507993	5.89279088865299	0.825552475047902	0.363562028660313	0.82358746793208
LOC687	LOC687399	0.0548932320367772	2.61311764852941	0.825501092658911	0.363576669386386	0.82358746793208
Tnfsf13	Tnfsf13	0.106102211562	1.20509830536612	0.825450333863188	0.363591420318078	0.82358746793208
Fbxl22	Fbxl22	-0.0731754167330367	3.45853737809533	0.825137182924667	0.363682442628898	0.82358746793208
Tmem22	Tmem229b	-0.0303278819456485	8.22764838065281	0.824703298804822	0.363808591681348	0.82358746793208
Ubqln4	Ubqln4	0.0206879273675463	7.10954091440797	0.824286758993445	0.363929792105909	0.82358746793208
Ambra1	Ambra1	0.028323589511071	6.59408573054585	0.824178952785495	0.363961164585548	0.82358746793208
Tmem16	Tmem168	0.0334099308295668	4.86869054284777	0.824068880169413	0.363993200490515	0.82358746793208
Jam3	Jam3	-0.0394469702577094	4.43314133624508	0.823958181633493	0.364025422491526	0.82358746793208
Micalcl	Micalcl	-0.147172958057834	-0.039254411616426	0.823919691535636	0.364036627089442	0.82358746793208
Maco1	Maco1	0.0237940006677159	7.02492828256513	0.823014357315905	0.364300310191612	0.823976715062758
Cdc34	Cdc34	0.0689760523498037	5.31636415362732	0.822790180182324	0.364365643746943	0.823976715062758
Npy	Npy	0.0964497116484127	6.05216411570256	0.822645982078023	0.364407674097643	0.823976715062758
Mrpl36	Mrpl36	0.0531408068973858	4.74131956188239	0.821956182018453	0.364598487381435	0.824260794923524
Sec16b	Sec16b	-0.0976591454534526	0.939098503744436	0.821744349253706	0.364670852299731	0.824274588540533
Kcnn4	Kcnn4	0.19201197827504	-0.510206269438	0.820740482035057	0.364963752310952	0.824787672085447
Tpr	Tpr	0.0483082428128298	7.48601015201554	0.820241023824821	0.365109705562779	0.824958089049033
Gimap8	Gimap8	0.117696877967509	0.876429584689755	0.820029990585667	0.365171397436708	0.824958089049033
Mrpl40	Mrpl40	-0.0461163460181621	3.36311609462437	0.819460982173475	0.36532780618036	0.825203081504693
Brms1	Brms1	0.0401572251542343	5.00572082359429	0.819295171371905	0.365386321759052	0.825203081504693
Cmtm4	Cmtm4	0.0620248119159464	4.79882682132843	0.819242376356371	0.365401770312971	0.825203081504693
Siglec15	Siglec15	-0.140967326438068	-0.323170506992525	0.817935644855872	0.365784426894433	0.825725682335463
Lrrc61	Lrrc61	0.0357562148465415	4.46981237076442	0.817380317057157	0.365947214552861	0.825725682335463
Rnaseh2	Rnaseh2a	0.0362302329261019	6.07517470586159	0.817270245166142	0.365979490737229	0.825725682335463
Rnf138	Rnf138	0.032418625791989	5.94373296818438	0.817055443097955	0.366042494044829	0.825725682335463
Gamt	Gamt	-0.0404565891627231	4.4507425648053	0.816881514890294	0.366093518101831	0.825725682335463
Zfp365	Zfp365	0.0430574167171477	5.0944553973624	0.816500036390906	0.366205464252012	0.825725682335463
Ddx56	Ddx56	-0.0486293381459354	5.38196479117817	0.816377168149259	0.36624153046594	0.825725682335463
Dmrta2	Dmrta2	-0.0752319959753296	2.97844471594657	0.816128441354465	0.366314555749776	0.825725682335463
Sirt3	Sirt3	-0.0432010466057765	4.83148508466125	0.815873530294286	0.36638941769678	0.825725682335463
Gabrq	Gabrq	-0.108964947607398	1.78708809445682	0.815439347798545	0.366516976712503	0.825725682335463
Ash2l	Ash2l	0.0278677790702338	6.0141909480759	0.815290911003459	0.366560600280177	0.825725682335463
Pdia5	Pdia5	0.077279163717952	2.75028795752258	0.815124590507011	0.366609488211938	0.825725682335463
Strn	Strn	0.0611341667416827	5.7774919407439	0.814752160554065	0.366718992384645	0.825725682335463
Mpst	Mpst	0.0454419903002722	5.62368094960451	0.814489943151673	0.366796412801003	0.825725682335463
Nfil3	Nfil3	0.0430633369064079	5.04102585194406	0.814488349254574	0.366796858730807	0.825725682335463
Ptprn2	Ptprn2	-0.0284828177686124	7.93620194757872	0.814297159296821	0.366852808341964	0.825725682335463
Anapc13	Anapc13	-0.0649234720741068	3.5038996545187	0.814189344559722	0.366884561254602	0.825725682335463
Ubb	Ubb	0.0424996780214739	10.3330553182031	0.813723836239177	0.367021582677097	0.825725682335463
Slc39a2	Slc39a2	-0.0544245368432303	3.32374215938261	0.813251619625259	0.367160651615053	0.825725682335463
Nab2	Nab2	0.0595613299187362	4.12574676980323	0.813250914898816	0.367160858918389	0.825725682335463
Insyn1	Insyn1	-0.0290998246888796	6.18390235350746	0.813194657011897	0.367177461320865	0.825725682335463
Spred2	Spred2	0.0311295342253408	5.35726690661894	0.813039622013235	0.367223108812967	0.825725682335463
Rpgrip1l	Rpgrip1l	0.0537194798850304	5.79678661273191	0.813009949427581	0.367231851953874	0.825725682335463
Elp2	Elp2	-0.0200806071226184	7.08732869659523	0.812682507594793	0.367328353154988	0.825793849065726
Tpmt	Tpmt	-0.0663088013689071	2.72398397132713	0.81241689060336	0.36740663020863	0.82582102776312
Il15	Il15	0.12175384038033	1.21914906088928	0.81215334978494	0.367484377086394	0.825847005200582
H19	H19	0.183439220871196	3.54632058557113	0.811465438142091	0.367687052710086	0.826153674889923
Ahsp	Ahsp	-0.176624141238279	-0.226375212063576	0.811072962191427	0.367803236982354	0.826265932000646
Hccs	Hccs	-0.0391663222588527	4.64858647235183	0.810683252360272	0.367918240929456	0.826375498123641
Snta1	Snta1	-0.0444793999634567	4.30135089911162	0.809437435339909	0.368286625379635	0.827050086962546
Lactb	Lactb	0.0427779530921786	3.91983022867897	0.808989346411039	0.368419219510607	0.827050086962546
Xkr5	Xkr5	0.122410721681916	0.43274916580119	0.808835703932587	0.36846489919769	0.827050086962546
Stard5	Stard5	0.0850080328428723	1.54903809626408	0.808708267346717	0.368502427610834	0.827050086962546
Prex2	Prex2	0.0762844358867492	3.02156080216735	0.808547570163569	0.368550010701075	0.827050086962546
Noct	Noct	-0.0309673490011356	5.44984714912058	0.807949495958919	0.36872717807587	0.82729886726378
P2rx2	P2rx2	-0.0680231461120223	3.99795461011455	0.807252476429258	0.368934102213124	0.827421916539851
Plk2	Plk2	0.038989170033432	8.05183874577896	0.807240445256653	0.368937373642675	0.827421916539851
Egf	Egf	0.0953370508741933	1.68159886327268	0.807093457875652	0.368980968409178	0.827421916539851
Zfp386	Zfp386	-0.0715978750732936	4.67118972257606	0.806562718711291	0.369138438274736	0.827494409002493
Psmg4	Psmg4	-0.0622323886571907	4.2920290808965	0.806463276308872	0.369167954352051	0.827494409002493
Timp4	Timp4	-0.101536063808083	2.64852305828905	0.806294072248193	0.369218182645388	0.827494409002493
Tprn	Tprn	-0.0364868955189845	4.37616307011868	0.806090640885465	0.369278582150581	0.827494409002493
Csmd1	Csmd1	0.0764178943905748	5.72370609982915	0.805121859150958	0.369566416183389	0.827827321657563
Trim32	Trim32	0.0220061625463652	7.12117503529761	0.805093546844098	0.369574822681209	0.827827321657563
RGD130	RGD130656	0.034919043248384	4.69226918725966	0.804858169583646	0.36966448144066	0.827827321657563
Bambi	Bambi	0.0925406333078275	1.31782534482061	0.804697680679382	0.369692541177842	0.827827321657563
R3hdm1	R3hdm1	0.0323748117710088	7.25151788534813	0.80366062067477	0.370001149840265	0.82827088898327
Cept1	Cept1	-0.0307738298237883	4.93610273233416	0.803585899993453	0.370023402334916	0.82827088898327
Sec14l1	Sec14l1	0.022878001297732	7.1381074368834	0.802975973845143	0.370205089102547	0.828528950086543
Sema3d	Sema3d	0.0780732105655732	3.47911049059251	0.802265102811504	0.370417002752988	0.828854545076942
Fn3krp	Fn3krp	0.0334909948892053	4.82362635836393	0.801485530916814	0.370649591032618	0.828962038886823
Plpp1	Plpp1	-0.0394910499714723	4.35646792055295	0.801127575603104	0.370756456657564	0.828962038886823
RGD131	RGD131151	0.0410507656448992	4.3408500874568	0.800880582654703	0.370830219844864	0.828962038886823
Gabrg3	Gabrg3	-0.0916454014091516	3.25842310559134	0.800761820849465	0.370865972706742	0.828962038886823
Sorcs1	Sorcs1	0.0454849362871421	5.91712327733861	0.800761617908236	0.370865755813172	0.828962038886823
Tbc1d17	Tbc1d17	-0.0277330177007001	5.91794705408749	0.800659697507221	0.370896502750694	0.828962038886823
Sqle	Sqle	0.0267913402719171	7.91735877469221	0.800529375518494	0.370935142688726	0.828962038886823
RGD130	RGD130866	0.0284791191101755	4.0739063589691	0.800211987137693	0.371029996414567	0.828962038886823
Art3	Art3	-0.131847554358575	0.635208603297744	0.800101588841337	0.371062994877346	0.828962038886823
Pstpip1	Pstpip1	-0.14858032039498	-0.150457759916543	0.799585951674544	0.37121719199579	0.829155800789095
Prr15	Prr15	-0.12841678547332	0.685175155671438	0.799238696010946	0.371321094402707	0.829155800789095
Ucp2	Ucp2	0.0691150438071286	5.17848806000301	0.799032622027525	0.371382757090335	0.829155800789095
Vasp	Vasp	0.0389670367415523	5.66694760889782	0.798895034651018	0.371423941448898	0.829155800789095
Lrtm2	Lrtm2	-0.0522344402539326	4.66071758373665	0.798227528577485	0.371623828229718	0.829155800789095
Mx1	Mx1	-0.155878151396076	-0.379407903287521	0.798127208639794	0.371653893943527	0.829155800789095
Coq8a	Coq8a	-0.0540658368988376	7.22638236013702	0.79795371441422	0.371705880316259	0.829155800789095
Gcn1	Gcn1	0.037362216517642	7.76371915047409	0.797804398791897	0.371750629956356	0.829155800789095
Rnf227	Rnf227	-0.0404025435405906	5.44429223756627	0.797581295208744	0.371817507754061	0.829155800789095
Ctsa	Ctsa	0.0367603422883584	6.06525419184837	0.797297350501339	0.371902647681229	0.829155800789095
Atxn10	Atxn10	-0.0274231388321163	8.58348920443356	0.797248036787025	0.37191743702044	0.829155800789095
Pdzd9	Pdzd9	-0.0828975898942006	0.978887932797617	0.797149154984666	0.371947094463272	0.829155800789095
Frs3	Frs3	-0.040557747455471	4.55776268059856	0.796065289034435	0.372272092982698	0.829614449659967
Dusp7	Dusp7	0.0266481110813608	6.39436359303517	0.796020280043557	0.372285810366767	0.829614449659967
Map3k10	Map3k10	-0.0307407308143546	8.2895871749026	0.795653709114996	0.372396024286229	0.829711641967728
Zfp54	Zfp54	0.13223191717798	0.412025982183623	0.79511567741001	0.372557725670836	0.829780634947429
Cacna2d2	Cacna2d2	-0.0789959996489592	6.62573785662006	0.794929151863712	0.372613807449275	0.829780634947429
Lpar3	Lpar3	0.247730974887643	0.22666527920366	0.794886927222933	0.372626504577258	0.829780634947429
Gripap1	Gripap1	-0.0321512600634882	6.82538885107343	0.793310874232638	0.373100863737308	0.830527867684642
Rps12	Rps12	-0.0341132294501984	8.19590122972378	0.793250929670435	0.373118922483745	0.830527867684642
Prpf3	Prpf3	0.0378900173668608	4.95474712457721	0.793108764381543	0.373161755729751	0.830527867684642
Traf7	Traf7	0.0262794474803442	6.61454300749623	0.792400679764071	0.373375198439085	0.830723670655602
Grm8	Grm8	-0.0696405609511011	3.64199003176395	0.792375166963751	0.373382892124438	0.830723670655602
Rab5b	Rab5b	-0.0172097671789771	7.72847105262441	0.791871769992028	0.373534742827872	0.830913351089919
Inpp5a	Inpp5a	-0.0361527309726762	5.4728208152957	0.790569495858215	0.373927978089415	0.83162282125533
Ap3b1	Ap3b1	0.0450940808308835	4.89688945489376	0.790140116348297	0.374057760758967	0.83162282125533
Scarf1	Scarf1	0.0685311946248855	3.07340037130548	0.790113335934933	0.374065857404457	0.83162282125533
Egr4	Egr4	-0.147925613127833	2.65889724525689	0.789998473796015	0.374130826555377	0.83162282125533
Reln	Reln	0.0749828051867673	8.26891782685605	0.789712922756266	0.374186945461844	0.83162282125533
Cers2	Cers2	0.0449852795417828	5.04562400217967	0.789065448461145	0.374382863012783	0.8316893219201595
Ddx18	Ddx18	-0.0386532355919646	3.82757304652138	0.788744647883822	0.374480047066504	0.8316893219201595
Sox14	Sox14	0.348767316891985	1.05276586265596	0.788492296998263	0.374556414473143	0.8316893219201595
Sart3	Sart3	0.0270867182344266	6.05806428694687	0.788455578772876	0.374567523450514	0.8316893219201595
Pex5	Pex5	0.0242094826564315	6.67999587839286	0.788430965607404	0.374574989075073	0.8316893219201595
Fez2	Fez2	-0.0414105287310558	4.71311232607711	0.788033906197263	0.374695291924821	0.8316893219201595
Psmb3	Psmb3	-0.0414213563274645	6.42605284509271	0.787829736007168	0.374757173428681	0.8316893219201595
Thbs1	Thbs1	-0.093241938146453	4.36497204631661	0.787800381642157	0.374766071558769	0.8316893219201595
Vav1	Vav1	0.156372079815836	0.133857197237926	0.787507927278363	0.374854735822463	0.8316893219201595
Rtn4rl1	Rtn4rl1	-0.0396475574262948	6.10353555969621	0.787407472196151	0.374885201954778	0.8316893219201595
Mios	Mios	0.0302962195020251	5.16814062529346	0.786949515874618	0.37502412166979	0.831816461583499
Hspa4	Hspa4	0.0214416140921982	8.49378912377912	0.786798852902976	0.375074089359422	0.831816461583499
Ell	Ell	0.0460549317391184	3.92114580252751	0.786220435176709	0.375245435271502	0.832008905253811
Ppwd1	Ppwd1	0.0490310688550191	3.41058025191086	0.786059767273132	0.375294230986307	0.832008905253811
Map2	Map2	0.0543809980213833	9.9496015276886	0.785469015789204	0.375473721702385	0.832137281782262
Prelid3b	Prelid3b	-0.0347379596580805	5.35313021759427	0.785413409035885	0.375490623146873	0.832137281782262
RGD156	RGD156545	0.0511992301149096	3.35178473435275	0.78521078581992	0.375552218779828	0.832137281782262
Thrb	Thrb	-0.057101435798685	4.66295143288726	0.784989869270776	0.375619385485016	0.83213832966813
Ttc25	Ttc25	-0.115729046222333	0.946130096978939	0.784523116218864	0.375761369800621	0.832305097150727
Map3k5	Map3k5	0.0595651074607205	4.7575768850024	0.784177368230658	0.375866589155896	0.832390385675818
Tcf3	Tcf3	0.0318015436134846	6.02282635432575	0.783874943489572	0.375958858064402	0.832446526471869
Rasa2	Rasa2	0.0682481668323929	4.2088373377675	0.78343886045665	0.376091442667875	0.832546488055705
Shmt2	Shmt2	0.0391545314716841	5.80045170292768	0.783204785746307	0.376162790987506	0.832546488055705
Jmjd1c	Jmjd1c	0.0735657177790774	7.19972017685835	0.782905187317709	0.37625410033532	0.832546488055705
Epc2	Epc2	-0.0285497049352051	6.27008535288774	0.782850700389538	0.376270709767717	0.832546488055705
Naxd	Naxd	-0.0419469097902576	4.9357983369794	0.782436930439218	0.376396874537288	0.832677979382011
Cldn9	Cldn9	-0.141272784538266	0.873177493303878	0.782024772234056	0.376522685654004	0.832808467155115
Add3	Add3	-0.0341555075536004	6.81590555802533	0.781375300881692	0.376720853678446	0.832809032290346
Atm	Atm	0.0638649138168168	5.02792420534831	0.781021940088834	0.376828776321403	0.832809032290346
Snrnp27	Snrnp27	0.0379103889849857	4.59848075889328	0.780382946082462	0.377024046856048	0.832809032290346
Lgals9	Lgals9	0.0800717899329538	2.82954910322871	0.780100756757115	0.377110327175115	0.832809032290346
Ndufa11	Ndufa11	-0.0539158988702615	6.07635859047468	0.780086178955614	0.377114784497502	0.832809032290346
LOC100	LOC100294	-0.0225901317788423	6.72633900778898	0.780022375510775	0.377134297260154	0.832809032290346
Vsig2	Vsig2	-0.103773923097457	1.32244912469827	0.779641358243381	0.37725084762111	0.832809032290346
Nrxn2	Nrxn2	-0.0312433819779657	10.2756850509423	0.779189361440991	0.37738917656497	0.832809032290346
Slc16a7	Slc16a7	0.0486804079612441	4.69449549525668	0.778522655426034	0.377593345086541	0.832809032290346
Ndufaf5	Ndufaf5	-0.0310320973091265	4.92617465359686	0.778212381120824	0.377688415322016	0.832809032290346
Rpusd2	Rpusd2	0.0379667699053032	3.85583536998635	0.777761316544044	0.377826584369263	0.832809032290346
Nsmce2	Nsmce2	-0.0375103738927806	4.49426733384605	0.777682348915846	0.377850898387464	0.832809032290346
Tnfrsf11	Tnfrsf11b	0.0709716053563438	2.76511159522296	0.777559854764291	0.377888463364262	0.832809032290346
Dbi	Dbi	-0.0532978264062301	6.64536935088533	0.777492067403355	0.377912320951425	0.832809032290346
Cops7a	Cops7a	0.0324238569523205	5.94524592642894	0.777262087580572	0.377979800828789	0.832809032290346
Ap3d1	Ap3d1	-0.0212011378005536	8.72631176277068	0.777045984241198	0.378046108091125	0.832809032290346
Trpm7	Trpm7	0.0651313471987989	6.06752758962712	0.77675050953836	0.378136795499753	0.832809032290346
Nsmf	Nsmf	-0.0302940853237483	8.75630904684014	0.776517203140017	0.378208423802172	0.832809032290346
Socs6	Socs6	-0.0347238588934882	4.53224699389525	0.77640473394392	0.378242960235561	0.832809032290346
Slc9a3	Slc9a3	-0.0515519624742166	5.80637164832969	0.776240880933768	0.378293288429473	0.832809032290346
Chi3l1	Chi3l1	0.15773653092112	-0.338859067219355	0.776217247934895	0.378300542305723	0.832809032290346
Dnajb9	Dnajb9	0.0664491812468484	2.26152069679742	0.775317176327206	0.378577150944658	0.832809032290346
Unc79	Unc79	0.0619046169518776	6.54401599902011	0.774903338310949	0.378704426893285	0.832809032290346
Nes	Nes	0.0543178840595063	6.28153357238464	0.774870367673685	0.378714569396522	0.832809032290346
Oas1b	Oas1b	-0.105227601915751	1.25040901524839	0.774770709898036	0.378745229283623	0.832809032290346
Mus81	Mus81	0.051599280920351	4.15770126842638	0.774138254646658	0.378939886946374	0.832809032290346
Add1	Add1	0.0220898970118334	8.91472529656559	0.774113174072902	0.378947609072654	0.832809032290346
Ndel1	Ndel1	0.032758473200848	6.98848878482032	0.774061156983365	0.378963625671161	0.832809032290346
Hivep1	Hivep1	0.0489323637492648	7.09918125368656	0.773644239157633	0.37909203347196	0.832809032290346
Dpyd	Dpyd	0.0644307821572895	3.29547422442257	0.773503851044453	0.379135285949881	0.832809032290346
Slc43a2	Slc43a2	0.0371554061839493	5.69072047423633	0.77338687498565	0.379171330527877	0.832809032290346
Dek	Dek	-0.0253390815489196	6.71516576589703	0.772671581980212	0.379391843989992	0.832809032290346
Naa20	Naa20	-0.0294745764779196	5.08503400810722	0.772419745095078	0.379469524218067	0.832809032290346
Angptl6	Angptl6	-0.0897123445760016	0.974596448565418	0.772392101544462	0.37947805237684	0.832809032290346
Zfp202	Zfp202	0.0523325614803042	4.63690540892315	0.772276788815516	0.379513625957168	0.832809032290346
Rnf103	Rnf103	0.0317778147830042	5.52546734388001	0.771838111981218	0.37964901767611	0.832809032290346
Grin2a	Grin2a	-0.0848710469052316	3.32690404179383	0.771582398094349	0.379727971103267	0.832809032290346
Mtmr2	Mtmr2	0.0222612633829837	6.35303836986373	0.771435735152686	0.379773262329688	0.832809032290346
Klhl41	Klhl41	-0.113040030200607	0.543150718102915	0.770974052888812	0.37991588985651	0.832809032290346
Hk1	Hk1	-0.0220950322258096	9.15090608719918	0.770752648990733	0.379984315012506	0.832809032290346
Ppp4r3a	Ppp4r3a	0.0352825220913369	6.24729878014968	0.770529512088288	0.380053293363082	0.832809032290346
Klf3	Klf3	0.0363367243735461	5.36745134752351	0.770252832153609	0.380138848042251	0.832809032290346
Trmt10c	Trmt10c	0.0361452674311548	4.5084045891294	0.770210052518667	0.380152078434909	0.832809032290346
Emc2	Emc2	-0.0362037068491877	5.85128911758898	0.770010475755271	0.380213811585706	0.832809032290346
Zswim2	Zswim2	-0.113719676562984	0.6650470532485	0.769892278921344	0.380250069367317	0.832809032290346
Auts2l1	Auts2l1	0.0613496846550686	7.02383605379042	0.769859165959517	0.38026062398589	0.832809032290346
Chmp4b	Chmp4bl1	-0.030035298960978	6.0878388912168	0.769741789601078	0.380296943627962	0.832809032290346
Gpatch1	Gpatch11	0.0356846433615995	4.56481782146466	0.769456238041677	0.380385322010994	0.832809032290346
Avp	Avp	-0.259805822596069	3.0998257865472	0.769446903148349	0.380388211656012	0.832809032290346
Zw10	Zw10	0.0327899360633861	4.49618123309098	0.768742320122811	0.380606407362868	0.832809032290346
Rrp1	Rrp1	-0.0215742032413437	6.20437802709006	0.768390280778497	0.380715492946405	0.832809032290346
Paqr3	Paqr3	0.0292044537550247	5.2730890255359	0.768263954410308	0.380754649275862	0.832809032290346
Xpot	Xpot	-0.022828705173852	6.67383971056282	0.768188919870422	0.380777629203861	0.832809032290346
Ppp2cb	Ppp2cb	-0.0251981575203293	7.76772850744327	0.768181958343767	0.380780066309464	0.832809032290346
Plaur	Plaur	-0.187357785890593	-0.475267034832164	0.768002582375765	0.3808356764888	0.832809032290346
Fbxo33	Fbxo33	0.042769455240378	4.05509224546484	0.767907744949127	0.380865087390428	0.832809032290346
Mapk15	Mapk15	-0.0870675521487037	3.77779980077633	0.767643162091801	0.380947167924248	0.832809032290346
Rgs12	Rgs12	0.0234167995249569	6.76053754632552	0.767231240433887	0.381074955443306	0.832809032290346
Rarg	Rarg	-0.186198137391394	5.58400347596482	0.767215636790084	0.381079797999953	0.832809032290346
Tbc1d7	Tbc1d7	0.0302542187313515	4.72315003242306	0.767063248044821	0.381126927305361	0.832809032290346
Acadl	Acadl	0.0477041856404118	4.51420125653412	0.766767075140564	0.381219045344652	0.832809032290346
Spg7	Spg7	0.0329498737138785	5.89650277009111	0.766649808520697	0.381255400379354	0.832809032290346
Hbs1l	Hbs1l	-0.0277907422275826	6.47682448607057	0.765037087281165	0.381756761507372	0.832809032290346
Strada	Strada	-0.03079661236269	4.99456148291059	0.76494432429993	0.381785618661811	0.832809032290346
Arhgdib	Arhgdib	0.0885958431912602	4.58312782312678	0.76491940728576	0.381793377874213	0.832809032290346
Tmc5	Tmc5	-0.103002638632544	1.22778487831413	0.764898858255594	0.381804128818296	0.832809032290346
Ebag9	Ebag9	-0.0333204050382785	4.55422298968911	0.764858870750705	0.381811904880382	0.832809032290346
Rapgef2	Rapgef2	0.0332706308041515	6.99126660674889	0.764599469849842	0.381864929282089	0.832809032290346
Nenf	Nenf	-0.0536181055386386	4.4376944910301	0.764479079835638	0.381930430756261	0.832809032290346
Rps13	Rps13	-0.04190142188989	7.76423857728616	0.764477071195287	0.381931056109532	0.832809032290346
Lemd2	Lemd2	-0.0265727861716968	6.15798031075479	0.764208125178165	0.382014800924399	0.832809032290346
Map1b	Map1b	0.0629412211540579	12.4134741216056	0.764166720748335	0.382027695437963	0.832809032290346
Smyd5	Smyd5	0.0244376552566436	6.00318040905515	0.763742605891444	0.382159815606297	0.832809032290346
Pmaip1	Pmaip1	-0.140087219701993	-0.304194864660687	0.763608636617022	0.382201563756679	0.832809032290346
Abcg4	Abcg4	-0.0400196455473184	6.16775320535058	0.76347588423078	0.38224290665563	0.832809032290346
Dip2a	Dip2a	0.0412872241518224	6.89117532511823	0.763458859487514	0.382248244354092	0.832809032290346
Fam20b	Fam20b	0.0224845669891885	6.92305573665068	0.763292646421153	0.382298883635275	0.832809032290346
Zfp213	Zfp213	0.0302408907658517	4.86306422255162	0.763161906045267	0.382340820700606	0.832809032290346
Mdm1	Mdm1	0.0502109607706771	5.36978596495828	0.762843761357402	0.382440037445329	0.832809032290346
Pacrg	Pacrg	0.0907933781712464	2.12906319775426	0.762841706993889	0.382440678866446	0.832809032290346
Stk16	Stk16	0.0267915727103543	5.9868244714195	0.76249006562869	0.382550384570577	0.832809032290346
Mpp4	Mpp4	0.176930316386398	0.771410888476821	0.762460247679634	0.382559912736035	0.832809032290346
Vps13b	Vps13b	0.0449171803788719	5.84813779777984	0.762452103195542	0.382582221030251	0.832809032290346
Eif1a	Eif1a	-0.0346382664185006	5.59528196209428	0.762436805462841	0.382567004870295	0.832809032290346
Tkfc	Tkfc	0.0621486249629889	2.42352440275347	0.761989864180087	0.382706516287674	0.832809032290346

Vamp3	Vamp3	0.0339041582503344	5.23995138272968	0.761953911649952	0.382717741804183	0.832809032290346
Mmip	Mmip	0.0367980389130673	4.09302797247924	0.76158239382432	0.382832769807735	0.832809032290346
Trub1	Trub1	0.0389316779471721	3.76923497271409	0.761494324906725	0.382954405811481	0.832809032290346
Ilvbl	Ilvbl	-0.035510388012445	4.45228687040619	0.761465882181177	0.382970167570591	0.832809032290346
RGD130	RGD130814	0.0475979037589686	3.70003249943474	0.761209725151303	0.382950207232681	0.832809032290346
Ung	Ung	-0.066067870970935	2.77115657946367	0.761198169242583	0.382953818588783	0.832809032290346
Eif3f	Eif3f	-0.029621856020195	8.08270506980652	0.760960709341482	0.383028038265024	0.832809032290346
Zfr	Zfr	-0.029298836554486	8.47011954539153	0.760883987917119	0.383052622416849	0.832809032290346
Ubxn11	Ubxn11	0.0478250628533437	3.8170458444287	0.760811928436169	0.38307455112147	0.832809032290346
Synpo2	Synpo2	-0.161320071347359	2.89946397881252	0.760732279952748	0.383099454640708	0.832809032290346
Ing5	Ing5	0.0620596677278684	4.12616171844315	0.760721409389109	0.383102854007066	0.832809032290346
Map4k3	Map4k3	0.0259839563785774	7.24784435708762	0.759921428965686	0.383349882627955	0.832809032290346
Ppp1r9b	Ppp1r9b	-0.022507508505230	9.02738154028656	0.759906728629765	0.383357713258531	0.832809032290346
Ndufb9	Ndufb9	-0.04447576706989	6.41790182332695	0.75989118354066	0.383362578623187	0.832809032290346
Mcm10	Mcm10	0.0731896114807784	2.42736078130912	0.759889912548815	0.383362876427212	0.832809032290346
Tmem25	Tmem250	0.0322598812009813	5.20017479071623	0.759733453882952	0.383411850440585	0.832809032290346
Abr	Abr	0.0215816990504942	9.17074270675367	0.759588882769364	0.383457211039966	0.832809032290346
Ogfod2	Ogfod2	0.0519491487551109	3.4454732808641	0.759575076491252	0.383461524083907	0.832809032290346
Apoc1	Apoc1	-0.129259481117335	0.133976890241813	0.759394893116557	0.383517955647661	0.832809032290346
Cxad	Cxad	-0.051265777890220	3.83870989379912	0.759352111844699	0.383531353653949	0.832809032290346
Id1	Id1	0.0672940165890537	4.07518038677829	0.758813882712666	0.383699958941449	0.832951906973242
Cmtm6	Cmtm6	0.0811763152572086	1.79689065655097	0.758715925788547	0.38373066933435	0.832951906973242
Ptafr	Ptafr	0.126582712966456	0.91494694385141	0.758436964622447	0.383818113604224	0.832996800041729
Cox14	Cox14	-0.049497064599987	5.37378515548838	0.757840617680685	0.38400514175098	0.833257765584817
Mboat1	Mboat1	0.0649880466251337	2.49772720820745	0.757613201614433	0.384076498745155	0.833267688200887
Ly6h	Ly6h	-0.028216172714300	8.52888098398645	0.757048225176533	0.384253854528534	0.833507535282817
Myorg	Myorg	-0.062477024702526	3.17653579546491	0.756591849598948	0.384397203564246	0.833673545779784
Bdh2	Bdh2	0.103273691665048	1.06254734107157	0.756160781541986	0.384532673187966	0.833719880328219
Orc6	Orc6	-0.041115055721038	4.00754403382129	0.756098635952327	0.384552208967612	0.833719880328219
Chrna2	Chrna2	-0.106724405657635	0.963998078220093	0.755008174100368	0.384895230726844	0.834281712752704
Tmem16	Tmem167b	0.0376012913218363	4.22187398588615	0.754849804263074	0.384945084581014	0.834281712752704
Tpt1	Tpt1	-0.036058469978888	9.67315067189994	0.754504255499981	0.385053893078937	0.834340224489431
[illegible]	[illegible]	[illegible]	[illegible]	[illegible]	[illegible]	[illegible]

Raly	Raly	[illegible]	[illegible]	[illegible]	[illegible]	[illegible]
Copb2	Copb2	[illegible]	[illegible]	[illegible]	[illegible]	[illegible]
Igsf11	Igsf11	[illegible]	[illegible]	[illegible]	[illegible]	[illegible]
Acy1	Acy1	[illegible]	[illegible]	[illegible]	[illegible]	[illegible]
Svbp	Svbp	[illegible]	[illegible]	[illegible]	[illegible]	[illegible]
Sgms2	Sgms2	[illegible]	[illegible]	[illegible]	[illegible]	[illegible]
Xrn2	Xrn2	[illegible]	[illegible]	[illegible]	[illegible]	[illegible]
Atp6v0e2	Atp6v0e2	[illegible]	[illegible]	[illegible]	[illegible]	[illegible]
Atp5pf	Atp5pf	[illegible]	[illegible]	[illegible]	[illegible]	[illegible]
Kif26a	Kif26a	[illegible]	[illegible]	[illegible]	[illegible]	[illegible]
Borcs5	Borcs5	[illegible]	[illegible]	[illegible]	[illegible]	[illegible]
Gne	Gne	[illegible]	[illegible]	[illegible]	[illegible]	[illegible]
Tbc1d23	Tbc1d23	[illegible]	[illegible]	[illegible]	[illegible]	[illegible]
Rplp2	Rplp2	[illegible]	[illegible]	[illegible]	[illegible]	[illegible]
Cmas	Cmas	[illegible]	[illegible]	[illegible]	[illegible]	[illegible]
Dhrs11	Dhrs11	[illegible]	[illegible]	[illegible]	[illegible]	[illegible]
Nacad	Nacad	[illegible]	[illegible]	[illegible]	[illegible]	[illegible]
Selenop	Selenop	[illegible]	[illegible]	[illegible]	[illegible]	[illegible]
Brcc3	Brcc3	[illegible]	[illegible]	[illegible]	[illegible]	[illegible]
Rabggta	Rabggta	[illegible]	[illegible]	[illegible]	[illegible]	[illegible]
Mnat1	Mnat1	[illegible]	[illegible]	[illegible]	[illegible]	[illegible]
Pmm2	Pmm2	[illegible]	[illegible]	[illegible]	[illegible]	[illegible]
Anks4b	Anks4b	[illegible]	[illegible]	[illegible]	[illegible]	[illegible]
Nutf2	Nutf2	[illegible]	[illegible]	[illegible]	[illegible]	[illegible]
Sgo2	Sgo2	[illegible]	[illegible]	[illegible]	[illegible]	[illegible]
Iqsec3	Iqsec3	[illegible]	[illegible]	[illegible]	[illegible]	[illegible]
Efhc1	Efhc1	[illegible]	[illegible]	[illegible]	[illegible]	[illegible]
Tm9sf2	Tm9sf2	[illegible]	[illegible]	[illegible]	[illegible]	[illegible]
Slco4a1	Slco4a1	[illegible]	[illegible]	[illegible]	[illegible]	[illegible]
Tspyl4	Tspyl4	[illegible]	[illegible]	[illegible]	[illegible]	[illegible]
Wdr46	Wdr46	[illegible]	[illegible]	[illegible]	[illegible]	[illegible]
Syt9	Syt9	[illegible]	[illegible]	[illegible]	[illegible]	[illegible]
Dnase2	Dnase2	[illegible]	[illegible]	[illegible]	[illegible]	[illegible]
Cops9	Cops9	[illegible]	[illegible]	[illegible]	[illegible]	[illegible]
Vps28	Vps28	[illegible]	[illegible]	[illegible]	[illegible]	[illegible]
Ints13	Ints13	[illegible]	[illegible]	[illegible]	[illegible]	[illegible]
Pstpip2	Pstpip2	[illegible]	[illegible]	[illegible]	[illegible]	[illegible]
Poldip3	Poldip3	[illegible]	[illegible]	[illegible]	[illegible]	[illegible]
Chn2	Chn2	[illegible]	[illegible]	[illegible]	[illegible]	[illegible]
Prr3	Prr3	[illegible]	[illegible]	[illegible]	[illegible]	[illegible]
Celsr3	Celsr3	[illegible]	[illegible]	[illegible]	[illegible]	[illegible]
Psmc3ip	Psmc3ip	[illegible]	[illegible]	[illegible]	[illegible]	[illegible]
Gpr137	Gpr137	[illegible]	[illegible]	[illegible]	[illegible]	[illegible]
Laptm4b	Laptm4b	[illegible]	[illegible]	[illegible]	[illegible]	[illegible]
Tdrd7	Tdrd7	[illegible]	[illegible]	[illegible]	[illegible]	[illegible]
LOC288913	LOC288913	[illegible]	[illegible]	[illegible]	[illegible]	[illegible]
Hey1	Hey1	[illegible]	[illegible]	[illegible]	[illegible]	[illegible]
Frzb	Frzb	[illegible]	[illegible]	[illegible]	[illegible]	[illegible]
Tmod2	Tmod2	[illegible]	[illegible]	[illegible]	[illegible]	[illegible]
Dync1li2	Dync1li2	[illegible]	[illegible]	[illegible]	[illegible]	[illegible]
B3gntl1	B3gntl1	[illegible]	[illegible]	[illegible]	[illegible]	[illegible]
Aasdhppt	Aasdhppt	[illegible]	[illegible]	[illegible]	[illegible]	[illegible]
Lekr1	Lekr1	[illegible]	[illegible]	[illegible]	[illegible]	[illegible]
Uba5	Uba5	[illegible]	[illegible]	[illegible]	[illegible]	[illegible]
Rwdd2b	Rwdd2b	[illegible]	[illegible]	[illegible]	[illegible]	[illegible]
Slc37a2	Slc37a2	[illegible]	[illegible]	[illegible]	[illegible]	[illegible]
Wnt2b	Wnt2b	[illegible]	[illegible]	[illegible]	[illegible]	[illegible]
Coq2	Coq2	[illegible]	[illegible]	[illegible]	[illegible]	[illegible]
Sox2	Sox2	[illegible]	[illegible]	[illegible]	[illegible]	[illegible]
Pcdhb4	Pcdhb4	[illegible]	[illegible]	[illegible]	[illegible]	[illegible]
RGD15657[illegible]	RGD15657[illegible]	[illegible]	[illegible]	[illegible]	[illegible]	[illegible]
Naa40	Naa40	[illegible]	[illegible]	[illegible]	[illegible]	[illegible]
Fto	Fto	[illegible]	[illegible]	[illegible]	[illegible]	[illegible]
Dzip3	Dzip3	[illegible]	[illegible]	[illegible]	[illegible]	[illegible]
Rasl10b	Rasl10b	[illegible]	[illegible]	[illegible]	[illegible]	[illegible]
Mrpl46	Mrpl46	[illegible]	[illegible]	[illegible]	[illegible]	[illegible]
Sgf29	Sgf29	[illegible]	[illegible]	[illegible]	[illegible]	[illegible]
Zfand5	Zfand5	[illegible]	[illegible]	[illegible]	[illegible]	[illegible]
Emc3	Emc3	[illegible]	[illegible]	[illegible]	[illegible]	[illegible]
Grhpr	Grhpr	[illegible]	[illegible]	[illegible]	[illegible]	[illegible]
Wnt3	Wnt3	[illegible]	[illegible]	[illegible]	[illegible]	[illegible]
Gins4	Gins4	[illegible]	[illegible]	[illegible]	[illegible]	[illegible]
Ank3	Ank3	[illegible]	[illegible]	[illegible]	[illegible]	[illegible]
Dclk3	Dclk3	[illegible]	[illegible]	[illegible]	[illegible]	[illegible]
Dpysl3	Dpysl3	[illegible]	[illegible]	[illegible]	[illegible]	[illegible]
Slc39a9	Slc39a9	[illegible]	[illegible]	[illegible]	[illegible]	[illegible]
Map3k11	Map3k11	[illegible]	[illegible]	[illegible]	[illegible]	[illegible]
Sh3tc1	Sh3tc1	[illegible]	[illegible]	[illegible]	[illegible]	[illegible]
Ubox5	Ubox5	[illegible]	[illegible]	[illegible]	[illegible]	[illegible]
Sdr39u1	Sdr39u1	[illegible]	[illegible]	[illegible]	[illegible]	[illegible]
Glb1l	Glb1l	[illegible]	[illegible]	[illegible]	[illegible]	[illegible]
Tph2	Tph2	[illegible]	[illegible]	[illegible]	[illegible]	[illegible]
Smpd5	Smpd5	[illegible]	[illegible]	[illegible]	[illegible]	[illegible]
Lama5	Lama5	[illegible]	[illegible]	[illegible]	[illegible]	[illegible]
Mid1	Mid1	[illegible]	[illegible]	[illegible]	[illegible]	[illegible]
Hsd17b1	Hsd17b1	[illegible]	[illegible]	[illegible]	[illegible]	[illegible]
Gls	Gls	[illegible]	[illegible]	[illegible]	[illegible]	[illegible]
Cdc73	Cdc73	[illegible]	[illegible]	[illegible]	[illegible]	[illegible]
Papolg	Papolg	[illegible]	[illegible]	[illegible]	[illegible]	[illegible]
Abhd4	Abhd4	[illegible]	[illegible]	[illegible]	[illegible]	[illegible]
Mad2l2	Mad2l2	[illegible]	[illegible]	[illegible]	[illegible]	[illegible]
Cyyr1	Cyyr1	[illegible]	[illegible]	[illegible]	[illegible]	[illegible]
Pde11a	Pde11a	[illegible]	[illegible]	[illegible]	[illegible]	[illegible]
Dync2h1	Dync2h1	[illegible]	[illegible]	[illegible]	[illegible]	[illegible]
Dnajc9	Dnajc9	[illegible]	[illegible]	[illegible]	[illegible]	[illegible]
Atp5md	Atp5md	[illegible]	[illegible]	[illegible]	[illegible]	[illegible]
Agap2	Agap2	[illegible]	[illegible]	[illegible]	[illegible]	[illegible]
Orc4	Orc4	[illegible]	[illegible]	[illegible]	[illegible]	[illegible]
Ube2f	Ube2f	[illegible]	[illegible]	[illegible]	[illegible]	[illegible]
Tspan15	Tspan15	[illegible]	[illegible]	[illegible]	[illegible]	[illegible]
F11r	F11r	[illegible]	[illegible]	[illegible]	[illegible]	[illegible]
Ddx52	Ddx52	[illegible]	[illegible]	[illegible]	[illegible]	[illegible]
Xbp1	Xbp1	[illegible]	[illegible]	[illegible]	[illegible]	[illegible]
Tm2d3	Tm2d3	[illegible]	[illegible]	[illegible]	[illegible]	[illegible]
Ppp1r10	Ppp1r10	[illegible]	[illegible]	[illegible]	[illegible]	[illegible]
Cab39l	Cab39l	[illegible]	[illegible]	[illegible]	[illegible]	[illegible]
Ankrd11	Ankrd11	[illegible]	[illegible]	[illegible]	[illegible]	[illegible]
Ppara	Ppara	[illegible]	[illegible]	[illegible]	[illegible]	[illegible]
Fbxo38	Fbxo38	[illegible]	[illegible]	[illegible]	[illegible]	[illegible]
Ipp	Ipp	[illegible]	[illegible]	[illegible]	[illegible]	[illegible]
Nup210	Nup210	[illegible]	[illegible]	[illegible]	[illegible]	[illegible]
Map1a	Map1a	[illegible]	[illegible]	[illegible]	[illegible]	[illegible]
Mcm7	Mcm7	[illegible]	[illegible]	[illegible]	[illegible]	[illegible]
Cd14	Cd14	[illegible]	[illegible]	[illegible]	[illegible]	[illegible]
St3gal6	St3gal6	[illegible]	[illegible]	[illegible]	[illegible]	[illegible]
Timm13	Timm13	[illegible]	[illegible]	[illegible]	[illegible]	[illegible]
Mppe1	Mppe1	[illegible]	[illegible]	[illegible]	[illegible]	[illegible]
Mybl2	Mybl2	[illegible]	[illegible]	[illegible]	[illegible]	[illegible]
Coro7	Coro7	[illegible]	[illegible]	[illegible]	[illegible]	[illegible]
Mtln	Mtln	[illegible]	[illegible]	[illegible]	[illegible]	[illegible]
Gpat3	Gpat3	[illegible]	[illegible]	[illegible]	[illegible]	[illegible]
Isca1	Isca1	[illegible]	[illegible]	[illegible]	[illegible]	[illegible]
Stag3	Stag3	[illegible]	[illegible]	[illegible]	[illegible]	[illegible]
Ccdc173	Ccdc173	[illegible]	[illegible]	[illegible]	[illegible]	[illegible]
Pxmp4	Pxmp4	[illegible]	[illegible]	[illegible]	[illegible]	[illegible]
Mpzl2	Mpzl2	[illegible]	[illegible]	[illegible]	[illegible]	[illegible]
Pcdhga11	Pcdhga11	[illegible]	[illegible]	[illegible]	[illegible]	[illegible]
Slc13a3	Slc13a3	[illegible]	[illegible]	[illegible]	[illegible]	[illegible]
Tra2a	Tra2a	[illegible]	[illegible]	[illegible]	[illegible]	[illegible]
Plekhh3	Plekhh3	[illegible]	[illegible]	[illegible]	[illegible]	[illegible]
Myh3	Myh3	[illegible]	[illegible]	[illegible]	[illegible]	[illegible]
Eif3l	Eif3l	[illegible]	[illegible]	[illegible]	[illegible]	[illegible]
Ufd1	Ufd1	[illegible]	[illegible]	[illegible]	[illegible]	[illegible]
Cdh20	Cdh20	[illegible]	[illegible]	[illegible]	[illegible]	[illegible]
Dmd	Dmd	[illegible]	[illegible]	[illegible]	[illegible]	[illegible]
Siglec5	Siglec5	[illegible]	[illegible]	[illegible]	[illegible]	[illegible]
Rnf217	Rnf217	[illegible]	[illegible]	[illegible]	[illegible]	[illegible]
Parpbp	Parpbp	[illegible]	[illegible]	[illegible]	[illegible]	[illegible]
Kbtbd11	Kbtbd11	[illegible]	[illegible]	[illegible]	[illegible]	[illegible]
Limk1	Limk1	[illegible]	[illegible]	[illegible]	[illegible]	[illegible]
Ifngr1	Ifngr1	[illegible]	[illegible]	[illegible]	[illegible]	[illegible]
Rad17	Rad17	[illegible]	[illegible]	[illegible]	[illegible]	[illegible]
Specc1	Specc1	[illegible]	[illegible]	[illegible]	[illegible]	[illegible]
Pgm2	Pgm2	[illegible]	[illegible]	[illegible]	[illegible]	[illegible]
Atg2a	Atg2a	[illegible]	[illegible]	[illegible]	[illegible]	[illegible]
Nmt2	Nmt2	[illegible]	[illegible]	[illegible]	[illegible]	[illegible]
Fbxl17	Fbxl17	[illegible]	[illegible]	[illegible]	[illegible]	[illegible]
Mbd1	Mbd1	[illegible]	[illegible]	[illegible]	[illegible]	[illegible]
Megf9	Megf9	[illegible]	[illegible]	[illegible]	[illegible]	[illegible]
Faf2	Faf2	[illegible]	[illegible]	[illegible]	[illegible]	[illegible]
LOC103693	LOC103693	[illegible]	[illegible]	[illegible]	[illegible]	[illegible]
Mllt11	Mllt11	[illegible]	[illegible]	[illegible]	[illegible]	[illegible]
Ero1a	Ero1a	[illegible]	[illegible]	[illegible]	[illegible]	[illegible]
Rpl8	Rpl8	[illegible]	[illegible]	[illegible]	[illegible]	[illegible]
Cetn4	Cetn4	[illegible]	[illegible]	[illegible]	[illegible]	[illegible]
Zfp787	Zfp787	[illegible]	[illegible]	[illegible]	[illegible]	[illegible]
B4galt6	B4galt6	[illegible]	[illegible]	[illegible]	[illegible]	[illegible]
Acvr1b	Acvr1b	[illegible]	[illegible]	[illegible]	[illegible]	[illegible]
Prpf8	Prpf8	[illegible]	[illegible]	[illegible]	[illegible]	[illegible]
Ccl27	Ccl27	[illegible]	[illegible]	[illegible]	[illegible]	[illegible]
Chd6	Chd6	[illegible]	[illegible]	[illegible]	[illegible]	[illegible]
Eif3i	Eif3i	[illegible]	[illegible]	[illegible]	[illegible]	[illegible]
LOC691141	LOC691141	[illegible]	[illegible]	[illegible]	[illegible]	[illegible]
C1ql4	C1ql4	[illegible]	[illegible]	[illegible]	[illegible]	[illegible]
Prrt2	Prrt2	[illegible]	[illegible]	[illegible]	[illegible]	[illegible]
Phldb1	Phldb1	[illegible]	[illegible]	[illegible]	[illegible]	[illegible]
Churc1	Churc1	[illegible]	[illegible]	[illegible]	[illegible]	[illegible]
Pheta2	Pheta2	[illegible]	[illegible]	[illegible]	[illegible]	[illegible]
Fxr1	Fxr1	[illegible]	[illegible]	[illegible]	[illegible]	[illegible]
LOC100909[illegible]	LOC100909[illegible]	[illegible]	[illegible]	[illegible]	[illegible]	[illegible]
Ltbr	Ltbr	[illegible]	[illegible]	[illegible]	[illegible]	[illegible]
Aagab	Aagab	[illegible]	[illegible]	[illegible]	[illegible]	[illegible]
Smg7	Smg7	[illegible]	[illegible]	[illegible]	[illegible]	[illegible]
Ube4b	Ube4b	[illegible]	[illegible]	[illegible]	[illegible]	[illegible]
Chrna3	Chrna3	[illegible]	[illegible]	[illegible]	[illegible]	[illegible]
Rnf2	Rnf2	[illegible]	[illegible]	[illegible]	[illegible]	[illegible]
Plekhm2	Plekhm2	[illegible]	[illegible]	[illegible]	[illegible]	[illegible]
Bhmt	Bhmt	[illegible]	[illegible]	[illegible]	[illegible]	[illegible]
Adgrl2	Adgrl2	[illegible]	[illegible]	[illegible]	[illegible]	[illegible]
Nat10	Nat10	[illegible]	[illegible]	[illegible]	[illegible]	[illegible]
Tmed4	Tmed4	[illegible]	[illegible]	[illegible]	[illegible]	[illegible]
Wscd1	Wscd1	[illegible]	[illegible]	[illegible]	[illegible]	[illegible]
Ppp2r5a	Ppp2r5a	[illegible]	[illegible]	[illegible]	[illegible]	[illegible]
Apc2	Apc2	[illegible]	[illegible]	[illegible]	[illegible]	[illegible]
Mtus1	Mtus1	[illegible]	[illegible]	[illegible]	[illegible]	[illegible]
Thap7	Thap7	[illegible]	[illegible]	[illegible]	[illegible]	[illegible]
Ctsb	Ctsb	[illegible]	[illegible]	[illegible]	[illegible]	[illegible]
Ndufab1	Ndufab1	[illegible]	[illegible]	[illegible]	[illegible]	[illegible]
Abo	Abo	[illegible]	[illegible]	[illegible]	[illegible]	[illegible]
Neurl1b	Neurl1b	[illegible]	[illegible]	[illegible]	[illegible]	[illegible]
Spdl1	Spdl1	[illegible]	[illegible]	[illegible]	[illegible]	[illegible]
Zbtb2	Zbtb2	[illegible]	[illegible]	[illegible]	[illegible]	[illegible]
Ybx1	Ybx1	[illegible]	[illegible]	[illegible]	[illegible]	[illegible]
Poldip2	Poldip2	[illegible]	[illegible]	[illegible]	[illegible]	[illegible]
Ctps1	Ctps1	[illegible]	[illegible]	[illegible]	[illegible]	[illegible]
Map3k12	Map3k12	[illegible]	[illegible]	[illegible]	[illegible]	[illegible]
Obsl1	Obsl1	[illegible]	[illegible]	[illegible]	[illegible]	[illegible]
Inka1	Inka1	[illegible]	[illegible]	[illegible]	[illegible]	[illegible]
Gng7	Gng7	[illegible]	[illegible]	[illegible]	[illegible]	[illegible]
Bax	Bax	[illegible]	[illegible]	[illegible]	[illegible]	[illegible]
Bmpr1b	Bmpr1b	[illegible]	[illegible]	[illegible]	[illegible]	[illegible]
Dcun1d4	Dcun1d4	[illegible]	[illegible]	[illegible]	[illegible]	[illegible]
Dbp	Dbp	[illegible]	[illegible]	[illegible]	[illegible]	[illegible]
Eaf1	Eaf1	[illegible]	[illegible]	[illegible]	[illegible]	[illegible]
Ednrb	Ednrb	[illegible]	[illegible]	[illegible]	[illegible]	[illegible]
Ncbp3	Ncbp3	[illegible]	[illegible]	[illegible]	[illegible]	[illegible]
Trappc11	Trappc11	[illegible]	[illegible]	[illegible]	[illegible]	[illegible]
Chac2	Chac2	[illegible]	[illegible]	[illegible]	[illegible]	[illegible]
Tp53i3	Tp53i3	[illegible]	[illegible]	[illegible]	[illegible]	[illegible]
Acbd4	Acbd4	[illegible]	[illegible]	[illegible]	[illegible]	[illegible]
Vps53	Vps53	[illegible]	[illegible]	[illegible]	[illegible]	[illegible]
Dctn1	Dctn1	[illegible]	[illegible]	[illegible]	[illegible]	[illegible]
Rmdn3	Rmdn3	[illegible]	[illegible]	[illegible]	[illegible]	[illegible]
Cdh11	Cdh11	[illegible]	[illegible]	[illegible]	[illegible]	[illegible]
Des	Des	[illegible]	[illegible]	[illegible]	[illegible]	[illegible]
Ywhag	Ywhag	[illegible]	[illegible]	[illegible]	[illegible]	[illegible]
Gtse1	Gtse1	[illegible]	[illegible]	[illegible]	[illegible]	[illegible]
Mustn1	Mustn1	[illegible]	[illegible]	[illegible]	[illegible]	[illegible]
Sdc2	Sdc2	[illegible]	[illegible]	[illegible]	[illegible]	[illegible]
Kcnt1	Kcnt1	[illegible]	[illegible]	[illegible]	[illegible]	[illegible]
Cyb561d2	Cyb561d2	[illegible]	[illegible]	[illegible]	[illegible]	[illegible]
Eif3d	Eif3d	[illegible]	[illegible]	[illegible]	[illegible]	[illegible]
Filip1	Filip1	[illegible]	[illegible]	[illegible]	[illegible]	[illegible]
Phlda1	Phlda1	[illegible]	[illegible]	[illegible]	[illegible]	[illegible]
Casp4	Casp4	[illegible]	[illegible]	[illegible]	[illegible]	[illegible]
Grb10	Grb10	[illegible]	[illegible]	[illegible]	[illegible]	[illegible]
Foxred2	Foxred2	[illegible]	[illegible]	[illegible]	[illegible]	[illegible]
Ccdc80	Ccdc80	[illegible]	[illegible]	[illegible]	[illegible]	[illegible]
Cyth2	Cyth2	[illegible]	[illegible]	[illegible]	[illegible]	[illegible]
Ift22	Ift22	[illegible]	[illegible]	[illegible]	[illegible]	[illegible]
Naxe	Naxe	[illegible]	[illegible]	[illegible]	[illegible]	[illegible]
Hacl1	Hacl1	[illegible]	[illegible]	[illegible]	[illegible]	[illegible]
Inava	Inava	[illegible]	[illegible]	[illegible]	[illegible]	[illegible]
Tceal7	Tceal7	[illegible]	[illegible]	[illegible]	[illegible]	[illegible]
Lca5l	Lca5l	[illegible]	[illegible]	[illegible]	[illegible]	[illegible]
Dalrd3	Dalrd3	[illegible]	[illegible]	[illegible]	[illegible]	[illegible]
Cyp1b1	Cyp1b1	[illegible]	[illegible]	[illegible]	[illegible]	[illegible]
Cyb561a3	Cyb561a3	[illegible]	[illegible]	[illegible]	[illegible]	[illegible]
Nfkbie	Nfkbie	[illegible]	[illegible]	[illegible]	[illegible]	[illegible]
Angpt1	Angpt1	[illegible]	[illegible]	[illegible]	[illegible]	[illegible]
Cuedc1	Cuedc1	[illegible]	[illegible]	[illegible]	[illegible]	[illegible]
Klhl15	Klhl15	[illegible]	[illegible]	[illegible]	[illegible]	[illegible]
Slc25a32	Slc25a32	[illegible]	[illegible]	[illegible]	[illegible]	[illegible]
Kdm4b	Kdm4b	[illegible]	[illegible]	[illegible]	[illegible]	[illegible]
Hyou1	Hyou1	[illegible]	[illegible]	[illegible]	[illegible]	[illegible]
Smarca5	Smarca5	[illegible]	[illegible]	[illegible]	[illegible]	[illegible]
Aqr	Aqr	[illegible]	[illegible]	[illegible]	[illegible]	[illegible]
Ca4	Ca4	[illegible]	[illegible]	[illegible]	[illegible]	[illegible]
Nde1	Nde1	[illegible]	[illegible]	[illegible]	[illegible]	[illegible]
Kti12	Kti12	[illegible]	[illegible]	[illegible]	[illegible]	[illegible]
Lsm12	Lsm12	[illegible]	[illegible]	[illegible]	[illegible]	[illegible]
Bace1	Bace1	[illegible]	[illegible]	[illegible]	[illegible]	[illegible]
Pcyt2	Pcyt2	[illegible]	[illegible]	[illegible]	[illegible]	[illegible]
Adamts1	Adamts1	[illegible]	[illegible]	[illegible]	[illegible]	[illegible]
Tnfaip2	Tnfaip2	[illegible]	[illegible]	[illegible]	[illegible]	[illegible]
Itga7	Itga7	[illegible]	[illegible]	[illegible]	[illegible]	[illegible]
Wars1	Wars1	[illegible]	[illegible]	[illegible]	[illegible]	[illegible]
Unc5d	Unc5d	[illegible]	[illegible]	[illegible]	[illegible]	[illegible]
Abhd13	Abhd13	[illegible]	[illegible]	[illegible]	[illegible]	[illegible]
Tmem230	Tmem230	[illegible]	[illegible]	[illegible]	[illegible]	[illegible]
Ankrd27	Ankrd27	[illegible]	[illegible]	[illegible]	[illegible]	[illegible]
Med6	Med6	[illegible]	[illegible]	[illegible]	[illegible]	[illegible]
Stk38	Stk38	[illegible]	[illegible]	[illegible]	[illegible]	[illegible]
Dnmt3b	Dnmt3b	[illegible]	[illegible]	[illegible]	[illegible]	[illegible]
Josd1	Josd1	[illegible]	[illegible]	[illegible]	[illegible]	[illegible]
Rbm7	Rbm7	[illegible]	[illegible]	[illegible]	[illegible]	[illegible]
Prss30	Prss30	[illegible]	[illegible]	[illegible]	[illegible]	[illegible]
Ndufa10	Ndufa10	[illegible]	[illegible]	[illegible]	[illegible]	[illegible]
Esco1	Esco1	[illegible]	[illegible]	[illegible]	[illegible]	[illegible]
Coro1b	Coro1b	[illegible]	[illegible]	[illegible]	[illegible]	[illegible]
Rbl2	Rbl2	[illegible]	[illegible]	[illegible]	[illegible]	[illegible]
Stac2	Stac2	[illegible]	[illegible]	[illegible]	[illegible]	[illegible]
Dynlt1	Dynlt1	[illegible]	[illegible]	[illegible]	[illegible]	[illegible]
Ptpn11	Ptpn11	[illegible]	[illegible]	[illegible]	[illegible]	[illegible]
Gtf2f1	Gtf2f1	[illegible]	[illegible]	[illegible]	[illegible]	[illegible]
Mapk6	Mapk6	[illegible]	[illegible]	[illegible]	[illegible]	[illegible]
Fgf1	Fgf1	[illegible]	[illegible]	[illegible]	[illegible]	[illegible]
Ndufv1	Ndufv1	[illegible]	[illegible]	[illegible]	[illegible]	[illegible]
Hes6	Hes6	[illegible]	[illegible]	[illegible]	[illegible]	[illegible]
Ccz1b	Ccz1b	[illegible]	[illegible]	[illegible]	[illegible]	[illegible]
Zc4h2	Zc4h2	[illegible]	[illegible]	[illegible]	[illegible]	[illegible]
Tmem86a	Tmem86a	[illegible]	[illegible]	[illegible]	[illegible]	[illegible]
Lamtor1	Lamtor1	[illegible]	[illegible]	[illegible]	[illegible]	[illegible]
Ptgfrn	Ptgfrn	[illegible]	[illegible]	[illegible]	[illegible]	[illegible]
Gm2a	Gm2a	[illegible]	[illegible]	[illegible]	[illegible]	[illegible]
Zfp955a	Zfp955a	[illegible]	[illegible]	[illegible]	[illegible]	[illegible]
Coq10a	Coq10a	[illegible]	[illegible]	[illegible]	[illegible]	[illegible]
Uck1	Uck1	[illegible]	[illegible]	[illegible]	[illegible]	[illegible]
[illegible]	[illegible]	[illegible]	[illegible]	[illegible]	[illegible]	[illegible]
Schip1	Schip1	[illegible]	[illegible]	[illegible]	[illegible]	[illegible]
Czib	Czib	[illegible]	[illegible]	[illegible]	[illegible]	[illegible]
Itgal	Itgal	[illegible]	[illegible]	[illegible]	[illegible]	[illegible]
Homez	Homez	[illegible]	[illegible]	[illegible]	[illegible]	[illegible]
Kcnma1	Kcnma1	[illegible]	[illegible]	[illegible]	[illegible]	[illegible]
Extl3	Extl3	[illegible]	[illegible]	[illegible]	[illegible]	[illegible]
Tspo	Tspo	[illegible]	[illegible]	[illegible]	[illegible]	[illegible]
Nars2	Nars2	[illegible]	[illegible]	[illegible]	[illegible]	[illegible]
Dr1	Dr1	[illegible]	[illegible]	[illegible]	[illegible]	[illegible]
RGD15652[illegible]	RGD15652[illegible]	[illegible]	[illegible]	[illegible]	[illegible]	[illegible]
Ddt	Ddt	[illegible]	[illegible]	[illegible]	[illegible]	[illegible]
Rhoc	Rhoc	[illegible]	[illegible]	[illegible]	[illegible]	[illegible]
Stau2	Stau2	[illegible]	[illegible]	[illegible]	[illegible]	[illegible]
Rdm1	Rdm1	[illegible]	[illegible]	[illegible]	[illegible]	[illegible]
Cp	Cp	[illegible]	[illegible]	[illegible]	[illegible]	[illegible]
Cdh9	Cdh9	[illegible]	[illegible]	[illegible]	[illegible]	[illegible]
Mtarc2	Mtarc2	[illegible]	[illegible]	[illegible]	[illegible]	[illegible]
Prtg	Prtg	[illegible]	[illegible]	[illegible]	[illegible]	[illegible]
Zscan25	Zscan25	[illegible]	[illegible]	[illegible]	[illegible]	[illegible]
Gtf3c6	Gtf3c6	[illegible]	[illegible]	[illegible]	[illegible]	[illegible]
Ift27	Ift27	[illegible]	[illegible]	[illegible]	[illegible]	[illegible]
Layn	Layn	[illegible]	[illegible]	[illegible]	[illegible]	[illegible]
Pde4d	Pde4d	[illegible]	[illegible]	[illegible]	[illegible]	[illegible]
Kcng3	Kcng3	[illegible]	[illegible]	[illegible]	[illegible]	[illegible]
Nthl1	Nthl1	[illegible]	[illegible]	[illegible]	[illegible]	[illegible]
Rabgef1	Rabgef1	[illegible]	[illegible]	[illegible]	[illegible]	[illegible]
Lrch4	Lrch4	[illegible]	[illegible]	[illegible]	[illegible]	[illegible]
Dvl2	Dvl2	[illegible]	[illegible]	[illegible]	[illegible]	[illegible]
Smarce1	Smarce1	[illegible]	[illegible]	[illegible]	[illegible]	[illegible]
Rasip1	Rasip1	[illegible]	[illegible]	[illegible]	[illegible]	[illegible]
Ndor1	Ndor1	[illegible]	[illegible]	[illegible]	[illegible]	[illegible]
Csnk2a1	Csnk2a1	[illegible]	[illegible]	[illegible]	[illegible]	[illegible]
Fmr1	Fmr1	[illegible]	[illegible]	[illegible]	[illegible]	[illegible]
Erg	Erg	[illegible]	[illegible]	[illegible]	[illegible]	[illegible]
Hmgb2l1	Hmgb2l1	[illegible]	[illegible]	[illegible]	[illegible]	[illegible]
Xpr1	Xpr1	[illegible]	[illegible]	[illegible]	[illegible]	[illegible]
Exoc5	Exoc5	[illegible]	[illegible]	[illegible]	[illegible]	[illegible]
Retreg1	Retreg1	[illegible]	[illegible]	[illegible]	[illegible]	[illegible]
Gng12	Gng12	[illegible]	[illegible]	[illegible]	[illegible]	[illegible]
Isyna1	Isyna1	[illegible]	[illegible]	[illegible]	[illegible]	[illegible]
Lamtor2	Lamtor2	[illegible]	[illegible]	[illegible]	[illegible]	[illegible]
Plcd3	Plcd3	[illegible]	[illegible]	[illegible]	[illegible]	[illegible]
Cbfa2t2	Cbfa2t2	[illegible]	[illegible]	[illegible]	[illegible]	[illegible]
Bsn	Bsn	[illegible]	[illegible]	[illegible]	[illegible]	[illegible]
Aamdc	Aamdc	[illegible]	[illegible]	[illegible]	[illegible]	[illegible]
Magi3	Magi3	[illegible]	[illegible]	[illegible]	[illegible]	[illegible]
Pqbp1	Pqbp1	[illegible]	[illegible]	[illegible]	[illegible]	[illegible]
Vapa	Vapa	[illegible]	[illegible]	[illegible]	[illegible]	[illegible]

Zfp865	Zfp865	0.0346854780816459	3.81738212356793	0.598001331231906	0.439341626446554	0.856376421365589
Npc2	Npc2	0.0265136774490258	5.27852130040106	0.597671358375059	0.439457890176792	0.856409861082582
Nploc4	Nploc4	0.0192841829439234	6.77008379878139	0.597597760296409	0.439496059981939	0.856409861082582
Prkn	Prkn	0.0470382821567838	3.08264261832614	0.597245110722739	0.439631075799658	0.856416756305108
Fam133b	Fam133b	-0.0284931289840322	5.34572837825566	0.597057680196604	0.439702869234784	0.856416756305108
Kpna3	Kpna3	0.0204316698231294	6.67031862694163	0.597050746753624	0.439705517423301	0.856416756305108
Nup107	Nup107	0.0217045720588587	5.05602306218802	0.596820169641177	0.439793853821765	0.856455113802743
Usp12	Usp12	0.023494424378213	5.2193460377494	0.59611441309373	0.440064405646453	0.856848250507303
Samhd1	Samhd1	0.0469817533988859	3.23842097859183	0.595409295627427	0.440334967881586	0.857227917591188
Macroh2	Macroh2a1	-0.0192398258620075	7.20753447330217	0.595176948951469	0.440424178148976	0.857227917591188
Pafah2	Pafah2	0.0600957541902865	2.39306314540333	0.594804238469068	0.440567328592545	0.857227917591188
Cat	Cat	0.0353791247005696	6.62826429878651	0.594619421889618	0.440638355831301	0.857227917591188
Sdc4	Sdc4	-0.0580158309810811	3.31610229580555	0.5845878963952	0.440650471723888	0.857227917591188
Prpf19	Prpf19	0.0219122563427198	8.27203473031713	0.594532856473585	0.440671624864496	0.857227917591188
Ppp2r2d	Ppp2r2d	-0.0218710262727714	6.16476362551374	0.594336324347935	0.440747170912732	0.857241223924887
Casp7	Casp7	-0.0421590447065263	3.70730928267488	0.593508355492709	0.441065657156211	0.857726964516529
Pex3	Pex3	0.0314164608935157	4.63532230512292	0.593325385767741	0.441136085984096	0.857730239200302
Pcbp4	Pcbp4	0.0237831502995969	8.53400719027791	0.593105092407086	0.441220904433002	0.857761487162756
Hspb1	Hspb1	0.102591023373634	1.39314688562854	0.592458549905132	0.441469984397102	0.858081158759827
RT1-M3-	RT1-M3-1	-0.0894087845676842	1.2220655589346	0.59232130625459	0.441522885247903	0.858081158759827
Ccdc71	Ccdc71	-0.0253685381173527	5.03010527947998	0.591772487094559	0.441734526315491	0.858358773530351
Xylt1	Xylt1	0.0364549441600549	5.96464031685615	0.591209167199224	0.441951821823537	0.858611075668007
RGD135	RGD135826	-0.229476882474133	5.75304652491845	0.590924218671105	0.442061951476553	0.858611075668007
Ubtd1	Ubtd1	-0.0507733819619207	3.5610442357265	0.590901270987246	0.442070814301538	0.858611075668007
Tppp	Tppp	-0.0486180667087014	3.22092884186591	0.590210856624175	0.442337593353581	0.858885360712429
Actn2	Actn2	-0.0540503861326366	4.02813307715406	0.590086963448726	0.442385492463006	0.858885360712429
Arhgap1	Arhgap17	0.0514115364168976	3.67893075432954	0.590001477984785	0.442418547190734	0.858885360712429
Txnl4b	Txnl4b	0.041704505975824	3.89081595419038	0.589278569542081	0.442698227062347	0.859134456098345
Dusp27	Dusp27	0.173811560550201	-0.531203882221621	0.589091330155043	0.442770710985261	0.859134456098345
Kcnk5	Kcnk5	-0.108572429955703	0.271237769461762	0.589074559589122	0.442777204061249	0.859134456098345
Crip1	Crip1	-0.0876725397092717	3.10984815158423	0.588892713804171	0.442847619115845	0.859134456098345
Elapor2	Elapor2	0.0364037105747995	7.55830201319191	0.588741790783891	0.442906073260175	0.859134456098345
Bcl	Bcl	-0.211236811440078	-0.674565642984275	0.588434021148416	0.443025312694326	0.859134456098345
RGD131	RGD1311703	-0.0252225347788964	7.26981963060918	0.588424895241532	0.443028860345977	0.859134456098345
Hipk2	Hipk2	0.0639963184717698	5.62446829284 78	0.588162856684875	0.443130411217486	0.859197846272039
Ubl3	Ubl3	-0.0325095171572336	6.94115037777004	0.587549147468675	0.443368416144409	0.859625750191702
Col7a1	Col7a1	0.0820517488652103	2.42248934952263	0.587287751840023	0.443469848917779	0.859656890606269
Trn2d1	Trn2d1	-0.0291174236994776	4.90296950539392	0.587066850106751	0.443555596252979	0.859656890606269
Etv6	Etv6	-0.0582597709002176	2.84371697957027	0.586959267884732	0.443597354022932	0.859656890606269
Dsc2	Dsc2	-0.0766212511843734	1.37387306612027	0.586237827985492	0.443877625922056	0.859783115958535
Ripk4	Ripk4	0.101710629670823	1.2913138477934	0.586232382221571	0.443879742108233	0.859783115958535
Armc6	Armc6	-0.0269208685707274	5.16020497425549	0.586142626610759	0.443914629558779	0.859783115958535
Golgb1	Golgb1	0.0659164205423305	6.32179581861987	0.585781147929168	0.444055175423385	0.859821941340615
Baz2b	Baz2b	0.0630892973400812	5.75228578425858	0.584564158960262	0.444528856623424	0.860603886354413
Polr2l	Polr2l	-0.0690888691076268	4.96364129663898	0.584513212644723	0.444548703184035	0.860603886354413
Fam220a	Fam220a	0.0303283363464967	6.0378758661901	0.583766639200036	0.444839694369923	0.860603886354413
Adss	Adss	0.0193498771410338	6.49731073334744	0.583622345371154	0.444895969612172	0.860603886354413
Zc3h12c	Zc3h12c	0.0484286576590813	3.73702555655179	0.583536192344354	0.444929574933347	0.860603886354413
Mfsd11	Mfsd11	0.0440799929105725	4.71542851849622	0.583530688070923	0.444931722093874	0.860603886354413
Tbxa2r	Tbxa2r	0.0793299367036552	1.08737401859168	0.583493752756581	0.444946130595483	0.860603886354413
Cnih2	Cnih2	0.0412073571672151	9.26321543285264	0.583136960052258	0.445085352962611	0.860603886354413
Mfng	Mfng	0.0500754098628788	3.56435931256385	0.58296929006417	0.445150800195425	0.860603886354413
Cox17	Cox17	-0.0504074547639825	3.33595039459034	0.582875586103462	0.445187385233239	0.860603886354413
Aph1a	Aph1a	0.0247449443273616	6.41608262728702	0.582596675268252	0.445257247344587	0.860603886354413
Rpp25l	Rpp25l	0.0460635863474657	3.00152576180571	0.582488082421222	0.445338721290986	0.860603886354413
Ccnt1	Ccnt1	0.0428459289450018	4.23717112323456	0.58196785758079	0.445542093885462	0.860603886354413
Smim26	Smim26	-0.0442610912028242	3.97279958401689	0.581909172580059	0.445564857750967	0.860603886354413
Cgrrf1	Cgrrf1	-0.0519238885165397	2.70976264681432	0.581698803349065	0.445651906401722	0.860603886354413
LOC690	LOC690120	-0.0867456221019882	0.859272160048945	0.581445460896646	0.445746303524292	0.860603886354413
C1qa	C1qa	0.100859230726808	2.39193003955287	0.581376222671878	0.445773390634032	0.860603886354413
Tspan13	Tspan13	-0.0234621989165861	8.03462537722357	0.580854717355663	0.445977493913404	0.860603886354413
Hddc2	Hddc2	0.0444503127127852	3.16468892904298	0.580459634952163	0.446132215090143	0.860603886354413
Il10ra	Il10ra	-0.104111671688597	0.562112667888129	0.580347640391694	0.446176080906534	0.860603886354413
Anxa4	Anxa4	0.0864268141540418	2.08776878775506	0.580342399006085	0.446178142747866	0.860603886354413
Atraid	Atraid	-0.0329283407965133	4.65754407987302	0.580321741450746	0.446186236188872	0.860603886354413
Itgb1bp	Itgb1bp2	0.15584902218867	0.594067971815442	0.580209550435598	0.446230195576012	0.860603886354413
Fance	Fance	-0.0338444744152495	4.81532607410564	0.579960156092362	0.446327768884554	0.860603886354413
Pkdcc	Pkdcc	0.0487563680031784	4.02271359269375	0.579954770926648	0.446330069829765	0.860603886354413
Kcns1	Kcns1	-0.0816097227118047	2.37200731681027	0.579905549797033	0.446349344775447	0.860603886354413
Hsd17b7	Hsd17b7	0.042134176520292	3.54828156626791	0.579844311758535	0.446373352219716	0.860603886354413
Gde1	Gde1	-0.0329483520125432	7.43418211139641	0.579660381116106	0.446444295036071	0.860603886354413
Kcnj5	Kcnj5	-0.101932062380546	0.718506136253975	0.579313935405423	0.446581362050682	0.860603886354413
Ubl5	Ubl5	-0.040329429209683	6.2840368640666	0.579269037719186	0.446598877507323	0.860603886354413
Bag4	Bag4	0.0211513414528544	5.32288336964266	0.579095790017846	0.446666960769792	0.860603886354413
Nicn1	Nicn1	-0.0238042856463835	6.37717523926006	0.578942685619488	0.446727053067182	0.860603886354413
Flvcr2	Flvcr2	0.0630244316891252	2.20629968975501	0.578733915372233	0.446809014002205	0.860603886354413
Cited2	Cited2	0.0247807097064545	6.50056120101223	0.578514736828268	0.446895086238406	0.860603886354413
Mycbp2	Mycbp2	0.0439173880228327	9.2049105058877	0.578509128776375	0.44689728 88791	0.860603886354413
Rpl18a	Rpl18a	-0.0355009179529444	8.73649546090694	0.578327629235801	0.446968584458169	0.860603886354413
Trib1	Trib1	0.0675376357262016	3.13742705649011	0.578213973335792	0.447013239089788	0.860603886354413
Romo1	Romo1	-0.0517272299746386	5.2484472078565	0.577979084413 67	0.447105547305665	0.860603886354413
Srxn3	Srxn3	-0.0249821301798306	7.43426785424376	0.577829189970586	0.447164469296115	0.860603886354413
Th	Th	-0.2031979729809	2.04763719821217	0.577824315080306	0.447166385768667	0.860603886354413
Arl3	Arl3	-0.0317107181862455	6.44549314567843	0.577288712442581	0.447377025719076	0.860790122381111
Tmco3	Tmco3	-0.026797519829888	5.14915512151931	0.576922358037891	0.447521193096044	0.860790122381111
Dgkz	Dgkz	-0.0228108314178346	8.21607629904348	0.576732760679704	0.447595830158452	0.860790122381111
Amelx	Amelx	0.198595473651203	-0.620761689475384	0.576526663426634	0.447676987505856	0.860790122381111
Iba57	Iba57	-0.0443053353428826	2.87275292376956	0.576492534421909	0.447690428857694	0.860790122381111
Vps25	Vps25	-0.0285038649781266	5.66627720586253	0.576442758143351	0.447710039849372	0.860790122381111
Dhx32	Dhx32	-0.0247393046113446	5.82087217928867	0.576351236250503	0.447746084340652	0.860790122381111
Nol10	Nol10	-0.0302929368840987	4.86312245580114	0.575850403815437	0.447943442289066	0.861026870927614
LOC294	LOC294154	0.0180633647486138	6.98918693065836	0.575598893052138	0.448042588764404	0.861094790957113
Sptssb	Sptssb	-0.0912291926626876	1.48628141630951	0.575312694886 62	0.448155482346 58	0.861120340738605
Mul1	Mul1	-0.0238211005112 08	4.97829297256608	0.575181200669086	0.448207359516158	0.861120340738605
Ube2h	Ube2h	0.0156139667174583	7.88104930442861	0.574961511546476	0.448294052229666	0.861120340738605
Cacna1c	Cacna1c	0.046738829021751	6.43648758 06307	0.574851382454018	0.448337520288369	0.861120340738605
Arhgef7	Arhgef7	0.0177249476894219	8.21758714388682	0.574379466872237	0.448523862118979	0.861356353477298
Purb	Purb	-0.0338492397022506	6.05290530909165	0.57381386140507	0.448747365020716	0.861652950656 12
Nhlrc2	Nhlrc2	-0.0600752189982047	4.34294107678176	0.573288647315437	0.448955046959489	0.861724832982307
Ogfrl1	Ogfrl1	-0.0324079210931195	4.49678598967305	0.573280600989362	0.448958229948527	0.861724832982307
Cyb5r1	Cyb5r1	0.040773244564861	4.75601678267568	0.573195247745261	0.448991996416046	0.861724832982307
Slc47a2	Slc47a2	-0.0908931775670744	1.06384042789887	0.573006496494855	0.449066682054427	0.861735618577836
Mctp1	Mctp1	-0.0911433608163197	3.05469264176673	0.572820706749837	0.449140214774642	0.861744188796434
Mthfsd1	Mthfsd1	0.0298824827745313	5.69523565940197	0.572128135926896	0.449414469003261	0.861841728790111
Tdrd1	Tdrd1	-0.111035308026547	-0.0816527323022 81	0.571995889769861	0.449466899569205	0.861841728790111
Nr3c1	Nr3c1	0.0355291168686 86	5.0983114354537	0.571959606598256	0.449481270037612	0.861841728790111
Lyn	Lyn	-0.0559720211614 56	3.67250137522283	0.571666588174907	0.449597697728911	0.861841728790111
Dgkq	Dgkq	-0.0547904890019456	6.21555293704704	0.571521361149166	0.449655000570411	0.861841728790111
LOC690	LOC690276	0.0961222229245324	1.48293029894 53	0.571461608926796	0.449678695828725	0.861841728790111
Fam160a	Fam160a2	-0.0216914838162873	5.82755682795426	0.571350802372415	0.449722642274791	0.861841728790111
Klhl26	Klhl26	-0.0320721295303132	5.0317935559054	0.571196409391483	0.449783886487 02	0.861841728790111
Med20	Med20	0.0423562564067762	4.94831317090697	0.571123727036465	0.449812722439786	0.861841728790111
Ppig	Ppig	0.0305800666415963	6.50328102260837	0.570665032178727	0.449994770887466	0.861915720796481
Spg21	Spg21	0.0274190388382187	6.19565058675461	0.570431173534658	0.450087630373185	0.861915720796481
Slc7a11	Slc7a11	0.102637566300503	5.62154522641564	0.570358905274205	0.450116322236257	0.861915720796481
Nat1	Nat1	0.125917520853912	0.0343584342999043	0.570196295961892	0.450180924144663	0.861915720796481
Slc5a2	Slc5a2	-0.10558170931563	1.34018230734681	0.569819942595022	0.450330474866856	0.861915720796481
Fgf11	Fgf11	0.0375217774403659	4.07783033980538	0.569536628618465	0.450443106583259	0.861915720796481
Arih2	Arih2	-0.0189016538142052	6.11288104571392	0.569477631720645	0.450466566299636	0.861915720796481
Fam43a	Fam43a	-0.0471967860624202	3.93910411985828	0.569405252253745	0.450495350109219	0.861915720796481
Tada1	Tada1	0.0265042242973908	5.07475242466328	0.569368657414003	0.450509904209159	0.861915720796481
LOC361	LOC361985	-0.0272514963276385	5.8205567682556	0.56922920544239	0.450565372252894	0.861915720796481
Hdhd5	Hdhd5	0.0292231777115203	4.20349711802181	0.569092496991104	0.450619759391659	0.861915720796481
Acad9	Acad9	-0.0215244923595436	6.13648965050703	0.568524145580184	0.450845678298925	0.861915720796481
Dbn1	Dbn1	0.021285041481767	9.88997748832567	0.568424531968613	0.450885645420359	0.861915720796481
Trex1	Trex1	0.0461916398662513	4.02388694274743	0.568387814015196	0.450900268246193	0.861915720796481
Ube3a	Ube3a	0.0383800753133764	6.5537269885443	0.568358374273956	0.450911993079 76	0.861915720796481
Hes7	Hes7	0.0440157240865627	4.09257803670045	0.568246316395317	0.450956626219955	0.861915720796481
Fcgbp	Fcgbp	-0.0824082255566286	1.99919085989723	0.568067018882942	0.451028055557989	0.861920209710029
Efcab11	Efcab11	-0.127899248310559	0.244080750646217	0.567458879214627	0.451270460650813	0.861974142042709
Rpusd3	Rpusd3	-0.0548085729302536	3.17835167403227	0.567432932824971	0.451280807436541	0.861974142042709
Fam98c	Fam98c	-0.0400565712502054	4.51682893245171	0.567076229032213	0.451423089758053	0.861974142042709
Pafah1b	Pafah1b1	-0.0210649426889351	9.72296252982291	0.567047836770705	0.451434417931291	0.861974142042709
Gtpbp1	Gtpbp1	0.0177862932272999	6.46230489618675	0.566912195280985	0.451488543266093	0.861974142042709
RGD135	RGD1358…	0.0192792968264094	6.14168603259255	0.566685880371798	0.451578880797553	0.861974142042709
Cacng8	Cacng8	-0.0298494554049213	5.87147340036717	0.566424782845502	0.451683120334307	0.861974142042709
Mea1	Mea1	-0.0326549498048142	6.84658930679609	0.566391021238346	0.451696602964203	0.861974142042709
Galnt2	Galnt2	0.0261187978869625	7.84548896705329	0.566111540872832	0.451808237063106	0.861974142042709
Nhlh2	Nhlh2	-0.0735995954917597	3.97994424830112	0.56584779340227	0.451913626488248	0.861974142042709
Socs2	Socs2	-0.045605297737242	3.63824484537617	0.565557349838429	0.452029727765913	0.861974142042709
Pomgnt	Pomgnt1	-0.0234776354259962	6.7498240441424	0.565464894402481	0.452066695494059	0.861974142042709
Arl1	Arl1	-0.0282970953226681	5.73658977236189	0.565344335130568	0.452114907472836	0.861974142042709
Fbxl12	Fbxl12	-0.0400936862301291	3.06524110120479	0.565286012846336	0.452138233600029	0.861974142042709
Usp42	Usp42	0.0214735853319894	6.23098208841024	0.565241064376863	0.452156212125 51	0.861974142042709
Trim65	Trim65	-0.0514435712277144	3.24777079343862	0.565227499119949	0.452161628187828	0.861974142042709
Slc6a2	Slc6a2	-0.0377097006508851	6.37385555414216	0.564742532417643	0.452355690000374	0.862017595162718
Prkaca	Prkaca	-0.0191685010433972	7.42350557970646	0.564706252813 21	0.452370212385911	0.862017595162718
Lrrc58	Lrrc58	0.0322475012530723	5.50204482289474	0.564558851385762	0.452429221677297	0.862017595162718
Map1s	Map1s	0.0181887531364185	6.7163764122174	0.564345462826559	0.452514668988791	0.862017595162718
Dynll2	Dynll2	-0.028572828274771	8.96683695482977	0.564246037919368	0.452554490396232	0.862017595162718
Aste1	Aste1	-0.0527360386576456	2.52987273222988	0.563892106967145	0.452696290461685	0.862017595162718
Efna1	Efna1	0.0637628511442784	2.24762998111643	0.563863582307782	0.452707721705804	0.862017595162718
Otub1	Otub1	-0.0227545375268063	7.51810361711648	0.563484517441402	0.452859674721346	0.862017595162718
Cd40	Cd40	0.115871576862537	0.494251163048333	0.5633242289 7249	0.452923852452069	0.862017595162718
Lmbrd1	Lmbrd1	-0.0218887693622172	5.69460038683737	0.563316874897687	0.452926901873334	0.862017595162718
Folh1	Folh1	-0.0818332977308645	1.44550517174626	0.563153081252139	0.452992600665644	0.862017595162718
Wnt9a	Wnt9a	0.0686754526862626	1.90948947846104	0.563075803513738	0.453023201304416	0.862017595162718
Usp54	Usp54	0.0429849845206637	5.69833627650544	0.562786722641949	0.453139603375987	0.862017595162718
Adrm1	Adrm1	-0.0253248671228168	6.9053271848974	0.562756655673816	0.453151671609543	0.862017595162718
Nrp1	Nrp1	0.064799215000713	6.33127947482588	0.562300826592733	0.453334687241387	0.862049366172388
Suv39h1	Suv39h1	-0.0250772049205614	4.81915215424699	0.562221382251185	0.453366595646767	0.862049366172388
Fbxo11	Fbxo11	0.0203573947734053	7.50501929865506	0.562198850768674	0.453375646455861	0.862049366172388
Mmp14	Mmp14	0.0465308500615641	5.61305287737327	0.561665932039707	0.453589800335478	0.862325151986406
Cyhr1	Cyhr1	0.0390101142547857	3.71807527229047	0.561276932603789	0.453746204118858	0.862370429786414
Mphosph	Mphosph6	-0.0412721151719773	3.57603908782135	0.561058702719293	0.453833989423474	0.862370429786414
Fgf18	Fgf18	0.0765209152671079	1.36200850688501	0.560969822158093	0.453869750231688	0.862370429786414
Oser1	Oser1	-0.0335086654405283	4.55523299519873	0.560874185 23321	0.453908234309915	0.862370429786414
Trk2	Trk2	-0.0357868607917782	6.9568542277609	0.560594368705 48	0.454020861490304	0.862370429786414
Gas7	Gas7	0.0280059611214631	9.27312277114714	0.560468184393592	0.454071665464319	0.862370429786414
Abhd5	Abhd5	0.0370388382462 71	4.11988798228858	0.560404182399623	0.454097437179067	0.862370429786414
Olig1	Olig1	-0.0352801113723242	7.29440344894908	0.559314191259064	0.454536897352618	0.862968450732781
Hdgf	Hdgf	0.0243069294003129	7.06610200467391	0.559100962517022	0.454622705363018	0.862968450732781
Carf	Carf	-0.0512608542242685	3.54149565442454	0.559000228669277	0.454663346293057	0.862968450732781
Slc2a9	Slc2a9	0.155004655322883	-0.556143371619586	0.55886669924647	0.454717228458638	0.862968450732781
Nfat5	Nfat5	0.0744681810268463	5.94626191304088	0.558763909636686	0.454758887069651	0.862968450732781
LOC100	LOC100912…	-0.0781545285478385	5.46967809337685	0.55855121493272	0.454844569718805	0.863001170374319
Eif3c	Eif3c	0.0273695000900975	8.57170765321446	0.55822115976892	0.454977853483527	0.863122802382728
Nme2	Nme2	-0.0418472626512961	8.16520992057054	0.558025309540935	0.455056971276629	0.863141658652858
Gabbr2	Gabbr2	-0.0267532529785486	8.24515813420573	0.557680036971306	0.455196504051105	0.863178524774507
P3h4	P3h4	0.0631299534893182	4.14378080283789	0.557634874927388	0.455214760071833	0.863178524774507
Phka2	Phka2	0.0732252910492934	2.01891450839733	0.557077807994567	0.455440040052608	0.863445722753 88
Sdccag8	Sdccag8	-0.0347791709588876	5.38191237231316	0.556665224920252	0.455607003294504	0.863445722753 88
Fam3a	Fam3a	0.0499825130349162	4.56752959896944	0.556535962977566	0.455659332154004	0.863445722753 88
Impa2	Impa2	0.0648278100324194	2.29108104204086	0.556325147032766	0.455744 69772285	0.863445722753 88
Lias	Lias	-0.0385499022268405	3.54371274666641	0.556261564562 27	0.455770446951834	0.863445722753 88
Bpgm	Bpgm	-0.0310520762968 12	6.45696456849617	0.556234958948522	0.455781225042555	0.863445722753 88
Axl	Axl	0.0478013478000952	4.18425324974471	0.556015952025035	0.455869944814 72	0.863445722753 88
Nmnat2	Nmnat2	0.0281692944042737	6.32613919391552	0.555918807381168	0.455909306 8448	0.863445722753 88
Heyl	Heyl	-0.0450922012134633	3.5086038136012	0.555440539421085	0.456103174253374	0.863601977768 79
Rhbdd1	Rhbdd1	0.0918806838527459	0.780125980769401	0.555373788366389	0.456130242325585	0.863601977768 79
Iqcb1	Iqcb1	0.0322748782582449	4.99165336003289	0.554952722530036	0.456301045731059	0.863602989024966
Fkbp1b	Fkbp1b	-0.0463009777732614	4.44217106007175	0.554861762197845	0.456337957095983	0.863602989024966
Pdp2	Pdp2	0.0329598276252731	4.16626431164126	0.55475113327001	0.456382855750944	0.863602989024966
Alg3	Alg3	0.0348899691735641	4.13831695681584	0.55461720064266	0.456437221507274	0.863602989024966
Farp2	Farp2	-0.0382108587539433	3.7055058949078	0.554519578913805	0.456476854421708	0.863602989024966
Imp4	Imp4	0.024405403621782	5.59328745547603	0.554323681580 39	0.456556400085022	0.863612482460508
Gatd1	Gatd1	-0.0262955321737268	4.90657580530436	0.554166350312272	0.456620305104 75	0.863612482460508
Stim2	Stim2	0.0198063993436286	6.41140545023186	0.553936910724317	0.456713521546644	0.863657867283644
Pclaf	Pclaf	0.0610072814743807	2.45693080399499	0.553708608706685	0.456806305598661	0.863702420814 44
Nudt12	Nudt12	0.0487005878835301	3.24512517581673	0.553209947421422	0.457009069548529	0.863872307285406
Thbs3	Thbs3	0.0798751924429723	3.88943682059468	0.553083163773834	0.457060664515036	0.863872307285406
Psmd2	Psmd2	-0.0165641858680557	8.34684696775798	0.552697625400057	0.457217526080287	0.863872307285406
Cep20	Cep20	-0.0269801817328471	5.35257502924219	0.552598076430364	0.457263760040254	0.863872307285406
Ahcy	Ahcy	-0.0332605566055974	5.90823342562878	0.552575489935123	0.457267255765 8	0.863872307285406
Hsdl2	Hsdl2	-0.0351076508956231	4.44658140061631	0.552380504481057	0.457346649406283	0.863872307285406
Praf2	Praf2	-0.0242423220581376	6.38378381368428	0.551955276044055	0.457519868223942	0.863872307285406
Nipsnap	Nipsnap2	-0.0342056188977836	5.01141391550775	0.551901261135903	0.457541878864826	0.863872307285406
Tbc1d2	Tbc1d2	0.0797601965716311	1.55142160988161	0.551595841880633	0.457666366211153	0.863872307285406
Nkx2-1	Nkx2-1	-0.14313970693673	3.50901676530918	0.551535689229914	0.457690889179612	0.863872307285406
Gria1	Gria1	-0.025573473347434	7.56217221402607	0.551420379315779	0.457737908054317	0.863872307285406
Fry	Fry	0.0427342618420742	8.38765100094263	0.551311454071 04	0.457782329333984	0.863872307285406
RGD131	RGD131174…	-0.0441438780886476	3.27461133980991	0.551248080350035	0.457808177180517	0.863872307285406
Bai1	Bai1	-0.0282607639521312	8.81546446528049	0.550473955031116	0.458124101025886	0.863872307285406
Cbln4	Cbln4	-0.0949527328286 09	4.73879806974977	0.550224852222613	0.458225824198685	0.863872307285406
Mapkapk	Mapkapk2	0.0325928769486342	5.8771740225059	0.550211388117859	0.458231333933583	0.863872307285406
Slc6a3	Slc6a3	-0.518449599093868	0.698474264096227	0.550111774151503	0.458272028876602	0.863872307285406
Rnf7	Rnf7	-0.0354017100381 41	5.47491743644262	0.550058882461588	0.458293625383324	0.863872307285406
Nr2f1	Nr2f1	0.0466258956465758	8.0929252987332	0.549906904985 41	0.458355735192459	0.863872307285406
Nab1	Nab1	0.0286354564446955	5.3748725865 07	0.549813467349857	0.458393821282582	0.863872307285406
Tbce	Tbce	-0.0248933274943714	5.685267645572	0.549758912488642	0.458416219084534	0.863872307285406
Tars2	Tars2	0.0278411304280524	5.24432645764575	0.549751190370948	0.458419375426687	0.863872307285406
Icam4	Icam4	-0.105952701700066	0.362699038547079	0.548877420110244	0.458776742746479	0.864415194681606
Gid8	Gid8	-0.0205490638306837	5.67398540888064	0.548017782492703	0.459128759689335	0.864820970290394
Ppm1g	Ppm1g	-0.0180927011589892	7.74644328038228	0.547865627638924	0.459191111278975	0.864820970290394
Rplp1	Rplp1	-0.0445558068369514	8.87169754209781	0.547691990802804	0.459262281883672	0.864820970290394
Vcp	Vcp	-0.0165784919907862	9.27830238950061	0.547599830583856	0.459300063787755	0.864820970290394
Mrpl55	Mrpl55	-0.042558507267602	4.29085198654736	0.547374133741954	0.459392611005083	0.864820970290394
Zfp180	Zfp180	0.0306649658449813	7.11707432380069	0.547214142978854	0.459458233262209	0.864820970290394
Fam227b	Fam227b	0.113941459820664	-0.234831085267738	0.547154724812117	0.459482608163423	0.864820970290394
Gba2	Gba2	-0.0334087920456566	7.04689883832544	0.546294371177609	0.459835777588573	0.86501446266311
Arl13b	Arl13b	-0.047147087901974	4.71345819642575	0.546119277932756	0.459907704996819	0.86501446266311
Cstf2t	Cstf2t	0.0193875443737894	6.80948179824322	0.545934619782933	0.459983580657851	0.86501446266311
Zdhhc1	Zdhhc1	-0.0345437770190775	3.87193317172322	0.545752782417801	0.460058312675062	0.86501446266311
Paqr5	Paqr5	0.159009207144272	-0.0278757823288 98	0.545743299549599	0.460062214823086	0.86501446266311
Otulinl	Otulinl	-0.0432573997151661	2.69401283895085	0.545640952618292	0.460104288988282	0.86501446266311
RGD156	RGD15648…	0.0415961790570056	3.90503212126998	0.545512457765469	0.460157121032308	0.86501446266311
Pomt2	Pomt2	0.0368885383531133	4.99382661410171	0.545418256295534	0.460195859091824	0.86501446266311
Mapk8ip	Mapk8ip2	-0.0160434400903356	8.36685327128022	0.545288145486481	0.460249372494683	0.86501446266311
Bzw1	Bzw1	-0.0209083673230237	8.54339371380767	0.545128654372 91	0.460314983212453	0.86501446266311
Spata9	Spata9	-0.0537751640386795	2.48671901480419	0.544852929149634	0.460428444769456	0.86501446266311
Brd2	Brd2	0.0166034714936045	8.14790898696033	0.544851054386754	0.460429216390448	0.86501446266311
Krt75	Krt75	0.0794340581079744	1.12038970131894	0.544528293867117	0.460562089790 37	0.86501446266311
Mir3587-	Mir3587-2	0.0975954300813391	0.650168746832514	0.544397375458374	0.460616003356925	0.86501446266311
Plppr3	Plppr3	0.0291657094753544	8.81948852585525	0.544035700688127	0.460764996849773	0.86501446266311
Gclc	Gclc	0.0209621025477052	5.74326821459396	0.543839256251758	0.460845865039263	0.86501446266311
Adamts5	Adamts5	-0.071512174548505	1.85384073352521	0.543805978321345	0.460859671692557	0.86501446266311
Timp2	Timp2	-0.0231792592106756	8.40894435976336	0.543772992033311	0.460873268774772	0.86501446266311
Hspe1	Hspe1	-0.0287922695960392	6.45174880062383	0.543574223230931	0.460955215561167	0.86501446266311
Txndc5	Txndc5	0.0266543506104703	6.40422316764441	0.543393489157104	0.461029747218117	0.86501446266311
Mien1	Mien1	-0.0532556712203833	5.72465974917846	0.543229066493865	0.461097568944602	0.86501446266311
Sp9	Sp9	-0.0871889551277396	4.76995555037516	0.543085022997232	0.461156997607 69	0.86501446266311
Ascc1	Ascc1	-0.0285844880229536	5.28717834859591	0.543042223762114	0.461174657821191	0.86501446266311
Galc	Galc	-0.0428973205416633	3.01619316119841	0.542480269506598	0.461406626210437	0.865319496496432
Exoc6b	Exoc6b	0.0199051094976869	6.91710440006489	0.542231624653077	0.461509327582031	0.865382026602193
Cdc123	Cdc123	-0.0239800205622881	6.11997561696076	0.541800067274707	0.461687676196128	0.865598346491222
Melk	Melk	0.0624654922382612	2.23827913812222	0.541614217534331	0.461764511273767	0.865600331605043
Aloxe3	Aloxe3	-0.0722168876074696	4.27600556953304	0.541326957430655	0.461883312002549	0.865692967281072
Mreg	Mreg	0.0881076810230473	0.608787304816329	0.540640882460128	0.462167244888107	0.866095003071026
Pcdh18	Pcdh18	0.0338003130973663	5.54768718098785	0.540441682750014	0.462249736438 95	0.866104391338672
Ddx23	Ddx23	0.0173304696405296	6.80918656638347	0.540094522999116	0.462393555284713	0.866104391338672
Mapk14	Mapk14	0.0263655238216721	6.11823148912564	0.539954940937037	0.462451400984936	0.866104391338672
Nt5c3a	Nt5c3a	-0.0239004951837264	6.15846129350407	0.539672132561641	0.462568837103807	0.866104391338672
Mta2	Mta2	0.0173589260130816	7.04327795977305	0.53933017763233	0.462710455172092	0.866104391338672
Katnbl1	Katnbl1	0.0365526511533962	4.1789726879684	0.539085485734979	0.462811978084911	0.866104391338672
Camk2d	Camk2d	-0.0675771853221851	6.39842820728415	0.539022728901386	0.462838021571767	0.866104391338672
Psip1	Psip1	-0.0188082871662873	8.10298517958896	0.538860215647375	0.462905474225715	0.866104391338672
LOC100	LOC100911…	-0.0427638573146602	4.4523037074434	0.538749708512725	0.462951350122 84	0.866104391338672
Lrrc3	Lrrc3	-0.0387487974492841	3.86422427488516	0.53867473108712	0.4629824800522	0.866104391338672
Ehd3	Ehd3	-0.0253025293729227	5.89481469808003	0.538447672149196	0.463076774352558	0.866104391338672
Bcl2l12	Bcl2l12	0.0716897655053202	1.67935254251802	0.538389674326794	0.463100864838 9	0.866104391338672
Brf1	Brf1	0.0230770797248966	5.82761270552707	0.537915867585774	0.463297744175179	0.866104391338672
Epha2	Epha2	0.0793073102044162	1.57246787248877	0.537858773664375	0.463321477224281	0.866104391338672
Hcls1	Hcls1	0.112637548221384	0.451136255612009	0.537672486123448	0.463398929343428	0.866104391338672
Wdr92	Wdr92	-0.0429545481470643	3.60584606282133	0.537663702640131	0.463402579812873	0.866104391338672
Tcta	Tcta	-0.0285622279608206	5.20977875731007	0.537505956615239	0.463445872949553	0.866104391338672
Igsf8	Igsf8	-0.0315042554057966	6.54194352130215	0.537422441679723	0.463502917593 85	0.866104391338672
Kazald1	Kazald1	-0.113243222256743	0.476957282905252	0.537266944184182	0.463567605644985	0.866104391338672
Sptlc1	Sptlc1	-0.0269233819845116	4.80583891456864	0.537125444252977	0.463626483118251	0.866104391338672
B3gnt7	B3gnt7	0.0297470954451235	4.91994228845684	0.536983431661341	0.463685585893322	0.866104391338672
Cacng3	Cacng3	-0.0622873168732148	4.27190113163615	0.536863476058073	0.463735518354257	0.866104391338672
Flot2	Flot2	-0.0217154855137255	6.80805382247387	0.536783509816757	0.463768809692527	0.866104391338672
Nkd1	Nkd1	0.0423690591864494	4.26896145556385	0.536262916852316	0.463985634807302	0.866379641647817
Tmem132	Tmem132b	-0.0350060615580106	6.16771311682511	0.535701649510912	0.464219581854919	0.866686775819815
Phf7	Phf7	0.0472951298815559	3.16533950294592	0.535348704125887	0.464366792705057	0.866703410187676
Pidd1	Pidd1	0.0606414694475784	2.57505587556731	0.535347200966378	0.46436741982084	0.866703410187676
Plxnd1	Plxnd1	0.0526633624804977	7.2338404181835	0.535126815565832	0.4644593788936	0.866745388940389
Nod2	Nod2	-0.112485295099093	-0.295529249948413	0.534951787583751	0.464532432624969	0.866752080432938
Bard1	Bard1	0.0648006408507171	2.00886300746741	0.534767267646256	0.464609413723 78	0.866766096745157
Acot2	Acot2	-0.0427494484509758	3.29786397588075	0.534550254040927	0.464700095296236	0.866805664376011
Gnas	Gnas	-0.0318018030043627	10.9200325025214	0.534329672472111	0.464792241548383	0.866847951808461
Ttk	Ttk	0.0605350301816179	2.81661514256811	0.534071296939 16	0.464900213053557	0.866919736701424
Fus	Fus	-0.0266728297499541	8.62214567311915	0.533709258950982	0.465051571061582	0.867072392727938
Calr	Calr	0.0300189210115159	9.23000342616087	0.533452587759826	0.465158921670064	0.867142965139308
Gimap1	Gimap1	0.107396187681744	0.277488725914965	0.533036279292133	0.465332134456987	0.867156414656046
Hs3st5	Hs3st5	-0.0394365193782436	5.58397443158945	0.532781190624107	0.465439930498504	0.867156414656046
Klf1	Klf1	-0.0986955137535135	0.396727159186945	0.532780956459472	0.465440029333286	0.867156414656046
Chat	Chat	-0.23205301167702	0.0396682786476532	0.532771142841085	0.465444137929914	0.867156414656046
Slc9a3	Slc9a3	-0.188118843540417	-0.611358017505605	0.53241885048152	0.465591696087386	0.867189610787204
Fkbp8	Fkbp8	-0.0232873690636516	8.26192860740452	0.532334182502179	0.465627170122776	0.867189610787204
Cnot3	Cnot3	0.0274916289179414	5.8429574408638	0.532222841524892	0.465673827112882	0.867189610787204
B3gnt5	B3gnt5	0.0455139782716932	4.05445729914412	0.531969449280709	0.465780037042688	0.867189610787204
Mif4gd	Mif4gd	-0.0488865121041281	2.86873756970437	0.531899242096426	0.465809471146577	0.867189610787204
Nudt9	Nudt9	-0.0299968185587582	5.31449857606544	0.531383902598008	0.466025618508941	0.867370309050721
Csnk1g1	Csnk1g1	0.0579268579682948	2.91916561151632	0.531179385415975	0.466111443032921	0.867370309050721
Srxn12	Srxn12	0.0275532346572879	4.79411079149276	0.531106066545661	0.466142217038941	0.867370309050721
Slc25a37	Slc25a37	0.0922116943738975	0.776307252459198	0.530926082588831	0.466217774775685	0.867370309050721
Riok2	Riok2	0.0302046701064143	4.02580742128695	0.530831580190 54	0.466257454361153	0.867370309050721
Smap2	Smap2	-0.017323156768113	7.85048981267973	0.530673989379565	0.466323628143162	0.867370309050721
Aspa	Aspa	0.127355768073924	0.814654943414248	0.530367603230433	0.466452352421227	0.867394911181255
Man2c1	Man2c1	-0.0440400932098219	6.60525904003493	0.530180214649 32	0.466531103366773	0.867394911181255
Apool	Apool	0.0588414540367487	2.43211450453073	0.529753234746934	0.466710623184663	0.867394911181255
Gtf2f2	Gtf2f2	0.0309932061940361	4.49457188979671	0.529718152729142	0.466725377599425	0.867394911181255
Serpinf2	Serpinf2	0.14260107 1151491	-0.493275971041478	0.529538011497507	0.466801153800258	0.867394911181255
Rnps1	Rnps1	-0.0177445144479495	7.90383722914203	0.529506765711719	0.466814298816523	0.867394911181255
Zic4	Zic4	-0.127977512703449	4.42432788253056	0.529388356913252	0.466864121313683	0.867394911181255
Zfp91	Zfp91	-0.0179014185609364	7.23979006055107	0.529319678988935	0.466893021038175	0.867394911181255
Ascc2	Ascc2	-0.0297808423887866	4.59782889459673	0.529113057847141	0.466979987203278	0.867427318783404
F8	F8	0.0755356668292213	4.74153304957186	0.528303553261154	0.467320955349974	0.867931461729924
Fosl2	Fosl2	0.0488394948740652	4.2794808849607	0.527999291785 42	0.467449215336086	0.868060461340726
Nphp1	Nphp1	0.0284589881341274	4.74917010748697	0.527292880798556	0.467751016064774	0.868352442372146
Usp51	Usp51	-0.0802771447513916	1.25538960824342	0.527138115765055	0.467812546328937	0.868352442372146
Grwd1	Grwd1	0.0287269218857023	4.51452952455553	0.527106226203244	0.467826009267067	0.868352442372146
Pgam2	Pgam2	-0.0483370629680925	2.51293537735809	0.526815584011345	0.467948773940 29	0.868451051952789
Dok4	Dok4	0.0324973528609114	7.20398685337105	0.526570513457855	0.468052266185617	0.868513988260508
Phactr1	Phactr1	0.0237829538645351	7.06280881061111	0.526254884312458	0.468185653622808	0.868632327174985
Sfxn2	Sfxn2	0.104385242716295	2.03352191120384	0.525934019803898	0.468321315159 67	0.868754841172755
Rufy1	Rufy1	0.0274144689565496	5.02451889333073	0.525139412290251	0.468657550757143	0.869249332658968
Eif5a2	Eif5a2	-0.0334308436551424	4.85976 64601167	0.524546936288935	0.468908506899815	0.869448175250 54
Ssh3	Ssh3	-0.0659185273012704	4.74158494 33432	0.524351880630249	0.468991174262087	0.869448175250 54
Dgke	Dgke	0.0299847247273982	5.42337941201 93	0.524152139476757	0.469076275684481	0.869448175250 54
Zfand2b	Zfand2b	0.0327149187703027	4.34021668742875	0.524118584752966	0.469090079159 46	0.869448175250 54
Haus3	Haus3	-0.0391073546059485	3.80002127034979	0.524064111993184	0.469113177509548	0.869448175250 54
Calb1	Calb1	-0.0697864749795126	5.58923265938681	0.523830646780112	0.46921219 563136	0.869457972062461
Gpatch2	Gpatch2	-0.0410801303721217	3.10023960158406	0.523612702952695	0.469304661116775	0.869457972062461
Fra10ac1	Fra10ac1	0.0369842895243093	4.00096117191029	0.523545119361157	0.469332915912533	0.869457972062461
Arpc3	Arpc3	0.0343655565794861	6.56897176222431	0.523115638046278	0.469515657080107	0.869604487899595
Asb14	Asb14	-0.112791536865256	0.770728812 29216	0.522727315972517	0.46968059970 89	0.869604487899595

Wdtc1	Wdtc1	0.0182073551634703	7.03718306934069	0.522635090668111	0.469719786790325	0.869604487899595
Rae1	Rae1	-0.0206241071240396	5.89617454218711	0.522613650963486	0.469728897403231	0.869604487899595
Ncl	Ncl	-0.0205452486719062	8.81516265169037	0.522552018308758	0.469755089239663	0.869604487899595
Rffl	Rffl	0.0514503587554314	2.45143328588773	0.522380056439992	0.469828179773386	0.869610769771924
Msl3l2	Msl3l2	0.0377500662710257	3.9648498534114	0.521859994675701	0.470050614152017	0.869646805469853
Zcchc9	Zcchc9	-0.0430647123513916	4.76146329343602	0.521698073667267	0.470118229632713	0.869646805469853
Ninj1	Ninj1	-0.0448520679996616	4.41215376773675	0.521672182988925	0.470129242464014	0.869646805469853
Nck1	Nck1	-0.0405395722614564	4.42640090937197	0.52157316907693	0.470171383284857	0.869646805469853
Mocs3	Mocs3	0.0363447285872397	3.78917201799146	0.521514906482437	0.470196181330418	0.869646805469853
Mtx2	Mtx2	-0.0321980517584816	5.34511933616745	0.52130377329398	0.470286058721882	0.869684178945847
Txlna	Txlna	0.0285879608694134	5.61367110701173	0.520355073492318	0.470690257049752	0.870092857289299
Ankrd46	Ankrd46	-0.0214536302106836	7.87912872664893	0.520342326873818	0.470695691581433	0.870092857289299
Palm2	Palm2	0.0432043508914016	5.36476105538338	0.520294084553598	0.470716260856197	0.870092857289299
Grik5	Grik5	-0.0260584930705725	8.42022774478219	0.520005149978651	0.470839485164818	0.870153822589307
Enc1	Enc1	0.0288352713072707	10.9028968825614	0.519745795249446	0.470949711909181	0.870153822589307
Ezh1	Ezh1	0.0265503565012282	5.4405352746931	0.519726271558568	0.470958470103037	0.870153822589307
H3f3b	H3f3b	-0.0257758447423136	9.47204779497354	0.519337254840309	0.47112452718472	0.870185100049665
Kcna2	Kcna2	0.061937958150562	3.34259034228835	0.519219379720028	0.471174862439064	0.870185100049665
Brk1	Brk1	-0.0349431010949563	6.61123867980121	0.518950246251137	0.471289820858436	0.870185100049665
Phlpp1	Phlpp1	0.0221887239648696	6.79375319380309	0.518850033801726	0.471332637452379	0.870185100049665
Abca8a	Abca8a	0.137483364360253	4.51426173329844	0.518657796426965	0.471414790034914	0.870185100049665
Efhd2	Efhd2	-0.0295225732338896	5.9911159866892	0.51855185157973	0.4714600754156	0.870185100049665
Hgh1	Hgh1	0.02633544453003	5.12198417892143	0.518543617938519	0.471463595121883	0.870185100049665
Abcb1b	Abcb1b	0.0765954656063577	1.69516715825282	0.518069875846973	0.471666609004153	0.870421042925738
Clcn5	Clcn5	-0.0586900783778756	3.14933014987318	0.517736528819995	0.47180881462776	0.87056471164013
RT1-S3	RT1-S3	0.0624448588870098	2.22001132152407	0.517396555190402	0.471954355194793	0.870704493532259
Prima1	Prima1	-0.138371066822684	1.95925746464263	0.517169968243195	0.47205139569752	0.870754770418752
Apbb1ip	Apbb1ip	0.0768632186596936	1.69580407316502	0.516966126023412	0.472138723323151	0.870787117650104
Cd164	Cd164	0.0290415911535134	5.89714100942109	0.516496167578836	0.47234015625613	0.870846921146511
Smim29	Smim29	-0.0384330723183205	4.84657199180264	0.516408659081446	0.47237767980328	0.870846921146511
Ptk2	Ptk2	0.01858765648851	7.50485607807041	0.516401994804447	0.472380537173967	0.870846921146511
Gnpda2	Gnpda2	0.0469159291719676	2.93220752057721	0.515785752612707	0.472644917303098	0.871152201366738
Senp7	Senp7	0.0259529031206259	5.92370086125019	0.515505019129975	0.472765436920486	0.871152201366738
Phf14	Phf14	0.0205092345265344	7.16016219700371	0.515478774708244	0.472776706260765	0.871152201366738
Pnrc2	Pnrc2	0.0275482396584702	5.73980317556102	0.515365350917385	0.472825415376737	0.871152201366738
Ephb6	Ephb6	0.0328512139318915	7.32752302375717	0.51503211632847	0.472968567821967	0.871287290818645
Rin1	Rin1	-0.078904852621747	2.21552925537154	0.514575682998203	0.473121329554752	0.871440039689199
Slc22a15	Slc22a15	-0.0459507196478391	4.70163351217985	0.513925914091018	0.473444278781451	0.871874156068276
Crabp1	Crabp1	-0.146285566984467	4.4957293892832	0.513632299915301	0.473570674730848	0.871874156068276
Eif3k	Eif3k	0.0278116643439353	5.31867195993384	0.51358782970452	0.473589823174563	0.871874156068276
Iffo2	Iffo2	0.0337398611251624	4.06324926613453	0.513388191057473	0.473675801113154	0.871874156068276
Grk5	Grk5	0.0341721497792887	4.36763631499099	0.51331712724047	0.473706412117243	0.871874156068276
Actl6b	Actl6b	-0.0200495815622617	7.15474902575698	0.512874961312278	0.473896948813613	0.871970509303678
Slc35a4	Slc35a4	0.0235251206009097	6.22105049288229	0.512871281119068	0.473898525191812	0.871970509303678
Ubr4	Ubr4	0.0532670420634625	8.31749471387918	0.512662389410551	0.473988593935786	0.87198115801516
Bnip1	Bnip1	-0.0314152265591485	4.29314572157572	0.512517359261725	0.474051126858426	0.87198115801516
Ccdc60	Ccdc60	-0.168801469156486	-0.26344701233081	0.512371652592865	0.474112983808195	0.87198115801516
Tmem13	Tmem138	-0.0460682120738194	2.98983941856027	0.511951024203107	0.474295490179801	0.872119712442461
Tceal1	Tceal1	-0.0320524400954442	4.78671768888511	0.511873129362286	0.474329115062478	0.872119712442461
Sfmbt1	Sfmbt1	-0.0340562992099364	5.38533974319423	0.511560485651543	0.474464112969692	0.872219203897079
Wdr48	Wdr48	0.0174903346236894	7.14767098059419	0.511164521091871	0.474635177905076	0.872219203897079
Cep104	Cep104	0.0253676288129277	5.78733889261611	0.511009736353337	0.47470207535663	0.872219203897079
Pex13	Pex13	-0.0342852470473201	4.31231347189666	0.510975829910578	0.474716731653002	0.872219203897079
Prr13	Prr13	0.0356211979195149	4.77469491326666	0.51082080347149	0.474783709036661	0.872219203897079
Rft1	Rft1	-0.0410488780148835	3.35678621832793	0.510777065976782	0.474802663530413	0.872219203897079
Slc46a1	Slc46a1	-0.0265531183365326	4.90571870937248	0.510444360908524	0.474946558900491	0.872242359148221
Ddhd2	Ddhd2	-0.0684368661388837	5.29955039780771	0.510330638700897	0.474995773126811	0.872242359148221
Akr7a2	Akr7a2	0.0400882726223772	4.45909928414529	0.510204754991122	0.475050232650427	0.872242359148221
Aldh16a1	Aldh16a1	-0.0344477694966436	4.18202537040858	0.510101547073305	0.475094900438512	0.872242359148221
Trim39	Trim39	0.0498547681403892	4.5595473129569	0.509927652330183	0.475170089490731	0.872252053342512
Sidt2	Sidt2	-0.0228159421485491	6.98192765037373	0.509687563019021	0.475274128941922	0.872261107307466
Optc	Optc	-0.102538043710898	0.583688304328131	0.509593580007114	0.475314840793737	0.872261107307466
Mcm3ap	Mcm3ap	0.0317874295874934	6.79812050995498	0.509320073482016	0.475433289279598	0.872261687230239
Nectin1	Nectin1	-0.0231002646249731	7.9276317462013	0.509270169034686	0.475454965096815	0.872261687230239
Sntb2	Sntb2	0.087946315394578	0.722593451886219	0.509106342346826	0.475525969732742	0.872263676009906
Ap2s1	Ap2s1	-0.0370204590606835	7.41994543142339	0.508911328337462	0.475610513842464	0.872290516127065
Prdx4	Prdx4	0.0464407655063037	4.76288307235193	0.508549494436139	0.475767443878743	0.872298822310581
Ramp2	Ramp2	0.0320368052890755	5.80590203956166	0.508450197353085	0.475810524484972	0.872298822310581
Sesn1	Sesn1	-0.0306549113862617	5.14595400784949	0.508417341993265	0.475824780367541	0.872298822310581
Jam2	Jam2	-0.0342179757876715	5.31460311810935	0.508251780759892	0.475896627867939	0.872302368651774
Fgfr3	Fgfr3	-0.0369934829896837	5.74081361947102	0.507846037996885	0.476072779303432	0.872497072175223
Flad1	Flad1	0.0229839178629736	5.10242442942121	0.507237225622447	0.476337291964047	0.8727418715732
Pth1r	Pth1r	0.0771169883930785	4.0949264548065	0.507216620022135	0.476346248730744	0.8727418715732
Nmd3	Nmd3	0.0253651121122692	4.73478170942513	0.506632235697253	0.476600380990905	0.872928459342853
Ada	Ada	0.0911318103220423	2.35961206326601	0.506191714070368	0.476792097436436	0.872928459342853
Zdhhc12	Zdhhc12	-0.037133043994298	3.60101146960779	0.506142142963739	0.476813678808157	0.872928459342853
Dnaja1	Dnaja1	-0.0219547027288815	8.725646059906	0.50597009991948	0.476888592147197	0.872928459342853
Fas	Fas	-0.113634452583768	-0.31290025674086	0.505926132905508	0.476907739943914	0.872928459342853
Sft2d1	Sft2d1	0.0299799431028965	5.10137013083385	0.505463135904378	0.477109452945816	0.872928459342853
Scmh1	Scmh1	-0.0258242133543783	6.16517303974512	0.505433932012075	0.477122180811362	0.872928459342853
Thbs4	Thbs4	-0.105590908052534	0.44217446890818	0.505402374656683	0.477135925003626	0.872928459342853
Eif3h	Eif3h	-0.0217592194662085	7.58652879670895	0.505268955434346	0.47719409257354	0.872928459342853
Usp19	Usp19	0.0254369548865057	7.02037538914949	0.505209637693362	0.477219952946909	0.872928459342853
Tmie	Tmie	-0.0589787633934736	2.75741169454347	0.505150114088053	0.477245905366584	0.872928459342853
Acss2	Acss2	-0.0298507352906746	5.11561049689416	0.505039869037954	0.477293978960505	0.872928459342853
Sel1l	Sel1l	0.0214859465423299	7.67455426066074	0.504852532399916	0.477375686144668	0.872928459342853
Polr2f	Polr2f	-0.0378574349044767	5.42822252611354	0.504421235637736	0.477563884695822	0.872928459342853
Drap1	Drap1	-0.0242831861658873	6.64944902331473	0.504016956202406	0.47774045403724	0.872928459342853
Nudcd3	Nudcd3	-0.0197008244550206	6.00607606721081	0.503889152744048	0.477796228199967	0.872928459342853
Cacul1	Cacul1	-0.0183484206038586	6.12135983829419	0.503864912321173	0.477806817627176	0.872928459342853
Mob3c	Mob3c	0.0922035930828142	0.292775182094972	0.503725352066216	0.47786779196806	0.872928459342853
Tmem25	Tmem252	0.117150452300549	0.469926441996363	0.503347524993487	0.478032880073977	0.872928459342853
Hpgd	Hpgd	-0.102061951195603	0.906038985484578	0.503134071467301	0.478126265947435	0.872928459342853
Sigirr	Sigirr	0.0836747062574462	0.430126004908886	0.503010040354539	0.478180513993529	0.872928459342853
Bmper	Bmper	0.0314146455126628	4.01877896854112	0.502918460388891	0.47822057526093	0.872928459342853
Fbxo3	Fbxo3	-0.0191765007287316	6.41304509693597	0.502899634852234	0.478228811091331	0.872928459342853
Magohb	Magohb	0.0487330300467154	2.87988121107479	0.502769245424488	0.478277109123857	0.872928459342853
Wwc2	Wwc2	-0.0301811310386896	5.54175148323967	0.502356800588518	0.478466388299137	0.872928459342853
Psmd13	Psmd13	-0.0247448846911982	7.07647137679459	0.502262691540409	0.478507599825343	0.872928459342853
Tomm7	Tomm7	-0.0629196242016025	4.89085600026915	0.502139082393995	0.478561732980378	0.872928459342853
Slc25a10	Slc25a10	-0.0301417990357916	5.19174748181324	0.501722571243872	0.478743777379376	0.872928459342853
Zkscan8	Zkscan8	0.0669703805077739	5.09591308525757	0.501709782234574	0.478749820148067	0.872928459342853
Agl	Agl	0.0273495743093106	6.12882698526606	0.501690241728213	0.478758384672967	0.872928459342853
Uvrag	Uvrag	-0.0275359794375483	4.16491799474661	0.501679709469286	0.478763000787091	0.872928459342853
Crebrf	Crebrf	0.0508966353619078	4.50695991580452	0.5016782731554	0.47876358647647	0.872928459342853
Gar1	Gar1	0.0415343637533804	4.05481805264693	0.501487628592478	0.478847199429537	0.872928459342853
Kcnd1	Kcnd1	0.0444676702993321	3.83120372047921	0.501361322923717	0.478902578239703	0.872928459342853
Csrp2	Csrp2	0.0377035823389865	5.97586642580793	0.500875271963821	0.479115787565239	0.872928459342853
Phosph	Phospho1	0.0906159156601562	0.887507177497708	0.500616661291602	0.479229291829814	0.872928459342853
Sec11a	Sec11a	0.0278876609368919	5.48935681370568	0.500591740183047	0.47924023203222	0.872928459342853
Cr1l	Cr1l	0.0237054040222653	5.18508904899957	0.500527927877981	0.479268290980848	0.872928459342853
Supt6h	Supt6h	0.0272134480751759	7.66359190275382	0.500227835018208	0.479400030567042	0.872928459342853
Ddx1	Ddx1	-0.0159273928352341	7.45994355949503	0.499967427270576	0.479514434710187	0.872928459342853
Acrbp	Acrbp	0.0968591388076866	1.30149909046878	0.499954441684451	0.479520140797985	0.872928459342853
Tef	Tef	-0.0247598824264401	6.52899033352471	0.499630290063323	0.479662611934476	0.872928459342853
Plscr1	Plscr1	0.126899125747487	-0.302980062836682	0.499598848992292	0.47967643749644	0.872928459342853
Pcnp	Pcnp	-0.0227467930684306	5.74852923427347	0.499572532444134	0.479688007948842	0.872928459342853
Dnajc6	Dnajc6	-0.0215151320738652	8.44549999374614	0.499472685729693	0.479731911162771	0.872928459342853
Micos13	Micos13	-0.0414973406186332	5.96957934936929	0.499248972988962	0.479830302922825	0.872928459342853
Abcc8	Abcc8	-0.0627299863165024	4.5059374784991	0.499131890513553	0.479881810486028	0.872928459342853
Mok	Mok	-0.0469105739555076	3.18290094558102	0.499125718336181	0.479884526035407	0.872928459342853
Rbm41	Rbm41	-0.0505919399318866	3.18467937119403	0.498953755749319	0.479960193881435	0.872928459342853
Dok7	Dok7	-0.0641332115936411	1.93487538775077	0.498922527713297	0.479973927096542	0.872928459342853
Slc16a6	Slc16a6	0.063183881000588	3.10837765785508	0.498818835944036	0.480019575660271	0.872928459342853
Pof1b	Pof1b	-0.140210489000515	-0.718247110228327	0.498534981797079	0.480144546696584	0.872982258728224
Pgf	Pgf	0.110086877135963	0.167380652640863	0.498332025647315	0.48023393387132	0.872982258728224
Nmral1	Nmral1	-0.0369405088846566	4.68522440873015	0.498081376720876	0.480344363834288	0.872982258728224
Ndufs3	Ndufs3	-0.0277655142180064	5.84066251861459	0.497985284770429	0.480386710703456	0.872982258728224
Eif1b	Eif1b	0.0273809736019272	7.0236795771851	0.497793535690192	0.480471231114752	0.872982258728224
Thra	Thra	0.0297334655187853	9.70785948262718	0.497788865292833	0.480473290067142	0.872982258728224
Gli2	Gli2	0.0675297857153031	2.84078627520881	0.497647636921755	0.480535557661733	0.872982258728224
Inhbb	Inhbb	0.0454321848681818	4.05383210227748	0.497124113888106	0.480766494484628	0.873185148687221
Pnma3	Pnma3	-0.064093429439138	5.38845448935809	0.497077181586477	0.48078720618337	0.873185148687221
Zfp251	Zfp251	0.0227163121265375	5.4905475860159	0.496439036746452	0.481068697415702	0.873328060779905
Tek	Tek	0.0607998380447626	4.18947501690529	0.496140187442833	0.481201017773082	0.873328060779905
Cdk5	Cdk5	-0.0222415096179083	6.38965498817502	0.496056022442893	0.481238216791063	0.873328060779905
Rad54l	Rad54l	0.0549131239068741	1.86410380180144	0.496021936579069	0.481253283311744	0.873328060779905
Zfyve21	Zfyve21	0.0396479014054736	3.60398252348884	0.495722052312532	0.481385870550574	0.873328060779905
Mxd3	Mxd3	0.0833208167842903	1.35289325218382	0.49564586036258	0.481419522476019	0.873328060779905
Disp2	Disp2	-0.0440531366179721	8.52386483203268	0.495461299563033	0.481501205413184	0.873328060779905
Nop56	Nop56	-0.0198561856788297	7.39693702444189	0.495328794260324	0.481559820184356	0.873328060779905
Cfd	Cfd	0.170348599185508	-0.427920467556234	0.495087686862498	0.481666534630367	0.873328060779905
Sertad4	Sertad4	-0.0376015736543382	6.26770352676542	0.494834485787706	0.481778640358	0.873328060779905
Slbp	Slbp	-0.0238535299506971	5.5658599882387	0.494727240150013	0.481826134818251	0.873328060779905
Prc1	Prc1	0.0477389426326554	4.62206815512412	0.494715155810241	0.481831486932685	0.873328060779905
Rpl3	Rpl3	-0.026162459073298	9.44100399718335	0.494704594520101	0.481836164573446	0.873328060779905
Galnt13	Galnt13	-0.0481486436343123	3.82817369276531	0.494570324775768	0.481851343304389	0.873328060779905
Cdkn1a	Cdkn1a	-0.0481654280637383	3.55272010726216	0.494327930078291	0.482003084001164	0.873369215837955
Mtch2	Mtch2	-0.020999652823256	7.36095012962338	0.494256252208576	0.48203480617096	0.873369215837955
Chmp6	Chmp6	-0.0281698728062216	5.89653154479447	0.494051632294806	0.482125509382572	0.873369215837955
Ap1s3	Ap1s3	-0.0977242327845403	2.14300509718304	0.493945534189988	0.48217256010298	0.873369215837955
Lipt1	Lipt1	0.0747683117790052	1.05474881621157	0.493841863030447	0.482218524317358	0.873369215837955
Sycp3	Sycp3	-0.101303209653149	0.99713212964227	0.493397687310678	0.482415487379891	0.873565939527498
Tmem79	Tmem79	-0.0711057649691173	0.730709459464588	0.493281436903828	0.482467170736875	0.873565939527498
Entpd4	Entpd4	-0.0232805721800016	5.55704746704865	0.492486645851486	0.482820160651094	0.873730502252712
Yipf1	Yipf1	0.0345998560244005	4.98918506434703	0.492409980230513	0.482855560918568	0.873730502252712
Cep85	Cep85	0.0459216884333147	4.93667044328044	0.492358845718115	0.482876960094609	0.873730502252712
Adamts1	Adamts14	-0.0967648882982931	0.842269407318038	0.492158203513881	0.48296615568074	0.873730502252712
Traf3	Traf3	-0.0345836527153347	6.0631983695953	0.492153271835534	0.482968348358551	0.873730502252712
Barhl2	Barhl2	-0.0782690670822386	2.27539227520169	0.491990802239343	0.483040594680269	0.873730502252712
Ctps2	Ctps2	-0.0196463998840471	7.22559030147587	0.491973591010989	0.48304824914211	0.873730502252712
Sv2b	Sv2b	-0.0294537805790296	6.93658861167101	0.491542879324413	0.483239867437074	0.873862287444696
Acp6	Acp6	0.0412145347405336	3.52807821966047	0.491494982917136	0.483261183246646	0.873862287444696
Cops3	Cops3	0.0187052632330058	6.25907736154588	0.4909081152529	0.48352249448544	0.87402600503796
Pde4b	Pde4b	-0.0286716207516126	5.72493757206609	0.490714972475971	0.483608564863563	0.87402600503796
Tor1a	Tor1a	-0.0212928008157296	5.30399028428875	0.490438591614414	0.483731723988114	0.87402600503796
Crhr1	Crhr1	0.0318478134306332	4.55498008819396	0.490299445778241	0.483793758955886	0.87402600503796
Tspan3	Tspan3	-0.0243453046703124	8.16138471701713	0.490244562539758	0.483818230988092	0.87402600503796
Kat5	Kat5	-0.0192621430549826	6.0563619605089	0.490172419517009	0.48385040213658	0.87402600503796
Gsk3b	Gsk3b	0.0313044630594018	6.66752511610268	0.490078780574549	0.48389216434452	0.87402600503796
Smim14	Smim14	0.0198153092581178	6.26172125389677	0.490034019001328	0.483912129742905	0.87402600503796
Scarb2	Scarb2	-0.0291984791983364	7.32833077275626	0.489437197742006	0.484178465085825	0.874038892998823
Rybp	Rybp	0.0179201663254144	7.22580955142574	0.489313098865424	0.484233875385419	0.874038892998823
Cyfip1	Cyfip1	0.0232567119785066	6.49682955155449	0.489049551059189	0.484351584520168	0.874038892998823
Ngef	Ngef	-0.0589907626726116	7.06305821901705	0.489030797584817	0.484359962239589	0.874038892998823
Miga2	Miga2	-0.0209092406866947	5.84141795708235	0.488530556506596	0.484583522727005	0.874038892998823
Ybx2	Ybx2	0.0959105082672986	3.6706251281559	0.488395182109699	0.484644051582144	0.874038892998823
Efr3a	Efr3a	0.028598321225637	5.55662332611663	0.488289685691839	0.48469122999605	0.874038892998823
Arsi	Arsi	0.128224792755	0.0479161855722381	0.488199561845249	0.484731529743586	0.874038892998823
Tp73	Tp73	0.0583199628240356	2.8764068142528	0.488059552229814	0.484794172913293	0.874038892998823
Liph	Liph	0.045231286421985	2.85646603504415	0.487961249298744	0.484838156825245	0.874038892998823
Fbxo25	Fbxo25	-0.0189413254655506	6.52598166185102	0.487827432037936	0.484898040674691	0.874038892998823
Btg3	Btg3	0.0433845174160354	3.35751287292587	0.487723937165214	0.484944364442885	0.874038892998823
Ftcd	Ftcd	0.0947381474585429	2.38231028220865	0.487607293328039	0.484996622173748	0.874038892998823
Kifbp	Kifbp	-0.020086023089645	6.62615458026267	0.487398842239465	0.485089921411202	0.874038892998823
Galt	Galt	-0.055710411054278	3.64753533554572	0.487191411686567	0.485182823445216	0.874038892998823
Dhx15	Dhx15	0.0201748683683965	7.34830885286524	0.487149657527553	0.48520153947247	0.874038892998823
Gemin2	Gemin2	-0.0431074084739472	3.43667976051825	0.486879823909732	0.485322454747337	0.874038892998823
Stub1	Stub1	-0.0248429488239362	6.37978511780712	0.486830744295119	0.485344453192367	0.874038892998823
Ccdc43	Ccdc43	-0.0220870788224967	5.07365775775315	0.486755069335857	0.485378375423206	0.874038892998823
Dhx36	Dhx36	-0.0289682129019196	7.34659596981674	0.486649889440187	0.485425520116291	0.874038892998823
Cyp2t1	Cyp2t1	0.109426500428763	1.02548897288808	0.48651724262055	0.485485009688818	0.874038892998823
Crcp	Crcp	-0.0255554161832816	4.81439882436126	0.486510126215983	0.4854882010375454	0.874038892998823
Antkmt	Antkmt	-0.0355011249630745	5.09475652655515	0.486326322359526	0.485570640034807	0.87411122142754
Odf3b	Odf3b	-0.124348325890238	0.255036577914233	0.485018604212826	0.486157866170096	0.874964249179983
Polr3k	Polr3k	-0.0536500125171056	5.62204696427733	0.484897543249915	0.486212265700049	0.874964249179983
Dnajb14	Dnajb14	0.0373844828098378	4.4106999046222	0.484802785758227	0.486254877279314	0.874964249179983
Srgap3	Srgap3	0.0410384827492621	7.70331083226896	0.484129508948669	0.486557748954415	0.875097817926787
Rnaseh2	Rnaseh2b	-0.0354647734340952	3.94212790579413	0.483956259099946	0.486635737654842	0.875097817926787
Gabrd	Gabrd	-0.0857651573417321	2.24292366643877	0.483908825762157	0.486657093633031	0.875097817926787
Clcn4	Clcn4	-0.0168955016849331	7.27541728589213	0.483901907364427	0.486660208623031	0.875097817926787
Tmem17	Tmem170b	0.0420643087773544	4.32787607289639	0.483858415435861	0.486679791520885	0.875097817926787
Rgs3	Rgs3	-0.0294232886365665	6.01601800562996	0.483520161879866	0.486832129735165	0.875245620672726
Adcyap1	Adcyap1	0.0520631270747953	4.79034072048486	0.4833179233122	0.486923265054162	0.875283327774208
Adam23	Adam23	0.0340420232032375	6.5037186086171	0.483122126964972	0.487011514563063	0.875315855258331
Ppp1r7	Ppp1r7	-0.0187434504552181	6.62980121942313	0.482339334833021	0.487364600522178	0.875824300837566
Mdh2	Mdh2	-0.0226348650108412	7.88131806217152	0.481652882914916	0.487674576476591	0.876046185667871
Sytl5	Sytl5	-0.102145584492689	0.588004475954416	0.481586503669092	0.487704573163625	0.876046185667871
Ring1	Ring1	-0.0324518590008827	4.97252841855299	0.481062923798575	0.487941249923683	0.876046185667871
Plk4	Plk4	0.0474525788232723	2.98869867836857	0.480988137646428	0.487975071498415	0.876046185667871
Sem1	Sem1	-0.0549601026028166	4.97010149201564	0.480888465790585	0.488020153532529	0.876046185667871
Elp4	Elp4	-0.0317786259808255	4.33492424030719	0.480877116976499	0.488025287090176	0.876046185667871
Ap2a2	Ap2a2	-0.0197487461464394	8.6723294846272	0.480467036524402	0.488210864408846	0.876046185667871
Galk2	Galk2	-0.0344759087912207	4.34923777957492	0.480362063322161	0.488258362582723	0.876046185667871
Vrk3	Vrk3	0.0291429466624397	4.54214181986335	0.480334715755639	0.488270743255815	0.876046185667871
Kifc1	Kifc1	-0.0477314288659736	3.57836287616909	0.480242617163782	0.488312441573	0.876046185667871
Pcdhga1	Pcdhga1	-0.117272246023694	3.33909267441143	0.479920546364141	0.4884583080563	0.876046185667871
Tjap1	Tjap1	0.0435708498082054	4.95935767085784	0.479830612360406	0.488499051823557	0.876046185667871
Lpcat2	Lpcat2	0.0543194601133905	3.27111737550875	0.479742358893489	0.488539040630305	0.876046185667871
Mrps22	Mrps22	0.0240912470688947	4.78109643012718	0.479721227810408	0.488548616101129	0.876046185667871
Zfp275	Zfp275	0.0273376643743634	5.68551067068926	0.479692600231502	0.488561589078616	0.876046185667871
Adgrl3	Adgrl3	-0.0264322138247671	7.12998847174074	0.479473309154514	0.48866098282153	0.876046185667871
Pard6a	Pard6a	-0.0400678789849513	3.59112249324044	0.47942782143994	0.488681690490373	0.876046185667871
Dap3	Dap3	-0.0216571640679796	5.6090838296129	0.479101482211433	0.488829590362191	0.876164425085345
Ncan	Ncan	0.0321863043699449	9.50024460894396	0.478657366388534	0.489031104672795	0.876164425085345
Chrdl1	Chrdl1	-0.0504075388542331	3.41540713612671	0.478611771782994	0.489051800716421	0.876164425085345
Dpp8	Dpp8	-0.0189503282622877	8.09889378938894	0.478454382908659	0.48912325297782	0.876164425085345
Itga8	Itga8	-0.0668393732701293	3.59658247828337	0.478201807298413	0.489237954956294	0.876164425085345
Ndufs7	Ndufs7	0.0340513922808801	5.69102110589357	0.478152737784524	0.489259788802083	0.876164425085345
Chchd2	Chchd2	-0.0450789522267017	7.02572488852518	0.478098776236862	0.48928475723691	0.876164425085345
Eral1	Eral1	0.0356143760565549	4.83829680033467	0.478044475727884	0.489309426464269	0.876164425085345
Insig2	Insig2	0.0324177038366231	3.30575327504789	0.477798532160858	0.489421186401771	0.876190232684336
Eno3	Eno3	-0.0589549918552375	2.31772543013468	0.477602821102266	0.48951015122939	0.876190232684336
Aatf	Aatf	0.0279431081110153	4.64651230385	0.477409003255623	0.489598281553069	0.876190232684336
Cyp26b1	Cyp26b1	-0.0553738470502431	5.3977529084057	0.477163743756662	0.489709840645028	0.876190232684336
Heca	Heca	-0.0247769102196681	6.30851185749979	0.477078398034493	0.489748671057304	0.876190232684336
Ddx17	Ddx17	0.0398412210794183	9.03172525296697	0.477072430191924	0.489751386485329	0.876190232684336
Glra2	Glra2	-0.0308748819859947	6.62973143673677	0.476931737812194	0.489815410192614	0.876190232684336
Kif2a	Kif2a	0.0170323087578666	8.00642712382689	0.476745702932028	0.489900088950344	0.876216085125207
Foxq1	Foxq1	0.0531424963002778	3.55394885797733	0.476035016073226	0.490223801889246	0.876590866603816
Foxm1	Foxm1	-0.0410422988516026	3.76811151929337	0.475782105309172	0.490339087046252	0.876590866603816
Coro1a	Coro1a	0.0322777686230524	6.72002587810162	0.475722479788336	0.490366272942485	0.876590866603816
Pkhd1l1	Pkhd1l1	-0.124487196535521	0.16795467510135	0.475681268508817	0.490385064430122	0.876590866603816
Exoc7	Exoc7	0.016659838189444	6.21733073486301	0.475371106654357	0.490526530419386	0.876606819784244
Alcam	Alcam	0.0250506866103531	7.43779974159611	0.475212616485059	0.490598864670293	0.876606819784244
Ankrd24	Ankrd24	0.0469042413650162	3.4437826559208	0.474800804026131	0.490786825383341	0.876606819784244
Zfp414	Zfp414	0.0257544519046181	5.24330465780477	0.474789188998004	0.490792129057127	0.876606819784244
Hspa13	Hspa13	0.024434853460324	5.34783582601738	0.474752660272287	0.490808809491142	0.876606819784244
Atg4b	Atg4b	-0.0158948574238343	6.16198911126226	0.47413771755317	0.491089758351085	0.876606819784244
Kif5a	Kif5a	-0.0261529136791507	9.22613067337578	0.473869294324906	0.491212477076689	0.876606819784244
Chuk	Chuk	-0.0608357074099565	5.03245438880937	0.473740664202467	0.491271302829817	0.876606819784244
Gmeb1	Gmeb1	0.0288184458698445	4.58156386898728	0.473693134509688	0.491293042318062	0.876606819784244
Mrps5	Mrps5	-0.0218072255389876	5.15669854030986	0.47366922894679	0.491303977031667	0.876606819784244
Cyp19a1	Cyp19a1	-0.124815260291184	0.0248899129547185	0.473636917315872	0.491318757435757	0.876606819784244
Gdf10	Gdf10	-0.047508455479509	4.30559267527134	0.473518735867358	0.491372823851206	0.876606819784244
Mgat4a	Mgat4a	0.0312205823293854	5.72267404863473	0.473265792503842	0.491488574981563	0.876606819784244
Armcx1	Armcx1	-0.0305597052999652	4.33255841352539	0.473251086419026	0.49149530661896	0.876606819784244
Carmil3	Carmil3	-0.0626601434196383	7.89911513348224	0.473125557281548	0.491552769323739	0.876606819784244
Usp4	Usp4	0.0150210629515548	7.07710089922941	0.473075305858252	0.491575775757706	0.876606819784244
Stx18	Stx18	-0.0259381890523913	4.85025099791827	0.473035576161323	0.491593966340495	0.876606819784244
Sigmar1	Sigmar1	0.0269477554094488	5.69605146986737	0.472736972788946	0.491730720478364	0.876725378585105
Shh	Shh	-0.0897840330855326	1.42623307234349	0.472240289478137	0.491958332067388	0.876804959664968
Txlng	Txlng	-0.0442048433083071	2.72990902201338	0.47220073129067	0.49197646769736	0.876804959664968
Sec11c	Sec11c	-0.0312945685532363	5.317098654246	0.472179555326086	0.491986176373683	0.876804959664968
Hnrnpu	Hnrnpu	0.0233182177720395	9.57040972086807	0.471936879445444	0.492097460383172	0.876820809258162
Zadh2	Zadh2	-0.0451580118638236	3.73625115782901	0.471841451199239	0.492141232502621	0.876820809258162
Taok3	Taok3	0.0243167501162572	6.1844269293783	0.471621024916011	0.49224236506075	0.876885777966445
Ube2q1	Ube2q1	-0.0158070572236116	6.62987535754396	0.471236571060388	0.492418827456657	0.876945515609392
rnf141	rnf141	-0.0197890605222996	5.98746107411649	0.471123480271416	0.492470768664351	0.876945515609392
Ccdc116	Ccdc116	0.0708125218949481	1.6206213038326	0.471088673274942	0.492486753857098	0.876945515609392
Mks1	Mks1	0.0458359006591424	3.11878293732164	0.470846475733062	0.492598607691678	0.877018456331202
Mns1	Mns1	0.0531795362080451	2.54398982757673	0.470345517440805	0.492828257310848	0.877302205374156
Gfra1	Gfra1	-0.0691756125716166	5.15597389818873	0.4697166920093	0.493117532336059	0.877629697408272
Mettl24	Mettl24	-0.114653440368388	0.176130070810681	0.469592676429283	0.49317921955726	0.877629697408272
Ica1l	Ica1l	-0.0322539646588813	4.69658880172486	0.469366682697599	0.493278694120725	0.877629697408272
Tcam1	Tcam1	0.108312030552367	0.0083249131811243	0.469322303584069	0.493298645341365	0.877629697408272
Alg10	Alg10	0.0320112165832739	5.03292833051265	0.469159239000533	0.493374212372452	0.877648984549629
Cetn3	Cetn3	-0.0281318102020922	5.38387990332999	0.468522002460183	0.493667903419991	0.877786677694112
Pdp1	Pdp1	-0.0278533391282634	5.61276079628002	0.468488499555114	0.493683352430857	0.877786677694112
RGD130	RGD130953	-0.0352483677076585	3.6811126521118	0.468313577983587	0.493764026232121	0.877786677694112
Vps16	Vps16	-0.018022143056876	6.16478671027135	0.467829554049409	0.493987373345677	0.877786677694112
Kif3c	Kif3c	0.0162666276097743	8.56939356036141	0.467710914402652	0.494042144085194	0.877786677694112
Xkr6	Xkr6	0.0605541300928664	2.2011069566367	0.467600550990582	0.494093103203182	0.877786677694112
Azin1	Azin1	-0.0176847834261346	8.64255438432745	0.467598959963278	0.494093839791207	0.877786677694112
Ngb	Ngb	-0.0605400824532096	3.05540078676229	0.467538848515957	0.494121597503563	0.877786677694112
Slc27a2	Slc27a2	-0.0818497995414847	4.14741287890859	0.46751610235269	0.49413210241284	0.877786677694112
Znhit1	Znhit1	-0.0342365314108536	5.52355531497244	0.467353672985823	0.494207128425749	0.877786677694112
Atp13a4	Atp13a4	0.0988881358592501	0.553668474878341	0.467274904943003	0.494243564420029	0.877786677694112
Kcnk4	Kcnk4	-0.0889124207992197	2.42997217911622	0.467076078680256	0.494335392348669	0.877786677694112
Cftr	Cftr	-0.184948740036466	-0.784854441858158	0.466968712273541	0.494385017938063	0.877786677694112
Hps5	Hps5	0.0573225783349332	3.82546209836419	0.466857249103967	0.494436544909371	0.877786677694112
Mcm2	Mcm2	0.0356364915762803	4.46754275067935	0.466535184288041	0.494585479104106	0.877821708776852
Rnf10	Rnf10	0.0186753281085088	8.64337655867956	0.466297126456823	0.494695613955405	0.877821708776852
Dcaf13	Dcaf13	0.0259530068990341	5.15589611161302	0.466256365693269	0.494714475610079	0.877821708776852
Thoc3	Thoc3	-0.0219582001536456	5.91145084552113	0.466114184872929	0.494780277892969	0.877821708776852
Plat	Plat	0.0361953651037235	5.34739225587797	0.466054179873424	0.494808053043809	0.877821708776852
Tacr2	Tacr2	0.0852836290925777	0.995981786258933	0.465639119494099	0.49500024825236	0.878037830174111
Cstb	Cstb	0.0383644013871733	4.90779699473126	0.465450066168756	0.495087821697099	0.878068354809482
Usp32	Usp32	0.0218323117935482	7.19234475254483	0.465000570402594	0.49529617599727	0.878313017043481
Lpin3	Lpin3	-0.0950318886447775	3.12254057032309	0.464702966155983	0.495434198746877	0.87835567640024
Xpo6	Xpo6	0.0167505060012544	7.40229315596557	0.464496757569336	0.4955298720347	0.87835567640024
Tubgcp!	Tubgcp5	0.0321091446539164	4.6372713382481	0.464239903659625	0.495649086417223	0.87835567640024
Stk35	Stk35	0.0274849496753293	4.194178559797	0.46423161487639	0.495652934319542	0.87835567640024
Slc39a12	Slc39a12	-0.190288740062437	1.44522957192317	0.464190068203706	0.495672222291746	0.87835567640024
Wdr37	Wdr37	-0.0201290333265771	6.43059750972609	0.464019741126492	0.495751309561625	0.878371072053449
Htr1b	Htr1b	0.0600687016925686	3.89857254787191	0.463771688695331	0.495866524741988	0.878447082775057
Cyp4f6	Cyp4f6	-0.0686694713526105	2.11230014356521	0.463624266765279	0.495935020523163	0.878447082775057
Afap1l2	Afap1l2	-0.0362661651042026	4.42670353982132	0.46340356512362	0.496037593622183	0.878474880020945
Psmd4	Psmd4	-0.0204648516198595	6.69346247637015	0.463287546256041	0.496091526783167	0.878474880020945
Elp1	Elp1	0.0238291287347492	7.54683472028187	0.463105845625044	0.496176018118433	0.878499812411479
Mab21l1	Mab21l1	-0.100063523364264	3.39438796808898	0.462858017412927	0.496291295351746	0.878526585303812
Nucks1	Nucks1	-0.0179086740409805	7.08755352672944	0.46277061185328	0.496331962796127	0.878526585303812
Pomp	Pomp	-0.033024217088899	6.61228865487281	0.462259212382011	0.496570481476571	0.878654362974461
Fxn	Fxn	-0.0348972938696671	4.4207563988672	0.462094801010551	0.496646589081923	0.878654362974461
Slc29a2	Slc29a2	0.0305300660221716	5.15698728061765	0.461879586552439	0.496746868948246	0.878654362974461
Erich2	Erich2	-0.104211763907022	0.980551027817077	0.46178361935074	0.496791574000354	0.878654362974461
Patl1	Patl1	0.0304841363466188	5.69151363944485	0.461566420606289	0.496892812974371	0.878654362974461
Hexim1	Hexim1	-0.0203326710117886	5.49406204365762	0.461532940921793	0.496908418561582	0.878654362974461
Map6	Map6	0.023725876144936	8.16529201153141	0.461381768084536	0.496978909142432	0.878654362974461
Pip4p1	Pip4p1	-0.0263914445897174	6.28417606029376	0.461039528330708	0.497138548601839	0.878654362974461
Ficd	Ficd	0.0348923967389642	3.36008351936466	0.460877714734089	0.497214057514288	0.878654362974461
Ndufa10	Ndufa10l1	-0.240711220655115	1.40453833922099	0.46051920565192	0.497381421673253	0.878654362974461
Mtch1	Mtch1	-0.0259496145450381	9.46448108025787	0.460515579660729	0.497383114894506	0.878654362974461
Usp53	Usp53	0.0379900433437439	4.18067421854916	0.460508836536242	0.497386263742627	0.878654362974461
Atp1a2	Atp1a2	0.0438327056723364	8.34434176703257	0.460454258358737	0.497411751444708	0.878654362974461
Metap1	Metap1	-0.0183628075752986	6.39351857851302	0.460341415539425	0.497464455403491	0.878654362974461
Caln1	Caln1	0.040714557566532	5.04918156082457	0.46016588291269	0.497546457789659	0.878654362974461
Dipk2a	Dipk2a	0.0289641054803186	5.2425542392046	0.459634340004335	0.497794914437461	0.878654362974461
Mafg	Mafg	0.0263469892379001	5.43315215004524	0.459618150562349	0.497802485083909	0.878654362974461
Wdr70	Wdr70	0.0240805828565711	4.73882408163267	0.459554247263711	0.497832369985712	0.878654362974461
Ndufb3	Ndufb3	-0.0410056263216002	5.14433595273786	0.459527582176349	0.497844861043484	0.878654362974461
Plekha5	Plekha5	-0.0538751495014346	6.13813301197279	0.459183269132633	0.498005862921189	0.878654362974461
Ltk	Ltk	-0.0808505344603934	2.22284351918675	0.459012354597334	0.498085912593749	0.878654362974461
Impdh2	Impdh2	0.021296118293098	6.42411359394782	0.45899938913152	0.498091981594775	0.878654362974461
Clcn3	Clcn3	-0.0193512479641797	7.19451030980279	0.458986640408654	0.498097949262602	0.878654362974461
Hspb8	Hspb8	-0.0682452739662187	2.66408663665322	0.458801745961424	0.498184511813956	0.878654362974461
Gdi1	Gdi1	-0.0182428463431336	10.0387940689066	0.458709882568767	0.498227538584039	0.878654362974461

**Pih1d2**	Pih1d2	[illegible]	[illegible]	[illegible]	[illegible]	[illegible]
**Arl2**	Arl2	[illegible]	[illegible]	[illegible]	[illegible]	[illegible]
**Nudt16**	Nudt16	[illegible]	[illegible]	[illegible]	[illegible]	[illegible]
**Slc3a2**	Slc3a2	[illegible]	[illegible]	[illegible]	[illegible]	[illegible]
**Ist1**	Ist1	[illegible]	[illegible]	[illegible]	[illegible]	[illegible]
**Pofut1**	Pofut1	[illegible]	[illegible]	[illegible]	[illegible]	[illegible]
**Qtrt2**	Qtrt2	[illegible]	[illegible]	[illegible]	[illegible]	[illegible]
**Penk**	Penk	[illegible]	[illegible]	[illegible]	[illegible]	[illegible]
**Dmrtc1a**	Dmrtc1a	[illegible]	[illegible]	[illegible]	[illegible]	[illegible]
**Htr3a**	Htr3a	[illegible]	[illegible]	[illegible]	[illegible]	[illegible]
**Drc7**	Drc7	[illegible]	[illegible]	[illegible]	[illegible]	[illegible]
**Mta1**	Mta1	[illegible]	[illegible]	[illegible]	[illegible]	[illegible]
**Pmf1**	Pmf1	[illegible]	[illegible]	[illegible]	[illegible]	[illegible]
**Fam20c**	Fam20c	[illegible]	[illegible]	[illegible]	[illegible]	[illegible]
**Unc50**	Unc50	[illegible]	[illegible]	[illegible]	[illegible]	[illegible]
**Trmo**	Trmo	[illegible]	[illegible]	[illegible]	[illegible]	[illegible]
**Eif4e**	Eif4e	[illegible]	[illegible]	[illegible]	[illegible]	[illegible]
**Clybl**	Clybl	[illegible]	[illegible]	[illegible]	[illegible]	[illegible]
**Rcor3**	Rcor3	[illegible]	[illegible]	[illegible]	[illegible]	[illegible]
**RGD131**	RGD131124	[illegible]	[illegible]	[illegible]	[illegible]	[illegible]
**Pik3c2g**	Pik3c2g	[illegible]	[illegible]	[illegible]	[illegible]	[illegible]
**Cap1**	Cap1	[illegible]	[illegible]	[illegible]	[illegible]	[illegible]
**Plec**	Plec	[illegible]	[illegible]	[illegible]	[illegible]	[illegible]
**Mfap1a**	Mfap1a	[illegible]	[illegible]	[illegible]	[illegible]	[illegible]
**Septin1**	Septin10	[illegible]	[illegible]	[illegible]	[illegible]	[illegible]
**Ccndbp**	Ccndbp1	[illegible]	[illegible]	[illegible]	[illegible]	[illegible]
**Sorbs3**	Sorbs3	[illegible]	[illegible]	[illegible]	[illegible]	[illegible]
**Fgd1**	Fgd1	[illegible]	[illegible]	[illegible]	[illegible]	[illegible]
**Larp4b**	Larp4b	[illegible]	[illegible]	[illegible]	[illegible]	[illegible]
**Aoc3**	Aoc3	[illegible]	[illegible]	[illegible]	[illegible]	[illegible]
**Emc1**	Emc1	[illegible]	[illegible]	[illegible]	[illegible]	[illegible]
**Ap1b1**	Ap1b1	[illegible]	[illegible]	[illegible]	[illegible]	[illegible]
**Dcps**	Dcps	[illegible]	[illegible]	[illegible]	[illegible]	[illegible]
**Bcas2**	Bcas2	[illegible]	[illegible]	[illegible]	[illegible]	[illegible]
**Eci1**	Eci1	[illegible]	[illegible]	[illegible]	[illegible]	[illegible]
**Ethe1**	Ethe1	[illegible]	[illegible]	[illegible]	[illegible]	[illegible]
**Clcn6**	Clcn6	[illegible]	[illegible]	[illegible]	[illegible]	[illegible]
**Cacnb4**	Cacnb4	[illegible]	[illegible]	[illegible]	[illegible]	[illegible]
**Atrx**	Atrx	[illegible]	[illegible]	[illegible]	[illegible]	[illegible]
**RGD156**	RGD156221	[illegible]	[illegible]	[illegible]	[illegible]	[illegible]
**Terf2ip**	Terf2ip	[illegible]	[illegible]	[illegible]	[illegible]	[illegible]
**Rnf4**	Rnf4	[illegible]	[illegible]	[illegible]	[illegible]	[illegible]
**Rbm22**	Rbm22	[illegible]	[illegible]	[illegible]	[illegible]	[illegible]
**Oxa1l**	Oxa1l	[illegible]	[illegible]	[illegible]	[illegible]	[illegible]
**Drd1**	Drd1	[illegible]	[illegible]	[illegible]	[illegible]	[illegible]
**Mtmr11**	Mtmr11	[illegible]	[illegible]	[illegible]	[illegible]	[illegible]
**Pdap1**	Pdap1	[illegible]	[illegible]	[illegible]	[illegible]	[illegible]
**Slfn13**	Slfn13	[illegible]	[illegible]	[illegible]	[illegible]	[illegible]
**Zfp157**	Zfp157	[illegible]	[illegible]	[illegible]	[illegible]	[illegible]
**Bbof1**	Bbof1	[illegible]	[illegible]	[illegible]	[illegible]	[illegible]
**Crlf2**	Crlf2	[illegible]	[illegible]	[illegible]	[illegible]	[illegible]
**Slc6a6**	Slc6a6	[illegible]	[illegible]	[illegible]	[illegible]	[illegible]
**Cldn3**	Cldn3	[illegible]	[illegible]	[illegible]	[illegible]	[illegible]
**Nol8**	Nol8	[illegible]	[illegible]	[illegible]	[illegible]	[illegible]
**Inpp4b**	Inpp4b	[illegible]	[illegible]	[illegible]	[illegible]	[illegible]
**Snrpf**	Snrpf	[illegible]	[illegible]	[illegible]	[illegible]	[illegible]
**Gpr27**	Gpr27	[illegible]	[illegible]	[illegible]	[illegible]	[illegible]
**Cbfb**	Cbfb	[illegible]	[illegible]	[illegible]	[illegible]	[illegible]
**B2m**	B2m	[illegible]	[illegible]	[illegible]	[illegible]	[illegible]
**Crispld1**	Crispld1	[illegible]	[illegible]	[illegible]	[illegible]	[illegible]
**Cd44**	Cd44	[illegible]	[illegible]	[illegible]	[illegible]	[illegible]
**Tatdn2**	Tatdn2	[illegible]	[illegible]	[illegible]	[illegible]	[illegible]
**Rab14**	Rab14	[illegible]	[illegible]	[illegible]	[illegible]	[illegible]
**Fam122a**	Fam122a	[illegible]	[illegible]	[illegible]	[illegible]	[illegible]
**Syde1**	Syde1	[illegible]	[illegible]	[illegible]	[illegible]	[illegible]
**Ndufb6**	Ndufb6	[illegible]	[illegible]	[illegible]	[illegible]	[illegible]
**Osm**	Osm	[illegible]	[illegible]	[illegible]	[illegible]	[illegible]
**Ccna1**	Ccna1	[illegible]	[illegible]	[illegible]	[illegible]	[illegible]
**Rab38**	Rab38	[illegible]	[illegible]	[illegible]	[illegible]	[illegible]
**Nfe2l3**	Nfe2l3	[illegible]	[illegible]	[illegible]	[illegible]	[illegible]
**Chrm5**	Chrm5	[illegible]	[illegible]	[illegible]	[illegible]	[illegible]
**Pecam1**	Pecam1	[illegible]	[illegible]	[illegible]	[illegible]	[illegible]
**P2ry13**	P2ry13	[illegible]	[illegible]	[illegible]	[illegible]	[illegible]
**Hsd17b8**	Hsd17b8	[illegible]	[illegible]	[illegible]	[illegible]	[illegible]
**Lrrc8b**	Lrrc8b	[illegible]	[illegible]	[illegible]	[illegible]	[illegible]
**Ankrd52**	Ankrd52	[illegible]	[illegible]	[illegible]	[illegible]	[illegible]
**C1qtnf5**	C1qtnf5	[illegible]	[illegible]	[illegible]	[illegible]	[illegible]
**Lrrfip1**	Lrrfip1	[illegible]	[illegible]	[illegible]	[illegible]	[illegible]
**Usp28**	Usp28	[illegible]	[illegible]	[illegible]	[illegible]	[illegible]
**Pcdhga9**	Pcdhga9	[illegible]	[illegible]	[illegible]	[illegible]	[illegible]
**Fut1**	Fut1	[illegible]	[illegible]	[illegible]	[illegible]	[illegible]
**Vwce**	Vwce	[illegible]	[illegible]	[illegible]	[illegible]	[illegible]
**Atp2b1**	Atp2b1	[illegible]	[illegible]	[illegible]	[illegible]	[illegible]
**Hba-a3**	Hba-a3	[illegible]	[illegible]	[illegible]	[illegible]	[illegible]
**Mgmt**	Mgmt	[illegible]	[illegible]	[illegible]	[illegible]	[illegible]
**Slc31a2**	Slc31a2	[illegible]	[illegible]	[illegible]	[illegible]	[illegible]
**Set**	Set	[illegible]	[illegible]	[illegible]	[illegible]	[illegible]
**Ift122**	Ift122	[illegible]	[illegible]	[illegible]	[illegible]	[illegible]
**Zcchc8**	Zcchc8	[illegible]	[illegible]	[illegible]	[illegible]	[illegible]
**Vti1a**	Vti1a	[illegible]	[illegible]	[illegible]	[illegible]	[illegible]
**Nsd2**	Nsd2	[illegible]	[illegible]	[illegible]	[illegible]	[illegible]
**Gtf3c1**	Gtf3c1	[illegible]	[illegible]	[illegible]	[illegible]	[illegible]
**Zdhhc22**	Zdhhc22	[illegible]	[illegible]	[illegible]	[illegible]	[illegible]
**Tufm**	Tufm	[illegible]	[illegible]	[illegible]	[illegible]	[illegible]
**Txndc12**	Txndc12	[illegible]	[illegible]	[illegible]	[illegible]	[illegible]
**Lman2l**	Lman2l	[illegible]	[illegible]	[illegible]	[illegible]	[illegible]
**Ost4**	Ost4	[illegible]	[illegible]	[illegible]	[illegible]	[illegible]
**Slc25a11**	Slc25a11	[illegible]	[illegible]	[illegible]	[illegible]	[illegible]
**Taf1c**	Taf1c	[illegible]	[illegible]	[illegible]	[illegible]	[illegible]
**Zfp689**	Zfp689	[illegible]	[illegible]	[illegible]	[illegible]	[illegible]
**Krt2**	Krt2	[illegible]	[illegible]	[illegible]	[illegible]	[illegible]
**Son**	Son	[illegible]	[illegible]	[illegible]	[illegible]	[illegible]
**Kif19**	Kif19	[illegible]	[illegible]	[illegible]	[illegible]	[illegible]
**Zfp286a**	Zfp286a	[illegible]	[illegible]	[illegible]	[illegible]	[illegible]
**Zfp382**	Zfp382	[illegible]	[illegible]	[illegible]	[illegible]	[illegible]
**Bloc1s6**	Bloc1s6	[illegible]	[illegible]	[illegible]	[illegible]	[illegible]
**Sart1**	Sart1	[illegible]	[illegible]	[illegible]	[illegible]	[illegible]
**Baiap2l2**	Baiap2l2	[illegible]	[illegible]	[illegible]	[illegible]	[illegible]
**Dars**	Dars	[illegible]	[illegible]	[illegible]	[illegible]	[illegible]
**Birc5**	Birc5	[illegible]	[illegible]	[illegible]	[illegible]	[illegible]
**Spa17**	Spa17	[illegible]	[illegible]	[illegible]	[illegible]	[illegible]
**Pdzd2**	Pdzd2	[illegible]	[illegible]	[illegible]	[illegible]	[illegible]
**Spg11**	Spg11	[illegible]	[illegible]	[illegible]	[illegible]	[illegible]
**Timp3**	Timp3	[illegible]	[illegible]	[illegible]	[illegible]	[illegible]
**Rps14**	Rps14	[illegible]	[illegible]	[illegible]	[illegible]	[illegible]
**RGD130**	RGD130813	[illegible]	[illegible]	[illegible]	[illegible]	[illegible]
**Snx18**	Snx18	[illegible]	[illegible]	[illegible]	[illegible]	[illegible]
**Mtf2**	Mtf2	[illegible]	[illegible]	[illegible]	[illegible]	[illegible]
**Serf2**	Serf2	[illegible]	[illegible]	[illegible]	[illegible]	[illegible]
**Casp12**	Casp12	[illegible]	[illegible]	[illegible]	[illegible]	[illegible]
**Utp3**	Utp3	[illegible]	[illegible]	[illegible]	[illegible]	[illegible]
**Ccdc125**	Ccdc125	[illegible]	[illegible]	[illegible]	[illegible]	[illegible]
**Fscn2**	Fscn2	[illegible]	[illegible]	[illegible]	[illegible]	[illegible]
**Mfsd6**	Mfsd6	[illegible]	[illegible]	[illegible]	[illegible]	[illegible]
**Zdhhc18**	Zdhhc18	[illegible]	[illegible]	[illegible]	[illegible]	[illegible]
**Fbxw4**	Fbxw4	[illegible]	[illegible]	[illegible]	[illegible]	[illegible]
**Npepl1**	Npepl1	[illegible]	[illegible]	[illegible]	[illegible]	[illegible]
**Prdx6**	Prdx6	[illegible]	[illegible]	[illegible]	[illegible]	[illegible]
**Aimp1**	Aimp1	[illegible]	[illegible]	[illegible]	[illegible]	[illegible]
**Xpo1**	Xpo1	[illegible]	[illegible]	[illegible]	[illegible]	[illegible]
**Shmt1**	Shmt1	[illegible]	[illegible]	[illegible]	[illegible]	[illegible]
**Rapgef6**	Rapgef6	[illegible]	[illegible]	[illegible]	[illegible]	[illegible]
**Xkr8**	Xkr8	[illegible]	[illegible]	[illegible]	[illegible]	[illegible]
**[illegible]**	[illegible]	[illegible]	[illegible]	[illegible]	[illegible]	[illegible]
**Timm23**	Timm23	[illegible]	[illegible]	[illegible]	[illegible]	[illegible]
**MGC108**	MGC108823	[illegible]	[illegible]	[illegible]	[illegible]	[illegible]
**Ripor2**	Ripor2	[illegible]	[illegible]	[illegible]	[illegible]	[illegible]
**Ddx46**	Ddx46	[illegible]	[illegible]	[illegible]	[illegible]	[illegible]
**Cpsf4**	Cpsf4	[illegible]	[illegible]	[illegible]	[illegible]	[illegible]
**Pgam1**	Pgam1	[illegible]	[illegible]	[illegible]	[illegible]	[illegible]
**Ipcef1**	Ipcef1	[illegible]	[illegible]	[illegible]	[illegible]	[illegible]
**Ywhaq**	Ywhaq	[illegible]	[illegible]	[illegible]	[illegible]	[illegible]
**Slc26a10**	Slc26a10	[illegible]	[illegible]	[illegible]	[illegible]	[illegible]
**Pik3r6**	Pik3r6	[illegible]	[illegible]	[illegible]	[illegible]	[illegible]
**Akt2**	Akt2	[illegible]	[illegible]	[illegible]	[illegible]	[illegible]
**Mrpl23**	Mrpl23	[illegible]	[illegible]	[illegible]	[illegible]	[illegible]
**Dctn2**	Dctn2	[illegible]	[illegible]	[illegible]	[illegible]	[illegible]
**Zfp422**	Zfp422	[illegible]	[illegible]	[illegible]	[illegible]	[illegible]
**Gsto2**	Gsto2	[illegible]	[illegible]	[illegible]	[illegible]	[illegible]
**Cdkn2d**	Cdkn2d	[illegible]	[illegible]	[illegible]	[illegible]	[illegible]
**Gpr26**	Gpr26	[illegible]	[illegible]	[illegible]	[illegible]	[illegible]
**Ankrd13**	Ankrd13c	[illegible]	[illegible]	[illegible]	[illegible]	[illegible]
**Tprg1l**	Tprg1l	[illegible]	[illegible]	[illegible]	[illegible]	[illegible]
**Psmd3**	Psmd3	[illegible]	[illegible]	[illegible]	[illegible]	[illegible]
**Rgcc**	Rgcc	[illegible]	[illegible]	[illegible]	[illegible]	[illegible]
**Nr3c2**	Nr3c2	[illegible]	[illegible]	[illegible]	[illegible]	[illegible]
**Alkbh2**	Alkbh2	[illegible]	[illegible]	[illegible]	[illegible]	[illegible]
**Spice1**	Spice1	[illegible]	[illegible]	[illegible]	[illegible]	[illegible]
**Abca7**	Abca7	[illegible]	[illegible]	[illegible]	[illegible]	[illegible]
**Tgds**	Tgds	[illegible]	[illegible]	[illegible]	[illegible]	[illegible]
**Lhfpl2**	Lhfpl2	[illegible]	[illegible]	[illegible]	[illegible]	[illegible]
**Smarcd2**	Smarcd2	[illegible]	[illegible]	[illegible]	[illegible]	[illegible]
**Pfas**	Pfas	[illegible]	[illegible]	[illegible]	[illegible]	[illegible]
**Klhdc10**	Klhdc10	[illegible]	[illegible]	[illegible]	[illegible]	[illegible]
**H1f0**	H1f0	[illegible]	[illegible]	[illegible]	[illegible]	[illegible]
**Mxd4**	Mxd4	[illegible]	[illegible]	[illegible]	[illegible]	[illegible]
**Etfdh**	Etfdh	[illegible]	[illegible]	[illegible]	[illegible]	[illegible]
**Vmp1**	Vmp1	[illegible]	[illegible]	[illegible]	[illegible]	[illegible]
**Ttc30a1**	Ttc30a1	[illegible]	[illegible]	[illegible]	[illegible]	[illegible]
**Emx2**	Emx2	[illegible]	[illegible]	[illegible]	[illegible]	[illegible]
**Mospd3**	Mospd3	[illegible]	[illegible]	[illegible]	[illegible]	[illegible]
**Abi3**	Abi3	[illegible]	[illegible]	[illegible]	[illegible]	[illegible]
**Pcdh8**	Pcdh8	[illegible]	[illegible]	[illegible]	[illegible]	[illegible]
**Sptbn2**	Sptbn2	[illegible]	[illegible]	[illegible]	[illegible]	[illegible]
**Pias3**	Pias3	[illegible]	[illegible]	[illegible]	[illegible]	[illegible]
**Dtx3**	Dtx3	[illegible]	[illegible]	[illegible]	[illegible]	[illegible]
**Slc44a5**	Slc44a5	[illegible]	[illegible]	[illegible]	[illegible]	[illegible]
**Ppp2ca**	Ppp2ca	[illegible]	[illegible]	[illegible]	[illegible]	[illegible]
**Mbip**	Mbip	[illegible]	[illegible]	[illegible]	[illegible]	[illegible]
**Gabra1**	Gabra1	[illegible]	[illegible]	[illegible]	[illegible]	[illegible]
**Klhl11**	Klhl11	[illegible]	[illegible]	[illegible]	[illegible]	[illegible]
**Mrps17**	Mrps17	[illegible]	[illegible]	[illegible]	[illegible]	[illegible]
**Lmf2**	Lmf2	[illegible]	[illegible]	[illegible]	[illegible]	[illegible]
**Hspd1**	Hspd1	[illegible]	[illegible]	[illegible]	[illegible]	[illegible]
**Tvp23b**	Tvp23b	[illegible]	[illegible]	[illegible]	[illegible]	[illegible]
**Imp3**	Imp3	[illegible]	[illegible]	[illegible]	[illegible]	[illegible]
**Eif2s2**	Eif2s2	[illegible]	[illegible]	[illegible]	[illegible]	[illegible]
**Kif22**	Kif22	[illegible]	[illegible]	[illegible]	[illegible]	[illegible]
**Pelp1**	Pelp1	[illegible]	[illegible]	[illegible]	[illegible]	[illegible]
**Rhno1**	Rhno1	[illegible]	[illegible]	[illegible]	[illegible]	[illegible]
**Trpc4**	Trpc4	[illegible]	[illegible]	[illegible]	[illegible]	[illegible]
**Pop4**	Pop4	[illegible]	[illegible]	[illegible]	[illegible]	[illegible]
**Zfp395**	Zfp395	[illegible]	[illegible]	[illegible]	[illegible]	[illegible]
**Tgfa**	Tgfa	[illegible]	[illegible]	[illegible]	[illegible]	[illegible]
**Wbp1**	Wbp1	[illegible]	[illegible]	[illegible]	[illegible]	[illegible]
**Tssk6**	Tssk6	[illegible]	[illegible]	[illegible]	[illegible]	[illegible]
**Cdk5rap**	Cdk5rap1	[illegible]	[illegible]	[illegible]	[illegible]	[illegible]
**Aoc2-ps**	Aoc2-ps1	[illegible]	[illegible]	[illegible]	[illegible]	[illegible]
**Col11a1**	Col11a1	[illegible]	[illegible]	[illegible]	[illegible]	[illegible]
**Eif2a**	Eif2a	[illegible]	[illegible]	[illegible]	[illegible]	[illegible]
**Tent5c**	Tent5c	[illegible]	[illegible]	[illegible]	[illegible]	[illegible]
**Srpx2**	Srpx2	[illegible]	[illegible]	[illegible]	[illegible]	[illegible]
**Med9**	Med9	[illegible]	[illegible]	[illegible]	[illegible]	[illegible]
**Tnfrsf1a**	Tnfrsf1a	[illegible]	[illegible]	[illegible]	[illegible]	[illegible]
**Kcns2**	Kcns2	[illegible]	[illegible]	[illegible]	[illegible]	[illegible]
**Septin3**	Septin3	[illegible]	[illegible]	[illegible]	[illegible]	[illegible]
**Wdr5b**	Wdr5b	[illegible]	[illegible]	[illegible]	[illegible]	[illegible]
**RGD135**	RGD135963	[illegible]	[illegible]	[illegible]	[illegible]	[illegible]
**Ust**	Ust	[illegible]	[illegible]	[illegible]	[illegible]	[illegible]
**Pip5k1a**	Pip5k1a	[illegible]	[illegible]	[illegible]	[illegible]	[illegible]
**Tmem20**	Tmem208	[illegible]	[illegible]	[illegible]	[illegible]	[illegible]
**Btbd10**	Btbd10	[illegible]	[illegible]	[illegible]	[illegible]	[illegible]
**Gtf3a**	Gtf3a	[illegible]	[illegible]	[illegible]	[illegible]	[illegible]
**RGD130**	RGD130916	[illegible]	[illegible]	[illegible]	[illegible]	[illegible]
**Pttg1ip**	Pttg1ip	[illegible]	[illegible]	[illegible]	[illegible]	[illegible]
**Coa8**	Coa8	[illegible]	[illegible]	[illegible]	[illegible]	[illegible]
**Mog**	Mog	[illegible]	[illegible]	[illegible]	[illegible]	[illegible]
**Ints2**	Ints2	[illegible]	[illegible]	[illegible]	[illegible]	[illegible]
**Anks3**	Anks3	[illegible]	[illegible]	[illegible]	[illegible]	[illegible]
**Fdps**	Fdps	[illegible]	[illegible]	[illegible]	[illegible]	[illegible]
**Eif4a3**	Eif4a3	[illegible]	[illegible]	[illegible]	[illegible]	[illegible]
**Haus4**	Haus4	[illegible]	[illegible]	[illegible]	[illegible]	[illegible]
**Wrap73**	Wrap73	[illegible]	[illegible]	[illegible]	[illegible]	[illegible]
**Irag1**	Irag1	[illegible]	[illegible]	[illegible]	[illegible]	[illegible]
**Map2k2**	Map2k2	[illegible]	[illegible]	[illegible]	[illegible]	[illegible]
**Stpg1**	Stpg1	[illegible]	[illegible]	[illegible]	[illegible]	[illegible]
**Tox2**	Tox2	[illegible]	[illegible]	[illegible]	[illegible]	[illegible]
**Rars2**	Rars2	[illegible]	[illegible]	[illegible]	[illegible]	[illegible]
**Slc37a3**	Slc37a3	[illegible]	[illegible]	[illegible]	[illegible]	[illegible]
**Cpq**	Cpq	[illegible]	[illegible]	[illegible]	[illegible]	[illegible]
**Fam91a1**	Fam91a1	[illegible]	[illegible]	[illegible]	[illegible]	[illegible]
**Tonsl**	Tonsl	[illegible]	[illegible]	[illegible]	[illegible]	[illegible]
**Upf2**	Upf2	[illegible]	[illegible]	[illegible]	[illegible]	[illegible]
**Nae1**	Nae1	[illegible]	[illegible]	[illegible]	[illegible]	[illegible]
**Zcrb1**	Zcrb1	[illegible]	[illegible]	[illegible]	[illegible]	[illegible]
**Lin37**	Lin37	[illegible]	[illegible]	[illegible]	[illegible]	[illegible]
**Ralgapb**	Ralgapb	[illegible]	[illegible]	[illegible]	[illegible]	[illegible]
**Jph1**	Jph1	[illegible]	[illegible]	[illegible]	[illegible]	[illegible]
**Slc38a6**	Slc38a6	[illegible]	[illegible]	[illegible]	[illegible]	[illegible]
**Pnisr**	Pnisr	[illegible]	[illegible]	[illegible]	[illegible]	[illegible]
**Blcap**	Blcap	[illegible]	[illegible]	[illegible]	[illegible]	[illegible]
**Ube2n**	Ube2n	[illegible]	[illegible]	[illegible]	[illegible]	[illegible]
**Fam172a**	Fam172a	[illegible]	[illegible]	[illegible]	[illegible]	[illegible]
**Ctnna2**	Ctnna2	[illegible]	[illegible]	[illegible]	[illegible]	[illegible]
**RGD156**	RGD156454	[illegible]	[illegible]	[illegible]	[illegible]	[illegible]
**Srp68**	Srp68	[illegible]	[illegible]	[illegible]	[illegible]	[illegible]
**Fam13b**	Fam13b	[illegible]	[illegible]	[illegible]	[illegible]	[illegible]
**Adnp**	Adnp	[illegible]	[illegible]	[illegible]	[illegible]	[illegible]
**Wdr13**	Wdr13	[illegible]	[illegible]	[illegible]	[illegible]	[illegible]
**Nolc1**	Nolc1	[illegible]	[illegible]	[illegible]	[illegible]	[illegible]
**Klhl8**	Klhl8	[illegible]	[illegible]	[illegible]	[illegible]	[illegible]
**Sf3b5**	Sf3b5	[illegible]	[illegible]	[illegible]	[illegible]	[illegible]
**Man2b2**	Man2b2	[illegible]	[illegible]	[illegible]	[illegible]	[illegible]
**Copb1**	Copb1	[illegible]	[illegible]	[illegible]	[illegible]	[illegible]
**Lonp1**	Lonp1	[illegible]	[illegible]	[illegible]	[illegible]	[illegible]
**Oprl1**	Oprl1	[illegible]	[illegible]	[illegible]	[illegible]	[illegible]
**Vps37d**	Vps37d	[illegible]	[illegible]	[illegible]	[illegible]	[illegible]
**Rad18**	Rad18	[illegible]	[illegible]	[illegible]	[illegible]	[illegible]
**Mbtps2**	Mbtps2	[illegible]	[illegible]	[illegible]	[illegible]	[illegible]
**Nup43**	Nup43	[illegible]	[illegible]	[illegible]	[illegible]	[illegible]
**Fgfbp3**	Fgfbp3	[illegible]	[illegible]	[illegible]	[illegible]	[illegible]
**Cda**	Cda	[illegible]	[illegible]	[illegible]	[illegible]	[illegible]
**Ugp2**	Ugp2	[illegible]	[illegible]	[illegible]	[illegible]	[illegible]
**Man2b1**	Man2b1	[illegible]	[illegible]	[illegible]	[illegible]	[illegible]
**Cmtr1**	Cmtr1	[illegible]	[illegible]	[illegible]	[illegible]	[illegible]
**Mapk1ip**	Mapk1ip1	[illegible]	[illegible]	[illegible]	[illegible]	[illegible]
**Ralyl**	Ralyl	[illegible]	[illegible]	[illegible]	[illegible]	[illegible]
**Ago2**	Ago2	[illegible]	[illegible]	[illegible]	[illegible]	[illegible]
**Mark2**	Mark2	[illegible]	[illegible]	[illegible]	[illegible]	[illegible]
**S100a11**	S100a11	[illegible]	[illegible]	[illegible]	[illegible]	[illegible]
**Lpar4**	Lpar4	[illegible]	[illegible]	[illegible]	[illegible]	[illegible]
**Zfhx2**	Zfhx2	[illegible]	[illegible]	[illegible]	[illegible]	[illegible]
**Unc5c**	Unc5c	[illegible]	[illegible]	[illegible]	[illegible]	[illegible]
**Ttyh3**	Ttyh3	[illegible]	[illegible]	[illegible]	[illegible]	[illegible]
**Acot5**	Acot5	[illegible]	[illegible]	[illegible]	[illegible]	[illegible]
**Rex1bd**	Rex1bd	[illegible]	[illegible]	[illegible]	[illegible]	[illegible]
**Grm6**	Grm6	[illegible]	[illegible]	[illegible]	[illegible]	[illegible]
**Plcb3**	Plcb3	[illegible]	[illegible]	[illegible]	[illegible]	[illegible]
**Mcrip1**	Mcrip1	[illegible]	[illegible]	[illegible]	[illegible]	[illegible]
**Fdft1**	Fdft1	[illegible]	[illegible]	[illegible]	[illegible]	[illegible]
**Nnat**	Nnat	[illegible]	[illegible]	[illegible]	[illegible]	[illegible]
**Lbh**	Lbh	[illegible]	[illegible]	[illegible]	[illegible]	[illegible]
**Tor1b**	Tor1b	[illegible]	[illegible]	[illegible]	[illegible]	[illegible]
**Abtb1**	Abtb1	[illegible]	[illegible]	[illegible]	[illegible]	[illegible]
**F3**	F3	[illegible]	[illegible]	[illegible]	[illegible]	[illegible]
**Tsc22d4**	Tsc22d4	[illegible]	[illegible]	[illegible]	[illegible]	[illegible]
**Dgcr2**	Dgcr2	[illegible]	[illegible]	[illegible]	[illegible]	[illegible]
**Tfap4**	Tfap4	[illegible]	[illegible]	[illegible]	[illegible]	[illegible]
**Adcy1**	Adcy1	[illegible]	[illegible]	[illegible]	[illegible]	[illegible]
**[illegible]**	[illegible]	[illegible]	[illegible]	[illegible]	[illegible]	[illegible]
**Pacsin2**	Pacsin2	[illegible]	[illegible]	[illegible]	[illegible]	[illegible]
**Dstyk**	Dstyk	[illegible]	[illegible]	[illegible]	[illegible]	[illegible]
**[illegible]**	[illegible]	[illegible]	[illegible]	[illegible]	[illegible]	[illegible]

Jade2	Jade2	0.0672312871110467	1.84459125025853	0.395984688543734	0.529170250856154	0.890724862108771
Sncaip	Sncaip	0.0292107358521522	5.97358672481731	0.395578747558556	0.529381454883246	0.890724862108771
Hdac9	Hdac9	0.0358802848387295	4.84278249577889	0.395409565977076	0.529468952177985	0.890724862108771
Med11	Med11	-0.0398344854785242	3.60388017158978	0.3953295894772	0.529521162422531	0.890724862108771
Tpgs1	Tpgs1	-0.0348867078804727	5.95388812601109	0.39525155953848	0.529551795231385	0.890724862108771
Mynn	Mynn	0.0244913889452493	4.92365563627128	0.395163722891642	0.529597540897401	0.890724862108771
Sec24a	Sec24a	0.0346281530643471	5.05571785199877	0.395139494512009	0.529610151044046	0.890724862108771
Gns	Gns	0.0205552097611006	6.01433195405737	0.395008329725904	0.529678489079885	0.890724862108771
Parvb	Parvb	0.0294157321396212	5.59658932151599	0.394905452672521	0.529732091682487	0.890724862108771
Pigm	Pigm	0.028264982553294	4.01601970252582	0.394812885116032	0.529780331947519	0.890724862108771
Wnt4	Wnt4	-0.04266257505163	3.51209363048509	0.394610312158196	0.529885825868545	0.890761895774096
Slc25a28	Slc25a28	-0.0354001767384125	5.45220310075594	0.394496741933835	0.529945143250089	0.890761895774096
Ric8b	Ric8b	-0.0237267825888934	5.08124342889335	0.394216814435694	0.530091151523855	0.890887297641151
Rnf139	Rnf139	-0.0220322550996076	5.20349952412839	0.394059588708842	0.530173191106881	0.890905172449924
Zc3h13	Zc3h13	0.0298990764367741	6.50494165619904	0.393912314048066	0.530250058963087	0.890914353040996
Cox11	Cox11	0.0248364916342995	4.56063428268547	0.39372245848443	0.530349180336046	0.890960915989342
Syce2	Syce2	0.0557516352093565	1.82954843681213	0.393522686847959	0.530453515815915	0.891016224668171
Ctla2a	Ctla2a	0.0839837586980783	0.704780495038078	0.393255437054151	0.530593150389548	0.891116538904298
Lpcat3	Lpcat3	0.0192422568642737	5.58261249470299	0.393135039982255	0.530656077908066	0.891116538904298
Slc7a8	Slc7a8	0.0323767508312598	5.78216285356404	0.392942015951235	0.530756893124706	0.891166061528322
Nemf	Nemf	0.0235431522544889	6.96417463856054	0.392655589775978	0.530906798486686	0.891297194152601
Morn2	Morn2	0.0807593770615679	0.535065834976735	0.392306664513669	0.531089406298877	0.891297194152601
Brinp1	Brinp1	-0.0252635965668907	6.96676836807388	0.392273062611551	0.531106897104252	0.891297194152601
Knl1	Knl1	0.0481063240848033	3.40507162924334	0.392246632840388	0.531120823415451	0.891297194152601
Bbs5	Bbs5	-0.0363015286979084	3.91291850154742	0.392104382394642	0.531195318102754	0.891302310915555
Ntrk2	Ntrk2	0.0237277866042896	8.80040781925388	0.391770317949735	0.531370312389115	0.891336516126082
Htt	Htt	0.0451025429633815	7.14063721180017	0.39173956672529	0.531386436633581	0.891336516126082
Bud31	Bud31	-0.0267043047491882	4.95498279387677	0.391533862854862	0.531494237799976	0.891336516126082
Capn10	Capn10	0.0194591677398815	6.07728470756009	0.391520092379366	0.53150145707927	0.891336516126082
Exoc3	Exoc3	-0.0169398135344662	6.56073518416982	0.39126489524121	0.531635246487881	0.891441065774666
Zfp598	Zfp598	0.0274738806029226	6.14426942370438	0.391083987253495	0.531730161766268	0.891480412302839
Pold2	Pold2	0.0265009914440178	4.61457441751147	0.390663210987634	0.531950943309949	0.891730742936751
RGD130	RGD130462	-0.0435809383355204	2.83711723778273	0.389935238225555	0.53233239266806	0.892160887536927
Ndufaf4	Ndufaf4	-0.0213487139605358	4.78266496328477	0.388817066472147	0.532395576079242	0.892160887536927
Crebl2	Crebl2	0.0289372460388156	4.22011704174112	0.388731128888956	0.532440765651828	0.892160887536927
Ap5b1	Ap5b1	-0.0317094049378696	3.94667921267892	0.388630751059762	0.532493558506652	0.892160887536927
Kctd16	Kctd16	-0.0625747501356954	1.83365092078389	0.388397548755568	0.532616245738291	0.89224662970954
Ndrg3	Ndrg3	0.0238758288205852	6.82521105166116	0.388140150598877	0.532751721631641	0.892353769740634
H2bc12	H2bc12	0.122075227155749	0.0938879486774402	0.38873764522782	0.532963696349869	0.892575207796022
Tnnt2	Tnnt2	0.132654688030366	2.80276096353737	0.388601751593157	0.533035297547782	0.892575207796022
Htr6	Htr6	-0.0912908042159892	1.27299123665022	0.388481754993066	0.533098537188103	0.892575207796022
Pxylp1	Pxylp1	0.0356911539036341	4.78461834039803	0.388322635622345	0.533182415912714	0.892595867984468
Rngtt	Rngtt	0.018908271821658	6.1337737539805	0.388185371080787	0.533254793178912	0.892597270524892
Fut10	Fut10	-0.0579000615296486	1.55845671847759	0.387906011575112	0.533402149583032	0.892724161029143
Mras	Mras	0.0148254127764004	7.38426445740855	0.387698736290854	0.533511530653683	0.89278748887956
Sox2ip	Sox2ip	0.0183867952588644	6.00893661709218	0.387407543481466	0.533665264327741	0.892924970221625
Polr1d	Polr1d	-0.0322924466279967	5.41242792106589	0.387111695182966	0.533821527952995	0.893066683968827
Phf10	Phf10	0.0217823810806587	5.5925135032428	0.386775270916424	0.533999045387357	0.893244380265189
Pgd	Pgd	-0.0204381645617944	6.34268205411339	0.386484444453224	0.534153138848927	0.893381866741866
Galr1	Galr1	-0.108609023808605	1.00285398738255	0.385894405231184	0.534465410258075	0.893596348805136
Gmeb2	Gmeb2	0.0294484509416394	3.79978362169456	0.385826431643309	0.534501405662622	0.893596348805136
Ppp1r17	Ppp1r17	-0.0842988759750006	1.79540052189401	0.385673809392344	0.534582242649635	0.893596348805136
Kcnn2	Kcnn2	0.059414161836251	4.08926098394083	0.385622489315296	0.534609429525765	0.893596348805136
LOC1003	LOC100363	-0.0268992144264835	5.32251864412526	0.385507255258982	0.53467048404931	0.893596348805136
RGD1308	RGD130811	0.0389012961743392	2.76443493427402	0.385408485824576	0.534722825174264	0.893596348805136
Zmynd11	Zmynd11	-0.016070174923134	8.16401880187422	0.385212999645285	0.53482644703901	0.893596348805136
Hr	Hr	-0.0277165453129244	5.05294847097711	0.384975532998561	0.53495183968187	0.893596348805136
Edem1	Edem1	0.0353292632036982	6.12410356987132	0.384908977900722	0.534987673021789	0.893596348805136
Dcaf7	Dcaf7	-0.0184615023369516	8.81685103450851	0.384815428989796	0.535037300751261	0.893596348805136
Ralgapa	Ralgapa1	-0.0281692457296554	7.28996302547797	0.384605562158299	0.535148865575699	0.893596348805136
Rabif	Rabif	0.0326806178922069	5.20028889968995	0.384096520570395	0.535418960918364	0.893596348805136
Fut8	Fut8	0.024071416453233	6.05654507075075	0.384081089011771	0.535427168397885	0.893596348805136
Cd63	Cd63	0.034193589017777	5.98621684011046	0.383888016013856	0.535529746080625	0.893596348805136
Psd	Psd	-0.0234694857479136	9.51902937049605	0.383761289943794	0.535581153854786	0.893596348805136
Eid1	Eid1	-0.025628912084735	9.40790607468584	0.383667330855552	0.535647068503504	0.893596348805136
Rpia	Rpia	0.0380050085252643	4.19425028971111	0.383568323955036	0.535699684780335	0.893596348805136
Pcdhb9	Pcdhb9	0.037242233219565	3.32867794292456	0.383348574106897	0.535816562125512	0.893596348805136
Wdr44	Wdr44	-0.0271608571623417	4.88088352330525	0.383192362768032	0.535899670315802	0.893596348805136
Atp5f1a	Atp5f1a	-0.0210103491804826	9.57395470782346	0.383062500124524	0.535968775008985	0.893596348805136
Mtss1l	Mtss1l	0.0176225802025134	8.62469435873689	0.382895190277239	0.536057837933514	0.893596348805136
Naa60	Naa60	-0.0142967691682115	6.75940445929044	0.382759582187927	0.536130058574219	0.893596348805136
Zfp410	Zfp410	0.018265297777612	5.30880927762676	0.382507289776578	0.536264368270882	0.893596348805136
Hint1	Hint1	-0.0289474634738264	7.28348881132264	0.382412519345564	0.536314916555518	0.893596348805136
Pcca	Pcca	0.0306440088177309	4.12632776062152	0.382392028729365	0.536325758681864	0.893596348805136
Kcnk13	Kcnk13	0.0481460516867634	2.82398429444715	0.382281027711613	0.536384990074688	0.893596348805136
Wsb2	Wsb2	-0.022090275114025	8.21028115862145	0.382274634049755	0.536388397765697	0.893596348805136
Fgf12	Fgf12	-0.023759888972607	5.80400472633593	0.382256694056676	0.53639795860185	0.893596348805136
Lnc001	Lnc001	0.0635205946177746	1.35978754357595	0.382245606872843	0.53640398912615	0.893596348805136
Ddx55	Ddx55	0.0351488893292996	4.15721116385591	0.382047881137936	0.5365092777363	0.893596348805136
Pip4k2b	Pip4k2b	-0.0168077800261586	7.56882139700786	0.38202689387589	0.536520415041985	0.893596348805136
Nr1d1	Nr1d1	-0.0364044574772317	4.29727606372579	0.381928005684998	0.536573202312101	0.893596348805136
Rpl15	Rpl15	-0.0282020618486446	9.04146312108502	0.381509870171875	0.536796285105551	0.893849555856247
Zfp706	Zfp706	-0.0181348729072481	7.02179604552473	0.381192620954302	0.536965655961487	0.894011274276867
Tmt1	Tmt1	0.0188892376105875	5.52665314875332	0.38070013729137	0.537228772740182	0.894141753406309
Haus1	Haus1	-0.0279293003904616	4.23575703804551	0.38058589272517	0.537289950111283	0.894141753406309
Mepce	Mepce	0.0190225320720351	5.45379770072973	0.380459399324053	0.53735747612443	0.894141753406309
Shbg	Shbg	-0.0847879685189145	0.574200901445401	0.380318982616586	0.537432571682418	0.894141753406309
Tubb3	Tubb3	-0.0243440959620562	11.8335753620873	0.380250460923065	0.537469224389491	0.894141753406309
Mapre2	Mapre2	0.0154159962935316	9.34736520031734	0.380094452802098	0.537552691148448	0.894141753406309
Bet1l	Bet1l	0.0261139155424302	4.70796169694048	0.379936488553042	0.537637228556184	0.894141753406309
Fez1	Fez1	-0.0225744103459061	8.85662821989619	0.379899952896399	0.537658390032626	0.894141753406309
Vwc2l	Vwc2l	-0.0388581485042523	3.25738931352322	0.379782981644624	0.537719403775149	0.894141753406309
Strn4	Strn4	-0.0145308938833762	8.32916821137319	0.379701941137206	0.537762795589536	0.894141753406309
Dnal4	Dnal4	0.0272576779751426	5.22988572978295	0.379572118589415	0.537832320214343	0.894141753406309
Dnajc13	Dnajc13	0.0294845540103269	6.68515204599044	0.379375439325841	0.537937680260007	0.89414407346414
Hs3st3b	Hs3st3b1	0.119207050672248	0.233369725185966	0.379301976545158	0.537977042517857	0.89414407346414
Daglb	Daglb	-0.0219244302445111	4.72840862405704	0.378599516224199	0.538352706130551	0.894411053549185
Gdap1	Gdap1	-0.020730650179286	6.69371976343037	0.378598806342659	0.538354087015983	0.894411053549185
Map3k4	Map3k4	0.0183319667833691	5.56034859273709	0.378585731668615	0.538361102292581	0.894411053549185
Veph1	Veph1	0.0487441021808857	2.75283918806874	0.378467747529839	0.538424414779817	0.894411053549185
Adal	Adal	0.057234201408904	2.62921245430692	0.37822111686069	0.538502118490599	0.894419643410817
Cyb5d1	Cyb5d1	0.0398186174565363	2.42122691845834	0.377959617894116	0.538697242161686	0.894419643410817
Tg	Tg	0.0942445127323807	-0.00142823524886	0.37788752516476	0.538735865623342	0.894419643410817
Mapk3	Mapk3	0.0170131232434261	7.62574974596606	0.377511211008853	0.538938159133309	0.894419643410817
Itpa	Itpa	-0.0249795373288073	6.26226954443749	0.377430449682448	0.538981634472352	0.894419643410817
Procr	Procr	0.0827620724504823	0.839467683948266	0.37736623892954	0.53901616205296	0.894419643410817
Pde4c	Pde4c	-0.102551399768903	-0.133435284016991	0.37734932257672	0.539025259047727	0.894419643410817
Enpp4	Enpp4	-0.0313468735041337	3.89444119318234	0.377261195498241	0.53905651926373	0.894419643410817
Rpl6	Rpl6	-0.0241347390902016	8.88983602268937	0.377094058162925	0.539162565001914	0.894419643410817
Dusp14	Dusp14	-0.056471519798501	2.06961602567589	0.377020521230889	0.539202132168617	0.894419643410817
Slc25a53	Slc25a53	0.0404508815087689	2.60237904832583	0.376875282093447	0.539280294387008	0.894419643410817
Pik3r5	Pik3r5	-0.114189411888509	-0.160945739387967	0.376736008508644	0.539355266704151	0.894419643410817
Jade1	Jade1	0.027123720494408	5.2018089374919	0.376724436362238	0.539361496916165	0.894419643410817
Cmpk2	Cmpk2	-0.0291173616482871	4.40255669089838	0.376124876711956	0.539684467115552	0.894763073616413
Ess2	Ess2	-0.0233356684246087	4.89674805312963	0.376027705851101	0.539736845626568	0.894763073616413
Nr2f2	Nr2f2	0.0438093822043544	6.45885951148663	0.375940732081546	0.539783734480303	0.894763073616413
Edf1	Edf1	-0.0366013125938232	6.4961204018336	0.375267811176954	0.540146768604789	0.89511334452022
Gask1b	Gask1b	0.0692553465413928	2.60655295640192	0.374872557217515	0.540360212917795	0.89511334452022
Pcmtd2	Pcmtd2	0.0286582777268041	6.05311096792062	0.374836585572638	0.540379645938866	0.89511334452022
Masp2	Masp2	0.0460061751987002	4.15086297682332	0.374830237267204	0.540383075627472	0.89511334452022
Pdcl	Pdcl	0.0242364372062322	5.09648576775554	0.374581498205302	0.540517488988551	0.89511334452022
Lamc2	Lamc2	-0.0589040347422706	2.47505545597786	0.374574348225778	0.540521353824452	0.89511334452022
Pdxk	Pdxk	0.0250225603659822	4.64710895246905	0.374569918535748	0.540524144196584	0.89511334452022
Pygo2	Pygo2	0.0205292916469645	6.48294297256628	0.374191843202858	0.54072817375736	0.89511334452022
Pex6	Pex6	0.0221405630245723	6.15221494688176	0.373934310748659	0.540867503185503	0.89511334452022
Col9a2	Col9a2	-0.0528491933165836	4.22488836815784	0.373643772248551	0.541024768599882	0.89511334452022
Tagln	Tagln	0.0797109544665077	2.79343714526198	0.37342676571221	0.541142286745259	0.89511334452022
Tmem12	Tmem120b	-0.0306402876108133	4.08532967170201	0.373312586835227	0.541204132810745	0.89511334452022
Cnpy2	Cnpy2	0.0261527475139007	5.10669692872154	0.373280687334891	0.541221420728673	0.89511334452022
Kif5c	Kif5c	-0.0197581345037441	9.57861534918559	0.373182667942993	0.54127453197646	0.89511334452022
Gabra3	Gabra3	-0.023564809283117	5.89513742200178	0.373009652713733	0.541368302588636	0.89511334452022
Ptpmt1	Ptpmt1	-0.0348778039155977	3.97874870382115	0.372824191655724	0.541468851958196	0.89511334452022
Naprt	Naprt	0.0452085674904096	2.52265143308011	0.372705927834986	0.541532987823672	0.89511334452022
Abcb6	Abcb6	-0.0286983113149717	6.71817493737221	0.372697964976908	0.541537306442641	0.89511334452022
Gosr1	Gosr1	-0.0212516033461784	5.07853824017597	0.372628521883122	0.541574972184228	0.89511334452022
Pdpk1	Pdpk1	0.0242093731401503	5.65887476763453	0.372622532125302	0.541578221781758	0.89511334452022
Rfc2	Rfc2	-0.029427320712518	4.14316804058988	0.372552178492512	0.541616388286944	0.89511334452022
Ip6k1	Ip6k1	0.0187570895625553	6.64400121964526	0.372545522841715	0.541619998807525	0.89511334452022
Fbxl19	Fbxl19	0.0138586151045922	7.98061132406342	0.372499282327063	0.541645087050385	0.89511334452022
Ndufa9	Ndufa9	-0.0194453758824415	6.66642680823744	0.37219565599864	0.541809873340049	0.895132925377551
Kcmf1	Kcmf1	-0.0161313644326692	6.56379633641102	0.372069904821523	0.541879777965027	0.895132925377551
Aldh7a1	Aldh7a1	-0.0300955740880404	5.35280664225342	0.371988254455493	0.541923575017073	0.895132925377551
Flii	Flii	0.0240328853548123	6.91647509962869	0.371643498321617	0.542109780884106	0.895132925377551
Acot13	Acot13	-0.0355758399570023	2.96014698950639	0.371561982354059	0.542154082624782	0.895132925377551
Usp2	Usp2	-0.0488034407783837	3.05582180065905	0.371486971720543	0.542194854780023	0.895132925377551
Slc17a8	Slc17a8	-0.0899270590800536	2.0459609508613	0.371477278978119	0.542200123688249	0.895132925377551
Mink1	Mink1	-0.0421965299025445	7.52527145445489	0.371420708779026	0.54223087681362	0.895132925377551
Mybbp1a	Mybbp1a	0.0156145576875251	6.8543409064264	0.370861976555313	0.542518465538489	0.895465987905846
Lrrn4cl	Lrrn4cl	0.113222289491178	-0.528635985421841	0.37076748541422	0.542586219795885	0.895465987905846
Dlgap2	Dlgap2	0.0437964214053055	4.18080084203215	0.370654108012857	0.542647808872258	0.895465987905846
Dph6	Dph6	-0.041446290295328	3.22783516849849	0.370417060168517	0.542777018089547	0.895481637246859
Bmp8a	Bmp8a	-0.0837128373913264	0.165460116341329	0.370373106105923	0.542800963572813	0.895481637246859
Myo16	Myo16	0.0216909652073969	7.84294845654727	0.37018682618956	0.542902446476629	0.895520653373864
Ncbp2	Ncbp2	0.0207112481204865	6.39774526235563	0.369700980121173	0.543167304706901	0.895849102556925
Esrrg	Esrrg	-0.0398841139128659	4.67631839080861	0.369480689892533	0.5432858359489	0.895826166420092
Cir1	Cir1	-0.0218072563121361	5.12572263868313	0.36908649381698	0.543502628775787	0.89616523050555
Tmco1	Tmco1	-0.0279453020706462	4.29352274768305	0.368927170343873	0.543589629896408	0.896189086411501
Spata6l	Spata6l	-0.0779792393604306	0.62608611579932	0.3686424198021	0.543745197133872	0.896189086411501
Sctr	Sctr	0.0741195228947694	1.08323172118537	0.368437252253898	0.543857334180055	0.896189086411501
Zfp763	Zfp763	-0.029102718512566	4.89465206246082	0.36821847047954	0.543976858344466	0.896189086411501
Irf8	Irf8	0.0962329515954804	0.453041503126256	0.368036125871299	0.544076898608083	0.896189086411501
Pclo	Pclo	0.0632096424019768	7.33898761132089	0.367995335681513	0.544099014744111	0.896189086411501
Pan2	Pan2	0.0470789236716364	4.9075721240919	0.367961028968177	0.544101371027059	0.896189086411501
Gja1	Gja1	0.0423940508864259	6.8957465168854	0.36796318361554	0.544171326188786	0.896189086411501
Fils1	Fils1	0.0295760038631165	5.78028861584966	0.36781370123127	0.544198406710281	0.896189086411501
Chd8	Chd8	0.0304009691044524	7.56972009159993	0.367640226868616	0.5442933647491	0.896189086411501
Zfp870	Zfp870	0.0325545233172457	3.38712071044995	0.367614962072253	0.544307196988567	0.896189086411501
Prr22	Prr22	0.0791379458433208	1.42526608254773	0.367429498634067	0.544408756389836	0.896189905131306
Cldn23	Cldn23	-0.0676658667868956	1.30905364442635	0.367352670414884	0.544451385404768	0.896189905131306
Prdx2	Prdx2	-0.0262160760906146	8.85889882363002	0.367055201960646	0.544613827276472	0.896248704083671
Ddb1	Ddb1	0.0154909530412403	8.52071564330049	0.366868829925323	0.54471599070818	0.896248704083671
Adamts6	Adamts6	0.0686823906922399	2.58024968659893	0.366581861046399	0.544872367619464	0.896248704083671
Cct7	Cct7	-0.0180508692801743	8.52610054545528	0.366501684894517	0.544917346867758	0.896248704083671
Tp53rk	Tp53rk	0.0385598204484271	3.05894714929173	0.366423080284243	0.544960480925854	0.896248704083671
LOC1001	LOC100125	-0.0536344302071386	1.68761213155007	0.366353795612478	0.54499852108732	0.896248704083671
Anapc10	Anapc10	-0.0327039445686385	3.85265321974311	0.366187610660766	0.545089716221034	0.896248704083671
Rpa1	Rpa1	0.0261052152077721	5.12367254977046	0.366083782068672	0.545146714114557	0.896248704083671
Srcin1	Srcin1	-0.0229349574288044	8.12189013187654	0.365861138621404	0.545259800575934	0.896248704083671
Lage3	Lage3	-0.0332610305058326	5.71361872009089	0.365843036296792	0.545278823333466	0.896248704083671
Sema4d	Sema4d	0.025834810211614	5.53713767562162	0.365554571311172	0.545437425586977	0.896248704083671
Mpc2	Mpc2	-0.0249725021790655	6.60014351015551	0.365446480226112	0.545496849549424	0.896248704083671
Smyd4	Smyd4	0.0296880704658943	3.91578394945015	0.365310292573899	0.545571723762466	0.896248704083671
Nlgn1	Nlgn1	0.025327051311201	5.99496234649347	0.365049862181237	0.545714859709305	0.896248704083671
Fah	Fah	-0.0629184720023371	1.8452067469457	0.364832228491451	0.545834710640792	0.896248704083671
Tmau1a	Tmau1ap	0.031203889720491	5.46778788076531	0.364599562148566	0.545962787079787	0.896248704083671
RGD156	RGD156272	-0.0634679202251777	1.42858930524221	0.364522326859926	0.5460047965819	0.896248704083671
Alox12	Alox12	0.109169635551031	-0.206016958830006	0.364465304160078	0.546036717793453	0.896248704083671
Eif4g2	Eif4g2	-0.0184259193780786	10.8084570252967	0.364349148059617	0.54610069548671	0.896248704083671
Enoph1	Enoph1	-0.0189770318927091	5.99952556960692	0.364345268999966	0.546102821725683	0.896248704083671
Ptger2	Ptger2	0.107090231169018	-0.347016609008478	0.364250059709477	0.546155283421118	0.896248704083671
Stox1	Stox1	-0.075743800156683	0.859638847007868	0.364235581706261	0.54616326022493	0.896248704083671
Ina	Ina	-0.0186842289985892	9.19083926527542	0.364176375649421	0.546195880657736	0.896248704083671
Adat2	Adat2	0.041742834710219	2.74805878250272	0.36388582723172	0.54635602126449	0.896248704083671
Slc30a6	Slc30a6	-0.0282258424322097	3.72512821296481	0.363787719882533	0.546410121158921	0.896248704083671
Crip2	Crip2	0.027298486901653	8.34865741381159	0.363781740607975	0.546413418330525	0.896248704083671
Armc8	Armc8	-0.0136028469372417	6.19516004540356	0.363690602295883	0.54646357960494	0.896248704083671
Ifi30	Ifi30	0.0421270020021669	3.67562666618523	0.36359432454902	0.546516779005346	0.896248704083671
Acd	Acd	-0.0405290052054758	5.25279941489726	0.363373139229239	0.546638822328073	0.896248704083671
Mrpl16	Mrpl16	0.0271438871055452	4.00760452937762	0.363367322442954	0.546642032381828	0.896248704083671
Ppan	Ppan	0.0245282792954321	5.07304765197196	0.362946105815887	0.546874579036699	0.896326615198387
Extl2	Extl2	0.0262230277056781	4.48178168642296	0.36274922872564	0.546983334659432	0.896326615198387
Ap3m1	Ap3m1	0.0176395118376001	6.19927821467199	0.36243128218846	0.547159054434385	0.896326615198387
Lrfn3	Lrfn3	-0.0228880334692624	5.78490002708253	0.36239258497681	0.547180468422118	0.896326615198387
Nrcam	Nrcam	0.0273189243101201	8.64075135254031	0.3623151258309	0.547222376965968	0.896326615198387
Kcnj11	Kcnj11	-0.0265481968280474	3.75262281146292	0.362259134032101	0.547254239523827	0.896326615198387
Siah3	Siah3	0.0503486453627391	2.62754849389031	0.36216966536068	0.547303732255219	0.896326615198387
Ptch2	Ptch2	-0.0632012600534731	2.37876403098832	0.362127147978018	0.54732170736527	0.896326615198387
Rbbp9	Rbbp9	0.0255336532888228	4.57174036311773	0.361823900394455	0.547495028743543	0.896326615198387
Arhgap1	Arhgap11a	-0.0385837304077845	4.06412366882516	0.361801079433988	0.54750765968212	0.896326615198387
Uri1	Uri1	0.0211299803581435	5.86052953235097	0.361758590671497	0.547532294889153	0.896326615198387
Dxo	Dxo	-0.0286880912255702	3.84243352597403	0.361721676452355	0.547551611849171	0.896326615198387
Fam162b	Fam162b	-0.0459841364332183	5.52765791261714	0.360921641713773	0.547994824345834	0.896697916610627
Psmb5	Psmb5	-0.0312522945876024	7.26317053424559	0.360801841407493	0.54806125024535	0.896697916610627
Mgat4c	Mgat4c	-0.0555173401726106	3.75479624616189	0.360740935435174	0.548095026647872	0.896697916610627
Rad51d	Rad51d	0.0369785122944074	2.86129982778735	0.360677151028959	0.548130403492525	0.896697916610627
Oma1	Oma1	0.0634230968231313	1.49360493820988	0.360623715266492	0.548160043929454	0.896697916610627
Foxj2	Foxj2	0.0221409692601755	6.17023964857886	0.360465387252906	0.548247879348959	0.896697916610627
Tm2d2	Tm2d2	0.0265003906615284	5.57556393157661	0.360283370799614	0.54834890073268	0.896697916610627
Synm	Synm	0.0454789699013407	4.41934279767526	0.360191527125536	0.548399885485522	0.896697916610627
Cyp51	Cyp51	-0.0187135982272305	8.15093768121748	0.359901801217973	0.548560777642323	0.896697916610627
Tmprss5	Tmprss5	-0.139081802453264	-0.630046966721187	0.359644721387752	0.548703614361584	0.896697916610627
Sh2d1a	Sh2d1a	0.0491192184139539	3.23413961826228	0.359551822686711	0.548755247097467	0.896697916610627
Septin8	Septin8	0.0200530653803739	6.02468893701327	0.359543195335576	0.548760042605783	0.896697916610627
Tdrkh	Tdrkh	0.0169111470816561	5.91232257609715	0.359431821027606	0.548821856976558	0.896697916610627
Kantr	Kantr	-0.0428518560682396	2.21555309542661	0.35924818496882	0.54892437104034	0.896697916610627
Iftap	Iftap	-0.0320011695336486	3.66111604837964	0.358959507496202	0.549084666969951	0.896697916610627
Zfpm1	Zfpm1	-0.0300093827041132	4.36386681445306	0.358919700695068	0.549106819019328	0.896697916610627
Gcg	Gcg	0.146710229611655	0.0262495900778967	0.358808000352525	0.549168807953388	0.896697916610627
Brd8	Brd8	0.0172999688169003	6.4063355027631	0.358807001386445	0.549169544006137	0.896697916610627
Engase	Engase	-0.0327768530361215	4.00117417619381	0.358718291765875	0.549218826482001	0.896697916610627
Trappc3	Trappc3	-0.0262148322927596	6.2182791033321	0.358640941847752	0.549259851256465	0.896697916610627
Mtg2	Mtg2	-0.0218382208858103	5.03221129604557	0.358594838986171	0.549287863433119	0.896697916610627
Ctdsp1	Ctdsp1	0.0354296719847	4.82910551050867	0.358215545248612	0.549498850012728	0.896925483949347
Slc35f5	Slc35f5	-0.0271744811292407	4.89735406817984	0.357819088408821	0.549719858804248	0.897052589637352
Gmps	Gmps	0.0259420363451877	5.66886469037101	0.357817915398286	0.549720613859061	0.897052589637352
Amz2	Amz2	-0.0137688152478535	8.19057356390477	0.357321885877326	0.549997366635189	0.897386837126618
Tbx6	Tbx6	0.120088518049544	-0.228675401894723	0.35706940154499	0.550138326856885	0.897499480774496
Aco1	Aco1	0.0139175857037924	6.93435616176916	0.356718220275027	0.550334525296471	0.897702166573841
Prkra	Prkra	-0.0181711356916721	6.50301879913993	0.356418900556186	0.550501864582882	0.897857714380882
Nomo1	Nomo1	0.0160484453267278	6.92190495711879	0.356079187439121	0.550691859215841	0.897952254907069
Ola1	Ola1	-0.0197545797440861	7.11674920559338	0.356057940986688	0.550703747258867	0.897952254907069
Abraxas2	Abraxas2	-0.0212370802938055	4.90594214061514	0.355725781011863	0.550889663536779	0.898138028476818
Cyp4x1	Cyp4x1	-0.0400656697253206	4.5784663212616	0.355499241292335	0.551016529651491	0.898227494181036
Tmem26	Tmem263	0.0291150395151321	3.82252167020076	0.355353900604662	0.551097948415054	0.898242861185451
Adrb1	Adrb1	0.058545707528123	3.0731844988047	0.354992721654384	0.551300387637566	0.898247920680679
Nipa1	Nipa1	0.0186179490056384	5.82588542351238	0.354924894684892	0.551338419288217	0.898247920680679
Zfp52	Zfp52	-0.058603200131476	2.20807893824894	0.354697142536804	0.551471769554105	0.898247920680679
Ppcdc	Ppcdc	0.0356750220384671	2.71746287992559	0.354594420138026	0.551529403172834	0.898247920680679
Celf5	Celf5	0.0203261524963477	9.02970902248057	0.354475642843909	0.551590446256756	0.898247920680679
Mas1	Mas1	-0.0797467792951731	1.92539278080678	0.35428772169692	0.551695832806575	0.898247920680679
Hs6st3	Hs6st3	0.0320580356739658	5.05971030968136	0.354161667311772	0.551766712685734	0.898247920680679
Golga7	Golga7	-0.016038824945305	7.1262696180049	0.354124149314963	0.551787782403657	0.898247920680679
Elf1	Elf1	0.0236480725127069	4.63571555881968	0.354098178795127	0.551802368089169	0.898247920680679
Mphosp	Mphosph10	-0.0217583715986697	4.61129942840251	0.353888134719135	0.551920360598884	0.898247920680679
Xylt2	Xylt2	-0.0200910642531322	5.22915898042931	0.353785809463176	0.551977859107438	0.898247920680679
Qrfpr	Qrfpr	0.0736880734574659	0.861158585851172	0.353723666794398	0.552007163395094	0.898247920680679
LOC6859	LOC685933	-0.0184574423996161	5.37174954555143	0.353681013476056	0.552036757635926	0.898247920680679
Sec22b	Sec22b	-0.0222459769829117	5.72500987684805	0.353358070210383	0.552218235877883	0.898425893434392
Kif23	Kif23	0.0392071593984415	3.52352606695676	0.352907160011064	0.55247205281774	0.89872148670234
Sprn	Sprn	-0.0270495738882456	4.60952255952877	0.352467759018431	0.5527195048408	0.898899034901685
Jakmip3	Jakmip3	0.039642414357934	4.73683267192733	0.352215266400279	0.55286179237909	0.898899034901685
Exosc5	Exosc5	0.0296407302935373	4.29228590837086	0.352140552582947	0.552903808637232	0.898899034901685
Wnt10a	Wnt10a	-0.120515910907898	-0.674968207336281	0.351772971030158	0.553111206036631	0.898899034901685
Hunk	Hunk	0.043391195257893	3.87966602676341	0.351725382780131	0.553138054033754	0.898899034901685
Hsp90b1	Hsp90b1	0.0252259814574264	8.64019210052956	0.351538752567745	0.553243368861602	0.898899034901685
Dync1h1	Dync1h1	0.0515860884877285	10.078978539257	0.351393389422293	0.553325423988646	0.898899034901685
Pask	Pask	-0.0531414976990055	3.87721913085367	0.351227606100093	0.553419032347851	0.898899034901685
Psmc3	Psmc3	-0.019762510238055	7.5306185170787	0.351163066977364	0.553455482361565	0.898899034901685
Hpf1	Hpf1	0.0204915792503099	5.48265965132562	0.350898491207182	0.553604956165184	0.898899034901685
Cntnap1	Cntnap1	-0.0582374607641836	4.79182060064687	0.350855117278582	0.553629802503767	0.898899034901685
Stxbp2	Stxbp2	-0.0521908569719657	2.76720827583598	0.350829380769895	0.553644012834645	0.898899034901685
Gpr149	Gpr149	-0.047553652371031	2.9585875685832	0.350659816807195	0.553739861243884	0.898899034901685
Rgs17	Rgs17	-0.0338767583764096	5.03315931680218	0.350647355939557	0.553746906158719	0.898899034901685
Ipo13	Ipo13	0.019948979664284	6.58348440609141	0.35059068891516	0.553778945170478	0.898899034901685
Eif2b3	Eif2b3	0.0241493770979814	4.87209610821833	0.350536261515117	0.553809722321654	0.898899034901685
Prpsap1	Prpsap1	-0.0194628230877557	6.09334285031112	0.350398634640257	0.553887558383541	0.898899034901685
Fastk	Fastk	-0.020576241924099	6.17621045768597	0.350322489637643	0.554100410829262	0.898899034901685
Tram1l1	Tram1l1	-0.0232267373791305	5.62539000492861	0.349972082242004	0.554128924059197	0.898899034901685
Rpl17	Rpl17	0.0409003855082828	8.17887861928155	0.349968821119546	0.554130780206567	0.898899034901685
Pigl	Pigl	-0.0429968769905001	3.06241095581204	0.349821141063074	0.554214395233193	0.898899034901685
Mkrn1	Mkrn1	0.0144033667984647	6.98313039814929	0.349706732302661	0.554279188184642	0.898899034901685
Pdzd8	Pdzd8	0.0200515778705142	6.72605841476088	0.349670206033089	0.554299677815073	0.898899034901685
Ehmt2	Ehmt2	0.0181260187183904	8.42659560301982	0.349358293610521	0.554476609432857	0.8991687881328
Nceh1	Nceh1	-0.0350067311340831	4.61178640869811	0.348909092644475	0.554758654762948	0.89950925737197
Sgsm3	Sgsm3	-0.0142822857065207	6.21540418096559	0.348669674711488	0.55486716266188	0.899521972785646
Acvr2a	Acvr2a	0.0375624481253769	6.29083959365403	0.34859289227216	0.554910695624038	0.899521972785646
Fbxl16	Fbxl16	0.0219729820988702	6.46028537414319	0.348285722976076	0.555085148486814	0.899634425625176
Gpr173	Gpr173	-0.0259446317032644	3.72175456686915	0.348216856480853	0.55512426407696	0.899634425625176
Zfp105	Zfp105	0.033989117887074	3.82556339048476	0.34785122166366	0.555332628956706	0.899854250038029
Abcc1	Abcc1	0.0633005203765283	6.06896810249723	0.347489464115256	0.555537735548003	0.900005865102962
G0s2	G0s2	-0.0789818880880455	0.577488967832122	0.347433205464291	0.555569738929472	0.900005865102962
Wdr83os	Wdr83os	0.0280402642409895	6.22271534396275	0.34717192953083	0.555718414526908	0.900038982928897
Spag1	Spag1	-0.0532463598512405	1.69322307210492	0.347031707822104	0.555798226807679	0.900038982928897
Rpf1	Rpf1	0.0271135744145416	3.6914073090405	0.346970700681386	0.555832818365576	0.900038982928897
Pi4k2b	Pi4k2b	0.0511879508439425	2.52572552031162	0.346694151051921	0.555990483049643	0.900038982928897
Zfp444	Zfp444	0.0209167187602543	5.85595218580753	0.346638271781622	0.556022319704118	0.900038982928897
Taf9b	Taf9b	-0.0240870752819226	5.02914796200728	0.346455034497508	0.556126752960714	0.900038982928897
Mfn1	Mfn1	0.0378207605068835	6.44739235257822	0.346409804070277	0.556152522851536	0.900038982928897
Slc25a27	Slc25a27	-0.0498125996579486	5.57020354295338	0.346341315849946	0.556191565371063	0.900038982928897
Pheta1	Pheta1	-0.024302810594251	5.32208458822505	0.346259349485617	0.556238299657526	0.900038982928897
Pdzrn4	Pdzrn4	-0.0331912678257694	4.12750886275757	0.34611504003982	0.556320593530155	0.900053429132265
Cpne1	Cpne1	0.02901570809369	4.89753083400976	0.345585757287154	0.556622625731668	0.900200901559017
Nsun2	Nsun2	0.0153211750628745	6.44370264284823	0.345511982664856	0.556664748940965	0.900200901559017
Atf7ip	Atf7ip	0.0286603896945511	7.50383015289038	0.345305369839199	0.556782738597588	0.900200901559017
Zfp282	Zfp282	0.0133044349128038	6.42307542705175	0.345219553839591	0.556831779861339	0.900200901559017
Rasgrp1	Rasgrp1	-0.0757042610077614	5.04623871670323	0.344996249636097	0.556959391521646	0.900200901559017
Smim20	Smim20	-0.0400518185151501	3.87545677630421	0.344670888357982	0.557145425157174	0.900200901559017
Srrm2	Srrm2	0.0613289593648636	9.86857743637073	0.344660736254312	0.557151231782134	0.900200901559017
Prim1	Prim1	-0.049613436118117	5.86855642751234	0.344540126623599	0.557220224785576	0.900200901559017
Niban3	Niban3	0.108587949380944	3.04014543694142	0.34453904521466	0.557220843473524	0.900200901559017
Klhl24	Klhl24	0.0222242127524001	7.28208803768024	0.34428613880236	0.557365556807961	0.900200901559017
Hdac3	Hdac3	-0.0184367660618221	6.56358590426775	0.344226673501659	0.557399607129346	0.900200901559017
Cep57l1	Cep57l1	-0.0282704473279065	3.485650019054	0.344125097651286	0.557457760309697	0.900200901559017
Ptprx	Ptprx	0.0231022421758925	10.2082789771839	0.344084380687016	0.557481068687887	0.900200901559017
Plcxd3	Plcxd3	-0.0628763737967725	4.91080807134837	0.344057676082187	0.557496366195879	0.900200901559017
Sin3b	Sin3b	0.0199860420385694	6.47865870055168	0.343995822346567	0.557531787815238	0.900200901559017
Tnip2	Tnip2	-0.0694629041660881	0.944052452465768	0.343935867783507	0.557566127053625	0.900200901559017
Xab2	Xab2	0.0168882174380589	5.58058210589652	0.343741111528887	0.557677700451281	0.900202012785211
Selenow	Selenow	-0.0325564713275355	9.00531131539474	0.343633113092295	0.55773958969966	0.900202012785211
Clip1	Clip1	0.0203594116137526	6.97538425197563	0.343485179598341	0.557824385218643	0.900202012785211
Usp39	Usp39	-0.0200015943479129	5.31742945937315	0.343327587056606	0.557914744298923	0.900202012785211
Capzb	Capzb	-0.0179714388075791	7.82657403916838	0.343305239687709	0.557927559889658	0.900202012785211
Large1	Large1	0.0144060285402599	7.76807592961082	0.342691973161763	0.558279470218983	0.900653342374241
LOC6919	LOC691995	-0.0282506328589536	9.06091385330302	0.342534244989935	0.558370047620979	0.9006830102349
Cx3cr1	Cx3cr1	0.101570845288248	1.81298758636352	0.342388887770922	0.558456418229222	0.900705885502327
Rfx1	Rfx1	0.0479732353078152	5.51742589454	0.341701884052696	0.558948505685722	0.901018269813406
Mrpl21	Mrpl21	-0.0289865680981575	4.1053605139589	0.341646937687443	0.558880117480386	0.901018269813406
Trpc7	Trpc7	0.0413898651842605	3.52402474125539	0.341602149552241	0.558905887536822	0.901018269813406
Slc2a5	Slc2a5	-0.0966915281522725	0.305324888434789	0.341497698319142	0.558971750471952	0.901018269813406
Nkap	Nkap	0.0204313321962746	5.33214305871328	0.341250328596495	0.559108396184151	0.901018269813406
Actr5	Actr5	-0.0212440327825326	4.41627515055331	0.341216366426415	0.559127953419435	0.901018269813406
Dgcr6	Dgcr6	-0.0230355876785754	6.34980558148609	0.341111246538392	0.559188490605417	0.901018269813406
Ywhae	Ywhae	-0.02071177870008	10.8439345190946	0.341042969967049	0.559227817689368	0.901018269813406
Stag2	Stag2	0.0215849033295271	6.92523000634094	0.340827150784101	0.55935216351539	0.901102252335239
Myo1e	Myo1e	0.0423903253603803	3.45761718046551	0.340449182130001	0.559570060178731	0.901328901736383
Scarf2	Scarf2	0.040806308523159	4.50514153888216	0.340315418209642	0.559647213121459	0.901344814523873
Asic1	Asic1	-0.0174189289437221	7.56260680177357	0.34006812187186	0.559789897552488	0.901458253970365

**Emc8**	Emc8	-0.027407710150647	4.79637447749649	0.339389318842763	0.560181930902463	0.901907562701322
**Slc26a6**	Slc26a6	-0.039653454518028	3.65353926878537	0.339334727577167	0.560213491681113	0.901907562701322
**Endog**	Endog	-0.048807072167068	2.92045072079071	0.338757262345789	0.560547468165604	0.902137002872745
**Plcb1**	Plcb1	0.02171945504209	7.18831490452683	0.338454104909374	0.560722903858577	0.902137002872745
**Rwdd1**	Rwdd1	-0.021046248126085	5.16591756971408	0.338285330671617	0.560820637578722	0.902137002872745
**Irx2**	Irx2	-0.146953520061476	2.34689182549539	0.338146037626714	0.560901323920667	0.902137002872745
**Dpy19l4**	Dpy19l4	0.030050411582635	4.06317390237953	0.338142703265255	0.560903255642309	0.902137002872745
**Zfp846**	Zfp846	-0.033102722563724	5.56238266968832	0.338104255792757	0.560925520637326	0.902137002872745
**LOC100**	LOC10091	-0.066389346635046	2.21694070034305	0.338062920410422	0.560949480667254	0.902137002872745
**Cmss1**	Cmss1	0.0293488725411991	4.21621392432307	0.338041618430672	0.560961823958924	0.902137002872745
**Morf4l2**	Morf4l2	-0.015925516856283	8.77678827603214	0.337885376377834	0.561052373294315	0.902137002872745
**Palb2**	Palb2	-0.050629684315049	1.93917713013264	0.337839377931367	0.561079038811133	0.902137002872745
**Eef1b2**	Eef1b2	-0.022050741714945	7.65231997431509	0.337331167936711	0.56137378819288	0.902293485951002
**Nrgn**	Nrgn	-0.048298104322939	8.80964929489713	0.337217118742103	0.561439675180726	0.902293485951002
**Mageh1**	Mageh1	-0.022306842158903	6.09135477452355	0.336949819708103	0.561595157412357	0.902293485951002
**Frmpd1**	Frmpd1	-0.035007212238579	4.37862778779971	0.33682846628507	0.561665637111841	0.902293485951002
**Ddx51**	Ddx51	-0.027541510779242	5.01569657011792	0.336810820145118	0.561675887058922	0.902293485951002
**Myh6**	Myh6	0.0893492058209783	1.73902152655891	0.336779311851416	0.561694189877254	0.902293485951002
**Gpr165**	Gpr165	-0.064827814363653	3.37238973714862	0.336725437052181	0.561725497802585	0.902293485951002
**Tnc**	Tnc	0.0408686275169804	8.0517109444112	0.336477774808998	0.561869407313819	0.902293485951002
**Nol4**	Nol4	0.0219182614947447	6.42391905488358	0.336255019985586	0.561998813302898	0.902293485951002
**Txndc11**	Txndc11	-0.016271501410012	5.72538183918089	0.336031699998344	0.562128805420224	0.902293485951002
**Ptrh2**	Ptrh2	0.0218976834509328	5.22971519680349	0.335726820891139	0.562306228819256	0.902293485951002
**Lsm8**	Lsm8	-0.026784113312044	4.52241212794725	0.335722317745926	0.562308850216214	0.902293485951002
**Adgrg2**	Adgrg2	-0.0628947767414414	1.66538814713759	0.335639187530191	0.562357246677224	0.902293485951002
**Snap47**	Snap47	-0.02760885839857	4.54348913460077	0.335497155121402	0.5624398521383	0.902293485951002
**Mir3577**	Mir3577	0.0524091598190258	1.2947894181503	0.335456619724738	0.562463561470646	0.902293485951002
**Rbpms**	Rbpms	0.0913137473410217	0.938731408947488	0.335357373135366	0.562521371983298	0.902293485951002
**Rapgef3**	Rapgef3	-0.041341842984891	5.34452626524627	0.335289694939831	0.562560800673468	0.902293485951002
**Sema6a**	Sema6a	-0.033385239488371	6.31469840512857	0.334885410825123	0.562796443521983	0.902293485951002
**Cryab**	Cryab	-0.062007725835127	1.3012195562146	0.334878666083497	0.562800376412436	0.902293485951002
**Rai1**	Rai1	0.0267341241840894	7.26586644246687	0.334877485502091	0.562801064818674	0.902293485951002
**Clta**	Clta	-0.022519428025706	8.11256288502885	0.334844129328118	0.562820515733665	0.902293485951002
**Slc7a10**	Slc7a10	0.0309626923764397	4.09738206631206	0.334830582806298	0.562828415457456	0.902293485951002
**Med12**	Med12	0.0617763235615734	5.98424059001488	0.334811333712963	0.562839641332031	0.902293485951002
**Slc39a11**	Slc39a11	0.0251495250977666	3.88504103762428	0.334598324205515	0.562963891445223	0.902376730930376
**Isg20l2**	Isg20l2	0.0291759971221233	3.66852180977605	0.334386874917087	0.563087392983668	0.902458579610049
**Cnpy3**	Cnpy3	0.0203357920436817	6.00221233052465	0.334105545727674	0.563251526103964	0.902491235295004
**Ndufaf3**	Ndufaf3	0.0286519360525281	4.96341651206636	0.333885414054286	0.563380122823425	0.902491235295004
[illegible]	[illegible]	[illegible]	[illegible]	[illegible]	[illegible]	[illegible]

**Snip1**	Snip1	0.0195014608941409	4.99651522412348	0.290066456789624	0.590177946355205	0.910666613489227
**Cpt1c**	Cpt1c	-0.0246258385523103	5.60246117704662	0.289005148527039	0.590858760853966	0.911464701354271
**Mfsd13a**	Mfsd13a	0.0230074740685 88	4.30866127705043	0.288943773759655	0.590898181177796	0.911464701354271
**Rtn4**	Rtn4	-0.0152260761434306	10.2145793534033	0.288938834830579	0.590901353631057	0.911464701354271
**Alg9**	Alg9	-0.0169427715922973	5.66119469475138	0.288791819096581	0.590995803176032	0.911477705342307
**Rps3a**	Rps3a	-0.0225883399860504	8.63644695655555	0.288372217029853	0.591265545004686	0.911696768848009
**Rpl10a**	Rpl10a	-0.0243348133532216	7.7202590327276	0.288343550098711	0.591283982809817	0.911696768848009
**Abca1**	Abca1	0.0478525142061486	4.62574691614178	0.288202354113977	0.591374813537916	0.911724150316641
**Agrn**	Agrn	0.0305871816925711	9.76891913932285	0.287697136168074	0.591700052858636	0.912023722406849
**Anxa3**	Anxa3	0.0575972492052894	2.86274201916325	0.287673426390896	0.591715325305237	0.912023722406849
**Cage1**	Cage1	0.0538538125966 86	1.56439544907573	0.287495154376153	0.591830183643941	0.912088093049092
**Rtraf**	Rtraf	-0.0158239012513027	6.55555915688777	0.287216376655429	0.592009888944296	0.912149923649498
**Taf9**	Taf9	-0.0246193180880832	6.67563556186443	0.287198712304061	0.592021278961963	0.912149923649498
**Has1**	Has1	0.114563937649822	0.00440343855095 01	0.287092738766773	0.592089623438109	0.912149923649498
**Tamalin**	Tamalin	0.0383549986488457	2.67408699233543	0.286854559142771	0.592243290207275	0.912156793416368
**Slc25a30**	Slc25a30	0.0277111131056776	4.02985410411527	0.286769841055352	0.592298890183379	0.912156793416368
**Rpp25**	Rpp25	-0.0294216513557173	4.20799881809624	0.286681026958661	0.592355298881834	0.912156793416368
**Sema6d**	Sema6d	-0.0280645169059883	6.84400238090285	0.286632682180159	0.592386510944264	0.912156793416368
**Nt5dc3**	Nt5dc3	-0.0242547331001427	7.42035261252276	0.286492908533916	0.592476677238236	0.912183204469744
**Fau**	Fau	-0.0241983921725832	8.35717274806004	0.286084381412081	0.592740749998287	0.912300096096267
**Ppp1r36**	Ppp1r36	-0.0376578874375571	2.58169852968605	0.286011648931073	0.592787768348248	0.912300096096267
**Tpd52l3**	Tpd52l3	-0.0448276503625291	1.48009600787288	0.28601084377 97	0.592788289930665	0.912300096096267
**Zc3h15**	Zc3h15	0.0138028121022 68	7.00180763132787	0.285851756225732	0.592891168811958	0.912300096096267
**Fam76a**	Fam76a	0.0186526989829659	4.89081125381198	0.285809829753383	0.592918287989471	0.912300096096267
**Atp2b2**	Atp2b2	-0.0252084214804616	8.12377325330463	0.285442557777571	0.593155958944361	0.912461187455974
**Uimc1**	Uimc1	-0.0219885352575266	5.07202938271639	0.28525107 80985	0.593279948083714	0.912461187455974
**Adm**	Adm	-0.0811830673534725	-0.0950830872990 18	0.285201944354291	0.593311772375199	0.912461187455974
**Sdf4**	Sdf4	-0.0173515995973462	5.97581236602228	0.285121934351203	0.593363603000236	0.912461187455974
**Isoc1**	Isoc1	-0.0185600318990 02	7.41645203616968	0.285103263188597	0.593375634769398	0.912461187455974
**Chst11**	Chst11	-0.0238822347581755	4.57158128805342	0.284817909331795	0.593560616775979	0.912613162725064
**Lrrc73**	Lrrc73	-0.0234213665786804	6.38635145045666	0.284700361767264	0.593636852937317	0.912617916966351
**Flgn**	Flgn	0.0689450068889618	3.2041041182999	0.284434782945638	0.593809126149 72	0.912747985117555
**Map3k2**	Map3k2	0.0491757094923228	3.57449971920397	0.284293649920826	0.593900717052952	0.912747985117555
**Baiap2l1**	Baiap2l1	0.0590965079569431	1.32310343064095	0.284221701124504	0.593940829098295	0.912747985117555
**Dvl1**	Dvl1	-0.0241555234148 75	7.11306180059419	0.283964984698722	0.594114123687385	0.912900171443 9347
**Hipk4**	Hipk4	-0.0443715270938234	2.71293213658 94	0.28361163252495	0.594343736853235	0.913117430934165
**Tm7sf2**	Tm7sf2	0.0259215843862532	5.32761483592 65	0.283523739478127	0.594400879477044	0.913117430934165
**Gfm2**	Gfm2	0.0318730784232952	4.36340789 09131	0.283256736227003	0.594574538270322	0.913271760925619
[illegible]	[illegible]	[illegible]	[illegible]	[illegible]	[illegible]	[illegible]

**Ran**	Ran	0.0197544968965008	8.02082153772548	0.240921877234697	0.623541077942847	0.923561834976699
**Agap3**	Agap3	-0.014100209844255	8.2646470705778	0.240903761332525	0.623554121429327	0.923561834976699
**Kbtbd4**	Kbtbd4	0.0172550234427282	4.30937379430828	0.240630930780078	0.623750794280454	0.923743461155753
**Camkk1**	Camkk1	-0.015409314482080	5.86182157945564	0.239865759856142	0.62430308896897	0.924366622186464
**Card10**	Card10	0.0402895988159675	2.22432886770923	0.239842697005777	0.624219750352527	0.924366622186464
**Zfp709l1**	Zfp709l1	0.0455012303668812	2.43049603679992	0.239660525878378	0.624451408412926	0.924385142760608
**Gdpd1**	Gdpd1	0.0130241638218452	6.20768855932288	0.239608361154758	0.624489120137958	0.924385142760608
**Mir6315**	Mir6315	-0.028446179870256	2.85582414120463	0.239517912692143	0.62455452059585	0.924385142760608
**Zfp800**	Zfp800	-0.027685282332153	3.48830156686408	0.239225184976767	0.624756287845528	0.924548787329153
**Babam2**	Babam2	-0.01717721259094	5.14930953795112	0.239160248502543	0.624813286427781	0.924548787329153
**Eif2b1**	Eif2b1	-0.019474018503961	5.3091304180833	0.238840786915389	0.625044615925856	0.924556953616654
**Pcid2**	Pcid2	-0.01783378407026	4.90176238902654	0.238666051058079	0.625171227284322	0.924556953616654
**Ap1ar**	Ap1ar	0.0135896599161443	6.51308144238329	0.23858081248973	0.625233010888713	0.924556953616654
**Nbl1**	Nbl1	0.0351936826082882	5.17375148734473	0.238515925503606	0.625280052189629	0.924556953616654
**Psma4**	Psma4	-0.016889239242759	6.97137127802101	0.238419782301971	0.62534976724439	0.924556953616654
**Sdhaf2**	Sdhaf2	-0.017012394296131	5.30072262463046	0.238396705439399	0.625366504155958	0.924556953616654
**Neto2**	Neto2	-0.014042885957666	6.85468204551494	0.238238956814733	0.625480936113142	0.924556953616654
**Col9a3**	Col9a3	0.0399756577870131	5.17580070582112	0.238168148954884	0.625532315885251	0.924556953616654
**Nell1**	Nell1	0.0363869865584251	2.44760935823568	0.238159644200678	0.625538487761875	0.924556953616654
**Spint2**	Spint2	-0.027705669438121	6.08094618858451	0.238017280032341	0.625641821258586	0.924556953616654
**Klhl36**	Klhl36	-0.022535271012301	3.92022828617113	0.237791942694145	0.625805458298541	0.924556953616654
**Ndufs8**	Ndufs8	-0.023191787540681	5.863681194806	0.237616884271276	0.625932649656987	0.924556953616654
**Rbm5**	Rbm5	0.0323729479130081	7.52327153288664	0.237552322668165	0.62597957250727	0.924556953616654
**Ttbk1**	Ttbk1	0.01725192476837	6.63066174267353	0.237503446576284	0.626015100923473	0.924556953616654
**Kifap3**	Kifap3	-0.012835679847535	8.82689150732899	0.237279647816592	0.626177827467755	0.924556953616654
**Htr1d**	Htr1d	-0.103005314764327	-0.325083429803886	0.237246332725395	0.626202070180396	0.924556953616654
**Has2**	Has2	0.0372637699956471	2.79182954570043	0.237221614838066	0.62621277738839	0.924556953616654
**Camsap2**	Camsap2	0.0170035521679731	8.33548237159117	0.237158975092539	0.626267080161837	0.924556953616654
**Surf2**	Surf2	0.033661080597118	4.80985476827635	0.237026300421803	0.626362175575475	0.924556953616654
**Dok3**	Dok3	-0.058970606035750	0.648600371657926	0.237020305832708	0.626366528774321	0.924556953616654
**Pomk**	Pomk	-0.013702497887379	6.09099988571002	0.236992975089315	0.626386432490401	0.924556953616654
**Slc34a1**	Slc34a1	-0.059405573801084	0.473043874986099	0.236870003865306	0.626475859309542	0.924556953616654
**Hnrnpa2b1**	Hnrnpa2b1	-0.018207080459629	9.26148919483052	0.236805233087303	0.626523125978104	0.924556953616654
**Shd**	Shd	-0.016196022033069	6.04593340538655	0.236590782742589	0.626679340336402	0.924625294851529
**Rhbdd2**	Rhbdd2	0.0186892186200111	5.54385490250932	0.236538222261736	0.626717649960678	0.924625294851529
**Slf2d3**	Slf2d3	0.0272464328392673	3.2967657762996	0.236212331192618	0.626955253710789	0.924732986854197
**Ptpn18**	Ptpn18	-0.056456523192830	2.11915933097923	0.236167301484201	0.62698810032805	0.924732986854197
**Mrpl12**	Mrpl12	-0.020754040180409	6.11339065082302	0.235898980303503	0.627183905700933	0.924732986854197
**Scara3**	Scara3	0.0241332861267005	3.94360095045491	0.235801006145536	0.627255435910736	0.924732986854197
**Spata33**	Spata33	-0.025979646013457	3.24095754582327	0.235741175707723	0.627299126703931	0.924732986854197
**Ptgis**	Ptgis	0.0502759870879389	1.2736348273438	0.23547840331905	0.627491095717477	0.924732986854197
**Slc7a6os**	Slc7a6os	-0.017372363071503	5.2488201782584	0.235431038194527	0.627525712500486	0.924732986854197
**Kcnmb4**	Kcnmb4	0.0250724875053527	5.66822295003358	0.235086375142572	0.627777738984283	0.924732986854197
**Hps4**	Hps4	-0.026504617150579	5.17408315102568	0.235080750451175	0.627781853805338	0.924732986854197
**Armcx6**	Armcx6	-0.024010949599290	4.16184740927507	0.23495816930221	0.627871544630315	0.924732986854197
**Depdc7**	Depdc7	0.0577007325850336	0.801674165248123	0.2348655657729	0.627939320432182	0.924732986854197
**Cpped1**	Cpped1	0.0367252537197527	2.30846380028129	0.234848476366615	0.627951828374862	0.924732986854197
**Chtf18**	Chtf18	-0.030172939293502	3.09944702634344	0.234775304452704	0.628005397870161	0.924732986854197
**Cox16**	Cox16	-0.024564498817934	3.44717760488829	0.23471864957795	0.628046881111968	0.924732986854197
**Hsf2**	Hsf2	0.0161611473541025	6.21593479464267	0.234572825977647	0.628080438113008	0.924732986854197
**Chaf1b**	Chaf1b	0.0323456148550702	2.44968613696905	0.234546182980452	0.628099950127286	0.924732986854197
**Phkg1**	Phkg1	-0.063803743953271	0.48850821715097	0.234544461975293	0.628101211333142	0.924732986854197
**Cdc20**	Cdc20	0.0381022803058012	3.9350256717107	0.234512372938486	0.628124714561939	0.924732986854197
**Cdca7**	Cdca7	0.0428779935474516	4.2988586855652	0.234413461317715	0.628270449276949	0.924781082835826
**Psd2**	Psd2	0.0129892084280947	7.44597072208582	0.234365470785072	0.628305621479534	0.924781082835826
**Gtpbp10**	Gtpbp10	-0.025772834724011	3.28983593084865	0.233836673087715	0.62869347130421	0.925076042666064
**Prlr**	Prlr	0.0871281412150455	-0.234272049069387	0.233802073274958	0.62871886759768	0.925076042666064
**Zc3hc1**	Zc3hc1	0.0184580053551201	5.4091360886273	0.233789022216577	0.628729447688404	0.925076042666064
**Slc39a6**	Slc39a6	0.0135913698208616	7.05714105389916	0.233486827773554	0.628950364940141	0.925097356265466
**Epor**	Epor	-0.076443371859760	0.0349712081484089	0.233465175742893	0.628966271963031	0.925097356265466
**Pck2**	Pck2	0.0196891803634697	4.46126395823592	0.233444007228613	0.628981824642214	0.925097356265466
**Paics**	Paics	-0.017711026648090	5.67965453172795	0.233365502438396	0.629039510343529	0.925097356265466
**Orc3**	Orc3	-0.029198207326256	2.01445269725493	0.233121983051838	0.629218525741444	0.925251566962403
**Ifitm3**	Ifitm3	0.0690127680349888	1.57114023060243	0.232947735290097	0.629346889123895	0.925330973391332
**Atp23**	Atp23	-0.030617392573865	2.75353230172749	0.232236795600429	0.629870215505732	0.925991596425711
**Mir770**	Mir770	-0.10519474336797	4.81780018752974	0.231965742820327	0.630070074308998	0.926176286186778
**Crhr2**	Crhr2	-0.088469245284423	0.0431266321012282	0.231351282974089	0.630523677732284	0.926652316020398
**Nudt6**	Nudt6	-0.039406554210882	1.94071324588763	0.231307482234425	0.630556032216939	0.926652316020398
**Dlk1**	Dlk1	-0.082046794851674	4.0210525687396	0.231225369950266	0.630616719981119	0.926652316020398
**Tmem158**	Tmem158	0.0319131993385439	7.25270954041739	0.231101471953451	0.630708303715264	0.926677756177031
**Mr1**	Mr1	0.078247500697737	0.39392531846996	0.230434509461652	0.631201837745101	0.927248920559468
**Clasrp**	Clasrp	-0.047901796311468	6.14623925667852	0.230226003523498	0.631356300912971	0.927248920559468
**Thsd1**	Thsd1	0.0293549071784669	3.48492885239034	0.230160786439619	0.631404633401136	0.927248920559468
**Gabrb1**	Gabrb1	-0.043546056545286	3.53862520314832	0.23008169928643	0.631463256323225	0.927248920559468
**Apom**	Apom	-0.068079697584019	0.0522566210128765	0.229871916388589	0.631618816815652	0.927248920559468
**Lig4**	Lig4	0.0261648504928926	4.12772285383619	0.229834556338474	0.631646529602967	0.927248920559468
**Urm1**	Urm1	-0.024184222623964	5.4770933229225	0.229823901268897	0.631654433786657	0.927248920559468
**Dynlrb1**	Dynlrb1	-0.019804711165587	7.61534822354559	0.229677222518447	0.631763266454738	0.927248920559468
**Nhs**	Nhs	0.0484069520438505	3.65077928841239	0.229648478355159	0.631784599050689	0.927248920559468
**Phyhd1**	Phyhd1	-0.049478194294555	3.06310844714979	0.229545730877885	0.631860124541509	0.927248920559468
**Npff**	Npff	-0.082918752073182	0.827151072265426	0.229397089595905	0.631971253076117	0.927248920559468
**Phactr2**	Phactr2	0.0374332956932253	3.05393835882955	0.22937367225822	0.631988830414179	0.927248920559468
**LOC100910**	LOC100910	0.0428817010080972	2.39693402843418	0.229250678440785	0.632079990556463	0.927273617523221
**Slc9a8**	Slc9a8	-0.023729438590359	4.30156793847208	0.228341347519724	0.632756430865031	0.927889925940957
**Crlf1**	Crlf1	0.0729607906362733	4.51727312381878	0.22827650078437	0.63280324246927	0.927889925940957
**Nelfe**	Nelfe	-0.016751865463108	5.08453674322507	0.228265071315256	0.632813246481262	0.927889925940957
**Maea**	Maea	-0.013839792947071	6.8341505665965	0.228221194860474	0.632945823880616	0.927889925940957
**Gps1**	Gps1	-0.013280859702514	7.09742398270226	0.228171365208937	0.632882061016187	0.927889925940957
**Mtif2**	Mtif2	-0.026541240309029	4.50934163030514	0.227997154008747	0.633012901648738	0.927889925940957
**Kctd7**	Kctd7	0.026234692319442	3.82978574540188	0.227987010268151	0.633020463714789	0.927889925940957
**Hpx**	Hpx	0.0765436388425235	0.0203735893881106	0.22785649740463	0.633117778296534	0.927923237376816
**Tbc1d10b**	Tbc1d10b	0.0108202720120161	7.08267703761094	0.227616403576135	0.633296889381529	0.927920749497101
**Cldn6**	Cldn6	-0.096041049811253	-0.436790485203353	0.22756124112648	0.633338057200744	0.927920749497101
**Tmem205**	Tmem205	0.0308297459499566	3.52480266051169	0.227550575487314	0.633346017689934	0.927920749497101
**Crb1**	Crb1	-0.058040217485289	1.86405278715559	0.227180264477596	0.633622548007103	0.927948567642904
**Swap70**	Swap70	0.0258995313965115	3.77575414000283	0.227069990840221	0.633704948456932	0.927948567642904
**Wipi2**	Wipi2	-0.01446097333703	5.49373329701073	0.226992428686472	0.633762920411778	0.927948567642904
**Yars1**	Yars1	-0.017168926443717	7.02548899925757	0.226982870078797	0.633769318080414	0.927948567642904
**Rps4x**	Rps4x	-0.017744200956428	8.7056846405597	0.226974960951296	0.633775977924311	0.927948567642904
**Adamts16**	Adamts16	0.0452240133299516	1.48620870657213	0.226884079907542	0.633843823433502	0.927948567642904
**Akap5**	Akap5	-0.077365469507129	4.90214318550243	0.226837454758041	0.633878768332852	0.927948567642904
**Rnf125**	Rnf125	0.0704597613903308	-0.0139032591746211	0.226301343620399	0.634279992502476	0.92795843100928
**Yipf6**	Yipf6	0.0175085988654587	4.94661792303264	0.226294593153007	0.634285047986525	0.92795843100928
**Fxr2**	Fxr2	0.0121526880672388	6.89025019993387	0.226244911121064	0.634322258222985	0.92795843100928
**Kcnv1**	Kcnv1	-0.035055204278971	4.31462638698872	0.226137370962704	0.63440281946687	0.92795843100928
**Ggct**	Ggct	-0.027362499434530	3.00032809833396	0.226133844343792	0.634405461750283	0.92795843100928
**Rp2**	Rp2	0.0268974610032052	3.54203429339791	0.226121064493647	0.634415037134902	0.92795843100928
**Fbxw11**	Fbxw11	0.0098303051653749	7.49667331558136	0.226118242227066	0.634417151780948	0.92795843100928
**Apobec2**	Apobec2	-0.074505459182948	0.0352401171599011	0.225996574163958	0.634508329673587	0.92795843100928
**Iqcg**	Iqcg	-0.088070567430943	0.595749817422026	0.225934462518874	0.634554887662994	0.92795843100928
**Rnf8**	Rnf8	0.0147180122820093	4.92438754391525	0.225692828411219	0.63473608775817	0.92801723942444
**Mfsd9**	Mfsd9	-0.102848229498122	-0.58938400088313	0.225644622211192	0.634772251547954	0.92801723942444
**Pik3r3**	Pik3r3	0.0291079557571978	4.83086400062118	0.225583333372199	0.634818236634415	0.92801723942444
**Cop1**	Cop1	0.0152118098257439	6.12826971547593	0.224932462513223	0.635307058789158	0.928502746210142
**Plekha4**	Plekha4	-0.033808840478160	2.57007239778441	0.224777327018288	0.635423697700088	0.928502746210142
**Scn4b**	Scn4b	-0.074056286238131	1.20796870952187	0.224739118408067	0.635452432480649	0.928502746210142
**Rspo2**	Rspo2	0.0304792312577696	3.23727456886501	0.224634239166228	0.635531322280412	0.928502746210142
**LOC684871**	LOC684871	0.0477021247949833	1.36845105589331	0.224400728774967	0.635707069023104	0.928502746210142
**Prickle3**	Prickle3	0.0532828563607422	1.00158624602125	0.224395436729111	0.635711032815231	0.928502746210142
**Zdhhc20**	Zdhhc20	0.0247487730237296	4.31680727774554	0.224349816877584	0.635745377307542	0.928502746210142
**Dusp4**	Dusp4	0.0341114951878125	4.95457292494344	0.224314520361418	0.635771952903919	0.928502746210142
**Plpp7**	Plpp7	0.0272128363189056	4.27227164140075	0.224130236028543	0.635910746800652	0.928502746210142
**C2cd2**	C2cd2	0.0217379347411095	4.66005396739161	0.223943274358307	0.636051628128692	0.928502746210142
**Hsd3b7**	Hsd3b7	0.0430042142062897	2.60394563199825	0.223915168737676	0.636072812826025	0.928502746210142
**Golga2**	Golga2	-0.013531907894673	6.87232663239231	0.223738936025043	0.636205685870852	0.928502746210142
**Nubp1**	Nubp1	-0.015855842672478	5.59816795142362	0.223421282103679	0.636445347183178	0.928502746210142
**Mesp2**	Mesp2	-0.048263052119498	1.22608133885943	0.223419481695061	0.636446706136797	0.928502746210142
**Mtbp**	Mtbp	-0.036798820879846	1.78233074725645	0.223417460161855	0.636448227475022	0.928502746210142
**Prkcg**	Prkcg	-0.045115707444948	6.70501990538141	0.223053647385555	0.63672297800982	0.928502746210142
**Vps26a**	Vps26a	0.017755740002452	5.05546481001856	0.222944206419662	0.636805679816804	0.928502746210142
**Bcar1**	Bcar1	0.0154057392973129	6.65946090166373	0.222836351661044	0.636887207257207	0.928502746210142
**Ctu2**	Ctu2	-0.022172650676038	4.38055018066702	0.222740762391766	0.636959483380431	0.928502746210142
**Nutm1**	Nutm1	0.0595649618549105	0.852786224800477	0.222563811418951	0.637093328062517	0.928502746210142
**Steap3**	Steap3	-0.037605892009057	2.48393493135821	0.222447120281245	0.637181628154162	0.928502746210142
**Wdr81**	Wdr81	0.0218655865261067	5.40521217786049	0.222432978700098	0.637192330300772	0.928502746210142
**Hmgcs1**	Hmgcs1	0.0162814969666167	10.2153403605123	0.222283343989923	0.637305605302687	0.928502746210142
**Hras**	Hras	-0.020497866162618	6.71497616593769	0.222265279889825	0.637319283086337	0.928502746210142
**LOC691921**	LOC691921	-0.055864226713921	1.83034249685695	0.222227759401999	0.637347695780383	0.928502746210142
**Dhx16**	Dhx16	-0.020720106617720	5.57384361445156	0.222099054408233	0.637445177807601	0.928502746210142
**Cyc1**	Cyc1	-0.018185016699999	7.30022710385289	0.221910467845376	0.637588077518123	0.928502746210142
**Snrpb**	Snrpb	0.0224190735817953	6.56145490862385	0.221666549534286	0.637750062193444	0.928502746210142
**Glp1r**	Glp1r	-0.154126594155857	-0.754545299110069	0.221633177589865	0.637798326729793	0.928502746210142
**Thap11**	Thap11	0.0217673537180146	5.0836706341002	0.221536374525826	0.637871763351613	0.928502746210142
**Rnf26**	Rnf26	0.0163928528208313	5.28561513462915	0.221531246646968	0.637875654003353	0.928502746210142
**Rpl13**	Rpl13	-0.026391337710032	8.84776652506954	0.221432885911391	0.637950285847386	0.928502746210142
**Cryga**	Cryga	-0.054273373893210	1.64341426521028	0.221239515798359	0.638097088232174	0.928502746210142
**Mogat2**	Mogat2	0.042300095125440	3.50698185644329	0.221230561140885	0.638103887959707	0.928502746210142
**Ttc30b**	Ttc30b	0.0210301328890749	4.55879588041959	0.221170244452146	0.638149693872798	0.928502746210142
**Vps45**	Vps45	-0.015083365988548	5.95514239383566	0.221044085335194	0.638245526742376	0.928502746210142
**Mrpl20**	Mrpl20	-0.026261306148668	5.629783532873	0.221033058285293	0.638255777980707	0.928502746210142
**Acvr1c**	Acvr1c	0.0528022752633357	1.22327789976802	0.221000708306043	0.638278484474913	0.928502746210142
**Trpc4ap**	Trpc4ap	-0.010345981217101	7.75809798022193	0.220922605381865	0.638337826793463	0.928502746210142
**Dot1l**	Dot1l	0.0467962756258213	6.66334481688419	0.220785352493522	0.638442169654833	0.928502746210142
**Mtmr7**	Mtmr7	0.0128198228410276	5.95157593962728	0.220730555287659	0.638483824840387	0.928502746210142
**Dyrk3**	Dyrk3	0.0245141268929614	3.62130385523432	0.220540759988953	0.638628194968107	0.928502746210142
**Emd**	Emd	0.0181044442089297	5.63910182639835	0.220540415412145	0.638628457123951	0.928502746210142
**Canx**	Canx	-0.014850918003132	9.51580147864392	0.220537561913353	0.638630628103022	0.928502746210142
**Borcs6**	Borcs6	-0.02229779259812	4.84252505358876	0.220300632177015	0.63881094716809	0.928502746210142
**Oprk1**	Oprk1	-0.081435150410528	3.20834850413838	0.220291172227348	0.638818149266225	0.928502746210142
**Tmem176a**	Tmem176a	0.0493756730004668	3.19731025493351	0.220152454520888	0.638924541859614	0.928502746210142
**Ptprc**	Ptprc	0.0786777257857416	0.472240644720148	0.220123770937818	0.638945627314006	0.928502746210142
**Slc19a1**	Slc19a1	0.0244537578272534	4.24228186993474	0.219926719408612	0.639095760173447	0.928502746210142
**Gabarap**	Gabarap	-0.017299229598265	7.28314414288744	0.219898703022608	0.639117112419902	0.928502746210142
**Plekhh2**	Plekhh2	0.0470384108531163	3.00939870947943	0.219833467723859	0.63916683690412	0.928502746210142
**Dock11**	Dock11	-0.029868248219499	5.52606515588628	0.21970760317781	0.639262800427756	0.928502746210142
**Cdk5rap3**	Cdk5rap3	0.0170352310246281	5.51682339955867	0.219648808037491	0.63930763938411	0.928502746210142
**Lancl2**	Lancl2	0.0142633220769077	7.40554711599956	0.219631727238422	0.639320667091322	0.928502746210142
**Pigx**	Pigx	-0.019770550599057	5.68050847992149	0.219619262702103	0.639330174315756	0.928502746210142
**Dnaaf6**	Dnaaf6	-0.142894289472651	-0.258355473965536	0.219374262623234	0.639517112979339	0.928502746210142
**Map2k5**	Map2k5	-0.013978531116408	5.50082943729686	0.218926580369953	0.639859030177359	0.928502746210142
**Ubc**	Ubc	0.0282930004467679	5.84319921386144	0.218657096993084	0.640065053538168	0.928502746210142
**RGD130**	RGD1306	-0.017420147638340	4.25844632670834	0.218496379571079	0.640187997647687	0.928502746210142
**Ggnbp2**	Ggnbp2	-0.013373420452936	6.59634832755909	0.218405546246331	0.640257506845078	0.928502746210142
**Sgms1**	Sgms1	0.0200613526425572	4.11861963175564	0.21836246843931	0.640290477817238	0.928502746210142
**Cryl1**	Cryl1	-0.045741840466731	2.93857022953016	0.218352449743549	0.640298146513179	0.928502746210142
**RGD156**	RGD156164	0.0684241220492199	0.542135474127376	0.218271168920296	0.640360369913477	0.928502746210142
**Ik**	Ik	-0.011431872700472	6.82983007201935	0.218149094535178	0.640453848778857	0.928502746210142
**Wbp4**	Wbp4	0.0237696793701641	4.82416575950581	0.218130460033308	0.640468121013277	0.928502746210142
**Etaa1l1**	Etaa1l1	-0.024701655296880	3.46331562184244	0.218124619563582	0.640472594403651	0.928502746210142
**Spef2**	Spef2	-0.080108904190046	0.843727053505485	0.218109076290198	0.640484498284924	0.928502746210142
**Kdelr1**	Kdelr1	-0.012301231595141	6.67325841980098	0.218070754643975	0.640512854595386	0.928502746210142
**Gemin8**	Gemin8	0.021739423628162	3.89071376479685	0.218053789754952	0.640526866201282	0.928502746210142
**Ap3b2**	Ap3b2	-0.019298347109013	8.18760696575781	0.217898562714083	0.640645794187469	0.928502746210142
**Cep43**	Cep43	0.0292431089627304	3.82603865634012	0.217843375642559	0.640688092923127	0.928502746210142
**Tmem128**	Tmem128	-0.021099571421047	4.998099572325	0.217822768671965	0.640703890408093	0.928502746210142
**Poglut2**	Poglut2	0.0395764700769106	4.48092118957539	0.217804657068877	0.640717774794089	0.928502746210142
**Lars2**	Lars2	-0.017217483848663	4.57852010415502	0.217794600356289	0.640733151126281	0.928502746210142
**Ppp1r12b**	Ppp1r12b	0.0296641341531564	6.94093951669094	0.217663779845537	0.640825795529685	0.928502746210142
**Ube2i**	Ube2i	0.0123901684027397	6.86101705864853	0.217596780526043	0.640877194418661	0.928502746210142
**Klhl20**	Klhl20	-0.015861679504924	5.10957148674496	0.217592480549539	0.640880472097599	0.928502746210142
**Tpd52**	Tpd52	-0.026125762120436	4.78197214810232	0.217028408981648	0.641313509808906	0.928579515856832
**Rpl13a**	Rpl13a	-0.021585898169581	9.12340544065661	0.216904989768033	0.641408340166547	0.928579515856832
**Vti1b**	Vti1b	-0.014898020511553	5.98683235190774	0.21687961168292	0.641427853001586	0.928579515856832
**Ap5s1**	Ap5s1	0.0300074388270434	2.97607378649718	0.21684312450197	0.641455898840313	0.928579515856832
**Opcml**	Opcml	0.0215054128623468	7.52085785027482	0.216742989680974	0.641532874446334	0.928579515856832
**Tpsg1**	Tpsg1	-0.065797869793825	0.0670726911712586	0.216727418922034	0.641544854823382	0.928579515856832
**Dnaaf2**	Dnaaf2	0.0188003636648729	4.36527549078336	0.216635580047918	0.641615480028963	0.928579515856832
**Phlda3**	Phlda3	-0.033647083959064	3.62267495987615	0.216378780741201	0.64181307805502	0.928579515856832
**Cenpw**	Cenpw	0.0428204002482736	1.98118875645848	0.216268873927035	0.641897674586847	0.928579515856832
**Slc35e1**	Slc35e1	0.01211026548508	6.48057038431899	0.216254843903648	0.641908477026513	0.928579515856832
**Pibf1**	Pibf1	-0.026804411638077	4.147805784136	0.216205420812429	0.641946523460255	0.928579515856832
**Mir186**	Mir186	-0.088679724621176	1.11056366905446	0.21607737012576	0.642045158797174	0.928579515856832
**Uhrf2**	Uhrf2	0.0295291278823781	6.62393203673204	0.215975685877936	0.642123501877328	0.928579515856832
**Tmem17**	Tmem17	-0.022796499895789	4.05274314024618	0.215973431997099	0.642125238664851	0.928579515856832
**Nagk**	Nagk	0.021096449761268	5.39425405185646	0.215741103003595	0.642304322875277	0.928579515856832
**Hacd3**	Hacd3	0.0116812786151297	7.50472891282826	0.215740833533722	0.642304530656555	0.928579515856832
**Pou2f1**	Pou2f1	0.0545234828076 84	3.01987873417057	0.215689140753128	0.642344392546572	0.928579515856832
**Noa1**	Noa1	0.0149474209119762	5.11738880080523	0.215677432335376	0.64235342207352	0.928579515856832
**Col6a4**	Col6a4	0.0682291881529442	1.63661923014645	0.215604947942026	0.64240932864075	0.928579515856832
**Zc3h8**	Zc3h8	0.0304049418254617	4.10027136553036	0.215553456671358	0.642449050339796	0.928579515856832
**Htra4**	Htra4	0.0703749864549562	1.55757342981722	0.215492151683364	0.642496350115575	0.928579515856832
**Rpusd4**	Rpusd4	0.0224531253273273	3.76849664909883	0.215289560922578	0.642652716917354	0.928659907875966
**Cnot11**	Cnot11	-0.016361020481334	4.7075293890761	0.215165767573898	0.642747526744937	0.928659907875966
**Prtfdc1**	Prtfdc1	0.023192421267034	4.71171570851123	0.214922726358084	0.642936080638652	0.928659907875966
**Ibsp**	Ibsp	-0.072058669614490	0.0597754367254218	0.214743946002429	0.643074287171943	0.928659907875966
**Zfp667**	Zfp667	0.0288142113486899	4.39587006469615	0.214506882835995	0.643257656394378	0.928659907875966
**Prrt3**	Prrt3	-0.017700181826896	5.21362031202027	0.214288692036021	0.643426528046505	0.928659907875966
**Scamp5**	Scamp5	-0.020146024418578	6.47388605602315	0.214046094709119	0.64361443232921	0.928659907875966
**Ptdss1**	Ptdss1	-0.011069837114628	6.46491503643446	0.213974167413831	0.64367016564908	0.928659907875966
**Spon1**	Spon1	-0.022954592842919	6.98290104576551	0.213939840831755	0.643700642909238	0.928659907875966
**Zbtb22**	Zbtb22	-0.018698342417135	5.61332800926583	0.213840169081568	0.643774026215364	0.928659907875966
**Slc25a1**	Slc25a1	0.0145497450208312	6.74400042482504	0.213822661031443	0.643787598544561	0.928659907875966
**Cenpj**	Cenpj	0.0282683696760626	4.12635283870062	0.213808360208517	0.643798685748692	0.928659907875966
**Dennd5a**	Dennd5a	-0.013370806129780	8.0468703411 4011	0.21366327 38222	0.643911116843879	0.928659907875966
**Fnbp1l**	Fnbp1l	0.0137111291236728	8.62967982967529	0.21363632481826	0.643932097379557	0.928659907875966
**Accs**	Accs	-0.035968825395202	2.12420987840487	0.213582324813288	0.64397398725678	0.928659907875966
**Gstm5**	Gstm5	-0.025814094009440	5.22952814998025	0.213520157213978	0.644022221009368	0.928659907875966
**Bsg**	Bsg	0.022731749758188	8.68326686522205	0.213467612416267	0.64406298539849	0.928659907875966
**Rimbp2**	Rimbp2	-0.024843499785080	7.11918029250775	0.213438015675109	0.644085964940108	0.928659907875966
**Cenpk**	Cenpk	0.041566638363792	1.66020601699643	0.213357940835351	0.644148119388107	0.928659907875966
**Nap1l1**	Nap1l1	-0.012116559803894	8.01678564922984	0.213308149937887	0.644186774441038	0.928659907875966
**Zdhhc6**	Zdhhc6	-0.016704383515572	5.36861366371592	0.213256103195071	0.644227186666381	0.928659907875966
**LOC361346**	LOC361346	-0.026401250850634	3.47882941861376	0.213194686839701	0.644274881641923	0.928659907875966
**Scamp2**	Scamp2	0.0182959300627876	4.71779952146491	0.213169780788554	0.64429421567584	0.928659907875966
**Tnn**	Tnn	-0.060432752198430	1.36765048845799	0.213113055617029	0.644338288248957	0.928659907875966
**Epb41l1**	Epb41l1	-0.014152108814733	9.24606481028205	0.212628306002827	0.644715118199883	0.929047585247226
**Tmem214**	Tmem214	-0.017112276472253	6.310366304089	0.212575506195442	0.644756194650615	0.929047585247226
**Rabep1**	Rabep1	-0.012823638585697	7.56030444133133	0.212358791302883	0.644924856278989	0.929162282960165
**Zim1**	Zim1	-0.046939097535157	4.01078542615413	0.212281885382893	0.64498473455188	0.929162282960165
**Map4**	Map4	-0.013576803958405	9.00825954127205	0.211779403418269	0.645376286551603	0.929404491915145
**Jtb**	Jtb	-0.014342218629522	5.4945520683394	0.211694151640245	0.645442773638846	0.929404491915145
**Dctpp1**	Dctpp1	-0.026887515779813	3.72116879530477	0.21168521726419	0.645449742416618	0.929404491915145
**Sil1**	Sil1	-0.031315415060809	3.21109144303522	0.211683896300833	0.645450772777901	0.929404491915145
**Atp2b3**	Atp2b3	-0.023228358898533	6.08782969551071	0.211315582646364	0.645738212265403	0.929465525734746
**Crppa**	Crppa	0.0297511015884018	2.61555806603877	0.211282568345425	0.645763992113988	0.929465525734746
**Ssbp1**	Ssbp1	-0.027236318701805	3.26489697761704	0.211107955178875	0.645900382736356	0.929465525734746
**Atg4a**	Atg4a	-0.033482701831656	3.22964849511382	0.210967246406199	0.646010340296463	0.929465525734746
**Slc27a5**	Slc27a5	-0.086614287552461	-0.362768587900856	0.210950731070355	0.646023249188222	0.929465525734746
**Mea6**	Mea6	-0.014554204333685	6.09930416373847	0.210949840341584	0.646023945437011	0.929465525734746
**Fam83f**	Fam83f	0.0628162144074726	0.885165786717337	0.210909946131991	0.646055130600171	0.929465525734746
**Stard6**	Stard6	-0.058686644820671	0.829018900109694	0.210866412914307	0.646089164438362	0.929465525734746
**Gltld1**	Gltld1	-0.03074296098129	5.37839804994769	0.210703886876246	0.646216263283521	0.929520316568073
**Pcdhga10**	Pcdhga10	0.0281229494101346	4.72134894988553	0.210567268368024	0.646323147803886	0.929520316568073
**Mrps26**	Mrps26	-0.023013222818196	5.50781006794153	0.210359664493666	0.646485648645507	0.929520316568073
**Gna15**	Gna15	-0.073658010257092	0.16970786505266	0.210327552650909	0.646510792687906	0.929520316568073
**Alg11**	Alg11	0.0149982510965423	4.86609528706467	0.210258144381264	0.646565148316355	0.929520316568073
**Senp3**	Senp3	0.0130441983219529	6.55593361885461	0.210172482585218	0.646632246740964	0.929520316568073
**Kcnip3**	Kcnip3	0.0328043350470862	2.89271036631178	0.210141927698226	0.646656185285796	0.929520316568073
**Glrx**	Glrx	-0.018913194511790	6.28370043224488	0.209567429708969	0.647106659570561	0.929699068188935
**Cnep1r1**	Cnep1r1	-0.016847820643240	4.82239100217673	0.209510077717376	0.647151671292798	0.929699068188935
**Agtrap**	Agtrap	-0.028010543404202	3.08783023843577	0.209440351061716	0.647206405094736	0.929699068188935
**Pmpca**	Pmpca	-0.012545026400680	5.76485339946882	0.209316929310184	0.647303315318232	0.929699068188935
**Ubr3**	Ubr3	-0.013880449421855	7.58979179919024	0.209290641060548	0.647323961200932	0.929699068188935
**Shroom3**	Shroom3	0.0344290769985029	3.40922953836717	0.209252303115804	0.647354073308699	0.929699068188935
**Msra**	Msra	-0.030336759552154	5.59079547956076	0.209232468066062	0.647369653834927	0.929699068188935
**Zbtb5**	Zbtb5	0.0118908048920655	6.24805193319485	0.208987732264845	0.647561968501278	0.929699068188935
**Mvb12a**	Mvb12a	0.0179870952602267	4.96440140388399	0.208981799981565	0.647566621809992	0.929699068188935
**Etf1**	Etf1	-0.011541314838199	6.58242231253268	0.208631988800811	0.647841756482181	0.929699068188935
**Cyp4f17**	Cyp4f17	-0.045289449332168	2.82044378929808	0.208558862571484	0.647900879229438	0.929699068188935
**Nphp4**	Nphp4	0.0251417251534521	4.56292226557337	0.208461384448853	0.647976037058648	0.929699068188935
**Dda1**	Dda1	-0.015080898672576	7.03520480259819	0.208444400630981	0.647989408445586	0.929699068188935
**RGD130**	RGD130526	-0.020571045553109	5.63471636594236	0.208433589014178	0.647997917642296	0.929699068188935
**Vldlr**	Vldlr	0.0192845295096087	5.97683677949915	0.208292807361659	0.648108787238932	0.929699068188935
**Rbs1**	Rbs1	-0.015749173793140	7.11283372473077	0.208191582304267	0.648188522717394	0.929699068188935
**Metrnl**	Metrnl	-0.041100023961731	2.39914894226852	0.208168819550991	0.648206465927095	0.929699068188935
**Ppp1r14a**	Ppp1r14a	0.0643867610075514	0.553474538481703	0.208058380796903	0.648295092322496	0.929699068188935
**Zfp653**	Zfp653	-0.019547261385612	4.66103586191716	0.207993258545649	0.648344834562733	0.929699068188935
**Yy2**	Yy2	-0.026198831792815	2.98042659362808	0.20795376241152	0.648375973212009	0.929699068188935
**Mthfd2**	Mthfd2	0.0308622621647553	4.50678320746452	0.2078805611566	0.648433894337039	0.929699068188935
**Hipk3**	Hipk3	0.0210669297027688	5.42172552949847	0.207627445104777	0.648633377693322	0.929699068188935
**Nfe2l1**	Nfe2l1	-0.012690347614894	7.32423209567415	0.207522745398814	0.648716018309277	0.929699068188935
**Dnajc5**	Dnajc5	-0.011568346187389	6.97090163894168	0.207323240988226	0.648873559020762	0.929699068188935
**Clk2**	Clk2	0.026968832221436	5.22751509375683	0.207240110647732	0.648939230762284	0.929699068188935
**Lrfn4**	Lrfn4	-0.013274443940655	7.10797278588033	0.207008682276219	0.649122121641414	0.929699068188935
**Kirrel2**	Kirrel2	0.0720391985743644	1.76252570052796	0.206969872671053	0.649152824695105	0.929699068188935
**Casp3**	Casp3	0.0153256468555405	7.49459039290708	0.206920959262543	0.649191504238803	0.929699068188935
**Slc25a20**	Slc25a20	0.0162774606652126	5.60986487093202	0.206880731428825	0.649223318133342	0.929699068188935
**Actr6**	Actr6	-0.019920085627444	4.2339588625592	0.206840004754064	0.649255521136418	0.929699068188935
**Stmn1**	Stmn1	0.0236107042231864	11.5378101915453	0.206699300376265	0.649390093844896	0.929699068188935
**Polk**	Polk	-0.033860152736256	2.10600348266666	0.206557339796973	0.64947921217785	0.929699068188935
**Lrrc24**	Lrrc24	0.017401486676 5477	4.6241929873282	0.206506639572048	0.649519352276601	0.929699068188935
**Mob3b**	Mob3b	0.0401329806002872	2.1822914697779	0.206493614133372	0.649529665663974	0.929699068188935
**Eif4ebp3**	Eif4ebp3	-0.038453924600011	2.89529460239945	0.206296778979564	0.649685565193157	0.929699068188935
**Trappc2b**	Trappc2b	0.0257119529531338	3.56189042324337	0.206265874423181	0.649710050705805	0.929699068188935
**Fnbp1**	Fnbp1	0.0182161291988558	5.86000314835818	0.20598649462211	0.649931502286141	0.929699068188935
**Nup214**	Nup214	0.0261630413349084	6.06752388086718	0.205639882023419	0.650206498103483	0.929699068188935
**Trak2**	Trak2	0.0138323399233494	7.51258572046062	0.205630420457311	0.650214008646031	0.929699068188935
**Irak2**	Irak2	-0.042881808476996	1.55895556714133	0.205480479832765	0.650333058561842	0.929699068188935
**Ndc80**	Ndc80	0.0485790888842967	2.21844379440799	0.205391532078839	0.650403705285883	0.929699068188935
**Pcgf5**	Pcgf5	-0.047159037174626	1.56364701577231	0.205371116148427	0.65041992421036	0.929699068188935
**Lpxn**	Lpxn	0.0236399671627141	1.97848594764308	0.205368036663756	0.650422370594328	0.929699068188935
**Spaca6**	Spaca6	-0.063495548958776	2.81879626072932	0.205238263690575	0.650525484089362	0.929699068188935
**Laptm4a**	Laptm4a	0.0230857946100767	7.71143338923443	0.205152659455862	0.650593524012417	0.929699068188935
**Tor2a**	Tor2a	0.0171148719693638	4.64979261762146	0.205130539978839	0.650611107787472	0.929699068188935
**Pnpla1**	Pnpla1	0.129886154867334	1.4830366911797	0.205037489984306	0.650685089961181	0.929699068188935
**Alg13**	Alg13	-0.020234761987498	4.10748250305877	0.205029480609724	0.650691458988862	0.929699068188935
**Rragd**	Rragd	-0.015285499548099	5.51026184871343	0.204997459617573	0.650716923521688	0.929699068188935
**Stx19**	Stx19	-0.112590726048007	0.0327204921095206	0.204912882195139	0.650784186936525	0.929699068188935
**Wdr31**	Wdr31	-0.022582846495952	3.39611227834508	0.204911075817849	0.650785621652266	0.929699068188935
**Scn2b**	Scn2b	-0.029237807708844	3.58698533409864	0.204897602250512	0.650796349951978	0.929699068188935
**LOC257650**	LOC257650	-0.046194820655258	2.20692439001704	0.204829614907766	0.650850440347647	0.929699068188935
**Krt77**	Krt77	-0.090678930609254	1.07996269889485	0.204826123878286	0.650853218084568	0.929699068188935
**Tbca**	Tbca	-0.021838385154340	6.43818234071615	0.204803286447047	0.650871312064627	0.929699068188935
**Kcnb2**	Kcnb2	0.0292761671255288	3.4644067921955	0.204543083345904	0.651078522366074	0.929888590151272
**Atp2a3**	Atp2a3	0.0776221603727108	0.896154436720468	0.204323110602076	0.651253753499077	0.930032400412955
**Prr32c**	Prr32c	-0.055883977858892	0.697293011118805	0.204033082161647	0.651484964184411	0.930187899840668
**Pcdhgb8**	Pcdhgb8	0.0339808948226536	3.34611180613002	0.203999504268552	0.651521746149523	0.930187899840668
**Cckar**	Cckar	0.0509078866295596	0.842725074762825	0.203522818394035	0.651892229411632	0.930212848221111
**Ift52**	Ift52	0.0133634508613835	5.4901404978537	0.203519854617674	0.651894596728681	0.930212848221111
**Nfyb**	Nfyb	0.0183624056540266	5.79345403720354	0.203499036168504	0.651911226053634	0.930212848221111
**Lypd3**	Lypd3	0.0358067719986661	2.44398733789614	0.2034609991656	0.651941611831568	0.930212848221111
**RGD130**	RGD130762	0.0330829967624857	2.70137156779448	0.203246979168171	0.652112664996278	0.930212848221111
**Tspan11**	Tspan11	-0.018698638017739	4.98480483069935	0.203078878282859	0.652247058089432	0.930212848221111
**LOC100**	LOC100910	0.0113675999863885	7.91496388398639	0.203043800522402	0.652275109828841	0.930212848221111
**Ccdc47**	Ccdc47	-0.010846103950431	7.76608977131164	0.202958282036413	0.652343527815278	0.930212848221111
**Creg1**	Creg1	0.0256534108896775	3.87385470066479	0.20279921892579	0.652470821455149	0.930212848221111
**Dcaf10**	Dcaf10	-0.017093052577332	5.08767822831878	0.202659795009083	0.652582467803237	0.930212848221111
**Ercc2**	Ercc2	0.0212226480950114	4.99107728247167	0.20252089175358	0.652693623115135	0.930212848221111
**Mzf1**	Mzf1	-0.025760367889064	5.72032078252719	0.202461609561723	0.652741199635844	0.930212848221111
**Mpp5**	Mpp5	-0.025697227498246	3.8426750645852	0.202434219695157	0.652763146897632	0.930212848221111
**Arsa**	Arsa	-0.022867302288809	4.30386490982508	0.202266985080819	0.652897181975582	0.930212848221111
**Rgl2**	Rgl2	-0.025796362005584	5.96348482315889	0.202254637285094	0.652907089720618	0.930212848221111
**Slc25a19**	Slc25a19	0.0244299666028576	3.64951377409478	0.202241540038081	0.652917590710906	0.930212848221111
**Syngr1**	Syngr1	-0.015809485251204	6.07295942367018	0.202232298903603	0.652925000228716	0.930212848221111
**Asmt**	Asmt	0.0557872164281157	1.59237684300833	0.202174400636876	0.652971422490289	0.930212848221111
**Uspl1**	Uspl1	0.0116488058933452	6.07443623966406	0.20214132922608	0.652997950347073	0.930212848221111
**Gatc**	Gatc	0.0136718950824677	5.6058415370142	0.20209746858301	0.653033212979188	0.930212848221111

Rpa3	Rpa3	0.0274642676488251	3.65411515092014	0.201947431033837	0.653153506456661	0.930212848221111
Myh7	Myh7	-0.0361457022100701	2.95018278154725	0.201927610358595	0.653169412780729	0.930212848221111
Arrb1	Arrb1	-0.0160941167620982	6.190130227658	0.201568744124714	0.653457569560573	0.930517016138098
Cacna1i	Cacna1i	-0.0291614759927756	6.32737060971552	0.201397004910476	0.653595579075445	0.930542402640646
Vipas39	Vipas39	-0.0122232355899061	6.22346150772385	0.201360954015158	0.65362455585271	0.930542402640646
Cep89	Cep89	-0.017883371764117	4.66760236475541	0.201096389983334	0.653837323293865	0.930663656319314
Agmo	Agmo	0.0823811576709261	-0.537391619248735	0.201051759853385	0.653873231813206	0.930663656319314
Cdc5l	Cdc5l	0.0122558518036259	6.46686142982072	0.200975867302039	0.653932499635442	0.930663656319314
Trmt9b	Trmt9b	0.0296995764723616	2.61403500215931	0.200866126026426	0.654022640499899	0.930666312983868
Dgat1	Dgat1	-0.0288027440223737	5.22193396167367	0.200755455394727	0.654111753984696	0.930666312983868
Dennd2b	Dennd2b	0.0321957529492885	4.9589447661022	0.200696623882124	0.654159137919061	0.930666312983868
Rars1	Rars1	-0.0162669542494758	6.11833080394488	0.200579927588258	0.654253151845955	0.930693943174323
Pdss2	Pdss2	-0.0231572985254412	4.10670891764427	0.199847121383009	0.654844273532498	0.931379912184688
Polr3e	Polr3e	-0.0177878898017835	5.39588506165814	0.199797106881434	0.654884665351949	0.931379912184688
Slc25a40	Slc25a40	-0.0436763330015721	1.45023935188265	0.199640183218515	0.655011428464488	0.931454022447164
Prkg2	Prkg2	0.0396924007067436	2.01105362158809	0.199105618574073	0.655443735465454	0.931962562668649
Dhx57	Dhx57	0.0158069408903292	7.63956269206814	0.198875142493641	0.655630334629067	0.932025982907073
Arg2	Arg2	0.0203935909005256	4.7300064892954	0.198832881231127	0.655664556360001	0.932025982907073
Tuba1b	Tuba1b	-0.0237065777764543	10.787862607619	0.198764478670014	0.655719676877557	0.932025982907073
Vcan	Vcan	0.0614463041641354	7.82178837944455	0.198504245049392	0.655930894760358	0.932025982907073
Cnot2	Cnot2	0.0114843571832718	5.97123722112991	0.198457919025486	0.655968458113532	0.932025982907073
Arfgef2	Arfgef2	0.0199991788900218	5.60080823212222	0.198397948211493	0.656017095413405	0.932025982907073
Gorasp2	Gorasp2	0.0107261521712472	6.80682152372962	0.198316467639664	0.656083190233219	0.932025982907073
Adipor1	Adipor1	0.0160124017946817	6.61005876058701	0.19831309467991	0.656085834749766	0.932025982907073
Slc4a4	Slc4a4	0.041788756741226	5.42160544718125	0.197932335817126	0.656394194639154	0.932301161320579
Pld5	Pld5	-0.0654510591708671	1.46631959676629	0.197890380596661	0.656429085693779	0.932301161320579
Gpaa1	Gpaa1	-0.0132252489439526	6.2604417891536	0.197502674268165	0.65674421169581	0.932370889354266
Tmem12	Tmem120b	0.0289487312204472	3.49747800442636	0.197280112237593	0.656925276927756	0.932370889354266
Ino80	Ino80	0.0225272517781887	5.56073266422873	0.197253082697117	0.656947274652447	0.932370889354266
Insyn2a	Insyn2a	0.0284262497620217	4.06917237502021	0.196935945438071	0.657205513500826	0.932370889354266
Gmppa	Gmppa	0.0219669044966511	4.39917199400608	0.196908412437622	0.657227944839052	0.932370889354266
Etv3	Etv3	0.0400809560880972	3.01796621912952	0.196857290953602	0.65726959886717	0.932370889354266
Tyk2	Tyk2	0.0351470502285896	4.27618986922536	0.19675191077593	0.65735548356601	0.932370889354266
Gorab	Gorab	0.0277679774530082	3.57621828332216	0.196721667210763	0.657371885185105	0.932370889354266
Epas1	Epas1	0.0258449466286576	5.61053157968386	0.196566759376353	0.65750644838952	0.932370889354266
Palm	Palm	0.0116625344124819	9.23276868491345	0.196234247921879	0.65777778252106	0.932370889354266
Wdfy2	Wdfy2	0.0297239916086527	4.88380546859451	0.196213625079693	0.657794615577537	0.932370889354266
Serbp1	Serbp1	-0.0122607005862657	8.48460515164528	0.196198689348364	0.657806810375707	0.932370889354266
Lars1	Lars1	0.0131332380831382	6.9967222189711	0.196144617087292	0.657850964201317	0.932370889354266
Asap1	Asap1	0.0161050847801117	6.30925685166296	0.196040332491656	0.657936140512566	0.932370889354266
Taf6	Taf6	0.0133982574054175	5.97899512949705	0.196001581579758	0.657967789748967	0.932370889354266
Bod1	Bod1	0.0183549962765242	6.00111237382234	0.195845307761815	0.658095503200465	0.932370889354266
Ccdc146	Ccdc146	-0.0753633178025606	0.927671722847887	0.195781738498226	0.658147468751982	0.932370889354266
Shld2	Shld2	0.0281092034821845	2.79261629935834	0.195734113815924	0.658186406800956	0.932370889354266
Tnks2	Tnks2	-0.0160719021831001	8.05092607790968	0.195680269294658	0.658230437035468	0.932370889354266
Qk	Qk	-0.0247276765233441	6.1607345936486	0.195635751675724	0.658266845845736	0.932370889354266
Ddx31	Ddx31	0.0379557362539345	3.24003986810251	0.195594322727388	0.658300733037987	0.932370889354266
Cntfr	Cntfr	-0.0194849187654996	5.85312270382174	0.1955567701729	0.658331453205877	0.932370889354266
Papss2	Papss2	-0.0445878642989622	2.0584580435111	0.195534300345244	0.658349826514644	0.932370889354266
Dlgap5	Dlgap5	-0.0339123811636565	3.06267112969288	0.195456580111891	0.658413429291072	0.932370889354266
Mb21d2	Mb21d2	-0.0138443422176284	6.20446272159206	0.195392398004273	0.658465960688962	0.932370889354266
Kcnh4	Kcnh4	-0.0373268262650304	4.70381681519087	0.195264444618019	0.658570713119535	0.932370889354266
Acat1	Acat1	0.0172330744063643	7.45080362838415	0.195234530618052	0.658595208924452	0.932370889354266
Mamdc4	Mamdc4	-0.0407659637954785	2.42988355967834	0.195171480251886	0.658646846594378	0.932370889354266
Prss36	Prss36	0.0460853298573315	1.82708190351259	0.194975434542826	0.658807469940499	0.932370889354266
Alkal2	Alkal2	-0.0735357771384561	0.0978357108831233	0.194852404893481	0.658908319361851	0.932370889354266
Ggt1	Ggt1	0.0551712540511293	0.642480894541941	0.194849769941494	0.658910479691274	0.932370889354266
Ggact	Ggact	-0.0389426496094416	2.57653240844992	0.194693513273251	0.659038621735742	0.932370889354266
Utp18	Utp18	-0.0173268823106155	5.22471961973367	0.194659309863702	0.659066679260518	0.932370889354266
Chmp3	Chmp3	0.0147662062133592	5.14535502685432	0.194623607948138	0.659095869171619	0.932370889354266
Saxo2	Saxo2	-0.0897210265321836	-0.224967736979179	0.194521082803622	0.659180097996572	0.932370889354266
Lyar	Lyar	-0.0175404405799205	4.2304702781631	0.194444730210087	0.659242769108276	0.932370889354266
Pthlh	Pthlh	-0.0545388502027835	0.736996744179918	0.194346225376357	0.659323643698184	0.932370889354266
Glod4	Glod4	-0.015584022054384	6.09608823148702	0.194186228263784	0.659455058939847	0.932370889354266
Mast3	Mast3	0.0199794685760794	7.18022954266984	0.193957060594997	0.659643395963571	0.932370889354266
Map9	Map9	0.0147522321579258	7.47390816019448	0.193955561137308	0.659644628520401	0.932370889354266
Dag1	Dag1	-0.0122153365762754	7.92124382894793	0.193899870498868	0.659690664060711	0.932370889354266
Tcirg1	Tcirg1	-0.0350894125005244	2.94683374190028	0.193700792740586	0.659854164370927	0.932370889354266
Wdr12	Wdr12	-0.0146609969726995	5.49402442534058	0.193669481957635	0.659867307063631	0.932370889354266
Fasn	Fasn	0.0223566625841299	10.4136796012778	0.193628566779473	0.659913599300567	0.932370889354266
Eif4a2	Eif4a2	-0.0147252164887723	9.07232123881432	0.193619986654284	0.659920658135412	0.932370889354266
Castor2	Castor2	0.0168151767455365	7.41877931888659	0.193540870543814	0.65998577729951	0.932370889354266
Pus7	Pus7	0.019399458689644	4.27318937467608	0.193509940169037	0.660011228989519	0.932370889354266
Tanc1	Tanc1	0.0299384075596137	3.46012261048021	0.193412051267131	0.660091848317818	0.932370889354266
Itih3	Itih3	0.0535807291467039	4.92308026426222	0.193306827558264	0.660178424870889	0.932370889354266
Hmgcll1	Hmgcll1	-0.0170952606684307	5.09135652523889	0.193253429118565	0.660222496377195	0.932370889354266
Entr1	Entr1	-0.0298033223764605	6.50329337907352	0.193125963498153	0.660327623212987	0.932370889354266
Wrnip1	Wrnip1	-0.0140745559888131	6.14155057207607	0.193032690739969	0.660404430877031	0.932370889354266
Ruvbl1	Ruvbl1	-0.0169258027312514	6.19270769132389	0.192991103395912	0.660438720855414	0.932370889354266
Cyp4f1	Cyp4f1	0.0738190082492617	-0.280221136147348	0.192843863346267	0.660560159914448	0.932436927158341
Pla2g3	Pla2g3	0.0494357357232936	3.14458116820765	0.192658462331967	0.660713051871725	0.932446484801375
Ciart	Ciart	-0.0508813038551845	1.55731023075801	0.192637303062938	0.660730617949823	0.932446484801375
Casq1	Casq1	0.0612818673871155	0.832258721363553	0.192346072945583	0.660971132501353	0.932446484801375
Cdc42se	Cdc42se1	0.0135840621186995	5.68725209284801	0.192311178229609	0.660999965123438	0.932446484801375
Grm5	Grm5	-0.0201673824282274	7.36617234222598	0.192209962318771	0.661083614920622	0.932446484801375
Bmp2	Bmp2	0.0647441637769297	1.48696643740229	0.192204369225379	0.661088238093075	0.932446484801375
Gga3	Gga3	0.0174077314962119	6.518426479079	0.192203447681578	0.661088999835935	0.932446484801375
Ufm1	Ufm1	-0.0165213729080463	5.25914648634087	0.191702629600435	0.661503295704658	0.932765472049739
C2cd4c	C2cd4c	0.0191822224193501	5.26262513081587	0.191672178812034	0.661528506551181	0.932765472049739
Alox15	Alox15	-0.0745422180572487	3.64535231632599	0.191595038379219	0.661592383415885	0.932765472049739
Dnaaf3	Dnaaf3	0.0469642110595251	2.356253288344	0.191485189424967	0.661683371332267	0.932765472049739
Sdcbp	Sdcbp	0.0146678532237364	7.43235165237533	0.191406345862134	0.661748696646064	0.932765472049739
Cers5	Cers5	0.0129479443463141	6.29138801129171	0.191386859999007	0.661764843999702	0.932765472049739
Arhgap1	Arhgap15	-0.0637169658939992	0.102900167558256	0.190989006751671	0.662094747458146	0.932878975386591
Prcc	Prcc	0.0118558510347135	6.82704960101484	0.190869338847268	0.662194057514615	0.932878975386591
Ptpa	Ptpa	-0.0126568045335055	7.81919564459008	0.190847607553472	0.662212095500799	0.932878975386591
Nlrx1	Nlrx1	-0.0319821504082727	3.49493181206839	0.190804612128119	0.662247787925721	0.932878975386591
Ndufb4	Ndufb4	-0.0230973605295846	6.0667006826656	0.190764038900383	0.662281473966803	0.932878975386591
Chml	Chml	-0.0317387871562595	3.00869341075919	0.190692667138627	0.662340740937416	0.932878975386591
Sez6l	Sez6l	-0.0193718535481976	7.93321269309632	0.190658947888503	0.662368745928493	0.932878975386591
Eno4	Eno4	0.0635868118960323	1.32058800020995	0.190497148663084	0.662503166761675	0.932962981002887
Zfp385d	Zfp385d	-0.0526026622362365	2.26003150655469	0.190330278525906	0.662641871653053	0.933052999956567
Anapc15	Anapc15	0.0218231473903814	4.50491205177408	0.190155545917179	0.662787189539774	0.933071095809471
Fbxo6	Fbxo6	-0.0166928817962673	4.58571085859997	0.190062382848517	0.662864701892568	0.933071095809471
Cited1	Cited1	0.0415877408034684	2.11112009951438	0.190045113074056	0.662879072954649	0.933071095809471
Xrcc1	Xrcc1	-0.0259422087365124	6.3498512267835	0.189781547160649	0.663098496111121	0.933112896909279
Itgb6	Itgb6	0.103176478479089	0.287271038364318	0.189732905375067	0.663139011047002	0.933112896909279
Camsap1	Camsap1	0.0105050296745592	8.41493525755341	0.189643184971764	0.663213757582906	0.933112896909279
Fam207a	Fam207a	-0.0189537256225605	5.22180235081812	0.189455797253267	0.663369939138466	0.933112896909279
Sema3b	Sema3b	-0.0235007150687153	3.87150356763264	0.189398309325782	0.663417871867441	0.933112896909279
Rnf187	Rnf187	-0.0151681035863977	9.3248044165012	0.189394616070487	0.663420951554185	0.933112896909279
Ifnar1	Ifnar1	-0.0092438916507841	7.17312713655197	0.189381035734428	0.663432277606654	0.933112896909279
Ube2d1	Ube2d1	0.0123247808266999	6.80092937926667	0.189262300888089	0.663531308494589	0.933146984589525
Fhl2	Fhl2	0.0219729960873335	4.26268103807181	0.188885517518628	0.663845814867008	0.933191488281351
Ccdc28b	Ccdc28b	-0.0194313063364764	5.27867720788256	0.188832242996632	0.663890313716941	0.933191488281351
Aard	Aard	-0.0381141669891348	2.38364820362616	0.188791335122252	0.663924504949528	0.933191488281351
Exosc3	Exosc3	0.0239988769413881	3.18541135251112	0.188768572312711	0.663942506244958	0.933191488281351
Lrp4	Lrp4	0.0288350629150251	4.93215767652146	0.188738576938526	0.663968567305186	0.933191488281351
Slc25a45	Slc25a45	0.0886330943925125	-0.417972916278027	0.188689958959778	0.664011704250885	0.933191488281351
Adcy9	Adcy9	-0.0185747379741096	4.74208008291545	0.188406354241856	0.664246317894696	0.933237141952124
Hif1an	Hif1an	0.0181609413654259	4.25761375675148	0.188206760441687	0.664413324896371	0.933237141952124
Rwdd3	Rwdd3	0.038194795283398	2.18556418030549	0.188192001445067	0.664425678219356	0.933237141952124
Lgals5	Lgals5	0.115007515672777	-0.303815334553138	0.188155463834846	0.664456263276634	0.933237141952124
Bcl2l13	Bcl2l13	0.0185605748539631	3.99431742122053	0.188116890029598	0.66448855662971	0.933237141952124
Sulf1	Sulf1	0.0329997068030451	4.61651320970358	0.188108686571013	0.664495424937079	0.933237141952124
Nanp	Nanp	-0.0207435222052852	3.65509274689156	0.18795960207909	0.66462027624304	0.933237141952124
Dkk3	Dkk3	-0.0225976096678415	6.47053794985834	0.187932998378827	0.664642561785322	0.933237141952124
Arpc1a	Arpc1a	0.0146735731076828	7.63462426041958	0.18736235571473	0.665121031643689	0.933727520657741
Nup88	Nup88	-0.0104737084367547	6.29523079378051	0.187273583316987	0.665195544245121	0.933727520657741
Hfm1	Hfm1	-0.0440591063762573	3.30674182462553	0.187124822724115	0.665320453951628	0.933727520657741
Rnf114	Rnf114	0.0139892698010255	6.34024321806848	0.187117958846258	0.665326218761658	0.933727520657741
Ehbp1	Ehbp1	0.0117769449893846	7.37727765740001	0.187070616026489	0.665365984304558	0.933727520657741
Med18	Med18	0.0193302154328775	4.19262630902606	0.186654927087076	0.665715398450138	0.934076942411582
Plekha1	Plekha1	-0.0352606792915704	6.92595780880768	0.186596210197672	0.665764708970121	0.934076942411582
Gpr160	Gpr160	0.0764429916102181	-0.435971305268662	0.186398327702067	0.66593130937191	0.934205639299227
Prex1	Prex1	0.0156671995288857	7.36710383688635	0.186189429262555	0.666107228705276	0.934345558625704
Csnk1a1	Csnk1a1	-0.0105798332465123	7.56588978621085	0.186102079208645	0.666180819864174	0.934345558625704
Fam50a	Fam50a	0.0169238093234919	4.91792543256504	0.185797167764406	0.666437864019274	0.934379280063308
Akip1	Akip1	-0.0282960270819607	2.69061209861288	0.185794400976135	0.666440197920035	0.934379280063308
Slc4a8	Slc4a8	0.034806123850654	5.19438136065937	0.185785670073169	0.666447561053151	0.934379280063308
Tceal8	Tceal8	-0.0157361953337582	5.83408601545599	0.185685652065011	0.666531835808347	0.934379280063308
Garem2	Garem2	-0.0247854521034792	4.15381157000539	0.185580481789	0.666620695065172	0.934379280063308
Sgce	Sgce	0.0166937747673385	4.68314998582149	0.185540784671353	0.666654191802802	0.934379280063308
Ier2	Ier2	0.0311199884451106	3.88752628689462	0.185318093466243	0.666842284972709	0.934527929866842
Nrep	Nrep	-0.0154598054282112	8.70821232165124	0.185131360325585	0.667000018574183	0.934588356949055
Ccdc120	Ccdc120	0.0149761423076743	5.66860696431776	0.185098165363414	0.667028077081096	0.934588356949055
Prodh1	Prodh1	0.0327450949527036	3.36198859421722	0.184750547431747	0.667322085265175	0.934895313030644
Cyfip2	Cyfip2	-0.0142564657885312	9.31237485631647	0.184625914224276	0.667427601964986	0.934938243880996
Abi2	Abi2	0.0111108998811691	8.51985880195608	0.184370894705047	0.667643562455381	0.934993057906284
Scn3a	Scn3a	-0.0203734940332806	7.54260898445069	0.184324202985555	0.667683126492098	0.934993057906284
Mast2	Mast2	0.0238553958884273	8.44709655204044	0.184314198861998	0.667691604227378	0.934993057906284
P3h3	P3h3	0.0499368703174975	6.77939749662897	0.184162571618216	0.667820130052459	0.935068091422346
Nrbp1	Nrbp1	0.0105537248681159	6.69659905571124	0.183977182666694	0.667977358077498	0.935182291823714
Ftx	Ftx	-0.0385208465294081	3.36434491225202	0.183754884515764	0.668166014300719	0.935342461621236
Amt	Amt	-0.030723930461057	5.46013114897766	0.183606713819742	0.668291825765812	0.935413645372452
RGD156	RGD156394	0.0588395951003687	0.722271302772008	0.183450350887739	0.668424678979137	0.935428248238883
Sixl	Sixl	0.122267506222737	-0.765946476874727	0.183417951895308	0.668452212975688	0.935428248238883
Mstn	Mstn	-0.0529574579908232	0.523934224013174	0.183161407360373	0.668670326752618	0.935595231319033
Ogfod1	Ogfod1	0.0235981406346274	3.52345303121861	0.183001490439992	0.668806395570508	0.935595231319033
Rbbp4	Rbbp4	0.0148241896928422	6.16889911576298	0.182900995928021	0.668840859839942	0.935595231319033
Gpr75	Gpr75	0.0243959800264778	3.09359367702642	0.182933420517315	0.66886433314396	0.935595231319033
Sertad1	Sertad1	0.0475269414558078	1.47446181084283	0.182810441681042	0.668969023876367	0.935626794717569
Alp	Alp	-0.0209153378719256	5.46061660815776	0.182450036359455	0.669276117972106	0.935851370089438
Arpc5l	Arpc5l	-0.0152292407560252	5.6715295252021	0.182220626008895	0.669471774378807	0.936120063759316
Srprb	Srprb	-0.0153822455105816	5.34540554362886	0.181916567498799	0.669816736715091	0.936414692210978
Ndufa8	Ndufa8	-0.0215444320544916	6.03017826956083	0.181798019400091	0.669832582905184	0.936414692210978
Rnf19b	Rnf19b	-0.0142099997050706	6.33515890118533	0.181505287204176	0.670082713783749	0.936642872459685
Klhl12	Klhl12	-0.0151710387269205	4.81889226938453	0.181431459192403	0.670145942329696	0.936642872459685
Atp7b	Atp7b	0.0326738631881769	2.3810740013392	0.181340870022431	0.670223441448779	0.936646267904258
Zfp580	Zfp580	-0.0164436719351526	6.22310053116454	0.181252308268235	0.670299228220679	0.936647269628112
Ptger4	Ptger4	-0.0456163897286876	0.658745019337722	0.180968223929327	0.670542481255454	0.936842896297894
Kcnj14	Kcnj14	0.0292794061168067	3.74271174681272	0.180913463353917	0.670589397037468	0.936842896297894
Cpd	Cpd	0.0196517921953516	6.09206059948533	0.180275469568809	0.671136614033055	0.936897890152758
Ppil3	Ppil3	-0.0285955205606981	3.01428807217386	0.180258547841395	0.671152860042737	0.936897890152758
Tfpi	Tfpi	0.0322573648246405	4.06446469222219	0.180218049891396	0.671185818773477	0.936897890152758
Rcan3	Rcan3	-0.0380688804874684	4.03419090332803	0.180152801780036	0.671241857988139	0.936897890152758
Ap4b1	Ap4b1	0.0144380224783868	4.92793389803	0.180150852864127	0.671243631168658	0.936897890152758
Pitx2	Pitx2	0.179007547252503	1.31504339151535	0.180144879522928	0.671248762935469	0.936897890152758
Ltb	Ltb	0.05407992286248	0.689908885795124	0.180122465878284	0.671268016430014	0.936897890152758
Drd2	Drd2	-0.0674877591712855	4.17368755134279	0.179978073617249	0.671392084402557	0.936897890152758
Prmt3	Prmt3	0.0152331860734229	5.56333532676209	0.17984321260667	0.671508015769436	0.936897890152758
Msantd1	Msantd1	0.0366106548285488	2.40202953689639	0.179773544203101	0.671567925321382	0.936897890152758
Zbtb7b	Zbtb7b	0.0495935250665392	1.17873054979698	0.179769682761763	0.671571238808738	0.936897890152758
RGD156	RGD156626	-0.0209452922941176	3.96311933070243	0.179452804657283	0.671843064300877	0.936897890152758
Rpn1	Rpn1	-0.0138005598929892	8.40832149046992	0.179376000198353	0.67191004536676	0.936897890152758
Erbb3	Erbb3	-0.0342364645718955	2.51745116027158	0.179316684692907	0.671961129706558	0.936897890152758
Atf6b	Atf6b	0.0112291290274362	6.99287086369335	0.179177481937536	0.672081054427739	0.936897890152758
Hrh2	Hrh2	-0.0479442105374086	1.22437198416061	0.179063267944772	0.67217954383572	0.936897890152758
Bbs2	Bbs2	0.0195264251309864	4.80186135064233	0.178828207089055	0.672382200703828	0.936897890152758
Cpsf7	Cpsf7	0.0203767288704338	6.58642920064956	0.178767707111945	0.672434399263281	0.936897890152758
Mterf2	Mterf2	0.0298088424014203	2.23888744659604	0.178750616045342	0.672449147089877	0.936897890152758
Map1lc3	Map1lc3a	-0.0225578016809464	8.00312612237498	0.178663165571704	0.672524620705529	0.936897890152758
Slc35b2	Slc35b2	0.0155424194383686	5.44167504254763	0.178475806413459	0.672686393635216	0.936897890152758
Egln3	Egln3	0.0282202293604166	4.57497203258618	0.178465999130495	0.672694864360504	0.936897890152758
Pgap1	Pgap1	0.0315473375483848	6.20462642969154	0.178402060528949	0.672750095997504	0.936897890152758
Pwwp2b	Pwwp2b	-0.0299016921271176	3.12294538049335	0.178286925094662	0.67284966849367	0.936897890152758
Lypd2	Lypd2	-0.0204094334639757	4.40654107357399	0.178185681537457	0.672937095713673	0.936897890152758
Ctsf	Ctsf	-0.01332491067629	6.00904211119153	0.178162533856458	0.67295710839328	0.936897890152758
Ppp6r2	Ppp6r2	0.0113083432422641	7.45548019479799	0.178112449628823	0.673000415581582	0.936897890152758
Ppp1r1b	Ppp1r1b	-0.0637068382545777	6.2865737196411	0.178041886622548	0.673061354446912	0.936897890152758
Nup37	Nup37	-0.0242268211839457	3.03047409116736	0.178038572409775	0.673064307219829	0.936897890152758
Setd3	Setd3	-0.0361004651362966	2.46717709073551	0.177842147562913	0.673234260682365	0.936897890152758
Sbds	Sbds	0.016748756973552	4.79713165041024	0.177798675804283	0.673271888866362	0.936897890152758
Gbx2	Gbx2	-0.114745838068172	3.37266462883787	0.177622535764809	0.673423541083154	0.936897890152758
Dnttip1	Dnttip1	-0.0156812310680606	5.31440762259404	0.177543968980362	0.673492465413915	0.936897890152758
Cobl	Cobl	-0.0199039857680596	6.08027436765474	0.177515606836291	0.673517039822509	0.936897890152758
Eif2b2	Eif2b2	0.0173945826061623	5.41043311784652	0.177505867518143	0.67352547843607	0.936897890152758
Wbp2	Wbp2	-0.0094507534919625	6.83605643295217	0.177440772078143	0.673581888456642	0.936897890152758
Vax1	Vax1	-0.0646362354453023	1.69118163983767	0.177369362750227	0.673643783943165	0.936897890152758
Dok6	Dok6	-0.0224008036890107	5.12988745417274	0.177187071272456	0.673801855337567	0.936897890152758
Itfg2	Itfg2	-0.0279031271424l8	3.72008998477004	0.177094842973705	0.673881865380419	0.936897890152758
Ermp1	Ermp1	0.0163858626341117	5.72421749721229	0.177082002844784	0.673893007380165	0.936897890152758
Pycr2	Pycr2	-0.0200434935108891	5.1702051955229	0.176972777168203	0.673987799791866	0.936897890152758
Dcaf5	Dcaf5	0.0092443850040554	6.98743653223662	0.176923644179212	0.674030451492147	0.936897890152758
Fpgs	Fpgs	0.0217872015506673	4.83093513311592	0.176669910095157	0.674250825719621	0.936897890152758
Syt2	Syt2	-0.0502793102171546	3.04015393879498	0.176664882896734	0.674255192975644	0.936897890152758
Zfp3	Zfp3	0.0224211242409536	3.37880641830538	0.176604240403663	0.674307891775577	0.936897890152758
Glra1	Glra1	-0.121471830010611	-0.387373675478266	0.176573294028923	0.674334768077648	0.936897890152758
Ndst4	Ndst4	-0.0773290104307065	2.55539113034929	0.176502322208535	0.674396482007857	0.936897890152758
Cnot8	Cnot8	0.01282769034988	6.23610235967032	0.176128052965577	0.67472206513799	0.936897890152758
Dhrs7b	Dhrs7b	-0.0197424915211517	4.34450397217741	0.176126532060835	0.674723389902394	0.936897890152758
Ccdc62	Ccdc62	-0.0476206155l8946	3.14169723134265	0.175940815906188	0.674885099822454	0.936897890152758
Lrrc4c	Lrrc4c	-0.01357586171898	6.27671138435255	0.175931635152976	0.67489309644736	0.936897890152758
Abcb8	Abcb8	0.0125971465063	5.62885587177617	0.175685625593061	0.675107467244945	0.936897890152758
Iqsec2	Iqsec2	-0.0159239653169296	6.91029341751442	0.17560810655583	0.675175053283251	0.936897890152758
Kntc1	Kntc1	0.0246054529721011	4.56668472679072	0.17560529942088	0.675177501053679	0.936897890152758
Slc25a28	Slc25a28	-0.0159746237167317	4.56066726545449	0.175587615324972	0.675192921777757	0.936897890152758
Rpl36	Rpl36	-0.0269843903836432	7.85388800120596	0.175517064226767	0.675254452190653	0.936897890152758
Asb6	Asb6	0.0164079023037338	4.9576362134664	0.175471685007161	0.675294028168057	0.936897890152758
Usp34	Usp34	0.0244575101011385	8.08129418925356	0.175448244068178	0.675314480390332	0.936897890152758
RGD131	RGD131035	-0.0136188371369077	6.6731007734629	0.175415579117036	0.675342988883095	0.936897890152758
Fam169a	Fam169a	0.0133027897422343	8.12298053366238	0.175375532546262	0.6753779314326	0.936897890152758
Pth2r	Pth2r	-0.0611145629415851	1.36200158725146	0.175319905374295	0.675426479362372	0.936897890152758
Tmem25	Tmem25	-0.0322183311723603	2.93253592201292	0.175123206558844	0.675598218463672	0.936897890152758
Fkbp11	Fkbp11	-0.0370885278622496	2.06621081582732	0.175109261179557	0.675610398572966	0.936897890152758
Zfp612	Zfp612	0.0277796880403524	5.28870002447186	0.174965125353694	0.675736322425785	0.936897890152758
Hat1	Hat1	-0.0201150592342305	4.6561877139747	0.174906482116043	0.675787573364865	0.936897890152758
Ppfia1	Ppfia1	0.0184175489978332	5.98477875924594	0.174525660671819	0.676120636061096	0.936897890152758
Dnajb1	Dnajb1	0.013482481452286	6.36617041089702	0.174421686863526	0.676211635939898	0.936897890152758
Gsk3a	Gsk3a	-0.0119552036653223	8.39270534230499	0.174382547180256	0.676245896476957	0.936897890152758
Asph	Asph	-0.0181363159925056	4.07995043984213	0.174336924410618	0.676285861136186	0.936897890152758
Hoxa5	Hoxa5	-0.607929497575576	1.39856853912732	0.17429163107752	0.676325545611525	0.936897890152758
Slc37a1	Slc37a1	0.0267697728266906	3.19940375483121	0.174168458517215	0.676433429683324	0.936897890152758
Sh2d4a	Sh2d4a	-0.0669754022303546	0.239640562141761	0.174119612914126	0.676476233918148	0.936897890152758
Ighmbp2	Ighmbp2	0.0134885427139852	5.26208526580965	0.174103380330635	0.676490457700367	0.936897890152758
Bcl7c	Bcl7c	-0.0209604877928265	4.59486197248735	0.174033489421348	0.676551713527322	0.936897890152758
Btg2	Btg2	0.0242312229947607	3.81312890052534	0.173985410083183	0.676593866925035	0.936897890152758
Pebp1	Pebp1	-0.0189484547418516	8.84304889437732	0.173898511710732	0.676670065792123	0.936897890152758
Utp11	Utp11	0.0153534186578635	5.59754613869799	0.173872927840286	0.67669250338648	0.936897890152758
Fam234b	Fam234b	-0.0067350107469255	7.35154520322239	0.173867384664007	0.676697365706438	0.936897890152758
Clint1	Clint1	0.0156351705612252	5.16809739485227	0.173844657544294	0.67671730029343	0.936897890152758
E2f1	E2f1	0.0216746820941082	4.15579635050977	0.173688920369102	0.676853842857966	0.936897890152758
Sh3bgrl	Sh3bgrl	0.0145666024370791	8.26295775485881	0.173649249753311	0.67688876103171	0.936897890152758
Larp6	Larp6	-0.0152188735420865	4.97676360529801	0.173622480916592	0.676912258187583	0.936897890152758
Foxp3	Foxp3	-0.0840827913893595	-0.447304699526669	0.173611188308683	0.676922171253215	0.936897890152758
Gpx4	Gpx4	0.0239126967670204	7.57200697312841	0.17358277l5532	0.676947118l9816	0.936897890152758
Kcnj6	Kcnj6	-0.0283409061815547	6.27640674007865	0.173460162158364	0.677054783933128	0.936897890152758
Aldh1a1	Aldh1a1	-0.0932206538301543	2.24404821613614	0.173366598185103	0.6771369743684	0.936897890152758
Slc52a3	Slc52a3	0.0554963668507251	0.833618824172549	0.173338580606774	0.677161591199613	0.936897890152758
Sugp2	Sugp2	-0.0069591845039015	7.52607184167607	0.17314433817349	0.677332320136721	0.937020188286491
Ccdc12	Ccdc12	-0.023157470926726	3.73520501814258	0.172798441961405	0.677636625628338	0.937282836376226
Hrob	Hrob	-0.0552674268075341	0.584528542498289	0.172766004200611	0.677665181289582	0.937282836376226
Kcna4	Kcna4	-0.0269394379911734	3.72563668850489	0.172669145823777	0.677750466435776	0.937296893118951
RGD156	RGD156026	-0.0298041137188745	6.76281945923529	0.172553332032152	0.677852479111427	0.937324077565474
Eif2ak4	Eif2ak4	0.0267199821519039	5.57232936536272	0.17237961080799	0.67800557393445	0.937461882311629
Polr1f	Polr1f	-0.0204907363955037	4.69307491029303	0.172052709430218	0.678293806297589	0.937736628130?1
Ybey	Ybey	0.0286210440762956	2.71458749645555	0.171812535016611	0.678505848837887	0.937763448087l5
Vom2r44	Vom2r44	-0.141571719807167	-0.174144995953968	0.171795562154205	0.678520928336197	0.93776344808715
Scn8a	Scn8a	-0.0535114560866636	5.75286688899657	0.171677074898426	0.678625618980113	0.93776344808715
Rhbdf1	Rhbdf1	-0.0206318247614011	4.5594018899679	0.171623155764728	0.678672268395267	0.93776344808715
Mirlet7c2	Mirlet7c2	-0.0934186588348626	-0.261317982387155	0.171605240009001	0.678689502847396	0.93776344808715
Spc25	Spc25	-0.0331068980879347	2.52995717750901	0.171423450918518	0.678849827985544	0.937881663578301
Epb41l5	Epb41l5	-0.0287086655514607	3.47826180380254	0.171093452530727	0.679141864648417	0.938181221842149
Tgm4	Tgm4	0.0946142660576769	1.318235208176	0.170965414536774	0.679255234695932	0.938181741348995
Nup85	Nup85	0.0134565996791106	5.45670490665483	0.170903834678583	0.67930978733296	0.938181741348995
March9	March9	-0.0151288207046196	6.89950620426068	0.170757549195713	0.679439425518297	0.938181741348995
Mrpl54	Mrpl54	-0.0274271051521567	5.06603386226476	0.170630157040025	0.679552237340772	0.938181741348995
Mrpl22	Mrpl22	-0.0295136115034175	3.42794377022022	0.170559171552248	0.679586468477436	0.938181741348995
Necap2	Necap2	-0.0167763771562143	4.80101082980034	0.170491136552897	0.679675687494657	0.938181741348995
T2	T2	-0.0694625890371058	-0.303396169761171	0.170471617923326	0.679693005638967	0.938181741348995
Ddx41	Ddx41	0.0132621905723589	5.61727140049666	0.170414417763602	0.679743763889112	0.938181741348995
Arl14ep	Arl14ep	0.0198384688462653	4.4607519077197	0.170155662981912	0.679973502119945	0.938229705357472
Rras	Rras	-0.0325676431014386	2.4600977774541	0.170139627617104	0.679987746017681	0.938229705357472
Rpap2	Rpap2	-0.0140684404912411	4.62556306981523	0.170096611185115	0.680025859852641	0.938229705357472
Ntpcr	Ntpcr	0.0217149432946543	4.19370856148032	0.170036577288329	0.680079302336537	0.938229705357472
Pex7	Pex7	-0.0279088914816546	2.76117115535243	0.169889285553367	0.680210221845267	0.938285617772581
Klhl17	Klhl17	0.0327830371088181	5.04246409762125	0.169821794508977	0.680270223098562	0.938285617772581
Yae1	Yae1	-0.015954529699028	5.12529790623681	0.169681522357614	0.680395004029898	0.938322293704129
Rbms2	Rbms2	0.0261130612040501	3.48470754371358	0.169622925866092	0.680447231981643	0.938322293704129
Ctnnb1	Ctnnb1	-0.0121381557914856	9.30598096150712	0.169497922000352	0.680558496257523	0.938372012965865
Cct2	Cct2	-0.0123403623640255	8.23102296613827	0.169398958273405	0.680646525738639	0.938389690789061
Kctd9	Kctd9	0.0298998524377556	3.20557419782461	0.169095067240107	0.680917305357677	0.938659288360153
B4galt7	B4galt7	0.015208392090087	5.17157014285713	0.168908507881852	0.681083678493576	0.938784915661108
Mfsd12	Mfsd12	0.0214193098509151	5.19889705167563	0.168660369033889	0.681305193489275	0.938888512666042
Rnasel	Rnasel	0.0210337108415779	3.93822237942124	0.168558442987865	0.681396155485117	0.939008154626442
RGD156	RGD156085	-0.0236302901827806	3.82208765819127	0.168327522101265	0.681602489077161	0.939147555004591
Klf13	Klf13	-0.0259008923847864	3.31951162073846	0.168222401480499	0.681696471750555	0.939147555004591
Gars	Gars	-0.0131817776339886	7.86795087779771	0.168192598754644	0.681723123000447	0.939147555004591
Tacc3	Tacc3	0.025820726890612	3.65398166342545	0.167818045564861	0.682058303751014	0.939265697364354
Ppp6c	Ppp6c	0.0118730174223387	5.84514165747333	0.167774778884073	0.682097051441306	0.939265697364354
Psph	Psph	-0.0320273200084025	3.94347950055118	0.167766559582162	0.682104412767164	0.939265697364354
Vegfa	Vegfa	0.0287406672545572	6.78620947186851	0.167760320067472	0.682110001107511	0.939265697364354
Cdkn1b	Cdkn1b	0.0176242348521214	6.40921498586216	0.167500055744725	0.682343321165715	0.939322572967
Alas1	Alas1	-0.0126164358584027	5.02757643918661	0.167435834729993	0.682400789805175	0.939322572967
Smf12	Smf12	0.0246293827550822	3.72015309838873	0.167361586211605	0.682467373874523	0.939322572967
Mlx	Mlx	0.0142416001881774	5.2591341880909	0.167302880674576	0.682520031781898	0.939322572967
Pepd	Pepd	0.0185895575681179	5.82931564374435	0.167261984488132	0.682556721368106	0.939322572967
Trank1	Trank1	-0.0440154111731237	7.39491938044282	0.167168852953044	0.682640291989524	0.939322572967
Lmcd1	Lmcd1	0.0440246220306747	1.4915210430329	0.167126512226343	0.68267828539671	0.939322572967
Agpat4	Agpat4	0.0105461183196853	5.77668448077728	0.167001145817125	0.682790853316871	0.939338052556057
Phf5a	Phf5a	0.0192677755515017	5.06904059539546	0.166937931385263	0.682847627475769	0.939338052556057
Lamtor4	Lamtor4	-0.0252376029679235	4.71543206849929	0.166862486671718	0.682915402319147	0.939338052556057
Rell1	Rell1	0.0454265599863383	0.894036722164431	0.166603579216769	0.68314812147241	0.939654580280794
Rpe	Rpe	-0.0167739869558944	4.61699035070457	0.166405348657866	0.68332554964929	0.939564396093l9
Rbmxl1	Rbmxl1	0.0124409675226262	6.90685581163001	0.166297213867004	0.683423777973454	0.93956439609319
Serpinb6	Serpinb6a	0.0260477904871283	4.01554529457109	0.166222379979217	0.683491155008886	0.93956439609319
Rhoa	Rhoa	0.0125747351737447	7.23022603908487	0.166217133455142	0.683495879404452	0.93956439609319
Gpr137c	Gpr137c	-0.0225992856787805	3.93363115408216	0.166154367981022	0.683552405268916	0.93956439609319
Aco2	Aco2	-0.0117824388749842	8.75473060749211	0.166093666628491	0.683607084053376	0.93956439609319
Scrn1	Scrn1	0.0211452958683706	3.37586565657253	0.165888869198613	0.683791654729415	0.939595141497649
Mob1b	Mob1b	0.0219069814201117	4.82628304654845	0.165808588160985	0.683864034036292	0.939595141497649
Ccdc32	Ccdc32	-0.0172475155568748	5.87125425727065	0.165557636364727	0.684090257291831	0.939595141497649
Yju2	Yju2	-0.0152849488132881	5.00370707500359	0.165532472070097	0.684113051509489	0.939595141497649
Ikbkb	Ikbkb	0.0234133645087352	4.69881127314085	0.165447405611303	0.684189969044722	0.939595141497649
Syt4	Syt4	0.0171750421240198	9.22469169385837	0.165393429669649	0.684238690125098	0.939595141497649
Dync1i2	Dync1i2	0.0101009774674371	8.57731254573276	0.165162232403773	0.684447498823475	0.939595141497649
Dis3l2	Dis3l2	0.0133051601120649	5.67757412420069	0.165150371107828	0.684458290549404	0.939595141497649
LOC100	LOC10012	0.0133459042575766	4.6711459759288	0.165112345661889	0.684492653102137	0.939595141497649
Ube3b	Ube3b	0.0101191730542124	6.84780958230825	0.165025020816449	0.684571616591446	0.939595141497649
Acot3	Acot3	-0.0245651854145353	3.98688893789678	0.164975327682114	0.684616556785143	0.939595141497649
Fzd5	Fzd5	0.0714477932038669	1.17468833208964	0.164795044614721	0.684779652749612	0.939595141497649
Pnma1	Pnma1	0.0245445925575287	2.62632682985513	0.164683182760374	0.684880917917351	0.939595141497649
Hebp1	Hebp1	0.0287383375864238	3.42568312958364	0.164592822326871	0.684962740398068	0.939595141497649
Maged1	Maged1	-0.010577161872672	9.45132821977355	0.164520874414094	0.685027909728281	0.939595141497649
Dhcr24	Dhcr24	-0.0136638324746561	7.17032121050921	0.164478267602636	0.685066391837056	0.939595141497649
Pot1	Pot1	0.0331390918888803	3.91854003194133	0.164290947577331	0.685145625907463	0.939595141497649
Actn3	Actn3	-0.0692359346160786	-0.104156966336716	0.164380282688082	0.685155301727586	0.939595141497649

Riox2	Riox2	0.0195745662675827	3.85094724233801	0.164158879036378	0.685356047130325	0.939595141497849
Usf1	Usf1	0.0125201136157182	6.91433212949488	0.164144284843822	0.685369385135261	0.939595141497849
Nipsnap	Nipsnap3b	0.0200661018940883	5.82653392120316	0.164122696671396	0.685387961285335	0.939595141497849
Reck	Reck	-0.0417129774156701	5.06605169525126	0.164103919194797	0.685405803313072	0.939595141497849
Mfsd10	Mfsd10	-0.0330757300662367	5.04424134249569	0.164081443739036	0.685426294461884	0.939595141497849
Msrb2	Msrb2	-0.0356297460439906	2.28464897459716	0.164069862915802	0.685436801948659	0.939595141497849
Pus3	Pus3	0.0220570147675583	3.90106585407742	0.163780526644061	0.685699462127833	0.939811947827192
Suco	Suco	0.0161679002076273	5.64344262715489	0.163729722888258	0.685745809763466	0.939811947827192
Gsta4	Gsta4	0.0326595106100077	3.76116881514221	0.163240091952233	0.686190794148678	0.940058150449038
Zbtb11o	Zbtb11os1	-0.0330529400658271	1.9390716186781	0.163048686956913	0.686365035379024	0.940058150449038
Abhd17a	Abhd17a	0.018403148380695	6.99065279675336	0.163017292501863	0.686393825968193	0.940058150449038
Coil	Coil	-0.013583957348261	4.91262585862849	0.162997289730292	0.686411843944912	0.940058150449038
Grpel1	Grpel1	-0.0209367844379751	5.45846700048782	0.162937258680401	0.686466526388297	0.940058150449038
Gla	Gla	-0.0151987451012876	4.75138155594975	0.162897629485357	0.686502621147274	0.940058150449038
Preb	Preb	0.0136444380915295	5.84291533621544	0.162881924255984	0.686516941039725	0.940058150449038
Txnrd2	Txnrd2	0.0209391627500729	3.87540475800941	0.162820880591121	0.686572559764181	0.940058150449038
Rps6kb2	Rps6kb2	-0.0354613246138367	5.07936590008223	0.162787114802342	0.686603344156666	0.940058150449038
Qars1	Qars1	-0.0089180275919386	6.45332044144751	0.162661512461284	0.686717854956745	0.940080859921869
Cadm3	Cadm3	0.0132958185967567	8.52397645750599	0.162512165575585	0.686854080413284	0.940080859921869
Tbxas1	Tbxas1	-0.0759748597262577	-0.548593858688588	0.162429992235303	0.68692908514328	0.940080859921869
Osbpl9	Osbpl9	0.0111736614017589	5.90309822373194	0.162366982942174	0.686986741568026	0.940080859921869
Sbsn	Sbsn	-0.0612292408022626	0.944803018446041	0.162332706868348	0.687017870218623	0.940080859921869
Hoxd3	Hoxd3	-0.473581479456048	-0.165008821732559	0.162155656779589	0.687179561233156	0.940080859921869
Kyat1	Kyat1	0.0179305211471687	4.17493305096709	0.162052473279554	0.687274755850086	0.940080859921869
Tmem14	Tmem140	0.0598031867027835	0.165664057432349	0.161694618297616	0.687601091371588	0.940080859921869
Arhgef1	Arhgef11	0.0214988291227749	6.79490892207965	0.161632676458848	0.687656863066142	0.940080859921869
Crb2	Crb2	0.0277011404059514	2.94386185812535	0.161387970777326	0.687881847888318	0.940080859921869
Dmrt3	Dmrt3	0.113166508765751	-0.326059665213469	0.161362200539532	0.687905456187522	0.940080859921869
Mrpl41	Mrpl41	-0.0202240889466016	4.9988773608975	0.16127240617493	0.68798773519402	0.940080859921869
Cntln	Cntln	0.0315018331927638	2.74384820710618	0.161146466443356	0.688103179209499	0.940080859921869
Fam181b	Fam181b	-0.0218370247404304	4.56688800399686	0.161119531007046	0.688127876657194	0.940080859921869
Plppr5	Plppr5	0.0123819707787137	6.24140207528288	0.161096472598839	0.688149021132797	0.940080859921869
Syap1	Syap1	0.018628349526464	4.54050061570349	0.161023255204164	0.688216172824695	0.940080859921869
Wif1	Wif1	-0.0231603698811116	4.02491628376178	0.160965081470408	0.688269529788442	0.940080859921869
Marchf6	Marchf6	-0.0127328407928135	8.82782002099088	0.160855135059152	0.688370432148595	0.940080859921869
Zbtb20	Zbtb20	0.0544988604121966	3.7786449331815	0.160770358670928	0.688448254557542	0.940080859921869
Iqub	Iqub	0.0384624808225331	2.42398087979041	0.160770145344244	0.688448450421172	0.940080859921869
Lig1	Lig1	-0.0151277953912086	5.93663997792834	0.160735717552789	0.688480006102053	0.940080859921869
Htra2	Htra2	-0.0145350279918964	5.79380705041334	0.160529414492004	0.688669564873332	0.940080859921869
Pcdhga7	Pcdhga7	-0.0217166679904696	4.61880484959871	0.160523455867548	0.688675040387756	0.940080859921869
Camta2	Camta2	-0.0186050213406703	6.98478549153305	0.160360564448624	0.688824770689811	0.940080859921869
Slc46a3	Slc46a3	-0.042436679071412	1.06578013592983	0.160343194842369	0.688840742091917	0.940080859921869
Hint2	Hint2	-0.0256421621708509	3.7752239328226	0.160313371272565	0.688868167290271	0.940080859921869
Psma2	Psma2	-0.015156192878231	6.70511879656727	0.160135429411621	0.689031861772359	0.940080859921869
Ctif	Ctif	0.0173203042987193	5.92993363750624	0.160080645547501	0.689082279722082	0.940080859921869
Hcfc1r1	Hcfc1r1	-0.0187216843510349	6.80305223412019	0.160023234629023	0.689135126129921	0.940080859921869
Wars2	Wars2	0.0254569427680804	2.74371338187606	0.159907912674786	0.689241312543633	0.940080859921869
Ocel1	Ocel1	-0.0207012024600194	5.12916921518793	0.159864632280222	0.689281176171955	0.940080859921869
Lrpprc	Lrpprc	-0.0098745544209346	7.47347825345867	0.159821999660146	0.689320450205031	0.940080859921869
Chrd	Chrd	-0.0383448181278132	4.19514520461862	0.159797176446205	0.689343320114706	0.940080859921869
Ikbip	Ikbip	0.0264826749673313	3.50910662104466	0.159696135278963	0.689436421758383	0.940080859921869
Cdc16	Cdc16	0.0095173545829386	6.65513367686712	0.159648963407577	0.68947991336571	0.940080859921869
Hmgcs2	Hmgcs2	0.0312608769957208	4.05284324209845	0.159600309663452	0.6895222516439	0.940080859921869
Ints14	Ints14	0.0110660248591425	5.58567378846582	0.1595110755568	0.689607056953308	0.940080859921869
Prkcd	Prkcd	-0.0306648341809416	4.48632931074102	0.159467219351384	0.689647509225677	0.940080859921869
Dync2i2	Dync2i2	-0.0169333381869457	5.46652025131084	0.159412924881764	0.689697598524219	0.940080859921869
Jmjd4	Jmjd4	0.023218739425437	3.25863720976392	0.159282519716588	0.689817020984111	0.940080859921869
Azi2	Azi2	0.0112967569901257	5.90404335367361	0.159248107070759	0.689849711604419	0.940080859921869
Tiprl	Tiprl	0.012667889527749	6.05804814107227	0.158810436693948	0.690254088217471	0.940080859921869
Ptger1	Ptger1	0.0238230042682226	4.91024434097432	0.158666480548391	0.690387234667079	0.940080859921869
Gpbp1	Gpbp1	0.0101572997946138	7.02667992734296	0.158631364298033	0.690419724647655	0.940080859921869
Tubgcp	Tubgcp3	0.015585041056622	5.72318856664059	0.158582885826192	0.690464584366919	0.940080859921869
Zfp641	Zfp641	-0.0220505180136447	4.56358346232781	0.158528114298406	0.690515276944867	0.940080859921869
Gabrb3	Gabrb3	0.0146868977772325	7.34465930200261	0.158505566575158	0.690536148440124	0.940080859921869
Uqcc3	Uqcc3	-0.0247272208382146	3.58855471994228	0.158494520970197	0.690546383710682	0.940080859921869
Ugt1a6	Ugt1a6	0.0789022317145564	0.0970200058022344	0.158441223842772	0.690595717305254	0.940080859921869
Tcte1	Tcte1	-0.016088877317008	4.74591123235831	0.158381734993313	0.69065080488559	0.940080859921869
Tom1l1	Tom1l1	-0.0184223894501011	3.21141006639883	0.15825386860469	0.690769159028906	0.940080859921869
Skdb	Skdb	-0.0182058304810496	4.70292358042	0.158244359334965	0.690779062643819	0.940080859921869
Rbsn	Rbsn	0.013091668744643	6.09072287950834	0.158236885229798	0.690784978839975	0.940080859921869
Slc11a2	Slc11a2	0.0167687511841694	6.04786196764681	0.158232686862362	0.690788878371888	0.940080859921869
Thap1	Thap1	0.0203810368945294	3.19120693403903	0.158147929096828	0.690867428888274	0.940080859921869
Ciao2a	Ciao2a	0.0237194551584143	3.78848750038592	0.15803856798532	0.69096881693923	0.940080859921869
Plk5	Plk5	-0.0315320605721792	3.6396203179173	0.157987325724065	0.691016337332574	0.940080859921869
Pkn1	Pkn1	0.0148360093713542	7.04798053156737	0.157945655097208	0.691054887878118	0.940080859921869
Dctd	Dctd	0.0284093439412259	3.22236704403286	0.157934546998412	0.691065291913391	0.940080859921869
Cyp2s1	Cyp2s1	-0.0259348838269448	2.85787962559465	0.157826987817671	0.691165087094582	0.940114116011711
Cstf3	Cstf3	-0.0151225198024955	4.9995575294203	0.157611002806746	0.691365600522344	0.940284346818976
Trim35	Trim35	0.0299592126038225	3.02820789486261	0.157309056558638	0.691646184316396	0.940563426889991
Dnajc7	Dnajc7	-0.0096180073657609	7.42196688409882	0.15708309867945	0.691856359792416	0.940610064396695
Rps11	Rps11	-0.0162501273126862	8.37238971817331	0.156992209581659	0.691940969918146	0.940610064396695
Smarcb1	Smarcb1	0.0114813850050851	6.94296938733013	0.15693740341564	0.691991971494275	0.940610064396695
Cdc37	Cdc37	-0.011242860674798	7.41891707574882	0.156918146084429	0.692009901475075	0.940610064396695
Gtf2a1	Gtf2a1	0.018323403279498	4.62592615575466	0.156715522721683	0.692198825814497	0.940610064396695
Nrtn	Nrtn	-0.0526552136205439	1.17517321923038	0.156712547748398	0.692201407916258	0.940610064396695
Cnih3	Cnih3	-0.0280867625244716	2.87754547582613	0.156614663035604	0.692292634584601	0.940610064396695
Slc38a10	Slc38a10	0.0138509550126303	6.26777113895452	0.156593552453778	0.692312313562794	0.940610064396695
Ankrd34	Ankrd34b	-0.0379846258182659	3.33496390147083	0.15654110340283	0.692361212449161	0.940610064396695
Slitrk1	Slitrk1	0.0118928659870867	6.56010848917903	0.156453725758418	0.69244269673014	0.940610064396695
Tpm2	Tpm2	-0.0419600461743072	2.85832498529081	0.156237051098978	0.692644870918279	0.940610064396695
Lto1	Lto1	0.0190357880543683	4.93453740477177	0.156121676017065	0.692752590921555	0.940610064396695
Hnrnpf	Hnrnpf	0.0127761632546406	7.82635278070456	0.156095431483969	0.692777100566796	0.940610064396695
Mocs1	Mocs1	-0.0250698047148961	2.89562255829603	0.155814230285564	0.693039862655124	0.940610064396695
Depp1	Depp1	0.0709724992199864	-0.19405257158833	0.155693076759036	0.693152156296978	0.940610064396695
Pcdha6	Pcdha6	-0.0278285388873106	4.03331252960128	0.155451104958388	0.693379582646082	0.940610064396695
Pabpn1	Pabpn1	-0.0322391003652899	7.85496083372561	0.155376173178723	0.693449741729876	0.940610064396695
P2ry12	P2ry12	0.0648413459543371	1.96498095739655	0.155322182169456	0.693500305958801	0.940610064396695
Alg5	Alg5	-0.0160588821560854	4.16898845479706	0.155168988044547	0.693643821295773	0.940610064396695
Tmpo	Tmpo	0.0164410985022057	6.7187923918071	0.155125281457629	0.693684795568052	0.940610064396695
Gpc5	Gpc5	0.0283671974758893	4.87321227477602	0.155115746579369	0.693693732873666	0.940610064396695
Dus4l	Dus4l	0.03198418914111	2.00880998081234	0.155059675427298	0.693747223880425	0.940610064396695
Atp5pb	Atp5pb	-0.0157736735686897	7.70678103368403	0.155055070008597	0.693750614142177	0.940610064396695
Dynll1	Dynll1	-0.0159994760651535	8.35356412818455	0.155014480047655	0.693788603008968	0.940610064396695
Tmem63	Tmem63c	0.0252618575326471	5.59841514548354	0.154891948049723	0.693903596623942	0.940610064396695
Brd9	Brd9	-0.0121197377379483	6.22581105107725	0.154866112026561	0.693927825242672	0.940610064396695
Arhgap1	Arhgap18	-0.0281121758380254	3.86764213189764	0.154703376263996	0.694080562983926	0.940610064396695
Ikbke	Ikbke	0.0521587489098806	1.12584970725346	0.154698439475213	0.694096463512294	0.940610064396695
Psmd14	Psmd14	-0.0145420749970047	6.51721825012699	0.154645944991677	0.694134578355245	0.940610064396695
Gstm4	Gstm4	-0.0225550382914162	4.32112581546974	0.154598638626261	0.694192056962165	0.940610064396695
Qdpr	Qdpr	-0.0171429377045445	6.48692371189276	0.154555651230908	0.694219283471983	0.940610064396695
Krit1	Krit1	-0.0282152855670471	5.1615319282076	0.15449441634857	0.694276808236244	0.940610064396695
Klhl4	Klhl4	-0.0270533920380684	3.99553686065181	0.154443954798918	0.69432422230031	0.940610064396695
Hsdl1	Hsdl1	0.0113768644856855	6.65323732115845	0.154406530332947	0.694361272054067	0.940610064396695
Nudt12	Nudt12	-0.0282741242019994	2.30582028101562	0.154357012886017	0.694405894571977	0.940610064396695
Uqcr11	Uqcr11	-0.0303753272952197	5.99869914904471	0.154274108951697	0.694482876521343	0.940610064396695
Polr2i	Polr2i	0.0219614918885633	4.55375338738939	0.154220731881949	0.69453407161472	0.940610064396695
Nubp2	Nubp2	-0.0186586019430507	4.62130176276037	0.154208894133468	0.694545205040214	0.940610064396695
Ttll3	Ttll3	-0.0366571709044732	2.86559693051497	0.154095922313875	0.694651479137728	0.940651888995163
Rragc	Rragc	0.0115034177811354	6.12894404648706	0.153847377427269	0.694885450004326	0.940866604417144
Ankrd16	Ankrd16	-0.0275871886205488	2.87452916955722	0.153714094401437	0.695011006982913	0.940934498074065
Selenoo	Selenoo	-0.0235745117856672	4.22689937111524	0.153238562150584	0.69532264951057	0.941254279911401
Fndc3c1	Fndc3c1	0.107751418821825	-0.413894903670465	0.153180460005359	0.695514338152118	0.941363286435481
Plp1	Plp1	-0.0421182480335985	4.63671303204548	0.153075825452348	0.695613149388851	0.941363286435481
Ndufb11	Ndufb11	-0.0203200198135562	6.15780653294773	0.152986996376399	0.695697065350422	0.941363286435481
Med31	Med31	-0.0200251930188125	3.77096700237789	0.152919685492974	0.695760662544109	0.941363286435481
Cars2	Cars2	-0.018036939251909	4.87686152995715	0.152872298411857	0.695805460959638	0.941363286435481
Susd2	Susd2	0.0562011328155581	1.0598216994048	0.15276093110532	0.695910753789101	0.941363286435481
Pfkfb2	Pfkfb2	0.0132005524277041	5.99006685632898	0.15269193520605	0.695976008537603	0.941363286435481
Oas1f	Oas1f	0.0723198493837891	-0.0683487158194722	0.152587079058104	0.696075211640207	0.941363286435481
Tgm1	Tgm1	-0.0727635392834146	-0.439880258949917	0.152579687997815	0.696082205710808	0.941363286435481
Ppm1d	Ppm1d	0.0187543909583023	5.06175593946376	0.152234322174841	0.696409239236205	0.941682699832046
Tspan9	Tspan9	-0.0385898887124291	6.80782485899442	0.152138722360082	0.696499829111321	0.941682699832046
Slc16a3	Slc16a3	0.0686287584749287	0.0261985872738768	0.152074325921859	0.696560887643267	0.941682699832046
Hrh1	Hrh1	-0.0622355227581344	-0.0336463245246688	0.151857237574924	0.696756701481193	0.941682699832046
Immt	Immt	0.0100751333971845	6.84596405319824	0.151807215462483	0.696814266472542	0.941682699832046
Tars1	Tars1	0.0120391305161103	6.34726359076693	0.15175682327979	0.696862096874136	0.941682699832046
Slc26a1	Slc26a1	0.0260078283596944	2.71219503331016	0.151715668988092	0.696901167694525	0.941682699832046
Rab2b	Rab2b	0.0117572299670522	5.24621491036122	0.151693534896585	0.696922180833401	0.941682699832046
Hiatl3	Hiatl3	-0.033716849507738	2.14285104483375	0.151466261378211	0.69713806608338	0.941822588901033
Gpsm1	Gpsm1	-0.0084682032425996	8.29745201789325	0.151400467715611	0.697200597750732	0.941822588901033
Zbtb4	Zbtb4	0.0153095455454093	6.08268504916249	0.15130912098607	0.697287441528474	0.941822588901033
Bbs4	Bbs4	-0.0150092326425566	5.62111148539005	0.151259054273666	0.697335052988937	0.941822588901033
Dhx37	Dhx37	0.0263017551172534	5.59803946156825	0.151147422832707	0.697441262771851	0.94187020099195
Cacna1h	Cacna1h	0.0270796906645789	7.51370445768804	0.150999686643015	0.697581869919627	0.94187020099195
Gpr158	Gpr158	-0.0332740063587841	4.42800093790997	0.150951769850858	0.697627468453445	0.94187020099195
Snd1	Snd1	0.0084640300579180	7.52016468244318	0.150912506605607	0.697664855138864	0.94187020099195
Stard3nl	Stard3nl	-0.0132693277750351	5.9670104680792	0.150674870801652	0.697891260112131	0.941961054672156
Dguok	Dguok	0.025382647327613	3.39385147474574	0.150385128674074	0.69816758694654	0.941961054672156
Ppp6r1	Ppp6r1	-0.0127019663322318	7.23885052145036	0.150380532149431	0.698171972160827	0.941961054672156
Dffb	Dffb	-0.0206498618585005	3.4277483626396	0.150379171365593	0.698173271638567	0.941961054672156
Ercc6	Ercc6	0.0262515303295862	5.608766870731	0.150372020832134	0.698180095342124	0.941961054672156
Rapsn	Rapsn	-0.0768826292568074	-0.30884647978261	0.150366747563169	0.698185127323663	0.941961054672156
Pard6b	Pard6b	0.0249644948153191	3.2835635725792	0.149788026798355	0.698738009811377	0.942588146636122
Cenpq	Cenpq	0.0377525578046893	1.60968009519345	0.149621517028581	0.698887742766736	0.942588146636122
Psmd5	Psmd5	-0.0137133482114597	5.52867203197447	0.149627696537088	0.698891398957769	0.942588146636122
Gmip	Gmip	0.0235528823385552	5.70663653037778	0.149494451838677	0.699018947045979	0.942588146636122
Tubgcp	Tubgcp6	0.0196368503284354	5.26126690923911	0.149485351646078	0.699027660561356	0.942588146636122
Pkib	Pkib	0.069397653214889	0.785974020688151	0.149293144624181	0.699211771760475	0.942640036908622
RGD156	RGD15661	-0.0713408767231926	0.362108985549183	0.149287434918364	0.699217243054363	0.942640036908622
Hmgcl	Hmgcl	0.0183046559039414	4.26225445407874	0.149058706103934	0.699436520138094	0.94283377936074
Mme	Mme	0.0243433856338703	3.26626171785662	0.148752021338919	0.699729875158295	0.942909837279518
Actr3	Actr3	-0.0095301207166405	8.55845477362289	0.148745706817849	0.699736898889586	0.942909837279518
Gch1	Gch1	0.0751753638703344	0.795541875601316	0.148697985668662	0.699782727747894	0.942909837279518
Mocs2	Mocs2	-0.0171823783444519	4.56210388178041	0.148601879480527	0.699875049672042	0.942909837279518
Mirlet7b	Mirlet7b	-0.080524891701916	-0.314698660250919	0.148488314590551	0.699984184875103	0.942909837279518
Porcn	Porcn	0.0149288113096474	5.2018448472114	0.14836759205383	0.700100252168819	0.942909837279518
Gins1	Gins1	0.0313149818642858	2.43095145120078	0.148326037764633	0.700140216606836	0.942909837279518
Slirp	Slirp	-0.0247236030215885	3.54551881161724	0.148272164596577	0.700192038178831	0.942909837279518
Atcay	Atcay	-0.017753403735117	8.68178054275553	0.148164910326841	0.700295240188786	0.942909837279518
Abhd11	Abhd11	0.0173778239404877	4.55755563671334	0.148040229610807	0.700415264162842	0.942909837279518
Plk1	Plk1	0.0232225622702673	3.53152177091758	0.148036091740217	0.700419248481376	0.942909837279518
Rorb	Rorb	0.0138436909148205	6.42233061396397	0.147963782140433	0.700488884106262	0.942909837279518
Stt3b	Stt3b	0.0136589342314366	6.65187965872964	0.147815345854958	0.700631895457037	0.942909837279518
Ugcg	Ugcg	-0.0089258313732909	7.58328387822148	0.147806418525818	0.700640499086203	0.942909837279518
Tlcd5	Tlcd5	0.0287273675802126	2.56561517212014	0.147745601794711	0.70069911857412	0.942909837279518
Cep70	Cep70	-0.0185813361196244	4.38865485142092	0.147598824975202	0.700839485032094	0.942909837279518
Ndufa7	Ndufa7	-0.0206349748610996	5.56916831920719	0.147567191258384	0.700871163263777	0.942909837279518
Atl3	Atl3	-0.0267193712961522	3.1935426909675	0.147553315250292	0.700884549165861	0.942909837279518
Rnf113a	Rnf113a1	-0.0221273692796457	3.21842703631823	0.14744667098265	0.700987450814416	0.942909837279518
Ppid	Ppid	-0.0130142459323782	6.59177692804839	0.147244331952251	0.701182806326852	0.942909837279518
Evpl	Evpl	0.0675044952679637	0.144777234631475	0.147166779578995	0.701257722878978	0.942909837279518
Lypd5	Lypd5	0.0650714633401724	1.02334918467232	0.147159586785862	0.701264662691695	0.942909837279518
Rftn1	Rftn1	-0.0334775760374804	4.4244790614174	0.147102499253236	0.701319825633883	0.942909837279518
Mrpl38	Mrpl38	0.0125004323954365	5.38084470061973	0.147097003238878	0.701325147040756	0.942909837279518
Dpy30	Dpy30	0.0210447011456562	5.1874809490795	0.147037934080259	0.701382228303615	0.942909837279518
Mad2l1b	Mad2l1bp	0.02255982596322	3.48751402574892	0.146718107540302	0.70169159069506	0.943071252709201
Ckap4	Ckap4	-0.0125235197069226	6.28038893784899	0.146609211452144	0.701797008382649	0.943071252709201
Il13ra1	Il13ra1	0.0332264578120745	2.24480617982167	0.146608909779644	0.701797300481353	0.943071252709201
Epha4	Epha4	-0.0201314658151832	8.03418496618577	0.146475858038912	0.70192616333324	0.943071252709201
Kif16b	Kif16b	0.0199750363020338	3.67186555147519	0.146474352135797	0.701927622209722	0.943071252709201
Smad9	Smad9	-0.0344196707888416	1.65712518458673	0.146402028856997	0.701997697087727	0.943071252709201
Sdhaf1	Sdhaf1	0.0211031394457677	6.05229579310052	0.146205054209886	0.702188648816305	0.943071252709201
Nudt1	Nudt1	-0.0368598159589181	1.71808026331261	0.14613596755941	0.702255657918785	0.943071252709201
Cdk11b	Cdk11b	0.0097161383931175	6.85373597921759	0.146130161508903	0.702261290200713	0.943071252709201
Stx2	Stx2	0.0327394308655337	3.647447942279	0.146123618201472	0.702267637823883	0.943071252709201
Malrd1	Malrd1	-0.0669645448827792	0.254975010975843	0.146055487717703	0.702333740496425	0.943071252709201
Cct6b	Cct6b	0.0666482880779674	-0.282684509767258	0.145799888152537	0.702581880382484	0.943240460603302
Rnls	Rnls	0.0241086652550086	2.4955291732822	0.145697137768521	0.702681423360176	0.943240460603302
Dscc1	Dscc1	0.0378359614709646	1.50026062976092	0.145587751991485	0.702788032446777	0.943240460603302
Zbtb24	Zbtb24	0.0156086362392345	4.03804976139697	0.145510348789749	0.702863291496885	0.943240460603302
Pde8b	Pde8b	-0.0418725686350476	3.82734686082403	0.145477067365007	0.702895458039665	0.943240460603302
Vps41	Vps41	-0.0118358905909763	7.2215357414245	0.145458407354546	0.70291380695561	0.943240460603302
Shtn1	Shtn1	0.0097120715170710	8.29183219826935	0.145296750735668	0.703071091670375	0.943240460603302
Ubr2	Ubr2	0.0273366043189515	6.45559845084666	0.145141350884487	0.703222383323537	0.943240460603302
Kctd1	Kctd1	0.0177836422192715	5.36368335108164	0.145135859741785	0.703227730996936	0.943240460603302
Eri2	Eri2	-0.0205598678212687	3.80602852961656	0.145079580906536	0.703282543169242	0.943240460603302
Zfp637	Zfp637	-0.0156736450949932	6.07795598627035	0.145070556871035	0.703291326458485	0.943240460603302
Sf3a3	Sf3a3	-0.0142184380660609	6.14583367644539	0.144909652362156	0.703448127947833	0.94334934255171
Nudt18	Nudt18	-0.02844226018191	3.05705331059468	0.144720916776521	0.703632168167951	0.943377827329429
P2rx7	P2rx7	0.029844284586183	3.12719709235674	0.144592115888214	0.703757802833383	0.943377827329429
Mical1	Mical1	0.0246582574162412	5.34045030689083	0.144574928146525	0.70377461838544	0.943377827329429
Dazap2	Dazap2	-0.0089757444252345	6.57765185554219	0.144497041118557	0.703850651295677	0.943377827329429
Rmdn2	Rmdn2	-0.0355532518648722	0.936532014134274	0.144445661922759	0.703900820239163	0.943377827329429
Ctr9	Ctr9	-0.0091110936921467	7.42002003623773	0.144422925702798	0.703923024159412	0.943377827329429
Slc16a1	Slc16a1	-0.0159166596651001	5.8923068160686	0.144250138117357	0.704091831842989	0.943488383632061
Rit1	Rit1	-0.0118549505927023	5.58922870055238	0.144130124535323	0.704209149185467	0.943488383632061
Rdh11	Rdh11	0.0136290490082135	5.29812289589552	0.143928962762601	0.704405817034804	0.943488383632061
RGD156	RGD156298	-0.0162182594641332	5.82127235194479	0.143811767977482	0.704520624487804	0.943488383632061
RGD156	RGD156028	-0.0614667363040252	1.03287428082708	0.143809200816676	0.704523137761668	0.943488383632061
Afap1l1	Afap1l1	-0.0280398198562584	3.24596032976556	0.143726978900069	0.704603647342589	0.943488383632061
Traf3ip1	Traf3ip1	0.0251546992206793	4.18302094442868	0.143651982904771	0.704677104460826	0.943488383632061
Rd3l	Rd3l	-0.057175537071388	0.696133767388731	0.143569608033388	0.70475791431978	0.943488383632061
Six3	Six3	-0.0813607823176436	3.96458319899942	0.143567209135625	0.704760165126256	0.943488383632061
Ankrd23	Ankrd23	-0.06177034307565	0.142618316570352	0.143492414130222	0.70483347196773	0.943488383632061
Thap6	Thap6	-0.0357782747646758	1.42229924156688	0.143488490051688	0.704837318573676	0.943488383632061
Pde4dip	Pde4dip	0.0139976541781373	8.18621985372228	0.143208280103089	0.705112152007967	0.943668334976264
Zfp511	Zfp511	-0.0191871050588557	3.37347751081875	0.143179474803496	0.70514042202583	0.943668334976264
Sf3b1	Sf3b1	-0.0213438184140335	8.8074067506809	0.14311939357961	0.705199397238651	0.943668334976264
Il2rg	Il2rg	0.0423316729494012	1.30727912159647	0.142599362483281	0.705710467577603	0.944129161205404
Nusap1	Nusap1	-0.0285960521941076	3.52184066998107	0.142348675750185	0.705957185432566	0.944129161205404
Acadm	Acadm	-0.0251088088880664	3.80226664944327	0.142347252514079	0.705958488482994	0.944129161205404
RT1-DM	RT1-DMb	-0.0641381036225952	0.926031340978201	0.142332181151517	0.705973428904167	0.944129161205404
Crmp1	Crmp1	0.0137196147629381	11.2987776578375	0.142289487950251	0.70601547710328	0.944129161205404
Col2a1	Col2a1	-0.0548467390454706	5.04338133418493	0.142273471612782	0.706031253327116	0.944129161205404
Dck	Dck	-0.0189363315488032	4.30611817656489	0.142231388834979	0.706072710043089	0.944129161205404
Shank2	Shank2	0.0189538505086623	8.0192877557885	0.142021772866656	0.706279311991534	0.944304219429717
Mau2	Mau2	0.0236358758072889	7.28173156653411	0.141929579304744	0.706370235056533	0.944324592606918
Atp5mc2	Atp5mc2	-0.0190678911450683	7.41333610990852	0.141778717344849	0.706519090791354	0.944422401521719
Tafa4	Tafa4	-0.0650648722108664	1.37108490620579	0.14163927471337	0.70665675949475	0.944505237087948
Coq4	Coq4	-0.0150352469215522	3.99281081301655	0.14143488734144	0.706858686615319	0.944519132816747
Arhgef9	Arhgef9	-0.0125859790015108	6.64110124881936	0.141404176097183	0.706889042571698	0.944519132816747
Igfbpl1	Igfbpl1	0.0326376486775266	6.12295203482644	0.141380160641836	0.706912782525509	0.944519132816747
Snx6	Snx6	0.0112631866886714	6.49252939521777	0.141188617300081	0.70710221360283	0.944519132816747
Sgsm2	Sgsm2	0.0105822518735429	7.16816630490757	0.14116994149024	0.707120691278416	0.944519132816747
Gulo	Gulo	0.160004371223465	-1.25682292022682	0.141100349828837	0.707189559787125	0.944519132816747
Chid1	Chid1	0.0139019095695627	5.55710844955126	0.14105456805742	0.707234871633212	0.944519132816747
Tcerg1	Tcerg1	0.022574163039921	7.52099667080718	0.14101629314365	0.707272762515578	0.944519132816747
Fbxl4	Fbxl4	-0.0204545482641898	3.62152472885678	0.140756488046279	0.707530116070555	0.94476168287375
Slc35a5	Slc35a5	0.013348695746777	5.2180910214732	0.140517415452322	0.707767172235563	0.944949592477342
Samd5	Samd5	-0.0318318666934586	3.56334339889275	0.140461916296587	0.707822256179149	0.944949592477342
Tbc1d25	Tbc1d25	-0.0130967346822662	4.81222240247937	0.139873764434256	0.708406529768777	0.945278044224814
Use1	Use1	-0.0144698131482486	5.60921062334289	0.139731107547583	0.70854847419305	0.945278044224814
Ube2l3	Ube2l3	-0.0088063323613437	7.55498006322462	0.139306486278365	0.708971434356314	0.945278044224814
Tagap	Tagap	0.0558422683826829	-0.190823036191613	0.139210761065343	0.709066886511606	0.945278044224814
Mxra8	Mxra8	-0.0337747456859776	3.45016863380232	0.139148624304568	0.709128866038298	0.945278044224814
Rab3c	Rab3c	0.0225223666109713	5.42104492815887	0.139145485239624	0.709131997578262	0.945278044224814
Smpd3	Smpd3	0.0095876088214615	8.32985303139194	0.139102741761469	0.709174642596435	0.945278044224814
Tfg	Tfg	0.0094407054991090	6.6515608612555	0.139054459192501	0.70922282292384	0.945278044224814
Noc2l	Noc2l	0.0237749564983002	6.52661114996493	0.139002240042331	0.709274962222308	0.945278044224814
Rfxank	Rfxank	0.0235263023298023	2.59278376218562	0.138919850121788	0.70935719728008	0.945278044224814
Nfatc2ip	Nfatc2ip	0.0232795409054847	2.84159012909049	0.138713725974775	0.709562105788159	0.945278044224814
Bud23	Bud23	-0.0179181614007316	4.32187457407889	0.138699362857208	0.70957745927115	0.945278044224814
Ccdc150	Ccdc150	-0.063919573990619	0.179357400090486	0.138642329119165	0.709634468570811	0.945278044224814
Mir2985	Mir2985	-0.0663793927222371	0.968276782044032	0.138569408002982	0.7097083764579	0.945278044224814
Tmed2	Tmed2	-0.0109005972850517	7.58818031629177	0.138549648282009	0.709727126392311	0.945278044224814
Ostf1	Ostf1	-0.0220027610796266	3.7756695118024	0.138436070587403	0.709840746269823	0.945278044224814
Dmc1	Dmc1	0.0767164602028372	0.519458990447773	0.138370852028316	0.709906007290688	0.945278044224814
Grin2c	Grin2c	-0.0614341191973176	2.1220501231809	0.13827173810769	0.710005219270999	0.945278044224814
C1qtnf1	C1qtnf1	0.0290820155563525	2.21028242930255	0.138257664728826	0.710019309854788	0.945278044224814
Fam8a1	Fam8a1	0.0085395755077021	7.35180792580068	0.138165711173478	0.71011139592207	0.945278044224814
Clk4	Clk4	-0.0291546133008264	4.78100267827233	0.138151236530803	0.710125894800659	0.945278044224814
Snrnp48	Snrnp48	0.0293274043259297	3.70273571075649	0.138137188578623	0.710139986741825	0.945278044224814
Ccnt2	Ccnt2	0.0401514902717005	6.29889704035683	0.138112217932957	0.710164982297482	0.945278044224814
Cacng4	Cacng4	0.0107075650413399	7.3258566146396	0.138002487721344	0.710274940529365	0.945278044224814
Nisch	Nisch	0.0130128845668953	7.87365316708935	0.137935951370082	0.710341628374041	0.945278044224814
Tle2	Tle2	-0.0386375699795452	4.72717485079469	0.137869229638121	0.710408096413019	0.945278044224814
Zmynd1	Zmynd10	-0.0389970966636084	4.35363007106216	0.137582046707067	0.710696716317904	0.945278044224814
Pkp1	Pkp1	0.0553038652498878	0.762245931465676	0.137561810447394	0.710717025303961	0.945278044224814
Fbxo41	Fbxo41	0.0112161664711179	7.52511398571411	0.137537274807876	0.710741673526275	0.945278044224814
Trpm8	Trpm8	-0.0364542016327287	1.70339799016175	0.137521707296798	0.710757307420957	0.945278044224814
Ppt1	Ppt1	-0.0145709613935804	5.59689689988561	0.137471384842294	0.710807841304343	0.945278044224814
Rbks	Rbks	0.0312437218543521	2.19720595336598	0.13745982174389	0.710819366369462	0.945278044224814
Cryz	Cryz	-0.0218535597658576	3.11634822909654	0.137125577150407	0.711155465907927	0.945278044224814
Sstr4	Sstr4	-0.0250108841703884	3.4583623830181	0.137111580922053	0.711169626237731	0.945278044224814
LOC100	LOC100369	0.0311165054263094	1.2885661912871	0.136934122087347	0.711348125734469	0.945278044224814
Kctd4	Kctd4	-0.0229986790216713	4.71044511819474	0.136886355536802	0.711396229225493	0.945278044224814
Psrc1	Psrc1	0.0270237888531561	2.50925167746865	0.136873834144247	0.711408887899418	0.945278044224814
Ppef2	Ppef2	-0.0404988012386754	2.48914035465018	0.13680380658964	0.711479365676766	0.945278044224814
Trrap	Trrap	0.0375662996330638	8.20203691821662	0.13678989493792	0.711493379145666	0.945278044224814
Cndp2	Cndp2	-0.0144007446535066	5.65344273482956	0.136756795901604	0.711526723685366	0.945278044224814
Tbc1d22	Tbc1d22b	-0.0121372138573152	6.31612159299428	0.136665770819398	0.711618467581559	0.945278044224814
Pdcd4	Pdcd4	0.0114627352091205	6.1420029330794	0.136561132562065	0.711723892071311	0.945278044224814
RGD156	RGD156584	-0.0358675403512917	1.51912313776662	0.136465204035986	0.711820676756091	0.945278044224814
Hopx	Hopx	-0.0342245202646474	2.90061113880906	0.136389064371258	0.711897491728091	0.945278044224814
Gabpb1	Gabpb1	0.0121707657482642	5.64508359944798	0.1362801106456	0.712007454190633	0.945278044224814
Pmvk	Pmvk	0.0164742657962821	5.59272553675348	0.136254338268099	0.712033472485258	0.945278044224814
Slc26a4	Slc26a4	-0.0648897855433054	-0.0927305529023901	0.136178853624997	0.712109693477022	0.945278044224814
Mad1l1	Mad1l1	-0.0152298037901181	4.22919456949827	0.13617225803776	0.712116354544149	0.945278044224814
Psmg2	Psmg2	0.0177832853858759	4.03319485949302	0.136085264149671	0.71220422924965	0.945278044224814
Cilp	Cilp	0.077843281570338	-0.366113631567865	0.135996686381404	0.712293726664764	0.945278044224814
Alg1	Alg1	-0.0196133841239136	3.80564321282829	0.135962210395277	0.712328593437618	0.945278044224814
Stk26	Stk26	-0.0907409668562396	0.567858013853527	0.135848542147542	0.712443509742968	0.945278044224814
Stk38l	Stk38l	-0.0231179145893062	3.73623624652388	0.13573485731736	0.712558507451991	0.945278044224814
Hmgb1	Hmgb1	0.0107034777255639	8.50360174861383	0.135682484691188	0.712611503301421	0.945278044224814

Pcyox1l	Pcyox1l	-0.0122688115785156	5.23377275211496	0.13286616948794	0.715478811447033	0.966186612259995
Prkag1	Prkag1	-0.0150154111980332	6.48796870521348	0.132847070843098	0.715498171589532	0.966186612259995
Cps1	Cps1	0.0416363771273603	0.970791092163019	0.132516281881175	0.715837206231076	0.966453997584141
Zfp956	Zfp956	-0.015616777540586	4.11485088937521	0.132493359850921	0.715860718421207	0.966453997584141
Arnt2	Arnt2	0.0096253738971043	8.82771081929763	0.132422180258261	0.715933730973008	0.966453997584141
Lpcat4	Lpcat4	-0.0166823895584545	5.40798789241578	0.132341102412326	0.716016846743175	0.966453997584141
Clns1a	Clns1a	-0.0147983561830285	6.24144510832805	0.132280025488356	0.716079645523306	0.966453997584141
Depdc5	Depdc5	0.0106910074688688	5.97141685328852	0.13204443271087	0.716321647963049	0.966592715662249
Lamp5	Lamp5	0.107100234747577	2.77102447382829	0.131986683652666	0.716381005465251	0.966592715662249
Adamts4	Adamts4	-0.0447122921548301	1.48694643779957	0.131955340563463	0.716412199567082	0.966592715662249
Mrps9	Mrps9	0.0135126727265116	4.93015422729821	0.131865152200188	0.716505868848225	0.966606366574906
Pigc	Pigc	0.0145380433701087	4.85136077751522	0.131741681393913	0.716632984901048	0.966606366574906
Tbc1d31	Tbc1d31	0.0243229624484856	4.06057035428682	0.131725025882275	0.716650125342355	0.966606366574906
Tfrc	Tfrc	0.0150149360909232	6.74988530682358	0.131609422160977	0.716769136889412	0.966628977587791
Mir124-2	Mir124-2	0.0361690798939693	1.01740181472653	0.131553653351183	0.716826565708418	0.966628977587791
Cort	Cort	-0.0504128265542046	0.623785236605566	0.131384520358452	0.717000817981165	0.966768886226161
Mphosp	Mphosph8	0.0122224199096653	6.03635232486381	0.13127508211501	0.717113636304923	0.966817655045003
Foxo3	Foxo3	0.0150613399589317	4.81284549059517	0.131023406532414	0.717373287072074	0.966872525925703
Dhrs4	Dhrs4	0.0213760838211256	2.8990501354515	0.131014259347552	0.717382729426123	0.966872525925703
Mtpn	Mtpn	-0.00896708597007707	8.21375411481389	0.130657722916279	0.717751060688291	0.94706588511215
Rmdn1	Rmdn1	-0.0154746701322506	3.79231236686634	0.130533947553118	0.717879065002506	0.94706588511215
Cct4	Cct4	0.0107588322165351	8.19243982982707	0.13042973676572	0.717986888630554	0.94706588511215
Stk36	Stk36	0.0377523753221857	4.64955897880244	0.130356759305627	0.718062425108104	0.94706588511215
Phf20l1	Phf20l1	0.0205817412519066	6.38081330685494	0.130344002737857	0.718075621449758	0.94706588511215
Zc3h12a	Zc3h12a	0.0448913868537533	0.200193474249368	0.130282552328808	0.718139258727798	0.94706588511215
Arid4b	Arid4b	-0.0209098175585864	5.99979485784841	0.130262506788682	0.718160018045983	0.94706588511215
Coq10b	Coq10b	-0.0177182980442176	4.24189370647712	0.130248785653642	0.718174228801617	0.94706588511215
Syt6	Syt6	0.0244724838007216	4.27718057355849	0.130170960498859	0.71825484705629	0.94706588511215
Cfap126	Cfap126	0.0448093063953955	2.72098108868292	0.130025067023467	0.718406049982672	0.94706588511215
Ptcd3	Ptcd3	-0.0117193649466106	6.08984557728 86	0.129944898283682	0.718489177087985	0.94706588511215
LOC688	LOC688316	-0.0250534273961414	2.36585410271841	0.129874488638961	0.718562208872489	0.94706588511215
Plac9	Plac9	0.0763896145663285	0.0679484586105386	0.129854235540909	0.718583116807114	0.94706588511215
Pcbp2	Pcbp2	0.0084822950749452	7.93058929896339	0.12984351501332	0.718594343077213	0.94706588511215
Rassf1	Rassf1	-0.0151080527154024	4.75341555827908	0.129783557714035	0.718656658278377	0.94706588511215
Bola1	Bola1	-0.0225775743784086	4.05707782870143	0.129705210172631	0.718737883052357	0.94706588511215
Irf2bp1	Irf2bp1	0.0120710021818514	6.64381402021132	0.129636011928419	0.718809733048811	0.94706588511215
Cd55	Cd55	-0.0362368191650435	2.42524370330353	0.129626373175597	0.718819742887879	0.94706588511215
Psme4	Psme4	0.0197124966123737	7.12655009481449	0.129539673278074	0.71890979958328	0.94708452849758
Sema3e	Sema3e	-0.0301894383857756	3.60809704616025	0.129406461781031	0.719048235044601	0.947166884916754
Rbfox3	Rbfox3	0.0116523124298377	6.40272895399569	0.129312582152675	0.719145843523285	0.947195470245965
Nudcd1	Nudcd1	0.0155388424813003	3.9155507581236	0.12922209608532	0.719239963590931	0.947219445400 47
Cant1	Cant1	-0.0138464997248771	5.31952471587114	0.129083640858894	0.719384049714403	0.947273515351 68
Ddx47	Ddx47	-0.0109834832954441	5.76772575045923	0.128959902834083	0.719512880962685	0.94727351535168
Spsb1	Spsb1	-0.0209468052487416	3.24526448025779	0.128892556832184	0.719583048420345	0.94727351535168
Epha7	Epha7	0.013550684304958	6.48196538888042	0.128890965715705	0.719584706139555	0.94727351535168
Rdh13	Rdh13	0.015709670822684	4.18059650247051	0.128536331638962	0.719954473461906	0.94735212720 3647
Pcnx4	Pcnx4	0.0081318712708168	6.76314542375284	0.128377567163767	0.720120199423022	0.947352127203647
Eif4e2	Eif4e2	-0.0108644834396986	5.49612504730433	0.128335069741595	0.720164579883928	0.947352127203647
Dbf4	Dbf4	0.0265880206174953	2.51271476106116	0.128320888888595	0.720179390926 08	0.947352127203647
Grk2	Grk2	-0.00873510697121 22	7.49779185588977	0.128308562896244	0.720192265432506	0.947352127203647
Slc2a6	Slc2a6	-0.0107742010267663	5.68290012933265	0.128304752973637	0.720196243984962	0.947352127203647
Tfap2b	Tfap2b	0.104708786818937	3.13688367212237	0.128298145853691	0.720203146582059	0.947352127203647
Lama3	Lama3	-0.0460698929924775	4.00644089458581	0.128215253416684	0.720289749865168	0.947352127203647
Ubiad1	Ubiad1	0.014076211 2090198	4.58307867286031	0.128178868534206	0.720327773565841	0.947352127203647
Oscp1	Oscp1	-0.0146271848972871	4.30557707586425	0.128073437698983	0.720437887523312	0.947397214410662
Dennd1	Dennd1a	0.0121195280503604	6.23114027017499	0.127980762494857	0.720534809425647	0.947424881451194
Depdc1	Depdc1	0.0364984328189173	1.55258972816908	0.127681969477884	0.720847663983032	0.947476298437375
Tnfrsf1b	Tnfrsf1b	-0.0413436556480725	1.12841042885335	0.127626810535536	0.720905445399036	0.947476298437375
Suox	Suox	-0.0166679522217656	4.03395191328368	0.127615480298667	0.720917316084852	0.947476298437375
Rab27b	Rab27b	-0.0362576765064452	2.18942188996855	0.127570943810156	0.720963882640752	0.947476298437375
Ager	Ager	-0.0530243866930007	2.55296823231136	0.127421612840482	0.721120521744561	0.947476298437375
Vta1	Vta1	0.015285009779 3865	6.37303357354579	0.127318513282304	0.721228659988116	0.947476298437375
Tfb1m	Tfb1m	0.0172931391567566	4.37989983501278	0.127264866122111	0.721284947988176	0.947476298437375
Itpr3	Itpr3	0.0306345062841561	3.16559889098762	0.127235473098253	0.721315793581298	0.947476298437375
Gpn1	Gpn1	-0.00980435251167 46	5.71818333323204	0.127187514978544	0.721366130359339	0.947476298437375
Mrpl18	Mrpl18	-0.0160421718526124	4.46219761117626	0.127157667248973	0.721397463896972	0.947476298437375
Dhx34	Dhx34	0.0157971663233279	4.26197060109104	0.127061908415001	0.721498017557866	0.947476298437375
Trpc1	Trpc1	0.0135792740677704	5.24063063225738	0.127026103421727	0.721535626344184	0.947476298437375
Abcd2	Abcd2	0.0246154669688415	3.76052986649401	0.126938655161055	0.721732614433988	0.947476298437375
Slc11a1	Slc11a1	0.0664309707780213	0.177002954999107	0.126812156001478	0.721760476648904	0.947476298437375
Hmces	Hmces	-0.0159188066636471	4.56454519566529	0.126751935508409	0.721823804554798	0.947476298437375
Fam189b	Fam189b	-0.0156761070848353	6.07200304362018	0.126647639640623	0.721933522262422	0.947476298437375
Nppa	Nppa	-0.0434899998448611	1.08696726788074	0.12664485070826	0.721936456876563	0.947476298437375
Polr2e	Polr2e	-0.0152957624522655	6.05164315024191	0.126640585589547	0.721940944878105	0.947476298437375
Ndufb10	Ndufb10	-0.0142988861023234	6.19890153788231	0.12651587198602	0.722072212671103	0.947510194931721
Mdp1	Mdp1	-0.0162646123882274	4.30240561934688	0.126229856837043	0.722372534614389	0.947510194931721
Lysmd1	Lysmd1	-0.0122254278817301	5.07399957167807	0.126213622094276	0.722390649724376	0.947510194931721
Pctp	Pctp	-0.0613511797151934	-0.256119340933002	0.12615026018364	0.722457458436925	0.947510194931721
Dym	Dym	-0.0103896455348 07	6.25743224803 31	0.126148880241601	0.722458914688706	0.947510194931721
Top3b	Top3b	-0.0136792045216491	5.49092437860157	0.126055635843613	0.722557267920321	0.947510194931721
Rnf34	Rnf34	0.010961893749428	5.61778770158792	0.125956345053197	0.722662043867026	0.947510194931721
Trmt5	Trmt5	0.0170381606389194	3.68851171331678	0.125926611050851	0.722693429522318	0.947510194931721
Ccnq	Ccnq	0.022980206455626	3.48128331575018	0.125914982883899	0.722705704741034	0.947510194931721
Pcdhgc5	Pcdhgc5	-0.0562594073619 52	2.17170334489278	0.125800202501011	0.722826806356349	0.947510194931721
Rufy3	Rufy3	0.0092926401137569	8.24450237252229	0.125746516973727	0.722883616614251	0.947510194931721
Synpo	Synpo	-0.026371228722699	4.04135735584404	0.125677618036434	0.722956417402305	0.947510194931721
Ing4	Ing4	0.0122779432913354	6.12018829940286	0.125540568741513	0.723101294814637	0.947510194931721
Sirt5	Sirt5	-0.0109453403974841	6.70313379162769	0.125458810743879	0.723187765137608	0.947510194931721
Letmd1	Letmd1	-0.0175708170369241	5.45227570948241	0.12542156403687	0.723227169164988	0.947510194931721
Gemin4	Gemin4	0.0160891112606078	3.99778642195 36	0.125390703844197	0.723259821760931	0.947510194931721
Slc41a2	Slc41a2	-0.0138549347702397	5.89573573243768	0.125343106624236	0.723310192361057	0.947510194931721
Tmem87	Tmem87b	0.0195736871227318	4.58931391425393	0.125260903713908	0.723397210395782	0.947510194931721
Mrpl10	Mrpl10	-0.0148027991425417	5.02494845862546	0.12515804589539	0.723506128487676	0.947510194931721
Sec13	Sec13	-0.0117829052495604	5.61737300878807	0.125150271142417	0.723514374123659	0.947510194931721
Eci2	Eci2	0.0355881688836425	3.60307552389152	0.125079455616444	0.723589400808217	0.947510194931721
Wdr75	Wdr75	0.0130748316360592	5.01288833406546	0.124971219229813	0.723704119830635	0.947510194931721
Gapdhs	Gapdhs	-0.0319980594288634	1.92058813778629	0.124894520583766	0.723785446049811	0.947510194931721
RGD130	RGD130501	-0.022073109308234 6	3.33828974844503	0.124758029378995	0.723930041882947	0.947510194931721
Atg7	Atg7	-0.0159277188711007	4.07208859926722	0.124668239901119	0.724025543089723	0.947510194931721
Uba52	Uba52	-0.0181044206718253	8.42027139880089	0.124661087520565	0.724033136157265	0.947510194931721
Txnl1	Txnl1	-0.00958030957736 76	6.60737142181 12	0.124619530318292	0.724077258830008	0.947510194931721
Slc30a3	Slc30a3	0.0292063374 0977	4.13745977096744	0.124580807782564	0.724118379289 38	0.947510194931721
Map4k1	Map4k1	-0.0206448235137246	2.78629478939531	0.124489704800915	0.724215152247795	0.947510194931721
Apba3	Apba3	-0.020554795341974	4.10711014716892	0.124423629707167	0.724285364572483	0.947510194931721
Lrrc75b	Lrrc75b	0.0154760642105456	4.90616352561774	0.124284113239518	0.72443368562582	0.947510194931721
Pus10	Pus10	-0.0284459820883694	2.43422806394693	0.124238851455402	0.724481823890837	0.947510194931721
Ei24	Ei24	-0.0092580088383694	7.01225411155814	0.124122775815948	0.724605321455068	0.947510194931721
Spidr	Spidr	0.0385355282 0478	1.53724738141092	0.124035763635469	0.724697828805037	0.947510194931721
Epcam	Epcam	-0.0432498794668276	2.67556422111701	0.123965801891472	0.72477243 5651	0.947510194931721
Golga1	Golga1	-0.0187234483138115	5.29279575519755	0.123902021423547	0.724840370213866	0.947510194931721
Rdh16	Rdh16	-0.0640001886315845	-0.451893792623773	0.123879526150077	0.724864335318033	0.947510194931721
Arhgap8	Arhgap8	0.0484521730557221	0.58605269 0526938	0.12387685226895	0.724867184071918	0.947510194931721
Racgap1	Racgap1	0.0220027567804957	3.98602751921571	0.12372516797592	0.725028845182444	0.947510194931721
Kcnq3	Kcnq3	0.0201977858600578	6.76602166925814	0.123545581669589	0.725220387553724	0.947510194931721
Fam117a	Fam117a	0.0310944853292346	1.79406604132685	0.12350904633395	0.725259374395367	0.947510194931721
Acap2	Acap2	0.0211332266054357	4.99587199039955	0.123502677578898	0.725266171154337	0.947510194931721
Nell2	Nell2	0.0128213759141955	9.45771069230104	0.123491234822836	0.725278383400658	0.947510194931721
Mat2a	Mat2a	0.0198612352993351	6.98009782176301	0.123463339978258	0.725308156751853	0.947510194931721
Mkln1	Mkln1	0.0110339407002672	5.52598329056864	0.123030101613821	0.725771056328867	0.947850216886572
Rp9	Rp9	0.0158334130269446	3.63495090338975	0.123020238690856	0.725781605155225	0.947850216886572
Tmem17	Tmem179b	0.0171994748023622	8.25866585103705	0.123006459823969	0.725796343049589	0.947850216886572
Rxfp2	Rxfp2	-0.0724532450188321	-0.206260956151176	0.122925581608958	0.725882858493105	0.947863990101357
Med27	Med27	0.0184967140331154	4.39672356125103	0.12255791602405	0.726276613230857	0.948198241031472
Fbh1	Fbh1	-0.0092717419099415	7.27521670829396	0.122544659628872	0.726290822276 01	0.948198241031472
Serpine1	Serpine1	-0.0518882788901877	0.117087536293327	0.122368567579983	0.726479650802648	0.948232423129334
Fam193b	Fam193b	0.0242521519941388	6.38330129346676	0.122356073557867	0.726493054291074	0.948232423129334
Srek1	Srek1	0.030804641464705	5.48919019783666	0.122303496992657	0.726549466433929	0.948232423129334
Cds2	Cds2	-0.0196860426033576	5.5682055455406	0.12207818611877	0.726791368476747	0.948232423129334
Clip4	Clip4	-0.0245685147916491	3.37872079656417	0.122072574947282	0.726797396032445	0.948232423129334
Mettl7a	Mettl7a	0.0219099728437939	3.5197681555577	0.122012280150649	0.726862174893636	0.948232423129334
Serp1	Serp1	-0.0078194413247621	7.78386367908399	0.121996329522673	0.726879314757826	0.948232423129334
Kcnv2	Kcnv2	0.158023901317384	-0.921782170305948	0.121907485300135	0.726974795228361	0.948232423129334
Trim35	Trim35	0.0088972947891707	8.54449717582425	0.121778082060423	0.727113960557562	0.948232423129334
Zhx2	Zhx2	0.0206968839614328	4.25307308011255	0.121512705212737	0.727399694866371	0.948232423129334
Lztfl1	Lztfl1	0.0168717812647508	4.20933653550199	0.121504313829107	0.727408632506784	0.948232423129334
Mmp15	Mmp15	0.0112126266493284	6.81666585274864	0.121477147391872	0.727437893549882	0.948232423129334
Nrxn1	Nrxn1	0.0160461274655475	8.54365592526049	0.121438470896463	0.727479658435946	0.948232423129334
Tap2	Tap2	0.0406483621653539	1.62526478806533	0.121337292941845	0.727588689254886	0.948232423129334
Ccdc124	Ccdc124	-0.0180261153210637	6.20092020351318	0.121281536320423	0.727648716739356	0.948232423129334
Cyp7b1	Cyp7b1	0.0282915853898064	2.49440877350056	0.121240815002871	0.727692602753493	0.948232423129334
Egfl8	Egfl8	0.0346895723517702	2.04095969097274	0.121180269846647	0.727757900582367	0.948232423129334
Nadsyn1	Nadsyn1	0.0292155928941953	3.96914088120262	0.121160076806774	0.727779682802146	0.948232423129334
Ruvbl2	Ruvbl2	0.011098514831892	6.05376834364971	0.121126505374254	0.727818058635242	0.948232423129334
Necab3	Necab3	0.0240213177709233	4.68027954539598	0.121046567151571	0.72790432212948	0.948232423129334
Sco1	Sco1	-0.0145360228307906	4.04693061450387	0.121036552380868	0.727912971766672	0.948232423129334
Cdc42bp	Cdc42bpa	0.0183883078095749	7.76223885017788	0.120826156723965	0.728140177651362	0.948340293207113
Fam122b	Fam122b	0.0274529402538864	2.32838656387713	0.120787610843216	0.728181827105931	0.948340293207113
Pramef8	Pramef8	0.0204877578465145	2.6125101562599	0.120629351788352	0.728352906716212	0.948340293207113
Micos10	Micos10	-0.0156556759025 34	5.55640954932881	0.120517767500715	0.728473606048209	0.948340293207113
Bicdl1	Bicdl1	-0.0169886591260567	6.22873556445686	0.120475870098338	0.728527859935889	0.948340293207113
Zbtb17	Zbtb17	-0.0138504635632625	5.75985996827418	0.120436652974596	0.728559138818173	0.948340293207113
RGD130	RGD130694	-0.0129151238537731	5.00807918547598	0.120428018172326	0.72857073221646	0.948340293207113
Pelo	Pelo	-0.0152236622647276	4.7625662827717	0.120361781267285	0.728642439395369	0.948340293207113
Selenoi	Selenoi	0.0168864514219276	6.52537601855275	0.120327239895559	0.728679842187752	0.948340293207113
Taf9	Taf9	-0.0126832382177432	4.72911500542636	0.120141567649085	0.728880988723098	0.948503151967886
Adam8l1	Adam8l1	-0.0477661925892871	0.57257404758325 5	0.119982512673137	0.729052372059244	0.948611263864 07
Bicd1	Bicd1	-0.0206027451026661	5.11978356063261	0.119924060247769	0.729116868908401	0.94861126386407
Mctp2	Mctp2	-0.0617556788366832	-0.280572106240767	0.11985471339743	0.72919211799794	0.94861126386407
Med8	Med8	0.0195923849113885	3.50987088835468	0.119776006902796	0.729277555160984	0.948623442008299
Fam89b	Fam89b	0.013580975519345	7.2724441289256	0.11955204571602	0.72952084073565	0.948840980908862
Cript	Cript	-0.0151317952445021	5.65821081643743	0.119345573740915	0.729745354167964	0.948856752148 74
C17h6or	C17h6orf52	0.054240950213884	0.136308624174682	0.119299234631626	0.729795772238473	0.94885675214874
Rnaseh1	Rnaseh1	-0.0251365262650855	3.46392808 85993	0.119236849904837	0.729863665641235	0.94885675214874
Nek8	Nek8	0.0309106443649832	2.27249088186409	0.119210773670673	0.729892050341703	0.94885675214874
Nup54	Nup54	0.0132995065559242	5.0141618181338	0.119141695330313	0.729967260819122	0.94885675214874
Zpbp2	Zpbp2	0.0624001562576593	-0.205380329055508	0.119056586826492	0.730059658027267	0.94885675214874
Gk	Gk	0.015149905526 7729	4.31027275159759	0.118945532170255	0.730180968565421	0.94885675214874
Hmgcr	Hmgcr	0.0092463350371 0568	9.14292297715874	0.118931085283542	0.730196717601016	0.94885675214874
Lhb	Lhb	0.042612182340031	0.814282160322454	0.118911807821945	0.730217721486304	0.94885675214874
Exosc8	Exosc8	-0.0187425706535561	3.81120077842197	0.118842359474719	0.730293451220985	0.94885675214874
Dcaf12l1	Dcaf12l1	-0.017868833599687	4.45462227502456	0.118692268940958	0.730469092744148	0.948872531895 55
Mdm2	Mdm2	0.0112096725060871	5.64252597107018	0.1186267552388	0.730528682 97835	0.94887253189555
Crip3	Crip3	0.0630964660671081	-0.260205451788447	0.118475163472607	0.730693125732497	0.94887253189555
Mpp7	Mpp7	0.0331661627675619	1.59628643887824	0.118470961975483	0.730698807735967	0.94887253189555
Dpf3	Dpf3	0.0308334369583412	2.32228958168616	0.118429772942675	0.730743806576142	0.94887253189555
Zc3h6	Zc3h6	0.0192900191263845	4.18828636060457	0.118413218714267	0.73076189460482	0.94887253189555
Arhgap4	Arhgap4	-0.0425625444159324	4.01883169264539	0.118218033438637	0.730975269928678	0.948892649034496
LOC682	LOC682870	-0.0314947508525397	1.6449836730534	0.118185993178727	0.731010314957325	0.948892649034496
Chka	Chka	-0.0130247957213014	5.86564212411258	0.118005402976365	0.731207940520705 7	0.948892649034496
RGD130	RGD130517	0.0145871465096193	5.06734153084823	0.117982039847464	0.73123352027156	0.948892649034496
Nap1l4	Nap1l4	0.0095663934960181 2	8.15709238289 29	0.117980963784532	0.73123469880 6986	0.948892649034496
Pim2	Pim2	0.0149931158270494	4.63488626836895	0.117674103236801	0.731570922284375	0.949320237666556
Tmem19	Tmem196	-0.0242112221909067	3.28627759508001	0.117600902118106	0.731651212814645	0.949320237666556
Tma7	Tma7	-0.0163005132752011	6.01770040044782	0.117535282083923	0.731723082122246	0.949320237666556
Ddx58	Ddx58	0.0343807000506 15	1.67987830303847	0.117167471500196	0.732127124400482	0.949403956495176
Knop1	Knop1	0.016705486814 2565	3.83840664886992	0.117156032712444	0.732139707888942	0.949403956495176
Hspbap1	Hspbap1	0.0265920409580259	3.0215841008577	0.117083880223547	0.732219032542975	0.949403956495176
Fendrr	Fendrr	-0.0664447652309356	-0.295679182398113	0.117056158843781	0.732249518152177	0.949403956495176
Pomtd1	Pomtd1	-0.0106858343503005	6.49279950582025	0.116950266245624	0.732366003114652	0.949403956495176
Edem2	Edem2	0.0147302968360291	4.44274140630378	0.116932133814643	0.732385955226337	0.949403956495176
Col11a2	Col11a2	-0.0360491270408377	3.8152537349855	0.116806821 97796	0.732523889982047	0.949403956495176
Aifm2	Aifm2	-0.013294611056829	4.50213248896424	0.116784829633993	0.732548106184209	0.949403956495176
Tatdn3	Tatdn3	0.0280066167539088	3.2146009113091	0.116784372618071	0.73254860945043	0.949403956495176
Dpysl5	Dpysl5	0.0119658945181 4827	10.9625174585468	0.116532831175761	0.732825768589554	0.949403956495176
mrpl11	mrpl11	-0.0148946295605377	4.72594443925286	0.116457845719211	0.732908455391826	0.949403956495176
Fdx2	Fdx2	0.0173909444956158	4.51016839656217	0.116362007536615	0.733014179888259	0.949403956495176
Rcor2	Rcor2	0.0172636931657715	5.40996855862206	0.116359283031642	0.733017186122959	0.949403956495176
Zfp394	Zfp394	0.0179114986107402	3.55790817680578	0.116289859983695	0.733093800953574	0.949403956495176
Cep63	Cep63	-0.0240013418366214	3.66160504582966	0.116119246251216	0.733282201134775	0.949403956495176
Cdc42bp	Cdc42bpb	-0.00848365387121 76	8.67379498142302	0.116108229122169	0.733294372041163	0.949403956495176
Slc9a5	Slc9a5	-0.040937029417934	5.99019727292617	0.116099588839418	0.733303917635112	0.949403956495176
Ltbp1	Ltbp1	-0.0271377265904847	2.61398089227815	0.116074237086497	0.73332019279 6681	0.949403956495176
Brpf1	Brpf1	0.016286742293838	5.61727713659388	0.115994460049194	0.733420093375484	0.949403956495176
Irf2bpl	Irf2bpl	0.0104331514449688	7.68324143429227	0.115908725442495	0.733524880490699	0.949403956495176
Epb41l4	Epb41l4a	-0.0253904012854334	4.33805419651335	0.115901258393762	0.733523137818618	0.949403956495176
Gpat4	Gpat4	0.0069334204978716 1	7.12555790147606	0.115900113790758	0.733524403587543	0.949403956495176
Cradd	Cradd	0.041939448886378	0.951864478983039	0.115895029210641	0.733530026497836	0.949403956495176
Peli3	Peli3	-0.0250221740187233	2.72948592932192	0.115774984163892	0.733662821325761	0.949454841173997
Fdxr	Fdxr	-0.0164681903370935	3.96931517745819	0.115721930767208	0.733721523910389	0.949454841173997
Snrf3	Snrf3	0.011465988 7763288	8.37978493511854	0.115600183331765	0.733856313820414	0.949461218168689
Med21	Med21	-0.0230547148569093	3.69753461286093	0.115580020290821	0.73387865576812	0.949461218168689
Odf2	Odf2	-0.0160092180565904	6.05190847270817	0.115488297274894	0.733980067832389	0.949494225167933
Dtnb	Dtnb	-0.0162852667070652	5.27646476485334	0.115393515890332	0.734085315299582	0.949520168881224
RGD131	RGD131116	0.0119191085997388	4.83870577729564	0.115185677267668	0.734315834174697	0.949614430556569
Agtr2	Agtr2	-0.0932598475188383	-0.664393732620465	0.115107011858811	0.734403144395827	0.949614430556569
Dscaml1	Dscaml1	0.0198700493775388	6.08895926038043	0.115036263489358	0.734481695966042	0.949614430556569
Nek9	Nek9	0.0123105823846183	6.38403236586876	0.114856801989625	0.734681072083284	0.949614430556569
Epm2a	Epm2a	0.0270949488151445	2.82105439108414	0.114845017 68374	0.734694170175142	0.949614430556569
Agap1	Agap1	-0.0118017549310121	8.02363533627595	0.114781271014046	0.734765036558952	0.949614430556569
Arc	Arc	-0.0456188043706393	2.9790019581765	0.114767665741685	0.734780163105 18	0.949614430556569
Ccpg1os	Ccpg1os	0.0322297990296 85	1.83320114500772	0.114736339787065	0.734814999245083	0.949614430556569
Rangrf	Rangrf	-0.0207355361230 87	3.23914617100805	0.114719002195422	0.734834281239374	0.949614430556569
Ppox	Ppox	0.0136098708686813	4.62137388736879	0.114615842497981	0.734947921092999	0.949662938716586
Clec1a	Clec1a	0.0353209223542701	1.02312100988674	0.114474704097525	0.735106150109053	0.949718739160538
Csnk1e	Csnk1e	0.0096111728899 6035	7.76877727936241	0.114173987684403	0.735441248792034	0.949718739160538
Tmc7	Tmc7	0.0254797729909805	2.82427795067305	0.114137704706167	0.735481713395579	0.949718739160538
Znhit6	Znhit6	0.0123903800228427	5.55007749229347	0.114087601108938	0.735537603232802	0.949718739160538
Washc3	Washc3	0.0189194439936418	3.2146981713247	0.113976201890253	0.735661916454262	0.949718739160538
Chrm2	Chrm2	0.0262780404844966	3.26253603578158	0.113965642034614	0.735673703977042	0.949718739160538
Ripk2	Ripk2	0.0199019469206917	3.17065515703153	0.113879364554478	0.735770034697775	0.949718739160538
Mecr	Mecr	-0.0180987535490632	3.71243017829481	0.113791867167151	0.73586776899378	0.949718739160538
Fhl4	Fhl4	-0.0410996076926566	0.362977953819208	0.113767981055182	0.735872110738687	0.949718739160538
Ccdc22	Ccdc22	-0.0145815135786841	4.45568211081411	0.113687380869166	0.735984534692006	0.949718739160538
Chek1	Chek1	0.0218893674738778	3.00227427462657	0.113532801816014	0.736157389778331	0.949718739160538
Lsm7	Lsm7	0.0210798195039547	5.29442983115615	0.113517636122305	0.736174355582737	0.949718739160538
Arntl2	Arntl2	-0.0474707424945206	1.04728799153674	0.113429332659308	0.736273165415853	0.949718739160538
Ypel3	Ypel3	-0.017157229598339	6.44776274238951	0.113381210898083	0.736327020745075	0.949718739160538
Nuf2	Nuf2	0.0215641693229379	3.12663109433355	0.113361291743537	0.736349331072 67	0.949718739160538
Gmfg	Gmfg	0.0287929778395807	1.95390572468704	0.11322011282325	0.736507449340058	0.949718739160538
Lgmn	Lgmn	0.0190983248956014	5.49341560659471	0.113141091639221	0.736595899785449	0.949718739160538
Tmbim1	Tmbim1	0.0329568188556269	1.52558364369585	0.113137168325053	0.736600397113691	0.949718739160538
Tmem21	Tmem216	-0.0184930850 80484	3.65557214321813	0.113047149876159	0.736701314896966	0.949718739160538
Pdgfb	Pdgfb	0.0194210671819623	4.14030675937913	0.112764489146343	0.737018490871349	0.949718739160538
Hpse	Hpse	-0.0337369247215859 6	1.75705154073494	0.112680345478026	0.737112984613 68	0.949718739160538
Thumpd	Thumpd3	0.0120683746611351	5.40187898551064	0.112661564871729	0.737134092915445	0.949718739160538
Tmf1	Tmf1	-0.011247622941553	6.08396149355232	0.112464762587198	0.737355299022276	0.949718739160538
Tada2b	Tada2b	0.0082674326301717 1	6.03737946815686	0.112419379072 74	0.737406342100537	0.949718739160538
Rpp14	Rpp14	-0.0128594943878326	5.22906195700341	0.11235346039237	0.737480499972082	0.949718739160538
Sfswap	Sfswap	-0.0206290818392586	6.83290712574098	0.112186280850011	0.737668693898881	0.949718739160538
Arl6	Arl6	-0.0149076901675366	4.7606674977084	0.112166881465306	0.737690520711655	0.949718739160538
Aamp	Aamp	-0.0121048566073461	7.04539308077603	0.11209855587466	0.737767493001296	0.949718739160538
Pcdh15	Pcdh15	-0.0322785577840471	3.58547309091645	0.112095878216579	0.737770509659935	0.949718739160538
Spatc1l	Spatc1l	-0.0562030111639382	-0.211541876421507	0.112060934690945	0.737809880827839	0.949718739160538
Syndig1	Syndig1	0.019141409698586	4.92917229742007	0.111972181752937	0.737909889894575	0.949718739160538
Vps37c	Vps37c	0.0155962211341759	4.59427231534628	0.111863494041266	0.73803246724409	0.949718739160538
Kcnq2	Kcnq2	0.0157461297152763	7.56223595809391	0.111835988048583	0.738063493690417	0.949718739160538
Zfp707	Zfp707	0.0206133050644314	2.91448060031182	0.111834150047706	0.738065557085662	0.949718739160538
Myl12b	Myl12b	0.0112235828527189	8.00609101488435	0.111821470840141	0.738079870646457	0.949718739160538
Med16	Med16	-0.0098622769940355	7.09417369445574	0.111804627681686	0.738098873002877	0.949718739160538
Sash3	Sash3	0.061653815904 4689	-0.22471792274 2288	0.111742212621252	0.738169303200321	0.949718739160538
Plekhg5	Plekhg5	0.0177060582430524	4.82708283492565	0.111689689736238	0.738228587847284	0.949718739160538
Cdadc1	Cdadc1	-0.0149245402510476	4.34275523159401	0.111641922802086	0.738282517722	0.949718739160538
Fastkd1	Fastkd1	0.0211990190961317	4.49554795910423	0.111550788883193	0.738385445423432	0.949718739160538
Dagla	Dagla	-0.0129620016854835	6.8239664214874	0.111543214869997	0.738394001704624	0.949718739160538
Kcnab3	Kcnab3	0.028312963515283	2.17925737375895	0.111511877501101	0.738429406625207	0.949718739160538
Ddo	Ddo	0.0483649898877858	1.2764694007155	0.111486376762258	0.738458221409221	0.949718739160538
Ypel5	Ypel5	-0.0089023473859265	6.34805542182542	0.111465243357763	0.738482104061131	0.949718739160538
Lnc012	Lnc012	-0.0985073677469332	-0.721883084204791	0.111288549507049	0.738680751439821	0.949718739160538
Egfr	Egfr	0.0309799443951521	3.62468064837389	0.111205354479026	0.738776007882923	0.949718739160538
Ly6e	Ly6e	-0.0114585432925636	5.86588859473951	0.111148086015376	0.738840823106285	0.949718739160538
Cdc42ep	Cdc42ep3	0.0131013950861437	5.71780024356128	0.111080453679646	0.73891739148571	0.949718739160538
RGD130	RGD130296	-0.015149139234801	4.79556137832198	0.111078174802145	0.738919672266118	0.949718739160538
Eif1	Eif1	-0.0114292535489 48	8.49431999949919	0.110995470780807	0.739013640395662	0.949718739160538
Sox13	Sox13	-0.0199085480659017	3.89813942929545	0.110987651852952	0.739022497887896	0.949718739160538
Kcnq5	Kcnq5	-0.0127635621270935	5.99626854754732	0.110984051193377	0.739026576928684	0.949718739160538
Coro2a	Coro2a	-0.0144118517324693	4.40529625791943	0.110977646101539	0.739033833178419	0.949718739160538
Polr2d	Polr2d	-0.0165578459687243	4.19211206880516	0.110917958881108	0.739101463272327	0.949718739160538
Nupr2	Nupr2	0.0230162126183979	4.34335878293385	0.110700889984315	0.739347589236631	0.949825302952401
Sult2b1	Sult2b1	0.0171750293977262	4.66018108846887	0.110652522938729	0.739402457249078	0.949825302952401
Rnf6	Rnf6	-0.013610875374677	4.96191614158731	0.110636581450059	0.739420557632177	0.949825302952401
Fiz1	Fiz1	-0.0089591863268012 6	5.25405928209726	0.110545253422003	0.739524224484784	0.949870676229838
Napa	Napa	-0.0105304595567181	6.62061586578172	0.110434371519887	0.739650150967 11	0.949891804915904
Ankrd49	Ankrd49	-0.0117295239214613	4.49312305015087	0.110285361139681	0.739819489850581	0.949891804915904
Kcnc1	Kcnc1	-0.0209842717950317	5.39669004418074	0.110234486478916	0.739877324089735	0.949891804915904
Aar2	Aar2	0.012630632452 1777	5.48243564035167	0.110232860041645	0.739879163357921	0.949891804915904
Smarcal1	Smarcal1	-0.0140805925674584	3.92113985269206	0.110178452538964	0.739941060418906	0.949891804915904
Anxa11	Anxa11	0.0262534260465852	3.58429346566165	0.10986981554797	0.74017850298447	0.949891804915904
Ormdl3	Ormdl3	0.012138346568344	5.61001653534415	0.109855021617733	0.74030092520 0585	0.949891804915904
Tmem65	Tmem65	-0.0080587615941886	7.18047948089049	0.109816182632784	0.74035352634017	0.949891804915904
Ypel4	Ypel4	-0.0339568275659401	3.07834905 83409	0.109800586286013	0.740371279750061	0.949891804915904
Ralgds	Ralgds	0.0072463269408720	7.90158246969908	0.109761837382386	0.740392647623116	0.949891804915904
Bmk1	Bmk1	-0.0091817490676555	8.86451722939242	0.109727571622571	0.740454504983528	0.949891804915904
[illegible]	[illegible]	[illegible]	[illegible]	[illegible]	[illegible]	[illegible]

**Ubl7**	Ubl7	0.0135297396262532	6.32996722445529	0.105557284703039	0.745259921242572	0.951496483535381
**Mst1r**	Mst1r	-0.0359779384087496	1.57638452609716	0.105546539998466	0.745272446827309	0.951496483535381
**Sec5d**	Sec5d	-0.0456401473884545	2.47887908411404	0.105532035426819	0.745289343078567	0.951496483535381
**Ppp1ca**	Ppp1ca	-0.0126919802841503	7.86994550522695	0.105249589537635	0.745618618055726	0.951819489764814
**Rap2c**	Rap2c	-0.0092409151173014	6.20831300286957	0.105137143801791	0.745749843014975	0.951878100155035
**Slc25a17**	Slc25a17	-0.0098003595603392	5.55994859582998	0.104964040658569	0.745952007268012	0.951878100155035
**Dtymk**	Dtymk	0.0135346977307781	5.03886607628725	0.104923343088579	0.745999564013139	0.951878100155035
**Npas1**	Npas1	0.0175299782215206	5.51426006521277	0.104780193038181	0.746166821616147	0.951878100155035
**Pmpcb**	Pmpcb	0.0118356474680695	6.10980374577181	0.104723981080895	0.746232673894906	0.951878100155035
**Npbwr1**	Npbwr1	0.0510934762517096	0.110284804577991	0.104612224316469	0.746363456003866	0.951878100155035
**Inpp5b**	Inpp5b	-0.0190044708189023	5.89958603886347	0.104537909941058	0.746450464295562	0.951878100155035
**Cdc42**	Cdc42	-0.0100143216147476	8.38330141719052	0.104188591130295	0.746859909686773	0.951878100155035
**Tspy26**	Tspy26	0.0121898064652486	5.21958562274476	0.104137007580654	0.746920436502355	0.951878100155035
**Matn4**	Matn4	0.0309436985256267	3.12849096841587	0.103953677744641	0.747135694331181	0.951878100155035
[illegible]	[illegible]	[illegible]	[illegible]	[illegible]	[illegible]	[illegible]

Sptbn4	Sptbn4	-0.0166152514630463	7.4934439212114	0.0807327654947585	0.776306834978624	0.957553207828738
C1galt1	C1galt1	-0.0146634287642821	3.10928640120604	0.0806462810197033	0.776423493952974	0.957553207828738
Slc29a1	Slc29a1	-0.0218821031444258	5.18255364441663	0.0806130592113145	0.776458326060156	0.957553207828738
Fubp3	Fubp3	-0.0171793725901446	5.27645411147867	0.0805475718658784	0.776556725440227	0.957553207828738
Gtf2ird1	Gtf2ird1	-0.0125163383663326	5.71909825947158	0.0805011220596157	0.776619451026909	0.957553207828738
Ngly1	Ngly1	0.0108032458995237	4.72313065236754	0.0804694346765231	0.776652252731086	0.957553207828738
Apex2	Apex2	0.017256827614542	2.8583027682016	0.0804512343209254	0.776686840962018	0.957553207828738
Ubac1	Ubac1	0.0098829671216195	6.3228746181009	0.0804101178216543	0.776742399388331	0.957553207828738
Magoh	Magoh	-0.0128114023692861	4.59153825871906	0.0803866744375412	0.776774084013462	0.957553207828738
Ccn3	Ccn3	-0.02594824530997	4.7482750968579	0.0802070699977691	0.777016892917709	0.957553207828738
Mgme1	Mgme1	-0.0259448129994056	1.60285879644225	0.0802002875183396	0.777026021839663	0.957553207828738
Rab18	Rab18	-0.0073313022961015	7.2238711110662	0.0800332673356544	0.777252158873172	0.957553207828738
Tmem17	Tmem170a	-0.0291879590193482	1.40290885814076	0.0800243228849853	0.777264426983254	0.957553207828738
Vapb	Vapb	-0.0096532625297236	5.93534344387875	0.07994672595665	0.777369619080348	0.957553207828738
Reps1	Reps1	0.010441246034586	5.85058505127778	0.0799338825381852	0.777387031092073	0.957553207828738
Mtmr9	Mtmr9	0.0093060240940824	6.27208579456153	0.0798943494620622	0.777440637509681	0.957553207828738
Golga5	Golga5	-0.0106419691840873	5.03936607737533	0.0798909446540472	0.777445389359649	0.957553207828738
Adra1b	Adra1b	-0.0236143723752726	3.60360214541788	0.0798794589317566	0.777460820751645	0.957553207828738
LRRTM1	LRRTM1	0.0117794303537675	6.52590823419585	0.0798765540909209	0.777467483161875	0.957553207828738
Adcy8	Adcy8	-0.0185208072227916	4.81480496538564	0.0797423318087453	0.777646898741251	0.957553207828738
Glb1l2	Glb1l2	-0.0415505835055276	0.209362434739615	0.0797252243800273	0.777670123952029	0.957553207828738
Gemin7	Gemin7	0.0199818161671219	3.36518130580634	0.0796757788637024	0.777737266793445	0.957553207828738
Coq8a	Coq8a	0.0131685765535726	3.8208397746943	0.0796608900195253	0.777757488994461	0.957553207828738
Clba1	Clba1	0.0153653960509177	3.40989915324958	0.0796400627522047	0.777785780025434	0.957553207828738
Mmd	Mmd	-0.0087248216041041	8.57992140226714	0.0796381903270102	0.777788323907432	0.957553207828738
Ask1	Ask1	0.0113756561610808	7.65181094762938	0.0796289327904944	0.777800900564583	0.957553207828738
Pggt1b	Pggt1b	-0.0185648587186736	4.95678131538415	0.0796181610061666	0.777815525368974	0.957553207828738
Spata25	Spata25	-0.0241205318414706	1.49400707561835	0.0795176854388168	0.777952081798544	0.957553207828738
Fxyd1	Fxyd1	0.0281969853813803	2.26088625325797	0.0795118516583528	0.777960027159488	0.957553207828738
Tsc22d2	Tsc22d2	0.0113576000934753	5.20164055878586	0.0794461604083381	0.778049364442675	0.957553207828738
Hcrtr2	Hcrtr2	0.0197632856711906	2.26970806730368	0.0794083096711429	0.778100857519383	0.957553207828738
Cacna1b	Cacna1b	0.0199245634765243	7.77751285378479	0.0793225258111505	0.778217610385404	0.957553207828738
Klhdc2	Klhdc2	-0.0086188592674967	7.8111131759055	0.0792887405357803	0.778263611092315	0.957553207828738
Adam11	Adam11	0.0247191032024832	6.10212177852219	0.0792400299723539	0.778329852111507	0.957553207828738
Nabp2	Nabp2	0.010772403184781	6.51163170985812	0.079203032749053	0.778380353570232	0.957553207828738
Pnlip	Pnlip	-0.0479280951780685	1.12111951185274	0.0791121985951264	0.778504155903476	0.957553207828738
Fam234a	Fam234a	-0.0129500262802562	4.53293109749805	0.0791076852244572	0.778510309330149	0.957553207828738
Gsr	Gsr	-0.0095435539249651	5.82776442457521	0.0790200031733335	0.778629890645206	0.957553207828738
Jagn1	Jagn1	0.0119532440722061	5.0232701550337	0.0788493228170833	0.778862889952297	0.957553207828738
Slc30a10	Slc30a10	-0.012223794762848	4.94545306251449	0.0788073668390084	0.778920182998818	0.957553207828738
Tymp	Tymp	-0.0082377234909035	5.82144341794694	0.0787599185721888	0.778985017286042	0.957553207828738
Nmt1	Nmt1	-0.0081500404529100	6.27598966477009	0.0786937910927534	0.779075410338486	0.957553207828738
Katna1	Katna1	0.0157266020699489	3.85271647543414	0.0786739486186576	0.779102542087455	0.957553207828738
Josd2	Josd2	0.0174940521993621	3.44231960742418	0.0786251916041323	0.779169225906255	0.957553207828738
Ptpn1	Ptpn1	0.0093882845610320	5.74585374482566	0.078605814660051	0.779195723488279	0.957553207828738
Ddb2	Ddb2	-0.0187152368950037	2.62817184328971	0.0785850342692953	0.779224164896116	0.957553207828738
Olfm4	Olfm4	-0.0358834120757636	1.36412409205048	0.0784975853109557	0.779343855656435	0.957553207828738
Plp2	Plp2	0.0309018092176899	1.41459635631943	0.0784576462454783	0.779384849561386	0.957553207828738
Rabl2	Rabl2	0.0127054591562781	4.50891768555015	0.0783685662984297	0.779520574421878	0.957553207828738
LOC100	LOC100912	0.0162462713436396	4.4604300517596	0.0783447014459426	0.779553279561621	0.957553207828738
Shisa5	Shisa5	-0.0127019986612181	5.08800976461529	0.0782832657446733	0.779637497687207	0.957553207828738
Hs2st1	Hs2st1	0.0063024949687331	7.2223830888067	0.0782432266354789	0.779692403825472	0.957553207828738
Cdk18	Cdk18	0.035614067122821	1.65509501403574	0.0782041308977597	0.779746030924909	0.957553207828738
Tmem18	Tmem181	0.0076775298560518	5.95522588217412	0.0781569968587377	0.779810703273848	0.957553207828738
Dync1li1	Dync1li1	0.0123903177634161	8.38256235935297	0.0780585436782673	0.779945858185178	0.957824923496996
Nelfa	Nelfa	0.0089616747548910	5.14112130376068	0.0779127925035894	0.780146111820227	0.957776546018004
Mtpap	Mtpap	-0.0139177479238952	4.49872757505717	0.077785660366338	0.780320948890505	0.957890938162958
Srsf11	Srsf11	0.0106210653776487	5.14073973837215	0.0777246058443666	0.780404968205781	0.957905832582575
Hnrnpa1	Hnrnpa1	-0.0111928441137146	10.1246618651268	0.0774490767597626	0.780784576367341	0.958169415016876
Invs	Invs	0.031943226757626	3.75935799928893	0.0774113196030157	0.780836853008493	0.958169415016876
Npffr1	Npffr1	0.0398136770540194	0.667366480259428	0.0774015766623251	0.780850093122674	0.958169415016876
Stard3	Stard3	-0.0085088803492631	5.74276766716427	0.0772398808759149	0.781073281142449	0.958277397461234
Fndc3b	Fndc3b	0.0201431390118299	4.35052331514624	0.0771903224448778	0.781141736816373	0.958277397461234
Mrps28	Mrps28	-0.0190425736569037	2.95026796561789	0.0771709498825928	0.781168502811143	0.958277397461234
Actg1	Actg1	0.0110485487015271	11.8317415091649	0.0769932405237341	0.781414203018268	0.958364027871096
Polr2h	Polr2h	0.0119311453284748	4.48729221987534	0.0769880843347792	0.781421326499978	0.958364027871096
Mpp3	Mpp3	0.0100524942166301	6.22462529480126	0.0769323811234281	0.781498403318432	0.958364027871096
Syt7	Syt7	0.0122357377866407	6.87831036625764	0.0768793894947298	0.781571802574042	0.958364027871096
Cdc25a	Cdc25a	-0.014046747647371	4.53157931427788	0.0768387719507331	0.781628018819268	0.958364027871096
Itgb3bp	Itgb3bp	-0.0194304021889012	2.79310747562546	0.0767868185149894	0.781699984903754	0.958364027871096
Eef1a1	Eef1a1	-0.0144225023961769	12.1486045410014	0.0766683427820125	0.781864194038144	0.958393109042893
Hadha	Hadha	0.0071194480756568	6.75630278476423	0.0766179758254495	0.781934047825725	0.958393109042893
Cntnap4	Cntnap4	0.0154558158557835	5.7908989752152	0.0766034887517151	0.781954143628809	0.958393109042893
Mutyh	Mutyh	0.0319123073253742	4.45225333371422	0.0761899568251465	0.782528647325033	0.958868262915731
Irf7	Irf7	0.0568852918808882	0.0782256975465378	0.076134639496388	0.782605624650989	0.958868262915731
Pten	Pten	-0.0107715881241701	6.37182341681405	0.0761153564978868	0.782632467938243	0.958868262915731
Vkorc1l1	Vkorc1l1	-0.0109575323754192	4.61205220785065	0.0761033165397293	0.782649225738063	0.958868262915731
Hirip3	Hirip3	0.0123659053562524	4.97422809383146	0.0758881157086672	0.782949043464644	0.959105059646445
Ttc33	Ttc33	0.0114924870251594	4.37675571785673	0.0758433225851 7	0.783011492757813	0.959105059646445
Tsg101	Tsg101	0.0076803177598603	7.03001073022185	0.0757679741579125	0.783116623608234	0.959105059646445
Cask	Cask	-0.0100531676921106	7.47858584239192	0.0757433371212315	0.783151006379274	0.959105059646445
Ext1	Ext1	-0.0255546527914192	2.61516861819709	0.0756880091320076	0.783226818366297	0.959105059646445
Mrps33	Mrps33	0.0165655311453779	4.64677264703621	0.0755541287079495	0.7834152611619	0.959210843512959
Trdmt1	Trdmt1	-0.0306684210212091	3.57435346488768	0.0755171161274717	0.783466865771465	0.959210843512959
Scfd1	Scfd1	-0.0100744597169842	5.51901126008723	0.0754577292253416	0.783550092754462	0.959218382915576
Tug1	Tug1	0.0099398236825352	6.96654371867035	0.0754001702472635	0.783630547408832	0.959222832318317
Ccdc114	Ccdc114	0.028440509800441	2.25949013726349	0.0753391719181825	0.783715809172189	0.959223215485655
Chm	Chm	0.0102109840797725	4.65633212504994	0.0751007646055868	0.784049896048396	0.959547871799493
Prlhr	Prlhr	-0.0521907465862892	0.0216390927 2917	0.0749493543908812	0.784262303936213	0.959697411938754
Pak1ip1	Pak1ip1	0.0110262032385001	4.40011741755053	0.0747945568941209	0.784479702140481	0.959697411938754
Klc2	Klc2	-0.0073219570870356	7.81389756650655	0.0747416825148556	0.784554000648884	0.959697411938754
Cdca8	Cdca8	-0.0181474332196492	3.47385595321846	0.0747362223321402	0.784561688951636	0.959697411938754
Hsf1	Hsf1	0.0182354542168831	5.16829695418285	0.0746459792384916	0.784688592983325	0.959697411938754
Tmem59	Tmem59	-0.0098933592959814	7.05954409419363	0.0745280204902059	0.784854594924733	0.959697411938754
Ppp2r3b	Ppp2r3b	-0.0132980880444101	5.17509409139626	0.0745192765421142	0.784866805451566	0.959697411938754
Spag4	Spag4	-0.0299206152800846	4.04580855482854	0.0745069464096275	0.784884266709067	0.959697411938754
Slc4a1ap	Slc4a1ap	-0.0082118712255725	5.66839241445126	0.0743862085455191	0.785054351450781	0.959697411938754
Phka1	Phka1	0.0105154698899696	4.92493205584221	0.074362100632051	0.785088330289161	0.959697411938754
Cthrc1	Cthrc1	0.0232521718176409	2.40233880973668	0.0742925344149015	0.785186413407484	0.959697411938754
Rab11fip	Rab11fip4	0.0101578575152877	7.1771942582325	0.0742737889603103	0.785212879730828	0.959697411938754
Sh2b1	Sh2b1	0.0064152086143469	6.91221032766642	0.0742403822647814	0.785260004580628	0.959697411938754
Ccdc174	Ccdc174	-0.0109539611356447	4.53323017765588	0.0742247853155931	0.785281981955282	0.959697411938754
Arl6ip5	Arl6ip5	0.0144469060825238	5.78523886687593	0.0741937012187454	0.785325845627529	0.959697411938754
Eif1ax	Eif1ax	-0.0075017512000295	7.72827149556639	0.0741373882118737	0.785405335692263	0.959700555620011
Dlgap1	Dlgap1	0.0077282865581598	7.69930739510001	0.0739748946452039	0.785634889594448	0.959880546417967
Actr2	Actr2	0.0066187451233460	8.08907885975012	0.0739242422020112	0.785706501413238	0.959880546417967
Ankrd55	Ankrd55	-0.0162026876180826	3.59264596250043	0.073711566368596	0.786007467884027	0.959912950272096
Stam	Stam	0.0062667586485027	6.78285182582586	0.073642982431374	0.786104809105861	0.959912950272096
Cfap77	Cfap77	0.0236148946722108	1.36391036852885	0.0736004215825972	0.786164809017304	0.959912950272096
Tll1	Tll1	0.0316419305715733	3.12958565058752	0.0733404584541404	0.786532756734267	0.959912950272096
Ndel1	Ndel1	-0.0066548348759170	6.26848570411421	0.0732962422203158	0.786596557450995	0.959912950272096
Dcun1d1	Dcun1d1	0.0107436460507885	4.79088740207644	0.0732864153126 3	0.786610517457955	0.959912950272096
Nufip1	Nufip1	-0.0080292708583635	5.53854443506266	0.0732471390930982	0.786666322886571	0.959912950272096
Wdpcp	Wdpcp	0.0196721648759212	2.96789035912365	0.073222041396681	0.786701891255588	0.959912950272096
Dennd2c	Dennd2c	0.0241211759055348	2.13142011941626	0.0732169157436431	0.786709276539186	0.959912950272096
Ate1	Ate1	-0.0077782704671752	6.34477379528679	0.0732009823529864	0.786731925019326	0.959912950272096
Pcdhb3	Pcdhb3	0.018582740642701	3.05782627217659	0.0731920292 4673	0.786744853973335	0.959912950272096
Snapin	Snapin	0.0090313415809851	5.44496252486357	0.0731539467214534	0.786798941342649	0.959912950272096
Nsg2	Nsg2	0.0091967750298228	10.7731413539601	0.0731150601620669	0.786854104532501	0.959912950272096
Alkbh6	Alkbh6	0.0128760034921821	4.51192697505891	0.0730832746610162	0.786899322353739	0.959912950272096
Usp40	Usp40	0.0254783930235699	4.99979972356572	0.0730205215727011	0.786988625428008	0.959912950272096
Cd109	Cd109	-0.0282893732075582	1.05206772542311	0.0730119575015067	0.787000816034957	0.959912950272096
Ppp1r35	Ppp1r35	0.0179544127453043	3.39412533541181	0.0728716073266042	0.787200708205667	0.959912950272096
Sde2	Sde2	0.0097716143291934	5.50919533480818	0.0728538577887737	0.787226002491553	0.959912950272096
Bloc1s1	Bloc1s1	-0.0147937515060376	5.17056094349262	0.0728345636326395	0.787253501715488	0.959912950272096
Elmod1	Elmod1	-0.0105365150265842	7.99632135239925	0.0728217864329606	0.787271717571 08	0.959912950272096
Csf2rb	Csf2rb	0.0631041000901184	-0.225335251280594	0.0726581905716444	0.787505060821114	0.960103639238556
Cinp	Cinp	0.0104795495823065	4.96520019938771	0.0725340992565933	0.787682247334546	0.960225832925562
Rasgrf2	Rasgrf2	0.0160302257963447	5.27807221711873	0.0724768995386	0.787763875945744	0.960221645943244
Hnrnpab	Hnrnpab	0.01081733933395	8.89210100887735	0.0723187442214197	0.787990133028673	0.960413489298848
Pde6a	Pde6a	-0.0166257715401511	5.26144947352398	0.0721091005070775	0.788290325983565	0.960685524264672
Acap1	Acap1	-0.0470497365157303	1.31493101065628	0.0720150430573199	0.788425160744 52	0.960749057371277
Cast	Cast	-0.0152421208686068	4.87192294544181	0.0719653321274752	0.788496461287319	0.960749057371277
Plekhd1	Plekhd1	-0.0411687887802211	1.64612713189202	0.0718682129357913	0.788635692292543	0.960824873814477
Bccip	Bccip	-0.0099681986551650	4.22907181905434	0.0718011750885736	0.788732099943037	0.960820426351158
Adra1a	Adra1a	-0.0406853933889206	1.03057262412594	0.0716053862812345	0.789013522140589	0.960820426351158
Ckb	Ckb	-0.0108780874942996	9.48515675648457	0.0715446521209147	0.789101092128755	0.960820426351158
Nuak2	Nuak2	0.0215270814621729	2.17769162651293	0.0715283849135488	0.789124315035561	0.960820426351158
Dcaf6	Dcaf6	-0.0059734747738765	6.48721555136396	0.0715129054300714	0.789146552115608	0.960820426351158
Ormdl2	Ormdl2	0.0106343183531506	3.75799646705383	0.0714879767915022	0.789182481553818	0.960820426351158
Calr3	Calr3	0.0318501082605298	0.807240619497567	0.071465071590417	0.789215460861424	0.960820426351158
Bysl	Bysl	0.011749631190653	5.07800234243469	0.0714367026597813	0.789256314793 06	0.960820426351158
Erg28	Erg28	-0.0100878901508636	5.38180002896137	0.0712980878846423	0.789456058216082	0.960966240294536
Irak1bp1	Irak1bp1	-0.0115613317905426	4.93825232959799	0.0712391221472651	0.789541089264226	0.960966240294536
Rida	Rida	0.0110348271886925	3.908456857988	0.0711990258015707	0.789598893677934	0.960966240294536
LOC688	LOC688613	-0.0116331344452937	4.66551663241519	0.0711137119361211	0.789722065956668	0.960969194 08364
Hacl1	Hacl1	-0.0151150822260366	3.73112285803265	0.0710906258250947	0.789755399225266	0.960969194 08364
Egr2	Egr2	-0.0470264268535685	1.11730104141979	0.0709571185122542	0.789948280774263	0.961110160821 11
Xpa	Xpa	-0.017156173293781	3.20307148416024	0.0709028816681565	0.790026679164571	0.961111824876789
Mettl26	Mettl26	0.0150391992562222	3.34689971767121	0.0707526738620672	0.790244024171062	0.961275034602951
Prkab1	Prkab1	-0.0100942645528469	5.2317119847378	0.070703708503089	0.790314823856462	0.961275034602951
Sbno2	Sbno2	0.0155851657254213	5.50369972857369	0.0706363051836547	0.790412563908002	0.961300082817 15
Kcnj10	Kcnj10	-0.0215160791926474	2.96108192497713	0.0704938418211913	0.790619099592897	0.961457554154053
Rnf5	Rnf5	-0.0108523426859882	6.6104253370268	0.0703808099822344	0.790783126303295	0.961502250115068
Tut1	Tut1	0.0096321394612313	4.83978174236094	0.0703623158066335	0.790809977613275	0.961502250115068
Nedd4	Nedd4	0.0076135232505569	10.1140017218595	0.0703032523612421	0.790895755902173	0.961512946789114
Zhx1	Zhx1	0.0077218568648022	6.9141924271383	0.0702432825166233	0.790982730165665	0.961516047158 51
Pdgfa	Pdgfa	0.0090723490328734	5.67730407017384	0.0701443062527645	0.791126786253691	0.961516047158 51
Flcn	Flcn	-0.0097214458588935	5.72923079073839	0.0700727548851461	0.791230878579769	0.961516047158 51
Plaat1	Plaat1	-0.0512307413759197	-0.155224886484842	0.0699791256020319	0.791367175604402	0.961516047158 51
Tomm70	Tomm70	0.0074508169013918	7.72407008431482	0.0698640695921461	0.791534798846371	0.961516047158 51
Slc1a6	Slc1a6	0.0134457904441367	4.67346540013759	0.069839409294281	0.791570744951 48	0.961516047158 51
Tmem13	Tmem134	0.0125301881129177	4.92937201262772	0.0697486366316724	0.791703116801454	0.961516047158 51
Gbx1	Gbx1	-0.115787318424374	0.434215339240879	0.0697464326960215	0.791706332935324	0.961516047158 51
Ncstn	Ncstn	-0.0064016925455834	6.64538328959754	0.0697052758134132	0.791766236259967	0.961516047158 51
Ttll1	Ttll1	-0.0080729983263062	6.1577057453867	0.0696959895953597	0.791779923935 62	0.961516047158 51
Arl10	Arl10	-0.0109546563652081	5.26159823535035	0.0696731820086285	0.791813222052323	0.961516047158 51
Mindy1	Mindy1	0.0119112953494362	3.61908736110865	0.0696638651550927	0.791826821858007	0.961516047158 51
Zhx3	Zhx3	-0.011714598288967	6.01835853537562	0.0696136015276068	0.791900208431582	0.961516047158 51
Phactr3	Phactr3	-0.0072042846005126	6.77383784921451	0.0693387823186598	0.792301955583887	0.961780220389 76
Gipc1	Gipc1	-0.0088067022242205	6.42834738321088	0.0693093628408033	0.792345011538553	0.961780220389 76
Caskin2	Caskin2	-0.0102126182101591	4.99387332706681	0.0692936674640734	0.792367988481589	0.961780220389 76
Tsc22d1	Tsc22d1	-0.0073118271129193	9.481464274607	0.0692221266849202	0.792472747141727	0.961780220389 76
Srsf1	Srsf1	0.0079175702678626	8.53667523051417	0.0692012364597628	0.792503201557035	0.961780220389 76
Mier3	Mier3	0.0090822467132483	5.56326051230121	0.0690757943264728	0.792687205100375	0.961909964796477
Tmem23	Tmem231	0.012481928705984	4.2241865887493	0.0690035963384119	0.792792104613682	0.961944915517 35
Pawr	Pawr	0.0236481534540917	2.00728997516477	0.0688250002154405	0.793055322886652	0.962169512230798
Rtel1	Rtel1	-0.0218978603623803	6.52202393629939	0.0686092319458087	0.793372803529835	0.962234574687097
Mettl3	Mettl3	0.0110566179657434	5.71298885588807	0.0685989614581378	0.793387719270499	0.962234574687097
Foxj1	Foxj1	-0.0328472867082867	4.43291191601365	0.0684952410030348	0.793540439003 27	0.962234574687097
Irf3	Irf3	-0.0247752530819991	5.24952844145505	0.0684916064924506	0.793545795703229	0.962234574687097
Vrk1	Vrk1	-0.0112919858891864	3.99872698452647	0.068470355438329	0.793577099299386	0.962234574687097
Apba1	Apba1	-0.0096543548568935	8.4174413850503	0.0683066829674495	0.793818390315616	0.962523727489014
Tmem18	Tmem184c	-0.0089545658966040	5.59478322042551	0.0681138125105286	0.794103124365196	0.962615947451334
Ciao2b	Ciao2b	-0.013492255445296	4.73597992235281	0.0680972456204358	0.794127603561589	0.962615947451334
Gpr180	Gpr180	0.0092755183029435	4.81554828459108	0.068071852181923	0.794165128753858	0.962615947451334
Exosc4	Exosc4	-0.0179834004686103	4.12121818561261	0.0680517747622247	0.794194803483533	0.962615947451334
Speg	Speg	0.0105423035538889	6.89508673903143	0.0678871768655114	0.794438258140273	0.962689763138716
Arhgap9	Arhgap9	0.0388237758003386	0.0780076256624114	0.0677202333216087	0.7946855043712	0.962689763138716
Usp24	Usp24	0.0176529616103865	7.57732534630486	0.0676686270010407	0.794752000039427	0.962689763138716
Otud6b	Otud6b	-0.0076875175085322	5.15674790508505	0.0676465692461469	0.794794707069879	0.962689763138716
Rnf208	Rnf208	-0.0082345168581485	6.87868119923386	0.0676117062298687	0.794846409406015	0.962689763138716
Lnc-hc	Lnc-hc	0.0218550196537112	2.19327734156436	0.0675716186711597	0.794905879109435	0.962689763138716
Ino80e	Ino80e	-0.0070268049640935	5.64843535082369	0.0675560186186104	0.794929026803686	0.962689763138716
Smu1	Smu1	0.0084362382693961	5.99543338072907	0.0675143798221711	0.794990825322238	0.962689763138716
Eif3j	Eif3j	-0.0080703117137506	6.23886552386763	0.0674700717529414	0.795056607808148	0.962689763138716
RGD130	RGD130677	0.015357137564612	2.91604722938279	0.067467557693436	0.795060341040 03	0.962689763138716
Cfap206	Cfap206	-0.060981840604957	0.441698726552143	0.0674379687144781	0.795104432888071	0.962689763138716
Brpf3	Brpf3	-0.0117752109340406	4.99304375471416	0.0672613893524724	0.795366770733605	0.962701170053404
Alg6	Alg6	0.0145032127453922	3.22540079119806	0.0672450749828046	0.795391008185772	0.962701170053404
Lrp2	Lrp2	0.0320016227800264	3.37391435979783	0.0672344942230296	0.795406748494287	0.962701170053404
Cpsf1	Cpsf1	-0.0096965422632991	6.75674542454559	0.0672239158961379	0.795422486341311	0.962701170053404
Sidt1	Sidt1	0.0170452704401033	5.69484192506579	0.0670376407311153	0.795699825760307	0.962721870702825
Hspa8	Hspa8	-0.0095094017217470	11.0168534938505	0.0669501763285112	0.795830205327528	0.962721870702825
Dnajc30	Dnajc30	0.0145828095723537	4.26417146725755	0.0669438141201155	0.795839690325112	0.962721870702825
Hlf	Hlf	0.0241232990165705	2.16855559978878	0.0669432045213441	0.795840601023918	0.962721870702825
Rpusd1	Rpusd1	-0.0120974031319898	4.42014318418819	0.0669291797426794	0.795861515190 83	0.962721870702825
Dcun1d2	Dcun1d2	-0.012544898649645	3.61222378413478	0.066901667945757	0.795902548393014	0.962721870702825
Rgs2	Rgs2	0.0200919988076875	4.99576982805395	0.0668401267765972	0.795994368208164	0.962739821796555
Deup1	Deup1	0.042005021543004	0.575183481914484	0.0667094579309691	0.796189476595632	0.962882242850024
Slitrk3	Slitrk3	-0.0142264176366085	4.97458175334983	0.0664011715535935	0.796650604104442	0.963217957908457
Zfp639	Zfp639	-0.0093002045164054	4.57380289198808	0.0663204266480406	0.796771568988584	0.963217957908457
Decr2	Decr2	0.0205810958557116	2.55210442012794	0.0663005483367693	0.796796864516238	0.963217957908457
Rsph10b	Rsph10b	-0.0225927124342596	3.34834023729731	0.0661986622664754	0.796954132733716	0.963217957908457
Olfml1	Olfml1	0.0670340619799271	1.91189712532357	0.0661780287052096	0.796985088526124	0.963217957908457
Tpp2	Tpp2	0.0074481822160555	7.11777904206223	0.0660045251774264	0.797245580809902	0.963217957908457
Fig4	Fig4	0.0073269649889279	6.06897963139707	0.0659830894471156	0.797277789004 86	0.963217957908457
Rflna	Rflna	0.0204913474699207	1.60229237277142	0.0659586454792844	0.797314524024981	0.963217957908457
Scaf1	Scaf1	-0.0055646346823072	8.21107400406889	0.0659551828686242	0.797319756718 54	0.963217957908457
Clcn2	Clcn2	-0.0178449956354516	6.3268997535561	0.065789119539815	0.797569491837112	0.963217957908457
Unc13c	Unc13c	-0.0253098809873	4.5776386231769	0.0656455649232441	0.797785672558578	0.963217957908457
Atm	Atm	0.0096754058961150	8.11826641292549	0.0656219815530514	0.797821196066662	0.963217957908457
Cd2ap	Cd2ap	-0.0219148885970742	3.44398829515768	0.0656118051305725	0.797836548434707	0.963217957908457
Slc18a3	Slc18a3	0.0510089224135155	1.97601238796032	0.065598560842749	0.797901737788005	0.963217957908457
Fam174a	Fam174a	0.0129149482518604	4.85856558269854	0.0655651157740351	0.797906882101716	0.963217957908457
Osbpl1a	Osbpl1a	0.0115370366146178	7.078878434631	0.0655452526580583	0.797936882108863	0.963217957908457
Rsrc2	Rsrc2	0.0073137194197292	6.43318547999048	0.0655375256110347	0.797948536507 46	0.963217957908457
Odf2l	Odf2l	-0.024297142063783	3.12970355982977	0.0655332063286096	0.797955050585837	0.963217957908457
Fcgr3a	Fcgr3a	0.0428052248960082	0.0221385509442319	0.0655264225826642	0.797965281879578	0.963217957908457
Stat5b	Stat5b	-0.0173403530137964	4.59018304867603	0.0655073415297807	0.797994063085147	0.963217957908457
Cyp4f5	Cyp4f5	0.0339684944684784	1.17186140479598	0.0654659824889592	0.798056462979545	0.963217957908457
Rps6ka2	Rps6ka2	0.0110196368708397	6.92492412444013	0.0651054485009155	0.798601305525705	0.963499635051906
Dlc1	Dlc1	-0.01074625763595	7.24113244456556	0.0651019625174598	0.798606581412188	0.963499635051906
Rasef	Rasef	-0.0415358562762085	-0.292821165579844	0.0650417551109257	0.798697726489396	0.963499635051906
Sertm1	Sertm1	-0.0202796771587942	6.81192407097719	0.0650087794889013	0.798747665728806	0.963499635051906
Sfi1	Sfi1	0.0210167967368923	4.95446590548274	0.0649998967821261	0.798761120265672	0.963499635051906
Smoc2	Smoc2	0.034276435628282	1.21918312875536	0.0649718829696618	0.798803557369637	0.963499635051906
Mat2b	Mat2b	0.0106495645850304	5.0883583808614	0.0649184640688745	0.798884511133336	0.963499635051906
Lsm3	Lsm3	-0.0140052452795615	4.32852393682069	0.0648368620463344	0.799008241245561	0.963499635051906
Ide	Ide	0.0083969168765949	6.09992866571625	0.0648213192790084	0.799016648543886	0.963499635051906
Dync2li1	Dync2li1	-0.0129046785410967	5.09416887992045	0.0647475511873239	0.799143755045 85	0.963512524575 4
Rtn2	Rtn2	-0.0079259661691386	7.72606452635094	0.0647119092462773	0.799197863314499	0.963512524575 4
Dclre1c	Dclre1c	0.0273064546390843	2.19523419050578	0.0645716455942311	0.799259006921166	0.963512524575 4
Adgrb3	Adgrb3	-0.0071613617982694	7.4585948210266	0.0645880341769924	0.799386042374885	0.963572567936571
Serpinb9	Serpinb9	0.018823632760006	2.97761879008695	0.0644890056436012	0.799536615184906	0.963660968681109
Tmem17	Tmem177	0.0192430988253413	2.58194005686052	0.0641595845328524	0.800038387288133	0.964172602935771
Toe1	Toe1	-0.0117371858289563	3.97715141647746	0.0640618214786812	0.800187563002971	0.964258245082873
Arhgap2	Arhgap24	0.0229755809581488	2.77763042949764	0.0639659192350983	0.800332489332 66	0.964340379548108
Mttp	Mttp	-0.0308986755754866	0.915174279097767	0.0638439183417461	0.800520495742417	0.964340379548108
Eif2s3y	Eif2s3y	-0.107572599770905	5.21592461935668	0.063837982351182	0.800529755746838	0.964340379548108
Csf2ra	Csf2ra	-0.022264747650125	3.88742584772704	0.0637463878411353	0.800669707748356	0.964340379548108
Gpx2	Gpx2	0.0538191669437479	0.121296064217434	0.0637335312479337	0.800689386118298	0.964340379548108
Mkks	Mkks	0.0103318531978859	5.05113745385194	0.0637118347088972	0.800722599744851	0.964340379548108
Prkar1a	Prkar1a	-0.0061998438030241	8.34200722587627	0.0636082822218782	0.800881203517855	0.964340379548108
Kif21a	Kif21a	0.014128099627541	7.38909530666052	0.0635552475286829	0.800962488053342	0.964340379548108
Vpreb3	Vpreb3	-0.0322369546576477	0.367453897296284	0.0634393262195232	0.801140276141405	0.964340379548108
Zdhhc7	Zdhhc7	-0.0082572500384026	5.50732917880016	0.0633377708142575	0.801296175056 77	0.964340379548108
Prph	Prph	0.0272145979975105	3.55664808397911	0.0632760210672814	0.801391032810031	0.964340379548108
Scgn	Scgn	-0.032382155796817	1.32020677982851	0.0631621223567188	0.801566127590553	0.964340379548108
Calu	Calu	0.0086840726178896	7.25324439965992	0.0630887924103334	0.801678967977561	0.964340379548108
Lamp2	Lamp2	0.0118401854609221	5.45282185949753	0.0630723223489724	0.801704296914671	0.964340379548108
Polr2g	Polr2g	0.0113605912731911	5.08322007956092	0.0630712474389091	0.801705851425363	0.964340379548108
Dtwd1	Dtwd1	0.0160237874200711	2.64640064585752	0.0630180974858163	0.801787778829135	0.964340379548108
Apbb3	Apbb3	-0.0161788844331765	5.8170361449328	0.0629950905057513	0.801823210772584	0.964340379548108
Cyp27a1	Cyp27a1	-0.0506901578309005	0.0368872871914935	0.0629552031801808	0.801884655626463	0.964340379548108
Bmyc	Bmyc	0.012709765416553	5.87621908577232	0.062887762750922	0.801988590493583	0.964340379548108
Chd5	Chd5	-0.0150852333657005	8.42048680416033	0.0628379561272965	0.802065389982876	0.964340379548108
Fibin	Fibin	0.0358795890671896	1.29715830506266	0.0628175557790627	0.802096855074469	0.964340379548108
Nr2c1	Nr2c1	0.0148552079980613	4.15874904537984	0.0627964983466427	0.802129328335522	0.964340379548108
Dhx40	Dhx40	-0.0086273814587577	5.36893739226401	0.0627098090000899	0.802262121712595	0.964340379548108
Eapp	Eapp	-0.0099122359184931	4.7314785587541	0.0626845592517461	0.802302121043736	0.964340379548108
Golga7b	Golga7b	-0.0088033034244404	5.80826487079558	0.0626391801142682	0.802372194329197	0.964340379548108
Gpd1l	Gpd1l	0.0078374175022778	6.22168848931368	0.0626380479973108	0.802373858732065	0.964340379548108
Taf12	Taf12	-0.010716339321964	3.68821676152883	0.0625890104472475	0.802449730689523	0.964340379548108
Npl	Npl	0.0162096885465327	2.72976491191757	0.0625761389988284	0.802469624624 94	0.964340379548108
Ppm1l	Ppm1l	0.0121510862492341	7.03824919485062	0.0625267415959456	0.802545860943681	0.964340379548108
Tmem26	Tmem265	0.0204239488566775	3.215460168364	0.0623331989528424	0.802845518349082	0.964340379548108
Marchf5	Marchf5	-0.0059684423410726	6.68227408619979	0.0623280912124216	0.802853429745495	0.964340379548108
Whrn	Whrn	0.0107362769000115	5.2432760179894	0.0623247723984922	0.802858570450635	0.964340379548108
Gpr17	Gpr17	-0.0186309939469691	4.54723153327991	0.0622938892237653	0.802906445184747	0.964340379548108
Dnpep	Dnpep	0.0075145851928894	7.15763269253496	0.0621938360791268	0.803061496472195	0.964340379548108
Eci2	Eci2	0.0106399707598766	4.82814212272836	0.0621820374210458	0.8030797988231	0.964340379548108
Adrp	Adrp	-0.0380217920147025	0.180599937499008	0.0621305830612755	0.803159615312952	0.964340379548108
Zfp518a	Zfp518a	0.0180111804483937	3.9109303977175	0.0620735328304446	0.803248151521385	0.964340379548108
Dusp6	Dusp6	0.0093647130521722	5.24392212678098	0.0620572977924638	0.803273356729 46	0.964340379548108
Pym1	Pym1	-0.0095072588333252	5.07074698564289	0.0620002827549442	0.803361895493653	0.964340379548108
Pdia2	Pdia2	0.0411345358637437	2.46559672381876	0.0620000456049473	0.803362263854775	0.964340379548108
Armcx2	Armcx2	-0.0071526755852115	7.61701953029452	0.0619604618600489	0.803423759349 41	0.964340379548108
Atrip	Atrip	0.0182894685658277	4.86933126985473	0.0618811913563206	0.803546972330872	0.964395495745 7
Adk	Adk	0.0115760337776501	5.50331620032893	0.0617615278122862	0.803732002250541	0.964425733910459
Pygm	Pygm	0.028456214014973	2.70072867488577	0.0617447214733033	0.803759089288927	0.964425733910459
Ptov1	Ptov1	-0.0091449290512890	8.74426597008647	0.0616858144347958	0.803851012586257	0.964425733910459
Gal3st4	Gal3st4	0.0211129426053881	1.84758167452154	0.0616408603554177	0.803921061218608	0.964425733910459
Gzf1	Gzf1	0.0095286526482322	4.65016489071499	0.0616112768980711	0.803966984724109	0.964425733910459
Crnde	Crnde	-0.0622873650424947	-0.594227255067913	0.0613275686306594	0.804409825835681	0.964627512259021
Tmem9	Tmem9	0.0095307555219195	6.09470240172661	0.0613223703457 6075	0.804415874490098	0.964627512259021
Uhrf1bp	Uhrf1bp1l	-0.0076003169677794	6.58935473626071	0.0613037970375245	0.804446978071481	0.964627512259021
Gnb1	Gnb1	-0.0072086759232572	10.1549233430536	0.0612938070872495	0.804462589280312	0.964627512259021
Tlcd4	Tlcd4	-0.0156309469907086	3.24043502026201	0.0612192658517685	0.804579116870909	0.964627512259021
Fth1	Fth1	-0.0099062894059277	9.37144650327178	0.0612118406480667	0.804590728546063	0.964627512259021
Acsl1	Acsl1	-0.0063799947610814	6.26654760158771	0.0610971593434783	0.804770113078887	0.964664965559308
Adam15	Adam15	-0.0082344807498604	5.50669498560637	0.0610895374174945	0.804782096257532	0.964664965559308
Panx2	Panx2	-0.0130486290836955	6.28524027431279	0.0610323709128067	0.804885711642 64	0.964664965559308
Ppp3cc	Ppp3cc	-0.0086893310607096	5.40918901050147	0.0609942359319215	0.804931229977382	0.964664965559308
Pigw	Pigw	0.0159057096737224	3.17553409918621	0.0609053892752343	0.805070616453428	0.964728347050463
Rps6ka4	Rps6ka4	-0.0104634354086062	5.15072068676601	0.0606825367330527	0.805420429007849	0.965019170214404
Itgb1	Itgb1	0.0090116125252217	7.19363313534835	0.0606580864406112	0.805458815309193	0.965019170214404
Mrpl37	Mrpl37	0.0087913875978697	5.88715512598703	0.0605751527570391	0.805589186748216	0.965051862954413
Atg5	Atg5	-0.010632070914367	5.06485523302989	0.0605162736432674	0.805681801956933	0.965051862954413
Polr2k	Polr2k	-0.01634302269171	3.82905607412118	0.0604931792702246	0.805718141809282	0.965051862954413
Gpt	Gpt	0.0263945902571481	4.73212790739185	0.0603989747083489	0.805866452437818	0.965126852281307
Sgta	Sgta	0.0071654397034325	7.37112757879112	0.0603135415808378	0.806001059904974	0.965205415532 62
Nnmt	Nnmt	0.0423736615038881	-0.141555945113298	0.0601216784071568	0.806303726118031	0.965461981372056
Mcart1	Mcart1	0.0071880473299216	6.18379027588988	0.0600579860526035	0.806404314689824	0.965461981372056
Acaa1b	Acaa1b	-0.0252905510244026	1.9352397134448	0.0600394964760931	0.806450906342474	0.965461981372056
[illegible]	[illegible]	[illegible]	[illegible]	[illegible]	[illegible]	[illegible]

Abcc10	Abcc10	0.0159119532535921	4.19006772582798	0.0584527582408754	0.808958345298914	0.966235452038731
Ston1	Ston1	-0.0240040692917037	2.31564767615334	0.0583585392988793	0.80911260928947	0.96642715164639
Slc25a44	Slc25a44	-0.00667321481465344	6.05483072142538	0.0580146168929119	0.809651891584037	0.96662371986208
Rgs16	Rgs16	0.0197732294211508	2.83988568352875	0.0580145268925065	0.809652036390612	0.96662371986208
Tbk1	Tbk1	0.00958530313426225	5.03010716224416	0.0579951803324121	0.80969316688821	0.96662371986208
Abcg3l4	Abcg3l4	0.0375488977178662	0.712154494076529	0.0579649479678525	0.809741885674517	0.96662371986208
Kat7	Kat7	0.00624263738861 54	6.6818266642 1067	0.0579536906 39728	0.809759946309001	0.96662371986208
Sgpl1	Sgpl1	0.0145405569421439	5.72008843805654	0.0579312100803975	0.809796140379546	0.96662371986208
Lrif1	Lrif1	0.0175849787019128	3.12037655875415	0.0579167114259036	0.809819487354305	0.96662371986208
Myo10	Myo10	0.0142513966451409	5.7866896 1635686	0.0577908211952831	0.810022336574936	0.966653968572231
Parn2	Parn2	-0.0141661394523203	3.32284490557804	0.0577426817617663	0.810099966424925	0.966653968572231
Eml5	Eml5	-0.0280011937286716	5.16558414223353	0.0576980299551053	0.81017200262471	0.966653968572231
Slc35b1	Slc35b1	-0.00623352867130 55	6.38095941761637	0.0576355216900879	0.81027289596 7357	0.966653968572231
Zfp142	Zfp142	0.0165063465283031	6.10755231330452	0.0574946229 35446	0.810516697499206	0.966653968572231
Arntl	Arntl	0.00900021527055145	5.41876088664441	0.05737374404 4692	0.810696055886351	0.966653968572231
Ppp5c	Ppp5c	-0.00761797869092 86	7.02848528761899	0.0573521526498714	0.810731001500405	0.966653968572231
Akap8	Akap8	-0.0169233710794 682	7.06454300427121	0.0573264102382325	0.810772679228629	0.966653968572231
Slc25a23	Slc25a23	-0.0081815412261685	6.801449622922	0.0573175112839124	0.81078709 8473414	0.966653968572231
Anp32a	Anp32a	0.00839008914647 83	7.22377467241529	0.0573085040370174	0.810801674270651	0.966653968572231
Lyrm1	Lyrm1	0.0113405358997918	4.27251814333039	0.0572829162127491	0.810843116276668	0.966653968572231
Camlg	Camlg	0.00857120823 62973	5.65145354079389	0.0572523891688732	0.810892570640174	0.966653968572231
Gpr89	Gpr89	-0.0252038807934397	6.2371031461633	0.0572217227852882	0.810926058468682	0.966653968572231
Acta1	Acta1	-0.0110291556409625	4.03473884773579	0.0571917474116788	0.810990852749554	0.966653968572231
Sox8	Sox8	-0.015495275660587	4.84291702605939	0.0571269354430714	0.811096116651302	0.966653968572231
Dpp3	Dpp3	0.00705936707714800	6.56559259472919	0.0571224853034629	0.811103317 3599831	0.966653968572231
Mmgt1	Mmgt1	-0.00880261027159186	6.24758072173774	0.0570605745079433	0.811203823318025	0.966653968572231
Ekf1	Ekf1	-0.0114336772061436	5.16815824959502	0.0570308812929952	0.811251825689612	0.966653968572231
Madd	Madd	-0.00988312711144 84	7.71361776532049	0.0569865309856257	0.811351470517386	0.966653968572231
RGD130[illegible]	RGD1309[illegible]	-0.0385497349193515	0.0190042057840505	0.0569431544949879	0.811394324197303	0.966653968572231
Pogluf3	Pogluf3	-0.0127152600803871	4.63217792816707	0.0567509964414086	0.811706828982501	0.96690095094 1823
Phyh	Phyh	-0.0114849243862116	5.01544210719415	0.0567173197580962	0.811761666355342	0.96690095094 1823
Syngap1	Syngap1	-0.00842565021919 11	9.22982546501801	0.0565354361452179	0.812058679954948	0.967167684574064
Bag1	Bag1	0.00920038570603111	6.01580553119186	0.0564589597435017	0.81218286 3084164	0.96717018 8382448
Trmt10a	Trmt10a	-0.0117791313669057	3.65576683170556	0.0564258264409574	0.812236852934196	0.96717018 8382448
Sirt1	Sirt1	-0.0123343232419706	4.82990173067261	0.0563916697946496	0.812292730949721	0.96717018 8382448
Zfp467	Zfp467	0.0144870019597073	4.52347971505529	0.0563251548562213	0.812401401817812	0.967207279626034
Atf5	Atf5	0.0103724262165336	4.52710385672122	0.0561046598025764	0.812752128156255	0.967378041333344
Pex2	Pex2	0.0111016829195515	3.96322861387557	0.0560895847108619	0.81278680015053	0.967378041333344
Oxsr1	Oxsr1	0.00621122444210711	6.90908464763799	0.0560561266945525	0.812841626300401	0.967378041333344
Pdgfra	Pdgfra	0.00959488425991 09	7.12654294943717	0.0560479864500394	0.812854963960871	0.967378041333344
Pcgf3	Pcgf3	0.00811932638790 98	5.58710811125877	0.0559502877256861	0.813015121827938	0.967476363857623
Ikzf5	Ikzf5	-0.00918839905490 34	4.84398409172934	0.0557718053186136	0.813308090389041	0.96765017 7342424
Crk	Crk	0.00720657539619 32	6.05075900894462	0.0557668113788665	0.813316294765569	0.96765017 7342424
Alkbh3	Alkbh3	0.0116338052094826	4.48472087319697	0.0556107673300232	0.813572849971417	0.967742350407837
Pax6	Pax6	-0.0221055435606621	3.85335717272737	0.0554926783083087	0.813783727843816	0.967742350407837
[illegible]	[illegible]	[illegible]	[illegible]	[illegible]	[illegible]	[illegible]

Spire1	Spire1	-0.00570309417666	7.50614716784079	0.0412414479909326	0.839072577804333	0.970880203419093
Inpp5f	Inpp5f	0.00737983419797246	6.59328414201699	0.0410725460288096	0.839397952882348	0.97102648071234
Cyp20a1	Cyp20a1	0.0077221284633579	4.20784640339071	0.0410461717685564	0.839448824769856	0.97102648071234
Arl5a	Arl5a	0.00676691471443548	5.16526310225546	0.0410209565603097	0.839497475872135	0.97102648071234
Nadk	Nadk	-0.00571737282711	5.776648620975	0.0410142110744	0.839510493486673	0.97102648071234
Sec61a1	Sec61a1	0.00484894934977058	7.15612962030079	0.0409147263530867	0.839702615127907	0.97102648071234
Tusc2	Tusc2	0.00787071615027446	6.25018884445033	0.0408356388243405	0.839855512971337	0.97102648071234
[illegible]	[illegible]	[illegible]	[illegible]	[illegible]	[illegible]	[illegible]

**Copg1**	Copg1	-0.0040639576762676	7.48662694089809	0.0264699299859998	0.870757937956319	0.97688140210177
**Tceal9**	Tceal9	-0.0074748443617413	5.94829930612899	0.0264592225334184	0.870782850917583	0.97688140210177
**Rala**	Rala	0.0048276923397158	6.84014676098567	0.0264526915157031	0.870802079855775	0.97688140210177
**Amer1**	Amer1	0.0075170614001042	4.84008294495915	0.0264198465438028	0.870879190534748	0.97688140210177
**Pam16**	Pam16	0.0103818952983075	4.70137548052445	0.0263719600770145	0.870995234575773	0.97688140210177
**Pdzd3**	Pdzd3	-0.0172978516185673	2.48838480524349	0.0263148051550957	0.871133880658583	0.97688140210177
**Galnt10**	Galnt10	0.0095209804927348	4.01243362708543	0.0262697775021792	0.871243217342941	0.97688140210177
**Lsamp**	Lsamp	-0.0066817547641142	6.12700271158007	0.0262372730433865	0.87132196175421	0.97688140210177
**Sertm2**	Sertm2	0.0356375923876678	1.17249540966493	0.0262235366707131	0.871355600015436	0.97688140210177
**Ric3**	Ric3	-0.0075255033444022	5.23602990057712	0.0261761217884261	0.871470941710009	0.97688140210177
**Pbxip1**	Pbxip1	-0.010220267821662	5.15175444804229	0.0261721704278557	0.871480558650587	0.97688140210177
**Nabp1**	Nabp1	-0.0125768009057596	2.48859073308943	0.0261548832409559	0.871522641478725	0.97688140210177
**Sptb**	Sptb	-0.0116728131523241	5.70668139630433	0.0261350162656839	0.871571022008044	0.97688140210177
**Nek4**	Nek4	0.0079980815424895	4.0902603245169	0.0261090185294464	0.8716343608227	0.97688140210177
**Zfp523**	Zfp523	0.0047077466248852	6.02958753204896	0.0261011676677612	0.871653494397612	0.97688140210177
**Bola3**	Bola3	0.0085576715549977	3.93544557687702	0.0260283670151864	0.871831059913383	0.976824359720243
**Dleu7**	Dleu7	-0.0133526768340683	3.92321693088522	0.0259834548818958	0.871940730420553	0.976824359720243
**Mpdz**	Mpdz	-0.0099458839639562	5.91884526871407	0.0259471420308302	0.872029473903094	0.976824359720243
**Jsrp1**	Jsrp1	0.029944406145263	-0.466645696397504	0.0258650556371975	0.87222031608621	0.976824359720243
**Satb2**	Satb2	-0.0152127347969996	6.78653668367326	0.0258063802090839	0.872374054181635	0.976824359720243
**MGC116**	MGC116197	0.0175062826494044	1.17852826805853	0.0258012510200061	0.872386653889078	0.976824359720243
**Sspo**	Sspo	-0.0634310060119054	3.22323820973208	0.0257903574460556	0.872413365747558	0.976824359720243
**Dnaaf1**	Dnaaf1	-0.0217502112735776	1.01898818260805	0.0257868765843643	0.872421902061972	0.976824359720243
**Sumf1**	Sumf1	-0.0081338245062547	3.82918744146034	0.025751660018912	0.872508301626534	0.976824359720243
**Nop14**	Nop14	0.0045707437147929	5.49345817875079	0.0257354750710732	0.872548029641431	0.976824359720243
**Afg3l1**	Afg3l1	-0.0071414660051605	5.69608025403051	0.0257122276571806	0.872605115806079	0.976824359720243
**Smim38**	Smim38	-0.0312770586675297	-0.367158772171449	0.0256849453570993	0.872672143776201	0.976824359720243
**Lactb2**	Lactb2	-0.0089146035684681	3.63058765039399	0.0256696658320337	0.872709698921361	0.976824359720243
**Nrbp2**	Nrbp2	-0.0146021298532173	7.72701334465947	0.0255465847823189	0.873012626562679	0.977175801127052
**Gata3**	Gata3	0.0646570650264967	0.772346549619342	0.0255123993314328	0.873096908313921	0.977182465467598
**Slc17a7**	Slc17a7	-0.0089313143489581	7.61173003244961	0.0254633582453749	0.873217903677043	0.977210609037879
**Dpysl2**	Dpysl2	0.0075127709103572	11.4541819890788	0.0254377423525406	0.873281148868745	0.977210609037879
**Cers4**	Cers4	-0.0094995719629936	5.53215978071667	0.0253871850949707	0.873406077097325	0.977210609037879
**Il18bp**	Il18bp	-0.0209648023507593	2.90907404462375	0.0253753505920429	0.873435328285827	0.977210609037879
**Gstp1**	Gstp1	0.0074272725259036	6.41556589639093	0.0252396894942422	0.873771265769451	0.977498797005778
**Tmem11**	Tmem115	0.0057853417129167	5.28465492720473	0.0251682177517279	0.873948369916213	0.977608551644858
**Gmnn**	Gmnn	-0.0112170538681006	3.02032522664036	0.0250552890952065	0.874229370356656	0.977608551644858
**Ddit3**	Ddit3	-0.0082635641522910	3.2849243232714	0.0250534852826974	0.874223860074791	0.977608551644858
**Prmt2**	Prmt2	-0.0046604554126396	7.10127957132932	0.0250175177978775	0.87432341833024	0.977608551644858
**Srrm1**	Srrm1	0.0080707986032719	7.40780725335111	0.025012682561414	0.874335460520418	0.977608551644858
**Cep95**	Cep95	-0.0194741139640372	4.98868837276009	0.0250110657020599	0.874339490887631	0.977608551644858
**Hebp2**	Hebp2	0.0147041975945896	1.15810640645576	0.0249364898431281	0.874525418265542	0.977728821119997
**Tfap2d**	Tfap2d	-0.0293212329420703	2.45369757861246	0.0248424444598072	0.874760292439177	0.977903787186267
**Eps8l1**	Eps8l1	-0.0305414754957973	-0.547471220487354	0.02479226520461	0.874885799329112	0.977956470008003
**Fbxl20**	Fbxl20	0.0090109135919432	3.52798242279114	0.0247509697951109	0.874989185376195	0.977984419816876
**Foxj3**	Foxj3	0.0041309137409558	7.23690163082608	0.0247118330561875	0.875087345706766	0.978006410308431
**Gsap**	Gsap	0.0166158537769187	1.58415316912827	0.0245949205463762	0.875380910243011	0.978148434737066
**Atpaf2**	Atpaf2	0.0062493447823808	4.39703106436937	0.0245805298810637	0.87541682421418	0.978148434737066
**Hspb11**	Hspb11	-0.0111621105798367	2.30741415684338	0.0245675264319232	0.875449512760184	0.978148434737066
**Lrp11**	Lrp11	-0.0070048802879331	7.62175412438904	0.0245121754104076	0.875588755017546	0.978215788903728
**Rbck1**	Rbck1	-0.0041406335112555	6.21693749782998	0.0244812593554968	0.875666598242024	0.978215788903728
**Trpv3**	Trpv3	0.02977294465369	-0.0244560799415803	0.0243832372763819	0.875913740542944	0.978404274015606
**Atp6v1f**	Atp6v1f	-0.0074620322809653	6.54967850429463	0.0242292492659573	0.876303019182182	0.978622436076402
**Noxo1**	Noxo1	0.0282844543276306	-0.315104688922185	0.0242265546375252	0.876309969024124	0.978622436076402
**Kdm4c**	Kdm4c	-0.0078487999072781	4.82879711317067	0.0242129285220614	0.876344351869972	0.978622436076402
**Lmo4**	Lmo4	0.0072019513898532	8.37435178325541	0.0241025016562304	0.876624386274584	0.978847544974772
**Adgrl1**	Adgrl1	0.0054784964877733	9.73657401672292	0.0240520675283236	0.876752502039213	0.978848162995327
**Adipor2**	Adipor2	-0.0059358349622123	5.13645461636582	0.0240344805433281	0.876797209883788	0.978848162995327
**Itpk1**	Itpk1	0.0062296927000859	6.06027865079363	0.0240096750921677	0.876860296369221	0.978848162995327
**Stard4**	Stard4	-0.0080795304435852	4.02930093848858	0.0239604468365968	0.876985594761115	0.978887017440006
**Pcif1**	Pcif1	0.0042179200644996	6.32071532931954	0.0239159223891186	0.877099031877179	0.978887017440006
**Cyp3a62**	Cyp3a62	-0.0244198407969106	-0.291967999122603	0.0238897609269131	0.877165739166451	0.978887017440006
**Tex26**	Tex26	-0.0277240142441717	-0.0756958345541398	0.0238728318466173	0.877208823779491	0.978887017440006
**Rcan1**	Rcan1	-0.0057192023170662	6.64072924958589	0.0237544599662609	0.877511319267657	0.979008178871744
**Gnai2**	Gnai2	-0.0049573273274362	8.56403980582554	0.0237354774072216	0.877559883954371	0.979008178871744
**Rab11b**	Rab11b	0.0047445715660209	8.52369796371828	0.0236913828599406	0.877672769582062	0.979008178871744
**Golga3**	Golga3	0.0054028871211940	5.71475600671054	0.0236195889919068	0.87785677914217	0.979008178871744
**Lrrc71**	Lrrc71	0.0091017923239711	4.48901805451075	0.0236000104865237	0.877907061032416	0.979008178871744
**Hyal3**	Hyal3	-0.0075974733423050	4.10193934451042	0.023587776907263	0.877938441762189	0.979008178871744
**Sgip1**	Sgip1	-0.0074637422539046	7.92848581676042	0.0235504469330506	0.878034315904123	0.979008178871744
**Gadd45g**	Gadd45g	-0.0130134429605405	2.95972056295711	0.0235102411322146	0.878137652354789	0.979008178871744
**Oat**	Oat	0.0049271251840034	6.78348839712856	0.0234211406944627	0.878341229760899	0.979008178871744
**Gfm1**	Gfm1	-0.00583477183802	4.962012572031	0.0234015590702725	0.878417453054061	0.979008178871744
**C2**	C2	0.013141048993188	2.39051279042533	0.0233992652571865	0.8784233630288	0.979008178871744
**Med17**	Med17	-0.0062190059187020	5.32167108056427	0.0233834388991951	0.878464167949429	0.979008178871744
**Rsph4a**	Rsph4a	-0.0282304538284993	1.97292989646767	0.0233687879552207	0.878501952329962	0.979008178871744
**Cdkn2b**	Cdkn2b	0.0259238505876856	-0.131711990192773	0.0233658371647039	0.878509563824041	0.979008178871744
**Spr**	Spr	0.0095318315968474	3.87280185424709	0.0233521490858806	0.878544878332659	0.979008178871744
**Clic2**	Clic2	0.0250592578257007	0.0931112500306849	0.0233412749704911	0.878572940516704	0.979008178871744
**Spag6l**	Spag6l	0.0248828266253935	2.39445677036396	0.0232194061056744	0.878887904156053	0.979106686457526
**Nxpe3**	Nxpe3	-0.0071232982929407	4.46644803551053	0.0231865879593585	0.8789728588096	0.979106686457526
**Plekhn1**	Plekhn1	0.019158759491148	4.27790386849459	0.0231804326189433	0.878988800644661	0.979106686457526
**Mfap3l**	Mfap3l	0.0087999938911355	3.9800341405728	0.0231406155132632	0.879091976091727	0.979106686457526
**Pla1a**	Pla1a	-0.0147524352184011	0.96935557603101	0.023034852602315	0.879366474390504	0.979106686457526
**Rbm25**	Rbm25	-0.0081850945245096	7.32821448251881	0.0230324746328421	0.879372653603059	0.979106686457526
**Tuba1c**	Tuba1c	0.0107552167533837	4.19722461955027	0.0230033821197617	0.879448251649844	0.979106686457526
**Ccne1**	Ccne1	0.0110046600270091	3.58380413281358	0.0229729990972398	0.87952730775757	0.979106686457526
**Rer1**	Rer1	0.0054051355286232	5.98149470709452	0.0229582993241166	0.879565564084862	0.979106686457526
**Rad51c**	Rad51c	-0.0154256437131531	1.09000708580602	0.0229345389660836	0.879627425927	0.979106686457526
**Ablim2**	Ablim2	-0.0080882324332683	5.28454468994715	0.0229311552769218	0.879636238276925	0.979106686457526
**Ift43**	Ift43	0.0112930109387579	2.7483718982318	0.022897772159709	0.87972321565685	0.979106686457526
**Slc36a1**	Slc36a1	0.0070600296023125	4.63049776134399	0.0228888228401268	0.879746540888776	0.979106686457526
**Fgf14**	Fgf14	0.0093759415042380	3.30562863687882	0.0228836753881244	0.879759963282466	0.979106686457526
**Mrpl51**	Mrpl51	-0.0052087567909105	5.20358548544874	0.0226518808823641	0.880365876390723	0.979489798032597
**Prpf39**	Prpf39	-0.0151711470046654	5.84898447844917	0.0226274507086828	0.880429821429013	0.979489798032597
**Lmnb2**	Lmnb2	0.0054198845627974	6.24508972248251	0.022621255456194	0.88044616824183	0.979489798032597
**Lrrc41**	Lrrc41	-0.0040896626882555	6.60842543576772	0.0225957971638167	0.880512955525685	0.979489798032597
**Fnbp4**	Fnbp4	-0.0127256319420773	6.27519912932445	0.0225754602738419	0.880566324988402	0.979489798032597
**Agk**	Agk	-0.0047159979931563	5.7757819100295	0.0225536516131299	0.880623604812341	0.979489798032597
**Recql**	Recql	0.0056899697561989	4.82611266823685	0.0225421851670475	0.88065372720443	0.979489798032597
**L3mbtl2**	L3mbtl2	-0.0079378191322011	5.94298931918358	0.0224984094306601	0.880768814083044	0.979494195703722
**Ccdc163**	Ccdc163	-0.0137772574621144	1.54762081886477	0.0224735370849842	0.880834230015053	0.979494195703722
**Csnk1g3**	Csnk1g3	-0.0040719571249636	6.67154258505587	0.0224511356918029	0.880893193074574	0.979494195703722
**Nrip1**	Nrip1	0.0067275256558980	6.42118234626548	0.022419974479277	0.880975263032783	0.979498160475865
**Arid3a**	Arid3a	0.0127565018789309	2.7908926339221	0.0223508411375803	0.881157549451333	0.979613528670969
**Ppp1r11**	Ppp1r11	-0.0050610639094960	6.00430112204351	0.0223178907656774	0.881244522326053	0.979622953477582
**Sv2c**	Sv2c	-0.0212234254687007	4.02146267096466	0.0222594604861257	0.881398889000107	0.979649550298002
**Gtf2b**	Gtf2b	-0.0062686485044477	4.39703280651776	0.02211922793159	0.881770362068056	0.979649550298002
**Nfxl1**	Nfxl1	-0.0185347373799825	4.85443704569915	0.0220165883457729	0.882042978909231	0.979649550298002
**Ccdc90b**	Ccdc90b	-0.0063900342959585	4.38448541322725	0.0219410188310292	0.882244111388647	0.979649550298002
**Tesk2**	Tesk2	0.0126114924884901	1.7196056455841	0.0219372508263772	0.882253882988237	0.979649550298002
**Ctrl**	Ctrl	0.0184867526285138	1.78376524546066	0.0219093270982285	0.88232856604038	0.979649550298002
**Tbc1d10**	Tbc1d10c	0.0325372315665639	-0.32024595923801	0.0218944727767791	0.882368172503158	0.979649550298002
**Dmac1**	Dmac1	-0.0062145699408819	4.06426399209808	0.0218591482857946	0.882462414292327	0.979649550298002
**Thsd7b**	Thsd7b	-0.0089348159336011	4.81826841747264	0.0218511306540847	0.882483815269107	0.979649550298002
**Trpm4**	Trpm4	0.0093198071109668	5.45036176480736	0.0218454018566376	0.882499109257267	0.979649550298002
**Pdha2**	Pdha2	0.0131056184705424	3.39061921799302	0.0217734896316841	0.88269126561865	0.979649550298002
**Tmem14**	Tmem147	-0.0067674979150900	5.76052036175081	0.0217682763869931	0.882705208524347	0.979649550298002
**Vac14**	Vac14	0.0036004808415233	6.40032573652576	0.0217293879042941	0.882809270089932	0.979649550298002
**Gosr2**	Gosr2	-0.0043413578985828	6.42539715621252	0.0217241736536948	0.882823220120353	0.979649550298002
**Lrp3**	Lrp3	0.0049403205851317	7.46774295471437	0.021687425554326	0.8829216638041	0.979649550298002
**Gphn**	Gphn	-0.0042377550657508	6.74552940526052	0.0216853203981434	0.882927305265021	0.979649550298002
**Chmp1a**	Chmp1a	-0.0047853690862231	7.04801014780573	0.0216718891957228	0.882963305213935	0.979649550298002
**B4galnt1**	B4galnt1	0.0041247972464160	6.99302676482386	0.0216691457682856	0.882970659884251	0.979649550298002
**Tgfbi**	Tgfbi	0.0171769874506896	2.55092675707927	0.0216512565363101	0.883018361387535	0.979649550298002
**Ptprt**	Ptprt	0.0113140674827573	6.66049276554127	0.021582635821547	0.883202822785833	0.979649550298002
**Stk11ip**	Stk11ip	-0.0042235317469366	5.84310693552661	0.0215716870837355	0.883232229591715	0.979649550298002
**Slc6a15**	Slc6a15	0.0040098468738899	6.98989606285334	0.0215475646888947	0.883297077393242	0.979649550298002
**Tm9sf4**	Tm9sf4	0.0040965340428724	5.62026583736305	0.021536040936347	0.883328064799719	0.979649550298002
**Vstm2b**	Vstm2b	-0.0077972747980317	5.84755057284714	0.0215143994978035	0.883386281590563	0.979649550298002
**Tmem69**	Tmem69	0.0070849512431764	4.44208401778323	0.021498750642607	0.883428398602141	0.979649550298002
**Mitd1**	Mitd1	0.0108724346866381	2.72864810560388	0.0214788555768166	0.883481961848862	0.979649550298002
**Asnsd1**	Asnsd1	0.0043530368570258	6.05528664613431	0.0214659950150065	0.883516600963545	0.979649550298002
**Dele1**	Dele1	-0.0052345096881626	5.47013678946501	0.0214263123096537	0.88362354800035	0.979649550298002
**Ddx20**	Ddx20	-0.0062838311047186	4.65217304445682	0.0213712725275172	0.883772057223642	0.979649550298002
**Snrpd1**	Snrpd1	-0.0049912667269084	5.81795612032913	0.0213663413612721	0.883785371862632	0.979649550298002
**Zfp512b**	Zfp512b	-0.0082189058331817	7.89997894988916	0.0213400472182741	0.883856395170282	0.979649550298002
**Gadd45b**	Gadd45b	0.0187770600826863	0.867895427802757	0.0213308826539418	0.883881160145603	0.979649550298002
**Ppp2r2a**	Ppp2r2a	0.0037541643971014	7.20518415956433	0.0213215411908507	0.883906408738639	0.979649550298002
**Gfra3**	Gfra3	0.0281321534581858	-0.489806684280788	0.0213098497511197	0.883938016931546	0.979649550298002
**Xrcc2**	Xrcc2	-0.0090268612254848	3.15357007399347	0.0211979524458919	0.884240981356355	0.979822913565076
**Wdr83**	Wdr83	-0.0066221327839891	4.34887219288232	0.0211542354898237	0.884359054407724	0.979822913565076
**Cdk6**	Cdk6	-0.018579837108034	1.62335657532776	0.021044577779918	0.884657582885356	0.979822913565076
**Ptms**	Ptms	0.0066563327676003	10.4333372832943	0.0210307635093095	0.884759158660389	0.979822913565076
**Tango2**	Tango2	-0.0058206473825300	5.08591395635312	0.0209669605349703	0.884939998878645	0.979822913565076
**Gimap6**	Gimap6	0.020267668526871	0.5119752652606	0.0209273068857611	0.884977162784259	0.979822913565076
**Bub1b**	Bub1b	0.0118364950051095	3.46879455229052	0.0209201768823561	0.884996622524685	0.979822913565076
**Htatip2**	Htatip2	0.0156831716224863	1.26042355550549	0.0208959345907402	0.885062811654719	0.979822913565076
**Rab3ip**	Rab3ip	0.0053277235260205	4.89331884717451	0.0208682992222486	0.885143778885497	0.979822913565076
**Nmt1**	Nmt1	0.0089477510645560	4.90742322154833	0.0208264197853367	0.885252826782037	0.979822913565076
**LOC499**	LOC499407	0.0238223591948057	0.712658223820724	0.0207661316811425	0.885417883487157	0.979822913565076
**Ctbs**	Ctbs	-0.0091238136460042	2.70717696529101	0.020754524856585	0.88544968870202	0.979822913565076
**Eed**	Eed	-0.0062790155774155	4.439703173627	0.0207045352003306	0.885586775050355	0.979822913565076
**Ankrf1**	Ankrf1	-0.0097274517044127	5.36425052537847	0.0206940292599853	0.885615606904	0.979822913565076
**Sh3gl1**	Sh3gl1	-0.0042391270245845	6.7770534109844	0.0206808434278969	0.885651803880455	0.979822913565076
**Exosc2**	Exosc2	0.0052956566807617	4.9121828971017	0.0206757314881543	0.885665840019329	0.979822913565076
**Agpat5**	Agpat5	-0.0038808938396434	6.17918435822197	0.0206555802987724	0.885721242547667	0.979822913565076
**Wnt7a**	Wnt7a	-0.0070555313132197	5.6858465487416	0.0206153359855108	0.885831805820012	0.979822913565076
**Stip1**	Stip1	-0.0040518899185927	7.71271363662893	0.0206008900746042	0.885871528692701	0.979822913565076
**Armh4**	Armh4	-0.0060582101134181	5.59676551035898	0.0205072523220232	0.886129435630431	0.979822913565076
**Vipr1**	Vipr1	0.0190177135061617	0.55816857972826	0.0205044162037211	0.886137259038781	0.979822913565076
**Nifk**	Nifk	-0.0055764343668703	5.31254896845917	0.0204493616354142	0.886289182567702	0.979822913565076
**Slc1a1**	Slc1a1	-0.0046884790299281	5.92508027371056	0.0204476896027046	0.886293799818811	0.979822913565076
**Blmh**	Blmh	-0.0045987138800551	6.6512030882569	0.0204140427029103	0.886386755355436	0.979822913565076
**Dclk2**	Dclk2	-0.0038895720212815	8.11308206823161	0.0203116534499017	0.886670105730221	0.979822913565076
**Tnni3**	Tnni3	0.0258550785785736	0.438641503408095	0.0202700586419979	0.886785422630038	0.979822913565076
**Fabp3**	Fabp3	-0.0091630099330435	4.78008671207591	0.020218482668426	0.886928578952983	0.979822913565076
**Nlgn2**	Nlgn2	0.0041706149717235	8.84392430637756	0.0202175818054258	0.886931081078463	0.979822913565076
**Ltbp4**	Ltbp4	0.0088490542085894	5.87958812257964	0.0201699169008265	0.887062555418574	0.979822913565076
**Mars1**	Mars1	-0.0039911847944035	7.09968349367069	0.020169535270476	0.887064611681937	0.979822913565076
**Prdx3**	Prdx3	0.0063374576422984	5.76649301945856	0.020159811432876	0.887091655840101	0.979822913565076
**Gkap1**	Gkap1	0.0055362512541307	5.14324825267993	0.0201191883864595	0.887204709605817	0.979822913565076
**Ilf2**	Ilf2	0.0039833329690142	7.57908718032856	0.0200798368950572	0.887314325714323	0.979822913565076
**Klhl21**	Klhl21	-0.0077558072359826	4.70483414003988	0.0200291465886124	0.887455711314944	0.979822913565076
**Zfp622**	Zfp622	0.0050066452503160	5.37715329056149	0.0200275688043305	0.887460114692445	0.979822913565076
**Txnrd1**	Txnrd1	-0.0032711791548493	7.23727976266071	0.0199851561847666	0.887578548810799	0.979822913565076
**Igdcc4**	Igdcc4	-0.0066581797886424	6.89891750476521	0.0199777745658167	0.887599174530084	0.979822913565076
**Mrgpre**	Mrgpre	-0.0065629883744256	4.32816941562541	0.0199331895770456	0.887723826620852	0.979822913565076
**Paxx**	Paxx	-0.0060712828027087	5.36278990405154	0.0199265052581126	0.887742528598456	0.979822913565076
**Cygb**	Cygb	-0.0061073795782096	4.65159878902705	0.0198878891332868	0.887850644886364	0.979822913565076
**Rexo2**	Rexo2	-0.0061570139663286	5.89857012618773	0.0198827023223992	0.887921213340502	0.979822913565076
**Lax1l**	Lax1l	0.0116976223675796	6.32294250371121	0.0198611813232219	0.887925476339459	0.979822913565076
**Mark3**	Mark3	-0.0032646768946783	7.49828697927058	0.0198488208432579	0.887960126000449	0.979822913565076
**Rora**	Rora	-0.0095854144073090	2.94420124285076	0.0198438890654177	0.887974008562381	0.979822913565076
**Clvs1**	Clvs1	0.0049209718502513	6.23434270780453	0.0198431250403033	0.887976096530407	0.979822913565076
**Gnb4**	Gnb4	-0.0069734717238701	6.9447850141013	0.0198238654808628	0.888030113156495	0.979822913565076
**Trim5**	Trim5	0.0129453534572575	2.2068179059227	0.0198059071536238	0.888080790478581	0.979822913565076
**Nus1**	Nus1	-0.0034957617508116	6.70248957180833	0.0197608968220866	0.88820691792146	0.979822913565076
**Faxc**	Faxc	0.0066301662568931	5.157861589499	0.0197531837114724	0.888228594589434	0.979822913565076
**Ubr7**	Ubr7	0.0040985160265210	5.69742197302887	0.019727518585178	0.88830075350147	0.979822913565076
**Cc2d1b**	Cc2d1b	-0.0110842292921388	5.42819388153459	0.019724298925997	0.888309806305441	0.979822913565076
**Dgkg**	Dgkg	0.0098021195601481	4.17794690482624	0.0195923775182429	0.888678679301862	0.979822913565076
**Ccdc112**	Ccdc112	0.0063775364779206	4.75688805221717	0.0195560200185173	0.888784163653444	0.979822913565076
**Ceacam1**	Ceacam1	-0.0202475113771461	1.80610162408008	0.019533929097264	0.888846589607244	0.979822913565076
**Spcs1**	Spcs1	-0.0055009192781288	5.86022090640723	0.0195178956450448	0.888891920205529	0.979822913565076
**Abhd6**	Abhd6	-0.0046061321726431	5.09658808028059	0.0195041435557641	0.888930815942906	0.979822913565076
**Mtmr10**	Mtmr10	-0.0067883247326483	3.85589306759354	0.0194909736989819	0.888968078034731	0.979822913565076
**Zcchc17**	Zcchc17	0.0050562958926387	5.74243425521554	0.0194906488794331	0.888968897218223	0.979822913565076
**Thyn1**	Thyn1	0.0044528579828303	5.50389539766669	0.0194616297606203	0.889051148376443	0.979822913565076
**Iqgap3**	Iqgap3	0.0109110218611252	3.70651421780521	0.0194528834982127	0.889075820683444	0.979822913565076
**Timm44**	Timm44	0.0068597140750222	5.16036059944476	0.0194194158437995	0.88917076467376	0.979822913565076
**Myef2**	Myef2	-0.0056128661295536	7.97222510502967	0.0193657361617028	0.889323061660554	0.979822913565076
**Ell3**	Ell3	-0.0128661998811326	1.77219561586217	0.0193638847322362	0.88932831826862	0.979822913565076
**Edn1**	Edn1	-0.0216552052976207	0.155480500225226	0.0192867890381123	0.889547437465285	0.979822913565076
**Cpsf6**	Cpsf6	0.0062470208895381	7.81265556976783	0.0192785426768812	0.889570897747528	0.979822913565076
**Rbm8a**	Rbm8a	-0.0067429172826291	5.9227472092967	0.0192399513023247	0.889680776380339	0.979822913565076
**Foxo1**	Foxo1	0.0114417043464382	4.50726243601758	0.019223942496609	0.889726388601303	0.979822913565076
**Mcat**	Mcat	-0.0063498794367590	4.5816302682184	0.0192158827561962	0.88974935685484	0.979822913565076
**Pank4**	Pank4	-0.0065782198543762	5.37265762129735	0.0191893946355606	0.889824888226582	0.979822913565076
**Chrna7**	Chrna7	-0.0078647685559146	3.80604098308547	0.0191472825406756	0.889945073725783	0.979822913565076
**Slc44a4**	Slc44a4	-0.0260276410174486	0.0388726923864628	0.0191030960617979	0.890071330155339	0.979822913565076
**Zbtb42**	Zbtb42	-0.0164106438719677	0.873438277145026	0.0190814032223675	0.890133367340436	0.979822913565076
**Slf1**	Slf1	-0.0081807838968026	4.41803063479178	0.0190800089934413	0.890137355788317	0.979822913565076
**Unc93b1**	Unc93b1	0.0127065826200979	2.55098828462058	0.0190441090878437	0.890240105175996	0.979822913565076
**Ppp1r3e**	Ppp1r3e	0.0091233640382315	3.5485689438856	0.0190432485758206	0.890242282910151	0.979822913565076
[illegible]	[illegible]	[illegible]	[illegible]	[illegible]	[illegible]	[illegible]

MGC942	MGC94207	-0.0047168742294445	4.96261742399758	0.0154602600684999	0.901046615344653	0.988080435227233
Vps33a	Vps33a	-0.0038373570347781	5.82003187755597	0.015459681446557	0.901048478685189	0.988080435227233
Polr3c	Polr3c	-0.0045802725955230	4.69461801109065	0.015458639163490	0.901051797216236	0.988080435227233
Plcl2	Plcl2	0.0032000574527387	6.32747137109341	0.015423322635867	0.901164310112796	0.988080435227233
Slc5a7	Slc5a7	-0.015783512114182	3.68268630232027	0.015348512542829	0.9014030723919	0.988080435227233
Atp13a1	Atp13a1	-0.0050317012442696	6.73521505997315	0.015266781838491	0.901664599952469	0.988080435227233
Gal3st3	Gal3st3	-0.0037389499263621	6.00796838690951	0.015248159779787	0.901724287452076	0.988080435227233
Rgs10	Rgs10	0.0070209628353638	5.38814563157021	0.015197080819518	0.901888196154122	0.988080435227233
Mef2d	Mef2d	0.0047562008445234	6.2226070727763	0.015180216079798	0.901942375279189	0.988080435227233
Pld1	Pld1	-0.0092026371532663	3.60326035287577	0.0151761117195	0.901955565437934	0.988080435227233
Prpf38a	Prpf38a	-0.0046344491180973	5.50153451336904	0.015174240804725	0.901961578586955	0.988080435227233
Mpv17	Mpv17	-0.0058988637541511	4.26334740039876	0.015170020667127	0.901975143555988	0.988080435227233
Cfap52	Cfap52	0.0132373665650887	3.26430631617578	0.015168424010539	0.901980276256951	0.988080435227233
Pgam5	Pgam5	0.0039779108539187	5.96826448878113	0.015154718892720	0.902024345418485	0.988080435227233
Slc25a26	Slc25a26	0.0064726885261722	3.48054949548107	0.015114659436437	0.902152627113016	0.988080435227233
Pim3	Pim3	-0.0043242606632591	5.92728007111601	0.015090222695825	0.902232003050124	0.988080435227233
Plaa	Plaa	0.0031672167849689	6.18403257885407	0.015040307623984	0.902393023911682	0.988080435227233
Ephb1	Ephb1	0.0044542368606435	7.15242641027237	0.015029100582112	0.902429184655751	0.988080435227233
Zkscan3	Zkscan3	0.0046137454622028	5.30901800831185	0.015012147598854	0.902483884916925	0.988080435227233
Cfp	Cfp	0.015883618496884	3.30911431673501	0.015008917142793	0.902494425301478	0.988080435227233
Fscn1	Fscn1	-0.0038145874523827	9.62857405373913	0.014964970565969	0.902636568792332	0.988080435227233
Efhb	Efhb	-0.0298308722077723	0.1329252182143	0.014946133895033	0.902697559529068	0.988080435227233
Arl2bp	Arl2bp	0.0034279352866297	7.67780262261507	0.014925189394049	0.902765388154099	0.988080435227233
Hnrnpk	Hnrnpk	-0.0034973932087117	9.53495452985758	0.014919991548389	0.902782269442483	0.988080435227233
Smpd4	Smpd4	-0.0051600616129644	5.91356735445514	0.014889994239313	0.902879563990735	0.988080435227233
Cyth1	Cyth1	-0.0044760622375846	5.25185594210896	0.014873929908971	0.902932032808037	0.988080435227233
Serinc4	Serinc4	0.02111509871029	0.0663482227470585	0.014767136910336	0.9032790879929	0.988080435227233
Ctsd	Ctsd	-0.0048679125698171	7.42368883815377	0.014722092805499	0.903425988805053	0.988080435227233
RGD131	RGD131055	0.0045865663120970	5.10664835223759	0.014703396858735	0.903487030850265	0.988080435227233
Clic3	Clic3	0.0115829393126756	1.69554255412279	0.014657435028311	0.903637258725248	0.988080435227233
Pxt	Pxt	-0.0148269230555265	3.09975605474769	0.014654685279168	0.903646253949581	0.988080435227233
Rab10	Rab10	-0.0030519323456146	8.80597578456372	0.014599451617158	0.90382712090389	0.988080435227233
[illegible]	[illegible]	[illegible]	[illegible]	[illegible]	[illegible]	[illegible]

**Prrg2**	Prrg2	-0.0143726048205782	-0.441350887274699	0.00733567001174151	0.931745821959606	0.985619561888611
**Dtwd2**	Dtwd2	0.00544577638030165	2.21236876265165	0.00732525858167638	0.931747721370564	0.985619561888611
**Coq7**	Coq7	-0.0041647702769723	4.23900910091093	0.00729479963290596	0.931982317326432	0.985619561888611
**Zgpat**	Zgpat	0.0063877384424586	5.36988079186223	0.00728369014700547	0.931987484556885	0.985619561888611
**Atpsckmt**	Atpsckmt	-0.0039011390936524	4.20170354869246	0.007256845877329	0.932112627946967	0.985619561888611
**Bace2**	Bace2	-0.0094612349182106	1.87263726735159	0.00724998111250841	0.932144667722491	0.985619561888611
**B3galt6**	B3galt6	0.0029575292367172	5.56481424647501	0.00722980351377782	0.932238782929682	0.985619561888611
**G2e3**	G2e3	-0.0070548636380776	4.79495785805842	0.0072001480322382	0.932377712125261	0.985619561888611
**Dnm2**	Dnm2	0.0026121221498280	6.62075177572414	0.00717575141159443	0.932492099090983	0.985619561888611
**Lingo1**	Lingo1	-0.0029788655721700	8.49614781547012	0.00717116372609894	0.93251363096081	0.985619561888611
**Sort1**	Sort1	0.0021190177346103	8.10008557450723	0.00715468406785646	0.932591032955082	0.985619561888611
**Dyrk1a**	Dyrk1a	0.0023142016632101	7.46821241571984	0.00714025451064515	0.932658881622954	0.985619561888611
**Nsa2**	Nsa2	-0.0026750020439546	6.20925552440351	0.0071229368352661	0.932748400422856	0.985619561888611
**Src**	Src	0.0021172414944521	6.82533007360255	0.00711488844579966	0.932778296689076	0.985619561888611
**L1cam**	L1cam	0.0043134396692228	9.51390662648589	0.00706923965187773	0.932992330729508	0.985619561888611
**Cep55**	Cep55	-0.0079327002975481	1.81521906758804	0.00706411836588217	0.933018622021543	0.985619561888611
**Pacs1**	Pacs1	0.0027161137535394	7.64214999435511	0.00705219888569709	0.933074422940309	0.985619561888611
**Cnnm3**	Cnnm3	-0.0031228396810142	6.1642845478613	0.00702651924148859	0.933219610382901	0.985619561888611
**Cul2**	Cul2	-0.0021772899937547	6.89644999759384	0.00702168098816983	0.933219001395117	0.985619561888611
**S100pbp**	S100pbp	0.0039394078232764	5.55570189304655	0.00700915899076904	0.933279482642455	0.985619561888611
**Pank2**	Pank2	-0.0032508277349660	4.42607932272363	0.00700264227066327	0.933309434437963	0.985619561888611
**Espl1**	Espl1	0.0056385964672339	3.47231933282273	0.00699245215317745	0.93335786247537	0.985619561888611
**Bcl2l2**	Bcl2l2	0.0038170420346107	5.66348908649756	0.00698260511180138	0.933404893831362	0.985619561888611
**Sf3b2**	Sf3b2	0.0019824447037831	8.25510880516683	0.0069807345915649	0.933412593560178	0.985619561888611
**Gopc**	Gopc	-0.0019297565559015	6.77708985452703	0.00696493923886444	0.933488793557718	0.985619561888611
**Lat**	Lat	-0.0155201210944217	0.406683649433174	0.00688502891388686	0.933870567758998	0.985619561888611
**Cxcl15**	Cxcl15	-0.0031101212082080	5.68181056688313	0.00682699001281464	0.934149676386216	0.985619561888611
**Ubxn10**	Ubxn10	-0.0167148596826104	0.372410233983864	0.00682281459745582	0.934169328479827	0.985619561888611
**Ttc8**	Ttc8	0.0038540356765241	4.25696535179286	0.00681051658682463	0.934228559892057	0.985619561888611
**Vps50**	Vps50	-0.0021955199270592	6.60757840060744	0.00678173017628581	0.934367392486109	0.985619561888611
**Thop1**	Thop1	0.0028113945298660	6.24308103944449	0.00677814381459285	0.934384709742343	0.985619561888611
**Clk1**	Clk1	0.0079349208547331	6.08271544447728	0.00677021703108355	0.934422518500882	0.985619561888611
**Aspm**	Aspm	0.0072547797652283	4.26010677479347	0.00676634997670078	0.934441890509901	0.985619561888611
**Fgf22**	Fgf22	0.0045247329120661	3.17017760356303	0.00675939628632705	0.934475310011421	0.985619561888611
**Adprh**	Adprh	0.0029000801548876	5.81602420114648	0.00674793285746142	0.934530766256359	0.985619561888611
**Oard1**	Oard1	0.0049538159870617	3.00352304759812	0.00672653463217188	0.934634424646263	0.985619561888611
**Dnai1**	Dnai1	0.0116408873711287	0.684499005805523	0.00670447361537185	0.934741462682921	0.985619561888611
**Cep57**	Cep57	0.0026988062022591	5.42308145785309	0.00665810934549915	0.934966897284082	0.985619561888611
**Olig2**	Olig2	-0.0039947160659350	5.83300076080412	0.00661279552798533	0.935188187119078	0.985619561888611
**Lrrc45**	Lrrc45	0.0052484014130228	5.86588806467235	0.00658194013365687	0.935339238088046	0.985619561888611
**Nod1**	Nod1	-0.0072738406787981	3.01245259245702	0.00656312132036874	0.935431539616239	0.985619561888611
**Dact3**	Dact3	-0.0040370911949056	7.15977389519732	0.00654922894307219	0.935499763708851	0.985619561888611
**Pced1b**	Pced1b	-0.0042664843449232	3.14052675600246	0.00654224161485928	0.93553410521616	0.985619561888611
**Arfrp1**	Arfrp1	0.0030754146151215	4.82403004797255	0.00653376145884197	0.93557580873539	0.985619561888611
**Akr1c19**	Akr1c19	-0.0192573885466771	0.373726088795604	0.00649702522252937	0.935756784346188	0.985619561888611
**Shisa7**	Shisa7	-0.0044598603550010	7.84057537557876	0.00649651670737826	0.93581037394589	0.985619561888611
**Phtf1**	Phtf1	-0.0047815052200223	5.12880079089854	0.00649411857640951	0.935820489215614	0.985619561888611
**Naca**	Naca	-0.0031433993244342	8.12308975769457	0.00646998232607041	0.935890321511083	0.985619561888611
**Mmadhc**	Mmadhc	0.0032856763336685	4.99049940748867	0.00646878941017803	0.935896234166699	0.985619561888611
**Mob3a**	Mob3a	-0.0027080765932646	6.32777354830967	0.00645550143142515	0.935961966625772	0.985619561888611
**Mylpf**	Mylpf	-0.010848212244363	0.190742469898104	0.00643752948168697	0.936050977120345	0.985619561888611
**Sesn3**	Sesn3	0.0043410493184308	6.09800544293192	0.0064226318302213	0.93612386414099	0.985619561888611
**Dusp16**	Dusp16	0.0060983742517255	2.36744709387235	0.00641327832934735	0.936171285856499	0.985619561888611
**Ntn1**	Ntn1	-0.0142972885829122	4.25124183654576	0.00640841047062679	0.936195462628991	0.985619561888611
[illegible]	[illegible]	[illegible]	[illegible]	[illegible]	[illegible]	[illegible]

Pc	Pc	-0.0016202482157783	5.77197972585668	0.00191217256761433	0.965120904573365	0.992599765473563
Nsg1	Nsg1	-0.0016573513230282	9.81852100741274	0.00190874790973794	0.96515213252144	0.992599765473563
Smk	Smk	0.00103887123224606	6.68143661551221	0.00190859682553679	0.965153510837963	0.992599765473563
Tmtc3	Tmtc3	-0.0022234628306347	4.60589648939778	0.00189147469581741	0.965310068914641	0.992642773649645
Kif18a	Kif18a	-0.0041307807586520	1.92264825712789	0.00188663547515233	0.965354445416751	0.992642773649645
Rnaset2	Rnaset2	-0.0028065103390134	3.91021381789669	0.00188654540916559	0.965549356772439	0.9927613781994
Sptbn1	Sptbn1	0.0020116536233775	10.0819837086841	0.00182132857733881	0.96586572697207	0.99287116015257
Cdh8	Cdh8	0.0016708750744206	7.37488203824744	0.00181852235632908	0.96588539875692	0.99287116015257
Dnah1	Dnah1	0.0065832480091113	2.12754968047167	0.00181826033709715	0.965986726203884	0.99287116015257
Trappc4	Trappc4	0.0019810707239467	5.24276316705145	0.00181218579433828	0.966044489313639	0.99287116015257
RGD130	RGD130590	-0.0018663511832675	5.63227956462109	0.00181116907351964	0.966054010118795	0.99287116015257
Dynlrb2	Dynlrb2	-0.0083762918626705	1.31936619220051	0.00179694328467406	0.966187506807756	0.992921144182593
Cyc1	Cyc1	-0.0032598594128490	2.03314588817699	0.00177527675194256	0.966391849697395	0.992921144182593
Prox1	Prox1	0.0058042782655176	4.63228239738004	0.00176831403423705	0.96645778189305	0.992921144182593
Hdac10	Hdac10	-0.0040857500279136	5.97946650811228	0.0017669480155007	0.966470732409448	0.992921144182593
Adamts1	Adamts10	0.0028287406629750	4.82959644601234	0.00176293387836414	0.96651005154211	0.992921144182593
Stambp	Stambp	-0.0015292934875276	4.91997843879123	0.00175543041563486	0.966580124818725	0.992921144182593
Pole2	Pole2	0.0017269904201261	5.44267197918628	0.00170213737210645	0.967091038753224	0.993282812600322
Esrrb	Esrrb	0.0106718031524393	0.80010300728246	0.00170208824254641	0.967091416804417	0.993282812600322
Hecw2	Hecw2	-0.0025857678420541	5.29793944613623	0.00169242232005765	0.967185026129484	0.993297190799043
Golga4	Golga4	-0.0025157966436782	6.52826934580036	0.00167758806482254	0.967329083999971	0.993307948752718
Klhl23	Klhl23	-0.0010611944885967	6.88576021740904	0.00166965641472672	0.967406366609214	0.993307948752718
Nox4	Nox4	0.0059538723719601	0.843773867379536	0.00165418882451552	0.967557802955308	0.993307948752718
Fbxo15	Fbxo15	-0.0039776139321814	1.44772399805743	0.00165126541561154	0.967586271165467	0.993307948752718
Chek2	Chek2	-0.0040112609174503	1.40076689742682	0.00165052277290556	0.967593556875894	0.993307948752718
Tmem67	Tmem67	-0.0030428280428347	5.15270099264218	0.00163970508259581	0.967699670757775	0.993325358901255
Acyp1	Acyp1	0.0034075564646875	3.40597467171797	0.00162113790935337	0.967882167028745	0.993441779780985
Ilm	Ilm	0.0055715779640815	0.985744711263763	0.00160880003089294	0.968005546553844	0.993480781787979
Dbt	Dbt	0.0018048535892071	4.62272145919712	0.00160127571771085	0.968080415562896	0.993480781787979
Traf6	Traf6	0.0027245041833129	3.15958826652624	0.00156058222299029	0.968488289927071	0.99363527071689
Chrnb2	Chrnb2	0.0011524773845782	6.11074167980525	0.00154215250230294	0.968674925706073	0.99363527071689
Atp13a5	Atp13a5	0.0039285454445974	2.62141362238526	0.00151191188939424	0.968983422197921	0.99363527071689
Hnrnpd	Hnrnpd	0.0013478944853454	8.1414755570991	0.00151137680390434	0.968988908552488	0.99363527071689
Ccdc130	Ccdc130	-0.0019014604940368	4.15614589957838	0.00150827777367368	0.969020702499469	0.99363527071689
Wdr19	Wdr19	-0.0019513534596575	4.84967626701687	0.00150480223496885	0.969056398108179	0.99363527071689
Tlr2	Tlr2	-0.0052291477189274	2.28713024543104	0.00149427664368318	0.969164754034728	0.99363527071689
Poglut1	Poglut1	0.0014382817091731	5.00504406373333	0.00148609069415784	0.969249389935875	0.99363527071689
Rhbg	Rhbg	-0.0064193467129837	-0.0932045204317729	0.00148515869852348	0.969258928254338	0.99363527071689
Sf3b6	Sf3b6	-0.0022878983405932	4.65542136352858	0.00148274887192201	0.969283866370956	0.99363527071689
Eif2s3	Eif2s3	0.0033096324920905	8.09022361864321	0.00147757293166251	0.969337498323714	0.99363527071689
Rps5	Rps5	0.0017881245177569	8.24815644469898	0.00146853483079212	0.969431375188132	0.99363527071689
Pcsk6	Pcsk6	0.0017713181289210	4.20169293866859	0.00146543517832498	0.969463637221885	0.99363527071689
Btbd16	Btbd16	0.0056635726184129	-0.0326089410325169	0.00146372596412903	0.969481441809172	0.99363527071689
Pbdc1	Pbdc1	0.0022490043983774	4.59314330493625	0.00145647259165571	0.969557115014723	0.99363527071689
Tp53inp	Tp53inp2	-0.0010362805961964	8.0047305041647	0.00145532170601825	0.969569129341016	0.99363527071689
Malsu1	Malsu1	0.0022589273296078	4.17275660888502	0.00144490560474253	0.96967818290846	0.99363527071689
Kcnj9	Kcnj9	-0.0018674281631060	4.43719822737585	0.00143654905888013	0.969765950377817	0.99363527071689
Stxbp5	Stxbp5	0.0020393678294712	6.41696696918521	0.00143448227405328	0.969787686636728	0.99363527071689
Egfl7	Egfl7	-0.0029343411035173	4.25481472925026	0.00142105439239411	0.969823799358042	0.99363527071689
Ocrl	Ocrl	0.0011921907491571	6.07082770075141	0.00139310410769156	0.970226422516955	0.993966259441954
Pacsin2	Pacsin2	0.0012995857546238	5.07504148999153	0.00137194516231176	0.970453289144091	0.994036366051622
Ppp4c	Ppp4c	-0.0014755453127602	5.68815689806413	0.00136248768237124	0.970555258722616	0.994036366051622
Stx17	Stx17	0.0023676164978012	2.99572005769661	0.00135472736150888	0.97062819470195	0.994036366051622
Mrps27	Mrps27	-0.0012043495118042	4.54145350410162	0.00134717840558096	0.970721075849927	0.994036366051622
Atg12	Atg12	0.0010312088316345	6.4997314923942	0.00134106311683979	0.970787575031126	0.994036366051622
Dnaja2	Dnaja2	0.0010081955139860	7.62168769936493	0.00134057608858775	0.970792842982505	0.994036366051622
Slc22a3	Slc22a3	0.0041802798284011	3.52683542833685	0.00132618051325675	0.970950016510319	0.994036366051622
Uqcc1	Uqcc1	-0.0015022480585827	4.76781851144188	0.00132505134767058	0.970962413694925	0.994036366051622
Slc1a2	Slc1a2	-0.0024852594624384	5.7458412514757	0.00132205386654758	0.971011880214589	0.994036366051622
Ncln	Ncln	0.0015191559786465	7.45051702446646	0.00130899515448846	0.971138802882719	0.994075912800223
Dohh	Dohh	-0.0016855998955082	6.26545504726579	0.00128623938095089	0.971389546291562	0.994075912800223
Lix1	Lix1	-0.0027243778081844	3.86310010043475	0.00128550787243853	0.971398799099708	0.994075912800223
Kcnj12	Kcnj12	0.002311092828857	2.95986292014586	0.00127473682198342	0.971518814244505	0.994075912800223
Rsrp1	Rsrp1	-0.0035053711157431	6.9354804544162	0.00127462522317501	0.97152008045691	0.994075912800223
Hps6	Hps6	-0.0023626657562285	3.61548760215907	0.00127000124916776	0.971571744005828	0.994075912800223
Gpr84	Gpr84	-0.0073416045979480	-0.46296726317507	0.00126369981737184	0.971642329914886	0.994075912800223
Gstt2	Gstt2	-0.0024659294473608	2.72574960345043	0.00125964050121752	0.971687892322796	0.994075912800223
Idh1	Idh1	-0.0021654877499846	8.40290079645552	0.00124966777821101	0.971800143324179	0.994088805289063
Ndufaf2	Ndufaf2	0.0027650787364582	2.84115297635409	0.00123876237579668	0.971923406938433	0.994088805289063
Pdcd2l	Pdcd2l	-0.0020072242265651	4.0843801424255	0.0012372407454902	0.971939951566345	0.994088805289063
Idua	Idua	0.0041493688561216	5.30715314536784	0.00122164223964871	0.972118005310382	0.994140490664712
Gpr174	Gpr174	0.0245772738898899	-1.47128050085464	0.00121889211845359	0.97214940506261	0.994140490664712
Kcnb1	Kcnb1	-0.0014019905295081	5.56851151576143	0.0011996049109495	0.972370543782399	0.994285139712587
Arhgef1	Arhgef1	-0.0036686960240644	6.50819683799221	0.00117821720773748	0.972617854955052	0.994458525139243
Tmem80	Tmem80	0.0016564593005146	4.26454973933422	0.00116504628666547	0.972771273531032	0.994485428246855
Maf1	Maf1	0.0013063374614276	6.98265755438834	0.00115410515793712	0.972899380290938	0.994485428246855
Clcc1	Clcc1	-0.0014269110289614	4.98651611407781	0.00112403519101179	0.973254626862981	0.994485428246855
Meis2	Meis2	-0.0031425988499867	7.84553665425434	0.00112357271717656	0.973260127435903	0.994485428246855
Hpcal4	Hpcal4	0.0016302282744041	5.42983831071704	0.00111648911047268	0.973344520515075	0.994485428246855
Slc25a14	Slc25a14	-0.0012482003713742	4.62593166112277	0.00110952040832233	0.973427806560584	0.994485428246855
Pdxp	Pdxp	0.0012872519830727	7.58414312675564	0.00110899069192615	0.973434406881977	0.994485428246855
Lcn12	Lcn12	-0.0060990564145664	1.26274691322496	0.00109079600530038	0.973652896215724	0.994485428246855
Bdh1	Bdh1	-0.0011036790264052	6.1216644732288	0.00109036186316303	0.973658137969775	0.994485428246855
Dscam	Dscam	-0.0012227587244809	6.71816422199802	0.00108943159138164	0.973669373422606	0.994485428246855
Nup160	Nup160	0.0013835261657936	5.30452200434535	0.00108808196366522	0.973685561354025	0.994485428246855
Plod2	Plod2	-0.0017522129682923	3.88958293265897	0.0010749916745425	0.973844392777077	0.994485428246855
Trim27	Trim27	-0.0011833804066973	6.08802495494515	0.00107402598036898	0.973856140815085	0.994485428246855
Prxl2b	Prxl2b	-0.0019268806445865	4.10917312909836	0.00107048413261444	0.973899266623905	0.994485428246855
Thumpd	Thumpd3-as	0.0025113405022977	3.25910025174946	0.00106812071827278	0.973928085490264	0.994485428246855
Rpap1	Rpap1	-0.0013230409551141	5.1572578605743	0.00105958518326415	0.974032307572228	0.994485428246855
Aga	Aga	-0.0017105399472900	3.70438460628133	0.00105897689231682	0.974039882287511	0.994485428246855
Fbxo5	Fbxo5	-0.0023512832288022	2.49445499226738	0.00105563827234789	0.974080822201236	0.994485428246855
Rock1	Rock1	-0.0039641139610513	6.80524949273542	0.00104613486702476	0.974197714072457	0.994516416808336
Stbd1	Stbd1	-0.0037030961870169	1.08670703562199	0.00103547297290163	0.97432948978984	0.994516416808336
Fgfr2	Fgfr2	-0.0014853219640977	5.63253097042232	0.00103337963201924	0.974350298875138	0.994516416808336
Ermn	Ermn	0.0067984258893183	-0.248901921133563	0.000980298648471489	0.975022537726275	0.994941158667971
Gpr135	Gpr135	-0.0017761188114385	3.45282533265077	0.000979247988770066	0.975035822068706	0.994941158667971
Htr5a	Htr5a	0.0029273291659504	2.38838720637703	0.000977066049301638	0.975063740817585	0.994941158667971
Pdzd7	Pdzd7	0.0037872940971462	3.97709438570553	0.000975389200152663	0.975085396184335	0.994941158667971
Prps2	Prps2	0.0015731933131056	5.30208185001198	0.000956008197982965	0.975332752077106	0.995112171686645
Clec11a	Clec11a	0.0016870326919714	3.16505824837227	0.000915784909999218	0.975858151492482	0.99556881571314
Akt	Akt	0.0026840536438868	2.54028226988237	0.000896553341806465	0.976112576350761	0.995624158582921
Tmco4	Tmco4	0.0036149866283841	0.37777522179885	0.000892949901071916	0.97616094815102	0.995624158582921
Aph1b	Aph1b	0.0018969277612671	3.12329314171733	0.000892754644659988	0.976162553894659	0.995624158582921
Mdga2	Mdga2	0.0013977908533732	4.99412063422526	0.000871811012054025	0.97644472729336	0.995699754252777
Pdlim7	Pdlim7	0.0011298083720610	4.87497688037579	0.000871516080776401	0.976448711085132	0.995699754252777
Avpr2	Avpr2	-0.0055252488167255	1.12010560922017	0.000869969772537615	0.976469607246179	0.995699754252777
Nhej1	Nhej1	0.0044120445039808	-0.0172085345847378	0.000864249027067267	0.976547078049904	0.995699754252777
Tmem14	Tmem143	0.0012589683689507	3.9828181537841	0.000849893928790085	0.976755675338847	0.995820363967614
Eme1	Eme1	0.0045180234561851	0.167708945412523	0.000827936838540211	0.977044823730579	0.996064571728608
Mrpl32	Mrpl32	-0.0016018992092853	3.45505895171471	0.000813877405285268	0.977240608305813	0.996063745482579
Dsn1	Dsn1	0.0022426812786745	2.61930642433413	0.000806577734095981	0.977342875188718	0.996063745482579
Gpx8	Gpx8	-0.0017266742260045	3.96435651171709	0.000804557835152764	0.977371255282177	0.996063745482579
Triqk	Triqk	0.0012957962797986	4.91238434135135	0.000801031844759882	0.97742088191871	0.996063745482579
Epop	Epop	-0.0022848460380181	3.50509645427602	0.000798053091642714	0.977462891695816	0.996063745482579
Prpf40b	Prpf40b	0.0016610920827776	6.64095379158608	0.00078888898977425	0.977592629723763	0.996078330600695
Tpk1	Tpk1	-0.0017922411140154	2.89864917470321	0.000781707737978265	0.977694822273377	0.996078330600695
Rspo1	Rspo1	-0.0032664262586641	0.700070261827955	0.000778042835477777	0.977747157108962	0.996078330600695
Rce1	Rce1	0.0012893717396139	4.71801564972941	0.000774582757757375	0.97779652708402	0.996078330600695
Emp2	Emp2	-0.0026794254667055	2.44388453922524	0.000747453218409078	0.978188879885646	0.996098748942771
Noxa1	Noxa1	0.0059147773924909	-0.519252010871762	0.000747083481749062	0.978194127852658	0.996098748942771
Ins2	Ins2	-0.0008447052830940	6.70252558120379	0.000745996574801574	0.978210126334928	0.996098748942771
Trafd1	Trafd1	-0.0014406912948423	7.2324544620443	0.000744616204400472	0.978230301785659	0.996098748942771
Wwp2	Wwp2	0.0008258531467526	5.12238599329062	0.000743497486027422	0.978246657376559	0.996098748942771
Cops4	Cops4	-0.0006997275888635	6.92485758946765	0.000732116215878752	0.978427822042408	0.996098748942771
RGD156	RGD156001	-0.0031827642970944	0.796212225193152	0.000728182861763571	0.978471805675928	0.996098748942771
Zfp317	Zfp317	-0.0011458911077880	5.35501061474572	0.000727083475461174	0.978486243442586	0.996098748942771
Ptdss2	Ptdss2	-0.0008343239186670	6.15298496419768	0.000723906800950216	0.978535093836964	0.996098748942771
Gstcd	Gstcd	0.0013247330739589	4.00430020934037	0.000702981536136349	0.978847527477506	0.996296657174168
Timm10b	Timm10b	0.0016803403601148	3.70290209881314	0.000702211864540279	0.978889228090194	0.996296657174168
Stx3	Stx3	-0.0026514470160927	3.2568563559326	0.000692490654287781	0.979005817574469	0.996308074003276
Tmem22	Tmem222	0.0010286865287112	5.22960002287354	0.000683752148209121	0.979138754137975	0.996308074003276
Polr2c	Polr2c	-0.0010514913349133	5.47977524517742	0.00068306723905294	0.979139985848312	0.996308074003276
Mcm8	Mcm8	-0.0020869205805166	2.28423497791638	0.000666676597198711	0.979400813752844	0.996430820034777
Ptx3	Ptx3	-0.0019704278252703	3.44081180061666	0.000664991075552734	0.979426895188756	0.996430820034777
Bclaf3	Bclaf3	0.0013537202701372	4.49473763074348	0.000652322141902083	0.979639371784111	0.996430820034777
Mrps24	Mrps24	0.0014103985192350	5.02749001080087	0.000647896684433746	0.979692987105518	0.996430820034777
Tmem24	Tmem243	0.0020317889423472	1.94546290114301	0.000647070652740964	0.979705861479406	0.996430820034777
Trmt11	Trmt11	0.0021339811740016	1.81927173255097	0.000644931285754001	0.979739785219736	0.996430820034777
Glipr1	Glipr1	-0.0016067651162016	2.72473830402841	0.000637817967941601	0.979851520795047	0.996403234571689
Fam227a	Fam227a	0.0035418172008462	5.10043517035021	0.000619773251132835	0.980138517957632	0.996502511928034
Ddx27	Ddx27	0.0006896087183512	5.70138641617625	0.000612388824057796	0.980257494175944	0.996502511928034
As3mt	As3mt	-0.0025858207265817	2.24007906411219	0.000599919515565261	0.980459164238731	0.996502511928034
Jph4	Jph4	-0.0008228559806252	8.34847529882002	0.000598945368328047	0.98050928477747	0.996502511928034
Uqcc2	Uqcc2	-0.0018707039378755	4.63370461511934	0.000596640391734127	0.98051263127982	0.996502511928034
Sgcd	Sgcd	0.0023307839083514	1.01371089085712	0.000592340032767424	0.980582973170823	0.996502511928034
Rgs7bp	Rgs7bp	-0.0012621190530281	4.43988669560971	0.000589252675298504	0.980631981940189	0.996502511928034
Slc44a2	Slc44a2	-0.0011255280606794	6.15686585986638	0.000583321013381	0.980731323989596	0.996502511928034
Hbegf	Hbegf	0.0011843243240836	3.8903371746832	0.000582326854898249	0.980747755897065	0.996502511928034
Septin4	Septin4	-0.0012932077716877	4.21230051256531	0.0005759091186893	0.980854118999139	0.996502511928034
Ift80	Ift80	-0.0012042912007552	3.67247790996868	0.000565724452030736	0.981024124691115	0.996502511928034
Pdlim1	Pdlim1	-0.0023853284193898	2.45713642400024	0.000565291409799329	0.981031397362321	0.996502511928034
Mchr1	Mchr1	0.0024337403873335	2.39926278874299	0.00056481388196695	0.98103940769648	0.996502511928034
Far1	Far1	0.0010390315422398	5.13254278631479	0.00055819986577346	0.981150730892967	0.996502511928034
Btbd3	Btbd3	-0.0016234594974484	6.00672854189505	0.000557456802567913	0.981163278246536	0.996502511928034
Myom1	Myom1	0.0035809903846151	0.192982601409433	0.000552126282102344	0.981258182984408	0.996502511928034
Cenpu	Cenpu	-0.0029797944130137	0.984151125072639	0.000544152885723292	0.981389367025304	0.996502511928034
Arse	Arse	-0.0022789178077265	5.05655735205842	0.000525230038943682	0.981715763768978	0.996502511928034
Dock8	Dock8	-0.0029359025549676	1.61942349809922	0.000519289434574081	0.981819459076322	0.996502511928034
Def8	Def8	0.0007208340556399	5.95528317367141	0.000516244398241383	0.981872814799506	0.996502511928034
Slc25a35	Slc25a35	0.0031360041922536	0.660995423897723	0.000508886699080335	0.98200242386367	0.996502511928034
Phospho	Phospho2	0.0009856844500441	4.42594341006898	0.000506826653356995	0.982038892062422	0.996502511928034
Ncapd3	Ncapd3	0.0010326758759007	4.95172040249724	0.000505921343105342	0.982054808811508	0.996502511928034
Stxbp5l	Stxbp5l	0.0017513100916370	4.31298923517361	0.000501780398394658	0.98212851715366	0.996502511928034
Chp1	Chp1	0.0006564147601874	6.67538659360629	0.000499148702495233	0.98217543637654	0.996502511928034
Nosip	Nosip	0.0008721422255486	4.84911155055327	0.000498965345335444	0.982178709968032	0.996502511928034
C1s	C1s	0.0025814409924332	1.97119209130936	0.000497211456842272	0.982210053745559	0.996502511928034
Klf15	Klf15	0.0012091408033251	3.45686589607949	0.000494922878252421	0.98225292908749	0.996502511928034
Cope	Cope	-0.0009993483081516	5.78565728452112	0.000492960066668679	0.982286260719536	0.996502511928034
Fam104a	Fam104a	0.0007074102861696	5.53068855387362	0.000485863913425533	0.982414196482299	0.996502511928034
Gas6	Gas6	0.0010035215649165	6.65217325563327	0.000484286705528802	0.982442758523065	0.996502511928034
Tamm41	Tamm41	0.0010980191284223	4.2518347807151	0.00047570509134416	0.982598980929148	0.996502511928034
Lgalsl	Lgalsl	-0.0006902761482766	7.17762106152106	0.000469500037513448	0.982712920529051	0.996502511928034
Fn3k	Fn3k	-0.0022098114816632	0.903407775153001	0.000468822076719277	0.982725314269178	0.996502511928034
Snf8	Snf8	-0.0008480851427644	5.13493032681072	0.000467082433955989	0.98275738936342	0.996502511928034
Ncor2	Ncor2	0.0011877581844741	7.73270304490768	0.000464050868686172	0.982813427738801	0.996502511928034
Ccdc61	Ccdc61	0.0012986416103863	3.3447103053473	0.000462334920449337	0.982845228162731	0.996502511928034
Krcc1	Krcc1	0.0014121254567543	3.51080783129026	0.000458864312759839	0.98294700710456	0.996524726386881
Vps54	Vps54	0.0011052873162148	5.80700150130805	0.000452337737298782	0.983030448253056	0.996525269326478
Parm1	Parm1	-0.0015518091851465	3.08375862565234	0.000445485371997734	0.98316058034147	0.996525269326478
Atp6ap1	Atp6ap1	-0.0006832157851265	8.39082885550799	0.000442169509821876	0.983222457987116	0.996525269326478
Csrp1	Csrp1	-0.0016534859164471	4.19972860745385	0.000437390117639325	0.983314358346941	0.996525269326478
Snapc4	Snapc4	0.0017763603108542	5.17859708619258	0.000433155158470289	0.983395322101132	0.996525269326478
Dlk2	Dlk2	-0.0012069136004905	5.40637805880116	0.000425039811190686	0.983551583217452	0.996525269326478
Plin5	Plin5	0.0040535794345403	-0.501277372619007	0.00042494957268957	0.983553329111562	0.996525269326478
Dcakd	Dcakd	0.0005621937390613	7.17642162013426	0.000421144614534211	0.983627124376839	0.996525269326478
Ergic2	Ergic2	-0.0005647631092376	5.83383853863289	0.000418117080329239	0.98368606406279	0.996525269326478
Tarbp2	Tarbp2	0.0006134742138438	5.58945480716793	0.00041503817735844	0.983746232451249	0.996525269326478
Fam76b	Fam76b	-0.0012767210671856	5.07129814605544	0.000405070332533288	0.983942572787449	0.996614950369489
Spns1	Spns1	-0.0008313548144733	6.88514354453671	0.000402452557555305	0.983994515797205	0.996614950369489
Cnnm2	Cnnm2	-0.0008720642291997	4.65292934543284	0.00039579432881709	0.98412746852461	0.996668703300064
Kcnf1	Kcnf1	0.0012514260785021	5.15371628906854	0.000385362939908873	0.984338002800945	0.99669423801258
Ppp6r3	Ppp6r3	-0.0005052589669173	7.0954552759852	0.000381808765716585	0.984410385596946	0.99669423801258
Ubald1	Ubald1	-0.0007403808049044	6.04088785841147	0.000379132438649038	0.984485611597078	0.99669423801258
Megf8	Megf8	-0.0013763845885096	8.02552535241943	0.000376404168401212	0.984521102305653	0.99669423801258
Atg16l2	Atg16l2	-0.0020183148686873	1.6570441806621	0.000371598909132635	0.984620376432374	0.99669423801258
Csk	Csk	-0.0006212198066136	7.02140326182609	0.000371509186560732	0.984622088200934	0.99669423801258
Rbmx2	Rbmx2	0.0009302111982820	3.65434756380712	0.000368282247433958	0.98473061713065	0.996704345535355
Matr3	Matr3	-0.0006061457720731	9.50880358244147	0.000357998092916034	0.984904256568947	0.996704345535355
Bcl11b	Bcl11b	0.0014495347629612	7.96275309321459	0.00035218986310781	0.985027200962165	0.996704345535355
Eya2	Eya2	0.0030032108170760	2.42810604263752	0.000348246621626913	0.98511124748093	0.996704345535355
Umps	Umps	0.0006323581968456	5.10653324380191	0.000347168906699835	0.985134300690388	0.996704345535355
Dmpk	Dmpk	0.0023472889689893	5.14067660505196	0.000346011863385343	0.98515909067848	0.996704345535355
Trmu	Trmu	0.0011645188793808	3.52384904762732	0.000340737252992529	0.985272629649213	0.996704345535355
Kcnh8	Kcnh8	-0.0016247045509767	2.73323158448768	0.000340341657377508	0.985281180414491	0.996704345535355
Thap3	Thap3	0.0009504932982645	4.25677599954959	0.000335926186992145	0.985379126143619	0.996704345535355
Rhob	Rhob	0.0006927100435409	8.73245634036653	0.000332999237803288	0.98544078559581	0.996704345535355
Rpain	Rpain	-0.0011222257247691	3.01696480576658	0.000322505349960041	0.985664981873643	0.996709245829486
Prkd1	Prkd1	0.0011836800471817	3.81650570101016	0.000319930234429967	0.985729123274558	0.996709245829486
Scrn2	Scrn2	-0.0011493989676225	2.89430071215268	0.000319086036697058	0.985748162376328	0.996709245829486
B4galt5	B4galt5	-0.0004975979016493	6.24982260845718	0.000318224597259334	0.985765175405615	0.996709245829486
Ppat	Ppat	0.0006014423344552	5.6866991782605	0.000314503006921368	0.985850752041732	0.99671500147676
Ggh	Ggh	-0.0008503457391225	4.70126810412871	0.000309957367909056	0.985953486575568	0.996728101760598
Dars2	Dars2	0.0008177721857666	3.61005697008759	0.00028965207694051	0.98642130075412	0.997087789928953
Mrps18b	Mrps18b	-0.0007612357221634	4.85470609555732	0.000288037498024527	0.986459219274104	0.997087789928953
Rps7	Rps7	-0.0006258428658097	9.07321755938376	0.000282109238577721	0.98659927493006	0.997111824289077
Catsper	Catsperg	-0.0024092453105792	4.05350349236141	0.000279485441183169	0.986661732379276	0.997111824289077
Bnc2	Bnc2	0.0036579714913998	0.574000791597785	0.000275934161760778	0.986722745411205	0.997111824289077
Ifi44	Ifi44	0.0020398425353132	1.4978265173041	0.000268448083025442	0.986927736891113	0.997180344710453
Abcb4	Abcb4	-0.0018297521451513	1.32626370982332	0.000267489799128384	0.986951097691706	0.997180344710453
Rnf123	Rnf123	-0.0006701837872246	6.85126742943393	0.000260828035045461	0.987114579959093	0.997265474829928
Scn1a	Scn1a	-0.0011526436651003	6.64625539496918	0.000242379200275741	0.987553014676372	0.997590183806482
Ap1m1	Ap1m1	-0.0004043902845045	7.50897979259664	0.000241705153449345	0.987595880492162	0.997590183806482
Ddx39b	Ddx39b	-0.0008795432972137	8.52680921934002	0.000235603789510817	0.987753439682799	0.997620138199167
Nt5dc1	Nt5dc1	-0.0010583758179785	2.80283725771866	0.000234372328576779	0.987785460231827	0.997620138199167
Lrrtm3	Lrrtm3	0.0005730715540248	5.38817901205551	0.000225702374259674	0.988012516589195	0.997769700241472
U2af1l4	U2af1l4	0.0006206930442082	4.68978207439109	0.000216305574248585	0.988265670985001	0.99794357211109
Zfp24	Zfp24	-0.0006958060495443	4.32122104565836	0.000212812111456628	0.988360808111196	0.997958873740627
Capn12	Capn12	-0.0019021622508144	2.29781379316508	0.000206795521194181	0.988526506931065	0.998045414062966
Dbr1	Dbr1	-0.0006918722282155	3.97854174762805	0.000202356571151086	0.988650308145175	0.99808964274839
Enpp6	Enpp6	-0.0022475159775726	-0.331420660031096	0.000189504188647049	0.989016826517889	0.998145970521934
Vps72	Vps72	0.0004849464655222	6.63481748907408	0.000182486747817734	0.989221892309994	0.998145970521934
Scly	Scly	-0.0007695442902886	3.16565040528881	0.000182175862633471	0.989231076480071	0.998145970521934
Wnt7b	Wnt7b	-0.0007770456557915	4.8693644525352	0.000182148712378183	0.98923187889536	0.998145970521934
Spata18	Spata18	0.0025888591786311	0.803603240109525	0.000177935792911196	0.989356011888719	0.998145970521934
Il11	Il11	0.0011526382036808	1.77618854822517	0.000174222070029373	0.989468771503246	0.998145970521934
Zfp746	Zfp746	-0.0004348959088856	5.53906332674442	0.00017053669260747	0.989580745750316	0.998145970521934
Pnn	Pnn	-0.0011149156842326	7.03025794482593	0.00016732220447943	0.989679374078096	0.998145970521934
Farp1	Farp1	0.0004225441435181	8.34660625538944	0.000162857897144875	0.989818009559428	0.998145970521934
Pou3f4	Pou3f4	0.0016839473434474	3.22344766145453	0.000162637722759828	0.989824894091362	0.998145970521934
Sncg	Sncg	-0.0034132573870385	1.10895792014749	0.00016036229514792	0.989896319931881	0.998145970521934
Gnpda1	Gnpda1	-0.0005249283032214	4.42600750982826	0.000156032236510839	0.990033654597422	0.998145970521934
LOC360	LOC360919	-0.0016157243438947	1.9393983049557	0.000152395616936474	0.990150475454198	0.998145970521934
Eif1ad	Eif1ad	-0.0006443820113629	4.80656568803221	0.000148386838532133	0.990280678484813	0.998145970521934
Naa50	Naa50	0.0006667532104261	5.82841841296366	0.000145282292559833	0.990383092886289	0.998145970521934
Dnai4	Dnai4	0.0007478915756287	3.69291347539499	0.000141109937679573	0.990522175825498	0.998145970521934
Prdx5	Prdx5	-0.0005885723184810	6.21173222979121	0.000140789222911053	0.990532952049116	0.998145970521934
Snx2	Snx2	0.0003977100146278	5.86051434657421	0.000140698485699231	0.990536124849008	0.998145970521934
Ppp4r3b	Ppp4r3b	0.0003590617436552	6.44025245103394	0.000140659838034729	0.990537303006654	0.998145970521934
Cyb561d	Cyb561d1	0.0013219123544585	2.98890677644574	0.000138608394176654	0.990606557545752	0.998145970521934
Yif1b	Yif1b	0.0006221293846560	5.50388389543492	0.000137654131565649	0.990632168948289	0.998145970521934
Dmap1	Dmap1	-0.0006411315887237	5.36759014950323	0.000137418010652368	0.990646978266808	0.998145970521934
Acap3	Acap3	-0.0004130568023307	7.72165322486096	0.000135860138350067	0.990700143257555	0.998145970521934
Prpf4b	Prpf4b	0.0006341081305207	7.08125552524179	0.000135154298243111	0.990724320941174	0.998145970521934
Lypla2	Lypla2	0.0003857650501045	6.65253782789191	0.000131482838526058	0.990851145341288	0.998145970521934
Efcab1	Efcab1	0.0017842629579908	1.89096233344981	0.000130779004120202	0.990875733784066	0.998145970521934
Tmub1	Tmub1	-0.0006675416212290	5.35655341808905	0.000129909838307185	0.990906068480496	0.998145970521934
Tomm34	Tomm34	0.0003551311176126	5.52902994206515	0.000128769383955785	0.990946071720382	0.998145970521934
Pphln1	Pphln1	0.0003494405283791	5.50236364573083	0.000125118382754863	0.991075342335191	0.998195596247674
Mpnd	Mpnd	0.0005294041699845	6.25322402909883	0.00011996211465523	0.991261130683232	0.998302133145204
Ankrd37	Ankrd37	-0.0016581191123662	-0.0050435676935286	0.000112214536337976	0.991544293185341	0.998482074482121
C1qb	C1qb	-0.0014030518880334	2.74918127794347	0.000110843323653853	0.991599854506092	0.998482074482121
Aspdh	Aspdh	0.0010451045437462	2.89092373145271	0.00010295910372804	0.991904102784729	0.998494047516418
Oxnad1	Oxnad1	-0.0005000251523791	3.67145437373332	0.000101209202590289	0.991973394541444	0.998494047516418
Gpn3	Gpn3	-0.0006458422268823	4.19935170423769	9.91798510980857e-05	0.992054072308574	0.998494047516418
Ccnl2	Ccnl2	-0.0010033771325823	5.25379044273951	9.69285458575531e-05	0.992144770200864	0.998494047516418
Pmm1	Pmm1	0.0003722394942291	6.50015534596378	9.68160324977597e-05	0.992149330463816	0.998494047516418
Adsl	Adsl	0.0003085758160144	6.45098571341115	9.63658244828025e-05	0.992167804538764	0.998494047516418
Gli4	Gli4	-0.0005383882795820	3.52744695760509	9.43630113354743e-05	0.992249421359006	0.998494047516418
Lmntd2	Lmntd2	0.0013121180674316	0.3907723611596	9.21447838564404e-05	0.992341058183979	0.998494047516418
Ythdf1	Ythdf1	-0.0002042217436960	6.74400293212697	9.1194450682508e-05	0.992380654511611	0.998494047516418
Rnf166	Rnf166	-0.0003487498207147	4.61440451527227	9.04453312458031e-05	0.992412012763573	0.998494047516418
Kif11	Kif11	-0.0007025577548373	4.10919659960422	8.54518953019579e-05	0.99262444465628	0.998672725334432
Eif2s1	Eif2s1	-0.0002038358691893	6.15649040472791	7.95485545065997e-05	0.992883762237925	0.998807603115484
Agbl5	Agbl5	0.0003169289851845	5.56904235894298	7.08871476930995e-05	0.993282329755764	0.999127983257229
Cyth4	Cyth4	0.0010441872432405	1.61077805006688	6.91287378771221e-05	0.993366169485235	0.999131777240409
Cep41	Cep41	0.0004615014703865	3.95692847584674	6.68023800542983e-05	0.993478744928308	0.999164460682153
Tp53bp1	Tp53bp1	0.0003557447014321	8.29483699252672	6.17617852753838e-05	0.993729966167007	0.999200352120885
B4galt2	B4galt2	0.0002707080541615	7.74836491868881	6.12991327598843e-05	0.993753125375728	0.999200352120885
Odf3	Odf3	0.0010412584478574	0.307318862634057	6.04000454629272e-05	0.993799105787086	0.999200352120885
Adat3	Adat3	-0.0005243109946975	2.9596339967217	5.53085954209109e-05	0.994066207259551	0.999200352120885
Gria2	Gria2	0.0004763010831606	8.75217445509221	5.41864848095282e-05	0.99412616560126	0.999200352120885
Ttc14	Ttc14	0.0007703857963210	4.60435040372789	5.29358981253836e-05	0.994194877712337	0.999200352120885
Spata24	Spata24	0.0007880742974368	1.15997592275046	4.91006005844952e-05	0.994409122362942	0.999200352120885
Lrrc43	Lrrc43	-0.0008041127988717	1.27469998730114	4.78869694672362e-05	0.994479803174349	0.999200352120885
Borcs8	Borcs8	0.0003556348032871	3.6941412001277	4.67957305907768e-05	0.994541821339653	0.999200352120885
Acss1	Acss1	-0.0004365765444343	4.02349819516113	4.67891685644872e-05	0.994542304032138	0.999200352120885
Gimap5	Gimap5	-0.0010935769256482	0.231786115818123	4.60779267299403e-05	0.994582943437759	0.999200352120885
Nrarp	Nrarp	0.0003425875081610	5.28967318312546	4.47036473759544e-05	0.994665320839659	0.999200352120885
Gmppb	Gmppb	-0.0003056284829925	3.62069298086207	4.43282532043217e-05	0.994687732158506	0.999200352120885
Mms22l	Mms22l	0.0005635008526765	2.86184303462659	4.39066748227832e-05	0.994713087077451	0.999200352120885
Ralgps2	Ralgps2	0.0002902493626711	5.2271277045248	4.38189598098825e-05	0.994718370623446	0.999200352120885
Mrs2	Mrs2	-0.0006625002857896	5.4023419296619	4.0684407697084e-05	0.994910403965864	0.999301983872255
Snx1	Snx1	-0.0002228901650126	4.9329919759704	3.85627213077555e-05	0.995045257292781	0.999301983872255
LOC679	LOC679894	0.0006098812136516	5.25823302295067	3.83057104187401e-05	0.99506179574406	0.999301983872255
Slc9a3r2	Slc9a3r2	-0.0003241271426647	5.250262236559	3.71444302231794e-05	0.995137224462032	0.999301983872255
Pdcl3	Pdcl3	-0.0002055094471824	5.77555446848871	3.42555545387313e-05	0.995330148361841	0.999354962985011
Ilf3	Ilf3	0.0002906381900003	7.58670941141477	3.39623815364121e-05	0.995350174321931	0.999354962985011
Hmox1	Hmox1	0.0006296551109390	2.42315585622502	3.2743045380812e-05	0.995434406655152	0.999359115854227
Slc39a5	Slc39a5	-0.0008517702940781	4.22852915459667	2.98577968891323e-05	0.995640197862215	0.999485296381596
Mif	Mif	-0.0002619265941456	7.6771091160019	2.72457834179224e-05	0.995835185055875	0.999490611741122
Rab3gap	Rab3gap2	-0.0002402752889516	6.07427111715288	2.27507007792838e-05	0.996194289013213	0.999880632613455
Crbn	Crbn	-0.0001589655798824	5.32225409482565	2.10139996141834e-05	0.996342427862167	0.999902442303118
Irf4	Irf4	-0.0009555595335404	-0.417765685603979	2.00153354938498e-05	0.996430395916418	0.999902442303118
Zdhhc21	Zdhhc21	-0.0002429112750473	5.26352046247998	1.80884248521807e-05	0.99660656818544	0.999902442303118
Dhfr	Dhfr	0.0003529164610373	2.35390320515451	1.71310052650142e-05	0.996697595664378	0.999902442303118
Mrps11	Mrps11	-0.0001525525906200	5.06136450105161	1.58810499963954e-05	0.996820355851043	0.999902442303118
Mtfr1l	Mtfr1l	0.0001153374139061	6.9798824197241	1.53791348225241e-05	0.996871004774776	0.999902442303118
Rcbtb2	Rcbtb2	-0.0001474036589771	5.60698804712334	1.53296123237864e-05	0.99687614846585	0.999902442303118
Hsd17b1	Hsd17b10	0.0002121518331700	4.94833406706867	1.52625747631419e-05	0.996882984756876	0.999902442303118
H2aj	H2aj	-0.0003090874982320	3.95286341000209	1.24227390898568e-05	0.99718778937791	0.999902442303118
S1pr5	S1pr5	-0.0005567179298632	-0.331075895052435	1.18042437549093e-05	0.997258889108583	0.999902442303118
Arglu1	Arglu1	0.0002055258638442	6.82895828967025	1.16807653808387e-05	0.997275400038905	0.999902442303118
Fbxl6	Fbxl6	0.0002195426068246	4.88303408920384	1.12009882116126e-05	0.997329654714883	0.999902442303118
Zzz3	Zzz3	-0.0001632177812221	5.6972297065056	1.06217033890976e-05	0.997399622638792	0.999902442303118
Tbpl1	Tbpl1	9.48525340577483e-0	5.75505142948924	8.08278539021048e-06	0.997731598109415	0.999902442303118
Trim11	Trim11	-0.0001068144691275	4.6262638765544	6.56857695702229e-06	0.997955085123492	0.999902442303118
Kctd18	Kctd18	0.0001089857788991	5.12632542108456	6.29139526608824e-06	0.997999895889525	0.999902442303118
Rab33a	Rab33a	0.0001222168583964	5.18303283134938	6.02228671188191e-06	0.998041965500996	0.999902442303118
Ncoa4	Ncoa4	-5.65146517589495e-	7.25334245986421	5.79407511480667e-06	0.998079423092759	0.999902442303118
Mcts1	Mcts1	-9.96814978754587e-	5.50435075109658	5.12636157168345e-06	0.998193472620413	0.999902442303118
Rbm19	Rbm19	-7.95441979293956e-0	4.9771813247097	4.95558506941052e-06	0.998223819201908	0.999902442303118
Taz	Taz	-0.0001451793466987	5.46040227302041	4.95420322161852e-06	0.998224067397412	0.999902442303118
Dpagt1	Dpagt1	8.24076488749003e-0	5.17181203293895	4.63860317267972e-06	0.998281564103632	0.999902442303118
Ufsp2	Ufsp2	8.77678504018426e-0	4.72135486479425	4.61479253743845e-06	0.998285880263971	0.999902442303118
Grik5	Grik5	-4.31754899816393e-	6.7662722292811	4.52385729232674e-06	0.998302951781239	0.999902442303118
Id2	Id2	-0.0001308241375054	7.06777354576892	3.51152164057567e-06	0.998504840647016	0.999902442303118
Tsnaxip1	Tsnaxip1	0.0001028113959657	-0.081085020710921	3.45613187846538e-06	0.998516679618232	0.999902442303118
Trim44	Trim44	-7.2048549523148e-0	6.56901324247066	3.40659040643031e-06	0.998527351372863	0.999902442303118
Atxn7l3b	Atxn7l3b	5.0145536930646E-05	9.44538532680376	3.37930077219026e-06	0.998534186606448	0.999902442303118
Pspc1	Pspc1	5.3985442819477e-05	6.64049358087983	3.16430768521059e-06	0.998580683716196	0.999902442303118
Pla2g7	Pla2g7	0.0005355120513635	0.422700612711834	2.87729013016852e-06	0.998646585188506	0.999902442303118
Trip6	Trip6	-0.0001037675637042	3.58991613614928	2.55216352229581e-06	0.998725340681439	0.999902442303118
Ctnnd2	Ctnnd2	4.2184014069948e-05	8.87409591987569	2.06581603279163e-06	0.99885320515018	0.999902442303118
Ube2m	Ube2m	4.31688240287897e-0	7.71510303742359	1.44557914438792e-06	0.999040685754097	0.999902442303118
Ranbp6	Ranbp6	3.70760631177685e-0	6.10700898358068	1.39098138791065e-06	0.999059236481946	0.999902442303118
Erc1	Erc1	4.2889180208729e-05	7.03363696226965	1.10196201319458e-06	0.999162425795341	0.999902442303118

**Izumo1**	Izumo1	-0.000187433706229	0.346709380963083	1.00535935132484e-06	0.999199980358428	0.999902442303118
**Slc33a1**	Slc33a1	3.16110774059508e-0	5.09112684983446	8.79666564843626e-07	0.999251659819182	0.999902442303118
**Mapk1**	Mapk1	2.16165522136147e-0	8.69805612840346	7.97965029164516e-07	0.999287258686407	0.999902442303118
**Rap1b**	Rap1b	2.28766274731701e-0	6.2581370381575	4.5316988206423e-07	0.999462880959578	0.999902442303118
**RGD156**	RGD156021	-1.55609961605359e-	5.88884309575161	2.09532799999579e-07	0.999634770326887	0.999902442303118
**Kpna6**	Kpna6	-1.42911138446905e-	5.41732048274586	1.85106621586328e-07	0.999656718005094	0.999902442303118
**Gcdh**	Gcdh	1.73433715832153e-0	5.40818303458376	1.33356124365491e-07	0.999708628859076	0.999902442303118
**Rufy2**	Rufy2	2.91810341925729e-0	4.87287267270651	9.42582829566163e-08	0.999755037380904	0.999902442303118
**Nr2c2ap**	Nr2c2ap	-9.55147142994659e-	4.47989455520891	5.20255696201319e-08	0.999818009599921	0.999902442303118
**Abraxas1**	Abraxas1	2.71803822003956e-0	1.78877943012344	2.8271524906919e-08	0.999865842610286	0.999902442303118
**Bet1**	Bet1	-7.16381246813822e-	4.0052693484319	1.49500607449227e-08	0.999902442303118	0.999902442303118

Supplementary Table 12 - Control vs MARA PC17 corrected DE genes

gene_id	gene_name	logFC	logCPM	LR	PValue	FDR
Mfrp	Mfrp	1.46538360900543	3.70103966133294	26.1797691336024	3.11064422085671e-07	0.00388115079448768
Sav1	Sav1	0.149831132792263	4.99581423006526	20.9129587587727	4.80632102921138e-06	0.0299842237407352
Grin3b	Grin3b	0.276258809237002	4.20051938533434	18.7593020198331	1.48298233475871e-05	0.0600085906775867
Cyp1b1	Cyp1b1	-0.292446686199682	2.87687553266504	18.2632657027174	1.92381472076896e-05	0.0600085906775867
Mt1	Mt1	-0.352358213961111	2.43575548069438	16.8194991872035	4.11087251659543e-05	0.098164421359175
Polq	Polq	0.560289764412309	1.57744249072624	15.9971532257144	6.34377984860018e-05	0.098164421359175
Efna2	Efna2	0.148501523968146	4.89650102813456	15.9820628790876	6.39454944276307e-05	0.098164421359175
Gli1	Gli1	-0.261092189890945	2.64588557025882	15.9434122347514	6.52645196214228e-05	0.098164421359175
Bach2	Bach2	0.171722846127332	5.76826161381358	15.4405075955319	8.51435177116444e-05	0.098164421359175
Tbccd1	Tbccd1	0.197143106226845	3.91710293407378	15.2181924796395	9.57762947935844e-05	0.098164421359175
Pcsk9	Pcsk9	-0.8839030160797	0.475664654778186	15.2127968900338	9.60503281119155e-05	0.098164421359175
Fbll1	Fbll1	0.102370314299305	6.68757355245799	15.1867711292994	9.74864559749185e-05	0.098164421359175
Lmo7	Lmo7	-0.109662459247726	6.19068579660454	14.769266219069	0.000121499691393452	0.098164421359175
Iffo1	Iffo1	-0.133075814446722	5.03836802454872	14.7626488955585	0.000121862189705158	0.098164421359175
Ddx46	Ddx46	-0.0850221581664966	6.90690733880401	14.7375950612282	0.000123557788936583	0.098164421359175
Kiaa0408L	Kiaa0408L	0.118995253914791	8.26345430512181	14.6928039996432	0.000126451420497236	0.098164421359175
Sorbs2	Sorbs2	0.128313500265504	6.61317970059241	14.58819634673	0.000133749712529514	0.098164421359175
Plxna2	Plxna2	0.125072961024907	8.45332463466354	14.4459622055031	0.000144238751972189	0.0999814949087083
Ctdspl2	Ctdspl2	0.284110345299452	4.62923584830344	14.2673606710175	0.000158591187304741	0.104144328621645
Ifrd1	Ifrd1	0.108265586703896	5.79108261418758	14.0807222486023	0.000175129547063811	0.109254567935758
Ttr	Ttr	0.937433618628324	9.3647075224497	13.8820424873624	0.000193616480560927	0.110940363823831
Mbd3	Mbd3	0.110860132815712	7.03699727632128	13.8727383773341	0.000195615453420236	0.110940363823831
Rnf165	Rnf165	0.146569630870339	5.62830080971164	13.6677129099478	0.000218173506476207	0.114159288485967
Col23a1	Col23a1	-0.198572197402301	4.2927664893099	13.6277405661645	0.000222867824051125	0.114159288485967
Thbs2	Thbs2	-0.302883936845589	2.61130006195245	13.5789228419919	0.00022873945757387	0.114159288485967
Kdm6b	Kdm6b	0.113884778027457	7.01876663579617	13.3582065362931	0.000257294242358622	0.11664019189498
Col16a1	Col16a1	-0.179099480644192	4.63721562869947	13.2741181716891	0.000269095423627402	0.11664019189498
Fzd8	Fzd8	0.136240142930057	4.7002748188995	13.2713850347755	0.000269488033016238	0.11664019189498
Helz	Helz	0.126193082454255	4.95323043499039	13.2620939498876	0.000270639221363662	0.11664019189498
Pcdha11	Pcdha11	-0.252213052838177	2.92246798888373	13.1716133919952	0.000284221821485783	0.118207897145937
Creb1	Creb1	0.216275531069829	4.22571824205434	12.8456916019681	0.000338257166661983	0.128671430311255
Plxnb3	Plxnb3	-0.41064134780326	2.57029109582935	12.7777286960269	0.000350770571122663	0.128671430311255
Renbp	Renbp	-0.291574162564554	1.31679156806668	12.7474894690353	0.000356487332860937	0.128671430311255
Banp	Banp	0.191690194747088	4.77409253002959	12.6945030159499	0.000366732057583242	0.128671430311255
Gng5	Gng5	0.15390389528144	4.1234672160303	12.6811282703761	0.000369364821695109	0.128671430311255
Mtmr4	Mtmr4	0.0806474666880972	7.49481858615923	12.6715741514358	0.000371256822186705	0.128671430311255
Sf3b1	Sf3b1	-0.0749166797074336	8.80737104085462	12.3822860222624	0.000433426169358361	0.146158332840116
Cntn2	Cntn2	0.153381250332172	7.3584625173445	12.23223018459	0.000468971020582107	0.152303986549309
Tuft1	Tuft1	-0.270622106754497	2.30232194988884	12.1772331797206	0.000483763475361654	0.152303986549309
Fam107a	Fam107a	-0.214781162988688	4.64999834918293	12.1301929281469	0.000496119871305987	0.152303986549309
Slc7a11	Slc7a11	-0.188128018724475	5.62197112086344	12.0828849510696	0.000509867955355585	0.152303986549309
Pnisr	Pnisr	-0.122417948247928	7.43705295545311	12.0433853638559	0.000519754890932621	0.152303986549309
Ephx1	Ephx1	-0.21964979529808	3.73021883896714	12.0250904491574	0.000524991514115594	0.152303986549309
Cep83	Cep83	-0.0957423489477651	5.73961265466341	11.94382085845	0.000549289204482482	0.153548082156277
Stc1	Stc1	0.241560229460896	2.57680465741607	11.9252159434366	0.00055379207678388	0.153548082156277
Sh2b2	Sh2b2	0.112487609453416	5.69095916863376	11.8125073424958	0.000588341468529558	0.159581228325393
Rhobtb2	Rhobtb2	0.0861849331297256	6.35258367903824	11.7547012363346	0.000606897519483295	0.161111922353044
Mapk7	Mapk7	0.0824278721831099	5.96340603696011	11.6882490418525	0.000625589383764587	0.161694408366344
Kcnj13	Kcnj13	0.444049776786161	1.89128673649878	11.6249456725528	0.000650731411680533	0.161694408366344
Aldh18a1	Aldh18a1	0.0941375352548245	6.68216944693272	11.5858580318062	0.000671702727639362	0.161694408366344
Vwa5b2	Vwa5b2	-0.228845862739765	6.20191750384836	11.5602699997406	0.000673761773018798	0.161694408366344
Slc35c1	Slc35c1	0.0917469765856642	5.76112605866304	11.5473678032392	0.000678452404349583	0.161694408366344
Pag1	Pag1	0.185917752274597	3.14521367444505	11.5245030597346	0.000686984809196251	0.161694408366344
Nfyc	Nfyc	0.0919978753076589	5.76108536487585	11.4865862575646	0.000701002727157576	0.161970574569353
Tmem97	Tmem97	0.190397196815599	5.09962038876221	11.4415416000385	0.00071820180623356	0.162927344297748
Zbed5	Zbed5	0.242142398648887	3.17426564555955	11.3485705471473	0.000755060628928219	0.168230204849177
Zfhx4	Zfhx4	0.157012377988911	5.77221360027757	11.2951557265251	0.000777095821263879	0.168810372102264
Hoxa5	Hoxa5	3.53738326352883	1.39403859482953	11.257645088588	0.000792957456368505	0.168810372102264
Dynlt3	Dynlt3	-0.107463180496574	4.52867507437128	11.2452849029715	0.000798255390150258	0.168810372102264
Tspan2	Tspan2	-0.108299671239799	6.06479197706416	11.1021915738353	0.000862257638183824	0.179306475860326
Igf1r	Igf1r	0.170827612867179	5.16788264336059	11.0208921982034	0.000900906886975444	0.181768590295648
Mmd2	Mmd2	-0.120079851274742	6.10597742956712	10.9643722309878	0.000928804070427848	0.181768590295648
Dus3l	Dus3l	-0.115702503609723	5.3894167577161	10.929122460704	0.000946642228229149	0.181768590295648
Fam89a	Fam89a	-0.17955952340945	3.16207458669324	10.8829336710317	0.000970543865764434	0.181768590295648
Arhgef40	Arhgef40	-0.128996323892977	6.37533937665346	10.7863064912394	0.00102253707425752	0.181768590295648
Epsti1	Epsti1	-0.598908517502027	0.0469855574495665	10.779696196036	0.00103261952312479	0.181768590295648
Bicral	Bicral	0.123262261068799	5.40194514306561	10.7794808808672	0.00103269922929271	0.181768590295648
Bcorl1	Bcorl1	0.128863582639841	5.83483824690379	10.7622180069875	0.00103359316088173	0.181768590295648
Armc5	Armc5	-0.108847937191827	5.44849276763328	10.7153689769426	0.0010624938293128	0.181768590295648
Srsf5	Srsf5	-0.132483128910654	7.72144573674629	10.7077221466781	0.00106669298910989	0.181768590295648
Kmt5a	Kmt5a	0.10359104243788	5.171058714148	10.7058592865529	0.00106791002515564	0.181768590295648
Mycn	Mycn	0.0938887648820658	6.75408801422276	10.7043156794192	0.00106885914467515	0.181768590295648
Mtif3	Mtif3	0.134965273550996	3.63385548940849	10.6941589787777	0.00107474373941584	0.181768590295648
Olfml1	Olfml1	-0.307425541814533	1.91745690197963	10.6894693451281	0.00107805367330913	0.181768590295648
Cdc6	Cdc6	0.281498210319457	1.54773808136491	10.5803180745317	0.00113681417437351	0.182167408778355
Per2	Per2	0.105719376911149	4.90311609037054	10.5881962975482	0.00113811959086446	0.182167408778355
Trio	Trio	0.0713288350592461	8.51926032186265	10.5742188947139	0.00114657571288305	0.182167408778355
Neu4	Neu4	-0.27818552643513	2.54798949019199	10.5594654401166	0.00115596020296589	0.182167408778355
Lrrc27	Lrrc27	0.312855475653434	2.01235751054115	10.5511116623016	0.0011611822860433	0.182167408778355
Gab2	Gab2	0.112488123638386	5.16576347375957	10.5402589938247	0.0011680205740377	0.182167408778355
Ccdc134	Ccdc134	0.21822639407867	2.51983137590193	10.5105713525293	0.00118693550145147	0.182832027797654
Spen	Spen	0.119693994077457	7.37817590801774	10.4417510648039	0.00123198418076295	0.187456910041211
Sox11	Sox11	0.197461050841155	7.93439673269877	10.4139784717	0.00125065016823856	0.188004363390645
Shprh	Shprh	0.0881680557233119	6.59111668521928	10.2597183441786	0.00135966436674542	0.201254103485809
Tbc1d2b	Tbc1d2b	0.0996925835536539	5.47094055201828	10.2443341759748	0.00137105062687399	0.201254103485809
Cdr2	Cdr2	0.15082509256665	4.30016564899912	10.1694806272776	0.00142784491763782	0.203621791156824
Sat1	Sat1	-0.124432425120262	4.98407069331923	10.1669351521396	0.0014298176059172	0.203621791156824
Arpc4	Arpc4	-0.0690190779197355	7.56944641578753	10.1588010117906	0.00143613990717324	0.203621791156824
Nr1d2	Nr1d2	-0.148371978970258	4.93750674308226	10.0966938381486	0.00148535568991116	0.206806771537356
LOC10091	LOC100911	0.803641565654684	9.07515230752856	10.0875208960206	0.00149275802398675	0.206806771537356
Vxn	Vxn	-0.123753091706768	5.66869025110899	10.0425702256167	0.00152963629072358	0.206806771537356
Gpt2	Gpt2	0.113451976276963	5.88429731543353	10.0416805062569	0.0015303752668806	0.206806771537356
Ccdc77	Ccdc77	0.573701831776105	2.70906557079439	10.0283642280718	0.00154147870104786	0.206806771537356
Zfp112	Zfp112	0.226752154110882	2.71453186295653	9.99805719616193	0.00156705459513247	0.208001491313487
Mxra8	Mxra8	-0.180781560750559	3.45205623537972	9.92958923797459	0.00162644582892914	0.211836029887845
Il17ra	Il17ra	-0.176889041655928	4.04003271359565	9.92566475172051	0.00162989972503271	0.211836029887845
Igf2bp3	Igf2bp3	0.251161838801462	3.11281180788167	9.89698616452361	0.00165333991023977	0.211989510026114
Sdha	Sdha	-0.0676050986719882	7.32363757373796	9.88629831572494	0.0016651400162346	0.211989510026114
Chst12	Chst12	0.115348919051834	4.71754649740367	9.85029585309414	0.00169880443950301	0.214005049664555
Srebf1	Srebf1	-0.130341251775978	5.14721497250728	9.81655185706718	0.00172948282388222	0.215480949089228
Fbxl14	Fbxl14	0.10851215236604	5.18223651821807	9.80086759412915	0.00174429557249435	0.215480949089228
Niban2	Niban2	0.0651423078514767	6.31160587165363	9.764681573628	0.00177898259705045	0.217049736879498
Srgap1	Srgap1	0.111575412100813	5.98457318142644	9.75147722805759	0.00179178671945085	0.217049736879498
Slco1a5	Slco1a5	0.894538912464616	1.253481379661	9.69899766698866	0.0018557551706402	0.222638288789209
Pstk	Pstk	-0.153064760743149	3.71712426457751	9.64889459982751	0.00189465564138297	0.223967071939866
Ncs1	Ncs1	0.0827281290146563	7.28293209858543	9.62071909782077	0.00192394385852493	0.223967071939866
Map3k13	Map3k13	0.157626653074062	4.38723968263774	9.60811578641274	0.0019371930674742	0.223967071939866
Amd1	Amd1	0.0741631108065645	7.18526517641372	9.60674222094104	0.00193864260395172	0.223967071939866
Dmgdh	Dmgdh	-0.412437952877177	2.39676105087959	9.58095941057343	0.00196605641008784	0.225050328703358
RGD1305	RGD130546	-0.188807002067061	3.04246947968293	9.53810889506741	0.00201248982943296	0.228271244109367
Cdk2ap1	Cdk2ap1	0.119490567935153	6.49677398800404	9.4939416774408	0.00206151459631519	0.231257820719937
Mafk	Mafk	0.128393893194771	4.23305018593634	9.47365292621731	0.00208444003615769	0.231257820719937
Clic6	Clic6	0.708717546373777	3.30365557380569	9.46488924632181	0.00209442444027834	0.231257820719937
Tfeb	Tfeb	-0.225286008238564	1.98717210892112	9.37622402322233	0.00219817877180021	0.240584881892555
Rnf111	Rnf111	0.079485522589091	5.89382913915686	9.34799786022858	0.00223229201075405	0.242183977549377
Pdik1l	Pdik1l	0.107818457309124	5.53183263990851	9.32816570819291	0.00225658128837139	0.242718651508705
Hoxd3	Hoxd3	2.13015744183397	-0.168907489117801	9.28499987015585	0.00231037938089968	0.244226456781213
Noc3l	Noc3l	0.110562841095338	4.55086871262614	9.27720482759263	0.00232023226449523	0.244226456781213
Zfp111	Zfp111	0.181741387758667	3.44647208503036	9.24114031559303	0.00236637572637678	0.244226456781213
Shisa3	Shisa3	-0.784712276768998	0.991896506734967	9.23528932962893	0.00237380627280237	0.244226456781213
Aplp2	Aplp2	-0.0531262147844405	8.65062204969706	9.2334114944198	0.00237638474544063	0.244226456781213
Btd	Btd	-0.136476736815587	4.1311346862106	9.21315297348918	0.00240262825075549	0.244226456781213
Apoe	Apoe	-0.122149465707683	9.06751257113059	9.20219183004623	0.00241725522136484	0.244226456781213
Ptgfr	Ptgfr	-0.463413582088003	0.517764792884189	9.19468203254094	0.00242719248544285	0.244226456781213
Tifa	Tifa	-0.322993452610344	1.1521701932398	9.14893640452604	0.00248862679140751	0.244492340914498
Echdc1	Echdc1	0.138666595635172	5.00307727974525	9.12374559879454	0.00252312694069622	0.244492340914498
Zfp386	Zfp386	-0.119511287926984	4.67040252810278	9.11711254184276	0.00253229348721574	0.244492340914498
Nedd4l	Nedd4l	0.0762696194935634	7.56614978232919	9.11581437324382	0.00253409117012885	0.244492340914498
Dcbld2	Dcbld2	0.110458536609922	5.33388106249048	9.10312652851428	0.00255172943641453	0.244492340914498
Dtx5	Dtx5	0.172945906530672	4.51637843827337	9.08998240886555	0.00257013344317498	0.244492340914498
Ppm1a	Ppm1a	-0.0673685055123972	6.71258208550248	9.0861525318738	0.00257552123028108	0.244492340914498
Dab2	Dab2	-0.136550734820183	4.72977824604735	9.07820383077987	0.00258659846122575	0.244492340914498
Dusp26	Dusp26	-0.108758932773178	7.01651018599427	9.02547796732882	0.00266242299634338	0.248836852553223
Obi1	Obi1	0.0959372464983303	4.72471403818265	8.99642285398745	0.00270508577403048	0.248836852553223
Nol3	Nol3	-0.172686822041803	4.53888192633994	8.97846697018304	0.0027317974847378	0.248836852553223
Ndfip1	Ndfip1	-0.0495481589225332	9.2410957816583	8.97140777489696	0.00274237215788358	0.248836852553223
Glul	Glul	-0.0863238544959866	8.7293724869319	8.96573394449134	0.00275090250518818	0.248836852553223
Mrpl49	Mrpl49	0.100783249856828	4.72130116413493	8.96485663787564	0.0027522229424016	0.248836852553223
Chga	Chga	-0.0781871298749567	6.94911903170958	8.9479188023023	0.00277785882590642	0.248916672534338
Sdf2l1	Sdf2l1	0.269763757445115	2.84094886507805	8.93196557381468	0.00280222621329795	0.248916672534338
Ptbp3	Ptbp3	0.126170187186134	4.01653648556	8.92498551007939	0.00281295580505263	0.248916672534338
Fam163a	Fam163a	-0.228239640843669	3.27721632925787	8.90667772191262	0.00284129713495602	0.249653974315819
Lrch3	Lrch3	0.0796785187105338	5.77520750087655	8.89321480837154	0.00286232218479444	0.249742701804826
Secisbp2l	Secisbp2l	0.0670184539511961	7.04810398611269	8.87351455486925	0.00289337573135716	0.250688951388884
Crocc	Crocc	-0.154063520738269	5.01334361844795	8.85668737554012	0.00292017043446473	0.251275631109079
Lmod1	Lmod1	-0.53254188787013	-0.0890469596251772	8.84132956362677	0.00294498535026011	0.251663256405448
Anapc2	Anapc2	-0.0642230858433854	7.55644287005248	8.80800695192195	0.00299911480968434	0.254233577557618
Optn	Optn	-0.101041179124478	5.73212843407274	8.79722750160983	0.00301688665426582	0.254233577557618
Col5a2	Col5a2	-0.138873087061436	5.78873204810482	8.75867315512312	0.00307964235165722	0.257883876655216
Gga2	Gga2	0.0717447233942386	5.90214632526555	8.72241679916783	0.00314321099243271	0.258466234022686
Chuk	Chuk	-0.115352751661313	5.03182197182094	8.71362894727616	0.00315839880893597	0.258466234022686
Sostdc1	Sostdc1	0.427618211632314	3.24455211335636	8.70825638732679	0.00316772045135217	0.258466234022686
Mrpl34	Mrpl34	0.0974921125809173	4.98974467153006	8.70725645112542	0.00316945850808257	0.258466234022686
Barhl1	Barhl1	-1.13383719924574	1.41287647072136	8.68010171942376	0.00321703003085075	0.259949247295044
Egfl7	Egfl7	-0.147863072908517	4.2554584772136	8.66231706391476	0.00324857950545108	0.259949247295044
Timeless	Timeless	0.134906222437318	4.03634829555194	8.65247034652582	0.00326618220822232	0.259949247295044
Vwa3a	Vwa3a	-0.238949282729985	3.70024144404862	8.64301507928421	0.0032831763617574	0.259949247295044
Nelfb	Nelfb	0.0719487007950723	6.91264521987579	8.63476661587912	0.00329807488415317	0.259949247295044
Tle3	Tle3	-0.0949155479585051	4.60466616246716	8.61895899145751	0.00332681921572514	0.259949247295044
Cd2ap	Cd2ap	-0.142246786462674	3.44299630075727	8.6023755582062	0.00335724802965584	0.259949247295044
Rnf182	Rnf182	0.159991875754213	4.55055961501084	8.58091526377075	0.00339704611860393	0.259949247295044
Wdr91	Wdr91	-0.0971488049136163	5.0515528664989	8.57370867225742	0.00341051752725933	0.259949247295044
Ppl	Ppl	-0.516189391938461	-0.486002741161212	8.5622855120731	0.00343198300213143	0.259949247295044
Atp1b1	Atp1b1	-0.0835632029705987	9.4588289270817	8.55340412433096	0.00344876705184545	0.259949247295044
Bach1	Bach1	0.0966852892066702	5.54221681159893	8.53655308634241	0.00348094188038999	0.259949247295044
Rgs19	Rgs19	0.113984798454112	4.53308668828937	8.50153441549462	0.00354947112445183	0.259949247295044
Dcaf11	Dcaf11	-0.0600731751015371	6.52020072976567	8.45341099058392	0.00364359442838865	0.259949247295044
Insc	Insc	0.610007331182743	-0.625093645818614	8.44790209862417	0.00365464775265319	0.259949247295044
Rnf112	Rnf112	-0.107675827205127	7.11957807800517	8.4436989153829	0.00366312832958962	0.259949247295044
Tmem135	Tmem135	-0.0769083479437327	5.65554486571274	8.43824009132352	0.00367411650632506	0.259949247295044
Fam107b	Fam107b	0.112344749514211	5.59600678662953	8.43481715337098	0.00368103892280852	0.259949247295044
Cyb5a	Cyb5a	0.126175637918553	4.88909345791578	8.42911517447421	0.00369259987543814	0.259949247295044
Podxl	Podxl	0.0943262810522007	6.22337671564914	8.42705173504186	0.00369679265793546	0.259949247295044
Fnip2	Fnip2	0.102019729813304	5.3988902827677	8.41694484917013	0.00371760374697371	0.259949247295044
Ciao3	Ciao3	-0.0962007223074867	4.38390208096852	8.41282773049259	0.0037258269871406	0.259949247295044
Ercc3	Ercc3	-0.0780901826058359	5.38008423765752	8.40370266786012	0.00374457528497527	0.259949247295044
Zfp786	Zfp786	0.142629495384835	4.38698422394798	8.40319160517251	0.00374562914389094	0.259949247295044
Alyref	Alyref	0.0932251072311242	6.36835256148271	8.40018258898627	0.0037518332257122	0.259949247295044
Hoxb3	Hoxb3	2.1084231760818	-0.312683051593486	8.39305454941611	0.00376657419320984	0.259949247295044
Ubap2	Ubap2	0.0638188729503173	7.00636890801795	8.39164702750503	0.00376949194005772	0.259949247295044
Vsnl1	Vsnl1	-0.112324826538806	7.36118421461653	8.38885154197491	0.0037773217258847	0.259949247295044
Slc66a2	Slc66a2	0.0759705395477332	5.61365890198268	8.38090387351564	0.00379183802257739	0.259949247295044
Des	Des	-0.235055769198652	1.0566165205487	8.34550417766444	0.00386642857761945	0.262079893687497
Cd24	Cd24	0.0809543700375286	9.70548518188017	8.34199375252546	0.00387390626268061	0.262079893687497
Armc7	Armc7	0.151217014546529	3.76070863150243	8.33636240684099	0.00388593254245306	0.262079893687497
Acadvl	Acadvl	-0.113288211967652	5.01244710520317	8.32199858784219	0.00391678014995327	0.262740139413801
Mt2A	Mt2A	-0.264466684529743	2.61247189830032	8.30663528316425	0.00395005007400655	0.263554965312181
Slc35e3	Slc35e3	0.0653005348318648	6.22825991140755	8.29828201786611	0.00400779037329752	0.265985108976772
Crebzf	Crebzf	-0.083765268153274	6.65547852446228	8.2672079880779	0.00403675370809175	0.266489820189739
Rc3h2	Rc3h2	0.12918580189773	5.47334863457585	8.2119662621518	0.00416150318549736	0.271105594946204
Grid2ip	Grid2ip	-0.272808404143255	2.91116180870839	8.21184949559676	0.00416177786788624	0.271105594946204
Ube2b	Ube2b	-0.0634543289455126	6.91326564737227	8.20745686066346	0.00417185815738329	0.271105594946204
Chp2	Chp2	-0.52133144263936	-0.316712795762565	8.18904407944218	0.00421441246980696	0.271163805010867
Ddhd2	Ddhd2	-0.118631903561557	5.29899939569246	8.18630137317857	0.00422078889495662	0.271163805010867
Chst7	Chst7	-0.167006327579587	3.95717851580468	8.17890938219439	0.00423795319204288	0.271163805010867
Bap1	Bap1	-0.0435731235352024	8.236458600977	8.16671477047532	0.00426661166239012	0.271604661794089
Srebf2	Srebf2	0.0624078465200831	8.29768789353368	8.12672657703487	0.00436174261748604	0.274148924710531
Gfod2	Gfod2	0.103632753982504	5.42619668896123	8.12102264397742	0.00437548702780515	0.274148924710531
Ccdc151	Ccdc151	-0.375679482908802	0.461103876268265	8.11385876559806	0.00439281186592338	0.274148924710531
Tmem254	Tmem254	-0.12892290452479	5.31058000897069	8.11317520019108	0.00439446861762492	0.274148924710531
Oaf	Oaf	-0.143137730880365	4.15820792138678	8.07966685731057	0.00447639332224139	0.277068676688401
Pcdha12	Pcdha12	0.217721380270486	3.42795706358858	8.07593980550399	0.00448569834728746	0.277068676688401
Nr4a1	Nr4a1	-0.202077317041279	3.68406584408097	8.06220920860117	0.00451965422977676	0.277610638595644
Eng	Eng	0.11215558731517	4.90413225580876	8.05454770946658	0.00453895730331894	0.277610638595644
Epc1	Epc1	-0.0660479095114479	6.46226348760772	8.04477829151409	0.00456350034325609	0.277750213574865
Ntn3	Ntn3	0.110938047237999	4.87717369881678	7.99663733488977	0.00468642022092508	0.283487389058024
Eml5	Eml5	-0.106465722036066	5.16483878467527	7.97884940421764	0.00473008765787716	0.283487389058024
Rnf38	Rnf38	0.0970134874026089	5.33277625764304	7.97219564186693	0.00475012872822756	0.283487389058024
Dram2	Dram2	0.137402974678846	3.89128332749407	7.96257783965009	0.00477542524972304	0.283487389058024
Rnpepl1	Rnpepl1	0.100308138749865	5.38599692658365	7.95311202354659	0.00480047587181557	0.283487389058024
Mgat2	Mgat2	0.0814661966727096	5.65277356481046	7.9467489091411	0.00482271622581773	0.283487389058024
Calb2	Calb2	-0.149853280440145	6.72564975505322	7.92828137065631	0.00486653122132608	0.283487389058024
Panx1	Panx1	0.0725714915976103	6.23953893983961	7.92240730411016	0.00488262627278941	0.283487389058024
Chgb	Chgb	-0.0862980468300845	7.35423888189468	7.91774339166599	0.00489522925769224	0.283487389058024
Abcf3	Abcf3	-0.0672258281232659	6.05536796937172	7.91361617176626	0.00490640961292683	0.283487389058024
Bop1	Bop1	0.0619775267270218	6.46894557781954	7.9059644370191	0.00492720651808932	0.283487389058024
Slc4a7	Slc4a7	0.0761708662717896	5.13147026668877	7.90478760116372	0.00493041303824751	0.283487389058024
Ctsh	Ctsh	-0.129636710428675	4.47405685730808	7.8821822774611	0.00498965755573144	0.285577785875283
Phf2	Phf2	0.0702916598717075	6.81704322749691	7.85481670620979	0.0050686549062557	0.288182251482764
Plin2	Plin2	0.163453068482829	5.64416332670203	7.84623135215256	0.00509267586421112	0.288182251482764
Xpo7	Xpo7	0.0582487535505182	7.00921252725315	7.83107688054764	0.00513558497344139	0.288182251482764
Rere	Rere	0.0657900070050181	7.63164755969898	7.82724374028325	0.00514545192064913	0.288182251482764
Isg15	Isg15	0.29816327117415	0.761963813326169	7.81821775059991	0.00517221761141783	0.288182251482764
Zswim5	Zswim5	0.0975954810910132	5.11519485862814	7.80795134832979	0.00520169457103134	0.288182251482764
Smarca4	Smarca4	0.0576652552976937	8.79270220118094	7.80488723101328	0.00521051176227115	0.288182251482764
Nkain3	Nkain3	0.187756740486204	3.2775135472672	7.79965372569575	0.00522562476946085	0.288182251482764
Adamts10	Adamts10	-0.110997104898989	4.82928702379326	7.7926431554172	0.00524303687477658	0.288182251482764
Lrrc23	Lrrc23	-0.142788808056096	4.21403917913698	7.78212071936824	0.00527658192154491	0.288754002785596
Ash1l	Ash1l	0.0686891453323589	7.32412781631605	7.75357472151073	0.00536063285588802	0.291265864671939
Zfp398	Zfp398	0.0975377548961969	4.35823470327076	7.75070047681153	0.00536917118494398	0.291265864671939
Ubqln1	Ubqln1	0.0622880842512615	7.58866330799273	7.73077427329774	0.00542874645907303	0.292578915851826
Itga8	Itga8	-0.196743756044738	3.5950732390519	7.72894413656695	0.00544027478567152	0.292578915851826
Phc3	Phc3	0.126866389879921	4.61217988010004	7.70579030672301	0.00552439684018898	0.294527017288322
Smc5	Smc5	-0.0685757687101929	6.28724206261447	7.6994683212701	0.00552370938891298	0.294527017288322
Chmp7	Chmp7	-0.0491944926829943	7.48026780724047	7.68517560609106	0.00556762620014418	0.295381982170967
Mllt3	Mllt3	0.11852691190162	5.77353013153478	7.67293274548109	0.00560552754032163	0.295381982170967
Cipc	Cipc	0.0667409133299508	6.43429663883542	7.66568409272251	0.00562809176462952	0.295381982170967
Pcdhb6	Pcdhb6	0.506104893870383	1.91353944128171	7.63753803464638	0.00571658899415838	0.295381982170967
Gas8	Gas8	-0.142573885010785	3.62969424364082	7.62929235614105	0.00574278302752833	0.295381982170967
Gpr139	Gpr139	0.236284901822679	2.07258445650409	7.62081816438217	0.00576983005498396	0.295381982170967
Rbm33	Rbm33	-0.0732862496512388	6.45968328878061	7.61267607933045	0.00579559404847475	0.295381982170967
RGD1359	RGD135682	-0.336114909415793	0.0122822688935374	7.60213942725569	0.00582990828429736	0.295381982170967
Rcor1	Rcor1	0.101783084568376	5.51125855322687	7.60123214233651	0.00583294264644016	0.295381982170967
Arid1a	Arid1a	0.0613158605312823	8.1629018840469	7.59873930460282	0.00584091279990186	0.295381982170967
Acer2	Acer2	0.225896013777635	2.58453632396974	7.59376653046825	0.00585704539171104	0.295381982170967
Ubqln2	Ubqln2	0.0520561035899898	8.34613210327378	7.59009778519429	0.00586897659981098	0.295381982170967
Prr11	Prr11	0.2977339148866	1.23422891771894	7.57804425908525	0.00590835097092129	0.295381982170967
Sgcz	Sgcz	0.392571526245565	0.124232788761018	7.5720068805128	0.00592620085771592	0.295381982170967
Mgst3	Mgst3	-0.0944193360082713	6.98906290136245	7.57200112265114	0.00592819297148299	0.295381982170967
Septin11	Septin11	0.0693671372285255	7.17793007977363	7.56661769142954	0.00594592615157638	0.295381982170967
Plpbp	Plpbp	0.0898951557034245	4.52785221567132	7.55693746467952	0.0059779493766112	0.295381982170967
Prkag2	Prkag2	0.0652046192520827	6.56963480191569	7.54758016947634	0.00600907157887808	0.295381982170967
Lrrcc1	Lrrcc1	-0.0940964269567336	4.44146356867158	7.54256273136765	0.00602582752038896	0.295381982170967
Tecta	Tecta	-0.433913587060407	0.564524590910411	7.54172459925056	0.00602863115312578	0.295381982170967
Cbfa2t3	Cbfa2t3	0.119403802041183	5.04019134757175	7.53458243496563	0.00605254275839125	0.295381982170967
Pld2	Pld2	-0.104277372028849	5.29206551519078	7.53220325436597	0.00606057445185344	0.295381982170967
Tpm1	Tpm1	-0.0579452760338603	7.41463706272439	7.51664932517359	0.00611312854204669	0.296784065464033
Impact	Impact	-0.109099973005807	6.96458881319148	7.50659329513397	0.00614725347028478	0.297288873657144
Zbed4	Zbed4	0.092750751886482	5.24543764822843	7.46915352903893	0.00627650550960846	0.302325525219295
Sfxn1	Sfxn1	-0.062183027472966	7.00283194688543	7.46243788917422	0.00629996306466002	0.302325525219295
Zbtb39	Zbtb39	0.0950405682989169	4.94436472372614	7.4460944971772	0.00636237177464594	0.304150622112098
Bicra	Bicra	0.100186803328889	6.21651127902449	7.42334109124868	0.00644026357826969	0.30451329689459
Ugt8	Ugt8	-0.157315123721719	3.52783309015474	7.42881201575915	0.00641877030591866	0.30451329689459
Erich1	Erich1	0.150489964853968	3.29489890937318	7.36154484630559	0.00666335170598496	0.310488356048392
Slc20a2	Slc20a2	-0.0848159716095608	5.25741351947412	7.36013520200043	0.00666685777110585	0.310488356048392
Ubb	Ubb	0.0706751243050203	10.3330621151951	7.35589529739077	0.00668483216431361	0.310488356048392
Grin2b	Grin2b	0.180946737556304	5.94193737646743	7.35270348927153	0.00669619874351032	0.310488356048392
Zfp362	Zfp362	-0.103249029046103	5.68057835231257	7.34623316064327	0.00672034178195059	0.310488356048392
Cdh3	Cdh3	0.276783872594053	2.1448758070018	7.34260231217621	0.00673392865557338	0.310488356048392
Thnsl1	Thnsl1	0.132558815883273	3.79007252122002	7.33869058493494	0.00674863552091908	0.310488356048392
Pak5	Pak5	0.0757638326046112	6.67578632760565	7.33787270155676	0.00675166925168335	0.310488356048392
Cdk4	Cdk4	0.0720496145502898	7.55593139325326	7.32494033165906	0.00680042219508389	0.310488356048392
Tmem38a	Tmem38a	-0.0895611700064356	4.94220572122483	7.31717483899926	0.00682986946780446	0.310488356048392
Cd74	Cd74	-0.481666282831918	-0.264391347293886	7.30485380484507	0.00687685917433671	0.310488356048392
Fat4	Fat4	0.0977713315691514	7.7363389871508	7.29881520549636	0.00689617029123705	0.310488356048392
Znrd1as1	Znrd1as1	0.418832682685866	0.958810680774965	7.29325242695471	0.00692102088441307	0.310488356048392
Rai14	Rai14	0.0976264110362053	4.87714303611476	7.2898884758569	0.00693445525636728	0.310488356048392
Cilp2	Cilp2	0.114988621036899	5.54762161610729	7.28794763380435	0.00694187355365206	0.310488356048392
Pglyrp1	Pglyrp1	-0.379676320705435	0.115990584120585	7.28768848771291	0.00694287499699458	0.310488356048392
Rptor	Rptor	0.0653585488795249	6.40955253624163	7.27228702514592	0.00700222931736184	0.31089407773483
Midn	Midn	0.0747759412071603	7.76368014356071	7.26520689720577	0.00703031685970366	0.31089407773483
Zfp84	Zfp84	0.109486068169954	5.14720309732053	7.25434115882162	0.00707298165047745	0.31089407773483
Pnp	Pnp	-0.105492407403636	5.46934746327886	7.24373823690653	0.00711489900734271	0.31089407773483
Gls	Gls	-0.0647924809713623	7.9932752567583	7.23869211665115	0.007134993466099	0.31089407773483
Maml1	Maml1	0.0927185031275009	4.79745942702719	7.23453657530185	0.00715142675385759	0.31089407773483
Elavl3	Elavl3	0.0656412676676814	8.20713666329822	7.23225309302116	0.00716012966584147	0.31089407773483
Plekhg2	Plekhg2	0.0964556451826525	5.27745674013236	7.22169864238273	0.00720275161243761	0.31089407773483
Trim36	Trim36	0.14608629781385	4.28860824773695	7.21825465143781	0.00721256502773512	0.31089407773483
Hyal2	Hyal2	0.0886323900636607	4.9413211242331	7.21523564362449	0.00722873236187384	0.31089407773483
Pwp1	Pwp1	0.0883048088375458	5.19635014690115	7.21080238327225	0.00724660907641052	0.31089407773483
Osbpl2	Osbpl2	0.0521984511776967	6.56483353607799	7.20361553068672	0.00727568543408571	0.31089407773483
Ptrhd1	Ptrhd1	-0.151478073416799	3.00770339396287	7.18246337287596	0.00736195587882791	0.31089407773483
Prkcd	Prkcd	-0.166781565887948	4.49889242954408	7.18137567113151	0.00736642040886152	0.31089407773483
Zfp799	Zfp799	0.115235234676073	4.72974619448762	7.17799378034007	0.00738031882059666	0.31089407773483
Pum3	Pum3	0.0903246465956231	6.11541257654799	7.17691222259197	0.00738475942786786	0.31089407773483
Virma	Virma	0.0825853467766246	5.82050893286742	7.17484924103478	0.00739326603898966	0.31089407773483
Fignl1	Fignl1	0.266534246181298	1.23294494671455	7.15877813500911	0.00745979955977762	0.31089407773483
Cbx7	Cbx7	-0.238686706706147	1.86643688823419	7.15595838297401	0.00747153614026182	0.31089407773483
Cmtm5	Cmtm5	-0.210112103096721	2.60864438499461	7.15568384478655	0.00747267985121183	0.31089407773483
Chrd	Chrd	-0.141557061523645	4.19473888845224	7.15507611508598	0.00747521225618729	0.31089407773483
Rbc3	Rbc3	0.0925478234521523	5.82040629284972	7.12457259059399	0.00760345381880468	0.315177054143608
Stk36	Stk36	-0.0949158391335064	4.64872459506579	7.11387922326541	0.00764894095489569	0.315838866752382
Fut4	Fut4	0.176541509563745	3.04143248956586	7.10893955529427	0.00767004701658827	0.315838866752382
Cptp	Cptp	0.120978242459536	4.357334254462	7.09400414183224	0.00773422550183764	0.316772134760377
RGD1565	RGD156561	0.0702967079988429	7.08790633801404	7.08917177082609	0.00775510788114356	0.316772134760377
Dld	Dld	-0.0614802702152651	6.88838208091482	7.08599283949169	0.00776887659186305	0.316772134760377
Gtpbp3	Gtpbp3	-0.105809720384835	3.95016578034716	7.07819633881392	0.00780275102171447	0.317117017908572
Kpna2	Kpna2	0.0924457050012106	6.25369883141323	7.06307638456931	0.00786887568952115	0.318766110318686
Utp23	Utp23	0.176906619735257	2.88803221542433	7.03762115267069	0.00798149736250527	0.322282014860771
Ccsap	Ccsap	-0.119293826673112	4.46709236933918	7.02678843219877	0.00802992337449443	0.323106934843852
Jakmip2	Jakmip2	0.066107554756994	6.66965553335372	7.01865495115972	0.00806648029117191	0.323106934843852
Vegfd	Vegfd	0.300799066463363	1.56996436078588	7.00875280951379	0.00811121654747894	0.323106934843852
Dnajb12	Dnajb12	-0.0690296026474521	5.19703213020333	7.00842687180033	0.00811269338722433	0.323106934843852
Blvra	Blvra	-0.188908848968439	4.16303223971253	7.00420181836336	0.00813140799398649	0.323106934843852
Mfsd14b	Mfsd14b	0.078547417421741	5.325510888917	6.99408380729536	0.00817795562580988	0.323924929468841
Naa15	Naa15	0.074717712922031	7.02600467858363	6.986915881289	0.00821123012723001	0.324213665498256
Dnajc16	Dnajc16	-0.0825939673714842	4.660601956284	6.97389494610899	0.00827072798715514	0.325532722699479
Gpm6b	Gpm6b	-0.0586174543817155	8.33281315822505	6.96761305336767	0.00829981376268541	0.325650240775365
Mis18a	Mis18a	0.435251701330149	1.16078622936126	6.95639280580834	0.00835207199829554	0.326045605019714
Bag3	Bag3	-0.234797212182185	1.9720668192397	6.95422449836349	0.00836215385159161	0.326045605019714
Plxdc1	Plxdc1	0.186994745431095	2.09903894797375	6.92878853788449	0.00848191233928039	0.329684798309039
Etv1	Etv1	-0.0809076864980107	6.79321420447509	6.91164365888347	0.00856362235835398	0.330672219054518
Rasd2	Rasd2	-0.119942908858289	5.13171563878647	6.91001835582255	0.00857141109731718	0.330672219054518
Cracr2b	Cracr2b	-0.154371654898493	2.79668043625209	6.90680565542137	0.00858682627802066	0.330672219054518
Mapk13	Mapk13	0.697451728081429	-0.49864587165235	6.89961687921996	0.00862142339085605	0.332080372000034
Baz1a	Baz1a	0.18249190322376	2.69316441819815	6.87492431447856	0.00874133613394931	0.334532642344151
Lrrc14b	Lrrc14b	0.19866980385237	1.91031154446246	6.86857859737905	0.00876753234323453	0.334532642344151
Hook1	Hook1	-0.0739349574433639	5.98470518203663	6.85408399613258	0.00884393990245153	0.336416400941699
Clasp2	Clasp2	-0.0438119158465174	9.57958967503732	6.84550688061221	0.00888644824580064	0.337009771317943
Rnf41	Rnf41	0.0468025926825225	7.13542207728892	6.83250197964003	0.00895137906797658	0.338443534186678
Emc4	Emc4	-0.0747964230984426	5.35418637878251	6.81212342256552	0.00905410350543569	0.341293200716982
Flna	Flna	-0.0839232835985101	8.00473724793999	6.79942576647173	0.00911871959325277	0.342693567364503
Ccdc24	Ccdc24	-0.359622176765757	0.163969737216367	6.79826159686103	0.00918569377184692	0.343355582064932
Rad51	Rad51	0.176768546644822	2.42567802855964	6.79525822126464	0.00919137230584334	0.343355582064932

Mcmbp	Mcmbp	0.0727457812717189	6.26675986975641	6.7779985075665	0.00922883185929259	0.343725776462966
Ppp1r14b	Ppp1r14b	0.0759725323528632	7.59974025992952	6.75233056528938	0.00936253117404136	0.34505150721885
Tnrc6b	Tnrc6b	0.10213205634271	7.08027877173576	6.74876492662338	0.00938126019816118	0.34505150721885
Fbxo36	Fbxo36	-0.385393257852552	0.0429178017608596	6.74018867843408	0.00942646548178042	0.34505150721885
Taf3	Taf3	0.0898101757161661	4.83771257820645	6.73899103342264	0.00942750952220332	0.34505150721885
Lyst	Lyst	0.0865473918293587	5.70941566296635	6.73645512374075	0.00944621464881404	0.34505150721885
Tma16	Tma16	-0.136236989094441	3.28299162161564	6.73225287815292	0.00946849372828786	0.34505150721885
Igdcc3	Igdcc3	0.132457289754035	3.682128020904	6.72943876589455	0.00948344341521633	0.34505150721885
Arhgap39	Arhgap39	0.0534204829283041	6.85205850139767	6.72072336049343	0.00952988671532972	0.34505150721885
Farsb	Farsb	0.0717157221960672	6.05403208089256	6.71725936090844	0.00954842458528893	0.34505150721885
Papola	Papola	0.0625583351192906	7.45599161274258	6.7124503140265	0.00957420789728336	0.34505150721885
Gatm	Gatm	-0.105634103080463	6.72230943681017	6.70202102348416	0.00963036904839693	0.34505150721885
Arl13b	Arl13b	-0.102993230253779	4.71299431798691	6.69643295032899	0.00966059927053143	0.34505150721885
Cnmd	Cnmd	0.814043086885882	-0.665172123999659	6.69619826005635	0.00966187101758555	0.34505150721885
Wnt16	Wnt16	-0.379627164681041	0.449589744388988	6.69370373264434	0.00967539904723799	0.34505150721885
Rprd1b	Rprd1b	0.078279832136962	5.34768832279008	6.69299390885632	0.00967925202585536	0.34505150721885
Plcl1	Plcl1	0.0841588074088581	5.26022130749713	6.67852103782022	0.00976911102394705	0.346358144161989
Sugp1	Sugp1	-0.0766160920880666	5.66873945743475	6.67809883874319	0.00977142476116214	0.346358144161989
Emc7	Emc7	-0.0735568750965748	5.33210489628932	6.66632754160656	0.00982514288942216	0.347057027387935
Cnih2	Cnih2	0.095009991284776	9.263223565803	6.66240870006261	0.00984577307809	0.347057027387935
Kdm1a	Kdm1a	0.055700697836646	7.32232172967672	6.64860778923258	0.00992233329042297	0.347992858642478
Mest	Mest	0.100467312843852	5.77757015725804	6.64757158549278	0.00992910616948964	0.347992858642478
Lrrc34	Lrrc34	-0.260071048952569	1.85315508521691	6.63527769149508	0.00999786092502523	0.349421038897226
Aldoa	Aldoa	-0.0626205534633423	8.99251285676335	6.61709405225452	0.0101004504873077	0.352020448967912
Zmynd10	Zmynd10	-0.14094954705468	4.35328303670732	6.58976515531802	0.0102566700835449	0.356489283098578
Nop53	Nop53	-0.0862790785405907	6.61023860037642	6.57203298037891	0.0103593553600216	0.359037984254414
Igf2bp2	Igf2bp2	0.146113685189776	4.10385234316069	6.56663624513864	0.0103908158165614	0.359130772695947
Dnajc25	Dnajc25	-0.270267988530272	3.6104198258437	6.56117886108126	0.0104227296611592	0.359238669459345
Mis12	Mis12	0.117294077862308	4.02124697880556	6.55189747518949	0.0104772366211694	0.359490566676711
Hsd11b1	Hsd11b1	-0.22279627520152	2.39823030383454	6.5501277928182	0.0104876626008113	0.359490566676711
Clptm1l	Clptm1l	0.0499336242395672	7.21927132047351	6.54459058530024	0.0105203525092725	0.359623152699161
Ccm2	Ccm2	0.0616085837560457	6.01626881377728	6.52504114536594	0.0106366088249501	0.362603738889814
Leng1	Leng1	0.166760846825591	2.79719564061927	6.50740986449375	0.0107425866457296	0.364369755814622
Lmnb1	Lmnb1	0.0744174553750689	6.94447890306107	6.50394239282786	0.0107635567085372	0.364369755814622
Trabd	Trabd	0.0872251539244115	5.12656850822992	6.50188425935251	0.0107760230770614	0.364369755814622
Rbm3	Rbm3	-0.0874744861680603	7.92877708306931	6.4727054570502	0.0109543647525329	0.367788890177975
Mideas	Mideas	0.0978992108770955	4.17659078966534	6.47026589989291	0.0109694121222903	0.367788890177975
Cytl1	Cytl1	-0.492993472628233	-0.230602721560091	6.46918743750872	0.0109760705788804	0.367788890177975
Hace1	Hace1	0.0726316543871017	5.14123546069621	6.4617088816718	0.0110223592287817	0.367788890177975
Mir1249	Mir1249	-0.341038369038667	0.229996443306851	6.46125916259051	0.0110245287269827	0.367788890177975
Mink1	Mink1	-0.0816306940866943	7.52518236375178	6.44904524388465	0.01110119954574	0.369160854089662
Snrpn	Snrpn	-0.24512147235848	0.823687255235451	6.44060585898296	0.0111540610704858	0.369160854089662
Slc25a11	Slc25a11	0.0510981843892982	6.4109422873101	6.44054921521258	0.0111544154830345	0.369160854089662
Lnx2	Lnx2	0.121157373309913	4.34283490461214	6.42551947251268	0.011249200443939	0.371312894018588
Samd4a	Samd4a	0.158860794998396	2.71053941331342	6.41387685207338	0.0113231924165596	0.372769054822226
Tmem18	Tmem18	-0.0807048841875255	4.53224005412792	6.40254854666811	0.0113956663199286	0.373253196769985
Cfap70	Cfap70	-0.187624700123222	2.87342093073762	6.39886003516906	0.011419366526654	0.373253196769985
Crtapl1	Crtapl1	-0.174789880053694	2.30345462804331	6.39423533131365	0.0114491536542532	0.373253196769985
Htr1a	Htr1a	0.193050155408503	2.05124253767018	6.39256412496874	0.0114599468770532	0.373253196769985
Plcd4	Plcd4	-0.144550210117564	3.17642653998824	6.38018397625294	0.0115401468614884	0.373253196769985
Maip1	Maip1	-0.0930207192282356	4.51092121261092	6.37479387442078	0.0115752485685407	0.373253196769985
Pklr	Pklr	-0.175464124023493	2.33511051801695	6.37455016669762	0.0115768838042261	0.373253196769985
Ralgapa1	Ralgapa1	-0.0450447505955542	7.28887798889921	6.35091649470812	0.0117320673332358	0.373253196769985
Tab1	Tab1	0.0636774202712081	6.09062153262138	6.34530471914701	0.0117692390687548	0.373253196769985
Ascl1	Ascl1	0.135037336547013	4.16687646896914	6.34291325328013	0.0117851166084855	0.373253196769985
Pitpnb	Pitpnb	0.0472946457933943	6.91563272099496	6.33311382128355	0.0118504075361637	0.373253196769985
Smarca1	Smarca1	0.0703703728807376	6.44284166515788	6.3327327255666	0.0118529541623789	0.373253196769985
Cldn2	Cldn2	0.495955708888123	2.31621706079779	6.32826277329346	0.0118828660642987	0.373253196769985
Smim3	Smim3	-0.106267482804494	3.54655802472032	6.31899725531925	0.0119451158090404	0.373253196769985
Itprid2	Itprid2	-0.0885613204803239	4.55554057046045	6.31784042598328	0.011952911480258	0.373253196769985
Bicd2	Bicd2	0.0582176889035019	7.0260952036396	6.30752559894037	0.0120226518099111	0.373253196769985
Nrip3	Nrip3	-0.112421115221702	6.08592434844775	6.30603499448748	0.012032754546667	0.373253196769985
Plxna4	Plxna4	0.0792798945556462	8.24350993087995	6.29776119586286	0.0120890556765748	0.373253196769985
Ccdc65	Ccdc65	-0.0847531089184421	4.5046767856144	6.29707650460556	0.0120937261025854	0.373253196769985
Eppin	Eppin	0.234412915283022	1.48562239220104	6.29686841076679	0.0120951458222782	0.373253196769985
Sucla2	Sucla2	-0.056638976518699	6.50369421979472	6.29643310483026	0.0120981165587905	0.373253196769985
N6amt1	N6amt1	-0.145313367138205	2.82948773359431	6.29612571212407	0.0121002147348472	0.373253196769985
Ptprj	Ptprj	0.071437082959068	6.26283636631705	6.29066378321613	0.012137558706485	0.373253196769985
Nf1	Nf1	0.0687319308644875	6.82404140808381	6.29065749249197	0.0121376017852336	0.373253196769985
Uckl1	Uckl1	-0.0929852526469059	4.87863274443372	6.28852523406873	0.0121453580340468	0.373253196769985
Mical2	Mical2	-0.147554010812702	5.44179958241512	6.28189944377464	0.0121979357619198	0.373253196769985
Ppp1r2	Ppp1r2	-0.0626656078901807	6.61114517412159	6.28112201934163	0.0122030813658962	0.373253196769985
Cdk12	Cdk12	0.0935806342568265	5.26171137035063	6.2805642995262	0.0122069224109874	0.373253196769985
Jam3	Jam3	-0.0929179068589664	4.43358484535208	6.26840799692148	0.0122909527397062	0.373253196769985
Syt10	Syt10	-0.270875420959121	0.91312200825552	6.25854292580183	0.0123595816005531	0.373253196769985
Micb	Micb	0.237251789917598	1.61680953451455	6.25757883289682	0.0123663094281081	0.373253196769985
Glb1	Glb1	-0.118653273256744	4.4510815444337	6.24614227028801	0.0124464087127147	0.373253196769985
Pafah1b3	Pafah1b3	0.0630315998968798	7.06350550247542	6.24407376519798	0.0124609526757443	0.373253196769985
Hoxc4	Hoxc4	2.02455572526024	0.326540721910015	6.24083212172513	0.012483780793968	0.373253196769985
Dnajc22	Dnajc22	-0.46593023655835	-0.318602234643969	6.23828461221582	0.0124939176006553	0.373253196769985
Eea1	Eea1	0.105363621398315	5.0383020441148	6.23777222479787	0.0125053682596659	0.373253196769985
Il23a	Il23a	-0.348972912843432	0.0867571251724795	6.23211448728289	0.0125453844794578	0.373253196769985
Atp6v0e1	Atp6v0e1	0.116111711811914	3.45163229286375	6.2316983604811	0.0125483257770468	0.373253196769985
RGD1560	RGD156010	0.0617811340859967	8.67747079805545	6.22813005564875	0.0125736446252901	0.373253196769985
Tnxa-ps1	Tnxa-ps1	-0.436798310090117	-0.154454929510519	6.22580315881281	0.0125899765737757	0.373253196769985
Get1	Get1	0.0534557058056608	7.06536054738594	6.22326800773574	0.0126075913958893	0.373253196769985
Shoc2	Shoc2	0.0667681451422294	6.58368028838536	6.21598028474287	0.0126602231232435	0.373253196769985
Sacm1l	Sacm1l	-0.0872407953920163	5.74868451499517	6.21076067937598	0.0126976059959756	0.373253196769985
Kbtbd7	Kbtbd7	0.109325004523263	5.65198247347225	6.21038801521188	0.0127002788269622	0.373253196769985
Maz	Maz	0.0776529588416436	8.21699126803362	6.20812746285244	0.0127093258704359	0.373253196769985
Pcsk4	Pcsk4	-0.223074862072406	2.36428500012372	6.20847621428122	0.0127140024546961	0.373253196769985
Supt5h	Supt5h	-0.0440093983428502	7.76369100148285	6.20206464345858	0.0127601381489817	0.373728271560668
Net1	Net1	-0.0924017280656458	5.06828678743412	6.19568785679049	0.0128061942165583	0.374188794473064
Gpcpd1	Gpcpd1	-0.0889942034435537	6.18865732347449	6.18440065108133	0.0128881348667245	0.375481795596659
Tpst2	Tpst2	0.0890368845995038	4.25142267847647	6.17784655427321	0.0129359620578768	0.375481795596659
Faim2	Faim2	-0.0727361229449937	6.20654443658659	6.17724184236537	0.0129403840752235	0.375481795596659
Prdm2	Prdm2	0.0693718191642681	6.58202458329553	6.17089080184141	0.0129869196799201	0.375957772889473
Rps6kl1	Rps6kl1	-0.0793209404614581	6.42298324525815	6.15894891418361	0.0130674982310919	0.376170004432608
Bnip3	Bnip3	-0.0564521217110552	6.26753908663075	6.15832094012228	0.0130795308052163	0.376170004432608
Tab2	Tab2	0.0756210918258919	6.69170353042739	6.15762225150061	0.0130846983989542	0.376170004432608
Hip1r	Hip1r	-0.0720643187918309	6.51609945434647	6.15309019538415	0.0131182690882249	0.376207008762778
Ptprcap	Ptprcap	-0.361123982784813	-0.312093499993605	6.13623537132549	0.0132438982603582	0.376207008762778
Egr1	Egr1	-0.197377780668586	3.97578688832189	6.13119683489204	0.0132816988800217	0.376207008762778
Pgpep1	Pgpep1	0.0801044078729841	5.19033655372665	6.13020249915423	0.0132891648376041	0.376207008762778
Smr2	Smr2	0.142950245263826	4.64640891201984	6.13000800495428	0.013290626987403	0.376207008762778
Htatsf1	Htatsf1	-0.0684182238497199	6.66017009020682	6.12987581285	0.0132916207109442	0.376207008762778
Rbm14	Rbm14	0.0791086575740687	6.41646135965829	6.12915380745348	0.013297049840858	0.376207008762778
Man1b1	Man1b1	-0.0487546516148226	7.32063082436703	6.12245050203196	0.0133475644316942	0.376781813054408
Nfya	Nfya	0.123342727917089	5.29298212597314	6.11825052851325	0.0133785581168876	0.376804220371122
Etv6	Etv6	-0.167188671071741	2.84288968752287	6.10987443376648	0.0134428578870814	0.377305296080036
Sgk1	Sgk1	-0.0894779769036768	5.70290476947243	6.10803104151768	0.0134568960961517	0.377305296080036
Ppp1r16a	Ppp1r16a	-0.0679750294433432	6.47346494451288	6.10407233438811	0.0134870727459999	0.377305296080036
Stxbp2	Stxbp2	-0.17318350757902	2.76694629533059	6.09581148934066	0.0135502683066063	0.378225274410575
Rrn3	Rrn3	0.0549057181550352	6.24051417684872	6.08799707706441	0.0136103288573894	0.379053737503008
Edar	Edar	-0.413279270257112	-0.136591860328224	6.07375791110789	0.0137204745218235	0.381270290888178
Setd4	Setd4	-0.0895065894696287	4.81029085001878	6.06680536191951	0.0137745879583098	0.381585164094908
Ttc5	Ttc5	0.0542242258175949	6.36335875953194	6.06444978084939	0.0137929717882739	0.381585164094908
Calca	Calca	0.537764420067031	-0.448284757659944	6.05439545391977	0.0138717237152443	0.382914815918368
Dchs1	Dchs1	0.0565194244701606	7.98510406811584	6.04281623280237	0.0139629929810921	0.384583362969285
Zfand3	Zfand3	0.0493867462965022	6.72846870449172	6.03865549477797	0.0140120425937003	0.385084263087222
RGD1305	RGD130511	-0.0646529756940908	6.32064675990383	6.03053850467537	0.0140604430665374	0.385419572144935
C2cd3	C2cd3	0.0904931601078236	5.57042234249172	6.0238086085349	0.0141141558625795	0.385419572144935
Atp1a3	Atp1a3	-0.0790676241867295	10.5914042594041	6.02161320775227	0.0141317234792271	0.385419572144935
Vamp7	Vamp7	0.0930169845970738	4.11698986943058	6.01263205469257	0.0142038253728674	0.385419572144935
Whamm	Whamm	-0.139859776812161	2.79436129352203	6.01196116090416	0.0142092265699024	0.385419572144935
Abhd17b	Abhd17b	-0.06760338069034	5.16904995029314	6.01191653795681	0.0142095858929767	0.385419572144935
Ttyh1	Ttyh1	-0.0709861736828957	8.28215831948038	6.00570783152129	0.0142596715215657	0.385939092352656
Psca	Psca	0.586823002783495	0.0732899466464954	5.99450489822124	0.0143505080372607	0.386257961069665
Ska1	Ska1	-0.264195435891814	1.27336571139899	5.99349932982919	0.0143586903842808	0.386257961069665
Vps26c	Vps26c	0.0782342982077603	5.29511607087958	5.99280711686093	0.0143643258745151	0.386257961069665
Wdr93	Wdr93	-0.40762297201499	-0.096825891173082	5.98816153535089	0.0144022051964244	0.386443686528575
Col15a1	Col15a1	-0.16019361884066	3.85889159868829	5.97944199882152	0.0144735809645633	0.387187201198051
Pask	Pask	-0.128882369120808	3.87529171485016	5.97560521689355	0.0145051029338758	0.387187201198051
Eif4b	Eif4b	-0.0431898790319079	8.24011943807047	5.96863321727124	0.0145625638732097	0.387187201198051
Apmap	Apmap	-0.0804877888876322	5.12941056861029	5.95807553276999	0.0146498524215663	0.387187201198051
N4bp1	N4bp1	0.070840184069469	5.95579409162722	5.95317829328804	0.0146907757056861	0.387187201198051
Rfc4	Rfc4	-0.0932851620472968	4.50607196814835	5.95228316562014	0.0146982370636869	0.387187201198051
Slc9b2	Slc9b2	-0.148288168147555	3.14445494706688	5.95111281705892	0.0147079884176118	0.387187201198051
Trmt6	Trmt6	-0.097524698350782	4.2898228530653	5.9426325669689	0.0147789280763542	0.387187201198051
Xpo4	Xpo4	0.0797559178269158	4.91710255337297	5.93826284829689	0.014815614225653	0.387187201198051
Itsn1	Itsn1	0.0690816840057082	7.45539021502177	5.93759244879164	0.0148212508938095	0.387187201198051
Epha3	Epha3	0.105294272236969	5.48877816162874	5.93744117932096	0.0148225230590722	0.387187201198051
Hoxa3	Hoxa3	1.95507746523434	-0.513797005296934	5.93545244884971	0.0148392585972147	0.387187201198051
Pttg1ip	Pttg1ip	-0.0613189214082109	6.08295641146153	5.93493622260485	0.0148436058171375	0.387187201198051
Prkcsh	Prkcsh	-0.0540748887360726	7.11343205454765	5.93247343300175	0.0148643626796318	0.387187201198051
Sipa1l3	Sipa1l3	0.0763647963674174	6.47803620158563	5.92525263712035	0.0149245511992762	0.387666600481819
Mapkapk3	Mapkapk3	-0.164265542744235	2.69228972173033	5.91984223067226	0.0149712990981461	0.387666600481819
Dennd1c	Dennd1c	-0.15292638220388	2.22498552593768	5.91829145544685	0.0149759799176274	0.387666600481819
Ppfia4	Ppfia4	-0.065030182287891	6.93482996967742	5.9140628947461	0.015020490505989	0.388013788909369
Plaat3	Plaat3	-0.203519326578973	2.64938017264476	5.89035516388679	0.0152240296651092	0.390597880111595
Map4k5	Map4k5	0.0707746810089095	6.00426284734332	5.88902727742157	0.0152355137023894	0.390597880111595
Pou3f3	Pou3f3	0.0893002972242507	8.1828483251328	5.88296795251861	0.0152880302732115	0.390597880111595
Tk2	Tk2	-0.0779536356984902	5.02791455872073	5.88174772874959	0.0152986285695815	0.390597880111595
Shfl	Shfl	-0.139782618258159	4.34588536630234	5.88068237861974	0.0153078878936503	0.390597880111595
Klhl9	Klhl9	0.0657687714065882	7.65546114194776	5.8792914586274	0.0153199855376223	0.390597880111595
Pygo1	Pygo1	0.12343699870221	3.36080685712794	5.8770315995545	0.0153396618782305	0.390597880111595
Adh5	Adh5	0.0524952723660126	6.498685151704	5.87163043834571	0.0153867946969728	0.39100007624059
Ddah2	Ddah2	0.117481453616172	7.28049945041706	5.86497680606703	0.0154450640234501	0.391683056545908
Pomc	Pomc	0.593449030838201	1.59075190431941	5.8447125632073	0.015623925899859	0.394819645760317
Atl3	Atl3	-0.129842040800902	3.19289405201911	5.84380393797228	0.0156319957526326	0.394819645760317
Tf	Tf	0.315580695954871	3.57618538954693	5.82920970291437	0.0157622029315861	0.39608323494625
Slain2	Slain2	0.0561341792521437	7.06392460890566	5.82660303319047	0.0157855137394172	0.39608323494625
Clcnka	Clcnka	-0.481189431480814	0.489634537845804	5.82504160082552	0.0157995947921437	0.39608323494625
Ppp2r3a	Ppp2r3a	0.0836161357359411	6.69000990311289	5.82399981827927	0.0158090447225481	0.39608323494625
Mib2	Mib2	-0.0583341364343566	7.25528897644833	5.81590497267417	0.0158818792272085	0.397110635506774
Mettl18	Mettl18	0.172574571720069	2.56583264571107	5.80453155697049	0.0159849256045233	0.397287477946222
Trip11	Trip11	0.0834690303510705	5.03029903099048	5.80394101829003	0.0159902948241718	0.397287477946222
Bri3bp	Bri3bp	0.117194654457361	3.83353681592987	5.80191555149204	0.0160087246821437	0.397287477946222
Stk32b	Stk32b	0.0842570333577257	5.64235635900692	5.80108171880302	0.0160163181379298	0.397287477946222
Enkur	Enkur	-0.157482262055757	3.41934851958279	5.79668811708691	0.0160563906686592	0.397491242803295
Abhd10	Abhd10	0.171442396259946	3.69687439754495	5.77693075309616	0.016238796056822	0.401210808714789
Lpgat1	Lpgat1	0.0557970151455684	6.58174396303909	5.76387946492296	0.016358913522683	0.403379770795732
Ackr3	Ackr3	0.138055167432181	3.63489905377471	5.75721685651284	0.0164210627305817	0.403802391780309
Gipc2	Gipc2	-0.338607632588521	0.472244969068119	5.75510850372144	0.0164407802375899	0.403802391780309
Cenpl	Cenpl	0.118518692536978	3.2541245765206	5.72915545794685	0.0166855050939188	0.407901561869029
Neurod2	Neurod2	0.0867810638079183	7.38976711648515	5.72858468812629	0.0166909290009809	0.407901561869029
Ebf4	Ebf4	-0.140049755454734	4.84605288949915	5.72702562840981	0.0167057544373707	0.407901561869029
Spast	Spast	0.0503009895554888	7.71699043008629	5.71528491252314	0.0168167813887223	0.409113930637001
Uck2	Uck2	0.0658947887805449	5.77512126774952	5.71495597870393	0.0168209863281864	0.409113930637001
Pbxip1	Pbxip1	-0.0870551020814148	5.15229761022847	5.70325987898194	0.0169324684448645	0.411024141608122
Rai2	Rai2	0.12124195628299	3.75266627976823	5.69896738012493	0.0169748896978058	0.411033651413931
Myl4	Myl4	0.446199448995789	-0.147711235414005	5.69603410447039	0.01700329082507	0.411033651413931
Slc9a1	Slc9a1	-0.0659818372111676	6.04861892638429	5.69310531481275	0.0170316901323237	0.411033651413931
Bin3	Bin3	-0.0958627472988117	4.0773117128832	5.68145864262559	0.0171451083538791	0.412456103753056
Eif5	Eif5	-0.0481324501885933	8.17394456868181	5.68026811332472	0.0171567458401728	0.412456103753056
Tspyl4	Tspyl4	0.0546040484236294	8.24303687004243	5.67568464120315	0.0172016258597307	0.4127397756855
Set	Set	0.135869271158928	6.87477543333328	5.67075229730006	0.0172500568433228	0.413107407358184
Slc6a20	Slc6a20	-0.232722522577232	3.16645239026595	5.661557818766	0.0173407143054043	0.413957813204323
Mrpl42	Mrpl42	0.107587325940708	4.26073790407159	5.660424536257	0.0173519224417617	0.413957813204323
Zfp346	Zfp346	0.057220727275547	5.86825299674588	5.65185615157917	0.0174369058499037	0.415191363147421
Zfp9	Zfp9	0.135542283260838	5.16285929742276	5.6446022985915	0.0175091871126137	0.416118338293487
Tp53i11	Tp53i11	0.0600162693946337	8.09966613273872	5.62961424502848	0.0176595172284482	0.416907256216719
Wdr45	Wdr45	-0.107548254847916	3.99730001397106	5.6250104682061	0.0177059600607459	0.416907256216719
Chchd3	Chchd3	0.0671836528798438	5.69356505620547	5.62362981911055	0.0177199125845772	0.416907256216719
Atic	Atic	0.0741429248513411	6.6945163495854	5.62258535433085	0.0177306775696704	0.416907256216719
Fam222a	Fam222a	0.12840305252726	3.78393589033982	5.61789148681656	0.0177780245764308	0.416907256216719
Cd38	Cd38	-0.201660973274228	1.6135559472614	5.61020082219753	0.017856216465305	0.416907256216719
Fam43a	Fam43a	-0.119450021712091	3.94021089155476	5.61010347575124	0.0178572084701368	0.416907256216719
Lix1l	Lix1l	0.069755644928755	6.70250799236994	5.60623010282576	0.0178967261165656	0.416907256216719
Pcdhga2	Pcdhga2	0.215287420652179	4.33845411662447	5.60620210730846	0.017897012066029	0.416907256216719
Pak2	Pak2	0.0864719639227512	5.98496282799538	5.605084594825	0.0179084303210428	0.416907256216719
Kcnq1	Kcnq1	0.302149667002216	-0.011221914851083	5.60493714784637	0.0179099374214468	0.416907256216719
Neu3	Neu3	-0.275294256854333	1.17791179112845	5.60151473923316	0.0179449559590203	0.416944525383046
Tigd3	Tigd3	0.127308922060291	3.2717866800068	5.59464560137717	0.0180154553536519	0.417235182107068
Dab2ip	Dab2ip	0.0481027383322794	7.97495425696208	5.59137712335378	0.0180491007670272	0.417235182107068
Cacfd1	Cacfd1	0.0801100295079681	4.84238383195794	5.59053440058162	0.0180577861936216	0.417235182107068
Asf1a	Asf1a	0.0919290128482648	4.81725828518394	5.58530905788913	0.0181117370173687	0.417708212136247
Auh	Auh	-0.0811016455151369	5.27114017092249	5.57935530104645	0.0181734115012715	0.418357297603984
Derl3	Derl3	0.167622264025764	2.25031268847641	5.57185817161633	0.0182513822595644	0.419378446505682
Stam2	Stam2	0.0816065218286641	4.77535642995702	5.56574065340257	0.0183152608564359	0.420072628135572
Rgs14	Rgs14	-0.217382491455599	3.11980960051333	5.55260459342033	0.0184532078527346	0.422459952988202
Mrpl2	Mrpl2	-0.0946630855411208	4.04014537601858	5.54433793575563	0.018540569049151	0.423682564150653
Qser1	Qser1	0.0882926477180708	5.58259986002334	5.53763853350731	0.0186116809840086	0.423944641634041
Akr1b1	Akr1b1	-0.0772012250793233	6.28802279623057	5.53685710862102	0.0186199938779718	0.423944641634041
Aqp4	Aqp4	-0.137032122586203	7.05632868091769	5.52818774568637	0.0187124773604097	0.425274280557071
Fndc8	Fndc8	-0.362454152772203	0.249722354748487	5.52073945546536	0.0187923243171687	0.425959000436277
Cav2	Cav2	-0.152149958028491	3.41809002256768	5.51494566074184	0.0188546482009015	0.425959000436277
Timm17b	Timm17b	0.0948881929036488	4.20995451237456	5.51230626848989	0.0188831315542161	0.425959000436277
Jmjd7	Jmjd7	0.245694043142226	3.11618031405861	5.50963191880696	0.0189120265485966	0.425959000436277
Hapln1	Hapln1	-0.25322522987737	2.2385536728234	5.5070556432683	0.0189399050996899	0.425959000436277
P2rx6	P2rx6	-0.158465310104976	2.67724080330093	5.50204812681865	0.0189942142972092	0.425959000436277
Tob1	Tob1	0.10856996280985	4.55554267756887	5.50069026752121	0.0190089687229178	0.425959000436277
Rapgef3	Rapgef3	-0.0898432632883982	5.34426924496169	5.50006912346692	0.0190157219878982	0.425959000436277
Klf9	Klf9	-0.18803485594015	1.75193933253565	5.48095971086674	0.0192247012764477	0.429888454885732
Rfng	Rfng	-0.0647832878457903	6.07195001081338	5.47315549713076	0.0193107288192147	0.430033652321836
Mroh7	Mroh7	-0.298633557205101	0.562363638057581	5.47158282064096	0.0193281130075075	0.430033652321836
Sfmbt1	Sfmbt1	-0.0663650567911579	5.3853904425807	5.46922657023865	0.0193541888608461	0.430033652321836
Polr3g	Polr3g	-0.126772015661971	3.27804514403399	5.4678036262971	0.0193699537236909	0.430033652321836
Lcn2	Lcn2	0.440623762962218	0.187580067080226	5.45979248502512	0.0194589575934238	0.431242298211631
Lin7b	Lin7b	-0.132331439668831	4.67945919894221	5.45259455538738	0.0195392872212693	0.431491526572282
LOC67989	LOC679894	-0.0801228097148257	5.25854454624927	5.44854705510092	0.0195846082394302	0.431491526572282
Tmem98	Tmem98	0.172234752457259	2.49792679492129	5.44505453506244	0.0196238023255603	0.431491526572282
Zcchc4	Zcchc4	0.152545745169275	2.78116998852609	5.44452517722667	0.019629750007796	0.431491526572282
Drg1	Drg1	-0.0805527916143725	5.66588076812704	5.4398923048181	0.0196818829622283	0.431491526572282
Zfp687	Zfp687	0.110201396787793	4.55485931899006	5.43772215455579	0.0197063525180339	0.431491526572282
Nt5m	Nt5m	-0.133108433810387	2.88272482638423	5.43187347982429	0.0197724560576549	0.431491526572282
Spata6	Spata6	-0.100304481118412	3.51386873300611	5.43159081477421	0.0197756566278599	0.431491526572282
Slc7a6	Slc7a6	-0.0661354755122501	5.71237291372805	5.42945214086627	0.0197998898688844	0.431491526572282
Top1mt	Top1mt	0.0841645826075104	4.55522186585615	5.42802890041446	0.0198160325281836	0.431491526572282
Zmym6	Zmym6	-0.0825146323908585	4.77508224322157	5.42289003819473	0.019874436570938	0.432009312013229
Tgfbi	Tgfbi	-0.191320720758107	2.55480785251697	5.4175781514899	0.0199349921252202	0.432370628406528
Cercam	Cercam	-0.170857158550497	2.40244380029982	5.41023760404017	0.0200189909875835	0.432370628406528
Parl	Parl	-0.0753609866900539	4.83808788028676	5.41022986443687	0.0200190797547491	0.432370628406528
Tnip1	Tnip1	-0.0763591411078715	4.6304166085726	5.40692481951523	0.0200570189693239	0.432370628406528
Ppp1r18	Ppp1r18	0.0796532438042957	5.65609925077063	5.40628902737537	0.0200643258673864	0.432370628406528
Rbpjl	Rbpjl	-0.249795706112273	2.09134699790587	5.39739164863457	0.0201668891164738	0.432764060980949
Rprd1a	Rprd1a	0.0557825096093274	6.5966282695004	5.39491565075706	0.0201955015554879	0.432764060980949
Akap8	Akap8	-0.0632827527466318	7.06441289544863	5.39371356079356	0.0202094176822892	0.432764060980949
Auts2l1	Auts2l1	0.0791452568983687	7.02369681563958	5.3926858426852	0.0202213230385424	0.432764060980949
Htr1b	Htr1b	0.142642745606003	3.89799684039537	5.38892453460439	0.0202649569127357	0.432955252397808
Srf	Srf	0.060208943307726	6.19085273484176	5.37458274439336	0.0204322276281836	0.43423884191103
Itm2b	Itm2b	-0.0446078754492815	8.19823551204977	5.37405091694008	0.0204384578268663	0.43423884191103
Pde7a	Pde7a	0.0710628825516857	5.43477891519294	5.37320167397575	0.0204484105097192	0.43423884191103
Ccp110	Ccp110	0.0796434163033218	5.85878515483725	5.37185085703868	0.020464254054956	0.43423884191103
Cpeb1	Cpeb1	-0.227486012932397	1.69576130680497	5.36789942782596	0.0205106635405253	0.434484803047978
Tgfb3	Tgfb3	-0.147087681360656	2.74780047887935	5.36196249938056	0.0205806010383718	0.434966350093602
Mybph	Mybph	-0.0914734935866207	4.9698643371273	5.36005543155311	0.0206031187709641	0.434966350093602
Kcnip4	Kcnip4	-0.175875356202801	3.14222989827264	5.35303092674513	0.020686280829761	0.435821503734619
Man2c1	Man2c1	-0.077956144227876	6.60514265243804	5.34962676308807	0.0207267070800288	0.435821503734619
H1f0	H1f0	-0.0822570808937701	6.90924107862742	5.34732930152186	0.020754039232228	0.435821503734619
Rcl1	Rcl1	0.103594471337175	3.93911270314154	5.34086416385676	0.0208311440822718	0.435821503734619
Dusp22	Dusp22	-0.105116014495979	3.04764885032072	5.3378681189309	0.0208669772307939	0.435821503734619
Rec114	Rec114	-0.288396234062941	0.728197857944243	5.33368719731449	0.0209170883223319	0.435821503734619
Sec22a	Sec22a	0.0880110029544602	3.8408454131315	5.33342342832762	0.0209202528508309	0.435821503734619
Ccnjl	Ccnjl	0.116825666366695	3.18011600558802	5.32632629929436	0.0210056164215702	0.435821503734619
Hrh3	Hrh3	-0.13472901219905	4.08009124070565	5.32258652435962	0.0210507424350316	0.435821503734619
Abhd14b	Abhd14b	0.179542597025292	2.91397999961282	5.31900244059645	0.0210940828631172	0.435821503734619
Spcs3	Spcs3	0.0973809798691588	4.39686233832079	5.31626449914619	0.0211272555033087	0.435821503734619
Lgr4	Lgr4	-0.0714578510152249	5.38021218548576	5.31570803181759	0.0211340038848349	0.435821503734619
Coq6	Coq6	-0.0757861458638173	5.38572267470293	5.31528000786991	0.0211391963051689	0.435821503734619
Lats1	Lats1	0.0729142638903521	6.81231922020821	5.31417989384601	0.0211525622317085	0.435821503734619
Gspt1	Gspt1	0.0443686663849235	7.29876929816572	5.31256223824022	0.021172196149487	0.435821503734619
Ldlr	Ldlr	-0.0912236865709652	4.94947508078153	5.31006986509663	0.0212025048302407	0.435821503734619
Spire1	Spire1	-0.0472171896846523	7.50612756193141	5.30315278766843	0.0212868605706245	0.436835788387224
En2	En2	-1.26025080315318	-0.572737069690259	5.2877280349405	0.0214762083738785	0.439967786328708
Plcg1	Plcg1	-0.051563506568321	8.38780584132324	5.28197748118127	0.0215473622380317	0.440296388502107
Zbtb45	Zbtb45	0.0693028626865481	5.62054321344885	5.28083862301566	0.0215613603730694	0.440296388502107
Jrkl	Jrkl	0.114707387851191	3.59661505532779	5.27396763005648	0.0216466205488168	0.4403228982574
Washc2c	Washc2c	-0.045944139309363	7.29479961429826	5.2718988687719	0.0216723595346641	0.4403228982574
Ttll9	Ttll9	-0.250315432510901	1.66842237438254	5.26896443826937	0.0217238804563699	0.4403228982574
Serpina11	Serpina11	0.431123614598261	-0.242763747510654	5.26616072777558	0.0217439179792832	0.4403228982574
Sh2d3c	Sh2d3c	-0.0688243691436067	5.59845267545215	5.26237557130455	0.0217912553022131	0.4403228982574
Zfp709	Zfp709	0.0880362376350069	4.12413261379818	5.25896523180746	0.0218591026069533	0.4403228982574
Cwf19l1	Cwf19l1	-0.0679078305360033	5.56005941785698	5.25613832078861	0.0218694915809846	0.4403228982574
Cdc42ep4	Cdc42ep4	0.0502685117512982	6.89444816906652	5.25611688870401	0.0218697508129742	0.4403228982574
Dnaja3	Dnaja3	0.0556742321326491	5.69445267570766	5.25528040707372	0.0218802754653833	0.4403228982574
Nmi	Nmi	-0.228853928256431	1.28526797264022	5.2348361148372	0.0221490782151705	0.444292543928848
Pnma2	Pnma2	0.0613858832278862	6.25360723200723	5.2281913480243	0.0222236300917245	0.444292543928848
Lrrc4	Lrrc4	0.0614724585451482	6.17673202655828	5.22787323151353	0.0222276952056451	0.444292543928848
Eya3	Eya3	0.0712175112943621	5.06170499884704	5.22681238851759	0.0222412574147508	0.444292543928848
Aoc3	Aoc3	-0.436234968471236	-0.053621726916614	5.22569283011146	0.0222555774589669	0.444292543928848
Spats1	Spats1	0.170039851042503	1.84228661120431	5.22140335351018	0.0223105394870833	0.444578276645907
Nudt14	Nudt14	0.116768120816089	3.72355810317834	5.21099640017353	0.0224444852573299	0.446429496391229
Dctn6	Dctn6	-0.0483836155255385	6.37828524816254	5.20568834567428	0.0225130868867828	0.446429496391229
Phf20l1	Phf20l1	-0.0614627612243982	6.38068173898919	5.20389382069518	0.0225363505494301	0.446429496391229
Dnajb4	Dnajb4	-0.100528986509052	4.93586705663313	5.20320473049642	0.0225453988791385	0.446429496391229
Rabep2	Rabep2	0.0957835686330375	4.31531765690376	5.20073762342102	0.0225773016985546	0.446429496391229
Ptger3	Ptger3	-0.188836117470667	3.1502847178209	5.17593083481009	0.0229018977992404	0.450654405017697
Trappc2	Trappc2	-0.0751180897442653	5.77463322248948	5.17384835827476	0.0229293658690404	0.450654405017697
Col18a1	Col18a1	-0.118955954388492	5.9235553864639	5.16767140820957	0.0230110413158816	0.450654405017697
Cacna1i	Cacna1i	-0.0962911903721146	6.32772855888138	5.16537020081603	0.0230415463161502	0.450654405017697
Kat14	Kat14	0.0838513745514509	4.31858701891112	5.16204969359753	0.0230856377147008	0.450654405017697
Slc22a15	Slc22a15	-0.0789342246825357	4.70137701187813	5.16106544204559	0.0230987221591257	0.450654405017697
Ehd2	Ehd2	0.175185647825195	2.55113258822359	5.16071415329844	0.0231033955631734	0.450654405017697
Zfp319	Zfp319	0.0798280567743342	5.3685531863604	5.15978049036533	0.0231158187052733	0.450654405017697
Chchd5	Chchd5	0.152705803193932	3.19870225484434	5.15976393513645	0.0231160390497558	0.450654405017697
Ptpn12	Ptpn12	0.0619142461908927	5.9430549621614	5.15897713021172	0.0231530279981758	0.450571362173541
Pxdc1	Pxdc1	0.0638512284628304	5.77748515907535	5.14392327411355	0.0233278727774088	0.453367396641342
Nectin4	Nectin4	-0.155547381654136	-0.020704279860901	5.13085142136119	0.0235041952758631	0.455414279173507
N4bp2l2	N4bp2l2	0.084588778892885	4.76719647910631	5.13070374897	0.023506195061933	0.455414279173507
Drc1	Drc1	-0.141326710205653	3.37319862049563	5.12783684438144	0.0235450537984618	0.455459901152571
Hcn4	Hcn4	0.0682656773835848	6.00006304552365	5.12260615888016	0.0236161235551065	0.456127513308149
Sgsm1	Sgsm1	-0.08425989822611	6.54466581958	5.11665946634153	0.0236971920771481	0.456982729321488
Pdzrn3	Pdzrn3	-0.0889017786171825	6.07108941552882	5.11363397438554	0.0237412844022892	0.456982729321488
Ephx2	Ephx2	0.63480041060283	2.40200032538714	5.10989885583691	0.0237896923479295	0.456982729321488
Rnf10	Rnf10	0.0367334473516687	8.64328305310439	5.10372155040582	0.0238745674057194	0.456982729321488
Dlg5	Dlg5	0.0479173646732207	7.0954456708041	5.10330336597437	0.0238803235237198	0.456982729321488
Srfbp1	Srfbp1	0.0893624108937242	4.48765012820471	5.10088557871355	0.023913631496798	0.456982729321488
Lpl	Lpl	-0.175812458710061	5.66358825561878	5.09853174204145	0.0239323032946331	0.456982729321488
Rilpl1	Rilpl1	0.0851105740566159	4.3552390518226	5.09800262741642	0.0239534106737299	0.456982729321488
Ipmk	Ipmk	0.0727306217096387	5.68582199480469	5.09279448887307	0.0240254470671186	0.457657256574715
Mycl	Mycl	0.0906030219236844	5.6963522865174	5.0882454778659	0.0240885774217005	0.458160336113655
Afap1	Afap1	0.054296623030235	7.38186582581083	5.08442307005813	0.0241417077087373	0.458269472567513
Fzd1	Fzd1	0.0849763329008725	5.57932970346154	5.07896554960845	0.0242178148641412	0.458269472567513
Map4k2	Map4k2	-0.100708841213546	5.18006158626838	5.0769975789824	0.0242453196785481	0.458269472567513
Rorb	Rorb	0.151793848583291	4.36865903056894	5.07575036847273	0.0242627582503328	0.458269472567513
Pmepa1	Pmepa1	0.0950451536180335	4.58688404518735	5.07008402810307	0.0243422036504695	0.458269472567513
Ccdc62	Ccdc62	-0.136954856160323	3.13984016598041	5.06648809992546	0.0243930357167915	0.458269472567513
Usb1	Usb1	0.0979460237040007	3.37298442550308	5.06566946239131	0.0244042776473638	0.458269472567513
Bcl2l11	Bcl2l11	-0.16188889474626	3.61530480513763	5.06551549815066	0.0244064455280451	0.458269472567513
Loxl2	Loxl2	-0.163010671254009	2.48078003837048	5.06420700598567	0.0244248777155884	0.458269472567513
Zfp52	Zfp52	-0.144420603275419	2.20496275046685	5.05859758468447	0.02453245181397	0.459576654511278
Prpf39	Prpf39	-0.092962284238236	5.84855095329061	5.05406629898645	0.0245682163263808	0.459576654511278
Nme6	Nme6	0.238836674590031	2.28528651964947	5.05107821570267	0.0246106191260803	0.459980808901997
Adamts1	Adamts1	-0.196344341998148	2.58646361679902	5.04767508169243	0.0246590024741191	0.459889056837047
Scgb1c1	Scgb1c1	-0.324983885981156	-0.303890124943255	5.03842506820672	0.0247910168788925	0.461687972654398
Smurf2	Smurf2	0.0522553252381946	6.29534782677954	5.03362924741315	0.0248741094881434	0.462108878824826

**Six5**	Six5	0.137499477800521	2.74465812396658	5.03160881261745	0.0248887586491851	0.462108878624826
**Ccn1**	Ccn1	-0.293920504633741	1.24172581018462	5.0271576557375	0.024952817161265	0.462583937260941
**Nnat**	Nnat	0.0596091853397341	11.0546789541	5.024645195449	0.0249890446287991	0.462583937260941
**Aff4**	Aff4	0.0593034882246577	8.55022216404048	5.01827060039734	0.0250812070277166	0.463612177903437
**Myh10**	Myh10	0.0459449503305083	9.15400689181213	5.00841764438267	0.0252243521785565	0.465568423677588
**RGD1307**	RGD130755	0.0613185341755085	5.33012855538766	5.00211780689764	0.0253163221288658	0.4658222735677
**Golm2**	Golm2	0.0708382493690274	7.73966981494306	5.00198883298829	0.0253182096240203	0.4658222735677
**Ppp1r10**	Ppp1r10	-0.0578035469322503	6.61657204178162	4.98871963010535	0.0255131219234994	0.469293591690494
**Zfp707l1**	Zfp707l1	-0.158953220790582	2.65153457263496	4.97878609794554	0.0256599819931168	0.469293591690494
**Shroom4**	Shroom4	0.146598942907466	2.51136400240194	4.97616039084282	0.0256989588974594	0.469293591690494
**Snx18**	Snx18	-0.0656197653453953	5.36288176155564	4.97273912171336	0.0257498376929248	0.469293591690494
**Nav2**	Nav2	0.0789927583533658	7.2261140679298	4.97192841996157	0.0257619092275875	0.469293591690494
**Vezt**	Vezt	0.0603712744742373	6.74669836595316	4.96861807921922	0.0258112620034674	0.469293591690494
**Eloa**	Eloa	0.0555288693932233	5.54874076894824	4.96366307436414	0.0258852181646978	0.469293591690494
**Phyhip**	Phyhip	-0.120179724076745	6.06521706345739	4.96201524584646	0.0259099950205678	0.469293591690494
**Slc38a1**	Slc38a1	0.0533489648491008	6.84447740318642	4.95817025452374	0.0259676703639407	0.469293591690494
**Gpr37l1**	Gpr37l1	-0.149117096711628	4.77528579357951	4.95804621231488	0.0259695322217258	0.469293591690494
**Rit2**	Rit2	-0.0678017853708689	6.39207738806837	4.95684423479737	0.0259875917413787	0.469293591690494
**Kifc2**	Kifc2	-0.101499917108425	7.94918281610412	4.95654015474524	0.025992162307413	0.469293591690494
**Dmbx1**	Dmbx1	-0.990398987641929	0.570272405769745	4.95403914066721	0.0260297862452086	0.469293591690494
**Tmem109**	Tmem109	0.0744632330895279	4.97851980955795	4.95391615535514	0.0260316378204853	0.469293591690494
**Lrrc4b**	Lrrc4b	0.0456858239817601	9.06049269885181	4.95116627337849	0.0260730729868052	0.469293591690494
**Tjp3**	Tjp3	-0.221325869633375	2.29174246727319	4.94208102417763	0.0262104615233664	0.469293591690494
**Mlec**	Mlec	0.078948907322369	5.48245848669401	4.94128383760477	0.0262221035450614	0.469293591690494
**Gfap**	Gfap	-0.188851736177446	5.99310719438115	4.93899057072885	0.02625736679855	0.469293591690494
**Hs6st1**	Hs6st1	0.0580615241230882	7.10666500779669	4.93881932625298	0.0262603729605084	0.469293591690494
**Anapc1**	Anapc1	0.0535456991816645	7.54270177428642	4.93615541460118	0.0263004740154178	0.469293591690494
**Pex1**	Pex1	-0.0649468506533192	5.44453929642928	4.93500257932362	0.0263180223360489	0.469293591690494
**Fam131b**	Fam131b	-0.0602651904313932	7.7726552688226	4.9342893824325	0.0263288862848718	0.469293591690494
**Lratd1**	Lratd1	0.0625067063628194	6.69629037412941	4.92858454006112	0.0264159464059856	0.47017369944006
**Fam91a1**	Fam91a1	-0.0423962747243648	6.58954716641782	4.92572770487303	0.0264590562029592	0.470280812598749
**Dnajc3**	Dnajc3	0.1126293677796	4.30464101163993	4.91291601422024	0.0266556025172739	0.470576783069059
**Bcdin3d**	Bcdin3d	0.132195547954548	3.15764282646268	4.9075934114398	0.0267388711055296	0.470576783069059
**Osbpl11**	Osbpl11	0.0620442978377022	6.10158056749692	4.90185595224342	0.0268278475720187	0.470576783069059
**Rap2a**	Rap2a	0.0981739565831308	4.1238941859963	4.89646825764601	0.0269116801454251	0.470576783069059
**Cflar**	Cflar	-0.0772925227517736	4.55166765734905	4.89480331656668	0.0269376418943335	0.470576783069059
**Por**	Por	0.0457589048365558	6.82180829150976	4.89321769048041	0.0269623906876727	0.470576783069059
**Zfat**	Zfat	0.106171138171835	3.57715828887005	4.89319392676229	0.026962761779659	0.470576783069059
**Pfkp**	Pfkp	-0.119050061478494	7.62939569887346	4.88889983156236	0.0270142534841901	0.470576783069059
**Pkhd1l1**	Pkhd1l1	-0.384576271282047	0.167488273428251	4.88755174330529	0.0270510200752966	0.470576783069059
**Fgd3**	Fgd3	0.0891182147345509	5.39373149225888	4.88693879760343	0.0270606261990798	0.470576783069059
**Gprasp1**	Gprasp1	-0.0407904962031521	9.33862118338716	4.88628749889864	0.0270708372805422	0.470576783069059
**Elavl1**	Elavl1	0.0907287323296059	5.50403121958057	4.88127190039297	0.0271490063445877	0.470576783069059
**Sars2**	Sars2	-0.0921176175675332	3.59932676327924	4.87728220921368	0.0272124339007628	0.470576783069059
**Alkbh4**	Alkbh4	0.106888981911645	3.4148085552092	4.87716291146005	0.027214314865364	0.470576783069059
**S100a13**	S100a13	-0.196530841875208	2.51551855355206	4.87669987490627	0.0272216152544831	0.470576783069059
**Zfhx2**	Zfhx2	0.1137612089312	5.5858037583159	4.87362930025311	0.0272700905808717	0.470576783069059
**Rabggtb**	Rabggtb	-0.0789950198254347	6.05042065527801	4.87079522457342	0.0273149104944253	0.470576783069059
**Tvp23a**	Tvp23a	-0.0589502081657409	5.06651543090959	4.86753438952836	0.027366574256481	0.470576783069059
**Atp6v0a1**	Atp6v0a1	-0.0523495370363744	8.93829680922329	4.86560969061418	0.0273961636925029	0.470576783069059
**Jagn1**	Jagn1	0.0577500086689601	5.02348844263733	4.86315649982084	0.0274360961889018	0.470576783069059
**Eif4ebp2**	Eif4ebp2	0.0734031009645728	5.89366773298531	4.86293520466862	0.0274396184631348	0.470576783069059
**Prkci**	Prkci	0.0468961090095433	6.68760041927147	4.86277285311172	0.0274422992593897	0.470576783069059
**Fancb**	Fancb	-0.186326339118027	1.80652607579673	4.86165901960775	0.02745991867376	0.470576783069059
**Fam219a**	Fam219a	-0.0562941927845294	7.18241403366529	4.86060237973903	0.0274767410483195	0.470576783069059
**Zfp280b**	Zfp280b	0.118604489553652	3.19370978063452	4.85977909338006	0.0274898557170366	0.470576783069059
**Tep1**	Tep1	-0.0940116497744168	4.57327385785441	4.85892948849575	0.0275033964574413	0.470576783069059
**Ttll13**	Ttll13	0.223488665712901	1.48948307452617	4.85526441081632	0.0275618888997357	0.470576783069059
**Actl6a**	Actl6a	0.0910110232212323	4.1273763737512	4.85325411942885	0.0275940268826567	0.470576783069059
**Tnn**	Tnn	-0.24891572428779	1.34771037827488	4.85263112712832	0.0276039944014093	0.470576783069059
**Slc43a3**	Slc43a3	-0.171121874223418	2.34158570349281	4.85012466251121	0.0276441342576119	0.470576783069059
**Atp6v0a2**	Atp6v0a2	0.0768716000712097	4.60343834731438	4.84882642226334	0.0276649488215579	0.470576783069059
**Dio2**	Dio2	-0.149758296901546	4.1768794576261	4.84623346640798	0.0277065702841084	0.470576783069059
**Bcl6**	Bcl6	0.126712835774097	4.48461373783582	4.84381132506337	0.0277616013512717	0.470576783069059
**Apold1**	Apold1	-0.154941925391641	2.87343203713429	4.84262904684477	0.0277645257328547	0.470576783069059
**Tnnc1**	Tnnc1	-0.282681053061544	0.464684480394861	4.82855966850823	0.027813996234007	0.470875482055231
**Csnk2b**	Csnk2b	-0.0468267886664464	6.84412537469876	4.82521146145437	0.0278826038746499	0.471394512506766
**Serpinb6**	Serpinb6b	-0.205379358831546	1.64584168770045	4.829749117558	0.027972700846875	0.471394512506766
**Ca8**	Ca8	-0.156897005381368	3.68756465601157	4.82143954830025	0.0281078511527561	0.471394512506766
**G4**	G4	0.12559682928821	3.46697822808948	4.81454216022584	0.0282205675723247	0.471394512506766
**Fbxo34**	Fbxo34	-0.0713551601836694	5.09842959022929	4.81353185668382	0.0282371158340867	0.471394512506766
**Fahd1**	Fahd1	-0.0868920585799508	4.145726287279	4.812707130074	0.028250631826036	0.471394512506766
**Bhlhe22**	Bhlhe22	0.0748474359620961	8.00350046288148	4.80776165517912	0.0283318225711371	0.471394512506766
**Dusp19**	Dusp19	0.120498186435939	3.56186054335059	4.80389837258258	0.0283954156302743	0.471394512506766
**Ccn2**	Ccn2	-0.130527037581165	3.5754262244816	4.79694902617744	0.0285103182309897	0.471394512506766
**Col5a3**	Col5a3	-0.107199880858783	5.64991341961822	4.79648257175342	0.0285179028567625	0.471394512506766
**Neu1**	Neu1	-0.123417558982734	4.21875161329319	4.79520429373758	0.0285390718781159	0.471394512506766
**Phf21b**	Phf21b	0.124045609805092	3.45293597183238	4.79194134372756	0.0285931821584811	0.471394512506766
**Cdkn1c**	Cdkn1c	-0.188523830544351	5.91882313851218	4.78796144228515	0.0286593264260423	0.471394512506766
**Mertk**	Mertk	0.188195308861448	1.90706546103766	4.7856767536797	0.0286973688261501	0.471394512506766
**Plcd1**	Plcd1	-0.112265647891223	4.27849649011284	4.78532828842545	0.0287031758628411	0.471394512506766
**Phf3**	Phf3	0.0667232022347271	6.7267201775153	4.78396224127637	0.0287259519114468	0.471394512506766
**Ccdc81**	Ccdc81	-0.420100555347043	-0.318299297168289	4.78290337221111	0.0287436193365554	0.471394512506766
**Fnbp4**	Fnbp4	-0.0688381174875448	6.27492275858254	4.78050575012468	0.0287836658240598	0.471394512506766
**C1s**	C1s	-0.194227108149159	1.97517759825565	4.77945031677579	0.0288013128431328	0.471394512506766
**Pcdha9**	Pcdha9	-0.151263261686488	2.84955472877994	4.7775909032606	0.0288324297737836	0.471394512506766
**Manba**	Manba	-0.130504817448834	3.13754840863032	4.776989342314	0.0288473523755098	0.471394512506766
**Aff3**	Aff3	0.0703679033412171	6.7265700611391	4.77546574703281	0.0288680367302947	0.471394512506766
**Tmtc4**	Tmtc4	0.0794775822650617	6.46012114077953	4.77521582292786	0.0288722272120172	0.471394512506766
**Rgl2**	Rgl2	-0.0725622759488896	5.99336031976266	4.76610184499012	0.0290254748278541	0.471394512506766
**Hddc3**	Hddc3	-0.118979293262711	4.01174621185133	4.76230142922174	0.0290896273470688	0.471394512506766
**Zfyve19**	Zfyve19	0.0998506013614917	4.20828342765301	4.75829277982297	0.029157454850167	0.471394512506766
**Mettl25**	Mettl25	-0.115582294543033	2.9878262354255	4.75643743790432	0.0291888035084233	0.471394512506766
**Ccni**	Ccni	-0.0696631065338145	9.10854640427511	4.75587597073918	0.0291984275229286	0.471394512506766
**Klhl29**	Klhl29	0.0911916004924983	6.6303767717117	4.75543652219679	0.0292058840265853	0.471394512506766
**Tipin**	Tipin	-0.113649679930562	4.35571777971603	4.75209477709358	0.0292626512281687	0.471394512506766
**Prag1**	Prag1	0.0918081918091354	5.86758982785888	4.75143160017059	0.0292739304675489	0.471394512506766
**Nhlrc2**	Nhlrc2	-0.110081820877536	4.24226007117331	4.74825589905421	0.029326300874582	0.471394512506766
**Coro6**	Coro6	-0.276297070336184	1.09303490182716	4.74581301798752	0.0293696725740392	0.471394512506766
**Gfral**	Gfral	-0.467222361335252	-0.293075823358821	4.74479647711424	0.0293870295087961	0.471394512506766
**Polb**	Polb	0.0694011084205153	5.49694878336008	4.74441110272816	0.029393612379342	0.471394512506766
**Tsc2**	Tsc2	-0.0457218648107691	7.34019855147131	4.74370806222359	0.0294056255016727	0.471394512506766
**Mycbpap**	Mycbpap	0.233606570392914	1.1809847126666	4.74092128330918	0.0294532945965157	0.471394512506766
**Ift20**	Ift20	0.0754664266807967	5.25050679492026	4.73961171139426	0.029475722201362	0.471394512506766
**Tmem35b**	Tmem35b	0.241646223312431	1.39904415019408	4.73900231467729	0.0294861662022837	0.471394512506766
**Ddx6**	Ddx6	0.0754946236220841	7.23005118836864	4.73870975448536	0.0294901525142523	0.471394512506766
**Exosc9**	Exosc9	-0.0633187354453947	4.93523910306761	4.73668580469418	0.0295258986526752	0.471394512506766
**Fhod3**	Fhod3	-0.0623911759356555	6.85937018479126	4.73304576356109	0.0295884445459747	0.471394512506766
**RGD6210**	RGD621098	-0.0648868247165625	5.16210586752198	4.73261308406935	0.0295958883529092	0.471394512506766
**Zfp710**	Zfp710	0.0874423209406553	4.12618952109282	4.73200310267218	0.0296408318034539	0.471394512506766
**Rassf4**	Rassf4	-0.197161582728455	1.93014937308271	4.7226378734253	0.0297507384150358	0.471394512506766
**Tefm**	Tefm	0.131931199480364	2.40004259179176	4.72223657052841	0.0297749912899062	0.471394512506766
**Eny2**	Eny2	-0.0598497276204275	6.27283108858725	4.72078197472184	0.0298001883154823	0.471394512506766
**Nmnat3**	Nmnat3	-0.235528722331504	0.771939239395256	4.72045387797795	0.0298058747940027	0.471394512506766
**Lrrc1**	Lrrc1	-0.0732827638168174	4.98023607721747	4.71890655564469	0.0298327077829834	0.471394512506766
**Tmem72**	Tmem72	0.405436705317255	1.93572947958887	4.71881998186863	0.0298342098509801	0.471394512506766
**Magi1**	Magi1	-0.0465428130957504	6.46926567260591	4.71709762905268	0.0298641093145297	0.471394512506766
**Matk**	Matk	-0.109513147943611	6.47996253449467	4.71512456905995	0.0298983993438915	0.471394512506766
**Gtf3c2**	Gtf3c2	-0.0423998890809076	6.27282043922737	4.71324490489896	0.0299311043958824	0.471394512506766
**Fam221a**	Fam221a	-0.217840958953834	2.34383641149448	4.71229236317036	0.0299476922923812	0.471394512506766
**Slc22a4**	Slc22a4	-0.235466043892361	0.613392682069731	4.71034095013653	0.0299817048063655	0.471394512506766
**Tfpi**	Tfpi	-0.116885270959436	4.06547756222326	4.70912800000853	0.0300028664288459	0.471394512506766
**Irf2**	Irf2	-0.116785223883681	2.79084102368182	4.70723655428051	0.0300341491955089	0.471394512506766
**Eif2ak2**	Eif2ak2	-0.0853476927163005	4.07580609814835	4.70596209681953	0.0300581734293342	0.471394512506766
**Pgap3**	Pgap3	0.101590811928225	3.59182692408498	4.70497229250594	0.0300754866881437	0.471394512506766
**Tex**	Tex	0.0945176598015325	4.85828217722108	4.70257305523803	0.0301174963208689	0.471394512506766
**LOC1009**	LOC100910	0.334680538255983	0.0382058209487557	4.70075898882942	0.0301493004551093	0.471394512506766
**Aspg**	Aspg	-0.385418373009025	-0.002238044938058	4.69934423917086	0.0303225699911403	0.472540099753299
**Ier5l**	Ier5l	0.0993502560086349	4.7208146362765	4.68891921602864	0.0303577364457274	0.472540099753299
**Ankib1**	Ankib1	0.0498575190573709	6.6739138170489	4.6868738071646	0.0303938971955912	0.472540099753299
**Drgx**	Drgx	0.568296888298508	-0.238764484129161	4.68642646379952	0.0304018117533771	0.472540099753299
**Tspan7**	Tspan7	-0.0543689031359676	8.72153836473243	4.68367424473212	0.0304505522813265	0.472540099753299
**Maff**	Maff	-0.22578531469937	0.850987290761987	4.68317251556845	0.0304594464474611	0.472540099753299
**Bcar3**	Bcar3	-0.110603670924425	3.31367220014834	4.67916465119755	0.0305305912556288	0.472540099753299
**Zeb2os**	Zeb2os	0.131825054120974	3.71852569544164	4.67912558914772	0.0305312855096201	0.472540099753299
**Asphd2**	Asphd2	0.078742787079821	5.26093474300338	4.67436002028312	0.0306161080814563	0.472540099753299
**Psen1**	Psen1	0.0552666087811461	5.66062753828813	4.66797483047802	0.0307301436496621	0.472540099753299
**Zdhhc9**	Zdhhc9	0.0464476297306494	7.01498058251394	4.66761184768159	0.0307366377976093	0.472540099753299
**Enah**	Enah	0.0738614849729821	6.36318828191012	4.66496621846263	0.0307840326029567	0.472540099753299
**Sec14l2**	Sec14l2	-0.100323555692309	3.91396807613726	4.66410148205319	0.0307995346990532	0.472540099753299
**Ly86**	Ly86	-0.340651254360007	-0.272202835552435	4.66012632476631	0.0308709201423044	0.472540099753299
**Eml1**	Eml1	0.0708961376156585	6.34745492146431	4.65829423944732	0.0309038844021639	0.472540099753299
**Nt5c**	Nt5c	-0.173616649076672	1.94239901687275	4.65829032052145	0.0309861347848843	0.472540099753299
**Anxa11**	Anxa11	-0.110762365704918	3.58560797385153	4.65708470462882	0.0309256574966318	0.472540099753299
**Acp4**	Acp4	-0.252451211406427	0.992160414832203	4.65579359230437	0.0309489230950772	0.472540099753299
**Tti1**	Tti1	0.080104717293171	4.90501553548567	4.64972777530691	0.0310584725264895	0.472540099753299
**Pias1**	Pias1	0.0816742503827569	4.75864477118303	4.64774882425238	0.0310943000592406	0.472540099753299
**Mindy3**	Mindy3	-0.0531315294200405	5.92512352077437	4.64726582967726	0.0311030506765295	0.472540099753299
**Ca3**	Ca3	0.545700743724228	-0.435062741137938	4.64602777816258	0.0311254934436884	0.472540099753299
**Col9a2**	Col9a2	-0.137529549770326	4.22457188839718	4.64394050451025	0.0311633683772088	0.472540099753299
**Zcchc8**	Zcchc8	-0.0684092325807813	5.61203919121925	4.64248389117111	0.0311898289646553	0.472540099753299
**Slc16a1**	Slc16a1	-0.0747681065350942	5.89239787961837	4.63877493652089	0.031257307779655	0.472540099753299
**Hs6st2**	Hs6st2	0.0790331109568868	6.08096482911995	4.63764229272964	0.0312779451368417	0.472540099753299
**Snap23**	Snap23	-0.105348896348211	3.50317691849844	4.63730629230925	0.0312840896716822	0.472540099753299
**Arr3**	Arr3	-0.387503487900506	0.343089032621912	4.63557432023393	0.0313156613284818	0.472540099753299
**Unc13d**	Unc13d	0.21608587958805	1.0841381574829	4.63528819883192	0.0313208834251806	0.472540099753299
**Cntn3**	Cntn3	-0.101515107034178	4.80497160071262	4.63146315183006	0.0313907828264722	0.473022702084413
**Fchsd1**	Fchsd1	-0.0925184778521758	5.27276705244304	4.62873677182794	0.0314407043550251	0.473203459676536
**Tmem67**	Tmem67	-0.0693449605925491	5.15228661870345	4.61508925874848	0.0316892215363043	0.476161269786035
**Ywhah**	Ywhah	-0.0414280646209216	9.67540627760473	4.61262765422654	0.0317373665619544	0.476161269786035
**Orai1**	Orai1	0.126209080127803	3.54197049556197	4.60959770056593	0.0317934911097407	0.476161269786035
**Gpr12**	Gpr12	0.0707712489537963	4.98332860384052	4.60879389645285	0.0318083975270037	0.476161269786035
**Vhl**	Vhl	0.0564974360526743	6.31491416721168	4.60718382311293	0.0318382780642497	0.476161269786035
**Mccc2**	Mccc2	-0.0667053224086428	4.91141945060132	4.60251685229212	0.0319250555176154	0.476161269786035
**Akr1c19**	Akr1c19	-0.482682581003592	0.378227380795185	4.6020409285481	0.0319339184772432	0.476161269786035
**Ehmt1**	Ehmt1	0.0454134340854198	7.35261170480014	4.59677532655783	0.0320321517500008	0.476161269786035
**Adam23**	Adam23	0.0693881349848623	6.50361529429993	4.59153153620201	0.0321302917197211	0.476161269786035
**Ctns**	Ctns	0.0986252728642262	3.29004167851279	4.59144873052754	0.0321318438777784	0.476161269786035
**Pcdhb21**	Pcdhb21	-0.176407990484861	2.02590464097377	4.5894414716682	0.0321694955911648	0.476161269786035
**Ndrg4**	Ndrg4	-0.0691579007879863	9.65871603254932	4.58812349957468	0.0321754642485173	0.476161269786035
**Rgs20**	Rgs20	-0.0840739951966143	4.21904543579896	4.58811724602961	0.0321755816454629	0.476161269786035
**Mrfap1**	Mrfap1	-0.0422551565044764	8.51277009215347	4.5873256378033	0.0322092236798813	0.476161269786035
**Fign**	Fign	0.181440642161607	3.2030392735396	4.58724213946886	0.0322193028267293	0.476161269786035
**Kcnk10**	Kcnk10	0.0805634972175436	5.29894216095034	4.58527278580074	0.0322478378581969	0.476161269786035
**Zmynd8**	Zmynd8	0.044824129727396	6.9476163491309	4.5816213467918	0.03221682675058	0.47623833282619
**Cpxm1**	Cpxm1	-0.0693914974627345	5.36507468037982	4.58014698500831	0.0323444402750468	0.47623833282619
**Apoc4**	Apoc4	-0.458413743601681	-0.637059126505783	4.5785625911839	0.0323743613244441	0.47623833282619
**Zc3h10**	Zc3h10	0.0967206028819394	4.06577827498389	4.57326286851719	0.0324746559634614	0.477209558254097
**Kif26b**	Kif26b	0.104788720979462	6.18557843633727	4.5713956969427	0.0325100684872952	0.477209558254097
**Tigd5**	Tigd5	0.0813057694521716	3.78690860938542	4.56428745453466	0.0326452517566262	0.478630794556317
**Hsp90ab1**	Hsp90ab1	-0.034358746322994	11.1913477408875	4.56050046056474	0.0327175216190483	0.478739660870924
**Zfp951**	Zfp951	0.277521854524697	0.508502752977988	4.55987738327024	0.0327294165943471	0.478739660870924
**Nhs**	Nhs	0.121825791703917	3.64949072615043	4.55789028156017	0.0327679878202929	0.478742604255028
**Nit1**	Nit1	-0.0645248376542472	5.55797737965406	4.55426054034866	0.0328350229048047	0.479160913193862
**Cnot6**	Cnot6	0.0711971307476327	6.36609259140797	4.5492445319198	0.0329332742341318	0.480033250723438
**Rwdd2a**	Rwdd2a	-0.152215756092062	2.44681056450913	4.54403595800365	0.0330336198967214	0.48045841019679
**Hnrnpdl**	Hnrnpdl	-0.0719845293813989	7.58033799003523	4.54283870340909	0.0330567292017775	0.48045841019679
**Cd1d1**	Cd1d1	-0.316680770504947	-0.019407341508023	4.53742583242807	0.0331614243272926	0.48045841019679
**Camkv**	Camkv	-0.06026882501038	8.94910803572951	4.53311269921642	0.0332450963709087	0.48045841019679
**Dmtf1**	Dmtf1	0.0821467646317631	6.54661578679536	4.53300283603515	0.0332472305242579	0.48045841019679
**Dctn4**	Dctn4	0.0485922868275428	6.34423126066076	4.53128728497166	0.033280574627342	0.48045841019679
**Galnt6**	Galnt6	-0.11081389692295	3.50994300392679	4.53103821385083	0.0332854185684825	0.48045841019679
**Msh4**	Msh4	-0.443390084432444	-0.76510732211021	4.52842862947392	0.0333362141628826	0.48045841019679
**Med26**	Med26	-0.0844361170615294	4.52703668186059	4.5280880120004	0.0333428502578487	0.48045841019679
**Cstf2**	Cstf2	-0.048061021494448	6.93082937534934	4.52807473275361	0.0333821022525673	0.48045841019679
**Lrfn2**	Lrfn2	0.0713932293528604	5.35018815624083	4.52587363483253	0.0333860256183872	0.48045841019679
**Pigv**	Pigv	0.117364497715313	3.25083893631094	4.52260259939178	0.0334499103155756	0.480823192404881
**Zfp455**	Zfp455	0.231358290784247	1.33070897452365	4.51866244570628	0.0335270327725721	0.481169347618375
**Slc2a1**	Slc2a1	-0.0667115059108544	5.7182451222079	4.51743374622341	0.0335511208562464	0.481169347618375
**Jdp2**	Jdp2	-0.122767999152282	3.19345149171134	4.50912157283664	0.033714551242875	0.482958043464237
**Col4a3**	Col4a3	-0.266832625838057	0.756065761456623	4.50485519685611	0.0337987578172543	0.483809061107683
**Egfem1**	Egfem1	-0.158530446521524	3.76837994350218	4.50179597516127	0.0338592737453493	0.483919998305526
**Raph1**	Raph1	0.0803122025389831	6.39396475573974	4.49597031341672	0.033974827258188	0.485015926445096
**Fam214b**	Fam214b	0.0718886301728311	5.67023338054397	4.49127003623593	0.0340683588436399	0.48579532947668
**Lgals8**	Lgals8	-0.0766681789073306	4.89491881710847	4.48282009589451	0.034237182985881	0.487104968056702
**Mdk**	Mdk	0.111843525681298	4.94858924089428	4.48276518016843	0.0342382830206643	0.487104968056702
**Pi4k2a**	Pi4k2a	-0.0675569824759881	5.033354905878	4.48067274406359	0.0342802248226442	0.487146201722246
**Pik3cb**	Pik3cb	0.0730184854548378	5.00833421122659	4.47218732144009	0.0344508626289269	0.489014113236542
**RT1-A1**	RT1-A1	-0.395747606547131	0.934997017761321	4.46547134238075	0.0345865461047753	0.490250462236745
**Srm**	Srm	0.0555169149813023	6.83222955836626	4.46399010014442	0.0346165470249717	0.490250462236745
**Eln**	Eln	-0.117114162783754	6.55142981925244	4.45403284952285	0.0348189276288452	0.492302569051142
**Hexd**	Hexd	-0.069377447918352	5.3762864179772	4.45298192087013	0.0348403597609248	0.492302569051142
**Txnrd3**	Txnrd3	-0.0897732393499154	4.60520957361986	4.45067079584798	0.0348875400682132	0.492411583827259
**Rasd1**	Rasd1	-0.175490476391611	1.97492953565733	4.442558589737	0.0350331932994287	0.493493961783255
**Usp49**	Usp49	0.075678477555337	5.01188882256272	4.44308254057496	0.0350433217419017	0.493493961783255
**Rprml**	Rprml	-0.141394003561869	5.19385967067909	4.43912776671873	0.0351241879471284	0.493770313456656
**Twf2**	Twf2	0.0661073264765265	5.09812719626494	4.43825715860363	0.035142104540315	0.493770313456656
**Wdr24**	Wdr24	-0.0784937824666949	5.28995878889726	4.42853343944577	0.0353428637019181	0.496032520144918
**Ext2**	Ext2	0.058059667067763	5.97943374293135	4.42576052268881	0.0354003238287653	0.496280859894691
**Prdm8**	Prdm8	0.0917849312238066	6.41077069798912	4.42321240358155	0.0354511536301746	0.496425524976081
**Myo9b**	Myo9b	-0.0646108625975687	6.80375446571374	4.4197585463934	0.035525063098339	0.496912794633604
**RGD1563**	RGD156307	-0.0751147125707472	4.9904105515634	4.41251180618937	0.0356762729308735	0.498005419096274
**Pip4p2**	Pip4p2	0.0699052019069169	5.7804123146012	4.40893531075427	0.0357511478666615	0.498005419096274
**Minpp1**	Minpp1	0.0766255806534646	5.46000785404797	4.40890585289784	0.0357517631615639	0.498005419096274
**Cops3**	Cops3	0.047338447139457	6.25909053148821	4.40837790730627	0.0357628320661587	0.498005419096274
**Ankrd13d**	Ankrd13d	-0.0609793629430105	6.46096575123708	4.39934625481833	0.0359527100113314	0.499612504614323
**Ica1**	Ica1	-0.0564328501350178	5.70899489029906	4.39907990425205	0.0359583256506902	0.499612504614323
**Rock1**	Rock1	-0.0779547899213158	6.80496051391108	4.39697962670746	0.0360026393162472	0.499671780588227
**Rtn4r**	Rtn4r	0.0641501659917478	5.96746111620114	4.39336797494515	0.0360789750946626	0.50017485806234
**Utp4**	Utp4	0.065243807308364	5.03628928622834	4.38385282072838	0.0362808993100351	0.501945166294229
**Prkcg**	Prkcg	-0.119625375020371	6.70481095830873	4.38356002642639	0.0362871315217917	0.501945166294229
**Pdgfc**	Pdgfc	0.159958783894286	2.27078515990729	4.37324341602553	0.0365052983242039	0.504403772190578
**Mavs**	Mavs	0.113737425477659	3.60761523326322	4.37053027049975	0.0365656113837476	0.504578244729003
**Slc6a13**	Slc6a13	-0.196130586806596	4.42505171647333	4.36780208685295	0.036624202528712	0.504769807697279
**Skap2**	Skap2	0.14342568689587	2.88646595242552	4.36645554312388	0.0366531574716466	0.504769807697279
**Trerf1**	Trerf1	0.0743754661307926	4.69996202357572	4.36379989272289	0.036710332594114	0.504999801297421
**Bcas1**	Bcas1	-0.14345629349622	4.84833660453873	4.35328800040553	0.0369375676179571	0.507566113622522
**Atp2b1**	Atp2b1	-0.0453223607155098	8.25780288679722	4.35058945975587	0.0369961608223778	0.507759659645478
**Sptlc2**	Sptlc2	-0.0559618825126946	6.21629998883179	4.34889145056933	0.0370330446231835	0.507759659645478
**Zbtb12**	Zbtb12	0.0965350988577328	5.64583160447468	4.34286767806034	0.0371642773972748	0.508359463307392
**Gpr85**	Gpr85	0.0675078732128386	7.42315723032358	4.34211446844645	0.0371807209816278	0.508359463307392
**Trim16**	Trim16	0.112602313242292	4.52593798099353	4.3412766154586	0.0371990213993457	0.508359463307392
**Lyrm2**	Lyrm2	0.0775785858585352	4.62516474627198	4.33794943201406	0.0372717870465554	0.508796594069882
**Pced1a**	Pced1a	0.0610830577441139	5.08189057149601	4.33451241938292	0.0373471112413367	0.509267657877769
**Cyb5r2**	Cyb5r2	0.291224686893648	0.870431804352718	4.32593946859717	0.0375357099931521	0.510396276092943
**Ncoa2**	Ncoa2	0.0728352824318358	6.1145566789893	4.32532945219383	0.0375491567866659	0.510396276092943
**Rhbdf2**	Rhbdf2	-0.183100498457376	1.77728874034634	4.32517288288321	0.0375525992989759	0.510396276092943
**Fbp2**	Fbp2	-0.374180069717313	-0.28667500872983	4.320642668068	0.0376526991730167	0.510819503298209
**Mcrip1**	Mcrip1	0.0506941295266358	6.39468223708413	4.32005882225371	0.0376565620183891	0.510819503298209
**Lonp2**	Lonp2	-0.0592142292987214	6.23888735149776	4.31594403654617	0.0377568157923259	0.511500315570655
**L2hgdh**	L2hgdh	0.173479920496272	3.31167458358466	4.31015425813024	0.0378855261002262	0.512184332423097
**Kctd1**	Kctd1	0.0687440357852602	5.36355257055872	4.30997987614278	0.0378894076161352	0.512184332423097
**Zkscan5**	Zkscan5	-0.0565417076280386	5.11677860519939	4.30745423069004	0.0379457064852444	0.512332677827936
**Prrc2c**	Prrc2c	0.051060105974851	7.54378964824536	4.30580474682236	0.0379825207975348	0.512332677827936
**Mcfd2**	Mcfd2	0.0642742153422572	5.04488232826999	4.30045891913404	0.0381020901015814	0.513278935855289
**Ophn1**	Ophn1	0.0687788568092327	5.22763272156426	4.29899357087366	0.0381349341618861	0.513278935855289
**Sec24b**	Sec24b	0.0462674444529872	6.58247943517783	4.29550496446703	0.03821324665257	0.513556095277189
**Plcd3**	Plcd3	-0.103557150931717	3.56316772728006	4.29346072045254	0.0382592143013764	0.513556095277189
**Ado**	Ado	0.0752746297202459	4.91312149466179	4.2911613632731	0.0383109877925806	0.513556095277189
**Gclm**	Gclm	0.077458235258205	4.41011306122685	4.29050915009998	0.0383256867210688	0.513556095277189
**Tex10**	Tex10	0.0684017879746295	5.15526728101277	4.28892881275618	0.0383613273061104	0.513556095277189
**Dkk3**	Dkk3	-0.0663346207989222	6.47065721065403	4.28320575817643	0.0384874044786364	0.51412221048893
**Abhd17c**	Abhd17c	0.0455887785041786	7.00872472692137	4.28023769412583	0.03855795585567	0.51412221048893
**Cfap45**	Cfap45	-0.198203232972071	1.76458295128878	4.27871899195114	0.0385924234018915	0.51412221048893
**Ramp3**	Ramp3	-0.198610404959468	2.16308219452851	4.27815132194698	0.0386053151896721	0.51412221048893
**Echs1**	Echs1	0.0647992585263263	5.23841755642676	4.27796081287165	0.0386096426405589	0.51412221048893
**Eml2**	Eml2	-0.0711015018732491	6.8137727967637	4.2753350519157	0.0386693394911226	0.514365357848558
**Pias4**	Pias4	0.0608226123533375	5.48596094401901	4.27306744497766	0.0387209715435572	0.514365357848558
**Snx1**	Snx1	-0.222635160815837	2.48824571298344	4.27113724112429	0.0387649780822446	0.514365357848558
**Srrm4**	Srrm4	0.0696915293063841	6.31683598698176	4.26991798134625	0.0387928028961684	0.514365357848558
**Chrna3**	Chrna3	0.165606565997846	2.29996280788982	4.25751972201484	0.0390769366660376	0.516039157255929
**Mto1**	Mto1	-0.0720000871322244	4.59245036822627	4.25739585366983	0.0390797863717923	0.516039157255929
**Abcb6**	Abcb6	-0.05590736213781	6.71810485660573	4.25566132279187	0.0391197137604556	0.516039157255929
**Cpne9**	Cpne9	-0.261405411050985	2.27478679782462	4.25561467401491	0.0391207881652528	0.516039157255929
**Mical1**	Mical1	-0.0858565290819961	5.3402390006678	4.24976734837929	0.0392557078267547	0.516039157255929
**Robo1**	Robo1	0.0633772207705686	7.75380055767524	4.24606875377754	0.0393413003159012	0.516039157255929
**Ddx3x**	Ddx3x	-0.0523006707685661	8.83048923707104	4.24364229271627	0.0393975594850415	0.516039157255929
**Cldn15**	Cldn15	0.405127782572183	-0.558118289061118	4.2434583092512	0.0394018287090501	0.516039157255929
**Piwil1**	Piwil1	-0.373162871651285	-0.063597996661414	4.24275358308009	0.0394181858898774	0.516039157255929
**Inpp4a**	Inpp4a	-0.0400071335306192	7.92743492980345	4.24216312369404	0.039431896436271	0.516039157255929
**Samd10**	Samd10	0.0597919746115856	5.2140250967247	4.23809776862933	0.0395031545803843	0.516039157255929
**Evl**	Evl	0.033524607551919	9.20031869895487	4.23811563482148	0.0395260141170579	0.516039157255929
**Ano6**	Ano6	0.0631014458985146	5.23976271851638	4.23678455669657	0.0395570176174608	0.516039157255929
**RGD1565**	RGD156568	-0.0940081036916378	4.34955169204021	4.23468694198088	0.0396059270989854	0.516039157255929
**Zfp592**	Zfp592	0.0562066908936574	5.56235535245064	4.23421232190553	0.0396146698833271	0.516039157255929
**Cpsf1**	Cpsf1	-0.0409464732628837	6.75667581951608	4.23399704152583	0.0396220271723232	0.516039157255929
**Arhgap4**	Arhgap4	-0.104653680426917	4.01728849307802	4.23399186103187	0.0396221457602934	0.516039157255929
**LOC3633**	LOC363337	-0.186777852178854	2.2595213318642	4.22221261507409	0.0398857499145007	0.519080762964781
**Tspyl2**	Tspyl2	-0.0677465072962412	5.64452094395054	4.22052103035531	0.039937895229577	0.519067832062809
**Ndst3**	Ndst3	0.141813562008028	2.65551347943573	4.20857967289853	0.0402200005182778	0.521868439420368
**Mybpc1**	Mybpc1	-0.43787114188153	-0.152844165991883	4.20498471122609	0.0403053378895055	0.521868439420368
**Pinx1**	Pinx1	0.104487633681495	3.13489806136978	4.20341547055275	0.0403426478830141	0.521868439420368
**Tigd2**	Tigd2	-0.103312841002914	3.17196481152513	4.20280581012124	0.0403571529182328	0.521868439420368
**Safb2**	Safb2	-0.0778944621396922	6.78632458572417	4.19991442312768	0.0404260193592427	0.521868439420368
**Rdh10**	Rdh10	0.144499936924303	3.02198015175398	4.19909252780826	0.0404456175965689	0.521868439420368
**Col11a1**	Col11a1	-0.113280835733846	5.1781667164908	4.19412302465432	0.0405643289427529	0.521868439420368
**Rn45s**	Rn45s	0.484517846733463	3.10370370332697	4.19398714676764	0.0405675798227264	0.521868439420368
**Fat3**	Fat3	0.0846314476874727	6.71434197810723	4.19268753556257	0.0405986879155987	0.521868439420368
**Sra1**	Sra1	-0.0738354542347784	5.37321690267424	4.19045955259375	0.0406520760320164	0.521868439420368
**Otop3**	Otop3	-0.69809249544854	0.294089704601635	4.18859866237601	0.0406967242145652	0.521868439420368
**Skiv2l**	Skiv2l	-0.0557984956496045	5.79224048479924	4.18697292181227	0.0407381757560321	0.521868439420368
**Lap3**	Lap3	0.0628386280109535	5.16825116338043	4.18655189539044	0.0407458915608272	0.521868439420368
**Araf**	Araf	-0.0468675677388095	7.01563172658263	4.18392332512933	0.0408091264623155	0.521868439420368
**Adarb1**	Adarb1	-0.0683689913036057	7.32860109732414	4.18366950062278	0.0408152381128694	0.521868439420368
**Tcf4**	Tcf4	0.0737291944997386	6.86724187849575	4.18326374971112	0.0408226013264013	0.521868439420368
**Cluap1**	Cluap1	-0.11114006784869	5.66608126270957	4.18058433043538	0.0408890005025678	0.522189912480592
**Cep120**	Cep120	-0.0522042209624027	6.45715213840954	4.17341490885622	0.041062956312571	0.523867592857003
**Mypop**	Mypop	0.100236805774795	3.84475645582661	4.17071426580907	0.0411284575480453	0.52409397020639
**Hemgn**	Hemgn	-0.402763827139214	-0.60863405474222	4.16822148982598	0.0411647103211904	0.52409397020639
**Mcm6**	Mcm6	0.0682823543093568	5.66834149444585	4.16205626403713	0.0413391890778224	0.525331073487791
**Heatr5b**	Heatr5b	0.0455795316061349	7.07326513414703	4.1617736876358	0.0413460858013145	0.525331073487791
**Mcpt2**	Mcpt2	0.773794135370069	-0.347785128757348	4.15842110790567	0.0414280047806729	0.525836435064207
**Hmg20a**	Hmg20a	0.0561427575235958	7.14455433382936	4.1556292222194	0.0414963534193169	0.526168700826034
**Snx14**	Snx14	-0.0971185640736088	4.83518309863637	4.14794884730226	0.0416849899029551	0.527577965870158
**Klc1**	Klc1	-0.0365939047618151	9.87924778859369	4.14668820887178	0.0417160382560047	0.527577965870158
**Htr2a**	Htr2a	0.146755483730315	2.88364241337599	4.14594527514208	0.0417343473842947	0.527577965870158
**Retsat**	Retsat	0.341132566740782	5.00330878283337	4.1416320165568	0.0418408118282924	0.528388470957868
**Nat1**	Nat1	0.111281075039997	3.04312019792555	4.13559601614455	0.0419902782205391	0.52925172494641
**Emid1**	Emid1	-0.10534527746864	4.63221680139749	4.13544578015654	0.041994005598034	0.52925172494641
**Ubr2**	Ubr2	-0.0416325685306802	6.4553683768723	4.13229996642778	0.0420721323679803	0.529701219811494
**LOC6800**	LOC680039	-0.0402510169570622	7.35493968880691	4.12703130286476	0.0422033255026853	0.53048214466704
**Hmox2**	Hmox2	-0.0538442158814138	5.3834331771431	4.12008442986432	0.0423769634037963	0.53048214466704
**Agt**	Agt	-0.176243969085672	4.20329587574907	4.11896517982508	0.0424050093558348	0.53048214466704
**Acot4**	Acot4	-0.204532519181754	2.04684128104823	4.11578229580668	0.0424848721431247	0.53048214466704
**Arpc3**	Arpc3	0.058234904864065	6.56901823622486	4.11562988501412	0.0424887002970213	0.53048214466704
**Bub1**	Bub1	0.111845875061169	3.64090821185771	4.11352384816338	0.0425416354851623	0.53048214466704
**Sp4**	Sp4	0.0751220855113231	5.33625809488913	4.11341459347218	0.0425443834903089	0.53048214466704
**Mrpl36**	Mrpl36	0.0775728923762116	4.74173866112968	4.10930221295171	0.0426479550899365	0.53048214466704
**Cpe**	Cpe	-0.0472204795503282	10.040950837102	4.10804140815134	0.0426797518250595	0.53048214466704
**Myh8**	Myh8	0.425309627291284	-0.313265205738964	4.1077359808771	0.0426874708151351	0.53048214466704
**Zbtb9**	Zbtb9	0.106260917371294	3.86613747262434	4.10755584525391	0.0426920180689487	0.53048214466704
**Fzd6**	Fzd6	0.103135813827427	3.47793011737578	4.10691877307869	0.0427081040872437	0.53048214466704
**C1qtnf2**	C1qtnf2	-0.153909925777696	2.38549170862209	4.10466357570896	0.0427650988573995	0.53048214466704
**Akirin2**	Akirin2	0.0447483245205039	6.93752089214289	4.10432223506682	0.0427737323993438	0.53048214466704
**Zpr1**	Zpr1	0.045550928419491	6.02063995251717	4.10323491028139	0.0428012465661034	0.53048214466704
**Dctn5**	Dctn5	-0.0435043665808639	6.12840403378812	4.10209788581128	0.0428300378531192	0.53048214466704
**Ehd1**	Ehd1	0.0408914468887799	7.64928699235781	4.10094117547343	0.0428593487191971	0.53048214466704

Pign	Pign	0.167521757171698	3.73271505401901	4.09935983774987	0.0428994527123542	0.53048214466704
Hmgn1	Hmgn1	0.0724257371375729	6.88459094328876	4.09744467682933	0.0429480777847028	0.530557590613601
Vps37a	Vps37a	0.070519663876083	4.27726110960534	4.09553046482799	0.0429967356918578	0.530633304750287
Arid5a	Arid5a	-0.197391007316941	1.87911076601668	4.09301427868373	0.0430607825223092	0.53089861279816
Ccng2	Ccng2	0.0705432737401353	6.30960571267026	4.0892940640832	0.0431559183697943	0.53154629170772
Tmem150b	Tmem150b	-0.167360035031897	3.29879299855603	4.08423363222886	0.0432850762355058	0.53223531935312
Tek	Tek	-0.0971789462314802	4.19010870400981	4.0823874246733	0.0433067565705231	0.53223531935312
Ntn1	Ntn1	0.181793743639281	4.25022540899642	4.08164861793601	0.0433513416785466	0.52237665507112
Zfp458	Zfp458	0.181011362906111	2.2848376686959	4.07187622812615	0.0436028174540689	0.53422316950158
March8	March8	0.0449364878043283	6.49626481021444	4.07091135045862	0.0436277300419886	0.53422316950158
Hipk1	Hipk1	0.0435412923496777	7.2828795590795	4.07081756767963	0.0436301522579224	0.53422316950158
RGD1306	RGD130674	-0.0763930856671343	3.98065925205771	4.06804418003414	0.0437018471435517	0.53450329078
Hexim2	Hexim2	-0.10856988243440	2.4580253614888	4.06559475602807	0.0437626791174222	0.53450329078
Opn3	Opn3	-0.111431111202786	2.81150874261593	4.06496674370816	0.0437815471014635	0.53450329078
Snw1	Snw1	0.060371538093684	5.52940642408412	4.05965870486443	0.0439193751778796	0.535661822184168
Clvs2	Clvs2	0.0932857517695643	2.9593973527384	4.0526379067503	0.0441031620371231	0.53695977800081
Ly6g6c	Ly6g6c	0.345244871439862	0.258092762478743	4.05021369877548	0.044165753653032	0.53695977800081
Fam120b	Fam120b	0.0515150197540277	6.84340221405475	4.04701142664239	0.0442496166199334	0.53695977800081
Tnnt2	Tnnt2	-0.238928348249425	2.80705699882145	4.04423605230453	0.0443224352926215	0.53695977800081
Itgbl1	Itgbl1	-0.120925962484015	2.9628382701791	4.04246576840938	0.0443689489020521	0.53695977800081
Rbm34	Rbm34	-0.0534345063675447	5.36455279892391	4.03865047780876	0.0444746397864141	0.53695977800081
Prpf19	Prpf19	0.0307331555177297	8.27205377147841	4.03643512237971	0.0445277880390189	0.53695977800081
Efhd1	Efhd1	-0.253479402069459	1.46981624523599	4.03600616495822	0.0445391088826238	0.53695977800081
Rasa1	Rasa1	0.0592346253926925	6.70435484092069	4.03564580960557	0.0445486190983366	0.53695977800081
Jmjd8	Jmjd8	-0.0836497774925347	5.11738529188394	4.03509719401859	0.0445631086089894	0.53695977800081
Pelp1	Pelp1	-0.0395781320118733	7.04561506058814	4.03357050137812	0.0446034479195288	0.53695977800081
Ccdc86	Ccdc86	0.083494879497841	3.89863092484042	4.03316951569704	0.0446140494786846	0.53695977800081
Ctnnal1	Ctnnal1	-0.0824986028399182	4.68358497548018	4.0321032618256	0.0446422698046398	0.53695977800081
Skor1	Skor1	0.737521566189274	0.524942210470905	4.03049635847069	0.0446847922321225	0.53695977800081
Col5a1	Col5a1	-0.103218807865255	5.51138115812349	4.02570885842201	0.0448117845257807	0.53695977800081
Trpv2	Trpv2	0.0431629026893285	6.39472975858757	4.02195524247911	0.0449116179807201	0.53695977800081
Rb1	Rb1	0.0660809655613799	6.09115052343596	4.02152474800096	0.044923082623697	0.53695977800081
Lmx1b	Lmx1b	-0.884764887886212	0.0536396485233257	4.02123675254063	0.0449307540645773	0.53695977800081
N4bp3	N4bp3	0.117864679983308	4.13378735273626	4.02065088624626	0.0449463642378106	0.53695977800081
Zfp597	Zfp597	0.0720274134943873	4.41003233502286	4.02055263983964	0.0449489825204786	0.53695977800081
Myom3	Myom3	0.319466740251711	0.177198383209446	4.01984515976687	0.0449678418263295	0.53695977800081
Zfp397	Zfp397	0.10490329470097	3.48218612469732	4.01966720035485	0.0449725870010559	0.53695977800081
[illegible]	[illegible]	[illegible]	[illegible]	[illegible]	[illegible]	[illegible]

Abcg1	Abcg1	-0.0621538928899008	5.1324239496732	3.47977813789146	0.0621230681348581	0.575538712822253
Acaca	Acaca	0.0481541706322851	8.0773591069401	3.4785806417463	0.0621680411065429	0.575538712822253
Stx6	Stx6	0.0362788130970833	6.9003896104137	3.47824887752865	0.062180506823491	0.575538712822253
F2rl1	F2rl1	-0.25136224394629	0.251019014756144	3.47491229764053	0.0623060248203713	0.576272996059134
Slc15a3	Slc15a3	-0.111544455687418	4.26255592081234	3.46951129067903	0.0625297762686704	0.577547772921584
Tfpt	Tfpt	0.0792252910429706	4.29183031830052	3.46754518779381	0.0625841230976485	0.577547772921584
Calm3	Calm3	-0.0352412798641993	9.60542938442246	3.46693832213292	0.0626109753747927	0.577547772921584
LOC49812	LOC498122	-0.0859361340803653	4.0291299131185	3.46625940177275	0.0626290083163343	0.577547772921584
Tbl3	Tbl3	-0.045293407529313	5.54509825771274	3.46461545778357	0.0626950838592247	0.577730102523003
Ccser1	Ccser1	0.0905166811196632	3.58284617717324	3.46247215527723	0.0627763918614178	0.577791471068518
Sec23ip	Sec23ip	-0.0743050328094684	6.37174734725886	3.46199889889306	0.0627943604046574	0.577791471068518
Adamts14	Adamts14	-0.257907842927864	0.844202582615936	3.45926210873379	0.0628845742475146	0.578287106032601
Znfx1	Znfx1	0.0668931936349771	4.68749420252525	3.45725116177604	0.0629749257367415	0.578599520188487
Igsf3	Igsf3	0.0455765781128994	8.08903948809654	3.45554359430974	0.0630400029539832	0.578771241248693
Eva1a	Eva1a	0.175971766106476	1.55213429492952	3.45353424817441	0.0631166730866366	0.579049066251444
Cep95	Cep95	-0.0988382184528879	4.98779691091536	3.44807621410557	0.0632756460059394	0.580080995750262
Arl4	Arl4	0.0352913254012117	8.29206215842878	3.44781769925726	0.0633353421796702	0.580201956223014
Rbx	Rbx	0.150185826589666	2.73647892721828	3.44603390736611	0.0634037408851102	0.580452402805224
Decr1	Decr1	-0.117069012387576	3.56715717520274	3.44468282769799	0.0634555998421596	0.58045126043301
Lbp	Lbp	-0.349430974513402	-0.402452161018288	3.43701679659351	0.0637507052914185	0.582723479795625
Mdfic	Mdfic	0.128108456502229	3.03857694634278	3.43449015818123	0.0638482891138883	0.58313689278042
Man2b1	Man2b1	-0.0707359698783951	5.1513009309102	3.43295644325422	0.0639076018295569	0.58313689278042
Pnpla7	Pnpla7	-0.104160009112581	2.84137970159024	3.43221883999236	0.0639361440509429	0.58313689278042
Pop1	Pop1	0.0954648549405159	3.75867887965049	3.42578744092104	0.0641856265550359	0.583756233394781
Atp6v1b2	Atp6v1b2	-0.0349609809737127	8.38614972248574	3.42528188877078	0.0642052773678039	0.583756233394781
Tmem151	Tmem151a	-0.0680000913752509	6.63145887778161	3.42527652320199	0.0642054898915679	0.583756233394781
Naaa	Naaa	-0.160467064919289	1.5221911167945	3.42499781769549	0.0642163281011513	0.583756233394781
Fndc3a	Fndc3a	0.0504716882863102	6.02822201410134	3.42444112152717	0.0642379825640005	0.583756233394781
Tbp	Tbp	0.0709738888915741	4.39009997799818	3.41853407409818	0.0644682363274864	0.584917257193346
Dock9	Dock9	-0.05656975780591	7.93875758232761	3.41826550742508	0.0644787258248008	0.584917257193346
Gria4	Gria4	-0.0654249132502001	6.72476811824613	3.41690277642233	0.0645319785260846	0.584917257193346
Supt20h	Supt20h	-0.0579964885775115	5.75780957986469	3.41606947251891	0.0645646045478302	0.584917257193346
Pbx3	Pbx3	0.119016274566967	5.99176584600517	3.4142480901332	0.0646359312700643	0.584917257193346
Pgap4	Pgap4	-0.0427637855916984	6.61483086567031	3.41396290858896	0.0646470223346657	0.584917257193346
Cxcl17	Cxcl17	-0.360571273087477	-0.589969279710406	3.40884455670596	0.0648478377707507	0.585274575507959
Cnbp	Cnbp	-0.0373967462113448	8.12366457075812	3.40866585623053	0.0648548668785146	0.585274575507959
Efnb1	Efnb1	0.0662366145245567	6.01128101756945	3.40471905084731	0.065010182855091	0.585274575507959
Crem	Crem	0.106590511842186	3.34188264746529	3.40446390420619	0.0650210267922671	0.585274575507959
Tbc1d23	Tbc1d23	-0.0431924440274696	6.45918192383373	3.40425932328517	0.0650243603222193	0.585274575507959
Ctnnd1	Ctnnd1	0.0380186240043082	7.44190236297596	3.40352398772267	0.0650572927373882	0.585274575507959
Spata7	Spata7	-0.0715497909715909	4.6684267071772	3.40325549687804	0.0650678815438847	0.585274575507959
Commd4	Commd4	-0.075092931075922	5.97307999863321	3.4029248443955	0.0650809244234829	0.585274575507959
Ccdc92	Ccdc92	0.0464073601814804	5.84925254755566	3.4021032526016	0.0651133448200842	0.585274575507959
Itga6	Itga6	0.055601756832487	5.51060735768859	3.40070824244862	0.0651685192452462	0.585274575507959
Cc2d1a	Cc2d1a	-0.0532867091669626	7.13187049509687	3.39935539440948	0.0652219022173943	0.585274575507959
Mt3	Mt3	-0.110201452165124	6.02711435291382	3.39750854464508	0.0652949729455861	0.585274575507959
Gtf2h3	Gtf2h3	0.0721869681738207	4.43756401839302	3.3970046179621	0.0653149258525843	0.585274575507959
Larp7	Larp7	-0.0518706009534859	5.35455940950561	3.39625441243233	0.0653446423428231	0.585274575507959
Map3k9	Map3k9	0.0549261213357482	6.38900221559097	3.39322560531189	0.0654647636567743	0.585274575507959
Caprin1	Caprin1	0.0456712945216772	8.8512041427471	3.38957037938134	0.0656100424779691	0.585274575507959
Dus2	Dus2	0.0780678954741712	4.50093618882772	3.38947496143133	0.0656138395197871	0.585274575507959
Zfp358	Zfp358	0.0551573692989499	6.03644069315211	3.38879524330431	0.0656408948690303	0.585274575507959
Lin54	Lin54	-0.0632070795787675	4.61299124250827	3.3885067300502	0.0656523823809649	0.585274575507959
Lca5	Lca5	0.105865807003848	3.77994782069728	3.38828499562271	0.0656612124802416	0.585274575507959
Fbxo21	Fbxo21	0.0361901658262231	7.47148210163771	3.38787517762108	0.065677525893215	0.585274575507959
Arih1	Arih1	0.0343601487694599	7.35213219670389	3.38694729783777	0.0657184964564119	0.585274575507959
Sec5d	Sec5d	-0.188535226190469	2.47805208944183	3.38120490264212	0.0659438295871594	0.586862454892288
Synj2bp	Synj2bp	0.0532584846436041	5.8854476835319	3.37726492289378	0.0661097652661998	0.587308260287594
LOC25765	LOC257650	-0.136818006091682	2.2054763008132	3.37475340702584	0.0662024861400852	0.587308260287594
Sowahc	Sowahc	0.0613277088343707	4.9638693221516	3.37437261612367	0.066217786822513	0.587308260287594
Daw1	Daw1	-0.290085607878835	0.205261108864656	3.3743687899157	0.0662179405941435	0.587308260287594
Il1rapl1	Il1rapl1	0.103584158500344	2.36202671996547	3.37266952524712	0.0662862655070206	0.587308260287594
Ybx1-ps3	Ybx1-ps3	-0.150742461078921	5.59560457624525	3.37177659705063	0.0663221990077305	0.587308260287594
Mrm3	Mrm3	0.0936609193158478	3.52720544084163	3.37128195327092	0.0663376943763457	0.587308260287594
LOC10254	LOC102549	0.103282327059568	3.19053991904263	3.37057731925497	0.0663704934684225	0.587308260287594
Sephs1	Sephs1	0.0405288733289935	6.45402589315888	3.36646755773495	0.0665362771299007	0.588357994241347
Gipr	Gipr	-0.142638972445389	6.15312670254339	3.36343920119994	0.0666587210751911	0.589023273676741
Gnl3	Gnl3	-0.0628672394914175	5.67584284826366	3.36001305914216	0.0667975387647292	0.589690982811425
Shc4	Shc4	-0.12829687140061	2.25506633795586	3.3589186303382	0.0668419470226035	0.589690982811425
Cdk5r1	Cdk5r1	0.0515289746231053	8.15293184281887	3.35807817686455	0.0668760712253079	0.589690982811425
Acp1	Acp1	-0.0624517025100806	5.32504839425851	3.3528429616899	0.0670890516843024	0.590582134898936
Dhdds	Dhdds	0.0659689778286045	5.51640157068238	3.35278598080104	0.0670913737799831	0.590582134898936
Csrp1	Csrp1	-0.0783171734559507	4.2008672302905	3.35116502975741	0.0671574670646205	0.590582134898936
Cnn3	Cnn3	0.0492239112510673	7.71132920683711	3.35094435655397	0.0671664702590038	0.590582134898936
Ccr10	Ccr10	-0.287066246387543	0.192282741722359	3.34908282501026	0.0672424698057037	0.590737259719599
Fkbp1a	Fkbp1a	-0.0288198480295431	8.56492153858997	3.34611309200608	0.0673638049161656	0.590737259719599
Dcun1d5	Dcun1d5	-0.0488907767008643	5.86937620625218	3.34497747023607	0.0674103401522434	0.590737259719599
Sgsm2	Sgsm2	0.0372565326531208	7.16812111976315	3.34387862877215	0.0674555082047638	0.590737259719599
Ppp1r9a	Ppp1r9a	0.0557715842246163	6.68006031800692	3.3438522099538	0.0674565091249481	0.590737259719599
Nlrx1	Nlrx1	-0.123407528158896	3.49483207014538	3.34354384587463	0.0674691461442158	0.590737259719599
Lynx1	Lynx1	-0.116995318643286	4.90758211547746	3.34200745073481	0.0675321711854505	0.590737259719599
Avpr1a	Avpr1a	0.176098613303102	1.50322191633032	3.34008379780491	0.0676111656706282	0.590737259719599
Mtmr11	Mtmr11	-0.10915081718823	3.50626274301652	3.33817313175178	0.0676485964643615	0.590737259719599
Hspa5	Hspa5	0.0819462940142525	8.54712849519223	3.33760546424544	0.0677130826655256	0.590737259719599
Rab7b	Rab7b	-0.195407403026735	1.1663062091726	3.33514325032951	0.0678145014280047	0.590737259719599
Amz1	Amz1	-0.084201691305072	4.5040014616692	3.33321440969038	0.0678899358670858	0.590737259719599
Aga	Aga	-0.0813937742810704	3.70505096323854	3.33217744617365	0.0679368775785748	0.590737259719599
Nuak1	Nuak1	0.0701679185847182	5.45436401073307	3.33208692731842	0.0679406163136907	0.590737259719599
Luzp1	Luzp1	0.0527536532382338	5.29034163943558	3.33076921140646	0.0679951996490547	0.590737259719599
Slc22a12	Slc22a12	-0.12808037112517	2.62953968377584	3.33034324459476	0.0680126792665607	0.590737259719599
Zfp39	Zfp39	0.0903935035467738	4.04787118725425	3.32980324233732	0.0680301734277935	0.590737259719599
Ngrn	Ngrn	-0.0431896155034522	6.33521350200485	3.32857641907917	0.0680857820390919	0.590737259719599
Hspb8	Hspb8	-0.15253948228799	2.66740385277112	3.32753618158804	0.0681288613751639	0.590737259719599
Trim32	Trim32	0.0355481585163547	7.12119774189669	3.32748372713481	0.0681310344422941	0.590737259719599
Snx13	Snx13	0.0624755540877685	5.44241974419149	3.32456428612493	0.0682520971034549	0.591375983027643
Ryk	Ryk	0.0668505547232024	5.33821096292896	3.32194386385385	0.0683609560631122	0.591724565852176
S100pbp	S100pbp	0.0513470588546343	5.55556645300278	3.32090307632587	0.0684039532761544	0.591724565852176
Ypel2	Ypel2	0.102758215860339	3.16199554755352	3.31900585229703	0.0684832293499348	0.591724565852176
Acat2	Acat2	0.0512508998409151	8.13553551475251	3.31803077942754	0.0685238613673865	0.591724565852176
Chchd1	Chchd1	-0.0745147395967121	4.71812995742241	3.31764804106493	0.0685399007401722	0.591724565852176
Ubp1	Ubp1	-0.0489919174289831	7.10536068585392	3.31581455469707	0.0686163089224034	0.591724565852176
Cdk9	Cdk9	-0.0454591790152675	6.44005061642508	3.31446909598456	0.0686716828576457	0.591724565852176
Lama3	Lama3	-0.106352799647299	4.00488468903142	3.3130230347065	0.068732944296351	0.591724565852176
Tpcn1	Tpcn1	-0.05688751472828	5.93761962779079	3.31150494825059	0.0687964620502531	0.591724565852176
Sytl2	Sytl2	-0.136301316921083	3.09441964441987	3.31142357189506	0.0687998686643408	0.591724565852176
Lss	Lss	0.092170947954312	7.14555718640485	3.31108592372404	0.0688140053740087	0.591724565852176
Commd5	Commd5	-0.0750297962252855	4.15256102543936	3.30887623996311	0.0689065975341257	0.591974920714142
Tgfbr2	Tgfbr2	0.119845152975202	3.44389849818181	3.3074477811834	0.0689665250337126	0.591974920714142
Rcn2	Rcn2	0.039256952627348	7.31179486011637	3.30699680980022	0.0689854560165294	0.591974920714142
Spint2	Spint2	-0.0655596980811141	6.08134559402203	3.30511376859885	0.0690645828640363	0.592246426704179
Tmod1	Tmod1	0.162745984543117	1.51849073950892	3.2978001240747	0.0693727321008644	0.593728220981385
Trim46	Trim46	0.0377373963121003	7.30229980311119	3.29678287198183	0.0694157116884508	0.593728220981385
Scrg1	Scrg1	-0.10465502704685	3.06142670496653	3.29660157748943	0.0694227743779649	0.593728220981385
Tcirg1	Tcirg1	-0.124193717179674	2.94672571493854	3.29649913210784	0.0694277049300185	0.593728220981385
Lyn	Lyn	-0.0899998035839079	3.67318551244251	3.29440481183887	0.0695162975630896	0.594078660749773
Pou3f4	Pou3f4	0.146721871191603	3.22080537394442	3.28710481171535	0.0698260446974338	0.595970396896002
Rab11a	Rab11a	0.0470576292534085	5.82887195541817	3.28693680677848	0.0698331907025459	0.595970396896002
Cirbp	Cirbp	-0.0644455893839351	7.05768281611821	3.28510349644753	0.069911220551275	0.596043407811154
Elovl6	Elovl6	0.0675175514248684	5.503942355275	3.28449151766092	0.0699372885237444	0.596043407811154
Fbxo30	Fbxo30	0.0695932518594643	5.47104279021818	3.27970045989899	0.0701417295038841	0.597377719465933
Clic4	Clic4	0.0851796751775962	4.42755708874124	3.27724668857284	0.0702468829939206	0.597449932672523
Rgs11	Rgs11	-0.166847730341114	3.02603091236729	3.27637372892623	0.0702840520474865	0.597449932672523
Letm2	Letm2	-0.0879319108912093	3.52970195147386	3.27595117747671	0.0703021628283738	0.597449932672523
Smtnl2	Smtnl2	-0.121002769542782	2.59285501540185	3.27368493905096	0.0703993266111863	0.597449932672523
RGD1311	RGD131108	-0.229664472492191	0.720752524518277	3.27212210988057	0.0704664158745503	0.597449932672523
Igf2bp1	Igf2bp1	0.202312494438805	1.71326125368942	3.27177458155522	0.0704813439045522	0.597449932672523
Fam217b	Fam217b	0.0649832117374263	4.72344616263415	3.27168023336275	0.0704853972023663	0.597449932672523
Prr18	Prr18	0.0749151737048023	4.55248579044553	3.27002503520254	0.0705565468854892	0.59764700304637
Zranb2	Zranb2	-0.0637520598989164	7.24429714942621	3.26760097447164	0.070658300880361	0.59810286302103
Hddc2	Hddc2	0.0936362812222415	3.16496436394294	3.26421935428496	0.0708023902476292	0.598260713223027
Thbs1	Thbs1	-0.150001564010532	4.34448290245404	3.26399028922714	0.0708165606308691	0.598260713223027
Casp8ap2	Casp8ap2	0.0747852976155028	4.81210587439792	3.26389325751344	0.0708207961407498	0.598260713223027
Enpp2	Enpp2	0.117128046840759	5.90681558466013	3.26029417274353	0.0709763924829065	0.598611707987345
Lnc134	Lnc134	0.117522268838995	2.7793229094628	3.25841809179474	0.0710142968035104	0.598611707987345
Rad51ap1	Rad51ap1	-0.0904249625686676	3.27395281121154	3.25806167777671	0.0710730921707622	0.598611707987345
Nsun7	Nsun7	-0.155609887184831	1.49126047044821	3.25677529021311	0.0711298713246136	0.598611707987345
Rassf3	Rassf3	-0.0777916425338051	4.52165749827689	3.25623708451231	0.0711476888239595	0.598611707987345
Abl2	Abl2	0.0660242732621876	4.65761021792929	3.25628361174666	0.0711503094209531	0.598611707987345
Rap2b	Rap2b	0.0768773927214685	3.98878225336811	3.2522755805397	0.0713243724746887	0.59867263838709
Itch	Itch	0.0661019218561233	5.11951181724072	3.25070757588272	0.0713926326761869	0.599707726201735
Chchd4	Chchd4	-0.171799389573201	3.99368087756077	3.24892890516705	0.0714527072694806	0.599707726201735
Snrpa1	Snrpa1	-0.0691481460869758	5.16103139252803	3.24879170446067	0.0714765564086637	0.599707726201735
Orai2	Orai2	0.102756762593504	6.51227963989061	3.24525284585602	0.0716306375457979	0.599707726201735
Srpx2	Srpx2	-0.376547073245406	-0.352469872166978	3.24454430089598	0.0716616157435435	0.599707726201735
Sppl2b	Sppl2b	-0.0534634526384232	6.5938209072369	3.24452523077947	0.071662449703933	0.599707726201735
Aldh4a1	Aldh4a1	-0.0740401951450809	4.58849542514615	3.2444658877434	0.0716650011835206	0.599707726201735
Pfn1	Pfn1	0.0449552668994067	7.86734002511616	3.24063031776588	0.0718329974231063	0.600710662850448
Ascc2	Ascc2	-0.0541689864634748	4.58755268223213	3.23828131582797	0.0720191064696173	0.601258227406327
Ears2	Ears2	0.133544355494149	2.36672289963336	3.23625628808846	0.0720250432044118	0.601258227406327
Cnp	Cnp	-0.06091412369319	6.42778847749741	3.23436427037225	0.07210828447389	0.601258227406327
Natd1	Natd1	0.0464271366771279	5.4081144265859	3.23285110209459	0.0721749320964584	0.601258227406327
G2e3	G2e3	-0.0809714958457466	4.7942808130385	3.23269473742417	0.0721818229557189	0.601258227406327
Msra	Msra	-0.0665592430820304	5.59093747749086	3.23025364438691	0.0722894913902543	0.601258227406327
Mkx8	Mkx8	-0.0694415503084361	4.73053269469652	3.2283445532077	0.0723738150059547	0.601258227406327
Lonrf3	Lonrf3	0.146239826477308	2.32750062011367	3.22529661676405	0.0725091024342351	0.601258227406327
Manf	Manf	0.0824611161111449	5.18801492367544	3.2228534994742	0.0726169407282765	0.601258227406327
Otp	Otp	0.647549940094324	2.36318667296801	3.22188631346016	0.0726598551881349	0.601258227406327
Gfer	Gfer	0.0836132331253424	3.9818902767826	3.22180023173295	0.0726636758894607	0.601258227406327
Creg1	Creg1	0.109811583705231	3.87489685903729	3.2199162267713	0.0727473529422047	0.601258227406327
Nek9	Nek9	-0.0456632529737677	6.38405656187795	3.21946686752757	0.0727673222252791	0.601258227406327
Rpl12	Rpl12	-0.055927067713199	8.0082040895393	3.21628455827897	0.0729044884950383	0.601258227406327
Akap10	Akap10	0.0634379477845767	4.73315124637907	3.21264757107471	0.0730800873091219	0.601258227406327
Rspo3	Rspo3	0.148284904174194	2.71848956347338	3.21192743320001	0.0731033205849138	0.601258227406327
Tepp	Tepp	0.159030912109459	1.33704109674586	3.21188041156668	0.0731054213159222	0.601258227406327
Gper1	Gper1	0.166613247081545	0.935433055820981	3.211823215654	0.0731079722108041	0.601258227406327
Flrt3	Flrt3	0.0767379727975172	4.7942434401732	3.21126872401153	0.0731327548729817	0.601258227406327
Sox12	Sox12	0.0639681223694263	7.32481582839111	3.21028558892117	0.0731767097215802	0.601258227406327
Pbx1	Pbx1	0.0585939457022253	8.96452223398717	3.20809458180121	0.0732747699602011	0.601258227406327
Capg	Capg	0.293999962001467	1.56234992550962	3.2080574581406	0.0732764318145723	0.601258227406327
Ifitm3	Ifitm3	-0.163245134930586	1.57624231455645	3.20720940701651	0.0733144255069964	0.601258227406327
Rfxap	Rfxap	-0.0575896545182504	6.03536760668698	3.2071388048548	0.0733175895165612	0.601258227406327
Col7a1	Col7a1	-0.129620079451053	2.43203274350359	3.20708251589765	0.0733201121991372	0.601258227406327
Ankzf1	Ankzf1	-0.0665189875147011	5.36403979604362	3.20368882140048	0.073472377899618	0.601258227406327
Ano1	Ano1	-0.198673650464437	1.41887278740697	3.20285508621282	0.0735098371973023	0.601258227406327
Tmem198	Tmem198b	0.0829354088580746	3.75358541461523	3.20065818961393	0.0736086408373397	0.601258227406327
Tufm	Tufm	0.0446207402009946	6.39770978911652	3.197897687491	0.0737329942918586	0.601258227406327
P2rx3	P2rx3	0.242060558688388	0.0525676216184205	3.19723689404995	0.0737582845769819	0.601258227406327
Tbck	Tbck	0.06292367728411	5.79181465836394	3.19711975933707	0.0737680786656495	0.601258227406327
Gpd1	Gpd1	-0.135365772158484	3.19768479086072	3.19668756208925	0.0737875783713727	0.601258227406327
Aldh7a1	Aldh7a1	-0.0563667152431037	5.35291032429518	3.19523946387472	0.0738529886640389	0.601258227406327
R3hdm4	R3hdm4	-0.0396682500758834	7.7399422329647	3.19503674367468	0.0738621103628889	0.601258227406327
Abhd1	Abhd1	-0.0837216004282191	3.56476094365573	3.19330321450235	0.0739404636167482	0.601258227406327
Klhl42	Klhl42	0.0847282456901823	4.07870324932995	3.19257579282562	0.0739733688909868	0.601258227406327
Pfkfb4	Pfkfb4	-0.0573662524313876	4.6502679818958	3.19012271098805	0.0740844503426023	0.601258227406327
RT1-DOb	RT1-DOb	-0.232929345785315	1.02663652313657	3.18997155175262	0.0740913010633339	0.601258227406327
Tpbg	Tpbg	0.090084336145903	5.09267652344286	3.18956489319361	0.074109734701238	0.601258227406327
Csrnp2	Csrnp2	0.0463924098594156	6.86281118323399	3.18910384675353	0.074130635136425	0.601258227406327
Tmem68	Tmem68	-0.0493454550134552	5.98311123923924	3.18791046088183	0.0741847801156666	0.601258227406327
Rbm11	Rbm11	-0.130898692527789	2.45968238713908	3.18749427159453	0.074203671383257	0.601258227406327
Cd164l2	Cd164l2	0.271952057795046	0.0914759580467326	3.1854981109811	0.0742943510768834	0.601258227406327
Dus1l	Dus1l	-0.0716459384386043	4.62356482673622	3.18500083201261	0.0743169594973172	0.601258227406327
Kdsr	Kdsr	-0.0673392227367672	3.88846299799227	3.18490618345666	0.0743203526769574	0.601258227406327
Faf1	Faf1	0.0430378731781958	6.46113986245514	3.1826667791086	0.0743776477369227	0.601258227406327
Pgr	Pgr	-0.123551568290025	3.32982798360526	3.18214244047441	0.0744470575343849	0.601258227406327
Marcksl1	Marcksl1	0.0566790576936876	9.3972715771912	3.18188431756514	0.0744588178828729	0.601258227406327
Acads	Acads	0.0852700323260767	4.18636202865727	3.18123651842985	0.0744846944240512	0.601258227406327
Mtfr1	Mtfr1	0.122702094570783	3.0396712493705	3.18008432137588	0.0745408820667803	0.601258227406327
Mn1	Mn1	0.0700305218539058	7.62110431409044	3.17958285149536	0.0745633069726734	0.601258227406327
Riiad1	Riiad1	-0.22777779382429	0.577322737976568	3.17956731252292	0.0745644724166163	0.601258227406327
RGD1559	RGD155988	0.0369012459307934	7.29091582579103	3.17949724494393	0.0745676696785025	0.601258227406327
Sf3b5	Sf3b5	0.0689920974356449	5.5637055283519	3.17846028948341	0.0746150090675124	0.601258227406327
Atl2	Atl2	-0.0496227475905051	5.86141808905328	3.17783300325675	0.0746436616043247	0.601258227406327
Igsf10	Igsf10	0.0676509670901115	4.88397868285788	3.17708797209851	0.0746777077266285	0.601258227406327
Xrcc1	Xrcc1	-0.0666414767641573	6.34904265508813	3.17707899630558	0.0746781179998582	0.601258227406327
Samd15	Samd15	0.140770774152157	1.68779612426987	3.17607669664301	0.0747239472543183	0.601258227406327
LOC36719	LOC367195	0.260572342600393	0.45057756630651	3.17568980770125	0.0747416454842681	0.601258227406327
Oxt	Oxt	-0.848543437223051	1.58289405392468	3.17440816515641	0.074800306375022	0.601342411495586
Sik1	Sik1	0.0763894707287762	4.85269626456029	3.17182587929101	0.0749181894011506	0.60176536865378
Utp20	Utp20	0.0730854960810496	5.64675256308539	3.17115806182265	0.0749493774856115	0.60176536865378
Uap1	Uap1	0.0525464044190686	5.60210145427638	3.16897948857589	0.0750493722541685	0.602025667541457
Cbx6	Cbx6	0.0460119194802797	7.56597089615389	3.16810870368264	0.0750893530642651	0.602025667541457
Vps16	Vps16	-0.0380740458427059	6.16475405777776	3.16729982976386	0.0751265500009657	0.602025667541457
Zdhhc8	Zdhhc8	0.0602760462098031	5.81512832860311	3.16553389424459	0.0752078417629603	0.602119350199801
Gpx4	Gpx4	0.0454211343853238	7.57207381889869	3.16446283159431	0.0752571895343197	0.602119350199801
Smyd5	Smyd5	0.0395910047313738	6.00321282827651	3.16390259250322	0.075283015653738	0.602119350199801
Zic2	Zic2	-0.147581818577118	6.09964285744508	3.16093713058213	0.0754196773701535	0.602827552817044
Mfsd4a	Mfsd4a	-0.14422322309981	2.26634552365032	3.15967035049733	0.0754784231582632	0.602877375315429
Ltn1	Ltn1	0.0432557152757527	6.20907248126212	3.15871185842371	0.0755227488673572	0.602877375315429
Ptgr2	Ptgr2	-0.0683786899099118	4.97064124281768	3.15614747606202	0.0756414940068649	0.603439207623819
Qrsl1	Qrsl1	0.080012254231175	3.54553574849064	3.15487976541485	0.0757002678529855	0.60345050230095
Tut4	Tut4	0.0637407929020719	5.85245427100457	3.15227293321508	0.0758212806011715	0.60345050230095
Afmid	Afmid	-0.135088697311843	1.8974880797273	3.15159909852832	0.0758525947439308	0.60345050230095
Prom1	Prom1	-0.0611774510590382	4.66121254110141	3.15021053571609	0.0759171572561493	0.60345050230095
Lrrc14	Lrrc14	0.0543271998509544	4.81175098121724	3.14897485110198	0.0759281331918954	0.60345050230095
Plau	Plau	0.394365520040544	-0.267063151500216	3.14826719682921	0.0759564129849012	0.60345050230095
Sipa1	Sipa1	0.0755024892103456	4.54500633268732	3.14682914179871	0.0759814655252069	0.60345050230095
Mcm4	Mcm4	0.0645016328365392	5.49211092408933	3.1446614496321	0.0761664583408161	0.604523121282391
Wdr55	Wdr55	0.0689150439156647	3.91943420476415	3.14377374088578	0.0762172711234347	0.604523121282391
Nle1	Nle1	-0.0772007524962381	4.16310844701738	3.14279758170075	0.0762628940436358	0.604523121282391
Stard7	Stard7	-0.033827927610403	6.74882542572497	3.13903313572374	0.0764391087045275	0.604716241285583
Itpkc	Itpkc	0.0703257066257921	4.37724861590199	3.13889684905373	0.0764454965238322	0.604716241285583
Cep162	Cep162	-0.069590228446213	4.03048104604441	3.13777298824978	0.0764981942878748	0.604716241285583
Cd59	Cd59	-0.0601180984979047	6.03615785821343	3.13615219840642	0.0765742622473034	0.604716241285583
Pex11b	Pex11b	-0.0527905290379503	5.1441822530912	3.13560308240423	0.0765991121093913	0.604716241285583
H6pd	H6pd	-0.103549728530089	2.86338696710491	3.13522485681248	0.0766178301558523	0.604716241285583
Cycs	Cycs	-0.0523072438756722	6.99514011839121	3.13398985899231	0.0766801045756768	0.604716241285583
Pik3ca	Pik3ca	0.0401716103089921	6.94828573153186	3.13130818332137	0.0768020802486607	0.604716241285583
Slco1a4	Slco1a4	-0.218635832403031	1.61519166651051	3.13091863302147	0.0768205256799532	0.604716241285583
Exo1	Exo1	0.163281715213416	1.37476627666083	3.13002984537282	0.0768623332537614	0.604716241285583
Ep400	Ep400	0.0398769337560164	7.63655193572839	3.1295580089551	0.0768842024413723	0.604716241285583
Diaph1	Diaph1	0.0371980690353286	6.45891554790855	3.12913720941688	0.076904431184324	0.604716241285583
Gtf2e1	Gtf2e1	0.0626459802341074	5.08760014229577	3.12888564074096	0.0769162999855911	0.604716241285583
Chrna5	Chrna5	-0.103552252837137	3.17728067416928	3.12640322827614	0.0770336656266489	0.605123929625791
Col27a1	Col27a1	-0.112829407201636	3.68916703595932	3.12541949503829	0.0770800778939866	0.605123929625791
Gtpbp6	Gtpbp6	-0.0644045550873172	5.86578793209096	3.12470889617435	0.0771136529698652	0.605123929625791
Coasy	Coasy	-0.056193586841351	5.13861821341517	3.12343644241336	0.0771738728002208	0.605215845963768
Tusc3	Tusc3	-0.0361996301704738	8.12272141073792	3.11979275219798	0.0773466203878014	0.606189551919662
Taf13	Taf13	-0.0647667581864623	4.89613265498123	3.11774388922083	0.0774439398104281	0.606571272451168
Cdca7	Cdca7	0.12178183161483	4.29768528207509	3.11558545130699	0.0775461304946849	0.606990633740489
Zmiz1	Zmiz1	0.0485389234003484	8.61202188339868	3.11219046978538	0.0777083861425159	0.607757113529358
Doc2g	Doc2g	0.163995896182903	1.95275776582797	3.10729685375261	0.0779426928636374	0.607757113529358
Kpnb1	Kpnb1	0.0361172930996611	7.81215805741206	3.10618243920063	0.077995575191735	0.607757113529358
LOC4978	LOC497899	0.361721840476437	-0.385663808197054	3.1044113726098	0.0780804515552123	0.607757113529358
Slc25a20	Slc25a20	0.0427726971413428	5.61300089161737	3.10384030634342	0.0781078404577286	0.607757113529358
Usp31	Usp31	0.0619568228173689	5.43443116813295	3.10366543996975	0.0781162292836303	0.607757113529358
Fen1	Fen1	-0.0695632262655928	3.99318831304394	3.103227146067	0.0781372597343071	0.607757113529358
Cabyr	Cabyr	-0.132113248008706	1.89916652952009	3.1018032955648	0.078205621755706	0.607757113529358
Rbm25	Rbm25	-0.0415147219925282	7.3391238028146	3.10148120600492	0.0782210948725639	0.607757113529358
Sertad2	Sertad2	0.0968672993350836	3.04561549632501	3.1007640222271	0.0782555600754549	0.607757113529358
Fads2	Fads2	0.0408752464496878	7.87300527958757	3.10048458653086	0.0782689931481176	0.607757113529358
Ss18	Ss18	0.0676808035450913	5.29440001177305	3.10043464582205	0.0782713941664557	0.607757113529358
Tgfb1	Tgfb1	0.102270261191767	3.15088222929403	3.10031213047015	0.0782772847192177	0.607757113529358
Ywhab	Ywhab	-0.0327590668460356	9.8318264929645	3.09776261761112	0.0783999729681574	0.608331141293967
Grhpr	Grhpr	-0.0878829176140245	4.11655190998224	3.09609355288704	0.0784804058665839	0.608576770663373
Slc4a10	Slc4a10	-0.0542327491826566	6.29637531983368	3.09327936193799	0.0786162224323832	0.608824703047149
A2m	A2m	-0.0960480485551125	4.21672876505866	3.09315952674663	0.0786220114518056	0.608824703047149
Rexo4	Rexo4	0.134479435434726	5.26544471484454	3.09298709055862	0.0786303423348564	0.608824703047149
LOC3628	LOC362863	-0.157554566251296	1.4638580516056	3.09191367562047	0.0786822234464084	0.60862870548099
Ern1	Ern1	0.101902868081306	3.20627802783611	3.0887367319028	0.0788359900397467	0.609072525006697
Ssh2	Ssh2	0.0506615744075144	6.40542955658518	3.08806933549644	0.0788682336689451	0.609072525006697
Smim17	Smim17	-0.0952943764375174	3.29534123585602	3.08767400227112	0.0788874992040927	0.609072525006697
Lasp1	Lasp1	0.0327782757098073	8.56853793650146	3.08669747960522	0.0789348619809112	0.609072525006697
Eif3d	Eif3d	0.0388161179039382	6.90624242980681	3.08345610532091	0.0790922930477356	0.609913099107909
Mief1	Mief1	0.0545101485612439	5.00042345259344	3.08230529532914	0.0791482634656438	0.609964720676428
Hira	Hira	0.0307386686239978	7.41101212793132	3.07837176032627	0.0792911483054435	0.61068867741174
Krt10	Krt10	-0.133395301282321	2.39727611326011	3.07529586161631	0.0794901293680025	0.610982819199872
Zeb2	Zeb2	0.0785107888692615	7.80900321463919	3.07513282181221	0.0794980996757907	0.610982819199872
Sh2d1a	Sh2d1a	0.112224284132335	3.23320126172342	3.07432971442115	0.0795378636474459	0.610982819199872
RGD1359	RGD135916	0.0380882115249972	6.14172827125933	3.07406347207662	0.0795503991941073	0.610982819199872
Smg7	Smg7	0.0335588714026733	7.0620946236544	3.07102777290972	0.0796990697292273	0.610982819199872
Zfp347	Zfp347	-0.0774966150053928	4.18742310790042	3.07020392380979	0.0797394686013473	0.610982819199872
Reck	Reck	-0.124434189760142	5.06550436286297	3.07011385742021	0.0797438865011431	0.610982819199872
Pdxp	Pdxp	0.0391086327123436	7.5841727778062	3.06944672206644	0.0797766187661694	0.610982819199872
Spag5	Spag5	-0.106811805314546	3.39608538849606	3.06863595909468	0.0798164174535676	0.610982819199872
Pigp	Pigp	-0.0889441282412242	3.42947101142729	3.06717442232312	0.0798882153493861	0.610982819199872
Zmat3	Zmat3	0.0575135495868167	6.32781403856473	3.06686352090506	0.0799034973265576	0.610982819199872
Kdm2b	Kdm2b	0.0352467553287885	7.78427493825665	3.06576897456313	0.0799573225271885	0.610982819199872
Vsig2	Vsig2	-0.180602552176944	1.32115722383687	3.06556737839465	0.079967241624861	0.610982819199872
Naa35	Naa35	0.0442817518409928	6.17508122572654	3.06178185217142	0.080153727800914	0.611538930000982
Plp2	Plp2	-0.139568979472491	2.25268234896024	3.05984522460434	0.0802493129797692	0.611538930000982
Scp2	Scp2	-0.0473074800311296	6.40444239844504	3.05946058739236	0.0802682119515376	0.611538930000982
Fubp1	Fubp1	0.0677277048873672	7.40388308344373	3.0593528789099	0.08027363300987	0.611538930000982
Elovl5	Elovl5	0.0485141287812638	5.23410602451507	3.05880081314123	0.0803009124789358	0.611538930000982
LOC10254	LOC102549	0.0829827699005676	5.20594041047297	3.05815581131267	0.0803327968742026	0.611538930000982
Fgfr1op2	Fgfr1op2	-0.0503526128370182	5.0023629476857	3.05492481487094	0.0804927203716207	0.612382726876044
Tbrg4	Tbrg4	-0.0589661498784404	5.4097138842032	3.05117264271156	0.0806788711997079	0.613424909176573
Abca8a	Abca8a	-0.139712444377162	4.51351085160744	3.04807277717674	0.0808330102906722	0.614222575758267
Mchr1	Mchr1	0.137824632995267	2.39893089689505	3.04313907341226	0.0810789912977797	0.615188049640213
Rhbdl1	Rhbdl1	-0.0722482014940034	5.83628030092619	3.04175529901019	0.0811481273917431	0.615188049640213
Uchl5	Uchl5	-0.0660413288589687	4.40961462873387	3.04051715264217	0.0812100415238551	0.615188049640213
Grik3	Grik3	0.0581338473310785	6.43836559165633	3.04040791530221	0.081215506447503	0.615188049640213
Myh1	Myh1	-0.180986198244699	0.9252672982737	3.03913794685295	0.0812790694007008	0.615188049640213
Sox1	Sox1	0.0451230248927958	6.90826599816833	3.03858290987213	0.0813068663064243	0.615188049640213
Atn1	Atn1	0.0357056935552644	8.4963991259665	3.03845570777081	0.0813132381817102	0.615188049640213
Tada1	Tada1	0.0532658549621641	5.07488151340901	3.03760557547584	0.0813558372931275	0.615188049640213
Prodh2	Prodh2	-0.205868653754198	0.275923568825401	3.0338184228354	0.0815458949455511	0.616011534805011
Ddi2	Ddi2	0.0660055995264226	5.19343206518789	3.03349488876978	0.0815621588120444	0.616011534805011
Imp3	Imp3	0.0621730503736727	5.15123639229181	3.03249016342468	0.0816126732824062	0.61602016003907
Ppp1r32	Ppp1r32	-0.184921049760979	1.94756263655778	3.0311438297361	0.0816804156868839	0.616040152801783
Pex13	Pex13	-0.0610787360680496	4.31270139527556	3.03047541889368	0.0817140701199768	0.616040152801783
Pigu	Pigu	0.0527411191941209	5.14492979383284	3.02821421914127	0.081822988795369	0.616488786856412
Selenoo	Selenoo	-0.0668996862129155	4.22626552264824	3.0240422783728	0.0820387443286206	0.617645297145807
Zbtb49	Zbtb49	0.0749350292904203	3.64858487624827	3.02222374655729	0.0821307760145728	0.617645297145807
Lrba	Lrba	0.0678870743188235	4.74026357816521	3.02211445876908	0.0821362103638969	0.617645297145807
Camsap3	Camsap3	0.0322285164218354	7.38990020386494	3.02032748881681	0.0822278729675558	0.617645297145807
Nlgn3	Nlgn3	0.0414545481206985	7.68965111451509	3.01954424270924	0.0822665826116272	0.617645297145807
LOC4993	LOC499331	0.0548398648842718	5.11727647782778	3.01940784994848	0.0822735018520717	0.617645297145807
Atp2a3	Atp2a3	-0.236192242667635	0.903589689727184	3.01395410082481	0.0825509914981659	0.619154527982528
Hif3a	Hif3a	-0.247543386367979	0.919067318436647	3.01350059476137	0.0825737865322535	0.619154527982528
Sec24c	Sec24c	0.0327804568493407	7.26290202037035	3.0087246660596	0.0828174271834515	0.620314731757517
Mpdz	Mpdz	-0.0444203014806426	5.91865209218597	3.00850651168514	0.0828285747291566	0.620314731757517
Gtf3a	Gtf3a	0.0540291710433456	5.31921509074232	3.00754615354922	0.0828776675354477	0.620314731757517
Tmem106	Tmem106a	0.26580243717216	0.565484947458036	3.00461964930653	0.0830274467851269	0.62106322154558

Zfp867	Zfp867	0.0874288360161628	3.43008824851901	2.98421221584938	0.0843801921685623	0.623324763512532
Prpf4b	Prpf4b	-0.0452142521458378	7.08141270105602	2.98221651337258	0.0841839132234353	0.623607004744149
Oga	Oga	-0.044260581382377	7.66722545295923	2.98146703271175	0.0842229010917678	0.623607004744149
Tpcn2	Tpcn2	-0.178924916827089	2.14328321342784	2.98039075896622	0.0842789228234299	0.623607004744149
Zfp2	Zfp2	0.0636841696094056	4.90288879594196	2.97957984956808	0.0843211585293662	0.623607004744149
LOC10091	LOC100912	0.234639630349607	0.0680103617359516	2.97869780138478	0.0843671254214437	0.623607004744149
Pknox1	Pknox1	0.0633942979950981	3.92225152220702	2.97552359276555	0.084532769981615	0.624461438241007
Fsip1	Fsip1	-0.15672953672762	1.40358948070355	2.97058596658713	0.0847906123245247	0.625985544362778
Armcx2	Armcx2	0.037004309860718	7.61702852181258	2.96794837277561	0.08492950112395	0.626650139280618
Arfip2	Arfip2	-0.0381806423686967	6.25341802800459	2.96657570339671	0.0850015535596298	0.626811101515072
Arhgef25	Arhgef25	-0.0483010674812654	5.99138236824314	2.96420269825188	0.0851211504693187	0.627322264858647
Gprc5c	Gprc5c	-0.154475761860886	1.56268825711513	2.96236314766072	0.0852233011867271	0.62748650745401
Slc10a1	Slc10a1	-0.242570746674871	0.154011185886978	2.96197007447749	0.0852440194064717	0.62748650745401
Sipa1l2	Sipa1l2	0.0541490523072199	6.77514286884129	2.9604014776617	0.0853267517389845	0.627506421119034
Tbr1	Tbr1	0.0653734395814313	7.79051439815134	2.95861087825695	0.0853684823501833	0.627506421119034
Rtbd10	Rtbd10	0.0359585062368205	6.40468808221282	2.95845275314264	0.0854296540889587	0.627506421119034
Lat	Lat	-0.256089482795367	0.403946075735389	2.95787631860745	0.0854601184189312	0.627506421119034
Usp24	Usp24	-0.0318813473820592	7.57722749725401	2.95654522455295	0.0855305211031291	0.627506421119034
Glt8d1	Glt8d1	-0.0660689951434449	5.37811321956872	2.95552501611374	0.0855845055779867	0.627506421119034
Kcnd3	Kcnd3	-0.0541295042858872	6.64161285476707	2.95525549645144	0.085598776047495	0.627506421119034
Rogdi	Rogdi	-0.0547671877034884	6.35587225192396	2.95321722425919	0.0857014791205096	0.627890402223487
Fbxo27	Fbxo27	-0.074600480318585	3.82412854102416	2.95230561662389	0.0857551345292517	0.627914793300644
Rdh14	Rdh14	0.0620756506522439	4.27528478488909	2.95068106798551	0.0858413749274809	0.627978774490625
Rps6	Rps6	-0.0453724018985871	8.69544749622311	2.95015357316993	0.0858693977295085	0.627978774490625
Zbtb17	Zbtb17	-0.0520862329787647	5.75981263321243	2.94829011685146	0.085963152020611	0.627978774490625
B3galt4	B3galt4	0.120712588583641	2.5811222813918	2.94743110150545	0.0860141846253378	0.627978774490625
Fbxl5	Fbxl5	-0.0411682908281751	6.40630235890638	2.9474058910135	0.0860155266173342	0.627978774490625
Cavin1	Cavin1	0.0916272649303112	4.30198280995483	2.94561837277936	0.0861107359407762	0.627985025598477
Plekha5	Plekha5	-0.0549361606680226	6.13784232574984	2.9454742572662	0.0861184169911834	0.627985025598477
Ca5b	Ca5b	-0.278936255824165	-0.047533743126715	2.94244110872108	0.0862802494969748	0.628499130627415
Dlg1	Dlg1	0.0599637174032088	4.86255496453815	2.94197279180746	0.0863052655829687	0.628499130627415
Sp110	Sp110	-0.162738232798331	1.57792978102758	2.94091714770455	0.086361684064093	0.628499130627415
Zbtb11	Zbtb11	0.0655855330745306	5.5647953866575	2.93893805982539	0.086414049610846	0.628499130627415
Klk1	Klk1	0.461577090322844	-0.463708606001757	2.93846405530386	0.0864294096653859	0.628499130627415
Arl6ip6	Arl6ip6	0.0879062395380078	3.99252299472177	2.93795515020805	0.0865201997951979	0.628720170556019
RGD1311	RGD131184	0.0712451966831259	3.90771375709366	2.93611758099363	0.086618698326252	0.629089557125388
Acsl6	Acsl6	-0.0560512711762669	5.7678914950803	2.93426291967575	0.0867128650087093	0.629354451325797
Lcp2	Lcp2	0.189371792744904	1.27594206877535	2.93350760319642	0.0867588087118355	0.629354451325797
Kcnh1	Kcnh1	-0.0914328223838827	4.36436664481841	2.93056096426015	0.086917286861424	0.629837600105573
Sdc4	Sdc4	-0.120362357675749	3.31792950655038	2.93038921160677	0.0869263723158044	0.629837600105573
Gxylt1	Gxylt1	-0.06059864502062	5.22369770740394	2.92915702846933	0.0869929045106993	0.629872295180333
Gemin5	Gemin5	0.0629522141490314	5.28501220235683	2.92809399757277	0.0870502080033238	0.629872295180333
Rpl22l1	Rpl22l1	-0.0909654720986243	5.53814723674105	2.92749327036913	0.0870826087357463	0.629872295180333
Gan	Gan	0.0913492530010697	4.32161279820429	2.92611870250508	0.0871567963589372	0.629922608579795
Pygm	Pygm	-0.138062187354857	2.7024095658186	2.92468941316231	0.0872459014760694	0.629922608579795
Cxcl14	Cxcl14	-0.10781427617067	5.18721842397721	2.92290737617441	0.0873303836326318	0.629922608579795
Htr1f	Htr1f	-0.317333008752775	-0.658224525256227	2.92248382306865	0.0873533066012386	0.629922608579795
Smpdl3b	Smpdl3b	-0.184765277276077	1.49004148525938	2.92165541502195	0.0873981594204593	0.629922608579795
Kcnab2	Kcnab2	-0.0645125588954725	4.66716219475943	2.92145838450979	0.0874088309914523	0.629922608579795
Fem1b	Fem1b	0.0523585319793946	7.17131623535214	2.91818212054723	0.0875864870731681	0.629922608579795
Csad	Csad	-0.0933235644143392	3.83966597280275	2.91707820136514	0.0876465440269561	0.629922608579795
Mical3	Mical3	0.0442268140971016	6.87830690359923	2.91646230088867	0.0876799011510666	0.629922608579795
Rlbp1	Rlbp1	-0.0661459622580753	5.51979766006496	2.9151836596924	0.0877494220348734	0.629922608579795
Oprm1	Oprm1	0.144181826344882	2.13093842969997	2.91487403911373	0.087766265365903	0.629922608579795
Hadh	Hadh	-0.0698798052973384	4.23448289127954	2.91420859245135	0.087802477457283	0.629922608579795
Pltp	Pltp	-0.0692351678304488	5.41781238309417	2.91306380143745	0.0878648122567143	0.629922608579795
LOC6888	LOC688801	-0.232812603390223	0.152494612159087	2.91298282811066	0.0878692231351801	0.629922608579795
Nalcn	Nalcn	-0.0563305567946781	6.72475471854058	2.91295135894885	0.0878709374267402	0.629922608579795
Cpne6	Cpne6	-0.143582002287513	5.37778244556908	2.91211377338301	0.0879165784582202	0.629922608579795
Angptl2	Angptl2	-0.144567318273241	2.28836961587446	2.91117491224853	0.087967758647686	0.629922608579795
Cdc37l1	Cdc37l1	0.0480576209589276	5.40733462556258	2.90900480257127	0.0880862147052288	0.629922608579795
Dhx58	Dhx58	-0.214286742927983	0.774835737946384	2.90879811527311	0.0880975048677326	0.629922608579795
Ccnh	Ccnh	0.0591297348074814	5.49435802404355	2.90730098859394	0.0881793311259553	0.629922608579795
Kctd3	Kctd3	-0.0280120700192196	7.37791939838303	2.90727815197619	0.0881805798113029	0.629922608579795
Dyrk1b	Dyrk1b	0.0636017067058274	4.89978815215613	2.90691808276633	0.0882002722761002	0.629922608579795
Elavl4	Elavl4	0.0509040911828084	7.6238754527775	2.90589627693004	0.0882561813019654	0.629961312416832
Cavin4	Cavin4	-0.135175366230886	2.49352989430686	2.90402713649677	0.0883585524101803	0.630331422768336
Pitpnm1	Pitpnm1	-0.0404186779934554	8.11736468365259	2.89877343082308	0.0886469829004524	0.631032495023774
Abhd2	Abhd2	0.0675930993466883	5.93504495151434	2.89819210963736	0.0886789603136764	0.631032495023774
Trim41	Trim41	-0.0450208050315587	5.97911102525024	2.89751251382031	0.0887163594869642	0.631032495023774
Luc7l3	Luc7l3	-0.075401578006749	7.85458848640368	2.89573933647814	0.0888140206150195	0.631032495023774
Cntf	Cntf	-0.158461490934093	1.54380863151139	2.89485388617706	0.0888626321045895	0.631032495023774
Vps52	Vps52	-0.0697979826731377	7.3091271174822	2.89396186648396	0.0889120351478745	0.631032495023774
Adora2b	Adora2b	-0.138600411196897	2.24666283280028	2.89386241897327	0.0889175224171673	0.631032495023774
Mrps2	Mrps2	0.0461630097253324	6.35693186216742	2.89371772507267	0.0889255308267991	0.631032495023774
Dync2li1	Dync2li1	-0.050122384266092	5.09387853334198	2.89323753968832	0.0889520101232477	0.631032495023774
Syt1	Syt1	-0.0515988194336491	7.94317500165769	2.89306527361484	0.0889615202081517	0.631032495023774
Gpatch4	Gpatch4	0.0529448519745088	4.43126683088635	2.89162932722301	0.089040835669096	0.631224742280327
Dgat2	Dgat2	-0.058467212668814	5.02025149649794	2.88867182929298	0.0892044367386708	0.631224742280327
Sdhaf1	Sdhaf1	0.0553260050670271	6.0524361327114	2.88698444165451	0.0893034683576496	0.631224742280327
Rasal1	Rasal1	-0.0919192341234605	6.44901075881527	2.8860465514652	0.0893499332611049	0.631224742280327
Ccdc25	Ccdc25	-0.0554352807836485	5.3806784498856	2.88592948419843	0.0893619750505866	0.631224742280327
Card6	Card6	0.160972104136308	1.69360819471073	2.88517493061139	0.0893982967139716	0.631224742280327
Kctd13	Kctd13	0.0431651049545476	6.8555943846675	2.88499764219043	0.089408137280064	0.631224742280327
Mrpl50	Mrpl50	0.0728117332813635	3.81724562854965	2.88471841630184	0.089423638508045	0.631224742280327
Hr	Hr	-0.0717609823186607	5.05247754651497	2.88423359873353	0.0894450063599307	0.631224742280327
Them6	Them6	0.0855723760771522	3.77939546381288	2.88323249980678	0.0895006205508682	0.631237723513779
Hdhd2	Hdhd2	-0.0543192004973023	5.21341734559751	2.88021837550515	0.0896738598613843	0.631237723513779
Cbx2	Cbx2	0.104930026103205	4.01706216476339	2.88003013444656	0.0896843430969966	0.631237723513779
Prkx	Prkx	0.0639300672581384	5.97674173831699	2.87981907042288	0.0896960995719043	0.631237723513779
Hacd1	Hacd1	-0.119612384369852	2.43543404481526	2.87975252720641	0.0896998063468726	0.631237723513779
Pcbd1	Pcbd1	-0.0985732368511713	3.83127077094	2.87643911300822	0.0898845896614137	0.631864301656039
Nampt	Nampt	0.0470282243352946	5.764488396307	2.87623990480492	0.0898901286719138	0.631864301656039
Tnfaip8l2	Tnfaip8l2	0.158265172974484	1.35515567976755	2.87525267562039	0.0899452692111948	0.63189589609858
Drd1	Drd1	0.137974672590768	4.47113395561023	2.87347373169476	0.090050312646324	0.632277865564584
Usp40	Usp40	-0.0700151067526886	4.99920850811585	2.87200983776669	0.0901322422125336	0.632497180025749
RGD1304	RGD130458	0.0732005742026056	4.30125438840101	2.87041331909054	0.090221696880738	0.632542424013597
Oxld1	Oxld1	0.119269450215358	2.67493840027196	2.87008537112406	0.0902400829321313	0.632542424013597
Pank3	Pank3	0.0437582088169205	8.33662675799029	2.8665502219428	0.090438525621571	0.632698923301884
Cwh43	Cwh43	-0.338290826342829	-0.357275595942342	2.86628504484297	0.0904472576478624	0.632698923301884
Reep3	Reep3	-0.0666450115402219	5.35521489034227	2.86607837882762	0.0904650590915075	0.632698923301884
RGD1562	RGD156221	-0.0557647058144222	5.00618785362315	2.86468570824755	0.0905433845737814	0.632698923301884
Zbtb21	Zbtb21	0.0546299117175811	4.78999831451431	2.86354181430589	0.0906077916730092	0.632698923301884
Map1b	Map1b	0.0578252397836609	12.413470617627	2.86295682852357	0.0906407434696614	0.632698923301884
Frmd6	Frmd6	-0.0619454441035714	4.67574181368076	2.86267215476985	0.0906567826275236	0.632698923301884
Slc22a17	Slc22a17	-0.042656467939187	9.32628323659851	2.86229551130888	0.0906780080675886	0.632698923301884
Pick1	Pick1	-0.0453842343021373	6.08824275508779	2.85800136660459	0.0908638931972251	0.632698923301884
Map7	Map7	-0.0901842471138039	4.10445284234419	2.85864727821134	0.090895200012013	0.632698923301884
Pex11g	Pex11g	0.193718081022932	1.10894760897016	2.85798812542231	0.0909211516965985	0.632698923301884
Cox6c	Cox6c	-0.0655271267332164	6.99154555241942	2.85693195382554	0.090980877900649	0.632698923301884
Snx4	Snx4	-0.0361841001490752	6.69300413575636	2.85681263906881	0.0909876277934452	0.632698923301884
Pdia6	Pdia6	0.0445282829984266	7.80302455417159	2.85679952449922	0.0909906327739318	0.632698923301884
Trappc13	Trappc13	-0.0417907529889124	5.97368034169544	2.85474133067399	0.0911048928544003	0.632698923301884
Lypd3	Lypd3	0.126046286700511	2.44504922453316	2.85456754466058	0.0911147390346084	0.632698923301884
Stam	Stam	0.0312412953952887	6.78285067860844	2.85431758897077	0.0911289028253327	0.632698923301884
Ap1g1	Ap1g1	0.0408509508213801	7.09298478831001	2.85269568151578	0.0912208663080904	0.632698923301884
Ufl1	Ufl1	0.0536525443425672	4.59838803095968	2.85179344916443	0.0912720672617856	0.632698923301884
Nin	Nin	0.0561285075444938	5.86988153186751	2.85109391371222	0.0913117868074725	0.632698923301884
Ano4	Ano4	-0.0666216319785943	4.06727509343362	2.85089468106137	0.0913231026502118	0.632698923301884
Irag1	Irag1	-0.17784421997856	0.927361240802867	2.84893795275214	0.091377463283008	0.632695121743669
Leprotl1	Leprotl1	0.0480987393129035	5.42132202857722	2.84860409281978	0.0914533108631013	0.632791247349173
Pkmyt1	Pkmyt1	-0.0851524849461124	3.23713850768565	2.84762332979053	0.0915091233689706	0.632791247349173
Slc38a2	Slc38a2	0.187304475182192	3.92230829886565	2.84558080310772	0.0916254780307878	0.632791247349173
Khdrbs1	Khdrbs1	0.0362274154776611	8.49622996487069	2.84469571960723	0.0916610346123522	0.632791247349173
Ipo4	Ipo4	-0.032001988769847	7.69472926884662	2.8439663581436	0.0917175600466214	0.632791247349173
Mpst	Mpst	0.0447363743487083	5.62396877146327	2.84352295048561	0.0917429892253265	0.632791247349173
Prdm4	Prdm4	0.0474193880549485	5.59163739326466	2.84346173748874	0.0917463626235897	0.632791247349173
Eno3	Eno3	-0.108432029394294	2.31650731121577	2.84211639805154	0.0918231988159489	0.632971299241212
Amigo1	Amigo1	0.0610557421818112	4.17347651540973	2.83897548588826	0.092002857558021	0.633317326884855
Spaca6	Spaca6	-0.128559248270474	2.81619689863274	2.83880534791822	0.0920126002566028	0.633317326884855
Tmem200a	Tmem200a	0.0753980232615332	4.85223229205486	2.83857709989576	0.0920256723284347	0.633317326884855
Cbll1	Cbll1	0.0520696577768506	5.56968563973684	2.83604774979078	0.0921706656165912	0.633965494894161
Lrrc8d	Lrrc8d	0.0425721372361638	6.30207301168744	2.83396399089446	0.0922903038954746	0.634213962206592
Fat1	Fat1	0.0603789427576351	7.73577497808898	2.8336481585589	0.0923084519810188	0.634213962206592
Ptprn	Ptprn	-0.0513381832480261	7.49483370826257	2.83069741139309	0.0924781918128811	0.634610161132495
Inpp5j	Inpp5j	-0.0842586191777101	4.43946450384355	2.82997691585307	0.0925196890550121	0.634610161132495
Snrnp70	Snrnp70	-0.0576975579082391	7.52183558652165	2.8295286584641	0.0925455173228098	0.634610161132495
Pf4	Pf4	0.274849529645446	0.355687337601254	2.82858057604963	0.0926001703257558	0.634610161132495
Gstz1	Gstz1	0.0865544168582259	4.27436193027986	2.82822928232125	0.092620429864733	0.634610161132495
Poc5	Poc5	-0.0654280127535443	4.06818607253742	2.82561521989626	0.092771337362716	0.634809106127198
Bzw2	Bzw2	0.0409233400213084	7.70085887507102	2.82358784380282	0.09288855417903	0.634809106127198
Cblb	Cblb	0.0596111996561189	5.30922237363815	2.82335925946607	0.0929020298250835	0.634809106127198
Eif3i	Eif3i	0.0408266834624258	6.50758818125518	2.82130243041904	0.0930208953401049	0.634809106127198
Myef2	Myef2	-0.035606516018895	7.9721817730945	2.820837767164	0.0930420281430613	0.634809106127198
Ficd	Ficd	0.221623151814772	2.38191389635693	2.82074550701248	0.0930531746751864	0.634809106127198
Rps6ka2	Rps6ka2	0.0450132921403268	6.92488216895299	2.81869147427734	0.0931722319328673	0.634809106127198
Spsb4	Spsb4	0.0519968533605573	5.13970111513098	2.81841723721848	0.0931882533492369	0.634809106127198
Prss2	Prss2	-0.0590701904671331	4.98283596092492	2.81824488680307	0.0931982610274079	0.634809106127198
Galns	Galns	0.129121530865405	3.36132072346612	2.81813294128528	0.0932047618777032	0.634809106127198
Tmem185	Tmem185b	-0.0627025559691947	4.58972279221478	2.81680068235203	0.0932821659568468	0.634809106127198
Sox9	Sox9	0.102057143641358	5.18769634666113	2.81672235288006	0.0932867191259237	0.634809106127198
Rab3il1	Rab3il1	0.0916381518416478	3.1270904178884	2.81620790552589	0.0933169267652854	0.634809106127198
LOC6883	LOC688390	0.164738244449052	1.17967355027846	2.81543184357183	0.0933617624238005	0.634809106127198
Slmap	Slmap	0.0478927460081707	6.05037641898836	2.81428852734703	0.0934283122425289	0.634906404368612
Aida	Aida	0.047313676186129	5.99825730209343	2.8134380720413	0.0934779444197435	0.634906404368612
Scn4a	Scn4a	-0.212922439187079	0.455714900166326	2.81058007652556	0.093638672028285	0.635852726276884
Zdhhc4	Zdhhc4	0.0912968234267212	3.94793631508002	2.80911939840795	0.0937298135011942	0.635736568110903
Rab11fip2	Rab11fip2	-0.0558112643641072	4.54151658076387	2.80872385165547	0.0937529282138384	0.635736568110903
Ptgfrn	Ptgfrn	-0.0426608467702238	7.57948252310504	2.80739888732964	0.0938304125235267	0.635915380801761
Cmpk1	Cmpk1	-0.0369943398679145	7.45975100520303	2.80391598930745	0.0940343246581899	0.636770512709227
Sqstm1	Sqstm1	-0.0527430940095522	8.52800213195443	2.80350454649171	0.0940585120560315	0.636770512709227
[truncated: 3,552,723 more chars]
